# Global trends in research of achilles tendon injury/rupture: A bibliometric analysis, 2000–2021

**DOI:** 10.3389/fsurg.2023.1051429

**Published:** 2023-03-27

**Authors:** Chenguang Wang, Zhaohui Jiang, Ran Pang, Huafeng Zhang, Hui Li, Zhijun Li

**Affiliations:** ^1^ Department of Orthopedics, Tianjin Medical University General Hospital, Tianjin, China; ^2^ Tianjin Chinese & Western Medicine Hospital, Tianjin, China

**Keywords:** bibliometrics, achilles tendon rupture, achilles tendon injury, biblioshiny, visualized study, co-authorship analysis

## Abstract

**Background:**

The Achilles tendon is the strongest and most susceptible tendon in humans. Achilles tendon injuries and ruptures have gradually attracted research attention. However, a bibliometric analysis of global research in this field is lacking. This study involved a bibliometric analysis of the developmental trends and research hotspots in Achilles tendon injuries/ruptures from 2000 to 2021.

**Methods:**

Articles published between 2001 and 2021 were retrieved from an extended database of the Science Citation Index using Web of Science. VOSviewer and CiteSpace were used to analyze the relationships between publications, countries, institutions, journals, authors, references, and keywords.

**Results:**

This study included 3,505 studies of 73 countries, 3,274 institutions, and 12,298 authors and explored the cooperation between them and the relationships between citations. Over the past 22 years, the number of publications has significantly increased. *Foot Ankle International* has published the most papers on Achilles tendon injuries/ruptures, and *British Journal of Sports Medicine* is the most famous journal. Re-rupture, exosomes, acute Achilles tendon rupture, and tendon adhesions gradually become the research focus over the past few years.

**Conclusion:**

Achilles tendon injury and rupture are important research topics. A vast number of newly published papers on this topic have demonstrated that clinicians and researchers are interested in their study. Over time, these recent studies will be widely cited; therefore, this bibliometric analysis should be constantly updated.

## 1. Introduction

The Achilles tendon is the strongest and most frequently ruptured tendon in the human body (1, 2). Epidemiological studies have reported that the incidence of Achilles tendon rupture (ATR) has increased from 11 to 37 per 100,00 population over the past few decades (3). In many nations, more than 60% of ATR are associated with sports games (2); however, there is no difference in the distribution of sports. Therefore, it is important to quantitatively analyze the status, focus areas, and outlook of ATR.

By analyzing published literature, a bibliometric analysis qualitatively and quantitatively evaluates the status of a certain field and predicts development trends (4). Visualizations based on bibliometric analysis can present results in an intuitive and understandable manner for readers (5). Moreover, it provides convincing evidence for experimental strategies and funding decisions (6). In the scientific and social sciences, bibliometric methodologies have been continuously developed and extensively used (6–8). However, few bibliometric studies have been conducted on ATR. Therefore, the present study aimed to systematically analyze ATR research, compare the contributions of different regions, organizations, and authors, and investigate the current status and global research trends.

## 2. Materials and methods

### 2.1. Research methods and data sources

The Web of Science (WOS) database is considered the superior bibliometric database due to its extensive publication coverage (9). During this study, articles published between 2000 and 2021 containing the words “Achilles tendon rupture” and “Achilles tendon injuries” were retrieved from the Web of Science Core Collection.

**Figure 1:**
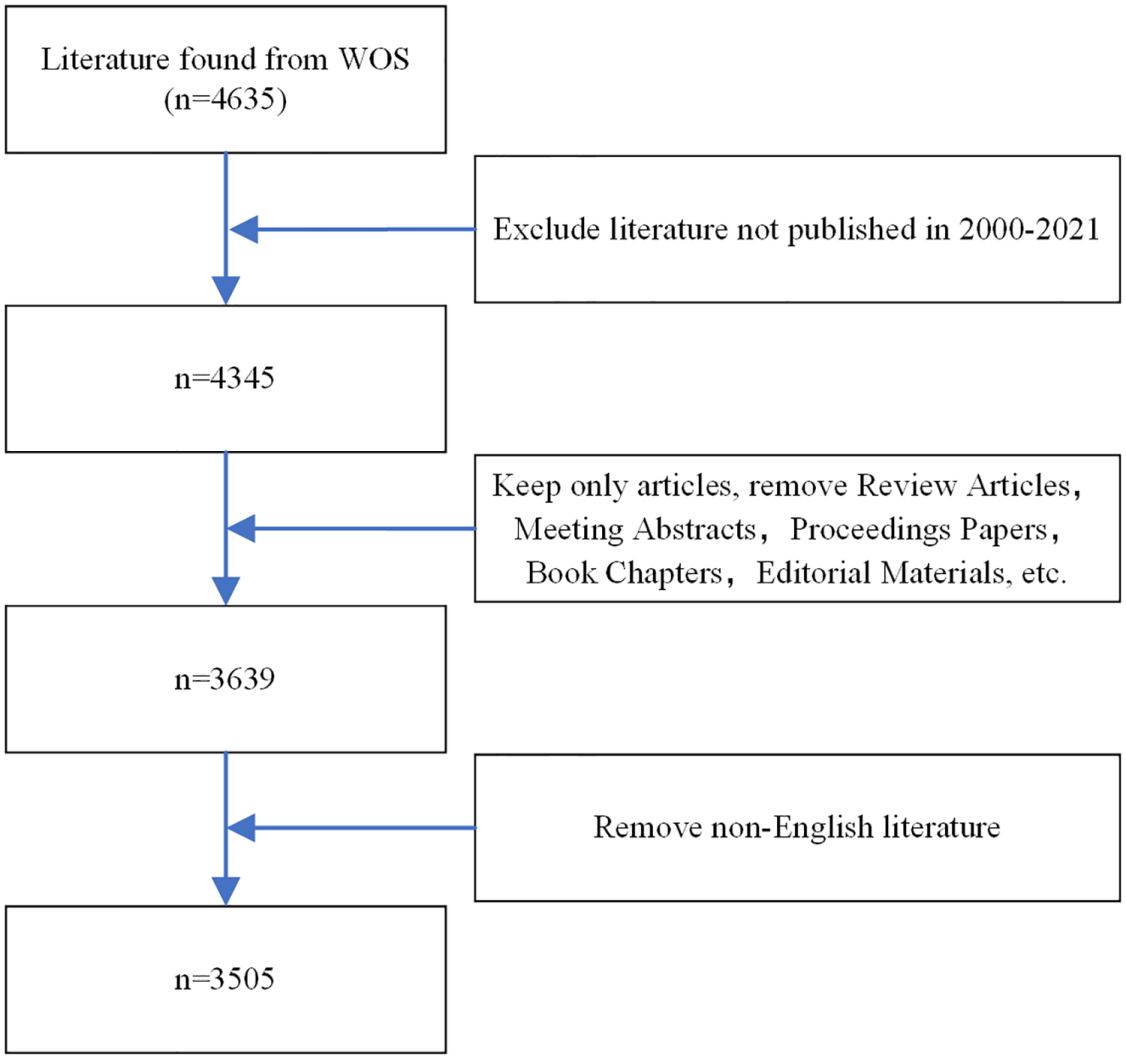
Flowchart of the screening process.

To avoid variation in the results, a literature search was conducted on the same day (July 25, 2022). The search terms were as follows: (((TS = (Achilles tendon rupture)) OR TS = (Achilles tendon injuries)) OR AK = (Achilles tendon rupture)) OR AK = (Achilles tendon injuries). Only original English-language articles were included in this search. Original articles were the only publications included, whereas letters, reviews, editorials, conference abstracts, and news reports were excluded. A total of 3,505 publications were evaluated in this analysis. Figure 1 illustrates the evaluation process. All applicable data were extracted independently by two authors and then compiled for analysis.

### 2.2. Data gathering and cleaning

We downloaded the “txt” format of the data from the WOS, including the complete records and references cited. The collection also included information regarding publication, citations, years of publication, affiliations, references, keywords, H-index, countries, regions, authors, and journals. The data analysis began by merging duplicate words, correcting misspelled words, and removing unnecessary words.

### 2.3. Visualization and bibliometric analysis

Publications and citations are the most common bibliometric indicators used to determine a bibliography's content. A typical measure of productivity is the number of publications (Np), whereas a measure of impact is the number of citations (Nc), excluding self-references. Research quality was assessed using these methods. In recent years, the H-index has been extensively used to assess academic contributions and predict future scientific breakthroughs (10). Medical journals are also widely acknowledged to be evaluated by their impact factor (IF) derived from the most recent edition of *Journal Citation Reports* (11). We used the current journal IF (2022) unless stated otherwise. Microsoft Excel 2019 (Microsoft Corporation, Redmond, WA, USA) was used to analyze the annual publications and citations. Dual-map overlays were performed using CiteSpace 6.1.R2 (Drexel University, Philadelphia, PA, USA).

Co-occurrence keywords, co-citation publications, and collaborative networks were analyzed using VOSviewer 1.6.17 (Leiden, CA, USA). A chord diagram of cooperation among countries/regions and institutions was created using the Charticulator online tool (Charticulator. com). We also used the R packages Bibliometrix and Biblioshiny to analyze topic trends in author keywords.

### 2.4. Ethics in research

Neither human nor animal *in vivo* data was used in this study; only scientometric data were used. Consequently, ethical approval was not required.

## 3. Results

### 3.1. Trends in global publication and citation

**Figure 2:**
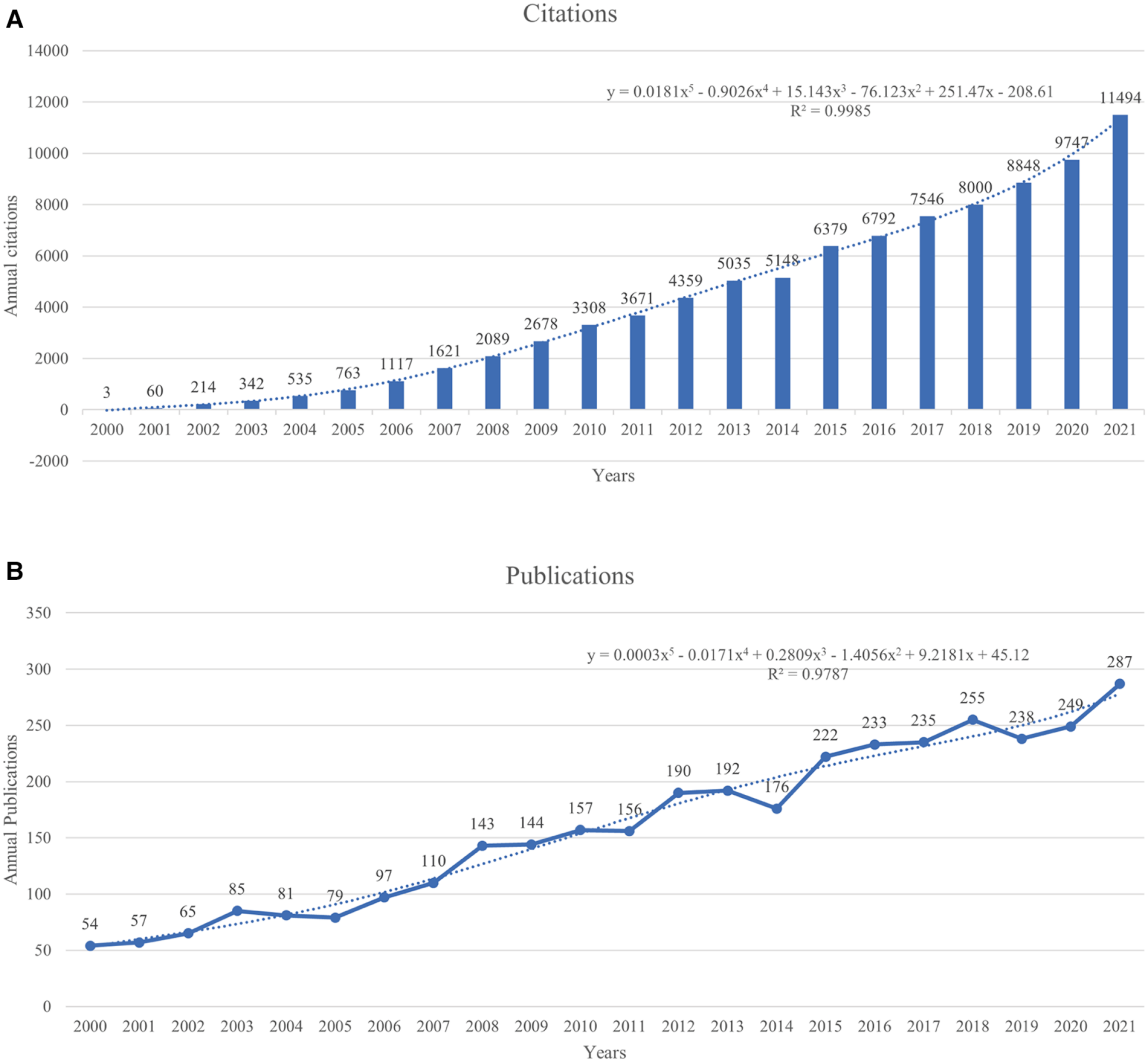
(**A**) The number of annual publications from 2000 to 2021. (**B**)The number of citations from 2000 to 2021.

The search and exclusion criteria identified 3,505 articles published between 2000 and 2021, averaging 159.3 publications per year with a total H-index of 126. Studies on Achilles tendon injuries (ATI)/ATR have increased annually starting with 54 in 2000 and peaking at 287 in 2021. A temporal prediction curve model was developed using the temporal trends as the basis for predicting future publication volumes (Figure 2A; R^2^ = 0.9787). The results of the analysis showed few studies on ATI or ATR before 2005; however, the number increased from 2006 and peaked in 2021. A similar increase in citations was observed, peaking in 2021 at 11,494 (Figure 2B; R^2 ^= 0.9985). Thus, the number of studies focusing on ATI/ATR has increased.

### 3.2. Analyzing affiliations and countries/regions

**Figure 3:**
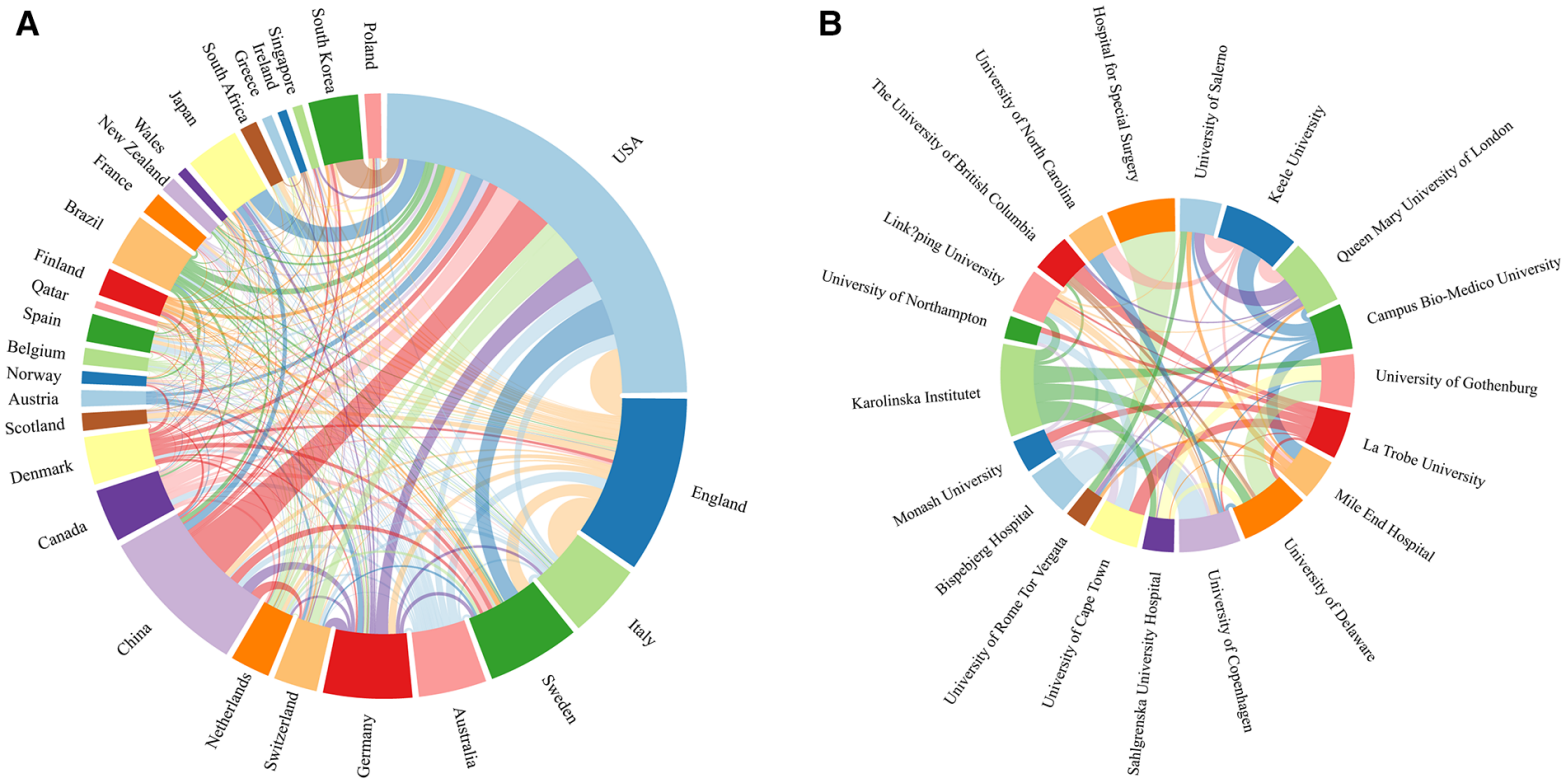
(**A**) Collaboration network between countries/regions. (**B**) Collaboration network between affiliations.

According to the statistical analysis, among 73 countries or regions, 3,274 institutions published articles related to ATI or ATR. Among all authors in the study, we ranked the 10 countries/regions with the highest Np output (Table 1). Articles published in the United States accounted for 31.30% of all publications, followed by those published in England (11.84%) and China (10.07%). USA publications were mentioned 27,572 times, followed by those in England (13,975) and Sweden (9,370). Moreover, the USA ranked first in terms of H-index (81), followed by England (67) and Sweden (56). Table 2 lists the 10 institutions that published the most studies on ATI and ATR. The University of London (139) and Queen Mary University of London (93), followed by the University of Copenhagen (87) and Karolinska Institute, had the highest number of citations per paper (Np) (86). In addition, the University of London ranked first in Nc and H-index. Keele University ranked second and third in terms of Nc and H-index, Keele University ranked second and third, respectively. Of note, seven of the top 10 institutions were located in Europe. Although the United States topped the list of publications by country, only three US institutions were in the top 10 because of the large number of universities and institutions participating in the publications. Not even one university in China has been able to form a world-class research center in this field. Three of the remaining universities were located in the USA. Collaborative ties between nations and institutions were identified by identifying nations and institutions with publication volumes and total link strengths (TLS) exceeding 15. Figure 3A shows that the USA has the most publications and collaborative links with other countries, particularly China, Sweden, Canada, and Japan. Of the 30 countries that met this criterion, the five with the largest TLS were the United States, England, Italy, Sweden, and Australia. Figure 3B demonstrates that the University of Salerno has the most TLS; the Hospital for Special Surgery had quite a few publications but few relationships with other universities, primarily with the University of Rome Tor Vergata.

**Table 1:** Top 10 countries in terms of number of articles published.

Rank	Country/Region	Np	% of (3505)	Nc	H-index
1	USA	1097	31.30%	27,572	81
2	England	415	11.84%	13,975	67
3	China	353	10.07%	4,856	38
4	Sweden	217	6.19%	9,370	56
5	Germany	208	5.93%	5,508	40
6	Italy	177	5.05%	4,078	36
7	Australia	161	4.59%	5,839	43
8	Turkey	142	4.05%	1,550	18
9	Canada	126	3.60%	4,870	37
10	Japan	125	3.57%	2,078	26

**Table 2:** Top 10 institutions in terms of number of articles issued.

Rank	Affiliations	Countries	Np	Nc	H-index
1	University of London	England	136	4,264	42
2	Queen Mary University London	England	93	2,656	35
3	University of Copenhagen	Denmark	87	3,274	32
4	Karolinska Institutet	Sweden	86	2,092	30
5	University of Pennsylvania	USA	74	1,715	24
6	Keele University	England	57	3,353	33
7	Harvard University	USA	55	1,309	23
8	Bispebjerg Hospital	Denmark	54	2,367	28
9	Karolinska University Hospital	Sweden	53	1,054	20
10	Hosp Special Surg	USA	48	1,038	18

### 3.3. Author activity analysis

A total of 12,298 authors contributed to the published ATI and ATR research. A list of the 11 most prolific authors is presented in Table 3. Among them, 495 articles were published, representing 14.1% of all published papers. Among those who investigated ATI/ATR, Maffulli N from Europe received the highest ratings for Np (120), Nc (5,455), and H-index (43), followed by Silbernagel KG from the University of Delaware and Soslowsky LJ from the University of Pennsylvania. Additionally, eight of the top 10 authors were from Europe.

**Table 3:** Top 10 authors in terms of number of articles published.

Rank	Author	Country	Np	Nc	H-index
1	Maffulli N	England	120	5,455	43
2	Silbernagel KG	USA	58	2,527	28
3	Soslowsky LJ	USA	43	1,206	20
4	Ackermann PW	Sweden	39	754	16
5	Karlsson J	Sweden	38	2,209	22
6	Longo UG	England	36	1,634	25
7	Denaro V	England	34	1,513	24
8	Kjaer M	Denmark	34	1,581	19
9	Collins M	South Africa	33	1,310	19
10	Alfredson H	Sweden	30	2,624	23
10	Magnusson SP	Denmark	30	1,578	19

### 3.4. Co-citation analysis

**Figure 4:**
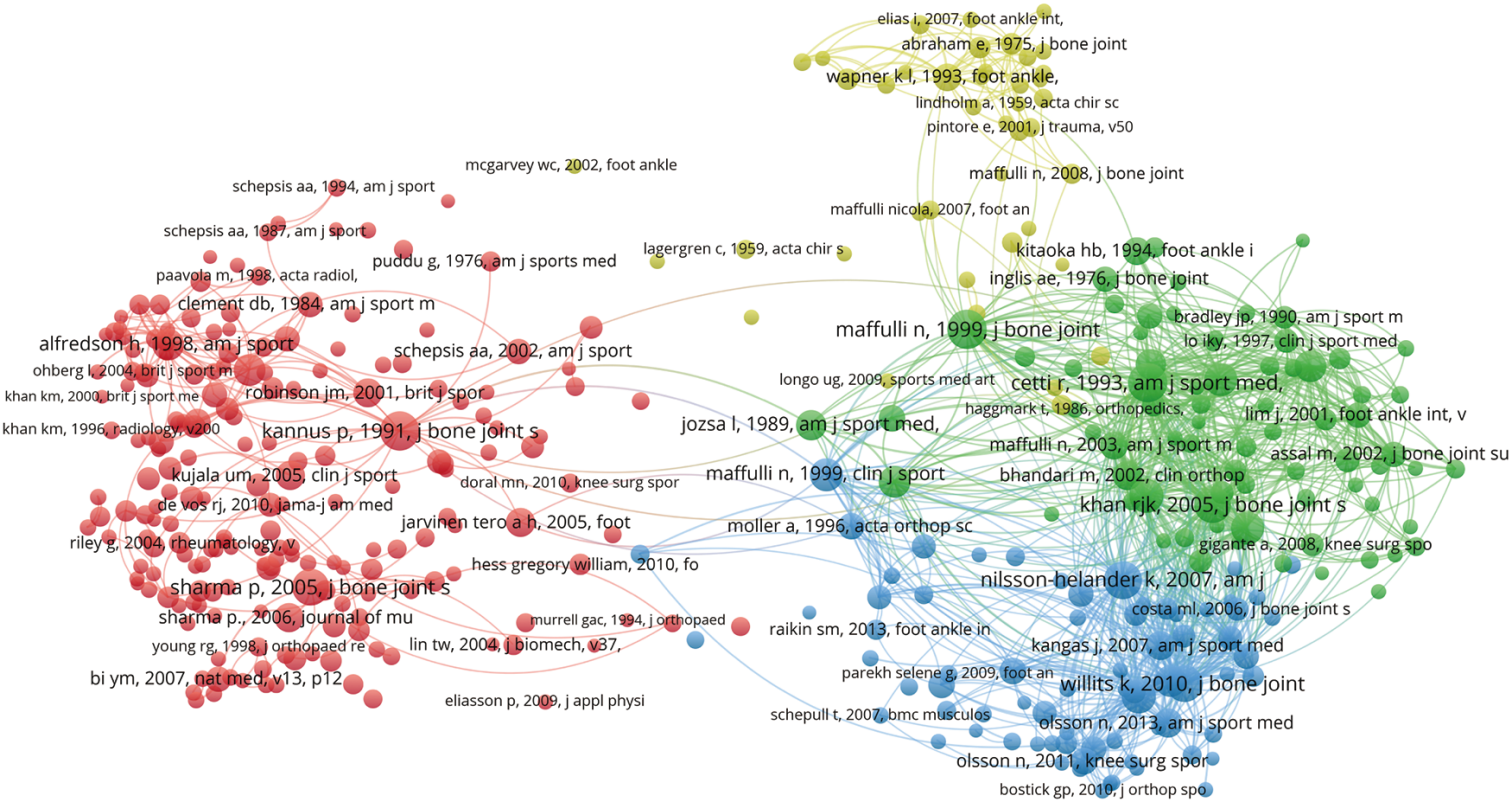
Mapping of co-cited references related to the field.

Two or more publications were simultaneously cited as other publications when they were in a co-citation relationship. According to a VOSviewer analysis of co-citations, the relatedness of publications is determined by the number of times they are cited together; 385 references (defined as the minimum number of citations of a cited reference used more than 30 times) were analyzed. A document's co-citations are represented by the size of its nodes, which corresponds to its TLS. As shown in Figure 4, an article cited in two publications is indicated by a shorter line between its nodes, which indicates a closer relationship. As shown in the red cluster, the strongest connections are represented by (Kannus *P*, 1991, J Bone Joint Surg Am, v73a, p1507, doi:10.2106/00004623-199173100-00009) (12). The green cluster shows the highest connection strength exhibited by (Cetti R, 1993, Am J Sports Med v21, p791, doi 10.1177/0363546593021 00606) (13). In the blue cluster, (Willits K, 2010, J Bone Joint Surg Am, v92a, p2767, doi:10.2106/jbjs.i.01401) (14) indicates the strongest connection strength. In the yellow cluster, (Wapner KL, 1993, Foot Ankle, v14, p443) (15) exhibits the strongest connection. These articles examined the pathology of ATR, surgical and non-surgical treatments, postoperative complications, and reconstruction.

### 3.5. Journal analysis

As shown in Table 4, 3,505 papers were published in 668 distinct journals, and the 10 journals with the highest number of publications were mentioned. *Foot and Ankle International* published the most papers on ATI/ATR (197, IF: 3.569), *American Journal of Sports Medicine* (176, IF:7.01), and *Knee Surgery and Sports Traumatology Arthroscopy* (156, IF:4.114) ranked second and third, respectively. *British Journal of Sports Medicine* (62, IF: 18.473) had the highest IF among the top 10 journals.

**Table 4:** Top 10 journals in terms of number of articles published.

Rank	Journal	Np	Nc	H-index	IF(2021)
1	Foot Ankle International	197	4,319	38	3.569
2	American Journal of Sports Medicine	176	10,427	62	7.01
3	Knee Surgery Sports Traumatology Arthroscopy	156	4,517	38	4.114
4	Journal of Foot Ankle Surgery	137	1,577	21	1.345
5	Journal of Orthopaedic Research	129	5,473	43	3.102
6	Scandinavian Journal of Medicine Science in Sports	79	2,874	31	4.645
7	Journal of Bone and Joint Surgery American Volume	67	4,253	37	6.558
8	British Journal of Sports Medicine	62	4,738	38	18.473
9	Journal of Biomechanics	54	1,015	21	2.789
10	Orthopaedic Journal of Sports Medicine	47	294	11	3.401

**Figure 5:**
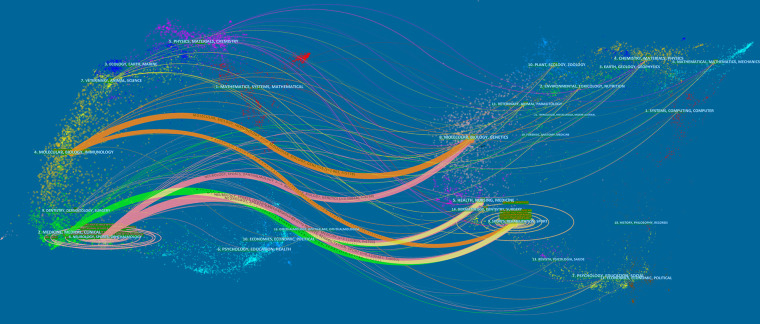
The dual-map overlay of journals related to ATI or ATR.

Using a series of dual-map overlays, Figure 5 shows the number of articles in a journal and describes the publication's genre or subject matter. The research subjects covered by the journals are represented by labels on the map. Citing journals appear on the left side, whereas cited journals appear on the right side. The paths of a citation link are indicated by lines of different colors and widths that depart from and converge on a citing map. To calculate path widths, the z-score–scale citation frequency was considered. Overall, most articles on ATI/ATR research were published in the fields of (i) molecular biology and immunology; (ii) dentistry, dermatology, and surgery; (iii) medicine, medical, and clinical; and (iv) neurology, sports, and ophthalmology. The most cited papers were published in journals of (i) molecular biology and genetics; (ii) health, nursing, and medicine; (iii) dermatology, dentistry, and surgery; and (iv) sports rehabilitation and sports.

### 3.6. Keyword analysis of research hotspots

**Figure 6:**
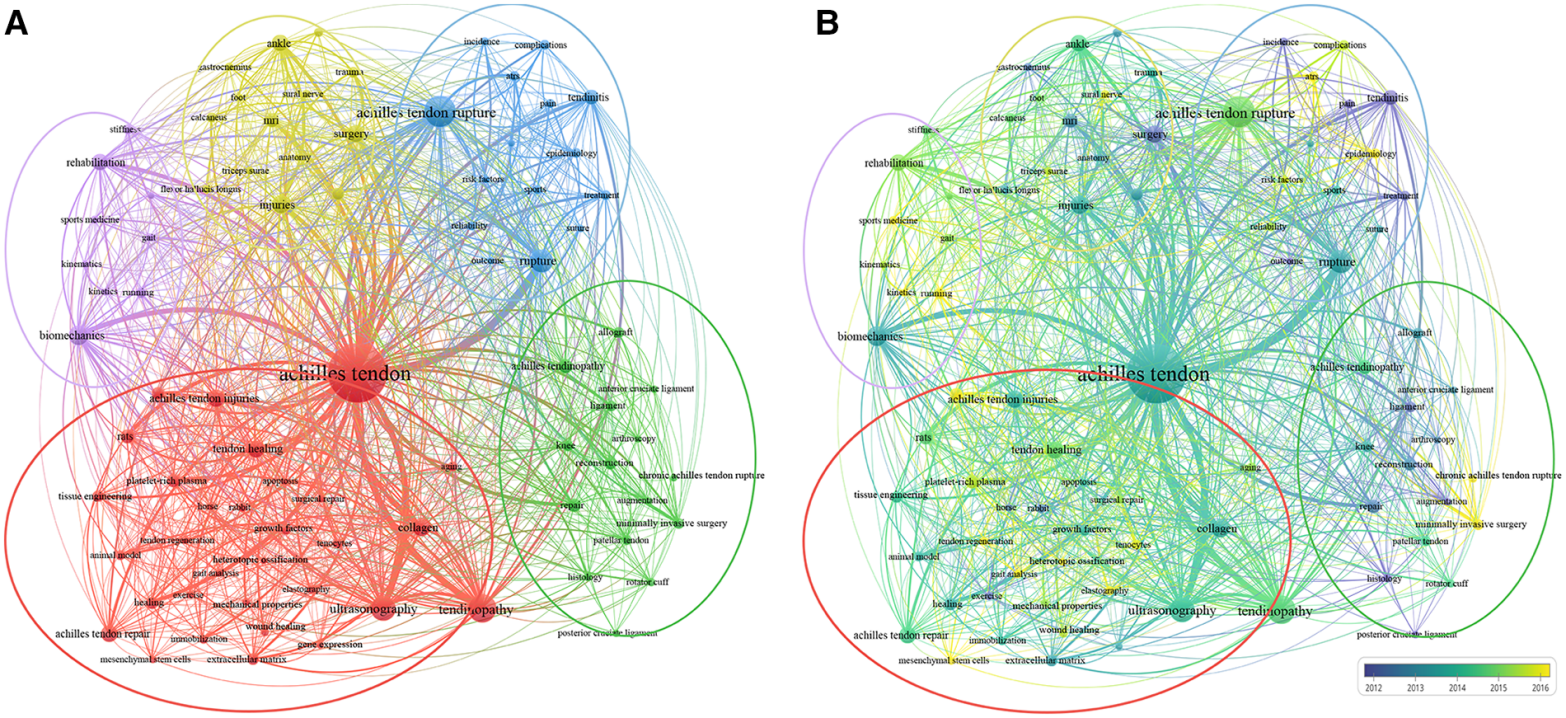
Author keyword co-occurrence visualization of ATI or ATR. (**A**) 85 keywords were divided into five clusters according to different colors: cluster 1: red, cluster 2: green, cluster 3: blue, cluster 4: yellow and cluster 5: purple. The size of the node indicates how often it occurs. (**B**) The distribution of keywords by average frequency of occurrence; Yellow keywords appear later than blue keywords.

The distribution of author keywords was analyzed using the VOSviewer software. To optimize the cluster analysis and visualization, we performed a synonym merge before the analysis and adjusted the minimum number of keyword occurrences to 15. Of the 5,198 keywords, 85 met this threshold. The keyword with the highest frequency was Achilles tendon (1,089), followed by Achilles tendon rupture (302), and tendinopathy (243). As shown in Figure 6A, the largest node was Achilles tendon, followed by Achilles tendinopathy, ATR, ATI, and biomechanics, with an important connection among them. We further performed a clustering analysis of these co-occurring keywords, which could be divided into five clusters: Cluster 1 (animal model research, red nodes), Cluster 2 (treatment study, green nodes), Cluster 3 (clinical study, blue nodes), Cluster 4 (anatomical studies, yellow nodes), and Cluster 5 (kinematics research, purple nodes). The most frequently used keywords were “tendinopathy,” “biomechanics,” “ultrasound,” and “collagen,” indicating that research related to ATI/ATR has mainly focused on pathogenesis and clinical research. The keywords were color-coded using VOSviewer based on the average time they appeared in all included publications (Figure 6B). Blue nodes represent keywords that appeared earlier, whereas yellow nodes represent recent occurrences. These clusters are the most prominent topics in research on ATI/ATR. This study mainly focused on tendinitis and surgery in 2012. Subsequently, research into minimally invasive surgery, heterotopic ossification, and tendon regeneration has gradually increased. The latest research trends mainly involved elastography, chronic ATR, kinetics, and mesenchymal stem cells, which will be of great interest in the future.

**Figure 7:**
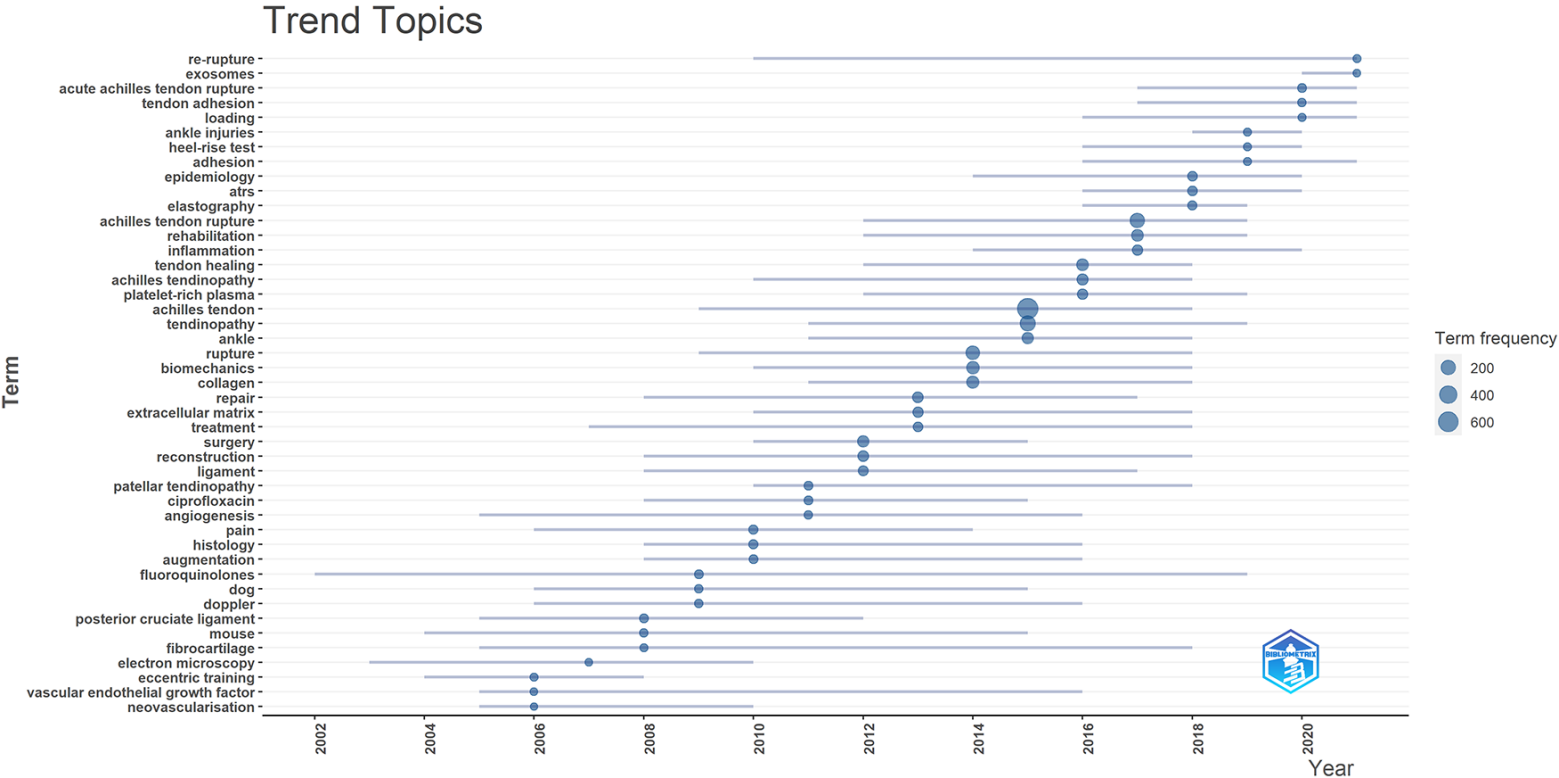
The keywords with the strongest citation bursts from 2000 to 2021.

Using a time-based literature productivity analysis, Figure 7 shows that the circle size represents the number of articles published, while the horizontal line represents the years in which the keywords co-occur. Accordingly, research on the involvement of ATI/ATR, re-rupture, exosomes, acute ATR, and tendon adhesion has steadily gained prominence as rising subjects over the last several years, whereas eccentric training, vascular endothelial growth factor, and neovascularization have gradually receded from prominence.

## 4. Discussion

Using bibliometrics and visual analysis methods, this study examined the current research and future trends in ATI. The number of publications on ATI has increased exponentially over the past 21 years. The variation trend in the number of citations was consistent with this. Research in this field has attracted widespread attention and is developing rapidly worldwide. ATI are on the rise, which is the main reason for the increase (2, 16). ATR tends to occur at a younger age; for example, Lantto et al. reported that the peak incidence was at 30–39 years (2). Sports-related injuries remain the leading cause of ATI, but non-sports-related injuries are increasing at a faster rate (a multifactorial effect) (2, 17). This may cause an increasing number of researchers to explore the epidemiology of ATI, including its etiology, diagnosis, and surgical or non-surgical treatment.

The economic status of a country is closely related to the strength of its scientific research (18). Research in the ATI field is consistent with this rule in terms of national, regional, and institutional contributions. With approximately one-third of the total number of publications in the discipline, the US ranked first in the overall number of papers, citations, and H-index. Britain was second only to the US. As developed countries, the USA and the United Kingdom have strong scientific research strengths, which have greatly promoted research progress in the field of ATI. With the development of the global economy, China has invested considerably in scientific research and contributed to a rapid increase in academic achievements in this field, ranking among the top in the world. From the analysis of institutional contributions, three of the top 10 institutions were from the USA, while the rest were from Europe, which again confirms the correlation between the national economy and scientific research level. It is also noteworthy that although the US has the highest number of publications, the top 10 authors in terms of publications were mainly from Europe, which may be due to the fact that the therapeutic theories for ATI/ATR were initially presented in a greater concentration in European universities. In addition, compared to China, the US, England, and Sweden have higher H-indices, suggesting that Chinese scholars and units should work more on the quality of papers in this field. Similarly, Turkey has conflicting publication quantity and quality. Overall, ATI is a multidisciplinary field with close collaboration between countries/regions and institutions, with the US at the center, which will enhance future research.

A collaborative and co-citation analysis of the authors' contributions can identify core authors in the field of ATI research. Professor Maffulli N specializes in foot and ankle diseases and is the most prolific author in the field of ATI, with the highest H-index and number of citations. In 1998, Maffulli et al. first proposed the term tendinopathy for pain, swelling, and limited function in the tendon region to avoid confusion with tendinitis such as Achilles tendinopathy. This name has since been accepted by most doctors and is still in use. Professor Maffulli N performed in-depth research on the epidemiology (19), histopathology (20), treatment (21), and rehabilitation (22) of ATI. Silbernagel KG et al. from the University of Delaware worked on the conservative management of Achilles tendinopathy (physical therapy) study (23). Soslowsky (University of Pennsylvania) focused on biomechanics after ATI and proposed that fatigue load is a sensitive indicator for tendon healing (24). Other researchers' results leading these rankings are also noteworthy. As scholars who have made outstanding contributions to the field, their research results are significant and representative, and young scholars should focus on their publications.

The research with the highest co-citation analysis was considered to have a high academic influence. A study by Kannus et al. published in *Journal of Bone and Joint Surgery* in 1991 found degeneration on histological examination in 97% of 891 patients with spontaneous ATR, including hypoxic degenerative tendinopathy, myxoid degeneration, tenonolipomatosis, and calcific tendinopathy (12). A prospective randomized study by Cietti et al. published in 1993 compared the surgical and non-surgical treatments for ATR. The findings of this study indicate that surgical treatment is desirable, but non-surgical treatment is an acceptable alternative (13).. This laid the foundation for a comparative study of surgery and conservative treatment in many aspects. A randomized controlled study by Willits K. et al. published in *Journal of Bone and Joint Surgery* in 2010 compared surgical and non-surgical treatments for ATR. Compared with the study by Cetti et al. published 17 years ago. It is important to note that ATR was treated with emphasis on accelerated functional recovery. Considering these cases, ATI research and treatment philosophy have advanced significantly in recent years. More notably, Willits et al. supported accelerated functional rehabilitation and non-surgical treatment of acute ATR (14). In 1993, Wapner KL published a new technique in *Foot Ankle*: *Reconstruction* of chronic ATR using the flexor hallucis longus tendon (15). The academic impact of these studies was profound, laying the foundation for subsequent research on ATI.

According to the journal analysis, the top 10 journals account for nearly one-third of all publications, and researchers can now track the latest developments in the field of ATR by monitoring the latest research in these journals. *Foot Ankle International*, with the largest number of publications (197 articles), is a well-known journal in this field. One of the ATR studies published in 2021 proposed the concept of slowed recovery (25) to compare the impact of different levels of positive rehabilitation on clinical outcomes. Slow-paced rehabilitation after acute Achilles tendon repair is more effective than traditional rehabilitation after percutaneous repair (25). *The American Journal of Sports Medicine*, which had the highest H-index, ranked second. A recent article in this journal in 2020 investigated the effect of quercetin on collagenase-induced tendinopathy, a mechanistic study aimed at screening new therapeutic agents for the treatment of Achilles tendinopathy (26). The *British Journal of Sports Medicine* had the highest IF value (18.473). The guidelines published in 2021 provide a comprehensive, evidence-based overview of the risk factors, prevention, diagnosis, imaging, treatment, and prognosis of Achilles tendinopathy, which can help other investigators in their medical practice (27). In general, top journals publish articles on popular topics in the field of ATI, and researchers continue to pay attention to these journals.

Changes in the ATI and ATR domains can be identified using keyword co-occurrence analysis (Figure 6). It is roughly divided into five subfields. Figure 6B shows the changes in the research focus in each subfield in the temporal dimension. Regarding the establishment of animal models, most of them are rat, rabbit, and horse. For example, researchers have established a rat model of ATI to examine the mechanism of ectopic ossification; the endothelium–mesenchymal transition is central to the pathological process (28). In animal studies and basic medical research, the development of computer hardware has greatly contributed to the study of heel ATI, allowing for a more comprehensive assessment of Achilles tendon repair and function in terms of the gait analysis (29) and elastography. Shear wave elastography (SWE) is a powerful tool for estimating tissue stiffness and sensitive to the early detection or monitoring of the tendinopathy (30). SWE and compression elastography are two major ultrasound elastography techniques that have undergone significant developments in recent years (31). Computer science is expected to intersect more with the fields of ATI and ATR as hardware advances and computing power increases. In basic research, Achilles tendon repair has become a hot topic, and exosomes and stem cells have gradually gained attention in recent years.

In 2019, Chamberlain et al. utilized exosome-educated macrophage–treated ATI exhibiting (a) improved mechanical properties, (b) reduced inflammation, and (c) earlier angiogenesis (32). Meanwhile, extracellular vesicles derived from mesenchymal stem cells have gained much momentum in the treatment of tendinopathies (33). Mesenchymal stem cells are a hot topic in the field of tendon regeneration engineering (34). Similar to the effects of platelet-rich plasma on tendon repair, more in-depth studies on tendon repair and regeneration are expected in the future (35). In 2012, the primary research directions were tendinitis and surgery. A review of specific studies during this period revealed that many studies comparing surgical treatment with non-surgical treatments of ATR (36–38) or investigated different surgical approaches (39, 40). Since then, increasing studies on minimally invasive surgery, heterotopic ossification, and tendon regeneration have come into view. Studies comparing the efficacy and complications of open and minimally invasive surgery for acute ATR concluded no significant intergroup difference in clinical outcomes after treatment with conventional open or minimally invasive surgery (41). Tendon ossification is usually caused by ATI or chronic tendon degeneration.

Ectopic ossification has attracted increasing attention from scholars (28, 42, 43). In recent years, the treatment of acute ATR remains controversial, including open surgery, minimally invasive surgery, or conservative treatment, especially when combined with early functional rehabilitation programs, which may achieve similar or equivalent clinical outcomes in terms of re-rupture rates and functional outcomes (44, 45). However, surgical treatment causes significantly more complications than conservative treatment, such as infections, deep venous thrombosis, peroneal nerve damage, and Achilles tendon adhesions (37, 46, 47). Therefore, these hot terms will continue to be discussed, and future clinical treatment studies will further expand on them.

## 5. Conclusion

From 2000 to 2021, the total number of publications and the annual production of studies on ATI/ATR increased. The US ranked first in the total number of papers, number of citations, and H-index and had the strongest scientific research level in this field. However, the research results from institutions and scholars in Europe are limited. The University of London, Queen Mary University London, and the University of Copenhagen were the main contributing institutions. Three of the top 10 were from the US, while the rest were from Europe. Maffulli N, Silbernagel KG, and Soslowsky LJ were the main contributors to this study. *Foot and Ankle International* and *American Journal of Sports Medicine* are major publications on ATI. Research by Kannus P, Cetti R, Willits K., and Wapner KL, among others, are the most influential in this field worldwide. ATI/ATR is divided into five main subfields: animal models, therapeutic, clinical, anatomical, and kinesiology. According to the latest research trends, it is predicted that basic research will focus on the application of stem cells and exosomes for Achilles tendon reconstruction, while clinical research will focus on the choice of surgical treatment, conservative treatment, and postoperative complications. Future research will continue in these popular research directions.

## Data Availability

The original contributions presented in the study are included in the article/Supplementary Material, further inquiries can be directed to the corresponding author/s.

## References

[1] Kauwe M. Acute achilles tendon rupture: clinical evaluation, conservative management, and early active rehabilitation. Clin Podiatr Med Surg. (2017) 34(2):229–43. 10.1016/j.cpm.2016.10.009 28257676

[2] Lantto I Lantto I Heikkinen J Flinkkila T Ohtonen P Leppilahti J Epidemiology of a chilles tendon ruptures: increasing incidence over a 33-year period. Scand J Med Sci Sports. (2015) 25(1):e133–8. 10.1111/sms.12253 24862178

[3] Manent A Lopez L Corominas H Santamaria A Dominguez A Llorens N Acute achilles tendon ruptures: efficacy of conservative and surgical (percutaneous, open) treatment—a randomized, controlled, clinical trial. J Foot Ankle Surg. (2019) 58(6):1229–34. 10.1053/j.jfas.2019.02.002 31679677

[4] Ellegaard O Wallin JA. The bibliometric analysis of scholarly production: how great is the impact? Scientometrics. (2015) 105(3):1809–31. 10.1007/s11192-015-1645-z 26594073 PMC4643120

[5] Liu Y Xu Y Cheng X Lin Y Jiang S Yu H Research trends and most influential clinical studies on anti-PD1/PDL1 immunotherapy for carcinomas: a bibliometric analysis. Front Immunol. (2022) 13:1631. doi: 10.3389/fimmu.2022.862084 PMC9044908 35493449

[6] Ding Y Chowdhury GG Foo S. Bibliometric cartography of information retrieval research by using co-word analysis. Inf Process Manag. (2001) 37(6):817–42. 10.1016/S0306-4573(00)00051-0

[7] Liu W Hu G Tang L Wang Y. China's global growth in social science research: uncovering evidence from bibliometric analyses of SSCI publications (1978–2013). J Informetr. (2015) 9(3):555–69. 10.1016/j.joi.2015.05.007

[8] Bornmann L Thor A Marx W Schier H. The application of bibliometrics to research evaluation in the humanities and social sciences: an exploratory study using normalized G oogle S cholar data for the publications of a research institute. J Assoc Inf Sci Technol. (2016) 67(11):2778–89. 10.1002/asi.23627

[9] Wu H Wang Y Tong L Yan H Sun Z. Global research trends of ferroptosis: a rapidly evolving field with enormous potential. Front Cell Dev Biol. (2021) 9:866. 10.3389/fcell.2021.646311 PMC8116802 33996807

[10] Balandin S Stancliffe RJ. Impact factors and the h-index: what researchers and readers need to know. Augment Altern Commun. (2009) 25(1):1–3. 10.1080/07434610902813549 19280418

[11] Shi S Gao Y Sun Y Liu M Shao L Zhang J The top-100 cited articles on biomarkers in the depression field: a bibliometric analysis. Psychol Health Med. (2021) 26(5):533–42. 10.1080/13548506.2020.1752924 32290683

[12] Kannus P Jozsa L. Histopathological changes preceding spontaneous rupture of a tendon. A controlled study of 891 patients. J Bone Joint Surg Am. (1991) 73(10):1507–25. 10.2106/00004623-199173100-00009 1748700

[13] Cetti R Christensen SE Ejsted R Jensen NM Jorgensen U. Operative versus nonoperative treatment of achilles tendon rupture: a prospective randomized study and review of the literature. Am J Sports Med. (1993) 21(6):791–9. 10.1177/036354659302100606 8291628

[14] Willits K Operative versus nonoperative treatment of acute achilles tendon ruptures: a multicenter randomized trial using accelerated functional rehabilitation. JBJS. (2010) 92(17):2767–75. 10.2106/JBJS.I.01401 21037028

[15] Wapner KL Pavlock GS Hecht PJ Naselli F Walther R. Repair of chronic achilles tendon rupture with flexor hallucis longus tendon transfer. Foot Ankle. (1993) 14(8):443–9. 10.1177/107110079301400803 8253436

[16] Egger AC Berkowitz MJ. Achilles tendon injuries. Curr Rev Musculoskelet Med. (2017) 10(1):72–80. 10.1007/s12178-017-9386-7 28194638 PMC5344857

[17] Raikin SM Garras DN Krapchev PV. Achilles tendon injuries in a United States population. Foot Ankle Int. (2013) 34(4):475–80. 10.1177/1071100713477621 23386750

[18] Hather GJ Haynes W Higdon R Kolker N Stewart EA Arzberger P The United States of America and scientific research. PLoS One. (2010) 5(8):e12203. 10.1371/journal.pone.0012203 20808949 PMC2922381

[19] Maffulli N Wong J Almekinders LC. Types and epidemiology of tendinopathy. Clin Sports Med. (2003) 22(4):675–92. 10.1016/S0278-5919(03)00004-8 14560540

[20] Maffulli N Longo UG Maffulli GD Rabitti C Khanna A Denaro V. Marked pathological changes proximal and distal to the site of rupture in acute achilles tendon ruptures. Knee Surg Sports Traumatol Arthrosc. (2011) 19(4):680–7. 10.1007/s00167-010-1193-2 20563556

[21] Maffulli N D'Addona A Gougoulias N Oliva F Maffulli GD. Ipsilateral free semitendinosus graft with interference screw fixation for surgical management of insertional acute achilles tendon tears. Injury. (2020) 51:S73–9. 10.1016/j.injury.2019.11.013 31761423

[22] Maffulli N Tallon C Wong J Lim KP Bleakney R. Early weightbearing and ankle mobilization after open repair of acute midsubstance tears of the achilles tendon. Am J Sports Med. (2003) 31(5):692–700. 10.1177/03635465030310051001 12975188

[23] Silbernagel KG Hanlon S Sprague A. Current clinical concepts: conservative management of achilles tendinopathy. J Athl Train. (2020) 55(5):438–47. 10.4085/1062-6050-356-19 32267723 PMC7249277

[24] Freedman BR Sarver JJ Buckley MR Voleti PB Soslowsky LJ. Biomechanical and structural response of healing achilles tendon to fatigue loading following acute injury. J Biomech. (2014) 47(9):2028–34. 10.1016/j.jbiomech.2013.10.054 24280564 PMC4017004

[25] Maffulli N Gougoulias N Maffulli GD Oliva F Migliorini F. Slowed-Down rehabilitation following percutaneous repair of achilles tendon rupture. Foot Ankle Int. (2022) 43(2):244–52. 10.1177/10711007211038594 34581220 PMC8841642

[26] Semis HS Gur C Ileriturk M Kandemir FM Kaynar O. Evaluation of therapeutic effects of quercetin against achilles tendinopathy in rats via oxidative stress, inflammation, apoptosis, autophagy, and metalloproteinases. Am J Sports Med. (2022) 50(2):486–98. 10.1177/03635465211059821 34908488

[27] de Vos RJ van der Vlist AC Zwerver J Meuffels DE Smithuis F van Ingen R Dutch Multidisciplinary guideline on achilles tendinopathy. Br J Sports Med. (2021) 55(20):1125–34. 10.1136/bjsports-2020-103867 34187784 PMC8479731

[28] Zhang C Zhang Y Zhong B Luo CF. SMAD 7 Prevents heterotopic ossification in a rat achilles tendon injury model via regulation of endothelial–mesenchymal transition. FEBS J. (2016) 283(7):1275–85. 10.1111/febs.13667 26807862

[29] Aufwerber S Naili JE Gravare Silbernagel K Ackermann PW. No effects of early functional mobilization on gait patterns after acute achilles tendon rupture repair. J Orthop Res. (2022) 40(8):1932–42. 10.1002/jor.25199 34674300

[30] Dirrichs T Quack V Gatz M Tingart M Kuhl CK Schrading S. Shear wave elastography (SWE) for the evaluation of patients with tendinopathies. Acad Radiol. (2016) 23(10):1204–13. 10.1016/j.acra.2016.05.012 27318786

[31] Prado-Costa R Rebelo J Monteiro-Barroso J Preto AS. Ultrasound elastography: compression elastography and shear-wave elastography in the assessment of tendon injury. Insights Imaging. (2018) 9(5):791–814. 10.1007/s13244-018-0642-1 30120723 PMC6206379

[32] Chamberlain CS Clements AEB Kink JA Choi U Baer GS Halanski MA Extracellular vesicle-educated macrophages promote early achilles tendon healing. Stem Cells. (2019) 37(5):652–62. 10.1002/stem.2988 30720911 PMC6850358

[33] Xu T Lin Y Yu X Jiang G Wang J Xu K Comparative effects of exosomes and ectosomes isolated from adipose-derived mesenchymal stem cells on achilles tendinopathy in a rat model. Am J Sports Med. (2022) 50(10):2740–52. 10.1177/03635465221108972 35867349

[34] Zhang C Yuan H Liu H Chen X Lu P Zhu T Well-aligned chitosan-based ultrafine fibers committed teno-lineage differentiation of human induced pluripotent stem cells for achilles tendon regeneration. Biomaterials. (2015) 53:716–30. 10.1016/j.biomaterials.2015.02.051 25890767

[35] de Vos RJ Weir A van Schie HT Bierma-Zeinstra SM Verhaar JA Weinans H Platelet-rich plasma injection for chronic achilles tendinopathy: a randomized controlled trial. JAMA. (2010) 303(2):144–9. 10.1001/jama.2009.1986 20068208

[36] Jiang N Wang B Chen A Dong F Yu B. Operative versus nonoperative treatment for acute achilles tendon rupture: a meta-analysis based on current evidence. Int Orthop. (2012) 36(4):765–73. 10.1007/s00264-011-1431-3 22159659 PMC3311794

[37] Soroceanu A Sidhwa F Aarabi S Kaufman A Glazebrook M. Surgical versus nonsurgical treatment of acute achilles tendon rupture: a meta-analysis of randomized trials. J Bone Joint Surg Am. (2012) 94(23):2136. 10.2106/JBJS.K.00917 23224384 PMC3509775

[38] Wilkins R Bisson LJ. Operative versus nonoperative management of acute achilles tendon ruptures: a quantitative systematic review of randomized controlled trials. Am J Sports Med. (2012) 40(9):2154–60. 10.1177/0363546512453293 22802271

[39] Maffulli N Spiezia F Testa V Capasso G Longo UG Denaro V. Free gracilis tendon graft for reconstruction of chronic tears of the achilles tendon. JBJS. (2012) 94(10):906–10. 10.2106/JBJS.K.00869 22617918

[40] Marx RG Hetsroni I. Surgical technique: medial collateral ligament reconstruction using achilles allograft for combined knee ligament injury. Clin Orthop Relat Res. (2012) 470(3):798–805. 10.1007/s11999-011-1941-8 21660595 PMC3270177

[41] Kolodziej L Bohatyrewicz A Kromuszczynska J Jezierski J Biedron M. Efficacy and complications of open and minimally invasive surgery in acute achilles tendon rupture: a prospective randomised clinical study—preliminary report. Int Orthop. (2013) 37(4):625–9. 10.1007/s00264-012-1737-9 23250350 PMC3609980

[42] Agarwal S Loder S Cholok D Li J Breuler C Drake J Surgical excision of heterotopic ossification leads to re-emergence of mesenchymal stem cell populations responsible for recurrence. Stem Cells Transl Med. (2017) 6(3):799–806. 10.5966/sctm.2015-0365 28297577 PMC5442786

[43] Zhang K Asai S Hast MW Liu M Usami Y Iwamoto M Tendon mineralization is progressive and associated with deterioration of tendon biomechanical properties, and requires BMP-smad signaling in the mouse achilles tendon injury model. Matrix Biol. (2016) 52:315–24. 10.1016/j.matbio.2016.01.015 26825318 PMC4875838

[44] She G Teng Q Li J Zheng X Chen L Hou H. Comparing surgical and conservative treatment on achilles tendon rupture: a comprehensive meta-analysis of RCTs. Front Surg. (2021) 8:607743. 10.3389/fsurg.2021.607743 33681281 PMC7931800

[45] Ochen Y Beks RB van Heijl M Hietbrink F Leenen LPH van der Velde D Operative treatment versus nonoperative treatment of achilles tendon ruptures: systematic review and meta-analysis. Br Med J. (2019) 364. Aticle ID: . 10.1136/bmj.k5120 30617123 PMC6322065

[46] Khan RJ Smith RLC. Surgical interventions for treating acute achilles tendon ruptures. Cochrane Database Syst Rev. (2010) (9):CD003674. 10.1002/14651858.cd003674.pub4 20824836

[47] Muller SA Evans CH Heisterbach PE Majewski M. The role of the paratenon in achilles tendon healing: a study in rats. Am J Sports Med. (2018) 46(5):1214–9. 10.1177/0363546518756093 29505741

