# Supplementary material for: Global trends in research of achilles tendon injury/rupture: A bibliometric analysis, 2000–2021
Source: Front Surg. 2023 Mar 27;10:1051429. doi: 10.3389/fsurg.2023.1051429 (PMC10083236; doi:10.3389/fsurg.2023.1051429)
Supplement: Supplementary file 3 [file Datasheet2.pdf]

editage

Editing Certificate

This document certifies that the manuscript listed below has been edited to ensure language and grammar accuracy and is error free in these aspects. The logical presentation of ideas and the structure of the paper were also checked during the editing process. The edit was performed by professional editors at Editage, a division of Cactus Communications. The author's core research ideas were not altered in any way during the editing process. The quality of the edit has been guaranteed, with the assumption that our suggested changes have been accepted and the text has not been further altered without the knowledge of our editors.

MANUSCRIPT TITLE

Global Trends in Research of Achilles Tendon Injury/Rupture: A Bibliometric Analysis, 2000–2021

AUTHORS

Chenguang Wang, Zhaohui Jiang, Ran Pang Huafeng Zhang, Hui Li, Zhijun Li

ISSUED ON

February 13, 2023

JOB CODE

FNHMI_1

THE EDITAGE Promise QUALITY GUARANTEE

Vikas Narang

Vikas Narang
Chief Operating Officer - Editage

editage

Editage, a brand of Cactus Communications, offers professional English language editing and publication support services to authors engaged in over 500 areas of research. Through its community of experienced editors, which includes doctors, engineers, published scientists, and researchers with peer review experience, Editage has successfully helped authors get published in internationally reputed journals. Authors who work with Editage are guaranteed excellent language quality and timely delivery.

GLOBAL :
+1(833) 979-0061 | request@editage.com
