# Supplementary material for: Global trends in research of achilles tendon injury/rupture: A bibliometric analysis, 2000–2021
Source: Front Surg. 2023 Mar 27;10:1051429. doi: 10.3389/fsurg.2023.1051429 (PMC10083236; doi:10.3389/fsurg.2023.1051429)
Supplement: Supplementary file 1 [file Table1.docx]

```
FN Clarivate Analytics Web of Science
VR 1.0
PT J
AU Kraemer, R
   Wuerfel, W
   Lorenzen, J
   Busche, M
   Vogt, PM
   Knobloch, K
AF Kraemer, Robert
   Wuerfel, Waldemar
   Lorenzen, Johan
   Busche, Marc
   Vogt, Peter M.
   Knobloch, Karsten
TI Analysis of hereditary and medical risk factors in Achilles tendinopathy
   and Achilles tendon ruptures: a matched pair analysis
SO ARCHIVES OF ORTHOPAEDIC AND TRAUMA SURGERY
LA English
DT Article
DE Achilles tendon injury; Risk factors; Family history; Achilles
   tendinopathy; Achilles tendon rupture
ID INJURIES; EPIDEMIOLOGY; GENE
AB In Achilles tendon injuries, it is suggested that a pathological continuum might be evident from the healthy Achilles tendon to Achilles tendinopathy to Achilles tendon rupture. As such, risk factors for both tendinopathy and rupture should be the same.
   Hereditary and medical risk factors for Achilles tendinopathy and Achilles tendon rupture are the same to a similar extent in a matched pair analysis.
   Matched pair study; level of evidence: 3.
   Recreational sportsmen as well as athletes on national level.
   566 questionnaires were analysed. 310 subjects were allocated to 3 groups (A, B, C) after matching the pairs for age, weight, height and gender: (A) healthy Achilles tendons ( = 89, age 39 +/- A 11 years, BMI 25.1 +/- A 3.9, females 36%), (B) chronic Achilles tendinopathy ( = 161, age 41 +/- A 11 years, BMI 24.4 +/- A 3.7, females 34%), (C) acute Achilles tendon rupture ( = 60, age 40 +/- A 9 years, BMI 25.2 +/- A 3.2, females 27%).
   We found a positive family history of Achilles tendinopathy as a risk factor for Achilles tendinopathy (OR: 4.8, 95% CI: 1.1-21.4; = 0.023), but not for Achilles tendon rupture (OR: 4.0, 95% CI 0.7-21.1, = 0.118). Smoking and cardiac diseases had a lower incidence in Achilles tendinopathy than in healthy subjects (both = 0.001), while cardiovascular medication did not change the risk profile.
   Identifying risk factors associated with Achilles tendon disorders has a high clinical relevance regarding the development and implementation of prevention strategies and programs. This cross-sectional study identified a positive family history as a significant solitary risk factor for Achilles tendinopathy, increasing the risk fivefold. However, in this matched pair analysis excluding age, weight, height and gender as risk factors no further factor necessarily increases the risk for either Achilles tendinopathy or Achilles tendon rupture.
C1 [Kraemer, Robert; Wuerfel, Waldemar] Hannover Med Sch, Dept Otolaryngol, D-30625 Hannover, Germany.
   [Lorenzen, Johan] Hannover Med Sch, Dept Nephrol, D-30625 Hannover, Germany.
RP Kraemer, R (通讯作者)，Hannover Med Sch, Dept Otolaryngol, Carl Neuberg Str 1, D-30625 Hannover, Germany.
EM Kraemer.Robert@mh-hannover.de
RI Knobloch, Karsten/AAI-3312-2020; Busche, Marc/AAS-3845-2021; Vogt, Peter
   M./AAL-1332-2020
OI Knobloch, Karsten/0000-0003-1468-4258; Lorenzen,
   Johan/0000-0002-9132-948X
CR Beeharry D, 2006, ANN RHEUM DIS, V65, P312, DOI 10.1136/ard.2005.040766
   Collins M, 2010, BRIT J SPORT MED, V44, P1063, DOI 10.1136/bjsm.2008.056184
   de Jonge S, 2011, BRIT J SPORT MED, V45, P1026, DOI 10.1136/bjsports-2011-090342
   Gaida JE, 2010, BMC MUSCULOSKEL DIS, V11, DOI 10.1186/1471-2474-11-41
   Gaida JE, 2009, MED SCI SPORT EXER, V41, P1194, DOI 10.1249/MSS.0b013e31819794c3
   HOLMES GB, 1992, FOOT ANKLE, V13, P70, DOI 10.1177/107110079201300204
   Holmes GB, 2006, FOOT ANKLE INT, V27, P952, DOI 10.1177/107110070602701115
   Knobloch K, 2008, FOOT ANKLE INT, V29, P671, DOI 10.3113/FAI.2008.0671
   LEES JF, 1994, J BIOL CHEM, V269, P24354
   Longo UG, 2009, AM J SPORT MED, V37, P1400, DOI 10.1177/0363546509332250
   Mokone GG, 2005, AM J SPORT MED, V33, P1016, DOI 10.1177/0363546504271986
   Parekh Selene G, 2009, Foot Ankle Spec, V2, P283, DOI 10.1177/1938640009351138
   Sankey RA, 2008, AM J SPORT MED, V36, P2415, DOI 10.1177/0363546508322889
   September AV, 2009, BRIT J SPORT MED, V43, P357, DOI 10.1136/bjsm.2008.048793
   Smithard A, 2007, SKELETAL RADIOL, V36, P171, DOI 10.1007/s00256-006-0139-8
   Sode J, 2007, EUR J CLIN PHARMACOL, V63, P499, DOI 10.1007/s00228-007-0265-9
   Tallon C, 2001, MED SCI SPORT EXER, V33, P1983, DOI 10.1097/00005768-200112000-00002
   Taunton JE, 2002, BRIT J SPORT MED, V36, P95, DOI 10.1136/bjsm.36.2.95
   Vandenbroucke JP, 2007, ANN INTERN MED, V147, pW163, DOI 10.7326/0003-4819-147-8-200710160-00010-w1
   VANMECHELEN W, 1992, SPORTS MED, V14, P82, DOI 10.2165/00007256-199214020-00002
NR 20
TC 29
Z9 30
U1 1
U2 22
PU SPRINGER
PI NEW YORK
PA 233 SPRING ST, NEW YORK, NY 10013 USA
SN 0936-8051
EI 1434-3916
J9 ARCH ORTHOP TRAUM SU
JI Arch. Orthop. Trauma Surg.
PD JUN
PY 2012
VL 132
IS 6
BP 847
EP 853
DI 10.1007/s00402-012-1476-9
PG 7
WC Orthopedics; Surgery
WE Science Citation Index Expanded (SCI-EXPANDED)
SC Orthopedics; Surgery
GA 944ND
UT WOS:000304209500015
PM 22350054
DA 2022-07-22
ER

PT J
AU Park, HG
   Youn, D
   Baik, JM
   Hwang, JH
AF Park, Hong-Gi
   Youn, Dukyoung
   Baik, Jong-Min
   Hwang, Jae Ho
TI Epidemiology of Achilles Tendon Rupture in South Korea: Claims Data of
   the National Health Insurance Service from 2009 to 2017
SO CLINICS IN ORTHOPEDIC SURGERY
LA English
DT Article
DE Achilles tendon; Tendon injuries; Rupture; Incidence
ID INCREASING INCIDENCE; METAANALYSIS
AB Background: The incidence of Achilles tendon rupture and its trend has not been studied in Asia. The purpose of this nationwide study was to analyze the trend of incidence and surgical treatment of tendon ruptures in South Korea based on sex, age, and income level of patients, as well as seasonal variation. Methods: A descriptive epidemiologic study was performed based on the data collected retrospectively from the Korea National Health Insurance Service. Data of all outpatients and inpatients were collected from approximately 52 million residents of South Korea, primarily diagnosed with Achilles tendon rupture from 2009 to 2017. Results: A total of 112,350 patients had Achilles tendon rupture, of which 44,248 patients underwent surgical treatment during the study period. The overall, age-specific, and sex-specific incidence of Achilles tendon rupture and surgical treatment showed an increasing trend. Patients in the age group of 41 to 50 years showed the highest increase in incidence. Regarding season, higher incidence was reported during spring and summer, whereas the lowest incidence was found in winter. Higher income level was associated with increased incidence of the condition. Conclusions: The incidence of Achilles tendon rupture and surgical treatments increased rapidly in patients between 41 and 50 years of age. Patients in the higher income quintile groups experienced more Achilles tendon injury than those in lower income groups, and fewer ruptures were observed during winter.
C1 [Park, Hong-Gi; Baik, Jong-Min; Hwang, Jae Ho] Gachon Univ, Coll Med, Gil Med Ctr, Dept Orthopaed Surg, 21 Namdong Daero 774beon Gil, Incheon 21565, South Korea.
   [Youn, Dukyoung] Samsung Bioepis, Incheon, South Korea.
RP Baik, JM (通讯作者)，Gachon Univ, Coll Med, Gil Med Ctr, Dept Orthopaed Surg, 21 Namdong Daero 774beon Gil, Incheon 21565, South Korea.
EM bbaik@hanmail.net
OI Baik, Jong-Min/0000-0002-6646-2449
CR Bartel AFP, 2014, J FOOT ANKLE SURG, V53, P744, DOI 10.1053/j.jfas.2014.07.009
   CROLLA R M P H, 1987, Acta Orthopaedica Belgica, V53, P492
   El-Akkawi AI, 2018, J FOOT ANKLE SURG, V57, P346, DOI 10.1053/j.jfas.2017.06.006
   Ganestam A, 2016, KNEE SURG SPORT TR A, V24, P3730, DOI 10.1007/s00167-015-3544-5
```

김석규, 2019, [Journal of Korean Physical Education Association for Girls and Women, 한국여성체육학회지], V33, P129

Hess Gregory William, 2010, Foot Ankle Spec, V3, P29, DOI 10.1177/1938640009355191

Ho G, 2017, INJURY, V48, P1701, DOI 10.1016/j.injury.2017.04.007

Houshian S, 1998, INJURY, V29, P651, DOI 10.1016/S0020-1383(98)00147-8

Huttunen TT, 2014, AM J SPORT MED, V42, P2419, DOI 10.1177/0363546514540599

Jarvinen Tero A H, 2005, Foot Ankle Clin, V10, P255, DOI 10.1016/j.fcl.2005.01.013

Kim IG, 2014, J PHYS THER SCI, V26, P1887, DOI 10.1589/jpts.26.1887

Korean Statistical Information Service, 2014, STAT DAT EXCH RAT KO

Lantto I, 2015, SCAND J MED SCI SPOR, V25, pE133, DOI 10.1111/sms.12253

Lemme NJ, 2018, ORTHOP J SPORTS MED, V6, DOI 10.1177/2325967118808238

Moller A, 1996, ACTA ORTHOP SCAND, V67, P479, DOI 10.3109/17453679608996672

Moller M, 2002, SCAND J MED SCI SPOR, V12, P9, DOI 10.1034/j.1600-0838.2002.120103.x

NILLIUS SA, 1976, ACTA ORTHOP SCAND, V47, P118, DOI 10.3109/17453677608998983

Nilsson-Helander K, 2010, AM J SPORT MED, V38, P2186, DOI 10.1177/0363546510376052

Nyyssonen T, 2008, SCAND J SURG, V97, P272, DOI 10.1177/145749690809700312

Ochen Y, 2019, BMJ-BRIT MED J, V364, DOI 10.1136/bmj.k5120

이찬희, 2014, [Health Policy and Mangemnet, 보건행정학회지], V24, P254, DOI 10.4332/KJHPA.2014.24.3.254

Raikin SM, 2013, FOOT ANKLE INT, V34, P475, DOI 10.1177/1071100713477621

Song SO, 2014, DIABETES METAB J, V38, P395, DOI 10.4093/dmj.2014.38.5.395

Soroceanu A, 2012, J BONE JOINT SURG AM, V94A, P2136, DOI 10.2106/JBJS.K.00917

Suchak AA, 2005, FOOT ANKLE INT, V26, P932, DOI 10.1177/107110070502601106

Tuite DJ, 1997, SCAND J MED SCI SPOR, V7, P72

Yasui Y, 2017, BIOMED RES INT, V2017, DOI 10.1155/2017/7021862

김준범, 2019, [journal of korean foot and ankle society, 대한족부족관절학회지], V23, P83

```
   Zhou K, 2018, J FOOT ANKLE SURG, V57, P1191, DOI 10.1053/j.jfas.2018.05.007
NR 29
TC 0
Z9 0
U1 0
U2 0
PU KOREAN ORTHOPAEDIC ASSOC
PI SEOUL
PA #1111 LIFECOMBI BLDG, YOEIDO-DONG, YOUNGDEUNGPO-GU, SEOUL, 150-732,
   SOUTH KOREA
SN 2005-291X
EI 2005-4408
J9 CLIN ORTHOP SURG
JI Clin. Orthop. Surg.
PD DEC
PY 2021
VL 13
IS 4
BP 539
EP 548
DI 10.4055/cios20255
PG 10
WC Orthopedics
WE Science Citation Index Expanded (SCI-EXPANDED)
SC Orthopedics
GA XD4PU
UT WOS:000722693900015
PM 34868504
OA gold, Green Published
DA 2022-07-22
ER

PT J
AU Corradino, B
   Di Lorenzo, S
   Calamia, C
   Moschella, F
AF Corradino, B.
   Di Lorenzo, S.
   Calamia, C.
   Moschella, F.
TI Surgical repair of acute Achilles tendon rupture with an end-to-end
   tendon suture and tendon flap
SO INJURY-INTERNATIONAL JOURNAL OF THE CARE OF THE INJURED
LA English
DT Article
DE Achilles tendon rupture; Surgical treatment of tendon rupture; Achilles
   tendon injury
AB Background: Achilles tendon ruptures are becoming more common. Complications after open or minimally invasive surgery are: recurrent rupture (2-8%), wound breakdown, deep infections, granuloma, and fistulas. The authors expose their experience with a personal technique.
   Materials: In 8 patients with acute rupture of Achilles tendon the surgery was performed at least 25 days after trauma. Clinical exam and MR demonstrated in all case a total lesion of tendon. After a posterolateral skin incision the tendon stumps were debrided and suture in end-to-end fashion. A tendon flap was harvested from the proximal part of the tendon, in order to protect and reinforce the suture itself. A plaster cast was applied for 3 weeks and the patients started the rehabilitation protocol.
   Results: After 4 months all patients returned to pre-injury daily activities. The mean follow up was 13 months (ranged between 6 and 24 months). No major complications occurred.
   Conclusion: The posterolateral skin incision, not above the tendon, preserves the vascularity of the soft tissues, allows identifying and not accidentally injuring the sural nerve, and prevents the cutaneous scar is overlapped the tendon. In this way is favoured physiological tendon sliding.
   The preparation of the flap tendon does not weaken the overall strength of the tendon and protects the tendon suture. The tension on sutured stumps is less than being spread over a larger area.
   In our sample of 8 patients the absence of short-and long-term complications and the rapid functional recovery after surgery suggest that the technique used is safe and effective. (C) 2015 Elsevier Ltd. All rights reserved.
C1 [Corradino, B.; Di Lorenzo, S.; Calamia, C.; Moschella, F.] Univ Palermo, Dipartimento Discipline Chirurg Oncol & Stomatol, Sez Chirurg Plast, I-90129 Palermo, Italy.
RP Corradino, B (通讯作者)，Univ Palermo, Dipartimento Discipline Chirurg Oncol & Stomatol, Sez Chirurg Plast, Via Vespro 127, I-90129 Palermo, Italy.
EM bartolo.corradino@unipa.it; dilsister@libero.it
CR Branch JP, 2011, J FOOT ANKLE SURG, V50, P257, DOI 10.1053/j.jfas.2010.12.015
   Gigante A, 2008, KNEE SURG SPORT TR A, V16, P204, DOI 10.1007/s00167-007-0448-z
   Massoud Elsayed Ibraheem Elsayed, 2011, Foot Ankle Surg, V17, P131, DOI 10.1016/j.fas.2010.03.002
   Porter KJ, 2014, FOOT ANKLE SURG, V20, P90, DOI 10.1016/j.fas.2013.11.005
   Strauss EJ, 2007, INJURY, V38, P832, DOI 10.1016/j.injury.2006.06.005
   Takao M, 2003, ARCH ORTHOP TRAUM SU, V123, P471, DOI 10.1007/s00402-002-0443-2
   Winter E, 1998, ARCH ORTHOP TRAUM SU, V117, P364, DOI 10.1007/s004020050267
NR 7
TC 8
Z9 13
U1 0
U2 3
PU ELSEVIER SCI LTD
PI OXFORD
PA THE BOULEVARD, LANGFORD LANE, KIDLINGTON, OXFORD OX5 1GB, OXON, ENGLAND
SN 0020-1383
EI 1879-0267
J9 INJURY
JI Injury-Int. J. Care Inj.
PD AUG
PY 2015
VL 46
IS 8
BP 1637
EP 1640
DI 10.1016/j.injury.2015.05.014
PG 4
WC Critical Care Medicine; Emergency Medicine; Orthopedics; Surgery
WE Science Citation Index Expanded (SCI-EXPANDED)
SC General & Internal Medicine; Emergency Medicine; Orthopedics; Surgery
GA CS2IR
UT WOS:000361894200037
PM 26093961
DA 2022-07-22
ER

PT J
AU Hollenberg, GM
   Adams, MJ
   Weinberg, WP
AF Hollenberg, GM
   Adams, MJ
   Weinberg, WP
TI Sonographic appearance of nonoperatively treated Achilles tendon
   ruptures
SO SKELETAL RADIOLOGY
LA English
DT Article
DE Achilles tendon; rupture; tendon injuries; ultrasonography; Doppler,
   color
ID DOPPLER SONOGRAPHY
AB Objective. This report describes the gray-scale and color Doppler sonographic appearance of healed Achilles tendon ruptures that have been treated nonoperatively.
   Design and patients. Eleven patients with acute Achilles tendon ruptures were studied with sonography in the acute setting and following nonoperative management of their torn tendon.
   Results. On sonographic examination, healed tendons treated nonoperatively are characterized by mild residual distortion of the normal fibrillar architecture of the tendon, anterior bulging or irregularity of the healed tendon, and a hypoechoic area about the site of rupture. Less frequent observations include mildly increased color flow and calcification at the rupture site. The previous ly described findings of acute tears, including hematomas, gaps at the rupture site, hyperemic torn tendon ends, and markedly distorted fibrillar architecture, were seen to have resolved in this series.
   Conclusion. The Achilles tendon tear treated nonoperatively has a different sonographic appearance from that of a normal or acutely ruptured tendon.
C1 Univ Med Imaging, Rochester, NY 14618 USA.
   Univ Rochester, Sch Med & Dent, Dept Radiol, Rochester, NY 14642 USA.
RP Hollenberg, GM (通讯作者)，Univ Med Imaging, 2180 S Clinton Ave,Suite B, Rochester, NY 14618 USA.
CR Astrom M, 1996, SKELETAL RADIOL, V25, P615, DOI 10.1007/s002560050146
   CETTI R, 1993, AM J SPORT MED, V21, P791, DOI 10.1177/036354659302100606
   COTRAN RS, 1994, ROBBINS PATHOLOGIC B, P79
   EVARTS CM, 1990, SURG MUSCULOSKELETAL, P3915
   FORNAGE BD, 1986, RADIOLOGY, V159, P759, DOI 10.1148/radiology.159.3.3517959
   HAGGMARK T, 1986, ORTHOPEDICS, V9, P160
   HOLLENBERG GM, 1998, EMERG RADIOL, V5, P317
   McComis GP, 1997, J BONE JOINT SURG AM, V79A, P1799, DOI 10.2106/00004623-199712000-00005
   NEWMAN JS, 1994, AM J ROENTGENOL, V163, P385, DOI 10.2214/ajr.163.2.8037037
   OREILLY MAR, 1993, CLIN RADIOL, V48, P202, DOI 10.1016/S0009-9260(05)80284-3
   THERMANN H, 1992, ORTHOP T, V16, P729
   Weinberg EP, 1998, AM J ROENTGENOL, V171, P743, DOI 10.2214/ajr.171.3.9725308
NR 12
TC 19
Z9 19
U1 0
U2 1
PU SPRINGER VERLAG
PI NEW YORK
PA 175 FIFTH AVE, NEW YORK, NY 10010 USA
SN 0364-2348
J9 SKELETAL RADIOL
JI Skeletal Radiol.
PD MAY
PY 2000
VL 29
IS 5
BP 259
EP 264
DI 10.1007/s002560050604
PG 6
WC Orthopedics; Radiology, Nuclear Medicine & Medical Imaging
WE Science Citation Index Expanded (SCI-EXPANDED)
SC Orthopedics; Radiology, Nuclear Medicine & Medical Imaging
GA 321WM
UT WOS:000087477700002
PM 10883444
DA 2022-07-22
ER

PT J
AU Biggs, A
   Scott, G
   Solan, MC
   Williamson, M
AF Biggs, A.
   Scott, G.
   Solan, M. C.
   Williamson, M.
TI Achilles tendon rupture: what you need to know
SO BRITISH JOURNAL OF HOSPITAL MEDICINE
LA English
DT Article
DE Achilles tendon; Ankle injuries; Rupture; Tendon injuries; Venous
   thrombosis
ID RANDOMIZED CONTROLLED-TRIAL; MANAGEMENT; METAANALYSIS; OUTCOMES; CAST
AB Heel pain and a history of a 'pop' or feeling 'something go' are the buzz phrases classically associated with Achilles tendon rupture. However, the diagnosis is often missed in clinical practice because of the assumption that this is a sporting injury suffered only by the young or middle-aged. In a sedentary older patient, the injury may be dismissed as an ankle sprain. If swelling is present but no injury is recalled then deep vein thrombosis is suspected, but Achilles rupture is not. The diagnosis of Achilles tendon rupture is clinical, based on history and examination. Radiological imaging (ultrasound scan) is useful to plan orthopaedic management and exclude concomitant deep vein thrombosis. In most cases, non-operative management with the ankle held plantar flexed in a boot is the current best practice.
C1 [Biggs, A.; Scott, G.; Solan, M. C.; Williamson, M.] Royal Surrey Hosp, Dept Trauma & Orthopaed, Guildford, Surrey, England.
RP Biggs, A (通讯作者)，Royal Surrey Hosp, Dept Trauma & Orthopaed, Guildford, Surrey, England.
EM a.biggs@nhs.net
CR APLEY AG, 1966, P ROY SOC MED, V59, P117, DOI 10.1177/003591576605900212
   Bleakney Robert R, 2005, Foot Ankle Clin, V10, P239, DOI 10.1016/j.fcl.2005.01.006
   Boyd RPR, 2015, BRIT J GEN PRACT, V65, P668, DOI 10.3399/bjgp15X688069
   CARDEN DG, 1987, J BONE JOINT SURG BR, V69, P416, DOI 10.1302/0301-620X.69B3.3294839
   Costa ML, 2020, LANCET, V395, P441, DOI 10.1016/S0140-6736(19)32942-3
   Dakin SG, 2018, BRIT J SPORT MED, V52, P359, DOI 10.1136/bjsports-2017-098161
   Deng SL, 2017, J FOOT ANKLE SURG, V56, P1236, DOI 10.1053/j.jfas.2017.05.036
   Domeij-Arverud E, 2016, BONE JOINT J, V98B, P1635, DOI 10.1302/0301-620X.98B12.BJJ-2016-0008.R1
   Edwards G, 2020, BMJ-BRIT MED J, V368, DOI 10.1136/bmj.m54
   Eriksen HA, 2002, J ORTHOPAED RES, V20, P1352, DOI 10.1016/S0736-0266(02)00064-5
   Garras DN, 2012, CLIN ORTHOP RELAT R, V470, P2268, DOI 10.1007/s11999-012-2355-y
   Healy B, 2010, J BONE JOINT SURG BR, V92B, P646, DOI 10.1302/0301-620X.92B5.23241
   Hess Gregory William, 2010, Foot Ankle Spec, V3, P29, DOI 10.1177/1938640009355191
   Hutchison AM, 2015, BONE JOINT J, V97B, P510, DOI 10.1302/0301-620X.97B4.35314
   JOZSA L, 1989, AM J SPORT MED, V17, P338, DOI 10.1177/036354658901700305
   Khan RJK, 2005, J BONE JOINT SURG AM, V87A, P2202, DOI 10.2106/JBJS.D.03049
   Lantto I, 2016, AM J SPORT MED, V44, P2406, DOI 10.1177/0363546516651060
   Lawrence JE, 2017, BONE JOINT J, V99B, P87, DOI 10.1302/0301-620X.99B1.BJJ-2016-0452.R1
   Leppilahti J, 1998, SPORTS MED, V25, P79, DOI 10.2165/00007256-199825020-00002
   Maempel JF, 2020, BONE JOINT J, V102B, P933, DOI 10.1302/0301-620X.102B7.BJJ-2019-0783.R3
   Nilsson-Helander K, 2009, KNEE SURG SPORT TR A, V17, P1234, DOI 10.1007/s00167-009-0727-y
   Ring J, 2014, BONE JOINT J, V96B, P1510, DOI 10.1302/0301-620X.96B11.33963
   Sheth U, 2017, BONE JOINT J, V99B, P1629, DOI 10.1302/0301-620X.99B12.BJJ-2017-0465.R1
   Sheth U, 2017, BONE JOINT J, V99B, P78, DOI [10.1302/0301-620X.99B1.BJJ-2016-0434.R1, 10.1302/0301-620x.99b1.bjj-2016-0434.r1]
   SIMMONDS F A, 1957, Practitioner, V179, P56
   Suchak AA, 2005, FOOT ANKLE INT, V26, P932, DOI 10.1177/107110070502601106
   THOMPSON T C, 1962, J Trauma, V2, P126, DOI 10.1097/00005373-196203000-00003
   van der Linden PD, 2003, ARCH INTERN MED, V163, P1801, DOI 10.1001/archinte.163.15.1801
   Willits K, 2010, J BONE JOINT SURG AM, V92A, P2767, DOI 10.2106/JBJS.I.01401
NR 29
TC 0
Z9 0
U1 1
U2 6
PU MA HEALTHCARE LTD
PI LONDON
PA ST JUDES CHURCH, DULWICH ROAD, LONDON SE24 0PB, ENGLAND
SN 1750-8460
EI 1759-7390
J9 BRIT J HOSP MED
JI Br. J. Hosp. Med.
PD FEB 2
PY 2021
VL 82
IS 2
DI 10.12968/hmed.2020.0593
PG 7
WC Medicine, General & Internal
WE Science Citation Index Expanded (SCI-EXPANDED)
SC General & Internal Medicine
GA QR7BN
UT WOS:000625370200013
PM 33646025
DA 2022-07-22
ER

PT J
AU Zietek, P
   Karaczun, M
   Kruk, B
   Szczypior, K
AF Zietek, Pawel
   Karaczun, Maciej
   Kruk, Bartosz
   Szczypior, Karina
TI Percutaneous, Minimally Invasive Repair of Traumatic and Simultaneous
   Rupture of Both Achilles Tendons: A Case Report
SO JOURNAL OF FOOT & ANKLE SURGERY
LA English
DT Article
DE Achilles tendon rupture; percutaneous repair; tendon injury; trauma
ID SINGLE-BLIND; REHABILITATION; SURGERY
AB Achilles injury is a common musculoskeletal disorder. Bilateral rupture of the Achilles tendon, however, is much less common and usually occurs spontaneously. Complete, traumatic, and bilateral ruptures are rare and typically require long periods of immobilization before the patient can return to full weightbearing. A 52-year-old male was hospitalized for bilateral traumatic rupture to both Achilles tendons. No risk factors for tendon rupture were found. Blood samples revealed no peripheral blood pathologic features. Both tendons were repaired with percutaneous, minimally invasive surgery using the Achillon (R) tendon suture system. Rehabilitation was begun 4 weeks later. An ankle-foot orthosis was prescribed to provide ankle support with an adjustable range of movement, and active plantar flexion was set at 0 degrees to 30 degrees. The patient remained non-weightbearing with the ankle-foot orthosis device and performed active range-of-motion exercises. At 8 weeks after surgery, we recommended that he begin walking with partial weightbearing using a foot-tibial orthosis with the range of motion set to 45 degrees plantar flexion and 15 degrees dorsiflexion. At 10 weeks postoperatively, he was encouraged to return to full weightbearing on both feet. Beginning rehabilitation as soon as possible after minimally invasive surgery, compared with 6 weeks of immobilization after surgery, provided a rapid resumption to full weightbearing. We emphasize the clinical importance of a safe, simple treatment program that can be followed for a patient with damage to the Achilles tendons. To our knowledge, ours is the first report of minimally invasive repair of bilateral simultaneous traumatic rupture of the Achilles tendon. (C) 2016 by the American College of Foot and Ankle Surgeons. All rights reserved.
C1 [Zietek, Pawel; Karaczun, Maciej; Kruk, Bartosz; Szczypior, Karina] Pomeranian Med Univ, Dept Orthopaed & Traumatol, Szczecin, Poland.
RP Zietek, P (通讯作者)，SPSK1, Klin Ortopedii, Ul Unii Lubelskiej 1, PL-71252 Szczecin, Poland.
EM paulz@wp.pl
OI Zietek, Pawel/0000-0002-2967-6376
CR Assal M, 2002, J BONE JOINT SURG AM, V84A, P161, DOI 10.2106/00004623-200202000-00001
   Audenaert EA, 2004, J FOOT ANKLE SURG, V10, P49
   Basic-Jukic N, 2009, KIDNEY BLOOD PRESS R, V32, P32, DOI 10.1159/000201792
   Calder JDF, 2005, BRIT J SPORT MED, V39, P857, DOI 10.1136/bjsm.2004.017509
   Cretnik A, 2004, WIEN KLIN WOCHENSCHR, V116, P33
   Ding WG, 2013, J FOOT ANKLE SURG, V52, P143, DOI 10.1053/j.jfas.2012.12.006
   Garofalo R, 2004, AM J SPORT MED, V32, P1778, DOI 10.1177/0363546503262647
   Garrick James G, 2012, Clin J Sport Med, V22, P379, DOI 10.1097/JSM.0b013e3182603905
   Gigante A, 2008, KNEE SURG SPORT TR A, V16, P204, DOI 10.1007/s00167-007-0448-z
   HABUSTA SF, 1995, CLIN ORTHOP RELAT R, P231
   HAINES JF, 1983, ANN RHEUM DIS, V42, P652, DOI 10.1136/ard.42.6.652
   Henriquez H, 2012, CLIN ORTHOP RELAT R, V470, P998, DOI 10.1007/s11999-011-1830-1
   Houshian S, 1998, INJURY, V29, P651, DOI 10.1016/S0020-1383(98)00147-8
   Ibrahim Talal, 2007, J Foot Ankle Surg, V46, P65, DOI 10.1053/j.jfas.2006.12.002
   Jiang N, 2012, INT ORTHOP, V36, P765, DOI 10.1007/s00264-011-1431-3
   Kearney RS, 2012, J BONE JOINT SURG BR, V94B, P28, DOI 10.1302/0301-620X.94B1.28008
   Kearney RS, 2012, PHYSIOTHERAPY, V98, P24, DOI 10.1016/j.physio.2011.04.349
   KITAOKA HB, 1994, FOOT ANKLE INT, V15, P349, DOI 10.1177/107110079401500701
   Kotnis RA, 1999, J ACCID EMERG MED, V16, P378
   Magnussen RA, 2011, AM J SPORT MED, V39, P1522, DOI 10.1177/0363546510397815
   Martinelli B, 1999, J BONE JOINT SURG AM, V82, P1019
   Metz R, 2011, AM J SPORT MED, V39, P820, DOI 10.1177/0363546510392012
   Mortensen NHM, 1999, J BONE JOINT SURG AM, V81A, P983, DOI 10.2106/00004623-199907000-00011
   Nilsson-Helander K, 2007, AM J SPORT MED, V35, P421, DOI 10.1177/0363546506294856
   Raisbeck CC, 2000, J BONE JOINT SURG AM, V82A, P1804, DOI 10.2106/00004623-200012000-00016
   Schepull T, 2011, AM J SPORT MED, V39, P38, DOI 10.1177/0363546510383515
   SIMMONDS F A, 1957, Practitioner, V179, P56
   Talbot JC, 2012, J FOOT ANKLE SURG, V51, P147, DOI 10.1053/j.jfas.2011.10.045
   THOMPSON T C, 1962, Acta Orthop Scand, V32, P461, DOI 10.3109/17453676208989608
   Twaddle BC, 2007, AM J SPORT MED, V35, P2033, DOI 10.1177/0363546507307503
   van der Eng DM, 2013, J FOOT ANKLE SURG, V52, P622, DOI 10.1053/j.jfas.2013.03.027
   Zietek P, 2015, EUR J PHYS REHAB MED, V51, P245
NR 32
TC 0
Z9 2
U1 0
U2 3
PU ELSEVIER SCIENCE INC
PI NEW YORK
PA 360 PARK AVE SOUTH, NEW YORK, NY 10010-1710 USA
SN 1067-2516
EI 1542-2224
J9 J FOOT ANKLE SURG
JI J. Foot Ankle Surg.
PD MAY-JUN
PY 2016
VL 55
IS 3
BP 642
EP 644
DI 10.1053/j.jfas.2015.03.005
PG 3
WC Orthopedics; Surgery
WE Science Citation Index Expanded (SCI-EXPANDED)
SC Orthopedics; Surgery
GA DK8WG
UT WOS:000375208700037
PM 26002678
DA 2022-07-22
ER

PT J
AU Abdelatif, NMN
   Batista, JP
AF Abdelatif, Nasef Mohamed N.
   Pablo Batista, Jorge
TI Endoscopic Flexor Hallucis Longus Transfer for the Management of Acute
   Achilles Tendon Ruptures in Professional Soccer Players
SO FOOT & ANKLE INTERNATIONAL
LA English
DT Article
DE acute Achilles rupture; Achilles injury; Achilles tendon tear
   management; endoscopic flexor hallucis longus tendon transfer; Achilles
   tendon management; athletes; soccer players; footballers
ID RESTING ANGLE; NONOPERATIVE MANAGEMENT; CONSERVATIVE MANAGEMENT;
   CONSENSUS STATEMENT; OPEN REPAIR; FOLLOW-UP; RETURN; SPORT; OUTCOMES;
   PERFORMANCE
AB Background: Acute Achilles tendon ruptures (AATRs) that occur in athletes can be a career-ending injury. The aim of this study was to describe return to play and clinical outcomes of isolated endoscopic flexor hallucis longus (FHL) transfer in active soccer players with AATR.
   Methods: Twenty-seven active male soccer players who underwent endoscopically assisted FHL tendon transfer for acute Achilles tendon ruptures were included in this study. Follow up was 46.2 (+/- 10.9) months after surgery. Return to play criteria and clinical outcome measures were evaluated.
   Results: All players returned to playing professional competitive soccer games. Return to active team training was at a mean of 5.8 (+/- 1.1) months postoperatively. However, return to active competitive match play occurred at a mean of 8.3 (+/- 1.4) months. Twenty-two players (82%) were able to return to their preinjury levels and performances and resumed their professional careers at the same soccer club as their preinjury state. One player (3.7%) shifted his career to professional indoor soccer. At 26 months postoperatively, the mean Tegner activity scale score was 9.7 (+/- 0.4), the mean Achilles tendon total rupture score was 99 (+/- 2), and the mean American Orthopaedic Foot & Ankle Society anklehindfoot score was 99 (+/- 3). No patients reported any great toe complaints or symptomatic deficits of flexion strength.
   Conclusion: The current study demonstrated satisfactory and comparable return to play criteria and clinical results with minimal complications when using an advanced endoscopically assisted technique involving FHL tendon transfer to treat acute Achilles tendon ruptures in this specific subset of patient cohort.
C1 [Abdelatif, Nasef Mohamed N.] DrNasef OrthoClin, Head Orthoped Dept, Cairo, Egypt.
   [Pablo Batista, Jorge] Ctr Artroscop Jorge Batista SA, Orthopaed Dept, Head Ankle & Knee Sect, Buenos Aires, Caba, Argentina.
   [Pablo Batista, Jorge] Club Atlet Boca Jr, Dept Sport Med, Buenos Aires, DF, Argentina.
RP Abdelatif, NMN (通讯作者)，Sierras 2 G03,POB 229, Cairo 11571, Egypt.
EM docnasef@hotmail.com
OI Batista, Jorge Pablo/0000-0003-0910-4140
CR Amin NH, 2013, AM J SPORT MED, V41, P1864, DOI 10.1177/0363546513490659
   Amlang MH, 2005, UNFALLCHIRURG, V108, P529, DOI 10.1007/s00113-005-0938-8
   Ardern CL, 2016, BRIT J SPORT MED, V50, P853, DOI 10.1136/bjsports-2016-096278
   Braunstein M, 2018, KNEE SURG SPORT TR A, V26, P846, DOI 10.1007/s00167-015-3795-1
   Brumann M, 2014, INJURY, V45, P1782, DOI 10.1016/j.injury.2014.06.022
   Byrne PA, 2017, J FOOT ANKLE SURG, V56, P121, DOI 10.1053/j.jfas.2016.07.007
   Caldwell JME, 2019, FOOT ANKLE CLIN, V24, P439, DOI 10.1016/j.fcl.2019.04.003
   Carmont MR, 2013, FOOT ANKLE SURG, V19, P245, DOI 10.1016/j.fas.2013.06.007
   Carmont MR, 2020, ORTHOP J SPORTS MED, V8, DOI 10.1177/2325967120909556
   Carmont MR, 2015, ASIA-PAC J SPORT MED, V2, P49, DOI 10.1016/j.asmart.2014.12.002
   Carmont MR, 2011, BMC SPORTS SCI MED R, V3, DOI 10.1186/1758-2555-3-28
   Chan Y. H., 2003, SMJ Singapore Medical Journal, V44, P391
   Cukelj F, 2015, INJURY, V46, pS137, DOI 10.1016/j.injury.2015.10.070
   Davies GJ, 2017, CURR REV MUSCULOSKE, V10, P307, DOI 10.1007/s12178-017-9420-9
   De la Fuente Carlos, 2016, Foot (Edinb), V26, P15, DOI 10.1016/j.foot.2015.10.003
   Del Buono A, 2014, BRIT MED BULL, V109, P45, DOI 10.1093/bmb/ldt029
   Del Buono A, 2013, INT ORTHOP, V37, P715, DOI 10.1007/s00264-012-1743-y
   Deng SL, 2017, J FOOT ANKLE SURG, V56, P1236, DOI 10.1053/j.jfas.2017.05.036
   Ebinesan AD, 2008, DISABIL REHABIL, V30, P1721, DOI 10.1080/09638280701786815
   Ekstrand J, 2011, BRIT J SPORT MED, V45, P553, DOI 10.1136/bjsm.2009.060582
   ENWEMEKA CS, 1992, AM J PHYS MED REHAB, V71, P31, DOI 10.1097/00002060-199202000-00008
   Fanchini M, 2018, PHYS THER SPORT, V29, P70, DOI 10.1016/j.ptsp.2017.04.008
   Farber J, 2014, ORTHOP J SPORTS MED, V2, DOI 10.1177/2325967114559892
   Fuller CW, 2006, BRIT J SPORT MED, V40, P193, DOI 10.1136/bjsm.2005.025270
   Gajhede-Knudsen M, 2013, BRIT J SPORT MED, V47, P763, DOI 10.1136/bjsports-2013-092271
   Grassi A, 2020, BRIT J SPORT MED, V54, P480, DOI 10.1136/bjsports-2019-100556
   Gwynne-Jones DP, 2011, FOOT ANKLE INT, V32, P337, DOI 10.3113/FAI.2011.0337
   Habets B, 2018, SPORTS MED, V48, P705, DOI 10.1007/s40279-017-0833-9
   Hagen M, 2019, JAAOS GLOB RES REV, V3, DOI 10.5435/JAAOSGlobal-D-19-00016
   Hamilton B, 2008, J SCI MED SPORT, V11, P566, DOI 10.1016/j.jsams.2007.03.008
   Hansen MS, 2016, KNEE SURG SPORT TR A, V24, P1365, DOI 10.1007/s00167-015-3974-0
   Hurmeydan OM, 2020, FOOT ANKLE INT, V41, P140, DOI 10.1177/1071100719879659
   Jiang N, 2012, INT ORTHOP, V36, P765, DOI 10.1007/s00264-011-1431-3
   Johns W, 2021, FOOT ANKLE INT, V42, P495, DOI 10.1177/1071100720969633
   Jones Matthew P, 2012, J Bone Joint Surg Am, V94, pe88, DOI 10.2106/JBJS.J.01829
   Kangas J, 2007, AM J SPORT MED, V35, P59, DOI 10.1177/0363546506293255
   Keller A, 2014, AM J SPORT MED, V42, P731, DOI 10.1177/0363546513511418
   Lansdaal JR, 2007, INJURY, V38, P839, DOI 10.1016/j.injury.2006.12.010
   Lantto I, 2015, SCAND J MED SCI SPOR, V25, pE133, DOI 10.1111/sms.12253
   Li QB, 2016, J ORTHOP SURG RES, V11, DOI 10.1186/s13018-016-0401-2
   Lin TW, 2004, J BIOMECH, V37, P865, DOI 10.1016/j.jbiomech.2003.11.005
   Lubberts B, 2019, BRIT J SPORT MED, V53, P959, DOI 10.1136/bjsports-2017-097710
   Maffulli N, 1998, AM J SPORT MED, V26, P266, DOI 10.1177/03635465980260021801
   Maffulli N, 2011, FOOT ANKLE INT, V32, P9, DOI 10.3113/FAI.2011.0009
   Meyers MC, 2017, AM J SPORT MED, V45, P708, DOI 10.1177/0363546516671715
   Miller D, 2005, SCOT MED J, V50, P160, DOI 10.1177/003693300505000408
   Minhas SV, 2016, AM J SPORT MED, V44, P1056, DOI 10.1177/0363546515623028
   Moller M, 2001, J BONE JOINT SURG BR, V83B, P843
   Nilsson-Helander K, 2007, AM J SPORT MED, V35, P421, DOI 10.1177/0363546506294856
   Ochen Y, 2019, BMJ-BRIT MED J, V364, DOI 10.1136/bmj.k5120
   Batista JP, 2020, J FOOT ANKLE SURG, V59, P927, DOI 10.1053/j.jfas.2019.12.008
   Parekh Selene G, 2009, Foot Ankle Spec, V2, P283, DOI 10.1177/1938640009351138
   Plaass C, 2013, FOOT ANKLE INT, V34, P1580, DOI 10.1177/1071100713494780
   Raikin SM, 2013, FOOT ANKLE INT, V34, P475, DOI 10.1177/1071100713477621
   Reda Y, 2020, FOOT ANKLE SURG, V26, P280, DOI 10.1016/j.fas.2019.03.010
   Rettig AC, 2005, AM J SPORT MED, V33, P119, DOI 10.1177/0363546504268720
   Richardson DR, 2009, FOOT ANKLE INT, V30, P627, DOI 10.3113/FAI.2009.0627
   Rozis M, 2018, FOOT ANKLE INT, V39, P689, DOI 10.1177/1071100718757971
   Saltzman Bryan M, 2018, HSS J, V14, P223, DOI 10.1007/s11420-017-9603-3
   Saltzman C L, 1998, J Am Acad Orthop Surg, V6, P316
   Schepull T, 2013, AM J SPORT MED, V41, P2550, DOI 10.1177/0363546513501785
   Schipper ON, 2018, FOOT ANKLE INT, V39, P664, DOI 10.1177/1071100718758257
   Sochacki KR, 2019, J ISAKOS, V4, P15, DOI 10.1136/jisakos-2018-000246
   Spennacchio Pietro, 2016, Joints, V4, P52, DOI 10.11138/jts/2016.4.1.052
   Suchak AA, 2008, J BONE JOINT SURG AM, V90A, P1876, DOI 10.2106/JBJS.G.01242
   Suttinark Piyachart, 2009, J Med Assoc Thai, V92 Suppl 6, pS226
   Taglialavoro G, 2011, STRATEG TRAUMA LIMB, V6, P147, DOI 10.1007/s11751-011-0124-1
   Thompson J, 2011, CLIN PODIATR MED SUR, V28, P117, DOI 10.1016/j.cpm.2010.10.002
   Trofa DP, 2018, ORTHOP J SPORTS MED, V6, DOI 10.1177/2325967118810772
   Trofa DP, 2017, AM J SPORT MED, V45, P2864, DOI 10.1177/0363546517713001
   Uquillas CA, 2015, J BONE JOINT SURG AM, V97A, P1187, DOI 10.2106/JBJS.O.00002
   Vadala Antonio, 2014, Muscles Ligaments Tendons J, V4, P177
   van der Eng DM, 2013, J FOOT ANKLE SURG, V52, P622, DOI 10.1053/j.jfas.2013.03.027
   van Dijk CN, 2000, ARTHROSCOPY, V16, P871, DOI 10.1053/jars.2000.19430
   Ververidis AN, 2016, J ORTHOP, V13, P57, DOI 10.1016/j.jor.2015.09.004
   Vosseller JT, 2013, FOOT ANKLE INT, V34, P49, DOI 10.1177/1071100712460223
   Wapner K L, 1993, Foot Ankle, V14, P443
   Weber M, 2003, AM J SPORT MED, V31, P685, DOI 10.1177/03635465030310050901
   Wilcox DK, 2000, FOOT ANKLE INT, V21, P1004, DOI 10.1177/107110070002101204
   Wilkins R, 2012, AM J SPORT MED, V40, P2154, DOI 10.1177/0363546512453293
   Wong J, 2002, AM J SPORT MED, V30, P565, DOI 10.1177/03635465020300041701
   Worth N, 2007, J Orthop Surg (Hong Kong), V15, P311
   Wu YH, 2019, AM J SPORT MED, V47, P2251, DOI 10.1177/0363546518824601
   Yang B, 2017, INT J SURG, V40, P178, DOI 10.1016/j.ijsu.2017.03.021
   Zellers JA, 2018, FOOT ANKLE INT, V39, P343, DOI 10.1177/1071100717742372
   Zellers JA, 2016, BRIT J SPORT MED, V50, P1325, DOI 10.1136/bjsports-2016-096106
   Zhao XY, 2019, J FOOT ANKLE SURG, V58, P1235, DOI 10.1053/j.jfas.2018.09.032
NR 87
TC 1
Z9 1
U1 2
U2 2
PU SAGE PUBLICATIONS INC
PI THOUSAND OAKS
PA 2455 TELLER RD, THOUSAND OAKS, CA 91320 USA
SN 1071-1007
EI 1944-7876
J9 FOOT ANKLE INT
JI Foot Ankle Int.
PD FEB
PY 2022
VL 43
IS 2
BP 164
EP 175
DI 10.1177/10711007211036439
EA SEP 2021
PG 12
WC Orthopedics
WE Science Citation Index Expanded (SCI-EXPANDED)
SC Orthopedics
GA YY5PC
UT WOS:000702340400001
PM 34558318
DA 2022-07-22
ER

PT J
AU Binkley, HM
   Douglass, D
   Phillips, K
   Wise, SL
AF Binkley, Helen M.
   Douglass, Dillon
   Phillips, Kristi
   Wise, Stephanie L.
TI Rehabilitation and Return to Sport After Nonsurgical Treatment of
   Achilles Tendon Rupture
SO STRENGTH AND CONDITIONING JOURNAL
LA English
DT Article
DE achilles tendon rupture; tendon loading; tendon injury outcomes;
   functional rehabilitation; return to sport
ID EARLY FUNCTIONAL REHABILITATION; NONOPERATIVE TREATMENT; OPERATIVE
   TREATMENT; SURGICAL REPAIR; EARLY MOTION; MANAGEMENT; INJURY;
   METAANALYSIS; RECOVERY; BIOLOGY
AB This article presents an overview of the rehabilitation and return to sport following a nonsurgical Achilles tendon (AT) rupture. A brief description of the recognition and diagnosis of the injury, selection of nonoperative treatment, outcome measures, importance of range of motion, purpose of functional rehabilitation, description of the rehabilitation phases, and return to play progressions are included. A well-designed early functional rehabilitation program is more important than the method of treatment in outcomes and functional performance with AT ruptures.
C1 [Binkley, Helen M.; Douglass, Dillon; Phillips, Kristi; Wise, Stephanie L.] Middle Tennessee State Univ, Dept Hlth & Human Performance, Murfreesboro, TN 37130 USA.
RP Binkley, HM (通讯作者)，Middle Tennessee State Univ, Dept Hlth & Human Performance, Murfreesboro, TN 37130 USA.
EM Helen.Binkley@mtsu.edu
CR Alfredson H, 1998, AM J SPORT MED, V26, P360, DOI 10.1177/03635465980260030301
   Andarawis-Puri N, 2015, J ORTHOP RES, V33, P780, DOI 10.1002/jor.22869
   Balsalobre-Fernandez C, 2015, J SPORT SCI, V33, P1574, DOI 10.1080/02640414.2014.996184
   Barfod KW, 2016, TRIALS, V17, DOI 10.1186/s13063-016-1697-2
   Bleakney Robert R, 2005, Foot Ankle Clin, V10, P239, DOI 10.1016/j.fcl.2005.01.006
   Braunstein M, 2018, KNEE SURG SPORT TR A, V26, P846, DOI 10.1007/s00167-015-3795-1
   Carmont MR, 2015, ASIA-PAC J SPORT MED, V2, P49, DOI 10.1016/j.asmart.2014.12.002
   Cary David V, 2009, JAAPA, V22, P39
   CETTI R, 1993, AM J SPORT MED, V21, P791, DOI 10.1177/036354659302100606
   Dams OC, 2017, INJURY, V48, P2383, DOI 10.1016/j.injury.2017.09.013
   Doral MN, 2010, KNEE SURG SPORT TR A, V18, P638, DOI 10.1007/s00167-010-1083-7
   Ecker TM, 2016, AM J SPORT MED, V44, P1004, DOI 10.1177/0363546515623501
   Egger AC, 2017, CURR REV MUSCULOSKE, V10, P72, DOI 10.1007/s12178-017-9386-7
   Eliasson P, 2016, EUR J NUCL MED MOL I, V43, P1868, DOI 10.1007/s00259-016-3379-4
   Forlizzi JM, 2018, ORTHO RES ONLINE J, V4, P329
   Frankewycz B, 2017, ARCH ORTHOP TRAUM SU, V137, P333, DOI 10.1007/s00402-017-2627-9
   Freedman Benjamin R, 2014, Muscles Ligaments Tendons J, V4, P245
   Gallardo-Fuentes F, 2016, J STRENGTH COND RES, V30, P2049, DOI 10.1519/JSC.0000000000001304
   Galloway MT, 2013, J BONE JOINT SURG AM, V95A, P1620, DOI 10.2106/JBJS.L.01004
   Gheidi N, 2018, PHYS THER SPORT, V32, P260, DOI 10.1016/j.ptsp.2018.05.007
   Glazebrook M, 2019, FOOT ANKLE CLIN, V24, P387, DOI 10.1016/j.fcl.2019.05.001
   Silbernagel KG, 2007, BRIT J SPORT MED, V41, P276, DOI 10.1136/bjsm.2006.033464
   Griffin MJ, 2017, FOOT ANKLE INT, V38, P36, DOI 10.1177/1071100716669983
   Gulati V, 2015, WORLD J ORTHOP, V6, P380, DOI 10.5312/wjo.v6.i4.380
   Hansen MS, 2016, KNEE SURG SPORT TR A, V24, P1365, DOI 10.1007/s00167-015-3974-0
   Hiramatsu K, 2018, ORTHOP J SPORTS MED, V6, DOI 10.1177/2325967118789883
   Huang JZ, 2015, AM J SPORT MED, V43, P1008, DOI 10.1177/0363546514531014
   Hutchison AM, 2015, BONE JOINT J, V97B, P510, DOI 10.1302/0301-620X.97B4.35314
   Ingvar J, 2005, ACTA ORTHOP, V76, P597, DOI 10.1080/17453670510041619
   James R, 2008, J HAND SURG-AM, V33A, P102, DOI 10.1016/j.jhsa.2007.09.007
   Jiang N, 2012, INT ORTHOP, V36, P765, DOI 10.1007/s00264-011-1431-3
   KELLAM JF, 1985, CLIN ORTHOP RELAT R, P80
   Khan RJ, 2004, COCHRANE DB SYST REV, V3, DOI DOI 10.1002/14651858.CD003674.PUB2
   Killian ML, 2012, J SHOULDER ELB SURG, V21, P228, DOI 10.1016/j.jse.2011.11.002
   Lantto I, 2016, AM J SPORT MED, V44, P2406, DOI 10.1177/0363546516651060
   LaStayo PC, 2003, J ORTHOP SPORT PHYS, V33, P557, DOI 10.2519/jospt.2003.33.10.557
   Maffulli N, 1999, J BONE JOINT SURG AM, V81A, P1019, DOI 10.2106/00004623-199907000-00017
   Malliaras P, 2013, SPORTS MED, V43, P267, DOI 10.1007/s40279-013-0019-z
   Maquirriain Javier, 2011, Yale Journal of Biology and Medicine, V84, P289
   Mark-Christensen Troels, 2016, Knee Surg Sports Traumatol Arthrosc, V24, P1852, DOI 10.1007/s00167-014-3180-5
   McCormack R, 2015, BRIT J SPORT MED, V49, P1329, DOI 10.1136/bjsports-2015-094935
   Morelli V, 2004, PRIMARY CARE, V31, P1039, DOI 10.1016/j.pop.2004.07.009
   Mortensen NHM, 1999, J BONE JOINT SURG AM, V81A, P983, DOI 10.2106/00004623-199907000-00011
   Murtaugh B, 2013, CURR SPORT MED REP, V12, P175, DOI 10.1249/JSR.0b013e3182933761
   Neumayer F, 2010, ARCH ORTHOP TRAUM SU, V130, P363, DOI 10.1007/s00402-009-0865-1
   Nilsson-Helander K, 2007, AM J SPORT MED, V35, P421, DOI 10.1177/0363546506294856
   Nilsson-Helander K, 2010, AM J SPORT MED, V38, P2186, DOI 10.1177/0363546510376052
   Ochen Y, 2019, BMJ-BRIT MED J, V354, P1
   Olsson N, 2014, SCAND J MED SCI SPOR, V24, P152, DOI 10.1111/j.1600-0838.2012.01497.x
   Olsson N, 2013, AM J SPORT MED, V41, P2867, DOI 10.1177/0363546513503282
   Parekh Selene G, 2009, Foot Ankle Spec, V2, P283, DOI 10.1177/1938640009351138
   Pedowitz D, 2013, CURR REV MUSCULOSKE, V6, P285, DOI 10.1007/s12178-013-9185-8
   Poposka A, 2012, Prilozi, V33, P209
   Revak A, 2017, J ATHL TRAINING, V52, P89, DOI 10.4085/1062-6050-52.1.04
   Romero-Franco N, 2017, EUR J SPORT SCI, V17, P386, DOI 10.1080/17461391.2016.1249031
   Saxena A, 2011, J FOOT ANKLE SURG, V50, P558, DOI 10.1053/j.jfas.2011.04.045
   Schwieterman B, 2013, INT J SPORTS PHYS TH, V8, P416
   Sharma P., 2006, Journal of Musculoskeletal & Neuronal Interactions, V6, P181
   Silbernagel KG, 2015, J ORTHOP SPORT PHYS, V45, P876, DOI 10.2519/jospt.2015.5885
   Silbernagel KG, 2012, J ORTHOP SPORT PHYS, V42, P521, DOI 10.2519/jospt.2012.3913
   Silbernagel KG, 2006, KNEE SURG SPORT TR A, V14, P1207, DOI 10.1007/s00167-006-0150-6
   Spennacchio Pietro, 2016, Joints, V4, P52, DOI 10.11138/jts/2016.4.1.052
   Strom AC, 2009, FOOT ANKLE CLIN, V14, P773, DOI 10.1016/j.fcl.2009.08.003
   Tippett SR, 1995, FUNCTIONAL PROGR SPO, P29
   Twaddle BC, 2007, AM J SPORT MED, V35, P2033, DOI 10.1177/0363546507307503
   Wallace RGH, 2004, J BONE JOINT SURG AM, V86A, P1198, DOI 10.2106/00004623-200406000-00011
   Wang KC, 2017, OPER TECHN SPORT MED, V25, P214, DOI 10.1053/j.otsm.2017.07.009
   Wertz J, 2013, SPORTS HEALTH, V5, P407, DOI 10.1177/1941738112472165
   Wilkins R, 2012, AM J SPORT MED, V40, P2154, DOI 10.1177/0363546512453293
   Willits J, 2010, J BONE JOINT SURG AM, V91, P1
   Zellers JA, 2019, KNEE SURG SPORT TR A, V27, P2831, DOI 10.1007/s00167-018-5277-8
   Zellers JA, 2016, INT J SPORTS PHYS TH, V11, P1150
   Zellers JA, 2016, BRIT J SPORT MED, V50, P1325, DOI 10.1136/bjsports-2016-096106
NR 73
TC 0
Z9 0
U1 6
U2 12
PU LIPPINCOTT WILLIAMS & WILKINS
PI PHILADELPHIA
PA TWO COMMERCE SQ, 2001 MARKET ST, PHILADELPHIA, PA 19103 USA
SN 1524-1602
EI 1533-4295
J9 STRENGTH COND J
JI Strength Cond. J.
PD JUN
PY 2020
VL 42
IS 3
BP 90
EP 99
DI 10.1519/SSC.0000000000000536
PG 10
WC Sport Sciences
WE Science Citation Index Expanded (SCI-EXPANDED)
SC Sport Sciences
GA LW1AA
UT WOS:000538877200009
DA 2022-07-22
ER

PT J
AU White, DW
   Wenke, JC
   Mosely, DS
   Mountcastle, SB
   Basamania, CJ
AF White, Daniel W.
   Wenke, Joseph C.
   Mosely, Dan S.
   Mountcastle, Sally B.
   Basamania, Carl J.
TI Incidence of major tendon ruptures and anterior cruciate ligament tears
   in US army soldiers
SO AMERICAN JOURNAL OF SPORTS MEDICINE
LA English
DT Article
DE pectoralis major tendon rupture; quadriceps tendon rupture; patellar
   tendon rupture; Achilles tendon rupture; race; mechanism of injury
ID ACHILLES-TENDON; BILATERAL RUPTURE; INJURY; BLACK; BASKETBALL; AMERICAN;
   RACE; MEN
AB Background: Although a rare event, the prevalence of major tendon rupture has increased in recent decades. Identification of risk factors is important for prevention purposes.
   Hypothesis: Race is a risk factor for major tendon ruptures.
   Study Design: Cohort study (prevalence); Level of evidence, 2. Methods: All patients admitted for surgical management of a rupture of a major tendon at Womack Army Medical Center, Fort Bragg, North Carolina, in 1995 and 1996 were identified and evaluated for risk factors.
   Results: The authors identified 52 major tendon ruptures: 29 Achilles, 12 patellar, 7 pectoralis major, and 4 quadriceps tendon ruptures. All patients were active-duty soldiers, and 1 was a female soldier. Forty-one tendon ruptures occurred among black soldiers, 8 occurred among white soldiers, and 3 occurred among Latino soldiers. The population at risk included 93 224 exposures during the 2-year period, of which 67.1 % were white, 24.5% were black, and 8.4% were self-classified as other race. The rate ratio for tendon rupture, adjusted for gender and age, was 13.3 (95% confidence interval, 6.2-28.5) between blacks and whites and 2.9 (95% confidence interval, 0.8-10.9) between Latinos and whites.
   Conclusion: The rate of major tendon rupture was 13 times greater for black men in this study population when compared with whites. Interventions among those at a higher risk for injury should be considered.
C1 Tripler Army Med Ctr, Honolulu, HI 96859 USA.
   USA, Inst Surg Res, Ft Sam Houston, TX 78234 USA.
   DeWitt Army Hosp, Ft Belvoir, VA USA.
   Keller Army Hosp, West Point, NY USA.
   Duke Univ, Med Ctr, Durham, NC USA.
RP White, DW (通讯作者)，Tripler Army Med Ctr, 1 Jarrett White Rd, Honolulu, HI 96859 USA.
EM daniel.w.white@us.army.mil
CR AMA PFM, 1986, J APPL PHYSIOL, V61, P1758, DOI 10.1152/jappl.1986.61.5.1758
   AMA PFM, 1990, MED SCI SPORT EXER, V22, P508
   ARENDT E, 1995, AM J SPORT MED, V23, P694, DOI 10.1177/036354659502300611
   Bak K, 2000, KNEE SURG SPORT TR A, V8, P113, DOI 10.1007/s001670050197
   COETZER P, 1993, J APPL PHYSIOL, V75, P1822, DOI 10.1152/jappl.1993.75.4.1822
   Corps AN, 2005, RHEUMATOLOGY, V44, P1514, DOI 10.1093/rheumatology/kei087
   DAVID HG, 1995, J BONE JOINT SURG BR, V77B, P159, DOI 10.1302/0301-620X.77B1.7822381
   Davis JJ, 1999, MIL MED, V164, P872, DOI 10.1093/milmed/164.12.872
   DEFRANCO P, 1994, AM J KIDNEY DIS, V24, P951, DOI 10.1016/S0272-6386(12)81067-5
   Duey WJ, 1997, ANN HUM BIOL, V24, P323, DOI 10.1080/03014469700005072
   Garratty G, 2004, TRANSFUSION, V44, P703, DOI 10.1111/j.1537-2995.2004.03338.x
   HABUSTA SF, 1995, CLIN ORTHOP RELAT R, P231
   HAINES JF, 1983, ANN RHEUM DIS, V42, P652, DOI 10.1136/ard.42.6.652
   JARVINEN M, 1992, CLIN SPORT MED, V11, P493
   JOZSA L, 1989, AM J SPORT MED, V17, P338, DOI 10.1177/036354658901700305
   KANNUS P, 1991, J BONE JOINT SURG AM, V73A, P1507, DOI 10.2106/00004623-199173100-00009
   Kowatari K, 2004, J ORTHOP SCI, V9, P186, DOI 10.1007/s00776-003-0761-4
   LIOW RYL, 1995, BRIT J SPORT MED, V29, P77, DOI 10.1136/bjsm.29.2.77
   MALINA RM, 1988, CAN J SPORT SCI, V13, P136
   MALINA RM, 1974, VITAL HLTH STAT 11, V143, P1
   O'Shea K, 2002, INJURY, V33, P257, DOI 10.1016/S0020-1383(01)00110-3
   OBRIEN M, 1992, CLIN SPORT MED, V11, P505
   PELITON J, 2005, J AM ACAD ORTHOP SUR, V13, P59
   PLEIS JR, 2003, VITAL HLTH STAT, V212, P1
   Puranik Gururaj S, 2006, Acta Orthop Belg, V72, P176
   Rao S, 2005, ETHNIC DIS, V15, P191
   SIWEK CW, 1981, J BONE JOINT SURG AM, V63, P932, DOI 10.2106/00004623-198163060-00010
   Teitz, 1997, J Am Acad Orthop Surg, V5, P87
   Teitz C C, 1997, Instr Course Lect, V46, P569
   Trojian TH, 2006, AM J SPORT MED, V34, P895, DOI 10.1177/0363546505284384
   Van der Linden PD, 2001, ARTHRIT RHEUM-ARTHR, V45, P235, DOI 10.1002/1529-0131(200106)45:3<235::AID-ART254>3.0.CO;2-7
   WOLF SW, 1992, AM J SPORTS MED, V20, P694
NR 32
TC 51
Z9 52
U1 0
U2 5
PU SAGE PUBLICATIONS INC
PI THOUSAND OAKS
PA 2455 TELLER RD, THOUSAND OAKS, CA 91320 USA
SN 0363-5465
EI 1552-3365
J9 AM J SPORT MED
JI Am. J. Sports Med.
PD AUG
PY 2007
VL 35
IS 8
BP 1308
EP 1314
DI 10.1177/0363546507301256
PG 7
WC Orthopedics; Sport Sciences
WE Science Citation Index Expanded (SCI-EXPANDED)
SC Orthopedics; Sport Sciences
GA 196AB
UT WOS:000248452500011
PM 17468380
DA 2022-07-22
ER

PT J
AU Sugimoto, K
   Kasanami, R
   Iwai, M
   Takakura, Y
   Kawate, K
AF Sugimoto, K
   Kasanami, R
   Iwai, M
   Takakura, Y
   Kawate, K
TI Achilles tendon rupture associated with injury of the calcaneofibular
   ligament
SO JOURNAL OF ORTHOPAEDIC TRAUMA
LA English
DT Article
DE achilles tendon rupture; calcaneofibular ligament rupture; combined
   injury; combined ankle lesions
ID FRACTURE; ANKLE; STRAIN
AB A 49-year-old man collided against an infielder when he slid into second base during a recreational baseball game. He was unable to continue in the game due to diffuse pain and swelling of his hindfoot. A rupture of the Achilles tendon was diagnosed incidentally on palpation and observation of a positive Thompson's squeeze test. Subcutaneous hemorrhage at the lateral aspect of the heel and a small bone fragment under the lateral malleolus on an anteroposterior plain radiograph indicated a fracture of the calcaneal wall. At surgery, a complete rupture of the Achilles tendon and an avulsion of the calcaneofibular ligament from the calcaneal wall were seen. Both injuries were surgically repaired, and the patient subsequently did well. The mechanism of injury was thought to be impact hyperdorsiflexion of the ankle with rupture of the Achilles tendon accompanied by an inversion injury. Using a literature search, it was found that this combination of injuries has not been previously reported.
C1 Saiseikai Nara Hosp, Dept Orthopaed Surg, Nara 6308145, Japan.
   Nara Med Univ, Dept Orthopaed Surg, Nara, Japan.
RP Sugimoto, K (通讯作者)，Saiseikai Nara Hosp, Dept Orthopaed Surg, 4-643 Hachijo, Nara 6308145, Japan.
CR Assal M, 2002, J ORTHOP TRAUMA, V16, P358, DOI 10.1097/00005131-200205000-00013
   BARRON JL, 1993, AM J SPORT MED, V21, P629, DOI 10.1177/036354659302100426
   CLAYTON M L, 1962, Clin Orthop, V23, P52
   COLVILLE MR, 1990, AM J SPORT MED, V18, P196, DOI 10.1177/036354659001800214
   Lubin J W, 2000, Am J Orthop (Belle Mead NJ), V29, P707
   LUGGER LJ, 1977, ZBL CHIR, V102, P1320
   MARTIN JW, 1986, CLIN ORTHOP RELAT R, P216
   OBIRST J, 1989, UNFALLCHIRURG, V15, P141
   Pieper HG, 1998, INT J SPORTS MED, V19, P68, DOI 10.1055/s-2007-971883
   RENSTROM P, 1988, FOOT ANKLE, V9, P59, DOI 10.1177/107110078800900201
   Sugimoto K, 1998, J BONE JOINT SURG BR, V80B, P785, DOI 10.1302/0301-620X.80B5.7921
NR 11
TC 8
Z9 8
U1 0
U2 0
PU LIPPINCOTT WILLIAMS & WILKINS
PI PHILADELPHIA
PA 530 WALNUT ST, PHILADELPHIA, PA 19106-3621 USA
SN 0890-5339
J9 J ORTHOP TRAUMA
JI J. Orthop. Trauma
PD AUG
PY 2003
VL 17
IS 7
BP 534
EP 535
DI 10.1097/00005131-200308000-00012
PG 2
WC Orthopedics; Sport Sciences
WE Science Citation Index Expanded (SCI-EXPANDED)
SC Orthopedics; Sport Sciences
GA 709EG
UT WOS:000184611300012
PM 12902795
DA 2022-07-22
ER

PT J
AU Agres, AN
   Arampatzis, A
   Gehlen, T
   Manegold, S
   Duda, GN
AF Agres, Alison N.
   Arampatzis, Adamantios
   Gehlen, Tobias
   Manegold, Sebastian
   Duda, Georg N.
TI Muscle Fascicles Exhibit Limited Passive Elongation Throughout the
   Rehabilitation of Achilles Tendon Rupture After Percutaneous Repair
SO FRONTIERS IN PHYSIOLOGY
LA English
DT Article
DE muscle-tendon unit; achilles tendon rupture; ultrasonography;
   ultrasound; rehabilitation; achilles tendon - injuries
ID STRENGTH; OUTCOMES; LENGTH; ARCHITECTURE; PERFORMANCE; PLASTICITY;
   ATROPHY; ANKLE; KNEE; GAIT
AB Achilles tendon rupture (ATR) results in long-term functional and structural deficits, characterized by reduced ankle mobility and plantarflexor muscle atrophy. However, it remains unclear how such functional impairments develop after surgical repair. While it is known that this injury negatively affects the tendon's function, to date, limited work has focused on the short-term effect of ATR on the structure of the muscles in series. The aim of this study was to characterize changes in medial gastrocnemius architecture and its response to passive lengthening during the post-surgical rehabilitative period following ATR. Both injured and contralateral limbs from 10 subjects (1 female, BMI: 27.2 +/- 3.9 kg/m(2); age: 46 +/- 10 years) with acute, unilateral ATR were assessed at 8, 12, and 16 weeks after percutaneous surgical repair. To characterize the component tissues of the muscle-tendon unit, resting medial gastrocnemius muscle thickness, fascicle length, and pennation angle were determined from ultrasound images with the ankle in both maximal plantarflexion and dorsiflexion. The ankle range of motion (ROM) was determined using motion capture; combined ultrasound and motion capture determined the relative displacement of the musculotendinous junction (MTJ) of the AT with the medial gastrocnemius. The ATR-injured gastrocnemius muscle consistently exhibited lower thickness, regardless of time point and ankle angle. Maximal ankle plantarflexion angles and corresponding fascicle lengths were lower on the injured ankle compared to the contralateral throughout rehabilitation. When normalized to the overall ankle ROM, both injured fascicles and MTJ displacement exhibited a comparably lower change in length when the ankle was passively rotated. These results indicate that when both ankles are passively exposed to the same ROM following ATR surgery, both ipsilateral Achilles tendon and gastrocnemius muscle fascicles exhibit limited lengthening compared to the contralateral MTU tissues. This appears to be consistent throughout the rehabilitation of gait, suggesting that current post-operative rehabilitative exercises do not appear to induce muscle adaptations in the affected MTU.
C1 [Agres, Alison N.; Duda, Georg N.] Charite Univ Med Berlin, Julius Wolff Inst, Berlin, Germany.
   [Agres, Alison N.; Arampatzis, Adamantios; Duda, Georg N.] Free Univ Berlin, Berlin, Germany.
   [Agres, Alison N.; Arampatzis, Adamantios; Duda, Georg N.] Humboldt Univ, Berlin, Germany.
   [Agres, Alison N.; Arampatzis, Adamantios; Duda, Georg N.] Berlin Inst Hlth, Berlin, Germany.
   [Arampatzis, Adamantios] Humboldt Univ, Dept Training & Movement Sci, Berlin, Germany.
   [Gehlen, Tobias] Charite Univ Med Berlin, Ctr Musculoskeletal Surg, Berlin, Germany.
   [Manegold, Sebastian] BG Unfallklin Frankfurt Main, Dept Foot & Ankle Surg, Frankfurt, Germany.
RP Agres, AN (通讯作者)，Charite Univ Med Berlin, Julius Wolff Inst, Berlin, Germany.; Agres, AN (通讯作者)，Free Univ Berlin, Berlin, Germany.; Agres, AN (通讯作者)，Humboldt Univ, Berlin, Germany.; Agres, AN (通讯作者)，Berlin Inst Hlth, Berlin, Germany.
EM alison.agres@charite.de
RI ; Duda, Georg/H-2085-2016
OI Agres, Alison/0000-0002-9620-5689; Duda, Georg/0000-0001-7605-3908
FU German Research Foundation (DFG) [AG 293/2-1]; Berlin-Brandenburg School
   for Regenerative Therapies [DFG GSC 203]; Open Access Publication Fund
   of the Charite -Universitatsmedizin Berlin; German Research Foundation
   (DFG)
FX This work was supported by the German Research Foundation (DFG) through
   Grant AG 293/2-1 and also through the Berlin-Brandenburg School for
   Regenerative Therapies (DFG GSC 203). The authors acknowledge
   publication support from the German Research Foundation (DFG) and the
   Open Access Publication Fund of the Charite -Universitatsmedizin Berlin.
CR Agres AN, 2015, SCAND J MED SCI SPOR, V25, P860, DOI 10.1111/sms.12456
   Agres AN, 2018, GAIT POSTURE, V62, P179, DOI 10.1016/j.gaitpost.2018.03.007
   Amin NH, 2013, AM J SPORT MED, V41, P1864, DOI 10.1177/0363546513490659
   Amlang Michael H, 2006, Oper Orthop Traumatol, V18, P287, DOI 10.1007/s00064-006-1178-y
   Amlang Michael H, 2011, ISRN Orthop, V2011, P869703, DOI 10.5402/2011/869703
   Arampatzis A, 2010, J BIOMECH, V43, P3073, DOI 10.1016/j.jbiomech.2010.08.014
   Bohm S, 2014, J EXP BIOL, V217, P4010, DOI 10.1242/jeb.112268
   Bolsterlee B, 2017, J APPL PHYSIOL, V122, P727, DOI 10.1152/japplphysiol.00976.2016
   Bolsterlee B, 2016, PLOS ONE, V11, DOI 10.1371/journal.pone.0157273
   Brumann M, 2014, INJURY, V45, P1782, DOI 10.1016/j.injury.2014.06.022
   Chiodo CP, 2010, J BONE JOINT SURG AM, V92A, P2466
   Cohen J., 1988, STAT POWER ANAL BEHA, V2nd, DOI DOI 10.4324/9780203771587
   De Monte G, 2006, J ELECTROMYOGR KINES, V16, P413, DOI 10.1016/j.jelekin.2005.10.001
   Eliasson P, 2018, AM J SPORT MED, V46, P2492, DOI 10.1177/0363546518781826
   Finni T, 2006, CLIN BIOMECH, V21, P67, DOI 10.1016/j.clinbiomech.2005.08.007
   Franchi MV, 2018, SCAND J MED SCI SPOR, V28, P846, DOI 10.1111/sms.12961
   Grieve D., 1978, BIOMECHANICS 6, P405
   Heikkinen J, 2017, J BONE JOINT SURG AM, V99, P1509, DOI 10.2106/JBJS.16.01491
   Heikkinen J, 2017, AM J SPORT MED, V45, P1395, DOI 10.1177/0363546517694610
   Herbert RD, 2002, J PHYSIOL-LONDON, V539, P637, DOI 10.1113/jphysiol.2001.012756
   Herbert RD, 2011, J PHYSIOL-LONDON, V589, P5257, DOI 10.1113/jphysiol.2011.212175
   Holm C, 2015, SCAND J MED SCI SPOR, V25, pE1, DOI 10.1111/sms.12209
   Hullfish TJ, 2019, PEERJ, V7, DOI 10.7717/peerj.6788
   Ishikawa M, 2007, GAIT POSTURE, V25, P380, DOI 10.1016/j.gaitpost.2006.05.002
   Kangas J, 2007, AM J SPORT MED, V35, P59, DOI 10.1177/0363546506293255
   Kastoft R, 2019, KNEE SURG SPORT TR A, V27, P2781, DOI 10.1007/s00167-018-5058-4
   Lantto I, 2015, SCAND J MED SCI SPOR, V25, pE133, DOI 10.1111/sms.12253
   Lichtwark GA, 2018, J APPL PHYSIOL, V125, P1812, DOI 10.1152/japplphysiol.00480.2018
   McGraw KO, 1996, PSYCHOL METHODS, V1, P30, DOI 10.1037/1082-989X.1.4.390
   Mersmann F, 2014, SCAND J MED SCI SPOR, V24, pE283, DOI 10.1111/sms.12166
   Metz Roderik, 2009, Foot Ankle Spec, V2, P219, DOI 10.1177/1938640009348338
   Mullaney MJ, 2006, AM J SPORT MED, V34, P1120, DOI 10.1177/0363546505284186
   Olsson N, 2011, KNEE SURG SPORT TR A, V19, P1385, DOI 10.1007/s00167-011-1511-3
   Rosso C, 2013, KNEE SURG SPORT TR A, V21, P1369, DOI 10.1007/s00167-013-2407-1
   Sharma P, 2005, J BONE JOINT SURG AM, V87A, P187, DOI 10.2106/JBJS.D.01850
   Suchak AA, 2008, J BONE JOINT SURG AM, V90A, P1876, DOI 10.2106/JBJS.G.01242
   Suetta C, 2008, J APPL PHYSIOL, V105, P180, DOI 10.1152/japplphysiol.01354.2007
   Suydam SM, 2015, KNEE SURG SPORT TR A, V23, P868, DOI 10.1007/s00167-013-2512-1
   Trofa DP, 2017, AM J SPORT MED, V45, P2864, DOI 10.1177/0363546517713001
   Wang HK, 2013, ARCH PHYS MED REHAB, V94, P1590, DOI 10.1016/j.apmr.2013.01.015
   Wulf M, 2018, KNEE SURG SPORT TR A, V26, P2030, DOI 10.1007/s00167-017-4624-5
NR 41
TC 1
Z9 1
U1 1
U2 2
PU FRONTIERS MEDIA SA
PI LAUSANNE
PA AVENUE DU TRIBUNAL FEDERAL 34, LAUSANNE, CH-1015, SWITZERLAND
SN 1664-042X
J9 FRONT PHYSIOL
JI Front. Physiol.
PD JUL 21
PY 2020
VL 11
AR 746
DI 10.3389/fphys.2020.00746
PG 10
WC Physiology
WE Science Citation Index Expanded (SCI-EXPANDED)
SC Physiology
GA MY6KY
UT WOS:000558524900001
PM 32792966
OA gold, Green Published
DA 2022-07-22
ER

PT J
AU Rettig, AC
   Liotta, FJ
   Klootwyk, TE
   Porter, DA
   Mieling, P
AF Rettig, AC
   Liotta, FJ
   Klootwyk, TE
   Porter, DA
   Mieling, P
TI Potential risk of rerupture in primary Achilles tendon repair in
   athletes younger than 30 years of age
SO AMERICAN JOURNAL OF SPORTS MEDICINE
LA English
DT Article
DE athletic injury; Achilles tendon rupture; repair; rerupture
ID MANAGEMENT; RUPTURE
AB Background: Complete Achilles tendon ruptures are found more often in athletes who participate in sports involving explosive acceleration or maximal effort. In most studies, the consensus for athletes is surgery. This form of treatment has been shown to exhibit the best functional performance with a lower rerupture rate.
   Hypothesis: Achilles tendon ruptures in a young population (< 30 years) have a higher rerupture rate than similar injuries in an older age group (31-50 years), in which the injury is more common.
   Study Design: Cohort study; Level of evidence, 4.
   Methods: Retrospective study was carried out by chart review. Magnetic resonance images were obtained comparing appearance of repair in young and old patients at 8 to 12 weeks after operation.
   Results: There were a total of 4 reruptures in the 89 Achilles tendon repairs. This was an overall rerupture rate of 4.5%, which was consistent with the literature. When the reruptures were critically analyzed, it was noted that the 4 reruptures of the repaired tendon occurred in a young population. Of the 89, there was a subgroup of athletes (n = 24) who were 30 years of age or younger at the time of injury. The incidence of rerupture for these individuals was 16.6%. In the remaining athletes (n = 65) older than 30 years, the incidence of rerupture was zero. There were no significant differences (P <= .05) in all parameters measured (average days in a boot, average days to active range of motion, average time to full weight-bearing, average days to bike or use a stair climbing machine, average return to sports) between age groups except in the time from injury to surgery (7.1 days, for athletes <= 30 years vs 2.65 days for athletes > 31 years).
   Conclusions: The results of Achilles tendon repair with an early weightbearing and an early range of motion rehabilitation program are good. However, caution may need to be taken in the younger athlete (: 30 years) during rehabilitation.
   Clinical Relevance: Although the authors recommend aggressive rehabilitation for Achilles tendon repairs, caution should be observed in the younger athlete.
C1 Methodist Sports Med Ctr, Dept Res & Educ, Indianapolis, IN 46280 USA.
RP Rettig, AC (通讯作者)，Methodist Sports Med Ctr, Dept Res & Educ, 201 Penn Pkwy,Suite 235, Indianapolis, IN 46280 USA.
EM jaimeg@methodistsports.com
RI Porter, David/AAA-7974-2022
CR CETTI R, 1994, CLIN ORTHOP RELAT R, P155
   DEMAIO M, 1995, ORTHOPEDICS, V18, P195
   Dugan Dirk H., 1994, Journal of Athletic Training, V29, P349
   KANNUS P, 1991, J BONE JOINT SURG AM, V73A, P1507, DOI 10.2106/00004623-199173100-00009
   MANDELBAUM BR, 1995, AM J SPORT MED, V23, P392, DOI 10.1177/036354659502300403
   NISTOR L, 1981, J BONE JOINT SURG AM, V63, P394, DOI 10.2106/00004623-198163030-00012
   SOMA CA, 1995, ORTHOP CLIN N AM, V26, P239
   WILLS CA, 1986, CLIN ORTHOP RELAT R, V207, P156
   Wong J, 2002, AM J SPORT MED, V30, P565, DOI 10.1177/03635465020300041701
NR 9
TC 52
Z9 55
U1 0
U2 4
PU SAGE PUBLICATIONS INC
PI THOUSAND OAKS
PA 2455 TELLER RD, THOUSAND OAKS, CA 91320 USA
SN 0363-5465
EI 1552-3365
J9 AM J SPORT MED
JI Am. J. Sports Med.
PD JAN
PY 2005
VL 33
IS 1
BP 119
EP 123
DI 10.1177/0363546504268720
PG 5
WC Orthopedics; Sport Sciences
WE Science Citation Index Expanded (SCI-EXPANDED)
SC Orthopedics; Sport Sciences
GA 910IB
UT WOS:000227923300015
PM 15611008
DA 2022-07-22
ER

PT J
AU Majewski, M
   Rohrbach, M
   Czaja, S
   Ochsner, P
AF Majewski, M
   Rohrbach, M
   Czaja, S
   Ochsner, P
TI Avoiding sural nerve injuries during percutaneous Achilles tendon repair
SO AMERICAN JOURNAL OF SPORTS MEDICINE
LA English
DT Article
DE Achilles tendon rupture; percutaneous suture repair; sural nerve injury
ID NON-SURGICAL TREATMENT; NONOPERATIVE TREATMENT; RUPTURE; ANKLE; FOOT
AB Background: Sural nerve injury is a reported risk during percutaneous repair of the Achilles tendon.
   Hypothesis: Exposure of the sural nerve during percutaneous repair can minimize the risk of nerve injury.
   Study Design: Case control study; Level of evidence, 3.
   Methods: The authors retrospectively examined the results of 84 patients who were treated for acute Achilles tendon rupture at 2 different hospitals. Both hospitals used the same percutaneous repair technique, except that the sural nerve was exposed in the 38 patients (mean age, 38 years; range, 23-68 years) of one hospital; the nerve was not exposed in the 46 patients (mean age, 42 years; range, 24-71 years) of the other hospital (the nonexposure group).
   Results: All patients recovered and returned to work after 44 days (range, 5-202 days). All patients returned to their previous sports levels within 1 year. On the 100-point Hannover Achilles Tendon Score, the mean score was 81 points (range, 44-100 points). The overall incidence of sural nerve related complications was 18%. All sural nerve lesions occurred in the nonexposure group. In the total study population, there were 3 cases of deep vein thrombosis, 1 rerupture, and 1 case of infection.
   Conclusion: Sural nerve injuries can be minimized during surgery by carefully placing the stab incisions to expose the nerve so as to avoid it during repair. If the sural nerve is exposed, percutaneous repair of the ruptured Achilles tendon is a safe and reliable method of treating Achilles tendon ruptures.
C1 Kantonsspital Liestal, Dept Orthopaed Surg, CH-4410 Liestal, Switzerland.
RP Majewski, M (通讯作者)，Kantonsspital Liestal, Dept Orthopaed Surg, Rheinstr 26, CH-4410 Liestal, Switzerland.
EM majewski0l@yahoo.de
CR ARACIL J, 1992, FOOT ANKLE, V13, P350, DOI 10.1177/107110079201300610
   BRADLEY JP, 1990, AM J SPORT MED, V18, P188, DOI 10.1177/036354659001800213
   Buchgraber A, 1997, CLIN ORTHOP RELAT R, P113
   CETTI R, 1993, AM J SPORT MED, V21, P791, DOI 10.1177/036354659302100606
   FITZGIBBONS RE, 1993, AM J SPORT MED, V21, P724, DOI 10.1177/036354659302100516
   HEIM U, 1977, HELV CHIR ACTA, V44, P581
   HOCKENBURY RT, 1990, FOOT ANKLE, V11, P67, DOI 10.1177/107110079001100202
   Jacobs D, 1978, Am J Sports Med, V6, P107, DOI 10.1177/036354657800600302
   KAKIUCHI M, 1995, J BONE JOINT SURG BR, V77B, P60, DOI 10.1302/0301-620X.77B1.7822398
   KLEIN W, 1991, Chirurgia degli Organi di Movimento, V76, P223
   LAWRENCE SJ, 1994, FOOT ANKLE INT, V15, P490, DOI 10.1177/107110079401500906
   LEA RB, 1972, J BONE JOINT SURG AM, VA 54, P1398, DOI 10.2106/00004623-197254070-00002
   Lim J, 2001, FOOT ANKLE INT, V22, P559, DOI 10.1177/107110070102200705
   Lo IKY, 1997, CLIN J SPORT MED, V7, P207, DOI 10.1097/00042752-199707000-00010
   MA GWC, 1977, CLIN ORTHOP RELAT R, P247
   Majewski M, 2002, SPORTVERLETZ SPORTSC, V16, P167, DOI 10.1055/s-2002-37065
   Majewski M, 2000, ORTHOPADE, V29, P670
   MANDELBAUM BR, 1995, AM J SPORT MED, V23, P392, DOI 10.1177/036354659502300403
   Motta P, 1997, AM J SPORT MED, V25, P172, DOI 10.1177/036354659702500205
   NISTOR L, 1981, J BONE JOINT SURG AM, V63, P394, DOI 10.2106/00004623-198163030-00012
   Soldatis JJ, 1997, AM J SPORT MED, V25, P90, DOI 10.1177/036354659702500118
   Thermann H, 1996, FUNKTIONELLE BEHANDL
   THOMPSON T C, 1962, J Trauma, V2, P126, DOI 10.1097/00005373-196203000-00003
   Webb J, 2000, FOOT ANKLE INT, V21, P475, DOI 10.1177/107110070002100604
   Webb JM, 1999, J BONE JOINT SURG BR, V81B, P877, DOI 10.1302/0301-620X.81B5.9784
   WILLS CA, 1986, CLIN ORTHOP RELAT R, V207, P156
   Wong J, 2002, AM J SPORT MED, V30, P565, DOI 10.1177/03635465020300041701
NR 27
TC 71
Z9 82
U1 1
U2 2
PU SAGE PUBLICATIONS INC
PI THOUSAND OAKS
PA 2455 TELLER RD, THOUSAND OAKS, CA 91320 USA
SN 0363-5465
EI 1552-3365
J9 AM J SPORT MED
JI Am. J. Sports Med.
PD MAY
PY 2006
VL 34
IS 5
BP 793
EP 798
DI 10.1177/0363546505283266
PG 6
WC Orthopedics; Sport Sciences
WE Science Citation Index Expanded (SCI-EXPANDED)
SC Orthopedics; Sport Sciences
GA 036OK
UT WOS:000237083500012
PM 16627630
DA 2022-07-22
ER

PT J
AU Assal, M
   Stern, R
   Peter, R
AF Assal, M
   Stern, R
   Peter, R
TI Fracture of the ankle associated with rupture of the Achilles tendon:
   Case report and review of the literature
SO JOURNAL OF ORTHOPAEDIC TRAUMA
LA English
DT Article
DE ankle fracture; Achilles tendon rupture; combination injury;
   misdiagnosed ankle injury; combined ankle lesions
ID DISLOCATION; EPIDEMIOLOGY; ENTRAPMENT; POPULATION; INJURY
AB A thirty-five-year-old man fell two meters from a ladder and sustained a closed fracture of the medial malleolus with an ipsilateral complete Achilles tendon rupture. The Achilles tendon rupture was diagnosed by means of the patient's complaints and physical findings. The ankle fracture was diagnosed incidentally on routine radiographs. Such a combination of injuries has been reported infrequently in the literature, and striking similarities have been described in the mechanism of injury and fracture pattern. Remarkably, in three of four reports the combined injury was initially misdiagnosed.
C1 Hop Univ Geneve, Clin & Policlin Orthoped & Chirurg Appareil Moteu, Geneva, Switzerland.
RP Assal, M (通讯作者)，3731 Meridan Ave, Seattle, WA 98103 USA.
RI peter, robin/N-2514-2014
CR Anderson JG, 1996, FOOT ANKLE INT, V17, P114, DOI 10.1177/107110079601700211
   Barrett JA, 1999, J CLIN EPIDEMIOL, V52, P243, DOI 10.1016/S0895-4356(98)00167-X
   BARRON JL, 1993, AM J SPORT MED, V21, P629, DOI 10.1177/036354659302100426
   Bos M, 1997, FOOT ANKLE INT, V18, P823, DOI 10.1177/107110079701801213
   CLAYTON M L, 1962, Clin Orthop, V23, P52
   Court-Brown CM, 1998, ACTA ORTHOP SCAND, V69, P43, DOI 10.3109/17453679809002355
   Davis JJ, 1999, MIL MED, V164, P872, DOI 10.1093/milmed/164.12.872
   DEZWART DF, 1983, J BONE JOINT SURG AM, V65, P260, DOI 10.2106/00004623-198365020-00017
   Ebraheim N A, 1995, Am J Orthop (Belle Mead NJ), VSuppl, P22
   FUNK JR, SAE 2000 WORLD C
   GIBLIN MM, 1980, AUST NZ J SURG, V50, P59, DOI 10.1111/j.1445-2197.1980.tb04494.x
   Houshian S, 1998, INJURY, V29, P651, DOI 10.1016/S0020-1383(98)00147-8
   Jensen SL, 1998, ACTA ORTHOP SCAND, V69, P48, DOI 10.3109/17453679809002356
   KELBEL M, 1982, J TRAUMA, V22, P1026, DOI 10.1097/00005373-198212000-00009
   LEITSCHUH PH, 1995, FOOT ANKLE INT, V16, P232, DOI 10.1177/107110079501600413
   Leppilahti J, 1996, ANN CHIR GYNAECOL FE, V85, P369
   Leppilahti J, 1996, ACTA ORTHOP SCAND, V67, P277, DOI 10.3109/17453679608994688
   Levi N, 1997, INJURY, V28, P311, DOI 10.1016/S0020-1383(96)00200-8
   Lubin J W, 2000, Am J Orthop (Belle Mead NJ), V29, P707
   LUGGER LJ, 1977, ZBL CHIR, V102, P1320
   Maffulli N, 1999, CLIN J SPORT MED, V9, P157, DOI 10.1097/00042752-199907000-00007
   MARTIN JW, 1986, CLIN ORTHOP RELAT R, P216
   Moller A, 1996, ACTA ORTHOP SCAND, V67, P479, DOI 10.3109/17453679608996672
   MONTO RR, 1991, FOOT ANKLE, V11, P400, DOI 10.1177/107110079101100612
   MORITZ JR, 1959, AM J SURG, V98, P493, DOI 10.1016/0002-9610(59)90543-4
   Penney K E, 2000, Am J Orthop (Belle Mead NJ), V29, P41
   Pieper HG, 1998, INT J SPORTS MED, V19, P68, DOI 10.1055/s-2007-971883
   PIETU G, 1993, REV CHIR ORTHOP, V79, P127
   SCHAFFER JJ, 1987, J TRAUMA, V27, P795, DOI 10.1097/00005373-198707000-00018
   Sell S, 1996, ARCH ORTHOP TRAUM SU, V115, P28, DOI 10.1007/BF00453213
   SOBALLE K, 1988, CLIN ORTHOP RELAT R, P140
   STEIN RE, 1985, J BONE JOINT SURG AM, V67A, P493, DOI 10.2106/00004623-198567030-00024
   THERMANN H, 1995, UNFALLCHIRURG, V98, P570
   THOMPSON T C, 1962, J Trauma, V2, P126, DOI 10.1097/00005373-196203000-00003
NR 34
TC 12
Z9 12
U1 0
U2 0
PU LIPPINCOTT WILLIAMS & WILKINS
PI PHILADELPHIA
PA 530 WALNUT ST, PHILADELPHIA, PA 19106-3621 USA
SN 0890-5339
J9 J ORTHOP TRAUMA
JI J. Orthop. Trauma
PD MAY
PY 2002
VL 16
IS 5
BP 358
EP 361
DI 10.1097/00005131-200205000-00013
PG 4
WC Orthopedics; Sport Sciences
WE Science Citation Index Expanded (SCI-EXPANDED)
SC Orthopedics; Sport Sciences
GA 549FN
UT WOS:000175434000013
PM 11972082
DA 2022-07-22
ER

PT J
AU Byrne, PA
   Hopper, GP
   Wilson, WT
   Mackay, GM
AF Byrne, Paul A.
   Hopper, Graeme P.
   Wilson, William T.
   Mackay, Gordon M.
TI Knotless Repair of Achilles Tendon Rupture in an Elite Athlete: Return
   to Competition in 18 Weeks
SO JOURNAL OF FOOT & ANKLE SURGERY
LA English
DT Article
DE Achilles tendon rupture; injury; minimally invasive; rehabilitation;
   surgical repair
ID SUTURE BRIDGE TECHNIQUE; SURGICAL-TREATMENT; PERCUTANEOUS REPAIR;
   REHABILITATION; INSTABILITY; TENDINOSIS
AB Rupture of the Achilles tendon is an increasingly common injury, particularly in physically active males, and current evidence favors minimally invasive surgical repair. We describe the case of a 36-year-old male elite bobsled athlete with complete rupture of the Achilles tendon. He was treated with surgical repair of the ruptured tendon using an innovative, minimally invasive procedure based on an internal bracing concept and was able to undergo early mobilization and aggressive physiotherapy rehabilitation. His recovery was such that he returned to training at 13 weeks postoperatively and participated in an international competition at 18 weeks, winning a World Cup silver medal. He subsequently raced at the 2014 Winter Olympic Games at 29 weeks after surgery. At >2 years since his injury, he has experienced no complications or reinjury. This represents an exceptional recovery that far exceeds the standard expected for such injuries. The use of this technique for athletes could enable accelerated return to sporting activity and attainment of their preinjury activity levels. (C) 2016 by the American College of Foot and Ankle Surgeons. All rights reserved.
C1 [Byrne, Paul A.] Royal Infirm Edinburgh NHS Trust, NHS South East Scotland, Edinburgh EH16 4SA, Midlothian, Scotland.
   [Hopper, Graeme P.] Queen Elizabeth Univ Hosp, NHS Greater Glasgow & Clyde, Trauma & Orthopaed, Glasgow, Lanark, Scotland.
   [Wilson, William T.] Univ Glasgow, Glasgow, Lanark, Scotland.
   [Mackay, Gordon M.] BMI Ross Hall Hosp, Glasgow, Lanark, Scotland.
RP Byrne, PA (通讯作者)，Royal Infirm Edinburgh NHS Trust, NHS South East Scotland, Edinburgh EH16 4SA, Midlothian, Scotland.
EM paul.a.byrne@doctors.org.uk
OI Wilson, William Thomas/0000-0001-9025-081X; Hopper,
   Graeme/0000-0001-8770-6097
CR Al-Mouazzen L, 2015, J ORTHOP SURG-HONG K, V23, P352, DOI 10.1177/230949901502300320
   Alentorn-Geli E, 2016, KNEE SURG SPORT TR A, V24, P440, DOI 10.1007/s00167-015-3895-y
   Clanton TO, 2015, AM J SPORT MED, V43, P1957, DOI 10.1177/0363546515587082
   Cooper MT, 2015, CLIN SPORT MED, V34, P595, DOI 10.1016/j.csm.2015.06.001
   Costa ML, 2006, J BONE JOINT SURG BR, V88B, P69, DOI 10.1302/0301-620X.88B1.16549
   Fanter NJ, 2012, AM J SPORT MED, V40, P2085, DOI 10.1177/0363546512453294
   Greenhagen Robert M, 2013, Foot Ankle Spec, V6, P185, DOI 10.1177/1938640012473150
   Hani R, 2015, PAN AFR MED J, V21, DOI 10.11604/pamj.2015.21.202.6796
   Hegewald KW, 2016, J FOOT ANKLE SURG, V55, P166, DOI 10.1053/j.jfas.2015.08.001
   HOLMLUND DEW, 1976, ANN SURG, V184, P189, DOI 10.1097/00000658-197608000-00010
   Hsu AR, 2015, FOOT ANKLE INT, V36, P1279, DOI 10.1177/1071100715589632
   Huang JZ, 2015, AM J SPORT MED, V43, P1008, DOI 10.1177/0363546514531014
   Jallageas R, 2013, ORTHOP TRAUMATOL-SUR, V99, P577, DOI 10.1016/j.otsr.2013.03.024
   James R, 2008, J HAND SURG-AM, V33A, P102, DOI 10.1016/j.jhsa.2007.09.007
   Kangas J, 2007, AM J SPORT MED, V35, P59, DOI 10.1177/0363546506293255
   Karabinas Panagiotis K, 2014, Eur J Orthop Surg Traumatol, V24, P607, DOI 10.1007/s00590-013-1350-7
   Kujala UM, 2005, CLIN J SPORT MED, V15, P133, DOI 10.1097/01.jsm.0000165347.55638.23
   Lantto I, 2015, SCAND J MED SCI SPOR, V25, pE133, DOI 10.1111/sms.12253
   Lubowitz JH, 2014, ARTHROSC TEC, V3, pE505, DOI 10.1016/j.eats.2014.05.008
   MACKAY GM, 2015, ORTHOP MUSCUL SYST, V4, P188, DOI DOI 10.4172/2161-0533.1000188
   Mackay Gordon M, 2015, Surg Technol Int, V26, P239
   Maffulli N, 1999, CLIN J SPORT MED, V9, P157, DOI 10.1097/00042752-199907000-00007
   Majewski M, 2008, DISABIL REHABIL, V30, P1726, DOI 10.1080/09638280701786831
   McCormack R, 2015, BRIT J SPORT MED, V49, P1329, DOI 10.1136/bjsports-2015-094935
   McMahon Samuel E, 2011, Foot Ankle Surg, V17, P211, DOI 10.1016/j.fas.2010.11.001
   Miyamoto W, 2014, AM J SPORT MED, V42, P1441, DOI 10.1177/0363546514527418
   Ollivere BJ, 2014, FOOT ANKLE SURG, V20, pE27, DOI 10.1016/j.fas.2014.01.006
   Rigby RB, 2013, J FOOT ANKLE SURG, V52, P575, DOI 10.1053/j.jfas.2012.11.004
   Saxena A, 2008, J AM PODIAT MED ASSN, V98, P95
   Saxena A, 2011, J FOOT ANKLE SURG, V50, P558, DOI 10.1053/j.jfas.2011.04.045
   Schuh R, 2015, KNEE SURG SPORT TR A, V10, P1
   Singh D, 2015, BMJ-BRIT MED J, V351, DOI 10.1136/bmj.h4722
   Smoot MK, 2014, CLIN J SPORT MED, V24, P438, DOI 10.1097/JSM.0000000000000057
   Sveen TM, 2015, DAN MED J, V62
   Vadala Antonio, 2014, Muscles Ligaments Tendons J, V4, P177
   van der Meijden OA, 2013, ARTHROSCOPY, V29, P37, DOI 10.1016/j.arthro.2012.07.012
   Webb JM, 1999, J BONE JOINT SURG BR, V81B, P877, DOI 10.1302/0301-620X.81B5.9784
   Witt BL, 2012, J FOOT ANKLE SURG, V51, P487, DOI 10.1053/j.jfas.2012.03.006
NR 38
TC 9
Z9 10
U1 0
U2 7
PU ELSEVIER SCIENCE INC
PI NEW YORK
PA STE 800, 230 PARK AVE, NEW YORK, NY 10169 USA
SN 1067-2516
EI 1542-2224
J9 J FOOT ANKLE SURG
JI J. Foot Ankle Surg.
PD JAN-FEB
PY 2017
VL 56
IS 1
BP 121
EP 124
DI 10.1053/j.jfas.2016.07.007
PG 4
WC Orthopedics; Surgery
WE Science Citation Index Expanded (SCI-EXPANDED)
SC Orthopedics; Surgery
GA EH3DU
UT WOS:000391651300029
PM 27555353
DA 2022-07-22
ER

PT J
AU McGee, R
   Watson, T
   Eudy, A
   Brady, C
   Vanier, C
   LeCavalier, D
   Hoang, V
AF McGee, Roddy
   Watson, Troy
   Eudy, Adam
   Brady, Candice
   Vanier, Cheryl
   LeCavalier, Daniel
   Hoang, Victor
TI Anatomic relationship of the sural nerve when performing Achilles tendon
   repair using the percutaneous Achilles repair system, a cadaveric study
SO FOOT AND ANKLE SURGERY
LA English
DT Article
DE Achilles; Tendon; Trauma; Rupture; Percutaneous; Repair; Sports; Injury
ID MANAGEMENT; RUPTURES
AB Background: Minimally invasive techniques for Achilles tendon repair are increasing due to reports of similar rerupture rates using open and percutaneous techniques with fewer wound complications and quicker recovery with percutaneous methods. The goal of this study was to investigate quantitatively the relationship and risk of injury to the sural nerve during Achilles tendon repair when using the Percutaneous Achilles Repair System (PARS) (Arthrex (R), Naples, FL), by recording the distance between the passed needles and the sural nerve as well identifying any direct violation of the nerve with needle passage or nerve entrapment within the suture after the jig was removed. The hypothesis of the study is that the PARS technique can be performed safely and without significant risk of injury to the sural nerve.
   Methods: A total of five needles were placed through the PARS jig in each of 10 lower extremity cadaveric specimens using the proximal portion after simulation of a midsubstance Achilles tendon rupture. Careful dissection was performed to measure the distance of the sural nerve in relation to the passed needles. The sutures were then pulled out through the incision as the jig was removed from the proximal portion of the tendon and observation of the suture in relation to the tendon was documented.
   Results: Of the 10 cadaveric specimens, none had violation of the sural nerve. Zero of the 50 (0%) needles directly punctured the sural nerve. In addition, upon retraction of the jig, all sutures were noted to reside within the tendon sheath with no entrapment of the sural nerve noted.
   Conclusion: This study demonstrated the variable course of the sural nerve and identifies the potential risk for sural nerve injury when using the PARS for Achilles tendon repair. However, this study provides additional evidence of safety from an anatomic standpoint that explains the outcomes demonstrated in the clinical trials. With this information the authors believe surgeons should feel comfortable they can replicate those outcomes while minimizing risk of sural nerve injury when the technique is used correctly. (C) 2020 European Foot and Ankle Society. Published by Elsevier Ltd. All rights reserved.
C1 [McGee, Roddy] Total Sports Med & Orthoped, 10105 Banburry Cross Dr, Las Vegas, NV 89144 USA.
   [McGee, Roddy; Watson, Troy; Eudy, Adam; LeCavalier, Daniel; Hoang, Victor] Valley Hosp Med Ctr, Dept Orthoped Surg, 620 Shadow Lane, Las Vegas, NV 89106 USA.
   [Watson, Troy; Brady, Candice] Desert Orthopaed Ctr, 2800 East Desert Inn Rd, Las Vegas, NV 89121 USA.
   [Vanier, Cheryl] Touro Univ Nevada, 874 Amer Pacific Dr, Henderson, NV 89014 USA.
RP Eudy, A (通讯作者)，Valley Hosp Med Ctr, Dept Orthoped Surg, 620 Shadow Lane, Las Vegas, NV 89106 USA.
EM adameudy@gmail.com
OI McGee, William/0000-0002-7952-0994; Vanier, Cheryl/0000-0003-0497-6150;
   Hoang, Victor/0000-0003-3342-8081
FU Arthrex(R) [RDGT7001]
FX The authors would like to thank Goode Surgical (Las Vegas, NV) for the
   use of their lab facility for this study. Funding for cadaveric
   specimens and PARS implants supplied by Arthrex (R) as part of an
   educational research grant [grant number RDGT7001] but they were
   otherwise not involved in the study or publication.
CR Aibinder WR, 2013, FOOT ANKLE INT, V34, P870, DOI 10.1177/1071100712473097
   Aktas S, 2009, FOOT ANKLE INT, V30, P391, DOI 10.3113/FAI.2009.0391
   [Anonymous], PROPCIS VARIOUS CONF
   Grassi A, 2018, J BONE JOINT SURG AM, V100, P1969, DOI 10.2106/JBJS.17.01364
   Haji A, 2004, FOOT ANKLE INT, V25, P215, DOI 10.1177/107110070402500404
   Hsu AR, 2015, FOOT ANKLE INT, V36, P1279, DOI 10.1177/1071100715589632
   Kadakia AR, 2017, J AM ACAD ORTHOP SUR, V25, P23, DOI 10.5435/JAAOS-D-15-00187
   Kammar H, 2014, ORTHOPEDICS, V37, pE298, DOI 10.3928/01477447-20140225-64
   Kocher MS, 2002, AM J SPORT MED, V30, P783, DOI 10.1177/03635465020300060501
   McWilliam JR, 2016, FOOT ANKLE INT, V37, P794, DOI 10.1177/1071100716653373
   Newcombe RG, 1998, STAT MED, V17, P857, DOI 10.1002/(SICI)1097-0258(19980430)17:8<857::AID-SIM777>3.0.CO;2-E
   Porter KJ, 2014, FOOT ANKLE SURG, V20, P90, DOI 10.1016/j.fas.2013.11.005
   Webb J, 2000, FOOT ANKLE INT, V21, P475, DOI 10.1177/107110070002100604
   Yang X, 2018, BONE JOINT RES, V7, P561, DOI 10.1302/2046-3758.710.BJR-2018-0004.R2
NR 14
TC 3
Z9 3
U1 0
U2 0
PU ELSEVIER
PI AMSTERDAM
PA RADARWEG 29, 1043 NX AMSTERDAM, NETHERLANDS
SN 1268-7731
EI 1460-9584
J9 FOOT ANKLE SURG
JI Foot Ankle Surg.
PD JUN
PY 2021
VL 27
IS 4
BP 427
EP 431
DI 10.1016/j.fas.2020.05.011
EA JUN 2021
PG 5
WC Orthopedics
WE Science Citation Index Expanded (SCI-EXPANDED)
SC Orthopedics
GA SR7YS
UT WOS:000661263000015
PM 32553425
DA 2022-07-22
ER

PT J
AU Gooyers, CE
   Frost, DM
   McGill, SM
   Callaghan, JP
AF Gooyers, Chad E.
   Frost, David M.
   McGill, Stuart M.
   Callaghan, Jack P.
TI Partial rupture of the Achilles tendon during a simulated fire ground
   task: Insights obtained from a case report for the prevention and
   reporting of musculoskeletal injury
SO CLINICAL BIOMECHANICS
LA English
DT Article
DE Biomechanics; Injury; Achilles tendon; Rupture; Prevention; Reporting
ID MOVEMENT; REPRESENTATION
AB Background: In this case report an incumbent firefighter partially ruptured his right Achilles tendon during a study of the physical demands of firefighting.
   Methods: Kinematics and kinetics of the lower limbs and trunk were collected while the firefighter performed two simulated fire ground tasks. From this unexpected event, two insights were obtained that should be considered in all future injury prevention and reporting efforts.
   Findings: (i) Consider the full anatomical linkage the right ankle and knee kinematics leading up to the onset of injury trial were comparable to all preceding repetitions. However, there was a notable difference in the left knee starting position before the initiation of movement of the 37th hose-advance trial. (ii) Consider the cumulative load the task in question comprised forward and backward phases. A marked difference was observed in the frontal-plane ankle moment during the return phase of the trial preceding the injury. Additionally, the magnitude of the left side vertical ground reaction force was comparable across all trials, suggesting that loads experienced by the right limb were also similar. This would indicate that the tolerance of the Achilles tendon and not the magnitude of the loading was altered.
   Interpretation: The unfortunate injury captured in this work provides insight into the complexity of characterizing the pathways of injury. It is recommended that future injury prevention and reporting efforts consider individuals physical demands (at work and in life) and document the nature of loading (i.e., frequency, duration, magnitude, type) when considering the mechanism for injury. Crown Copyright (C) 2013 Published by Elsevier Ltd. All rights reserved.
C1 [Gooyers, Chad E.; Frost, David M.; McGill, Stuart M.; Callaghan, Jack P.] Univ Waterloo, Dept Kinesiol, Fac Appl Hlth Sci, Waterloo, ON N2L 3G1, Canada.
RP Callaghan, JP (通讯作者)，Univ Waterloo, Dept Kinesiol, Fac Appl Hlth Sci, Waterloo, ON N2L 3G1, Canada.
EM callagha@uwaterloo.ca
RI Callaghan, Jack P/A-3332-2008
OI Callaghan, Jack P/0000-0002-8245-7084
CR Begon M, 2007, GAIT POSTURE, V25, P353, DOI 10.1016/j.gaitpost.2006.04.010
   Cereatti A, 2006, J NEUROENG REHABIL, V3, DOI 10.1186/1743-0003-3-7
   COLE GK, 1993, J BIOMECH ENG-T ASME, V115, P344, DOI 10.1115/1.2895496
   Dempster WT, 1955, WADC TECHNICAL REPOR, P55
   Hess Gregory William, 2010, Foot Ankle Spec, V3, P29, DOI 10.1177/1938640009355191
   KUMAR S, 1990, SPINE, V15, P1311, DOI 10.1097/00007632-199012000-00014
   Kumar S, 2001, ERGONOMICS, V44, P17, DOI 10.1080/00140130150203866
   Leppilahti J, 1998, SPORTS MED, V25, P79, DOI 10.2165/00007256-199825020-00002
   Malvankar S, 2011, Foot (Edinb), V21, P193, DOI 10.1016/j.foot.2011.08.004
   McGill SM, 1997, J BIOMECH, V30, P465, DOI 10.1016/S0021-9290(96)00172-8
   Schwartz MH, 2005, J BIOMECH, V38, P107, DOI 10.1016/j.jbiomech.2004.03.009
   WOLTRING HJ, 1991, HUM MOVEMENT SCI, V10, P603, DOI 10.1016/0167-9457(91)90048-3
   Wren TAL, 2001, CLIN BIOMECH, V16, P245, DOI 10.1016/S0268-0033(00)00089-9
NR 13
TC 3
Z9 3
U1 0
U2 7
PU ELSEVIER SCI LTD
PI OXFORD
PA THE BOULEVARD, LANGFORD LANE, KIDLINGTON, OXFORD OX5 1GB, OXON, ENGLAND
SN 0268-0033
J9 CLIN BIOMECH
JI Clin. Biomech.
PD APR
PY 2013
VL 28
IS 4
BP 436
EP 440
DI 10.1016/j.clinbiomech.2013.02.002
PG 5
WC Engineering, Biomedical; Orthopedics; Sport Sciences
WE Science Citation Index Expanded (SCI-EXPANDED)
SC Engineering; Orthopedics; Sport Sciences
GA 170CD
UT WOS:000320826300012
PM 23466057
DA 2022-07-22
ER

PT J
AU Yang, YL
   Jia, HL
   Zhang, WP
   Xu, SH
   Wang, F
   Wang, BM
   Li, QH
   Wang, YH
   Han, SM
AF Yang Yongliang
   Jia Honglei
   Zhang Wupeng
   Xu Shihong
   Wang Fu
   Wang Bomin
   Li Qinghu
   Wang Yonghui
   Han Shumei
TI Intraoperative ultrasonography assistance for minimally invasive repair
   of the acute Achilles tendon rupture
SO JOURNAL OF ORTHOPAEDIC SURGERY AND RESEARCH
LA English
DT Article
DE Intraoperative ultrasonography; Minimally invasive; Acute Achilles
   tendon rupture; Sural nerve injury
ID SURAL NERVE; NONOPERATIVE TREATMENT; PERCUTANEOUS REPAIR; COMPLICATIONS;
   ANATOMY; INJURY
AB Background Minimally invasive repair is a better option for Achilles tendon rupture with low re-rupture and wound-related complications than conservative treatment or traditional open repair. The major problem is sural nerve injury. The purpose of this study was to evaluate the effect and advantage of the intraoperative ultrasonography assistance for minimally invasive repair of the acute Achilles tendon rupture. Methods A retrospective study was performed on 36 cases of acute Achilles tendon rupture treated with minimally invasive repair assisted with intraoperative ultrasonography from January 2015 to December 2017. The relationship of the sural nerve and small saphenous vein was confirmed on the preoperative MRI. The course of the small saphenous vein and the sural nerve was identified and marked by intraoperative ultrasonography. The ruptured Achilles tendon was repaired with minimally invasive Bunnell suture on the medial side of the small saphenous vein (SSV). Results All patients were followed up for at least 12 months. No sural nerve injury or other complications was found intraoperatively and postoperatively. All the patients returned to work and light sporting activities at a mean of 12.78 +/- 1.40 weeks and 17.28 +/- 2.34 weeks, respectively. The Mean American Orthopaedic Foot & Ankle Society (AOFAS) scores improved from 59.17 +/- 5.31 preoperatively to 98.92 +/- 1.63 at the time of 12 months follow-up. There was a statistically significant difference (P< 0.001). No patient complained of a negative effect on their life. Conclusions The minimally invasive repair assisted with intraoperative ultrasonography can yield good clinical outcomes, less surgical time, and less complications, especially sural nerve injury. It is an efficient, reliable, and safe method for acute Achilles tendon (AT) rupture.
C1 [Yang Yongliang; Jia Honglei; Zhang Wupeng; Xu Shihong; Wang Fu; Wang Bomin; Li Qinghu; Wang Yonghui] Shandong Univ, Dept Trauma & Emergency, Shandong Prov Hosp, 324 Jingwu Rd, Jinan 250021, Peoples R China.
   [Yang Yongliang; Jia Honglei; Xu Shihong; Wang Fu; Wang Bomin; Li Qinghu; Wang Yonghui] Shandong First Med Univ, Dept Trauma & Emergency, Shandong Prov Hosp, 324 Jingwu Rd, Jinan 250021, Peoples R China.
   [Han Shumei] Shandong First Med Univ & Shandong Acad Med Sci, Shandong Canc Hosp & Inst, Dept Oncol, Jinan 250021, Peoples R China.
RP Wang, YH (通讯作者)，Shandong Univ, Dept Trauma & Emergency, Shandong Prov Hosp, 324 Jingwu Rd, Jinan 250021, Peoples R China.; Wang, YH (通讯作者)，Shandong First Med Univ, Dept Trauma & Emergency, Shandong Prov Hosp, 324 Jingwu Rd, Jinan 250021, Peoples R China.; Han, SM (通讯作者)，Shandong First Med Univ & Shandong Acad Med Sci, Shandong Canc Hosp & Inst, Dept Oncol, Jinan 250021, Peoples R China.
EM trauma1973@hotmail.com; 15553115908@163.com
CR Assal M, 2002, J BONE JOINT SURG AM, V84A, P161, DOI 10.2106/00004623-200202000-00001
   Chen H, 2015, J ORTHOP SURG RES, V10, DOI 10.1186/s13018-015-0310-9
   Cretnik A, 2005, AM J SPORT MED, V33, P1369, DOI 10.1177/0363546504271501
   Cretnik Andrej, 2004, J Foot Ankle Surg, V43, P72, DOI 10.1053/j.jfas.2004.01.008
   Eid EM, 2011, CLIN ANAT, V24, P237, DOI 10.1002/ca.21068
   Flavin R, 2007, INJURY, V38, P845, DOI 10.1016/j.injury.2006.10.016
   Galletti S, 2015, MLTJ-MUSCLE LIGAMENT, V5, P325, DOI 10.11138/mltj/2015.5.4.325
   Giannetti S, 2014, ORTHOPEDICS, V37, P820, DOI 10.3928/01477447-20141124-04
   Haji A, 2004, FOOT ANKLE INT, V25, P215, DOI 10.1177/107110070402500404
   Jiang N, 2012, INT ORTHOP, V36, P765, DOI 10.1007/s00264-011-1431-3
   Jones Matthew P, 2012, J Bone Joint Surg Am, V94, pe88, DOI 10.2106/JBJS.J.01829
   KAKIUCHI M, 1995, J BONE JOINT SURG BR, V77B, P60, DOI 10.1302/0301-620X.77B1.7822398
   Kammar H, 2014, ORTHOPEDICS, V37, pE298, DOI 10.3928/01477447-20140225-64
   Khan RJK, 2005, J BONE JOINT SURG AM, V87A, P2202, DOI 10.2106/JBJS.D.03049
   KLEIN W, 1991, Chirurgia degli Organi di Movimento, V76, P223
   Lacoste S, 2014, ORTHOP TRAUMATOL-SUR, V100, P925, DOI 10.1016/j.otsr.2014.09.018
   MA GWC, 1977, CLIN ORTHOP RELAT R, P247
   Mavrodontidis A, 2015, ACTA ORTHOP TRAUMATO, V49, P661, DOI 10.3944/AOTT.2015.14.0196
   McMahon Samuel E, 2011, Foot Ankle Surg, V17, P211, DOI 10.1016/j.fas.2010.11.001
   Nilsson-Helander K, 2010, AM J SPORT MED, V38, P2186, DOI 10.1177/0363546510376052
   Paavola M, 2000, AM J SPORT MED, V28, P77, DOI 10.1177/03635465000280012501
   Pajala A, 2002, J BONE JOINT SURG AM, V84A, P2016, DOI 10.2106/00004623-200211000-00017
   Porter KJ, 2014, FOOT ANKLE SURG, V20, P90, DOI 10.1016/j.fas.2013.11.005
   Poynton AR, 2001, FOOT ANKLE INT, V22, P572, DOI 10.1177/107110070102200707
   Ricci S, 2010, EUR J VASC ENDOVASC, V39, P636, DOI 10.1016/j.ejvs.2009.11.024
   Rodriguez-Acevedo O, 2017, PHLEBOLOGY, V32, P49, DOI 10.1177/0268355515627262
   Saxena A, 2008, J AM PODIAT MED ASSN, V98, P95
   Tasatan E, 2016, J FOOT ANKLE SURG, V55, P971, DOI 10.1053/j.jfas.2016.04.016
   Wang D, 2015, FOOT ANKLE SURG, V21, P250, DOI 10.1016/j.fas.2015.01.009
   Willits K, 2010, J BONE JOINT SURG AM, V92A, P2767, DOI 10.2106/JBJS.I.01401
   Yang B, 2017, INT J SURG, V40, P178, DOI 10.1016/j.ijsu.2017.03.021
   Zappia M, 2018, FOOT ANKLE SURG, V24, P342, DOI 10.1016/j.fas.2017.03.006
NR 32
TC 3
Z9 3
U1 0
U2 1
PU BMC
PI LONDON
PA CAMPUS, 4 CRINAN ST, LONDON N1 9XW, ENGLAND
SN 1749-799X
J9 J ORTHOP SURG RES
JI J. Orthop. Surg. Res.
PD JUL 11
PY 2020
VL 15
IS 1
AR 258
DI 10.1186/s13018-020-01776-6
PG 8
WC Orthopedics
WE Science Citation Index Expanded (SCI-EXPANDED)
SC Orthopedics
GA MP2MS
UT WOS:000552043900001
PM 32653002
OA Green Published, gold, Green Submitted
DA 2022-07-22
ER

PT J
AU Chinen, T
   Sasabuchi, Y
   Matsui, H
   Yasunaga, H
AF Chinen, Takashi
   Sasabuchi, Yusuke
   Matsui, Hiroki
   Yasunaga, Hideo
TI Association Between Third-Generation Fluoroquinolones and Achilles
   Tendon Rupture: A Self-Controlled Case Series Analysis
SO ANNALS OF FAMILY MEDICINE
LA English
DT Article
DE fluoroquinolone; antibiotics; adverse effects; Achilles tendon rupture;
   tendon injuries; self-controlled case series; precision medicine
ID RISK
AB PURPOSE We investigated whether use of third-generation fluoroquinolones is associated with occurrence of Achilles tendon rupture using a case series analysis in which patients served as their own control.
   METHODS We used administrative claims data to identify residents in a single Japanese prefecture who were enrolled in National Health Insurance and Elderly Health Insurance from April 2012 to March 2017 and experienced Achilles tendon rupture after receiving an antibiotic prescription. Antibiotics were categorized into 3 groups: first- and second-generation fluoroquinolones, third-generation fluoroquinolones, and nonfluoroquinolones. We used a conditional Poisson regression model to estimate the incidence rate ratio (IRR) of Achilles tendon rupture during the antibiotic exposure period relative to the nonexposure period for each patient. An exposure period was defined as 30 days from start of the prescription.
   RESULTS Analyses were based on 504 patients with Achilles tendon rupture who had received antibiotic prescriptions. Risk of rupture was not significantly elevated during exposure to third-generation fluoroquinolones (IRR = 1.05; 95% CI, 0.33-3.37) and nonfluoroquinolones (IRR = 1.08; 95% CI, 0.80-1.47). In contrast, risk was significantly elevated during exposure to first- and second-generation fluoroquinolones (IRR = 2.94; 95% CI, 1.90-4.54). Findings were similar across subgroups stratified by sex and by recent corticosteroid use.
   CONCLUSIONS Our analysis showed that third-generation fluoroquinolone use was not associated with an increased risk of Achilles tendon rupture. These antibiotics may be a safer option for patients in whom this risk is elevated, such as athletes.
C1 [Chinen, Takashi] Jichi Med Univ, Dept Clin Oncol, 3311-1 Yakushiji, Shimotsuke, Tochigi 3290498, Japan.
   [Chinen, Takashi; Sasabuchi, Yusuke; Matsui, Hiroki; Yasunaga, Hideo] Jichi Med Univ, Data Sci Ctr, Shimotsuke, Tochigi, Japan.
   [Matsui, Hiroki; Yasunaga, Hideo] Univ Tokyo, Sch Publ Hlth, Dept Clin Epidemiol & Hlth Econ, Tokyo, Japan.
RP Chinen, T (通讯作者)，Jichi Med Univ, Dept Clin Oncol, 3311-1 Yakushiji, Shimotsuke, Tochigi 3290498, Japan.
EM m02061tc@jichi.ac.jp
OI Chinen, Takashi/0000-0002-9816-215X
FU Ministry of Education, Culture, Sports, Science and Technology
   [JP19K19394]
FX This work was supported by a Grant-in-Aid for Scientific Research from
   the Ministry of Education, Culture, Sports, Science and Technology
   (grant number JP19K19394).
CR Adriaenssens N, 2011, J ANTIMICROB CHEMOTH, V66, P47, DOI 10.1093/jac/dkr457
   Ball P, 2000, J ANTIMICROB CHEMOTH, V46, P17
   Bisaccia DR, 2019, BRIT MED BULL, V130, P39, DOI [10.1093/bmb/ldz00, 10.1093/bmb/ldz006]
   DiDiodato G, 2019, AM J INFECT CONTROL, V47, P9, DOI 10.1016/j.ajic.2018.06.016
   FARRINGTON CP, 1995, BIOMETRICS, V51, P228, DOI 10.2307/2533328
   Farrington P, 2018, SELF CONTROLLED CASE
   Gault N, 2017, BMC MED RES METHODOL, V17, DOI 10.1186/s12874-016-0278-0
   Hashimoto H, 2019, BMJ OPEN, V9, DOI 10.1136/bmjopen-2018-026251
   Hori K, 2012, PHARMACOEPIDEM DR S, V21, P886, DOI 10.1002/pds.3285
   Kashida Y, 1997, ANTIMICROB AGENTS CH, V41, P2389, DOI 10.1128/AAC.41.11.2389
   Lewis T, 2014, J ATHL TRAINING, V49, P422, DOI 10.4085/1062-6050-49.2.09
   Nordmann S, 2012, PLOS ONE, V7, DOI 10.1371/journal.pone.0049444
   Pasternak B, 2018, BMJ-BRIT MED J, V360, DOI 10.1136/bmj.k678
   Petersen I, 2016, BMJ-BRIT MED J, V354, DOI 10.1136/bmj.i4515
   Sasabuchi Y, 2018, BMJ OPEN, V8, DOI 10.1136/bmjopen-2017-021294
   Sode J, 2007, EUR J CLIN PHARMACOL, V63, P499, DOI 10.1007/s00228-007-0265-9
   Stephenson AL, 2013, DRUG SAFETY, V36, P709, DOI 10.1007/s40264-013-0089-8
   Takeuchi Y, 2017, THER INNOV REGUL SCI, V51, P332, DOI 10.1177/2168479016679865
   Tsutsui A, 2018, J INFECT CHEMOTHER, V24, P414, DOI 10.1016/j.jiac.2018.01.003
   van der Linden PD, 2003, ARCH INTERN MED, V163, P1801, DOI 10.1001/archinte.163.15.1801
   Whitaker HJ, 2018, BIOMETRICAL J, V60, P1110, DOI 10.1002/bimj.201800019
   Wise BL, 2012, AM J MED, V125, DOI 10.1016/j.amjmed.2012.05.027
   Yamana H, 2017, J EPIDEMIOL, V27, P476, DOI 10.1016/j.je.2016.09.009
NR 23
TC 3
Z9 3
U1 0
U2 1
PU ANNALS FAMILY MEDICINE
PI LEAWOOD
PA 11400 TOMAHAWK CREEK PARKWAY, LEAWOOD, KS 66211-2672 USA
SN 1544-1709
EI 1544-1717
J9 ANN FAM MED
JI Ann. Fam. Med.
PD MAY-JUN
PY 2021
VL 19
IS 3
BP 212
EP 216
DI 10.1370/afm.2673
PG 5
WC Primary Health Care; Medicine, General & Internal
WE Science Citation Index Expanded (SCI-EXPANDED)
SC General & Internal Medicine
GA SB7NL
UT WOS:000650175800005
PM 34180840
OA gold, Green Published
DA 2022-07-22
ER

PT J
AU Liu, JY
   Duan, WF
   Shen, S
   Ye, Y
   Sun, YQ
   He, W
AF Liu, Jun-Yi
   Duan, Wei-Feng
   Shen, Sheng
   Ye, Ye
   Sun, Yong-Qiang
   He, Wei
TI Achillon versus modified minimally invasive repair treatment in acute
   Achilles tendon rupture
SO JOURNAL OF ORTHOPAEDIC SURGERY
LA English
DT Article
DE Achilles tendon rupture; Achillon; minimally invasive repair; sports
   injury
ID PERCUTANEOUS REPAIR; SURGICAL REPAIR; COMPLICATIONS
AB Purpose:
   To date, the best treatment of acute Achilles tendon rupture (AATR) is still inconclusive. Achillon seems to be a promising approach with satisfactory function and low complication rate. We hypothesize a modified minimally invasive repair (MMIR), which provides direct visualization of proximal tendon stump without specialized equipment that could provide comparable results. This trial is aimed to evaluate the functional and surgical outcomes of MMIR comparing with Achillon.
   Methods:
   From February 2013 to February 2017, 114 patients with AATR were enrolled in this trial, underwent an alternative operation (Achillon or MMIR), and accelerated rehabilitation protocol. Forty-four patients took the Achillon and the other 70 patients took the MMIR at their subjective choice. One hundred eleven full follow-up data were obtained including Achilles tendon total rupture score (ATRS), time back to work, rerupture rate, overall complication rate, and operation time.
   Results:
   There was no significant difference between groups in demographic characters. There was no statistical difference between both groups regarding to time return to work and ATRS at 3rd, 6th, 12th, and 24th month, respectively. Five reruptures and two Achilles tendons tethering to skins were found in the Achillon group, and two reruptures and one sural nerve injury in the MMIR group. No wound infection and dehiscence occurred. Overall complication rate in the Achillon group is higher (16.3% vs. 4.4%, p = 0.044). The operation time of Achillon is less than MMIR (34.84 vs. 39.71, p < 0.001).
   Conclusion:
   Both techniques combining with accelerated rehabilitation showed to be reliable and effective. MMIR is safer and more economical, and Achillon is faster.
C1 [Liu, Jun-Yi; Duan, Wei-Feng] Guangzhou Univ Chinese Med, Sch Clin Med 1, Guangzhou, Peoples R China.
   [Liu, Jun-Yi; Duan, Wei-Feng; Shen, Sheng; Ye, Ye; Sun, Yong-Qiang] Orthped Hosp Henan Prov, Orthped Trauma Ctr, 100 Yongping Rd, Zhengzhou 450000, Peoples R China.
   [He, Wei] Guangzhou Univ Chinese Med, Affiliated Hosp 1, Guangzhou, Peoples R China.
RP Liu, JY (通讯作者)，Orthped Hosp Henan Prov, Orthped Trauma Ctr, 100 Yongping Rd, Zhengzhou 450000, Peoples R China.
EM liujunyi0101@gmail.com
OI Liu, Junyi/0000-0002-3227-161X
CR Aibinder WR, 2013, FOOT ANKLE INT, V34, P870, DOI 10.1177/1071100712473097
   Alcelik I, 2018, FOOT ANKLE SURG, V24, P427, DOI 10.1016/j.fas.2017.04.016
   Bartel AFP, 2014, J FOOT ANKLE SURG, V53, P744, DOI 10.1053/j.jfas.2014.07.009
   Bhattacharyya M, 2009, INT ORTHOP, V33, P151, DOI 10.1007/s00264-008-0564-5
   Carmont MR, 2013, KNEE SURG SPORT TR A, V21, P1361, DOI 10.1007/s00167-013-2423-1
   Chen H, 2015, J ORTHOP SURG RES, V10, DOI 10.1186/s13018-015-0310-9
   Daghino W, 2016, INJURY, V47, P2591, DOI 10.1016/j.injury.2016.09.009
   Davies H, 2017, INJURY, V48, P781, DOI 10.1016/j.injury.2017.01.018
   Deng SL, 2017, J FOOT ANKLE SURG, V56, P1236, DOI 10.1053/j.jfas.2017.05.036
   DeVries JG, 2017, CLIN PODIATR MED SUR, V34, P251, DOI 10.1016/j.cpm.2016.10.011
   Gigante A, 2008, KNEE SURG SPORT TR A, V16, P204, DOI 10.1007/s00167-007-0448-z
   Grassi A, 2018, J BONE JOINT SURG AM, V100, P1969, DOI 10.2106/JBJS.17.01364
   Holm C, 2015, SCAND J MED SCI SPOR, V25, pE1, DOI 10.1111/sms.12209
   Jiang N, 2012, INT ORTHOP, V36, P765, DOI 10.1007/s00264-011-1431-3
   Jung HG, 2008, FOOT ANKLE INT, V29, P803, DOI 10.3113/FAI.2008.0803
   Kolodziej L, 2013, INT ORTHOP, V37, P625, DOI 10.1007/s00264-012-1737-9
   Majewski M, 2006, AM J SPORT MED, V34, P793, DOI 10.1177/0363546505283266
   Makulavicius A, 2020, FOOT ANKLE SURG, V26, P580, DOI 10.1016/j.fas.2019.07.011
   McMahon Samuel E, 2011, Foot Ankle Surg, V17, P211, DOI 10.1016/j.fas.2010.11.001
   Nilsson-Helander K, 2007, AM J SPORT MED, V35, P421, DOI 10.1177/0363546506294856
   Olsson N, 2013, AM J SPORT MED, V41, P2867, DOI 10.1177/0363546513503282
   Soroceanu A, 2012, J BONE JOINT SURG AM, V94A, P2136, DOI 10.2106/JBJS.K.00917
   Tarnita DN, 2016, ROM J MORPHOL EMBRYO, V57, P211
   Vadala Antonio, 2014, Muscles Ligaments Tendons J, V4, P177
   Willits K, 2010, J BONE JOINT SURG AM, V92A, P2767, DOI 10.2106/JBJS.I.01401
   Yang B, 2017, INT J SURG, V40, P178, DOI 10.1016/j.ijsu.2017.03.021
NR 26
TC 2
Z9 2
U1 1
U2 2
PU SAGE PUBLICATIONS LTD
PI LONDON
PA 1 OLIVERS YARD, 55 CITY ROAD, LONDON EC1Y 1SP, ENGLAND
SN 1022-5536
EI 2309-4990
J9 J ORTHOP SURG-HONG K
JI J. Orthop. Surg.
PD JAN
PY 2020
VL 28
IS 1
AR 2309499020908354
DI 10.1177/2309499020908354
PG 7
WC Orthopedics; Surgery
WE Science Citation Index Expanded (SCI-EXPANDED)
SC Orthopedics; Surgery
GA LB0KK
UT WOS:000524326800001
PM 32129145
OA gold
DA 2022-07-22
ER

PT J
AU Steginsky, BD
   Van Dyke, B
   Berlet, GC
AF Steginsky, Brian D.
   Van Dyke, Bryan
   Berlet, Gregory C.
TI The Missed Achilles Tear Now what?
SO FOOT AND ANKLE CLINICS
LA English
DT Article
DE Achilles; Chronic Achilles tendon ruptures; Neglected Achilles tendon
   ruptures; Tendon transfer for Achilles ruptures; V-Y advancement
ID NONAUGMENTED SURGICAL REPAIR; HALLUCIS LONGUS TRANSFER; GRACILIS TENDON
   GRAFT; THIGH FREE-FLAP; NEGLECTED RUPTURE; CARBON-FIBER; GASTROCNEMIUS
   RECESSION; CALCANEAL TENDON; PLANTARIS TENDON; LOWER-EXTREMITY
AB Chronic Achilles tendon ruptures are debilitating injuries and are often associated with large tendon gaps that can be challenging for the foot and ankle surgeon to treat. Preoperative evaluation should include the patient's functional goals, medical comorbidities, MRI assessment of gastrocsoleus muscle viability, condition of adjacent flexor tendons, and size of the tendon defect. Although several surgical techniques have been described, the surgeon must formulate an individualized treatment plan for the patient. This article reviews the principles of diagnosis, treatment options, and clinical outcomes, and outlines the authors' preferred techniques.
C1 [Steginsky, Brian D.] Illinois Bone & Joint Inst, 720 Florsheim Dr, Libertyville, IL 60048 USA.
   [Van Dyke, Bryan; Berlet, Gregory C.] Orthoped Foot & Ankle Ctr, 300 Polaris Pkwy,Suite 2000, Westerville, OH 43082 USA.
   [Steginsky, Brian D.] 720 Florsheim Dr, Libertyville, IL 60048 USA.
   [Van Dyke, Bryan] 300 Polaris Pkwy,Suite 2000, Westerville, OH 43082 USA.
RP Berlet, GC (通讯作者)，Orthoped Foot & Ankle Ctr, 300 Polaris Pkwy,Suite 2000, Westerville, OH 43082 USA.
EM ofacresearch@orthofootankle.com
CR ABRAHAM E, 1975, J BONE JOINT SURG AM, VA 57, P253, DOI 10.2106/00004623-197557020-00019
   Ahmad Jamal, 2016, Foot Ankle Spec, V9, P400, DOI 10.1177/1938640016640895
   AMIS AA, 1984, J BONE JOINT SURG BR, V66, P131, DOI 10.1302/0301-620X.66B1.6229543
   AMIS AA, 1988, J BONE JOINT SURG BR, V70, P628, DOI 10.1302/0301-620X.70B4.3403613
   ARNER O, 1959, Acta Chir Scand Suppl, V116, P1
   Ballas MT, 1998, AM FAM PHYSICIAN, V57, P267
   Barber FA, 2008, FOOT ANKLE INT, V29, P329, DOI 10.3113/FAI.2008.0329
   BARNES MJ, 1986, J BONE JOINT SURG BR, V68, P121, DOI 10.1302/0301-620X.68B1.3941128
   Basiglini L, 2010, KNEE SURG SPORT TR A, V18, P644, DOI 10.1007/s00167-010-1085-5
   BOSWORTH DM, 1956, J BONE JOINT SURG AM, V38, P111, DOI 10.2106/00004623-195638010-00011
   Bruggeman NB, 2004, CLIN ORTHOP RELAT R, P63, DOI 10.1097/01.blo.0000144475.05543.e7
   BUGG EI, 1968, CLIN ORTHOP RELAT R, P73
   Bullock MJ, 2016, FOOT ANKLE SPEC
   Choksey A, 1996, INJURY, V27, P215, DOI 10.1016/0020-1383(95)00214-6
   CHRISTENSEN I, 1953, ACTA CHIR SCAND, V106, P50
   Cienfuegos A, 2013, J FOOT ANKLE SURG, V52, P95, DOI 10.1053/j.jfas.2012.06.006
   Coull R, 2003, FOOT ANKLE INT, V24, P931, DOI 10.1177/107110070302401211
   Cuttica DJ, 2015, J FOOT ANKLE SURG, V54, P99, DOI 10.1053/j.jfas.2014.09.014
   Den Hartog BD, 2003, FOOT ANKLE INT, V24, P233, DOI 10.1177/107110070302400306
   Den Hartog BD, 2008, FOOT ANKLE INT, V29, P456, DOI 10.3113/FAI.2008.0456
   Duhamel P, 2010, J BONE JOINT SURG AM, V92A, P2598, DOI 10.2106/JBJS.I.01578
   Elias I, 2007, FOOT ANKLE INT, V28, P1238, DOI 10.3113/FAI.2007.1238
   Gallant G G, 1995, Am J Orthop (Belle Mead NJ), V24, P257
   Geiger SE, 2016, WOUNDS, V28, P306
   GERDES MH, 1992, CLIN ORTHOP RELAT R, P241
   Gilcreest EL, 1933, J AMER MED ASSOC, V100, P153, DOI 10.1001/jama.1933.02740030001001
   Grove JR, 2008, FOOT ANKLE J, V1
   Guclu B, 2016, FOOT ANKLE INT, V37, P737, DOI 10.1177/1071100716642753
   Hahn F, 2008, FOOT ANKLE INT, V29, P794, DOI 10.3113/FAI.2008.0794
   Hanna T, 2014, FOOT ANKLE INT, V35, P168, DOI 10.1177/1071100713514037
   Hansen ST, 1991, DISORDERS FOOT ANKLE, P2355
   Heikkinen J, 2016, J BONE JOINT SURG AM, V98, P85, DOI 10.2106/JBJS.O.00496
   Hollawell S, 2015, J FOOT ANKLE SURG, V54, P1146, DOI 10.1053/j.jfas.2014.09.006
   HOWARD CB, 1984, J BONE JOINT SURG BR, V66, P206, DOI 10.1302/0301-620X.66B2.6707056
   Jennings AG, 2004, ANN ROY COLL SURG, V86, P445, DOI 10.1308/147870804957
   Jennings AG, 2002, J BONE JOINT SURG BR, V84B, P361, DOI 10.1302/0301-620X.84B3.11559
   Kissel C G, 1994, J Foot Ankle Surg, V33, P46
   KUWADA G T, 1990, Journal of Foot Surgery, V29, P361
   Lee Daniel K, 2007, J Foot Ankle Surg, V46, P451, DOI 10.1053/j.jfas.2007.05.007
   Lee JW, 2000, BRIT J PLAST SURG, V53, P574, DOI 10.1054/bjps.2000.3407
   Lee YS, 2005, ORTHOPEDICS, V28, P647, DOI 10.3928/0147-7447-20050701-10
   LEITNER A, 1992, FOOT ANKLE, V13, P176, DOI 10.1177/107110079201300402
   Lepow Gary M, 2006, J Foot Ankle Surg, V45, P351, DOI 10.1053/j.jfas.2006.06.004
   Leslie Hamish D H, 2005, Foot Ankle Clin, V10, P357, DOI 10.1016/j.fcl.2005.01.009
   Lewis JS, 2013, JBJS REV, V1, DOI 10.2106/JBJS.RVW.M.00034
   Lin JL, 2009, FOOT ANKLE CLIN, V14, P729, DOI 10.1016/j.fcl.2009.08.007
   LINDHOLM A, 1959, Acta Chir Scand, V117, P261
   LYNN TA, 1966, J BONE JOINT SURG AM, VA 48, P268, DOI 10.2106/00004623-196648020-00005
   Maffulli N, 2005, CLIN J SPORT MED, V15, P56, DOI 10.1097/01.jsm.0000152714.05097.ef
   Maffulli N, 2008, J BONE JOINT SURG AM, V90A, P1348, DOI 10.2106/JBJS.G.01241
   Maffulli N, 2012, J BONE JOINT SURG AM, V94A, P906, DOI 10.2106/JBJS.K.00869
   MANN RA, 1991, J BONE JOINT SURG AM, V73A, P214, DOI 10.2106/00004623-199173020-00009
   Mao HJ, 2015, ACTA ORTHOP BELG, V81, P553
   Matles A L, 1975, Bull Hosp Joint Dis, V36, P48
   Myerson M S, 1999, Instr Course Lect, V48, P219
   Nellas ZJ, 1996, J FOOT ANKLE SURG, V35, P190
   OBRIEN T, 1984, J BONE JOINT SURG AM, V66A, P1099, DOI 10.2106/00004623-198466070-00018
   Ofili KP, 2016, J FOOT ANKLE SURG, V55, P1245, DOI 10.1053/j.jfas.2016.01.001
   OZAKI J, 1989, CLIN ORTHOP RELAT R, P204
   Padanilam TG, 2009, FOOT ANKLE CLIN, V14, P711, DOI 10.1016/j.fcl.2009.08.001
   Pajala A, 2009, J BONE JOINT SURG AM, V91A, P1092, DOI 10.2106/JBJS.G.01089
   PARKER RG, 1979, J AM POD ASSOC, V69, P548, DOI 10.7547/87507315-69-9-548
   PARSONS JR, 1989, FOOT ANKLE, V9, P179, DOI 10.1177/107110078900900406
   Patil SSD, 2014, FOOT ANKLE INT, V35, P699, DOI 10.1177/1071100714531228
   PEREZTEUFFER A, 1974, ORTHOP CLIN N AM, V5, P89
   Porter DA, 1997, FOOT ANKLE INT, V18, P557, DOI 10.1177/107110079701800905
   Richardson DR, 2009, FOOT ANKLE INT, V30, P627, DOI 10.3113/FAI.2009.0627
   RUSH JH, 1980, AUST NZ J SURG, V50, P420, DOI 10.1111/j.1445-2197.1980.tb04156.x
   SILFVERSKIOLD N, 1941, ACTA CHIR SCAND, V84, P393
   STRAYER LM, 1950, J BONE JOINT SURG AM, V32-A, P671, DOI 10.2106/00004623-195032030-00022
   STRAYER LM, 1958, J BONE JOINT SURG AM, V40, P1019, DOI 10.2106/00004623-195840050-00004
   Takao M, 2003, ARCH ORTHOP TRAUM SU, V123, P471, DOI 10.1007/s00402-002-0443-2
   THOMPSON T C, 1962, Acta Orthop Scand, V32, P461, DOI 10.3109/17453676208989608
   TOBIN W J, 1953, Am Surg, V19, P514
   TURCO VJ, 1987, FOOT ANKLE, V7, P253, DOI 10.1177/107110078700700407
   Uquillas CA, 2015, J BONE JOINT SURG AM, V97A, P1187, DOI 10.2106/JBJS.O.00002
   Us AK, 1997, ARCH ORTHOP TRAUM SU, V116, P408
   Wapner K L, 1993, Foot Ankle, V14, P443
   Yasuda T, 2016, J BONE JOINT SURG AM, V98, P1168, DOI 10.2106/JBJS.15.00865
   Zadek I, 1940, J BONE JOINT SURG, V22, P1070
NR 80
TC 5
Z9 6
U1 0
U2 3
PU W B SAUNDERS CO-ELSEVIER INC
PI PHILADELPHIA
PA 1600 JOHN F KENNEDY BOULEVARD, STE 1800, PHILADELPHIA, PA 19103-2899 USA
SN 1083-7515
EI 1558-1934
J9 FOOT ANKLE CLIN
JI Foot Ankle Clin.
PD DEC
PY 2017
VL 22
IS 4
BP 715
EP +
DI 10.1016/j.fcl.2017.07.004
PG 21
WC Orthopedics
WE Science Citation Index Expanded (SCI-EXPANDED)
SC Orthopedics
GA FN7MO
UT WOS:000416202500006
PM 29078824
DA 2022-07-22
ER

PT J
AU Caldwell, JME
   Vosseller, JT
AF Caldwell, Jon-Michael E.
   Vosseller, J. Turner
TI Maximizing Return to Sports After Achilles Tendon Rupture in Athletes
SO FOOT AND ANKLE CLINICS
LA English
DT Article
DE Achilles tendon rupture; Return to play; Athlete; Sports injury;
   Tendonitis; Sports medicine
ID EARLY WEIGHT-BEARING; REPAIR; PERFORMANCE; PLAY; TENDINOPATHY;
   METAANALYSIS; COMPETITION; OUTCOMES; COMMON; SCORE
AB Achilles tendon ruptures are devastating injuries to athletes, with return-to-sports rates around 70% and some risk for diminished performance post-injury. Surgical management in athletes is often favored for a number of reasons, although evidence guiding the optimal treatment is limited. Functional rehabilitation has been supported as a key component of operative and nonoperative treatment plans. Return-to-play protocols in the literature are sparse and varied due to often ambiguous definitions of what it means to return to sport and a lack of explicit criteria. Optimal sport-specific return-to-play milestones should be defined to guide the rehabilitation of injured athletes.
C1 [Caldwell, Jon-Michael E.; Vosseller, J. Turner] Columbia Univ, Dept Orthoped Surg, Irving Med Ctr, NewYork Presbyterian Hosp, 622 West 168th St,PH11 Ctr Wing, New York, NY 10032 USA.
RP Vosseller, JT (通讯作者)，Columbia Univ, Dept Orthoped Surg, Irving Med Ctr, NewYork Presbyterian Hosp, 622 West 168th St,PH11 Ctr Wing, New York, NY 10032 USA.
EM turner.vosseller@gmail.com
RI Caldwell, Jon-Michael/AAG-6503-2021
OI Caldwell, Jon-Michael/0000-0003-3767-5813
CR Amin Nirav H, 2016, Clin Res Foot Ankle, V4, DOI 10.4172/2329-910X.1000205
   Amin NH, 2013, AM J SPORT MED, V41, P1864, DOI 10.1177/0363546513490659
   Ardern CL, 2016, BRIT J SPORT MED, V50, P853, DOI 10.1136/bjsports-2016-096278
   Barfod KW, 2014, J BONE JOINT SURG AM, V96A, P1497, DOI 10.2106/JBJS.M.01273
   Bostick GP, 2010, J ORTHOP SPORT PHYS, V40, P345, DOI 10.2519/jospt.2010.3204
   Byrne PA, 2017, J FOOT ANKLE SURG, V56, P121, DOI 10.1053/j.jfas.2016.07.007
   Davies GJ, 2017, CURR REV MUSCULOSKE, V10, P307, DOI 10.1007/s12178-017-9420-9
   ENWEMEKA CS, 1992, AM J PHYS MED REHAB, V71, P31, DOI 10.1097/00002060-199202000-00008
   Fanchini M, 2018, PHYS THER SPORT, V29, P70, DOI 10.1016/j.ptsp.2017.04.008
   Gajhede-Knudsen M, 2013, BRIT J SPORT MED, V47, P763, DOI 10.1136/bjsports-2013-092271
   Habets B, 2018, SPORTS MED, V48, P705, DOI 10.1007/s40279-017-0833-9
   Hansen MS, 2016, KNEE SURG SPORT TR A, V24, P1365, DOI 10.1007/s00167-015-3974-0
   Hardy A, 2018, ORTHOP TRAUMATOL-SUR, V104, P719, DOI 10.1016/j.otsr.2018.05.003
   Ho G, 2017, INJURY, V48, P1701, DOI 10.1016/j.injury.2017.04.007
   Jack RA, 2017, FOOT ANKLE INT, V38, P1092, DOI 10.1177/1071100717718131
   Kangas J, 2007, AM J SPORT MED, V35, P59, DOI 10.1177/0363546506293255
   Lin TW, 2004, J BIOMECH, V37, P865, DOI 10.1016/j.jbiomech.2003.11.005
   Maffulli N, 2011, FOOT ANKLE INT, V32, P9, DOI 10.3113/FAI.2011.0009
   Mai HT, 2016, AM J SPORT MED, V44, P2255, DOI 10.1177/0363546516651426
   Minhas SV, 2016, AM J SPORT MED, V44, P1056, DOI 10.1177/0363546515623028
   Nilsson-Helander K, 2007, AM J SPORT MED, V35, P421, DOI 10.1177/0363546506294856
   Noback PC, 2017, INJURY, V48, P2342, DOI 10.1016/j.injury.2017.08.050
   Parekh Selene G, 2009, Foot Ankle Spec, V2, P283, DOI 10.1177/1938640009351138
   Saltzman Bryan M, 2017, HSS J, V13, P108, DOI 10.1007/s11420-016-9540-6
   Schepull T, 2013, AM J SPORT MED, V41, P2550, DOI 10.1177/0363546513501785
   Silbernagel KG, 2015, J ORTHOP SPORT PHYS, V45, P876, DOI 10.2519/jospt.2015.5885
   Soroceanu A, 2012, J BONE JOINT SURG AM, V94A, P2136, DOI 10.2106/JBJS.K.00917
   Suchak AA, 2008, J BONE JOINT SURG AM, V90A, P1876, DOI 10.2106/JBJS.G.01242
   Trofa DP, 2017, AM J SPORT MED, V45, P2864, DOI 10.1177/0363546517713001
   Willits K, 2010, J BONE JOINT SURG AM, V92A, P2767, DOI 10.2106/JBJS.I.01401
   Zellers JA, 2016, BRIT J SPORT MED, V50, P1325, DOI 10.1136/bjsports-2016-096106
NR 31
TC 8
Z9 9
U1 1
U2 12
PU W B SAUNDERS CO-ELSEVIER INC
PI PHILADELPHIA
PA 1600 JOHN F KENNEDY BOULEVARD, STE 1800, PHILADELPHIA, PA 19103-2899 USA
SN 1083-7515
EI 1558-1934
J9 FOOT ANKLE CLIN
JI Foot Ankle Clin.
PD SEP
PY 2019
VL 24
IS 3
BP 439
EP +
DI 10.1016/j.fcl.2019.04.003
PG 8
WC Orthopedics
WE Science Citation Index Expanded (SCI-EXPANDED)
SC Orthopedics
GA IX7JC
UT WOS:000485858600006
PM 31370995
DA 2022-07-22
ER

PT J
AU Park, YH
   Kim, TJ
   Choi, GW
   Kim, HJ
AF Park, Young Hwan
   Kim, Tae Jin
   Choi, Gi Won
   Kim, Hak Jun
TI Achilles tendinosis does not always precede Achilles tendon rupture
SO KNEE SURGERY SPORTS TRAUMATOLOGY ARTHROSCOPY
LA English
DT Article
DE Achilles tendon rupture; Achilles tendinosis; Associated factor;
   Histology
ID INJURIES; TENDINOPATHY; SCORE; RISK
AB Purpose Degenerative change is a typical histologic finding of Achilles tendinosis and is thought to be a factor associated with Achilles tendon rupture; however, this relationship remains controversial. Therefore, the aim of this study was to review the histological findings of ruptured Achilles tendons and analyze demographics and injury patterns. Methods Retrospective analysis was performed for all patients who underwent Achilles tendon repair for acute Achilles tendon rupture at our institution between 2008 and 2015. The presence of Achilles tendinosis was determined by light microscopic histology of ruptured Achilles tendons. Patients were grouped according to the presence of Achilles tendinosis and patient demographics, sports activities, and clinical outcomes were compared. Ankle activity score was used to evaluate the level of sports activity and Achilles tendon total rupture score (ATRS) was used to evaluate clinical outcomes. Results A total of 152 Achilles tendon ruptures were included: 112 (77%) showed Achilles tendinosis at a ruptured Achilles tendon on histology and 40 (23%) did not. Patients who had no Achilles tendinosis prior to rupture had a lower ankle activity score (P < 0.05) and higher incidence of non-sports related injury (P < 0.05). ATRS was not significantly different between the groups at final follow-up (P = n.s.). Conclusion In contrast to previous reports, Achilles tendon rupture can occur without prior Achilles tendinosis, especially in non-sports-related injuries and in patients with low sports activity. Although the direct clinical applications of these results are limited, the findings of this study can improve our understanding of the etiology of Achilles tendon rupture.
C1 [Park, Young Hwan; Kim, Tae Jin; Kim, Hak Jun] Korea Univ, Guro Hosp, Dept Orthoped Surg, Seoul, South Korea.
   [Choi, Gi Won] Korea Univ, Ansan Hosp, Dept Orthoped Surg, Seoul, South Korea.
RP Kim, HJ (通讯作者)，Korea Univ, Guro Hosp, Dept Orthoped Surg, Seoul, South Korea.
EM hjunkimos@gmail.com
RI Kim, Hak Jun/AAR-5929-2020; Choi, Gi Won/AAX-7187-2020
OI Kim, Hak Jun/0000-0003-3633-6174;
CR ARNER O, 1959, Acta Chir Scand, V116, P484
   ASTROM M, 1995, CLIN ORTHOP RELAT R, P151
   Ganestam A, 2016, KNEE SURG SPORT TR A, V24, P3730, DOI 10.1007/s00167-015-3544-5
   Halasi T, 2004, AM J SPORT MED, V32, P899, DOI 10.1177/0363546503262181
   Hess Gregory William, 2010, Foot Ankle Spec, V3, P29, DOI 10.1177/1938640009355191
   Jozsa L, 1997, SCAND J MED SCI SPOR, V7, P113
   KANNUS P, 1991, J BONE JOINT SURG AM, V73A, P1507, DOI 10.2106/00004623-199173100-00009
   Khan KM, 1998, CLIN J SPORT MED, V8, P151, DOI 10.1097/00042752-199807000-00001
   KRACKOW KA, 1988, ORTHOPEDICS, V11, P909, DOI 10.3928/0147-7447-19880601-11
   Krolo I, 2007, COLLEGIUM ANTROPOL, V31, P275
   KVIST M, 1991, ANN CHIR GYNAECOL FE, V80, P188
   KVIST M, 1994, SPORTS MED, V18, P173, DOI 10.2165/00007256-199418030-00004
   LEADBETTER WB, 1992, CLIN SPORT MED, V11, P533
   LEPPILAHTI J, 1991, ANN CHIR GYNAECOL FE, V80, P202
   Leppilahti J, 1996, ACTA ORTHOP SCAND, V67, P277, DOI 10.3109/17453679608994688
   Maffulli N, 1998, ARTHROSCOPY, V14, P840, DOI 10.1016/S0749-8063(98)70021-0
   Maffulli N, 2000, AM J SPORT MED, V28, P499, DOI 10.1177/03635465000280040901
   Maffulli N, 2004, J ROY SOC MED, V97, P472, DOI 10.1258/jrsm.97.10.472
   Maffulli N, 2000, AM J SPORT MED, V28, P857, DOI 10.1177/03635465000280061401
   Maffulli N, 1999, CLIN J SPORT MED, V9, P157, DOI 10.1097/00042752-199907000-00007
   Nestorson J, 2000, ACTA ORTHOP SCAND, V71, P64, DOI 10.1080/00016470052943928
   Nilsson-Helander K, 2007, AM J SPORT MED, V35, P421, DOI 10.1177/0363546506294856
   Nyyssonen T, 2008, SCAND J SURG, V97, P272, DOI 10.1177/145749690809700312
   Park DY, 2006, FOOT ANKLE INT, V27, P1086, DOI 10.1177/107110070602701215
   Park YH, 2017, INJURY, V48, P776, DOI 10.1016/j.injury.2017.01.020
   Raikin SM, 2013, FOOT ANKLE INT, V34, P475, DOI 10.1177/1071100713477621
   Selvanetti A, 1997, OPER TECHN SPORT MED, V5, P110, DOI 10.1016/S1060-1872(97)80031-7
   Tallon C, 2001, MED SCI SPORT EXER, V33, P1983, DOI 10.1097/00005768-200112000-00002
   van der Linden PD, 2003, ARCH INTERN MED, V163, P1801, DOI 10.1001/archinte.163.15.1801
   Yasui Y, 2017, BIOMED RES INT, V2017, DOI 10.1155/2017/7021862
   Young SW, 2014, J BONE JOINT SURG AM, V96A, P1073, DOI 10.2106/JBJS.M.00248
NR 31
TC 4
Z9 4
U1 0
U2 2
PU SPRINGER
PI NEW YORK
PA 233 SPRING ST, NEW YORK, NY 10013 USA
SN 0942-2056
EI 1433-7347
J9 KNEE SURG SPORT TR A
JI Knee Surg. Sports Traumatol. Arthrosc.
PD OCT
PY 2019
VL 27
IS 10
BP 3297
EP 3303
DI 10.1007/s00167-018-5172-3
PG 7
WC Orthopedics; Sport Sciences; Surgery
WE Science Citation Index Expanded (SCI-EXPANDED)
SC Orthopedics; Sport Sciences; Surgery
GA IZ5PR
UT WOS:000487136100028
PM 30267184
DA 2022-07-22
ER

PT J
AU Ufberg, J
   Harrigan, RA
   Cruz, T
   Perron, AD
AF Ufberg, J
   Harrigan, RA
   Cruz, T
   Perron, AD
TI Orthopedic pitfalls in the ED: Achilles tendon rupture
SO AMERICAN JOURNAL OF EMERGENCY MEDICINE
LA English
DT Article
DE Tendinopathy; Achilles tendon; tendon rupture
ID NON-SURGICAL TREATMENT
AB Achilles tendon rupture is a relatively uncommon occurrence in a general ED population. The history can be subtle, and physical findings may not be clear-cut. Prompt diagnosis and treatment of these injuries, however, is important to improved clinical outcome. The emergency physician needs to remain vigilant for this diagnosis to avoid this orthopedic pitfall. This review article examines the clinical presentation, diagnostic technique, and management options applicable to the emergency physician in the treatment of Achilles tendon rupture.
C1 Maine Med Ctr, Dept Emergency Med, Portland, ME 04102 USA.
   Temple Univ Hosp & Med Sch, Philadelphia, PA 19122 USA.
   Sch Med, Philadelphia, PA 19122 USA.
RP Perron, AD (通讯作者)，Maine Med Ctr, Dept Emergency Med, 22 Bramhall St, Portland, ME 04102 USA.
EM perrona@mmc.org
CR ARNER O, 1958, ACTA CHIR SCAND, V116, P484
   BESKIN JL, 1987, AM J SPORT MED, V15, P1, DOI 10.1177/036354658701500101
   CARDEN DG, 1987, J BONE JOINT SURG BR, V69, P416, DOI 10.1302/0301-620X.69B3.3294839
   CETTI R, 1993, AM J SPORT MED, V21, P791, DOI 10.1177/036354659302100606
   CETTI R, 1993, CLIN ORTHOP RELAT R, P215
   CHRISTENSEN I, 1953, ACTA CHIR SCAND, V106, P50
   COPELAND SA, 1990, ANN ROY COLL SURG, V72, P270
   HAGGMARK T, 1986, ORTHOPEDICS, V9, P160
   Haims AH, 2000, SKELETAL RADIOL, V29, P640, DOI 10.1007/s002560000273
   HAINES JF, 1983, ANN RHEUM DIS, V42, P652, DOI 10.1136/ard.42.6.652
   INGLIS AE, 1976, J BONE JOINT SURG AM, V58, P990, DOI 10.2106/00004623-197658070-00015
   Jacobs D, 1978, Am J Sports Med, V6, P107, DOI 10.1177/036354657800600302
   JOZSA L, 1989, AM J SPORT MED, V17, P338, DOI 10.1177/036354658901700305
   KAINBERGER FM, 1990, AM J ROENTGENOL, V155, P1031, DOI 10.2214/ajr.155.5.2120931
   KANNUS P, 1991, J BONE JOINT SURG AM, V73A, P1507, DOI 10.2106/00004623-199173100-00009
   LAGERGREN C, 1959, Acta Chir Scand, V116, P491
   LANDVATER SJ, 1992, CLIN SPORT MED, V11, P741
   LEA RB, 1972, J BONE JOINT SURG AM, VA 54, P1398, DOI 10.2106/00004623-197254070-00002
   Lindholm Arner O, 1959, ACTA CHIR SCAND, V239, P7
   NISTOR L, 1981, J BONE JOINT SURG AM, V63, P394, DOI 10.2106/00004623-198163030-00012
   OBRIEN T, 1984, J BONE JOINT SURG AM, V66A, P1099, DOI 10.2106/00004623-198466070-00018
   THOMPSON T, 1967, ANAT RES, V156, P126
   VANDERLINDEN PD, 2002, BRIT MED J, V3241, P306
   WILLS CA, 1986, CLIN ORTHOP RELAT R, P156
NR 24
TC 15
Z9 17
U1 0
U2 1
PU W B SAUNDERS CO-ELSEVIER INC
PI PHILADELPHIA
PA 1600 JOHN F KENNEDY BOULEVARD, STE 1800, PHILADELPHIA, PA 19103-2899 USA
SN 0735-6757
EI 1532-8171
J9 AM J EMERG MED
JI Am. J. Emerg. Med.
PD NOV
PY 2004
VL 22
IS 7
BP 596
EP 600
DI 10.1016/j.ajem.2004.09.007
PG 5
WC Emergency Medicine
WE Science Citation Index Expanded (SCI-EXPANDED)
SC Emergency Medicine
GA 886VN
UT WOS:000226261000019
PM 15666269
DA 2022-07-22
ER

PT J
AU Eidelman, M
   Nachtigal, A
   Katzman, A
   Bialik, V
AF Eidelman, M
   Nachtigal, A
   Katzman, A
   Bialik, V
TI Acute rupture of achilles tendon in a 7-year-old girl
SO JOURNAL OF PEDIATRIC ORTHOPAEDICS-PART B
LA English
DT Article
DE achilles; avulsion; rupture; tendon
AB Ruptures of Achilles tendon are a relatively common injury. Ruptures typically occur after the age of 30 years, and are more frequent in people of middle age or older. In children, rupture of the tendon is usually due to avulsion of the tendon from the calcaneus but not within the tendon itself. We describe a 7-year-old girl successfully treated conservatively for acute tear of her Achilles tendon. No general predisposing disease was found. We were unable to find any description of an acute rupture of the Achilles tendon in a child under the age of 10 years. (c) 2004 Lippincott Williams R Wilkins.
C1 Rambam Med Ctr, Pediat Orthoped Unit, IL-31096 Haifa, Israel.
   Rambam Med Ctr, Dept Diagnost Radiol, IL-31096 Haifa, Israel.
   Technion Israel Inst Technol, Fac Med, Haifa, Israel.
RP Eidelman, M (通讯作者)，Rambam Med Ctr, Pediat Orthoped Unit, POB 9602, IL-31096 Haifa, Israel.
EM markeid@yahoo.com
CR ARNER O, 1959, ACTA CHIR SCAND    S, V239, P1
   Maffulli N, 1999, J BONE JOINT SURG AM, V81A, P1019, DOI 10.2106/00004623-199907000-00017
   RALSTON EL, 1971, J TRAUM, V11, P15, DOI 10.1097/00005373-197101000-00001
   Staheli LT., 2001, PRACTICE PEDIAT ORTH
NR 4
TC 8
Z9 9
U1 1
U2 2
PU LIPPINCOTT WILLIAMS & WILKINS
PI PHILADELPHIA
PA TWO COMMERCE SQ, 2001 MARKET ST, PHILADELPHIA, PA 19103 USA
SN 1060-152X
EI 1473-5865
J9 J PEDIATR ORTHOP B
JI J. Pediatr. Orthop.-Part B
PD JAN
PY 2004
VL 13
IS 1
BP 32
EP 33
DI 10.1097/01.bpb.0000078723.48512.7e
PG 2
WC Orthopedics; Pediatrics
WE Science Citation Index Expanded (SCI-EXPANDED)
SC Orthopedics; Pediatrics
GA 916UH
UT WOS:000228411200006
PM 15091256
DA 2022-07-22
ER

PT J
AU Suchak, AA
   Bostick, G
   Reid, D
   Blitz, S
   Jomha, N
AF Suchak, AA
   Bostick, G
   Reid, D
   Blitz, S
   Jomha, N
TI The incidence of Achilles tendon ruptures in Edmonton, Canada
SO FOOT & ANKLE INTERNATIONAL
LA English
DT Article
DE Achilles tendon; epidemiology; incidence; rupture
AB Background: The incidence of Achilles tendon ruptures specific to the North American population has not been previously reported and current epidemiological data are primarily reported from European communities. The purpose of this study was to determine the incidence of Achilles tendon ruptures in the city of Edmonton, Alberta, Canada, and to compare this data to those reported in European studies. Methods: A retrospective chart review from all five acute care hospitals in Edmonton from 1998 to 2002 (inclusive) were reviewed for Achilles tendon ruptures. Data such as gender, age, side, mechanism of injury, and season of injury were obtained. Results: The incidence of Achilles tendon ruptures ranged from an annual average of 5.5 ruptures to 9.9 ruptures per 100,000 inhabitants with an overall mean of 8.3 ruptures per 100,000 people. There was a statistically significant difference in Achilles tendon ruptures over the last two study years for both genders (women, p < 0.02; men, p < 0.03). The mean age for an Achilles tendon rupture was 40.6 years for men and 44.5 years for women. The Achilles tendon ruptures occurred most frequently in the 30 to 39 and 40 to 49 year old age groups in both men and women, respectively (p < 0.02). Most ruptures occurred in the spring season, but there was no statistical difference in the incidence of Achilles tendon ruptures by season (p > 0.05). Conclusions: The incidence of Achilles tendon ruptures in this community was comparable to those reported in European communities (range 6 to 37 ruptures per 100,000 people), although a bimodal age distribution of rupture previously reported was not observed in this study.
C1 Univ Alberta, Dept Orthopaed Surg, Edmonton, AB T6G 2B7, Canada.
RP Jomha, N (通讯作者)，Univ Alberta, Dept Orthopaed Surg, Edmonton, AB T6G 2B7, Canada.
EM nadr@interbaun.com
CR CETTI R, 1993, CLIN ORTHOP RELAT R, P215
   Houshian S, 1998, INJURY, V29, P651, DOI 10.1016/S0020-1383(98)00147-8
   JARVINEN M, 1992, CLIN SPORT MED, V11, P493
   JOZSA L, 1989, AM J SPORT MED, V17, P338, DOI 10.1177/036354658901700305
   Leppilahti J, 1996, ACTA ORTHOP SCAND, V67, P277, DOI 10.3109/17453679608994688
   Levi N, 1997, INJURY, V28, P311, DOI 10.1016/S0020-1383(96)00200-8
   Maffulli N, 1999, CLIN J SPORT MED, V9, P157, DOI 10.1097/00042752-199907000-00007
   Moller A, 1996, ACTA ORTHOP SCAND, V67, P479, DOI 10.3109/17453679608996672
NR 8
TC 133
Z9 146
U1 0
U2 11
PU SAGE PUBLICATIONS INC
PI THOUSAND OAKS
PA 2455 TELLER RD, THOUSAND OAKS, CA 91320 USA
SN 1071-1007
EI 1944-7876
J9 FOOT ANKLE INT
JI Foot Ankle Int.
PD NOV
PY 2005
VL 26
IS 11
BP 932
EP 936
DI 10.1177/107110070502601106
PG 5
WC Orthopedics
WE Science Citation Index Expanded (SCI-EXPANDED)
SC Orthopedics
GA 980YG
UT WOS:000233053500006
PM 16309606
DA 2022-07-22
ER

PT J
AU Al-Mouazzen, L
   Rajakulendran, K
   Najefi, A
   Ahad, N
AF Al-Mouazzen, Louay
   Rajakulendran, Karthig
   Najefi, Ali
   Ahad, Nurul
TI Percutaneous repair followed by accelerated rehabilitation for acute
   Achilles tendon ruptures
SO JOURNAL OF ORTHOPAEDIC SURGERY
LA English
DT Article
DE Achilles tendon; rupture
ID POSTOPERATIVE REHABILITATION; NONOPERATIVE TREATMENT; MOBILIZATION;
   SCORE
AB Purpose. To evaluate the outcome after percutaneous repair followed by accelerated rehabilitation for acute Achilles tendon ruptures.
   Methods. 21 men and 9 women (mean age, 41 years) underwent percutaneous repair by a single senior surgeon for acute Achilles tendon ruptures, followed by early weight bearing and accelerated rehabilitation. Outcome measures included the Achilles tendon re-rupture rate, the Achilles tendon total rupture score (ATRS) at 3 and 6 months, the incidence of sural nerve injury, wound infection, wound dehiscence, patient satisfaction, and the time to return to pre-rupture activity.
   Results. The mean follow-up period was 12.5 months. The mean ATRS score improved from 57.65 at 3 months to 86.95 at 6 months (p<0.001). No patient had intra-operative complications, tendon re-rupture, sural nerve injury, wound dehiscence, or deep infection. Two patients developed a superficial wound infection, which was resolved with oral flucloxacillin. Two patients had a mass at the transverse incision, but neither had any symptoms or functional restriction. All patients were able to bear full weight comfortably without the walker boot at 8 weeks, and return to their work by 3 months. The mean time to return to pre-rupture activity, including sports, was 10.4 months. The mean satisfaction rate was 87% at 6 months.
   Conclusion. Percutaneous repair of the Achilles tendon followed by early weight bearing and accelerated rehabilitation achieves good functional outcome.
C1 [Al-Mouazzen, Louay] North Bristol NHS Fdn Trust, Dept Orthopaed, Bristol, Avon, England.
   [Rajakulendran, Karthig; Najefi, Ali] Royal Natl Orthopaed Hosp, Dept Orthopaed, Stanmore HA7 4LP, Middx, England.
   [Ahad, Nurul] Queens Hosp, Dept Trauma & Orthopaed, Romford, Essex, England.
RP Al-Mouazzen, L (通讯作者)，17 Rushy Way, Bristol BS16 7ER, Avon, England.
EM almouazzen@yahoo.com
CR Barrie KA, 2000, J HAND SURG-AM, V25A, P714, DOI 10.1053/jhsu.2000.9414
   Buchgraber A, 1997, CLIN ORTHOP RELAT R, P113
   Carmont MR, 2008, KNEE SURG SPORT TR A, V16, P199, DOI 10.1007/s00167-007-0411-z
   Carmont MR, 2011, BMC SPORTS SCI MED R, V3, DOI 10.1186/1758-2555-3-28
   CETTI R, 1994, CLIN ORTHOP RELAT R, P155
   Costa ML, 2003, INJURY, V34, P874, DOI 10.1016/S0020-1383(02)00205-X
   Haji A, 2004, FOOT ANKLE INT, V25, P215, DOI 10.1177/107110070402500404
   Hrnack SA, 2012, FOOT ANKLE INT, V33, P848, DOI 10.3113/FAI.2012.0848
   Jiang N, 2012, INT ORTHOP, V36, P765, DOI 10.1007/s00264-011-1431-3
   Kangas J, 2003, J TRAUMA, V54, P1171, DOI 10.1097/01.TA.0000047945.20863.A2
   Kearney RS, 2012, HEALTH QUAL LIFE OUT, V10, DOI 10.1186/1477-7525-10-24
   Keating JF, 2011, J BONE JOINT SURG BR, V93B, P1071, DOI 10.1302/0301-620X.93B8.25998
   Khan RJK, 2010, COCHRANE DB SYST REV, DOI 10.1002/14651858.CD003674.pub4
   KLEIN W, 1991, Chirurgia degli Organi di Movimento, V76, P223
   Leppilahti J, 1998, SPORTS MED, V25, P79, DOI 10.2165/00007256-199825020-00002
   Leppilahti J, 1998, CLIN ORTHOP RELAT R, P152
   Lim J, 2001, FOOT ANKLE INT, V22, P559, DOI 10.1177/107110070102200705
   Maes Renaud, 2006, Acta Orthop Belg, V72, P179
   Maffulli N, 2003, AM J SPORT MED, V31, P692, DOI 10.1177/03635465030310051001
   Maffulli N, 2011, FOOT ANKLE INT, V32, P9, DOI 10.3113/FAI.2011.0009
   Maffulli N, 2011, ARCH ORTHOP TRAUM SU, V131, P33, DOI 10.1007/s00402-010-1097-0
   Maffulli N, 2010, CLIN ORTHOP RELAT R, V468, P1039, DOI 10.1007/s11999-009-0944-1
   Majewski M, 2008, DISABIL REHABIL, V30, P1726, DOI 10.1080/09638280701786831
   MARTI R, 1974, HELV CHIR ACTA, V41, P293
   Moller M, 2001, J BONE JOINT SURG BR, V83B, P843
   Mortensen NHM, 1999, J BONE JOINT SURG AM, V81A, P983, DOI 10.2106/00004623-199907000-00011
   Nilsson-Helander K, 2007, AM J SPORT MED, V35, P421, DOI 10.1177/0363546506294856
   Nilsson-Helander K, 2010, AM J SPORT MED, V38, P2186, DOI 10.1177/0363546510376052
   Olsson N, 2011, KNEE SURG SPORT TR A, V19, P1385, DOI 10.1007/s00167-011-1511-3
   Ozkaya U, 2009, INJURY, V40, P669, DOI 10.1016/j.injury.2008.10.033
   Rettig AC, 2005, AM J SPORT MED, V33, P119, DOI 10.1177/0363546504268720
   ROWLEY DI, 1982, INJURY, V14, P252, DOI 10.1016/0020-1383(82)90138-3
   SIMMONDS F A, 1957, Practitioner, V179, P56
   Soroceanu A, 2012, J BONE JOINT SURG AM, V94A, P2136, DOI 10.2106/JBJS.K.00917
   Talbot JC, 2012, J FOOT ANKLE SURG, V51, P147, DOI 10.1053/j.jfas.2011.10.045
   Wagnon Raymond, 2005, J Foot Ankle Surg, V44, P437, DOI 10.1053/j.jfas.2005.07.015
   Webb J, 2000, FOOT ANKLE INT, V21, P475, DOI 10.1177/107110070002100604
   Willits K, 2010, J BONE JOINT SURG AM, V92A, P2767, DOI 10.2106/JBJS.I.01401
NR 38
TC 6
Z9 6
U1 0
U2 4
PU SAGE PUBLICATIONS LTD
PI LONDON
PA 1 OLIVERS YARD, 55 CITY ROAD, LONDON EC1Y 1SP, ENGLAND
SN 1022-5536
EI 2309-4990
J9 J ORTHOP SURG-HONG K
JI J. Orthop. Surg.
PY 2015
VL 23
IS 3
BP 352
EP 356
DI 10.1177/230949901502300320
PG 5
WC Orthopedics; Surgery
WE Science Citation Index Expanded (SCI-EXPANDED)
SC Orthopedics; Surgery
GA CZ9YT
UT WOS:000367454500020
PM 26715717
OA gold
DA 2022-07-22
ER

PT J
AU Krill, MK
   Hoffman, J
   Yang, J
   Hodax, JD
   Owens, BD
   Hewett, TE
AF Krill, Michael K.
   Hoffman, Joshua
   Yang, JaeWon
   Hodax, Johnathan D.
   Owens, Brett D.
   Hewett, Timothy E.
TI Previous foot injuries associated with a greater likelihood of Achilles
   tendon ruptures in professional American football players
SO PHYSICIAN AND SPORTSMEDICINE
LA English
DT Article
DE Achilles tendon rupture; football (American); previous injury history
ID ANTERIOR CRUCIATE LIGAMENT; UPPER EXTREMITY INJURIES; MUSCLE STRAINS;
   LEAGUE; NFL; EPIDEMIOLOGY; TENDINOPATHY; OUTCOMES; KNEE; TIME
AB Objectives: The purpose of this study was to build on current understanding of Achilles tendon (AT) ruptures in football through the examination of lower extremity injury rates (IR) in the season prior to AT rupture.
   Methods: An online review was conducted to document AT ruptures in the National Football League (NFL) from the 2010-11 through 2016-17 seasons. Player-specific information was gathered through the online search, injury reports, or video analysis. Injury incidence was recorded for each season. Descriptive statistics were calculated for all variables. IRs per 1000 athlete-exposures with 95% confidence intervals (CI) were established. Injury rate ratios (IRR) with 95% CI were calculated to evaluate differences in IR with statistical significance established at p < 0.05.
   Results: From the 2010-11 to 2016-17 NFL seasons, 109 AT ruptures were identified. 36 athletes participated in the NFL the season prior and sustained a total of 40 injuries. Thirty-two (32/40, 80%) of the injuries involved the lower extremity. The most frequent body parts injured in the NFL season prior to AT rupture were the knee (n = 8, 22.5%), upper leg and thigh (n = 7, 17.5%), lower leg and shin (n = 5, 12.5%), and ankle (n = 5, 12.5%). The overall IR for the AT rupture group was not significantly different than the NFL group (IRR: 0.94, 0.68-1.31 95% CI, p = 0.73). The AT rupture group demonstrated a significantly increased rate of foot injuries compared to the NFL cohort (IRR: 2.58, 1.16-5.77 95% CI, p = 0.02).
   Conclusion: There was an increased identified incidence of AT ruptures in the NFL from 2010 to 2017 compared to AT ruptures reported from 1997 to 2002. There was no significant difference in lower extremity IR the season prior to AT rupture. Only foot injuries demonstrated an increased IR the season prior to AT rupture compared to an NFL cohort. Eleven athletes sustained 2 AT ruptures and all 11 players sustained the subsequent AT rupture to the contralateral limb.
C1 [Krill, Michael K.; Hoffman, Joshua] Ohio State Univ, Wexner Med Ctr, Mot Anal & Performance Lab, James Crane Sports Med Inst, Columbus, OH 43210 USA.
   [Krill, Michael K.] Florida Atlantic Univ, Charles E Schmidt Coll Med, Boca Raton, FL 33431 USA.
   [Krill, Michael K.] Washington Univ, Div Neurorehabil, Dept Neurol, Phys Med & Rehabil, St Louis, MO USA.
   [Yang, JaeWon] Brown Univ, Warren Alpert Med Sch, Providence, RI 02912 USA.
   [Hodax, Johnathan D.; Owens, Brett D.] Brown Univ, Warren Alpert Med Sch, Dept Orthopaed Surg, Providence, RI 02912 USA.
   [Hewett, Timothy E.] Mayo Clin, Sports Med Ctr, Rochester, MN 55905 USA.
   [Hewett, Timothy E.] Mayo Clin, Dept Orthoped Surg, 200 First St SW, Rochester, MN 55905 USA.
   [Hewett, Timothy E.] Mayo Clin, Dept Physiol & Biomed Engn, Rochester, MN 55905 USA.
   [Hewett, Timothy E.] Mayo Clin, Dept Phys Med & Rehabil, Rochester, MN 55905 USA.
RP Hewett, TE (通讯作者)，Mayo Clin, Dept Orthoped Surg, 200 First St SW, Rochester, MN 55905 USA.
EM hewett.timothy@mayo.edu
RI Hewett, Timothy Edwin/GLT-1556-2022; Krill, Michael/AAF-7180-2020
OI Krill, Michael/0000-0002-5248-4786; Owens, Brett/0000-0002-9972-0096
CR Amin NH, 2013, AM J SPORT MED, V41, P1864, DOI 10.1177/0363546513490659
   Aroen A, 2004, SCAND J MED SCI SPOR, V14, P30, DOI 10.1111/j.1600-0838.2004.00344.x
   Arthur R, 2016, WALL STREET J   0229
   Carcia Christopher R, 2010, J Orthop Sports Phys Ther, V40, pA1, DOI 10.2519/jospt.2010.0305
   Carlisle JC, 2008, AM J SPORT MED, V36, P1945, DOI 10.1177/0363546508318198
   CLEMENT DB, 1984, AM J SPORT MED, V12, P179, DOI 10.1177/036354658401200301
   Cotchett MP, 2011, J FOOT ANKLE RES, V4, DOI 10.1186/1757-1146-4-5
   Egger AC, 2017, CURR REV MUSCULOSKE, V10, P72, DOI 10.1007/s12178-017-9386-7
   Elliott MCCW, 2011, AM J SPORT MED, V39, P843, DOI 10.1177/0363546510394647
   Frankewycz B, 2017, ARCH ORTHOP TRAUM SU, V137, P333, DOI 10.1007/s00402-017-2627-9
   Fredberg U, 2002, AM J SPORT MED, V30, P488, DOI 10.1177/03635465020300040701
   Ganestam A, 2016, KNEE SURG SPORT TR A, V24, P3730, DOI 10.1007/s00167-015-3544-5
   Hoffman J, 2016, MED SCI SPORT EXER, V48, P871, DOI 10.1249/01.mss.0000487610.94985.6f
   Holm C, 2015, SCAND J MED SCI SPOR, V25, pE1, DOI 10.1111/sms.12209
   Jarvinen Tero A H, 2005, Foot Ankle Clin, V10, P255, DOI 10.1016/j.fcl.2005.01.013
   Johnston JT, 2018, AM J SPORT MED, V46, P862, DOI 10.1177/0363546518756328
   Kirsch JM, 2018, CURR REV MUSCULOSKE, V11, P6, DOI 10.1007/s12178-018-9453-8
   Knobloch K, 2008, DISABIL REHABIL, V30, P1685, DOI 10.1080/09638280701786658
   Krill MK, 2017, PHYSICIAN SPORTSMED, V45, P259, DOI 10.1080/00913847.2017.1343652
   Kujala UM, 2005, CLIN J SPORT MED, V15, P133, DOI 10.1097/01.jsm.0000165347.55638.23
   Lawrence DW, 2015, ORTHOP J SPORTS MED, V3, DOI 10.1177/2325967115583653
   Lynch TS, 2013, AM J SPORT MED, V41, P2904, DOI 10.1177/0363546513504284
   Maffulli N, 1998, ARTHROSCOPY, V14, P840, DOI 10.1016/S0749-8063(98)70021-0
   Maffulli N, 2003, CLIN SPORT MED, V22, P675, DOI 10.1016/S0278-5919(03)00004-8
   Maffulli N, 2000, AM J SPORT MED, V28, P499, DOI 10.1177/03635465000280040901
   Mai HT, 2016, AM J SPORT MED, V44, P2255, DOI 10.1177/0363546516651426
   Mall NA, 2008, AM J SPORT MED, V36, P1938, DOI 10.1177/0363546508318197
   Mark-Christensen T, 2016, KNEE SURG SPORT TR A, V24, P1852, DOI 10.1007/s00167-014-3180-5
   Minhas SV, 2016, AM J SPORT MED, V44, P1056, DOI 10.1177/0363546515623028
   Myer GD, 2011, J ORTHOP SPORT PHYS, V41, P702, DOI 10.2519/jospt.2011.0107
   Orishimo KF, 2008, J FOOT ANKLE SURG, V47, P34, DOI 10.1053/j.jfas.2007.10.008
   Parekh Selene G, 2009, Foot Ankle Spec, V2, P283, DOI 10.1177/1938640009351138
   Quagliarella L, 2010, FOOT ANKLE INT, V31, P306, DOI 10.3113/FAI.2010.0306
   Rebolledo BJ, 2018, ARTHROSCOPY, V34, P1280, DOI 10.1016/j.arthro.2017.10.005
   Rees JD, 2009, AM J SPORT MED, V37, P1855, DOI 10.1177/0363546508324283
   Schrock JB, 2018, SPORTS HEALTH, V10, P453, DOI 10.1177/1941738118758312
   Sochacki Kyle R, 2018, Hand (N Y), V13, P682, DOI 10.1177/1558944717726137
   Valderrabano V, 2016, FOOT ANKLE SPORTS OR
   Vopat B, 2017, ORTHOP J SPORTS MED, V5, DOI 10.1177/2325967117723285
   Williams DSB, 2008, J ORTHOP SPORT PHYS, V38, P761, DOI 10.2519/jospt.2008.2911
   Yang J, FOOT ANKLE SUR UNPUB
NR 41
TC 5
Z9 5
U1 0
U2 3
PU TAYLOR & FRANCIS LTD
PI ABINGDON
PA 2-4 PARK SQUARE, MILTON PARK, ABINGDON OR14 4RN, OXON, ENGLAND
SN 0091-3847
EI 2326-3660
J9 PHYSICIAN SPORTSMED
JI Physician Sportsmed.
PY 2018
VL 46
IS 3
BP 342
EP 348
DI 10.1080/00913847.2018.1482188
PG 7
WC Primary Health Care; Orthopedics; Sport Sciences
WE Science Citation Index Expanded (SCI-EXPANDED)
SC General & Internal Medicine; Orthopedics; Sport Sciences
GA GS7WR
UT WOS:000443915700013
PM 29860909
DA 2022-07-22
ER

PT J
AU Syed, S
   Bhatti, A
   Shah, MM
AF Syed, Shakir
   Bhatti, Arshad
   Shah, Mian Munawar
TI Spontaneous Atraumatic Achilles Tendon Rupture in Healthy Individuals:
   Biomechanical Aspect
SO JCPSP-JOURNAL OF THE COLLEGE OF PHYSICIANS AND SURGEONS PAKISTAN
LA English
DT Article
DE Achilles tendon; Rupture; Spontaneous
ID OVERUSE INJURIES
AB Achilles tendon rupture bilaterally secondary to predisposing factors such as steroid, fluoroquinolones, SLE, RA etc. Other factors are well-described in the literature but this is an extremely rare phenomenon in the absence of risk factors. Available reports in healthy athletes, all had history of severe trauma. We report an unusual presentation of bilateral and unilateral spontaneous rupture of Achilles tendon in healthy lifeguards without any marked trauma. Knowledge of training environment will alert the trainers, athletes and surgeons to recognise training environment at risk and take preventive measures.
C1 [Syed, Shakir] Royal Shrewsbury Hosp, Dept Trauma & Orthopaed, Shrewsbury, Salop, England.
   [Bhatti, Arshad; Shah, Mian Munawar] Manor Teaching Hosp, Dept Trauma & Orthopaed, Walsall, W Midlands, England.
RP Syed, S (通讯作者)，Flat 6,Khan House,Bridge Rd, Birmingham B8 3SF, W Midlands, England.
EM drshakirsyed@yahoo.com
CR Baruah D R, 1984, Br J Sports Med, V18, P128
   Battista V, 2003, AM J SPORT MED, V31, P1007, DOI 10.1177/03635465030310060201
   GALLOWAY MT, 1992, CLIN SPORT MED, V11, P771
   Hayes Timothy, 2003, Bull Hosp Jt Dis, V61, P140
   Kotnis RA, 1999, J ACCID EMERG MED, V16, P378
   MAFFULLI N, 1991, Journal of Foot Surgery, V30, P529
   McGarvey WC, 1996, FOOT ANKLE INT, V17, P496, DOI 10.1177/107110079601700811
   PRICE AE, 1986, CLIN ORTHOP RELAT R, P249
   Van der Linden PD, 2001, ARTHRIT RHEUM-ARTHR, V45, P235, DOI 10.1002/1529-0131(200106)45:3<235::AID-ART254>3.0.CO;2-7
   Wilder RP, 2004, CLIN SPORT MED, V23, P55, DOI 10.1016/S0278-5919(03)00085-1
NR 10
TC 5
Z9 5
U1 0
U2 2
PU COLL PHYSICIANS & SURGEONS PAKISTAN
PI KARACHI
PA SEVENTH CENTRAL ST, DEFENCE HOUSING AUTHORITY, KARACHI, 75500, PAKISTAN
SN 1022-386X
EI 1681-7168
J9 JCPSP-J COLL PHYSICI
JI JCPSP-J. Coll. Physicians Surg.
PD MAR
PY 2009
VL 19
IS 3
BP 195
EP 197
PG 3
WC Medicine, General & Internal
WE Science Citation Index Expanded (SCI-EXPANDED)
SC General & Internal Medicine
GA 430OZ
UT WOS:000264999700016
PM 19268024
DA 2022-07-22
ER

PT J
AU El Khoury, L
   Posthumus, M
   Collins, M
   van der Merwe, W
   Handley, C
   Cook, J
   Raleigh, SM
AF El Khoury, L.
   Posthumus, M.
   Collins, M.
   van der Merwe, W.
   Handley, C.
   Cook, J.
   Raleigh, S. M.
TI ELN and FBN2 Gene Variants as Risk Factors for Two Sports-related
   Musculoskeletal Injuries
SO INTERNATIONAL JOURNAL OF SPORTS MEDICINE
LA English
DT Article
DE gene; achilles tendinopathy; tendon rupture; ligament rupture; injury
   prevention
ID CRUCIATE LIGAMENT RUPTURES; ACHILLES-TENDON PATHOLOGY; COL5A1 GENE;
   EXTRACELLULAR-MATRIX; ETHICAL STANDARDS; PATHOGENESIS; MUSCLE; TISSUE
AB The proteins ELN and FBN2 are important in extracellular matrix function. The ELN rs2071307 and FBN2 rs331079 gene variants have been associated with soft tissue pathologies. We aimed to determine whether these variants were predisposing factors for both Achilles tendinopathy (AT) and anterior cruciate ligament (ACL) ruptures. For the AT study, 135 cases (TEN group) and 239 asymptomatic controls were recruited. For the ACL rupture study our cohort consisted of 141 cases (ACL group) and 219 controls. Samples were genotyped for both the ELN rs2071307 and FBN2 rs331079 variants using TaqMan assays. Analysis of variance and chi-squared tests were used to determine whether either variant was associated with AT or ACL rupture with significance set at p < 0.05. The GG genotype of the FBN2 variant was significantly over-represented within the TEN group (p = 0.035; OR = 1.83; 95 % CI 1.04-3.25) compared to the CON group. We also found that the frequency of the G allele was significantly different between the TEN (p = 0.017; OR = 1.90; 95 % CI 1.11-3.27) and ACL groups (p = 0.047; OR = 1.76; 95 % CI 1.00-3.10) compared to controls. The ELN rs207137 variant was not associated with either AT or ACL rupture. In conclusion, DNA sequence variation within the FBN2 gene is associated with both AT and ACL rupture.
C1 [El Khoury, L.; Raleigh, S. M.] Univ Northampton, Div Hlth & Life Sci, Northampton NN2 7AL, England.
   [Posthumus, M.; Collins, M.] Univ Cape Town, Human Biol, UCT MRC Res Unit Exercise Sci & Sports Med, ZA-7925 Cape Town, South Africa.
   [van der Merwe, W.] Sports Sci Orthopaed Clin, Sports Sci, Cape Town, South Africa.
   [Handley, C.] La Trobe Univ, Sch Publ Hlth & Human Biosci, Bundoora, Vic 3086, Australia.
   [Handley, C.] La Trobe Univ, Musculoskeletal Res Ctr, Bundoora, Vic 3086, Australia.
   [Cook, J.] Monash Univ, Dept Physiotherapy, Clayton, Vic 3800, Australia.
RP Raleigh, SM (通讯作者)，Univ Northampton, Hlth & Life Sci, Boughton Green Rd, Northampton NN2 7AL, England.
EM stuart.raleigh@northampton.ac.uk
RI Khoury, Louis El/R-7232-2019; Collins, Malcolm/G-4046-2011
OI Khoury, Louis El/0000-0002-8147-435X; Collins,
   Malcolm/0000-0002-2564-0480; raleigh, stuart/0000-0002-8882-0040
CR BERGET SM, 1977, P NATL ACAD SCI USA, V74, P3171, DOI 10.1073/pnas.74.8.3171
   Charbonneau Noe L, 2004, Birth Defects Res C Embryo Today, V72, P37, DOI 10.1002/bdrc.20000
   Collins M, 2009, MED SPORT SCI, V54, P136, DOI 10.1159/000235701
   Cook CS, 1996, EUR J APPL PHYSIOL O, V72, P380, DOI 10.1007/BF00599700
   El Khoury L, 2013, J SCI MED SPORT, V16, P493, DOI 10.1016/j.jsams.2013.02.006
   Ellis SG, 2012, AM J CARDIOL, V110, P88, DOI 10.1016/j.amjcard.2012.02.053
   Gianotti SM, 2009, J SCI MED SPORT, V12, P622, DOI 10.1016/j.jsams.2008.07.005
   Gosline J, 2002, PHILOS T R SOC B, V357, P121, DOI 10.1098/rstb.2001.1022
   Gupta PA, 2002, HUM MUTAT, V19, P39, DOI 10.1002/humu.10017
   Harriss DJ, 2013, INT J SPORTS MED, V34, P1025, DOI 10.1055/s-0033-1358756
   Harriss DJ, 2011, INT J SPORTS MED, V32, P819, DOI 10.1055/s-0031-1287829
   Jarvinen Tero A H, 2005, Foot Ankle Clin, V10, P255, DOI 10.1016/j.fcl.2005.01.013
   Jelinsky SA, 2011, J ORTHOP RES, V29, P1604, DOI 10.1002/jor.21427
   LAHIRI DK, 1991, NUCLEIC ACIDS RES, V19, P5444, DOI 10.1093/nar/19.19.5444
   Ljungqvist A, 2008, CLIN SPORT MED, V27, P231, DOI 10.1016/j.csm.2007.10.007
   Mokone GG, 2006, SCAND J MED SCI SPOR, V16, P19, DOI 10.1111/j.1600-0838.2005.00439.x
   Mokone GG, 2005, AM J SPORT MED, V33, P1016, DOI 10.1177/0363546504271986
   Ng PC, 2003, NUCLEIC ACIDS RES, V31, P3812, DOI 10.1093/nar/gkg509
   Parkkari J, 2008, BRIT J SPORT MED, V42, P422, DOI 10.1136/bjsm.2008.046185
   Perneger TV, 1998, BRIT MED J, V316, P1236, DOI 10.1136/bmj.316.7139.1236
   Posthumus M, 2009, BRIT J SPORT MED, V43, P352, DOI 10.1136/bjsm.2008.056150
   Posthumus M, 2011, SCAND J MED SCI SPOR, P321
   Posthumus M, 2010, RHEUMATOLOGY, V49, P2090, DOI 10.1093/rheumatology/keq072
   Posthumus M, 2010, BRIT J SPORT MED, V44, P1160, DOI 10.1136/bjsm.2009.060756
   Posthumus M, 2009, AM J SPORT MED, V37, P2234, DOI 10.1177/0363546509338266
   Pruna R, 2015, MED CLIN-BARCELONA, V144, P105, DOI 10.1016/j.medcli.2013.09.026
   Puddu G, 1976, Am J Sports Med, V4, P145, DOI 10.1177/036354657600400404
   Radermecker M A, 2003, Interact Cardiovasc Thorac Surg, V2, P389, DOI 10.1016/S1569-9293(03)00107-5
   Raleigh SM, 2013, INT J SPORTS MED, V34, P364, DOI 10.1055/s-0032-1316361
   Raleigh SM, 2009, BRIT J SPORT MED, V43, P514, DOI 10.1136/bjsm.2008.053892
   Robinson PN, 2000, J MED GENET, V37, P9, DOI 10.1136/jmg.37.1.9
   ROSE JR, 1993, FASEB J, V7, pA1208, DOI 10.1096/fasebj.7.13.8405806
   Ruigrok YM, 2006, HUM MOL GENET, V15, P3361, DOI 10.1093/hmg/ddl412
   Saracini C, 2012, J VASC SURG, V55, P171, DOI 10.1016/j.jvs.2011.07.051
   September AV, 2009, BRIT J SPORT MED, V43, P357, DOI 10.1136/bjsm.2008.048793
   ZHANG H, 1995, J CELL BIOL, V129, P1165, DOI 10.1083/jcb.129.4.1165
NR 36
TC 20
Z9 21
U1 1
U2 29
PU GEORG THIEME VERLAG KG
PI STUTTGART
PA RUDIGERSTR 14, D-70469 STUTTGART, GERMANY
SN 0172-4622
EI 1439-3964
J9 INT J SPORTS MED
JI Int. J. Sports Med.
PD APR
PY 2015
VL 36
IS 4
BP 333
EP 337
DI 10.1055/s-0034-1390492
PG 5
WC Sport Sciences
WE Science Citation Index Expanded (SCI-EXPANDED)
SC Sport Sciences
GA CE4YY
UT WOS:000351837700011
PM 25429546
OA Green Accepted
DA 2022-07-22
ER

PT J
AU Park, YH
   Jeong, SM
   Choi, GW
   Kim, HJ
AF Park, Young Hwan
   Jeong, Seong Min
   Choi, Gi Won
   Kim, Hak Jun
TI How early must an acute Achilles tendon rupture be repaired?
SO INJURY-INTERNATIONAL JOURNAL OF THE CARE OF THE INJURED
LA English
DT Article
DE Acute achilles tendon rupture; Optimal surgical timing; Early achilles
   tendon repair
ID SURGICAL REPAIR; MANAGEMENT; SCORE
AB An acute Achilles tendon rupture is the most common tendon rupture of the lower extremities, yet the optimal timing for an early surgical repair is unclear. To identify the optimal time for an early surgical repair with favorable results, we evaluated the isokinetic muscle strength and clinical outcomes of early surgical repairs of acute Achilles tendon ruptures performed at different time points after injury. Between January 2011 and July 2015, a total of 65 patients underwent an acute Achilles tendon rupture repair within 1 week after injury. To compare the outcomes at different time points post-injury, we divided patients into 3 groups: group 1, surgical treatment at <= 24 h; group 2, surgery at >= 24 h and <= 48 h; and group 3, surgery at >= 48 hours and <= 1 week. The isokinetic muscle strength in both ankles were measured using a Cybex dynamometer, and the Achilles tendon total rupture score, the modified Tegner scoring system, the visual analog scale was used to assess clinical outcomes. Kruskal-Wallis and Fisher's exact tests were used to compare multiple results in the 3 groups. No significant differences were found among the groups in terms of ankle isokinetic muscle strength or clinical outcome scores (P > 0.05). The complication rate was low in all groups. There were no significant differences in isokinetic muscle strength or clinical outcomes following acute Achilles tendon rupture repairs performed within 1 week after injury. (C) 2017 Elsevier Ltd. All rights reserved.
C1 [Park, Young Hwan; Jeong, Seong Min; Kim, Hak Jun] Korea Univ, Guro Hosp, Dept Orthoped Surg, 148 Gurodong Ro, Seoul, South Korea.
   [Choi, Gi Won] Korea Univ, Ansan Hosp, Dept Orthoped Surg, Ansan, South Korea.
RP Kim, HJ (通讯作者)，Korea Univ, Guro Hosp, Dept Orthoped Surg, 148 Gurodong Ro, Seoul, South Korea.
EM hjunkimos@gmail.com
RI Kim, Hak Jun/AAR-5929-2020; Choi, Gi Won/AAX-7187-2020
OI Kim, Hak Jun/0000-0003-3633-6174;
CR BESKIN JL, 1987, AM J SPORT MED, V15, P1, DOI 10.1177/036354658701500101
   Bhattacharyya M, 2009, INT ORTHOP, V33, P151, DOI 10.1007/s00264-008-0564-5
   BOYDEN EM, 1995, CLIN ORTHOP RELAT R, P150
   Choi GW, 2016, KNEE SURG SPORTS TRA
   Flint JH, 2014, AM J SPORT MED, V42, P235, DOI 10.1177/0363546513490656
   Halasi T, 2004, AM J SPORT MED, V32, P899, DOI 10.1177/0363546503262181
   Inglis AE, 1981, CLIN ORTHOP RELAT R, V16
   KAKIUCHI M, 1995, J BONE JOINT SURG BR, V77B, P60, DOI 10.1302/0301-620X.77B1.7822398
   Khan RJ, 2010, COCHRANE DB SYST REV, V9, P1
   Lantto I, 2015, AM J SPORT MED, V43, P2302, DOI 10.1177/0363546515591267
   Leppilahti J, 1996, ACTA ORTHOP SCAND, V67, P277, DOI 10.3109/17453679608994688
   Lim J, 2001, FOOT ANKLE INT, V22, P559, DOI 10.1177/107110070102200705
   McComis GP, 1997, J BONE JOINT SURG AM, V79A, P1799, DOI 10.2106/00004623-199712000-00005
   MURPHY PG, 1994, BIOCHEM CELL BIOL, V72, P403, DOI 10.1139/o94-054
   Neumayer F, 2010, ARCH ORTHOP TRAUM SU, V130, P363, DOI 10.1007/s00402-009-0865-1
   Ng E S, 2006, J Orthop Surg (Hong Kong), V14, P142
   Nilsson-Helander K, 2007, AM J SPORT MED, V35, P421, DOI 10.1177/0363546506294856
   NISTOR L, 1981, J BONE JOINT SURG AM, V63, P394, DOI 10.2106/00004623-198163030-00012
   Olsson N, 2013, AM J SPORT MED, V41, P2867, DOI 10.1177/0363546513503282
   Rosenbaum Andrew J, 2010, HSS J, V6, P164, DOI 10.1007/s11420-009-9152-5
   Sharma P, 2005, SURG-J R COLL SURG E, V3, P309, DOI 10.1016/S1479-666X(05)80109-X
   Tegner Y, 1985, CLIN ORTHOP RELAT R, V4, P3
   Uquillas CA, 2015, J BONE JOINT SURG AM, V97A, P1187, DOI 10.2106/JBJS.O.00002
   Wallace RGH, 2004, J BONE JOINT SURG AM, V86A, P1198, DOI 10.2106/00004623-200406000-00011
   Wilkins R, 2012, AM J SPORT MED, V40, P2154, DOI 10.1177/0363546512453293
   Woo SLY, 1999, CLIN ORTHOP RELAT R, pS312
NR 26
TC 14
Z9 16
U1 0
U2 2
PU ELSEVIER SCI LTD
PI OXFORD
PA THE BOULEVARD, LANGFORD LANE, KIDLINGTON, OXFORD OX5 1GB, OXON, ENGLAND
SN 0020-1383
EI 1879-0267
J9 INJURY
JI Injury-Int. J. Care Inj.
PD MAR
PY 2017
VL 48
IS 3
BP 776
EP 780
DI 10.1016/j.injury.2017.01.020
PG 5
WC Critical Care Medicine; Emergency Medicine; Orthopedics; Surgery
WE Science Citation Index Expanded (SCI-EXPANDED)
SC General & Internal Medicine; Emergency Medicine; Orthopedics; Surgery
GA EQ5CA
UT WOS:000398097700032
PM 28088376
DA 2022-07-22
ER

PT J
AU Il Chun, D
   Lee, S
   Won, SH
   Cho, J
AF Il Chun, Dong
   Lee, Sanghyeon
   Won, Sung Hun
   Cho, Jaeho
TI Fatal pulmonary thromboembolism after Achilles tendon open repair A rare
   case report
SO MEDICINE
LA English
DT Article
DE Achilles tendon rupture; pulmonary thromboembolism; venous
   thromboembolism
ID DEEP-VEIN THROMBOSIS; VENOUS THROMBOEMBOLISM; ORTHOPEDIC-SURGERY;
   THROMBOPROPHYLAXIS; PREVENTION; RUPTURE; VTE; ARTHROPLASTY;
   METAANALYSIS; RIVAROXABAN
AB Rationale: The operative procedure for Achilles tendon rupture is relatively simple, but venous thromboembolism is serious complication with a high incidence after Achilles tendon rupture. However, the guideline for thromboprophylaxis in Achilles tendon rupture is unclear.
   Patient concerns: The patient was 32-year-old male and underwent Achilles tendon open repair surgery. He was healthy and there are no abnormal findings other than Achilles tendon rupture. At 3 weeks after operation, the episode of loss of consciousness with convulsive movement occurred. The next day, suddenly cardiac arrest occurred.
   Diagnoses: Extensive pulmonary thromboembolism in both pulmonary arteries was identified in chest computed tomography and thrombus was also identified at the left popliteal vein on ultrasonography.
   Intervention: Anticoagulant therapy with heparin sodium was performed to manage the pulmonary thromboembolism.
   Outcomes: Brain swelling after ischemic brain damage, acute kidney injury, and pneumonia gradually occurred and aggravated. His condition became worse and he died about 2 weeks after the cardiac arrest episode.
   Lessons: Although the incidence of venous thromboembolism in Achilles tendon rupture is higher than that in lower leg injury patients, guideline for thromboprophylaxis is unclear. We suggest that thromboprophylaxis for Achilles tendon rupture should be considered and appropriate guidelines should be established.
C1 [Il Chun, Dong; Won, Sung Hun] Soonchunhyang Univ, Coll Med, Seoul Hosp, Dept Orthoped Surg, Seoul, South Korea.
   [Lee, Sanghyeon; Cho, Jaeho] Hallym Univ, Dept Orthopaed Surg, Chuncheon Sacred Heart Hosp, Chunchon, South Korea.
RP Cho, J (通讯作者)，Hallym Univ, Dept Orthopaed Surg, Chuncheon Sacred Heart Hosp, 77,Sakju Ro, Chuncheon Si 200704, Gangwon Do, South Korea.
EM hohotoy@nate.com
FU Soonchunhyang University Hospital
FX This work was supported by Soonchunhyang University Hospital. The
   authors report no conflicts of interest.
CR Bjornara BT, 2006, J BONE JOINT SURG BR, V88B, P386, DOI 10.1302/0301-620X.88B3.17207
   Calder JDF, 2016, KNEE SURG SPORT TR A, V24, P1409, DOI 10.1007/s00167-015-3976-y
   Domeij-Arverud E, 2015, BONE JOINT J, V97B, P675, DOI 10.1302/0301-620X.97B5.34581
   Eriksson BI, 2008, NEW ENGL J MED, V358, P2765, DOI 10.1056/NEJMoa0800374
   Falck-Ytter Y, 2012, CHEST, V141, pE278S, DOI 10.1378/chest.11-2404
   Francis CW, 2013, J THROMB THROMBOLYS, V35, P359, DOI 10.1007/s11239-013-0889-9
   Khan RJK, 2005, J BONE JOINT SURG AM, V87A, P2202, DOI 10.2106/JBJS.D.03049
   Kyrle PA, 2005, LANCET, V365, P1163, DOI 10.1016/S0140-6736(05)71880-8
   Lapidus LJ, 2007, J ORTHOP TRAUMA, V21, P52, DOI 10.1097/01.bot.0000250741.65003.14
   Lassen MR, 2008, NEW ENGL J MED, V358, P2776, DOI 10.1056/NEJMoa076016
   Leppilahti J, 1996, ACTA ORTHOP SCAND, V67, P277, DOI 10.3109/17453679608994688
   Makhdom Asim M, 2013, Case Rep Orthop, V2013, P401968, DOI 10.1155/2013/401968
   Makhdom AM, 2013, J FOOT ANKLE SURG, V52, P584, DOI 10.1053/j.jfas.2013.03.001
   Mangwani Jitendra, 2015, Foot (Edinb), V25, P173, DOI 10.1016/j.foot.2014.07.007
   Meissner Mark H, 2005, Semin Intervent Radiol, V22, P147, DOI 10.1055/s-2005-921948
   Nilsson-Helander K, 2009, KNEE SURG SPORT TR A, V17, P1234, DOI 10.1007/s00167-009-0727-y
   Ramirez Miguel A, 2007, J Foot Ankle Surg, V46, P283, DOI 10.1053/j.jfas.2007.03.014
   Saragas Nikiforos Pandelis, 2011, Foot Ankle Surg, V17, P263, DOI 10.1016/j.fas.2010.12.002
   Yusof M I, 2007, Singapore Med J, V48, pe234
NR 19
TC 0
Z9 0
U1 0
U2 2
PU LIPPINCOTT WILLIAMS & WILKINS
PI PHILADELPHIA
PA TWO COMMERCE SQ, 2001 MARKET ST, PHILADELPHIA, PA 19103 USA
SN 0025-7974
EI 1536-5964
J9 MEDICINE
JI Medicine (Baltimore)
PD NOV
PY 2017
VL 96
IS 47
AR e8887
DI 10.1097/MD.0000000000008887
PG 4
WC Medicine, General & Internal
WE Science Citation Index Expanded (SCI-EXPANDED)
SC General & Internal Medicine
GA FP5FP
UT WOS:000417645700107
PM 29382016
OA Green Published, gold
DA 2022-07-22
ER

PT J
AU Turgut, A
   Gunal, I
   Maralcan, G
   Kose, N
   Gokturk, E
AF Turgut, A
   Gunal, I
   Maralcan, G
   Kose, N
   Gokturk, E
TI Endoscopy, assisted percutaneous repair of the Achilles tendon ruptures:
   a cadaveric and clinical study
SO KNEE SURGERY SPORTS TRAUMATOLOGY ARTHROSCOPY
LA English
DT Article
DE Achilles tendon ruptures; endoscopy assisted; percutaneous repair
AB Endoscopy-assisted percutaneous repair of Achilles tendon ruptures were investigated in a cadaveric and clinical study. Sixteen above-knee fresh amputation specimens in which different types of Achilles tendon ruptures were created were repaired percutaneously with the visualization of the tendon ends by endoscopy. Neither malalignment nor damage to the neurovascular structures was observed. Eleven patients were treated in this way. No reruptures, wound problems, or neurovascular injury were observed. All patients returned to daily activities 10-11 weeks after the repair. This technique seems to overcome certain problems of conservative, surgical, or percutaneous repair of the Achilles tendon ruptures.
C1 Osmangazi Univ Hosp, Dept Orthoped, TR-26480 Eskisehir, Turkey.
   Dokuz Eylul Univ Hosp, Dept Orthoped, TR-35340 Izmir, Turkey.
   Kocatepe Univ Hosp, Dept Orthoped, TR-03610 Afyon, Turkey.
RP Gunal, I (通讯作者)，Ruzgar Sok Cankaya Apt 51-20, TR-35330 Izmir, Turkey.
EM izge.gunal@deu.edu.tr
RI Kose, Nusret/AAG-6118-2019
OI Kose, Nusret/0000-0002-1517-9635
CR ARACIL J, 1992, FOOT ANKLE, V13, P350, DOI 10.1177/107110079201300610
   BOGOSAVLJEVIC M, 1997, J BONE JOINT SURG S2, V9, P180
   BRADLEY JP, 1990, AM J SPORT MED, V18, P188, DOI 10.1177/036354659001800213
   CARDEN DG, 1987, J BONE JOINT SURG BR, V69, P416, DOI 10.1302/0301-620X.69B3.3294839
   Esemenli BT, 1996, FOOT ANKLE INT, V17, P217, DOI 10.1177/107110079601700406
   GARRET WE, 1990, AM J SPORTS MED, V18, P195
   GILLIES H, 1970, J BONE JOINT SURG AM, VA 52, P337, DOI 10.2106/00004623-197052020-00015
   HOCKENBURY RT, 1990, FOOT ANKLE, V11, P67, DOI 10.1177/107110079001100202
   INGLIS AE, 1976, J BONE JOINT SURG AM, V58, P990, DOI 10.2106/00004623-197658070-00015
   KAKIUCHI M, 1995, J BONE JOINT SURG BR, V77B, P60, DOI 10.1302/0301-620X.77B1.7822398
   Lo IKY, 1997, CLIN J SPORT MED, V7, P207, DOI 10.1097/00042752-199707000-00010
   MA GWC, 1977, CLIN ORTHOP RELAT R, P247
   Maffulli N, 1999, J BONE JOINT SURG AM, V81A, P1019, DOI 10.2106/00004623-199907000-00017
   Maquirriain J, 1998, ARTHROSCOPY, V14, P182, DOI 10.1016/S0749-8063(98)70038-6
   NISTOR L, 1981, J BONE JOINT SURG AM, V63, P394, DOI 10.2106/00004623-198163030-00012
   OBRIEN T, 1984, J BONE JOINT SURG AM, V66A, P1099, DOI 10.2106/00004623-198466070-00018
   SCOTT BW, 1992, J BONE JOINT SURG BR, V74, P314, DOI 10.1302/0301-620X.74B2.1544978
   Steinfeld R, 1998, ARTHROSCOPY, V14, P212, DOI 10.1016/S0749-8063(98)70043-X
   Webb J, 2000, FOOT ANKLE INT, V21, P475, DOI 10.1177/107110070002100604
   WEBB J, 1992, J BONE JOINT SURG BR, V1, P877
NR 20
TC 17
Z9 17
U1 0
U2 2
PU SPRINGER
PI NEW YORK
PA 233 SPRING ST, NEW YORK, NY 10013 USA
SN 0942-2056
EI 1433-7347
J9 KNEE SURG SPORT TR A
JI Knee Surg. Sports Traumatol. Arthrosc.
PD MAR
PY 2002
VL 10
IS 2
BP 130
EP 133
DI 10.1007/s00167-001-0258-7
PG 4
WC Orthopedics; Sport Sciences; Surgery
WE Science Citation Index Expanded (SCI-EXPANDED)
SC Orthopedics; Sport Sciences; Surgery
GA 541TX
UT WOS:000175001600013
PM 11914773
DA 2022-07-22
ER

PT J
AU Bian, DL
   Wang, XM
   Huang, K
   Zhang, Z
AF Bian, Donglin
   Wang, Xuemei
   Huang, Kun
   Zhang, Zhen
TI Evaluation of Functional Recovery Following Achilles Tendon Ruptures by
   Shear Wave Elastography: A Before-and-After Study
SO IRANIAN JOURNAL OF RADIOLOGY
LA English
DT Article
DE Elastography; Ultrasonography; Achilles Tendon; Rupture
ID QUANTITATIVE ASSESSMENT; FEASIBILITY; FORCE
AB Background: Sonoelastography is extensively used for clinical diagnosis. Different types of elastography can be used to assess the elastic properties (stiffness) of tissues. For quantitative analyses, shear wave elastography (SWE) has been applied as a non-invasive method in the past decade. Today, SWE is widely used for diagnosing diseases. However, a limited number of studies have investigated musculoskeletal injuries and neoplastic diseases.
   Objectives: This study aimed to evaluate the postoperative functional recovery of Achilles tendon ruptures by SWE to determine the Achilles tendon function and provide reliable evidence for clinicians.
   Methods: SWE was used to measure elasticity in 67 patients with Achilles tendon ruptures. In the postoperative stage, 17 patients were lost to follow-up. The remaining 50 patients with Achilles tendon ruptures were examined. Each patient was followed-up for one, three, and six months postoperatively. The 0 degrees flexion of the ankle joint was considered as the standard position. The mean elasticity (E-mean) of the Achilles tendon was measured and recorded at different time points before and after surgery. Besides, the thickness and width of the Achilles tendon were measured at the corresponding time points.
   Results: The mean elasticity (Emean) of normal and ruptured Achilles tendons was 447.4 +/- 59.57 and 63.84 +/- 45.18 kPa, respectively in 50 patients with ruptured Achilles tendons before surgery. On the other hand, the E-mean values of ruptured Achilles tendons in one-, three-, and six-month follow-ups were 110.3 +/- 35.60, 183.4 +/- 46.08, and 279.1 +/- 48.18 kPa in 50 patients, respectively. Regarding the E-mean, the difference between the five time points was significant (F = 418.5, P < 0.01). With prolongation of postoperative recovery, the E-mean of the Achilles tendon gradually increased; the E-mean value significantly increased at six months after surgery.
   Conclusions: The quantitative analysis of the postoperative function of Achilles tendon, based on the E-mean value obtained by SWE, can be helpful in guiding clinicians to objectively examine the prognosis of patients.
C1 [Bian, Donglin; Wang, Xuemei; Huang, Kun; Zhang, Zhen] China Med Univ, Affiliated Hosp 1, Dept Ultrasound, Shenyang, Peoples R China.
RP Zhang, Z (通讯作者)，China Med Univ, Affiliated Hosp 1, Dept Ultrasound, Shenyang, Peoples R China.
EM 2662898158@qq.com
FU National Natural Science Foundation of China [81971639]
FX This study was supported in part by a grant (81971639) from the National
   Natural Science Foundation of China.
CR Akagi R, 2015, ACTA RADIOL, V56, P1487, DOI 10.1177/0284185114559765
   Arda K, 2011, AM J ROENTGENOL, V197, P532, DOI 10.2214/AJR.10.5449
   Au FWF, 2015, ACAD RADIOL, V22, P269, DOI 10.1016/j.acra.2014.10.007
   Aubry S, 2015, RADIOLOGY, V274, P821, DOI 10.1148/radiol.14140434
   Barr RG, 2014, RADIOL CLIN N AM, V52, P1145, DOI 10.1016/j.rcl.2014.07.002
   Botanlioglu H, 2013, SKELETAL RADIOL, V42, P659, DOI 10.1007/s00256-012-1520-4
   Brandenburg JE, 2015, J ULTRAS MED, V34, P663, DOI 10.7863/ultra.34.4.663
   Chen XM, 2013, J ULTRAS MED, V32, P449, DOI 10.7863/jum.2013.32.3.449
   Correas JM, 2015, RADIOLOGY, V275, P280, DOI 10.1148/radiol.14140567
   Efimenko N A, 2011, Klin Med (Mosk), V89, P64
   Garra BS, 2015, ABDOM IMAGING, V40, P680, DOI 10.1007/s00261-014-0305-8
   Han ZL, 2015, PHYS MED BIOL, V60, P3531, DOI 10.1088/0031-9155/60/9/3531
   Itoigawa Y, 2015, CLIN ANAT, V28, P213, DOI 10.1002/ca.22498
   Jung WS, 2015, ANN SURG ONCOL, V22, P111, DOI 10.1245/s10434-014-3627-4
   Koo TK, 2013, J BIOMECH, V46, P2053, DOI 10.1016/j.jbiomech.2013.05.016
   Nakamura M, 2014, ULTRASOUND MED BIOL, V40, P1991, DOI 10.1016/j.ultrasmedbio.2014.03.024
   Park AY, 2015, EUR J RADIOL, V84, P407, DOI 10.1016/j.ejrad.2014.11.019
   Zaleska-Dorobisz U, 2015, POSTEP HIG MED DOSW, V69, P221, DOI 10.5604/17322693.1140338
   Zhao H, 2011, ULTRASOUND MED BIOL, V37, P1884, DOI 10.1016/j.ultrasmedbio.2011.07.012
NR 19
TC 0
Z9 0
U1 2
U2 5
PU KOWSAR PUBL
PI HOENSBROEK
PA PATERSWEG 22,, HOENSBROEK, LIMBURG 6431 GC, NETHERLANDS
SN 1735-1065
EI 2008-2711
J9 IRAN J RADIOL
JI Iran. J. Radiol.
PD APR
PY 2021
VL 18
IS 2
AR e106415
DI 10.5812/iranjradiol.106415
PG 10
WC Radiology, Nuclear Medicine & Medical Imaging
WE Science Citation Index Expanded (SCI-EXPANDED)
SC Radiology, Nuclear Medicine & Medical Imaging
GA TL0YL
UT WOS:000674580900016
OA gold
DA 2022-07-22
ER

PT J
AU Mikashima, Y
   Kawamura, K
   Miyawaki, M
   Murakoshi, K
   Usami, N
   Momohara, S
AF Mikashima, Yoshinori
   Kawamura, Koichiro
   Miyawaki, Motoko
   Murakoshi, Kaoru
   Usami, Norio
   Momohara, Shigeki
TI Neglected Spontaneous Rupture of the Achilles Tendon in Elderly Patients
   With Rheumatoid Arthritis
SO JCR-JOURNAL OF CLINICAL RHEUMATOLOGY
LA English
DT Article
DE Achilles tendon; corticosteroid; rheumatoid arthritis; spontaneous
   rupture
ID STEROID-THERAPY
AB Spontaneous Achilles tendon rupture associated with rheumatoid arthritis (RA) is a very rare complication. In this report, we have experienced neglected spontaneous rupture of the Achilles tendon in 5 elderly RA patients, and report its clinical features and management. These patients had taken corticosteroids for a long time for RA control. Moreover, they did not show any signs, such as click or impact on the rear foot at the moment of the rupture, and had few complaints or obvious symptoms around their ankles or the rear of the foot after the rupture. This suggests that the lack of severe symptoms prevented the early diagnosis of the Achilles tendon rupture, and that this injury had been neglected during treatment for RA. Therefore, careful diagnosis of the Achilles tendon rupture is needed in elderly patients with RA, even if they have little or no history of trauma.
C1 [Mikashima, Yoshinori; Kawamura, Koichiro; Miyawaki, Motoko; Murakoshi, Kaoru; Momohara, Shigeki] Tokyo Womens Med Univ, Dept Orthopaed Surg, Inst Rheumatol, Shinjuku Ku, Tokyo 1620054, Japan.
   [Usami, Norio] Shiseikai Daini Hosp, Dept Orthopaed Surg, Setagaya Ku, Tokyo, Japan.
RP Kawamura, K (通讯作者)，Tokyo Womens Med Univ, Dept Orthoped Surg, Inst Rheumatol, Shinjuku Ku, 10-22 Kawada, Tokyo 1620054, Japan.
EM buchan2002da@hotmail.com
CR ABRAHAM E, 1975, J BONE JOINT SURG AM, VA 57, P253, DOI 10.2106/00004623-197557020-00019
   BARUAH DR, 1984, UNFALLHEILKUNDE, V87, P35
   Boer R, 2009, STRATEG TRAUMA LIMB, V4, P41, DOI 10.1007/s11751-009-0051-6
   Chiou YM, 2005, LUPUS, V14, P321, DOI 10.1191/0961203305lu2046cr
   GABEL S, 1994, FOOT ANKLE INT, V15, P512, DOI 10.1177/107110079401500912
   HAINES JF, 1983, ANN RHEUM DIS, V42, P652, DOI 10.1136/ard.42.6.652
   Jarvinen TAH, 2001, CURR OPIN RHEUMATOL, V13, P150
   KARPAKKA JA, 1992, AM J SPORT MED, V20, P262, DOI 10.1177/036354659202000305
   Kirschenbaum S E, 1980, J Foot Surg, V19, P4
   MATSUMOTO K, 1992, CLIN ORTHOP RELAT R, P235
   MELMED E P, 1965, J Bone Joint Surg Br, V47, P104
   OZAKI J, 1989, CLIN ORTHOP RELAT R, P204
   RASK MR, 1978, JAMA-J AM MED ASSOC, V239, P435, DOI 10.1001/jama.239.5.435
   STAUFFER RN, 1977, CLIN ORTHOP RELAT R, P189
   Theobald P, 2005, INJURY, V36, P1267, DOI 10.1016/j.injury.2005.02.012
NR 15
TC 4
Z9 5
U1 0
U2 1
PU LIPPINCOTT WILLIAMS & WILKINS
PI PHILADELPHIA
PA TWO COMMERCE SQ, 2001 MARKET ST, PHILADELPHIA, PA 19103 USA
SN 1076-1608
EI 1536-7355
J9 JCR-J CLIN RHEUMATOL
JI JCR-J. Clin. Rheumatol.
PD AUG
PY 2010
VL 16
IS 5
BP 221
EP 224
DI 10.1097/RHU.0b013e3181e96cd6
PG 4
WC Rheumatology
WE Science Citation Index Expanded (SCI-EXPANDED)
SC Rheumatology
GA 631HU
UT WOS:000280338200005
PM 20661068
DA 2022-07-22
ER

PT J
AU Gunaratne, R
   Chong, YC
   Heng, Y
   Hahn, J
   Lek, J
   Randazzo, A
   Brankov, B
AF Gunaratne, Rajitha
   Chong, Yu Chuan
   Heng, Yi
   Hahn, Jayhee
   Lek, Jeremy
   Randazzo, Amy
   Brankov, Boris
TI Chronic Achilles tendon rupture: a novel modification of surgical
   technique described by El Shewy
SO ANZ JOURNAL OF SURGERY
LA English
DT Article
DE Achilles tendon; chronic rupture; neglected rupture; novel modified
   surgical technique
ID REPAIR; RECONSTRUCTION; MANAGEMENT; GRAFT
AB Background: Chronic Achilles tendon rupture is commonly defined as a rupture presenting 6 weeks after the time of injury and operative management is recommended. This research aims to describe a novel modified surgical technique in the repair of chronic Achilles tendon rupture and to report the result of this technique.
   Methods: This is a retrospective study performed between January 2007 and January 2017, and a novel modification of El Shewy et al.'s surgical technique is described. Fifteen patients with chronic rupture of Achilles tendon repaired with the technique by a single experienced surgeon were identified. Patients were contacted via phone call and questionnaires completed. Achilles Tendon Rupture Score and pain score were assessed via questionnaires.
   Results: Thirteen patients were contacted and two patients were uncontactable. Ten patients were able to return to their premorbid level of function. Twelve patients were satisfied or very satisfied with the outcome. Only one patient was very dissatisfied with the outcome. The average Achilles Tendon Rupture score was 72 (n = 7, 54%). The average pain score was 1.23 (n = 13, 100%).
   Conclusions: This novel modified surgical technique demonstrated good functional outcomes and high levels of patient satisfaction in patient with chronic Achilles tendon rupture. It can be considered in the repair of chronic Achilles tendon rupture.
C1 [Gunaratne, Rajitha; Chong, Yu Chuan; Heng, Yi; Randazzo, Amy; Brankov, Boris] Fremantle Hosp, Orthopaed Dept, Alma St, Fremantle, WA 6160, Australia.
   [Gunaratne, Rajitha] Curtin Univ, Perth, WA, Australia.
   [Gunaratne, Rajitha; Heng, Yi; Hahn, Jayhee; Lek, Jeremy] Joondalup Hlth Campus, Orthopaed Dept, Perth, WA, Australia.
RP Chong, YC (通讯作者)，Fremantle Hosp, Orthopaed Dept, Alma St, Fremantle, WA 6160, Australia.
EM yuchuan15@gmail.com
OI GUNARATNE, RAJITHA/0000-0002-2054-5783; Chong, Yu
   Chuan/0000-0002-7828-3472
CR ABRAHAM E, 1975, J BONE JOINT SURG AM, VA 57, P253, DOI 10.2106/00004623-197557020-00019
   Cienfuegos A, 2013, J FOOT ANKLE SURG, V52, P95, DOI 10.1053/j.jfas.2012.06.006
   El Shewy MT, 2009, AM J SPORT MED, V37, P1570, DOI 10.1177/0363546509333009
   Goodman AD, 2018, ORTHOP J SPORTS MED, V6, DOI 10.1177/2325967117750105
   Guclu B, 2016, FOOT ANKLE INT, V37, P737, DOI 10.1177/1071100716642753
   Hadi M, 2013, BRIT MED BULL, V108, P95, DOI 10.1093/bmb/ldt019
   Jennings AG, 2002, J BONE JOINT SURG BR, V84B, P361, DOI 10.1302/0301-620X.84B3.11559
   Kearney RS, 2012, HEALTH QUAL LIFE OUT, V10, DOI 10.1186/1477-7525-10-24
   Lee JH, 2012, ANNU REP NMR SPECTRO, V75, P115, DOI 10.1016/B978-0-12-397018-3.00003-X
   Leslie Hamish D H, 2005, Foot Ankle Clin, V10, P357, DOI 10.1016/j.fcl.2005.01.009
   Maffulli N, 2005, CLIN J SPORT MED, V15, P56, DOI 10.1097/01.jsm.0000152714.05097.ef
   Maffulli Nicola, 2017, Open Orthop J, V11, P660, DOI 10.2174/1874325001711010660
   Maffulli N, 2010, AM J SPORT MED, V38, P2304, DOI 10.1177/0363546510376619
   Malagelada Francesc, 2016, Foot (Edinb), V28, P54, DOI 10.1016/j.foot.2016.05.001
   Miskulin Mladen, 2005, J Foot Ankle Surg, V44, P49, DOI 10.1053/j.jfas.2004.11.003
   Netto CD, 2019, FOOT ANKLE SURG, V25, P303, DOI 10.1016/j.fas.2017.12.003
   Nilsson-Helander K, 2007, AM J SPORT MED, V35, P421, DOI 10.1177/0363546506294856
   Periasamy M, 2019, INDIAN J PLAST SURG, V52, P109, DOI [10.1055/5-0039-1687923, 10.1055/s-0039-1687923]
   Porter DA, 1997, FOOT ANKLE INT, V18, P557, DOI 10.1177/107110079701800905
   Shoaib A, 2017, FOOT ANKLE SURG, V23, P179, DOI 10.1016/j.fas.2016.04.006
   Vadala A, 2014, INT J ORTHOP, V1, P57
   Wegrzyn J, 2010, INT ORTHOP, V34, P1187, DOI 10.1007/s00264-009-0859-1
   Wilcox DK, 2000, FOOT ANKLE INT, V21, P1004, DOI 10.1177/107110070002101204
NR 23
TC 0
Z9 0
U1 0
U2 3
PU WILEY
PI HOBOKEN
PA 111 RIVER ST, HOBOKEN 07030-5774, NJ USA
SN 1445-1433
EI 1445-2197
J9 ANZ J SURG
JI ANZ J. Surg.
PD JUL
PY 2021
VL 91
IS 7-8
BP 1447
EP 1450
DI 10.1111/ans.16921
EA MAY 2021
PG 4
WC Surgery
WE Science Citation Index Expanded (SCI-EXPANDED)
SC Surgery
GA UB5VN
UT WOS:000647967600001
PM 33956383
DA 2022-07-22
ER

PT J
AU Wiegand, N
   Vamhidy, L
   Kereskai, L
   Lorinczy, D
AF Wiegand, N.
   Vamhidy, L.
   Kereskai, L.
   Lorinczy, D.
TI Differential scanning calorimetric examination of the ruptured Achilles
   tendon in human
SO THERMOCHIMICA ACTA
LA English
DT Article
DE Achilles tendon rupture; DSC
ID DSC EXAMINATION; COLLAGEN; INJURIES
AB The Achilles tendon rupture is a common injury of the foot in middle age and physically active population in Europe. The aetiology of the degenerative changes in the collagen structures of the tendon which could be disposed for the rupture is still not clear. Our hypothesis was that before the injury there is a clear pathological abnormality in the tissue elements building up the Achilles tendon, which is responsible for the disease, and could be monitored besides the classical histological methods by differential scanning calorimetry.
   The thermal denaturation of human samples was monitored by a SETARAM Micro DSC-II calorimeter. All the experiments were performed between 0 and 100 degrees C. The heating rate was 0.3 K/min. DSC scans clearly demonstrated significant differences between the control and ruptured samples (control: T-m = 59.7 degrees C, T-1/2 = 1.4 degrees C and Delta H-cal = 8.54 J/g; ruptured: T-m = 62.75 degrees C, T-1/2 = 2.6 degrees C and Delta H-cal = 1.54 J/g). These observations could be explained with the structural alterations caused by the biochemical and structural processes.
   With our investigations we could demonstrate that DSC is a useful and well applicable method for the investigation of collagen tissue of the human Achilles tendon. We can prove with this method, that the earlier series of microtraumas which result a scar formation in the tendon tissue increases the thermal stability of collagen in ruptured tendon. (C) 2009 Elsevier B.V. All rights reserved.
C1 [Lorinczy, D.] Univ Pecs, Fac Med, Dept Biophys, H-7624 Pecs, Hungary.
   [Wiegand, N.; Vamhidy, L.] Univ Pecs, Fac Med, Dept Traumatol, H-7624 Pecs, Hungary.
   [Kereskai, L.] Univ Pecs, Fac Med, Dept Pathol, H-7624 Pecs, Hungary.
RP Lorinczy, D (通讯作者)，Univ Pecs, Fac Med, Dept Biophys, Szigeti Str 12, H-7624 Pecs, Hungary.
EM denes.lorinczy@aok.pte.hu
FU Hungarian Scientific Research Fund [CO-272]
FX The SETARAM Micro DSC-II was purchased with a grant (CO-272) from the
   Hungarian Scientific Research Fund (Denes Lorinczy).
CR Comerford EJ, 2005, J ORTHOP RES, V23, P61, DOI 10.1016/j.orthres.2004.05.016
   Comerford EJ, 2004, AM J VET RES, V65, P1136, DOI 10.2460/ajvr.2004.65.1136
   DEHL RE, 1970, SCIENCE, V170, P738, DOI 10.1126/science.170.3959.738
   Eriksen HA, 2002, J ORTHOPAED RES, V20, P1352, DOI 10.1016/S0736-0266(02)00064-5
   FRANCHI M, 2007, THESCIENTIFICWORLDJO, V30, P404
   Glazebrook MA, 2008, J ORTHOP RES, V26, P840, DOI 10.1002/jor.20546
   Jarvinen TAH, 2004, J ORTHOP RES, V22, P1303, DOI 10.1016/j.orthres.2004.04.003
   Kannus P, 2000, SCAND J MED SCI SPOR, V10, P312, DOI 10.1034/j.1600-0838.2000.010006312.x
   KAROUSOU E, 2008, CLIN ORTHOP RELAT RE
   Lorinczy D, 2009, J THERM ANAL CALORIM, V95, P713
   Maffulli N, 2000, AM J SPORT MED, V28, P857, DOI 10.1177/03635465000280061401
   MILES CA, 1995, J MOL BIOL, V245, P437, DOI 10.1006/jmbi.1994.0035
   MILES CA, 1994, EQUINE VET J, V26, P291, DOI 10.1111/j.2042-3306.1994.tb04389.x
   Nedvig K, 2009, J THERM ANAL CALORIM, V95, P775, DOI 10.1007/s10973-008-9416-1
   Selvanetti A, 1997, OPER TECHN SPORT MED, V5, P110, DOI 10.1016/S1060-1872(97)80031-7
   September AV, 2007, BRIT J SPORT MED, V41, P241, DOI 10.1136/bjsm.2006.033035
   Sohar G, 2007, J THERM ANAL CALORIM, V89, P853, DOI 10.1007/s10973-007-8256-8
   Strauss EJ, 2007, INJURY, V38, P832, DOI 10.1016/j.injury.2006.06.005
   WIEGAND N, 2006, MAGY TRAUMA, V49, P202
   Wilhelm F, 2007, J THERM ANAL CALORIM, V89, P863, DOI 10.1007/s10973-006-7927-1
   Willett TL, 2008, J ORTHOP RES, V26, P1605, DOI 10.1002/jor.20672
   Willett TL, 2007, ANN BIOMED ENG, V35, P1961, DOI 10.1007/s10439-007-9375-x
   Willett TL, 2009, J BONE JOINT SOC D 2, V91, P217
NR 23
TC 12
Z9 12
U1 0
U2 5
PU ELSEVIER
PI AMSTERDAM
PA RADARWEG 29, 1043 NX AMSTERDAM, NETHERLANDS
SN 0040-6031
EI 1872-762X
J9 THERMOCHIM ACTA
JI Thermochim. Acta
PD JAN 20
PY 2010
VL 498
IS 1-2
BP 7
EP 10
DI 10.1016/j.tca.2009.09.003
PG 4
WC Thermodynamics; Chemistry, Analytical; Chemistry, Physical
WE Science Citation Index Expanded (SCI-EXPANDED)
SC Thermodynamics; Chemistry
GA 561BC
UT WOS:000274948900002
DA 2022-07-22
ER

PT J
AU Calder, JDF
   Saxby, TS
AF Calder, JDF
   Saxby, TS
TI Independent evaluation of a recently described Achilles tendon repair
   technique
SO FOOT & ANKLE INTERNATIONAL
LA English
DT Article
DE achilles tendon rupture; operative procedure; suture technique; tendon
   repair
ID NON-SURGICAL TREATMENT; RUPTURES
AB Background: We investigated a previously reported technique for the repair of acute Achilles tendon ruptures using the percutaneous Achillon((R)) suture system (Intega Life Sciences Corporation, Plainsboro, NJ). Methods: Twenty-five patients with Achilles tendon ruptures were studied prospectively with a minimum of 12 months followup. A single 2- to 3-cm horizontal incision and the Achillon((R)) suture system were used. Early rehabilitation and an active range-of-motion brace were instituted. Results: There were no wound problems, sural nerve injuries, or reruptures. All patients were able to return to their previous sporting activities by 6 months. Conclusions: This independent study confirms that the technique offers patients a safe operative procedure for repair of acute Achilles tendon ruptures that allows early active rehabilitation.
RP Saxby, TS (通讯作者)，Level 9,Arnold Janssen Ctr,259 Wickham Terrace, Brisbane, Qld, Australia.
EM forefoot@bigpond.com
CR Assal M, 2002, J BONE JOINT SURG AM, V84A, P161, DOI 10.2106/00004623-200202000-00001
   INGLIS AE, 1976, J BONE JOINT SURG AM, V58, P990, DOI 10.2106/00004623-197658070-00015
   KAKIUCHI M, 1995, J BONE JOINT SURG BR, V77B, P60, DOI 10.1302/0301-620X.77B1.7822398
   KITAOKA HB, 1994, FOOT ANKLE INT, V15, P349, DOI 10.1177/107110079401500701
   LEPPILAHTI J, 1998, CLIN ORTHOP RELAT R, V346, P152
   Lim J, 2001, FOOT ANKLE INT, V22, P559, DOI 10.1177/107110070102200705
   Lo IKY, 1997, CLIN J SPORT MED, V7, P207, DOI 10.1097/00042752-199707000-00010
   MA GWC, 1977, CLIN ORTHOP RELAT R, P247
   Mortensen NHM, 1999, J BONE JOINT SURG AM, V81A, P983, DOI 10.2106/00004623-199907000-00011
   NISTOR L, 1981, J BONE JOINT SURG AM, V63, P394, DOI 10.2106/00004623-198163030-00012
   Poynton AR, 2001, FOOT ANKLE INT, V22, P572, DOI 10.1177/107110070102200707
   Webb J, 2000, FOOT ANKLE INT, V21, P475, DOI 10.1177/107110070002100604
NR 12
TC 17
Z9 19
U1 0
U2 0
PU SAGE PUBLICATIONS INC
PI THOUSAND OAKS
PA 2455 TELLER RD, THOUSAND OAKS, CA 91320 USA
SN 1071-1007
EI 1944-7876
J9 FOOT ANKLE INT
JI Foot Ankle Int.
PD FEB
PY 2006
VL 27
IS 2
BP 93
EP 96
DI 10.1177/107110070602700204
PG 4
WC Orthopedics
WE Science Citation Index Expanded (SCI-EXPANDED)
SC Orthopedics
GA 014MH
UT WOS:000235483800005
PM 16487460
DA 2022-07-22
ER

PT J
AU Gamal, O
   Shams, A
   Mesregah, MK
AF Gamal, Osama
   Shams, Ahmed
   Mesregah, Mohamed Kamal
TI Augmented Repair of Acute Total Achilles Tendon Rupture With Peroneus
   Brevis Tendon Transfer Using Oblique Transosseous Calcaneal Tunnel: A
   Prospective Case Series
SO JOURNAL OF FOOT & ANKLE SURGERY
LA English
DT Article
DE Achilles tendon rupture; augmented repair; peroneus brevis tendon;
   reconstruction; re-rupture; transosseous tunnel
ID NONOPERATIVE TREATMENT; MANAGEMENT; COMPLICATIONS; INJURY
AB Acute Achilles tendon rupture is one of the most common tendon injuries. This prospective study aimed to evaluate the functional outcomes of augmented repair of acute Achilles tendon rupture with peroneus brevis tendon transfer and fixation to an oblique calcaneal transosseous tunnel. Functional evaluation was performed using the American Orthopaedic Foot & Ankle Society (AOFAS) score and Achilles Tendon Total Rupture Score (ATRS). Postoperative complications were recorded. This study included 33 males and 9 females, with a mean age of 26.1 +/- 4.2 years. The Achilles tendon ruptures were right in 24 patients and left in 18 patients. The cause of rupture was sports injuries in all patients. The mean distance from the calcaneal insertion to the tear site was 4.4 +/- 1.3 cm. The mean time from injury to surgery was 2.4 +/- 1.6 days. The mean follow-up time was 40.3 +/- 4.9 months. The mean AOFAS and ATRS scores were 99.3 +/- 1.2 and 95.9 +/- 1.9, respectively, at 12 months of follow-up. The mean time to return to work was 12.1 +/- 1.2 weeks. The mean time to return to light sports activities was 16.1 +/- 1.8 weeks postoperatively. None of the patients developed re-rupture. Augmented repair of acute Achilles tendon ruptures with peroneus brevis tendon transfer is a robust repair with excellent functional outcomes and early weight-bearing and return to sports activities. (c) 2021 by the American College of Foot and Ankle Surgeons. All rights reserved.
C1 [Gamal, Osama; Shams, Ahmed; Mesregah, Mohamed Kamal] Menoufia Univ, Fac Med, Dept Orthopaed Surg, Menoufia 32511, Egypt.
RP Mesregah, MK (通讯作者)，Menoufia Univ, Fac Med, Dept Orthopaed Surg, Menoufia 32511, Egypt.
EM mohamed.mesregah@med.menofia.edu.eg
RI Gamal, Osama/AAE-5036-2022; Mesregah, Mohamed/Y-5819-2019; shams,
   ahmed/AAC-6944-2021
OI Gamal, Osama/0000-0002-3425-6704; Mesregah, Mohamed/0000-0002-8047-9159;
   shams, ahmed/0000-0002-2831-3389
CR Abubeih H, 2018, INT ORTHOP, V42, P2699, DOI 10.1007/s00264-018-3976-x
   Akhil AT, 2013, MALAYS ORTHOP J, V7, P19, DOI 10.5704/MOJ.1303.011
   Bergkvist D, 2012, J BONE JOINT SURG AM, V94A, P1229, DOI 10.2106/JBJS.J.01601
   Boyd RPR, 2015, BRIT J GEN PRACT, V65, P668, DOI 10.3399/bjgp15X688069
   Clanton TO, 2013, FOOT ANKLE INT, V34, P486, DOI 10.1177/1071100713477625
   Dalal RB, 2003, ANN ROY COLL SURG, V85, P283, DOI 10.1308/003588403766275088
   Demirel M, 2011, INDIAN J ORTHOP, V45, P45, DOI 10.4103/0019-5413.73654
   DOWNIE WW, 1978, ANN RHEUM DIS, V37, P378, DOI 10.1136/ard.37.4.378
   Gallant G G, 1995, Am J Orthop (Belle Mead NJ), V24, P257
   Garabito A, 2005, INT ORTHOP, V29, P42, DOI 10.1007/s00264-004-0598-2
   Hahn F, 2008, CLIN BIOMECH, V23, P109, DOI 10.1016/j.clinbiomech.2007.08.015
   Huttunen TT, 2014, AM J SPORT MED, V42, P2419, DOI 10.1177/0363546514540599
   Ibrahim Talal, 2007, J Foot Ankle Surg, V46, P65, DOI 10.1053/j.jfas.2006.12.002
   Jacob HAC, 2001, CLIN BIOMECH, V16, P783, DOI 10.1016/S0268-0033(01)00070-5
   Jiang N, 2012, INT ORTHOP, V36, P765, DOI 10.1007/s00264-011-1431-3
   Jones Matthew P, 2012, J Bone Joint Surg Am, V94, pe88, DOI 10.2106/JBJS.J.01829
   Karousou E, 2008, CLIN ORTHOP RELAT R, V466, P1577, DOI 10.1007/s11999-008-0255-y
   Khalid Mohammed A, 2019, Foot Ankle Spec, V12, P345, DOI 10.1177/1938640018803695
   Khalil LS, 2020, ORTHOP J SPORTS MED, V8, DOI 10.1177/2325967120966041
   KITAOKA HB, 1994, FOOT ANKLE INT, V15, P349, DOI 10.1177/107110079401500701
   Lantto I, 2016, AM J SPORT MED, V44, P2406, DOI 10.1177/0363546516651060
   Lerch TD, 2020, FOOT ANKLE INT, V41, P784, DOI 10.1177/1071100720919029
   Lo Torto F, 2020, FOOT ANKLE SURG, V26, P218, DOI 10.1016/j.fas.2019.02.004
   Lui TH, 2007, ARTHROSCOPY, V23, DOI 10.1016/j.arthro.2006.07.053
   Maffulli N, 2015, BONE JOINT J, V97B, P353, DOI 10.1302/0301-620X.97B3.33732
   Maffulli N, 2018, FOOT ANKLE SURG, V24, P164, DOI 10.1016/j.fas.2017.02.002
   Mavrodontidis A, 2015, ACTA ORTHOP TRAUMATO, V49, P661, DOI 10.3944/AOTT.2015.14.0196
   Mohamed A, 2017, MLTJ-MUSCLE LIGAMENT, V7, P396, DOI 10.11138/mltj/2017.7.2.396
   Nilsson-Helander K, 2007, AM J SPORT MED, V35, P421, DOI 10.1177/0363546506294856
   Ochen Y, 2019, BMJ-BRIT MED J, V364, DOI 10.1136/bmj.k5120
   Park SH, 2020, CLIN ORTHOP SURG, V12, P1, DOI 10.4055/cios.2020.12.1.1
   PEREZTEUFFER A, 1974, ORTHOP CLIN N AM, V5, P89
   Pintore E, 2001, J TRAUMA, V50, P71, DOI 10.1097/00005373-200101000-00013
   Porter KJ, 2014, FOOT ANKLE SURG, V20, P90, DOI 10.1016/j.fas.2013.11.005
   Sebastian Hermann, 2007, J Foot Ankle Surg, V46, P424, DOI 10.1053/j.jfas.2007.07.003
   Sheth U, 2017, BONE JOINT J, V99B, P78, DOI [10.1302/0301-620X.99B1.BJJ-2016-0434.R1, 10.1302/0301-620x.99b1.bjj-2016-0434.r1]
   Singh A, 2014, J ORTHOP SURG-HONG K, V22, P52, DOI 10.1177/230949901402200114
   Spina Andreo A, 2007, J Can Chiropr Assoc, V51, P158
   Stenroos Antti J, 2020, J Am Podiatr Med Assoc, V110, DOI 10.7547/18-014
   TURCO VJ, 1987, FOOT ANKLE, V7, P253, DOI 10.1177/107110078700700407
   Wang D, 2015, FOOT ANKLE SURG, V21, P250, DOI 10.1016/j.fas.2015.01.009
   Willits K, 2010, J BONE JOINT SURG AM, V92A, P2767, DOI 10.2106/JBJS.I.01401
   Yang B, 2017, INT J SURG, V40, P178, DOI 10.1016/j.ijsu.2017.03.021
   Yang X, 2018, BONE JOINT RES, V7, P561, DOI 10.1302/2046-3758.710.BJR-2018-0004.R2
   Yang YL, 2020, J ORTHOP SURG RES, V15, DOI 10.1186/s13018-020-01776-6
   Zellers JA, 2016, BRIT J SPORT MED, V50, P1325, DOI 10.1136/bjsports-2016-096106
NR 46
TC 0
Z9 0
U1 0
U2 0
PU ELSEVIER SCIENCE INC
PI NEW YORK
PA STE 800, 230 PARK AVE, NEW YORK, NY 10169 USA
SN 1067-2516
EI 1542-2224
J9 J FOOT ANKLE SURG
JI J. Foot Ankle Surg.
PD SEP-OCT
PY 2021
VL 60
IS 5
BP 923
EP 928
DI 10.1053/j.jfas.2021.03.014
EA SEP 2021
PG 6
WC Orthopedics; Surgery
WE Science Citation Index Expanded (SCI-EXPANDED)
SC Orthopedics; Surgery
GA UR5EQ
UT WOS:000696772800011
PM 33875333
DA 2022-07-22
ER

PT J
AU Hussien, DG
   Villarreal, JV
   Panchbhavi, V
   Jupiter, DC
AF Hussien, Doha G.
   Villarreal, Joseph V.
   Panchbhavi, Vinod
   Jupiter, Daniel C.
TI Predisposing Factors for 30-Day Complications Following Achilles Tendon
   Repair
SO JOURNAL OF FOOT & ANKLE SURGERY
LA English
DT Article
DE Achilles tendon rupture; lower extremity repairs; patient risk factors;
   postoperative complications; surgical outcomes; tendon injuries
AB Certain risk factors for Achilles tendon repair complications, including tobacco use, diabetes mellitus, steroid use, and obesity, have been well-reviewed. This study analyzes the impact of a range of demographic factors on unique surgical complications within the 30-day postoperative period. We extracted data from the American College of Surgeons National Surgical Quality Improvement Program database from 2012 to 2017 and searched for Achilles tendon repairs using CPT codes 27650, 27652, and 27654, identifying 4,040 patients. Twenty-two demographic variables and their association with each of 4 complications were analyzed using t tests or chi-squared tests. A logistic regression was conducted to determine independent risk factors for each outcome, based on results from the bivariate analyses. Variables having p < .2 on bivariate analysis were included in the multivariate analysis of the corresponding surgical complication. Patients with open or infected wounds preoperatively were more likely to return with a postoperative surgical infection (p < .001). Likewise, each additional year of the patient?s age increased their likelihood of developing a surgical infection (p = .03). Patients with ?clean/contaminated? wound sites prior to repair were more likely to return for an additional service (p = .02). Furthermore, each additional inch of the patient?s height (p = .03) and every additional minute of operative time increased a patient?s risk of developing a new-onset deep vein thrombosis (p = .01). This study offers providers a more complete picture of which preoperative characteristics affect Achilles tendon repair patients, directing management to reduce postoperative complication rates. ? 2020 by the American College of Foot and Ankle Surgeons. All rights reserved.
C1 [Hussien, Doha G.; Villarreal, Joseph V.] Univ Texas Med Branch, Sch Med, 300 Univ Blvd, Galveston, TX 77555 USA.
   [Panchbhavi, Vinod; Jupiter, Daniel C.] Univ Texas Med Branch, Dept Orthoped Surg & Rehabil, Galveston, TX 77555 USA.
   [Jupiter, Daniel C.] Univ Texas Med Branch, Dept Prevent Med & Populat Hlth, Galveston, TX 77555 USA.
RP Hussien, DG (通讯作者)，Univ Texas Med Branch, Sch Med, 300 Univ Blvd, Galveston, TX 77555 USA.
EM dghussie@utmb.edu
RI Panchbhavi, Vinod Kumar/AAN-1355-2021
OI Panchbhavi, Vinod Kumar/0000-0003-2420-7316; Villarreal,
   Joseph/0000-0002-8055-8578
CR Barfod KW, 2017, J FOOT ANKLE SURG, V56, P440, DOI 10.1053/j.jfas.2017.01.004
   Bhandari M, 2002, CLIN ORTHOP RELAT R, P190
   Bruggeman NB, 2004, CLIN ORTHOP RELAT R, P63, DOI 10.1097/01.blo.0000144475.05543.e7
   Burrus M Tyrrell, 2016, Foot Ankle Spec, V9, P208, DOI 10.1177/1938640015609983
   Egger AC, 2017, CURR REV MUSCULOSKE, V10, P72, DOI 10.1007/s12178-017-9386-7
   Jildeh TR, 2018, ORTHOP J SPORTS MED, V6, DOI 10.1177/2325967118774302
   Lemme NJ, 2018, ORTHOP J SPORTS MED, V6, DOI 10.1177/2325967118808238
   Metz R, 2011, AM J SPORT MED, V39, P820, DOI 10.1177/0363546510392012
   Olsson N, 2014, AM J SPORT MED, V42, P1448, DOI 10.1177/0363546514527409
   Pean CA, 2018, J ORTHOP, V15, P226, DOI 10.1016/j.jor.2018.01.017
   Pedowitz D, 2013, CURR REV MUSCULOSKE, V6, P285, DOI 10.1007/s12178-013-9185-8
   Roetker NS, 2017, J THROMB HAEMOST, V15, P1334, DOI 10.1111/jth.13719
   Stavenuiter XJR, 2019, FOOT ANKLE INT, V40, P679, DOI 10.1177/1071100719831371
   Yang X, 2018, BONE JOINT RES, V7, P561, DOI 10.1302/2046-3758.710.BJR-2018-0004.R2
   Zoller B, 2017, CIRC-CARDIOVASC GENE, V10, DOI 10.1161/CIRCGENETICS.116.001651
NR 15
TC 1
Z9 1
U1 0
U2 1
PU ELSEVIER SCIENCE INC
PI NEW YORK
PA STE 800, 230 PARK AVE, NEW YORK, NY 10169 USA
SN 1067-2516
EI 1542-2224
J9 J FOOT ANKLE SURG
JI J. Foot Ankle Surg.
PD MAR-APR
PY 2021
VL 60
IS 2
BP 288
EP 291
DI 10.1053/j.jfas.2020.08.029
EA MAR 2021
PG 4
WC Orthopedics; Surgery
WE Science Citation Index Expanded (SCI-EXPANDED)
SC Orthopedics; Surgery
GA RA9EI
UT WOS:000631721500013
PM 33461920
DA 2022-07-22
ER

PT J
AU Barfod, KW
   Sveen, TM
   Ganestam, A
   Ebskov, LB
   Troelsen, A
AF Barfod, Kristoffer Weisskirchner
   Sveen, Thor Magnus
   Ganestam, Ann
   Ebskov, Lars Bo
   Troelsen, Anders
TI Severe Functional Debilitations After Complications Associated With
   Acute Achilles Tendon Rupture With 9 Years of Follow-Up
SO JOURNAL OF FOOT & ANKLE SURGERY
LA English
DT Article
DE Achilles tendon rupture; complication; infection; repeat rupture; sural
   nerve lesion
ID DEFICITS; ELONGATION
AB The purpose of the present study was to investigate the long-term effect of deep infection, sural nerve injury, and repeat rupture in the treatment of acute Achilles tendon rupture. A total of 324 patients had made a claim to the Danish Patient Insurance Association from 1992 to 2010 for a complication after acute Achilles tendon rupture. Of the 324 patients, 119 (36.7%) (77 [64.7%] males and 42 [35.3%] females) returned the Achilles tendon total rupture score and the 36-item short-form survey questionnaires. Patients with deep infection (n = 10), sural nerve injury (n = 10), and repeat rupture (n = 16) participated in a follow-up investigation. The mean follow-up period was 8.9 (range 3 to 21) years. The mean Achilles tendon total rupture score was 49 +/- 27. The summary scores of the physical component and mental components scales of the 36-item Short Form Survey were 43 +/- 11 and 52 +/- 11, respectively. No significant differences were found among the subpopulations with deep infection, injury to the sural nerve, or repeat rupture. The physical evaluation investigating tendon length and heel rise work revealed a statistically significant difference between the affected and unaffected limb after repeat rupture (p < .01) but not after injury to the sural nerve (p > .05) or deep infection (p > .05). In conclusion, patients with from a complication after acute Achilles tendon rupture had a remarkable reduction of the Achilles tendon total rupture score and physical component scale score at mean follow-up point of 9 years. Patients with repeat rupture had a significant elongation of the tendon and reduction of strength in the affected limb. (C) 2017 by the American College of Foot and Ankle Surgeons. All rights reserved.
C1 [Barfod, Kristoffer Weisskirchner; Sveen, Thor Magnus; Ganestam, Ann; Troelsen, Anders] Copenhagen Univ Hosp, Clin Orthopaed Res Hvidovre, Dept Orthopaed Surg, Hvidovre, Denmark.
   [Ebskov, Lars Bo] Copenhagen Univ Hosp, Dept Orthopaed Surg, Kettegaards Alle 30, DK-2650 Hvidovre, Denmark.
RP Barfod, KW (通讯作者)，Copenhagen Univ Hosp, Dept Orthopaed Surg, Kettegaards Alle 30, DK-2650 Hvidovre, Denmark.
EM kbarfod@dadlnet.dk
RI Barfod, Kristoffer/AAY-3288-2021
OI Barfod, Kristoffer/0000-0003-2620-5891; Troelsen,
   Anders/0000-0003-0132-8182
CR Barfod KW, 2015, KNEE SURG SPORT TR A, V23, P3398, DOI 10.1007/s00167-014-3175-2
   Barfod KW, 2014, J BONE JOINT SURG AM, V96A, P1497, DOI 10.2106/JBJS.M.01273
   Barfod KW, 2014, DAN MED J, V61
   Ganestam A, 2016, KNEE SURG SPORT TR A, V24, P3730, DOI 10.1007/s00167-015-3544-5
   Ganestam A, 2013, J FOOT ANKLE SURG, V52, P736, DOI 10.1053/j.jfas.2013.07.004
   Holm C, 2015, SCAND J MED SCI SPOR, V25, pE1, DOI 10.1111/sms.12209
   Horstmann T, 2012, INT J SPORTS MED, V33, P474, DOI 10.1055/s-0032-1301932
   Kangas J, 2007, AM J SPORT MED, V35, P59, DOI 10.1177/0363546506293255
   Karlsson J., 2014, ACHILLES TENDON DISO
   Khan RJK, 2010, COCHRANE DB SYST REV, DOI 10.1002/14651858.CD003674.pub4
   Magnusson SP, 2007, INT J EXP PATHOL, V88, P237, DOI 10.1111/j.1365-2613.2007.00551.x
   Nilsson-Helander K, 2007, AM J SPORT MED, V35, P421, DOI 10.1177/0363546506294856
   Nilsson-Helander K, 2010, AM J SPORT MED, V38, P2186, DOI 10.1177/0363546510376052
   Olsson N, 2014, AM J SPORT MED, V42, P1448, DOI 10.1177/0363546514527409
   Olsson N, 2011, KNEE SURG SPORT TR A, V19, P1385, DOI 10.1007/s00167-011-1511-3
   Pajala A, 2002, J BONE JOINT SURG AM, V84A, P2016, DOI 10.2106/00004623-200211000-00017
   Silbernagel KG, 2012, AM J SPORT MED, V40, P1564, DOI 10.1177/0363546512447926
   Silbernagel KG, 2010, KNEE SURG SPORT TR A, V18, P258, DOI 10.1007/s00167-009-0889-7
   Soroceanu A, 2012, J BONE JOINT SURG AM, V94A, P2136, DOI 10.2106/JBJS.K.00917
   SULLIVAN M, 1995, SOC SCI MED, V41, P1349, DOI 10.1016/0277-9536(95)00125-Q
   Sveen TM, 2015, DAN MED J, V62
   Ware JE, 2007, US POPULATION NORMS
   Wilkins R, 2012, AM J SPORT MED, V40, P2154, DOI 10.1177/0363546512453293
NR 23
TC 7
Z9 7
U1 0
U2 0
PU ELSEVIER SCIENCE INC
PI NEW YORK
PA STE 800, 230 PARK AVE, NEW YORK, NY 10169 USA
SN 1067-2516
EI 1542-2224
J9 J FOOT ANKLE SURG
JI J. Foot Ankle Surg.
PD MAY-JUN
PY 2017
VL 56
IS 3
BP 440
EP 444
DI 10.1053/j.jfas.2017.01.004
PG 5
WC Orthopedics; Surgery
WE Science Citation Index Expanded (SCI-EXPANDED)
SC Orthopedics; Surgery
GA EU2UC
UT WOS:000400884400005
PM 28216305
DA 2022-07-22
ER

PT J
AU Uchida, R
   Natsuume, T
   Yoneda, K
   Fuji, T
AF Uchida, Ryohei
   Natsuume, Takashi
   Yoneda, Kenji
   Fuji, Takeshi
TI Repair of Achilles Tendon Rupture Using Autologous Semitendinosus Graft
   in a Kidney Transplant Recipient
SO JOURNAL OF FOOT & ANKLE SURGERY
LA English
DT Article
DE Achilles tendon injury; renal transplantation; semitendinosus tendon
ID CRUCIATE LIGAMENT RECONSTRUCTION; QUADRICEPS TENDON; PLANTARIS TENDON;
   HYPERPARATHYROIDISM; HEMODIALYSIS
AB Insertional Achilles tendon injuries can be difficult to treat when minimal tendon tissue remains for anastomosis. Moreover, in the chronic case with tendon shortening, operative repair can be more difficult than acute rupture. It is particularly desirable to reinforce the tendons, in addition to performing primary repair, in patients with renal or systemic diseases because of the accelerated collagen degeneration. Many techniques have been described for the surgical management of Achilles tendon rupture; however, none has shown clear superiority. We report the case of a 50-year-old renal transplant patient with a spontaneous distal Achilles tendon injury that we repaired using the pull-out technique reinforced with an autologous semitendinosus graft. At 2 years postoperatively, the ankle-hindfoot scale score was 92 points, and the postoperative course was without complication. We believe that the free hamstring tendon autograft is advantageous for this repair, because it is easy to handle, has limited donor site morbidity, and preserves the structures around the ankle. (C) 2014 by the American College of Foot and Ankle Surgeons. All rights reserved.
C1 [Uchida, Ryohei] Osaka Univ, Grad Sch Med, Dept Orthopaed Surg, Suita, Osaka 5650871, Japan.
   [Uchida, Ryohei; Natsuume, Takashi; Yoneda, Kenji; Fuji, Takeshi] Osaka Kousei Nenkin Hosp, Dept Sports Med, Osaka, Japan.
   [Natsuume, Takashi] Sumitomo Hosp, Dept Orthopaed, Osaka, Japan.
RP Uchida, R (通讯作者)，Osaka Univ, Grad Sch Med, Dept Orthopaed Surg, 2-2 Yamada Oka, Suita, Osaka 5650871, Japan.
EM uchida3847@gmail.com
RI Uchida, Ryohei/AAW-7733-2021
CR Baer GS, 2010, TENDON INJURIES FOOT
   Besse J L, 1999, J Foot Ankle Surg, V38, P70
   BOSWORTH DM, 1956, J BONE JOINT SURG AM, V38, P111, DOI 10.2106/00004623-195638010-00011
   BUTLER DL, 1984, J BIOMECH, V17, P579, DOI 10.1016/0021-9290(84)90090-3
   CIRINCIONE RJ, 1975, J BONE JOINT SURG BR, V57, P852
   HANNA JR, 1993, J AM PODIAT MED ASSN, V83, P663, DOI 10.7547/87507315-83-12-663
   Hiemstra LA, 2007, CLIN BIOMECH, V22, P543, DOI 10.1016/j.clinbiomech.2007.01.009
   Hioki S, 2003, KNEE SURG SPORT TR A, V11, P223, DOI 10.1007/s00167-003-0361-z
   HOWARD CB, 1984, J BONE JOINT SURG BR, V66, P206, DOI 10.1302/0301-620X.66B2.6707056
   Janis L, 2000, J Foot Ankle Surg, V39, P49
   Jennings AG, 2002, J BONE JOINT SURG BR, V84B, P361, DOI 10.1302/0301-620X.84B3.11559
   Kocabey Yavuz, 2006, J Foot Ankle Surg, V45, P42, DOI 10.1053/j.jfas.2005.10.009
   Lepow Gary M, 2006, J Foot Ankle Surg, V45, P351, DOI 10.1053/j.jfas.2006.06.004
   LEVY M, 1984, CLIN ORTHOP RELAT R, P199
   LINDHOLM A, 1959, Acta Chir Scand, V117, P261
   LOTEM M, 1974, ANN RHEUM DIS, V33, P428, DOI 10.1136/ard.33.5.428
   LYNN TA, 1966, J BONE JOINT SURG AM, VA 48, P268, DOI 10.2106/00004623-196648020-00005
   MADDOX PA, 1986, J BONE JOINT SURG AM, V68A, P288, DOI 10.2106/00004623-198668020-00015
   Maffulli N, 1999, J BONE JOINT SURG AM, V81A, P1019, DOI 10.2106/00004623-199907000-00017
   Maffulli N, 2005, CLIN J SPORT MED, V15, P56, DOI 10.1097/01.jsm.0000152714.05097.ef
   Maniscalco P, 1998, J Foot Ankle Surg, V37, P96
   Maniscalco P, 1998, J FOOT ANKLE SURG, V37, P172
   MANN RA, 1991, J BONE JOINT SURG AM, V73A, P214, DOI 10.2106/00004623-199173020-00009
   Miyamoto W, 2012, KNEE SURG SPORT TR A, V20, P1863, DOI 10.1007/s00167-011-1792-6
   OZAKI J, 1989, CLIN ORTHOP RELAT R, P204
   PEREZTEUFFER A, 1974, ORTHOP CLIN N AM, V5, P89
   PIERIDES AM, 1977, CLIN NEPHROL, V7, P271
   PRESTON ET, 1972, J AMER MED ASSOC, V221, P406, DOI 10.1001/jama.221.4.406
   ROBERTSON DB, 1986, AM J SPORT MED, V14, P398, DOI 10.1177/036354658601400512
   SCHEDL R, 1979, J TRAUMA, V19, P189, DOI 10.1097/00005373-197903000-00011
   Shiota E, 2002, CLIN ORTHOP RELAT R, P236
   SPENCER JD, 1988, INJURY, V19, P86, DOI 10.1016/0020-1383(88)90079-4
   Tsourvakas S, 2004, ARCH ORTHOP TRAUM SU, V124, P278, DOI 10.1007/s00402-003-0628-3
   TURCO VJ, 1987, FOOT ANKLE, V7, P253, DOI 10.1177/107110078700700407
   Wapner K L, 1993, Foot Ankle, V14, P443
NR 35
TC 3
Z9 3
U1 0
U2 3
PU ELSEVIER SCIENCE INC
PI NEW YORK
PA 360 PARK AVE SOUTH, NEW YORK, NY 10010-1710 USA
SN 1067-2516
EI 1542-2224
J9 J FOOT ANKLE SURG
JI J. Foot Ankle Surg.
PD MAR-APR
PY 2014
VL 53
IS 2
BP 194
EP 198
DI 10.1053/j.jfas.2013.12.010
PG 5
WC Orthopedics; Surgery
WE Science Citation Index Expanded (SCI-EXPANDED)
SC Orthopedics; Surgery
GA AB4II
UT WOS:000331752600016
PM 24556487
DA 2022-07-22
ER

PT J
AU Jandacka, D
   Silvernail, JF
   Uchytil, J
   Zahradnik, D
   Farana, R
   Hamill, J
AF Jandacka, Daniel
   Silvernail, Julia Freedman
   Uchytil, Jaroslav
   Zahradnik, David
   Farana, Roman
   Hamill, Joseph
TI Do athletes alter their running mechanics after an Achilles tendon
   rupture?
SO JOURNAL OF FOOT AND ANKLE RESEARCH
LA English
DT Article
DE Achilles tendon; Injury; Ultrasonography; Knee
ID 2-JOINT MUSCLES; INJURY; REPAIR; LENGTH; BASKETBALL; OUTCOMES; RUNNERS;
   FLEXION; ANGLE
AB Background: Over the past thirty years, there has been dramatic increase in incidence of Achilles tendon rupture in the athletic population. The purpose of this study was to compare the lower extremity mechanics of Achilles tendon ruptured runners with healthy controls.
   Methods: The participants with a past history of an Achilles tendon repair (n = 11) and healthy control (n = 11) subgroups were matched on sex, age, type of regular physical activity, mass, height, footfall pattern and lateral dominancy. Running kinetics and kinematics of the ankle, knee and hip were recorded using a high-speed motion capture system interfaced with a force platform. Achilles tendon length was measured using ultrasonography. Main outcome measures were lower extremity joint angles and moments during stance phase of running and Achilles tendon lengths.
   Results: Athletes from Achilles tendon group had an affected gastro-soleus complex. Athletes with history of Achilles tendon rupture had reduced ankle range of motion during second half of the stance phase of running (Delta 7.6 degrees), an overextended knee during initial contact (Delta 5.2 degrees) and increased affected knee range of motion (Delta 4.4 degrees) during the first half of stance phase on their affected limb compared to the healthy control group. There was a 22% increase in the maximal hip joint moment on contralateral side of the Achilles tendon group compared to the healthy controls.
   Conclusion: These results suggest a compensation mechanism, relatively extended knee at initial ground contact against the deficit in the muscle-tendon complex of the triceps surae. Overextension during sporting activities may place the knee at risk for further injury. Avoidance of AT lengthening and plantarflexion strength deficit after surgery and during rehabilitation might help to manage AT rupture since these factors may be responsible for altered running kinematics.
C1 [Jandacka, Daniel; Uchytil, Jaroslav; Zahradnik, David; Farana, Roman; Hamill, Joseph] Univ Ostrava, Human Mot Diagnost Ctr, Dept Human Movement Studies, Varenska 40 A, Ostrava 70200, Czech Republic.
   [Silvernail, Julia Freedman] Univ Nevada, Dept Kinesiol & Nutr Sci, Las Vegas, NV 89154 USA.
   [Hamill, Joseph] Univ Massachusetts, Dept Kinesiol, Amherst, MA 01003 USA.
RP Jandacka, D (通讯作者)，Univ Ostrava, Human Mot Diagnost Ctr, Dept Human Movement Studies, Varenska 40 A, Ostrava 70200, Czech Republic.
EM daniel.jandacka@osu.cz
RI Jandacka, Daniel/B-6035-2011
OI Jandacka, Daniel/0000-0003-2653-2569; Freedman Silvernail,
   Julia/0000-0002-8792-696X
FU Grant SGS University of Ostrava [SGS05/PDF/2014]
FX This research was supported by the Grant SGS University of Ostrava
   (SGS05/PDF/2014). Funding was used in the design of the study and data
   collection.
CR Agres AN, 2015, SCAND J MED SCI SPOR, V25, P860, DOI 10.1111/sms.12456
   Amin NH, 2013, AM J SPORT MED, V41, P1864, DOI 10.1177/0363546513490659
   Aroen A, 2004, SCAND J MED SCI SPOR, V14, P30, DOI 10.1111/j.1600-0838.2004.00344.x
   Bressel E, 2004, CLIN BIOMECH, V19, P284, DOI 10.1016/j.clinbiomech.2003.12.008
   Carmont MR, 2015, ASIA-PAC J SPORT MED, V2, P49, DOI 10.1016/j.asmart.2014.12.002
   Cohen J., 1990, STAT POWER ANAL BEHA
   Cooper A, 2012, PHYSIOTHER RES INT, V17, DOI 10.1002/pri.528
   GRIMBY G, 1986, ACTA MED SCAND, P233
   Hamill J, 2008, CLIN BIOMECH, V23, P1018, DOI 10.1016/j.clinbiomech.2008.04.017
   Hanavan E.P, 1964, MATH MODEL HUMAN BOD
   Hespanhol LC, 2013, J PHYSIOTHER, V59, P263, DOI 10.1016/S1836-9553(13)70203-0
   Houshian S, 1998, INJURY, V29, P651, DOI 10.1016/S0020-1383(98)00147-8
   Jandacka D, 2013, BMJ CASE REP, V2013
   Krosshaug T, 2007, AM J SPORT MED, V35, P359, DOI 10.1177/0363546506293899
   Lantto I, 2015, SCAND J MED SCI SPOR, V25, pE133, DOI 10.1111/sms.12253
   Malvankar S, 2011, Foot (Edinb), V21, P193, DOI 10.1016/j.foot.2011.08.004
   McClay I, 1999, MED SCI SPORT EXER, V31, P1629, DOI 10.1097/00005768-199911000-00021
   McClay I, 1998, CLIN BIOMECH, V13, P195, DOI 10.1016/S0268-0033(97)00029-6
   Moraux A, 2013, BMC MUSCULOSKEL DIS, V14, DOI 10.1186/1471-2474-14-104
   Mullaney MJ, 2006, AM J SPORT MED, V34, P1120, DOI 10.1177/0363546505284186
   Nilsson-Helander K, 2007, AM J SPORT MED, V35, P421, DOI 10.1177/0363546506294856
   Nunley JA, 2010, ACHILLES TENDON TREA
   Parekh Selene G, 2009, Foot Ankle Spec, V2, P283, DOI 10.1177/1938640009351138
   Podraza JT, 2010, KNEE, V17, P291, DOI 10.1016/j.knee.2010.02.013
   PRILUTSKY BI, 1994, J BIOMECH, V27, P25, DOI 10.1016/0021-9290(94)90029-9
   Rees JD, 2008, RHEUMATOLOGY, V47, P1493, DOI 10.1093/rheumatology/ken262
   Robertson E, 2014, RES METHODS BIOMECHA
   ROBINSON RO, 1987, J MANIP PHYSIOL THER, V10, P172
   Rosso C, 2013, KNEE SURG SPORT TR A, V21, P1369, DOI 10.1007/s00167-013-2407-1
   Seeley MK, 2008, GAIT POSTURE, V28, P24, DOI 10.1016/j.gaitpost.2007.09.006
   Selbie W.S., 2013, RES METHODS BIOMECHA, Vsecond, P35
   Shultz SJ, 2009, SPORTS HEALTH, V1, P54, DOI 10.1177/1941738108326702
   Silbernagel KG, 2012, J ORTHOP SPORT PHYS, V42, P521, DOI 10.2519/jospt.2012.3913
   Suydam SM, 2013, KNEE SURG SPORT TR A, V23, P868
   Tengman T, 2015, EUR J PHYSIOTHER, V17, P200, DOI 10.3109/21679169.2015.1078842
   Umberger BR, 1998, STRENGTH COND, V20, P70, DOI 10.1519/1073-6840(1998)020<0070:MOTVJA>2.3.CO;2
   Willy RW, 2017, AM J SPORT MED, V45, P1124, DOI 10.1177/0363546516685055
NR 37
TC 16
Z9 16
U1 2
U2 12
PU BIOMED CENTRAL LTD
PI LONDON
PA 236 GRAYS INN RD, FLOOR 6, LONDON WC1X 8HL, ENGLAND
SN 1757-1146
J9 J FOOT ANKLE RES
JI J. Foot Ankle Res.
PD NOV 28
PY 2017
VL 10
AR 53
DI 10.1186/s13047-017-0235-0
PG 8
WC Orthopedics
WE Science Citation Index Expanded (SCI-EXPANDED)
SC Orthopedics
GA FN7PM
UT WOS:000416210700002
PM 29209417
OA Green Published, gold
DA 2022-07-22
ER

PT J
AU Lee, DK
AF Lee, Daniel K.
TI A preliminary study on the effects of acellular tissue graft
   augmentation in acute Achilles tendon ruptures
SO JOURNAL OF FOOT & ANKLE SURGERY
LA English
DT Article
DE acellular human dermal matrix; acute Achilles tendon rupture; Achilles
   tendon augmentation
ID DERMAL MATRIX; REPAIR; WEIGHTBEARING; MOTION
AB Acute Achilles tendon rupture injuries present surgical challenges because of the mechanical forces placed on this tendon. The purpose of this study was to evaluate the effectiveness of an acellular human dermal tissue matrix, Graft Jacket Matrix (Wright Medical Technology, Inc., Arlington, TN), as an augmentation material in acute Achilles tendon repair. Eleven consecutive patients with acute tendon ruptures were evaluated and followed up (20-31 months). Primary repair was followed by augmentation with the graft sutured circumferentially around the tendon. Patients were placed in an early functional rehabilitation program with postoperative evaluation at 3, 6, and 12 months. Outcome scores were calculated based on the American Orthopaedic Foot and Ankle Society ankle-hindfoot scoring system. At 20-month postoperative follow-up, there have been no cases of rerupture or recurrent pain. The average return-to-activity time was 11.8 +/- 0.75 weeks. These retrospective clinical results suggest that with an acellular human dermal tissue matrix to augment acute Achilles tendon, primary repair offers a desirable return-to-activity time without any rerupture or complications. ACFAS Level of Clinical Evidence: 2c.
C1 Univ Calif San Diego, Dept Orthoped Surg, San Diego, CA 92103 USA.
RP Lee, DK (通讯作者)，Univ Calif San Diego, Dept Orthoped Surg, 350 Dickinson St,MC 8894, San Diego, CA 92103 USA.
EM dklee@ucsd.edu
CR Adams JE, 2006, ARTHROSCOPY, V22, P700, DOI 10.1016/j.arthro.2006.03.016
   Aoki M, 1998, AM J SPORT MED, V26, P794, DOI 10.1177/03635465980260061001
   Barber FA, 2006, ARTHROSCOPY, V22, P534, DOI 10.1016/j.arthro.2005.12.021
   Beniker D, 2003, ORTHOPEDICS, V26, pS591
   Bhandari M, 2002, CLIN ORTHOP RELAT R, P190
   Brigido SA, 2004, ORTHOPEDICS, V27, pS145
   CETTI R, 1993, AM J SPORT MED, V21, P791, DOI 10.1177/036354659302100606
   JOZSA L, 1989, AM J SPORT MED, V17, P338, DOI 10.1177/036354658901700305
   Khan RJK, 2005, J BONE JOINT SURG AM, V87A, P2202, DOI 10.2106/JBJS.D.03049
   KITAOKA HB, 1994, FOOT ANKLE INT, V15, P349, DOI 10.1177/107110079401500701
   Lee MS, 2004, ORTHOPEDICS, V27, pS151
   Leppilahti J, 1998, SPORTS MED, V25, P79, DOI 10.2165/00007256-199825020-00002
   Leppilahti J, 1996, ACTA ORTHOP SCAND, V67, P277, DOI 10.3109/17453679608994688
   Maffulli N, 2003, AM J SPORT MED, V31, P692, DOI 10.1177/03635465030310051001
   Maffulli N, 2006, CLIN J SPORT MED, V16, P123, DOI 10.1097/00042752-200603000-00007
   MANDELBAUM BR, 1995, AM J SPORT MED, V23, P392, DOI 10.1177/036354659502300403
   Rettig AC, 2005, AM J SPORT MED, V33, P119, DOI 10.1177/0363546504268720
   Tallon C, 2001, MED SCI SPORT EXER, V33, P1983, DOI 10.1097/00005768-200112000-00002
NR 18
TC 58
Z9 58
U1 0
U2 8
PU ELSEVIER SCIENCE INC
PI NEW YORK
PA STE 800, 230 PARK AVE, NEW YORK, NY 10169 USA
SN 1067-2516
EI 1542-2224
J9 J FOOT ANKLE SURG
JI J. Foot Ankle Surg.
PD JAN-FEB
PY 2008
VL 47
IS 1
BP 8
EP 12
DI 10.1053/j.jfas.2007.08.015
PG 5
WC Orthopedics; Surgery
WE Science Citation Index Expanded (SCI-EXPANDED)
SC Orthopedics; Surgery
GA 339IA
UT WOS:000258570100003
PM 18156058
DA 2022-07-22
ER

PT J
AU Maffulli, N
   Oliva, F
   Migliorini, F
AF Maffulli, Nicola
   Oliva, Francesco
   Migliorini, Filippo
TI Check-rein technique for Achilles tendon elongation following
   conservative management for acute Achilles tendon ruptures: a two-year
   prospective clinical study
SO JOURNAL OF ORTHOPAEDIC SURGERY AND RESEARCH
LA English
DT Article
DE Achilles tendon; Achilles rupture; Tendon elongation; Check-rein
   procedure
ID NONSURGICAL TREATMENT; NONOPERATIVE TREATMENT; SURGICAL REPAIR;
   RANDOMIZED-TRIAL; RESTING ANGLE; GUIDELINES; DEFICITS; TENSION; CAST
AB Background Following conservative management for acute Achilles tendon (AT) ruptures, the tendon may heal in continuity, and some patients may present with an elongated Achilles tendon-gastrosoleus complex. This study investigated the efficacy and feasibility of a novel minimally invasive technique, which we named "check-rein procedure", in patients with intact and elongated AT following conservative management for AT ruptures. Methods All patients who underwent the check-rein procedure for elongation of the gastrosoleus-AT complex by one experienced surgeon were prospectively enrolled. The AT resting angle (ATRA) and AT rupture score (ATRS) were assessed at baseline and repeated at 2-year follow-up, as were calf circumference and isometric plantarflexion strength of both ankles. Results Forty-three patients (43 procedures) were analysed. The mean time elapsed from injury to surgery was 28.7 +/- 7.9 weeks. The mean age at surgery was 38.5 +/- 5.7 years. At the last follow-up, ATRS, ATRA, isometric strength difference, and calf circumference of the affected side were increased (P < 0.0001). The rate of the return to sport was 98% (42 of 43). No wound complications or rupture were experienced by any patient. Conclusion The check-rein technique for AT elongation after conservative management of AT tears is effective and feasible to restore tendon length and calf function. The surgical outcome was influenced by the preoperative performance status, and longer time elapsed from injury to surgery worsens the outcomes.
C1 [Maffulli, Nicola; Oliva, Francesco] Univ Salerno, Dept Med Surg & Dent, Via S Allende, I-84081 Baronissi, SA, Italy.
   [Maffulli, Nicola] Keele Univ, Sch Pharm & Bioengn, Sch Med, Thornburrow Dr, Stoke On Trent, Staffs, England.
   [Maffulli, Nicola] Queen Mary Univ London, Barts & London Sch Med & Dent, Ctr Sports & Exercise Med, Mile End Hosp, 275 Bancroft Rd, London E1 4DG, England.
   [Migliorini, Filippo] RWTH Aachen Univ Hosp, Dept Orthopaed Trauma & Reconstruct Surg, Pauwelsstr 30, D-52074 Aachen, Germany.
RP Migliorini, F (通讯作者)，RWTH Aachen Univ Hosp, Dept Orthopaed Trauma & Reconstruct Surg, Pauwelsstr 30, D-52074 Aachen, Germany.
EM migliorini.md@gmail.com
FU Projekt DEAL
FX Open Access funding enabled and organized by Projekt DEAL. The authors
   received no financial support for the research, authorship, and/or
   publication of this article.
CR Amin NH, 2013, AM J SPORT MED, V41, P1864, DOI 10.1177/0363546513490659
   Barfod KW, 2013, J FOOT ANKLE SURG, V52, P629, DOI 10.1053/j.jfas.2013.04.012
   Barfod KW, 2017, J FOOT ANKLE SURG, V56, P440, DOI 10.1053/j.jfas.2017.01.004
   Carmont MR, 2013, FOOT ANKLE SURG, V19, P245, DOI 10.1016/j.fas.2013.06.007
   Carmont MR, 2013, ORTHOP J SPORTS MED, V1, DOI 10.1177/2325967113494584
   Carmont MR, 2015, ASIA-PAC J SPORT MED, V2, P49, DOI 10.1016/j.asmart.2014.12.002
   Carmont MR, 2009, FOOT ANKLE INT, V30, P704, DOI 10.3113/FAI.2009.0704
   CETTI R, 1993, AM J SPORT MED, V21, P791, DOI 10.1177/036354659302100606
   Costa ML, 2020, LANCET, V395, P441, DOI 10.1016/S0140-6736(19)32942-3
   Costa ML, 2006, FOOT ANKLE INT, V27, P414, DOI 10.1177/107110070602700605
   Del Buono A, 2014, BRIT MED BULL, V109, P45, DOI 10.1093/bmb/ldt029
   Frizziero A, 2014, BRIT MED BULL, V110, P47, DOI 10.1093/bmb/ldu006
   FYFE I, 1992, CLIN SPORT MED, V11, P601
   Gatz M, 2021, ARCH ORTHOP TRAUM SU, V141, P383, DOI 10.1007/s00402-020-03437-z
   Heikkinen J, 2017, AM J SPORT MED, V45, P1395, DOI 10.1177/0363546517694610
   Horstmann T, 2012, INT J SPORTS MED, V33, P474, DOI 10.1055/s-0032-1301932
   Kangas J, 2007, AM J SPORT MED, V35, P59, DOI 10.1177/0363546506293255
   Lantto I, 2015, AM J SPORT MED, V43, P2302, DOI 10.1177/0363546515591267
   Lee SJ, 2008, FOOT ANKLE INT, V29, P427, DOI 10.3113/FAI.2008.0427
   Leppilahti J, 1998, SPORTS MED, V25, P79, DOI 10.2165/00007256-199825020-00002
   Lo IKY, 1997, CLIN J SPORT MED, V7, P207, DOI 10.1097/00042752-199707000-00010
   Maffulli N, 2014, OPER ORTHOP TRAUMATO, V26, P513, DOI 10.1007/s00064-012-0228-x
   Maffulli N, 1996, BRIT J SPORT MED, V30, P250, DOI 10.1136/bjsm.30.3.250
   Maffulli N, 2020, AM J SPORT MED, V48, P1181, DOI 10.1177/0363546520908592
   Maffulli N, 2020, LANCET, V395, P397, DOI 10.1016/S0140-6736(19)33133-2
   Maffulli N, 2020, INJURY, V51, pS73, DOI 10.1016/j.injury.2019.11.013
   Maffulli N, 2019, BMJ-BRIT MED J, V364, DOI 10.1136/bmj.k5344
   Maffulli N, 2008, BMC MUSCULOSKEL DIS, V9, DOI 10.1186/1471-2474-9-100
   Maffulli N, 2012, INT ORTHOP, V36, P2087, DOI 10.1007/s00264-012-1602-x
   Maquirriain Javier, 2011, Yale Journal of Biology and Medicine, V84, P289
   Mark-Christensen T, 2016, KNEE SURG SPORT TR A, V24, P1852, DOI 10.1007/s00167-014-3180-5
   Migliorini F, 2020, EXPERT OPIN BIOL TH, V20, P1373, DOI 10.1080/14712598.2020.1786532
   Moher David, 2010, BMJ, V340, pc869, DOI [10.1016/j.ijsu.2011.10.001, 10.1016/j.jclinepi.2010.03.004, 10.1136/bmj.c869]
   Ochen Y, 2019, BMJ-BRIT MED J, V364, DOI 10.1136/bmj.k5120
   Olsson N, 2013, AM J SPORT MED, V41, P2867, DOI 10.1177/0363546513503282
   Pajala A, 2009, J BONE JOINT SURG AM, V91A, P1092, DOI 10.2106/JBJS.G.01089
   Reda Y, 2020, FOOT ANKLE SURG, V26, P280, DOI 10.1016/j.fas.2019.03.010
   Rosso C, 2015, KNEE SURG SPORT TR A, V23, P890, DOI 10.1007/s00167-013-2726-2
   Saxena A, 2011, J FOOT ANKLE SURG, V50, P37, DOI 10.1053/j.jfas.2010.10.008
   Schepull T, 2012, SCAND J MED SCI SPOR, V22, P18, DOI 10.1111/j.1600-0838.2010.01154.x
   Schepull T, 2013, AM J SPORT MED, V41, P2550, DOI 10.1177/0363546513501785
   Schepull T, 2011, AM J SPORT MED, V39, P38, DOI 10.1177/0363546510383515
   Silbernagel KG, 2012, AM J SPORT MED, V40, P1564, DOI 10.1177/0363546512447926
   SJOSTROM M, 1983, VIRCHOWS ARCH A, V399, P177, DOI 10.1007/BF00619578
   Suydam SM, 2015, KNEE SURG SPORT TR A, V23, P868, DOI 10.1007/s00167-013-2512-1
   Wagner P, 2020, FOOT ANKLE SURG, V26, P421, DOI 10.1016/j.fas.2019.05.008
   Willits K, 2010, J BONE JOINT SURG AM, V92A, P2767, DOI 10.2106/JBJS.I.01401
   Zappia M, 2018, FOOT ANKLE SURG, V24, P342, DOI 10.1016/j.fas.2017.03.006
NR 48
TC 0
Z9 0
U1 0
U2 0
PU BMC
PI LONDON
PA CAMPUS, 4 CRINAN ST, LONDON N1 9XW, ENGLAND
SN 1749-799X
J9 J ORTHOP SURG RES
JI J. Orthop. Surg. Res.
PD NOV 24
PY 2021
VL 16
IS 1
AR 690
DI 10.1186/s13018-021-02830-7
PG 8
WC Orthopedics
WE Science Citation Index Expanded (SCI-EXPANDED)
SC Orthopedics
GA XC7PO
UT WOS:000722202000002
PM 34819119
OA gold, Green Published
DA 2022-07-22
ER

PT J
AU Wang, H
   Liu, NN
AF Wang, Hai
   Liu, Ningning
TI Research on the Examination Value of High-Frequency Ultrasound in the
   Postoperative Rehabilitation Treatment of Sports Injury Achilles Tendon
   Rupture
SO JOURNAL OF MEDICAL IMAGING AND HEALTH INFORMATICS
LA English
DT Article
DE High-Frequency Ultrasound; Achilles Tendon Rupture; Rehabilitation
   Treatment
AB The objective of the paper is to explore the examination value of high-frequency ultrasonography in the postoperative rehabilitation treatment of Achilles tendon injury occurred during sports events. The high-frequency ultrasound imaging technique was applied to examine the Achilles tendon injuries of patients. After the patients accepted Achilles tendon rupture repair surgeries, ultrasound imaging was applied to detect the rehabilitation conditions during the recovery processes. The diagnosis was mainly performed through the Achilles tendon sonograms examined 4 weeks, 6 weeks, 12 weeks, 24 weeks after the surgeries respectively: in addition, the changes in the American Orthopedic Foot and Ankle Score (AOFAS) and the elasticity modulus of repaired Achilles tendons were observed 8 weeks, 12 weeks, 24 weeks, and 48 weeks after the surgeries respectively. The results showed that the Achilles tendon was significantly improved after the postoperative rehabilitation, and the average AOFAS score was positively correlated with the elasticity modulus of the repaired Achilles tendon. In addition, the research results also indicated that the high-frequency ultrasound technology could accurately observe the thickness, area, and internal echo of the Achilles tendons of patients dynamically in real-time, which was convenient and quick. After the patient had undergone rehabilitation training, high-frequency ultrasound could be used to detect the postoperative rehabilitation conditions of patients, thereby the real-time rehabilitation conditions of the patients would be obtained.
C1 [Wang, Hai; Liu, Ningning] Shanxi Datong Univ, Datong 037000, Shanxi, Peoples R China.
RP Wang, H (通讯作者)，Shanxi Datong Univ, Datong 037000, Shanxi, Peoples R China.
CR Barfod KW, 2015, KNEE SURG SPORT TR A, V23, P3398, DOI 10.1007/s00167-014-3175-2
   Cheng H., 2015, J GUIYANG COLL NAT S, V10, P1
   Freedman B.R., 2016, J ORTHOPAEDIC RES OF, V34, P21
   Genovese E, 2011, J CLIN ULTRASOUND, V39, P141, DOI 10.1002/jcu.20789
   Geremia JM, 2015, CLIN BIOMECH, V30, P485, DOI 10.1016/j.clinbiomech.2015.03.005
   Gulati V, 2015, WORLD J ORTHOP, V6, P380, DOI 10.5312/wjo.v6.i4.380
   Maffulli N, 2015, INT ORTHOP, V39, P61, DOI 10.1007/s00264-014-2553-1
   Suydam SM, 2015, KNEE SURG SPORT TR A, V23, P868, DOI 10.1007/s00167-013-2512-1
   Zhang LN, 2016, MED SCI MONITOR, V22, P1186, DOI 10.12659/MSM.895674
NR 9
TC 2
Z9 2
U1 1
U2 5
PU AMER SCIENTIFIC PUBLISHERS
PI VALENCIA
PA 26650 THE OLD RD, STE 208, VALENCIA, CA 91381-0751 USA
SN 2156-7018
EI 2156-7026
J9 J MED IMAG HEALTH IN
JI J. Med. Imaging Health Inform.
PD APR
PY 2020
VL 10
IS 4
BP 918
EP 922
DI 10.1166/jmihi.2020.2954
PG 5
WC Mathematical & Computational Biology; Radiology, Nuclear Medicine &
   Medical Imaging
WE Science Citation Index Expanded (SCI-EXPANDED)
SC Mathematical & Computational Biology; Radiology, Nuclear Medicine &
   Medical Imaging
GA KU5LD
UT WOS:000519751600023
DA 2022-07-22
ER

PT J
AU Zhang, XF
   Ruan, F
   Wu, YP
   Lu, H
AF Zhang, Xiangfeng
   Ruan, Feng
   Wu, Yongping
   Lu, Huang
TI Chronic bilateral asynchronous achilles tendon rupture treated using
   modified whole flexor hallucis longus transfer reconstruction A case
   report
SO MEDICINE
LA English
DT Article
DE bilateral achilles tendon rupture; flexor hallucis longus tendon
   transfer
ID INVASIVE RECONSTRUCTION; AUGMENTATION; REPAIR; GRAFT
AB Introduction: Achilles tendon rupture is common, but bilateral ruptures are very rare. Treatment of chronic Achilles tendon rupture is very challenging due tendon retraction and atrophied. We report a case of bilateral asynchronous Achilles tendon rupture patient who was treated with modified minimally invasive whole flexor hallucis longus (FHL) tendon transfer to repair the defects. Patient concerns: A 52-year-old male farmer presented to our hospital complaining of bilateral heel pain that had disrupted his walking for 6 months. The patient had been misdiagnosed and under-treated for 1 year. Physical examination showed that his plantar flexors were tender and weak, with marked hypotrophy of the calf muscles. Bilateral ankle radiographs of both X-ray and computed topography (CT) revealed no bone injure. Diagnosis: Magnetic resonance imaging (MRI) indicated a bilateral Achilles tendon rupture. The diagnosis was further confirmed by postoperative histological examination, which revealed Achilles tendonitis accompanied by regional calcification and chondrometaplasia. Interventions: Surgical reconstruction of the ruptured Achilles tendons was done through a modified minimally invasive whole FHL tendon transfer followed by physiotherapy. Outcomes: The patient was immobilized in a cast for the next 6 weeks, gradual weight bearing gradually was then encouraged for another 6 weeks, and full weight-bearing started 3 months after surgery. By 6-month postoperation, the patient could walk and jog normally returned to his pre-injury working condition. Conclusion: Surgical intervention is among the primary treatment of chronic Achilles tendon rupture. However, one of the challenges in its treatment is providing suitable graft for tendon reconstruction. Our case presents a successful reconstruction procedure using less-invasive whole FHL transfer technique. This surgical technique provides satisfactory clinical and functional outcome and can be considered for future therapy.
C1 [Zhang, Xiangfeng; Wu, Yongping; Lu, Huang] Zhejiang Univ, Sch Med, Affiliated Hosp 2, Dept Orthoped Surg, Hangzhou 310000, Zhejiang, Peoples R China.
   [Ruan, Feng] Zhejiang Univ, Sch Med, Affiliated Hosp 2, Dept Emergency Med, Hangzhou, Zhejiang, Peoples R China.
RP Zhang, XF (通讯作者)，Zhejiang Univ, Sch Med, Affiliated Hosp 2, Dept Orthoped Surg, Hangzhou 310000, Zhejiang, Peoples R China.
EM 2318099@zju.edu.cn
CR BOYDEN EM, 1995, CLIN ORTHOP RELAT R, P150
   Flint JH, 2014, AM J SPORT MED, V42, P235, DOI 10.1177/0363546513490656
   Jennings AG, 2002, J BONE JOINT SURG BR, V84B, P361, DOI 10.1302/0301-620X.84B3.11559
   KUWADA G T, 1990, Journal of Foot Surgery, V29, P361
   Leslie Hamish D H, 2005, Foot Ankle Clin, V10, P357, DOI 10.1016/j.fcl.2005.01.009
   Lui TH, 2011, FOOT ANKLE INT, V32, P730, DOI 10.3113/FAI.2011.0730
   Maffulli N, 2013, AM J SPORT MED, V41, P1100, DOI 10.1177/0363546513479017
   Maffulli N, 2013, AM J SPORT MED, V41, P865, DOI 10.1177/0363546512473819
   Maffulli N, 2010, AM J SPORT MED, V38, P2304, DOI 10.1177/0363546510376619
   McClelland D, 2004, SURG-J R COLL SURG E, V2, P209, DOI 10.1016/S1479-666X(04)80002-7
   Panchbhavi VK, 2007, TECH FOOT ANKLE SURG, V6, P123
   Spratley EM, 2013, FOOT ANKLE INT, V34, P1286, DOI 10.1177/1071100713487724
   Sullivan RJ, 2006, FOOT ANKLE INT, V27, P363, DOI 10.1177/107110070602700508
   Tay D, 2010, ANN ACAD MED SINGAP, V39, P58
   Uchikawa Y, 2013, J PLAST RECONSTR AES, V66, pE281, DOI 10.1016/j.bjps.2013.03.039
   Us AK, 1997, ARCH ORTHOP TRAUM SU, V116, P408
   Wegrzyn J, 2010, INT ORTHOP, V34, P1187, DOI 10.1007/s00264-009-0859-1
NR 17
TC 0
Z9 0
U1 1
U2 2
PU LIPPINCOTT WILLIAMS & WILKINS
PI PHILADELPHIA
PA TWO COMMERCE SQ, 2001 MARKET ST, PHILADELPHIA, PA 19103 USA
SN 0025-7974
EI 1536-5964
J9 MEDICINE
JI Medicine (Baltimore)
PD AUG 28
PY 2020
VL 99
IS 35
AR e21742
DI 10.1097/MD.0000000000021742
PG 4
WC Medicine, General & Internal
WE Science Citation Index Expanded (SCI-EXPANDED)
SC General & Internal Medicine
GA OC4MP
UT WOS:000579132500037
PM 32871894
OA gold, Green Published
DA 2022-07-22
ER

PT J
AU Ding, HX
   Xu, YS
   Li, J
   Yue, K
AF Ding, Haixiang
   Xu, Yingshu
   Li, Jie
   Yue, Kai
TI Comparison of the curative effect of modified medial arc incision of
   achilles tendon and traditional straight incision in the treatment of
   old achilles tendon rupture
SO PAKISTAN JOURNAL OF PHARMACEUTICAL SCIENCES
LA English
DT Article
DE Old achilles tendon; rupture; incision; skin; necrosis
AB Modified medial arc incision of Achilles tendon and traditional straight incision were applied to treat 50 cases of old rupture of Achilles tendon. Among them, there were 20 cases involved in the modified medial arc incision of Achilles tendon (group A). Furthermore, 30 cases accepted traditional straight incision treatment (group B). The same rehabilitation program was applied in both groups, combined with the evaluation using the clinical objective examination, subjective satisfaction of patients and the American Orthopaedic Foot and Ankle Society (AOFAS) scores. There was significant difference between group A and group B in the recovery time to normal motion ranges (P<0.05). The average AOFAS score of group A was higher more than (P<0.05). In addition, patients from the two groups did not suffer from cutaneous sensory disturbance of the dorsum of foot due to sural nerve injury, or no deep venous thrombosis. Except for 1 patient with deep infection, all patients recovered to normal motion ranges. The overall curative effect of modified medial arc incision of Achilles tendon is superior to traditional straight incision in the treatment of old Achilles tendon rupture, which is conducive to the early recovery to normal motion ranges before injury.
C1 [Ding, Haixiang] Jiangnan Univ, Dept Emergency, Affiliated Hosp, Wuxi, Jiangsu, Peoples R China.
   [Xu, Yingshu; Li, Jie; Yue, Kai] First Peoples Hosp Longquanyi Dist Chengdu, Dept Orthoped, Chengdu, Sichuan, Peoples R China.
RP Yue, K (通讯作者)，First Peoples Hosp Longquanyi Dist Chengdu, Dept Orthoped, Chengdu, Sichuan, Peoples R China.
EM 1562629213@qq.com
CR ARNER O, 1959, Acta Chir Scand, V116, P484
   CARDEN DG, 1987, J BONE JOINT SURG BR, V69, P416, DOI 10.1302/0301-620X.69B3.3294839
   Elias I, 2007, FOOT ANKLE INT, V28, P1238, DOI 10.3113/FAI.2007.1238
   Flint JH, 2014, AM J SPORT MED, V42, P235, DOI 10.1177/0363546513490656
   [高武长 Gao Wuchang], 2015, [中国修复重建外科杂志, Chinese Journal of Reparative and Reconstructive Surgery], V29, P563
   Guclu B, 2016, FOOT ANKLE INT, V37, P737, DOI 10.1177/1071100716642753
   INGLIS AE, 1981, CLIN ORTHOP RELAT R, P160
   Jiang N, 2012, INT ORTHOP, V36, P765, DOI 10.1007/s00264-011-1431-3
   Lui TH, 2007, KNEE SURG SPORT TR A, V15, P1163, DOI 10.1007/s00167-007-0352-6
   Lui TH, 2010, FOOT ANKLE INT, V31, P828, DOI 10.3113/FAI.2010.0828
   Maffulli N, 2008, BMC MUSCULOSKEL DIS, V9, DOI 10.1186/1471-2474-9-100
   Movin Tomas, 2005, Foot Ankle Clin, V10, P331, DOI 10.1016/j.fcl.2005.01.003
   Myerson M S, 1999, Instr Course Lect, V48, P219
   Nestorson J, 2000, ACTA ORTHOP SCAND, V71, P64, DOI 10.1080/00016470052943928
   Neufeld SK, 2014, FOOT ANKLE CLIN, V19, P73, DOI 10.1016/j.fcl.2013.10.005
   Sun SH, 2007, CHINA J ORTHOP TRAUM, V20, P106
   Tian QS, 2014, J CLIN SURG, V22, P370
   Yang JJ, 2011, J PRAC ORTHOP, V17, P404
   Yang YM, 2014, CHINA MED HERALD, V11, P49
   Yepes H, 2010, J BONE JOINT SURG AM, V92A, P1215, DOI 10.2106/JBJS.I.00743
   Yu CL, 1994, CHIN J SPOR MED, V13, P17
   [张丙磊 Zhang Binglei], 2011, [中华创伤骨科杂志, Chinese Journal of Orthopaedic Trauma], V13, P943
   林勇彬, 2011, [中华创伤骨科杂志, Chinese Journal of Orthopaedic Trauma], V13, P693
NR 23
TC 0
Z9 0
U1 0
U2 1
PU UNIV KARACHI
PI KARACHI
PA UNIV CAMPUS, FAC PHARMACY, KARACHI, 75270, PAKISTAN
SN 1011-601X
J9 PAK J PHARM SCI
JI Pak. J. Pharm. Sci.
PD MAY
PY 2020
VL 33
IS 3
BP 1087
EP 1093
DI 10.36721/PJPS.2020.33.3.REG.1087-1093.1
PG 7
WC Pharmacology & Pharmacy
WE Science Citation Index Expanded (SCI-EXPANDED)
SC Pharmacology & Pharmacy
GA KZ9HT
UT WOS:000523571000023
PM 33191233
DA 2022-07-22
ER

PT J
AU Pedersen, MH
   Wahlsten, LR
   Gronborg, H
   Gislason, GH
   Petersen, MM
   Bonde, AN
AF Pedersen, Melissa Hornbaek
   Wahlsten, Liv Riisager
   Gronborg, Henrik
   Gislason, Gunnar Hilmar
   Petersen, Michael Mork
   Bonde, Anders Nissen
TI Symptomatic Venous Thromboembolism After Achilles Tendon Rupture: A
   Nationwide Danish Cohort Study of 28,546 Patients With Achilles Tendon
   Rupture
SO AMERICAN JOURNAL OF SPORTS MEDICINE
LA English
DT Article
DE risk factors; incidence; Achilles tendon rupture; venous
   thromboembolism; deep venous thrombosis; pulmonary embolism; Achilles
   tendon; cardiovascular physiology; injury prevention and epidemiology
ID LOWER-LIMB IMMOBILIZATION; DEEP-VEIN THROMBOSIS; CAST IMMOBILIZATION;
   SURGICAL-TREATMENT; RANDOMIZED-TRIAL; RISK; THROMBOPROPHYLAXIS;
   EPIDEMIOLOGY; KNEE; METAANALYSIS
AB Background: Venous thromboembolism (VTE) is a well-known complication of Achilles tendon rupture (ATR) and carries a high risk of morbidity and mortality. Although routine thromboprophylaxis for patients with ATR is not recommended, sparse knowledge is available regarding risk factors associated with VTE in patients with ATR. Purpose: To use Danish nationwide registers to identify incidence rates for symptomatic VTE and risk factors associated with increased risk of developing VTE in patients with ATR. Study Design: Cohort study; Level of evidence, 3. Methods: By crosslinking nationwide registers, we identified all patients with diagnosed ATR in Denmark from 1997 to 2015. We stratified patients into 4 groups by age and treatment modality (ie, operative vs nonoperative treatment). The main outcome was VTE within 180 days. We calculated crude incidence rates and considered age, sex, year, comorbidities, and medications as risk factors for VTE in Poisson regression models. Results: We identified 28,546 patients with ATR, of whom 389 (1.36%) were hospitalized with VTE during the follow-up period: 278 due to deep vein thromboses and 138 due to pulmonary embolism. Incidence rates were highest during the first month and ranged from 4.6 to 14.6 events per 100 person-years. VTEs were most frequent among nonoperatively treated patients aged >= 50 years. In Poisson regression analyses, having had VTE beforehand was associated with an increased risk of VTE, as was male sex in the nonoperative treatment group aged >= 50 years; among women <50 years of age, hormonal contraceptives led to a 4- to 6-fold higher risk of VTE compared with patients in the same group without the equivalent risk factor. Conclusion: In this nationwide cohort of patients with ATR, 1.36% developed symptomatic VTE during follow-up. Hormonal contraception, previous VTE, older age group, and male sex increased the risk of VTE. Taken together, the results of the present study suggest that focus on risk stratification and initiatives to prevent VTE might be warranted. A randomized controlled trial could answer this question.
C1 [Pedersen, Melissa Hornbaek; Wahlsten, Liv Riisager; Gronborg, Henrik; Gislason, Gunnar Hilmar; Petersen, Michael Mork; Bonde, Anders Nissen] Copenhagen Univ Hosp, Rigshosp, Dept Orthoped Surg & Trauma Ctr, Blegdamsvej 9, DK-2100 Copenhagen O, Denmark.
   [Pedersen, Melissa Hornbaek; Petersen, Michael Mork] Copenhagen Univ Hosp, Rigshosp, Trauma Ctr, Copenhagen, Denmark.
   [Pedersen, Melissa Hornbaek; Gronborg, Henrik] Copenhagen Univ Hosp Herlev Gentofte, Dept Orthoped Surg, Herlev, Denmark.
   [Wahlsten, Liv Riisager; Gislason, Gunnar Hilmar; Bonde, Anders Nissen] Copenhagen Univ Hosp Herlev Gentofte, Dept Cardiol, Hellerup, Denmark.
RP Pedersen, MH (通讯作者)，Copenhagen Univ Hosp, Rigshosp, Dept Orthoped Surg & Trauma Ctr, Blegdamsvej 9, DK-2100 Copenhagen O, Denmark.
EM 1510mhp@gmail.com
RI Gislason, Gunnar H/B-7561-2009
OI Gislason, Gunnar H/0000-0002-0548-402X; Petersen, Michael
   Mork/0000-0002-2324-6420
CR Agnelli G, 2013, NEW ENGL J MED, V369, P799, DOI 10.1056/NEJMoa1302507
   Anderson FA, 2003, CIRCULATION, V107, pI9, DOI 10.1161/01.CIR.0000078469.07362.E6
   Barfod KW, 2015, UGESKRIFT LAEGER, V177
   Bauersachs R, 2010, NEW ENGL J MED, V363, P2499, DOI 10.1056/NEJMoa1007903
   Bergan JJ, 2006, NEW ENGL J MED, V355, P488, DOI 10.1056/NEJMra055289
   Blanco JA, 2018, J FOOT ANKLE SURG, V57, P484, DOI 10.1053/j.jfas.2017.10.036
   Braithwaite I, 2016, J THROMB HAEMOST, V14, P331, DOI 10.1111/jth.13224
   Calder JDF, 2016, KNEE SURG SPORT TR A, V24, P1409, DOI 10.1007/s00167-015-3976-y
   Deng SL, 2017, J FOOT ANKLE SURG, V56, P1236, DOI 10.1053/j.jfas.2017.05.036
   Domeij-Arverud E, 2015, BONE JOINT J, V97B, P675, DOI 10.1302/0301-620X.97B5.34581
   Farrell JJ, 2016, CARDIOVASC DIAGN THE, V6, P623, DOI 10.21037/cdt.2016.11.22
   Ganestam A, 2016, KNEE SURG SPORT TR A, V24, P3730, DOI 10.1007/s00167-015-3544-5
   Healy B, 2010, J BONE JOINT SURG BR, V92B, P646, DOI 10.1302/0301-620X.92B5.23241
   Heit JA, 2008, ARTERIOSCL THROM VAS, V28, P370, DOI 10.1161/ATVBAHA.108.162545
   Helweg-Larsen K, 2011, SCAND J PUBLIC HEALT, V39, P26, DOI 10.1177/1403494811399958
   Heyes GJ, 2015, EUR J TRAUMA EMERG S, V41, P273, DOI 10.1007/s00068-014-0408-5
   Houshian S, 1998, INJURY, V29, P651, DOI 10.1016/S0020-1383(98)00147-8
   Kildemoes HW, 2011, SCAND J PUBLIC HEALT, V39, P38, DOI 10.1177/1403494810394717
   Kou J, 2010, J AM ACAD ORTHOP SUR, V18, P511, DOI 10.5435/00124635-201008000-00008
   Lapidus LJ, 2007, J ORTHOP TRAUMA, V21, P52, DOI 10.1097/01.bot.0000250741.65003.14
   Lapidus LJ, 2013, BMC MUSCULOSKEL DIS, V14, DOI 10.1186/1471-2474-14-177
   Lassen MR, 2008, NEW ENGL J MED, V358, P2776, DOI 10.1056/NEJMoa076016
   Lassen MR, 2009, NEW ENGL J MED, V361, P594, DOI 10.1056/NEJMoa0810773
   Lidegaard O, 2002, CONTRACEPTION, V65, P187, DOI 10.1016/S0010-7824(01)00307-9
   Lidegaard Ojvind, 2009, BMJ, V339, pb2890, DOI 10.1136/bmj.b2890
   Lynge E, 2011, SCAND J PUBLIC HEALT, V39, P30, DOI 10.1177/1403494811401482
   Makhdom AM, 2013, J FOOT ANKLE SURG, V52, P584, DOI 10.1053/j.jfas.2013.03.001
   Nilsson-Helander K, 2010, AM J SPORT MED, V38, P2186, DOI 10.1177/0363546510376052
   Nilsson-Helander K, 2009, KNEE SURG SPORT TR A, V17, P1234, DOI 10.1007/s00167-009-0727-y
   Olsson N, 2013, AM J SPORT MED, V41, P2867, DOI 10.1177/0363546513503282
   Patel A, 2012, CLIN ORTHOP RELAT R, V470, P270, DOI 10.1007/s11999-011-2166-6
   Pedersen CB, 2011, SCAND J PUBLIC HEALT, V39, P22, DOI 10.1177/1403494810387965
   Saragas Nikiforos Pandelis, 2011, Foot Ankle Surg, V17, P263, DOI 10.1016/j.fas.2010.12.002
   Severinsen MT, 2010, J CLIN EPIDEMIOL, V63, P223, DOI 10.1016/j.jclinepi.2009.03.018
   Sheth U, 2017, BONE JOINT J, V99B, P78, DOI [10.1302/0301-620X.99B1.BJJ-2016-0434.R1, 10.1302/0301-620x.99b1.bjj-2016-0434.r1]
   Sundboll J, 2016, BMJ OPEN, V6, DOI 10.1136/bmjopen-2016-012832
   Twaddle BC, 2007, AM J SPORT MED, V35, P2033, DOI 10.1177/0363546507307503
   van Stralen KJ, 2008, ARCH INTERN MED, V168, P21, DOI 10.1001/archinternmed.2007.5
   Wahlsten LR, 2015, J BONE JOINT SURG AM, V97A, P470, DOI 10.2106/JBJS.N.00307
   Willits K, 2010, J BONE JOINT SURG AM, V92A, P2767, DOI 10.2106/JBJS.I.01401
NR 40
TC 7
Z9 8
U1 0
U2 3
PU SAGE PUBLICATIONS INC
PI THOUSAND OAKS
PA 2455 TELLER RD, THOUSAND OAKS, CA 91320 USA
SN 0363-5465
EI 1552-3365
J9 AM J SPORT MED
JI Am. J. Sports Med.
PD NOV
PY 2019
VL 47
IS 13
BP 3229
EP 3237
AR 0363546519876054
DI 10.1177/0363546519876054
EA OCT 2019
PG 9
WC Orthopedics; Sport Sciences
WE Science Citation Index Expanded (SCI-EXPANDED)
SC Orthopedics; Sport Sciences
GA JI9IR
UT WOS:000491817400001
PM 31574237
DA 2022-07-22
ER

PT J
AU Westin, O
   Helander, KN
   Silbernagel, KG
   Moller, M
   Kalebo, P
   Karlsson, J
AF Westin, Olof
   Helander, Katarina Nilsson
   Silbernagel, Karin Gravare
   Moller, Michael
   Kalebo, Peter
   Karlsson, Jon
TI Acute Ultrasonography Investigation to Predict Reruptures and Outcomes
   in Patients With an Achilles Tendon Rupture
SO ORTHOPAEDIC JOURNAL OF SPORTS MEDICINE
LA English
DT Article
DE ultrasonography; Achilles tendon rupture; rerupture; diastasis
ID REPAIR
AB Background: The optimal treatment for acute Achilles tendon ruptures is still an ongoing debate. Acute ultrasonography (US) investigation to measure the diastasis between the tendon ends has previously been used to classify acute Achilles tendon ruptures; however, no study has used US to predict reruptures and functional outcomes.
   Purpose: To investigate whether acute US can be used to predict the risk of reruptures and outcomes after treatment of an acute Achilles tendon rupture.
   Study Design: Cohort study; Level of evidence, 2.
   Methods: Forty-five patients (37 men, 8 women) with amean age of 39 +/- 9.2 years (range, 23-59 years) from a cohort of 97 patients participating in a randomized controlled study comparing surgical and nonsurgical treatment were included. US was performed within 72 hours from the index injury. Diastasis between the tendon ends was documented. Reruptures were documented, and the patients' functional outcomes were measured 12 months after injury.
   Results: Patients with a diastasis of >10 mm treated nonsurgically had a higher degree of rerupture. In the nonsurgically treated group, 3 of 4 patients with a diastasis of >10 mm suffered from rerupture (P < .001). Moreover, in the nonsurgical group, there was significantly worse outcomes in patients with a diastasis of >5 mm in terms of patient-reported outcomes using the Achilles tendon Total Rupture Score (ATRS) (P = .004) and heel-rise height at 12 months (P = .048) compared with the group with a lesser degree of tendon separation.
   Conclusion: US may be a useful tool to predict the risk of rerupture and greater degree of functional deficit. It may be an important measure in a clinical treatment algorithm for deciding whether a patient will benefit from surgical intervention after an acute Achilles tendon rupture.
C1 [Westin, Olof; Helander, Katarina Nilsson; Silbernagel, Karin Gravare; Moller, Michael; Karlsson, Jon] Sahlgrens Univ Hosp, Gothenburg, Sweden.
   [Kalebo, Peter] Sahlgrens Univ Hosp, Dept Radiol, Gothenburg, Sweden.
   [Westin, Olof; Helander, Katarina Nilsson; Silbernagel, Karin Gravare; Moller, Michael; Karlsson, Jon] Univ Gothenburg, Sahlgrenska Acad, Inst Clin Sci, Dept Orthopaed, S-43180 Molndal, Sweden.
   [Westin, Olof; Moller, Michael; Karlsson, Jon] Sahlgrens Univ Hosp, Molndal, Sweden.
   [Helander, Katarina Nilsson] Hallands Sjukhus, Kungsbacka, Sweden.
   [Silbernagel, Karin Gravare] Univ Delaware, Dept Phys Therapy, Newark, DE USA.
RP Westin, O (通讯作者)，Univ Gothenburg, Sahlgrenska Acad, Inst Clin Sci, Dept Orthopaed, S-43180 Molndal, Sweden.
EM olof.westin@gmail.com
OI Gravare Silbernagel, Karin/0000-0001-7566-407X
CR Amlang Michael H, 2011, ISRN Orthop, V2011, P869703, DOI 10.5402/2011/869703
   Bhandari M, 2002, CLIN ORTHOP RELAT R, P190
   Carmont MR, 2013, FOOT ANKLE SURG, V19, P245, DOI 10.1016/j.fas.2013.06.007
   Ganestam A, 2016, KNEE SURG SPORT TR A, V24, P3730, DOI 10.1007/s00167-015-3544-5
   Gibbon WW, 1999, BRIT J SPORT MED, V33, P129, DOI 10.1136/bjsm.33.2.129
   KEENE JS, 1989, AM J SPORT MED, V17, P333, DOI 10.1177/036354658901700304
   Khan RJK, 2005, J BONE JOINT SURG AM, V87A, P2202, DOI 10.2106/JBJS.D.03049
   Kotnis R, 2006, AM J SPORT MED, V34, P1395, DOI 10.1177/0363546506288678
   Lin TW, 2004, J BIOMECH, V37, P865, DOI 10.1016/j.jbiomech.2003.11.005
   Maffulli N, 1998, Bull Hosp Jt Dis, V57, P152
   Maffulli N, 1998, AM J SPORT MED, V26, P266, DOI 10.1177/03635465980260021801
   Nilsson-Helander K, 2007, AM J SPORT MED, V35, P421, DOI 10.1177/0363546506294856
   Nilsson-Helander K, 2010, AM J SPORT MED, V38, P2186, DOI 10.1177/0363546510376052
   Olsson N, 2014, AM J SPORT MED, V42, P1448, DOI 10.1177/0363546514527409
   Olsson N, 2013, AM J SPORT MED, V41, P2867, DOI 10.1177/0363546513503282
   Olsson N, 2011, KNEE SURG SPORT TR A, V19, P1385, DOI 10.1007/s00167-011-1511-3
   Silbernagel KG, 2006, KNEE SURG SPORT TR A, V14, P1207, DOI 10.1007/s00167-006-0150-6
   THERMANN H, 1989, ORTHOPADE, V18, P321
   THERMANN H, 1995, UNFALLCHIRURG, V98, P21
   Voleti PB, 2012, ANNU REV BIOMED ENG, V14, P47, DOI 10.1146/annurev-bioeng-071811-150122
NR 20
TC 24
Z9 27
U1 0
U2 4
PU SAGE PUBLICATIONS INC
PI THOUSAND OAKS
PA 2455 TELLER RD, THOUSAND OAKS, CA 91320 USA
EI 2325-9671
J9 ORTHOP J SPORTS MED
JI Orthop. J. Sports Med.
PD OCT
PY 2016
VL 4
IS 10
AR 2325967116667920
DI 10.1177/2325967116667920
PG 7
WC Orthopedics; Sport Sciences
WE Science Citation Index Expanded (SCI-EXPANDED)
SC Orthopedics; Sport Sciences
GA DZ0SN
UT WOS:000385550100007
PM 27781212
OA Green Published, gold
DA 2022-07-22
ER

PT J
AU Santrock, RD
   Friedmann, AJ
   Hanselman, AE
AF Santrock, Robert D.
   Friedmann, Andrew J.
   Hanselman, Andrew E.
TI Acute Rupture Open Repair Techniques
SO CLINICS IN PODIATRIC MEDICINE AND SURGERY
LA English
DT Article
DE Achilles tendon; Acute rupture; Open repair; Limited open; Surgical
   technique
ID ACHILLES-TENDON RUPTURE; RANDOMIZED CONTROLLED-TRIALS; NONOPERATIVE
   TREATMENT; NONSURGICAL TREATMENTS; MANAGEMENT; SURGERY
AB Achilles tendon injuries can be serious injuries requiring either operative or nonoperative management. For appropriate surgical candidates, operative intervention may provide lower rerupture rates and adequate end-to-end tendon healing. Our preference is an open Achilles tendon repair, specifically a limited open technique using the PARS device (Arthrex, Naples, FL). Postoperatively, we use functional rehabilitation and early range of motion. Although the current literature remains controversial regarding operative versus nonoperative management, the authors have obtained satisfactory results in appropriately chosen surgical candidates.
C1 [Santrock, Robert D.; Friedmann, Andrew J.; Hanselman, Andrew E.] West Virginia Univ, Robert C Byrd Hlth Sci Ctr, Dept Orthopaed, POB 9196 South, Morgantown, WV 26506 USA.
RP Santrock, RD (通讯作者)，West Virginia Univ, Robert C Byrd Hlth Sci Ctr, Dept Orthopaed, POB 9196 South, Morgantown, WV 26506 USA.
EM rsantrock@hsc.wvu.edu
OI Hanselman, Andrew/0000-0002-9005-7355
CR CETTI R, 1993, AM J SPORT MED, V21, P791, DOI 10.1177/036354659302100606
   Chiodo CP, 2010, J AM ACAD ORTHOP SUR, V18, P503, DOI 10.5435/00124635-201008000-00007
   Costa ML, 2006, J BONE JOINT SURG BR, V88B, P69, DOI 10.1302/0301-620X.88B1.16549
   Coughlin MJ, 1999, SURG FOOT ANKLE, P835
   Cretnik A, 2005, AM J SPORT MED, V33, P1369, DOI 10.1177/0363546504271501
   Erikson B, 2015, ORTHOP J SPORTS MED, V3
   Fujikawa A, 2007, AM J ROENTGENOL, V189, P1169, DOI 10.2214/AJR.07.2260
   Hsu AR, 2015, FOOT ANKLE INT, V36, P1279, DOI 10.1177/1071100715589632
   INGLIS AE, 1976, J BONE JOINT SURG AM, V58, P990, DOI 10.2106/00004623-197658070-00015
   Khan RJK, 2010, COCHRANE DB SYST REV, DOI 10.1002/14651858.CD003674.pub4
   Lantto I, 2016, AM J SPORT MED, V44, P2406, DOI 10.1177/0363546516651060
   Lapidus LJ, 2013, BMC MUSCULOSKEL DIS, V14, DOI 10.1186/1471-2474-14-177
   Leppilahti J, 1996, ACTA ORTHOP SCAND, V67, P277, DOI 10.3109/17453679608994688
   Longo UG, 2013, FOOT ANKLE CLIN, V18, P319, DOI 10.1016/j.fcl.2013.02.009
   Maffulli N, 1999, J BONE JOINT SURG AM, V81A, P1019, DOI 10.2106/00004623-199907000-00017
   Makhdom AM, 2013, J FOOT ANKLE SURG, V52, P584, DOI 10.1053/j.jfas.2013.03.001
   Moller M, 2001, J BONE JOINT SURG BR, V83B, P843
   Mosser P, 2015, J FOOT ANKLE SURG, V54, P2, DOI 10.1053/j.jfas.2014.09.040
   Nilsson-Helander K, 2010, AM J SPORT MED, V38, P2186, DOI 10.1177/0363546510376052
   Sarrafian SK., 1993, ANATOMY FOOT ANKLE, V2nd ed.
   STROCCHI R, 1991, FOOT ANKLE, V12, P100, DOI 10.1177/107110079101200207
   Twaddle BC, 2007, AM J SPORT MED, V35, P2033, DOI 10.1177/0363546507307503
   Weber M, 2003, AM J SPORT MED, V31, P685, DOI 10.1177/03635465030310050901
   Wilkins R, 2012, AM J SPORT MED, V40, P2154, DOI 10.1177/0363546512453293
   Willits K, 2010, J BONE JOINT SURG AM, V92A, P2767, DOI 10.2106/JBJS.I.01401
NR 25
TC 1
Z9 1
U1 1
U2 3
PU W B SAUNDERS CO-ELSEVIER INC
PI PHILADELPHIA
PA 1600 JOHN F KENNEDY BOULEVARD, STE 1800, PHILADELPHIA, PA 19103-2899 USA
SN 0891-8422
EI 1558-2302
J9 CLIN PODIATR MED SUR
JI Clin. Podiatr. Med. Surg.
PD APR
PY 2017
VL 34
IS 2
BP 245
EP +
DI 10.1016/j.cpm.2016.10.010
PG 7
WC Orthopedics
WE Science Citation Index Expanded (SCI-EXPANDED)
SC Orthopedics
GA EQ3ZZ
UT WOS:000398013800011
PM 28257677
DA 2022-07-22
ER

PT J
AU Lantto, I
   Heikkinen, J
   Flinkkila, T
   Ohtonen, P
   Leppilahti, J
AF Lantto, I.
   Heikkinen, J.
   Flinkkila, T.
   Ohtonen, P.
   Leppilahti, J.
TI Epidemiology of Achilles tendon ruptures: Increasing incidence over a
   33-year period
SO SCANDINAVIAN JOURNAL OF MEDICINE & SCIENCE IN SPORTS
LA English
DT Article
DE Incidence; Achilles tendon rupture; complications
ID NONOPERATIVE TREATMENT; METAANALYSIS; REPAIR
AB We investigated the epidemiology of total Achilles tendon ruptures and complication rates after operative and nonoperative treatments over a 33-year period in Oulu, Finland. Patients with Achilles tendon ruptures from 1979 to 2011 in Oulu were identified from hospital patient records. Demographic data, treatment method, and complications were collected retrospectively from medical records. Overall and sex- and age-specific incidence rates were calculated with 95% confidence intervals (CIs). The overall incidence per 100000 person-years increased from 2.1 (95% CI 0.3-7.7) in 1979 to 21.5 (95% CI 14.6-30.6) in 2011. The incidence increased in all age groups. The mean annual increase in incidence was 2.4% (95% CI 1.3-4.7) higher for non-sports-related ruptures than for sports-related ruptures (P=0.036). The incidence of sports-related ruptures increased during the second 11-year period whereas the incidence of non-sports-related ruptures increased steadily over the entire study period. Infection was four times more common after operative treatment compared with nonoperative treatment, re-rupture rates were similar. The incidence of Achilles tendon ruptures increased in all age groups over a 33-year period. Increases were mainly due to sports-related injuries in the second 11-year period and non-sports-related injuries in the last 11-year period.
C1 [Lantto, I.; Heikkinen, J.; Flinkkila, T.; Leppilahti, J.] Oulu Univ Hosp, Div Orthopaed & Trauma Surg, Dept Surg, Oulu, Finland.
   [Ohtonen, P.] Oulu Univ Hosp, Dept Surg & Anesthesiol, Oulu, Finland.
RP Lantto, I (通讯作者)，Oulu Univ Hosp, Div Orthopaed & Trauma Surg, Dept Surg, POB 21, FIN-90029 Oys Oulu, Finland.
EM iikka.lantto@ppshp.fi
OI Leppilahti, Juhana/0000-0001-5778-246X; Heikkinen,
   Juuso/0000-0001-9137-788X
CR Beri A, 2009, J CARDIOVASC PHARM, V53, P401, DOI 10.1097/FJC.0b013e3181a0ce8b
   Bhandari M, 2002, CLIN ORTHOP RELAT R, P190
   Garras DN, 2012, CLIN ORTHOP RELAT R, V470, P2268, DOI 10.1007/s11999-012-2355-y
   Gwynne-Jones DP, 2011, FOOT ANKLE INT, V32, P337, DOI 10.3113/FAI.2011.0337
   Houshian S, 1998, INJURY, V29, P651, DOI 10.1016/S0020-1383(98)00147-8
   Jarvinen Tero A H, 2005, Foot Ankle Clin, V10, P255, DOI 10.1016/j.fcl.2005.01.013
   Jones Matthew P, 2012, J Bone Joint Surg Am, V94, pe88, DOI 10.2106/JBJS.J.01829
   JOZSA L, 1989, AM J SPORT MED, V17, P338, DOI 10.1177/036354658901700305
   Kangas J, 2003, J TRAUMA, V54, P1171, DOI 10.1097/01.TA.0000047945.20863.A2
   KANNUS P, 1991, J BONE JOINT SURG AM, V73A, P1507, DOI 10.2106/00004623-199173100-00009
   Keating JF, 2011, J BONE JOINT SURG BR, V93B, P1071, DOI 10.1302/0301-620X.93B8.25998
   Khan RJK, 2005, J BONE JOINT SURG AM, V87A, P2202, DOI 10.2106/JBJS.D.03049
   Leppilahti J, 1998, SPORTS MED, V25, P79, DOI 10.2165/00007256-199825020-00002
   Leppilahti J, 1996, ACTA ORTHOP SCAND, V67, P277, DOI 10.3109/17453679608994688
   Levi N, 1997, INJURY, V28, P311, DOI 10.1016/S0020-1383(96)00200-8
   Maffulli N, 1999, CLIN J SPORT MED, V9, P157, DOI 10.1097/00042752-199907000-00007
   Marie I, 2008, ARTHRIT RHEUM-ARTHR, V59, P367, DOI 10.1002/art.23309
   Moller A, 1996, ACTA ORTHOP SCAND, V67, P479, DOI 10.3109/17453679608996672
   Nyyssonen T, 2008, SCAND J SURG, V97, P272, DOI 10.1177/145749690809700312
   Pajala A, 2009, J BONE JOINT SURG AM, V91A, P1092, DOI 10.2106/JBJS.G.01089
   Soroceanu A, 2012, J BONE JOINT SURG AM, V94A, P2136, DOI 10.2106/JBJS.K.00917
   Suchak AA, 2005, FOOT ANKLE INT, V26, P932, DOI 10.1177/107110070502601106
   Waldecker Ute, 2012, Foot Ankle Surg, V18, P119, DOI 10.1016/j.fas.2011.04.007
   Wallace RGH, 2011, J BONE JOINT SURG BR, V93B, P1362, DOI 10.1302/0301-620X.93B10.26187
NR 24
TC 172
Z9 183
U1 2
U2 20
PU WILEY
PI HOBOKEN
PA 111 RIVER ST, HOBOKEN 07030-5774, NJ USA
SN 0905-7188
EI 1600-0838
J9 SCAND J MED SCI SPOR
JI Scand. J. Med. Sci. Sports
PD FEB
PY 2015
VL 25
IS 1
BP E133
EP E138
DI 10.1111/sms.12253
PG 6
WC Sport Sciences
WE Science Citation Index Expanded (SCI-EXPANDED)
SC Sport Sciences
GA CA3XT
UT WOS:000348839100015
PM 24862178
DA 2022-07-22
ER

PT J
AU Albano, AW
   Nelson, V
AF Albano, Andrew W., Jr.
   Nelson, Vicki
TI Approaching Foot and Ankle Injuries in the Ambulatory Setting
SO PRIMARY CARE
LA English
DT Article
DE Achilles tendon rupture; Ankle sprain; Syndesmotic injury; Metatarsal
   stress fracture; Jones fracture; Plantar fasciitis; Sports medicine
ID SHOCK-WAVE THERAPY; ACHILLES-TENDON; PLANTAR FASCIITIS;
   STRESS-FRACTURES; MANAGEMENT; OSTEOARTHRITIS; RULES; ACCURACY; RUPTURES;
   MRI
AB Foot and ankle injuries account for a significant volume of primary care office visits each year. Given the incidence of injury and concern for long-term sequelae, it is imperative that primary care physicians familiarize themselves with commonly encountered foot and ankle injuries. Coupling a sound understanding of key anatomic structures with an appropriately gathered history can help to quickly narrow the differential diagnosis in this clinical presentation. This article focuses on key elements from the history and physical examination as well as provides a concise review of imaging modalities and recommended treatment strategies.
C1 [Albano, Andrew W., Jr.] Prisma Hlth, Dept Family Med, Qual & Med Affairs, 877 West Faris Rd, Greenville, SC 29605 USA.
   [Albano, Andrew W., Jr.; Nelson, Vicki] Univ South Carolina, Sch Med Greenville, Greenville, SC USA.
   [Nelson, Vicki] Prisma Hlth, Dept Family Med, 877 West Faris Rd, Greenville, SC 29605 USA.
RP Albano, AW (通讯作者)，877 West Faris Rd, Greenville, SC 29605 USA.
EM Drew.Albano@prismahealth.org
RI Albano, Andrew/K-9471-2019
OI Albano, Andrew/0000-0002-7196-1269
CR Bachmann LM, 2003, BMJ-BRIT MED J, V326, P417, DOI 10.1136/bmj.326.7386.417
   Barelds I, 2018, SPORTS MED, V48, P2775, DOI 10.1007/s40279-018-0993-2
   Beard NM, 2018, ORTHOP CLIN N AM, V49, P109, DOI 10.1016/j.ocl.2017.08.013
   Birrer R B, 1988, J Am Board Fam Pract, V1, P274
   Bowes J, 2016, WORLD J ORTHOP, V7, P793, DOI 10.5312/wjo.v7.i12.793
   Brison RJ, 2016, BMJ-BRIT MED J, V355, DOI 10.1136/bmj.i5650
   Brown KW, 2004, AM J ROENTGENOL, V182, P131, DOI 10.2214/ajr.182.1.1820131
   Carbone A, 2017, J ORTHOP RES, V35, P397, DOI 10.1002/jor.23341
   Chimenti RL, 2017, FOOT ANKLE INT, V38, P1160, DOI 10.1177/1071100717723127
   Davda K, 2017, EFORT OPEN REV, V2, P281, DOI 10.1302/2058-5241.2.160047
   Doherty C, 2017, BRIT J SPORT MED, V51, P113, DOI 10.1136/bjsports-2016-096178
   Durrant B, 2011, J AM PODIAT MED ASSN, V101, P176, DOI 10.7547/1010176
   Egger AC, 2017, CURR REV MUSCULOSKE, V10, P72, DOI 10.1007/s12178-017-9386-7
   Ewalefo SO, 2018, CURR REV MUSCULOSKE, V11, P546, DOI 10.1007/s12178-018-9525-9
   Faleiro TB, 2016, ACTA ORTOP BRAS, V24, P52, DOI 10.1590/1413-785220162401139470
   Gill LE, 2018, ORTHOP RES REV, V10, P19, DOI 10.2147/ORR.S129990
   Goff JD, 2011, AM FAM PHYSICIAN, V84, P676
   Gollwitzer H, 2015, J BONE JOINT SURG AM, V97A, P701, DOI 10.2106/JBJS.M.01331
   Guelfi M, 2017, EUR REV MED PHARMACO, V21, P13
   Harmon KG, 2004, PRIMARY CARE, V31, P1025, DOI 10.1016/j.pop.2004.07.008
   Hemphill B, 2011, J FAM PRACTICE, V60, P759
   Hossain M, 2015, CLIN SPORT MED, V34, P769, DOI 10.1016/j.csm.2015.06.011
   Hsiao MY, 2015, RHEUMATOLOGY, V54, P1735, DOI 10.1093/rheumatology/kev010
   Kramer WC, 2011, INT J CLIN EXP MED, V4, P285
   Landorf Karl B, 2015, BMJ Clin Evid, V2015
   Li HY, 2016, BIOMED RES INT, V2016, DOI 10.1155/2016/6492597
   Maffulli G, 2017, MLTJ-MUSCLE LIGAMENT, V7, P46, DOI 10.11138/mltj/2017.7.1.046
   Maffulli N, 2014, JBJS REV, V2, DOI 10.2106/JBJS.RVW.M.00136
   Mayer SW, 2014, SPORTS HEALTH, V6, P481, DOI 10.1177/1941738113486588
   McCormack JR, 2016, SPORTS HEALTH, V8, P230, DOI 10.1177/1941738116631498
   Menz HB, 2013, J GERONTOL A-BIOL, V68, P1281, DOI 10.1093/gerona/glt048
   Miller L, 2015, AM FAM PHYSICIAN, V91, P794
   Molini L, 2014, J ULTRASOUND, V17, P125, DOI 10.1007/s40477-014-0072-y
   Mountjoy M, 2018, BRIT J SPORT MED, V52, P687, DOI 10.1136/bjsports-2018-099193
   Park JW, 2017, ULTRASONOGRAPHY, V36, P321, DOI 10.14366/usg.17008
   Pearce CJ, 2016, EFORT OPEN REV, V1, P383, DOI 10.1302/2058-5241.1.160024
   Pires RES, 2014, ANN MED HEALTH SCI R, V4, P432, DOI 10.4103/2141-9248.133473
   Porter DA, 2014, OPEN ACCESS J SPORTS, V5, P173, DOI 10.2147/OAJSM.S41564
   Reissig J, 2017, J AM OSTEOPATH ASSOC, V117, P98, DOI 10.7556/jaoa.2017.020
   Roussignol X, 2016, ORTHOP TRAUMATOL-SUR, V102, pS195, DOI 10.1016/j.otsr.2015.06.033
   Rubin A, 1996, AM FAM PHYSICIAN, V54, P5
   Rungprai Chamnanni, 2017, Open Orthop J, V11, P743, DOI 10.2174/1874325001711010743
   Saleh A, 2013, PRIMARY CARE, V40, P383, DOI 10.1016/j.pop.2013.02.015
   Schnetzke M, 2016, WORLD J ORTHOP, V7, P718, DOI 10.5312/wjo.v7.i11.718
   Schwieterman B, 2013, INT J SPORTS PHYS TH, V8, P416
   Smith MS, 2012, FRACTURE MANAGEMENT, P299
   STIELL IG, 1992, ANN EMERG MED, V21, P384, DOI 10.1016/S0196-0644(05)82656-3
   Tocci SL, 2007, FOOT ANKLE INT, V28, P166, DOI 10.3113/FAI.2007.0166
   Vuurberg G, 2018, BRIT J SPORT MED, V52, P956, DOI 10.1136/bjsports-2017-098106
   Willits K, 2010, J BONE JOINT SURG AM, V92A, P2767, DOI 10.2106/JBJS.I.01401
   Young CC, 2005, PRIMARY CARE, V32, P105, DOI 10.1016/j.pop.2004.11.002
   Yuen Chi Pan, 2017, Open Orthop J, V11, P670, DOI 10.2174/1874325001711010670
NR 52
TC 1
Z9 1
U1 1
U2 7
PU W B SAUNDERS CO-ELSEVIER INC
PI PHILADELPHIA
PA 1600 JOHN F KENNEDY BOULEVARD, STE 1800, PHILADELPHIA, PA 19103-2899 USA
SN 0095-4543
EI 1558-299X
J9 PRIMARY CARE
JI Primary Care
PD MAR
PY 2020
VL 47
IS 1
BP 133
EP +
DI 10.1016/j.pop.2019.10.007
PG 14
WC Primary Health Care; Medicine, General & Internal
WE Science Citation Index Expanded (SCI-EXPANDED)
SC General & Internal Medicine
GA KM2JG
UT WOS:000513948200011
PM 32014130
DA 2022-07-22
ER

PT J
AU Griffin, MJ
   Olson, K
   Heckmann, N
   Charlton, TP
AF Griffin, Matthew J.
   Olson, Kirstina
   Heckmann, Nathanael
   Charlton, Timothy P.
TI Realtime Achilles Ultrasound Thompson (RAUT) Test for the Evaluation and
   Diagnosis of Acute Achilles Tendon Ruptures
SO FOOT & ANKLE INTERNATIONAL
LA English
DT Article
DE Achilles tendon rupture; ultrasound
ID INJURY; ANKLE
AB Background: Acute complete Achilles tendon ruptures are commonly missed injuries. We propose the Realtime Achilles Ultrasound Thompson (RAUT) test, a Thompson test under ultrasound visualization, to aid in the diagnosis of these injuries. We hypothesized that RAUT testing would provide improved diagnostic characteristics compared with static ultrasound.
   Methods: Twenty-two consecutive patients with operatively confirmed acute Achilles tendon ruptures were prospectively evaluated with RAUT testing and static ultrasonography. RAUT video recordings and static ultrasound images from both ruptured and uninjured sides were randomized and graded by a group of novice reviewers and a group of expert attendings. From these observations, sensitivity, specificity, positive predictive value, and negative predictive value for RAUT and static ultrasound were calculated. In addition, interobserver coefficients were computed. Forty-seven novice reviewers and 11 foot and ankle attendings made a total of 4136 and 528 observations, respectively.
   Results: For static ultrasound, sensitivity and specificity were 76.8% and 74.8% for the novice reviewers and 79.6% and 86.4% for the attendings, respectively. For RAUT testing, sensitivity and specificity were 87.2% and 81.1% for the novice group and 86.4% and 91.7% for the attending group, respectively. The coefficient was 0.62 and 0.27 for novice and attending RAUT reviewers, indicating substantial and fair agreement, respectively, but only 0.46 and 0.12 for static ultrasonography, representing moderate and slight agreement, respectively.
   Conclusion: RAUT testing was a sensitive and specific test, providing a cost-effective adjunct to the clinical examination when diagnosing acute Achilles tendon ruptures. This test can be used by surgeons with minimal training in ultrasonography.
   Level of Evidence: Level II, diagnostic study.
C1 [Griffin, Matthew J.; Heckmann, Nathanael] Univ Southern Calif, Dept Orthopaed Surg, Keck Hosp, Los Angeles, CA USA.
   [Olson, Kirstina] Univ Calif San Francisco, Med Ctr, Dept Orthopaed Surg, San Francisco, CA 94143 USA.
   [Charlton, Timothy P.] Cedars Sinai Med Ctr, Dept Orthopaed Surg, 444 S San Vicente Blvd, Los Angeles, CA 90048 USA.
RP Charlton, TP (通讯作者)，Cedars Sinai Med Ctr, Dept Orthopaed Surg, 444 S San Vicente Blvd, Los Angeles, CA 90048 USA.
EM timothy.charlton@cshs.org
CR ARNER O, 1959, ACTA CHIR SCAND    S, V239, P1
   Ballas MT, 1998, AM FAM PHYSICIAN, V57, P267
   BESKIN JL, 1987, AM J SPORT MED, V15, P1, DOI 10.1177/036354658701500101
   Chiodo CP, 2010, J BONE JOINT SURG AM, V92A, P2466
   Garras DN, 2012, CLIN ORTHOP RELAT R, V470, P2268, DOI 10.1007/s11999-012-2355-y
   Hodgson RJ, 2014, BRIT J RADIOL, V85, P1016
   KAINBERGER FM, 1990, AM J ROENTGENOL, V155, P1031, DOI 10.2214/ajr.155.5.2120931
   Kayser R, 2005, BRIT J SPORT MED, V39, P838, DOI 10.1136/bjsm.2005.018416
   King J, MULTIRATER RELIABILI
   Kotnis R, 2006, AM J SPORTS MED, V34, P9
   LANDIS JR, 1977, BIOMETRICS, V33, P159, DOI 10.2307/2529310
   LAWRENCE GH, 1955, AM J SURG, V89, P795, DOI 10.1016/0002-9610(55)90146-X
   Maffulli N, 1998, AM J SPORT MED, V26, P266, DOI 10.1177/03635465980260021801
   OBRIEN T, 1984, J BONE JOINT SURG AM, V66A, P1099, DOI 10.2106/00004623-198466070-00018
   Qureshi AA, 2011, J BONE JOINT SURG AM, V93A, P2265, DOI [10.2106/JBJSJ.01757, 10.2106/JBJS.J.01757]
   Ryan ED, 2013, ULTRASOUND MED BIOL, V39, P2488, DOI 10.1016/j.ultrasmedbio.2013.06.013
   SCHELLER AD, 1980, ORTHOP CLIN N AM, V11, P801
   Slane LC, 2014, J BIOMECH, V47, P2831, DOI 10.1016/j.jbiomech.2014.07.032
   Sunding K, 2016, KNEE SURG SPORT TR A, V24, P1988, DOI 10.1007/s00167-014-3270-4
   THOMPSON T C, 1962, J Trauma, V2, P126, DOI 10.1097/00005373-196203000-00003
   Ufberg J, 2004, AM J EMERG MED, V22, P596, DOI 10.1016/j.ajem.2004.09.007
   Weinreb Jeffrey H, 2014, Muscles Ligaments Tendons J, V4, P66
NR 22
TC 8
Z9 9
U1 0
U2 9
PU SAGE PUBLICATIONS INC
PI THOUSAND OAKS
PA 2455 TELLER RD, THOUSAND OAKS, CA 91320 USA
SN 1071-1007
EI 1944-7876
J9 FOOT ANKLE INT
JI Foot Ankle Int.
PD JAN
PY 2017
VL 38
IS 1
BP 36
EP 40
DI 10.1177/1071100716669983
PG 5
WC Orthopedics
WE Science Citation Index Expanded (SCI-EXPANDED)
SC Orthopedics
GA EH5JB
UT WOS:000391808100006
PM 27672015
DA 2022-07-22
ER

PT J
AU Lin, YJ
   Duan, XJ
   Yang, L
AF Lin, Yang-Jing
   Duan, Xiao-Jun
   Yang, Liu
TI V-Y Tendon Plasty for Reconstruction of Chronic Achilles Tendon Rupture:
   A Medium-term and Long-term Follow-up
SO ORTHOPAEDIC SURGERY
LA English
DT Article
DE Achilles tendon; Chronic; Rupture; V-Y tendon plasty
ID BREVIS; REPAIR
AB Objective: To evaluate the surgical results of V-Y tendon plasty in the treatment of chronic Achilles tendon rupture during medium and long-term follow-up.
   Methods: Between June 2005 and September 2017, 20 patients with chronic Achilles tendon rupture underwent V-Y tendon plasty in our hospital. The mean injury-to-surgery time was 20.4weeks (range, 4-96weeks). The Matles test and an improved Thompson test was used to examine Achilles tendon rupture. These patients were not able to stand on the tiptoes of the injured lower extremity. X-ray tests ruled out the chance of fracture and were used to examine the Kager triangle. MRI was used to confirm the final diagnosis. The function of the ankle and the foot was assessed using the American Orthopaedic Foot & Ankle Society (AOFAS) score and the Achilles Tendon Total Rupture Score (ATRS). V-Y tendon plasty for Achilles tendon reconstruction was performed. A below-knee cast was used to keep the ankle in plantar flexion (up to 20 degrees) for 4weeks. Non-weight-bearing exercise was allowed with crutches. After 4weeks, partial weight-bearing was allowed. Physical exercises were performed under rehabilitation guidelines. At 10-12weeks postoperation, patients began to return to daily life activity levels without restrictions. Preoperative and postoperative MRI was obtained.
   Results: The mean follow-up period was 32.8months. The mean operative gap of the Achilles tendon after debridement was 5 cm (range, 4-9 cm), with 85% of the gap less than or equal to 6 cm. The mean AOFAS score increased from 59.25 12.28 preoperatively to 96.55 +/- 3.75 at final follow-up (P < 0.05). The mean ATRS score increased from 39.55 +/- 14.21 preoperatively to 94.05 +/- 4.89 at final follow-up (P < 0.05). All patients had no recurrent Achilles tendon rupture during the follow up. No patient had developed serious complications, such as sural nerve injury or deep vein thrombosis. Patients were able to return to daily life activity levels without restrictions. At the latest follow-up, all patients were able to perform repetitive single heel rise on the involved limb, and to walk without a visible limp. All of the postoperative MRI showed the continuity of the Achilles tendon with no signs of cysts or inflammation, indicating perfect healing at the final follow-up.
   Conclusions: V-Y tendon plasty can be used in most cases of chronic Achilles tendon rupture. It yields satisfactory functional results and low complication rates. The advantage of this procedure is that it is an easy and economic method without the need for expensive synthetic implants. V-Y tendon plasty should be considered an acceptable first-choice treatment.
C1 [Lin, Yang-Jing; Duan, Xiao-Jun; Yang, Liu] Army Med Univ, Third Med Univ 3, Southwest Hosp, Ctr Joint Surg, Chongqing 400038, Peoples R China.
RP Yang, L (通讯作者)，Army Med Univ, Third Med Univ 3, Southwest Hosp, Ctr Joint Surg, Chongqing 400038, Peoples R China.
EM jointsurgery@163.com
CR ABRAHAM E, 1975, J BONE JOINT SURG AM, VA 57, P253, DOI 10.2106/00004623-197557020-00019
   Ahmad Jamal, 2016, Foot Ankle Spec, V9, P400, DOI 10.1177/1938640016640895
   Anaim AA, 2018, FOOT ANKLE SPEC, V11, P514
   Cetti R, 1995, AM J SPORTS MED, V23, P571
   Chang Huan J, 2010, JAMA, V303, P188, DOI 10.1001/jama.303.2.188
   de Cesar Netto C, 2017, FOOT ANKLE SURG, V17, P31364
   Den Hartog BD, 2008, FOOT ANKLE INT, V29, P456, DOI 10.3113/FAI.2008.0456
   Diserens KA, 2015, SCHWEIZ ARCH TIERH, V157, P519, DOI 10.17236/sat00035
   Guclu B, 2016, FOOT ANKLE INT, V37, P737, DOI 10.1177/1071100716642753
   Ibrahim SAR, 2009, J FOOT ANKLE SURG, V48, P340, DOI 10.1053/j.jfas.2009.02.007
   Maffulli N, 1996, BRIT J SPORT MED, V30, P250, DOI 10.1136/bjsm.30.3.250
   Maffulli N, 2008, J BONE JOINT SURG AM, V90A, P1348, DOI 10.2106/JBJS.G.01241
   Maffulli Nicola, 2007, Foot Ankle Clin, V12, P583, DOI 10.1016/j.fcl.2007.07.007
   Maffulli Nicola, 2017, Open Orthop J, V11, P660, DOI 10.2174/1874325001711010660
   Matles A L, 1975, Bull Hosp Joint Dis, V36, P48
   McClelland D, 2004, SURG-J R COLL SURG E, V2, P209, DOI 10.1016/S1479-666X(04)80002-7
   Peterson KS, 2014, J FOOT ANKLE SURG, V53, P664, DOI 10.1053/j.jfas.2013.10.001
   Shoaib A, 2017, FOOT ANKLE SURG, V23, P179, DOI 10.1016/j.fas.2016.04.006
   Takao M, 2003, ARCH ORTHOP TRAUM SU, V123, P471, DOI 10.1007/s00402-002-0443-2
   Us AK, 1997, ARCH ORTHOP TRAUM SU, V116, P408
   Usuelli FG, 2017, JOINTS, V5, P212
   Wegrzyn J, 2010, INT ORTHOP, V34, P1187, DOI 10.1007/s00264-009-0859-1
   Wiegerinck Johannes I, 2012, J Bone Joint Surg Am, V94, pe67, DOI 10.2106/JBJS.K.01074
NR 23
TC 7
Z9 14
U1 0
U2 0
PU WILEY
PI HOBOKEN
PA 111 RIVER ST, HOBOKEN 07030-5774, NJ USA
SN 1757-7853
EI 1757-7861
J9 ORTHOP SURG
JI Orthop. Surg.
PD FEB
PY 2019
VL 11
IS 1
BP 109
EP 116
DI 10.1111/os.12429
PG 8
WC Orthopedics
WE Science Citation Index Expanded (SCI-EXPANDED)
SC Orthopedics
GA HP2ZQ
UT WOS:000461545600015
PM 30809943
OA Green Published, gold
DA 2022-07-22
ER

PT J
AU Maffulli, N
   D'Addona, A
   Maffulli, GD
   Gougoulias, N
   Oliva, F
AF Maffulli, Nicola
   D'Addona, Alessio
   Maffulli, Gayle D.
   Gougoulias, Nikolaos
   Oliva, Francesco
TI Delayed (14-30 Days) Percutaneous Repair of Achilles Tendon Ruptures
   Offers Equally Good Results As Compared With Acute Repair
SO AMERICAN JOURNAL OF SPORTS MEDICINE
LA English
DT Article
DE Achilles tendon; rupture; delayed repair; minimally invasive
ID NONSURGICAL TREATMENT; MANAGEMENT; SURGERY; TEARS; GRAFT
AB Background:
   Minimally invasive repair of acute Achilles tendon ruptures has been performed for several years, resulting in reduced morbidity as compared with open repair.
   Hypothesis:
   A minimally invasive technique can be used to manage Achilles tendon ruptures in patients presenting between 14 and 30 days from injury.
   Study Design:
   Cohort study; Level of evidence, 3.
   Methods:
   We prospectively recruited 21 consecutive patients who presented between 14 and 30 days after the index injury, and we compared them with 21 patients who were matched according to sex, age (+/- 2 years), and level of activity, who presented within 14 days of the index injury. All patients underwent the same minimally invasive procedure under local anesthesia: a core suture repair consisting of a modified Bunnell suture in the proximal stump and a modified Kessler suture in the distal stump.
   Results:
   At 12 months after minimally invasive repair, patients with delayed treatment had a median Achilles tendon rupture score of 91 (SD, 2.4; range, 87-96) as compared with 91 (SD, 2.2; range, 86-96) in patients treated acutely, who presented at a median 2.4 days (range 1-6 days) from the injury. There were no significant differences between groups in terms of mean (SD) Achilles tendon resting angle: delayed repair group, -3.9 degrees (2.0); acute repair group, -3.7 degrees (1.9) (P = .69). No patient in either group developed a wound infection. One patient in the acute group experienced an iatrogenic sural nerve injury.
   Conclusion:
   Patients with Achilles tendon rupture treated by percutaneous repair 14 to 30 days after injury achieved similar results at 1 year as patient treated <14 after injury.
C1 [Maffulli, Nicola; D'Addona, Alessio; Maffulli, Gayle D.; Gougoulias, Nikolaos; Oliva, Francesco] Univ Salerno, Fac Med & Surg, Dept Musculoskeletal Disorders, Via S Allende 24, I-84081 Salerno, Italy.
   [Maffulli, Nicola; Oliva, Francesco] Univ Salerno, Fac Med & Surg, Dept Musculoskeletal Disorders, Baronissi, Italy.
   [Maffulli, Nicola] Barts & London Queen Marys Sch Med & Dent, Ctr Sports & Exercise Med, London, England.
   [Maffulli, Nicola] Keele Univ, Fac Med, Sch Pharm & Bioengn, Newcastle Under Lyme, England.
   [D'Addona, Alessio] Sch Med & Surg Federico II, Dept Publ Hlth, Sect Orthopaed & Trauma Surg, Naples, Italy.
   [Maffulli, Gayle D.] Wholelife Clin, London, England.
   [Gougoulias, Nikolaos] Frimley Hlth NHS Fdn Trust, Frimley Pk Hosp, Frimley, England.
RP Maffulli, N (通讯作者)，Univ Salerno, Fac Med & Surg, Dept Musculoskeletal Disorders, Via S Allende 24, I-84081 Salerno, Italy.
EM n.maffulli@qmul.ac.uk
RI Maffulli, Nicola/A-1839-2014
OI Maffulli, Nicola/0000-0002-5327-3702
CR Anathattee MY, 2017, FOOT ANKLE SURG, V24, P296, DOI DOI 10.1016/J.FAS.2017.02.018
   Carmont MR, 2008, KNEE SURG SPORT TR A, V16, P199, DOI 10.1007/s00167-007-0411-z
   Carmont MR, 2020, KNEE SURG SPORT TR A, V28, P1587, DOI 10.1007/s00167-018-5340-5
   Carmont MR, 2017, ORTHOP J SPORTS MED, V5, DOI 10.1177/2325967117723347
   Carmont MR, 2013, ORTHOP J SPORTS MED, V1, DOI 10.1177/2325967113494584
   Carmont MR, 2015, ASIA-PAC J SPORT MED, V2, P49, DOI 10.1016/j.asmart.2014.12.002
   Craig CL, 2003, MED SCI SPORT EXER, V35, P1381, DOI 10.1249/01.MSS.0000078924.61453.FB
   Del Buono A, 2014, BRIT MED BULL, V109, P45, DOI 10.1093/bmb/ldt029
   Eberling PB, 2016, J BONE JOINT SURG AM, V98, pe8, DOI DOI 10.2106/JBJS.15.01291
   Ecker TM, 2016, AM J SPORT MED, V44, P1004, DOI 10.1177/0363546515623501
   Faul F, 2007, BEHAV RES METHODS, V39, P175, DOI 10.3758/BF03193146
   Froberg A, 2009, AM J SPORT MED, V37, P1200, DOI 10.1177/0363546508330126
   Guzzini M, 2017, J ORTHOP TRAUMATOL, V18, P251, DOI 10.1007/s10195-017-0455-x
   HAGGMARK T, 1986, ORTHOPEDICS, V9, P160
   Halasi T, 2004, AM J SPORT MED, V32, P899, DOI 10.1177/0363546503262181
   HOCKENBURY RT, 1990, FOOT ANKLE, V11, P67, DOI 10.1177/107110079001100202
   Hutchison AM, 2015, BONE JOINT J, V97B, P510, DOI 10.1302/0301-620X.97B4.35314
   INGLIS AE, 1976, J BONE JOINT SURG AM, V58, P990, DOI 10.2106/00004623-197658070-00015
   Julien TP, 2013, FOOT ANKLE INT, V34, P912, DOI 10.1177/1071100713477384
   Lee SJ, 2009, AM J SPORT MED, V37, P786, DOI 10.1177/0363546508328595
   Longo UG, 2012, KNEE SURG SPORT TR A, V20, P1392, DOI 10.1007/s00167-011-1868-3
   Maffulli N, 2005, CLIN J SPORT MED, V15, P56, DOI 10.1097/01.jsm.0000152714.05097.ef
   Maffulli N, 1998, AM J SPORT MED, V26, P266, DOI 10.1177/03635465980260021801
   Maffulli N, 1997, EUR J PEDIATR, V156, P317, DOI 10.1007/s004310050608
   MAFFULLI N, 1991, Acta Orthopaedica Belgica, V57, P154
   Maffulli N, 1992, THESIS
   Maffulli Nicola, 2007, Foot Ankle Clin, V12, P583, DOI 10.1016/j.fcl.2007.07.007
   Maffulli N, 2019, BMJ-BRIT MED J, V364, DOI 10.1136/bmj.k5344
   Maffulli N, 2018, FOOT ANKLE SURG, V24, P164, DOI 10.1016/j.fas.2017.02.002
   Maffulli N, 2013, AM J SPORT MED, V41, P865, DOI 10.1177/0363546512473819
   Movin Tomas, 2005, Foot Ankle Clin, V10, P331, DOI 10.1016/j.fcl.2005.01.003
   Nilsson-Helander K, 2010, AM J SPORT MED, V38, P2186, DOI 10.1177/0363546510376052
   Olsson N, 2013, AM J SPORT MED, V41, P2867, DOI 10.1177/0363546513503282
   Sharma P, 2005, J BONE JOINT SURG AM, V87A, P187, DOI 10.2106/JBJS.D.01850
   TEGNER Y, 1985, CLIN ORTHOP RELAT R, P43
   Wu YH, 2016, INT J SURG, V36, P305, DOI 10.1016/j.ijsu.2016.11.014
   Yang B, 2017, INT J SURG, V40, P178, DOI 10.1016/j.ijsu.2017.03.021
   Zappia M, 2018, FOOT ANKLE SURG, V24, P342, DOI 10.1016/j.fas.2017.03.006
   Zhang YJ, 2018, AM J SPORT MED, V46, P1767, DOI 10.1177/0363546517702872
NR 39
TC 9
Z9 9
U1 0
U2 2
PU SAGE PUBLICATIONS INC
PI THOUSAND OAKS
PA 2455 TELLER RD, THOUSAND OAKS, CA 91320 USA
SN 0363-5465
EI 1552-3365
J9 AM J SPORT MED
JI Am. J. Sports Med.
PD APR
PY 2020
VL 48
IS 5
BP 1181
EP 1188
AR 0363546520908592
DI 10.1177/0363546520908592
EA MAR 2020
PG 8
WC Orthopedics; Sport Sciences
WE Science Citation Index Expanded (SCI-EXPANDED)
SC Orthopedics; Sport Sciences
GA KZ1QM
UT WOS:000523841000001
PM 32176527
DA 2022-07-22
ER

PT J
AU Kessler, MW
   Barr, J
   Greenwald, R
   Lane, LB
   Dines, JS
   Dines, DM
   Drakos, MC
   Grande, DA
   Chahine, NO
AF Kessler, Michael W.
   Barr, Jerome
   Greenwald, Robert
   Lane, Lewis B.
   Dines, Joshua S.
   Dines, David M.
   Drakos, Mark C.
   Grande, Daniel A.
   Chahine, Nadeen O.
TI Enhancement of Achilles Tendon Repair Mediated by Matrix
   Metalloproteinase Inhibition via Systemic Administration of Doxycycline
SO JOURNAL OF ORTHOPAEDIC RESEARCH
LA English
DT Article
DE achilles tendon; matrix metalloproteinase; doxycycline; tendon rupture
   and repair
ID TISSUE INHIBITOR; GENE-EXPRESSION; TENDINOPATHY; GROWTH; SUTURES;
   RUPTURE; DISEASE; CELLS; MODEL; MMP-9
AB Collagenases or matrix metalloproteinases (MMPs) have been shown to play an important role in the matrix degradation cascade associated with Achilles tendon rupture and disease. The goal of this study was to examine the effects of daily administration of doxycycline (Doxy) through oral gavage on MMP activity and on the repair quality of Achilles tendons in vivo. Our findings indicate that Achilles tendon transection resulted in increasing MMP-8 activity from 2 to 6 weeks post-injury, with peak increases in activity occurring at 4 weeks post-injury. Doxy adiministration at clinically relevant serum concentrations was found to significantly inhibit MMP activity after continuous treatment for 4 weeks, but not for continuous administration for shorter durations (96h or 2 weeks). Extended doxy administration was also associated with improved collagen fibril organization, and enhanced biomechanical properties (stiffness, ultimate tensile strength, maximum load to failure, and elastic toughness). Our findings indicate that a temporal delay exists between Achilles tendon transection and associated increases in MMP-8 activity in situ. Our findings suggest that inhibition of MMP-8 at its peak activity levels ameliorates fibrosis development and improves biomechanical properties of the Achilles tendon. (c) 2013 Orthopaedic Research Society. Published by Wiley Periodicals, Inc. J Orthop Res 32:500-506, 2014.
C1 [Kessler, Michael W.; Barr, Jerome; Grande, Daniel A.] Feinstein Inst Med Res, Orthopaed Res Lab, Manhasset, NY USA.
   [Kessler, Michael W.] Georgetown Univ Hosp, Washington, DC 20007 USA.
   [Greenwald, Robert; Lane, Lewis B.; Dines, Joshua S.; Dines, David M.; Drakos, Mark C.] North Shore LIJ Hlth Syst, New Hyde Pk, NY USA.
   [Chahine, Nadeen O.] Feinstein Inst Med Res, Biomech & Bioengn Res Lab, Manhasset, NY 11030 USA.
   [Lane, Lewis B.; Grande, Daniel A.; Chahine, Nadeen O.] Hofstra North Shore LIJ Sch Med, Hempstead, NY USA.
RP Chahine, NO (通讯作者)，Feinstein Inst Med Res, Biomech & Bioengn Res Lab, Manhasset, NY 11030 USA.
EM dgrande@nshs.edu; nchahine@nshs.edu
RI Chahine, Nadeen/AAF-9975-2019; Chahine, Nadeen/O-5496-2015
OI Chahine, Nadeen/0000-0002-0478-6042; Dines, David/0000-0003-0605-5312
FU Department of Orthopedic Surgery; Feinstein Institute for Medical
   Research, North Shore LIJ Health System; American Orthopaedic Society
   for Sports Medicine
FX Grant sponsor: Department of Orthopedic Surgery; Grant sponsor: The
   Feinstein Institute for Medical Research, North Shore LIJ Health System;
   Grant sponsor: American Orthopaedic Society for Sports Medicine.
CR Bedi A, 2010, J SHOULDER ELB SURG, V19, P384, DOI 10.1016/j.jse.2009.07.010
   Bedi A, 2010, AM J SPORT MED, V38, P308, DOI 10.1177/0363546509347366
   DALTON S, 1995, ANN RHEUM DIS, V54, P571, DOI 10.1136/ard.54.7.571
   Del Buono A, 2012, J SHOULDER ELB SURG, V21, P200, DOI 10.1016/j.jse.2011.10.020
   Dines JS, 2007, J SHOULDER ELB SURG, V16, p215S, DOI 10.1016/j.jse.2007.03.001
   Dong M, 2012, PLOS ONE, V7, DOI 10.1371/journal.pone.0039695
   Gardner K, 2008, DISABIL REHABIL, V30, P1523, DOI 10.1080/09638280701785395
   Garofalo R, 2011, SPORTS MED ARTHROSC, V19, P207, DOI 10.1097/JSA.0b013e318227b07b
   Golub L M, 1998, Adv Dent Res, V12, P12
   Gotoh M, 2013, KNEE SURG SPORT TR A, V21, P1807, DOI 10.1007/s00167-012-2209-x
   Griffin MO, 2011, PHARMACOL RES, V63, P102, DOI 10.1016/j.phrs.2010.10.004
   Ireland D, 2001, MATRIX BIOL, V20, P159, DOI 10.1016/S0945-053X(01)00128-7
   Jacob J, 2012, ORTHOPEDICS, V35, pE474, DOI 10.3928/01477447-20120327-18
   Jones GC, 2006, ARTHRITIS RHEUM-US, V54, P832, DOI 10.1002/art.21672
   Karousou E, 2008, CLIN ORTHOP RELAT R, V466, P1577, DOI 10.1007/s11999-008-0255-y
   Kujala UM, 2005, CLIN J SPORT MED, V15, P133, DOI 10.1097/01.jsm.0000165347.55638.23
   Lo IKY, 2004, AM J SPORT MED, V32, P1223, DOI 10.1177/0363546503262200
   Maffulli N, 2011, FOOT ANKLE INT, V32, P9, DOI 10.3113/FAI.2011.0009
   Magra M, 2005, BRIT J SPORT MED, V39, P789, DOI 10.1136/bjsm.2005.017855
   Pasternak B, 2010, BRIT J SPORT MED, V44, P669, DOI 10.1136/bjsm.2008.049411
   Pasternak B, 2007, ACTA ORTHOP, V78, P680, DOI 10.1080/17453670710014392
   Pasternak Bjorn, 2006, Acta Orthop Belg, V72, P756
   Prall AK, 2002, J VASC SURG, V35, P923, DOI 10.1067/mva.2002.123757
   Raffetto JD, 2008, BIOCHEM PHARMACOL, V75, P346, DOI 10.1016/j.bcp.2007.07.004
   Riley GP, 2005, SCAND J MED SCI SPOR, V15, P241, DOI 10.1111/j.1600-0838.2005.00456.x
   Riley GP, 2002, MATRIX BIOL, V21, P185, DOI 10.1016/S0945-053X(01)00196-2
   Shah V, 2013, J ORTHOP RES, V31, P413, DOI 10.1002/jor.22222
   Soslowsky L J, 1996, J Shoulder Elbow Surg, V5, P383
   Tsuzaki M, 2003, J ORTHOP RES, V21, P256, DOI 10.1016/S0736-0266(02)00141-9
   Zimowska M, 2012, TISSUE ENG PT A, V18, P1183, DOI [10.1089/ten.tea.2011.0459, 10.1089/ten.TEA.2011.0459]
NR 30
TC 26
Z9 27
U1 0
U2 13
PU WILEY
PI HOBOKEN
PA 111 RIVER ST, HOBOKEN 07030-5774, NJ USA
SN 0736-0266
EI 1554-527X
J9 J ORTHOP RES
JI J. Orthop. Res.
PD APR
PY 2014
VL 32
IS 4
BP 500
EP 506
DI 10.1002/jor.22564
PG 7
WC Orthopedics
WE Science Citation Index Expanded (SCI-EXPANDED)
SC Orthopedics
GA AA0UA
UT WOS:000330811700003
PM 24346815
DA 2022-07-22
ER

PT J
AU Guclu, B
   Basat, HC
   Yildirim, T
   Bozduman, O
   Us, AK
AF Guclu, Berk
   Basat, H. Cagdas
   Yildirim, Tugrul
   Bozduman, Omer
   Us, Ali Kemal
TI Long-term Results of Chronic Achilles Tendon Ruptures Repaired With V-Y
   Tendon Plasty and Fascia Turndown
SO FOOT & ANKLE INTERNATIONAL
LA English
DT Article
DE chronic Achilles tendon tear; Achilles tendon reconstruction; neglected
   rupture
ID NEGLECTED TEARS; MANAGEMENT; DISORDERS
AB Background: This study aimed to evaluate the long-term follow-up results of V-Y tendon plasty with fascia turndown, for repairing chronic Achilles tendon ruptures.
   Methods: Seventeen patients (12 males, 5 females), who were diagnosed with chronic Achilles tendon rupture and met the inclusion criteria, were included in the study. These patients received treatment by means of V-Y tendon plasty with fascia turndown from January 1995 to December 2001. Clinical outcomes of the patients were assessed by using isokinetic strength testing, questioning the patient regarding residual discomfort, pain, or swelling and having the ability to perform heel rises and using American Orthopaedic Foot & Ankle Society's (AOFAS's) Ankle-Hind Foot Scale score. Mean follow-up duration was 16 years (13-18 years).
   Results: Mean time from the injury to operative treatment was 7 months. Mean operative defect of Achilles tendon in neutral position after debridement was 6 cm. During the follow-up, the mean calf atrophy was 3.4 cm. The mean 30 degrees/s plantarflex and 120 degrees/s plantarflex peak torques were 89 and 45 Nm, respectively. The mean 30 degrees/s plantarflex peak torque deficiency was 16%. The mean 120 degrees/s plantarflex peak torque deficiency was 17%. The average peak torque deficiency was 17%. The pre- and postoperative mean AOFAS Ankle-Hindfoot Scale scores were 64 and 95, respectively. No patient had a rerupture. Superficial wound infection was treated with oral antibiotic therapy in 2 patients (11%).
   Conclusions: The V-Y tendon plasty with fascia turndown for repairing chronic Achilles tendon ruptures yielded results comparable with the literature regarding clinical outcomes. This method did not require synthetic materials for augmentation and was an economic alternative compared to other repair methods.
   Level of Evidence: Level III, retrospective comparative study.
C1 [Guclu, Berk; Bozduman, Omer] Ufuk Univ, Dept Orthoped Surg, Fac Med, Ankara, Turkey.
   [Basat, H. Cagdas] Koru Hosp, Dept Orthoped Surg, Ankara, Turkey.
   [Yildirim, Tugrul] Kazan Hamdi Eris State Hosp, Dept Orthoped Surg, Ankara, Turkey.
   [Us, Ali Kemal] Ankara Univ, Dept Orthoped Surg, Fac Med, Ankara, Turkey.
RP Yildirim, T (通讯作者)，Kazan Hamdi Eris State Hosp, Dept Orthoped Surg, Ankara, Turkey.
EM mdtugrul@hotmail.com
RI Yildirim, Tugrul/AAN-5809-2020; US, Ali Kemal/AAJ-3320-2020
OI US, Ali Kemal/0000-0003-4835-2294; Yildirim, Tugrul/0000-0002-2316-0766
CR ABRAHAM E, 1975, J BONE JOINT SURG AM, VA 57, P253, DOI 10.2106/00004623-197557020-00019
   Badalihan A, 2015, J FOOT ANKLE SURG, V54, P153, DOI 10.1053/j.jfas.2014.12.002
   Basiglini L, 2010, KNEE SURG SPORT TR A, V18, P644, DOI 10.1007/s00167-010-1085-5
   Besse J L, 1999, J Foot Ankle Surg, V38, P70
   CARDEN DG, 1987, J BONE JOINT SURG BR, V69, P416, DOI 10.1302/0301-620X.69B3.3294839
   Choksey A, 1996, INJURY, V27, P215, DOI 10.1016/0020-1383(95)00214-6
   CHRISTENSEN RE, 1931, DANSK KIR SELSK FORH, V75, P39
   Coull R, 2003, FOOT ANKLE INT, V24, P931, DOI 10.1177/107110070302401211
   Den Hartog BD, 2008, FOOT ANKLE INT, V29, P456, DOI 10.3113/FAI.2008.0456
   Doral MN, 2009, ARCH ORTHOP TRAUM SU, V129, P1093, DOI 10.1007/s00402-009-0880-2
   Elias I, 2007, FOOT ANKLE INT, V28, P1238, DOI 10.3113/FAI.2007.1238
   Hoffmann A, 2011, EUR RADIOL, V21, P1996, DOI 10.1007/s00330-011-2136-0
   Hutchison AM, 2015, BONE JOINT J, V97B, P510, DOI 10.1302/0301-620X.97B4.35314
   Kosanovic M, 2008, ARCH ORTHOP TRAUM SU, V128, P211, DOI 10.1007/s00402-007-0514-5
   Lee KB, 2009, KNEE SURG SPORT TR A, V17, P316, DOI 10.1007/s00167-008-0693-9
   LEITNER A, 1992, FOOT ANKLE, V13, P176, DOI 10.1177/107110079201300402
   Leslie Hamish D H, 2005, Foot Ankle Clin, V10, P357, DOI 10.1016/j.fcl.2005.01.009
   LINDHOLM A, 1959, Acta Chir Scand, V117, P261
   Maffulli N, 2005, CLIN J SPORT MED, V15, P56, DOI 10.1097/01.jsm.0000152714.05097.ef
   Maffulli N, 2015, CLIN SPORT MED, V34, P607, DOI 10.1016/j.csm.2015.06.010
   Mulier T, 2003, FOOT ANKLE SURG, V9, P151, DOI DOI 10.1016/S1268-7731(03)00048-1
   Pintore E, 2001, J TRAUMA, V50, P71, DOI 10.1097/00005373-200101000-00013
   Rahm S, 2013, FOOT ANKLE INT, V34, P1100, DOI 10.1177/1071100713487725
   SILFVERSKIOLD N, 1941, ACTA CHIR SCAND, V84, P393
   Us AK, 1997, ARCH ORTHOP TRAUM SU, V116, P408
   Wallace RGH, 2011, J BONE JOINT SURG BR, V93B, P1362, DOI 10.1302/0301-620X.93B10.26187
   Wapner K L, 1993, Foot Ankle, V14, P443
   Wilcox DK, 2000, FOOT ANKLE INT, V21, P1004, DOI 10.1177/107110070002101204
   Wong J, 2002, AM J SPORT MED, V30, P565, DOI 10.1177/03635465020300041701
   Yeoman Thomas F M, 2012, Foot (Edinb), V22, P219, DOI 10.1016/j.foot.2012.05.004
NR 30
TC 25
Z9 31
U1 0
U2 7
PU SAGE PUBLICATIONS INC
PI THOUSAND OAKS
PA 2455 TELLER RD, THOUSAND OAKS, CA 91320 USA
SN 1071-1007
EI 1944-7876
J9 FOOT ANKLE INT
JI Foot Ankle Int.
PD JUL
PY 2016
VL 37
IS 7
BP 737
EP 742
DI 10.1177/1071100716642753
PG 6
WC Orthopedics
WE Science Citation Index Expanded (SCI-EXPANDED)
SC Orthopedics
GA DS0XZ
UT WOS:000380321400008
PM 27036138
DA 2022-07-22
ER

PT J
AU Park, YH
   Lim, JW
   Choi, GW
   Kim, HJ
AF Park, Young Hwan
   Lim, Jung Wook
   Choi, Gi Won
   Kim, Hak Jun
TI Quantitative Magnetic Resonance Imaging Analysis of the Common Site of
   Acute Achilles Tendon Rupture: 5 to 8 cm Above the Distal End of the
   Calcaneal Insertion
SO AMERICAN JOURNAL OF SPORTS MEDICINE
LA English
DT Article
DE Achilles tendon; tendon injuries; calcaneus; magnetic resonance imaging
ID ETHNIC-DIFFERENCES; BLOOD-FLOW; AGREEMENT
AB Background: Previous studies have not quantitatively analyzed the site of acute Achilles tendon rupture. Purpose: To investigate the site of acute Achilles tendon rupture via magnetic resonance imaging (MRI). Study Design: Cross-sectional study; Level of evidence, 4. Methods: The medical records and MRIs of 195 patients with acute Achilles tendon rupture were retrospectively reviewed. The rupture site was measured as the distance from the most distal fibers of the Achilles tendon at the calcaneal insertion to the proximal end of the distal stump. The relationship between the site of rupture and patient characteristics was analyzed, and the intra- and interobserver reliability of the measurements was assessed using intraclass correlation coefficients. Results: The rupture site had a mean distance of 6.4 cm (SD, 1.5) and the 10th to 90th percentile range was 5.0 to 8.4 cm. There was no significant difference in location of the rupture according to patient characteristics and the time of imaging. The intra- and interobserver reliability of measurements was excellent. Conclusion: Most acute Achilles tendon ruptures visible on MRI occurred 5 to 8 cm above the distal end of the calcaneal insertion, which is more proximal than the previously reported 2 to 6 cm location.
C1 Korea Univ, Med Ctr, Guro & Ansan Hosp, Seoul, South Korea.
   [Park, Young Hwan; Lim, Jung Wook; Kim, Hak Jun] Korea Univ, Guro Hosp, Dept Orthopaed Surg, 148 Gurodong Ro, Seoul 08308, South Korea.
   [Choi, Gi Won] Korea Univ, Ansan Hosp, Dept Orthopaed Surg, Ansan, South Korea.
RP Kim, HJ (通讯作者)，Korea Univ, Guro Hosp, Dept Orthopaed Surg, 148 Gurodong Ro, Seoul 08308, South Korea.
EM hjunkimos@gmail.com
RI Choi, Gi Won/AAX-7187-2020
OI Kim, Hak Jun/0000-0003-3633-6174
CR Ahmed IM, 1998, J ORTHOPAED RES, V16, P591, DOI 10.1002/jor.1100160511
   ASTROM M, 1994, J ORTHOP RES, V12, P246, DOI 10.1002/jor.1100120214
   Astrom M, 2000, SCAND J MED SCI SPOR, V10, P365, DOI 10.1034/j.1600-0838.2000.010006365.x
   Ballal MS, 2014, BONE JOINT J, V96B, P1344, DOI 10.1302/0301-620X.96B10.33771
   Barfod KW, 2015, KNEE SURG SPORT TR A, V23, P3398, DOI 10.1007/s00167-014-3175-2
   CAMPBELL P, 1993, BRIT J HOSP MED, V50, P321
   CARR AJ, 1989, J BONE JOINT SURG BR, V71, P100, DOI 10.1302/0301-620X.71B1.2914976
   de Vet HCW, 2006, J CLIN EPIDEMIOL, V59, P1033, DOI 10.1016/j.jclinepi.2005.10.015
   Edama M, 2016, J ANAT, V229, P610, DOI 10.1111/joa.12514
   Ganestam A, 2016, KNEE SURG SPORT TR A, V24, P3730, DOI 10.1007/s00167-015-3544-5
   Halasi T, 2004, AM J SPORT MED, V32, P899, DOI 10.1177/0363546503262181
   Hess Gregory William, 2010, Foot Ankle Spec, V3, P29, DOI 10.1177/1938640009355191
   Ho G, 2017, INJURY, V48, P1701, DOI 10.1016/j.injury.2017.04.007
   Kim PJ, 2011, J FOOT ANKLE SURG, V50, P41, DOI 10.1053/j.jfas.2010.10.007
   LAGERGREN C, 1959, Acta Chir Scand, V116, P491
   LAINE V A, 1955, Acta Orthop Scand, V24, P250
   Lamm BM, 2016, J FOOT ANKLE SURG, V55, P991, DOI 10.1053/j.jfas.2016.05.005
   LANDIS JR, 1977, BIOMETRICS, V33, P159, DOI 10.2307/2529310
   Langberg H, 1998, ACTA PHYSIOL SCAND, V163, P149, DOI 10.1046/j.1365-201X.1998.00361.x
   Lantto I, 2015, SCAND J MED SCI SPOR, V25, pE133, DOI 10.1111/sms.12253
   Lohrer H, 2008, CLIN ORTHOP RELAT R, V466, P2230, DOI 10.1007/s11999-008-0298-0
   McCarthy JP, 2006, J STRENGTH COND RES, V20, P511
   Nyyssonen T, 2008, SCAND J SURG, V97, P272, DOI 10.1177/145749690809700312
   Raikin SM, 2013, FOOT ANKLE INT, V34, P475, DOI 10.1177/1071100713477621
   REINHERZ R P, 1991, Journal of Foot Surgery, V30, P117
   Rosso C, 2012, CLIN J SPORT MED, V22, P483, DOI 10.1097/JSM.0b013e3182639a3e
   SCHMIDTROHLFING B, 1992, INT ORTHOP, V16, P29
   Sheth U, 2017, BONE JOINT J, V99B, P78, DOI [10.1302/0301-620X.99B1.BJJ-2016-0434.R1, 10.1302/0301-620x.99b1.bjj-2016-0434.r1]
   Stein V, 2000, ACTA ORTHOP SCAND, V71, P60, DOI 10.1080/00016470052943919
   Theobald P, 2005, INJURY, V36, P1267, DOI 10.1016/j.injury.2005.02.012
   Young SW, 2014, J BONE JOINT SURG AM, V96A, P1073, DOI 10.2106/JBJS.M.00248
   Zantop T, 2003, ARCH ORTHOP TRAUM SU, V123, P501, DOI 10.1007/s00402-003-0491-2
   Zengin A, 2016, BONE, V91, P180, DOI 10.1016/j.bone.2016.07.018
NR 33
TC 6
Z9 7
U1 0
U2 2
PU SAGE PUBLICATIONS INC
PI THOUSAND OAKS
PA 2455 TELLER RD, THOUSAND OAKS, CA 91320 USA
SN 0363-5465
EI 1552-3365
J9 AM J SPORT MED
JI Am. J. Sports Med.
PD AUG
PY 2019
VL 47
IS 10
BP 2374
EP 2379
DI 10.1177/0363546519858990
PG 6
WC Orthopedics; Sport Sciences
WE Science Citation Index Expanded (SCI-EXPANDED)
SC Orthopedics; Sport Sciences
GA IN4QK
UT WOS:000478659700014
PM 31287711
DA 2022-07-22
ER

PT J
AU Gibbon, WW
   Cooper, JR
   Radcliffe, GS
AF Gibbon, WW
   Cooper, JR
   Radcliffe, GS
TI Distribution of sonographically detected tendon abnormalities in
   patients with a clinical diagnosis of chronic Achilles tendinosis
SO JOURNAL OF CLINICAL ULTRASOUND
LA English
DT Article
DE ultrasonography; tendon injury; tendonitis; Achilles tendon
ID ULTRASONOGRAPHY; INJURY; US; TENDINITIS; RUPTURES
AB Purpose. We conducted a retrospective study of the distribution of sonographically detected abnormalities in the heels of patients who had a clinical diagnosis of Achilles tendinosis.
   Methods. One hundred eighteen symptomatic heels in 73 patients who had a clinical diagnosis of chronic Achilles tendinosis were examined over a 12-month period by the same experienced sonologist. The distribution of altered tendon architecture and features suggesting retrocalcaneal bursitis or Achilles paratendinosis were evaluated.
   Results. Sonograms of 118 symptomatic heels demonstrated that 96 (81%) had abnormalities confined to the proximal two thirds of the Achilles tendon, 9 (8%) had abnormalities in the distal third alone, and 13 (11%) had abnormalities at both sites. Of the 109 heels with proximal two-third Achilles tendon disease, 99 (91%) had medial tendon involvement; 22 of the 99 showed diffuse tendon changes. Lateral tendon segment changes were seen in 22 (19%) of the 118 symptomatic heels. No lateral tendon segment was involved in isolation. Of the 22 heels with distal third abnormalities, 14 (64%) had sonographic evidence of Achilles paratendinitis, and 13 (59%) had sonographic evidence of Achilles tendinosis. Eighteen of the 22 had sonographic evidence of retrocalcaneal bursitis. In all cases of distal third tendinosis, the deep surface of the tendon was primarily involved. In the heels with both proximal and distal changes, superficial segment involvement of the mid-Achilles tendon was present.
   Conclusions. Sonography provides information that helps to accurately diagnose clinical Achilles tendinopathy and may help to determine the biomechanical processes involved in the injury. (C) 2000 John Wiley & Sons, Inc.
C1 Gen Infirm, Dept Radiol, Leeds LS 3EX, W Yorkshire, England.
   Gen Infirm, Dept Orthopaed, Leeds LS 3EX, W Yorkshire, England.
RP Cooper, JR (通讯作者)，No Gen Hosp, Dept Diagnost Imaging, Herries Rd, Sheffield S5 7AU, S Yorkshire, England.
CR Astrom M, 1996, SKELETAL RADIOL, V25, P615, DOI 10.1007/s002560050146
   BARFRED T, 1973, ACTA ORTHOP SCAND S, V152, P1124
   BERTOLOTTO M, 1995, BRIT J RADIOL, V68, P986, DOI 10.1259/0007-1285-68-813-986
   Blazina M E, 1973, Orthop Clin North Am, V4, P665
   BLEI CL, 1986, RADIOLOGY, V159, P765, DOI 10.1148/radiology.159.3.3517960
   CLEMENT DB, 1984, AM J SPORT MED, V12, P179, DOI 10.1177/036354658401200301
   DILLEHAY GL, 1984, INVEST RADIOL, V19, P338, DOI 10.1097/00004424-198407000-00020
   FORNAGE BD, 1986, RADIOLOGY, V159, P759, DOI 10.1148/radiology.159.3.3517959
   FORNAGE BD, 1987, J ULTRAS MED, V6, P19
   FORNAGE BD, 1988, RADIOL CLIN N AM, V26, P87
   FUKASHIRO S, 1995, EUR J APPL PHYSIOL O, V71, P453, DOI 10.1007/BF00635880
   Gibbon WW, 1999, BRIT J SPORT MED, V33, P129, DOI 10.1136/bjsm.33.2.129
   GIBBON WW, 1998, BRIT J SPORT MED, V32, P266
   GROSS MT, 1992, J ORTHOP SPORT PHYS, V16, P248, DOI 10.2519/jospt.1992.16.6.248
   HANLON D P, 1992, Journal of Emergency Medicine, V10, P559, DOI 10.1016/0736-4679(92)90136-H
   INGLIS AE, 1976, J BONE JOINT SURG AM, V58, P990, DOI 10.2106/00004623-197658070-00015
   KAINBERGER FM, 1990, AM J ROENTGENOL, V155, P1031, DOI 10.2214/ajr.155.5.2120931
   KALEBO P, 1990, ACTA RADIOL, V31, P565, DOI 10.3109/02841859009173098
   KOMI PV, 1992, CLIN SPORT MED, V11, P521
   KVIST M, 1994, SPORTS MED, V18, P173, DOI 10.2165/00007256-199418030-00004
   LAINE HR, 1987, J ULTRAS MED, V6, P351, DOI 10.7863/jum.1987.6.7.351
   LEADBETTER WB, 1992, CLIN SPORT MED, V11, P533
   Ljungqvist R., 1968, ACTA ORTHOP SCAND S, V39, P1, DOI 10.3109/ort.1968.39.suppl-113.01
   Maffulli N, 1987, Br J Sports Med, V21, P158
   MAFFULLI N, 1989, J SPORT MED PHYS FIT, V29, P365
   MARTINOLI C, 1993, RADIOLOGY, V186, P839, DOI 10.1148/radiology.186.3.8430196
   MATHIESON JR, 1988, AM J ROENTGENOL, V151, P127
   PAVLOV H, 1982, RADIOLOGY, V144, P83, DOI 10.1148/radiology.144.1.7089270
   Puddu G, 1976, Am J Sports Med, V4, P145, DOI 10.1177/036354657600400404
   QUINN SF, 1987, RADIOLOGY, V164, P767, DOI 10.1148/radiology.164.3.3615878
   SELLA J, 1998, J FOOT ANKLE SURG, V37, P110
   SMART GW, 1980, MED SCI SPORT EXER, V12, P231
   WILLIAMS JGP, 1986, SPORTS MED, V3, P114, DOI 10.2165/00007256-198603020-00003
NR 33
TC 67
Z9 68
U1 0
U2 7
PU WILEY
PI HOBOKEN
PA 111 RIVER ST, HOBOKEN 07030-5774, NJ USA
SN 0091-2751
EI 1097-0096
J9 J CLIN ULTRASOUND
JI J. Clin. Ultrasound
PD FEB
PY 2000
VL 28
IS 2
BP 61
EP 66
DI 10.1002/(SICI)1097-0096(200002)28:2<61::AID-JCU1>3.0.CO;2-R
PG 6
WC Acoustics; Radiology, Nuclear Medicine & Medical Imaging
WE Science Citation Index Expanded (SCI-EXPANDED)
SC Acoustics; Radiology, Nuclear Medicine & Medical Imaging
GA 279VK
UT WOS:000085065600001
PM 10641001
DA 2022-07-22
ER

PT J
AU Vosseller, JT
   Ellis, SJ
   Levine, DS
   Kennedy, JG
   Elliott, AJ
   Deland, JT
   Roberts, MM
   O'Malley, MJ
AF Vosseller, J. Turner
   Ellis, Scott J.
   Levine, David S.
   Kennedy, John G.
   Elliott, Andrew J.
   Deland, Jonathan T.
   Roberts, Matthew M.
   O'Malley, Martin J.
TI Achilles Tendon Rupture in Women
SO FOOT & ANKLE INTERNATIONAL
LA English
DT Article
DE Achilles tendon; rupture; women; male; female ratio
ID ANTERIOR CRUCIATE LIGAMENT; MENSTRUAL-CYCLE; MECHANICAL-PROPERTIES;
   INCREASING INCIDENCE; IN-VIVO; ESTROGEN; INJURIES; PROGESTERONE;
   EPIDEMIOLOGY
AB Background: The incidence of Achilles rupture appears to be less in women, although this notion has not been specifically investigated in the literature.
   Methods: The medical records of 7 foot-and-ankle orthopaedic surgeons at 1 institution were reviewed by Current Procedural Terminology (code 27650) and International Classification of Diseases-9 (code 727.67) to establish all Achilles tendon ruptures seen and/or treated by these surgeons. Sex, age, side, and mechanism of injury were recorded. Whether the patient had an acute Achilles tendon rupture or nonacute Achilles pathology was also noted.
   Results: A total of 468 patients were identified, of whom 358 had acute ruptures: 302 male and 56 female (5.39:1). Patients with acute ruptures were significantly younger than those with nonacute pathology (43.8 vs 55.1, P < .001). For acute ruptures, the mean age was not significantly different between men and women (43.9 vs 43.2; P = .780). Athletic activity was causative in 243 of 302 men (80.5%) and in 40 of 56 women (71.4%). This difference was not statistically significant (P = .130). Six men (2.0%) and 6 women (10.7%) had comorbidities that were thought to increase their risk of rupture (P = .005).
   Conclusion: Achilles tendon rupture is more common in men than women. Previous studies using the aforementioned codes to identify patients without chart review may have overestimated the number of women with acute Achilles tendon rupture.
   Level of Evidence: Level III, retrospective comparative series.
C1 [Vosseller, J. Turner; Ellis, Scott J.; Levine, David S.; Kennedy, John G.; Elliott, Andrew J.; Deland, Jonathan T.; Roberts, Matthew M.; O'Malley, Martin J.] Hosp Special Surg, New York, NY 10021 USA.
RP Vosseller, JT (通讯作者)，New York Presbyterian Columbia, 622 W 168th St,PH 11, New York, NY 10032 USA.
EM turner.vosseller@gmail.com
RI Vosseller, J. Turner/S-2479-2019
OI Vosseller, J. Turner/0000-0002-9639-3739
CR Bridgeman JT, 2010, FOOT ANKLE INT, V31, P1081, DOI 10.3113/FAI.2010.1081
   Bryant AL, 2008, J APPL PHYSIOL, V105, P1035, DOI 10.1152/japplphysiol.01281.2007
   Burgess KE, 2009, CLIN BIOMECH, V24, P504, DOI 10.1016/j.clinbiomech.2009.03.011
   Burgess KE, 2010, J STRENGTH COND RES, V24, P2088, DOI 10.1519/JSC.0b013e3181aeb12b
   Clayton RAE, 2008, INJURY, V39, P1338, DOI 10.1016/j.injury.2008.06.021
   Davis JJ, 1999, MIL MED, V164, P872, DOI 10.1093/milmed/164.12.872
   Houshian S, 1998, INJURY, V29, P651, DOI 10.1016/S0020-1383(98)00147-8
   JONES BH, 1993, AM J SPORT MED, V21, P705, DOI 10.1177/036354659302100512
   JOZSA L, 1989, AM J SPORT MED, V17, P338, DOI 10.1177/036354658901700305
   KOBORI M, 1989, CLIN ORTHOP RELAT R, P306
   Leppilahti J, 1996, ACTA ORTHOP SCAND, V67, P277, DOI 10.3109/17453679608994688
   Levi N, 1997, INJURY, V28, P311, DOI 10.1016/S0020-1383(96)00200-8
   Liu SH, 1996, J ORTHOPAED RES, V14, P526, DOI 10.1002/jor.1100140405
   Maffulli N, 2002, MED SCI SPORT EXER, V34, P1057, DOI 10.1097/00005768-200207000-00001
   Maffulli N, 2000, AM J SPORT MED, V28, P857, DOI 10.1177/03635465000280061401
   Maffulli N, 1999, CLIN J SPORT MED, V9, P157, DOI 10.1097/00042752-199907000-00007
   Maffulli N, 2011, KNEE SURG SPORT TR A, V19, P680, DOI 10.1007/s00167-010-1193-2
   Moller A, 1996, ACTA ORTHOP SCAND, V67, P479, DOI 10.3109/17453679608996672
   NILLIUS SA, 1976, ACTA ORTHOP SCAND, V47, P118, DOI 10.3109/17453677608998983
   Nyyssonen T, 2008, SCAND J SURG, V97, P272, DOI 10.1177/145749690809700312
   Suchak AA, 2005, FOOT ANKLE INT, V26, P932, DOI 10.1177/107110070502601106
   Tallon C, 2001, MED SCI SPORT EXER, V33, P1983, DOI 10.1097/00005768-200112000-00002
   Wentorf FA, 2006, AM J SPORT MED, V34, P1948, DOI 10.1177/0363546506290060
   Wojtys EM, 2002, AM J SPORT MED, V30, P182, DOI 10.1177/03635465020300020601
   Wojtys EM, 1998, AM J SPORT MED, V26, P614, DOI 10.1177/03635465980260050301
   Yu WD, 2001, CLIN ORTHOP RELAT R, P268, DOI 10.1097/00003086-200102000-00031
NR 26
TC 39
Z9 41
U1 1
U2 15
PU SAGE PUBLICATIONS INC
PI THOUSAND OAKS
PA 2455 TELLER RD, THOUSAND OAKS, CA 91320 USA
SN 1071-1007
EI 1944-7876
J9 FOOT ANKLE INT
JI Foot Ankle Int.
PD JAN
PY 2013
VL 34
IS 1
BP 49
EP 53
DI 10.1177/1071100712460223
PG 5
WC Orthopedics
WE Science Citation Index Expanded (SCI-EXPANDED)
SC Orthopedics
GA 080WK
UT WOS:000314274600007
PM 23386761
DA 2022-07-22
ER

PT J
AU Zell, RA
   Santoro, VM
AF Zell, RA
   Santoro, VM
TI Augmented repair of acute Achilles tendon ruptures
SO FOOT & ANKLE INTERNATIONAL
LA English
DT Article
DE Achilles tendon rupture; augmented repair
AB Twenty-five patients who had an acute Achilles tendon rupture were managed with an augmented repair using the gastrocnemius-soleus fascia, All patients healed their repair and there were no re-ruptures. There was one infection, Augmented repair allowed early functional recovery as evidenced by full ankle motion by four to eight weeks, full unassisted weight bearing by three weeks, cessation of braces by four weeks, and return to work by one to six weeks post-operatively. Augmentation adds a sufficient amount of collagen to allow early range of motion and weight bearing without re-rupture. Disadvantages included a long incision, soft tissue prominence, one infection, and sural nerve injury.
C1 Univ Connecticut, Hartford, CT 06106 USA.
   Hartford Hosp, Hartford, CT 06106 USA.
RP Santoro, VM (通讯作者)，Univ Connecticut, 85 Seymour St,Suite 607, Hartford, CT 06106 USA.
CR ABRAHAMS M, 1967, MED BIOL ENG, V5, P433, DOI 10.1007/BF02479137
   ARNER O, 1959, Acta Chir Scand, V116, P484
   BARFRED T, 1971, ACTA ORTHOP SCAND, V42, P406, DOI 10.3109/17453677108989062
   BESKIN JL, 1987, AM J SPORT MED, V15, P1, DOI 10.1177/036354658701500101
   BRADLEY JP, 1990, AM J SPORT MED, V18, P188, DOI 10.1177/036354659001800213
   FOX JM, 1975, CLIN ORTHOP RELAT R, P221
   INGLIS AE, 1981, CLIN ORTHOP RELAT R, V156, P160
   JOZSA L, 1989, AM J SPORT MED, V17, P338, DOI 10.1177/036354658901700305
   KANNUS P, 1991, J BONE JOINT SURG AM, V73A, P1507, DOI 10.2106/00004623-199173100-00009
   KELLAM JF, 1985, CLIN ORTHOP RELAT R, V201, P80
   KLEINMAN M, 1983, J BONE JOINT SURG AM, V65, P1345, DOI 10.2106/00004623-198365090-00019
   LAGERGREN C, 1959, Acta Chir Scand, V116, P491
   LAWRENCE GH, 1955, AM J SURG, V89, P795, DOI 10.1016/0002-9610(55)90146-X
   LINDHOLM A, 1959, Acta Chir Scand, V117, P261
   LYNN TA, 1966, J BONE JOINT SURG AM, VA 48, P268, DOI 10.2106/00004623-196648020-00005
   Percy E C, 1978, Am J Sports Med, V6, P132, DOI 10.1177/036354657800600307
   PEREZTEUFFER A, 1974, ORTHOP CLIN N AM, V5, P89
   Puddu G, 1976, Am J Sports Med, V4, P145, DOI 10.1177/036354657600400404
   RALSTON EL, 1971, J TRAUM, V11, P15, DOI 10.1097/00005373-197101000-00001
   ROCKWOOD CA, 1996, FRACTURES ADULTS, V2, P2254
   THOMPSON T C, 1962, J Trauma, V2, P126, DOI 10.1097/00005373-196203000-00003
   Tonino P. M., 1987, TECHN ORTHOP, V2, P6
   TURCO VJ, 1987, FOOT ANKLE, V7, P253, DOI 10.1177/107110078700700407
NR 23
TC 17
Z9 20
U1 0
U2 2
PU SAGE PUBLICATIONS INC
PI THOUSAND OAKS
PA 2455 TELLER RD, THOUSAND OAKS, CA 91320 USA
SN 1071-1007
EI 1944-7876
J9 FOOT ANKLE INT
JI Foot Ankle Int.
PD JUN
PY 2000
VL 21
IS 6
BP 469
EP 474
DI 10.1177/107110070002100603
PG 6
WC Orthopedics
WE Science Citation Index Expanded (SCI-EXPANDED)
SC Orthopedics
GA 326XB
UT WOS:000087761600003
PM 10884104
DA 2022-07-22
ER

PT J
AU Tang, KL
   Thermann, H
   Dai, G
   Chen, GX
   Guo, L
   Yang, L
AF Tang, Kang-lai
   Thermann, Hajo
   Dai, Gang
   Chen, Guang-xing
   Guo, Lin
   Yang, Liu
TI Arthroscopically assisted percutaneous repair of fresh closed Achilles
   tendon rupture by Kessler's suture
SO AMERICAN JOURNAL OF SPORTS MEDICINE
LA English
DT Article
DE Achilles tendon; rupture; arthroscopy; Kessler's suture
AB Background: Achilles tendon ruptures are difficult to repair, and the healing rate is low due to this structure's anatomic and physiological characteristics. It is essential to develop new techniques to increase the healing rate and decrease the rate of complications.
   Objective: To propose and evaluate a new percutaneous method of repairing fresh closed Achilles tendon ruptures by Kessler's suture under arthroscopy.
   Study Design: Case series; Level of evidence, 4.
   Methods: Twenty patients were followed at least 12 months in this study. First, the torn ends of the Achilles tendon were debrided during arthroscopy. Then percutaneous repair of the Achilles tendon was performed using Kessler's suture by an inside-out technique. All cases were followed up for an average range of 21 months (range, 12-36 months). All patients were evaluated by clinical examination, magnetic resonance imaging, and the Lindholm scale.
   Results: The torn ends were well aligned and sutured after the debridement under arthroscopy. According to the Lindholm scale, excellent results were seen in 15 cases and good in 5 cases. No patients had complications such as nerve injury, infection, or re-rupture at follow-up. Magnetic resonance imaging results showed that the ruptured Achilles tendons were repaired and remodeled very well in all patients.
   Conclusion: The present method is an effective surgical technique for repair of a closed rupture of the Achilles tendon. The short-term follow-up results were good, and recovery time was short. Few complications were found in our study cases.
C1 Third Mil Med Univ, Ctr Joint Surg, Chongqing 400038, Peoples R China.
   ATOS Clin, Heidelberg, Germany.
RP Tang, KL (通讯作者)，Third Mil Med Univ, Ctr Joint Surg, Gaotanyan Str 30, Chongqing 400038, Peoples R China.
EM tangkanglai@hotmail.com
CR ARNER O, 1959, Acta Chir Scand Suppl, V116, P1
   Cretnik A, 2005, AM J SPORT MED, V33, P1369, DOI 10.1177/0363546504271501
   Khan RJK, 2005, J BONE JOINT SURG AM, V87A, P2202, DOI 10.2106/JBJS.D.03049
   LEA RB, 1972, J BONE JOINT SURG AM, VA 54, P1398, DOI 10.2106/00004623-197254070-00002
   Leppilahti J, 1996, ACTA ORTHOP SCAND, V67, P277, DOI 10.3109/17453679608994688
   Lim J, 2001, FOOT ANKLE INT, V22, P559, DOI 10.1177/107110070102200705
   MA GWC, 1977, CLIN ORTHOP RELAT R, P247
   Thermann H, 2001, FOOT ANKLE INT, V22, P158, DOI 10.1177/107110070102200214
   Thermann H, 2001, FOOT ANKLE INT, V22, P478, DOI 10.1177/107110070102200604
NR 9
TC 26
Z9 30
U1 0
U2 5
PU SAGE PUBLICATIONS INC
PI THOUSAND OAKS
PA 2455 TELLER RD, THOUSAND OAKS, CA 91320 USA
SN 0363-5465
EI 1552-3365
J9 AM J SPORT MED
JI Am. J. Sports Med.
PD APR
PY 2007
VL 35
IS 4
BP 589
EP 596
DI 10.1177/0363546506295080
PG 8
WC Orthopedics; Sport Sciences
WE Science Citation Index Expanded (SCI-EXPANDED)
SC Orthopedics; Sport Sciences
GA 155PJ
UT WOS:000245590100010
PM 17170162
DA 2022-07-22
ER

PT J
AU Maffulli, N
   Loppini, M
   Longo, UG
   Maffulli, GD
   Denaro, V
AF Maffulli, Nicola
   Loppini, Mattia
   Longo, Umile Giuseppe
   Maffulli, Gayle D.
   Denaro, Vincenzo
TI Minimally Invasive Reconstruction of Chronic Achilles Tendon Ruptures
   Using the Ipsilateral Free Semitendinosus Tendon Graft and Interference
   Screw Fixation
SO AMERICAN JOURNAL OF SPORTS MEDICINE
LA English
DT Article
DE Achilles tendon; chronic ruptures; semitendinosus tendon graft;
   mini-invasive surgery
ID CRUCIATE LIGAMENT RECONSTRUCTION; SURGICAL REPAIR; CHRONIC TEARS;
   REGIONAL PROPHYLAXIS; NEGLECTED TEARS; FIT FIXATION; BONE TUNNEL;
   REHABILITATION; TENDINOPATHY; REGENERATION
AB Background: Achilles tendon ruptures represent more than 40% of all tendon ruptures requiring surgical management. About 20% of acute Achilles tendon tears are not diagnosed at the time of injury and become chronic, necessitating more complicated management than fresh injuries. Several techniques for the reconstruction of chronic tears of the Achilles tendon have been described, but the superiority of one technique over the others has not been demonstrated.
   Hypothesis: Mini-invasive reconstruction of the Achilles tendon, with a gap lesion larger than 6 cm, using the ipsilateral free semitendinosus tendon graft will result in improvement of the overall function with a low rate of complications.
   Study Design: Case series; Level of evidence, 4.
   Methods: Between 2008 and 2010, the authors prospectively enrolled 28 consecutive patients (21 men and 7 women; median age, 46 years) with chronic closed ruptures of the Achilles tendon who had undergone reconstruction with a free semitendinosus tendon graft. They assessed the Achilles tendon Total Rupture Score (ATRS), maximum calf circumference, and isometric plantarflexion strength before surgery and at the last follow-up. Outcome of surgery and rate of complications were also recorded. The median follow-up after surgery was 31.4 months.
   Results: The overall result of surgery was excellent/good in 26 (93%) of 28 patients. The ATRS improved from 42 (range, 29-55) to 86 (range, 78-95) (P < .0001). In the operated leg, the maximum calf circumference and isometric plantarflexion strength were significantly improved after surgery (P < .0001); however, their values remained significantly lower than those of the opposite side (P < .0001). All patients were able to walk on tiptoes and returned to their preinjury working occupation. No infections were recorded.
   Conclusion: Mini-invasive reconstruction of the Achilles tendon, with a gap lesion larger than 6 cm, using the ipsilateral free semitendinosus tendon graft provides a significant improvement of symptoms and function, although calf circumference and ankle plantarflexion strength do not recover fully.
C1 [Maffulli, Nicola; Loppini, Mattia; Longo, Umile Giuseppe; Maffulli, Gayle D.; Denaro, Vincenzo] Univ London, London, England.
RP Maffulli, N (通讯作者)，Mile End Hosp, Barts & London Sch Med & Dent, Ctr Sports & Exercise Med, 275 Bancroft Rd, London E1 4DG, England.
EM n.maffulli@qmul.ac.uk
RI Denaro, Vincenzo/K-9567-2016; Longo, Umile Giuseppe/K-9147-2016;
   Maffulli, Nicola/A-1839-2014; Loppini, Mattia/J-5620-2018
OI Denaro, Vincenzo/0000-0003-4739-9795; Longo, Umile
   Giuseppe/0000-0003-4063-9821; Maffulli, Nicola/0000-0002-5327-3702;
   Loppini, Mattia/0000-0001-8569-0669
CR Ardern CL, 2010, ARTHROSCOPY, V26, P462, DOI 10.1016/j.arthro.2009.08.018
   BOYDEN EM, 1995, CLIN ORTHOP RELAT R, P150
   Carmont MR, 2008, KNEE SURG SPORT TR A, V16, P199, DOI 10.1007/s00167-007-0411-z
   Carmont MR, 2007, BMC MUSCULOSKEL DIS, V8, DOI 10.1186/1471-2474-8-100
   de Lalla F, 2000, ANTIMICROB AGENTS CH, V44, P316, DOI 10.1128/AAC.44.2.316-319.2000
   Elliot R R, 2007, Foot Ankle Clin, V12, P573, DOI 10.1016/j.fcl.2007.07.002
   Ferretti A, 2002, AM J SPORT MED, V30, P204, DOI 10.1177/03635465020300021001
   FERRIS L, 1995, FOOT ANKLE INT, V16, P464, DOI 10.1177/107110079501600802
   Idler CS, 2006, AM J SPORT MED, V34, P968, DOI 10.1177/0363546505284185
   Jacob HAC, 2001, CLIN BIOMECH, V16, P783, DOI 10.1016/S0268-0033(01)00070-5
   Jacob KM, 2007, ANZ J SURG, V77, P287, DOI 10.1111/j.1445-2197.2007.04035.x
   Jielile J, 2011, APPL BIOCHEM BIOTECH, V165, P1092, DOI 10.1007/s12010-011-9327-7
   JOZSA L, 1989, AM J SPORT MED, V17, P338, DOI 10.1177/036354658901700305
   Lazzarini L, 2003, J ARTHROPLASTY, V18, P342, DOI 10.1054/arth.2003.50053
   Leys T, 2012, AM J SPORT MED, V40, P595, DOI 10.1177/0363546511430375
   Longo UG, 2010, BRIT MED BULL, V94, P165, DOI 10.1093/bmb/ldp051
   Longo UG, 2009, SPORTS MED ARTHROSC, V17, P127, DOI 10.1097/JSA.0b013e3181a3d767
   Longo UG, 2008, DISABIL REHABIL, V30, P1709, DOI 10.1080/09638280701786922
   Loppini M., 2011, MUSCLES LIGAMENTS TE, V1, P133
   Maffulli N, 1999, J BONE JOINT SURG AM, V81A, P1019, DOI 10.2106/00004623-199907000-00017
   Maffulli N, 1997, AM J SPORT MED, V25, P835, DOI 10.1177/036354659702500618
   Maffulli N, 2003, AM J SPORT MED, V31, P692, DOI 10.1177/03635465030310051001
   Maffulli N, 2005, CLIN J SPORT MED, V15, P56, DOI 10.1097/01.jsm.0000152714.05097.ef
   Maffulli N, 1998, AM J SPORT MED, V26, P266, DOI 10.1177/03635465980260021801
   MAFFULLI N, 1989, J SPORT MED PHYS FIT, V29, P365
   Maffulli N, 2008, J BONE JOINT SURG AM, V90A, P1348, DOI 10.2106/JBJS.G.01241
   Maffulli Nicola, 2007, Foot Ankle Clin, V12, P583, DOI 10.1016/j.fcl.2007.07.007
   Maffulli N, 2008, BMC MUSCULOSKEL DIS, V9, DOI 10.1186/1471-2474-9-100
   Maffulli N, 2012, J BONE JOINT SURG AM, V94A, P906, DOI 10.2106/JBJS.K.00869
   Maffulli N, 2012, J BONE JOINT SURG AM, V94A, P901, DOI 10.2106/JBJS.K.00200
   Maffulli N, 2010, AM J SPORT MED, V38, P2304, DOI 10.1177/0363546510376619
   Maffulli N, 2010, KNEE SURG SPORT TR A, V18, P269, DOI 10.1007/s00167-009-0968-9
   Matles A L, 1975, Bull Hosp Joint Dis, V36, P48
   Myerson M S, 1999, Instr Course Lect, V48, P219
   Nilsson-Helander K, 2007, AM J SPORT MED, V35, P421, DOI 10.1177/0363546506294856
   Ozalay M, 2005, ARTHROSCOPY, V21, P992, DOI 10.1016/j.arthro.2005.05.002
   Padanilam TG, 2009, FOOT ANKLE CLIN, V14, P711, DOI 10.1016/j.fcl.2009.08.001
   Papandrea P, 2000, AM J SPORT MED, V28, P556, DOI 10.1177/03635465000280041901
   Pereira DS, 2002, AM J SPORT MED, V30, P432, DOI 10.1177/03635465020300032101
   Pintore E, 2001, J TRAUMA, V50, P71, DOI 10.1097/00005373-200101000-00013
   Prodromos CC, 2005, ARTHROSCOPY, V21, P130, DOI 10.1016/j.arthro.2004.09.010
   Sarzaeem MM, 2012, KNEE SURG SPORT TR A, V20, P1386, DOI 10.1007/s00167-011-1703-x
   SIMMONDS F A, 1957, Practitioner, V179, P56
   Suchak AA, 2008, J BONE JOINT SURG AM, V90A, P1876, DOI 10.2106/JBJS.G.01242
   Suchak AA, 2006, CLIN ORTHOP RELAT R, P216, DOI 10.1097/01.blo.0000203458.05135.74
   Wapner K L, 1993, Foot Ankle, V14, P443
   Webb J, 2000, FOOT ANKLE INT, V21, P475, DOI 10.1177/107110070002100604
   Weiler A, 2002, ARTHROSCOPY, V18, P124, DOI 10.1053/jars.2002.30657
   Weiler A, 2002, ARTHROSCOPY, V18, P113, DOI 10.1053/jars.2002.30656
   Wong J, 2002, AM J SPORT MED, V30, P565, DOI 10.1177/03635465020300041701
   Young SW, 2013, CLIN ORTHOP RELAT R, V471, P244, DOI 10.1007/s11999-012-2469-2
NR 51
TC 25
Z9 28
U1 0
U2 5
PU SAGE PUBLICATIONS INC
PI THOUSAND OAKS
PA 2455 TELLER RD, THOUSAND OAKS, CA 91320 USA
SN 0363-5465
EI 1552-3365
J9 AM J SPORT MED
JI Am. J. Sports Med.
PD MAY
PY 2013
VL 41
IS 5
BP 1100
EP 1107
DI 10.1177/0363546513479017
PG 8
WC Orthopedics; Sport Sciences
WE Science Citation Index Expanded (SCI-EXPANDED)
SC Orthopedics; Sport Sciences
GA 135OQ
UT WOS:000318298300023
PM 23467554
DA 2022-07-22
ER

PT J
AU De la Fuente, C
   Cruz-Montecinos, C
   Schimidt, HL
   Henriquez, H
   Ruidiaz, S
   Carpes, FP
AF De la Fuente, Carlos
   Cruz-Montecinos, Carlos
   Schimidt, Helen L.
   Henriquez, Hugo
   Ruidiaz, Sebastian
   Carpes, Felipe P.
TI Biomechanical properties of different techniques used in vitro for
   suturing mid-substance Achilles tendon ruptures
SO CLINICAL BIOMECHANICS
LA English
DT Article
DE Achilles tendon; Biomechanics; Injury
ID REPAIR; STRENGTH; PLANTARFLEXION; REHABILITATION; MOBILIZATION; ANKLE
AB Background: The Dresden technique preserves the paratenon during Achilles tendon repair and may improve the plantarflexor mechanism when combined with mobilization during early rehabilitation. However, the surgical repair design for Achilles tendon ruptures can affect rates of re-rupture or lengthening. Therefore, the aim of this study was to determine the biomechanical properties of the Krackow, Double-Kessler, Double-Dresden, and Triple-Dresden techniques used for repairing mid-substance Achilles tendon ruptures during cyclical and maximum traction.
   Methods: Sixty mid-substance bovine tendons repaired after transverse rupturing were divided randomly into four groups by repair technique: Krackow, Double-Kessler, Double-Dresden, and Triple-Dresden. Cyclical tractions of 4.7, 5.8, 7.9, and 11.7 mm (equivalent to 5, 8,10, and 15 of dorsal flexion, respectively) were applied to determine gapping, tensile strength, nominal suture stress, repair deformation, and specimens with clinical failure (gap > 5 mm). Maximal traction was applied to measure maximum strength and failure type (Le. suture, knot, or tendon).
   Findings: The Triple-Dresden technique resulted in decreased gapping, nominal suture stress, repair deformation, and quantity of specimens with clinical failure as compared to the other techniques. Furthermore, Triple Dresden tendons showed greater comparative tensile and maximum strength. During maximal traction testing, this technique presented tendon failure, whereas the Krackow, Double-Kessler, and Double-Dresden techniques had suture failures.
   Interpretation: Triple-Dresden repair results in better cyclical and maximum traction strengths, suggesting that this technique might be more appropriate when performing early mobilization after mid-substance Achilles tendon rupture repair.
C1 [De la Fuente, Carlos] Univ Andres Bello, Fac Ciencias Rehabil, Santiago 7591538, Chile.
   [De la Fuente, Carlos] Pontificia Univ Catolica, Carrera Kinesiol, UDA Ciencias Salud, Fac Med, Santiago 7820436, Chile.
   [Cruz-Montecinos, Carlos] Univ Chile, Lab Clin Biomech, Dept Phys Therapy, Fac Med, Santiago 8380453, Chile.
   [Cruz-Montecinos, Carlos] San Jose Hosp, Lab Biomech & Kinesiol, Santiago 8380419, Chile.
   [Schimidt, Helen L.; Carpes, Felipe P.] Univ Fed Pampa, Lab Neuromech, Campus Uruguaiana, BR-97508000 Uruguaiana, Brazil.
   [Henriquez, Hugo; Ruidiaz, Sebastian] Inst Traumatol, Serv Tobillo & Pie, Santiago 8340220, Chile.
RP Carpes, FP (通讯作者)，Univ Fed Pampa, Lab Neuromech, Campus Uruguaiana, BR-97508000 Uruguaiana, Brazil.
EM carpes@unipampa.edu.br
RI , cruz-montecinos/N-2030-2019; Cruz-Montecinos, Carlos/L-4472-2017;
   Carpes, Felipe P./A-1069-2010; Schimidt, Helen Lidiane/O-3419-2019;
   Schimidt, Helen L/F-5926-2019
OI , cruz-montecinos/0000-0002-3835-3368; Cruz-Montecinos,
   Carlos/0000-0002-3835-3368; Carpes, Felipe P./0000-0001-8923-4855;
   Schimidt, Helen Lidiane/0000-0003-4704-0897; Schimidt, Helen
   L/0000-0003-4704-0897; De la Fuente, Carlos/0000-0002-2429-837X
CR Ahn JH, 2011, J FOOT ANKLE SURG, V50, P93, DOI 10.1053/j.jfas.2010.07.008
   Amlang Michael H, 2006, Oper Orthop Traumatol, V18, P287, DOI 10.1007/s00064-006-1178-y
   Beer F., 2012, VECTOR MECH ENG STAT
   Davis WL, 1999, FOOT ANKLE INT, V20, P126, DOI 10.1177/107110079902000210
   De la Fuente Carlos, 2016, Foot (Edinb), V26, P15, DOI 10.1016/j.foot.2015.10.003
   De la Fuente C, 2017, KNEE SURG SPORT TR A, V25, P1849, DOI 10.1007/s00167-016-4182-2
   De la Fuente CI, 2016, J ATHL TRAINING, V51, P1013, DOI 10.4085/1062-6050-51.12.23
   Dinopoulos HT, 2000, J HAND SURG-AM, V25A, P489, DOI 10.1053/jhsu.2000.6456
   Finni T, 2006, CLIN BIOMECH, V21, P67, DOI 10.1016/j.clinbiomech.2005.08.007
   KAKIUCHI M, 1995, J BONE JOINT SURG BR, V77B, P60, DOI 10.1302/0301-620X.77B1.7822398
   Kearney RS, 2012, PHYSIOTHERAPY, V98, P24, DOI 10.1016/j.physio.2011.04.349
   Keller A, 2014, AM J SPORT MED, V42, P731, DOI 10.1177/0363546513511418
   Krackow KA, 2008, ORTHOPEDICS, V31, P931, DOI 10.3928/01477447-20080901-19
   Lantto I, 2015, SCAND J MED SCI SPOR, V25, pE133, DOI 10.1111/sms.12253
   Lee SJ, 2008, FOOT ANKLE INT, V29, P427, DOI 10.3113/FAI.2008.0427
   Longo UG, 2012, KNEE SURG SPORT TR A, V20, P1392, DOI 10.1007/s00167-011-1868-3
   Ma JX, 2014, P I MECH ENG H, V228, P665, DOI 10.1177/0954411914541435
   McCoy BW, 2010, FOOT ANKLE INT, V31, P701, DOI 10.3113/FAI.2010.0701
   Orishimo KF, 2008, J FOOT ANKLE SURG, V47, P34, DOI 10.1053/j.jfas.2007.10.008
   Ortiz C, 2012, J BONE JOINT SURG BR, V94B, P663, DOI 10.1302/0301-620X.94B5.27642
   Palmes D, 2002, J ORTHOPAED RES, V20, P939, DOI 10.1016/S0736-0266(02)00032-3
   Pneumaticos SG, 2000, FOOT ANKLE INT, V21, P551, DOI 10.1177/107110070002100704
   Sadoghi P, 2012, INT ORTHOP, V36, P1947, DOI 10.1007/s00264-012-1533-6
   Sebastin SJ, 2013, J HAND SURG-AM, V38A, P552, DOI 10.1016/j.jhsa.2012.11.033
   Silbernagel KG, 2012, AM J SPORT MED, V40, P1564, DOI 10.1177/0363546512447926
   Suchak AA, 2005, FOOT ANKLE INT, V26, P932, DOI 10.1177/107110070502601106
   Suydam SM, 2015, KNEE SURG SPORT TR A, V23, P868, DOI 10.1007/s00167-013-2512-1
   Taglialavoro G, 2011, STRATEG TRAUMA LIMB, V6, P147, DOI 10.1007/s11751-011-0124-1
NR 28
TC 6
Z9 6
U1 0
U2 6
PU ELSEVIER SCI LTD
PI OXFORD
PA THE BOULEVARD, LANGFORD LANE, KIDLINGTON, OXFORD OX5 1GB, OXON, ENGLAND
SN 0268-0033
EI 1879-1271
J9 CLIN BIOMECH
JI Clin. Biomech.
PD DEC
PY 2017
VL 50
BP 78
EP 83
DI 10.1016/j.clinbiomech.2017.10.008
PG 6
WC Engineering, Biomedical; Orthopedics; Sport Sciences
WE Science Citation Index Expanded (SCI-EXPANDED)
SC Engineering; Orthopedics; Sport Sciences
GA FQ2XA
UT WOS:000418220100012
PM 29032307
DA 2022-07-22
ER

PT J
AU Zhang, LN
   Wan, WB
   Wang, YX
   Jiao, ZY
   Zhang, LH
   Luo, YK
   Tang, PF
AF Zhang, Li-ning
   Wan, Wen-bo
   Wang, Yue-xiang
   Jiao, Zi-yu
   Zhang, Li-hai
   Luo, Yu-kun
   Tang, Pei-fu
TI Evaluation of Elastic Stiffness in Healing Achilles Tendon After
   Surgical Repair of a Tendon Rupture Using In Vivo Ultrasound Shear Wave
   Elastography
SO MEDICAL SCIENCE MONITOR
LA English
DT Article
DE Achilles Tendon; Elasticity Imaging Techniques; Foot Injuries;
   Ultrasonography
ID REAL-TIME SONOELASTOGRAPHY; PATELLAR TENDON; IMMOBILIZATION; MODULUS;
   INJURY; MODEL
AB Background: There has been no published report assessing the mechanical properties of a repaired Achilles tendon after surgery using shear wave elastography (SWE). The aim of this study was to investigate the changes in mechanical properties of the healing Achilles tendon after surgical repair of a tendon rupture using ultrasound SWE and how these changes correlate with tendon function.
   Material/Methods: Twenty-six patients who underwent surgical repair for Achilles tendon rupture were examined with ultrasound SWE coupled with a linear array transducer (4-15 MHz). The elasticity values of the repaired Achilles tendon in a longitudinal view were measured at 12, 24, and 48 weeks postoperatively. Functional outcomes were assessed with the American Orthopedic Foot and Ankle Society (AOFAS) rating system at 12, 24, and 48 weeks postoperatively. General linear regression analysis and correlation coefficients were used to analyze the relationship between elasticity and the AOFAS score.
   Results: There were significant differences with respect to the mean elasticity values and functional scores of the repaired Achilles tendon at 12, 24, and 48 weeks postoperatively (all P<0.05). Tendon function was positively correlated with the elasticity of the repaired Achilles tendon (P=0.0003).
   Conclusions: Our findings suggest that SWE can provide biomechanical information for evaluating the mechanical properties of healing Achilles tendon and predict Achilles tendon function.
C1 [Zhang, Li-ning; Zhang, Li-hai; Tang, Pei-fu] Chinese Peoples Liberat Army Gen Hosp, Dept Orthoped, Beijing, Peoples R China.
   [Wan, Wen-bo; Wang, Yue-xiang; Jiao, Zi-yu; Luo, Yu-kun] Chinese Peoples Liberat Army Gen Hosp, Dept Ultrasound, Beijing, Peoples R China.
RP Tang, PF (通讯作者)，Chinese Peoples Liberat Army Gen Hosp, Dept Orthoped, Beijing, Peoples R China.
EM pftang301@163.com
OI Tang, Peifu/0000-0003-4279-1704
CR Alexander R. M., 1988, ELASTIC MECH ANIMAL
   Arda K, 2011, AM J ROENTGENOL, V197, P532, DOI 10.2214/AJR.10.5449
   Atkinson TS, 1998, J SURG RES, V79, P25, DOI 10.1006/jsre.1998.5387
   Bavu E, 2011, ULTRASOUND MED BIOL, V37, P1361, DOI 10.1016/j.ultrasmedbio.2011.05.016
   Berg WA, 2012, RADIOLOGY, V262, P435, DOI 10.1148/radiol.11110640
   BOSCO C, 1982, ACTA PHYSIOL SCAND, V116, P343, DOI 10.1111/j.1748-1716.1982.tb07152.x
   Busilacchi A, 2014, KNEE SURG SPORTS TRA
   Chen XM, 2013, J ULTRAS MED, V32, P449, DOI 10.7863/jum.2013.32.3.449
   De Zordo T, 2010, ULTRASCHALL MED, V31, P394, DOI 10.1055/s-0028-1109809
   Fenwick SA, 2002, ARTHRITIS RES, V4, P252, DOI 10.1186/ar416
   Gehmert S, 2012, ULTRASCHALL MED, V33, P581, DOI 10.1055/s-0032-1325526
   Holmes GB, 2006, FOOT ANKLE INT, V27, P952, DOI 10.1177/107110070602701115
   JOZSA L, 1989, AM J SPORT MED, V17, P338, DOI 10.1177/036354658901700305
   Kannus P, 1997, SCAND J MED SCI SPOR, V7, P67
   KASPERCZYK WJ, 1993, CLIN ORTHOP RELAT R, P271
   KASPERCZYK WJ, 1991, ARCH ORTHOP TRAUM SU, V110, P158, DOI 10.1007/BF00395800
   KITAOKA HB, 1994, FOOT ANKLE INT, V15, P349, DOI 10.1177/107110079401500701
   Klauser AS, 2010, SEMIN MUSCULOSKEL R, V14, P323, DOI 10.1055/s-0030-1254521
   Koo TK, 2013, J BIOMECH, V46, P2053, DOI 10.1016/j.jbiomech.2013.05.016
   Kot BCW, 2012, PLOS ONE, V7, DOI 10.1371/journal.pone.0044348
   Maffulli N, 1999, CLIN J SPORT MED, V9, P157, DOI 10.1097/00042752-199907000-00007
   Pawlus A, 2015, POL J RADIOL, V80, P164, DOI 10.12659/PJR.893002
   Sandrin L, 2002, IEEE T ULTRASON FERR, V49, P426, DOI 10.1109/58.996560
   Sconfienza LM, 2010, CLIN EXP RHEUMATOL, V28, P373
   Strom AC, 2009, FOOT ANKLE CLIN, V14, P773, DOI 10.1016/j.fcl.2009.08.003
   Sudol-Szopinska I, 2014, POL J RADIOL, V79, P402, DOI 10.12659/PJR.890803
   Tan S, 2012, SKELETAL RADIOL, V41, P1067, DOI 10.1007/s00256-011-1339-4
   Witvrouw E, 2004, SPORTS MED, V34, P443, DOI 10.2165/00007256-200434070-00003
NR 28
TC 46
Z9 52
U1 0
U2 11
PU INT SCIENTIFIC LITERATURE, INC
PI SMITHTOWN
PA 361 FOREST LANE, SMITHTOWN, NY 11787 USA
SN 1643-3750
J9 MED SCI MONITOR
JI Med. Sci. Monitor
PD APR 9
PY 2016
VL 22
BP 1186
EP 1191
DI 10.12659/MSM.895674
PG 6
WC Medicine, Research & Experimental
WE Science Citation Index Expanded (SCI-EXPANDED)
SC Research & Experimental Medicine
GA DI8HD
UT WOS:000373741200001
PM 27072885
OA Green Published
DA 2022-07-22
ER

PT J
AU Nyyssonen, T
   Lantto, I
   Luthje, P
   Selander, T
   Kroger, H
AF Nyyssonen, Timo
   Lantto, Iikka
   Luthje, Peter
   Selander, Tuomas
   Kroeger, Heikki
TI Drug treatments associated with Achilles tendon rupture. A case-control
   study involving 1118 Achilles tendon ruptures
SO SCANDINAVIAN JOURNAL OF MEDICINE & SCIENCE IN SPORTS
LA English
DT Article
DE Achilles tendon; drugs; epidemiology; incidence; rupture
ID INCREASING INCIDENCE; EPIDEMIOLOGY; TENDINOPATHY; MANAGEMENT; REGISTER;
   SURGERY; INJURY
AB The incidence of Achilles tendon (AT) rupture, especially nonsport-related rupture, is increasing, while the reasons for this increase are largely unknown. The association between the AT rupture and the use of various drug treatments was studied. We collected AT rupture patients from the Finnish Hospital Discharge Register. We also acquired information about all the doctor-prescribed drugs they had purchased within 1 year before the rupture. For comparison, we randomly selected age- and sex-matched controls from the Finnish Population Register. There were 1118 AT rupture patients. Several drug groups had a statistically significant association with the AT rupture. Our study confirmed an association between fluoroquinolone antibiotics and AT rupture (OR 2.20, P = 0.005). A statistically significant association of renin-angiotensin II receptor antagonists with tendon rupture (OR 7.59, P = 0.003) was a previously unreported finding. The increasing incidence of AT rupture, especially in middle-aged and elderly patients, might partially be a consequence of the increased use of certain drug treatments. Some associations are probably explained by the symptomatic treatment of the painful tendon before the rupture.
C1 [Nyyssonen, Timo; Selander, Tuomas; Kroeger, Heikki] Kuopio Univ Hosp, Dept Orthopaed Traumatol & Hand Surg, Kuopio, Finland.
   [Lantto, Iikka] Oulu Univ Hosp, Oulu, Finland.
   [Luthje, Peter] North Kymi Hosp, Kouvola, Finland.
   [Kroeger, Heikki] Univ Eastern Finland, Kuopio Musculoskeletal Res Unit, Kuopio, Finland.
RP Nyyssonen, T (通讯作者)，Kuopio Univ Hosp, Dept Orthopaed Traumatol & Hand Surg, Kuopio, Finland.
EM timo.nyyssonen@kuh.fi
FU Medical Research Fund of Mikkeli Central Hospital, Finland
FX Medical Research Fund of Mikkeli Central Hospital, Finland.
CR Ames PRJ, 2008, DISABIL REHABIL, V30, P1646, DOI 10.1080/09638280701785882
   Budny AM, 2015, J FOOT ANKLE SURG, V54, P494, DOI 10.1053/j.jfas.2014.09.037
   Coombes BK, 2010, LANCET, V376, P1751, DOI 10.1016/S0140-6736(10)61160-9
   DesOliveira LP, 2007, AM J CARDIOL, V100, P152
   Finnish Medicines Agency Fimea and Social Insurance Institution, 2016, FINN STAT MED 2015 FINN STAT MED 2015
   Ganestam A, 2016, KNEE SURG SPORT TR A, V24, P3730, DOI 10.1007/s00167-015-3544-5
   Hart Lawrence, 2011, Clin J Sport Med, V21, P540, DOI 10.1097/01.jsm.0000407929.35973.b9
   Huttunen TT, 2014, AM J SPORT MED, V42, P2419, DOI 10.1177/0363546514540599
   Kannus P, 1997, SCAND J MED SCI SPOR, V7, P107
   Kawtharani F, 2016, J FOOT ANKLE SURG, V55, P276, DOI 10.1053/j.jfas.2014.07.005
   Keskimaki I., 1991, INT J HLTH SCI, V2, P15
   Kirchgesner T, 2014, JOINT BONE SPINE, V81, P485, DOI 10.1016/j.jbspin.2014.03.022
   Lantto I, 2015, SCAND J MED SCI SPOR, V25, pE133, DOI 10.1111/sms.12253
   Leppilahti J, 1996, THESIS
   Marie I, 2008, ARTHRIT RHEUM-ARTHR, V59, P367, DOI 10.1002/art.23309
   Mattila VM, 2015, BRIT J SPORT MED, V49, P1084, DOI 10.1136/bjsports-2013-092756
   Nyyssonen T, 2008, SCAND J SURG, V97, P272, DOI 10.1177/145749690809700312
   Official Statistics of Finland (OSF), POPULATION STRUCTURE
   Scott A, 2014, BMJ OPEN, V4, DOI 10.1136/bmjopen-2013-004320
   Sheth U, 2017, BONE JOINT J, V99B, P78, DOI [10.1302/0301-620X.99B1.BJJ-2016-0434.R1, 10.1302/0301-620x.99b1.bjj-2016-0434.r1]
   Stephenson AL, 2013, DRUG SAFETY, V36, P709, DOI 10.1007/s40264-013-0089-8
   Sund R, 2007, METHOD INFORM MED, V46, P558, DOI 10.1160/ME0382
   Sund R, 2012, SCAND J PUBLIC HEALT, V40, P505, DOI 10.1177/1403494812456637
   Teichtahl AJ, 2016, MED J AUSTRALIA, V204, P115, DOI 10.5694/mja15.00806
NR 24
TC 10
Z9 11
U1 0
U2 4
PU WILEY
PI HOBOKEN
PA 111 RIVER ST, HOBOKEN 07030-5774, NJ USA
SN 0905-7188
EI 1600-0838
J9 SCAND J MED SCI SPOR
JI Scand. J. Med. Sci. Sports
PD DEC
PY 2018
VL 28
IS 12
BP 2625
EP 2629
DI 10.1111/sms.13281
PG 5
WC Sport Sciences
WE Science Citation Index Expanded (SCI-EXPANDED)
SC Sport Sciences
GA GZ9ST
UT WOS:000449842400017
PM 30120842
DA 2022-07-22
ER

PT J
AU Barfod, KW
   Hansen, MS
   Holmich, P
   Troelsen, A
   Kristensen, MT
AF Barfod, Kristoffer Weisskirchner
   Hansen, Maria Swennergren
   Holmich, Per
   Troelsen, Anders
   Kristensen, Morten Tange
TI Efficacy of early controlled motion of the ankle compared with no motion
   after non-operative treatment of an acute Achilles tendon rupture: study
   protocol for a randomized controlled trial
SO TRIALS
LA English
DT Article
DE Achilles tendon; Achilles tendon rupture; Non-operative treatment;
   Dynamic mobilization; Early controlled motion
ID NONSURGICAL TREATMENT; EARLY MOBILIZATION; WEIGHT-BEARING; REPAIR;
   REHABILITATION; IMMOBILIZATION; RELIABILITY; ELONGATION; DEFICITS;
   ABILITY
AB Background: Early controlled ankle motion is widely used in the non-operative treatment of acute Achilles tendon rupture, though its safety and efficacy have never been investigated in a randomized setup. The objectives of this study are to investigate if early controlled motion of the ankle affects functional and patient-reported outcomes.
   Methods/design: The study is performed as a blinded, randomized, controlled trial with patients allocated in a 1: 1 ratio to one of two parallel groups. Patients aged from 18 to 70 years are eligible for inclusion. The intervention group performs early controlled motion of the ankle in weeks 3-8 after rupture. The control group is immobilized. In total, 130 patients will be included from one big orthopedic center over a period of 21/2 years. The primary outcome is the patient-reported Achilles tendon Total Rupture Score evaluated at 12 months post-injury. Secondary outcome measures are the heel-rise work test, Achilles tendon elongation, and the rate of re-rupture. The primary analysis will be conducted as intention-to-treat analyses.
   Discussion: This trial is the first to investigate the safety and efficacy of early controlled motion in the treatment of acute Achilles tendon rupture in a randomized setup. The study uses the patient-reported outcome measure, the Achilles tendon Total Rupture Score, as the primary endpoint, as it is believed to be the best surrogate measure for the tendon's actual capability to function in everyday life.
C1 [Barfod, Kristoffer Weisskirchner; Troelsen, Anders] Copenhagen Univ Hosp Hvidovre, Clin Orthoped Res Hvidovre, Dept Orthoped Surg, Kettegard Alle 30, DK-2650 Hvidovre, Denmark.
   [Hansen, Maria Swennergren; Kristensen, Morten Tange] Copenhagen Univ Hosp Hvidovre, Dept Physiotherapy & Occupat Therapy, Phys Med & Rehabil Res Copenhagen PMR C, Kettegard Alle 30, DK-2650 Hvidovre, Denmark.
   [Holmich, Per] Copenhagen Univ Hosp Hvidovre, Sports Orthoped Res Ctr Copenhagen SORC C Arthros, Dept Orthoped Surg, Hvidovre, Denmark.
RP Barfod, KW (通讯作者)，Copenhagen Univ Hosp Hvidovre, Clin Orthoped Res Hvidovre, Dept Orthoped Surg, Kettegard Alle 30, DK-2650 Hvidovre, Denmark.
EM kbarfod@dadlnet.dk
RI Barfod, Kristoffer/AAY-3288-2021; Kristensen, Morten/L-3527-2019
OI Barfod, Kristoffer/0000-0003-2620-5891; Kristensen,
   Morten/0000-0001-5868-4677; Troelsen, Anders/0000-0003-0132-8182;
   Holmich, Per/0000-0003-2098-0272
FU Department of Orthopedic Surgery; Department of Physiotherapy,
   Copenhagen University Hospital Hvidovre
FX The trial's main sponsors are the Department of Orthopedic Surgery and
   the Department of Physiotherapy, Copenhagen University Hospital
   Hvidovre. DJO Global is contributing 44,000(sic) according to a detailed
   research grant agreement. The Danish Rheumatism Association, the Danish
   Medical Research Grant, and a few private non-profit foundations have
   contributed amounts of less than 11,000(sic).
CR Amlang Michael H, 2011, ISRN Orthop, V2011, P869703, DOI 10.5402/2011/869703
   Aspenberg P, 2007, INT ORTHOP, V31, P783, DOI 10.1007/s00264-007-0398-6
   Barfod KW, 2013, J FOOT ANKLE SURG, V52, P629, DOI 10.1053/j.jfas.2013.04.012
   Barfod KW, 2015, KNEE SURG SPORT TR A, V23, P3398, DOI 10.1007/s00167-014-3175-2
   Barfod KW, 2014, J BONE JOINT SURG AM, V96A, P1497, DOI 10.2106/JBJS.M.01273
   Barfod KW, 2014, DAN MED J, V61
   Carmont MR, 2013, FOOT ANKLE SURG, V19, P245, DOI 10.1016/j.fas.2013.06.007
   CETTI R, 1994, CLIN ORTHOP RELAT R, P155
   Costa ML, 2006, J BONE JOINT SURG BR, V88B, P69, DOI 10.1302/0301-620X.88B1.16549
   Ganestam A, 2016, KNEE SURG SPORT TR A, V24, P3730, DOI 10.1007/s00167-015-3544-5
   Ganestam A, 2013, J FOOT ANKLE SURG, V52, P736, DOI 10.1053/j.jfas.2013.07.004
   Gelberman RH, 1989, J HAND THER, V2, P66, DOI DOI 10.1016/S0894-1130(89)80044-4
   Hansen MS, 2016, KNEE SURG SPORT TR A, V24, P1365, DOI 10.1007/s00167-015-3974-0
   Huttunen TT, 2014, AM J SPORT MED, V42, P2419, DOI 10.1177/0363546514540599
   Jiang N, 2012, INT ORTHOP, V36, P765, DOI 10.1007/s00264-011-1431-3
   Jones MP, 2012, J BONE JOINT SURG AM, V88, P1
   Kangas J, 2003, J TRAUMA, V54, P1171, DOI 10.1097/01.TA.0000047945.20863.A2
   Kangas J, 2007, AM J SPORT MED, V35, P59, DOI 10.1177/0363546506293255
   Kearney RS, 2012, J BONE JOINT SURG BR, V94B, P28, DOI 10.1302/0301-620X.94B1.28008
   Kearney RS, 2012, PHYSIOTHERAPY, V98, P24, DOI 10.1016/j.physio.2011.04.349
   Krapf D, 2012, ORTHOPEDICS, V35, pE1383, DOI 10.3928/01477447-20120822-26
   Matsumoto F, 2003, ARCH PHYS MED REHAB, V84, P662, DOI 10.1016/S0003-9993(03)04834-7
   Mortensen NHM, 1999, J BONE JOINT SURG AM, V81A, P983, DOI 10.2106/00004623-199907000-00011
   Nilsson-Helander K, 2007, AM J SPORT MED, V35, P421, DOI 10.1177/0363546506294856
   Nilsson-Helander K, 2010, AM J SPORT MED, V38, P2186, DOI 10.1177/0363546510376052
   Olsson N, 2014, SCAND J MED SCI SPOR, V24, P152, DOI 10.1111/j.1600-0838.2012.01497.x
   Olsson N, 2013, AM J SPORT MED, V41, P2867, DOI 10.1177/0363546513503282
   SALEH M, 1992, J BONE JOINT SURG BR, V74, P206, DOI 10.1302/0301-620X.74B2.1544953
   Silbernagel KG, 2012, AM J SPORT MED, V40, P1564, DOI 10.1177/0363546512447926
   Silbernagel KG, 2010, KNEE SURG SPORT TR A, V18, P258, DOI 10.1007/s00167-009-0889-7
   Soroceanu A, 2012, J BONE JOINT SURG AM, V94A, P2136, DOI 10.2106/JBJS.K.00917
   Strickland James W, 2005, J Hand Ther, V18, P94, DOI 10.1197/j.jht.2005.01.013
   THOMPSON T C, 1962, J Trauma, V2, P126, DOI 10.1097/00005373-196203000-00003
   Twaddle BC, 2007, AM J SPORT MED, V35, P2033, DOI 10.1177/0363546507307503
   Willits K, 2010, J BONE JOINT SURG AM, V92A, P2767, DOI 10.2106/JBJS.I.01401
NR 35
TC 10
Z9 12
U1 0
U2 3
PU BMC
PI LONDON
PA CAMPUS, 4 CRINAN ST, LONDON N1 9XW, ENGLAND
SN 1745-6215
J9 TRIALS
JI Trials
PD NOV 29
PY 2016
VL 17
AR 564
DI 10.1186/s13063-016-1697-2
PG 10
WC Medicine, Research & Experimental
WE Science Citation Index Expanded (SCI-EXPANDED)
SC Research & Experimental Medicine
GA ED5OH
UT WOS:000388901100001
PM 27894329
OA Green Published, gold
DA 2022-07-22
ER

PT J
AU Matsumoto, F
   Trudel, G
   Uhthoff, HK
   Backman, DS
AF Matsumoto, F
   Trudel, G
   Uhthoff, HK
   Backman, DS
TI Mechanical effects of immobilization on the Achilles' tendon
SO ARCHIVES OF PHYSICAL MEDICINE AND REHABILITATION
LA English
DT Article
DE Achilles tendon; biomechanics; contracture; immobilization;
   rehabilitation; tendon injuries
ID TENSILE-STRENGTH; SOLEUS MUSCLE; RABBIT; RUPTURE; LIGAMENTS
AB Objective: To measure the biomechanical effects of immobilization on the Achilles' tendon.
   Design: Experimental, controlled study.
   Setting: Physiatry research laboratory.
   Animals: Twenty adult rabbits.
   Intervention: One hindlimb immobilized in a cast for 4 weeks (n=10) or 8 weeks (n=10). The contralateral legs (n=20) served as controls.
   Main Outcome Measures: Cross-sectional Achilles' tendon area, mode of failure, mean failure load, and tendon stiffness.
   Results: The Achilles' tendon cross-sectional area did not change. Achilles' tendon- calcaneus units failed at insertion at 4 weeks and in controls. Calcaneus fracture was the most prevalent mode of failure at 8 weeks. The mean load to failure of both immobilized groups (4wk: 187.5+/-45.7N; 8wk: 162.6+/-39.3N) was significantly smaller than that of the control group (549.2+/-93.7N, both P<.005). The mean tendon stiffness of both immobilized groups (4wk: 64.6+/-24.8N/mm; 8wk: 53.9+/-19.9N/mm) was significantly lower than that of the control group (125.1+/-26.5N/mm, both P<.005).
   Conclusions: Immobility for up to 8 weeks caused decreased stiffness but no atrophy or rupture of the Achilles' tendon, suggesting that immobility does not constitute a risk factor for Achilles' tendon midsubstance rupture. Clinically, graded reloading is required after immobilization to restore the tendon insertion and to reverse calcaneal disuse osteoporosis.
C1 Univ Ottawa, Div Phys Med & Rehabil, Bone & Joint Lab, Rehabil Ctr, Ottawa, ON K1H 8M2, Canada.
   Univ Ottawa, Div Orthopaed Surg, Ottawa, ON K1H 8M2, Canada.
RP Trudel, G (通讯作者)，Univ Ottawa, Div Phys Med & Rehabil, Bone & Joint Lab, Rehabil Ctr, 505 Smyth Rd, Ottawa, ON K1H 8M2, Canada.
OI Backman, David/0000-0002-8765-8133
CR AMIEL D, 1982, ACTA ORTHOP SCAND, V53, P325, DOI 10.3109/17453678208992224
   ARNER O, 1959, ACTA CHIR SCAND    S, V239, P1
   BARFRED T, 1971, ACTA ORTHOP SCAND, V42, P406, DOI 10.3109/17453677108989062
   BINKLEY JM, 1986, CLIN ORTHOP RELAT R, V203, P301
   CARR AJ, 1989, J BONE JOINT SURG BR, V71, P100, DOI 10.1302/0301-620X.71B1.2914976
   ENWEMEKA CS, 1992, AM J PHYS MED REHAB, V71, P31, DOI 10.1097/00002060-199202000-00008
   HALAR EM, 1998, REHABILITATION MED P, P1015
   Hannafin JA, 1995, J ORTHOP RES, V13, P907, DOI 10.1002/jor.1100130615
   HARWOOD FL, 1992, J APPL PHYSIOL, V72, P1687, DOI 10.1152/jappl.1992.72.5.1687
   HERBERT RD, 1993, J ORTHOPAED RES, V11, P358, DOI 10.1002/jor.1100110307
   JOZSA L, 1984, FINN SPORTS EXERC ME, V3, P111
   Kannus P, 1997, SCAND J MED SCI SPOR, V7, P67
   KVIST M, 1994, SPORTS MED, V18, P173, DOI 10.2165/00007256-199418030-00004
   LARSEN NP, 1987, ACTA ORTHOP SCAND, V58, P260, DOI 10.3109/17453678709146480
   Leppilahti J, 1996, ACTA ORTHOP SCAND, V67, P277, DOI 10.3109/17453679608994688
   LOITZ B, 1989, CLIN ORTHOP RELAT R, V244, P265
   MAXWELL LC, 1992, ANAT REC, V234, P55, DOI 10.1002/ar.1092340107
   Moller A, 1996, ACTA ORTHOP SCAND, V67, P479, DOI 10.3109/17453679608996672
   NEWTON PO, 1995, J ORTHOP RES, V13, P191, DOI 10.1002/jor.1100130207
   NOYES FR, 1977, CLIN ORTHOP RELAT R, P210
   NOYES FR, 1974, J BONE JOINT SURG AM, VA 56, P236, DOI 10.2106/00004623-197456020-00002
   NOYES FR, 1974, J BONE JOINT SURG AM, VA 56, P1406, DOI 10.2106/00004623-197456070-00009
   OZKAYA N, 1998, MECH PROPERTIES BIOL, P196
   OZKAYA N, 1998, FUNDAMENTALS BIOMECH, P126
   Sano H, 1997, J ORTHOP RES, V15, P719, DOI 10.1002/jor.1100150514
   Sell S, 1996, ARCH ORTHOP TRAUM SU, V115, P28, DOI 10.1007/BF00453213
   TABARY JC, 1972, J PHYSIOL-LONDON, V224, P231, DOI 10.1113/jphysiol.1972.sp009891
   VIIDIK A, 1969, ACTA ORTHOP SCAND, V40, P261, DOI 10.3109/17453676908989506
   WOO SLY, 1975, ARTHRITIS RHEUM, V18, P257, DOI 10.1002/art.1780180311
NR 29
TC 65
Z9 68
U1 1
U2 13
PU W B SAUNDERS CO-ELSEVIER INC
PI PHILADELPHIA
PA 1600 JOHN F KENNEDY BOULEVARD, STE 1800, PHILADELPHIA, PA 19103-2899 USA
SN 0003-9993
EI 1532-821X
J9 ARCH PHYS MED REHAB
JI Arch. Phys. Med. Rehabil.
PD MAY
PY 2003
VL 84
IS 5
BP 662
EP 667
DI 10.1016/S0003-9993(03)04834-7
PG 6
WC Rehabilitation; Sport Sciences
WE Science Citation Index Expanded (SCI-EXPANDED)
SC Rehabilitation; Sport Sciences
GA 675NF
UT WOS:000182700900007
PM 12736878
DA 2022-07-22
ER

PT J
AU Wang, CC
   Chen, PY
   Wang, TM
   Wang, CL
AF Wang, Chen-Chie
   Chen, Pei-Yu
   Wang, Ting-Ming
   Wang, Chung-Li
TI Ultrasound-Guided Minimally Invasive Surgery for Achilles Tendon
   Rupture: Preliminary Results
SO FOOT & ANKLE INTERNATIONAL
LA English
DT Article
DE Achilles Tendon; Ultrasound; Acute Ruptures
ID NONOPERATIVE TREATMENT; PERCUTANEOUS REPAIR; SURGICAL REPAIRS;
   TENDINOPATHY; MANAGEMENT; VASCULARIZATION; METAANALYSIS; UPDATE; ANKLE
AB Background: Many surgeons prefer surgical repair for Achilles tendon ruptures in an attempt to reduce the risk of rerupture. To minimize wound complications, the use of minimally invasive surgery has become more popular recently. In line with this, the use of ultrasound to guide Achilles tendon repair is reported in this study. Methods: From March 2005 to January 2008, 23 patients with Achilles tendon rupture were repaired by the same surgeon. The ages of the patients ranged from 19 to 67 years old, with an average of 43 years old. The repair of the Achilles tendon was achieved through a stab wound under the guidance of ultrasonography. A control group consisted of 25 patients who received traditional open Achilles tendon repair. Results: The average operation time was 52 minutes, and the average wound size was 1.1 cm. The short leg cast was removed 4 weeks after the surgery, and serial casting was used for another 3 to 4 weeks. The postoperative AOFAS ankle-hindfoot scores were 98.7 in the experimental group, 96.5 in the control group with no significant difference. The rates of local infection, stiffness of the ankle, pain of the scar and sural nerve injury were better in the experimental group than in the control group with significant difference. Conclusions: Ultrasound-guided surgery was a good choice due to its availability and real-time soft tissue visualization. It can further minimize the size of the surgical wound. Our method has the potential to achieve reliable results.
RP Wang, CC (通讯作者)，289 Jianguo Rd, New Taipei City 23142, Taiwan.
EM xavier-wang@yahoo.com.tw
OI WANG, CHUNG-LI/0000-0002-3364-8183; Chen, Pei-yu/0000-0003-2793-2361
CR Ahmed IM, 1998, J ORTHOPAED RES, V16, P591, DOI 10.1002/jor.1100160511
   Aktas S, 2009, FOOT ANKLE INT, V30, P391, DOI 10.3113/FAI.2009.0391
   Alfredson H, 2003, CLIN SPORT MED, V22, P727, DOI 10.1016/S0278-5919(03)00010-3
   Assal M, 2002, J BONE JOINT SURG AM, V84A, P161, DOI 10.2106/00004623-200202000-00001
   Bauer G, 1999, SPORTVERLETZ SPORTSC, V13, P79, DOI 10.1055/s-2007-993321
   Bhandari M, 2002, CLIN ORTHOP RELAT R, P190
   BRADLEY JP, 1990, AM J SPORT MED, V18, P188, DOI 10.1177/036354659001800213
   Calder JDF, 2005, BRIT J SPORT MED, V39, P857, DOI 10.1136/bjsm.2004.017509
   CARTER TR, 1992, AM J SPORT MED, V20, P459, DOI 10.1177/036354659202000417
   CETTI R, 1993, AM J SPORT MED, V21, P791, DOI 10.1177/036354659302100606
   Cretnik A, 2005, AM J SPORT MED, V33, P1369, DOI 10.1177/0363546504271501
   Cretnik A, 2010, FOOT ANKLE INT, V31, P14, DOI 10.3113/FAI.2010.0014
   Elliot R R, 2007, Foot Ankle Clin, V12, P573, DOI 10.1016/j.fcl.2007.07.002
   Goren D, 2005, FOOT ANKLE INT, V26, P286, DOI 10.1177/107110070502600404
   Hufner TM, 2006, FOOT ANKLE INT, V27, P167, DOI 10.1177/107110070602700302
   Ismail M, 2008, FOOT ANKLE INT, V29, P808, DOI 10.3113/FAI.2008.0808
   Khan KM, 1998, CLIN J SPORT MED, V8, P151, DOI 10.1097/00042752-199807000-00001
   Khan RJK, 2005, J BONE JOINT SURG AM, V87A, P2202, DOI 10.2106/JBJS.D.03049
   KITAOKA HB, 1994, FOOT ANKLE INT, V15, P349, DOI 10.1177/107110079401500701
   MA GWC, 1977, CLIN ORTHOP RELAT R, P247
   Maffulli N, 2002, J BONE JOINT SURG BR, V84B, P1, DOI 10.1302/0301-620X.84B1.12792
   Maffulli N, 2010, CLIN ORTHOP RELAT R, V468, P1039, DOI 10.1007/s11999-009-0944-1
   Maffulli N, 2009, ORTHOP CLIN N AM, V40, P491, DOI 10.1016/j.ocl.2009.05.006
   Pajala A, 2002, J BONE JOINT SURG AM, V84A, P2016, DOI 10.2106/00004623-200211000-00017
   Quagliarella L, 2010, J STRENGTH COND RES, V24, P1229, DOI 10.1519/JSC.0b013e3181cb281a
   Quagliarella L, 2010, FOOT ANKLE INT, V31, P306, DOI 10.3113/FAI.2010.0306
   Reach JS, 2009, FOOT ANKLE INT, V30, P239, DOI 10.3113/FAI.2009.0239
   SALEH M, 1992, J BONE JOINT SURG BR, V74, P206, DOI 10.1302/0301-620X.74B2.1544953
   Silvestri E, 2003, INT J TISSUE REACT, V25, P149
   Soubeyrand M, 2010, FOOT ANKLE INT, V31, P1069, DOI 10.3113/FAI.2010.1069
   STEIN SR, 1976, ORTHOP CLIN N AM, V7, P241
   Testa V, 2002, MED SCI SPORT EXER, V34, P573, DOI 10.1097/00005768-200204000-00002
   Theobald P, 2005, INJURY, V36, P1267, DOI 10.1016/j.injury.2005.02.012
   THOMPSON T C, 1962, J Trauma, V2, P126, DOI 10.1097/00005373-196203000-00003
   Wallace RGH, 2004, J BONE JOINT SURG AM, V86A, P1198, DOI 10.2106/00004623-200406000-00011
   Webb JM, 1999, J BONE JOINT SURG BR, V81B, P877, DOI 10.1302/0301-620X.81B5.9784
   Weber M, 2003, AM J SPORT MED, V31, P685, DOI 10.1177/03635465030310050901
   Wijesekera NT, 2010, SKELETAL RADIOL, V39, P425, DOI 10.1007/s00256-009-0873-9
   Wong J, 2002, AM J SPORT MED, V30, P565, DOI 10.1177/03635465020300041701
   Young Jonathan S, 2005, Foot Ankle Clin, V10, P371, DOI 10.1016/j.fcl.2005.01.004
NR 40
TC 6
Z9 11
U1 0
U2 4
PU SAGE PUBLICATIONS INC
PI THOUSAND OAKS
PA 2455 TELLER RD, THOUSAND OAKS, CA 91320 USA
SN 1071-1007
EI 1944-7876
J9 FOOT ANKLE INT
JI Foot Ankle Int.
PD JUL
PY 2012
VL 33
IS 7
BP 582
EP 590
DI 10.3113/FAI.2012.0582
PG 9
WC Orthopedics
WE Science Citation Index Expanded (SCI-EXPANDED)
SC Orthopedics
GA 967UB
UT WOS:000305932200008
PM 22835396
DA 2022-07-22
ER

PT J
AU Jaakkola, JI
   Beskin, JL
   Griffith, LH
   Cernansky, G
AF Jaakkola, JI
   Beskin, JL
   Griffith, LH
   Cernansky, G
TI Early ankle motion after triple bundle technique repair vs. casting for
   acute Achilles tendon rupture
SO FOOT & ANKLE INTERNATIONAL
LA English
DT Article
DE achilles tendon; retrospective study; tendon injuries
ID SURGICAL REPAIR; NONOPERATIVE TREATMENT; OPERATIVE REPAIR; STRENGTH
AB The purpose of our study is to evaluate the triple bundle technique for acute Achilles tendon rupture repair followed by early (at 14 days) postoperative ankle range of motion compared to nonoperative treatment with delayed ankle range of motion.
   We retrospectively reviewed 73 patients with an acute Achilles tendon rupture treated with either a plantar flexed cast or surgical repair. We found operative treatment reduces immobilization time, allows safe early return to weight-bearing, and diminishes risk of rerupture compared to nonoperative treatment. However, at an average follow-up of greater than 3.5 years, there was no statistical difference in AOFAS hindfoot scores, strength, or patient satisfaction between the two groups. Significant complications were higher in the nonoperative group manifested by three reruptures (7.7%) vs. one deep wound dehiscence in the operative group (3%).
C1 Atlanta Med Ctr, Atlanta, GA USA.
   Peachtree Orthopaed Clin, Atlanta, GA USA.
   Physiotherapy Associates, Atlanta, GA USA.
RP Beskin, JL (通讯作者)，2001 Peachtree Rd NE,Ste 705, Atlanta, GA 30309 USA.
CR BESKIN JL, 1987, AM J SPORT MED, V15, P1, DOI 10.1177/036354658701500101
   CETTI R, 1994, CLIN ORTHOP RELAT R, P155
   CETTI R, 1993, AM J SPORT MED, V21, P791, DOI 10.1177/036354659302100606
   FENANDEZFAIREN M, 1997, AM J SPORTS MED, V25, P177
   Jaakkola JI, 2000, FOOT ANKLE INT, V21, P14, DOI 10.1177/107110070002100103
   Leppilahti J, 1998, SPORTS MED, V25, P79, DOI 10.2165/00007256-199825020-00002
   Leppilahti J, 2000, FOOT ANKLE INT, V21, P330, DOI 10.1177/107110070002100410
   Lo IKY, 1997, CLIN J SPORT MED, V7, P207, DOI 10.1097/00042752-199707000-00010
   Maffulli N, 1999, J BONE JOINT SURG AM, V81A, P1019, DOI 10.2106/00004623-199907000-00017
   MANDELBAUM BR, 1995, AM J SPORT MED, V23, P392, DOI 10.1177/036354659502300403
   MARTI RK, 1983, NETH J SURG, V35, P61
   Mortensen NHM, 1999, J BONE JOINT SURG AM, V81A, P983, DOI 10.2106/00004623-199907000-00011
   Motta P, 1997, AM J SPORT MED, V25, P172, DOI 10.1177/036354659702500205
   NISTOR L, 1981, J BONE JOINT SURG AM, V63, P394, DOI 10.2106/00004623-198163030-00012
   SAW Y, 1993, INJURY, V24, P479, DOI 10.1016/0020-1383(93)90155-Y
   Soldatis JJ, 1997, AM J SPORT MED, V25, P90, DOI 10.1177/036354659702500118
   SOLVEBORN SA, 1994, AM J SPORT MED, V22, P607, DOI 10.1177/036354659402200507
   SOMA CA, 1995, ORTHOP CLIN N AM, V26, P239
   Speck M, 1998, AM J SPORT MED, V26, P789, DOI 10.1177/03635465980260060901
   TROOP RL, 1995, FOOT ANKLE INT, V16, P705, DOI 10.1177/107110079501601106
   WATSON TW, 1995, FOOT ANKLE INT, V16, P191, DOI 10.1177/107110079501600404
   WEBER BG, 1972, RUPTURE ACHILLES TEN, P857
   WILLS CA, 1986, CLIN ORTHOP RELAT R, P156
NR 23
TC 20
Z9 22
U1 0
U2 1
PU AMER ORTHOPAEDIC FOOT & ANKLE SOC, INC
PI SEATTLE
PA 2517 EASTLAKE AVE EAST, STE 200, SEATTLE, WA 98102 USA
SN 1071-1007
J9 FOOT ANKLE INT
JI Foot Ankle Int.
PD DEC
PY 2001
VL 22
IS 12
BP 979
EP 984
DI 10.1177/107110070102201210
PG 6
WC Orthopedics
WE Science Citation Index Expanded (SCI-EXPANDED)
SC Orthopedics
GA 536HN
UT WOS:000174694800010
PM 11783925
DA 2022-07-22
ER

PT J
AU Silbernagel, KG
   Willy, R
   Davis, I
AF Silbernagel, Karin Graevare
   Willy, Richard
   Davis, Irene
TI Preinjury and Postinjury Running Analysis Along With Measurements of
   Strength and Tendon Length in a Patient With a Surgically Repaired
   Achilles Tendon Rupture
SO JOURNAL OF ORTHOPAEDIC & SPORTS PHYSICAL THERAPY
LA English
DT Article
DE Achilles tendon Total Rupture Score (ATRS); biomechanics; heel-rise test
ID NONOPERATIVE TREATMENT; MUSCLE; ANKLE; FOOT; KINEMATICS; ABILITY; RANGE;
   SCORE
AB STUDY DESIGN: Case report.
   BACKGROUND: The Achilles tendon is the most frequently ruptured tendon, and the incidence of Achilles tendon rupture has increased in the last decade. The rupture generally occurs without any preceding warning signs, and therefore preinjury data are seldom available. This case represents a unique opportunity to compare preinjury running mechanics with postinjury evaluation in a patient with an Achilles tendon rupture.
   CASE DESCRIPTION: A 23-year-old female sustained a right complete Achilles tendon rupture while playing soccer. Running mechanics data were collected preinjury, as she was a healthy participant in a study on running analysis. In addition, patient-reported symptoms, physical activity level, strength, ankle range of motion, heel-rise ability, Achilles tendon length, and running kinetics were evaluated 1 year after surgical repair.
   OUTCOMES: During running, greater ankle dorsiflexion and eversion and rearfoot abduction were noted on the involved side postinjury when compared to preinjury data. In addition, post injury, the magnitude of all kinetics data was lower on the involved limb when compared to the uninvolved limb. The involved side displayed differences in strength, ankle range of motion, heel rise, and tendon length when compared to the uninvolved side 1 year after injury.
   DISCUSSION: Despite a return to normal running routine and reports of only minor limitations with running, considerable changes were noted in running biomechanics 1 year after injury. Calf muscle weakness and Achilles tendon elongation were also found when comparing the involved and uninvolved sides. J Orthop Sports Phys Ther 2012;42(6):521-529, Epub 25 January 2012. doi:10.2519/jospt.2012.3913
C1 [Silbernagel, Karin Graevare] Univ Sci, Samson Coll Hlth Profess, Dept Phys Therapy, Philadelphia, PA 19104 USA.
   [Willy, Richard] Ohio Univ, Athens, OH 45701 USA.
   [Davis, Irene] Harvard Univ, Sch Med, Spaulding Natl Running Ctr, Cambridge, MA USA.
RP Silbernagel, KG (通讯作者)，Univ Sci, Samson Coll Hlth Profess, Dept Phys Therapy, 600 S 43rd St, Philadelphia, PA 19104 USA.
EM k.silbernagel@usciences.edu
OI Willy, Richard/0000-0002-1249-228X; Gravare Silbernagel,
   Karin/0000-0001-7566-407X
FU Swedish Research Council; DOD [W911NF-05-1-0097]; NIH [1 S10 RR022396];
   NATIONAL CENTER FOR RESEARCH RESOURCES [S10RR022396] Funding Source: NIH
   RePORTER
FX <SUP>1</SUP>Assistant Professor, University of the Sciences,
   Philadelphia, PA. <SUP>2</SUP>Assistant Professor, Ohio University,
   Athens, OH. <SUP>3</SUP>Director, Spaulding National Running Center,
   Harvard Medical School, Cambridge, MA. This case was seen at the
   Department of Physical Therapy and Department of Mechanical Engineering,
   University of Delaware, Newark, DE. At the time of the case, Karin
   Gravare Silbernagel was performing postdoctoral research at the
   Department of Mechanical Engineering and Richard Willy was a doctoral
   candidate under the mentorship of Irene Davis. The studies were approved
   by the Human Subjects Review Board at the University of Delaware, and
   the subject gave informed consent. This case study was funded by the
   Swedish Research Council, DOD W911NF-05-1-0097, and NIH 1 S10 RR022396.
   Address correspondence to Dr Karin Gravare Silbernagel, University of
   the Sciences, Samson College of Health Professions, Department of
   Physical Therapy, 600 South 43rd Street, Philadelphia, PA 19104. E-mail:
   k.silbernagel@usciences.edu
CR Aroen A, 2004, SCAND J MED SCI SPOR, V14, P30, DOI 10.1111/j.1600-0838.2004.00344.x
   Costa ML, 2005, J BONE JOINT SURG BR, V87B, P1085, DOI 10.1302/0301-620X.87B8.16540
   Crawford J, 2004, COCHRANE DB SYST REV, DOI DOI 10.1002/14651858.CD003674.PUB2
   DEMPSTER WT, 1959, AM J PHYS ANTHROPOL, V17, P289, DOI 10.1002/ajpa.1330170405
   Don R, 2007, CLIN BIOMECH, V22, P211, DOI 10.1016/j.clinbiomech.2006.10.001
   Fellin RE, 2010, J APPL BIOMECH, V26, P407, DOI 10.1123/jab.26.4.407
   Flemister AS, 2007, FOOT ANKLE INT, V28, P448, DOI 10.3113/FAI.2007.0448
   GRIMBY G, 1986, ACTA MED SCAND, P233
   Hof AL, 2002, ACTA PHYSIOL SCAND, V174, P17, DOI 10.1046/j.1365-201x.2002.00917.x
   Houshian S, 1998, INJURY, V29, P651, DOI 10.1016/S0020-1383(98)00147-8
   Jozsa L, 1997, SCAND J MED SCI SPOR, V7, P113
   Kangas J, 2007, AM J SPORT MED, V35, P59, DOI 10.1177/0363546506293255
   KANNUS P, 1991, J BONE JOINT SURG AM, V73A, P1507, DOI 10.2106/00004623-199173100-00009
   Kannus P, 1997, SCAND J MED SCI SPOR, V7, P107
   KELLAM JF, 1985, CLIN ORTHOP RELAT R, P80
   Lo IKY, 1997, CLIN J SPORT MED, V7, P207, DOI 10.1097/00042752-199707000-00010
   Maffulli N, 2000, AM J SPORT MED, V28, P499, DOI 10.1177/03635465000280040901
   MANDELBAUM BR, 1995, AM J SPORT MED, V23, P392, DOI 10.1177/036354659502300403
   Martin RRL, 2005, FOOT ANKLE INT, V26, P968, DOI 10.1177/107110070502601113
   McComis GP, 1997, J BONE JOINT SURG AM, V79A, P1799, DOI 10.2106/00004623-199712000-00005
   Moller A, 1996, ACTA ORTHOP SCAND, V67, P479, DOI 10.3109/17453679608996672
   MORTENSEN NHM, 1992, ORTHOPEDICS, V15, P899
   Mullaney MJ, 2006, AM J SPORT MED, V34, P1120, DOI 10.1177/0363546505284186
   Nilsson-Helander K, 2007, AM J SPORT MED, V35, P421, DOI 10.1177/0363546506294856
   Nilsson-Helander K, 2010, AM J SPORT MED, V38, P2186, DOI 10.1177/0363546510376052
   Pandy MG, 2010, J BIOMECH, V43, P2055, DOI 10.1016/j.jbiomech.2010.04.010
   Parekh Selene G, 2009, Foot Ankle Spec, V2, P283, DOI 10.1177/1938640009351138
   Rees JD, 2008, RHEUMATOLOGY, V47, P1493, DOI 10.1093/rheumatology/ken262
   Riley PO, 2008, MED SCI SPORT EXER, V40, P1093, DOI 10.1249/MSS.0b013e3181677530
   Roos EM, 2001, FOOT ANKLE INT, V22, P788, DOI 10.1177/107110070102201004
   Silbernagel KG, 2010, KNEE SURG SPORT TR A, V18, P258, DOI 10.1007/s00167-009-0889-7
   Suchak AA, 2005, FOOT ANKLE INT, V26, P932, DOI 10.1177/107110070502601106
   Tome J, 2006, J ORTHOP SPORT PHYS, V36, P635, DOI 10.2519/jospt.2006.2293
   van Sterkenburg MN, 2008, ACHILLES TENDINOPATH, P34
   Willits K, 2010, J BONE JOINT SURG AM, V92A, P2767, DOI 10.2106/JBJS.I.01401
NR 35
TC 23
Z9 23
U1 0
U2 18
PU J O S P T
PI ALEXANDRIA
PA 1111 NORTH FAIRFAX ST, STE 100, ALEXANDRIA, VA 22314-1436 USA
SN 0190-6011
EI 1938-1344
J9 J ORTHOP SPORT PHYS
JI J. Orthop. Sports Phys. Ther.
PD JUN
PY 2012
VL 42
IS 6
BP 521
EP 529
DI 10.2519/jospt.2012.3913
PG 9
WC Orthopedics; Rehabilitation; Sport Sciences
WE Science Citation Index Expanded (SCI-EXPANDED)
SC Orthopedics; Rehabilitation; Sport Sciences
GA 952IO
UT WOS:000304786400006
PM 22282229
DA 2022-07-22
ER

PT J
AU Fotiadis, E
   Chatzisimeon, A
   Samoladas, E
   Antonarakos, P
   Akritopoulos, P
   Akritopoulou, K
AF Fotiadis, Elias
   Chatzisimeon, Apostolos
   Samoladas, Efthimios
   Antonarakos, Petros
   Akritopoulos, Panagiotis
   Akritopoulou, Kiriaki
TI A combined repair technique for early neglected Achilles tendon ruptures
SO EUROPEAN JOURNAL OF TRAUMA AND EMERGENCY SURGERY
LA English
DT Article
DE Achilles tendon; neglected ruptures; reconstruction; augmented;
   plantaris
ID CALCANEAL TENDON; RECONSTRUCTION; INJURY; MARLEX
AB Objective: This study presents the long-term results of a combined technique, Using both Duthie's biological repair and a plantaris tendon transfer for the reconstruction of early neglected tendo Achilles ruptures.
   Materials and Method: Between January 2000 and November 2004, nine patients with an early neglected Achilles tendon rupture (>= 4 and <= 12 weeks from injury), underwent surgical reconstruction. Eight patients were male and one female, their age was average 41 years and the median follow-up period was 3.66 years. The average clinical defect of the Achilles tendon was 4.33 cm.
   Results: All patients were examined by the clinical ankle scoring system of Leppilahti et al. (Clin Orthop 346:152-161, 1998). Six patients presented excellent results and three patients had good results. All patients had equal plantarflexion and dorsiflexion with the healthy side and all of the study's patients returned to work 3 months post-operatively. An average muscle atrophy of 0.83 cm was documented compared with the uninjured side. Only four of nine patients (44.44%) had a normal one-legged jump. There were no complications postoperatively and no re-ruptures of the Achilles tendons.
   Conclusion: This combined method appears to be safe and effective, with a low risk for complications, allowing us to obtain excellent results for the majority of our patients.
C1 [Fotiadis, Elias; Samoladas, Efthimios; Antonarakos, Petros; Akritopoulos, Panagiotis; Akritopoulou, Kiriaki] Interbalkan Med Ctr, Thessaloniki 54622, Greece.
   [Chatzisimeon, Apostolos] King George Hosp, London, England.
RP Fotiadis, E (通讯作者)，Interbalkan Med Ctr, Ag Sofias 30, Thessaloniki 54622, Greece.
EM fotiad-h@otenet.gr
CR AZAR FM, 2003, CAMPBELLS OPERATIVE, P2449
   BOSWORTH DM, 1956, J BONE JOINT SURG AM, V38, P111, DOI 10.2106/00004623-195638010-00011
   CARDEN DG, 1987, J BONE JOINT SURG BR, V69, P416, DOI 10.1302/0301-620X.69B3.3294839
   Davis WL, 1999, FOOT ANKLE INT, V20, P126, DOI 10.1177/107110079902000210
   Fletcher MDA, 2001, BRIT J SPORT MED, V35, P131, DOI 10.1136/bjsm.35.2.131
   Hof AL, 2002, ACTA PHYSIOL SCAND, V174, P17, DOI 10.1046/j.1365-201x.2002.00917.x
   HOWARD CB, 1984, J BONE JOINT SURG BR, V66, P206, DOI 10.1302/0301-620X.66B2.6707056
   Kartus J, 2001, ARTHROSCOPY, V17, P971, DOI 10.1053/jars.2001.28979
   KEENE JS, 1994, ORTHOPAEDICS SPORTS, V2, P1794
   Lee YS, 2005, ORTHOPEDICS, V28, P647, DOI 10.3928/0147-7447-20050701-10
   Leppilahti J, 1998, SPORTS MED, V25, P79, DOI 10.2165/00007256-199825020-00002
   LEPPILAHTI J, 1998, CLIN ORTHOP RELAT R, V346, P152
   Maffulli N, 1999, J BONE JOINT SURG AM, V81A, P1019, DOI 10.2106/00004623-199907000-00017
   MAHMOUD SW, 1992, J BONE JOINT SURG AM, V74, P114
   MANN RA, 1991, J BONE JOINT SURG AM, V73A, P214, DOI 10.2106/00004623-199173020-00009
   MOHAMMED A, 1995, J BONE JOINT SURG BR, V77B, P64, DOI 10.1302/0301-620X.77B1.7822399
   Moller M, 2001, J BONE JOINT SURG BR, V83B, P843
   Muermans S, 1998, J HAND SURG-BRIT EUR, V23B, P64, DOI 10.1016/S0266-7681(98)80222-2
   Naim Fadel, 2005, Acta Orthop Traumatol Turc, V39, P1
   OZAKI J, 1989, CLIN ORTHOP RELAT R, V238, P204
   Pintore E, 2001, J TRAUMA, V50, P71, DOI 10.1097/00005373-200101000-00013
   Porter DA, 1997, FOOT ANKLE INT, V18, P557, DOI 10.1177/107110079701800905
   Stein H, 2005, ORTHOPEDICS, V28, P375, DOI 10.3928/0147-7447-20050401-11
   Us AK, 1997, ARCH ORTHOP TRAUM SU, V116, P408
   Wapner K L, 1993, Foot Ankle, V14, P443
   WAPNER KL, 1995, ORTHOP CLIN N AM, V26, P249
NR 26
TC 2
Z9 2
U1 0
U2 4
PU SPRINGER HEIDELBERG
PI HEIDELBERG
PA TIERGARTENSTRASSE 17, D-69121 HEIDELBERG, GERMANY
SN 1863-9933
EI 1863-9941
J9 EUR J TRAUMA EMERG S
JI Eur. J. Trauma Emerg. Surg.
PD FEB
PY 2008
VL 34
IS 1
BP 37
EP 42
DI 10.1007/s00068-007-7038-0
PG 6
WC Emergency Medicine
WE Science Citation Index Expanded (SCI-EXPANDED)
SC Emergency Medicine
GA 314FA
UT WOS:000256793700007
PM 26815489
DA 2022-07-22
ER

PT J
AU Wren, TAL
   Yerby, SA
   Beaupre, GS
   Carter, DR
AF Wren, TAL
   Yerby, SA
   Beaupre, GS
   Carter, DR
TI Influence of bone mineral density, age, and strain rate on the failure
   mode of human Achilles tendons
SO CLINICAL BIOMECHANICS
LA English
DT Article
DE Achilles tendon; rupture; avulsion; failure mode
ID ANTERIOR CRUCIATE LIGAMENT; MEDIAL COLLATERAL LIGAMENT;
   MECHANICAL-PROPERTIES; PATELLAR TENDON; TENSILE; CALCANEUS; FRACTURE;
   RABBITS
AB Objective, To examine the influence of strain rate. bone mineral density, and age in determining the mode by which human Achilles tendons fail.
   Design. Dual-energy X-ray absorptiometry and mechanical testing of excised Achilles tendon-calcaneus specimens.
   Background. The Achilles tendon can fail by tendon rupture or bony avulsion. These injuries are caused by similar loading mechanisms and can present similar symptoms. It is important to understand when each mode of injury is likely to occur so that accurate diagnoses can be made and appropriate treatments selected.
   Methods, Excised human Achilles tendons were loaded to failure at strain rates of 1% s(-1) and 10% s(-1) following dual-energy Xray absorptiometry examination to determine bone mineral density near the tendon insertion. Calcaneal bone mineral density, donor age, and strain rate were compared between specimens that failed by avulsion and those that failed by tendon rupture.
   Results. While strain rate was not observed to affect failure mode, the calcaneal bone mineral density of specimens that failed by avulsion was significantly lower than the bone mineral density of specimens that failed by tendon rupture (P = 0.004). There was a significant decrease in bone mineral density with age (P = 0.004), and the difference in age between the avulsed and ruptured specimens was close to statistical significance (P = 0.058). For the avulsed specimens, there was a significant linear relationship between failure load and bone mineral density squared (P = 0.002). Logistic regression indicated that the effect of age on failure mode is secondary to the primary effect of bone mineral density.
   Conclusions. The avulsions were primarily "premature" failures associated with low bone mineral density. Since bone mineral density decreases with age, older individuals are more likely to experience avulsions while younger individuals are more likely to experience tendon ruptures.
C1 Rehabil Res & Dev Ctr 153, Vet Affairs Hlth Care Syst, Palo Alto, CA 94304 USA.
   Stanford Univ, Dept Mech Engn, Biomech Engn Div, Stanford, CA 94305 USA.
RP Wren, TAL (通讯作者)，Rehabil Res & Dev Ctr 153, Vet Affairs Hlth Care Syst, 3801 Miranda Ave, Palo Alto, CA 94304 USA.
EM wren@stanfordalumni.org
RI Beaupre, Gary S/B-9880-2013; Wren, Tishya/AAS-4196-2021; YAMAMOTO,
   MASAHIRO/F-9094-2011
OI Beaupre, Gary S/0000-0002-6211-7621; YAMAMOTO,
   MASAHIRO/0000-0003-3859-0125
CR *AM AC ORTH SURG, 1998, ORTH KNOWL UPD FOOT, V2, P215
   Carter Dennis R., 1996, P333
   CARTER DR, 1977, J BONE JOINT SURG AM, V59, P954, DOI 10.2106/00004623-197759070-00021
   COUGHLIN MJ, 1999, SURG FOOT ANKLE, P786
   CROWNINSHIELD RD, 1976, J TRAUMA, V16, P99, DOI 10.1097/00005373-197602000-00004
   DANTO MI, 1993, J ORTHOP RES, V11, P58, DOI 10.1002/jor.1100110108
   HAUT RC, 1992, J BIOMECH, V25, P163, DOI 10.1016/0021-9290(92)90273-4
   HAUT RC, 1983, J BIOMECH ENG-T ASME, V105, P296, DOI 10.1115/1.3138422
   HUBBARD RP, 1984, J BIOMECH ENG-T ASME, V106, P144, DOI 10.1115/1.3138471
   JOHNSON GA, 1994, J ORTHOPAED RES, V12, P796, DOI 10.1002/jor.1100120607
   LANDVATER SJ, 1992, CLIN SPORT MED, V11, P741
   Levi N, 1997, J ORTHOP TRAUMA, V11, P61, DOI 10.1097/00005131-199701000-00019
   MICHAEL S, 1993, ARCH EMERG MED, V10, P336
   Nakagawa Y, 1996, EUR J APPL PHYSIOL O, V73, P7, DOI 10.1007/BF00262803
   NOYES FR, 1976, J BONE JOINT SURG AM, V58, P1074, DOI 10.2106/00004623-197658080-00006
   NOYES FR, 1974, J BONE JOINT SURG AM, VA 56, P236, DOI 10.2106/00004623-197456020-00002
   SANDERS R, 1999, SURG FOOT ANKLE, P1422
   SCHECHTMAN H, 1994, P I MECH ENG H, V208, P241, DOI 10.1243/PIME_PROC_1994_208_294_02
   SHARKEY NA, 1995, J BIOMECH, V28, P631, DOI 10.1016/0021-9290(94)00100-I
   WOO SLY, 1986, J ORTHOPAED RES, V4, P133, DOI 10.1002/jor.1100040201
   WOO SLY, 1981, J BIOMECH ENG-T ASME, V103, P293, DOI 10.1115/1.3138295
   WOO SLY, 1990, J ORTHOP RES, V8, P712, DOI 10.1002/jor.1100080513
   Wren TAL, 2000, J BONE MINER RES, V15, P1573, DOI 10.1359/jbmr.2000.15.8.1573
NR 23
TC 41
Z9 41
U1 2
U2 9
PU ELSEVIER SCI LTD
PI OXFORD
PA THE BOULEVARD, LANGFORD LANE, KIDLINGTON, OXFORD OX5 1GB, OXON, ENGLAND
SN 0268-0033
EI 1879-1271
J9 CLIN BIOMECH
JI Clin. Biomech.
PD JUL
PY 2001
VL 16
IS 6
BP 529
EP 534
DI 10.1016/S0268-0033(01)00033-X
PG 6
WC Engineering, Biomedical; Orthopedics; Sport Sciences
WE Science Citation Index Expanded (SCI-EXPANDED)
SC Engineering; Orthopedics; Sport Sciences
GA 459YR
UT WOS:000170279500009
PM 11427296
DA 2022-07-22
ER

PT J
AU Porter, KJ
   Robati, S
   Karia, P
   Portet, M
   Szarko, M
   Amin, A
AF Porter, Ken J.
   Robati, Shibby
   Karia, Priyesh
   Portet, Mark
   Szarko, Matthew
   Amin, Amit
TI An anatomical and cadaveric study examining the risk of sural nerve
   injury in percutaneous Achilles tendon repair using the Achillon device
SO FOOT AND ANKLE SURGERY
LA English
DT Article
DE Achilles tendon; Rupture; Percutaneous repair; Achillon; Sural nerve
ID RUPTURES
AB Background: Percutaneous Achilles tendon repairs are gaining in popularity. This study aims to quantify the risk of sural nerve injury when using the Achillon device.
   Methods: The Achillon device was instrumented into 15 cadaveric specimens and through dissection the rate of sural nerve puncture and the position of the sural nerve in relation to the Achilles tendon was documented.
   Results: The sural nerve was found lateral to the Achilles tendon insertion point over a range of 14.3 mm and crossed the lateral border of the Achilles tendon over a range of 57.7 mm.
   The sural nerve was punctured a total of 6 times and in 4 out of 15 cadaveric specimens (27%). Four out of the 6 punctures occurred when the Achillon device was instrumented distally.
   Conclusions: The sural nerve displays a highly variable anatomical course and there is a risk of puncture during percutaneous Achilles tendon repair using the Achillon device. (C) 2013 European Foot and Ankle Society. Published by Elsevier Ltd. All rights reserved.
C1 [Porter, Ken J.; Karia, Priyesh; Szarko, Matthew; Amin, Amit] St George Hosp, London, England.
   [Robati, Shibby] East Kent Univ Hosp, East Kent, OH USA.
   [Portet, Mark] Royal Surrey Cty Hosp, Guildford, Surrey, England.
RP Porter, KJ (通讯作者)，1 Whateley Close, Guildford GU2 9LW, Surrey, England.
EM kenjporter@hotmail.com
FU Integra
FX Funding for one Achillon (R) device was secured from Integra but they
   were otherwise not involved in the study or publication.
CR Aibinder WR, 2013, FOOT ANKLE INT, V34, P870, DOI 10.1177/1071100712473097
   Assal M, 2002, J BONE JOINT SURG AM, V84A, P161, DOI 10.2106/00004623-200202000-00001
   Calder JDF, 2005, BRIT J SPORT MED, V39, P857, DOI 10.1136/bjsm.2004.017509
   Citak M, 2007, BRIT J SPORT MED, V41, P456, DOI 10.1136/bjsm.2006.031328
   Flavin R, 2007, INJURY, V38, P845, DOI 10.1016/j.injury.2006.10.016
   Garrido Ignacio Martinez, 2010, Foot Ankle Surg, V16, P189, DOI 10.1016/j.fas.2009.10.014
   HASTAD K, 1959, Acta Chir Scand, V116, P251
   Huffard B, 2008, CLIN BIOMECH, V23, P1158, DOI 10.1016/j.clinbiomech.2008.05.007
   Jung HG, 2008, FOOT ANKLE INT, V29, P803, DOI 10.3113/FAI.2008.0803
   LAGERGREN C, 1959, Acta Chir Scand, V116, P491
   LAWRENCE SJ, 1994, FOOT ANKLE INT, V15, P490, DOI 10.1177/107110079401500906
   McMahon Samuel E, 2011, Foot Ankle Surg, V17, P211, DOI 10.1016/j.fas.2010.11.001
   Rippstein Pascal F, 2002, Foot Ankle Clin, V7, P611, DOI 10.1016/S1083-7515(02)00040-2
   Webb J, 2000, FOOT ANKLE INT, V21, P475, DOI 10.1177/107110070002100604
NR 14
TC 29
Z9 33
U1 0
U2 2
PU ELSEVIER SCIENCE BV
PI AMSTERDAM
PA PO BOX 211, 1000 AE AMSTERDAM, NETHERLANDS
SN 1268-7731
EI 1460-9584
J9 FOOT ANKLE SURG
JI Foot Ankle Surg.
PD JUN
PY 2014
VL 20
IS 2
BP 90
EP 93
DI 10.1016/j.fas.2013.11.005
PG 4
WC Orthopedics
WE Science Citation Index Expanded (SCI-EXPANDED)
SC Orthopedics
GA CI8FY
UT WOS:000355006900008
PM 24796825
DA 2022-07-22
ER

PT J
AU Cretnik, A
   Frank, A
AF Cretnik, A
   Frank, A
TI Incidence and outcome of rupture of the Achilles tendon
SO WIENER KLINISCHE WOCHENSCHRIFT
LA English
DT Article
DE Achilles tendon; rupture; incidence; treatment; outcome
ID NON-SURGICAL TREATMENT; FUNCTIONAL POSTOPERATIVE TREATMENT; PERCUTANEOUS
   REPAIR; NONOPERATIVE TREATMENT
AB We determined the incidence of complete rupture of the Achilles tendon in the Maribor region (273,609 inhabitants) between 1991 and 1996. During this period, 116 ruptures were treated at Maribor Teaching Hospital. The average incidence was 7 ruptures per 100,000 inhabitants, with a peak incidence of almost 9 per 100,000. Most injuries (65%) occurred during sports activities, with soccer as the major cause of rupture. The average age of patients was 37 years with a male-to-female ratio of 18:1.
   All patients underwent open surgical repair of the ruptured Achilles tendon, with a minimum follow-up of two years. 19.8% of cases developed complications and in 10.4% of these the complications were major. 1.9% of patients sustained a re-rupture. The mean AOFAS score was 96 points. The patients were subjectively very satisfied with their treatment in 88% of cases. Good functional results with a return to the usual pre-injury activities were achieved in 96% of patients.
C1 Maribor Teaching Hosp, Dept Traumatol, SLO-2000 Maribor, Slovenia.
RP Cretnik, A (通讯作者)，Maribor Teaching Hosp, Dept Traumatol, Ljubljanska 5, SLO-2000 Maribor, Slovenia.
EM andrej.cretnik@guest.arnes.si
CR BRADLEY JP, 1990, AM J SPORT MED, V18, P188, DOI 10.1177/036354659001800213
   Buchgraber A, 1997, CLIN ORTHOP RELAT R, P113
   CARTER TR, 1992, AM J SPORT MED, V20, P459, DOI 10.1177/036354659202000417
   CETTI R, 1993, AM J SPORT MED, V21, P791, DOI 10.1177/036354659302100606
   Cretnik A, 2000, MED SCI SPORT EXER, V32, P16
   DeLee JC., 1994, ORTHOPAEDIC SPORTS M
   FITZGIBBONS RE, 1993, AM J SPORT MED, V21, P724, DOI 10.1177/036354659302100516
   INGLIS AE, 1976, J BONE JOINT SURG AM, V58, P990, DOI 10.2106/00004623-197658070-00015
   INGLIS AE, 1981, CLIN ORTHOP RELAT R, V156, P160
   KITAOKA HB, 1994, FOOT ANKLE INT, V15, P349, DOI 10.1177/107110079401500701
   KOSANOVIC M, 1994, ARCH ORTHOP TRAUM SU, V113, P177, DOI 10.1007/BF00441827
   LEA RB, 1972, J BONE JOINT SURG AM, VA 54, P1398, DOI 10.2106/00004623-197254070-00002
   Leppilahti J, 1996, ACTA ORTHOP SCAND, V67, P277, DOI 10.3109/17453679608994688
   LEPPILAHTI J, 1998, CLIN ORTHOP RELAT R, V346, P152
   Lo IKY, 1997, CLIN J SPORT MED, V7, P207, DOI 10.1097/00042752-199707000-00010
   MA GWC, 1977, CLIN ORTHOP RELAT R, P247
   Maffulli N, 1999, J BONE JOINT SURG AM, V81A, P1019, DOI 10.2106/00004623-199907000-00017
   MARTI R, 1974, HELV CHIR ACTA, V41, P293
   McComis GP, 1997, J BONE JOINT SURG AM, V79A, P1799, DOI 10.2106/00004623-199712000-00005
   Moller A, 1996, ACTA ORTHOP SCAND, V67, P479, DOI 10.3109/17453679608996672
   NISTOR L, 1981, J BONE JOINT SURG AM, V63, P394, DOI 10.2106/00004623-198163030-00012
   Reilmann H, 1996, UNFALLCHIRURG, V99, P576
   SCHONBAUER HR, 1986, WIEN KLIN WOCHENSCHR, V98, P3
   SOLVEBORN SA, 1994, AM J SPORT MED, V22, P607, DOI 10.1177/036354659402200507
   THERMANN H, 1995, UNFALLCHIRURG, V98, P21
   THOMPSON T C, 1962, Acta Orthop Scand, V32, P461, DOI 10.3109/17453676208989608
   WASHBURN SD, 1992, CLIN ORTHOP RELAT R, V279, P237
   Webb JM, 1999, J BONE JOINT SURG BR, V81B, P877, DOI 10.1302/0301-620X.81B5.9784
   WILLS CA, 1986, CLIN ORTHOP RELAT R, V207, P156
   Winter E, 1998, ARCH ORTHOP TRAUM SU, V117, P364, DOI 10.1007/s004020050267
NR 30
TC 22
Z9 23
U1 0
U2 3
PU SPRINGER WIEN
PI WIEN
PA SACHSENPLATZ 4-6, PO BOX 89, A-1201 WIEN, AUSTRIA
SN 0043-5325
EI 1613-7671
J9 WIEN KLIN WOCHENSCHR
JI Wien. Klin. Wochen.
PY 2004
VL 116
SU 2
BP 33
EP 38
PG 6
WC Medicine, General & Internal
WE Science Citation Index Expanded (SCI-EXPANDED)
SC General & Internal Medicine
GA 841FB
UT WOS:000222913600009
PM 15506308
DA 2022-07-22
ER

PT J
AU Khalil, LS
   Jildeh, TR
   Tramer, JS
   Abbas, MJ
   Hessburg, L
   Mehran, N
   Okoroha, KR
AF Khalil, Lafi S.
   Jildeh, Toufic R.
   Tramer, Joseph S.
   Abbas, Muhammad J.
   Hessburg, Luke
   Mehran, Nima
   Okoroha, Kelechi R.
TI Effect of Achilles Tendon Rupture on Player Performance and Longevity in
   National Basketball Association Players
SO ORTHOPAEDIC JOURNAL OF SPORTS MEDICINE
LA English
DT Article
DE Basketball; NBA; Achilles tendon rupture; performance; Achilles tendon;
   general sports trauma
AB Background:
   National Basketball Association (NBA) players who return to sport (RTS) after Achilles tendon rupture have been reported to have poor outcomes.
   Purpose:
   To evaluate the effect of Achilles tendon ruptures on player performance and career longevity in NBA athletes.
   Study Design:
   Cohort study; Level of evidence, 3.
   Methods:
   NBA players who sustained Achilles tendon ruptures between 1970 and 2019 were identified using publicly available resources and were matched 1:1 to a healthy control group by age, position, height, and body mass index. Demographic characteristics, player utilization (games and minutes), and performance efficiency rating (PER) were documented for all athletes. The season of Achilles tendon rupture was set as the index year, and statistical analysis compared postindex versus preindex data both acutely and in the long term. Percentages relative to preoperative values were calculated to compare the injured and control groups in a standardized fashion.
   Results:
   Of 47 players, 34 (72.3%) with Achilles tendon ruptures returned to play at the NBA level after surgical intervention. A total of 7 players were excluded from the study. No differences were found in demographic characteristics or PER (2 years before injury) between the remaining 27 players and matched controls. The injured players had significantly shorter careers compared with control players (3.1 +/- 2.3 vs 5.8 +/- 3.5 seasons, respectively; P < .05). Injured players demonstrated significant declines in games per season (GPS), minutes per game (MPG), and PER at 1 year and 3 years after RTS compared with preindex baseline (P < .05). Injured players, compared with control players, had reduced relative percentages of games started (GS) (50% vs 125%, respectively), MPG (83% vs 103%), and PER (80% vs 96%) at 1 year after return (P < .05), but reductions at extended 3-year follow-up were seen only in GPS (71% vs 91%) and GS (39% vs 99%) (P < .05).
   Conclusion:
   Our study found that 72.3% of NBA players returned to play after Achilles tendon repair, but they had shorter careers compared with uninjured controls. Players returning from Achilles tendon repairs had decreased game utilization and performance at all time points relative to their individual preindex baseline. However, for the injured players when compared with controls, game utilization but not performance was found to be decreased at 3-year follow-up.
C1 [Khalil, Lafi S.; Jildeh, Toufic R.; Tramer, Joseph S.; Abbas, Muhammad J.; Hessburg, Luke; Mehran, Nima; Okoroha, Kelechi R.] Henry Ford Hosp, Dept Orthopaed Surg, 2799 W Grand Blvd, Detroit, MI 48202 USA.
   [Abbas, Muhammad J.; Hessburg, Luke] Wayne State Univ, Sch Med, Detroit, MI USA.
   [Mehran, Nima] Kaiser Permanente Los Angeles Med Ctr, Los Angeles, CA USA.
RP Khalil, LS (通讯作者)，Henry Ford Hosp, Dept Orthopaed Surg, 2799 W Grand Blvd, Detroit, MI 48202 USA.
EM lskhalil@gmail.com
OI Khalil, Lafi/0000-0001-5924-7639
CR Amin NH, 2013, AM J SPORT MED, V41, P1864, DOI 10.1177/0363546513490659
   Cukelj F, 2015, INJURY, V46, pS137, DOI 10.1016/j.injury.2015.10.070
   Gwynne-Jones DP, 2011, FOOT ANKLE INT, V32, P337, DOI 10.3113/FAI.2011.0337
   Harris JD, 2013, SPORTS HEALTH, V5, P562, DOI 10.1177/1941738113495788
   Jildeh TR, 2019, AM J SPORT MED, V47, P2717, DOI 10.1177/0363546519864581
   Keller RA, 2017, J SHOULDER ELB SURG, V26, P369, DOI 10.1016/j.jse.2016.11.045
   Keller RA, 2017, J SHOULDER ELB SURG, V26, P288, DOI 10.1016/j.jse.2016.11.008
   Kujala UM, 2005, CLIN J SPORT MED, V15, P133, DOI 10.1097/01.jsm.0000165347.55638.23
   Lemme NJ, 2019, AM J SPORT MED, V47, P2360, DOI 10.1177/0363546519858609
   Lu YN, 2020, J SHOULDER ELB SURG, V29, P50, DOI 10.1016/j.jse.2019.05.035
   Maffulli N, 2011, FOOT ANKLE INT, V32, P9, DOI 10.3113/FAI.2011.0009
   Marshall NE, 2018, ARTHROSCOPY, V34, P473, DOI 10.1016/j.arthro.2017.08.298
   Minhas SV, 2016, AM J SPORT MED, V44, P1056, DOI 10.1177/0363546515623028
   Okoroha KR, 2018, J SHOULDER ELB SURG, V27, P17, DOI 10.1016/j.jse.2017.07.027
   Okoroha KR, 2017, ORTHOP J SPORTS MED, V5, DOI 10.1177/2325967117698788
   Ryan J., 2015, THESIS HARVARD COLL
   Schipper ON, 2018, FOOT ANKLE INT, V39, P664, DOI 10.1177/1071100718758257
   Starkey C, 2000, J ATHL TRAINING, V35, P161
   Trofa DP, 2017, AM J SPORT MED, V45, P2864, DOI 10.1177/0363546517713001
   Wright S., 2015, ATHL TRAIN SPORTS HL, V11, P166
NR 20
TC 3
Z9 3
U1 1
U2 2
PU SAGE PUBLICATIONS INC
PI THOUSAND OAKS
PA 2455 TELLER RD, THOUSAND OAKS, CA 91320 USA
EI 2325-9671
J9 ORTHOP J SPORTS MED
JI Orthop. J. Sports Med.
PD NOV
PY 2020
VL 8
IS 11
AR 2325967120966041
DI 10.1177/2325967120966041
PG 8
WC Orthopedics; Sport Sciences
WE Science Citation Index Expanded (SCI-EXPANDED)
SC Orthopedics; Sport Sciences
GA PB7BM
UT WOS:000596472200001
PM 33294475
OA gold, Green Published
DA 2022-07-22
ER

PT J
AU Yasuda, T
   Kinoshita, M
   Okuda, R
AF Yasuda, Toshito
   Kinoshita, Mitsuo
   Okuda, Ryuzo
TI Reconstruction of chronic Achilles tendon rupture with the use of
   interposed tissue between the stumps
SO AMERICAN JOURNAL OF SPORTS MEDICINE
LA English
DT Article
DE Achilles tendon; neglected rupture; reconstruction; histologic
   examination; magnetic resonance imaging
ID NEGLECTED RUPTURE; CALCANEAL TENDON; REPAIR
AB Background: The gap between the tendon stumps in chronic Achilles tendon rupture has reportedly been filled with interposed scar tissue. In the authors' clinical experience, this interposed tissue is often thick and resists tension, so they considered it was possible to use the interposed tissue for reconstruction of Achilles tendon rupture.
   Hypothesis: Scar tissue interposed between the tendon stumps has the capacity to form tendon-like repair tissue in patients with chronic Achilles tendon rupture.
   Study Design: Case series; Level of evidence, 4.
   Methods: Six patients with chronic rupture of the Achilles tendon underwent tendon reconstruction with the use of interposed tissue between the stumps. The average time from the primary injury to surgery was 22 weeks (range, 9 to 30 weeks). Preoperative magnetic resonance imaging (MRI), histology of the interposed tissue, and clinical results were evaluated. The average postoperative follow-up period was 31 months (range, 24 to 43 months).
   Results: Preoperative T2-weighted MRI in all cases revealed that chronically ruptured Achilles tendons were thickened and fusiform-shaped with diffuse intratendinous high-signal alterations throughout. Longitudinal high-signal bands were seen throughout the tendon, except at the musculotendinous junction and insertion on the calcaneus. Histologically, scar tissue interposed between the tendon stumps consisted of dense collagen fibers, and degenerative changes were not seen. After surgery, no patient had difficulty in walking or stair climbing, and all were able to perform a single-limb toe raise. The mean preoperative and postoperative American Orthopaedic Foot and Ankle Society (AOFAS) ankle-hindfoot scores were 88.2 and 98.3 points, respectively; the difference was statistically significant (P = .0277).
   Conclusion: Interposed tissue between the tendon stumps is suitable for repair of chronic Achilles tendon rupture if preoperative MRI shows a thickened fusiform-shaped Achilles tendon with diffuse intratendinous high-signal alterations throughout.
C1 Osaka Med Coll, Dept Orthoped Surg, Osaka 5698686, Japan.
RP Yasuda, T (通讯作者)，Osaka Med Coll, Dept Orthoped Surg, 2-7 Daigaku Machi, Osaka 5698686, Japan.
EM ort028@poh.osaka-med.ac.jp
CR BARNES MJ, 1986, J BONE JOINT SURG BR, V68, P121, DOI 10.1302/0301-620X.68B1.3941128
   BESKIN JL, 1987, AM J SPORT MED, V15, P1, DOI 10.1177/036354658701500101
   BOSWORTH DM, 1956, J BONE JOINT SURG AM, V38, P111, DOI 10.2106/00004623-195638010-00011
   BUGG EI, 1968, CLIN ORTHOP RELAT R, P73
   GABEL S, 1994, FOOT ANKLE INT, V15, P512, DOI 10.1177/107110079401500912
   HOWARD CB, 1984, J BONE JOINT SURG BR, V66, P206, DOI 10.1302/0301-620X.66B2.6707056
   KANNUS P, 1991, J BONE JOINT SURG AM, V73A, P1507, DOI 10.2106/00004623-199173100-00009
   Karjalainen PT, 1997, AM J SPORT MED, V25, P164, DOI 10.1177/036354659702500204
   Kissel C G, 1994, J Foot Ankle Surg, V33, P46
   Lee YS, 2005, ORTHOPEDICS, V28, P647, DOI 10.3928/0147-7447-20050701-10
   Leppilahti J, 2000, FOOT ANKLE INT, V21, P330, DOI 10.1177/107110070002100410
   Leslie Hamish D H, 2005, Foot Ankle Clin, V10, P357, DOI 10.1016/j.fcl.2005.01.009
   Maffulli N, 1999, J BONE JOINT SURG AM, V81A, P1019, DOI 10.2106/00004623-199907000-00017
   MANDELBAUM BR, 1995, AM J SPORT MED, V23, P392, DOI 10.1177/036354659502300403
   MANN RA, 1991, J BONE JOINT SURG AM, V73A, P214, DOI 10.2106/00004623-199173020-00009
   Miskulin Mladen, 2005, J Foot Ankle Surg, V44, P49, DOI 10.1053/j.jfas.2004.11.003
   Myerson M S, 1999, Instr Course Lect, V48, P219
   Porter DA, 1997, FOOT ANKLE INT, V18, P557, DOI 10.1177/107110079701800905
   Shalabi A, 2001, ACTA RADIOL, V42, P269, DOI 10.1080/028418501127346819
   Us AK, 1997, ARCH ORTHOP TRAUM SU, V116, P408
   Wapner K L, 1993, Foot Ankle, V14, P443
   Yasuda T, 2000, ACTA ORTHOP SCAND, V71, P69, DOI 10.1080/00016470052943937
NR 22
TC 27
Z9 32
U1 0
U2 1
PU SAGE PUBLICATIONS INC
PI THOUSAND OAKS
PA 2455 TELLER RD, THOUSAND OAKS, CA 91320 USA
SN 0363-5465
EI 1552-3365
J9 AM J SPORT MED
JI Am. J. Sports Med.
PD APR
PY 2007
VL 35
IS 4
BP 582
EP 588
DI 10.1177/0363546506295939
PG 7
WC Orthopedics; Sport Sciences
WE Science Citation Index Expanded (SCI-EXPANDED)
SC Orthopedics; Sport Sciences
GA 155PJ
UT WOS:000245590100009
PM 17293468
DA 2022-07-22
ER

PT J
AU Yasuhara, T
   Kuwahara, K
   Sasada, S
   Toyoshima, A
   Morimoto, J
   Kin, K
   Manabe, H
   Miyoshi, Y
   Kusumegi, A
   Takahashi, Y
   Ito, K
   Date, I
AF Yasuhara, Takao
   Kuwahara, Ken
   Sasada, Susumu
   Toyoshima, Atsuhiko
   Morimoto, Jun
   Kin, Kyohei
   Manabe, Hiroaki
   Miyoshi, Yasuyuki
   Kusumegi, Akira
   Takahashi, Yuichi
   Ito, Kiyoshi
   Date, Isao
TI Surgery in the Standing Position by a Surgeon with Achilles Tendon
   Rupture
SO ACTA MEDICA OKAYAMA
LA English
DT Article
DE Achilles tendon rupture; electromyogram; genuflection; injured leg;
   standing position
AB Unexpected injuries can have a profound effect on a surgeon's performance and thus on patients and surgical departments. Here we describe a technique for performing surgery in the standing position, as done by a surgeon with an Achilles tendon rupture. During his prescribed 45-day non-weight-bearing period for the left ankle after surgery for an Achilles tendon rupture, the surgeon was able to participate in 15 surgeries as an operator or assistant, due to his use of a combination of injured-leg genuflection on a stool and a 'Surgical Body Support' device. Similarly injured surgeons may benefit from such support.
C1 [Yasuhara, Takao; Kuwahara, Ken; Sasada, Susumu; Toyoshima, Atsuhiko; Morimoto, Jun; Kin, Kyohei; Manabe, Hiroaki; Miyoshi, Yasuyuki; Kusumegi, Akira; Date, Isao] Okayama Univ, Grad Sch Med Dent & Pharmaceut Sci, Dept Neurol Surg, Okayama 7008558, Japan.
   [Takahashi, Yuichi] Shinkomonfi Hosp, Dept Spinal Surg, Kitakyushu, Fukuoka 8000057, Japan.
   [Ito, Kiyoshi] Shinshu Univ, Dept Neurosurg, Matsumoto, Nagano 3908621, Japan.
RP Yasuhara, T (通讯作者)，Okayama Univ, Grad Sch Med Dent & Pharmaceut Sci, Dept Neurol Surg, Okayama 7008558, Japan.
EM tyasu37@cc.okayama-u.ac.jp
OI kin, kyohei/0000-0002-5445-5647
CR Ito K, 2015, NEUROSURG REV, V38, P731, DOI 10.1007/s10143-015-0633-3
   Kikuta KI, 2004, ACTA NEUROCHIR, V146, P1119, DOI 10.1007/s00701-004-0337-x
   Takami T, 2015, J CRANIOVERTEBRAL JU, V6, P173, DOI 10.4103/0974-8237.167862
NR 3
TC 0
Z9 0
U1 0
U2 0
PU OKAYAMA UNIV MED SCHOOL
PI OKAYAMA
PA EDITORIAL OFFICE, ACTA MEDICA OKAYAMA OKAYAMA UNIVERSITY MEDICAL SCHOOL
   2-5-1 SHIKATA-CHO, KITA-KU, OKAYAMA, 700-8558, JAPAN
SN 0386-300X
J9 ACTA MED OKAYAMA
JI Acta Med. Okayama
PD DEC
PY 2016
VL 70
IS 6
BP 493
EP 496
PG 4
WC Medicine, Research & Experimental
WE Science Citation Index Expanded (SCI-EXPANDED)
SC Research & Experimental Medicine
GA EQ6NY
UT WOS:000398200200009
PM 28003675
DA 2022-07-22
ER

PT J
AU Miller, TL
   Shemory, S
AF Miller, Timothy L.
   Shemory, Scott
TI Insertional Achilies Tendon Injuries in the Athlete
SO OPERATIVE TECHNIQUES IN SPORTS MEDICINE
LA English
DT Article
DE Achilles; tendon; tendonitis; tendinosis; insertional; rupture
ID CHRONIC ACHILLES TENDINOPATHY; PLATELET-RICH PLASMA; SURGICAL-TREATMENT;
   TENDINOSIS; MANAGEMENT; DISORDERS; EXERCISE; ANATOMY
AB Insertional Achilles tendon injuries are common among running and jumping athletes of all age ranges. Injuries to this site are often due to an overuse mechanism and are especially common in individuals who continue to participate in sports into middle age and beyond. They present with a broad spectrum of severity and chronicity from mild acute tendonitis to partial or complete tendon rupture. The causes of injuries and conditions of the distal Achilles tendon are often multifactorial and may include biomechanical factors, training errors, and medications that predispose the tendon to degeneration or tear. It is this variability of cause, severity, and chronicity coupled with many athletes' unwillingness to alter or curtail the inciting activity that makes determination of an ideal treatment option a challenge. Nonsurgical management, which may consist of oral nonsteroidal anti-inflammatory medications, physical therapy, shoewear modification, and training alterations, has been the mainstay of treatment for patients with tendon inflammation and early degeneration. However, when injuries become more chronic and debilitating or when complete rupture occurs at the calcaneal insertion, debridement to remove degenerative tissue and repair using a calcaneal anchor-based suture bridge are required. Augmentation of the repair with transfer of the flexor hallucis longus tendon may be required if more than 50% of the tendon has been damaged or if extensive debridement leads to inadequate tendon length. Recently, biological treatment options as well as less invasive procedures have become available for stimulating tendon healing. (C) 2017 Elsevier Inc. All rights reserved.
C1 [Miller, Timothy L.; Shemory, Scott] Ohio State Univ, Dept Orthopaed Surg, Wexner Med Ctr, Columbus, OH 43210 USA.
RP Miller, TL (通讯作者)，CarePoint Gahanna, 920 N Hamilton Rd,Suite 600, Gahanna, OH 43230 USA.
EM timothy.miller@osumc.edu
CR Alfredson H, 2003, KNEE SURG SPORT TR A, V11, P334, DOI 10.1007/s00167-003-0391-6
   Ballal MS, 2014, BONE JOINT J, V96B, P1344, DOI 10.1302/0301-620X.96B10.33771
   Ceccarelli F, 2007, CLIN ORTHOP RELAT R, P188, DOI 10.1097/BLO.0b013e3180396f07
   Chen TM, 2009, CLIN ANAT, V22, P377, DOI 10.1002/ca.20758
   Clancy W G Jr, 1976, Am J Sports Med, V4, P46, DOI 10.1177/036354657600400202
   Cox JT, 2014, AM J SPORT MED, V42, P2727, DOI 10.1177/0363546514549004
   de Oliveira LP, 2015, FOOD CHEM TOXICOL, V77, P50, DOI 10.1016/j.fct.2014.12.014
   de Vos RJ, 2010, JAMA-J AM MED ASSOC, V303, P144, DOI 10.1001/jama.2009.1986
   Den Hartog BD, 2009, FOOT ANKLE CLIN, V14, P639, DOI 10.1016/j.fcl.2009.08.005
   DeOrio MJ, 2008, FOOT ANKLE INT, V29, P542, DOI 10.3113/FAI.2008.0542
   Doral MN, 2010, OPEN ACCESS J SPORTS, V1, P233, DOI 10.2147/OAJSM.S10670
   Elias I, 2009, FOOT ANKLE INT, V30, P197, DOI 10.3113/FAI.2009.0197
   Fredberg U, 2002, AM J SPORT MED, V30, P488, DOI 10.1177/03635465020300040701
   Gaida JE, 2010, BMC MUSCULOSKEL DIS, V11, DOI 10.1186/1471-2474-11-41
   Greenhagen Robert M, 2013, Foot Ankle Spec, V6, P185, DOI 10.1177/1938640012473150
   Guelfi M, 2015, FOOT ANKLE SURG, V21, P178, DOI 10.1016/j.fas.2014.11.005
   Hahn F, 2008, FOOT ANKLE INT, V29, P794, DOI 10.3113/FAI.2008.0794
   Heckman DS, 2009, AM J SPORT MED, V37, P1223, DOI 10.1177/0363546509335947
   Hegewald KW, 2016, J FOOT ANKLE SURG, V55, P166, DOI 10.1053/j.jfas.2015.08.001
   Holmes GB, 2006, FOOT ANKLE INT, V27, P952, DOI 10.1177/107110070602701115
   Irwin TA, 2010, FOOT ANKLE INT, V31, P933, DOI 10.3113/FAI.2010.0933
   Johansson K, 2016, MLTJ-MUSCLE LIGAMENT, V6, P111, DOI 10.11138/mltj/2016.6.1.111
   Kang S, 2012, FOOT ANKLE INT, V33, P487, DOI 10.3113/FAI.2012.0487
   Kedia M, 2014, INT J SPORTS PHYS TH, V9, P488
   Knobloch K, 2006, AM J SPORT MED, V34, P92, DOI 10.1177/0363546505278705
   KOMI PV, 1992, CLIN SPORT MED, V11, P521
   Langberg H, 2007, SCAND J MED SCI SPOR, V17, P61, DOI 10.1111/j.1600-0838.2006.00522.x
   Legerlotz K, 2012, RHEUMATOLOGY, V51, P1161, DOI 10.1093/rheumatology/kes002
   Lyman J, 2004, AM J SPORT MED, V32, P457, DOI 10.1177/0095399703258621
   Martin RL, 2005, FOOT ANKLE INT, V26, P691, DOI 10.1177/107110070502600905
   McCormack JR, 2016, SPORTS HEALTH, V8, P230, DOI 10.1177/1941738116631498
   Monto RR, 2012, FOOT ANKLE INT, V33, P379, DOI 10.3113/FAI.2012.0379
   Munteanu SE, 2011, J FOOT ANKLE RES, V4, DOI 10.1186/1757-1146-4-15
   Murphy K, 2013, SPORTS MED, V43, P1131, DOI 10.1007/s40279-013-0093-2
   Paavola M, 2002, J BONE JOINT SURG AM, V84A, P2062, DOI 10.2106/00004623-200211000-00024
   Phisitkul P, 2012, CURR REV MUSCULOSKE, V5, P156, DOI 10.1007/s12178-012-9115-1
   Puddu G, 1976, Am J Sports Med, V4, P145, DOI 10.1177/036354657600400404
   Reddy SS, 2009, J AM ACAD ORTHOP SUR, V17, P3, DOI 10.5435/00124635-200901000-00002
   Rigby RB, 2013, J FOOT ANKLE SURG, V52, P575, DOI 10.1053/j.jfas.2012.11.004
   Roche AJ, 2013, BONE JOINT J, V95B, P1299, DOI 10.1302/0301-620X.95B10.31881
   Rousseau R, 2015, KNEE SURG SPORT TR A, V23, P2494, DOI 10.1007/s00167-014-2986-5
   Schepsis AA, 2002, AM J SPORT MED, V30, P287, DOI 10.1177/03635465020300022501
   Schon LC, 2013, J BONE JOINT SURG AM, V95A, P54, DOI 10.2106/JBJS.K.00970
   Scott LA, 2015, SPORTS MED, V45, P95, DOI 10.1007/s40279-014-0237-z
   Sundararajan PP, 2014, J FOOT ANKLE SURG, V53, P147, DOI 10.1053/j.jfas.2013.12.009
   Uquillas CA, 2015, J BONE JOINT SURG AM, V97A, P1187, DOI 10.2106/JBJS.O.00002
   van Dijk CN, 2011, KNEE SURG SPORT TR A, V19, P835, DOI 10.1007/s00167-010-1374-z
   Verrall G, 2011, FOOT ANKLE INT, V32, P843, DOI 10.3113/FAI.2011.0843
   Webb J, 2000, FOOT ANKLE INT, V21, P475, DOI 10.1177/107110070002100604
   Weinfeld SB, 2014, MED CLIN N AM, V98, P331, DOI 10.1016/j.mcna.2013.11.005
   Yeo A, 2016, KNEE SURG SPORT TR A, V24, P2112, DOI 10.1007/s00167-014-3458-7
NR 51
TC 2
Z9 2
U1 0
U2 19
PU W B SAUNDERS CO-ELSEVIER INC
PI PHILADELPHIA
PA 1600 JOHN F KENNEDY BOULEVARD, STE 1800, PHILADELPHIA, PA 19103-2899 USA
SN 1060-1872
EI 1557-9794
J9 OPER TECHN SPORT MED
JI Oper. Tech. Sports Med.
PD JUN
PY 2017
VL 25
IS 2
BP 87
EP 98
DI 10.1053/j.otsm.2017.03.006
PG 12
WC Sport Sciences; Surgery
WE Science Citation Index Expanded (SCI-EXPANDED)
SC Sport Sciences; Surgery
GA EZ7ZU
UT WOS:000404945000007
DA 2022-07-22
ER

PT J
AU Dong, ZJ
   Qiu, B
   Pan, Y
   Wu, SZ
   Hong, X
   Liu, FY
AF Dong, Zhijun
   Qiu, Bing
   Pan, Yuan
   Wu, Shengzhong
   Hong, Xiao
   Liu, Fuyao
TI Improved Krackow Method Combined with Unilateral Mattress Suture for
   Treating Recent Achilles Tendon Rupture
SO JCPSP-JOURNAL OF THE COLLEGE OF PHYSICIANS AND SURGEONS PAKISTAN
LA English
DT Article
DE Achilles tendon rupture; Improved Krackow method; Unilateral mattress
   suture
AB The report describes the clinical efficacy of improved Krackow method combined with unilateral mattress suture for treating fresh Achilles tendon rupture. The clinical data of 19 patients, 18 males (94.7%) and one female (5.3%), with recent Achilles tendon rupture undergoing the above procedure at Department of Orthopaedics, Orthopaedic Hospital of Guizhou Province, Guiyang, China, from May 2013 to May 2016, were retrospectively analysed. The enrolled patients were aged from 19 to 56 years. The injury time to operation time was 1-10 days with an average of 2.9 days. Postoperative curative effectiveness was evaluated according to the American Orthopedic Foot and Ankle Society (AOFAS) Ankle-Hindfoot postoperative function score. All patients remained under an OPD follow-up from 7 to 18 months (average of 11.8 months). Among them, incision of 16 patients healed by primary intention. Incision edge of three patients showed darkening and effusion, without recovery after change of dressings. No sural nerve injury or re-rupture of Achilles tendon were noted and the AOFAS score was 97.4. Improved Krackow method with unilateral mattress suture has good curative effectiveness to skin and tendon, reducing the risk of Achilles tendon re-rupture, skin infections, and necrosis.
   The report describes the clinical efficacy of improved Krackow method combined with unilateral mattress suture for treating fresh Achilles tendon rupture. The clinical data of 19 patients, 18 males (94.7%) and one female (5.3%), with recent Achilles tendon rupture undergoing the above procedure at Department of Orthopaedics, Orthopaedic Hospital of Guizhou Province, Guiyang, China, from May 2013 to May 2016, were retrospectively analysed. The enrolled patients were aged from 19 to 56 years. The injury time to operation time was 1-10 days with an average of 2.9 days. Postoperative curative effectiveness was evaluated according to the American Orthopedic Foot and Ankle Society (AOFAS) Ankle-Hindfoot postoperative function score. All patients remained under an OPD follow-up from 7 to 18 months (average of 11.8 months). Among them, incision of 16 patients healed by primary intention. Incision edge of three patients showed darkening and effusion, without recovery after change of dressings. No sural nerve injury or re-rupture of Achilles tendon were noted and the AOFAS score was 97.4. Improved Krackow method with unilateral mattress suture has good curative effectiveness to skin and tendon, reducing the risk of Achilles tendon re-rupture, skin infections, and necrosis.
C1 [Dong, Zhijun; Qiu, Bing; Pan, Yuan; Wu, Shengzhong; Hong, Xiao; Liu, Fuyao] Orthopaed Hosp Guizhou Prov, Dept Orthopaed, 25 North Shachong Rd, Guiyang, Guizhou, Peoples R China.
RP Liu, FY (通讯作者)，Orthopaed Hosp Guizhou Prov, Dept Orthopaed, 25 North Shachong Rd, Guiyang, Guizhou, Peoples R China.
EM ecw07r@163.com
CR Coughlin MJ, 1999, SURG FOOT ANKLE, P835
   Jun C, 2012, ORTHOP J CHIN, V20, P945
   Labib SA, 2000, ORTHOPEDIC BOARD REV
   Leppilahti J, 1996, ACTA ORTHOP SCAND, V67, P277, DOI 10.3109/17453679608994688
   Longo UG, 2013, FOOT ANKLE CLIN, V18, P319, DOI 10.1016/j.fcl.2013.02.009
   Lynch Richard M, 2004, Accid Emerg Nurs, V12, P149, DOI 10.1016/j.aaen.2003.11.004
   [徐海林 Xu Hailin], 2012, [中华创伤骨科杂志, Chinese Journal of Orthopaedic Trauma], V14, P36
   Yongping Y, 2006, CHIN J BONE JOINT IN, V21, P151
NR 8
TC 0
Z9 0
U1 0
U2 1
PU COLL PHYSICIANS & SURGEONS PAKISTAN
PI KARACHI
PA SEVENTH CENTRAL ST, DEFENCE HOUSING AUTHORITY, KARACHI, 75500, PAKISTAN
SN 1022-386X
EI 1681-7168
J9 JCPSP-J COLL PHYSICI
JI JCPSP-J. Coll. Physicians Surg.
PD SEP
PY 2018
VL 28
IS 9
BP 711
EP 713
DI 10.29271/jcpsp.2018.09.711
PG 3
WC Medicine, General & Internal
WE Science Citation Index Expanded (SCI-EXPANDED)
SC General & Internal Medicine
GA GR9UR
UT WOS:000443119200014
PM 30158040
DA 2022-07-22
ER

PT J
AU Sen, B
   Guler, S
   Cecen, B
   Kumtepe, E
   Bagriyanik, A
   Ozkal, S
   Ozcan, MA
   Ozsan, H
   Sanli, N
   Tatari, MH
AF Sen, Baran
   Guler, Serkan
   Cecen, Berivan
   Kumtepe, Erdem
   Bagriyanik, Alper
   Ozkal, Sermin
   Ozcan, M. Ali
   Ozsan, Hayri
   Sanli, Namik
   Tatari, M. Hasan
TI The Effect of Autologous Platelet Rich Plasma in the Treatment of
   Achilles Tendon Ruptures: An Experimental Study on Rabbits
SO BALKAN MEDICAL JOURNAL
LA English
DT Article; Proceedings Paper
CT 12th Congress of the
   Turkish-Society-of-Sports-Traumatology-Arthroscopy-and-Knee-Surgery
CY SEP 23-27, 2014
CL Izmir, TURKEY
SP Turkish Soc Sports Traumatol Arthroscopy & Knee Surg
DE Achilles tendon; electron microscopy; platelet-rich plasma; tendon
   healing; tendon rupture
ID GROWTH-FACTOR; INJECTION; INJURY; ANGIOGENESIS; LIGAMENT; REPAIR; CELLS;
   BONE; PRP
AB Background: Achilles tendon ruptures are characterized by a long recovery period, high re-rupture rate and late return to work. To overcome these difficulties and augment tendon repair, many agents have been used.
   Aims: To determine the effect of autologous platelet rich plasma (PRP) in the treatment of Achilles tendon ruptures in rabbits.
   Study Design: Animal experimentation.
   Methods: The study included 14 New Zealand albino rabbits that were divided randomly into 2 groups, A and B, each containing seven rabbits. On day zero, all 28 Achilles tendons were tenotomized and repaired. In group A, the tendons were injected with PRP post-surgery, whereas those in group B were left untreated. On day 28, the right tendons in both groups were examined histopathologically via both light and electron microscopy, and the left tendons were subjected to biomechanical testing.
   Results: The histological and biomechanical findings in both light and electron microscopy in group A were better than those in group B, but the difference was not significant. According to Tang's scale, the mean value in Group A was 3.57, while it was 3.0 in Group B. The mean value of Group A for the length of collagen bands was 48.09 nm while the mean value of Group B was 46.58 nm (p=0.406). In biomechanical tests, although stiffness values were higher in group A, the difference between groups was not significant. In addition, maximum load values did not differ between groups A and B.
   Conclusion: PRP had no effect on the healing process 28 days post-Achilles tendon rupture.
C1 [Sen, Baran; Guler, Serkan; Tatari, M. Hasan] Dokuz Eylul Univ, Fac Med, Dept Orthopaed & Traumatol, Izmir, Turkey.
   [Cecen, Berivan; Kumtepe, Erdem] Dokuz Eylul Univ, Inst Hlth Sci, Dept Biomech, Izmir, Turkey.
   [Bagriyanik, Alper] Dokuz Eylul Univ, Fac Med, Dept Histol & Embriol, Izmir, Turkey.
   [Ozkal, Sermin] Dokuz Eylul Univ, Fac Med, Dept Pathol, Izmir, Turkey.
   [Ozcan, M. Ali; Ozsan, Hayri; Sanli, Namik] Dokuz Eylul Univ, Fac Med, Dept Hematol, Izmir, Turkey.
RP Tatari, MH (通讯作者)，Dokuz Eylul Univ, Fac Med, Dept Orthopaed & Traumatol, Izmir, Turkey.
EM hasan.tatari@deu.edu.tr
RI Bagriyanik, Husnu Alper/H-7577-2013; Cecen, Berivan/Q-4203-2019; Özcan,
   Mehmet Ali/Q-1897-2019; Ozkal, Sermin/AAH-2615-2020
OI Bagriyanik, Husnu Alper/0000-0003-4064-7637; Cecen,
   Berivan/0000-0002-0081-2004;
CR Anitua E, 2005, J ORTHOP RES, V23, P281, DOI 10.1016/j.orthres.2004.08.015
   Aspenberg P, 2004, ACTA ORTHOP SCAND, V75, P93, DOI 10.1080/00016470410001708190
   Badhe SP, 2008, J SHOULDER ELB SURG, V17, p35S, DOI 10.1016/j.jse.2007.08.005
   Bolt P, 2007, J BONE JOINT SURG AM, V89A, P1315, DOI 10.2106/JBJS.F.00257
   Coban Ibrahim, 2009, Foot Ankle Surg, V15, P22, DOI 10.1016/j.fas.2008.06.005
   De Carli A, 2015, KNEE SURG SPORTS TRA
   de Vos RJ, 2011, BRIT J SPORT MED, V45, P387, DOI 10.1136/bjsm.2010.076398
   de Vos RJ, 2010, JAMA-J AM MED ASSOC, V303, P144, DOI 10.1001/jama.2009.1986
   Efeoglu C, 2004, J ORAL MAXIL SURG, V62, P1403, DOI 10.1016/j.joms.2004.06.047
   Forslund C, 2003, AM J SPORT MED, V31, P555, DOI 10.1177/03635465030310041301
   Halici M, 2004, KNEE SURG SPORT TR A, V12, P562, DOI 10.1007/s00167-004-0536-2
   Hazard SW, 2011, EXP MOL PATHOL, V91, P660, DOI 10.1016/j.yexmp.2011.08.002
   Jelinsky SA, 2011, J ORTHOP RES, V29, P1604, DOI 10.1002/jor.21427
   Kajikawa Y, 2008, J CELL PHYSIOL, V215, P837, DOI 10.1002/jcp.21368
   Kaniki N, 2014, ARTHROSCOPY, V30, P1139, DOI 10.1016/j.arthro.2014.04.086
   Kaux JF, 2012, WOUND REPAIR REGEN, V20, P748, DOI 10.1111/j.1524-475X.2012.00826.x
   Khan RJK, 2005, J BONE JOINT SURG AM, V87A, P2202, DOI 10.2106/JBJS.D.03049
   Kim HJ, 2007, CONNECT TISSUE RES, V48, P309, DOI 10.1080/03008200701692610
   Konerding MA, 2010, INT J MOL MED, V25, P217, DOI 10.3892/ijmm_00000333
   Kurtz CA, 1999, AM J SPORT MED, V27, P363, DOI 10.1177/03635465990270031701
   Lee Daniel K, 2007, J Foot Ankle Surg, V46, P451, DOI 10.1053/j.jfas.2007.05.007
   Leek BT, 2012, AM J SPORT MED, V40, P1762, DOI 10.1177/0363546512450691
   Lui PPY, 2007, WOUND REPAIR REGEN, V15, P702, DOI 10.1111/j.1524-475X.2007.00276.x
   Lyras DN, 2009, FOOT ANKLE INT, V30, P1101, DOI 10.3113/FAI.2009.1101
   Lyras DN, 2007, FOOT ANKLE SURG, V16, P137
   Mae T, 2003, CLIN ORTHOP RELAT R, P305, DOI 10.1097/01.blo.0000079440.64912.c3
   Malcarney HL, 2005, AM J SPORT MED, V33, P907, DOI 10.1177/0363546504271500
   Nicholson GP, 2007, J SHOULDER ELB SURG, V16, p184S, DOI 10.1016/j.jse.2007.03.010
   Paoloni J, 2011, CLIN J SPORT MED, V21, P37, DOI 10.1097/JSM.0b013e31820758c7
   Petrou CG, 2009, J MUSCULOSKEL NEURON, V9, P147
   Sanchez M, 2007, AM J SPORT MED, V35, P245, DOI 10.1177/0363546506294078
   Sarrafian TL, 2010, J FOOT ANKLE SURG, V49, P128, DOI 10.1053/j.jfas.2009.12.005
   Sasaki K, 2012, J ELECTRON MICROSC, V61, P327, DOI 10.1093/jmicro/dfs057
   Schepull T, 2011, AM J SPORT MED, V39, P38, DOI 10.1177/0363546510383515
   STEINER M, 1982, J BIOMECH, V15, P951, DOI 10.1016/0021-9290(82)90013-6
   TANG JB, 1994, J HAND SURG-AM, V19A, P61, DOI 10.1016/0363-5023(94)90225-9
   Taylor DW, 2011, CLIN J SPORT MED, V21, P344, DOI 10.1097/JSM.0b013e31821d0f65
   Virchenko O, 2006, ACTA ORTHOP, V77, P806, DOI 10.1080/17453670610013033
   Xu K, 2015, J TISSUE ENG REGEN M
   Yokota K, 2008, ACTA ORTHOP, V79, P106, DOI 10.1080/17453670710014842
   Zhang F, 2003, PLAST RECONSTR SURG, V112, P1613, DOI 10.1097/01.PRS.0000086772.72535.A4
   Zheng MH, 2005, J BIOMED MATER RES B, V73B, P61, DOI 10.1002/jbm.b.30170
NR 42
TC 3
Z9 4
U1 0
U2 7
PU GALENOS PUBL HOUSE
PI ISTANBUL
PA  MOLLA GURANI MAHALLESI KACAMAK SOKAK NO 21-1, ISTANBUL, TURKEY
SN 2146-3123
EI 2146-3131
J9 BALK MED J
JI Balk. Med. J.
PD JAN
PY 2016
VL 33
IS 1
BP 94
EP 101
DI 10.5152/balkanmedj.2015.15549
PG 8
WC Medicine, General & Internal
WE Science Citation Index Expanded (SCI-EXPANDED); Conference Proceedings Citation Index - Science (CPCI-S)
SC General & Internal Medicine
GA DE9ON
UT WOS:000370967500015
PM 26966624
OA Green Submitted, Green Published, gold
DA 2022-07-22
ER

PT J
AU Zickmantel, B
   Krause, F
   Frauchiger, L
AF Zickmantel, Benjamin, Jr.
   Krause, Fabian
   Frauchiger, Lars
TI Isolated Rupture of the Distal Plantaris Muscle
SO JOURNAL OF FOOT & ANKLE SURGERY
LA English
DT Article
DE Achilles tendon; musculus gastrocnemius; musculus plantaris; tendon
   rupture
AB In the present case, the plantaris tendon was ruptured in isolation and at the distal part of the tendon. An injury of the Achilles tendon, gastrocnemius muscle, or soleus muscle was not detected. To the best of our knowledge, a similar case has not yet been reported. Our case basically demonstrates that a rupture of the musculus plantaris does not have to occur at the myotendiouns junction or the muscle belly itself. The initial nonoperative treatment with physiotherapy, antiinflammatory medicine, and an early return to sports, even for this more distal plantaris tendon rupture, led to a good result. (C) 2017 by the American College of Foot and Ankle Surgeons. All rights reserved.
C1 [Zickmantel, Benjamin, Jr.; Frauchiger, Lars] Spital STS AG Thun, Dept Orthopaed Surg & Traumatol, Thun, Switzerland.
   [Krause, Fabian] Univ Bern, Inselspital, Dept Orthopaed Surg & Traumatol, Bern Univ Hosp, Bern, Switzerland.
RP Zickmantel, B (通讯作者)，Altenbergstr 78, CH-3013 Bern, Switzerland.
EM h.zickmantel@web.de
CR Gopinath TN, 2012, J CLIN IMAG SCI, V2, DOI 10.4103/2156-7514.95433
   HELMS CA, 1995, RADIOLOGY, V195, P201, DOI 10.1148/radiology.195.1.7892469
   Spang C, 2016, BONE JOINT J, V98B, P1312, DOI 10.1302/0301-620X.98B10.37939
NR 3
TC 0
Z9 0
U1 0
U2 0
PU ELSEVIER SCIENCE INC
PI NEW YORK
PA 360 PARK AVE SOUTH, NEW YORK, NY 10010-1710 USA
SN 1067-2516
EI 1542-2224
J9 J FOOT ANKLE SURG
JI J. Foot Ankle Surg.
PD SEP-OCT
PY 2018
VL 57
IS 5
BP 995
EP 996
DI 10.1053/j.jfas.2017.11.027
PG 2
WC Orthopedics; Surgery
WE Science Citation Index Expanded (SCI-EXPANDED)
SC Orthopedics; Surgery
GA GR5LQ
UT WOS:000442674300027
PM 29622499
DA 2022-07-22
ER

PT J
AU Hufner, TM
   Brandes, DB
   Thermann, H
   Richter, M
   Knobloch, K
   Krettek, C
AF Hufner, TM
   Brandes, DB
   Thermann, H
   Richter, M
   Knobloch, K
   Krettek, C
TI Long-term results after functional nonoperative treatment of Achilles
   tendon rupture
SO FOOT & ANKLE INTERNATIONAL
LA English
DT Article
DE Achilles tendon rupture; nonoperative treatment
ID NON-SURGICAL TREATMENT; OPERATIVE TREATMENT
AB Background: Nonoperative treatment of complete Achilles tendon ruptures generally involves a long period of cast immobilization and is associated with frequent reruptures. Functional nonoperative treatment of complete Achilles tendon ruptures involves the use of a high-shaft boot with a 3-cm bindfoot elevation, in which physical therapy is begun after 3 weeks of wear. We reviewed our long-term results with this treatment protocol to determine its effectiveness. Methods: The indications for nonoperative treatment, defined by ultrasound, were a distance of 10 mm or less between the tendon ends with the ankle in neutral position and complete apposition of the tendon ends in 20 degrees of plantarflexion. From 1990 to 1996, 168 patients were treated; 125 (74%) were available for followup at a mean of 5.5 (2 to 12.7) years after the injury. Results: Good or excellent results were achieved in 92 (73.5%) with complete rehabilitation and return to sports activity at their pre-injury levels. Satisfactory (9%) and poor results (17.5%) were due to pain in the Achilles tendon region, a lengthened Achilles tendon, markedly reduced strength, or a marked reduction of calf size in 25 patients (76%). Eight patients (6.4%) sustained a rerupture. Conclusions: Functional nonoperative treatment achieved good results in patients who had precise sonographic evaluation and who were compliant. As a result of our study, we modified our protocol: (1) a repeat ultrasound examination is done by an experienced sonographer 2 to 5 days after the first to confirm the indications for nonoperative treatment, (2) the use of the 3-cm hindfoot elevation is extended from 6 to 8 weeks to provide a longer protection of the tendon, and (3) patients then wear shoes with 1-cm hindfoot elevation for another 3 months.
C1 Hannover Med Sch, Dept Trauma, D-30625 Hannover, Lower Saxony, Germany.
   Atos Clin, Ctr Knee & Foot Surg Sportstraumatol, Heidelberg, Germany.
RP Hufner, TM (通讯作者)，Hannover Med Sch, Dept Trauma, D-30625 Hannover, Lower Saxony, Germany.
EM huefner.tobias@mh-hannover.de
RI Knobloch, Karsten/AAI-3312-2020
OI Knobloch, Karsten/0000-0003-1468-4258; Krettek,
   Christian/0000-0001-9807-0020
CR GILLIES H, 1970, J BONE JOINT SURG AM, VA 52, P337, DOI 10.2106/00004623-197052020-00015
   KELLAM JF, 1985, CLIN ORTHOP RELAT R, P80
   LEA RB, 1972, J BONE JOINT SURG AM, VA 54, P1398, DOI 10.2106/00004623-197254070-00002
   LILDHOLDT T, 1976, ACTA ORTHOP SCAND, V47, P454
   Lo IKY, 1997, CLIN J SPORT MED, V7, P207, DOI 10.1097/00042752-199707000-00010
   Maffulli N, 1999, J BONE JOINT SURG AM, V81A, P1019, DOI 10.2106/00004623-199907000-00017
   NISTOR L, 1981, J BONE JOINT SURG AM, V63, P394, DOI 10.2106/00004623-198163030-00012
   Reilmann H, 1996, UNFALLCHIRURG, V99, P576
   THERMANN H, 1995, UNFALLCHIRURG, V98, P21
   WILLS CA, 1986, CLIN ORTHOP RELAT R, P156
   ZWIPP H, 1989, UNFALLCHIRURG, V92, P554
NR 11
TC 43
Z9 43
U1 1
U2 3
PU SAGE PUBLICATIONS INC
PI THOUSAND OAKS
PA 2455 TELLER RD, THOUSAND OAKS, CA 91320 USA
SN 1071-1007
EI 1944-7876
J9 FOOT ANKLE INT
JI Foot Ankle Int.
PD MAR
PY 2006
VL 27
IS 3
BP 167
EP 171
DI 10.1177/107110070602700302
PG 5
WC Orthopedics
WE Science Citation Index Expanded (SCI-EXPANDED)
SC Orthopedics
GA 020XP
UT WOS:000235946900002
PM 16539896
DA 2022-07-22
ER

PT J
AU Baxter, JR
   Hullfish, TJ
   Chao, W
AF Baxter, Josh R.
   Hullfish, Todd J.
   Chao, Wen
TI Functional deficits may be explained by plantarflexor remodeling
   following Achilles tendon rupture repair: Preliminary findings
SO JOURNAL OF BIOMECHANICS
LA English
DT Article
DE Achilles tendon rupture; Ultrasound; Muscle morphology
ID IN-VIVO; RANDOMIZED-TRIAL; FASCICLE LENGTH; MUSCLE-TENDON; MOMENT ARM;
   ADAPTATION; TENSION; ANKLE; REHABILITATION; IMMOBILIZATION
AB Achilles tendon ruptures are common injuries that often lead to long-term functional deficits. Despite the prevalence of these injuries, the mechanism responsible for limited function has not yet been established. Therefore, the purpose of this study was to present preliminary findings that support a hypothesis that skeletal muscle remodeling is the driving factor of poor outcomes in some patients. Biomechanical and ultrasonography assessments were performed on a patient that presented with poor functional outcomes 2.5 years after a surgically-repaired acute Achilles tendon rupture. Single-leg heel raise height was decreased by 75% in the affected limb (3.0 cm compared to 11.9 cm) while walking mechanics showed no deficits. Ultrasonography revealed that the affected medial gastrocnemius muscle was less thick and had shorter, more pennate fascicles compared to the unaffected limb. A simple computational model of a maximal-effort plantarflexion contraction was employed to test the implications of changes in muscle architecture on single-leg heel raise function. Subject-specific measurements of fascicle length and pennation were input into the model, which supported these architectural parameters as being drivers of heel raise function. These preliminary findings support the hypothesis that an Achilles tendon rupture elicits changes in skeletal muscle architecture, which reduces the amount of work and power the joint can generate. This multidisciplinary framework of biomechanical, imaging, and computational modeling provides a unique platform for studying the complex interactions between structure and function in patients recovering from Achilles tendon injuries. (C) 2018 Elsevier Ltd. All rights reserved.
C1 [Baxter, Josh R.; Hullfish, Todd J.; Chao, Wen] Univ Penn, Perelman Sch Med, Dept Orthopaed Surg, Philadelphia, PA 19104 USA.
RP Baxter, JR (通讯作者)，3737 Market St,Suite 1050, Philadelphia, PA 19104 USA.
EM josh.baxter@uphs.upenn.edu
RI Baxter, Josh/N-4020-2013
OI Baxter, Josh/0000-0003-0269-2170
CR Arnold EM, 2010, ANN BIOMED ENG, V38, P269, DOI 10.1007/s10439-009-9852-5
   Baxter JR, 2014, J APPL PHYSIOL, V116, P538, DOI 10.1152/japplphysiol.01140.2013
   Baxter JR, 2012, P ROY SOC B-BIOL SCI, V279, P2018, DOI 10.1098/rspb.2011.2358
   Blazevich AJ, 2007, J APPL PHYSIOL, V103, P1565, DOI 10.1152/japplphysiol.00578.2007
   Burkholder TJ, 1998, J EXP BIOL, V201, P309
   Delp SL, 2007, IEEE T BIO-MED ENG, V54, P1940, DOI 10.1109/TBME.2007.901024
   Demetracopoulos CA, 2014, FOOT ANKLE INT, V35, P612, DOI 10.1177/1071100714524550
   Fath F, 2010, J APPL PHYSIOL, V109, P1644, DOI 10.1152/japplphysiol.00656.2010
   Flanagan SP, 2005, J AGING PHYS ACTIV, V13, P160, DOI 10.1123/japa.13.2.160
   Fukunaga T, 1997, J APPL PHYSIOL, V82, P354, DOI 10.1152/jappl.1997.82.1.354
   Fukunaga T, 2001, P ROY SOC B-BIOL SCI, V268, P229, DOI 10.1098/rspb.2000.1361
   Heikkinen J, 2017, AM J SPORT MED, V45, P1395, DOI 10.1177/0363546517694610
   HOWELL JN, 1993, J PHYSIOL-LONDON, V464, P183, DOI 10.1113/jphysiol.1993.sp019629
   Ishikawa M, 2005, J APPL PHYSIOL, V99, P603, DOI 10.1152/japplphysiol.00189.2005
   Kangas J, 2003, J TRAUMA, V54, P1171, DOI 10.1097/01.TA.0000047945.20863.A2
   Kangas J, 2007, AM J SPORT MED, V35, P59, DOI 10.1177/0363546506293255
   Kawakami Y, 1995, EUR J APPL PHYSIOL O, V72, P37, DOI 10.1007/BF00964112
   Kawakami Y, 1998, J APPL PHYSIOL, V85, P398, DOI 10.1152/jappl.1998.85.2.398
   Koh TJ, 1998, J BIOMECH, V31, P593, DOI 10.1016/S0021-9290(98)00052-9
   Lantto I, 2015, SCAND J MED SCI SPOR, V25, pE133, DOI 10.1111/sms.12253
   Lantto I, 2015, AM J SPORT MED, V43, P2302, DOI 10.1177/0363546515591267
   Leppilahti J, 2000, RELATIONSHIP CALF MU
   Lichtwark GA, 2005, J EXP BIOL, V208, P4715, DOI 10.1242/jeb.01950
   MANDELBAUM BR, 1995, AM J SPORT MED, V23, P392, DOI 10.1177/036354659502300403
   Morse CI, 2005, J APPL PHYSIOL, V99, P1050, DOI 10.1152/japplphysiol.01186.2004
   Mortensen NHM, 1999, J BONE JOINT SURG AM, V81A, P983, DOI 10.2106/00004623-199907000-00011
   Nilsson-Helander K, 2007, AM J SPORT MED, V35, P421, DOI 10.1177/0363546506294856
   NYSTROM B, 1983, ACTA ORTHOP SCAND, V54, P620, DOI 10.3109/17453678308992899
   Osterberg U, 1998, CLIN BIOMECH, V13, P344, DOI 10.1016/S0268-0033(98)00100-4
   Seynnes OR, 2007, J APPL PHYSIOL, V102, P368, DOI 10.1152/japplphysiol.00789.2006
   Silbernagel KG, 2012, AM J SPORT MED, V40, P1564, DOI 10.1177/0363546512447926
   Takahashi M, 2010, J BONE JOINT SURG AM, V92A, P664, DOI 10.2106/JBJS.I.00694
   Wapner KL, 2006, FOOT ANKLE INT, V27, P591, DOI 10.1177/107110070602700805
   Westin O, 2016, ORTHOP J SPORTS MED, V4, DOI 10.1177/2325967116667920
   Willits K, 2010, J BONE JOINT SURG AM, V92A, P2767, DOI 10.2106/JBJS.I.01401
   Willy RW, 2017, AM J SPORT MED, V45, P1124, DOI 10.1177/0363546516685055
   Zhou GQ, 2015, ULTRASONICS, V57, P72, DOI 10.1016/j.ultras.2014.10.020
NR 37
TC 18
Z9 18
U1 0
U2 6
PU ELSEVIER SCI LTD
PI OXFORD
PA THE BOULEVARD, LANGFORD LANE, KIDLINGTON, OXFORD OX5 1GB, OXON, ENGLAND
SN 0021-9290
EI 1873-2380
J9 J BIOMECH
JI J. Biomech.
PD OCT 5
PY 2018
VL 79
BP 238
EP 242
DI 10.1016/j.jbiomech.2018.08.016
PG 5
WC Biophysics; Engineering, Biomedical
WE Science Citation Index Expanded (SCI-EXPANDED)
SC Biophysics; Engineering
GA GX2WJ
UT WOS:000447580500031
PM 30166224
OA Green Submitted
DA 2022-07-22
ER

PT J
AU Cho, J
   Kim, HJ
   Lee, JS
   Kim, J
   Won, SH
   Yi, Y
   Chun, DI
AF Cho, Jaeho
   Kim, Hyun-Joo
   Lee, Jeong Seok
   Kim, Jahyung
   Won, Sung Hun
   Yi, Young
   Chun, Dong-Il
TI Comparing Absorbable and Nonabsorbable Suture Materials for Repair of
   Achilles Tendon Rupture: A Magnetic Resonance Imaging-Based Study
SO DIAGNOSTICS
LA English
DT Article
DE Achilles tendon rupture; magnetic resonance imaging; suture material;
   tendon healing
ID INJURY
AB We aimed to compare magnetic resonance imaging (MRI) findings and corresponding clinical outcomes of repaired Achilles tendons using absorbable and nonabsorbable sutures. Patients who underwent Achilles tendon repair were divided into 2 groups, with 11 in the absorbable group (group A) and 11 in the nonabsorbable group (group B). For all patients, MRI findings taken 6 months postoperatively were evaluated for morphological changes in the tendon. Concurrently, the American Orthopedic Foot and Ankle Society (AOFAS) ankle-hind foot score and incidence of postoperative complications were evaluated. Regarding MRI findings, the extent to which the cross-sectional area of the repaired tendon was thicker than that of the preoperative tendon was significantly greater in group B than in group A (p = 0.0012). Notably, more stitches remained within the tendon in group B than in group A (p = 0.0063). No other MRI findings showed a significant difference between the two groups. No significant difference was observed in the AOFAS score, and there was one re-rupture each in both groups. Because nonabsorbable suture material in the treatment of Achilles tendon rupture yielded a thicker postoperative MRI cross-sectional area, enhanced rehabilitation is recommended in order to prevent scar formation.
C1 [Cho, Jaeho] Hallym Univ, Chuncheon Sacred Heart Hosp, Dept Orthopaed Surg, 77 Sakju Ro, Chuncheon Si 24253, South Korea.
   [Kim, Hyun-Joo] Soonchunhyang Univ, Seoul Hosp, Dept Radiol, 59 Daesagwan Ro, Seoul 04401, South Korea.
   [Lee, Jeong Seok; Kim, Jahyung; Won, Sung Hun; Chun, Dong-Il] Soonchunhyang Univ, Seoul Hosp, Dept Orthopaed Surg, 59 Daesagwan Ro, Seoul 04401, South Korea.
   [Yi, Young] Inje Univ, Seoul Paik Hosp, Seoul Foot & Ankle Ctr, Dept Orthopaed Surg, 85 2 Ga, Seoul 04551, South Korea.
RP Chun, DI (通讯作者)，Soonchunhyang Univ, Seoul Hosp, Dept Orthopaed Surg, 59 Daesagwan Ro, Seoul 04401, South Korea.
EM hohotoy@nate.com; reonora@schmc.ac.kr; 124856@schmc.ac.kr;
   hpsyndrome@naver.com; orthowon@gmail.com; 20vvin@naver.com;
   orthochun@gmail.com
OI WON, SUNG HUN/0000-0002-5471-7432; Cho, Jaeho/0000-0001-8680-4680; Chun,
   Dong-il/0000-0001-7255-8984
FU Soonchunhyang University Research Fund
FX This work was supported by Soonchunhyang University Research Fund.
CR Ackerman JE, 2019, J ORTHOP RES, V37, P2476, DOI 10.1002/jor.24397
   Baig M., 2017, IR MED J
   Barber FA, 2003, ARTHROSCOPY, V19, P985, DOI 10.1016/j.arthro.2003.09.018
   Brumann M, 2014, INJURY, V45, P1782, DOI 10.1016/j.injury.2014.06.022
   Byrne M, 2019, AESTHET SURG J, V39, pS67, DOI 10.1093/asj/sjz036
   Carr Brian J, 2009, Orthopedics, V32, P828, DOI 10.3928/01477447-20090922-11
   Deng SL, 2017, J FOOT ANKLE SURG, V56, P1236, DOI 10.1053/j.jfas.2017.05.036
   Elliot D, 2007, J HAND SURG-BRIT EUR, V32E, P346, DOI 10.1016/j.jhse.2007.03.005
   Froberg A, 2017, KNEE SURG SPORT TR A, V25, P1857, DOI 10.1007/s00167-016-4394-5
   GELBERMAN R H, 1984, Journal of Orthopaedic Research, V2, P39, DOI 10.1002/jor.1100020107
   HARAMATI N, 1994, JMRI-J MAGN RESON IM, V4, P209, DOI 10.1002/jmri.1880040219
   Karjalainen PT, 1996, ACTA RADIOL, V37, P639
   Karjalainen PT, 1997, AM J SPORT MED, V25, P164, DOI 10.1177/036354659702500204
   Khan KM, 1998, CLIN J SPORT MED, V8, P1, DOI 10.1097/00042752-199801000-00001
   Kitaoka HB, 1997, FOOT ANKLE INT, V18, P187, DOI 10.1177/107110079701800315
   Kocaoglu B, 2015, FOOT ANKLE INT, V36, P691, DOI 10.1177/1071100714568868
   Koob TJ, 2002, COMP BIOCHEM PHYS A, V133, P1171, DOI 10.1016/S1095-6433(02)00247-7
   Koob TJ, 2002, COMP BIOCHEM PHYS A, V133, P905, DOI 10.1016/S1095-6433(02)00255-6
   Kreszinger M, 2011, VET ARHIV, V81, P223
   Lee JE, 2015, KOREAN J RADIOL, V16, P363, DOI 10.3348/kjr.2015.16.2.363
   Maffulli N, 2001, SCAND J MED SCI SPOR, V11, P156
   MANSKE PR, 1984, J HAND SURG-BRIT EUR, V9B, P117, DOI 10.1016/S0266-7681(84)80005-4
   Miyashita H, 1997, ARCH ORTHOP TRAUM SU, V116, P454, DOI 10.1007/BF00387577
   Moller M, 2001, J BONE JOINT SURG BR, V83B, P843
   Nichols AEC, 2019, TRANSL RES, V209, P156, DOI 10.1016/j.trsl.2019.02.002
   Okoroha KR, 2020, AM J SPORT MED, V48, P1720, DOI 10.1177/0363546520909389
   Park JY, 2017, IEEE T SEMICONDUCT M, V30, P39, DOI 10.1109/TSM.2016.2618894
   POTENZA AD, 1962, J BONE JOINT SURG AM, V44, P49, DOI 10.2106/00004623-196244010-00004
   Sharma P, 2005, J BONE JOINT SURG AM, V87A, P187, DOI 10.2106/JBJS.D.01850
   Tempfer H, 2015, FRONT PHYSIOL, V6, DOI 10.3389/fphys.2015.00330
   Thomopoulos S, 2015, J ORTHOP RES, V33, P832, DOI 10.1002/jor.22806
   Wada A, 2001, J HAND SURG-AM, V26A, P398, DOI 10.1053/jhsu.2001.24134
   WHALEN WP, 1951, ANN SURG, V133, P567
   Wichelhaus DA, 2016, ARCH ORTHOP TRAUM SU, V136, P1021, DOI 10.1007/s00402-016-2472-2
   Yildirim Yakup, 2006, Acta Orthop Traumatol Turc, V40, P164
NR 35
TC 2
Z9 2
U1 0
U2 2
PU MDPI
PI BASEL
PA ST ALBAN-ANLAGE 66, CH-4052 BASEL, SWITZERLAND
EI 2075-4418
J9 DIAGNOSTICS
JI Diagnostics
PD DEC
PY 2020
VL 10
IS 12
AR 1085
DI 10.3390/diagnostics10121085
PG 11
WC Medicine, General & Internal
WE Science Citation Index Expanded (SCI-EXPANDED)
SC General & Internal Medicine
GA PK1AT
UT WOS:000602186700001
PM 33322173
OA Green Published, gold
DA 2022-07-22
ER

PT J
AU Wiegand, N
   Vamhidy, L
   Lorinczy, D
AF Wiegand, N.
   Vamhidy, L.
   Lorinczy, D.
TI Differential scanning calorimetric examination of ruptured lower limb
   tendons in human
SO JOURNAL OF THERMAL ANALYSIS AND CALORIMETRY
LA English
DT Article; Proceedings Paper
CT 10th Conference on Calorimetry and Thermal Analysis (CCTA 10)/2nd
   Czech-Hungarian-Polish-Slovakian Thermoanalytical Conference
CY AUG 30-SEP 03, 2009
CL Zakopane, POLAND
SP Polish Soc Calorimetry & Thermal Anal
DE Achilles tendon; Quadriceps tendon; Patellar ligament rupture; DSC
ID ACHILLES-TENDON; DSC EXAMINATION; COLLAGEN; MODEL; INJURIES; TISSUE
AB The tendon ruptures are serious injuries of the lover limb in middle age and physically active population. While the Achilles tendon rupture is common, the patellar ligament and quadriceps ligament ruptures are an absolutely rare injury. Usually there is no correlation between the velocity of the trauma and the supervening of the rupture. The aetiology of the degenerative changes in the collagen structures of the tendons and ligaments which could be disposed for the rupture are still not clear. Our hypothesis was that before the injury there are clear pathological abnormalities in the tissues of the tendons, which are predisposed for the rupture, and could be monitored besides the classical histological methods by differential scanning calorimetry. The thermal denaturation of human samples was monitored by a SETARAM Micro DSC-II calorimeter. All the experiments were performed between 0 and 100 A degrees C. The heating rate was 0.3 K/min. DSC scans clearly demonstrated significant differences between the control and ruptured samples (control: T (m) = 59.7 A degrees C, T (1/2) = 1.4 A degrees C and Delta H (cal) = 8.54 J/g; ruptured Achilles tendon: T (m) = 62.75 A degrees C, T (1/2) = 2.6 A degrees C and Delta H (cal) = 1.54 J/g, ruptured Quadriceps tendon: T (m) = 64.8 A degrees C, T (1/2) = 1.6 A degrees C and Delta H (cal) = 1.53 J/g, ruptured Patellar tendon: T (m) = 63.9 A degrees C, T (1/2) = 1.41 A degrees C and Delta H (cal) = 0.97 J/g). These observations could be explained with the structural alterations caused by the biochemical processes. With our investigations we could demonstrate that DSC is a useful and well applicable method for the investigation of collagen tissue of the degenerated human tendons and ligaments. We can prove with this method that the degenerative changes of the tissue elements increase the thermal stability of collagen tissues of the tendons which could be disposed for the rupture.
C1 [Lorinczy, D.] Univ Pecs, Fac Med, Dept Biophys, H-7624 Pecs, Hungary.
   [Wiegand, N.; Vamhidy, L.] Univ Pecs, Fac Med, Dept Traumatol, H-7624 Pecs, Hungary.
RP Lorinczy, D (通讯作者)，Univ Pecs, Fac Med, Dept Biophys, Szigeti Str 12, H-7624 Pecs, Hungary.
EM denes.lorinczy@aok.pte.hu
CR Amlang MH, 2006, CHIRURG, V77, P637, DOI 10.1007/s00104-006-1205-4
   Clayton RAE, 2008, INJURY, V39, P1338, DOI 10.1016/j.injury.2008.06.021
   Comerford EJ, 2005, J ORTHOP RES, V23, P61, DOI 10.1016/j.orthres.2004.05.016
   Comerford EJ, 2004, AM J VET RES, V65, P1136, DOI 10.2460/ajvr.2004.65.1136
   Eriksen HA, 2002, J ORTHOPAED RES, V20, P1352, DOI 10.1016/S0736-0266(02)00064-5
   Glazebrook MA, 2008, J ORTHOP RES, V26, P840, DOI 10.1002/jor.20546
   Jarvinen TAH, 2004, J ORTHOP RES, V22, P1303, DOI 10.1016/j.orthres.2004.04.003
   Kannus P, 2000, SCAND J MED SCI SPOR, V10, P312, DOI 10.1034/j.1600-0838.2000.010006312.x
   Lorinczy D, 2009, J THERM ANAL CALORIM, V95, P713
   Maffulli N, 2000, AM J SPORT MED, V28, P857, DOI 10.1177/03635465000280061401
   MILES CA, 1994, EQUINE VET J, V26, P255, DOI 10.1111/j.2042-3306.1994.tb04383.x
   MILES CA, 1995, J MOL BIOL, V245, P437, DOI 10.1006/jmbi.1994.0035
   Nedvig K, 2009, J THERM ANAL CALORIM, V95, P775, DOI 10.1007/s10973-008-9416-1
   RESCH H, 1995, ORTHOPADE, V24, P209
   Selvanetti A, 1997, OPER TECHN SPORT MED, V5, P110, DOI 10.1016/S1060-1872(97)80031-7
   Sohar G, 2007, J THERM ANAL CALORIM, V89, P853, DOI 10.1007/s10973-007-8256-8
   Strauss EJ, 2007, INJURY, V38, P832, DOI 10.1016/j.injury.2006.06.005
   Trobisch PD, 2010, KNEE SURG SPORT TR A, V18, P85, DOI 10.1007/s00167-009-0884-z
   Wilhelm F, 2007, J THERM ANAL CALORIM, V89, P863, DOI 10.1007/s10973-006-7927-1
   Willett TL, 2008, J ORTHOP RES, V26, P1605, DOI 10.1002/jor.20672
   Willett TL, 2007, ANN BIOMED ENG, V35, P1961, DOI 10.1007/s10439-007-9375-x
   Willett TL, 2009, J BONE JOINT SOC D 2, V91, P217
   Yepes H, 2008, J BONE JOINT SURG AM, V90A, P2135, DOI 10.2106/JBJS.G.01200
NR 23
TC 20
Z9 20
U1 0
U2 8
PU SPRINGER
PI DORDRECHT
PA VAN GODEWIJCKSTRAAT 30, 3311 GZ DORDRECHT, NETHERLANDS
SN 1388-6150
EI 1588-2926
J9 J THERM ANAL CALORIM
JI J. Therm. Anal. Calorim.
PD AUG
PY 2010
VL 101
IS 2
BP 487
EP 492
DI 10.1007/s10973-010-0820-y
PG 6
WC Thermodynamics; Chemistry, Analytical; Chemistry, Physical
WE Science Citation Index Expanded (SCI-EXPANDED); Conference Proceedings Citation Index - Science (CPCI-S)
SC Thermodynamics; Chemistry
GA 640DG
UT WOS:000281027500011
DA 2022-07-22
ER

PT J
AU Huttunen, TT
   Kannus, P
   Rolf, C
   Fellander-Tsai, L
   Mattila, VM
AF Huttunen, Tuomas T.
   Kannus, Pekka
   Rolf, Christer
   Fellander-Tsai, Li
   Mattila, Ville M.
TI Acute Achilles Tendon Ruptures Incidence of Injury and Surgery in Sweden
   Between 2001 and 2012
SO AMERICAN JOURNAL OF SPORTS MEDICINE
LA English
DT Article
DE Achilles tendon; injury; surgical treatment; incidence; epidemiology
ID NONOPERATIVE TREATMENT; III COLLAGEN; EPIDEMIOLOGY; TENDINOPATHY;
   METAANALYSIS; FINLAND; TRIALS
AB Background: Population-based incidence rates and trends of acute Achilles tendon ruptures are not known. It is also not known whether recent high-quality randomized controlled trials not favoring surgery have had an effect on treatment protocols.
   Purpose: To assess the incidence of acute Achilles tendon ruptures in Sweden and to examine the trends in surgical treatment.
   Study Design: Descriptive epidemiology study.
   Methods: We conducted a nationwide registry-based study including all adult (18 years of age) inpatient and outpatient hospital visits because of an acute Achilles tendon rupture in Sweden between 2001 and 2012.
   Results: We identified a total of 27,702 patients (21,979 men, 79%) with acute Achilles tendon ruptures between 2001 and 2012. In 2001, the sex-specific incidence of acute Achilles tendon ruptures was 47.0 (per 100,000 person-years) in men and 12.0 in women. In 2012, the corresponding values were 55.2 in men and 14.7 in women, with an increase of 17% in men and 22% in women. The proportion of surgically treated patients declined from 43% in 2001 to 28% in 2012 in men and from 34% in 2001 to 22% in 2012 in women.
   Conclusion: The incidence of acute Achilles tendon ruptures in Sweden is increasing. The most probable reason for this increase is the rise in the number of older adults participating in high-demand sports. The proportion of surgically treated patients is decreasing most likely because of recent high-quality randomized controlled trials and their meta-analyses supporting similar results between surgical and nonsurgical approaches.
C1 [Huttunen, Tuomas T.; Kannus, Pekka; Rolf, Christer; Fellander-Tsai, Li; Mattila, Ville M.] Karolinska Inst, Solna, Sweden.
RP Mattila, VM (通讯作者)，Tampere Univ Hosp, Dept Orthoped & Trauma Surg, Tampere 33100, Finland.
EM ville.mattila@uta.fi
OI Huttunen, Tuomas/0000-0001-7884-7533; Fellander-Tsai,
   Li/0000-0003-0693-6080
CR Barfod KW, 2013, J FOOT ANKLE SURG, V52, P629, DOI 10.1053/j.jfas.2013.04.012
   CETTI R, 1993, AM J SPORT MED, V21, P791, DOI 10.1177/036354659302100606
   Clayton RAE, 2008, INJURY, V39, P1338, DOI 10.1016/j.injury.2008.06.021
   Crawford J, 2004, COCHRANE DB SYST REV, DOI DOI 10.1002/14651858.CD003674.PUB2
   Eriksen HA, 2002, J ORTHOPAED RES, V20, P1352, DOI 10.1016/S0736-0266(02)00064-5
   Holmes GB, 2006, FOOT ANKLE INT, V27, P952, DOI 10.1177/107110070602701115
   Houshian S, 1998, INJURY, V29, P651, DOI 10.1016/S0020-1383(98)00147-8
   KANNUS P, 1991, J BONE JOINT SURG AM, V73A, P1507, DOI 10.2106/00004623-199173100-00009
   Khan RJK, 2005, J BONE JOINT SURG AM, V87A, P2202, DOI 10.2106/JBJS.D.03049
   Kujala UM, 2005, CLIN J SPORT MED, V15, P133, DOI 10.1097/01.jsm.0000165347.55638.23
   Leppilahti J, 1996, ACTA ORTHOP SCAND, V67, P277, DOI 10.3109/17453679608994688
   Levi N, 1997, INJURY, V28, P311, DOI 10.1016/S0020-1383(96)00200-8
   Ludvigsson JF, 2011, BMC PUBLIC HEALTH, V11, DOI 10.1186/1471-2458-11-450
   Maffulli N, 1999, CLIN J SPORT MED, V9, P157, DOI 10.1097/00042752-199907000-00007
   Mathiak G, 1999, ARCH ORTHOP TRAUM SU, V119, P280, DOI 10.1007/s004020050410
   Mattila VM, 2015, BRIT J SPORT MED, V49, P1084, DOI 10.1136/bjsports-2013-092756
   Metz R, 2008, AM J SPORT MED, V36, P1688, DOI 10.1177/0363546508319312
   Moller M, 2001, J BONE JOINT SURG BR, V83B, P843
   Ng N, 2011, GLOBAL HEALTH ACTION, V4, DOI 10.3402/gha.v4i0.6347
   Nilsson-Helander K., 2009, ACUTE ACHILLES TENDO
   Nilsson-Helander K, 2010, AM J SPORT MED, V38, P2186, DOI 10.1177/0363546510376052
   NISTOR L, 1981, J BONE JOINT SURG AM, V63, P394, DOI 10.2106/00004623-198163030-00012
   Nyyssonen T, 2008, SCAND J SURG, V97, P272, DOI 10.1177/145749690809700312
   Nyyssonen T, 2000, ANN CHIR GYNAECOL, V89, P53
   Pajala A, 2009, HISTOL HISTOPATHOL, V24, P1207, DOI 10.14670/HH-24.1207
   Soroceanu A, 2012, J BONE JOINT SURG AM, V94A, P2136, DOI 10.2106/JBJS.K.00917
   Suchak AA, 2005, FOOT ANKLE INT, V26, P932, DOI 10.1177/107110070502601106
   Suchak AA, 2008, J BONE JOINT SURG AM, V90A, P1876, DOI 10.2106/JBJS.G.01242
   Tallon C, 2001, MED SCI SPORT EXER, V33, P1983, DOI 10.1097/00005768-200112000-00002
   Tuomilehto J, 2013, CURR DIABETES REP, V13, P795, DOI 10.1007/s11892-013-0433-5
   Wallace RGH, 2011, J BONE JOINT SURG BR, V93B, P1362, DOI 10.1302/0301-620X.93B10.26187
   WHO, 1994, 25 NORDIC SEMINAR DE, V10th
   Willits K, 2010, J BONE JOINT SURG AM, V92A, P2767, DOI 10.2106/JBJS.I.01401
   Wndell PE, 2013, CURR DIABETES REV, V9, P342, DOI 10.2174/15733998113099990064
NR 34
TC 165
Z9 171
U1 3
U2 24
PU SAGE PUBLICATIONS INC
PI THOUSAND OAKS
PA 2455 TELLER RD, THOUSAND OAKS, CA 91320 USA
SN 0363-5465
EI 1552-3365
J9 AM J SPORT MED
JI Am. J. Sports Med.
PD OCT
PY 2014
VL 42
IS 10
BP 2419
EP 2423
DI 10.1177/0363546514540599
PG 5
WC Orthopedics; Sport Sciences
WE Science Citation Index Expanded (SCI-EXPANDED)
SC Orthopedics; Sport Sciences
GA AQ4XQ
UT WOS:000342805000020
PM 25056989
DA 2022-07-22
ER

PT J
AU Moller, M
   Kalebo, P
   Tidebrant, G
   Movin, T
   Karlsson, J
AF Moller, M
   Kalebo, P
   Tidebrant, G
   Movin, T
   Karlsson, J
TI The ultrasonographic appearance of the ruptured Achilles tendon during
   healing: a longitudinal evaluation of surgical and nonsurgical
   treatment, with comparisons to MRI appearance
SO KNEE SURGERY SPORTS TRAUMATOLOGY ARTHROSCOPY
LA English
DT Article
DE Achilles tendon; rupture; magnetic resonance imaging ultrasonography;
   prospective
ID ULTRASOUND; DIAGNOSIS; REPAIR
AB This prospective, randomized trial compared healing characteristics of the Achilles tendon after surgical and nonsurgical treatment for complete rupture of the Achilles tendon. Fifty-eight patients were examined by ultrasonography after 6, 12, and 24 months and by magnetic resonance imaging after 12 months. A standardized protocol was used, and the outcome was correlated with clinical findings. Common findings 1 year after the injury were tendon thickening and moderate heterogeneity of the tendon. Peritendinous reactions, edema, and defects were present only in a minority of patients. There were no significant differences between the treatment groups in any of the evaluated parameters apart from the gliding function of the tendon, which was significantly less in the surgically treated group than in the nonsurgically treated group. No correlations were found between the radiological findings and the clinical parameters, such as muscle strength, endurance, and range of motion. We conclude that the roles of ultrasonography and magnetic resonance imaging during the healing process after Achilles tendon rupture are limited, due to a weak correlation with clinical findings.
C1 Gothenburg Univ, Sahlgrens Univ Hosp Ostra, Dept Orthoped, S-41685 Gothenburg, Sweden.
   Gothenburg Univ, Sahlgrens Univ Hosp Ostra, Dept Radiol, S-41685 Gothenburg, Sweden.
   Huddinge Univ Hosp, Dept Orthoped, S-14186 Huddinge, Sweden.
RP Moller, M (通讯作者)，Gothenburg Univ, Sahlgrens Univ Hosp Ostra, Dept Orthoped, S-41685 Gothenburg, Sweden.
EM michael.moller@swipnet.se
CR BLEI CL, 1986, RADIOLOGY, V159, P765, DOI 10.1148/radiology.159.3.3517960
   BURCHHARDT H, 1991, UNFALLCHIRURG, V94, P589
   FORNAGE BD, 1986, RADIOLOGY, V159, P759, DOI 10.1148/radiology.159.3.3517959
   HAGGMARK T, 1986, ORTHOPEDICS, V9, P160
   Hollenberg GM, 2000, SKELETAL RADIOL, V29, P259, DOI 10.1007/s002560050604
   Karjalainen PT, 1996, ACTA RADIOL, V37, P639
   Karjalainen PT, 1997, AM J SPORT MED, V25, P164, DOI 10.1177/036354659702500204
   KEENE JS, 1989, AM J SPORT MED, V17, P333, DOI 10.1177/036354658901700304
   Maffulli N, 1999, J BONE JOINT SURG AM, V81A, P1019, DOI 10.2106/00004623-199907000-00017
   MAFFULLI N, 1990, INT J SPORTS MED, V11, P78, DOI 10.1055/s-2007-1024767
   MARCUS DS, 1989, J COMPUT ASSIST TOMO, V13, P480, DOI 10.1097/00004728-198905000-00021
   Moller M, 2001, J BONE JOINT SURG BR, V83B, P843
   Movin T, 1998, ACTA RADIOL, V39, P126
   QUINN SF, 1987, RADIOLOGY, V164, P767, DOI 10.1148/radiology.164.3.3615878
   REINIG JW, 1985, J COMPUT ASSIST TOMO, V9, P1131, DOI 10.1097/00004728-198511000-00029
   Rominger MB, 1998, ROFO-FORTSCHR RONTG, V168, P27, DOI 10.1055/s-2007-1015178
   RUPP S, 1995, BRIT J RADIOL, V68, P454, DOI 10.1259/0007-1285-68-809-454
   Schweitzer ME, 2000, AM J ROENTGENOL, V175, P613, DOI 10.2214/ajr.175.3.1750613
   Shalabi A, 2001, ACTA RADIOL, V42, P269, DOI 10.1080/028418501127346819
   Soila K, 1999, AM J ROENTGENOL, V173, P323, DOI 10.2214/ajr.173.2.10430128
   Thermann H, 2000, ORTHOPADE, V29, P235
   THERMANN H, 1989, UNFALLCHIRURG, V92, P266
   THOMPSON T C, 1962, J Trauma, V2, P126, DOI 10.1097/00005373-196203000-00003
NR 23
TC 56
Z9 56
U1 0
U2 6
PU SPRINGER
PI NEW YORK
PA 233 SPRING ST, NEW YORK, NY 10013 USA
SN 0942-2056
EI 1433-7347
J9 KNEE SURG SPORT TR A
JI Knee Surg. Sports Traumatol. Arthrosc.
PD JAN
PY 2002
VL 10
IS 1
BP 49
EP 56
DI 10.1007/s001670100245
PG 8
WC Orthopedics; Sport Sciences; Surgery
WE Science Citation Index Expanded (SCI-EXPANDED)
SC Orthopedics; Sport Sciences; Surgery
GA 528MA
UT WOS:000174246500011
PM 11819022
DA 2022-07-22
ER

PT J
AU Cottom, JM
   Baker, JS
   Richardson, PE
   Maker, JM
AF Cottom, James M.
   Baker, Joseph S.
   Richardson, Phillip E.
   Maker, Jared M.
TI Evaluation of a New Knotless Suture Anchor Repair in Acute Achilles
   Tendon Ruptures: A Biomechanical Comparison of Three Techniques
SO JOURNAL OF FOOT & ANKLE SURGERY
LA English
DT Article
DE Achilles repair; Achilles rupture; Achilles tendon; open Achilles
   repair; percutaneous Achilles repair
ID SURGICAL REPAIR; SEPARATION; ENDS
AB Acute ruptures of the Achilles tendon are a common injury, and debate has continued in published studies on how best to treat these injuries. Specifically, controversy exists regarding the surgical approaches for Achilles tendon repair when one considers percutaneous versus open repair. The present study investigated the biomechanical strength of 3 different techniques for Achilles tendon repair in a cadaveric model. A total of 36 specimens were divided into 3 groups, each of which received a different construct. The first group received a traditional Krackow suture repair, the second group was repaired using a jig-assisted percutaneous suture, and the third group received a repair using a jig-assisted percutaneous repair modified with suture anchors placed into the calcaneus. The specimens were tested with cyclical loading and to ultimate failure. Cyclical loading showed a trend toward a stronger repair with the use of suture anchors after 10 cycles (p = .295), 500 cycles (p = .120), and 1000 cycles (p = .040). The ultimate load to failure was greatest in the group repaired with the modified knotless technique using the suture anchors (p = .098). The results of the present study show a clear trend toward a stronger construct in Achilles repair using a knotless suture anchor technique, which might translate to a faster return to activity and be more resistant to an early and aggressive rehabilitation protocol. Further clinical studies are warranted to evaluate this technique in a patient population. (C) 2016 by the American College of Foot and Ankle Surgeons. All rights reserved.
C1 [Cottom, James M.] Florida Orthoped Foot & Ankle Ctr, 2030 Bee Ridge Rd,Suite B, Sarasota, FL 24239 USA.
   [Baker, Joseph S.; Richardson, Phillip E.; Maker, Jared M.] Florida Orthoped Foot & Ankle Ctr, Sarasota, FL USA.
RP Cottom, JM (通讯作者)，Florida Orthoped Foot & Ankle Ctr, 2030 Bee Ridge Rd,Suite B, Sarasota, FL 24239 USA.
EM drcottom@FLOFAC.COM
OI cottom, james/0000-0003-3091-0059
CR Aktas S, 2009, FOOT ANKLE INT, V30, P391, DOI 10.3113/FAI.2009.0391
   Demetracopoulos CA, 2014, FOOT ANKLE INT, V35, P612, DOI 10.1177/1071100714524550
   Ding WG, 2014, FOOT ANKLE SURG, V20, P105, DOI 10.1016/j.fas.2013.12.007
   FITZGIBBONS RE, 1993, AM J SPORT MED, V21, P724, DOI 10.1177/036354659302100516
   Gorschewsky O, 2004, FOOT ANKLE INT, V25, P219, DOI 10.1177/107110070402500405
   Haji A, 2011, FOOT ANKLE INT, V32, P879
   Henriquez H, 2012, CLIN ORTHOP RELAT R, V470, P998, DOI 10.1007/s11999-011-1830-1
   Huffard B, 2008, CLIN BIOMECH, V23, P1158, DOI 10.1016/j.clinbiomech.2008.05.007
   JOZSA L, 1989, AM J SPORT MED, V17, P338, DOI 10.1177/036354658901700305
   Kangas J, 2007, AM J SPORT MED, V35, P59, DOI 10.1177/0363546506293255
   Kanz Brian N, 2014, Foot Ankle Spec, V7, P176, DOI 10.1177/1938640014528041
   Keating JF, 2011, J BONE JOINT SURG BR, V93B, P1071, DOI 10.1302/0301-620X.93B8.25998
   Lee SJ, 2008, FOOT ANKLE INT, V29, P427, DOI 10.3113/FAI.2008.0427
   Lim J, 2001, FOOT ANKLE INT, V22, P559, DOI 10.1177/107110070102200705
   MANDELBAUM BR, 1995, AM J SPORT MED, V23, P392, DOI 10.1177/036354659502300403
   Mortensen NHM, 1999, J BONE JOINT SURG AM, V81A, P983, DOI 10.2106/00004623-199907000-00011
   MORTENSEN NHM, 1992, ORTHOPEDICS, V15, P899
   NYSTROM B, 1983, ACTA ORTHOP SCAND, V54, P620, DOI 10.3109/17453678308992899
   Shepard ME, 2008, FOOT ANKLE INT, V29, P513, DOI 10.3113/FAI.2008.0513
   SOLVEBORN SA, 1994, AM J SPORT MED, V22, P607, DOI 10.1177/036354659402200507
   Suchak AA, 2008, J BONE JOINT SURG AM, V90A, P1876, DOI 10.2106/JBJS.G.01242
NR 21
TC 16
Z9 16
U1 0
U2 7
PU ELSEVIER SCIENCE INC
PI NEW YORK
PA 360 PARK AVE SOUTH, NEW YORK, NY 10010-1710 USA
SN 1067-2516
EI 1542-2224
J9 J FOOT ANKLE SURG
JI J. Foot Ankle Surg.
PD MAY-JUN
PY 2017
VL 56
IS 3
BP 423
EP 427
DI 10.1053/j.jfas.2016.10.012
PG 5
WC Orthopedics; Surgery
WE Science Citation Index Expanded (SCI-EXPANDED)
SC Orthopedics; Surgery
GA EU2UC
UT WOS:000400884400002
PM 28476383
DA 2022-07-22
ER

PT J
AU Jung, HG
   Lee, KB
   Cho, SG
   Yoon, TR
AF Jung, Hong Geun
   Lee, Kenn Bae
   Cho, Sang Gwon
   Yoon, Taek Rim
TI Outcome of Achilles tendon ruptures treated by a limited open technique
SO FOOT & ANKLE INTERNATIONAL
LA English
DT Article
DE Achilles tendon rupture; limited open repair
ID FUNCTIONAL POSTOPERATIVE TREATMENT; OPEN REPAIR; PERCUTANEOUS REPAIR;
   REHABILITATION
AB Background: The optimal surgical management of Achilles tendon ruptures remains a topic of active debate. Recently, many authors have preferred the limited open method because it afforded sufficient visualization to ensure anatomic apposition of disrupted tendon fibers, minimized local blood supply disturbances, guaranteed free tendon movement, and produced excellent cosmesis. We report our initial experience with this technique and review the literature. Materials and Methods: The outcomes of 30 consecutive patients that underwent limited open repair for Achilles tendon rupture using Achillon (R) (Newdeal SA, Lyon, France) from June 2003 to May 2006 were retrospectively reviewed. There were 20 men and 10 women, of average age 38.6 years, and the average followup period was 18.5 months. Twenty of the injuries were sports-related, eight were caused by a fall, and two by a laceration. The clinical results were assessed using patient satisfaction, the ankle-hindfoot scale of the American Orthopaedic Foot and Ankle Society (AOFAS), and the occurrence of complications. Results: At last followup, sixteen patients were very satisfied, 11 were satisfied, and the remaining three were dissatisfied. Mean patient AOFAS score was 93.0 points. Surgical complications noted were re-rupture in two cases, deep infection in one, and sural nerve injury in one. All patients except the three patients with a re-rupture or infection, returned to work 2 months postoperatively and resumed light exercise at 3 months, and previous sporting activities by 6 months. Conclusion: The described limited open repair technique for Achilles tendon ruptures provided excellent cosmetic results, satisfactory functional results, and a high level of patient satisfaction.
C1 [Lee, Kenn Bae; Cho, Sang Gwon; Yoon, Taek Rim] Chonnam Natl Univ, Sch Med, Dept Orthoped Surg, Kwangju 501746, South Korea.
   [Jung, Hong Geun] Konkuk Univ, Sch Med, Dept Orthoped Surg, Seoul, South Korea.
RP Lee, KB (通讯作者)，Chonnam Natl Univ, Sch Med, Dept Orthoped Surg, 5 Hak Dong, Kwangju 501746, South Korea.
EM kblecos@chonnam.ac.kr
CR ARACIL J, 1992, FOOT ANKLE, V13, P350, DOI 10.1177/107110079201300610
   Assal M, 2002, J BONE JOINT SURG AM, V84A, P161, DOI 10.2106/00004623-200202000-00001
   BRADLEY JP, 1990, AM J SPORT MED, V18, P188, DOI 10.1177/036354659001800213
   Buchgraber A, 1997, CLIN ORTHOP RELAT R, P113
   Calder JDF, 2005, BRIT J SPORT MED, V39, P857, DOI 10.1136/bjsm.2004.017509
   CARTER TR, 1992, AM J SPORT MED, V20, P459, DOI 10.1177/036354659202000417
   Ceccarelli F, 2007, CLIN ORTHOP RELAT R, P188, DOI 10.1097/BLO.0b013e3180396f07
   CETTI R, 1993, AM J SPORT MED, V21, P791, DOI 10.1177/036354659302100606
   Haji A, 2004, FOOT ANKLE INT, V25, P215, DOI 10.1177/107110070402500404
   HATTRUP SJ, 1985, FOOT ANKLE, V6, P34, DOI 10.1177/107110078500600107
   KAKIUCHI M, 1995, J BONE JOINT SURG BR, V77B, P60, DOI 10.1302/0301-620X.77B1.7822398
   KITAOKA HB, 1994, FOOT ANKLE INT, V15, P349, DOI 10.1177/107110079401500701
   Leppilahti J, 1996, ACTA ORTHOP SCAND, V67, P277, DOI 10.3109/17453679608994688
   MA GWC, 1977, CLIN ORTHOP RELAT R, P247
   Maffulli N, 1999, J BONE JOINT SURG AM, V81A, P1019, DOI 10.2106/00004623-199907000-00017
   MANDELBAUM BR, 1995, AM J SPORT MED, V23, P392, DOI 10.1177/036354659502300403
   Rebeccato A, 2001, J Foot Ankle Surg, V40, P188
   Rippstein Pascal F, 2002, Foot Ankle Clin, V7, P611, DOI 10.1016/S1083-7515(02)00040-2
NR 18
TC 21
Z9 28
U1 0
U2 3
PU SAGE PUBLICATIONS INC
PI THOUSAND OAKS
PA 2455 TELLER RD, THOUSAND OAKS, CA 91320 USA
SN 1071-1007
EI 1944-7876
J9 FOOT ANKLE INT
JI Foot Ankle Int.
PD AUG
PY 2008
VL 29
IS 8
BP 803
EP 807
DI 10.3113/FAI.2008.0803
PG 5
WC Orthopedics
WE Science Citation Index Expanded (SCI-EXPANDED)
SC Orthopedics
GA 331VL
UT WOS:000258040600005
PM 18752778
DA 2022-07-22
ER

PT J
AU Kuskucu, M
   Mahirogullari, M
   Solakoglu, C
   Akmaz, I
   Rodop, O
   Kiral, A
   Kaplan, H
AF Kuskucu, M
   Mahirogullari, M
   Solakoglu, C
   Akmaz, I
   Rodop, O
   Kiral, A
   Kaplan, H
TI Treatment of rupture of the Achilles tendon with fibrin sealant
SO FOOT & ANKLE INTERNATIONAL
LA English
DT Article
DE Achilles tendon; fibrin sealant; rupture
ID NONOPERATIVE MANAGEMENT; FUNCTIONAL TREATMENT; REPAIR
AB Background: The optimal management strategy for acute Achilles tendon ruptures is controversial. These injuries historically were treated by nonoperative methods (cast immobilization, bandaging); however, operative repair of the ruptured tendon has become popular. Methods: Thirty-two patients who had rupture of the Achilles tendon were treated operatively with use of fibrin sealant, and clinical and functional performance measures were assessed after a mean followup of at least 6 months between November, 1998, and July, 2003. All of the patients were male. Average age was 38.18 (30 to 45) years. All of the patients were followed for at least 18 months after surgery. Average followup time was 22.4 (18 to 56) months. We evaluated all patients according to the scoring system of Thermann et al. Results: Our results were excellent in 24 patients and good in eight patients. One patient had rerupture 3 weeks after surgery. Conclusion: Fibrin sealants are biologically compatible, hemostatic agents derived from human plasma that can be used instead of suture or suture support. We think that the treatment of rupture of the Achilles tendon with fibrin sealant is a useful treatment, and there is less risk of complications, such as deep infection, than in other operative procedures. We had no wound closure problems, the incision size was small, and the operating time was short. However, it must be remembered that the risk of disease transfer by fibrin sealant application is still present.
C1 Gata Haydarpasa Training Hosp, Dept Orthoped & Traumatol, TR-34668 Istanbul, Turkey.
RP Mahirogullari, M (通讯作者)，Gata Haydarpasa Training Hosp, Dept Orthoped & Traumatol, TR-34668 Istanbul, Turkey.
EM mahirogullari@yahoo.com
RI Mahirogullari, Mahir/AAA-4742-2020; Rodop, Osman/AAO-3436-2020
OI Rodop, Osman/0000-0002-4209-4338
CR Bhandari M, 2002, CLIN ORTHOP RELAT R, P190
   Clark RAF, 2000, EXPERT OPIN INV DRUG, V9, P2371, DOI 10.1517/13543784.9.10.2371
   EDER G, 1986, FIBRIN SEALANT OPERA, V1, P51
   HAAS F, 1987, ZBL CHIR, V112, P99
   KLEIN W, 1991, Chirurgia degli Organi di Movimento, V76, P223
   Kocher MS, 2002, AM J SPORT MED, V30, P783, DOI 10.1177/03635465020300060501
   MA GWC, 1977, CLIN ORTHOP RELAT R, P247
   Maffulli N, 1999, J BONE JOINT SURG AM, V81A, P1019, DOI 10.2106/00004623-199907000-00017
   MANDELBAUM BR, 1995, AM J SPORT MED, V23, P392, DOI 10.1177/036354659502300403
   Pajala A, 2002, J BONE JOINT SURG AM, V84A, P2016, DOI 10.2106/00004623-200211000-00017
   PFLUGER H, 1986, FIBRIN SEALANT OPERA, V6, P39
   REDAELLI C, 1992, CHIRURG, V63, P572
   Thermann H, 1995, UNFALLCHIRURG, V98, P507
   THERMANN H, 1995, UNFALLCHIRURG, V98, P21
   Winter E, 1998, ARCH ORTHOP TRAUM SU, V117, P364, DOI 10.1007/s004020050267
   Wong J, 2002, AM J SPORT MED, V30, P565, DOI 10.1177/03635465020300041701
NR 16
TC 6
Z9 7
U1 0
U2 1
PU SAGE PUBLICATIONS INC
PI THOUSAND OAKS
PA 2455 TELLER RD, THOUSAND OAKS, CA 91320 USA
SN 1071-1007
EI 1944-7876
J9 FOOT ANKLE INT
JI Foot Ankle Int.
PD OCT
PY 2005
VL 26
IS 10
BP 826
EP 831
DI 10.1177/107110070502601007
PG 6
WC Orthopedics
WE Science Citation Index Expanded (SCI-EXPANDED)
SC Orthopedics
GA 970NY
UT WOS:000232318100007
PM 16221455
DA 2022-07-22
ER

PT J
AU Shim, VB
   Fernandez, JW
   Gamage, PB
   Regnery, C
   Smith, DW
   Gardiner, BS
   Lloyd, DG
   Besier, TF
AF Shim, Vickie B.
   Fernandez, Justin W.
   Gamage, Prasad B.
   Regnery, Camille
   Smith, David W.
   Gardiner, Bruce S.
   Lloyd, David G.
   Besier, Thor F.
TI Subject-specific finite element analysis to characterize the influence
   of geometry and material properties in Achilles tendon rupture
SO JOURNAL OF BIOMECHANICS
LA English
DT Article
DE Achilles tendon; Finite element analysis; Tendon rupture; Material
   property optimization; Subject-specific finite element analysis
ID MEDIAL COLLATERAL LIGAMENT; CROSS-SECTIONAL AREA; MECHANICAL-PROPERTIES;
   MUSCLE; MODEL; LOCOMOTION; FAILURE; FASCIA; FIBERS
AB Achilles tendon injuries including rupture are one of the most frequent musculoskeletal injuries, but the mechanisms for these injuries are still not fully understood. Previous in vivo and experimental studies suggest that tendon rupture mainly occurs in the tendon mid-section and predominantly more in men than women due to reasons yet to be identified. Therefore we aimed to investigate possible mechanisms for tendon rupture using finite element (FE) analysis. Specifically, we have developed a framework for generating subject-specific FE models of human Achilles tendon. A total of ten 3D FE models of human Achilles tendon were generated. Subject-specific geometries were obtained using ultrasound images and a mesh morphing technique called Free Form Deformation. Tendon material properties were obtained by performing material optimization that compared and minimized difference in uniaxial tension experimental results with model predictions. Our results showed that both tendon geometry and material properties are highly subject-specific. This subject-specificity was also evident in our rupture predictions as the locations and loads of tendon ruptures were different in all specimens tested. A parametric study was performed to characterize the influence of geometries and material properties on tendon rupture. Our results showed that tendon rupture locations were dependent largely on geometry while rupture loads were more influenced by tendon material properties. Future work will investigate the role of microstructural properties of the tissue on tendon rupture and degeneration by using advanced material descriptions. (C) 2014 Elsevier Ltd. All rights reserved.
C1 [Shim, Vickie B.; Fernandez, Justin W.; Gamage, Prasad B.; Regnery, Camille; Besier, Thor F.] Univ Auckland, Auckland Bioengn Inst, Auckland 1, New Zealand.
   [Shim, Vickie B.; Lloyd, David G.] Griffith Univ, Griffith Hlth Inst, Ctr Musculoskeletal Res, Gold Coast, Qld, Australia.
   [Fernandez, Justin W.; Besier, Thor F.] Univ Auckland, Dept Engn Sci, Auckland 1, New Zealand.
   [Smith, David W.; Gardiner, Bruce S.] Univ Western Australia, Sch Comp Sci & Software Engn, Nedlands, WA 6009, Australia.
   [Lloyd, David G.] Univ Western Australia, Sch Sport Sci Exercise & Hlth, Nedlands, WA 6009, Australia.
   [Regnery, Camille] MATMECH Bordeaux, ENSEIRB, Bordeaux, France.
RP Shim, VB (通讯作者)，Auckland Bioengn Inst, 70 Symonds St, Auckland, New Zealand.
EM v.shim@auckland.ac.nz
RI Besier, Thor F/P-6425-2019; Gardiner, Bruce/C-8593-2012; Lloyd,
   David/AAH-6066-2020; Smith, David W/H-5856-2014
OI Lloyd, David/0000-0002-0824-9682; Smith, David W/0000-0003-4864-0288;
   Shim, Vickie Bo Kyung/0000-0002-1680-4287; Babarenda Gamage, Thiranja
   Prasad/0000-0001-5109-0219; Gardiner, Bruce/0000-0001-8770-5908; Besier,
   Thor/0000-0003-0818-7554
FU Australian Research Council Linkage Grant [LP110100581]
FX This work was funded by the Australian Research Council Linkage Grant
   (LP110100581). The authors would like to thank Prof. Wren for proving
   the experimental dataset and for the guidance of Professor Ming Hao
   Zheng, Professor Jiake Xu, Professor Brett Kirk, Professor Peter Hunter,
   Dr. Paul Anderson, Dr. Allan Wang, and Assistant Prof. Jonas Rubenson in
   this research.
CR Bandak FA, 2001, INT J SOLIDS STRUCT, V38, P1681, DOI 10.1016/S0020-7683(00)00129-3
   Bayod J, 2012, J BIOMECH, V45, P1219, DOI 10.1016/j.jbiomech.2012.01.043
   Blemker SS, 2005, ANN BIOMED ENG, V33, P661, DOI 10.1007/s10439-005-1433-7
   BUTLER DL, 1984, J BIOMECH, V17, P579, DOI 10.1016/0021-9290(84)90090-3
   Cheung JTM, 2006, CLIN BIOMECH, V21, P194, DOI 10.1016/j.clinbiomech.2005.09.016
   Fernandez J., 2012, PATIENT SPECIFIC COM, V5, DOI [DOI 10.1007/978-94-007-4552-0_2, 10.1007/978-94-007-4552-0_2]
   Fernandez JW, 2004, BIOMECH MODEL MECHAN, V2, P139, DOI [10.1007/s10237-003-0036-1, 10.1007/S10237-003-0036-1]
   Fiorentino NM, 2014, MED SCI SPORT EXER, V46, P776, DOI 10.1249/MSS.0000000000000172
   Gamage TPB, 2011, INT J NUMER METH BIO, V27, P391, DOI 10.1002/cnm.1429
   Gardiner JC, 2003, J ORTHOP RES, V21, P1098, DOI 10.1016/S0736-0266(03)00113-X
   Hansen P, 2003, J APPL PHYSIOL, V95, P2375, DOI 10.1152/japplphysiol.00503.2003
   Hess Gregory William, 2010, Foot Ankle Spec, V3, P29, DOI 10.1177/1938640009355191
   Houghton L, 2013, J APPL BIOMECH, V29, P453, DOI 10.1123/jab.29.4.453
   Hunter P, 2002, PFLUG ARCH EUR J PHY, V445, P1, DOI 10.1007/s00424-002-0890-1
   Jarvinen Tero A H, 2005, Foot Ankle Clin, V10, P255, DOI 10.1016/j.fcl.2005.01.013
   JOZSA L, 1989, MATRIX, V9, P140, DOI 10.1016/S0934-8832(89)80032-0
   Kasashima Y., 2002, EQUINE VET J S, pS264
   Kongsgaard M, 2005, J APPL PHYSIOL, V99, P1965, DOI 10.1152/japplphysiol.00384.2005
   Lavagnino M, 2008, BIOMECH MODEL MECHAN, V7, P405, DOI 10.1007/s10237-007-0104-z
   LEMM M, 1992, J AM PODIAT MED ASSN, V82, P482, DOI 10.7547/87507315-82-9-482
   Lichtwark GA, 2007, J BIOMECH, V40, P1768, DOI 10.1016/j.jbiomech.2006.07.025
   Lichtwark GA, 2006, J EXP BIOL, V209, P4379, DOI 10.1242/jeb.02434
   Maganaris CN, 2006, MUSCLE NERVE, V33, P85, DOI 10.1002/mus.20441
   Magnusson SP, 2003, EUR J APPL PHYSIOL, V90, P549, DOI 10.1007/s00421-003-0865-8
   Munro JT, 2013, J BIOMECH, V46, P2529, DOI 10.1016/j.jbiomech.2013.07.038
   Oberhofer K, 2010, CLIN BIOMECH, V25, P88, DOI 10.1016/j.clinbiomech.2009.09.007
   Pennisi E, 2002, SCIENCE, V295, P1011, DOI 10.1126/science.295.5557.1011
   Pioletti D.P., 1998, COMPUT METHOD BIOMEC, V21, P221
   Quapp KM, 1998, J BIOMECH ENG-T ASME, V120, P757, DOI 10.1115/1.2834890
   Reese SP, 2010, J BIOMECH, V43, P1394, DOI 10.1016/j.jbiomech.2010.01.004
   Reeves ND, 2005, MUSCLE NERVE, V31, P355, DOI 10.1002/mus.20275
   Shim V, 2010, J BIOMECH, V43, P1635, DOI 10.1016/j.jbiomech.2010.01.017
   Shim VB, 2012, INT ORTHOP, V36, P1363, DOI 10.1007/s00264-012-1513-x
   Shim VB, 2011, IEEE T BIO-MED ENG, V58, P3532, DOI 10.1109/TBME.2011.2165955
   Stevens C, 2003, J BIOMECH, V36, P737, DOI 10.1016/S0021-9290(02)00452-9
   Theobald P, 2005, INJURY, V36, P1267, DOI 10.1016/j.injury.2005.02.012
   Wakabayashi I, 2003, J SHOULDER ELB SURG, V12, P612, DOI 10.1016/S1058-2746(03)00214-3
   Weiss JA, 2001, CRIT REV BIOMED ENG, V29, P303, DOI 10.1615/CritRevBiomedEng.v29.i3.20
   Weiss JA, 1996, COMPUT METHOD APPL M, V135, P107, DOI 10.1016/0045-7825(96)01035-3
   Wren TAL, 2001, CLIN BIOMECH, V16, P245, DOI 10.1016/S0268-0033(00)00089-9
   Wren TAL, 2003, ANN BIOMED ENG, V31, P710, DOI 10.1114/1.1569267
   Wren TAL, 2001, CLIN BIOMECH, V16, P529, DOI 10.1016/S0268-0033(01)00033-X
NR 42
TC 42
Z9 42
U1 0
U2 13
PU ELSEVIER SCI LTD
PI OXFORD
PA THE BOULEVARD, LANGFORD LANE, KIDLINGTON, OXFORD OX5 1GB, OXON, ENGLAND
SN 0021-9290
EI 1873-2380
J9 J BIOMECH
JI J. Biomech.
PD NOV 28
PY 2014
VL 47
IS 15
BP 3598
EP 3604
DI 10.1016/j.jbiomech.2014.10.001
PG 7
WC Biophysics; Engineering, Biomedical
WE Science Citation Index Expanded (SCI-EXPANDED)
SC Biophysics; Engineering
GA AU7UC
UT WOS:000345805500002
PM 25458149
DA 2022-07-22
ER

PT J
AU Reito, A
   Logren, HL
   Ahonen, K
   Nurmi, H
   Paloneva, J
AF Reito, Aleksi
   Logren, Hanna-Liina
   Ahonen, Katri
   Nurmi, Heikki
   Paloneva, Juha
TI Risk Factors for Failed Nonoperative Treatment and Rerupture in Acute
   Achilles Tendon Rupture
SO FOOT & ANKLE INTERNATIONAL
LA English
DT Article
DE Achilles; nonoperative; rupture; Achilles tendon; rerupture
ID NONSURGICAL TREATMENTS; EARLY WEIGHTBEARING; CONTROLLED-TRIALS;
   WEIGHT-BEARING; METAANALYSIS; MANAGEMENT; SURGERY
AB Background: Nonoperative treatment is feasible in most patients with acute Achilles tendon rupture. Risk factors associated with failed nonoperative treatment are poorly understood. We investigated risk factors associated with rerupture after nonoperative treatment and otherwise failed nonoperative treatment of Achilles tendon rupture.
   Methods: All patients diagnosed with acute Achilles tendon rupture between January 2009 and June 2016 and who underwent 8 weeks of nonoperative treatment with functional rehabilitation were included in the study. Patients with rerupture or otherwise failed nonoperative treatment were identified retrospectively. Time to rerupture and association of age, sex, time from injury, diabetes, and visits to the physiotherapist for cases of reruptures and otherwise failed nonoperative treatment were investigated. A total of 210 patients were included in the study.
   Results: Fifteen patients sustained a rerupture. Rerupture incidence was 7.1%. Incidence of late reruptures, those occurring after return to daily activities at 12 weeks, was 1.9%. Six patients had otherwise failed nonoperative treatment. Median time to rerupture was 23 days (6 to 61) after the end of the treatment. The incidence of all-cause failure was 10.0%. Male gender was associated with reruptures (P = .013) and failed nonoperative treatment for any reason (P = .029).
   Conclusion: It is important to highlight the increased risk of rerupture in male patients during the first month after the end of the nonoperative treatment. Age alone, even in male patients, was a poor indication for operative treatment since it did not predict early failure. Further studies will hopefully clarify the influence of activity level on the risk of rerupture.
   Level of Evidence: Level IV, retrospective case series.
C1 [Reito, Aleksi; Logren, Hanna-Liina; Ahonen, Katri; Nurmi, Heikki; Paloneva, Juha] Cent Finland Hosp, Keskussairaalantie 19, Jyvaskyla 40620, Keski Suomi, Finland.
   [Logren, Hanna-Liina] Univ Eastern Finland, Kuopio, Finland.
RP Reito, A (通讯作者)，Cent Finland Hosp, Keskussairaalantie 19, Jyvaskyla 40620, Keski Suomi, Finland.
EM aleksi.reito@fimnet.fi
RI Paloneva, Juha/K-2419-2019; Reito, Aleksi/AAC-3832-2020
OI Paloneva, Juha/0000-0003-3340-9390; Reito, Aleksi/0000-0002-6903-6461
CR American Academy of Orthopaedic Surgeons, 2009, DIAGN TREATM AC ACH
   Barfod KW, 2013, J FOOT ANKLE SURG, V52, P629, DOI 10.1053/j.jfas.2013.04.012
   Barfod KW, 2014, J BONE JOINT SURG AM, V96A, P1497, DOI 10.2106/JBJS.M.01273
   Ecker TM, 2016, AM J SPORT MED, V44, P1004, DOI 10.1177/0363546515623501
   Hutchison AM, 2015, BONE JOINT J, V97B, P510, DOI 10.1302/0301-620X.97B4.35314
   Huttunen TT, 2014, AM J SPORT MED, V42, P2419, DOI 10.1177/0363546514540599
   Ingvar J, 2005, ACTA ORTHOP, V76, P597, DOI 10.1080/17453670510041619
   Khan RJK, 2005, J BONE JOINT SURG AM, V87A, P2202, DOI 10.2106/JBJS.D.03049
   Kotnis R, 2006, AM J SPORT MED, V34, P1395, DOI 10.1177/0363546506288678
   Lantto I, 2016, AM J SPORT MED, V44, P2406, DOI 10.1177/0363546516651060
   Mattila VM, 2015, BRIT J SPORT MED, V49, P1084, DOI 10.1136/bjsports-2013-092756
   Mattila VM, 2011, J TRAUMA, V71, P939, DOI 10.1097/TA.0b013e3182231af9
   Nilsson-Helander K, 2010, AM J SPORT MED, V38, P2186, DOI 10.1177/0363546510376052
   Schepull T, 2012, SCAND J MED SCI SPOR, V22, P18, DOI 10.1111/j.1600-0838.2010.01154.x
   Silbernagel KG, 2012, AM J SPORT MED, V40, P1564, DOI 10.1177/0363546512447926
   Soroceanu A, 2012, J BONE JOINT SURG AM, V94A, P2136, DOI 10.2106/JBJS.K.00917
   van der Eng DM, 2013, J FOOT ANKLE SURG, V52, P622, DOI 10.1053/j.jfas.2013.03.027
   Wallace RGH, 2011, J BONE JOINT SURG BR, V93B, P1362, DOI 10.1302/0301-620X.93B10.26187
   Wang D, 2015, FOOT ANKLE SURG, V21, P250, DOI 10.1016/j.fas.2015.01.009
   Wilkins R, 2012, AM J SPORT MED, V40, P2154, DOI 10.1177/0363546512453293
   Young SW, 2014, J BONE JOINT SURG AM, V96A, P1073, DOI 10.2106/JBJS.M.00248
NR 21
TC 11
Z9 12
U1 0
U2 2
PU SAGE PUBLICATIONS INC
PI THOUSAND OAKS
PA 2455 TELLER RD, THOUSAND OAKS, CA 91320 USA
SN 1071-1007
EI 1944-7876
J9 FOOT ANKLE INT
JI Foot Ankle Int.
PD JUN
PY 2018
VL 39
IS 6
BP 694
EP 703
DI 10.1177/1071100717754042
PG 10
WC Orthopedics
WE Science Citation Index Expanded (SCI-EXPANDED)
SC Orthopedics
GA GH9YO
UT WOS:000434026300009
PM 29380628
DA 2022-07-22
ER

PT J
AU Ho, G
   Tantigate, D
   Kirschenbaum, J
   Greisberg, JK
   Vosseller, JT
AF Ho, Gavin
   Tantigate, Direk
   Kirschenbaum, Josh
   Greisberg, Justin K.
   Vosseller, J. Turner
TI Increasing age in Achilles rupture patients over time
SO INJURY-INTERNATIONAL JOURNAL OF THE CARE OF THE INJURED
LA English
DT Article
DE Achilles tendon rupture age; Male/female ratio
ID NONAUGMENTED SURGICAL REPAIR; PROSPECTIVE RANDOMIZED-TRIAL; MINIMALLY
   INVASIVE SURGERY; EARLY WEIGHT-BEARING; VS. OPEN REPAIR; TENDON-RUPTURE;
   NONOPERATIVE TREATMENT; NONSURGICAL TREATMENT; PERCUTANEOUS REPAIR;
   FOLLOW-UP
AB Background: The changing demographics of Achilles tendon rupture (ATR) patients have not fully been investigated. However, there has been a general suspicion that this injury is occurring in an increasingly older population, in terms of mean age. The aim of this study was to objectively show an increase in age in Achilles tendon rupture patients over time.
   Methods: Published literature on Achilles tendon ruptures was searched for descriptive statistics on the demographics of patients in the studies, specifically mean and median age of Achilles tendon rupture patients, gender ratio, percentage of athletics-related injuries, percentage of smokers, and BMI. Linear regression analyses were performed to determine the trend of patient demographics over time. A Welch one-way ANOVA was carried out to identify any possible differences in data obtained from different types of studies.
   Results: The patient demographics from 142 studies were recorded, with all ATR injuries occurring between the years 1953 and 2014. There was no significant difference in the mean age data reported by varying study types, i.e. randomized controlled trial, cohort study, case series, etc. (P = 0.182). There was a statistically significant rise in mean age of ATR patients over time (P < 0.0005). There was also a statistically significant drop in percentage of male ATR patients (P = 0.02). There is no significant trend for percentage of athletics-related injuries, smoking or BMI.
   Conclusion: Since 1953 to present day, the mean age at which ATR occurs has been increasing by at least 0.721 years every five years. In the same time period, the percentage of female study patients with ATR injuries has also been increasing by at least 0.6% every five years. (C) 2017 Elsevier Ltd. All rights reserved.
C1 [Ho, Gavin; Tantigate, Direk; Kirschenbaum, Josh; Greisberg, Justin K.; Vosseller, J. Turner] Columbia Univ, Med Ctr, New York Presbyterian Hosp, 622 West 168th St,PH-11, New York, NY 10032 USA.
RP Vosseller, JT (通讯作者)，Columbia Univ, Med Ctr, New York Presbyterian Hosp, 622 West 168th St,PH-11, New York, NY 10032 USA.
EM jtv2111@columbia.edu
RI Vosseller, J. Turner/S-2479-2019
OI Vosseller, J. Turner/0000-0002-9639-3739
CR Aktas Seref, 2007, J Foot Ankle Surg, V46, P336, DOI 10.1053/j.jfas.2007.06.006
   Aktas S, 2009, FOOT ANKLE INT, V30, P391, DOI 10.3113/FAI.2009.0391
   Aroen A, 2004, SCAND J MED SCI SPOR, V14, P30, DOI 10.1111/j.1600-0838.2004.00344.x
   Arts DGT, 2002, J AM MED INFORM ASSN, V9, P600, DOI 10.1197/jamia.M1087
   Assal M, 2002, J BONE JOINT SURG AM, V84A, P161, DOI 10.2106/00004623-200202000-00001
   Aujla R, 2016, FOOT ANKLE SURG, V22, P254, DOI 10.1016/j.fas.2015.11.003
   Badalihan A, 2015, J FOOT ANKLE SURG, V54, P153, DOI 10.1053/j.jfas.2014.12.002
   Barfod KW, 2015, KNEE SURG SPORT TR A, V23, P3398, DOI 10.1007/s00167-014-3175-2
   Barfod KW, 2014, J BONE JOINT SURG AM, V96A, P1497, DOI 10.2106/JBJS.M.01273
   Bergkvist D, 2012, J BONE JOINT SURG AM, V94A, P1229, DOI 10.2106/JBJS.J.01601
   BESKIN JL, 1987, AM J SPORT MED, V15, P1, DOI 10.1177/036354658701500101
   BOYDEN EM, 1995, CLIN ORTHOP RELAT R, P150
   BRADLEY JP, 1990, AM J SPORT MED, V18, P188, DOI 10.1177/036354659001800213
   Bruggeman NB, 2004, CLIN ORTHOP RELAT R, P63, DOI 10.1097/01.blo.0000144475.05543.e7
   Bryant AL, 2008, J APPL PHYSIOL, V105, P1035, DOI 10.1152/japplphysiol.01281.2007
   Buchgraber A, 1997, CLIN ORTHOP RELAT R, P113
   Calder JDF, 2005, BRIT J SPORT MED, V39, P857, DOI 10.1136/bjsm.2004.017509
   Carmont MR, 2015, ASIA-PAC J SPORT MED, V2, P49, DOI 10.1016/j.asmart.2014.12.002
   Carmont MR, ORTHOP J SPORTS MED
   Carpenter LJ, WOMEN INTERCOLLEGIAT
   CETTI R, 1994, CLIN ORTHOP RELAT R, P155
   CETTI R, 1981, ACTA ORTHOP SCAND, V52, P675, DOI 10.3109/17453678108992166
   CETTI R, 1983, CLIN ORTHOP RELAT R, P204
   CETTI R, 1993, AM J SPORT MED, V21, P791, DOI 10.1177/036354659302100606
   Cetti R, 1982, Br J Sports Med, V16, P230
   CETTI R, 1993, CLIN ORTHOP RELAT R, P215
   Chan Alexander Pak-Hin, 2011, Sports Med Arthrosc Rehabil Ther Technol, V3, P32, DOI 10.1186/1758-2555-3-32
   Chan S. K., 2008, Hong Kong Medical Journal, V14, P255
   Cheslock J., WOMENS SPORTS FDN WH, P32
   Clayton RAE, 2008, INJURY, V39, P1338, DOI 10.1016/j.injury.2008.06.021
   Cooper MT, 2015, CLIN SPORT MED, V34, P595, DOI 10.1016/j.csm.2015.06.001
   Corradino B, 2015, INJURY, V46, P1637, DOI 10.1016/j.injury.2015.05.014
   Costa ML, 2006, J BONE JOINT SURG BR, V88B, P69, DOI 10.1302/0301-620X.88B1.16549
   Cretnik A, 2005, AM J SPORT MED, V33, P1369, DOI 10.1177/0363546504271501
   Cukelj F, 2015, INJURY, V46, pS137, DOI 10.1016/j.injury.2015.10.070
   Davis JJ, 1999, MIL MED, V164, P872, DOI 10.1093/milmed/164.12.872
   Ding WG, 2013, J FOOT ANKLE SURG, V52, P143, DOI 10.1053/j.jfas.2012.12.006
   Docking SI, 2016, SCAND J MED SCI SPOR, V26, P557, DOI 10.1111/sms.12469
   Domeij-Arverud E, 2015, BONE JOINT J, V97B, P675, DOI 10.1302/0301-620X.97B5.34581
   Ebinesan AD, 2008, DISABIL REHABIL, V30, P1721, DOI 10.1080/09638280701786815
   Eime RM, 2016, BMC SPORTS SCI MED R, V8, DOI 10.1186/s13102-016-0031-3
   Erickson BJ, 2014, ORTHOP J SPORT MED, V2, P2005
   Fahlstrom M, 1998, SCAND J MED SCI SPOR, V8, P145
   Fine LG, 2003, BRIT MED J, V326, P25, DOI 10.1136/bmj.326.7379.25
   Finni T, 2006, CLIN BIOMECH, V21, P67, DOI 10.1016/j.clinbiomech.2005.08.007
   FOX JM, 1975, CLIN ORTHOP RELAT R, P221
   FRUENSGAARD S, 1992, INT ORTHOP, V16, P33
   Fu CY, 2015, SURG-J R COLL SURG E, V13, P207, DOI 10.1016/j.surge.2014.03.010
   Ganestam A, 2016, KNEE SURG SPORT TR A, V24, P3730, DOI 10.1007/s00167-015-3544-5
   Geremia JM, 2015, CLIN BIOMECH, V30, P485, DOI 10.1016/j.clinbiomech.2015.03.005
   Gigante A, 2008, KNEE SURG SPORT TR A, V16, P204, DOI 10.1007/s00167-007-0448-z
   Groetelaers RPTGC, 2014, FOOT ANKLE INT, V35, P771, DOI 10.1177/1071100714536167
   Guillo S, 2013, SURG-J R COLL SURG E, V11, P14, DOI 10.1016/j.surge.2011.12.002
   HAGGMARK T, 1986, ORTHOPEDICS, V9, P160
   Ham SA, 2009, J PHYS ACT HEALTH, V6, P6, DOI 10.1123/jpah.6.1.6
   Heikkinen J, 2016, J BONE JOINT SURG AM, V98, P85, DOI 10.2106/JBJS.O.00496
   Henriquez H, 2012, CLIN ORTHOP RELAT R, V470, P998, DOI 10.1007/s11999-011-1830-1
   Hess Gregory William, 2010, Foot Ankle Spec, V3, P29, DOI 10.1177/1938640009355191
   Heyes G, 2014, ANN ORTHOP RHEUMATOL, V2, P1017
   Houshian S, 1998, INJURY, V29, P651, DOI 10.1016/S0020-1383(98)00147-8
   Hsu AR, 2015, FOOT ANKLE INT, V36, P1279, DOI 10.1177/1071100715589632
   Huang XW, 2015, J FOOT ANKLE SURG, V54, P1004, DOI 10.1053/j.jfas.2014.12.029
   Huttunen TT, 2014, AM J SPORT MED, V42, P2419, DOI 10.1177/0363546514540599
   INGLIS AE, 1981, CLIN ORTHOP RELAT R, P160
   Jackson G, 2013, ORTHOPEDICS, V36, pE1053, DOI 10.3928/01477447-20130724-23
   Jallageas R, 2013, ORTHOP TRAUMATOL-SUR, V99, P577, DOI 10.1016/j.otsr.2013.03.024
   JOZSA L, 1989, AM J SPORT MED, V17, P338, DOI 10.1177/036354658901700305
   Kaalund S, 1989, Br J Sports Med, V23, P102
   KAKIUCHI M, 1995, J BONE JOINT SURG BR, V77B, P60, DOI 10.1302/0301-620X.77B1.7822398
   Kangas J, 2003, J TRAUMA, V54, P1171, DOI 10.1097/01.TA.0000047945.20863.A2
   Kangas J, 2007, AM J SPORT MED, V35, P59, DOI 10.1177/0363546506293255
   Kaniki N, 2014, ARTHROSCOPY, V30, P1139, DOI 10.1016/j.arthro.2014.04.086
   Karabinas Panagiotis K, 2014, Eur J Orthop Surg Traumatol, V24, P607, DOI 10.1007/s00590-013-1350-7
   Keating JF, 2011, J BONE JOINT SURG BR, V93B, P1071, DOI 10.1302/0301-620X.93B8.25998
   Keller A, 2014, AM J SPORT MED, V42, P731, DOI 10.1177/0363546513511418
   KELLER J, 1984, ACTA ORTHOP SCAND, V55, P548, DOI 10.3109/17453678408992957
   Kerkhoffs GMMJ, 2002, ARCH ORTHOP TRAUM SU, V122, P102, DOI 10.1007/s004020100312
   KOBORI M, 1989, CLIN ORTHOP RELAT R, P306
   Kocaoglu B, 2015, FOOT ANKLE INT, V36, P691, DOI 10.1177/1071100714568868
   Kolodziej L, 2013, INT ORTHOP, V37, P625, DOI 10.1007/s00264-012-1737-9
   Korkmaz M, 2015, J ORTHOP SCI, V20, P513, DOI 10.1007/s00776-015-0710-z
   Labib SA, 2016, J FOOT ANKLE SURG, V55, P39, DOI 10.1053/j.jfas.2015.04.024
   Lantto I, 2015, SCAND J MED SCI SPOR, V25, pE133, DOI 10.1111/sms.12253
   Lapidus LJ, 2007, J ORTHOP TRAUMA, V21, P52, DOI 10.1097/01.bot.0000250741.65003.14
   Leppilahti J, 1996, ACTA ORTHOP SCAND, V67, P277, DOI 10.3109/17453679608994688
   Levi N, 1997, INJURY, V28, P311, DOI 10.1016/S0020-1383(96)00200-8
   LILDHOLDT T, 1976, ACTA ORTHOP SCAND, V47, P454
   Lim J, 2001, FOOT ANKLE INT, V22, P559, DOI 10.1177/107110070102200705
   Lorimer AV, 2016, SPORTS MED, V46, P1921, DOI 10.1007/s40279-016-0526-9
   Lorimer AV, 2014, SPORTS MED, V44, P1459, DOI 10.1007/s40279-014-0209-3
   Macias R, 2014, BMC PUBLIC HEALTH, V14, DOI 10.1186/1471-2458-14-972
   Macquet AJ, 2011, ANZ J SURG, V81, P619, DOI 10.1111/j.1445-2197.2010.05532.x
   Maffulli N, 2003, AM J SPORT MED, V31, P692, DOI 10.1177/03635465030310051001
   Maffulli N, 2003, J SPORT MED PHYS FIT, V43, P367
   Maffulli N, 2005, CLIN J SPORT MED, V15, P56, DOI 10.1097/01.jsm.0000152714.05097.ef
   Maffulli N, 2002, MED SCI SPORT EXER, V34, P1057, DOI 10.1097/00005768-200207000-00001
   Maffulli N, 1998, AM J SPORT MED, V26, P266, DOI 10.1177/03635465980260021801
   Maffulli N, 1999, CLIN J SPORT MED, V9, P157, DOI 10.1097/00042752-199907000-00007
   Maffulli N, 1998, BRIT J SPORT MED, V32, P174, DOI 10.1136/bjsm.32.2.174
   Maffulli N, 2011, ARCH ORTHOP TRAUM SU, V131, P33, DOI 10.1007/s00402-010-1097-0
   Maffulli N, 2010, J BONE JOINT SURG AM, V92A, P2604, DOI 10.2106/JBJS.I.01744
   Maffulli N, 2010, CLIN ORTHOP RELAT R, V468, P1039, DOI 10.1007/s11999-009-0944-1
   Majewski M, 2000, ORTHOPADE, V29, P670
   Metz R, 2008, AM J SPORT MED, V36, P1688, DOI 10.1177/0363546508319312
   Moller M, 2001, J BONE JOINT SURG BR, V83B, P843
   Moller M, 2002, KNEE SURG SPORT TR A, V10, P49, DOI 10.1007/s001670100245
   Moller M, 2002, SCAND J MED SCI SPOR, V12, P9, DOI 10.1034/j.1600-0838.2002.120103.x
   Mortensen NHM, 1999, J BONE JOINT SURG AM, V81A, P983, DOI 10.2106/00004623-199907000-00011
   MORTENSEN NHM, 1992, ORTHOPEDICS, V15, P899
   Mosser P, 2015, J FOOT ANKLE SURG, V54, P2, DOI 10.1053/j.jfas.2014.09.040
   Muezzinoglu S, 2013, TECH FOOT ANKLE SURG, V12, P92, DOI 10.1097/BTF.0b013e318290ff37
   Munteanu SE, 2015, BRIT J SPORT MED, V49, P989, DOI 10.1136/bjsports-2014-093845
   Ng N, 2011, GLOBAL HEALTH ACTION, V4, DOI 10.3402/gha.v4i0.6347
   NILLIUS SA, 1976, ACTA ORTHOP SCAND, V47, P118, DOI 10.3109/17453677608998983
   Nilsson-Helander K, 2007, AM J SPORT MED, V35, P421, DOI 10.1177/0363546506294856
   Nilsson-Helander K, 2010, AM J SPORT MED, V38, P2186, DOI 10.1177/0363546510376052
   Nilsson-Helander K, 2009, KNEE SURG SPORT TR A, V17, P1234, DOI 10.1007/s00167-009-0727-y
   NISTOR L, 1976, ACTA ORTHOP SCAND, V47, P459, DOI 10.3109/17453677608988720
   NISTOR L, 1981, J BONE JOINT SURG AM, V63, P394, DOI 10.2106/00004623-198163030-00012
   Nyyssonen T, 2008, SCAND J SURG, V97, P272, DOI 10.1177/145749690809700312
   Oksanen MM, 2014, FOOT ANKLE SURG, V20, P253, DOI 10.1016/j.fas.2014.06.003
   Olsson N, 2014, AM J SPORT MED, V42, P1448, DOI 10.1177/0363546514527409
   Olsson N, 2013, AM J SPORT MED, V41, P2867, DOI 10.1177/0363546513503282
   Olsson N, 2011, KNEE SURG SPORT TR A, V19, P1385, DOI 10.1007/s00167-011-1511-3
   OZAKI J, 1989, CLIN ORTHOP RELAT R, P204
   Ozkaya U, 2009, INJURY, V40, P669, DOI 10.1016/j.injury.2008.10.033
   Pajala A, 2002, J BONE JOINT SURG AM, V84A, P2016, DOI 10.2106/00004623-200211000-00017
   Pajala A, 2009, J BONE JOINT SURG AM, V91A, P1092, DOI 10.2106/JBJS.G.01089
   Palacios-Cena D, 2012, BRIT J SPORT MED, V46, P1137, DOI 10.1136/bjsports-2012-091076
   Parekh Selene G, 2009, Foot Ankle Spec, V2, P283, DOI 10.1177/1938640009351138
   Patel A, 2012, CLIN ORTHOP RELAT R, V470, P270, DOI 10.1007/s11999-011-2166-6
   PERSSON A, 1979, INT ORTHOP, V3, P149
   Pintore E, 2001, J TRAUMA, V50, P71, DOI 10.1097/00005373-200101000-00013
   Porter MD, 2015, ANZ J SURG, V85, P373, DOI 10.1111/ans.12910
   Qureshi AA, 2011, J BONE JOINT SURG AM, V93A, P2265, DOI [10.2106/JBJSJ.01757, 10.2106/JBJS.J.01757]
   Raikin SM, 2013, FOOT ANKLE INT, V34, P475, DOI 10.1177/1071100713477621
   RALSTON EL, 1971, J TRAUM, V11, P15, DOI 10.1097/00005373-197101000-00001
   Ram R, 2013, CLIN INVEST MED, V36, pE197, DOI 10.25011/cim.v36i4.19953
   Rees JD, 2009, AM J SPORT MED, V37, P1855, DOI 10.1177/0363546508324283
   Richardson LC, 2003, FOOT ANKLE INT, V24, P439, DOI 10.1177/107110070302400512
   Rosso C, 2013, KNEE SURG SPORT TR A, V21, P1369, DOI 10.1007/s00167-013-2407-1
   RUBIN BD, 1980, J TRAUMA, V20, P248, DOI 10.1097/00005373-198003000-00011
   SALEH M, 1992, J BONE JOINT SURG BR, V74, P206, DOI 10.1302/0301-620X.74B2.1544953
   Sarman H, 2015, J FOOT ANKLE SURG, V54, P782, DOI 10.1053/j.jfas.2014.12.025
   Schepull T, 2013, AM J SPORT MED, V41, P2550, DOI 10.1177/0363546513501785
   Schepull T, 2011, AM J SPORT MED, V39, P38, DOI 10.1177/0363546510383515
   SEJBERG D, 1990, ACTA ORTHOP SCAND, V61, P549, DOI 10.3109/17453679008993580
   Silbernagel KG, 2010, KNEE SURG SPORT TR A, V18, P258, DOI 10.1007/s00167-009-0889-7
   Silbernagel KG, ORTHOP J SPORT MED
   Sinclair J, 2015, J ROY ARMY MED CORPS, V161, P140, DOI 10.1136/jramc-2014-000308
   Stamatakis E, 2008, BRIT J SPORT MED, V42, P901, DOI 10.1136/bjsm.2008.048082
   Stein BE, 2015, INT ORTHOP, V39, P901, DOI 10.1007/s00264-015-2725-7
   Steinbrueck K, 1997, ORTHOP T, V21, P1228
   Strauss EJ, 2007, INJURY, V38, P832, DOI 10.1016/j.injury.2006.06.005
   Suchak AA, 2005, FOOT ANKLE INT, V26, P932, DOI 10.1177/107110070502601106
   Tallon C, 2001, MED SCI SPORT EXER, V33, P1983, DOI 10.1097/00005768-200112000-00002
   Therbo M, 2003, SCAND J MED SCI SPOR, V13, P194, DOI 10.1034/j.1600-0838.2003.20205.x
   THERMANN H, 1995, UNFALLCHIRURG, V98, P21
   Twaddle BC, 2007, AM J SPORT MED, V35, P2033, DOI 10.1177/0363546507307503
   Van Tuyckom C, 2010, J SPORT SCI, V28, P1077, DOI 10.1080/02640414.2010.492229
   Vosseller JT, 2013, FOOT ANKLE INT, V34, P49, DOI 10.1177/1071100712460223
   Wang D, 2015, FOOT ANKLE SURG, V21, P250, DOI 10.1016/j.fas.2015.01.009
   Wapner K L, 1993, Foot Ankle, V14, P443
   Wearing SC, 2014, MED SCI SPORT EXER, V46, P1604, DOI 10.1249/MSS.0000000000000256
   Weber M, 2003, AM J SPORT MED, V31, P685, DOI 10.1177/03635465030310050901
   Weber O, 1999, TRAUMA BERUFSKRANKHE, V1, P258
   Wegrzyn J, 2010, INT ORTHOP, V34, P1187, DOI 10.1007/s00264-009-0859-1
   Wertz J, 2013, SPORTS HEALTH, V5, P407, DOI 10.1177/1941738112472165
   Willits K, 2010, J BONE JOINT SURG AM, V92A, P2767, DOI 10.2106/JBJS.I.01401
NR 169
TC 25
Z9 25
U1 0
U2 2
PU ELSEVIER SCI LTD
PI OXFORD
PA THE BOULEVARD, LANGFORD LANE, KIDLINGTON, OXFORD OX5 1GB, OXON, ENGLAND
SN 0020-1383
EI 1879-0267
J9 INJURY
JI Injury-Int. J. Care Inj.
PD JUL
PY 2017
VL 48
IS 7
BP 1701
EP 1709
DI 10.1016/j.injury.2017.04.007
PG 9
WC Critical Care Medicine; Emergency Medicine; Orthopedics; Surgery
WE Science Citation Index Expanded (SCI-EXPANDED)
SC General & Internal Medicine; Emergency Medicine; Orthopedics; Surgery
GA FB2TW
UT WOS:000405997100069
PM 28457569
DA 2022-07-22
ER

PT J
AU Akali, AU
   Niranjan, NS
AF Akali, A. U.
   Niranjan, N. S.
TI Management of bilateral Achilles tendon rupture associated with
   ciprofloxacin: A review and case presentation
SO JOURNAL OF PLASTIC RECONSTRUCTIVE AND AESTHETIC SURGERY
LA English
DT Article
DE fluoroquinolone; Achilles tendon rupture; Leeds-Keio ligament;
   perforator-based fasciocutaneous flap
ID TENDINITIS; NORFLOXACIN; DISORDERS
AB Fluoroquinolone antibiotics are increasingly being recognized as a cause of Achilles tendinitis and rupture. We report the case of a 62-year old man who developed bilateral Achilles tendon rupture six days following commencement of ciprofloxacin. Tendon exploration and repair was accomplished with the use of a prosthetic substitute (Leeds-Keio ligament) but heating was complicated by left wound breakdown that was successfully repaired with a perforator-based fasciocutaneous flap. A review of the current literature on fluoroquinolone associated achilles ruptures and the various methods of tendon and soft tissue management of the primary or complicated injuries are discussed. (C) 2007 British Association of Plastic, Reconstructive and Aesthetic Surgeons. Published by Elsevier Ltd. All rights reserved.
C1 [Akali, A. U.; Niranjan, N. S.] Broomfield Hosp, St Andrews Ctr Burns & Plast Surg, Dept Plast & Reconstruct Surg, Chelmsford, Essex, England.
RP Akali, AU (通讯作者)，26 Windsor Close, Normanton WF6 1TX, W Yorkshire, England.
EM auakali@dsl.pipex.com
CR [Anonymous], 2003, World Health Organ Tech Rep Ser, V921, P1
   BAILEY RR, 1983, NEW ZEAL MED J, V96, P590
   Berthe JV, 1998, PLAST RECONSTR SURG, V102, P1618, DOI 10.1097/00006534-199810000-00044
   Casparian JM, 2000, SOUTHERN MED J, V93, P488, DOI 10.1097/00007611-200005000-00008
   Chhajed PN, 2002, EUR RESPIR J, V19, P469, DOI 10.1183/09031936.02.00257202
   DELAGARZA EVA, 1997, ARCH MED RES, V28, P429
   Fama U, 2004, PLAST RECONSTR SURG, V114, P267, DOI 10.1097/01.PRS.0000127238.45551.12
   JANGOSE JT, 1996, NZ MED J, V109, P471
   LEE WT, 1992, AUST NZ J MED, V22, P500
   Maffulli N, 1999, J BONE JOINT SURG AM, V81A, P1019, DOI 10.2106/00004623-199907000-00017
   MARCACCI M, 1991, CLIN ORTHOP RELAT R, V267, P115
   Mathis AS, 2003, ANN PHARMACOTHER, V37, P1014, DOI 10.1345/aph.1C505
   McGarvey WC, 1996, FOOT ANKLE INT, V17, P496, DOI 10.1177/107110079601700811
   Melhus A, 2003, SCAND J INFECT DIS, V35, P768, DOI 10.1080/00365540310015863
   Movin T, 1997, FOOT ANKLE INT, V18, P297, DOI 10.1177/107110079701800510
   Poon CCH, 1997, MED J AUSTRALIA, V166, P665, DOI 10.5694/j.1326-5377.1997.tb123308.x
   RIBARD P, 1992, J RHEUMATOL, V19, P1479
   Royer RJ, 1996, THERAPIE, V51, P414
   ROYER RJ, 1994, THERAPIE, V49, P75
   Stanec S, 1999, PLAST RECONSTR SURG, V104, P1409, DOI 10.1097/00006534-199910000-00026
   van der Linden PD, 1999, BRIT J CLIN PHARMACO, V48, P433
   Van der Linden PD, 2001, ARTHRIT RHEUM-ARTHR, V45, P235, DOI 10.1002/1529-0131(200106)45:3<235::AID-ART254>3.0.CO;2-7
   Vanek D, 2003, J AM PODIAT MED ASSN, V93, P333, DOI 10.7547/87507315-93-4-333
   West MB, 1998, NEW ZEAL MED J, V111, P18
   Williams RJ, 2000, AM J SPORT MED, V28, P364, DOI 10.1177/03635465000280031401
   Wilton LV, 1996, BRIT J CLIN PHARMACO, V41, P277, DOI 10.1046/j.1365-2125.1996.03013.x
   2004, BRIT NATL FORMULARY, V48, P300
NR 27
TC 23
Z9 24
U1 0
U2 2
PU ELSEVIER SCI LTD
PI OXFORD
PA THE BOULEVARD, LANGFORD LANE, KIDLINGTON, OXFORD OX5 1GB, OXON, ENGLAND
SN 1748-6815
EI 1878-0539
J9 J PLAST RECONSTR AES
JI J. Plast. Reconstr. Aesthet. Surg.
PY 2008
VL 61
IS 7
BP 830
EP 834
DI 10.1016/j.bjps.2006.08.005
PG 5
WC Surgery
WE Science Citation Index Expanded (SCI-EXPANDED)
SC Surgery
GA 330BJ
UT WOS:000257914000027
PM 17409040
DA 2022-07-22
ER

PT J
AU Carmont, MR
   Zellers, JA
   Brorsson, A
   Nilsson-Helander, K
   Karlsson, J
   Silbernagel, KG
AF Carmont, Michael R.
   Zellers, Jennifer A.
   Brorsson, Annelie
   Nilsson-Helander, Katarina
   Karlsson, Jon
   Silbernagel, Karin Gravare
TI Age and Tightness of Repair Are Predictors of Heel-Rise Height After
   Achilles Tendon Rupture
SO ORTHOPAEDIC JOURNAL OF SPORTS MEDICINE
LA English
DT Article
DE Achilles tendon rupture; operative repair; outcome; heel-rise
ID TERM-FOLLOW-UP; RESTING ANGLE; NONSURGICAL TREATMENT; SURGICAL REPAIR;
   OPERATIVE TREATMENT; LENGTH; RELIABILITY; COMPLICATIONS; ENDURANCE;
   DEFICITS
AB Background:
   Achilles tendon rupture leads to weakness of ankle plantarflexion. Treatment of Achilles tendon rupture should aim to restore function while minimizing weakness and complications of management.
   Purpose:
   To determine the influence of factors (age, sex, body mass index [BMI], weight, time from injury to operative repair, and tightness of repair) in the initial surgical management of patients after an acute Achilles tendon rupture on 12-month functional outcome assessment after percutaneous and minimally invasive repair.
   Study Design:
   Cohort study; Level of evidence, 3.
   Methods:
   From May 2012 to January 2018, patients sustaining an Achilles tendon rupture receiving operative repair were prospectively evaluated. Tightness of repair was quantified using the intraoperative Achilles tendon resting angle (ATRA). Heel-rise height index (HRHI) was used as the primary 12-month outcome variable. Secondary outcome measures included Achilles tendon total rupture score (ATRS) and Tegner score. Stepwise multiple regression was used to create a model to predict 12-month HRHI.
   Results:
   A total of 122 patients met the inclusion criteria for data analysis (mean +/- SD age, 44.1 +/- 10.8 years; 78% male; mean +/- SD BMI, 28.1 +/- 4.3 kg/m(2)). The elapsed time to surgery was 6.5 +/- 4.0 days. At 12-month follow-up, patients had an HRHI of 82% +/- 16% and performed 82% +/- 17% of repetitions compared with the noninjured side. Participants had a mean ATRS of 87 +/- 15 and a median Tegner score of 5 (range, 1-9), with a reduction in Tegner score of 2 from preinjury levels. The relative ATRA at 12 months was -4.8 degrees +/- 3.9 degrees. Multiple regression identified younger age (B = +/- 0.006; P < .001) and greater intraoperative ATRA (B = 0.005; P = .053) as predictors of more symmetrical 12-month HRHI (R (2) = 0.19; P < .001; n = 120).
   Conclusion:
   Age was found to be the strongest predictor of outcome after Achilles tendon rupture. The most important modifiable risk factor was the tightness of repair. It is recommended that repair be performed as tight as possible to optimize heel-rise height 1 year after Achilles tendon rupture and possibly to reduce tendon elongation.
C1 [Carmont, Michael R.; Zellers, Jennifer A.; Brorsson, Annelie; Nilsson-Helander, Katarina; Karlsson, Jon; Silbernagel, Karin Gravare] Shrewsbury & Telford Hosp NHS Trust, Princess Royal Hosp, Telford, Shrops, England.
   [Carmont, Michael R.] Shrewsbury & Telford Hosp NHS Trust, Princess Royal Hosp, Dept Trauma & Orthopaed Surg, Telford, Shrops, England.
   [Zellers, Jennifer A.] Washington Univ, Sch Med St Louis, Program Phys Therapy, St Louis, MO USA.
   [Brorsson, Annelie; Nilsson-Helander, Katarina; Karlsson, Jon] Univ Gothenburg, Sahlgrenska Acad, Inst Clin Sci, Dept Orthopaed, Gothenburg, Sweden.
   [Silbernagel, Karin Gravare] Univ Delaware, Dept Phys Therapy, Newark, DE USA.
RP Carmont, MR (通讯作者)，Shrewsbury & Telford Hosp NHS Trust, Princess Royal Hosp, Telford, Shrops, England.
EM mcarmont@hotmail.com
OI Zellers, Jennifer/0000-0002-0601-1355
CR Aujla R, 2018, FOOT ANKLE SURG, V24, P336, DOI 10.1016/j.fas.2017.03.007
   Bostick GP, 2010, J ORTHOP SPORT PHYS, V40, P345, DOI 10.2519/jospt.2010.3204
   Brorsson A, 2017, AM J SPORT MED, V45, P3060, DOI 10.1177/0363546517717698
   Carmont MR, 2013, FOOT ANKLE SURG, V19, P245, DOI 10.1016/j.fas.2013.06.007
   Carmont MR, 2017, ORTHOP J SPORTS MED, V5, DOI 10.1177/2325967117723347
   Carmont MR, 2013, ORTHOP J SPORTS MED, V1, DOI 10.1177/2325967113494584
   Carmont MR, 2015, ASIA-PAC J SPORT MED, V2, P49, DOI 10.1016/j.asmart.2014.12.002
   Carmont MR, 2013, KNEE SURG SPORT TR A, V21, P1356, DOI 10.1007/s00167-012-2146-8
   Chandrakant V, 2012, FOOT ANKLE INT, V33, P1093, DOI 10.3113/FAI.2012.1093
   Domeij-Arverud E, 2016, BONE JOINT J, V98B, P1635, DOI 10.1302/0301-620X.98B12.BJJ-2016-0008.R1
   Ecker TM, 2016, AM J SPORT MED, V44, P1004, DOI 10.1177/0363546515623501
   Groetelaers RPTGC, 2014, FOOT ANKLE INT, V35, P771, DOI 10.1177/1071100714536167
   Hansen MS, 2017, FOOT ANKLE SURG, V23, P275, DOI 10.1016/j.fas.2016.08.002
   Heikkinen J, 2017, J BONE JOINT SURG AM, V99, P1509, DOI 10.2106/JBJS.16.01491
   Heikkinen J, 2017, AM J SPORT MED, V45, P1395, DOI 10.1177/0363546517694610
   Hsu AR, 2015, FOOT ANKLE INT, V36, P1279, DOI 10.1177/1071100715589632
   Hullfish TJ, 2019, PEERJ, V7, DOI 10.7717/peerj.6788
   KAKIUCHI M, 1995, J BONE JOINT SURG BR, V77B, P60, DOI 10.1302/0301-620X.77B1.7822398
   Kangas J, 2003, J TRAUMA, V54, P1171, DOI 10.1097/01.TA.0000047945.20863.A2
   Keller A, 2014, AM J SPORT MED, V42, P731, DOI 10.1177/0363546513511418
   Lantto I, 2016, AM J SPORT MED, V44, P2406, DOI 10.1177/0363546516651060
   Lawrence JE, 2017, BONE JOINT J, V99B, P87, DOI 10.1302/0301-620X.99B1.BJJ-2016-0452.R1
   Liechti DJ, 2018, ARTHROSC TEC, V7, pE171, DOI 10.1016/j.eats.2017.08.065
   Lim CS, 2017, FOOT ANKLE INT, V38, P1331, DOI 10.1177/1071100717728687
   Maffulli N, 1998, AM J SPORT MED, V26, P266, DOI 10.1177/03635465980260021801
   Manegold S, 2019, FOOT ANKLE SURG, V25, P529, DOI 10.1016/j.fas.2018.04.004
   Maquirriain Javier, 2011, Yale Journal of Biology and Medicine, V84, P289
   Matles A L, 1975, Bull Hosp Joint Dis, V36, P48
   Misir A, 2020, KNEE SURG SPORT TR A, V28, P2788, DOI 10.1007/s00167-019-05536-w
   Molloy A, 2009, FOOT ANKLE CLIN, V14, P745, DOI 10.1016/j.fcl.2009.07.004
   Mortensen NHM, 1999, J BONE JOINT SURG AM, V81A, P983, DOI 10.2106/00004623-199907000-00011
   Nilsson-Helander K, 2007, AM J SPORT MED, V35, P421, DOI 10.1177/0363546506294856
   Nilsson-Helander K, 2010, AM J SPORT MED, V38, P2186, DOI 10.1177/0363546510376052
   NYSTROM B, 1983, ACTA ORTHOP SCAND, V54, P620, DOI 10.3109/17453678308992899
   Ochen Y, 2019, BMJ-BRIT MED J, V364, DOI 10.1136/bmj.k5120
   Olsson N, 2014, AM J SPORT MED, V42, P1448, DOI 10.1177/0363546514527409
   Olsson N, 2013, AM J SPORT MED, V41, P2867, DOI 10.1177/0363546513503282
   Pajala A, 2009, J BONE JOINT SURG AM, V91A, P1092, DOI 10.2106/JBJS.G.01089
   Park YH, 2017, INJURY, V48, P776, DOI 10.1016/j.injury.2017.01.020
   Reda Y, 2020, FOOT ANKLE SURG, V26, P280, DOI 10.1016/j.fas.2019.03.010
   Rippstein Pascal F, 2002, Foot Ankle Clin, V7, P611, DOI 10.1016/S1083-7515(02)00040-2
   Rosso C, 2015, KNEE SURG SPORT TR A, V23, P890, DOI 10.1007/s00167-013-2726-2
   Schepull T, 2012, SCAND J MED SCI SPOR, V22, P18, DOI 10.1111/j.1600-0838.2010.01154.x
   Schepull T, 2013, AM J SPORT MED, V41, P2550, DOI 10.1177/0363546513501785
   Silbernagel KG, 2012, AM J SPORT MED, V40, P1564, DOI 10.1177/0363546512447926
   Silbernagel KG, 2015, ORTHOP J SPORTS MED, V3, DOI 10.1177/2325967115586768
   Silbernagel KG, 2010, KNEE SURG SPORT TR A, V18, P258, DOI 10.1007/s00167-009-0889-7
   Soroceanu A, 2012, J BONE JOINT SURG AM, V94A, P2136, DOI 10.2106/JBJS.K.00917
   Svedman S, 2018, AM J SPORT MED, V46, P2929, DOI 10.1177/0363546518793655
   TEGNER Y, 1985, CLIN ORTHOP RELAT R, P43
   van der Werken C., 2000, ORTHOP TRAUMATOL, V8, P285
   Westin O, 2018, ORTHOP J SPORTS MED, V6, DOI 10.1177/2325967118813904
   Westin O, 2016, ORTHOP J SPORTS MED, V4, DOI 10.1177/2325967116667920
   Willits K, 2010, J BONE JOINT SURG AM, V92A, P2767, DOI 10.2106/JBJS.I.01401
   Wu YH, 2019, AM J SPORT MED, V47, P2251, DOI 10.1177/0363546518824601
   Yang X, 2018, BONE JOINT RES, V7, P561, DOI 10.1302/2046-3758.710.BJR-2018-0004.R2
   Zellers JA, 2018, FOOT ANKLE INT, V39, P343, DOI 10.1177/1071100717742372
NR 57
TC 14
Z9 14
U1 0
U2 6
PU SAGE PUBLICATIONS INC
PI THOUSAND OAKS
PA 2455 TELLER RD, THOUSAND OAKS, CA 91320 USA
EI 2325-9671
J9 ORTHOP J SPORTS MED
JI Orthop. J. Sports Med.
PD MAR
PY 2020
VL 8
IS 3
AR 2325967120909556
DI 10.1177/2325967120909556
PG 8
WC Orthopedics; Sport Sciences
WE Science Citation Index Expanded (SCI-EXPANDED)
SC Orthopedics; Sport Sciences
GA LB1BQ
UT WOS:000524371600001
PM 32232072
OA Green Published, gold
DA 2022-07-22
ER

PT J
AU Zhao, H
   Guan, HG
   Gu, J
   Luo, ZP
   Zhang, W
   Chen, B
   Gu, QL
   Yang, HL
   Shi, Q
AF Zhao Huan
   Guan Hong-geng
   Gu Jun
   Luo Zong-ping
   Zhang Wen
   Chen Bing
   Gu Qiao-li
   Yang Hui-lin
   Shi Qin
TI Collagen membrane alleviates peritendinous adhesion in the rat Achilles
   tendon injury model
SO CHINESE MEDICAL JOURNAL
LA English
DT Article
DE collagen membrane; Achilles tendon rupture; peritendinous adhesion;
   tendon repair
ID REPAIR; REGENERATION; RUPTURE; REHABILITATION; TENDINOPATHY; SCAFFOLDS;
   BARRIER
AB Background Tendon adhesion is one of the most common causes of disability following tendon surgery. Therefore, prevention of peritendinous adhesion after surgical repair of tendon is a major challenge. The aim of this study was to explore the possible application of a collagen membrane for the prevention or attenuation of peritendinous adhesions.
   Methods Sprague-Dawley (SD) rat Achilles tendon was cut and sutured by a modified Kessler's technique with or without the collagen membrane wrapped. Macroscopic, morphological and biomechanical evaluations were applied to examine the recovery of the injured tendon at 4 and 8 weeks after surgery.
   Results The surgery group wrapped by collagen membranes had a better outcome than the group with surgery repair only. In the collagen membrane-treated group, less adhesion appeared, stronger tensile strength was detected, and more tendon fibers and collagen I expression were observed morphologically.
   Conclusion Wrapping the tendon with a collagen membrane may be an efficient approach for tendon repair and preventing tendon adhesion after its ruptures.
C1 [Zhao Huan; Luo Zong-ping; Zhang Wen; Gu Qiao-li; Yang Hui-lin; Shi Qin] Soochow Univ, Affiliated Hosp 1, Dept Orthoped Surg, Suzhou 215006, Jiangsu, Peoples R China.
   [Guan Hong-geng] Soochow Univ, Affiliated Hosp 1, Dept Gen Surg, Suzhou 215006, Jiangsu, Peoples R China.
RP Shi, Q (通讯作者)，Soochow Univ, Affiliated Hosp 1, Dept Orthoped Surg, Suzhou 215006, Jiangsu, Peoples R China.
EM shiqin@suda.edu.cn
FU Jiangsu Orthopedic Medical Center; Priority Academic Program Development
   of Jiangsu Higher Education Institutions; National High Technology
   Research and Development Program ("863" Program) of China [2012AA020501]
FX This work was supported by grants from the Jiangsu Orthopedic Medical
   Center, the Priority Academic Program Development of Jiangsu Higher
   Education Institutions and the National High Technology Research and
   Development Program ("863" Program) of China (No. 2012AA020501).
CR Bava ED, 2011, PHYSICIAN SPORTSMED, V39, P94, DOI 10.3810/psm.2011.09.1925
   Caliari SR, 2011, BIOMATERIALS, V32, P8990, DOI 10.1016/j.biomaterials.2011.08.035
   Chong AKS, 2007, J BONE JOINT SURG AM, V89A, P74, DOI 10.2106/JBJS.E.01396
   Gott M, 2011, DISCOV MED, V11, P75
   Han QQ, 2010, BIOMATERIALS, V31, P9212, DOI 10.1016/j.biomaterials.2010.08.040
   Helary C, 2012, J TISSUE ENG REGEN M, V6, P225, DOI 10.1002/term.420
   Ingraham JM, 2011, J HAND SURG-EUR VOL, V36E, P154, DOI 10.1177/1753193410382959
   Ishiyama N, 2011, J BONE JOINT SURG AM, V93A, P142, DOI 10.2106/JBJS.I.01634
   Li XA, 2011, BIOMATERIALS, V32, P8172, DOI 10.1016/j.biomaterials.2011.07.050
   Maquirriain Javier, 2011, Yale Journal of Biology and Medicine, V84, P289
   Mentzel M, 2000, J HAND SURG-BRIT EUR, V25B, P590, DOI 10.1054/jhsb.2000.0385
   Ozgenel GY, 2004, J BONE JOINT SURG BR, V86B, P301, DOI 10.1302/0301-620X.86B2.14435
   Pek YS, 2008, BIOMATERIALS, V29, P4300, DOI 10.1016/j.biomaterials.2008.07.030
   Rasmussen S, 2008, ACTA ORTHOP, V79, P249, DOI 10.1080/17453670710015058
   Schlee M, 2012, HEAD FACE MED, V8, DOI 10.1186/1746-160X-8-6
   Sharma P, 2005, J BONE JOINT SURG AM, V87A, P187, DOI 10.2106/JBJS.D.01850
   Sun WJ, 2009, BIOMATERIALS, V30, P4649, DOI 10.1016/j.biomaterials.2009.05.037
   Talbot JC, 2012, J FOOT ANKLE SURG, V51, P147, DOI 10.1053/j.jfas.2011.10.045
   Tan V, 2010, J HAND SURG-AM, V35A, P941, DOI 10.1016/j.jhsa.2010.02.033
   Tang JB, 1996, J HAND SURG-AM, V21A, P900, DOI 10.1016/S0363-5023(96)80212-7
   Temiz A, 2008, INT ORTHOP, V32, P389, DOI 10.1007/s00264-007-0335-8
   Zhao HM, 2011, CHINESE MED J-PEKING, V124, P4050, DOI 10.3760/cma.j.issn.0366-6999.2011.23.038
   Zobitz ME, 2000, J BIOMECH ENG-T ASME, V122, P604, DOI 10.1115/1.1324668
NR 23
TC 21
Z9 22
U1 1
U2 15
PU MEDKNOW PUBLICATIONS & MEDIA PVT LTD
PI MUMBAI
PA B-9, KANARA BUSINESS CENTRE, OFF LINK RD, GHAKTOPAR-E, MUMBAI, 400075,
   INDIA
SN 0366-6999
J9 CHINESE MED J-PEKING
JI Chin. Med. J.
PD FEB 20
PY 2013
VL 126
IS 4
BP 729
EP 733
DI 10.3760/cma.j.issn.0366-6999.20122566
PG 5
WC Medicine, General & Internal
WE Science Citation Index Expanded (SCI-EXPANDED)
SC General & Internal Medicine
GA 156BA
UT WOS:000319793800023
PM 23422197
DA 2022-07-22
ER

PT J
AU Gajhede-Knudsen, M
   Ekstrand, J
   Magnusson, H
   Maffulli, N
AF Gajhede-Knudsen, Mariann
   Ekstrand, Jan
   Magnusson, Henrik
   Maffulli, Nicola
TI Recurrence of Achilles tendon injuries in elite male football players is
   more common after early return to play: an 11-year follow-up of the UEFA
   Champions League injury study
SO BRITISH JOURNAL OF SPORTS MEDICINE
LA English
DT Article
DE Achilles tendon; Elite performance; Epidemiology; Lower extremity
   injuries; Soccer
ID MEDICAL-RESEARCH PROGRAM; PROFESSIONAL FOOTBALL; BADMINTON PLAYERS;
   SOCCER PLAYERS; TENDINOPATHY; EPIDEMIOLOGY; RUPTURE; TENDINOSIS; AUDIT;
   PAIN
AB Background There is limited information about Achilles tendon disorders in professional football.
   Aims To investigate the incidence, injury circumstances, lay-off times and reinjury rates of Achilles tendon disorders in male professional football.
   Methods A total of 27 clubs from 10 countries and 1743 players have been followed prospectively during 11 seasons between 2001 and 2012. The team medical staff recorded individual player exposure and time-loss injuries.
   Results A total of 203 (2.5% of all injuries) Achilles tendon disorders were registered. A majority (96%) of the disorders were tendinopathies, and nine were partial or total ruptures. A higher injury rate was found during the preseason compared with the competitive season, 0.25 vs 0.18/1000 h (rate ratio (RR) 1.4, 95% CI 1.1 to 2.0; p=0.027). The mean lay-off time for Achilles tendinopathies was 23 +/- 37 (median=10, Q(1)=4 and Q(3)=24) days, while a rupture of the Achilles tendon, on average, caused 161 +/- 65 (median=169, Q(1)=110 and Q(3)=189) days of absence. Players with Achilles tendon disorders were significantly older than the rest of the cohort, with a mean age of 27.2 +/- 4 years vs 25.6 +/- 4.6 years (p<0.001). 27% of all Achilles tendinopathies were reinjuries. A higher reinjury risk was found after short recovery periods (31%) compared with longer recovery periods (13%) (RR 2.4, 95% CI 2.1 to 2.8; p<0.001).
   Conclusions Achilles tendon disorders account for 3.8% of the total lay-off time and are more common in older players. Recurrence is common after Achilles tendinopathies and the reinjury risk is higher after short recovery periods.
C1 [Gajhede-Knudsen, Mariann; Ekstrand, Jan] Linkoping Univ, Div Community Med, Dept Med & Hlth Sci, Linkoping, Sweden.
   [Gajhede-Knudsen, Mariann; Ekstrand, Jan; Magnusson, Henrik] Linkoping Univ, Football Res Grp, Linkoping, Sweden.
   [Ekstrand, Jan] UEFA Med Comm, Nyon, Switzerland.
   [Magnusson, Henrik] Linkoping Univ, Div Physiotherapy, Dept Med & Hlth Sci, Linkoping, Sweden.
   [Maffulli, Nicola] Univ Salerno, Sch Med & Surg, Dept Musculoskeletal Disorders, I-84100 Salerno, Italy.
   [Maffulli, Nicola] Queen Mary Univ London, Ctr Sports & Exercise Med, London, England.
RP Ekstrand, J (通讯作者)，Solstigen 3, S-58943 Linkoping, Sweden.
EM jan.ekstrand@telia.com
RI Maffulli, Nicola/A-1839-2014
OI Maffulli, Nicola/0000-0002-5327-3702
FU Union of European Football Associations (UEFA); Swedish National Centre
   for Research in Sports; Praktikertjanst AB
FX This study was supported by grants from the Union of European Football
   Associations (UEFA), the Swedish National Centre for Research in Sports
   and Praktikertjanst AB.
CR Alfredson H, 2000, SPORTS MED, V29, P135, DOI 10.2165/00007256-200029020-00005
   Alfredson H, 2003, KNEE SURG SPORT TR A, V11, P334, DOI 10.1007/s00167-003-0391-6
   Bahr R, 2009, BRIT J SPORT MED, V43, P966, DOI 10.1136/bjsm.2009.066936
   Chomiak J, 2000, AM J SPORT MED, V28, pS58
   Collins M, 2011, EXERC SPORT SCI REV, V39, P191, DOI 10.1097/JES.0b013e318224e853
   Cook JL, 2002, MANUAL THER, V7, P121, DOI 10.1054/math.2002.0458
   Ekstrand J, 2012, SCAND J MED SCI SPOR, V22, P341, DOI 10.1111/j.1600-0838.2010.01171.x
   Ekstrand J, 2011, BRIT J SPORT MED, V45, P553, DOI 10.1136/bjsm.2009.060582
   Ekstrand J, 2011, AM J SPORT MED, V39, P1226, DOI 10.1177/0363546510395879
   Fahlstrom M, 2002, AM J SPORT MED, V30, P51, DOI 10.1177/03635465020300012201
   Fahlstrom M, 1998, AM J SPORT MED, V26, P467, DOI 10.1177/03635465980260032201
   Fredberg U, 2008, SCAND J MED SCI SPOR, V18, P3, DOI 10.1111/j.1600-0838.2007.00746.x
   Fredberg U, 2008, AM J SPORT MED, V36, P451, DOI 10.1177/0363546507310073
   Fuller CW, 2006, BRIT J SPORT MED, V40, P193, DOI 10.1136/bjsm.2005.025270
   Fuller CW, 2006, BRIT J SPORT MED, V40, P151, DOI 10.1136/bjsm.2005.021048
   Grobler LA, 2004, BRIT J SPORT MED, V38, P697, DOI 10.1136/bjsm.2003.006502
   Hagglund M, 2005, BRIT J SPORT MED, V39, P340, DOI 10.1136/bjsm.2005.018267
   Hagglund M, 2005, SCAND J MED SCI SPOR, V15, P21, DOI 10.1111/j.1600-0838.2004.00395.x
   Hagglund M, 2011, AM J SPORT MED, V39, P1906, DOI 10.1177/0363546511408877
   Hawkins RD, 1999, BRIT J SPORT MED, V33, P196, DOI 10.1136/bjsm.33.3.196
   Hawkins RD, 2001, BRIT J SPORT MED, V35, P43, DOI 10.1136/bjsm.35.1.43
   Jarvinen M, 1997, SCAND J MED SCI SPOR, V7, P86
   JOZSA L, 1989, AM J SPORT MED, V17, P338, DOI 10.1177/036354658901700305
   KANNUS P, 1991, J BONE JOINT SURG AM, V73A, P1507, DOI 10.2106/00004623-199173100-00009
   KOMI PV, 1992, CLIN SPORT MED, V11, P521
   Kujala UM, 2005, CLIN J SPORT MED, V15, P133, DOI 10.1097/01.jsm.0000165347.55638.23
   KVIST M, 1991, ANN CHIR GYNAECOL FE, V80, P188
   KVIST M, 1994, SPORTS MED, V18, P173, DOI 10.2165/00007256-199418030-00004
   Langberg H, 2007, SCAND J MED SCI SPOR, V17, P61, DOI 10.1111/j.1600-0838.2006.00522.x
   Maffulli N, 2003, CLIN SPORT MED, V22, P675, DOI 10.1016/S0278-5919(03)00004-8
   Maffulli N, 2002, J BONE JOINT SURG BR, V84B, P1, DOI 10.1302/0301-620X.84B1.12792
   Maffulli N, 2004, J ROY SOC MED, V97, P472, DOI 10.1258/jrsm.97.10.472
   Maffulli N, 2008, DISABIL REHABIL, V30, P1677, DOI 10.1080/09638280701786427
   Maganaris CN, 2004, SPORTS MED, V34, P1005, DOI 10.2165/00007256-200434140-00005
   Muramatsu T, 2001, J APPL PHYSIOL, V90, P1671, DOI 10.1152/jappl.2001.90.5.1671
   Muraoka T, 2005, J APPL PHYSIOL, V99, P665, DOI 10.1152/japplphysiol.00624.2004
   Nichols A W, 1989, J Am Board Fam Pract, V2, P196
   Oztekin Haluk H, 2009, Foot (Edinb), V19, P22, DOI 10.1016/j.foot.2008.07.003
   Parekh Selene G, 2009, Foot Ankle Spec, V2, P283, DOI 10.1177/1938640009351138
   Rees JD, 2009, AM J SPORT MED, V37, P1855, DOI 10.1177/0363546508324283
   rOen A, 2004, SCAND J MED SCI SPOR, V14, P30
   Sankey RA, 2008, AM J SPORT MED, V36, P2415, DOI 10.1177/0363546508322889
   Walden M, 2005, SCAND J MED SCI SPOR, V15, P118, DOI 10.1111/j.1600-0838.2004.00393.x
   Woods C, 2003, BRIT J SPORT MED, V37, P233, DOI 10.1136/bjsm.37.3.233
   Woods C, 2002, BRIT J SPORT MED, V36, P436, DOI 10.1136/bjsm.36.6.436
NR 45
TC 70
Z9 71
U1 1
U2 28
PU BMJ PUBLISHING GROUP
PI LONDON
PA BRITISH MED ASSOC HOUSE, TAVISTOCK SQUARE, LONDON WC1H 9JR, ENGLAND
SN 0306-3674
J9 BRIT J SPORT MED
JI Br. J. Sports Med.
PD AUG
PY 2013
VL 47
IS 12
BP 763
EP 768
DI 10.1136/bjsports-2013-092271
PG 6
WC Sport Sciences
WE Science Citation Index Expanded (SCI-EXPANDED)
SC Sport Sciences
GA 197RR
UT WOS:000322868800012
PM 23770660
DA 2022-07-22
ER

PT J
AU Ofili, KP
   Pollard, JD
   Schuberth, JM
AF Ofili, Kene P.
   Pollard, Jason D.
   Schuberth, John M.
TI The Neglected Achilles Tendon Rupture Repaired With Allograft: A Review
   of 14 Cases
SO JOURNAL OF FOOT & ANKLE SURGERY
LA English
DT Article
DE allograft; Achilles tendon; neglected rupture
ID RECONSTRUCTION
AB Various surgical techniques have been reported for the repair of neglected Achilles tendon ruptures, including V-Y advancements, synthetic augmentations, and collagen implants. The use of an Achilles tendon allograft allows bridging of large defects without donor site morbidity, with a relative ease of technique and adequate graft availability. The present retrospective report focused on the outcomes of a series of 14 patients with neglected ruptures treated with an Achilles tendon allograft. Patients were included in the present series if they had >= 12 months of postoperative follow-up data available and the allograft had been used without any adjunctive procedures. Of the 14 patients, 6 were female (43%) and 8 were male (57%), with a mean follow-up period of 16.1 +/- 3 (range 12 to 27) months. The mean interval from the initial injury to surgery was 6.9 +/- 5 (range 1 to 28) months. The mean intraoperative defect size was 7.0 +/- 3 (range 4 to 15) cm. A calcaneal block was used in 2 patients (14%). All patients were able to perform a single heel rise at a mean of 27 11 (range 12 to 37) weeks postoperatively. Weightbearing in normal shoe gear was achieved at a mean of 13.5 +/- 3 (range 12 to 17) weeks. Complications included 1 delayed union (7%) of the calcaneal bone block. Repair of the neglected Achilles tendon rupture with an allograft appears to be an acceptable approach, with good overall outcomes and low risk. These results suggest that this method of repair compares favorably with established alternatives. (C) 2016 by the American College of Foot and Ankle Surgeons. All rights reserved.
C1 [Ofili, Kene P.] Woodland Clin Med Grp, Dept Orthoped & Podiatry, Woodland, CA USA.
   [Pollard, Jason D.] Kaiser Oakland Med Ctr, Dept Podiatr Surg, Oakland, CA USA.
   [Schuberth, John M.] Kaiser San Francisco Med Ctr, Foot & Ankle Surg, San Francisco, CA USA.
RP Schuberth, JM (通讯作者)，Kaiser Fdn Hosp, Dept Orthoped Surg, French Campus,450 6th Ave, San Francisco, CA 94118 USA.
EM Jmfoot@aol.com
CR Badalihan A, 2015, J FOOT ANKLE SURG, V54, P153, DOI 10.1053/j.jfas.2014.12.002
   Ballas MT, 1998, AM FAM PHYSICIAN, V57, P267
   Cienfuegos A, 2013, J FOOT ANKLE SURG, V52, P95, DOI 10.1053/j.jfas.2012.06.006
   Cvetanovich GL, 2014, ARTHROSCOPY, V30, P1616, DOI 10.1016/j.arthro.2014.05.040
   Deese J Melvin, 2015, J Surg Orthop Adv, V24, P75
   Edgar CM, 2008, CLIN ORTHOP RELAT R, V466, P2238, DOI 10.1007/s11999-008-0305-5
   GABEL S, 1994, FOOT ANKLE INT, V15, P512, DOI 10.1177/107110079401500912
   Hanna T, 2014, FOOT ANKLE INT, V35, P168, DOI 10.1177/1071100713514037
   Haraguchi N, 2005, TECH FOOT ANKLE SURG, V4, P154, DOI 10.1097/01.btf.0000177228.45213.5d
   HOWARD CB, 1984, J BONE JOINT SURG BR, V66, P206, DOI 10.1302/0301-620X.66B2.6707056
   Lepow Gary M, 2006, J Foot Ankle Surg, V45, P351, DOI 10.1053/j.jfas.2006.06.004
   LINN RM, 1993, AM J SPORT MED, V21, P825, DOI 10.1177/036354659302100611
   Maffulli N, 1999, J BONE JOINT SURG AM, V81A, P1019, DOI 10.2106/00004623-199907000-00017
   Maffulli N, 1998, AM J SPORT MED, V26, P266, DOI 10.1177/03635465980260021801
   Maffulli N, 2011, KNEE SURG SPORT TR A, V19, P680, DOI 10.1007/s00167-010-1193-2
   McNally PD, 1998, ARTHROSCOPY, V14, P340, DOI 10.1016/S0749-8063(98)70154-9
   Nellas Z J, 1996, J Foot Ankle Surg, V35, P144
   Nestorson J, 2000, ACTA ORTHOP SCAND, V71, P64, DOI 10.1080/00016470052943928
   PARSONS JR, 1989, FOOT ANKLE, V9, P179, DOI 10.1177/107110078900900406
   Patterson RW, 2009, J HAND SURG-AM, V34A, P545, DOI 10.1016/j.jhsa.2008.12.019
   Scheffler SU, 2008, ARTHROSCOPY, V24, P448, DOI 10.1016/j.arthro.2007.10.011
   SCHUBERTH JM, ACHILLES TENDON
   WAPNER KL, 1995, ORTHOP CLIN N AM, V26, P249
   Yuen JC, 2001, PLAST RECONSTR SURG, V107, P1807, DOI 10.1097/00006534-200106000-00028
NR 24
TC 11
Z9 12
U1 0
U2 3
PU ELSEVIER SCIENCE INC
PI NEW YORK
PA STE 800, 230 PARK AVE, NEW YORK, NY 10169 USA
SN 1067-2516
EI 1542-2224
J9 J FOOT ANKLE SURG
JI J. Foot Ankle Surg.
PD NOV-DEC
PY 2016
VL 55
IS 6
BP 1245
EP 1248
DI 10.1053/j.jfas.2016.01.001
PG 4
WC Orthopedics; Surgery
WE Science Citation Index Expanded (SCI-EXPANDED)
SC Orthopedics; Surgery
GA EB1FJ
UT WOS:000387094700022
PM 26872523
DA 2022-07-22
ER

PT J
AU Netto, CD
   Bernasconi, A
   Roberts, L
   Saito, GH
   Hamilton, WG
   O'Malley, MJ
AF Netto, Cesar de Cesar
   Bernasconi, Alessio
   Roberts, Lauren
   Saito, Guilherme Honda
   Hamilton, William G.
   O'Malley, Martin J.
TI Open Re-rupture of the Achilles Tendon Following Minimally Invasive
   Repair: A Case Report
SO JOURNAL OF FOOT & ANKLE SURGERY
LA English
DT Article
DE Achilles tendon; ankle; athlete; foreign body reaction; injury;
   paratenon
ID RUPTURES; METAANALYSIS; REHABILITATION; TRIALS
AB In this report, we describe a rare complication of an open re-rupture of the Achilles tendon following a minimally invasive Achilles tendon repair on a healthy 29-year-old active male. The reinjury happened 19 weeks following the primary surgical repair of a spontaneous rupture, performed by minimally invasive technique with the help of a jig using partially absorbable sutures and four locking stitches. The wound of the open re-rupture was transverse, in a perpendicular orientation relative to the longitudinal approach used in the index procedure. Increased scar tissue formation, the absence of an adequate layer of paratenon overlying the primary tendon repair, and foreign-body reaction to the suture may have been involved in the occurrence of this unusual complication in the surgical treatment of Achilles tendon rupture. (C) 2018 by the American College of Foot and Ankle Surgeons. All rights reserved.
C1 [Netto, Cesar de Cesar; Bernasconi, Alessio; Roberts, Lauren; Saito, Guilherme Honda; Hamilton, William G.; O'Malley, Martin J.] Hosp Special Surg, 535 East 70th St, New York, NY 10021 USA.
RP Netto, CD (通讯作者)，Hosp Special Surg, 535 East 70th St, New York, NY 10021 USA.
EM cesardecesarnetto@gmail.com
RI Bernasconi, Alessio/AAE-7177-2020; de Cesar Netto, Cesar/D-3046-2018
OI Bernasconi, Alessio/0000-0002-9427-3178; de Cesar Netto,
   Cesar/0000-0001-6037-0685; Honda Saito, Guilherme/0000-0002-1211-9258
CR Adams SB, 2014, FOOT ANKLE INT, V35, P293, DOI 10.1177/1071100713519078
   Aktas S, 2009, FOOT ANKLE INT, V30, P391, DOI 10.3113/FAI.2009.0391
   Bhandari M, 2002, CLIN ORTHOP RELAT R, P190
   Carr Brian J, 2009, Orthopedics, V32, P828, DOI 10.3928/01477447-20090922-11
   Erickson BJ, 2015, ORTHOP J SPORTS MED, V3, DOI 10.1177/2325967115579188
   Garcia-German Diego, 2009, Foot Ankle Surg, V15, P152, DOI 10.1016/j.fas.2008.09.001
   Goren D, 2005, FOOT ANKLE INT, V26, P286, DOI 10.1177/107110070502600404
   Henriquez H, 2012, CLIN ORTHOP RELAT R, V470, P998, DOI 10.1007/s11999-011-1830-1
   Huang JZ, 2015, AM J SPORT MED, V43, P1008, DOI 10.1177/0363546514531014
   Kadakia AR, 2017, J AM ACAD ORTHOP SUR, V25, P23, DOI 10.5435/JAAOS-D-15-00187
   Khan RJK, 2005, J BONE JOINT SURG AM, V87A, P2202, DOI 10.2106/JBJS.D.03049
   Lantto I, 2016, AM J SPORT MED, V44, P2406, DOI 10.1177/0363546516651060
   Lim J, 2001, FOOT ANKLE INT, V22, P559, DOI 10.1177/107110070102200705
   Lock AM, 2017, PATIENT SAF SURG, V11, DOI 10.1186/s13037-017-0132-2
   McMahon Samuel E, 2011, Foot Ankle Surg, V17, P211, DOI 10.1016/j.fas.2010.11.001
   Ollivere BJ, 2014, ANKLE SURG, V20, pE27
   Sanchez M, 2007, AM J SPORT MED, V35, P245, DOI 10.1177/0363546506294078
   Saper D, 2016, ORTHOPEDICS, V39, pE790, DOI 10.3928/01477447-20160503-04
   Schepull T, 2011, AM J SPORT MED, V39, P38, DOI 10.1177/0363546510383515
   Soroceanu A, 2012, J BONE JOINT SURG AM, V94A, P2136, DOI 10.2106/JBJS.K.00917
   Stein BE, 2015, INT ORTHOP, V39, P901, DOI 10.1007/s00264-015-2725-7
   Willits K, 2010, J BONE JOINT SURG AM, V92A, P2767, DOI 10.2106/JBJS.I.01401
   Zhang H, 2015, MEDICINE, V94, DOI 10.1097/MD.0000000000001951
   Zhang YJ, 2017, AM J SPORTS MED
NR 24
TC 1
Z9 1
U1 0
U2 3
PU ELSEVIER SCIENCE INC
PI NEW YORK
PA STE 800, 230 PARK AVE, NEW YORK, NY 10169 USA
SN 1067-2516
EI 1542-2224
J9 J FOOT ANKLE SURG
JI J. Foot Ankle Surg.
PD NOV-DEC
PY 2018
VL 57
IS 6
BP 1272
EP 1277
DI 10.1053/j.jfas.2018.04.002
PG 6
WC Orthopedics; Surgery
WE Science Citation Index Expanded (SCI-EXPANDED)
SC Orthopedics; Surgery
GA GY2AP
UT WOS:000448340000044
PM 30115453
DA 2022-07-22
ER

PT J
AU Silbernagel, KG
   Steele, R
   Manal, K
AF Silbernagel, Karin Graevare
   Steele, Robert
   Manal, Kurt
TI Deficits in Heel-Rise Height and Achilles Tendon Elongation Occur in
   Patients Recovering From an Achilles Tendon Rupture
SO AMERICAN JOURNAL OF SPORTS MEDICINE
LA English
DT Article
DE Achilles tendon Total Rupture Score (ATRS); Foot and Ankle Outcome Score
   (FAOS); tendon elongation; calf raise; toe-raise; calf muscle strength
ID REPAIR; RELIABILITY; ENDURANCE; MULTICENTER; VALIDATION; SCORE
AB Background: Whether an Achilles tendon rupture is treated surgically or not, complications such as muscle weakness, decrease in heel-rise height, and gait abnormalities persist after injury.
   Purpose: The purpose of this study was to evaluate if side-to-side differences in maximal heel-rise height can be explained by differences in Achilles tendon length.
   Study Design: Case series; Level of evidence, 4.
   Method: Eight patients (mean [SD] age of 46 [13] years) with acute Achilles tendon rupture and 10 healthy subjects (mean [SD] age of 28 [8] years) were included in the study. Heel-rise height, Achilles tendon length, and patient-reported outcome were measured 3, 6, and 12 months after injury. Achilles tendon length was evaluated using motion analysis and ultrasound imaging.
   Results: The Achilles tendon length test-retest reliability (intraclass correlation coefficient = 0.97) was excellent. For the healthy subjects, there were no side-to-side differences in tendon length and heel-rise height. Patients with Achilles tendon ruptures had significant differences between the injured and uninjured side for both tendon length (mean [SD] difference, 2.6-3.1 [1.2-1.4] cm, P = .017-.028) and heel-rise height (mean [SD] difference, -4.1 to -6.1 [1.7-1.8] cm, P = .012-.028). There were significant negative correlations (r = -0.943, P = .002, and r = -0.738, P = .037) between the side-to-side difference in heel-rise height and Achilles tendon length at the 6- and 12-month evaluations, respectively.
   Conclusion: The side-to-side difference found in maximal heel-rise height can be explained by a difference in Achilles tendon length in patients recovering from an Achilles tendon rupture. Minimizing tendon elongation appears to be an important treatment goal when aiming for full return of function.
C1 [Silbernagel, Karin Graevare] Allegheny Univ Hlth Sci, Samson Coll Hlth Profess, Dept Phys Therapy, Philadelphia, PA 19104 USA.
   [Steele, Robert] First State Orthopaed, Newark, DE USA.
   [Manal, Kurt] Univ Delaware, Dept Mech Engn, Newark, DE 19716 USA.
RP Silbernagel, KG (通讯作者)，Allegheny Univ Hlth Sci, Samson Coll Hlth Profess, Dept Phys Therapy, 600 S 43rd St, Philadelphia, PA 19104 USA.
EM k.silbernagel@usciences.edu
OI Gravare Silbernagel, Karin/0000-0001-7566-407X
FU Swedish Research Council
FX One or more of the authors has declared the following potential conflict
   of interest or source of funding: This study was funded by the Swedish
   Research Council.
CR Aspenberg P, 2007, INT ORTHOP, V31, P783, DOI 10.1007/s00264-007-0398-6
   Bostick GP, 2010, J ORTHOP SPORT PHYS, V40, P345, DOI 10.2519/jospt.2010.3204
   Denegar CR., 1993, J SPORT REHABIL, V2, P35, DOI [10.1123/jsr.2.1.35, DOI 10.1123/JSR.2.1.35]
   Don R, 2007, CLIN BIOMECH, V22, P211, DOI 10.1016/j.clinbiomech.2006.10.001
   ENWEMEKA CS, 1992, AM J PHYS MED REHAB, V71, P31, DOI 10.1097/00002060-199202000-00008
   Hopkins WG, 2000, SPORTS MED, V30, P1, DOI 10.2165/00007256-200030010-00001
   Kangas J, 2007, AM J SPORT MED, V35, P59, DOI 10.1177/0363546506293255
   Khan RJK, 2005, J BONE JOINT SURG AM, V87A, P2202, DOI 10.2106/JBJS.D.03049
   Kjaer M., 2005, Journal of Musculoskeletal & Neuronal Interactions, V5, P41
   Maffulli N, 2011, KNEE SURG SPORT TR A, V19, P680, DOI 10.1007/s00167-010-1193-2
   Manal K, 2010, J APPL BIOMECH, V26, P224, DOI 10.1123/jab.26.2.224
   Moller M, 2001, J BONE JOINT SURG BR, V83B, P843
   Moller M, 2005, KNEE SURG SPORT TR A, V13, P60, DOI 10.1007/s00167-003-0441-0
   MORTENSEN NHM, 1992, ORTHOPEDICS, V15, P899
   Mullaney MJ, 2006, AM J SPORT MED, V34, P1120, DOI 10.1177/0363546505284186
   Nilsson-Helander K, 2007, AM J SPORT MED, V35, P421, DOI 10.1177/0363546506294856
   Nilsson-Helander K, 2010, AM J SPORT MED, V38, P2186, DOI 10.1177/0363546510376052
   Olsson N, 2011, KNEE SURG SPORT TR A, V19, P1385, DOI 10.1007/s00167-011-1511-3
   Parekh Selene G, 2009, Foot Ankle Spec, V2, P283, DOI 10.1177/1938640009351138
   Rees JD, 2008, RHEUMATOLOGY, V47, P1493, DOI 10.1093/rheumatology/ken262
   Rohner-Spengler M, 2007, J ORTHOP SPORT PHYS, V37, P199, DOI 10.2519/jospt.2007.2371
   Roos EM, 2001, FOOT ANKLE INT, V22, P788, DOI 10.1177/107110070102201004
   Schepull T, 2012, SCAND J MED SCI SPOR, V22, P18, DOI 10.1111/j.1600-0838.2010.01154.x
   Schepull T, 2007, BMC MUSCULOSKEL DIS, V8, DOI 10.1186/1471-2474-8-116
   Silbernagel KG, 2010, KNEE SURG SPORT TR A, V18, P258, DOI 10.1007/s00167-009-0889-7
   Willits K, 2010, J BONE JOINT SURG AM, V92A, P2767, DOI 10.2106/JBJS.I.01401
NR 26
TC 137
Z9 138
U1 0
U2 15
PU SAGE PUBLICATIONS INC
PI THOUSAND OAKS
PA 2455 TELLER RD, THOUSAND OAKS, CA 91320 USA
SN 0363-5465
EI 1552-3365
J9 AM J SPORT MED
JI Am. J. Sports Med.
PD JUL
PY 2012
VL 40
IS 7
BP 1564
EP 1571
DI 10.1177/0363546512447926
PG 8
WC Orthopedics; Sport Sciences
WE Science Citation Index Expanded (SCI-EXPANDED)
SC Orthopedics; Sport Sciences
GA 967NI
UT WOS:000305914500017
PM 22593092
DA 2022-07-22
ER

PT J
AU Kearney, RS
   Achten, J
   Lamb, SE
   Parsons, N
   Costa, ML
AF Kearney, Rebecca S.
   Achten, Juul
   Lamb, Sarah E.
   Parsons, Nicholas
   Costa, Matthew L.
TI The Achilles tendon total rupture score: a study of responsiveness,
   internal consistency and convergent validity on patients with acute
   Achilles tendon ruptures
SO HEALTH AND QUALITY OF LIFE OUTCOMES
LA English
DT Article
DE Achilles tendon; Rupture; Questionnaires; Treatment Outcome
ID QUESTIONNAIRE; INDEX; PERCEPTIONS; REPLACEMENT
AB Background: The Achilles tendon Total Rupture Score was developed by a research group in 2007 in response to the need for a patient reported outcome measure for this patient population. Beyond this original development paper, no further validation studies have been published. Consequently the purpose of this study was to evaluate internal consistency, convergent validity and responsiveness of this newly developed patient reported outcome measure within patients who have sustained an isolated acute Achilles tendon rupture.
   Methods: Sixty-four eligible patients with an acute rupture of their Achilles tendon completed the Achilles tendon Total Rupture Score alongside two further patient reported outcome measures (Disability Rating Index and EQ 5D). These were completed at baseline, six weeks, three months, six months and nine months post injury. The Achilles tendon Total Rupture Score was evaluated for internal consistency, using Cronbach's alpha, convergent validity, through correlation analysis and responsiveness, by analysing floor and ceiling effects and calculating its relative efficiency in comparison to the Disability Rating Index and EQ 5D scores.
   Results: The Achilles tendon Total Rupture Score demonstrated high internal consistency (Cronbachs alpha > 0.8) and correlated significantly (p < 0.001) with the Disability Rating Index at five time points (pre-injury, six weeks, three, six and nine months) with correlation coefficients between -0.5 and -0.9. However, the confidence intervals were wide. Furthermore, the ability of the new score to detect clinically important changes over time (responsiveness) was shown to be greater than the Disability Rating Index and EQ 5D.
   Conclusions: A universally accepted outcome measure is imperative to allow comparisons to be made across practice. This is the first study to evaluate aspects of validity of this newly developed outcome measure, outside of the developing centre. The ATRS demonstrated high internal consistency and responsiveness, with limited convergent validity. This research provides further support for the use of this outcome measure, however further research is required to advocate its universal use in patients with acute Achilles tendon ruptures. Such areas include inter-rater reliability and research to determine the minimally clinically important difference between scores. All authors have read and concur with the content of this manuscript. The material presented has not been and will not be submitted for publication elsewhere, except as an abstract. All authors have made substantial contributions to all of the following: (1) the conception and design of the study, or acquisition of data, or analysis and interpretation of data, (2) drafting the article or revising it critically for important intellectual content and (3) final approval of the submitted version. This research has been funded by Arthritis Research UK, no conflicts of interests have been declared by the authors.
C1 [Kearney, Rebecca S.; Achten, Juul; Parsons, Nicholas] Univ Hosp, Div Hlth Sci, Warwick Med Sch, Clin Sci Res Labs, Coventry CV2 2DX, W Midlands, England.
   [Lamb, Sarah E.; Costa, Matthew L.] Univ Warwick, Warwick Clin Trials Unit, Div Hlth Sci, Warwick Med Sch,Clin Trials Unit, Coventry CV4 7AL, W Midlands, England.
RP Kearney, RS (通讯作者)，Univ Hosp, Div Hlth Sci, Warwick Med Sch, Clin Sci Res Labs, Clifford Bridge Rd, Coventry CV2 2DX, W Midlands, England.
EM r.s.kearney@warwick.ac.uk
RI Parsons, Nick/AAA-8713-2019
OI Kearney, Rebecca/0000-0002-8010-164X; Parsons, Nick/0000-0001-9975-888X;
   Lamb, Sarah/0000-0003-4349-7195
FU Arthritis Research UK; National Institute for Health Research
   [NF-SI-0611-10020] Funding Source: researchfish; Versus Arthritis
   [18858] Funding Source: researchfish
FX The authors would like to thank the financial support of Arthritis
   Research UK.
CR BARR S, 1994, J RHEUMATOL, V21, P2106
   Bland JM, 1997, BRIT MED J, V314, P572, DOI 10.1136/bmj.314.7080.572
   Brooks R, 1996, HEALTH POLICY, V37, P53, DOI 10.1016/0168-8510(96)00822-6
   Dawson J, 1996, J BONE JOINT SURG BR, V78B, P185, DOI 10.1302/0301-620X.78B2.0780185
   Dawson J, 1998, J BONE JOINT SURG BR, V80B, P63, DOI 10.1302/0301-620X.80B1.7859
   DONOVAN JL, 1993, J EPIDEMIOL COMMUN H, V47, P158, DOI 10.1136/jech.47.2.158
   Fitzpatrick R, 1998, Health Technol Assess, V2, pi
   Hudak PL, 1996, AM J IND MED, V29, P602, DOI 10.1002/(SICI)1097-0274(199606)29:6<602::AID-AJIM4>3.0.CO;2-L
   Kearney R, 2011, BRIT J SPORTS MED
   KESSLER RC, 1995, MED CARE, V33, pAS109
   KITAOKA HB, 1994, FOOT ANKLE INT, V15, P349, DOI 10.1177/107110079401500701
   Leppilahti J, 1998, CLIN ORTHOP RELAT R, P152
   Naal FD, 2010, CLIN ORTHOP RELAT R, V468, P815, DOI 10.1007/s11999-009-1036-y
   Nilsson-Helander K, 2007, AM J SPORT MED, V35, P421, DOI 10.1177/0363546506294856
   Robinson JM, 2001, BRIT J SPORT MED, V35, P335, DOI 10.1136/bjsm.35.5.335
   SALEN BA, 1994, J CLIN EPIDEMIOL, V47, P1423, DOI 10.1016/0895-4356(94)90086-8
   Terwee CB, 2007, J CLIN EPIDEMIOL, V60, P34, DOI 10.1016/j.jclinepi.2006.03.012
   Thermann H, 1995, UNFALLCHIRURG, V98, P507
   THOMPSON T C, 1962, J Trauma, V2, P126, DOI 10.1097/00005373-196203000-00003
   Visentini P J, 1998, J Sci Med Sport, V1, P22, DOI 10.1016/S1440-2440(98)80005-4
NR 20
TC 46
Z9 46
U1 0
U2 2
PU BMC
PI LONDON
PA CAMPUS, 4 CRINAN ST, LONDON N1 9XW, ENGLAND
SN 1477-7525
J9 HEALTH QUAL LIFE OUT
JI Health Qual. Life Outcomes
PD FEB 29
PY 2012
VL 10
AR 24
DI 10.1186/1477-7525-10-24
PG 7
WC Health Care Sciences & Services; Health Policy & Services
WE Science Citation Index Expanded (SCI-EXPANDED); Social Science Citation Index (SSCI)
SC Health Care Sciences & Services
GA 916AE
UT WOS:000302068000001
PM 22376047
OA Green Published, gold
DA 2022-07-22
ER

PT J
AU Mendes, AGR
   de Sousa, GGV
   Franca, MD
   de Carvalho, CAM
   Batista, EDO
   Passos, ADF
   Oliveira, KRHM
   Herculano, AM
   de Moraes, SAS
AF Mendes, Aurea Gabriela Rodrigues
   de Sousa, Gabriel Gomes Vilar
   Franca, Martha de Souza
   de Carvalho, Carlos Alberto Marques
   Batista, Evander de Jesus Oliveira
   Passos, Adelaide da Conceicao Fonseca
   Oliveira, Karen Renata Herculano Matos
   Herculano, Anderson Manoel
   de Moraes, Suellen Alessandra Soares
TI Astrocyte reactivity in spinal cord and functional impairment after
   tendon injury in rats
SO HELIYON
LA English
DT Article
DE Achilles tendon; Tendon injury; Astrocyte reactivity; Gait; Mechanical
   sensitivity
ID NEUROPATHIC PAIN; GLIAL ACTIVATION; ACHILLES-TENDON; GAIT ANALYSIS;
   MODEL; ELECTROACUPUNCTURE; RECOVERY
AB Astrocyte reactivity in the spinal cord may occur after peripheral neural damage. However, there is no data to report such reactivity after Achilles tendon injury. We investigate whether changes occur in the spinal cord, mechanical sensitivity and gait in two phases of repair after Achilles tendon injury. Wistar rats were divided into groups: control (CTRL, without rupture), 2 days post-injury (RUP2) and 21 days post-injury (RUP21). Functional and mechanical sensitivity tests were performed at 2 and 21 days post-injury (dpi). The spinal cords were processed, cryosectioned and activated astrocytes were immunostained by GFAP at 21 dpi. Astrocyte reactivity was observed in the L5 segment of the spinal cord with predominance in the white matter regions and decrease in the mechanical threshold of the ipsilateral paw only in RUP2. However, there was gait impairment in both RUP2 and RUP21. We conclude that during the acute phase of Achilles tendon repairment, there was astrocyte reactivity in the spinal cord and impairment of mechanical sensitivity and gait, whereas in the chronic phase only gait remains compromised.
C1 [Mendes, Aurea Gabriela Rodrigues; de Sousa, Gabriel Gomes Vilar; de Moraes, Suellen Alessandra Soares] Univ Fed Para, Inst Ciencias Saude, Belem, Para, Brazil.
   [Franca, Martha de Souza; Passos, Adelaide da Conceicao Fonseca; Oliveira, Karen Renata Herculano Matos; Herculano, Anderson Manoel] Univ Fed Para, Inst Ciencias Biol, Belem, Para, Brazil.
   [de Carvalho, Carlos Alberto Marques] Univ Estado Para, Dept Patol, Belem, Para, Brazil.
   [Batista, Evander de Jesus Oliveira] Univ Fed Para, Nucleo Med Trop, Belem, Para, Brazil.
RP de Moraes, SAS (通讯作者)，Univ Fed Para, Inst Ciencias Saude, Belem, Para, Brazil.
EM sualessandra@yahoo.com.br
OI Moraes, Suellen/0000-0001-8616-6885; Carvalho,
   Carlos/0000-0001-6615-1387
FU Fundacao Amazonia de Amparo a Estudos e Pesquisas (FAPESPA);
   Pro-Reitoria de Pesquisa e Pos-Graduacao da Universidade Federal do Para
   (PROPESP/UFPA)
FX This work was supported by Fundacao Amazonia de Amparo a Estudos e
   Pesquisas (FAPESPA) and Pro-Reitoria de Pesquisa e Pos-Graduacao da
   Universidade Federal do Para (PROPESP/UFPA).
CR Abaei M, 2016, MOL PAIN, V12, DOI 10.1177/1744806916642445
   Aro AA, 2013, INJURY, V44, P884, DOI 10.1016/j.injury.2012.08.055
   Barmakian J.T., 1994, J HAND SURG, V19
   Bressan E, 2010, LIFE SCI, V87, P481, DOI 10.1016/j.lfs.2010.09.002
   Cirillo G, 2011, NEUROBIOL DIS, V41, P630, DOI 10.1016/j.nbd.2010.11.012
   Cirillo G, 2010, CELL MOL NEUROBIOL, V30, P51, DOI 10.1007/s10571-009-9430-2
   Clark AK, 2013, J PAIN RES, V6, P803, DOI 10.2147/JPR.S53660
   Colangelo AM, 2008, J NEUROSCI, V28, P2698, DOI 10.1523/JNEUROSCI.5201-07.2008
   Coulthard P., 2003, J NEUROSCI METHODS, V128
   Danielson P, 2009, BRIT J SPORT MED, V43, P265, DOI 10.1136/bjsm.2008.054593
   de Cesar Netto C., 2018, PLOS ONE, V13
   Gallo A, 2015, NEUROSCI RES, V95, P78, DOI 10.1016/j.neures.2015.02.004
   Ghilardi JR, 2004, J NEUROSCI, V24, P2727, DOI 10.1523/JNEUROSCI.5054-03.2004
   Goff J.R., 1997, NEUROSCIENCE, V82
   Hansen R.R., 2013, EUR J PHARM, V716
   Huang WY, 2015, PLOS ONE, V10, DOI 10.1371/journal.pone.0125022
   Larsson M, 2011, NEUROSCIENTIST, V17, P256, DOI 10.1177/1073858409349913
   Li HY, 2016, BIOMED RES INT, V2016, DOI 10.1155/2016/6492597
   Li L., 2019, FRONT CELL NEUROSCI, V13
   Liang JI, 2012, ANN BIOMED ENG, V40, P2532, DOI 10.1007/s10439-012-0619-z
   Longo UG, 2009, SPORTS MED ARTHROSC, V17, P112, DOI 10.1097/JSA.0b013e3181a3d625
   Lui P.P.Y., 2010, AM J SPORTS MED, V38
   Magni G, 2019, CEPHALALGIA, V39, P1809, DOI 10.1177/0333102419861710
   McMahon SB, 2009, NEURON, V64, P46, DOI 10.1016/j.neuron.2009.09.033
   Moraes SAS, 2013, CELL TISSUE RES, V353, P457, DOI 10.1007/s00441-013-1662-2
   MURRELL GAC, 1992, J ORTHOPAED RES, V10, P398, DOI 10.1002/jor.1100100313
   Olsson N., 2011, KNEE SURG SPORTS TRA, V19
   Pekny M, 2005, GLIA, V50, P427, DOI 10.1002/glia.20207
   Pekny M, 2014, PHYSIOL REV, V94, P1077, DOI 10.1152/physrev.00041.2013
   Pekny M, 2014, NEUROSCI LETT, V565, P30, DOI 10.1016/j.neulet.2013.12.071
   Previtali SC, 2003, J NEUROSCI, V23, P5520
   Rothman SM, 2007, BRAIN RES, V1181, P30, DOI 10.1016/j.brainres.2007.08.064
   Samad TA, 2001, NATURE, V410, P471, DOI 10.1038/35068566
   Shan S, 2007, NEUROBIOL DIS, V26, P558, DOI 10.1016/j.nbd.2007.02.007
   Sun S, 2008, BRAIN RES BULL, V75, P83, DOI 10.1016/j.brainresbull.2007.07.027
   Taccola G, 2016, EXP NEUROL, V286, P1, DOI 10.1016/j.expneurol.2016.09.008
   Tawfik VL, 2007, BRAIN BEHAV IMMUN, V21, P238, DOI 10.1016/j.bbi.2006.07.001
   Wang W., 2009, PLOS ONE, V4
   Yu PR, 2001, MUSCLE NERVE, V24, P231, DOI 10.1002/1097-4598(200102)24:2<231::AID-MUS80>3.0.CO;2-5
   Zhang HJ, 2012, J PAIN, V13, P293, DOI 10.1016/j.jpain.2011.12.002
NR 40
TC 0
Z9 1
U1 0
U2 1
PU ELSEVIER SCI LTD
PI OXFORD
PA THE BOULEVARD, LANGFORD LANE, KIDLINGTON, OXFORD OX5 1GB, OXON, ENGLAND
EI 2405-8440
J9 HELIYON
JI Heliyon
PD APR
PY 2021
VL 7
IS 4
AR e06845
DI 10.1016/j.heliyon.2021.e06845
EA APR 2021
PG 6
WC Multidisciplinary Sciences
WE Science Citation Index Expanded (SCI-EXPANDED)
SC Science & Technology - Other Topics
GA SB0XP
UT WOS:000649726700015
PM 33981899
OA gold, Green Published
DA 2022-07-22
ER

PT J
AU Cretnik, A
   Kosir, R
   Kosanovic, M
AF Cretnik, Andrej
   Kosir, Roman
   Kosanovic, Milos
TI Incidence and Outcome of Operatively Treated Achilles Tendon Rupture in
   the Elderly
SO FOOT & ANKLE INTERNATIONAL
LA English
DT Article
DE Achilles Tendon; Rupture; Elderly Person; Incidence; Treatment; Outcome
ID NON-SURGICAL TREATMENT; NONOPERATIVE TREATMENT; REPAIR
AB Background: Very little has been published about Achilles tendon rupture in the elderly. Optimal therapy is controversial with conservative treatment generally recommended. The purpose of our study was to find the incidence and outcome of operatively treated Achilles tendon ruptures in the elderly. Materials and Methods: We determined the incidence of a closed complete Achilles tendon rupture in a period from 1991 to 2000 in two centers caring for 572,929 people with 108,668 people over 60 years of age. Results: In a 10-year period there were 434 ruptures, all of which were treated operatively: 146 in an open fashion and 288 percutaneously. The average incidence was 7.6 ruptures per 100,000 people. The average age of patients was 38.7 years, with a male-to-female ratio of 16.7:1. There were 14 ruptures in 13 patients older than 60 years, with the incidence of 1.28 ruptures per 100,000 people. Seven of the ruptures were operated on in an open way under spinal anesthesia and seven percutaneously under local anesthesia. The average age of the patients was 67.9 years, with a male-to-female ratio of 1.6:1 and the mean ASA score 1.64. There were no major complications in either group. One patient in the percutaneous group had transient sural nerve injury and one patient in the open group had a superficial infection. All of the patients returned to their previous activities, four of them with some limitations. The average AOFAS score was 93.1 points. Conclusion: Achilles tendon rupture in the elderly is a rare injury. Operative treatment can yield a successful outcome.
C1 [Cretnik, Andrej; Kosir, Roman] Univ Med Ctr Maribor, Dept Traumatol, Maribor 2000, Slovenia.
   [Kosanovic, Milos] Gen & Teaching Hosp Celje, Dept Traumatol, Celje, Slovenia.
RP Cretnik, A (通讯作者)，Univ Med Ctr Maribor, Dept Traumatol, Ljubljanska 5, Maribor 2000, Slovenia.
EM andrej.cretnik@guest.arnes.si
CR BRADLEY JP, 1990, AM J SPORT MED, V18, P188, DOI 10.1177/036354659001800213
   CETTI R, 1993, AM J SPORT MED, V21, P791, DOI 10.1177/036354659302100606
   Cretnik A, 2005, AM J SPORT MED, V33, P1369, DOI 10.1177/0363546504271501
   Cretnik A, 2000, MED SCI SPORT EXER, V32, P16
   Gorschewsky O, 2004, FOOT ANKLE INT, V25, P219, DOI 10.1177/107110070402500405
   KHAN RJK, 2004, COCHRANE DATABASE SY, V3
   KITAOKA HB, 1994, FOOT ANKLE INT, V15, P349, DOI 10.1177/107110079401500701
   Kocher MS, 2002, AM J SPORT MED, V30, P783, DOI 10.1177/03635465020300060501
   LEA RB, 1972, J BONE JOINT SURG AM, VA 54, P1398, DOI 10.2106/00004623-197254070-00002
   Leppilahti J, 1996, ACTA ORTHOP SCAND, V67, P277, DOI 10.3109/17453679608994688
   Leppilahti J, 1998, CLIN ORTHOP RELAT R, P152
   LITTLE JP, 1995, ANAESTHESIA, V50, P658
   Lo IKY, 1997, CLIN J SPORT MED, V7, P207, DOI 10.1097/00042752-199707000-00010
   Maffulli N, 1999, J BONE JOINT SURG AM, V81A, P1019, DOI 10.2106/00004623-199907000-00017
   Maffulli N, 1999, CLIN J SPORT MED, V9, P157, DOI 10.1097/00042752-199907000-00007
   Maffulli Nicola, 2007, Foot Ankle Clin, V12, P583, DOI 10.1016/j.fcl.2007.07.007
   Moller A, 1996, ACTA ORTHOP SCAND, V67, P479, DOI 10.3109/17453679608996672
   Movin Tomas, 2005, Foot Ankle Clin, V10, P331, DOI 10.1016/j.fcl.2005.01.003
   Nestorson J, 2000, ACTA ORTHOP SCAND, V71, P64, DOI 10.1080/00016470052943928
   NISTOR L, 1981, J BONE JOINT SURG AM, V63, P394, DOI 10.2106/00004623-198163030-00012
   Ryf C, 1995, INJURY S1, V26, P1
   Suchak AA, 2005, FOOT ANKLE INT, V26, P932, DOI 10.1177/107110070502601106
   THERMANN H, 1995, UNFALLCHIRURG, V98, P21
   THOMPSON T C, 1962, Acta Orthop Scand, V32, P461, DOI 10.3109/17453676208989608
   WASHBURN SD, 1992, CLIN ORTHOP RELAT R, P237
   WILLS CA, 1986, CLIN ORTHOP RELAT R, P156
NR 26
TC 14
Z9 15
U1 1
U2 1
PU AMER ORTHOPAEDIC FOOT & ANKLE SOC, INC
PI SEATTLE
PA 2517 EASTLAKE AVE EAST, STE 200, SEATTLE, WA 98102 USA
SN 1071-1007
J9 FOOT ANKLE INT
JI Foot Ankle Int.
PD JAN
PY 2010
VL 31
IS 1
BP 14
EP 18
DI 10.3113/FAI.2010.0014
PG 5
WC Orthopedics
WE Science Citation Index Expanded (SCI-EXPANDED)
SC Orthopedics
GA 539IU
UT WOS:000273248400003
PM 20067717
DA 2022-07-22
ER

PT J
AU Hansen, MS
   Kristensen, MT
   Budolfsen, T
   Ellegaard, K
   Holmich, P
   Barfod, KW
AF Hansen, Maria Swennergren
   Kristensen, Morten Tange
   Budolfsen, Thomas
   Ellegaard, Karen
   Holmich, Per
   Barfod, Kristoffer Weisskirchner
TI Reliability of the Copenhagen Achilles length measure (CALM) on patients
   with an Achilles tendon rupture
SO KNEE SURGERY SPORTS TRAUMATOLOGY ARTHROSCOPY
LA English
DT Article
DE Achilles tendon rupture; Elongation; Ultrasonographic measure;
   Reliability
ID RESTING ANGLE; ELONGATION; STRENGTH; REPAIR; RETURN
AB Purpose The primary objective was to examine the reliability of the Copenhagen Achilles length measure (CALM) in patients with an Achilles tendon rupture and secondary to examine the reliability of Achilles tendon resting angle (ATRA) and Achilles tendon length measure (ATLM). Method The study was executed as a cross-sectional study on two different groups: one focused on CALM and the other on ATRA/ATLM. CALM was performed on 56 patients at four timepoints during the first year after injury, whereas ATRA/ATLM were carried out on 28 patients. Intra- and inter-rater reliabilities were determined using the intra-class correlation coefficient (ICC), the standard error of the measurement (SEM), and the minimal detectable change (MDC). Results For CALM, all measurements, both for injured and non-injured sides as well as for elongation, indicated excellent relative reliability (ICC >= 0.75). During the four timepoints, the inter-rater absolute reliability had an SEM that ranged between 0.3 and 0.8 cm (1-4 SEM%) for injured and non-injured sides and 0.3-0.6 cm (18-29 SEM%) for elongation. On an individual level, the inter-rater absolute reliability had an MDC ranging between 0.8 and 2.2 cm (4-11 MDC%) for injured and non-injured sides and 0.8-1.7 cm (47-81 MDC%) for elongation. In the case of ATRA, relative reliability was excellent (ICC >= 0.75), and for ATLM, it was fair to excellent (ICC 0.58-0.79). ATRA presented a lower measurement error than ATLM. Conclusion Copenhagen Achilles length measure showed excellent relative reliability, but had a significant measurement error at four timepoints the first year following an Achilles tendon rupture.
C1 [Hansen, Maria Swennergren; Kristensen, Morten Tange; Budolfsen, Thomas] Copenhagen Univ Hosp Amager, Dept Phys,Occupat Therapy,Phys Med,Rehabil Res PM, Hvidovre, Denmark.
   [Hansen, Maria Swennergren; Holmich, Per; Barfod, Kristoffer Weisskirchner] Copenhagen Univ Hosp Amager, Dept Orthped Surg Sports Orthped Res Ctr Copenhag, Hvidovre, Denmark.
   [Kristensen, Morten Tange] Copenhagen Univ Hosp Amager, Dept Orthpaed Surg, Hvidovre, Denmark.
   [Ellegaard, Karen] Frederiksberg Univ Hosp, Parker Inst, Frederiksberg, Denmark.
   [Hansen, Maria Swennergren] Hvidovre Univ Hosp, Fysio og Ergotraput afdeling, Kettegard 30, DK-2650 Hvidovre, Denmark.
RP Hansen, MS (通讯作者)，Copenhagen Univ Hosp Amager, Dept Phys,Occupat Therapy,Phys Med,Rehabil Res PM, Hvidovre, Denmark.; Hansen, MS (通讯作者)，Copenhagen Univ Hosp Amager, Dept Orthped Surg Sports Orthped Res Ctr Copenhag, Hvidovre, Denmark.; Hansen, MS (通讯作者)，Hvidovre Univ Hosp, Fysio og Ergotraput afdeling, Kettegard 30, DK-2650 Hvidovre, Denmark.
EM maria_swennergren@hotmail.com
RI Barfod, Kristoffer/AAY-3288-2021
OI Barfod, Kristoffer/0000-0003-2620-5891; Holmich,
   Per/0000-0003-2098-0272; Kristensen, Morten Tange/0000-0001-5868-4677
CR Barfod KW, 2018, DAN MED J, V65
   Barfod KW, 2016, TRIALS, V17, DOI 10.1186/s13063-016-1697-2
   Barfod KW, 2015, KNEE SURG SPORT TR A, V23, P3398, DOI 10.1007/s00167-014-3175-2
   Beckerman H, 2001, QUAL LIFE RES, V10, P571, DOI 10.1023/A:1013138911638
   BLAND JM, 1986, LANCET, V1, P307, DOI 10.1016/s0140-6736(86)90837-8
   Brouwer EF, 2018, KNEE SURG SPORT TR A, V26, P2088, DOI 10.1007/s00167-017-4815-0
   Carmont MR, 2013, FOOT ANKLE SURG, V19, P245, DOI 10.1016/j.fas.2013.06.007
   Carmont MR, 2015, ASIA-PAC J SPORT MED, V2, P49, DOI 10.1016/j.asmart.2014.12.002
   Eliasson P, 2018, AM J SPORT MED, V46, P2492, DOI 10.1177/0363546518781826
   Flansbjer UB, 2005, CLIN REHABIL, V19, P514, DOI 10.1191/0269215505cr854oa
   Fleiss JL., 1999, RELIABILITY MEASUREM, DOI [10.1002/9781118032923, DOI 10.1002/9781118032923]
   Ganestam A, 2016, KNEE SURG SPORT TR A, V24, P3730, DOI 10.1007/s00167-015-3544-5
   Hansen MS, 2017, FOOT ANKLE SURG, V23, P275, DOI 10.1016/j.fas.2016.08.002
   Hansen MS, 2016, KNEE SURG SPORT TR A, V24, P1365, DOI 10.1007/s00167-015-3974-0
   Harvill LM, 1991, ED MEASUREMENT ISSUE, V10, P33, DOI DOI 10.1111/J.1745-3992.1991.TB00195.X
   Heikkinen J, 2017, J BONE JOINT SURG AM, V99, P1509, DOI 10.2106/JBJS.16.01491
   Kangas J, 2007, AM J SPORT MED, V35, P59, DOI 10.1177/0363546506293255
   Kristensen MT, 2014, GAIT POSTURE, V39, P424, DOI 10.1016/j.gaitpost.2013.08.017
   Matles A L, 1975, Bull Hosp Joint Dis, V36, P48
   Moller M, 2002, SCAND J MED SCI SPOR, V12, P9, DOI 10.1034/j.1600-0838.2002.120103.x
   Olsson N, 2011, KNEE SURG SPORT TR A, V19, P1385, DOI 10.1007/s00167-011-1511-3
   Rees JD, 2008, RHEUMATOLOGY, V47, P1493, DOI 10.1093/rheumatology/ken262
   Schepull T, 2012, SCAND J MED SCI SPOR, V22, P18, DOI 10.1111/j.1600-0838.2010.01154.x
   Silbernagel KG, 2012, AM J SPORT MED, V40, P1564, DOI 10.1177/0363546512447926
   Silbernagel KG, 2016, MLTJ-MUSCLE LIGAMENT, V6, P104, DOI 10.11138/mltj/2016.6.1.104
   Vandenbroucke JP, 2007, EPIDEMIOLOGY, V18, P805, DOI [10.1371/journal.pmed.0040297, 10.1097/EDE.0b013e3181577511]
   Weir JP, 2005, J STRENGTH COND RES, V19, P231, DOI 10.1519/15184.1
   Zellers JA, 2018, FOOT ANKLE INT, V39, P343, DOI 10.1177/1071100717742372
   Zellers JA, 2016, BRIT J SPORT MED, V50, P1325, DOI 10.1136/bjsports-2016-096106
NR 29
TC 7
Z9 7
U1 0
U2 0
PU SPRINGER
PI NEW YORK
PA ONE NEW YORK PLAZA, SUITE 4600, NEW YORK, NY, UNITED STATES
SN 0942-2056
EI 1433-7347
J9 KNEE SURG SPORT TR A
JI Knee Surg. Sports Traumatol. Arthrosc.
PD JAN
PY 2020
VL 28
IS 1
SI SI
BP 281
EP 290
DI 10.1007/s00167-019-05672-3
PG 10
WC Orthopedics; Sport Sciences; Surgery
WE Science Citation Index Expanded (SCI-EXPANDED)
SC Orthopedics; Sport Sciences; Surgery
GA KJ0AA
UT WOS:000511719300037
PM 31432242
DA 2022-07-22
ER

PT J
AU Wei, SJ
   Chen, J
   Kong, CW
   Xu, F
   Zhi, XS
   Cai, XH
AF Wei, Shijun
   Chen, Jia
   Kong, Changwang
   Xu, Feng
   Zhi, Xiaosong
   Cai, Xianhua
TI Endoscopic "internal splinting" repair technique for acute Achilles
   tendon rupture
SO ARCHIVES OF ORTHOPAEDIC AND TRAUMA SURGERY
LA English
DT Article
DE Endoscopy; Arthroscopy; Achilles tendon rupture; Suture technique;
   Knotless anchor
AB Introduction Recently, endoscopically assisted Achilles tendon repair techniques have improved to overcome the surgical complications. However, the risk of sural nerve injury and the strength of repair are still the most concerning aspects. Material and methods Twenty three patients with acute Achilles tendon rupture were reviewed in the present study. We stitch the Achilles tendon above the ruptured site using the endoscopic locking loop suture technique, and the knotless anchor suture-bridge technique can be used to make the distal fixation of threads. The function was assessed using the muscle power (MRC0-5), ATRS scores, AOFAS ankle-hindfoot scores, and VAS scores at the final follow-up. Results The mean follow-up time was 15.74 +/- 2.43 months (12-18). At the final follow-up, the average of the muscle power (MRC0-5), ATRS score, AOFAS ankle-hindfoot score, and VAS score are 4.74 +/- 0.45, 97.83 +/- 2.77, 96.52 +/- 4.87, and 0.35 +/- 0.49, respectively. Every patient returned to previous sports activity at 6 months postoperative. No wound infection and sural nerve injuries were encountered. Only one case suffers local irritation at the medial knotless anchor site. Conclusions Endoscopic "internal splinting" repair for acute Achilles tendon rupture using locking loop stitch with suture-bridge technique leads to an expedited return to activity with a low risk of complications.
C1 [Wei, Shijun; Chen, Jia; Kong, Changwang; Xu, Feng; Zhi, Xiaosong; Cai, Xianhua] Gen Hosp Cent Theater Command, Dept Orthopaed, 627 Wuluo Rd, Wuhan 430030, Hubei, Peoples R China.
   [Wei, Shijun; Xu, Feng; Cai, Xianhua] Southern Med Univ, Clin Med Sch 1, Guangzhou, Guangdong, Peoples R China.
RP Wei, SJ; Xu, F (通讯作者)，Gen Hosp Cent Theater Command, Dept Orthopaed, 627 Wuluo Rd, Wuhan 430030, Hubei, Peoples R China.; Wei, SJ; Xu, F (通讯作者)，Southern Med Univ, Clin Med Sch 1, Guangzhou, Guangdong, Peoples R China.
EM wsj1974@yeah.net; gkxf79390@sohu.com
RI Cai, Xian-Hua/ABA-2969-2021
OI Cai, Xian-Hua/0000-0002-2080-6656
CR ARACIL J, 1992, FOOT ANKLE, V13, P350, DOI 10.1177/107110079201300610
   Assal M, 2002, J BONE JOINT SURG AM, V84A, P161, DOI 10.2106/00004623-200202000-00001
   Baumfeld D, 2019, FOOT ANKLE SURG, V25, P503, DOI 10.1016/j.fas.2018.03.003
   Cretnik A, 2005, AM J SPORT MED, V33, P1369, DOI 10.1177/0363546504271501
   Deng SL, 2017, J FOOT ANKLE SURG, V56, P1236, DOI 10.1053/j.jfas.2017.05.036
   FITZGIBBONS RE, 1993, AM J SPORT MED, V21, P724, DOI 10.1177/036354659302100516
   Flavin R, 2007, INJURY, V38, P845, DOI 10.1016/j.injury.2006.10.016
   Fortis AP, 2008, ARTHROSCOPY, V24, P683, DOI 10.1016/j.arthro.2008.02.018
   Gatz M, 2021, ARCH ORTHOP TRAUM SU, V141, P383, DOI 10.1007/s00402-020-03437-z
   Haji A, 2004, FOOT ANKLE INT, V25, P215, DOI 10.1177/107110070402500404
   Heckman DS, 2009, AM J SPORT MED, V37, P1223, DOI 10.1177/0363546509335947
   Henriquez H, 2012, CLIN ORTHOP RELAT R, V470, P998, DOI 10.1007/s11999-011-1830-1
   Hsu AR, 2015, FOOT ANKLE INT, V36, P1279, DOI 10.1177/1071100715589632
   LAGERGREN C, 1959, Acta Chir Scand, V116, P491
   Leppilahti J, 1996, ACTA ORTHOP SCAND, V67, P277, DOI 10.3109/17453679608994688
   Lim J, 2001, FOOT ANKLE INT, V22, P559, DOI 10.1177/107110070102200705
   Maffulli N, 1999, CLIN J SPORT MED, V9, P157, DOI 10.1097/00042752-199907000-00007
   Mavrodontidis A, 2015, ACTA ORTHOP TRAUMATO, V49, P661, DOI 10.3944/AOTT.2015.14.0196
   Paavola M, 2002, J BONE JOINT SURG AM, V84A, P2062, DOI 10.2106/00004623-200211000-00024
   Reda Y, 2020, FOOT ANKLE SURG, V26, P280, DOI 10.1016/j.fas.2019.03.010
   Rungprai C, 2018, ARTHROSCOPY, V34, P1262, DOI 10.1016/j.arthro.2017.09.027
   Sarman H, 2016, J FOOT ANKLE SURG, V55, P965, DOI 10.1053/j.jfas.2016.04.014
   SCHMIDTROHLFING B, 1992, INT ORTHOP, V16, P29
   Severyns M, 2019, ARTHROSC TEC, V8, pE489, DOI 10.1016/j.eats.2019.01.007
   Tang KL, 2007, AM J SPORT MED, V35, P589, DOI 10.1177/0363546506295080
   Tejwani Nirmal C, 2014, Am J Orthop (Belle Mead NJ), V43, pE221
   Tian J, 2020, ARCH ORTHOP TRAUM SU, V140, P1775, DOI 10.1007/s00402-020-03535-y
   Turgut A, 2002, KNEE SURG SPORT TR A, V10, P130, DOI 10.1007/s00167-001-0258-7
   WATSON TW, 1995, FOOT ANKLE INT, V16, P191, DOI 10.1177/107110079501600404
   Wolff KS, 2012, EUR J RADIOL, V81, P1211, DOI 10.1016/j.ejrad.2011.03.015
   Zappia M, 2018, FOOT ANKLE SURG, V24, P342, DOI 10.1016/j.fas.2017.03.006
   Zhou K, 2018, J FOOT ANKLE SURG, V57, P1191, DOI 10.1053/j.jfas.2018.05.007
NR 32
TC 2
Z9 3
U1 0
U2 5
PU SPRINGER
PI NEW YORK
PA ONE NEW YORK PLAZA, SUITE 4600, NEW YORK, NY, UNITED STATES
SN 0936-8051
EI 1434-3916
J9 ARCH ORTHOP TRAUM SU
JI Arch. Orthop. Trauma Surg.
PD OCT
PY 2021
VL 141
IS 10
BP 1753
EP 1760
DI 10.1007/s00402-021-03818-y
EA FEB 2021
PG 8
WC Orthopedics; Surgery
WE Science Citation Index Expanded (SCI-EXPANDED)
SC Orthopedics; Surgery
GA UT8SQ
UT WOS:000618589000001
PM 33594461
DA 2022-07-22
ER

PT J
AU Hullfish, TJ
   O'Connor, KM
   Baxter, JR
AF Hullfish, Todd J.
   O'Connor, Kathryn M.
   Baxter, Josh R.
TI Gastrocnemius fascicles are shorter and more pennate throughout the
   first month following acute Achilles tendon rupture
SO PEERJ
LA English
DT Article
DE Muscle; Remodeling; Pennation; Gastrocnemius; Plantarflexors; Achilles
   tendon; Rupture; Fascicle; Ultrasound
ID NONAUGMENTED SURGICAL REPAIR; HEEL-RISE HEIGHT; INCREASING INCIDENCE;
   SARCOMERE NUMBER; RANDOMIZED-TRIAL; MUSCLE FASCICLE; LENGTH; STRENGTH;
   ANKLE; ARCHITECTURE
AB The purpose of this study was to characterize the short-term effects of Achilles tendon ruptures on medial gastrocnemius. We hypothesized that the fascicles of the medial gastrocnemius muscle of the injured Achilles tendon would be shorter and more pennate immediately following the injury and would persist throughout 4 weeks post-injury. B-mode longitudinal ultrasound images of the medial gastrocnemius were acquired in 10 adults who suffered acute Achilles tendon ruptures and were treated non-operatively. Ultrasound images were acquired during the initial clinical visit following injury as well as 2 and 4 weeks following this initial clinical visit. Resting muscle structure was characterized by measuring fascicle length, pennation angle, muscle thickness, and muscle echo intensity in both the injured and contralateral (control) limbs. Fascicle length was 15% shorter (P < 0.001) and pennation angle was 21% greater (P < 0.001) in the injured muscle compared to the uninjured (control) muscle at the presentation of injury (week 0). These differences in fascicle length persisted through 4 weeks after injury (P < 0.002) and pennation angle returned to pre-injury levels. Muscle thickness changes were not detected at any of the post-injury visits (difference < 4%, P > 0.026). Echo intensity of the injured limb was 8% lower at the presentation of the injury but was not different compared to the contralateral muscle at 2 and 4 weeks post-injury. Our results suggest that Achilles tendon ruptures elicit rapid changes in the configuration of the medial gastrocnemius, which may explain long-term functional deficits.
C1 [Hullfish, Todd J.; O'Connor, Kathryn M.; Baxter, Josh R.] Univ Penn, Sch Med, Dept Orthopaed Surg, Philadelphia, PA 19104 USA.
RP Baxter, JR (通讯作者)，Univ Penn, Sch Med, Dept Orthopaed Surg, Philadelphia, PA 19104 USA.
EM josh.baxter@uphs.upenn.edu
RI Baxter, Josh/N-4020-2013
OI Baxter, Josh/0000-0003-0269-2170
FU Thomas B. McCabe and Jeannette E. Laws McCabe Fund
FX This work was supported by the Thomas B. McCabe and Jeannette E. Laws
   McCabe Fund. The funders had no role in study design, data collection
   and analysis, decision to publish, or preparation of the manuscript.
CR Baxter JR, 2018, J BIOMECH, V79, P238, DOI 10.1016/j.jbiomech.2018.08.016
   Baxter JR, 2014, J APPL PHYSIOL, V116, P538, DOI 10.1152/japplphysiol.01140.2013
   Baxter JR, 2018, 415679 BIORXIV, DOI [10.1101/415679, DOI 10.1101/415679]
   Bolsterlee B, 2016, J BIOMECH, V49, P1002, DOI 10.1016/j.jbiomech.2016.02.014
   Brorsson A, 2018, AM J SPORT MED, V46, P470, DOI 10.1177/0363546517737055
   Brorsson A, 2017, AM J SPORT MED, V45, P3060, DOI 10.1177/0363546517717698
   Burkholder TJ, 1998, J EXP BIOL, V201, P309
   Drazan JF, 2019, BIORXIV, DOI [10.1101/547042, DOI 10.1101/547042]
   Drazan JF, 2019, PEERJ PREPRINT, V7, DOI [10.7287/peerj.preprints.27475v1, DOI 10.7287/PEERJ.PREPRINTS.27475V1]
   Fukumoto Y, 2012, EUR J APPL PHYSIOL, V112, P1519, DOI 10.1007/s00421-011-2099-5
   Gao F, 2009, ARCH PHYS MED REHAB, V90, P819, DOI 10.1016/j.apmr.2008.11.004
   Hagan KL, 2018, J APPL PHYSIOL, V125, P1743, DOI 10.1152/japplphysiol.00723.2018
   Heikkinen J, 2017, J BONE JOINT SURG AM, V99, P1509, DOI 10.2106/JBJS.16.01491
   Heikkinen J, 2017, AM J SPORT MED, V45, P1395, DOI 10.1177/0363546517694610
   Heikkinen J, 2016, J BONE JOINT SURG AM, V98, P85, DOI 10.2106/JBJS.O.00496
   Hirata K, 2015, J BIOMECH, V48, P1210, DOI 10.1016/j.jbiomech.2015.01.039
   Hortobagyi T, 2000, J PHYSIOL-LONDON, V524, P293, DOI 10.1111/j.1469-7793.2000.00293.x
   Hug F, 2013, J BIOMECH, V46, P2534, DOI 10.1016/j.jbiomech.2013.07.015
   Huttunen TT, 2014, AM J SPORT MED, V42, P2419, DOI 10.1177/0363546514540599
   Kangas J, 2007, AM J SPORT MED, V35, P59, DOI 10.1177/0363546506293255
   Koh TJ, 1998, J PHYSIOL-LONDON, V508, P267, DOI 10.1111/j.1469-7793.1998.267br.x
   Kwah LK, 2013, J APPL PHYSIOL, V114, P761, DOI 10.1152/japplphysiol.01430.2011
   Lantto I, 2015, SCAND J MED SCI SPOR, V25, pE133, DOI 10.1111/sms.12253
   Lee SSM, 2009, J EXP BIOL, V212, P3700, DOI 10.1242/jeb.031096
   Leppilahti J, 2000, FOOT ANKLE INT, V21, P330, DOI 10.1177/107110070002100410
   Mangine GT, 2014, J ATHL TRAINING, V49, P794, DOI 10.4085/1062-6050-49.3.60
   Mortensen NHM, 1999, J BONE JOINT SURG AM, V81A, P983, DOI 10.2106/00004623-199907000-00011
   Mullaney MJ, 2006, AM J SPORT MED, V34, P1120, DOI 10.1177/0363546505284186
   NYSTROM B, 1983, ACTA ORTHOP SCAND, V54, P620, DOI 10.3109/17453678308992899
   Nyyssonen T, 2008, SCAND J SURG, V97, P272, DOI 10.1177/145749690809700312
   Olsson N, 2011, KNEE SURG SPORT TR A, V19, P1385, DOI 10.1007/s00167-011-1511-3
   Pajala A, 2009, J BONE JOINT SURG AM, V91A, P1092, DOI 10.2106/JBJS.G.01089
   Peng WC, 2017, CLIN BIOMECH, V43, P50, DOI 10.1016/j.clinbiomech.2017.02.002
   Raikin SM, 2013, FOOT ANKLE INT, V34, P475, DOI 10.1177/1071100713477621
   Rosso C, 2013, KNEE SURG SPORT TR A, V21, P1369, DOI 10.1007/s00167-013-2407-1
   Salzano MQ, 2018, J BIOMECH, V80, P1, DOI 10.1016/j.jbiomech.2018.09.001
   Sanchez GN, 2015, NEURON, V88, P1109, DOI 10.1016/j.neuron.2015.11.022
   Silbernagel KG, 2012, AM J SPORT MED, V40, P1564, DOI 10.1177/0363546512447926
   Suydam SM, 2015, KNEE SURG SPORT TR A, V23, P868, DOI 10.1007/s00167-013-2512-1
   WILLIAMS PE, 1990, ANN RHEUM DIS, V49, P316, DOI 10.1136/ard.49.5.316
   WILLIAMS PE, 1978, J ANAT, V127, P459
   Willits K, 2010, J BONE JOINT SURG AM, V92A, P2767, DOI 10.2106/JBJS.I.01401
   Wren TAL, 2010, J PEDIATR ORTHOPED, V30, P479, DOI 10.1097/BPO.0b013e3181e00c80
   Zellers JA, 2018, FOOT ANKLE INT, V39, P343, DOI 10.1177/1071100717742372
   Zellers JA, 2016, BRIT J SPORT MED, V50, P1325, DOI 10.1136/bjsports-2016-096106
NR 45
TC 11
Z9 11
U1 0
U2 1
PU PEERJ INC
PI LONDON
PA 341-345 OLD ST, THIRD FLR, LONDON, EC1V 9LL, ENGLAND
SN 2167-8359
J9 PEERJ
JI PeerJ
PD APR 23
PY 2019
VL 7
AR e6788
DI 10.7717/peerj.6788
PG 14
WC Multidisciplinary Sciences
WE Science Citation Index Expanded (SCI-EXPANDED)
SC Science & Technology - Other Topics
GA HU3SU
UT WOS:000465195300006
PM 31065459
OA Green Published, Green Submitted, gold
DA 2022-07-22
ER

PT J
AU Prejbeanu, R
   Vermesan, D
   Balanescu, A
   Mioc, ML
   Haragus, H
AF Prejbeanu, Radu
   Vermesan, Dinu
   Balanescu, Andrei
   Mioc, Mihail Lazar
   Haragus, Horia
TI Repair of acute Achilles tears with plantaris augmentation
SO INTERNATIONAL ORTHOPAEDICS
LA English
DT Article
DE Achilles tendon injuries; Achilles tendon surgery; Plantaris tendon;
   Suture techniques; Tendon surgery; Foot surgery
ID PROSPECTIVE RANDOMIZED-TRIAL; TENDON RUPTURES
AB Purpose To determine the benefits associated with using the fanned out plantaris tendon as a membrane to cover and augment the acute Achilles tendon midportion end-to-end suture repair. Methods Between 2014 and 2018, 31 (67.4%) patients had plantaris augmented reconstruction and 15 simple end-to-end suture. The clinician filled out the American Orthopaedic Foot and Ankle Society (AOFAS) hindfoot scale and the patient the Achilles tendon total rupture score (ATRS). Examination included clinical examination, limb symmetry index (LSI) using the heel rise test, and ultrasound determination of cross-sectional area (CSA). Results 80.4% were males, mean age 41 (SD 6.7, range 29-57), of which 82.6% were sports accidents. 39/46 were operated in less than two weeks from injury. 82.6% were available at the two year follow-up. Duration of surgery (62.3 min vs 58, p = 0.45), AOFAS (89.6 vs 88.4, p = 0.61 and 97.2 vs 96.8, p = 0.72), ATRS (86.3 vs 83.8, p = 0.33 and 95.6 vs 93.6, p = 0.12), LSI (60.8% vs 58.75, p = 0.24 and 80.5 vs 79, p = 0.29), CSA (3.39cm(2) vs 3.36, p = 0.82 and 2.57 vs 2.59, p = 0.87), return to sport (80% vs 57, p = 0.15 and 84.6 vs 85.7, p = 1.00), and complications at six months and two years were comparable between the two techniques. Conclusion Fanned out plantaris augmentation of acute Achilles tendon tears yields excellent and comparable clinical and ultrasonographic results to end-to-end suture at mid-term follow-up
C1 [Prejbeanu, Radu; Vermesan, Dinu; Mioc, Mihail Lazar; Haragus, Horia] Victor Babes Univ Med & Pharm, Dept Orthoped & Trauma, 2 Eftimie Murgu Sq, Timisoara 300041, Romania.
   [Balanescu, Andrei] Premiere Hosp, 113 Aradului Str, Timisoara 300643, Romania.
RP Mioc, ML (通讯作者)，Victor Babes Univ Med & Pharm, Dept Orthoped & Trauma, 2 Eftimie Murgu Sq, Timisoara 300041, Romania.
EM mihaillazarmioc@gmail.com
RI Haragus, Horia/Q-1800-2019; Haragus, Horia/ABD-8399-2021
OI Haragus, Horia/0000-0001-6801-3471; Mioc,
   Mihail-Lazar/0000-0002-9381-6871
CR Akgun Umut, 2006, Acta Orthop Traumatol Turc, V40, P228
   Aktas Seref, 2007, J Foot Ankle Surg, V46, P336, DOI 10.1053/j.jfas.2007.06.006
   Carmont MR, 2020, ORTHOP J SPORTS MED, V8, DOI 10.1177/2325967120909556
   Dederer KM, 2019, FOOT ANKLE CLIN, V24, P371, DOI 10.1016/j.fcl.2019.04.001
   Eliasson P, 2018, AM J SPORT MED, V46, P2492, DOI 10.1177/0363546518781826
   Ganestam A, 2016, KNEE SURG SPORT TR A, V24, P3730, DOI 10.1007/s00167-015-3544-5
   Hegewald KW, 2016, J FOOT ANKLE SURG, V55, P166, DOI 10.1053/j.jfas.2015.08.001
   Hiramatsu K, 2018, ORTHOP J SPORTS MED, V6, DOI 10.1177/2325967118789883
   Ilhami K, 2004, ARCH ORTHOP TRAUM SU, V124, P608, DOI 10.1007/s00402-003-0626-5
   Kearney RS, 2012, HEALTH QUAL LIFE OUT, V10, DOI 10.1186/1477-7525-10-24
   Lantto I, 2016, AM J SPORT MED, V44, P2406, DOI 10.1177/0363546516651060
   Lantto I, 2015, AM J SPORT MED, V43, P2302, DOI 10.1177/0363546515591267
   LYNN TA, 1966, J BONE JOINT SURG AM, VA 48, P268, DOI 10.2106/00004623-196648020-00005
   Moller M, 2002, KNEE SURG SPORT TR A, V10, P49, DOI 10.1007/s001670100245
   Nyyssonen T, 2003, ACTA ORTHOP SCAND, V74, P206, DOI 10.1080/00016470310013978
   Ochen Y, 2019, BMJ-BRIT MED J, V364, DOI 10.1136/bmj.k5120
   Park YH, 2019, KNEE SURG SPORT TR A, V27, P3297, DOI 10.1007/s00167-018-5172-3
   SCHEDL R, 1979, J TRAUMA, V19, P189, DOI 10.1097/00005373-197903000-00011
   Sconfienza LM, 2018, EUR RADIOL, V28, P5338, DOI 10.1007/s00330-018-5474-3
   Spang C, 2016, BONE JOINT J, V98B, P1312, DOI 10.1302/0301-620X.98B10.37939
   Svedman S, 2020, KNEE SURG SPORT TR A, V28, P3309, DOI 10.1007/s00167-020-05945-2
   Willits K, 2010, J BONE JOINT SURG AM, V92A, P2767, DOI 10.2106/JBJS.I.01401
   Zellers JA, 2020, KNEE SURG SPORT TR A, V28, P245, DOI 10.1007/s00167-019-05608-x
NR 23
TC 0
Z9 0
U1 0
U2 0
PU SPRINGER
PI NEW YORK
PA ONE NEW YORK PLAZA, SUITE 4600, NEW YORK, NY, UNITED STATES
SN 0341-2695
EI 1432-5195
J9 INT ORTHOP
JI Int. Orthop.
PD SEP
PY 2021
VL 45
IS 9
BP 2285
EP 2290
DI 10.1007/s00264-021-05169-0
EA AUG 2021
PG 6
WC Orthopedics
WE Science Citation Index Expanded (SCI-EXPANDED)
SC Orthopedics
GA WD2TA
UT WOS:000681190500002
PM 34350472
DA 2022-07-22
ER

PT J
AU Hansen, MS
   Christensen, M
   Budolfsen, T
   Ostergaard, TF
   Kallemose, T
   Troelsen, A
   Barfod, KW
AF Hansen, Maria Swennergren
   Christensen, Marianne
   Budolfsen, Thomas
   Ostergaard, Thomas Friis
   Kallemose, Thomas
   Troelsen, Anders
   Barfod, Kristoffer Weisskirchner
TI Achilles tendon Total Rupture Score at 3 months can predict patients'
   ability to return to sport 1 year after injury
SO KNEE SURGERY SPORTS TRAUMATOLOGY ARTHROSCOPY
LA English
DT Article
DE Achilles tendon rupture; Achilles tendon Total Rupture Score; Return to
   sport; Predictive value; Sex
ID RANDOMIZED CONTROLLED-TRIAL; NONOPERATIVE TREATMENT; WOMEN; RELIABILITY;
   EXERCISE; MEN
AB To investigate how the Achilles tendon Total Rupture Score (ATRS) at 3 months and 1 year after injury is associated with a patient's ability to return to work and sports as well as to investigate whether sex and age influence ATRS after 3 months and 1 year.
   This is a retrospective study analysing the data from the Danish Achilles tendon Database. A total of 366 patients were included. Logistic regression was conducted to describe the effect of ATRS on return to work and sports. The effect of age and sex on ATRS was analysed by linear regression.
   Three months after injury patients had a significantly increased chance of return to sport after 1 year with an increased ATRS (OR 1.06, p = 0.001) but a non-significant effect on return to work. After 1 year, patients had a significantly increased probability of having returned to sport (OR 1.11, p < 0.001) and also having returned to work (OR 1.05, p = 0.007) with an increased ATRS. Men had an average 7 (p = 0.006) points higher ATRS at 3 months and an average 22 (p = 0.006) points higher at 1 year.
   ATRS is associated with patients' ability to return to sports and work. ATRS at 3 months can be used as a predictor of the patient's ability to return to sports after 1 year. Hereby, ATRS might help to individualise rehabilitation by identifying patients who do not respond adequately to the chosen treatment.
   II.
C1 [Hansen, Maria Swennergren; Budolfsen, Thomas] Copenhagen Univ Hosp Hvidovre, Dept Physiotherapy & Occupat Therapy, Danish Achilles Tendon Database, Kettegard Alle 30, DK-2650 Hvidovre, Denmark.
   [Christensen, Marianne] Aalborg Univ Hosp, Dept Physiotherapy & Occupat Therapy, Danish Achilles Tendon Database, Hobrovej 18-22, DK-9000 Aalborg, Denmark.
   [Christensen, Marianne] Aalborg Univ Hosp, Dept Orthopaed Surg, Danish Achilles Tendon Database, Hobrovej 18-22, DK-9000 Aalborg, Denmark.
   [Ostergaard, Thomas Friis] Lillebaelt Hosp, Dept Orthopaed Surg, Danish Achilles Tendon Database, Skovvangen 2-8, DK-6000 Kolding, Denmark.
   [Kallemose, Thomas] Copenhagen Univ Hosp, Danish Achilles Tendon Database, Clin Res Ctr, Dept Orthopaed,Clin Orthopaed Res Hvidovre, Hvidovre, Denmark.
   [Troelsen, Anders; Barfod, Kristoffer Weisskirchner] Copenhagen Univ Hosp, Danish Achilles Tendon Database, Clin Orthopaed Res Hvidovre, Dept Orthopaed Surg, Hvidovre, Denmark.
RP Hansen, MS (通讯作者)，Copenhagen Univ Hosp Hvidovre, Dept Physiotherapy & Occupat Therapy, Danish Achilles Tendon Database, Kettegard Alle 30, DK-2650 Hvidovre, Denmark.
EM maria_swennergren@hotmail.com
RI Barfod, Kristoffer/AAY-3288-2021
OI Barfod, Kristoffer/0000-0003-2620-5891; Troelsen,
   Anders/0000-0003-0132-8182; Christensen, Marianne/0000-0003-3517-8151
CR Barfod KW, 2014, J BONE JOINT SURG AM, V96A, P1497, DOI 10.2106/JBJS.M.01273
   Bostick GP, 2010, J ORTHOP SPORT PHYS, V40, P345, DOI 10.2519/jospt.2010.3204
   Carmont MR, 2013, FOOT ANKLE SURG, V19, P245, DOI 10.1016/j.fas.2013.06.007
   Ganestam A, 2016, KNEE SURG SPORT TR A, V24, P3730, DOI 10.1007/s00167-015-3544-5
   Ganestam A, 2013, J FOOT ANKLE SURG, V52, P736, DOI 10.1053/j.jfas.2013.07.004
   Hosmer D.W., 2000, APPL LOGISTIC REGRES, V2nd ed., DOI [DOI 10.1002/0471722146, 10.1002/0471722146.ch8]
   Houshian S, 1998, INJURY, V29, P651, DOI 10.1016/S0020-1383(98)00147-8
   Hutchison AM, 2015, BONE JOINT J, V97B, P510, DOI 10.1302/0301-620X.97B4.35314
   Jiang N, 2012, INT ORTHOP, V36, P765, DOI 10.1007/s00264-011-1431-3
   Lantto I, 2015, SCAND J MED SCI SPOR, V25, pE133, DOI 10.1111/sms.12253
   Levi N, 1997, INJURY, V28, P311, DOI 10.1016/S0020-1383(96)00200-8
   Magnusson SP, 2007, INT J EXP PATHOL, V88, P237, DOI 10.1111/j.1365-2613.2007.00551.x
   Mark-Christensen T, 2016, KNEE SURG SPORT TR A, V24, P1852, DOI 10.1007/s00167-014-3180-5
   Metz R, 2008, AM J SPORT MED, V36, P1688, DOI 10.1177/0363546508319312
   Miller BF, 2007, J APPL PHYSIOL, V102, P541, DOI 10.1152/japplphysiol.00797.2006
   Ministry of Employment, 2008, AN SICK LEAV
   Moller A, 1996, ACTA ORTHOP SCAND, V67, P479, DOI 10.3109/17453679608996672
   Mortensen NHM, 1999, J BONE JOINT SURG AM, V81A, P983, DOI 10.2106/00004623-199907000-00011
   Nilsson-Helander K, 2007, AM J SPORT MED, V35, P421, DOI 10.1177/0363546506294856
   Nilsson-Helander K, 2010, AM J SPORT MED, V38, P2186, DOI 10.1177/0363546510376052
   Olsson N, 2014, SCAND J MED SCI SPOR, V24, P152, DOI 10.1111/j.1600-0838.2012.01497.x
   Olsson N, 2014, AM J SPORT MED, V42, P1448, DOI 10.1177/0363546514527409
   Olsson N, 2011, KNEE SURG SPORT TR A, V19, P1385, DOI 10.1007/s00167-011-1511-3
   Saxena A, 2011, J FOOT ANKLE SURG, V50, P37, DOI 10.1053/j.jfas.2010.10.008
   Silbernagel KG, 2012, AM J SPORT MED, V40, P1564, DOI 10.1177/0363546512447926
   Silbernagel KG, 2015, ORTHOP J SPORTS MED, V3, DOI 10.1177/2325967115586768
   Soroceanu A, 2012, J BONE JOINT SURG AM, V94A, P2136, DOI 10.2106/JBJS.K.00917
   Westh E, 2008, SCAND J MED SCI SPOR, V18, P23, DOI 10.1111/j.1600-0838.2007.00638.x
   Wilkins R, 2012, AM J SPORT MED, V40, P2154, DOI 10.1177/0363546512453293
   Willits K, 2010, J BONE JOINT SURG AM, V92A, P2767, DOI 10.2106/JBJS.I.01401
NR 30
TC 23
Z9 24
U1 0
U2 3
PU SPRINGER
PI NEW YORK
PA 233 SPRING ST, NEW YORK, NY 10013 USA
SN 0942-2056
EI 1433-7347
J9 KNEE SURG SPORT TR A
JI Knee Surg. Sports Traumatol. Arthrosc.
PD APR
PY 2016
VL 24
IS 4
BP 1365
EP 1371
DI 10.1007/s00167-015-3974-0
PG 7
WC Orthopedics; Sport Sciences; Surgery
WE Science Citation Index Expanded (SCI-EXPANDED)
SC Orthopedics; Sport Sciences; Surgery
GA DI7SX
UT WOS:000373703400056
PM 26733273
DA 2022-07-22
ER

PT J
AU Solakoglu, C
   Mahirogullari, M
   Cakmak, S
   Tamam, C
   Kuskucu, M
AF Solakoglu, Can
   Mahirogullari, Mahir
   Cakmak, Selami
   Tamam, Cuneyt
   Kuskucu, Mesih
TI Fibrin sealant in the treatment of acute ruptures of the Achilles tendon
   long-term results
SO EKLEM HASTALIKLARI VE CERRAHISI-JOINT DISEASES AND RELATED SURGERY
LA English
DT Article
DE Achilles tendon; fibrin tissue adhesive; rupture
ID FUNCTIONAL TREATMENT; GLUE; SUTURE; REPAIR
AB Objectives This study aims to point out the long-term results of fibrin sealant in the treatment of acute ruptures of the Achilles tendon
   Patients and methods Between January 1998 and December 2007, 42 patients (42 males, mean age 37 8 years range 27 to 56 years) admitted to our clinic for acute ruptures of the Achilles tendon were treated with a fibrin sealant The average time to surgery from injury was 2 0 (range 1 to 4) days The patients were divided into two groups, in the first 32 patients fibrin sealant was used which was heated for preparation In the second group of 10 patients we used fibrin sealant which did not require heating for preparation
   Results The mean follow-up period was 51 5 (range 24 to 92) months The Thermann scoring system was used to evaluate the patients postoperatively in both the early and the final follow-ups We found that the Thermann scores of some patients slightly decreased in the advanced age group when compared to younger patients long-term, but the overall result remained unchanged by time Only one patient needed to be re-operated for a re-rupture resulting from resumption of daily work and sports activities
   Conclusion Treatment of acute Achilles tendon ruptures with fibrin sealant can be one of the first choices selected because of its lower complication rate and equal results when compared to other treatment options
C1 [Solakoglu, Can; Mahirogullari, Mahir; Kuskucu, Mesih] GATA Haydarpasa Training Hosp, Dept Orthoped & Traumatol, Istanbul, Turkey.
   [Cakmak, Selami] Aksaz Mil Hosp, Dept Orthoped & Traumatol, Mugla, Turkey.
   [Tamam, Cuneyt] Kasimpasa Mil Hosp, Dept Orthoped & Traumatol, Istanbul, Turkey.
RP Solakoglu, C (通讯作者)，Haydarpasa Egitim Hastanesi, Ortopedi & Travmatol Klinigi, TR-34668 Istanbul, Turkey.
RI tamam, cüneyt/AAW-1543-2021; Mahirogullari, Mahir/AAA-4742-2020
CR ATIK OS, 1998, EKLEM HAST CERRAHISI, V9, P149
   BLUME M, 1987, Beitraege zur Orthopaedie und Traumatologie, V34, P309
   BOSCH P, 1981, ARCH ORTHOP TRAUM SU, V98, P305, DOI 10.1007/BF00378887
   CETTI R, 1993, AM J SPORT MED, V21, P791, DOI 10.1177/036354659302100606
   Clark RAF, 2000, EXPERT OPIN INV DRUG, V9, P2371, DOI 10.1517/13543784.9.10.2371
   HAAS F, 1987, ZBL CHIR, V112, P99
   HAIBOCK HF, 1986, IATROS ORTHOPADIE, V1, P17
   Hohendorff B, 2008, J FOOT ANKLE SURG, V47, P392, DOI 10.1053/j.jfas.2008.05.006
   Hohendorff B, 2009, J FOOT ANKLE SURG, V48, P439, DOI 10.1053/j.jfas.2009.04.005
   Khan RJK, 2005, J BONE JOINT SURG AM, V87A, P2202, DOI 10.2106/JBJS.D.03049
   Kuskucu M, 2005, FOOT ANKLE INT, V26, P826, DOI 10.1177/107110070502601007
   Lusardi D A, 1994, J Foot Ankle Surg, V33, P443
   Maffulli N, 1999, J BONE JOINT SURG AM, V81A, P1019, DOI 10.2106/00004623-199907000-00017
   Oktem H, 2010, EKLEM HAST CERRAHISI, V21, P50
   Pfluger H, 1986, FIBRIN SEALANT OPERA, P39
   Pneumaticos SG, 2000, FOOT ANKLE INT, V21, P551, DOI 10.1177/107110070002100704
   REDAELLI C, 1992, CHIRURG, V63, P572
   SCHLAG G, 1988, CLIN ORTHOP RELAT R, P269
   Thermann H, 2001, FOOT ANKLE INT, V22, P478, DOI 10.1177/107110070102200604
   Thermann H, 1995, UNFALLCHIRURG, V98, P507
   THERMANN H, 1995, UNFALLCHIRURG, V98, P21
   US K, 1996, EKLEM HAST CERRAHISI, V7, P37
   Wong J, 2002, AM J SPORT MED, V30, P565, DOI 10.1177/03635465020300041701
NR 23
TC 7
Z9 8
U1 0
U2 5
PU TURKISH JOINT DISEASES FOUNDATION
PI KAVAKLIDERE
PA BUGDAY SOKAK 6-27, KAVAKLIDERE, ANKARA 06700, TURKEY
SN 1305-8282
J9 EKLEM HAST CERRAHISI
JI Eklem Hast. Cerrahisi
PY 2010
VL 21
IS 3
BP 124
EP 129
PG 6
WC Orthopedics; Surgery
WE Science Citation Index Expanded (SCI-EXPANDED)
SC Orthopedics; Surgery
GA 697AI
UT WOS:000285483000002
PM 21067492
DA 2022-07-22
ER

PT J
AU Jennings, AG
   Sefton, GK
   Newman, RJ
AF Jennings, AG
   Sefton, GK
   Newman, RJ
TI Repair of acute rupture of the Achilles tendon: a new technique using
   polyester tape without external splintage
SO ANNALS OF THE ROYAL COLLEGE OF SURGEONS OF ENGLAND
LA English
DT Article
DE Achilles tendon rupture; surgical treatment
ID EARLY MOBILIZATION; CALCANEAL TENDON
AB Introduction: A new method of treating acute rupture of the Achilles tendon using polyester tape is reported. This requires no postoperative splintage, allows earlier mobilisation and prompt return to work and sport.
   Patients and Methods: Thirty patients were reviewed prospectively and at a mean of 3 years from surgery.
   Results: The average time of return to work, full weight-bearing and to driving was 40 days, 45 days and 49 days, respectively. The average time for return to sport was 122 days. Three patients required further surgery, two for infected wounds and one for scar release. There was one sural nerve injury. Twenty-two patients regained a normal range of ankle and subtalar movement, with the mean power of plantar flexion 84% of the opposite side. Of the 22 patients who played sport, 14 were still performing at the same or a higher level. There were no re-ruptures over this period.
   Conclusions: The technique is straightforward and avoids splintage. It also conveys other short-and long-term advantages over more established methods.
C1 Harrogate Dist Hosp, Dept Orthopaed Surg, Harrogate HG2 7SX, England.
RP Sefton, GK (通讯作者)，Harrogate Dist Hosp, Dept Orthopaed Surg, Lancaster Pk Rd, Harrogate HG2 7SX, England.
CR CANALE ST, 1998, CAMPBELLS OPERATIVE, P1414
   CARDEN DG, 1987, J BONE JOINT SURG BR, V69, P416, DOI 10.1302/0301-620X.69B3.3294839
   LEVY M, 1984, CLIN ORTHOP RELAT R, P199
   McComis GP, 1997, J BONE JOINT SURG AM, V79A, P1799, DOI 10.2106/00004623-199712000-00005
   NISTOR L, 1981, J BONE JOINT SURG AM, V63, P394, DOI 10.2106/00004623-198163030-00012
   SALEH M, 1992, J BONE JOINT SURG BR, V74, P206, DOI 10.1302/0301-620X.74B2.1544953
   SAW Y, 1993, INJURY, V24, P479, DOI 10.1016/0020-1383(93)90155-Y
   Webb JM, 1999, J BONE JOINT SURG BR, V81B, P877, DOI 10.1302/0301-620X.81B5.9784
   1973, LANCET, V1, P189
   1989, LANCET, V1, P1427
NR 10
TC 12
Z9 12
U1 0
U2 2
PU ROYAL COLL SURGEONS ENGLAND
PI LONDON
PA 35-43 LINCOLN'S INN FIELDS, LONDON WC2A 3PN, ENGLAND
SN 0035-8843
J9 ANN ROY COLL SURG
JI Ann. R. Coll. Surg. Engl.
PD NOV
PY 2004
VL 86
IS 6
BP 445
EP 448
DI 10.1308/147870804957
PG 4
WC Surgery
WE Science Citation Index Expanded (SCI-EXPANDED)
SC Surgery
GA 870NL
UT WOS:000225064600013
PM 15527585
OA Green Published
DA 2022-07-22
ER

PT J
AU Coull, R
   Flavin, R
   Stephens, MM
AF Coull, R
   Flavin, R
   Stephens, MM
TI Flexor hallucis longus tendon transfer: Evaluation of postoperative
   morbidity
SO FOOT & ANKLE INTERNATIONAL
LA English
DT Article
DE achilles tendon; pressure; tendon injuries; tendon transfer
ID ACHILLES-TENDON; RUPTURE; ANKLE; FOOT; TOES
AB Clinical and pedobarograph evaluation was performed on 16 patients following flexor hallucis longus (FHL) tendon transfers to determine the resulting morbidity due to the loss of FHL function. All patients underwent FHL tendon transfer for either chronic tendon Achilles rupture or chronic Achilles tendinosis. Clinical evaluation of hallux function was performed using the American Orthopaedic Foot and Ankle Society (AOFAS) hallux metatarsophalangeal-interphalangeaI scale, the SF-36 score, and a clinical questionnaire to assess alteration in the clinical function of the hallux during activities of daily living. Pedobarography was carried out using the Musgrave pedobarograph system to detect changes in forefoot loading in comparison to the contralateral normal foot. Fourteen of the 16 patients scored maximally on the hallux metatarsophalangeal-interphalangeaI scale and none of the patients noticed functional weakness of the hallux during activities of daily living at a mean follow-up of 43.6 months (range, 5-120 months). Pedobarograph readings showed a trend toward reduction in peak pressure loading on the distal phalanx, but this was not significant for the numbers of patients studied. There was no significant increase in loading of the first or second metatarsophalangeal joints to suggest that transfer metatarsalgia may complicate FHL tendon transfer. According to the results of the study morbidity from FHL transfer should be clinically insignificant.
C1 Cappagh Natl Orthopaed Hosp, Dublin, Ireland.
   Mater Misericordiae Hosp, Childrens Univ Hosp, Cent Remedial Clin, Cappagh Natl Orthopaed Hosp, Dublin 7, Ireland.
RP Stephens, MM (通讯作者)，Mater Private Hosp, Eccles St,Suite 8, Dublin 7, Ireland.
CR BORTON DC, 1994, J BONE JOINT SURG BR, V76B, P204, DOI 10.1302/0301-620X.76B2.8113277
   FRENETTE JP, 1977, J BONE JOINT SURG AM, V59, P673, DOI 10.2106/00004623-197759050-00015
   GABEL S, 1994, FOOT ANKLE INT, V15, P512, DOI 10.1177/107110079401500912
   Hansen Jr ST, 1991, DISORDERS FOOT ANKLE, V3, P2355
   HOLMES GB, 1991, FOOT ANKLE, V12, P105, DOI 10.1177/107110079101200208
   HUGHES J, 1990, J BONE JOINT SURG BR, V72, P245, DOI 10.1302/0301-620X.72B2.2312564
   Hughes J, 1993, Acta Orthop Belg, V59, P10
   KITAOKA HB, 1994, FOOT ANKLE INT, V15, P349, DOI 10.1177/107110079401500701
   Monroe MT, 2000, FOOT ANKLE INT, V21, P324, DOI 10.1177/107110070002100409
   O'Toole GC, 2003, FOOT ANKLE INT, V24, P256, DOI 10.1177/107110070302400310
   Rosenbaum D., 1997, FOOT ANKLE SURG, V3, P1, DOI [10.1046/J.1460-9584.1997.00043.X, DOI 10.1046/J.1460-9584.1997.00043.X, 10.1046/j.1460-9584.1997.00043.x.]
   SERRAFIAN SK, 1993, ANATOMY FOOT ANKLE, P240
   SILVER RL, 1985, J BONE JOINT SURG BR, V67, P432, DOI 10.1302/0301-620X.67B3.3997956
   VAUGHAN CL, 1992, DYNAMICS HUMAN GAIT, P107
   Wapner K L, 1993, Foot Ankle, V14, P443
   Wilcox DK, 2000, FOOT ANKLE INT, V21, P1004, DOI 10.1177/107110070002101204
NR 16
TC 89
Z9 95
U1 0
U2 3
PU AMER ORTHOPAEDIC FOOT & ANKLE SOC, INC
PI SEATTLE
PA 2517 EASTLAKE AVE EAST, STE 200, SEATTLE, WA 98102 USA
SN 1071-1007
J9 FOOT ANKLE INT
JI Foot Ankle Int.
PD DEC
PY 2003
VL 24
IS 12
BP 931
EP 934
DI 10.1177/107110070302401211
PG 4
WC Orthopedics
WE Science Citation Index Expanded (SCI-EXPANDED)
SC Orthopedics
GA 758XK
UT WOS:000187676900011
PM 14733350
DA 2022-07-22
ER

PT J
AU Jupiter, DC
   Fang, X
   Ashmore, Z
   Shibuya, N
   Mehta, HB
AF Jupiter, Daniel C.
   Fang, Xiao
   Ashmore, Zachary
   Shibuya, Naohiro
   Mehta, Hemalkumar B.
TI The Relative Risk of Achilles Tendon Injury in Patients Taking
   Quinolones
SO PHARMACOTHERAPY
LA English
DT Article
DE quinolones; Achilles tendon injury; propensity score; adverse events
ID FLUOROQUINOLONE-ASSOCIATED TENDINOPATHY; TRANSPLANT PATIENTS; DISORDERS;
   LEVOFLOXACIN; RUPTURE; SAFETY; CIPROFLOXACIN; ASSOCIATION; POPULATION;
   DATABASE
AB ObjectiveTo examine the association between quinolone use and Achilles tendon injury, comparing well-matched cohorts of users of quinolone and nonquinolone antibiotics, and well-matched cohorts of quinolone users and patients not using any nonquinolone antibiotics.
   Patients and MethodsThis retrospective cohort study used Clinformatics data from 2008-2014. Using the propensity score, we matched quinolone users with other antibiotic users and quinolone users with nonusers. The primary outcome was Achilles tendon injury within 6months. Bivariate analyses determined risk factors for Achilles tendon injury, and conditional logistic regression assessed the impact of quinolone use on these injuries.
   ResultsA total of 716,522 fluoroquinolone users were matched with other antibiotic users, and 645,034 fluoroquinolone users were matched with nonusers. Rates of Achilles tendon injury were less than 0.5% in all groups. Quinolone use increased the risk of Achilles tendon injury compared with other antibiotic users (odds ratio [OR] 1.24, 95% confidence interval [CI] 1.17-1.31) and nonusers (OR 1.54, 95% CI 1.44-1.64). Interaction with age did not significantly impact the relationship between quinolone use and Achilles injury; however, older quinolone users had a slightly higher relative risk of injury than nonusers versus younger patients. Furthermore, the youngest group of patients had similarly elevated relative risk for injury with quinolone use as did the elderly.
   ConclusionAlthough quinolone use increases the risk of Achilles tendon injury, the absolute risk increase is minimal, especially when compared with similar morbidity patients taking other nonquinolone antibiotics. In relatively healthy populations, such as the one studied here, quinolone use may not make a clinically significant contribution to risk of Achilles tendon injury, at any age range, among those in need of such drugs.
C1 [Jupiter, Daniel C.; Fang, Xiao] Univ Texas Med Branch, Dept Prevent Med & Community Hlth, 301 Univ Blvd, Galveston, TX 77555 USA.
   [Ashmore, Zachary] Univ Texas Med Branch, Sch Med, Galveston, TX 77555 USA.
   [Shibuya, Naohiro] Texas A&M Univ, Hlth Sci Ctr, Dept Surg, Coll Med, Round Rock, TX USA.
   [Shibuya, Naohiro] Cent Texas Vet Affairs Hlth Care Syst, Dept Surg, Sect Podiatry, Temple, TX USA.
   [Shibuya, Naohiro] Baylor Scott & White Hlth, Dept Surg, Temple, TX USA.
   [Mehta, Hemalkumar B.] Univ Texas Med Branch, Dept Surg, Galveston, TX 77555 USA.
RP Jupiter, DC (通讯作者)，Univ Texas Med Branch, Dept Prevent Med & Community Hlth, 301 Univ Blvd, Galveston, TX 77555 USA.
EM dajupite@utmb.edu
CR Abramowicz M, 2016, JAMA-J AM MED ASSOC, V316, P1404, DOI 10.1001/jama.2016.8383
   Austin PC, 2011, MULTIVAR BEHAV RES, V46, P119, DOI 10.1080/00273171.2011.540480
   Barge-Cabatlero E, 2008, J HEART LUNG TRANSPL, V27, P46, DOI 10.1016/j.healun.2007.09.021
   Bidell MR, 2016, PHARMACOTHERAPY, V36, P679, DOI 10.1002/phar.1761
   Chhajed PN, 2002, EUR RESPIR J, V19, P469, DOI 10.1183/09031936.02.00257202
   Claessen FMAP, 2014, SPORTS MED, V44, P1241, DOI 10.1007/s40279-014-0200-z
   Corrao G, 2006, DRUG SAFETY, V29, P889, DOI 10.2165/00002018-200629100-00006
   Daneman N, 2015, BMJ OPEN, V5, DOI 10.1136/bmjopen-2015-010077
   Food and Drug Administration, FDA DRUG SAF COMM FD
   Hori K, 2012, PHARMACOEPIDEM DR S, V21, P886, DOI 10.1002/pds.3285
   Khaliq Y, 2003, CLIN INFECT DIS, V36, P1404, DOI 10.1086/375078
   Lang TR, 2017, FUND CLIN PHARMACOL, V31, P4, DOI 10.1111/fcp.12228
   Leone R, 2003, DRUG SAFETY, V26, P109, DOI 10.2165/00002018-200326020-00004
   Mehta HB, 2017, SUCCESS ACAD SURG, P47, DOI 10.1007/978-3-319-43952-5_5
   Parson LS, 2004, P 29 ANN SAS US GROU
   Pierfitte C, 1996, THERAPIE, V51, P419
   Pugi A, 2013, AM J HEALTH-SYST PH, V70, P1039, DOI 10.2146/ajhp120665
   Seeger JD, 2006, PHARMACOEPIDEM DR S, V15, P784, DOI 10.1002/pds.1214
   Sode J, 2007, EUR J CLIN PHARMACOL, V63, P499, DOI 10.1007/s00228-007-0265-9
   Sohn M, 2015, PHARMACOEPIDEM DR S, V24, P583, DOI 10.1002/pds.3768
   Stephenson AL, 2013, DRUG SAFETY, V36, P709, DOI 10.1007/s40264-013-0089-8
   TANNE JH, 2008, BRIT MED J, V337
   Torre-Cisneros J, 2015, CLIN INFECT DIS, V60, P1642, DOI 10.1093/cid/civ156
   van der Linden PD, 1999, BRIT J CLIN PHARMACO, V48, P433
   van der Linden PD, 2003, ARCH INTERN MED, V163, P1801, DOI 10.1001/archinte.163.15.1801
   van der Linden PD, 2002, BRIT MED J, V324, P1306, DOI 10.1136/bmj.324.7349.1306
   Van der Linden PD, 2001, ARTHRIT RHEUM-ARTHR, V45, P235, DOI 10.1002/1529-0131(200106)45:3<235::AID-ART254>3.0.CO;2-7
   van der Linden PD, 2001, PHARM WORLD SCI, V23, P89, DOI 10.1023/A:1011254030271
   Wilton LV, 1996, BRIT J CLIN PHARMACO, V41, P277, DOI 10.1046/j.1365-2125.1996.03013.x
   Wise BL, 2012, AM J MED, V125, DOI 10.1016/j.amjmed.2012.05.027
   Yamaguchi H, 2007, CHEMOTHERAPY, V53, P85, DOI 10.1159/000099032
NR 31
TC 6
Z9 6
U1 0
U2 7
PU WILEY
PI HOBOKEN
PA 111 RIVER ST, HOBOKEN 07030-5774, NJ USA
SN 0277-0008
EI 1875-9114
J9 PHARMACOTHERAPY
JI Pharmacotherapy
PD SEP
PY 2018
VL 38
IS 9
BP 878
EP 887
DI 10.1002/phar.2162
PG 10
WC Pharmacology & Pharmacy
WE Science Citation Index Expanded (SCI-EXPANDED)
SC Pharmacology & Pharmacy
GA GS9WV
UT WOS:000444079700001
PM 29972705
DA 2022-07-22
ER

PT J
AU Hsu, AR
   Jones, CP
   Cohen, BE
   Davis, WH
   Ellington, JK
   Anderson, RB
AF Hsu, Andrew R.
   Jones, Carroll P.
   Cohen, Bruce E.
   Davis, W. Hodges
   Ellington, J. Kent
   Anderson, Robert B.
TI Clinical Outcomes and Complications of Percutaneous Achilles Repair
   System Versus Open Technique for Acute Achilles Tendon Ruptures
SO FOOT & ANKLE INTERNATIONAL
LA English
DT Article
DE Achilles tendon; rupture; treatment; limited incision repair; PARS;
   open; complications
ID LIMITED OPEN REPAIR; INJURIES
AB Background: Limited incision techniques for acute Achilles tendon ruptures have been developed in recent years to improve recovery and reduce postoperative complications compared with traditional open repair. The purpose of this retrospective cohort study was to analyze the clinical outcomes and postoperative complications between acute Achilles tendon ruptures treated using a percutaneous Achilles repair system (PARS [Arthrex, Inc, Naples, FL]) versus open repair and evaluate the overall outcomes for operatively treated Achilles ruptures.
   Methods: Between 2005 and 2014, 270 consecutive cases of operatively treated acute Achilles tendon ruptures were reviewed (101 PARS, 169 open). Patients with Achilles tendinopathy, insertional ruptures, chronic tears, or less than 3-month follow-up were excluded. Operative treatment consisted of a percutaneous technique (PARS) using a 2-cm transverse incision with FiberWire (Arthrex, Inc, Naples, FL) sutures or open repair using a 5- to 8-cm posteromedial incision with FiberWire in a Krackow fashion reinforced with absorbable sutures. Patient demographics were recorded along with medical comorbidities, activity at injury, time from injury to surgery, length of follow-up, return to baseline activities by 5 months, and postoperative complications.
   Results: The most common activity during injury for both groups was basketball (PARS: 39%, open: 47%). A greater number of patients treated with PARS were able to return to baseline physical activities by 5 months compared with the open group (PARS: 98%, open: 82%; P = .0001). There were no significant differences (P > .05) between groups in rates of rerupture (P = 1.0), sural neuritis (P = .16), wound dehiscence (P = .74), superficial (P = .29) and/or deep infection (P = .29), or reoperation (P = .13). There were no deep vein thromboses (DVTs) or reruptures in either group. In the PARS group, there were no cases of sural neuritis, 3 cases (3%) of superficial wound dehiscence, and 2 reoperations (2%) for superficial foreign-body reaction to FiberWire. In the open group, there were 5 cases (3%) of sural neuritis, 7 cases (4%) of superficial wound dehiscence, 3 cases (2%) of superficial infection, and 3 reoperations (2%) for deep infection.
   Conclusion: The present study reports the largest single-center series of acute Achilles tendon ruptures in the literature with lower complication rates for operatively treated Achilles ruptures compared with previous reports. The overall complication rate for all operatively treated Achilles ruptures was 8.5% with no reruptures, and most patients (88%) were able to return to baseline activities by 5 months after surgery. There were no significant differences in rates of postoperative complications between PARS and open repair for acute Achilles tendon ruptures.
   Level of Evidence: Level III, retrospective cohort study.
C1 [Hsu, Andrew R.; Jones, Carroll P.; Cohen, Bruce E.; Davis, W. Hodges; Ellington, J. Kent; Anderson, Robert B.] OrthoCarolina Foot & Ankle Inst, Charlotte, NC 28207 USA.
RP Hsu, AR (通讯作者)，OrthoCarolina Foot & Ankle Inst, 2001 Vail Ave,Suite 200B, Charlotte, NC 28207 USA.
EM andyhsu1@gmail.com
FU Arthrex, Inc.
FX The authors declared the following potential conflicts of interest with
   respect to the research, authorship, and/or publication of this article:
   Carroll P. Jones, MD, Bruce E. Cohen, MD, W. Hodges Davis, MD, and
   Robert B. Anderson, MD, receive royalties and are paid consultants for
   Arthrex, Inc, the manufacturer of the percutaneous Achilles repair
   system (PARS) discussed in the present article. J. Kent Ellington, MD,
   is also a paid consultant for Arthrex, Inc.
CR Assal M, 2002, J BONE JOINT SURG AM, V84A, P161, DOI 10.2106/00004623-200202000-00001
   Calder JDF, 2005, BRIT J SPORT MED, V39, P857, DOI 10.1136/bjsm.2004.017509
   Chiodo CP, 2010, J BONE JOINT SURG AM, V92A, P2466
   Chiodo CP, 2010, J AM ACAD ORTHOP SUR, V18, P503, DOI 10.5435/00124635-201008000-00007
   Cretnik A, 2005, AM J SPORT MED, V33, P1369, DOI 10.1177/0363546504271501
   Demetracopoulos CA, 2014, FOOT ANKLE INT, V35, P612, DOI 10.1177/1071100714524550
   Healy B, 2010, J BONE JOINT SURG BR, V92B, P646, DOI 10.1302/0301-620X.92B5.23241
   Holmes GB, 2006, FOOT ANKLE INT, V27, P952, DOI 10.1177/107110070602701115
   Hrnack SA, 2012, FOOT ANKLE INT, V33, P848, DOI 10.3113/FAI.2012.0848
   Jones Matthew P, 2012, J Bone Joint Surg Am, V94, pe88, DOI 10.2106/JBJS.J.01829
   Keller A, 2014, AM J SPORT MED, V42, P731, DOI 10.1177/0363546513511418
   Khan RJK, 2010, COCHRANE DB SYST REV, DOI 10.1002/14651858.CD003674.pub4
   KRACKOW KA, 1988, ORTHOPEDICS, V11, P909, DOI 10.3928/0147-7447-19880601-11
   Lansdaal JR, 2007, INJURY, V38, P839, DOI 10.1016/j.injury.2006.12.010
   Lim J, 2001, FOOT ANKLE INT, V22, P559, DOI 10.1177/107110070102200705
   Maffulli N, 2011, FOOT ANKLE INT, V32, P9, DOI 10.3113/FAI.2011.0009
   Majewski M, 2006, AM J SPORT MED, V34, P793, DOI 10.1177/0363546505283266
   McMahon Samuel E, 2011, Foot Ankle Surg, V17, P211, DOI 10.1016/j.fas.2010.11.001
   Metz R, 2008, AM J SPORT MED, V36, P1688, DOI 10.1177/0363546508319312
   Metz R, 2011, AM J SPORT MED, V39, P820, DOI 10.1177/0363546510392012
   Nilsson-Helander K, 2010, AM J SPORT MED, V38, P2186, DOI 10.1177/0363546510376052
   Nilsson-Helander K, 2009, KNEE SURG SPORT TR A, V17, P1234, DOI 10.1007/s00167-009-0727-y
   Park HG, 2001, FOOT ANKLE INT, V22, P985, DOI 10.1177/107110070102201211
   Raikin SM, 2013, FOOT ANKLE INT, V34, P475, DOI 10.1177/1071100713477621
   Suchak AA, 2005, FOOT ANKLE INT, V26, P932, DOI 10.1177/107110070502601106
   Tejwani Nirmal C, 2014, Am J Orthop (Belle Mead NJ), V43, pE221
NR 26
TC 79
Z9 86
U1 0
U2 12
PU SAGE PUBLICATIONS INC
PI THOUSAND OAKS
PA 2455 TELLER RD, THOUSAND OAKS, CA 91320 USA
SN 1071-1007
EI 1944-7876
J9 FOOT ANKLE INT
JI Foot Ankle Int.
PD NOV
PY 2015
VL 36
IS 11
BP 1279
EP 1286
DI 10.1177/1071100715589632
PG 8
WC Orthopedics
WE Science Citation Index Expanded (SCI-EXPANDED)
SC Orthopedics
GA CV3XZ
UT WOS:000364201300004
PM 26055259
DA 2022-07-22
ER

PT J
AU Lim, CS
   Lees, D
   Gwynne-Jones, DP
AF Lim, Che Siu
   Lees, David
   Gwynne-Jones, David P.
TI Functional Outcome of Acute Achilles Tendon Rupture With and Without
   Operative Treatment Using Identical Functional Bracing Protocol
SO FOOT & ANKLE INTERNATIONAL
LA English
DT Article
DE ATRS; Achilles tendon rupture treatment; functional bracing
ID RANDOMIZED CONTROLLED-TRIAL; NONOPERATIVE TREATMENT; SURGICAL REPAIR;
   ACCELERATED REHABILITATION; NONSURGICAL TREATMENT; CAST IMMOBILIZATION;
   FOLLOW-UP; MANAGEMENT; VALIDITY; SCORE
AB Background: The purpose of this study was to compare the functional results of operative and nonoperative treatment of acute Achilles tendon rupture using an identical rehabilitation program of functional bracing.
   Methods: Over a 10-year period, 200 patients (99 operative, 101 nonoperative) aged between 18 and 65 years were treated at our institution's physiotherapy department after acute Achilles tendon rupture. There were 132 patients (62 operative, 70 nonoperative) available for a minimum 2-year follow-up (average 6.5 years; range, 2-13 years). Functional outcome was assessed using the Achilles tendon total rupture score (ATRS).
   Results: With the numbers available, no significant difference could be detected in ATRS between operative (mean 84.8, median 90) and nonoperative groups (mean 85.3, median 91; P = 0.55). No significant difference could be detected in ATRS between male and female patients however treated (P = 0.30) or between patients younger and older than 40 years at time of injury (P = 0.68). There was no correlation between ATRS score and age at injury in all patients ( = -0.0168, P = 0.85). In male patients, there was a weak trend with older patients at follow-up having better scores ( = 0.21, P = 0.069). However, among female patients, there was a significant negative correlation between ATRS scores and increasing age ( = -0.29, P = 0.03). Logistic regression analysis failed to show any significant effect of age at rupture, gender, or mode of treatment on ATRS.
   Conclusions: This study showed no significant difference detectable in ATRS between operative and nonoperative patients in the treatment of acute Achilles tendon ruptures using an identical rehabilitation program with functional bracing.
   Level of Evidence: Level II, prospective comparative study.
C1 [Lim, Che Siu] Whangarei Hosp, Orthopaed Outpatients, Maunu Rd,Private Bag 9742, Whangarei 0148, New Zealand.
   [Lees, David] Waikato Dist Hlth Board, Hamilton, New Zealand.
   [Gwynne-Jones, David P.] Univ Otago, Dunedin Sch Med, Dept Surg Sci, Dunedin, New Zealand.
   [Gwynne-Jones, David P.] Southern Dist Hlth Board, Dunedin Hosp, Dept Orthopaed Surg, Dunedin, New Zealand.
RP Lim, CS (通讯作者)，Whangarei Hosp, Orthopaed Outpatients, Maunu Rd,Private Bag 9742, Whangarei 0148, New Zealand.
EM limchesiu@yahoo.com
RI Jones, David Gwynne-/AAJ-7059-2020
OI Jones, David Gwynne-/0000-0001-8484-614X
CR Barfod KW, 2014, J BONE JOINT SURG AM, V96A, P1497, DOI 10.2106/JBJS.M.01273
   Bergkvist D, 2012, J BONE JOINT SURG AM, V94A, P1229, DOI 10.2106/JBJS.J.01601
   Carmont MR, 2013, KNEE SURG SPORT TR A, V21, P1356, DOI 10.1007/s00167-012-2146-8
   Ganestam A, 2013, J FOOT ANKLE SURG, V52, P736, DOI 10.1053/j.jfas.2013.07.004
   Gross CE, 2016, FOOT ANKLE INT, V37, P233, DOI 10.1177/1071100715619606
   Gwynne-Jones DP, 2011, FOOT ANKLE INT, V32, P337, DOI 10.3113/FAI.2011.0337
   Hufner TM, 2006, FOOT ANKLE INT, V27, P167, DOI 10.1177/107110070602700302
   Hutchison AM, 2015, BONE JOINT J, V97B, P510, DOI 10.1302/0301-620X.97B4.35314
   Jones Matthew P, 2012, J Bone Joint Surg Am, V94, pe88, DOI 10.2106/JBJS.J.01829
   JOZSA L, 1989, AM J SPORT MED, V17, P338, DOI 10.1177/036354658901700305
   Kearney RS, 2012, HEALTH QUAL LIFE OUT, V10, DOI 10.1186/1477-7525-10-24
   Kearney RS, BR J SPORTS MED, V2011, P1102
   Keating JF, 2011, J BONE JOINT SURG BR, V93B, P1071, DOI 10.1302/0301-620X.93B8.25998
   Khan RJ, 2010, COCHRANE DB SYST REV, V9
   Lantto I, 2015, AM J SPORT MED, V43, P2302, DOI 10.1177/0363546515591267
   Maffulli N, 2011, FOOT ANKLE INT, V32, P9, DOI 10.3113/FAI.2011.0009
   McCormack R, 2015, BRIT J SPORT MED, V49, P1329, DOI 10.1136/bjsports-2015-094935
   Metz R, 2008, AM J SPORT MED, V36, P1688, DOI 10.1177/0363546508319312
   Moller A, 1996, ACTA ORTHOP SCAND, V67, P479, DOI 10.3109/17453679608996672
   Moller M, 2001, J BONE JOINT SURG BR, V83B, P843
   Nilsson-Helander K, 2007, AM J SPORT MED, V35, P421, DOI 10.1177/0363546506294856
   Nilsson-Helander K, 2010, AM J SPORT MED, V38, P2186, DOI 10.1177/0363546510376052
   Olsson N, 2013, AM J SPORT MED, V41, P2867, DOI 10.1177/0363546513503282
   Olsson N, 2011, KNEE SURG SPORT TR A, V19, P1385, DOI 10.1007/s00167-011-1511-3
   Porter MD, 2015, ANZ J SURG, V85, P373, DOI 10.1111/ans.12910
   Rosso C, 2015, KNEE SURG SPORT TR A, V23, P890, DOI 10.1007/s00167-013-2726-2
   Schepull T, 2013, AM J SPORT MED, V41, P2550, DOI 10.1177/0363546513501785
   Silbernagel KG, 2015, ORTHOP J SPORTS MED, V3, DOI 10.1177/2325967115586768
   Soroceanu A, 2012, J BONE JOINT SURG AM, V94A, P2136, DOI 10.2106/JBJS.K.00917
   Sorrenti SJ, 2006, FOOT ANKLE INT, V27, P407, DOI 10.1177/107110070602700603
   Uquillas CA, 2015, J BONE JOINT SURG AM, V97A, P1187, DOI 10.2106/JBJS.O.00002
   Wilkins R, 2012, AM J SPORT MED, V40, P2154, DOI 10.1177/0363546512453293
   Willits K, 2010, J BONE JOINT SURG AM, V92A, P2767, DOI 10.2106/JBJS.I.01401
NR 33
TC 14
Z9 14
U1 0
U2 7
PU SAGE PUBLICATIONS INC
PI THOUSAND OAKS
PA 2455 TELLER RD, THOUSAND OAKS, CA 91320 USA
SN 1071-1007
EI 1944-7876
J9 FOOT ANKLE INT
JI Foot Ankle Int.
PD DEC
PY 2017
VL 38
IS 12
BP 1331
EP 1336
DI 10.1177/1071100717728687
PG 6
WC Orthopedics
WE Science Citation Index Expanded (SCI-EXPANDED)
SC Orthopedics
GA FN8JA
UT WOS:000416268700006
PM 28891323
DA 2022-07-22
ER

PT J
AU Grubor, P
   Grubor, M
AF Grubor, Predrag
   Grubor, Milan
TI Treatment of Achilles tendon rupture using different methods
SO VOJNOSANITETSKI PREGLED
LA English
DT Article
DE achilles tendon; rupture; orthopedic procedures; treatment outcome
ID PERCUTANEOUS REPAIR; MANAGEMENT
AB Background/Aim. Today there are controversies about searching for the ideal surgical method (conservatively with plaster cast, with open and percutaneous tenorrhaphy) for repairing a ruptured Achilles tendon. The aim of this study study was to examine the results of treating Achilles tendon ruptures in patients by using the following methods: percutaneous suturing, open surgery technique and non-surgical treatment by plaster cast immobilisation. Methods. Forty two patients treated at our facility in the period August 2003 - September 2010 for Achilles tendon ruptures were included in the study. They were operated on by using different orthopedic procedures (percutaneous reconstruction of the Achilles tendon, open surgery, plaster cast only) and two anaesthesia technique (spinal aneasthesia and local infiltrational anaesthesia). The following parameters were monitored after interventions performed and compared: duration of hospital stay, postsurgical complications, incidence of the reruptures of the Achilles tendon and time for full leg functionality. Results. The patients sustained their respective injuries in the following manner: 8 of them while pursuing sports activities, 24 while pursuing recreational activities, 4 at workplace, 4 while performing everyday activities, and 2 of the patients did not know how they had sustained their injuries. The average age of the patients was 40.5, with 37 (88%) men and 5 (12%) women. Surgeries were performed under spinal anaesthesia in 29 (69%) patients, and in 5 (12%) patients tenorrhaphy was performed under local anaesthesia. Anaesthesia was not used in 8 (19%) patients treated with plaster cast. We performed percutaneous reconstruction of the Achilles tendon in 19 (45%) patients. A total of 14 (33%) patients were treated under spinal anaesthesia, and 5 (11.9%) under local infiltrational anaesthesia with 2% xylocain. We treated 15 (36%) patients with open surgery. The patients treated conservatively stayed in hospital on average for up to 5 hours. Those who underwent an percutaneous surgery stayed 2 days and those who underwent an open surgery stayed 9 days. A total of 28 (66%) patients from the given series experienced no complications. The patients treated with open surgical reconstruction experienced skin complications ranging from inflammatory changes on the skin in 6 (14%) patients to dehiscence and skin necrosis in 3 (7%). The 5 (11.9%) patients whose ruptured Achilles tendon was treated percutaneously experienced temporary redness and delayed healing of the incision(s) longer than 5 mm. A total of 3 (7%) patients treated with open surgery and 1 (2%) patient treated with percutaneous tenorrhaphy had temporary peroneal nerve prolapses. A total of 7 (16.6%) patients had reruptures: 4 were treated with plaster cast, 2 underwent open surgery, and 1 was treated percutaneously. Out of the 8 patients who were treated with plaster cast, 4 sustained reruptures and 3 of the 4 had diabetes. Conclusion. Surgical treatment, percutaneous tenorrhaphy, performed in a small operating theatre under local anaesthesia, should be preferred in cases of fresh ruptures of the Achilles tendon.
C1 [Grubor, Predrag] Univ Clin Ctr Banja Luka, Traumatol Clin, Banja Luka 78000, Republika Srpsk, Bosnia & Herceg.
   [Grubor, Milan] Univ Banja Luka, Fac Med, Banja Luka, Bosnia & Herceg.
RP Grubor, P (通讯作者)，Univ Clin Ctr Banja Luka, Traumatol Clin, Zdravka Kodre 1, Banja Luka 78000, Republika Srpsk, Bosnia & Herceg.
EM predraggrubor@gmail.com
RI Grubor, Predrag/AAE-2329-2021; Grubor, Predrag/Y-9975-2018
OI Grubor, Predrag/0000-0001-6826-1790; Grubor, Predrag/0000-0001-6826-1790
CR Amer O, 1959, ACTA CHIR SCAND, V239, P1
   Banovic D, 1998, TRAUMATOLOGY BONES A
   Burry HC, 1973, RHEUMATOL REHABIL, V12, P177, DOI DOI 10.1093/RHEUMATOLOGY/12.4.177
   Cretnik Andrej, 2004, J Foot Ankle Surg, V43, P72, DOI 10.1053/j.jfas.2004.01.008
   Gorschewsky O, 2004, FOOT ANKLE INT, V25, P219, DOI 10.1177/107110070402500405
   Haji A, 2004, FOOT ANKLE INT, V25, P215, DOI 10.1177/107110070402500404
   Jennings AG, 2002, J BONE JOINT SURG BR, V84B, P361, DOI 10.1302/0301-620X.84B3.11559
   Josey RA, 2003, FOOT ANKLE INT, V24, P775, DOI 10.1177/107110070302401007
   Leppilahti J, 1998, CLIN ORTHOP RELAT R, P152
   MA GWC, 1977, CLIN ORTHOP RELAT R, P247
   Matasovic T, 1988, ULTRASOUND DIAGNOSTI
   Myerson M, 1991, CURRENT THERAPY FOOT, P123
   NISTOR L, 1981, J BONE JOINT SURG AM, V63, P394, DOI 10.2106/00004623-198163030-00012
   Rostan Alain, 2003, Rev Med Suisse Romande, V123, P365
   THOMPSON T C, 1962, J Trauma, V2, P126, DOI 10.1097/00005373-196203000-00003
   Tomak Sanda L, 2004, Am J Orthop (Belle Mead NJ), V33, P9
   Wallace RGH, 2004, J BONE JOINT SURG AM, V86A, P1198, DOI 10.2106/00004623-200406000-00011
   Weber M, 2003, AM J SPORT MED, V31, P685, DOI 10.1177/03635465030310050901
NR 18
TC 9
Z9 14
U1 0
U2 11
PU MILITARY MEDICAL ACAD-INI
PI BELGRADE
PA CRNOTRAVSKA 17, PO BOX 33-35, BELGRADE, 11040, SERBIA
SN 0042-8450
EI 2406-0720
J9 VOJNOSANIT PREGL
JI Vojnosanit. Pregl.
PD AUG
PY 2012
VL 69
IS 8
BP 663
EP 668
DI 10.2298/VSP1208663G
PG 6
WC Medicine, General & Internal
WE Science Citation Index Expanded (SCI-EXPANDED)
SC General & Internal Medicine
GA 990FX
UT WOS:000307617500003
PM 22924261
OA Green Submitted, gold
DA 2022-07-22
ER

PT J
AU Nilsson-Helander, K
   Sward, L
   Silbernagel, KG
   Thomee, R
   Eriksson, BI
   Karlsson, J
AF Nilsson-Helander, Katarina
   Sward, Leif
   Silbernagel, Karin Gravare
   Thomee, Roland
   Eriksson, Bengt I.
   Karlsson, Jon
TI A new surgical method to treat chronic ruptures and reruptures of the
   Achilles tendon
SO KNEE SURGERY SPORTS TRAUMATOLOGY ARTHROSCOPY
LA English
DT Article
DE achilles tendon; rerupture; chronic rupture; free flap; augmentation
ID PERONEUS BREVIS TENDON; NEGLECTED RUPTURE; CALCANEAL TENDON; REPAIR;
   RECONSTRUCTION; FLAP; AUGMENTATION; DEFECTS; TISSUE; TEARS
AB In patients with a chronic rupture or rerupture of the Achilles tendon, the recommended treatment is surgical. Various surgical techniques have been reported in the literature; however, the outcome is rarely evaluated with a sufficiently long follow-up, using appropriate end-points. The purpose of this study was to evaluate the subjective and objective outcome following a new surgical treatment for chronic rupture or rerupture of the Achilles tendon using augmentation with a free gastrocnemius aponeurosis flap. A total of 28 consecutive patients (22 male and 6 female) with a mean (SD) age of 46 (10.4) years were evaluated at a median (range) of 29 (12-117) months after surgery. The surgical technique involved making a single incision and then using a free gastrocnemius aponeurosis flap to cover the tendon gap after an end-to-end suture. The patients were evaluated using the Achilles tendon rupture score (ATRS) and a detailed questionnaire relating to symptoms, physical activity and satisfaction with treatment. The functional evaluation consisted of a validated test battery measuring different aspects of muscle/tendon function of the gastrocnemius/soleus and Achilles tendon complex. The median (range) ATRS was 83 (24-100). There were no reruptures. In terms of surgical complications, there was one deep infection, three wound closure complications and deep venous thrombosis in two patients. All but one patient returned to work within 6 months of surgery. Sixteen (57%) patients were satisfied with the treatment. There was a significant decrease in the level of physical activity after the injury compared with before the injury (p = 0.004). Of the 25 patients who participated in recreational sports prior to injury, 13 (52%) returned to the same activity level after treatment. In terms of jump performance, no significant differences were found between the healthy and injured sides. There was, however, a significant decrease in strength, in terms of both concentric and eccentric-concentric toe raises and the toe-raise test for endurance compared with the healthy side. The use of a free gastrocnemius aponeurosis flap to treat chronic ruptures and reruptures of the Achilles tendon rendered a good overall subjective and objective outcome in the majority of patients. The use of a single incision in combination with a free flap augmentation produced favourable results.
C1 [Nilsson-Helander, Katarina] Univ Gothenburg, Dept Orthopaed, Kungsbacka Hosp, S-43440 Kungsbacka, Sweden.
   [Sward, Leif; Eriksson, Bengt I.; Karlsson, Jon] Univ Gothenburg, Sahlgrens Univ Hosp, Dept Orthopaed, Gothenburg, Sweden.
   [Silbernagel, Karin Gravare; Thomee, Roland] Univ Gothenburg, Sahlgrens Univ Hosp, Lundberg Lab Orthopaed Res, Gothenburg, Sweden.
RP Nilsson-Helander, K (通讯作者)，Univ Gothenburg, Dept Orthopaed, Kungsbacka Hosp, S-43440 Kungsbacka, Sweden.
EM Ina.nilsson@telia.com
RI Helander, Katarina Nilsson/AAE-1793-2020
OI Helander, Katarina Nilsson/0000-0002-1292-5102; Gravare Silbernagel,
   Karin/0000-0001-7566-407X
CR ABRAHAM E, 1975, J BONE JOINT SURG AM, VA 57, P253, DOI 10.2106/00004623-197557020-00019
   ARNER O, 1959, Acta Chir Scand Suppl, V116, P1
   BARNES MJ, 1986, J BONE JOINT SURG BR, V68, P121, DOI 10.1302/0301-620X.68B1.3941128
   BOSWORTH DM, 1956, J BONE JOINT SURG AM, V38, P111, DOI 10.2106/00004623-195638010-00011
   CARDEN DG, 1987, J BONE JOINT SURG BR, V69, P416, DOI 10.1302/0301-620X.69B3.3294839
   Cetti R, 2003, ACTA ORTHOP SCAND, V74, P78, DOI 10.1080/00016470310013707
   CHRISTENSEN I, 1953, ACTA CHIR SCAND, V106, P50
   GABEL S, 1994, FOOT ANKLE INT, V15, P512, DOI 10.1177/107110079401500912
   GERDES MH, 1992, CLIN ORTHOP RELAT R, P241
   Haas F, 2003, ANN PLAS SURG, V51, P376, DOI 10.1097/01.sap.0000068080.76814.D7
   INGLIS AE, 1976, J BONE JOINT SURG AM, V58, P990, DOI 10.2106/00004623-197658070-00015
   Kangas J, 2007, AM J SPORT MED, V35, P59, DOI 10.1177/0363546506293255
   Khan RJK, 2005, J BONE JOINT SURG AM, V87A, P2202, DOI 10.2106/JBJS.D.03049
   Kissel C G, 1994, J Foot Ankle Surg, V33, P46
   KOMI PV, 1992, CLIN SPORT MED, V11, P521
   Lapidus LJ, 2007, J ORTHOP TRAUMA, V21, P52, DOI 10.1097/01.bot.0000250741.65003.14
   Lerch K, 2003, ORTHOPADE, V32, P816, DOI 10.1007/s00132-003-0470-x
   Leslie Hamish D H, 2005, Foot Ankle Clin, V10, P357, DOI 10.1016/j.fcl.2005.01.009
   Maffulli N, 2005, CLIN J SPORT MED, V15, P56, DOI 10.1097/01.jsm.0000152714.05097.ef
   Moller M, 2001, J BONE JOINT SURG BR, V83B, P843
   Nilsson-Helander K, 2007, AM J SPORT MED, V35, P421, DOI 10.1177/0363546506294856
   OZAKI J, 1989, CLIN ORTHOP RELAT R, P204
   Pajala A, 2002, J BONE JOINT SURG AM, V84A, P2016, DOI 10.2106/00004623-200211000-00017
   PEREZTEUFFER A, 1974, ORTHOP CLIN N AM, V5, P89
   Pintore E, 2001, J TRAUMA, V50, P71, DOI 10.1097/00005373-200101000-00013
   Porter DA, 1997, FOOT ANKLE INT, V18, P557, DOI 10.1177/107110079701800905
   SALTIN B, 1968, CIRCULATION, V38, P1104, DOI 10.1161/01.CIR.38.6.1104
   Silbernagel KG, 2006, KNEE SURG SPORT TR A, V14, P1207, DOI 10.1007/s00167-006-0150-6
   SILFVERSKIOLD N, 1940, ACTA CHIR SCAND, V84, P393
   Takao M, 2003, ARCH ORTHOP TRAUM SU, V123, P471, DOI 10.1007/s00402-002-0443-2
   TURCO VJ, 1987, FOOT ANKLE, V7, P253, DOI 10.1177/107110078700700407
   WAPNER KL, 1995, ORTHOP CLIN N AM, V26, P249
   WHITE RK, 1959, SURG GYNECOL OBSTET, V108, P117
NR 33
TC 49
Z9 53
U1 0
U2 1
PU SPRINGER
PI NEW YORK
PA 233 SPRING ST, NEW YORK, NY 10013 USA
SN 0942-2056
EI 1433-7347
J9 KNEE SURG SPORT TR A
JI Knee Surg. Sports Traumatol. Arthrosc.
PD JUN
PY 2008
VL 16
IS 6
BP 614
EP 620
DI 10.1007/s00167-008-0492-3
PG 7
WC Orthopedics; Sport Sciences; Surgery
WE Science Citation Index Expanded (SCI-EXPANDED)
SC Orthopedics; Sport Sciences; Surgery
GA 307MC
UT WOS:000256322000014
PM 18273601
DA 2022-07-22
ER

PT J
AU Stalman, A
   Bring, D
   Ackermann, PW
AF Stalman, A.
   Bring, D.
   Ackermann, P. W.
TI Chemokine expression of CCL2, CCL3, CCL5 and CXCL10 during early
   inflammatory tendon healing precedes nerve regeneration: an
   immunohistochemical study in the rat
SO KNEE SURGERY SPORTS TRAUMATOLOGY ARTHROSCOPY
LA English
DT Article
DE Chemokines; Achilles tendon; Neuropeptides; Tendon rupture; Tendon
   repair; Nerve regeneration; Inflammation; Angiogenesis
ID ACHILLES-TENDON; WOUND REPAIR; SUBSTANCE-P; ANGIOGENESIS; INJURY;
   CHEMOATTRACTANT; MIP-1-ALPHA; PLASTICITY; INDUCTION; RANTES
AB Chemokines are major promoters of repair and may regulate nerve ingrowth that is essential in tendon healing. The purpose of this study was to assess the temporal occurrence of different chemokines during Achilles tendon healing in relation to sensory nerve regeneration. Chemokine presence in tendon healing has not been studied previously.
   Chemokine expression, nerve regeneration, angiogenesis and inflammatory cell occurrence during healing of Achilles tendon rupture in the rat were studied by immunohistochemistry and histology including semiquantitative assessment. Markers for chemokines (CCL5, CCL2, CCL3, CXCL10), nerves (PGP-9.5) and sensory neuropeptide substance P (SP) were analysed at different time points (1 day-16 weeks) post-rupture.
   In intact tendons (controls) immunoreactivity to all chemokines, PGP-9.5 and SP were confined to the tendon surroundings. After rupture, there was rapid increase in the tendon proper of the chemokines studied, all exhibiting their peak expression at week 1. Subsequently, at weeks 2-6, emerging inflammatory cells and maximum sprouting of PGP-/SP-positive nerves were observed close to newly formed blood vessels within the tendon proper, while chemokine expression already decreased. During weeks 6-8, PGP-/SP-positive nerves withdrew from the rupture site and relocated together with the chemokines in the surrounding tendon.
   Early chemokine expression in the healing tendon precedes ingrowth of new nerves, angiogenesis and emergence of inflammatory cells. The fine-tuned temporal and spatial appearance of chemokines suggests a chemoattractant role for inflammatory cell migration and possibly also a role in angiogenesis and neurogenesis. Chemokines may thus exhibit vital targets for biological modulation of tendon repair.
   III.
C1 [Stalman, A.] Karolinska Inst, Dept Clin Sci Intervent & Technol CLINTEC, Stockholm, Sweden.
   [Stalman, A.] Aleris Sabbatsberg Hosp, Stockholm, Sweden.
   [Bring, D.; Ackermann, P. W.] Karolinska Inst, Dept Mol Med & Surg, Integrat Orthoped Lab, Stockholm, Sweden.
   [Bring, D.; Ackermann, P. W.] Karolinska Univ Hosp, Dept Orthoped, Stockholm, Sweden.
RP Stalman, A (通讯作者)，Karolinska Inst, Dept Clin Sci Intervent & Technol CLINTEC, Stockholm, Sweden.
EM anders.stalman@ki.se
RI Stalman, Anders/AAE-5611-2019; Stålman, Anders/AAX-8051-2020; W,
   Ackermann P/B-9714-2008
OI Stålman, Anders/0000-0002-6261-2024; W, Ackermann P/0000-0002-5520-169X
FU Stockholm County Council [SLL20100168]; Karolinska Institutet
   [SLL20100168]; Swedish National Centre for Sports Research; Swedish
   Research Council [2012-3510]
FX This study was supported by the regional agreement on medical training
   and clinical research (ALF) between Stockholm County Council and
   Karolinska Institutet (project nr. SLL20100168) and the Swedish National
   Centre for Sports Research as well as the Swedish Research Council
   (project nr. 2012-3510).
CR Ackermann PW, 2013, KNEE SURG SPORT TR A, V21, P1801, DOI 10.1007/s00167-012-2197-x
   Ackermann PW, 2013, INT J EXP PATHOL, V94, P271, DOI 10.1111/iep.12028
   Ackermann PW, 2009, FRONT BIOSCI-LANDMRK, V14, P5165, DOI 10.2741/3593
   Ackermann PW, 2003, J ORTHOP RES, V21, P432, DOI 10.1016/S0736-0266(02)00207-3
   Ackermann PW, 2002, J ORTHOP RES, V20, P849, DOI 10.1016/S0736-0266(01)00159-0
   ANGIOLILLO AL, 1995, J EXP MED, V182, P155, DOI 10.1084/jem.182.1.155
   Baggiolini M, 1997, ANNU REV IMMUNOL, V15, P675, DOI 10.1146/annurev.immunol.15.1.675
   Behm B, 2012, J EUR ACAD DERMATOL, V26, P812, DOI 10.1111/j.1468-3083.2011.04415.x
   Bring DKI, 2005, J ORTHOP RES, V23, P294, DOI 10.1016/j.orthres.2004.08.017
   Burssens P, 2005, FOOT ANKLE INT, V26, P832, DOI 10.1177/107110070502601008
   Carlsson O, 2011, SCAND J MED SCI SPOR, V21, P562, DOI 10.1111/j.1600-0838.2009.01080.x
   Charo IF, 2006, NEW ENGL J MED, V354, P610, DOI 10.1056/NEJMra052723
   DiPietro LA, 1998, J CLIN INVEST, V101, P1693, DOI 10.1172/JCI1020
   DVORAK HF, 1995, AM J PATHOL, V146, P1029
   Frank S, 2000, BIOCHEM J, V347, P265, DOI 10.1042/0264-6021:3470265
   Glaser J, 2006, J NEUROSCI RES, V84, P724, DOI 10.1002/jnr.20982
   Kalehua AN, 2004, EXP CELL RES, V297, P197, DOI 10.1016/j.yexcr.2004.02.031
   Low QEH, 2001, AM J PATHOL, V159, P457, DOI 10.1016/S0002-9440(10)61717-8
   Mantovani A, 2013, J PATHOL, V229, P176, DOI 10.1002/path.4133
   Nicoletti M, 2012, INT J IMMUNOPATH PH, V25, P849, DOI 10.1177/039463201202500403
   Olsson N, 2011, KNEE SURG SPORT TR A, V19, P1385, DOI 10.1007/s00167-011-1511-3
   Sharma P., 2006, Journal of Musculoskeletal & Neuronal Interactions, V6, P181
   Sharma P, 2005, J BONE JOINT SURG AM, V87A, P187, DOI 10.2106/JBJS.D.01850
   Shiraha H, 1999, J CELL BIOL, V146, P243
   Suffee N, 2012, ANGIOGENESIS, V15, P727, DOI 10.1007/s10456-012-9285-x
   Suffee N, 2011, BIOCHEM SOC T, V39, P1649, DOI 10.1042/BST20110651
   Taskinen HS, 2000, J PERIPHER NERV SYST, V5, P75, DOI 10.1046/j.1529-8027.2000.00009.x
   Tonnesen MG, 2000, J INVEST DERM SYMP P, V5, P40, DOI 10.1046/j.1087-0024.2000.00014.x
   Wetzler C, 2000, J INVEST DERMATOL, V115, P245, DOI 10.1046/j.1523-1747.2000.00029.x
NR 29
TC 16
Z9 16
U1 0
U2 5
PU SPRINGER
PI NEW YORK
PA 233 SPRING ST, NEW YORK, NY 10013 USA
SN 0942-2056
EI 1433-7347
J9 KNEE SURG SPORT TR A
JI Knee Surg. Sports Traumatol. Arthrosc.
PD SEP
PY 2015
VL 23
IS 9
BP 2682
EP 2689
DI 10.1007/s00167-014-3010-9
PG 8
WC Orthopedics; Sport Sciences; Surgery
WE Science Citation Index Expanded (SCI-EXPANDED)
SC Orthopedics; Sport Sciences; Surgery
GA CP6ND
UT WOS:000360003300035
PM 24809505
DA 2022-07-22
ER

PT J
AU Hrnack, SA
   Crates, JM
   Barber, FA
AF Hrnack, Scott A.
   Crates, John M.
   Barber, F. Alan
TI Primary Achilles Tendon Repair with Mini-Dorsolateral Incision Technique
   and Accelerated Rehabilitation
SO FOOT & ANKLE INTERNATIONAL
LA English
DT Article
DE Achilles Tendon Rupture; Mini-Dorsolateral Incision; Accelerated
   Rehabilitation; Ankle; Surgical Repair
ID SURGICAL REPAIR; SURAL NERVE; RUPTURE; ANATOMY
AB Background: No consensus exists for the best primary repair of acute Achilles tendon ruptures. Problems with wound healing and nerve damage can occur. Prolonged immobilization leads to stiffness and calf atrophy. This study assesses the clinical outcome of acute Achilles tendon repairs using a mini-dorsolateral incision followed by a rapid rehabilitation program. Materials: A consecutive series of acute Achilles tendon ruptures repaired using a mini-dorsolateral incision were reviewed with a minimum 12 months follow up. Fifteen patients with an average age of 44 (range, 32 to 60) years were followed an average of 45 (range, 14 to 72) months. Two modified, buried core high strength sutures were placed in each torn end of the Achilles tendon reinforced with a running circumferential whip-stitch. Ankle Hindfoot scores, single toe raises, calf circumference, and adverse events were recorded. An accelerated postoperative rehabilitation protocol was followed. Results: Postoperative AOFAS Ankle Hindfoot scores averaged 98.3 [39 pain; 49.6 function; 9.3 alignment]. All patients could single heel raise. Eight of 15 demonstrated atrophy with an average calf circumference loss of 1.0 cm. The only postoperative complication was one case of superficial cellulitis successfully treated with oral antibiotics. There were no sural nerve injuries, wound break down, or re-ruptures at final followup. Conclusion: The repair of acute Achilles tendon ruptures through a minimal lateral incision provided excellent functional outcomes, avoided complications including sural nerve injury, and allowed a return to sports between 4 to 6 months.
C1 [Crates, John M.; Barber, F. Alan] Plano Orthoped Sports Med & Spine Ctr, Plano, TX 75093 USA.
   [Hrnack, Scott A.] Act Orthopaed & Sports Med, Decatur, TX USA.
RP Barber, FA (通讯作者)，Plano Orthoped Sports Med & Spine Ctr, 5228 W Plano Pkwy, Plano, TX 75093 USA.
CR Citak M, 2007, BRIT J SPORT MED, V41, P456, DOI 10.1136/bjsm.2006.031328
   Fortis AP, 2008, ARTHROSCOPY, V24, P683, DOI 10.1016/j.arthro.2008.02.018
   Hohendorff B, 2008, J FOOT ANKLE SURG, V47, P392, DOI 10.1053/j.jfas.2008.05.006
   Hufner TM, 2006, FOOT ANKLE INT, V27, P167, DOI 10.1177/107110070602700302
   Kangas J, 2007, AM J SPORT MED, V35, P59, DOI 10.1177/0363546506293255
   Khan RJK, 2005, J BONE JOINT SURG AM, V87A, P2202, DOI 10.2106/JBJS.D.03049
   KITAOKA HB, 1994, FOOT ANKLE INT, V15, P349, DOI 10.1177/107110079401500701
   Kosanovic M, 2008, ARCH ORTHOP TRAUM SU, V128, P211, DOI 10.1007/s00402-007-0514-5
   Pajala A, 2002, J BONE JOINT SURG AM, V84A, P2016, DOI 10.2106/00004623-200211000-00017
   Pajala A, 2009, J BONE JOINT SURG AM, V91A, P1092, DOI 10.2106/JBJS.G.01089
   Pearsall AW, 2006, FOOT ANKLE INT, V27, P146, DOI 10.1177/107110070602700215
   Sorrenti SJ, 2006, FOOT ANKLE INT, V27, P407, DOI 10.1177/107110070602700603
   Suchak AA, 2008, J BONE JOINT SURG AM, V90A, P1876, DOI 10.2106/JBJS.G.01242
   TROOP RL, 1995, FOOT ANKLE INT, V16, P705, DOI 10.1177/107110079501601106
   Webb J, 2000, FOOT ANKLE INT, V21, P475, DOI 10.1177/107110070002100604
NR 15
TC 10
Z9 12
U1 0
U2 5
PU SAGE PUBLICATIONS INC
PI THOUSAND OAKS
PA 2455 TELLER RD, THOUSAND OAKS, CA 91320 USA
SN 1071-1007
EI 1944-7876
J9 FOOT ANKLE INT
JI Foot Ankle Int.
PD OCT
PY 2012
VL 33
IS 10
BP 848
EP 851
DI 10.3113/FAI.2012.0848
PG 4
WC Orthopedics
WE Science Citation Index Expanded (SCI-EXPANDED)
SC Orthopedics
GA 015ZL
UT WOS:000309486800007
PM 23050708
DA 2022-07-22
ER

PT J
AU Osarumwense, D
   Wright, J
   Gardner, K
   James, L
AF Osarumwense, Donald
   Wright, Jonathan
   Gardner, Kikachukwu
   James, Laurence
TI Conservative treatment for acute Achilles tendon rupture: survey of
   current practice
SO JOURNAL OF ORTHOPAEDIC SURGERY
LA English
DT Article
DE Achilles tendon; disease management; questionnaires; tendon injuries
ID NON-SURGICAL TREATMENT; NONOPERATIVE TREATMENT; IMMOBILIZATION;
   MANAGEMENT
AB Purpose. To survey the practice of orthopaedic consultants in the Greater London area for treating Achilles tendon ruptures.
   Methods. 221 orthopaedic consultants working in 28 hospitals within the Greater London area were identified. A questionnaire regarding conservative treatment for acute Achilles tendon ruptures was sent. The choice of immobilisation, the period of immobilisation, the time to weight bearing, the use of heel raises, and the use of diagnostic ultrasonography were enquired about.
   Results. 62 of 86 respondents treated Achilles tendon ruptures conservatively by below-knee casts (n=51), above-knee casts (n=5), or functional braces (n=6). The most common immobilisation regimen (n=7) was to keep the foot in a sequence of an equinus position, a semi-equinus position, and a neutral position (3 weeks in each position). After cast removal, 45 of respondents preferred to use a heel raise for a median duration of 4 (range, 2-36) weeks. Respectively for foot and ankle specialists (n=24) and other orthopaedic specialists (n=38), the median immobilisation period prescribed was 8 (range, 3-13) and 9 (range, 6-36) weeks, respectively (p=0.625), whereas the median time to weight bearing prescribed was 6 (range, 0-9) and 6 (range, 0-12) weeks, respectively (p=0.402).
   Conclusion. Functional bracing was not as widely used as below-knee cast immobilisation. There was no consensus on the optimal immobilisation regimen.
C1 [Osarumwense, Donald; Wright, Jonathan; Gardner, Kikachukwu; James, Laurence] Univ Hosp Lewisham, London SE13 6LH, England.
RP Osarumwense, D (通讯作者)，Univ Hosp Lewisham, London SE13 6LH, England.
EM donaldosaru@btinternet.com
OI Wright, Jonathan/0000-0001-6055-648X
CR CETTI R, 1993, AM J SPORT MED, V21, P791, DOI 10.1177/036354659302100606
   Eames MHA, 1997, INJURY, V28, P289, DOI 10.1016/S0020-1383(96)00203-3
   FRUENSGAARD S, 1992, INT ORTHOP, V16, P33
   Goodwin D W, 2000, Foot Ankle Clin, V5, P135
   Hufner TM, 2006, FOOT ANKLE INT, V27, P167, DOI 10.1177/107110070602700302
   INGLIS AE, 1976, J BONE JOINT SURG AM, V58, P990, DOI 10.2106/00004623-197658070-00015
   Kangas J, 2003, J TRAUMA, V54, P1171, DOI 10.1097/01.TA.0000047945.20863.A2
   Keating JF, 2011, J BONE JOINT SURG BR, V93B, P1071, DOI 10.1302/0301-620X.93B8.25998
   KELLER J, 1984, ACTA ORTHOP SCAND, V55, P548, DOI 10.3109/17453678408992957
   LEA RB, 1972, J BONE JOINT SURG AM, VA 54, P1398, DOI 10.2106/00004623-197254070-00002
   LILDHOLDT T, 1976, ACTA ORTHOP SCAND, V47, P454
   Maffulli N, 1998, AM J SPORT MED, V26, P266, DOI 10.1177/03635465980260021801
   Maffulli N, 1999, CLIN J SPORT MED, V9, P157, DOI 10.1097/00042752-199907000-00007
   McComis GP, 1997, J BONE JOINT SURG AM, V79A, P1799, DOI 10.2106/00004623-199712000-00005
   PERSSON A, 1979, INT ORTHOP, V3, P149
   Petersen Oleg Funck, 2002, Ugeskr Laeger, V164, P3852
   SALEH M, 1992, J BONE JOINT SURG BR, V74, P206, DOI 10.1302/0301-620X.74B2.1544953
   Sekiya JK, 1999, AM J SPORT MED, V27, P632, DOI 10.1177/03635465990270051501
   Worth N, 2007, J Orthop Surg (Hong Kong), V15, P311
NR 19
TC 13
Z9 13
U1 0
U2 4
PU HONG KONG ACAD MEDICINE PRESS
PI HONG KONG
PA 9/F/, ROOM 901, 99 WONG CHUK HANG RD, ABERDEEN, HONG KONG, 00000,
   PEOPLES R CHINA
SN 1022-5536
EI 2309-4990
J9 J ORTHOP SURG-HONG K
JI J. Orthop. Surg.
PY 2013
VL 21
IS 1
BP 44
EP 46
DI 10.1177/230949901302100112
PG 3
WC Orthopedics; Surgery
WE Science Citation Index Expanded (SCI-EXPANDED)
SC Orthopedics; Surgery
GA AX0AG
UT WOS:000346615500012
PM 23629986
OA gold
DA 2022-07-22
ER

PT J
AU Schafer, FP
   Sander, V
   Pothmann, CEM
   Allemann, F
   Simmen, HP
   Pape, HC
AF Schaefer, Frank P.
   Sander, Victor
   Pothmann, Carina E. M.
   Allemann, Florin
   Simmen, Hans-Peter
   Pape, Hans-Christoph
TI Anterior Rectus Sheath Autograft in WRAP-Augmentation of Achilles Tendon
   Rupture
SO JOURNAL OF FOOT & ANKLE SURGERY
LA English
DT Article
DE Achilles tendon; anterior rectus sheath; augmentation; autologous tissue
   rupture
ID NONSURGICAL TREATMENT; REPAIR
AB Achilles tendon ruptures can be counted as the most common traumatic ankle injuries. As such, there is a comparatively large set of treatment options including surgical and nonsurgical approaches. The purpose of this case report is to demonstrate a new technique for a specific subgroup of Achilles tendon ruptures that present with a large tendinous gap. We used a 2-step procedure designed to grant additional stability through an autograft from the anterior rectus sheath of the patient. Two patients were treated after suffering traumatic Achilles tendon ruptures on the left side with a gap of >3.5 cm and a high demand in daily activities. The reconstruction was performed using an upper quadrant recuts sheath as a WRAP-augmentation. After securing the transplant tissue, the abdominal wall was reconstructed using a Vicryl (TM)-Prolene (TM) mesh (VYPRO (R), Johnson & Johnson Medical GmbH, Ethicon Deutschland, Norderstedt, Germany). After, a standard approach to the Achilles tendon was performed with a Kirchmayr-Kessler suture. The end result was then stabilized with a rectus sheath WRAP over a length of 14 to 15 cm. On the cases reported here, multiple clinical follow-ups were performed over a 5-year period. We can report highly satisfying results, with a return to sports activity after 6 months and no complications. As such we believe the rectus sheath autograft an effective solution for Achilles tendon ruptures with large gaps in healthy patients that demonstrate a high demand in daily activities. (C) 2018 The Author(s). This is an open access article under the CC BY-NC-ND license. (http://creativecommons.org/licenses/by-nc-nd/4.0/)
C1 [Schaefer, Frank P.; Sander, Victor; Pothmann, Carina E. M.; Allemann, Florin; Simmen, Hans-Peter; Pape, Hans-Christoph] Univ Hosp Zurich, Dept Trauma Surg, Zurich, Switzerland.
   [Simmen, Hans-Peter; Pape, Hans-Christoph] Univ Hosp Zurich, Dept Trauma Surg, Surg, Zurich, Switzerland.
RP Schafer, FP (通讯作者)，Univ Hosp Zurich, Ramistr 100, CH-8049 Zurich, Switzerland.
EM frankpeter.schaefer@usz.ch
RI Allemann, Florin/AAV-3854-2020
OI Simmen, Hans-Peter/0000-0002-5201-4549; Pape,
   Hans-Christoph/0000-0002-2059-4980
CR Aurora A, 2007, J SHOULDER ELB SURG, V16, p171S, DOI 10.1016/j.jse.2007.03.008
   Chen JM, 2009, EXPERT REV MED DEVIC, V6, P61, DOI 10.1586/17434440.6.1.61
   Coons DA, 2006, SPORTS MED ARTHROSC, V14, P185, DOI 10.1097/00132585-200609000-00011
   Craatz S, 2006, BJU INT, V97, P134, DOI 10.1111/j.1464-410X.2006.05876.x
   Heikkinen J, 2017, AM J SPORT MED, V45, P1395, DOI 10.1177/0363546517694610
   Jones Matthew P, 2012, J Bone Joint Surg Am, V94, pe88, DOI 10.2106/JBJS.J.01829
   KELLAM JF, 1985, CLIN ORTHOP RELAT R, P80
   Lantto I, 2016, AM J SPORT MED, V44, P2406, DOI 10.1177/0363546516651060
   Lepow Gary M, 2006, J Foot Ankle Surg, V45, P351, DOI 10.1053/j.jfas.2006.06.004
   Longo UG, 2010, BRIT MED BULL, V94, P165, DOI 10.1093/bmb/ldp051
   Moller M, 2001, J BONE JOINT SURG BR, V83B, P843
   Olsson N, 2013, AM J SPORT MED, V41, P2867, DOI 10.1177/0363546513503282
   Pagenstert G, 2010, ORTHOPADE, V39, P1135, DOI 10.1007/s00132-010-1691-4
   Peterson KS, 2014, J FOOT ANKLE SURG, V53, P664, DOI 10.1053/j.jfas.2013.10.001
   Salgado CJ, 2000, PLAST RECONSTR SURG, V106, P321, DOI 10.1097/00006534-200008000-00013
   Thermann H, 2000, ORTHOPADE, V29, P235
   Trofa DP, 2017, AM J SPORT MED, V45, P2864, DOI 10.1177/0363546517713001
   Ulmar B, 2014, UNFALLCHIRURG, V117, P921, DOI 10.1007/s00113-014-2627-y
   WILLS CA, 1986, CLIN ORTHOP RELAT R, P156
   Wong J, 2002, AM J SPORT MED, V30, P565, DOI 10.1177/03635465020300041701
   Yuen JC, 2001, PLAST RECONSTR SURG, V107, P1807, DOI 10.1097/00006534-200106000-00028
   2010, ACTAS UROL ESP, V34, P564, DOI DOI 10.1016/J.ACURO.2009.12.015
NR 22
TC 0
Z9 0
U1 0
U2 1
PU ELSEVIER SCIENCE INC
PI NEW YORK
PA STE 800, 230 PARK AVE, NEW YORK, NY 10169 USA
SN 1067-2516
EI 1542-2224
J9 J FOOT ANKLE SURG
JI J. Foot Ankle Surg.
PD MAY
PY 2019
VL 58
IS 3
BP 562
EP 566
DI 10.1053/j.jfas.2018.09.021
PG 5
WC Orthopedics; Surgery
WE Science Citation Index Expanded (SCI-EXPANDED)
SC Orthopedics; Surgery
GA HW1LN
UT WOS:000466444500029
PM 30683517
OA Green Accepted, hybrid
DA 2022-07-22
ER

PT J
AU Praxitelous, P
   Edman, G
   Ackermann, PW
AF Praxitelous, P.
   Edman, G.
   Ackermann, P. W.
TI Microcirculation after Achilles tendon rupture correlates with
   functional and patient-reported outcomes
SO SCANDINAVIAN JOURNAL OF MEDICINE & SCIENCE IN SPORTS
LA English
DT Article
DE Achilles tendon; blood flow; circulation; healing; laser-Doppler
   flowmetry; microcirculation; rupture; tendon; treatment outcome
ID DEEP-VEIN THROMBOSIS; REACTIVE HYPEREMIA; TENDINOPATHY; COMPRESSION;
   ELONGATION; ENDURANCE; DEFICITS; ABILITY; MUSCLE; MODEL
AB Patients with acute Achilles tendon rupture (ATR) display an extended healing process with varying clinical outcome. Poor microcirculatory blood flow has been suggested to be a significant factor for the healing process. However, whether microcirculation may predict healing outcome has been mostly unknown. Therefore, we investigated whether blood flow in the Achilles tendon may be associated with patient-reported and functional outcomes after ATR. In vivo laser-Doppler flowmetry was used to assess microvascular blood flow bilateral in the Achilles tendons, during post-occlusive reactive hyperemia, of nine patients with acute total ATR at 2weeks post-operatively. At 3months post-operatively, patient-reported outcome was assessed using Achilles tendon Total Rupture Score (ATRS). At 1year a uniform outcome score, Achilles Combined Outcome Score (ACOS), was obtained by combining validated, independent, patient-reported (ATRS), and functional outcome (heel-rise test) measures. An improved combined patient-reported and functional outcome, ACOS, at 1year was significantly correlated with higher maximum blood flow (r=.777, P=.040) in the injured limb. Furthermore, enhanced patient-reported outcome, ATRS, at 3months, was associated with an elevated ratio of maximum to resting blood flow (r=.809, P=.015) in the uninjured limb. Blood flow in early tendon healing is associated with long-term patient-reported and functional outcomes after ATR. The microcirculatory blood flow of both the healing and contralateral Achilles tendon seems to determine the healing potential after injury.
C1 [Praxitelous, P.; Ackermann, P. W.] Karolinska Inst, Integrat Orthoped Lab, Dept Mol Med & Surg, Stockholm, Sweden.
   [Praxitelous, P.; Ackermann, P. W.] Karolinska Univ Hosp, Dept Orthoped, Stockholm, Sweden.
   [Edman, G.] Tiohundra AB, Dept Psychiat, Norrtalje, Sweden.
RP Ackermann, PW (通讯作者)，Karolinska Univ Hosp, Dept Orthoped, Stockholm, Sweden.
EM paul.ackermann@karolinska.se
RI W, Ackermann P/B-9714-2008
OI W, Ackermann P/0000-0002-5520-169X
FU Vetenskapsradet [2012-3510]; Stockholms Lans Landsting [SLL20130150];
   Swedish Research Council for Sport Science [P2016-0090]
FX Vetenskapsradet, Grant/Award Number: 2012-3510; Stockholms Lans
   Landsting, Grant/Award Number: SLL20130150; Swedish Research Council for
   Sport Science: P2016-0090
CR Alim MA, 2016, BMJ OPEN SPORT EXERC, V2, P1
   Arverud ED, 2016, MLTJ-MUSCLE LIGAMENT, V6, P90, DOI 10.11138/mltj/2016.6.1.090
   Astrom M, 2000, SCAND J MED SCI SPOR, V10, P365, DOI 10.1034/j.1600-0838.2000.010006365.x
   Bostick GP, 2010, J ORTHOP SPORT PHYS, V40, P345, DOI 10.2519/jospt.2010.3204
   Cetti R, 2003, ACTA ORTHOP SCAND, V74, P78, DOI 10.1080/00016470310013707
   Cohen J., 1988, STAT POWER SOCIAL SC, P98
   Domeij-Arverud E, 2015, BONE JOINT J, V97B, P675, DOI 10.1302/0301-620X.97B5.34581
   Domeij-Arverud E, 2013, BONE JOINT J, V95B, P1227, DOI 10.1302/0301-620X.95B9.31162
   Domeij-Arverud E, 2015, ACUTE ACHILLES TENDO, P49
   Haggmark T, 1979, Am J Sports Med, V7, P121, DOI 10.1177/036354657900700208
   HASTAD K, 1959, Acta Chir Scand, V116, P251
   Jarvinen M, 1997, SCAND J MED SCI SPOR, V7, P86
   JOCHMANN W, 1993, VASA-J VASCULAR DIS, V22, P306
   Kangas J, 2007, AM J SPORT MED, V35, P59, DOI 10.1177/0363546506293255
   Karthikeyan S, 2015, J SHOULDER ELB SURG, V24, P1954, DOI 10.1016/j.jse.2015.07.014
   Kjaer M, 2000, SCAND J MED SCI SPOR, V10, P326, DOI 10.1034/j.1600-0838.2000.010006326.x
   Klein Erin E, 2013, Foot Ankle Spec, V6, P276, DOI 10.1177/1938640013489343
   Knobloch K, 2006, AM J SPORT MED, V34, P92, DOI 10.1177/0363546505278705
   Moller M, 2005, KNEE SURG SPORT TR A, V13, P60, DOI 10.1007/s00167-003-0441-0
   Morales F, 2005, MICROVASC RES, V69, P17, DOI 10.1016/j.mvr.2005.01.006
   Nilsson-Helander K, 2007, AM J SPORT MED, V35, P421, DOI 10.1177/0363546506294856
   Nilsson-Helander K, 2010, AM J SPORT MED, V38, P2186, DOI 10.1177/0363546510376052
   Olsson N, 2014, SCAND J MED SCI SPOR, V24, P152, DOI 10.1111/j.1600-0838.2012.01497.x
   Olsson N, 2014, AM J SPORT MED, V42, P1448, DOI 10.1177/0363546514527409
   Scott Ryan T, 2013, Foot Ankle Spec, V6, P283, DOI 10.1177/1938640013490019
   Silbernagel KG, 2012, AM J SPORT MED, V40, P1564, DOI 10.1177/0363546512447926
   Silbernagel KG, 2007, AM J SPORT MED, V35, P897, DOI 10.1177/0363546506298279
   Silbernagel KG, 2006, KNEE SURG SPORT TR A, V14, P1207, DOI 10.1007/s00167-006-0150-6
   Silbernagel KG, 2010, KNEE SURG SPORT TR A, V18, P258, DOI 10.1007/s00167-009-0889-7
   Svantesson U, 1998, SCAND J MED SCI SPOR, V8, P252, DOI 10.1111/j.1600-0838.1998.tb00479.x
   Wong J, 2002, AM J SPORT MED, V30, P565, DOI 10.1177/03635465020300041701
   Yvonne-Tee GB, 2008, CLIN HEMORHEOL MICRO, V38, P119
NR 32
TC 9
Z9 9
U1 1
U2 4
PU WILEY
PI HOBOKEN
PA 111 RIVER ST, HOBOKEN 07030-5774, NJ USA
SN 0905-7188
EI 1600-0838
J9 SCAND J MED SCI SPOR
JI Scand. J. Med. Sci. Sports
PD JAN
PY 2018
VL 28
IS 1
BP 294
EP 302
DI 10.1111/sms.12892
PG 9
WC Sport Sciences
WE Science Citation Index Expanded (SCI-EXPANDED)
SC Sport Sciences
GA FS7ZX
UT WOS:000422630100033
PM 28378372
DA 2022-07-22
ER

PT J
AU Saarensilta, IA
   Edman, G
   Ackermann, PW
AF Saarensilta, I. A.
   Edman, G.
   Ackermann, P. W.
TI Achilles tendon ruptures during summer show the lowest incidence, but
   exhibit an increased risk of re-rupture
SO KNEE SURGERY SPORTS TRAUMATOLOGY ARTHROSCOPY
LA English
DT Article
DE Epidemiology; Seasons; Deep venous thrombosis; Infection; Seasonal
   variation; Achilles tendon Total Rupture Score
ID DEEP-VEIN THROMBOSIS; INJURY; EPIDEMIOLOGY; METAANALYSIS; COMPRESSION;
   SURGERY
AB Purpose Achilles tendon rupture (ATR) is a common injury. The knowledge of seasonal factors ' impact is incomplete, but may provide means for preventive approaches for Achilles tendon related morbidity. The aim of this study was to investigate seasonal variations in ATR incidence in relation to injury mechanism, adverse events including risk of re-rupture, and patient-reported outcome in adults in Stockholm, Sweden. Methods In total, 349 patients with unilateral acute Achilles tendon rupture, prospectively treated with standardized surgical techniques, were retrospectively assessed. Date of injury was assigned to one of the four internationally defined meteorological seasons in the northern hemisphere. Injury mechanism and the rate of adverse events; deep venous thrombosis, infection and re-rupture in relation to per-operative complications. Patient-reported outcome at 1 year was assessed with the validated Achilles tendon Total Rupture Score. Results ATR incidence was significantly highest during winter and spring, and lowest during summer (p < 0.05). The most common sporting activities associated with ATR were badminton, floorball and soccer (> 50%). The rate of soccer-related ATR was highest during summer (p < 0.05). Patients sustaining an ATR during summer, compared to other seasons, exhibited more per-operative complications (p < 0.05), a significantly higher risk of re-rupture (p < 0.05) and a lower rate of good outcome (n.s.). The risk of other adverse events after ATR did not differ between the seasons. Conclusion Winter and spring are the high risk seasons for sports-related ATR and the risk sports are badminton, soccer and floorball. The reason for the higher risk of re-rupture after ATR repair during summer should be further investigated.
C1 [Saarensilta, I. A.; Ackermann, P. W.] Karolinska Inst, Dept Mol Med & Surg, Stockholm, Sweden.
   [Edman, G.] Tiohundra AB, Dept Psychiat, Norrtalje, Sweden.
   [Ackermann, P. W.] Karolinska Univ Hosp, Dept Orthoped Surg, S-17176 Stockholm, Sweden.
RP Ackermann, PW (通讯作者)，Karolinska Inst, Dept Mol Med & Surg, Stockholm, Sweden.; Ackermann, PW (通讯作者)，Karolinska Univ Hosp, Dept Orthoped Surg, S-17176 Stockholm, Sweden.
EM paul.ackermann@sll.se
RI W, Ackermann P/B-9714-2008
OI W, Ackermann P/0000-0002-5520-169X
FU Stockholm County Council (ALF project) [SLL20180348]; Swedish Research
   Council [2017-00202]
FX This study was supported by grants provided by the Stockholm County
   Council (ALF project): SLL20180348, the Swedish Research Council
   (Project No. 2017-00202). Neither funding agency had influence over or
   took part in the design or conduct of the study or in the analysis or
   interpretation of the study data.
CR Aufwerber S, 2020, KNEE SURG SPORT TR A, V28, P312, DOI 10.1007/s00167-019-05767-x
   Bohm S, 2015, SPORTS MED-OPEN, V1, DOI 10.1186/s40798-015-0009-9
   Caldwell JME, 2018, JAAOS GLOB RES REV, V2, DOI 10.5435/JAAOSGlobal-D-18-00043
   Dentali F, 2011, THROMB HAEMOSTASIS, V106, P439, DOI 10.1160/TH11-02-0116
   Domeij-Arverud E, 2015, BONE JOINT J, V97B, P675, DOI 10.1302/0301-620X.97B5.34581
   Domeij-Arverud E, 2013, BONE JOINT J, V95B, P1227, DOI 10.1302/0301-620X.95B9.31162
   Domeij-Arverud E, 2016, ADV EXP MED BIOL, V920, P221, DOI 10.1007/978-3-319-33943-6_21
   Egger AC, 2017, CURR REV MUSCULOSKE, V10, P72, DOI 10.1007/s12178-017-9386-7
   Ganestam A, 2016, KNEE SURG SPORT TR A, V24, P3730, DOI 10.1007/s00167-015-3544-5
   Gulati V, 2015, WORLD J ORTHOP, V6, P380, DOI 10.5312/wjo.v6.i4.380
   Hess Gregory William, 2010, Foot Ankle Spec, V3, P29, DOI 10.1177/1938640009355191
   Houshian S, 1998, INJURY, V29, P651, DOI 10.1016/S0020-1383(98)00147-8
   Huntley Samuel R, 2018, Foot (Edinb), V37, P61, DOI 10.1016/j.foot.2018.04.008
   Huttunen TT, 2014, AM J SPORT MED, V42, P2419, DOI 10.1177/0363546514540599
   Jarvinen Tero A H, 2005, Foot Ankle Clin, V10, P255, DOI 10.1016/j.fcl.2005.01.013
   Lapidus L, 2006, J THROMB HAEMOST, V4, P807, DOI 10.1111/j.1538-7836.2006.01832.x
   Lapidus LJ, 2007, J ORTHOP TRAUMA, V21, P52, DOI 10.1097/01.bot.0000250741.65003.14
   Lauersen JB, 2014, BRIT J SPORT MED, V48, DOI 10.1136/bjsports-2013-092538
   Nilsson-Helander K, 2007, AM J SPORT MED, V35, P421, DOI 10.1177/0363546506294856
   Nilsson-Helander K, 2009, KNEE SURG SPORT TR A, V17, P1234, DOI 10.1007/s00167-009-0727-y
   Nyyssonen T, 2000, ANN CHIR GYNAECOL, V89, P53
   Raikin SM, 2013, FOOT ANKLE INT, V34, P475, DOI 10.1177/1071100713477621
   Rosfors S, 2010, THROMB J, V8, DOI 10.1186/1477-9560-8-4
   Scott A, 2014, BMJ OPEN, V4, DOI 10.1136/bmjopen-2013-004320
   Suchak AA, 2005, FOOT ANKLE INT, V26, P932, DOI 10.1177/107110070502601106
   Svedman S, 2018, AM J SPORT MED, V46, P2929, DOI 10.1177/0363546518793655
   Thorborg K, 2017, BRIT J SPORT MED, V51, P562, DOI 10.1136/bjsports-2016-097066
NR 27
TC 6
Z9 6
U1 1
U2 5
PU SPRINGER
PI NEW YORK
PA ONE NEW YORK PLAZA, SUITE 4600, NEW YORK, NY, UNITED STATES
SN 0942-2056
EI 1433-7347
J9 KNEE SURG SPORT TR A
JI Knee Surg. Sports Traumatol. Arthrosc.
PD DEC
PY 2020
VL 28
IS 12
BP 3978
EP 3986
DI 10.1007/s00167-020-05982-x
EA APR 2020
PG 9
WC Orthopedics; Sport Sciences; Surgery
WE Science Citation Index Expanded (SCI-EXPANDED)
SC Orthopedics; Sport Sciences; Surgery
GA OS3EA
UT WOS:000527439000001
PM 32313989
OA hybrid, Green Published
DA 2022-07-22
ER

PT J
AU Metz, R
   van der Heijden, GJMG
   Verleisdonk, EJMM
   Kolfschoten, N
   Verhofstad, MHJ
   van der Werken, C
AF Metz, Roderik
   van der Heijden, Geert J. M. G.
   Verleisdonk, Egbert-Jan M. M.
   Kolfschoten, Nicky
   Verhofstad, Michiel H. J.
   van der Werken, Christiaan
TI Effect of Complications After Minimally Invasive Surgical Repair of
   Acute Achilles Tendon Ruptures Report on 211 Cases
SO AMERICAN JOURNAL OF SPORTS MEDICINE
LA English
DT Article
DE Achilles tendon rupture; surgery; complications; long-term outcome
ID PERCUTANEOUS REPAIR
AB Background: Complications of acute Achilles tendon rupture treatment are considered to negatively influence outcome, but the relevance of these effects is largely unknown.
   Purpose: The Achilles Tendon Total Rupture Score (ATRS) was used to determine level of disability in patients with minimally invasive surgical Achilles tendon rupture repair with a complicated postoperative course.
   Study Design: Case series; Level of evidence, 4.
   Methods: The charts of 340 consecutive patients treated for an acute Achilles tendon rupture by minimally invasive surgical repair were reviewed. Complications were categorized as rerupture, severe wound infection, sural nerve injury, and other. Level of disability was evaluated by the 10-item ATRS with a sum score of minimum 0 to maximum 100 points.
   Results: A total of 211 patients returned a completed questionnaire: mean follow-up was 6.2 years (range, 3-10 years). Mean ATRS for all 211 patients was 84 (95% confidence interval: 82, 87). The mean ATRS for the 135 (64%) uncomplicated cases was 89 of 100 points, 71 points for the 17 (8%) patients with a rerupture (95% confidence interval: 63, 79; P < .0001), 79 points for the 41 patients (19%) with a sural nerve injury (95% confidence interval: 74, 85; P = .0008), and 75 points for the 17 patients (8%) with another complication (95% confidence interval: 67, 83; P = .001). Of these other complications, 13 patients (6%) suffered a wound-healing complication considered minor. Their average ATRS score was 80 points (95% confidence interval: 71, 88.7; P = .0445). One patient suffered a severe wound infection as well, scoring 28 of 100 points. Rerupture significantly increased the risk of quitting or changing sport participation on the long term.
   Conclusion: Long-term outcome after minimally invasive Achilles tendon rupture repair is excellent. Rerupture and severe wound infection are the most important complications with lasting negative effect on outcome. It justifies the use of rerupture as a relevant outcome measure in treatment evaluation.
C1 St Elizabeth Hosp, Tilburg, Netherlands.
   Diakonessen Hosp, Utrecht, Netherlands.
   Univ Med Ctr, Utrecht, Netherlands.
RP Metz, R (通讯作者)，Erasmus MC, Gravendijkwal 230, NL-3015 CE Rotterdam, Netherlands.
EM metz_r@hotmail.com
RI van der Heijden, Geert/M-3733-2013
OI van der Heijden, Geert/0000-0003-4979-2099
CR Assal M, 2002, J BONE JOINT SURG AM, V84A, P161, DOI 10.2106/00004623-200202000-00001
   Khan RJ, 2004, COCHRANE DB SYST REV, V3, DOI DOI 10.1002/14651858.CD003674.PUB2
   KITAOKA HB, 1994, FOOT ANKLE INT, V15, P349, DOI 10.1177/107110079401500701
   Lansdaal JR, 2007, INJURY, V38, P839, DOI 10.1016/j.injury.2006.12.010
   LEPPILAHTI J, 1998, CLIN ORTHOP RELAT R, V346, P152
   MA GWC, 1977, CLIN ORTHOP RELAT R, P247
   Majewski M, 2006, AM J SPORT MED, V34, P793, DOI 10.1177/0363546505283266
   Nilsson-Helander K, 2007, AM J SPORT MED, V35, P421, DOI 10.1177/0363546506294856
   Pajala A, 2002, J BONE JOINT SURG AM, V84A, P2016, DOI 10.2106/00004623-200211000-00017
   SCOTT N, 1979, CLIN ORTHOP RELAT R, V140, P175
   van der Werken C., 2000, ORTHOP TRAUMATOL, V8, P285
   Webb JM, 1999, J BONE JOINT SURG BR, V81B, P877, DOI 10.1302/0301-620X.81B5.9784
NR 12
TC 26
Z9 27
U1 0
U2 5
PU SAGE PUBLICATIONS INC
PI THOUSAND OAKS
PA 2455 TELLER RD, THOUSAND OAKS, CA 91320 USA
SN 0363-5465
EI 1552-3365
J9 AM J SPORT MED
JI Am. J. Sports Med.
PD APR
PY 2011
VL 39
IS 4
BP 820
EP 824
DI 10.1177/0363546510392012
PG 5
WC Orthopedics; Sport Sciences
WE Science Citation Index Expanded (SCI-EXPANDED)
SC Orthopedics; Sport Sciences
GA 744CO
UT WOS:000289071300017
PM 21289275
DA 2022-07-22
ER

PT J
AU De Carli, A
   Vadala, A
   Ciardini, R
   Iorio, R
   Ferretti, A
AF De Carli, A.
   Vadala, A.
   Ciardini, R.
   Iorio, R.
   Ferretti, A.
TI Spontaneous Achilles tendon ruptures treated with a mini-open technique:
   clinical and functional evaluation
SO JOURNAL OF SPORTS MEDICINE AND PHYSICAL FITNESS
LA English
DT Article
DE Achilles tendon; Rupture; Subcutaneous tissue
ID NONOPERATIVE TREATMENT; PERCUTANEOUS REPAIR; SURGICAL REPAIR
AB Aim and methods. Spontaneous ruptures of the Achilles tendon are frequent among occasional and high level athletes. Despite the frequency of this problem, there is no agreement about the treatment of this pathology. The purpose of this study was to show the results of 20 consecutive patients (14 males and 6 females) surgically treated between 1995 and 2001 with a miniopen technique (mean follow-up: 52 months; range 20-95 months). Postoperatively, all these patients were assessed with a new functional scoring scale and a functional jumping test (Ergo-Jump Bosco System). Results. A very low rate of minor complications have been reported and most of these patients are now practicing their favoured sport at the same pre-injury level. Conclusion. Mini open repair should be considered an excellent method of treatment of this pathology, with a very low rate of complications. The authors recommend this technique for young professional and occasional athletes.
C1 Univ Roma La Sapienza, Orthoped Unit, S Andrea Hosp, Rome, Italy.
   Univ Roma La Sapienza, Kirk Kilgour Sports Injury Ctr, S Andrea Hosp, Rome, Italy.
EM angelo.decarli@tin.it
OI DE CARLI, ANGELO/0000-0001-5469-5782; IORIO,
   RAFFAELE/0000-0003-1599-7096
CR ARNER O, 1959, ACTA CHIR SCAND    S, V239, P1
   BESKIN JL, 1987, AM J SPORT MED, V15, P1, DOI 10.1177/036354658701500101
   BRADLEY JP, 1990, AM J SPORT MED, V18, P188, DOI 10.1177/036354659001800213
   Carlstedt C A, 1987, Acta Orthop Scand Suppl, V224, P1
   Casteleyn P P, 1980, Acta Orthop Belg, V46, P310
   CETTI R, 1993, AM J SPORT MED, V21, P791, DOI 10.1177/036354659302100606
   Dal Monte A., 1983, VALUTAZIONE FUNZIONA
   FOX JM, 1975, CLIN ORTHOP RELAT R, P221
   GILLIES H, 1970, J BONE JOINT SURG AM, VA 52, P337, DOI 10.2106/00004623-197052020-00015
   INGLIS AE, 1981, CLIN ORTHOP RELAT R, V156, P160
   JOZSA L, 1989, AM J SPORT MED, V17, P338, DOI 10.1177/036354658901700305
   KAKIUCHI M, 1995, J BONE JOINT SURG BR, V77B, P60, DOI 10.1302/0301-620X.77B1.7822398
   LAGERGREN C, 1959, Acta Chir Scand, V116, P491
   Lo IKY, 1997, CLIN J SPORT MED, V7, P207, DOI 10.1097/00042752-199707000-00010
   MA GWC, 1977, CLIN ORTHOP RELAT R, P247
   Maffulli N, 1999, J BONE JOINT SURG AM, V81A, P1019, DOI 10.2106/00004623-199907000-00017
   McComis GP, 1997, J BONE JOINT SURG AM, V79A, P1799, DOI 10.2106/00004623-199712000-00005
   NISTOR L, 1981, J BONE JOINT SURG AM, V63, P394, DOI 10.2106/00004623-198163030-00012
   PAPANDREA P, 1990, J SPORTS TRAUMATOL
   Soldatis JJ, 1997, AM J SPORT MED, V25, P90, DOI 10.1177/036354659702500118
   Webb JM, 1999, J BONE JOINT SURG BR, V81B, P877, DOI 10.1302/0301-620X.81B5.9784
   WILLS CA, 1986, CLIN ORTHOP RELAT R, V207, P156
NR 22
TC 8
Z9 8
U1 0
U2 0
PU EDIZIONI MINERVA MEDICA
PI TURIN
PA CORSO BRAMANTE 83-85 INT JOURNALS DEPT., 10126 TURIN, ITALY
SN 0022-4707
EI 1827-1928
J9 J SPORT MED PHYS FIT
JI J. Sports Med. Phys. Fit.
PD SEP
PY 2009
VL 49
IS 3
BP 292
EP 296
PG 5
WC Sport Sciences
WE Science Citation Index Expanded (SCI-EXPANDED)
SC Sport Sciences
GA 538IZ
UT WOS:000273179100009
PM 19861935
DA 2022-07-22
ER

PT J
AU Sommer, B
   Hollenstein, A
   Graf, E
AF Sommer, Bettina
   Hollenstein, Andreas
   Graf, Eveline
TI Stability boots for the treatment of Achilles tendon injuries: Gait
   analysis of healthy participants
SO GAIT & POSTURE
LA English
DT Article
DE Achilles tendon; Achilles tendon injury; Stability boots; Gait analysis
ID EARLY FUNCTIONAL REHABILITATION; RUPTURE; WALKING; MANAGEMENT
AB Background: Achilles tendon injuries are commonly treated with stability boots that secure the ankle at a specific position and aim to reduce loading of the tendon. These boots allow full weight bearing by limiting the range of movement. It is, however, unknown, to what extent these boots can reduce tendon loading and if the biomechanics are altered during walking. Research question: How do orthopedic boots influence lower extremity biomechanics during walking? Methods: For this cross-sectional study, ten healthy participants walked with three orthopedic boots (Oped Vacoped, Kuenzli Ortho Rehab Absolut, Orthotech Variostabil) commonly used to treat Achilles tendon injuries. Kinematics and kinetics of the lower extremity of the booted leg and spatiotemporal parameters of both sides were collected using motion-capturing system and dynamometry. Each boot was tested in the maximally plantarflexed position. Group differences between boot conditions were analyzed by means of repeated-measures ANOVA and post-hoc paired t-test. Results: The boot dorsiflexion range of motion differed significantly between boots with Vacoped (1.8 degrees (0.3)) showing the smallest range, followed by Kuenzli (5.0 degrees (1.3)) and Orthotech (7.9 degrees (1.7)). Orthotech displayed a higher peak plantarflexion moment (1.36 Nm/kg (0.09)) than both Kuenzli (1.06 Nm/kg (0.12)) and Vacoped (1.04 Nm/kg (0.14)). Concerning loading over time, significant differences in the plantarflexion impulse were found, with the highest impulse in Vacoped (0.42 Nms/kg (0.06)), followed by Orthotech (0.29 Nms/kg (0.03)) and Kuenzli (0.25 Nms/kg (0.05)). In addition, asymmetries were seen in stance and step length for the booted and contralateral sides. Significance: The lower extremity biomechanics were affected by the boots, with Kuenzli showing the lowest joint loading, Vacoped the smallest joint motion and Orthotech the most natural gait pattern. Future research is needed to determine the most relevant variable expressing the risk of re-rupture of the Achilles tendon in order to conclude which boot may be most favorable to use in clinical practice.
C1 [Sommer, Bettina; Hollenstein, Andreas; Graf, Eveline] Zurich Univ Appl Sci, Sch Hlth Profess, Inst Physiotherapy, Katharina Sulzer Pl 9, CH-8401 Winterthur, Switzerland.
RP Graf, E (通讯作者)，Zurich Univ Appl Sci, Sch Hlth Profess, Inst Physiotherapy, Katharina Sulzer Pl 9, CH-8401 Winterthur, Switzerland.
EM eveline.graf@zhaw.ch
OI Sommer, Bettina/0000-0003-1231-2987; Graf, Eveline/0000-0003-4740-8145
FU Research Fund Aargau, Switzerland
FX The authors would like to thank Nadine Hadfliger for her assistance with
   the literature search, Karen Linwood for proofreading, all partici-pants
   for voluntarily taking part in this study and Kuenzli SwissSchuh AG for
   providing the test boots. This study was funded by the Research Fund
   Aargau, Switzerland.
CR Agres AN, 2015, SCAND J MED SCI SPOR, V25, P860, DOI 10.1111/sms.12456
   Aufwerber S, 2019, AM J SPORT MED, V47, P894, DOI 10.1177/0363546518824326
   Balasubramanian CK, 2009, GAIT POSTURE, V29, P408, DOI 10.1016/j.gaitpost.2008.10.061
   Barfod KW, 2014, J BONE JOINT SURG AM, V96A, P1497, DOI 10.2106/JBJS.M.01273
   Bohm H, 2010, J BIOMECH, V43, P2467, DOI 10.1016/j.jbiomech.2010.05.029
   Bohannon RW, 2011, PHYSIOTHERAPY, V97, P182, DOI 10.1016/j.physio.2010.12.004
   Ecker TM, 2016, AM J SPORT MED, V44, P1004, DOI 10.1177/0363546515623501
   Frankewycz B, 2017, ARCH ORTHOP TRAUM SU, V137, P333, DOI 10.1007/s00402-017-2627-9
   Froberg A, 2009, AM J SPORT MED, V37, P1200, DOI 10.1177/0363546508330126
   Kauwe M, 2017, CLIN PODIATR MED SUR, V34, P229, DOI 10.1016/j.cpm.2016.10.009
   Kerrigan DC, 1998, AM J PHYS MED REHAB, V77, P2, DOI 10.1097/00002060-199801000-00002
   Kunzli SwissSchuh AG, ORTH REH ABS
   Lantto I, 2015, SCAND J MED SCI SPOR, V25, pE133, DOI 10.1111/sms.12253
   Lemme NJ, 2018, ORTHOP J SPORTS MED, V6, DOI 10.1177/2325967118808238
   List R, 2013, J STRENGTH COND RES, V27, P1529, DOI 10.1519/JSC.0b013e3182736034
   Lohrer H., 2003, ORTHOPADIESCHUHTECHN, V11
   McCormack R, 2015, BRIT J SPORT MED, V49, P1329, DOI 10.1136/bjsports-2015-094935
   Ochen Y, 2019, BMJ-BRIT MED J, V364, DOI 10.1136/bmj.k5120
   OPED, OPED PROD VACOPED M
   Orthotech GmbH, ORTH VAR STAB
   Pirker W, 2017, WIEN KLIN WOCHENSCHR, V129, P81, DOI 10.1007/s00508-016-1096-4
   Pollo FE, 1999, ORTHOPEDICS, V22, P503
   Reda Y, 2020, FOOT ANKLE SURG, V26, P280, DOI 10.1016/j.fas.2019.03.010
   SODERKVIST I, 1993, J BIOMECH, V26, P1473, DOI 10.1016/0021-9290(93)90098-y
   Zellers JA, 2019, GAIT POSTURE, V70, P59, DOI 10.1016/j.gaitpost.2019.02.027
NR 25
TC 0
Z9 0
U1 2
U2 3
PU ELSEVIER IRELAND LTD
PI CLARE
PA ELSEVIER HOUSE, BROOKVALE PLAZA, EAST PARK SHANNON, CO, CLARE, 00000,
   IRELAND
SN 0966-6362
EI 1879-2219
J9 GAIT POSTURE
JI Gait Posture
PD JAN
PY 2022
VL 91
BP 131
EP 136
DI 10.1016/j.gaitpost.2021.10.009
EA OCT 2021
PG 6
WC Neurosciences; Orthopedics; Sport Sciences
WE Science Citation Index Expanded (SCI-EXPANDED)
SC Neurosciences & Neurology; Orthopedics; Sport Sciences
GA WL9SD
UT WOS:000710736500014
PM 34689070
OA Green Published, hybrid
DA 2022-07-22
ER

PT J
AU Marian, IR
   Costa, ML
   Dutton, SJ
AF Marian, Ioana R.
   Costa, Matthew L.
   Dutton, Susan J.
TI Cast versus functional brace in the rehabilitation of patients with a
   rupture of the Achilles tendon: statistical analysis plan for the UK
   study of tendo Achilles rehabilitation (UK STAR) multi-centre randomised
   controlled trial
SO TRIALS
LA English
DT Article
DE Rehabilitation; Achilles tendon; Rupture; Statistical analysis plan;
   Randomised controlled trial
AB Background: The incidence of Achilles tendon rupture in the UK is increasing and the best rehabilitation strategy for patients treated non-operatively remains unclear. We describe a statistical analysis plan (SAP) for the UK study of tendo Achilles rehabilitation (UK STAR) multi-centre randomised trial.
   Methods/design: UK STAR is a 1:1, multi-centre, parallel, two-arm, superiority randomised controlled trial. This study aims to evaluate the use of functional bracing compared to plaster cast for the management of acute Achilles tendon rupture in adult patients treated non-operatively. The primary outcome is the Achilles Tendon Rupture Score measured at 9 months after injury and will be estimated based on a linear mixed effects regression model adjusted for the stratification factor (centre) and other key prognostic variables. Secondary outcomes include complications, quality of life and resource use evaluated at 8 weeks and at 3, 6 and 9 months after the injury. Missing data will be summarised and reported by treatment arm. Full details of the planned analysis methods are described in this paper. Further study design details are published in the UK STAR protocol.
   Discussion: The planned statistical analyses for UK STAR aim to reduce the risk of outcome reporting bias arising from prior data knowledge. Any changes or deviations from the current SAP will be described and justified in the final study report.
C1 [Marian, Ioana R.; Dutton, Susan J.] Univ Oxford, Nuffield Dept Orthopaed Rheumatol & Musculoskelet, Oxford Clin Trials Res Unit, Ctr Stat Med, Oxford OX3 7LD, England.
   [Costa, Matthew L.] Univ Oxford, John Radcliffe Hosp, Kadoorie Ctr, Oxford Trauma Nuffield Dept Orthopaed Rheumatol &, Oxford OX3 9DU, England.
RP Marian, IR (通讯作者)，Univ Oxford, Nuffield Dept Orthopaed Rheumatol & Musculoskelet, Oxford Clin Trials Res Unit, Ctr Stat Med, Oxford OX3 7LD, England.
EM ioana.marian@csm.ox.ac.uk
FU UK National Institute for Health Research Health Technology Assessment
   (HTA) Programme [13/115/62]; National Institute for Health Research
   (NIHR) Oxford Biomedical Research Centre
FX This project was funded by the UK National Institute for Health Research
   Health Technology Assessment (HTA) Programme (project no 13/115/62) and
   was supported by the National Institute for Health Research (NIHR)
   Oxford Biomedical Research Centre.
CR Achten J, 2017, BMJ OPEN, V7, DOI 10.1136/bmjopen-2017-019628
   American Academy of Orthopaedic Surgeons, 2009, DIAGN TREATM AC ACH
   Bell ML, 2014, SAGE OPEN, V4, DOI 10.1177/2158244014534858
   Calvert M, 2013, JAMA-J AM MED ASSOC, V309, P814, DOI 10.1001/jama.2013.879
   Costa ML, 2006, J BONE JOINT SURG BR, V88B, P69, DOI 10.1302/0301-620X.88B1.16549
   Dunn G, 2005, STAT METHODS MED RES, V14, P369, DOI 10.1191/0962280205sm403oa
   Finfer S, 2009, CRIT CARE RESUSC, V11, P5
   Gamble C, 2017, JAMA-J AM MED ASSOC, V318, P2337, DOI 10.1001/jama.2017.18556
   Kangas J, 2003, J TRAUMA, V54, P1171, DOI 10.1097/01.TA.0000047945.20863.A2
   Kearney RS, 2012, HEALTH QUAL LIFE OUT, V10, DOI 10.1186/1477-7525-10-24
   Kearney RS, 2011, BMC MED RES METHODOL, V11, DOI 10.1186/1471-2288-11-39
   Khan RJ, 2004, COCHRANE DB SYST REV, V3, DOI DOI 10.1002/14651858.CD003674.PUB2
   Maffulli N, 1999, CLIN J SPORT MED, V9, P157, DOI 10.1097/00042752-199907000-00007
   Nilsson-Helander K, 2007, AM J SPORT MED, V35, P421, DOI 10.1177/0363546506294856
   Sullivan TR, 2018, STAT METHODS MED RES, V27, P2610, DOI 10.1177/0962280216683570
   van Hout B, 2012, VALUE HEALTH, V15, P708, DOI 10.1016/j.jval.2012.02.008
NR 16
TC 1
Z9 1
U1 1
U2 2
PU BMC
PI LONDON
PA CAMPUS, 4 CRINAN ST, LONDON N1 9XW, ENGLAND
SN 1745-6215
J9 TRIALS
JI Trials
PD MAY 30
PY 2019
VL 20
AR 311
DI 10.1186/s13063-019-3380-x
PG 7
WC Medicine, Research & Experimental
WE Science Citation Index Expanded (SCI-EXPANDED)
SC Research & Experimental Medicine
GA IA8DP
UT WOS:000469788400001
PM 31146789
OA Green Published, gold
DA 2022-07-22
ER

PT J
AU Ocguder, DA
   Dogan, M
   Bektaser, SB
   Akgun, E
   Tolunay, T
   Ugurlu, M
AF Ocguder, Durmus Ali
   Dogan, Metin
   Bektaser, Suleyman Bulent
   Akgun, Erkan
   Tolunay, Tolga
   Ugurlu, Mahmut
TI Comparison of the open primary repair with augmentation and without
   augmentation in acute achilles tendon rupture
SO TURKISH JOURNAL OF MEDICAL SCIENCES
LA English
DT Article
DE Achilles tendon; injuries; augmentation; surgery
AB Aim: To compare the clinical and the functional efficiency of 2 surgical methods that are used in open primary repair of acute Achilles tendon ruptures: one that utilizes the augmentation of the plantaris tendon and one that does not.
   Materials and methods: Data were evaluated from 41 patients that underwent surgical repair for acute Achilles tendon ruptures with these 2 different surgery methods. In the first group, which included 21 patients (19 male, 2 female; mean age: 36.8 years; age range: 22 to 49), we performed augmentation with the plantaris tendon after an end-to-end primary repair with a modified Kessler technique. In the second group, which included 20 patients (19 male, 1 female; mean age: 41.4 years; age range: 23 to 52), we did not perform augmentation after the end-to-end primary repair with a modified Kessler technique. For both groups, we used polydioxanone suture (PDS) materials in Kessler knots.
   Results: The mean American Orthopedic Foot and Ankle Society (AOFAS) hindfoot clinical outcome score was 94 6.4 (range: 73-100) in the first group. In the second group, the mean AOFAS score was 94.4 +/- 6.1 (range: 76-100). The first group's mean Achilles tendon assessment score was 81.7 +/- 10.9 (good, range of 60-96); the mean Achilles tendon assessment score of the second group was 82.4 +/- 6.1 (good, range of 71 to 94). There was no statistically significant difference between these 2 groups in terms of functional scores (P > 0.05). In the first group, 2 patients (9.5%) had superficial skin infections and 1 patient (4.7%) had hypertrophic scar tissue. In the second group, 3 patients (15%) had superficial skin infections and 1 patient (5%) experienced a partial rerupture.
   Conclusion: Clinical and functional results did not show any statistically significant difference between methods with or without augmentation when appropriate and progressive rehabilitation programs were applied in treating acute Achilles tendon ruptures.
C1 [Ocguder, Durmus Ali; Bektaser, Suleyman Bulent; Akgun, Erkan; Tolunay, Tolga] Ataturk Training & Res Hosp, Dept Orthoped Surg, Ankara, Turkey.
   [Dogan, Metin; Ugurlu, Mahmut] Yildirim Beyazit Univ, Dept Orthoped Surg, Fac Med, Ankara, Turkey.
RP Ocguder, DA (通讯作者)，Umit Mahallesi,444 Sokak,Kermes Sitesi 3,Blok 27, TR-06580 Ankara, Turkey.
EM aliocguder@yahoo.com
RI Doğan, Metin/AAC-3199-2020
CR Akgun Umut, 2006, Acta Orthop Traumatol Turc, V40, P228
   Aktas Seref, 2007, J Foot Ankle Surg, V46, P336, DOI 10.1053/j.jfas.2007.06.006
   Bhandari M, 2002, CLIN ORTHOP RELAT R, P190
   CARDEN DG, 1987, J BONE JOINT SURG BR, V69, P416, DOI 10.1302/0301-620X.69B3.3294839
   Dalton GP, 2001, CLIN ORTHOP RELAT R, P133
   DeCarbo WT, 2008, J FOOT ANKLE SURG, V47, P69, DOI 10.1053/j.jfas.2007.09.001
   GERDES MH, 1992, CLIN ORTHOP RELAT R, P241
   KELLAM JF, 1985, CLIN ORTHOP RELAT R, P80
   KITAOKA HB, 1994, FOOT ANKLE INT, V15, P349, DOI 10.1177/107110079401500701
   Leppilahti J, 1998, CLIN ORTHOP RELAT R, P152
   Lui T H, 2009, Foot Ankle Surg, V15, P58, DOI 10.1016/j.fas.2008.06.004
   LYNN TA, 1966, J BONE JOINT SURG AM, VA 48, P268, DOI 10.2106/00004623-196648020-00005
   Maffulli N, 1999, J BONE JOINT SURG AM, V81A, P1019, DOI 10.2106/00004623-199907000-00017
   MANN RA, 1991, J BONE JOINT SURG AM, V73A, P214, DOI 10.2106/00004623-199173020-00009
   McComis GP, 1997, J BONE JOINT SURG AM, V79A, P1799, DOI 10.2106/00004623-199712000-00005
   MISKULIN M, J FOOT ANKLE SURG
   Moller M, 2001, J BONE JOINT SURG BR, V83B, P843
   Naim Fadel, 2005, Acta Orthop Traumatol Turc, V39, P1
   NISTOR L, 1981, J BONE JOINT SURG AM, V63, P394, DOI 10.2106/00004623-198163030-00012
   THERMANN H, 1995, UNFALLCHIRURG, V98, P21
   Vega Jordi, 2008, Foot Ankle Surg, V14, P204, DOI 10.1016/j.fas.2008.02.005
   Winter E, 1998, ARCH ORTHOP TRAUM SU, V117, P364, DOI 10.1007/s004020050267
   Wong MWN, 2005, CLIN ORTHOP RELAT R, P201, DOI 10.1097/01.blo.0000150460.92012.7a
   YEL M, 1999, ACTA ORTHOP TRAUMATO, V33, P313
   Yildirim Yakup, 2006, Acta Orthop Traumatol Turc, V40, P164
NR 25
TC 3
Z9 3
U1 0
U2 5
PU TUBITAK SCIENTIFIC & TECHNICAL RESEARCH COUNCIL TURKEY
PI ANKARA
PA ATATURK BULVARI NO 221, KAVAKLIDERE, ANKARA, 00000, TURKEY
SN 1300-0144
EI 1303-6165
J9 TURK J MED SCI
JI Turk. J. Med. Sci.
PD AUG
PY 2011
VL 41
IS 4
BP 639
EP 646
DI 10.3906/sag-1003-699
PG 8
WC Medicine, General & Internal
WE Science Citation Index Expanded (SCI-EXPANDED)
SC General & Internal Medicine
GA 812EU
UT WOS:000294274700011
OA Green Submitted, Bronze
DA 2022-07-22
ER

PT J
AU Silbernagel, KG
   Nilsson-Helander, K
   Thomee, R
   Eriksson, BI
   Karlsson, J
AF Silbernagel, Karin Gravare
   Nilsson-Helander, Katarina
   Thomee, Roland
   Eriksson, Bengt I.
   Karlsson, Jon
TI A new measurement of heel-rise endurance with the ability to detect
   functional deficits in patients with Achilles tendon rupture
SO KNEE SURGERY SPORTS TRAUMATOLOGY ARTHROSCOPY
LA English
DT Article
DE Achilles tendon; Rupture; Tendon elongation; Complications;
   Plantarflexion
ID NONOPERATIVE TREATMENT; OPERATIVE TREATMENT; MUSCLE FUNCTION; REPAIR;
   METAANALYSIS; RELIABILITY
AB Studies evaluating treatment effects on muscle function after an Achilles tendon rupture often use various tests for evaluating calf muscle strength. However, these tests rarely demonstrate the difference between treatment groups; therefore, new tests with a higher ability to detect possible differences in outcome are needed. The purpose of this study was to evaluate the validity and ability to detect differences in outcome of a heel-rise work test that would measure both the height of each heel-rise and the number of repetitions. Seventy-eight patients (65 men and 13 women) at a mean (standard deviation) age of 42 (9) years with Achilles tendon ruptures were included. The patients were evaluated with the new heel-rise test at 6 and 12 months after injury. The limb symmetry index (LSI = involved/uninvolved x 100) was calculated to determine the size of the difference in function between the injured and the uninjured side. The heel-rise height differed significantly between the injured and uninjured sides at the 6- and 12-month evaluations (P < 0.001). At the 6-month evaluation, the patients had achieved a mean LSI of 84% on the number of repetitions parameter but only a mean LSI of 61% on the work parameter. At the 12-month evaluation the mean, LSI of the heel-rise repetition parameter was 95%, indicating that the patients had fully recovered function, but on the work parameter the mean LSI was only 76%. The heel-rise work test in the present study has good validity and greater ability to detect differences between the injured and the uninjured sides than a test that measures only the number of heel-rise repetitions in patients with Achilles tendon rupture.
C1 [Silbernagel, Karin Gravare; Nilsson-Helander, Katarina; Thomee, Roland; Eriksson, Bengt I.; Karlsson, Jon] Univ Gothenburg, Sahlgrenska Univ Hosp, Sahlgrenska Acad, Dept Orthopaed,Inst Clin Sci, Gothenburg, Sweden.
   [Nilsson-Helander, Katarina] Kungsbacka Hosp, Dept Orthopaed, Kungsbacka, Sweden.
RP Silbernagel, KG (通讯作者)，Univ Gothenburg, Sahlgrenska Univ Hosp, Sahlgrenska Acad, Dept Orthopaed,Inst Clin Sci, Gothenburg, Sweden.
EM karin.gravare-silbernagel@orthop.gu.se
RI Helander, Katarina Nilsson/AAE-1793-2020
OI Helander, Katarina Nilsson/0000-0002-1292-5102; Gravare Silbernagel,
   Karin/0000-0001-7566-407X
FU Swedish National Centre for Research in Sports (CIF); local Research and
   Development Council of Halland, Sweden; Renee Eanders Foundation, Sweden
FX We thank physical therapist Annelie Brorsson for assistance with the
   evaluations. This study was supported by grants from the Swedish
   National Centre for Research in Sports (CIF), the local Research and
   Development Council of Halland, Sweden and Renee Eanders Foundation,
   Sweden.
CR Bhandari M, 2002, CLIN ORTHOP RELAT R, P190
   Crawford J, 2004, COCHRANE DB SYST REV, DOI DOI 10.1002/14651858.CD003674.PUB2
   Don R, 2007, CLIN BIOMECH, V22, P211, DOI 10.1016/j.clinbiomech.2006.10.001
   EKSTRAND J, 1982, ARCH PHYS MED REHAB, V63, P171
   HAGGMARK T, 1986, ORTHOPEDICS, V9, P160
   Houshian S, 1998, INJURY, V29, P651, DOI 10.1016/S0020-1383(98)00147-8
   Jarvinen TAH, 2001, CURR OPIN RHEUMATOL, V13, P150
   Jozsa L G., 1997, HUMAN TENDONS ANATOM
   Kangas J, 2003, J TRAUMA, V54, P1171, DOI 10.1097/01.TA.0000047945.20863.A2
   Kangas J, 2007, AM J SPORT MED, V35, P59, DOI 10.1177/0363546506293255
   Khan RJK, 2005, J BONE JOINT SURG AM, V87A, P2202, DOI 10.2106/JBJS.D.03049
   Kocher MS, 2002, AM J SPORT MED, V30, P783, DOI 10.1177/03635465020300060501
   LEADBETTER WB, 1992, CLIN SPORT MED, V11, P533
   Leppilahti J, 1998, CLIN ORTHOP RELAT R, P152
   Lo IKY, 1997, CLIN J SPORT MED, V7, P207, DOI 10.1097/00042752-199707000-00010
   Maffulli N, 2000, AM J SPORT MED, V28, P499, DOI 10.1177/03635465000280040901
   Moller M, 2001, J BONE JOINT SURG BR, V83B, P843
   Moller M, 2005, KNEE SURG SPORT TR A, V13, P60, DOI 10.1007/s00167-003-0441-0
   Moller M, 2002, SCAND J MED SCI SPOR, V12, P9, DOI 10.1034/j.1600-0838.2002.120103.x
   Mortensen NHM, 1999, J BONE JOINT SURG AM, V81A, P983, DOI 10.2106/00004623-199907000-00011
   MORTENSEN NHM, 1992, ORTHOPEDICS, V15, P899
   Movin Tomas, 2005, Foot Ankle Clin, V10, P331, DOI 10.1016/j.fcl.2005.01.003
   Mullaney MJ, 2006, AM J SPORT MED, V34, P1120, DOI 10.1177/0363546505284186
   Nilsson-Helander K, 2008, KNEE SURG SPORT TR A, V16, P614, DOI 10.1007/s00167-008-0492-3
   Nilsson-Helander K, 2007, AM J SPORT MED, V35, P421, DOI 10.1177/0363546506294856
   NISTOR L, 1981, J BONE JOINT SURG AM, V63, P394, DOI 10.2106/00004623-198163030-00012
   NORKIN CC, 1985, MEASUREMENT JOINT MO
   Schepull T, 2007, BMC MUSCULOSKEL DIS, V8, DOI 10.1186/1471-2474-8-116
   Silbernagel KG, 2006, KNEE SURG SPORT TR A, V14, P1207, DOI 10.1007/s00167-006-0150-6
   Suchak AA, 2005, FOOT ANKLE INT, V26, P932, DOI 10.1177/107110070502601106
   Suchak AA, 2008, J BONE JOINT SURG AM, V90A, P1876, DOI 10.2106/JBJS.G.01242
   Suchak AA, 2006, CLIN ORTHOP RELAT R, P216, DOI 10.1097/01.blo.0000203458.05135.74
   Svantesson U, 1998, SCAND J REHABIL MED, V30, P67, DOI 10.1080/003655098444156
   Weber M, 2003, AM J SPORT MED, V31, P685, DOI 10.1177/03635465030310050901
   Young Jonathan S, 2005, Foot Ankle Clin, V10, P371, DOI 10.1016/j.fcl.2005.01.004
NR 35
TC 107
Z9 107
U1 0
U2 13
PU SPRINGER
PI NEW YORK
PA 233 SPRING ST, NEW YORK, NY 10013 USA
SN 0942-2056
EI 1433-7347
J9 KNEE SURG SPORT TR A
JI Knee Surg. Sports Traumatol. Arthrosc.
PD FEB
PY 2010
VL 18
IS 2
BP 258
EP 264
DI 10.1007/s00167-009-0889-7
PG 7
WC Orthopedics; Sport Sciences; Surgery
WE Science Citation Index Expanded (SCI-EXPANDED)
SC Orthopedics; Sport Sciences; Surgery
GA 546ZI
UT WOS:000273853900021
PM 19690833
DA 2022-07-22
ER

PT J
AU Kosanovic, M
   Brilej, D
AF Kosanovic, Milos
   Brilej, Drago
TI Chronic rupture of Achilles tendon: is the percutaneous suture technique
   effective?
SO ARCHIVES OF ORTHOPAEDIC AND TRAUMA SURGERY
LA English
DT Article
DE achilles tendon; rupture; chronic; surgery
ID SURAL NERVE; REPAIR; AUGMENTATION; STRENGTH
AB Background We report the long-term results of the surgical treatment of chronic rupture of the Achilles tendon using percutaneous suturing under local anesthesia.
   Patients and methods We operated on 22 patients with median age of 50 years (29-72) with chronic rupture of Achilles tendon between 1991 and 2005. The median time from injury to surgery was 7.1 weeks (4-40). We used percutaneous surgical technique similar to a technique described by Ma and Griffith (1977, Clin Orthop Relat Res 128:247-255) and Kosanovic (1994, Arch Orthop Trauma Surgery 113:177-179). Eighteen (82%) patients attended the clinical review at a mean of 67 months (14-176). During follow up, patients were asked about pain, stiffness, weakness of the calf, footwear restrictions, occupation and level of activity before and after injury (Tegner score), influence of injury on ADL activities and satisfaction with treatment. The passive and active range of movement of the ankle and the power of isometric plantar flexion were measured, and the endurance test was performed. Functional assessment was performed using a Leppilahti clinical scoring scale and a modified Merkel score
   Results Complete healing of the tendon was achieved in 21 patients (95%). In 17 patients (77%) the postoperative course was complications free. There were no reruptures during the observation period. All were able to stand on the tiptoe of the injured leg. The operated leg had a mean of 87.5% of the isometric power compared with the uninjured leg (p = NS). On an average they could stand on toes and raise the heel 13 times on side with the ruptured tendon. The Leppilahti scoring scale revealed a result that was excellent for 11 patients (62%), good for 2 (11%), fair for 5 (28%) and no one had poor result. The average score was 83.3 (60-100). Eighty-three percent of patients stated that the result of surgery was very good and 11% rated it as good. Fourteen patients (78%) returned to same level of activity at median 7 months after surgery.
   Conclusions Our series is one of the largest to be reported for the treatment of chronic rupture. Our technique offers a considerable advantage; it is minimal invasive, easy to perform with no associated harvesting morbidity and increased patient acceptance. We recommend this technique for the treatment of chronic rupture of Achilles tendon.
C1 [Kosanovic, Milos; Brilej, Drago] Gen & Teaching Hosp Celje, Dept Traumatol, Celje 3000, Slovenia.
RP Brilej, D (通讯作者)，Gen & Teaching Hosp Celje, Dept Traumatol, Oblakova 5, Celje 3000, Slovenia.
EM Drago.Brilej@guest.arnes.si
CR Cretnik A, 2000, MED SCI SPORT EXER, V32, P16
   Cretnik Andrej, 2004, J Foot Ankle Surg, V43, P72, DOI 10.1053/j.jfas.2004.01.008
   GERDES MH, 1992, CLIN ORTHOP RELAT R, V280, P241
   Jennings AG, 2002, J BONE JOINT SURG BR, V84B, P361, DOI 10.1302/0301-620X.84B3.11559
   KOSANOVIC M, 1994, ARCH ORTHOP TRAUM SU, V113, P177, DOI 10.1007/BF00441827
   Leslie Hamish D H, 2005, Foot Ankle Clin, V10, P357, DOI 10.1016/j.fcl.2005.01.009
   MA GWC, 1977, CLIN ORTHOP RELAT R, P247
   Maes Renaud, 2006, Acta Orthop Belg, V72, P179
   Maffulli N, 1999, J BONE JOINT SURG AM, V81A, P1019, DOI 10.2106/00004623-199907000-00017
   Majewski M, 2006, AM J SPORT MED, V34, P793, DOI 10.1177/0363546505283266
   MULLIER T, 2003, J FOOT ANKLE SURG, V9, P151
   Porter DA, 1997, FOOT ANKLE INT, V18, P557, DOI 10.1177/107110079701800905
   Thermann H, 2002, FOOT ANKLE INT, V23, P606, DOI 10.1177/107110070202300704
   Webb J, 2000, FOOT ANKLE INT, V21, P475, DOI 10.1177/107110070002100604
   Wilcox DK, 2000, FOOT ANKLE INT, V21, P1004, DOI 10.1177/107110070002101204
   Yasuda T, 2000, ACTA ORTHOP SCAND, V71, P69, DOI 10.1080/00016470052943937
   Yasuda T, 2007, AM J SPORT MED, V35, P582, DOI 10.1177/0363546506295939
NR 17
TC 13
Z9 17
U1 0
U2 0
PU SPRINGER
PI NEW YORK
PA 233 SPRING ST, NEW YORK, NY 10013 USA
SN 0936-8051
EI 1434-3916
J9 ARCH ORTHOP TRAUM SU
JI Arch. Orthop. Trauma Surg.
PD FEB
PY 2008
VL 128
IS 2
BP 211
EP 216
DI 10.1007/s00402-007-0514-5
PG 6
WC Orthopedics; Surgery
WE Science Citation Index Expanded (SCI-EXPANDED)
SC Orthopedics; Surgery
GA 253TA
UT WOS:000252539300016
PM 18057945
DA 2022-07-22
ER

PT J
AU Ecker, TM
   Bremer, AK
   Krause, FG
   Muller, T
   Weber, M
AF Ecker, Timo M.
   Bremer, Anne K.
   Krause, Fabian G.
   Mueller, Thorsten
   Weber, Martin
TI Prospective Use of a Standardized Nonoperative Early Weightbearing
   Protocol for Achilles Tendon Rupture: 17 Years of Experience
SO AMERICAN JOURNAL OF SPORTS MEDICINE
LA English
DT Article
DE foot; Achilles tendon; rupture; biology of tendon
ID NONSURGICAL TREATMENT; CONTROLLED-TRIALS; WEIGHT-BEARING;
   IMMOBILIZATION; REHABILITATION; MOBILIZATION; METAANALYSIS; ATROPHY;
   SURGERY; RABBITS
AB Background: Acute traumatic rupture of the Achilles tendon can be treated operatively or nonoperatively. Throughout the literature, there is no consensus regarding the optimal treatment protocol.
   Purpose: To report on 17 years of experience with treating this injury with a standardized nonoperative treatment protocol.
   Study Design: Case Series; Level of evidence, 4.
   Methods: The treatment protocol was based on a combination of an equinus cast and rehabilitation boot, which promoted immediate full weightbearing and early functional rehabilitation. A total of 171 patients were consecutively treated and prospectively followed from 1996 to 2013. Assessed were subjective parameters such as pain, loss of strength, return to previous activity level, meteosensitivity, and general satisfaction with the treatment outcome. Clinical assessment included testing of plantar flexion strength and endurance, calf circumference, and tendon length. Subjective and clinical parameters were then used to calculate a modified Thermann score. The correlation between tendon lengthening and function was calculated using the Pearson correlation coefficient.
   Results: A total of 114 patients were followed for a minimum of 12 months (mean, 27 20 months; range, 12-88 months). The mean Thermann score was 82 +/- 13 (range, 41-100), and subjective satisfaction was rated very good and good in 90%. An inverse correlation was found between tendon length and muscle strength (R = -0.3). There were 11 reruptures (8 with and 3 without an adequate trauma). General complications were 5 deep venous thromboses, 1 complex regional pain syndrome, and minor problems such as transient heel pain (n = 3), heel numbness (n = 1), and cast-associated skin abrasions (n = 4).
   Conclusion: Seventeen years of experience with a nonoperative treatment protocol for acute rupture of the Achilles tendon confirmed good functional outcome and patient satisfaction. Reruptures mostly occurred with new traumatic events in the vulnerable phase from 6 to 12 weeks after the initial injury. Muscle strength correlated to tendon length, making its assessment a crucial follow-up parameter. The protective equinus cast and boot can protect against excessive tendon lengthening during the healing process.
C1 Univ Bern, Inselspital, CH-3010 Bern, Switzerland.
   Zieglerspital Bern, Bern, Switzerland.
   [Bremer, Anne K.; Weber, Martin] Siloah Hosp, Dept Orthopaed Surg, CH-3027 Bern, Switzerland.
   [Ecker, Timo M.; Krause, Fabian G.; Mueller, Thorsten] Univ Bern, Inselspital, Dept Orthopaed Surg, CH-3010 Bern, Switzerland.
RP Weber, M (通讯作者)，Siloah Hosp, Dept Orthopaed Surg, CH-3027 Bern, Switzerland.
EM martin.weber@siloah.ch
OI Ecker, Timo Michael/0000-0002-5909-2101
CR Barfod KW, 2014, J BONE JOINT SURG AM, V96A, P1497, DOI 10.2106/JBJS.M.01273
   BROWN TD, 1981, J TRAUMA, V21, P951, DOI 10.1097/00005373-198111000-00007
   Carmont MR, 2013, FOOT ANKLE SURG, V19, P245, DOI 10.1016/j.fas.2013.06.007
   CARTER TR, 1992, AM J SPORT MED, V20, P459, DOI 10.1177/036354659202000417
   CETTI R, 1993, AM J SPORT MED, V21, P791, DOI 10.1177/036354659302100606
   Costa ML, 2006, J BONE JOINT SURG BR, V88B, P69, DOI 10.1302/0301-620X.88B1.16549
   ENWEMEKA CS, 1988, AM J PHYS MED REHAB, V67, P264
   Gwynne-Jones DP, 2011, FOOT ANKLE INT, V32, P337, DOI 10.3113/FAI.2011.0337
   Healy B, 2010, J BONE JOINT SURG BR, V92B, P646, DOI 10.1302/0301-620X.92B5.23241
   Holm C, 2015, SCAND J MED SCI SPOR, V25, pE1, DOI 10.1111/sms.12209
   Keating JF, 2011, J BONE JOINT SURG BR, V93B, P1071, DOI 10.1302/0301-620X.93B8.25998
   Khan RJK, 2010, COCHRANE DB SYST REV, DOI 10.1002/14651858.CD003674.pub4
   Khan RJK, 2005, J BONE JOINT SURG AM, V87A, P2202, DOI 10.2106/JBJS.D.03049
   Kocher MS, 2002, AM J SPORT MED, V30, P783, DOI 10.1177/03635465020300060501
   McComis GP, 1997, J BONE JOINT SURG AM, V79A, P1799, DOI 10.2106/00004623-199712000-00005
   Moller M, 2001, J BONE JOINT SURG BR, V83B, P843
   Mortensen NHM, 1999, J BONE JOINT SURG AM, V81A, P983, DOI 10.2106/00004623-199907000-00011
   Neumayer F, 2010, ARCH ORTHOP TRAUM SU, V130, P363, DOI 10.1007/s00402-009-0865-1
   Nilsson-Helander K, 2009, KNEE SURG SPORT TR A, V17, P1234, DOI 10.1007/s00167-009-0727-y
   NYSTROM B, 1983, ACTA ORTHOP SCAND, V54, P620, DOI 10.3109/17453678308992899
   Olsson N, 2014, SCAND J MED SCI SPOR, V24, P152, DOI 10.1111/j.1600-0838.2012.01497.x
   Olsson N, 2014, AM J SPORT MED, V42, P1448, DOI 10.1177/0363546514527409
   Olsson N, 2013, AM J SPORT MED, V41, P2867, DOI 10.1177/0363546513503282
   Patel A, 2012, CLIN ORTHOP RELAT R, V470, P270, DOI 10.1007/s11999-011-2166-6
   PEACOCK EE, 1965, SURG CLIN N AM, V45, P461
   Qin L, 1997, ARCH PHYS MED REHAB, V78, P512, DOI 10.1016/S0003-9993(97)90166-0
   Rantanen J, 1999, SCAND J MED SCI SPOR, V9, P57
   SALEH M, 1992, J BONE JOINT SURG BR, V74, P206, DOI 10.1302/0301-620X.74B2.1544953
   Silbernagel KG, 2012, AM J SPORT MED, V40, P1564, DOI 10.1177/0363546512447926
   Soroceanu A, 2012, J BONE JOINT SURG AM, V94A, P2136, DOI 10.2106/JBJS.K.00917
   Thermann H, 2001, FOOT ANKLE INT, V22, P478, DOI 10.1177/107110070102200604
   Thermann H, 1995, UNFALLCHIRURG, V98, P507
   Thermann H, 2000, ORTHOPADE, V29, P235
   Twaddle BC, 2007, AM J SPORT MED, V35, P2033, DOI 10.1177/0363546507307503
   VIIDIK A, 1969, ACTA ORTHOP SCAND, V40, P261, DOI 10.3109/17453676908989506
   Weber M, 2003, AM J SPORT MED, V31, P685, DOI 10.1177/03635465030310050901
   Willits K, 2010, J BONE JOINT SURG AM, V92A, P2767, DOI 10.2106/JBJS.I.01401
NR 37
TC 31
Z9 31
U1 0
U2 17
PU SAGE PUBLICATIONS INC
PI THOUSAND OAKS
PA 2455 TELLER RD, THOUSAND OAKS, CA 91320 USA
SN 0363-5465
EI 1552-3365
J9 AM J SPORT MED
JI Am. J. Sports Med.
PD APR
PY 2016
VL 44
IS 4
BP 1004
EP 1010
DI 10.1177/0363546515623501
PG 7
WC Orthopedics; Sport Sciences
WE Science Citation Index Expanded (SCI-EXPANDED)
SC Orthopedics; Sport Sciences
GA DI7FX
UT WOS:000373665800029
PM 26818449
DA 2022-07-22
ER

PT J
AU Stavenuiter, XJR
   Lubberts, B
   Prince, RM
   Johnson, AH
   DiGiovanni, CW
   Guss, D
AF Stavenuiter, X. J. Ruben
   Lubberts, Bart
   Prince, Robert M., III
   Johnson, A. Holly
   DiGiovanni, Christopher W.
   Guss, Daniel
TI Postoperative Complications Following Repair of Acute Achilles Tendon
   Rupture
SO FOOT & ANKLE INTERNATIONAL
LA English
DT Article
DE Achilles tendon; acute; rupture; surgery; complications
ID VENOUS THROMBOEMBOLISM; NONSURGICAL TREATMENT; WOUND COMPLICATIONS; CAST
   IMMOBILIZATION; ANKLE SURGERY; FOOT; RISK; METAANALYSIS; PREVENTION;
   SYSTEM
AB Background: Controversy remains regarding which patients with acute Achilles tendon rupture would best be treated nonoperatively and which might benefit from operative repair. The primary aim of this study was to characterize the overall incidence of-and specific risk factors associated with-postoperative complications that follow operative repair. We also evaluated the specific differences between complications after the use of an open or minimally invasive surgical (MIS) approach. Methods: Retrospective chart review identified 615 adult patients who underwent operative repair for an acute Achilles tendon rupture between January 1, 2001, and May 1, 2016, at 3 level I trauma centers. Minimum follow-up was 3 months. Patient demographics, comorbidities, injury mechanism, procedural details, and surgeon subspecialty were collected. Assessed complications included wound healing issues, rerupture, hematoma, nerve injury, deep vein thrombosis, and pulmonary embolism. Results: Seventy-two patients (11.7%) developed a postoperative complication. Risk factors included advancing patient age (odds ratio [OR], 1.04, P = .007), active tobacco use (OR, 3.20, P = .007), and specific subspecialty training (OR, 2.04, P = .046). No difference in overall complication rate was found between the open and MIS approaches (11.6% vs 13.2%, P = .658). A subgroup analysis among orthopedic subspecialties demonstrated that patients treated by trauma surgeons had increased rates of wound complication (P = .043) and rerupture (P = .025) compared with those treated by other subspecialties. Patients treated by trauma surgeons were also more likely to be younger or have a body mass index (BMI) > 30, although neither factor was found to be independently predictive for postoperative complications. Conclusion: Approximately 1 in 9 patients undergoing operative repair of an acute Achilles tendon rupture developed a postoperative complication. Advancing age and active tobacco use were independent risk factors for developing such complications. Differences in subspecialty training also appear to impact complication rates, but the potential reason for this discrepancy remains unclear. As controversy remains regarding which patients who sustain acute Achilles tendon rupture should be treated nonoperatively and which would benefit most from surgical repair, a better understanding of postoperative complication rates and associated risk factors may enhance the decision-making processes in treating these injuries. It is not clear whether MIS techniques are superior to traditional open repair in terms of postoperative complications.
C1 [Stavenuiter, X. J. Ruben; Lubberts, Bart; Prince, Robert M., III] Harvard Med Sch, Massachusetts Gen Hosp, Orthopaed Foot & Ankle Serv, Boston, MA 02115 USA.
   [Johnson, A. Holly; DiGiovanni, Christopher W.; Guss, Daniel] Harvard Med Sch, Massachusetts Gen Hosp, Newton Wellesley Hosp, Orthopaed Foot & Ankle Serv, 55 Fruit St, Boston, MA 02114 USA.
RP Guss, D (通讯作者)，Harvard Med Sch, Massachusetts Gen Hosp, Newton Wellesley Hosp, Orthopaed Foot & Ankle Serv, 55 Fruit St, Boston, MA 02114 USA.
EM dguss@partners.org
CR Aktas S, 2009, FOOT ANKLE INT, V30, P391, DOI 10.3113/FAI.2009.0391
   Assal M, 2002, J BONE JOINT SURG AM, V84A, P161, DOI 10.2106/00004623-200202000-00001
   Bartel AFP, 2014, J FOOT ANKLE SURG, V53, P744, DOI 10.1053/j.jfas.2014.07.009
   Bhandari M, 2002, CLIN ORTHOP RELAT R, P190
   Bruggeman NB, 2004, CLIN ORTHOP RELAT R, P63, DOI 10.1097/01.blo.0000144475.05543.e7
   Burrus M Tyrrell, 2016, Foot Ankle Spec, V9, P208, DOI 10.1177/1938640015609983
   Calder JDF, 2016, KNEE SURG SPORT TR A, V24, P1409, DOI 10.1007/s00167-015-3976-y
   Cretnik A, 2005, AM J SPORT MED, V33, P1369, DOI 10.1177/0363546504271501
   Del Buono A, 2014, BRIT MED BULL, V109, P45, DOI 10.1093/bmb/ldt029
   Fleischer AE, 2015, J FOOT ANKLE SURG, V54, P497, DOI 10.1053/j.jfas.2015.02.022
   Griffiths JT, 2012, J BONE JOINT SURG BR, V94B, P210, DOI 10.1302/0301-620X.94B2.27579
   Guss D, 2015, JBJS REV, V3
   Haji A, 2004, FOOT ANKLE INT, V25, P215, DOI 10.1177/107110070402500404
   Healy B, 2010, J BONE JOINT SURG BR, V92B, P646, DOI 10.1302/0301-620X.92B5.23241
   Henriquez H, 2012, CLIN ORTHOP RELAT R, V470, P998, DOI 10.1007/s11999-011-1830-1
   Hsu AR, 2015, FOOT ANKLE INT, V36, P1279, DOI 10.1177/1071100715589632
   Jones Matthew P, 2012, J Bone Joint Surg Am, V94, pe88, DOI 10.2106/JBJS.J.01829
   Karabinas Panagiotis K, 2014, Eur J Orthop Surg Traumatol, V24, P607, DOI 10.1007/s00590-013-1350-7
   Khan RJK, 2010, COCHRANE DB SYST REV, DOI 10.1002/14651858.CD003674.pub4
   KOCK HJ, 1995, LANCET, V346, P459, DOI 10.1016/S0140-6736(95)91320-3
   Lansdaal JR, 2007, INJURY, V38, P839, DOI 10.1016/j.injury.2006.12.010
   Lapidus LJ, 2013, BMC MUSCULOSKEL DIS, V14, DOI 10.1186/1471-2474-14-177
   Lim J, 2001, FOOT ANKLE INT, V22, P559, DOI 10.1177/107110070102200705
   MA GWC, 1977, CLIN ORTHOP RELAT R, P247
   Nilsson-Helander K, 2009, KNEE SURG SPORT TR A, V17, P1234, DOI 10.1007/s00167-009-0727-y
   O'Brien RM, 2007, QUAL QUANT, V41, P673, DOI 10.1007/s11135-006-9018-6
   Olsson N, 2013, AM J SPORT MED, V41, P2867, DOI 10.1177/0363546513503282
   Patel A, 2012, CLIN ORTHOP RELAT R, V470, P270, DOI 10.1007/s11999-011-2166-6
   Pelet S, 2012, J BONE JOINT SURG AM, V94A, P502, DOI 10.2106/JBJS.J.01190
   Reis FP, 2008, SURG RADIOL ANAT, V30, P341, DOI 10.1007/s00276-008-0333-6
   Saragas Nikiforos Pandelis, 2011, Foot Ankle Surg, V17, P263, DOI 10.1016/j.fas.2010.12.002
   Saxena A, 2008, J AM PODIAT MED ASSN, V98, P95
   Soroceanu A, 2012, J BONE JOINT SURG AM, V94A, P2136, DOI 10.2106/JBJS.K.00917
   Strauss EJ, 2007, INJURY, V38, P832, DOI 10.1016/j.injury.2006.06.005
   Twaddle BC, 2007, AM J SPORT MED, V35, P2033, DOI 10.1177/0363546507307503
   Wallace RGH, 2004, J BONE JOINT SURG AM, V86A, P1198, DOI 10.2106/00004623-200406000-00011
   Webb JM, 1999, J BONE JOINT SURG BR, V81B, P877, DOI 10.1302/0301-620X.81B5.9784
   Wiewiorski M, 2015, FOOT ANKLE INT, V36, P479, DOI 10.1177/1071100714565792
   Willits K, 2010, J BONE JOINT SURG AM, V92A, P2767, DOI 10.2106/JBJS.I.01401
NR 39
TC 19
Z9 22
U1 0
U2 4
PU SAGE PUBLICATIONS INC
PI THOUSAND OAKS
PA 2455 TELLER RD, THOUSAND OAKS, CA 91320 USA
SN 1071-1007
EI 1944-7876
J9 FOOT ANKLE INT
JI Foot Ankle Int.
PD JUN
PY 2019
VL 40
IS 6
BP 679
EP 686
DI 10.1177/1071100719831371
PG 8
WC Orthopedics
WE Science Citation Index Expanded (SCI-EXPANDED)
SC Orthopedics
GA IC2CD
UT WOS:000470766300010
PM 30808187
DA 2022-07-22
ER

PT J
AU Elias, I
   Besser, M
   Nazarian, LN
   Raikin, SM
AF Elias, Ilan
   Besser, Marcus
   Nazarian, Levon N.
   Raikin, Steven M.
TI Reconstruction for missed or neglected Achilles tendon rupture with V-Y
   lengthening and flexor hallucis longus tendon transfer through one
   incision
SO FOOT & ANKLE INTERNATIONAL
LA English
DT Article
DE Achilles tendon rupture; dynamometry; flexor hallucis longus; retracted
   tendon; single incision technique; V-Y lengthening
ID REPAIR; RELIABILITY; TORQUE; TENDINOSIS; INJURY
AB Background. The purpose of this study was to introduce a novel operative technique and to evaluate the clinical outcomes in a cohort of patients with missed or neglected Achilles tendon ruptures. Methods: Fifteen consecutive patients with missed complete Achilles tendon ruptures and 5-cm or larger gaps had reconstruction with V-Y lengthening and flexor hallucis longus tendon transfer through a single incision. The patients were evaluated at an average of 106 weeks after surgery. At the time of followup, all patients were assessed with regard to their self-reported level of satisfaction and the American Orthopaedic Foot and Ankle Society (AOFAS) Ankle-Hindfoot Scale. Ankle strength and active range of motion were evaluated using Biodex (R) (Biodex Medical Systems, Shirley, NY) isokinetic dynamometry. In addition, seven patients were evaluated using diagnostic ultrasound. Results: We found a 7.7 N-m (-22.3%) loss of plantarflexion torque at 60 degrees/sec and a 3.5 N-m (-13.5%) loss of plantarflexion torque at 120 degrees/sec, as well as a 5 degrees loss of active range of motion. AOFAS scores were all good to excellent, with an average score of 94.1 of 100. All patients were satisfied with their outcomes (rated good or very good). Excellent exposure of the Achilles tendon repair was obtained with ultrasound. Conclusions: For patients with missed or neglected Achilles tendon rupture with a rupture gap of at least 5 cm, operative repair using V-Y lengthening and flexor hallucis longus tendon transfer through a single incision technique achieved a high percentage of satisfactory results.
C1 [Raikin, Steven M.] Thomas Jefferson Univ, Dept Orthopaed Surg, Rothman Inst, Philadelphia, PA 19107 USA.
RP Raikin, SM (通讯作者)，Thomas Jefferson Univ, Dept Orthopaed Surg, Rothman Inst, 925 Chestnut St, Philadelphia, PA 19107 USA.
EM steven.raikin@rothmaninstitute.com
CR ABRAHAM E, 1975, J BONE JOINT SURG AM, VA 57, P253, DOI 10.2106/00004623-197557020-00019
   Andersen H, 1996, ARCH PHYS MED REHAB, V77, P265, DOI 10.1016/S0003-9993(96)90109-4
   BAUMHAUER JF, 1995, AM J SPORT MED, V23, P571, DOI 10.1177/036354659502300509
   CETTI R, 1993, AM J SPORT MED, V21, P791, DOI 10.1177/036354659302100606
   Chester R, 2003, FOOT ANKLE INT, V24, P909, DOI 10.1177/107110070302401207
   Coull R, 2003, FOOT ANKLE INT, V24, P931, DOI 10.1177/107110070302401211
   Den Hartog BD, 2003, FOOT ANKLE INT, V24, P233, DOI 10.1177/107110070302400306
   Fessell David P., 1998, Semin Musculoskelet Radiol, V2, P271, DOI 10.1055/s-2008-1080107
   Hansen ST, 1991, DISORDERS FOOT ANKLE, P2357
   Hartgerink P, 2001, RADIOLOGY, V220, P406, DOI 10.1148/radiology.220.2.r01au41406
   Karjalainen PT, 1996, ACTA RADIOL, V37, P639
   Kissel C G, 1994, J Foot Ankle Surg, V33, P46
   KITAOKA HB, 1994, FOOT ANKLE INT, V15, P349, DOI 10.1177/107110079401500701
   MANN RA, 1991, J BONE JOINT SURG AM, V73A, P214, DOI 10.2106/00004623-199173020-00009
   Martin RL, 2005, FOOT ANKLE INT, V26, P691, DOI 10.1177/107110070502600905
   Monroe MT, 2000, FOOT ANKLE INT, V21, P324, DOI 10.1177/107110070002100409
   Myerson M S, 1999, Instr Course Lect, V48, P219
   SHIELDS CR, 1987, AM J SPORTS MED, V6, P369
   Takao M, 2003, ARCH ORTHOP TRAUM SU, V123, P471, DOI 10.1007/s00402-002-0443-2
   Tashjian RZ, 2003, FOOT ANKLE INT, V24, P673, DOI 10.1177/107110070302400903
   Us AK, 1997, ARCH ORTHOP TRAUM SU, V116, P408
   Wapner K L, 1993, Foot Ankle, V14, P443
   WAPNER KL, 1995, ORTHOP CLIN N AM, V26, P249
   Wapner KL, 1994, OPER TECH ORTHOP, V4, P132, DOI [10.1016/S1048-6666(06)80123-0, DOI 10.1016/S1048-6666(06)80123-0]
   Wilcox DK, 2000, FOOT ANKLE INT, V21, P1004, DOI 10.1177/107110070002101204
   Wong MWN, 2005, CLIN ORTHOP RELAT R, P201, DOI 10.1097/01.blo.0000150460.92012.7a
   WOODSON C, 1995, J ORTHOP SPORT PHYS, V22, P113, DOI 10.2519/jospt.1995.22.3.113
NR 27
TC 54
Z9 61
U1 0
U2 3
PU SAGE PUBLICATIONS INC
PI THOUSAND OAKS
PA 2455 TELLER RD, THOUSAND OAKS, CA 91320 USA
SN 1071-1007
EI 1944-7876
J9 FOOT ANKLE INT
JI Foot Ankle Int.
PD DEC
PY 2007
VL 28
IS 12
BP 1238
EP 1248
DI 10.3113/FAI.2007.1238
PG 11
WC Orthopedics
WE Science Citation Index Expanded (SCI-EXPANDED)
SC Orthopedics
GA 253DS
UT WOS:000252499500005
PM 18173986
DA 2022-07-22
ER

PT J
AU Frankewycz, B
   Krutsch, W
   Weber, J
   Ernstberger, A
   Nerlich, M
   Pfeifer, CG
AF Frankewycz, B.
   Krutsch, W.
   Weber, J.
   Ernstberger, A.
   Nerlich, M.
   Pfeifer, Christian G.
TI Rehabilitation of Achilles tendon ruptures: is early functional
   rehabilitation daily routine?
SO ARCHIVES OF ORTHOPAEDIC AND TRAUMA SURGERY
LA English
DT Article
DE Achilles tendon rupture; Non-operative treatment; Post-operative
   rehabilitation; Immobilization; Early function; Orthosis
ID RANDOMIZED CONTROLLED-TRIALS; ACCELERATED REHABILITATION; NONSURGICAL
   TREATMENT; SURGICAL REPAIR; WEIGHT-BEARING; EARLY MOBILIZATION;
   CALCANEAL TENDON; MUSCLE ATROPHY; IMMOBILIZATION; METAANALYSIS
AB Ruptures of the Achilles tendon are the most common tendon injuries of the lower extremities. Besides the initial operative or non-operative treatment, rehabilitation of patients plays a crucial role for tendon healing and long-term outcome. As only limited evidence is available for optimized rehabilitation regimen and guidelines for the initial (e.g., first 6 weeks) rehabilitation are limited, this study investigated the current rehabilitation concepts after Achilles tendon rupture.
   We analyzed 213 written rehabilitation protocols that are provided by orthopedic and trauma surgery institutions throughout Germany in terms of recommendations for weight-bearing, range of motion (ROM), physiotherapy, and choice of orthosis. All protocols for operatively and non-operatively treated Achilles tendon ruptures were included. Descriptive analysis was carried out and statistical analysis applied where appropriate.
   Of 213 institutions, 204 offered rehabilitation protocols for Achilles tendon rupture and, therefore, 243 protocols for operative and non-operative treatment could be analyzed. While the majority of protocols allowed increased weight-bearing over time, significant differences were found for durations of fixed plantar flexion between operative (o) and non-operative (n) treatments [fixed 30A degrees (or 20)A degrees to 15A degrees (or 10)A degrees: 3.6 weeks (+/- 0.1; o) vs 4.7 weeks (+/- 0.3; n) (p <= 0.0001) and fixed 15A degrees (or 10)A degrees to 0A degrees: 5.8 weeks (+/- 0.1; o) vs 6.6 weeks (+/- 0.2; n) (p <= 0.001)]. The mean time of the recommended start of physiotherapy is at 2.9 weeks (+/- 0.2; o) vs 3.3 weeks (+/- 0.4; n), respectively.
   Our study shows that a huge variability in rehabilitation after Achilles tendon rupture exists. This study shows different strategies in rehabilitation of Achilles tendon ruptures using a convertible vacuum brace system. To improve patient care, further clinical as well as biomechanical studies need to be conducted. This study might serve as basis for prospective randomized controlled trials to optimize rehabilitation for Achilles tendon ruptures.
C1 [Frankewycz, B.; Krutsch, W.; Weber, J.; Ernstberger, A.; Nerlich, M.; Pfeifer, Christian G.] Univ Med Ctr Regensburg, Dept Trauma & Orthoped Surg, FIFA Med Ctr Excellence, D-93053 Regensburg, Germany.
RP Pfeifer, CG (通讯作者)，Univ Med Ctr Regensburg, Dept Trauma & Orthoped Surg, FIFA Med Ctr Excellence, Franz Josef Strauss Allee 11, D-93053 Regensburg, Germany.
EM christian.pfeifer@ukr.de
CR Brumann M, 2014, INJURY, V45, P1782, DOI 10.1016/j.injury.2014.06.022
   CARDEN DG, 1987, J BONE JOINT SURG BR, V69, P416, DOI 10.1302/0301-620X.69B3.3294839
   CARTER TR, 1992, AM J SPORT MED, V20, P459, DOI 10.1177/036354659202000417
   Claessen FMAP, 2014, SPORTS MED, V44, P1241, DOI 10.1007/s40279-014-0200-z
   Costa ML, 2006, J BONE JOINT SURG BR, V88B, P69, DOI 10.1302/0301-620X.88B1.16549
   Crawford J, 2004, COCHRANE DB SYST REV, DOI DOI 10.1002/14651858.CD003674.PUB2
   Ecker TM, 2016, AM J SPORT MED, V44, P1004, DOI 10.1177/0363546515623501
   Eyfferth T, 2011, TRAUMA BERUFSKRANKH, V13, P183, DOI 10.1007/s10039-011-1751-y
   GILLIES H, 1970, J BONE JOINT SURG AM, VA 52, P337, DOI 10.2106/00004623-197052020-00015
   Groetelaers RPTGC, 2014, FOOT ANKLE INT, V35, P771, DOI 10.1177/1071100714536167
   Gruber J, 2013, REHABIL POSTSURGICAL, P554
   Haggmark T, 1979, Am J Sports Med, V7, P121, DOI 10.1177/036354657900700208
   Holm C, 2015, SCAND J MED SCI SPOR, V25, pE1, DOI 10.1111/sms.12209
   Huang JZ, 2015, AM J SPORT MED, V43, P1008, DOI 10.1177/0363546514531014
   Jackson G, 2013, ORTHOPEDICS, V36, pE1053, DOI 10.3928/01477447-20130724-23
   Jiang N, 2012, INT ORTHOP, V36, P765, DOI 10.1007/s00264-011-1431-3
   Jielile J, 2016, KNEE SURG SPORT TR A, V24, P2148, DOI 10.1007/s00167-015-3598-4
   Kangas J, 2003, J TRAUMA, V54, P1171, DOI 10.1097/01.TA.0000047945.20863.A2
   Kearney RS, 2012, PHYSIOTHERAPY, V98, P24, DOI 10.1016/j.physio.2011.04.349
   Khan K, 2003, CLIN SPORT MED, V22, P711, DOI 10.1016/S0278-5919(03)00035-8
   Khan RJK, 2005, J BONE JOINT SURG AM, V87A, P2202, DOI 10.2106/JBJS.D.03049
   LEA RB, 1972, J BONE JOINT SURG AM, VA 54, P1398, DOI 10.2106/00004623-197254070-00002
   Lopes AD, 2012, SPORTS MED, V42, P891, DOI [10.2165/11631170-000000000-00000, 10.1007/BF03262301]
   Maffulli N, 2003, CLIN SPORT MED, V22, P675, DOI 10.1016/S0278-5919(03)00004-8
   Mark-Christensen T, 2016, KNEE SURG SPORT TR A, V24, P1852, DOI 10.1007/s00167-014-3180-5
   MAXWELL LC, 1992, ANAT REC, V234, P55, DOI 10.1002/ar.1092340107
   McCormack R, 2015, BRIT J SPORT MED, V49, P1329, DOI 10.1136/bjsports-2015-094935
   Mortensen NHM, 1999, J BONE JOINT SURG AM, V81A, P983, DOI 10.2106/00004623-199907000-00011
   Nilsson-Helander K, 2010, AM J SPORT MED, V38, P2186, DOI 10.1177/0363546510376052
   Olsson N, 2013, AM J SPORT MED, V41, P2867, DOI 10.1177/0363546513503282
   Osarumwense D, 2013, J ORTHOP SURG-HONG K, V21, P44, DOI 10.1177/230949901302100112
   Palmes D, 2002, J ORTHOPAED RES, V20, P939, DOI 10.1016/S0736-0266(02)00032-3
   Porter DA, 2014, FOOT ANKLE INT, V35, P123, DOI 10.1177/1071100713514228
   Porter MD, 2015, ANZ J SURG, V85, P373, DOI 10.1111/ans.12910
   Rantanen J, 1999, SCAND J MED SCI SPOR, V9, P57
   RANTANEN J, 1993, ACTA ORTHOP SCAND, V64, P333, DOI 10.3109/17453679308993638
   Riley G, 2004, RHEUMATOLOGY, V43, P131, DOI 10.1093/rheumatology/keg448
   SALEH M, 1992, J BONE JOINT SURG BR, V74, P206, DOI 10.1302/0301-620X.74B2.1544953
   SMART GW, 1980, MED SCI SPORT EXER, V12, P231
   Soroceanu A, 2012, J BONE JOINT SURG AM, V94A, P2136, DOI 10.2106/JBJS.K.00917
   Sorrenti SJ, 2006, FOOT ANKLE INT, V27, P407, DOI 10.1177/107110070602700603
   Stockle U, 2000, UNFALLCHIRURG, V103, P215, DOI 10.1007/s001130050525
   Suchak AA, 2006, CLIN ORTHOP RELAT R, P216, DOI 10.1097/01.blo.0000203458.05135.74
   Thermann H, 1998, UNFALLCHIRURG, V101, P299, DOI 10.1007/s001130050273
   Weinreb Jeffrey H, 2014, Muscles Ligaments Tendons J, V4, P66
   WILLIAMS JGP, 1986, SPORTS MED, V3, P114, DOI 10.2165/00007256-198603020-00003
   WILLS CA, 1986, CLIN ORTHOP RELAT R, P156
   Young SW, 2014, J BONE JOINT SURG AM, V96A, P1073, DOI 10.2106/JBJS.M.00248
NR 48
TC 23
Z9 25
U1 2
U2 17
PU SPRINGER
PI NEW YORK
PA 233 SPRING ST, NEW YORK, NY 10013 USA
SN 0936-8051
EI 1434-3916
J9 ARCH ORTHOP TRAUM SU
JI Arch. Orthop. Trauma Surg.
PD MAR
PY 2017
VL 137
IS 3
BP 333
EP 340
DI 10.1007/s00402-017-2627-9
PG 8
WC Orthopedics; Surgery
WE Science Citation Index Expanded (SCI-EXPANDED)
SC Orthopedics; Surgery
GA EL2DW
UT WOS:000394431300005
PM 28097423
DA 2022-07-22
ER

PT J
AU Hodgens, BH
   Geller, JS
   Rizzo, MG
   Munoz, J
   Kaplan, J
   Aiyer, A
AF Hodgens, Blake H.
   Geller, Joseph S.
   Rizzo, Michael G.
   Munoz, Julianne
   Kaplan, Jonathan
   Aiyer, Amiethab
TI Performance Outcomes After Surgical Repair of Achilles Tendon Rupture in
   the Women's National Basketball Association
SO ORTHOPAEDIC JOURNAL OF SPORTS MEDICINE
LA English
DT Article
DE Achilles tendon rupture; Women's National Basketball Association;
   performance outcomes; basketball
ID EPIDEMIOLOGY; ESTROGEN
AB Background: Achilles tendon ruptures are devastating in elite athletes. There are currently no studies examining the effects of Achilles tendon rupture on performance outcomes in the Women's National Basketball Association (WNBA). Hypothesis: Athletes in the WNBA who sustained an Achilles tendon rupture and underwent subsequent surgical repair will sustain declines in performance metrics when compared with their preinjury statistics and matched controls. Study Design: Cohort study; Level of evidence, 3. Methods: Seventeen WNBA players who sustained an Achilles tendon rupture from 2000 to 2019 were identified through publicly available injury reports and player profiles. Athlete information collected included age, body mass index, position, and service in the WNBA when the tear occurred. Statistics were collected for 1 season before and 2 seasons after the injury, and the player efficiency rating (PER) was calculated. Players were matched to uninjured controls by service in the WNBA, position, and performance statistics. Results: On average, players were 28 years of age at the time of Achilles tendon rupture, with a service time in the WNBA of 6.5 years. Four players never returned to play in the WNBA, while 7 players failed to play more than 1 season. Players who did return played significantly fewer minutes per game compared with preinjury in both postinjury seasons 1 and 2 (mean difference, -6.11 and -6.54 min/game, respectively; P < .01 for both) and had a significantly decreased PER in postinjury season 2 (mean difference, -2.53; P = .024). After returning to play, the injured players experienced significant decreases when compared with controls in field goals (-0.85 vs +0.20; P = .047), free throws (-1.04 vs +0.12; P < .01), steals (-0.48 vs +0.24; P = .018), and points scored (-2.89 vs +0.58; P = .014). Conclusion: WNBA players experienced significant decreases in performance metrics after Achilles tendon rupture compared with their preinjury levels and compared with uninjured controls. Overall, 23.5% of players failed to return to the WNBA, while 41.2% failed to play for more than 1 season.
C1 [Hodgens, Blake H.] Univ Miami, Miller Sch Med, Miami, FL 33136 USA.
   [Geller, Joseph S.; Rizzo, Michael G.; Aiyer, Amiethab] Univ Miami, Dept Orthoped Surg, Jackson Mem Hosp, Miami, FL 33136 USA.
   [Munoz, Julianne] Univ Miami, Sports Med Inst, Miami, FL 33136 USA.
   [Kaplan, Jonathan] Hoag Orthopaed Inst, Orange, CA USA.
RP Aiyer, A (通讯作者)，Univ Miami, Jackson Mem Hosp, 1611 NW 12th Ave, Miami, FL 33136 USA.
EM tabsaiyer@gmail.com
OI Geller, Joseph/0000-0003-4213-8975
CR Amin Nirav H, 2016, Clin Res Foot Ankle, V4, DOI 10.4172/2329-910X.1000205
   Amin NH, 2013, AM J SPORT MED, V41, P1864, DOI 10.1177/0363546513490659
   Bryant AL, 2008, J APPL PHYSIOL, V105, P1035, DOI 10.1152/japplphysiol.01281.2007
   Chan JJ, 2020, INT ORTHOP, V44, P585, DOI 10.1007/s00264-019-04471-2
   Clayton RAE, 2008, INJURY, V39, P1338, DOI 10.1016/j.injury.2008.06.021
   Cukelj F, 2015, INJURY, V46, pS137, DOI 10.1016/j.injury.2015.10.070
   Deitch JR, 2006, AM J SPORT MED, V34, P1077, DOI 10.1177/0363546505285383
   FIRTH D, 1993, BIOMETRIKA, V80, P27, DOI 10.1093/biomet/80.1.27
   Gwynne-Jones DP, 2011, FOOT ANKLE INT, V32, P337, DOI 10.3113/FAI.2011.0337
   Jarvinen Tero A H, 2005, Foot Ankle Clin, V10, P255, DOI 10.1016/j.fcl.2005.01.013
   Khalil LS, 2020, ORTHOP J SPORTS MED, V8, DOI 10.1177/2325967120966041
   KOBORI M, 1989, CLIN ORTHOP RELAT R, P306
   Lemme NJ, 2019, AM J SPORT MED, V47, P2360, DOI 10.1177/0363546519858609
   Lemme NJ, 2018, ORTHOP J SPORTS MED, V6, DOI 10.1177/2325967118808238
   Maffulli N, 2000, AM J SPORT MED, V28, P857, DOI 10.1177/03635465000280061401
   Maffulli N, 2011, KNEE SURG SPORT TR A, V19, P680, DOI 10.1007/s00167-010-1193-2
   Mai HT, 2016, AM J SPORT MED, V44, P2255, DOI 10.1177/0363546516651426
   McCarthy MM, 2013, AM J SPORT MED, V41, P645, DOI 10.1177/0363546512474223
   Minhas SV, 2016, AM J SPORT MED, V44, P1056, DOI 10.1177/0363546515623028
   Muraoka T, 2005, J APPL PHYSIOL, V99, P665, DOI 10.1152/japplphysiol.00624.2004
   Namdari S, 2011, PHYSICIAN SPORTSMED, V39, P36, DOI 10.3810/psm.2011.02.1860
   Nilsson-Helander K, 2010, AM J SPORT MED, V38, P2186, DOI 10.1177/0363546510376052
   OBRIEN M, 1992, CLIN SPORT MED, V11, P505
   Parekh Selene G, 2009, Foot Ankle Spec, V2, P283, DOI 10.1177/1938640009351138
   Schepsis AA, 2002, AM J SPORT MED, V30, P287, DOI 10.1177/03635465020300022501
   Trofa DP, 2017, AM J SPORT MED, V45, P2864, DOI 10.1177/0363546517713001
   Vosseller JT, 2013, FOOT ANKLE INT, V34, P49, DOI 10.1177/1071100712460223
NR 27
TC 0
Z9 0
U1 1
U2 1
PU SAGE PUBLICATIONS INC
PI THOUSAND OAKS
PA 2455 TELLER RD, THOUSAND OAKS, CA 91320 USA
EI 2325-9671
J9 ORTHOP J SPORTS MED
JI Orthop. J. Sports Med.
PD SEP
PY 2021
VL 9
IS 9
AR 23259671211030473
DI 10.1177/23259671211030473
PG 6
WC Orthopedics; Sport Sciences
WE Science Citation Index Expanded (SCI-EXPANDED)
SC Orthopedics; Sport Sciences
GA UW8PE
UT WOS:000700414600001
PM 34527757
OA Green Published
DA 2022-07-22
ER

PT J
AU Kosaka, T
   Masaoka, T
   Yamamoto, K
AF Kosaka, T.
   Masaoka, T.
   Yamamoto, K.
TI Possible Molecular Mechanism of Promotion of Repair of Acute Achilles
   Tendon Rupture by Low Intensity-pulsed Ultrasound Treatment in a Rat
   Model
SO WEST INDIAN MEDICAL JOURNAL
LA English
DT Article
DE Achilles tendon rupture; Collagen I; Collagen III; COX2; EP4R; LIPUS
ID COLLAGEN EXPRESSION; EP4 RECEPTORS; GROWTH; OSTEOBLASTS;
   PROSTAGLANDIN-E2; STIMULATION; FRACTURES; CELLS
AB Objective: This study investigated the effect of Low Intensity-pulsed Ultrasound (LIP US) on the repair process of ruptured Achilles tendon using a rat model and also examined the regulation of a biological molecule that may contribute to this in vivo and in vitro.
   Methods: To investigate the effect of LIP US and its biological mechanism of promoting Achilles tendon repair after acute injury, ninety-eight male Sprague-Dawley (SD) rats (mean body weight, 258 +/- 9.8 g) aged 12 weeks were used in this study. To create the model, the Achilles tendon attachment site and musculotendinous junction were ruptured under direct vision. The leg on one side was exposed to LIP US (frequency at 1.5 MHz, the repetition cycle at 1.0 kHz, the burst width at 200 msec and the power output at 45 mW/cm2), for 20 minutes daily with a 0.7 mm diameter probe.
   Results: Low Intensity-pulsed Ultrasound treatment accelerated the repair of the Achilles tendon compared to the untreated group, judged by electron microscopy. Both cyclo-oxygenase (COX)-2* and EP4* expressions were over-expressed in the LIP US treated group in the inflammatory period, and TGF beta 1* expression was markedly induced in LIP US treated groups followed by collagen I* and III* expression in the repair and reconstitution process.
   Conclusion: These findings suggest that LIPUS is potentially able to accelerate the repair of acute ruptured Achilles tendon in several ways: by exaggerating inflammation by inducing COX-2 and EP4 and reconstituting tissue by inducing TGF beta 1 followed by collagen I and III. (* : p < 0.05, **: 0.001)
C1 [Kosaka, T.] Tokyo Med Univ, Dept Orthopaed Surg, Shinjuku Ku, Tokyo 1600023, Japan.
RP Kosaka, T (通讯作者)，Tokyo Med Univ, Dept Orthopaed Surg, Shinjuku Ku, 6-7-1 Nishi Shinjuku, Tokyo 1600023, Japan.
EM tikosaka@h6.dion.ne.jp
CR DUARTE LR, 1983, ARCH ORTHOP TRAUM SU, V101, P153, DOI 10.1007/BF00436764
   ENWEMEKA CS, 1990, ULTRASOUND MED BIOL, V16, P801, DOI 10.1016/0301-5629(90)90044-D
   ENWEMEKA CS, 1989, AM J PHYS MED REHAB, V68, P283, DOI 10.1097/00002060-198912000-00005
   Hatazawa R, 2007, AM J PHYSIOL-GASTR L, V293, pG788, DOI 10.1152/ajpgi.00131.2007
   HECKMAN JD, 1994, J BONE JOINT SURG AM, V76A, P26, DOI 10.2106/00004623-199401000-00004
   Ito M, 2000, ULTRASOUND MED BIOL, V26, P161, DOI 10.1016/S0301-5629(99)00110-6
   Kabashima K, 2007, LAB INVEST, V87, P49, DOI 10.1038/labinvest.3700491
   Kokubu T, 1999, BIOCHEM BIOPH RES CO, V256, P284, DOI 10.1006/bbrc.1999.0318
   KOSAKA T, 2009, JJBEPSRS, V23, P19
   Kristiansen TK, 1997, J BONE JOINT SURG AM, V79A, P961, DOI 10.2106/00004623-199707000-00002
   Li M, 2005, BONE, V37, P46, DOI 10.1016/j.bone.2005.03.016
   McCoy JM, 2002, J CLIN INVEST, V110, P651, DOI 10.1172/JCI200215528
   Naruse K, 2003, J BONE MINER RES, V18, P360, DOI 10.1359/jbmr.2003.18.2.360
   Rubin C, 2001, J BONE JOINT SURG AM, V83A, P259, DOI 10.2106/00004623-200102000-00015
   Sant'Anna EF, 2005, J ORTHOP RES, V23, P646, DOI 10.1016/j.orthres.2004.09.007
   Sugimoto Y, 2007, J BIOL CHEM, V282, P11613, DOI 10.1074/jbc.R600038200
   Tsai WC, 2007, CONNECT TISSUE RES, V48, P46, DOI 10.1080/03008200601071295
   Tsai WC, 2006, J ORTHOP RES, V24, P1310, DOI 10.1002/jor.20130
   Wang FS, 2004, BONE, V35, P114, DOI 10.1016/j.bone.2004.02.012
   Yeung CK, 2006, J ORTHOP RES, V24, P193, DOI 10.1002/jor.20020
NR 20
TC 13
Z9 13
U1 0
U2 2
PU UNIV WEST INDIES FACULTY MEDICAL SCIENCES
PI KINGSTON
PA MONA CAMPUS, KINGSTON 7, JAMAICA
SN 0043-3144
J9 W INDIAN MED J
JI West Ind. Med. J.
PD JUN
PY 2011
VL 60
IS 3
BP 263
EP 268
PG 6
WC Medicine, General & Internal
WE Science Citation Index Expanded (SCI-EXPANDED)
SC General & Internal Medicine
GA 858DN
UT WOS:000297776200004
PM 22224336
DA 2022-07-22
ER

PT J
AU Nilsson-Helander, K
   Thurin, A
   Karlsson, J
   Eriksson, BI
AF Nilsson-Helander, Katarina
   Thurin, Anders
   Karlsson, Jon
   Eriksson, Bengt I.
TI High incidence of deep venous thrombosis after Achilles tendon rupture:
   a prospective study
SO KNEE SURGERY SPORTS TRAUMATOLOGY ARTHROSCOPY
LA English
DT Article
DE Achilles tendon rupture; Deep venous thrombosis; Colour duplex
   sonography
ID MOLECULAR-WEIGHT HEPARIN; COLOR DOPPLER ULTRASOUND; VEIN-THROMBOSIS;
   TOTAL HIP; SURGERY; THROMBOPROPHYLAXIS; OUTPATIENTS; DIAGNOSIS;
   ACCURACY; INJURY
AB Deep venous thrombosis (DVT) is common after lower limb injury, but the effect of prophylactic treatment has not been documented in large randomised trials or meta-analyses. As a result, evidence-based recommendations have not been established. The purpose of this study was to evaluate the incidence of venous thromboembolism in patients with Achilles tendon rupture. A total of 100 consecutive patients with an acute Achilles tendon rupture were included in a prospective study and randomised to either surgical or non-surgical treatment. At 8 weeks after the initiation of treatment, 95/100 patients were screened for DVT using colour duplex sonography (CDS) with blinded interpretation by two experienced examiners and adjudication in cases of disagreement by a third person. A total of 95 patients (79 male and 16 female) with a median (range) age of 41 (24-63) years were screened for CDS at 8 weeks. Of the 95 patients, 32 had a CDS-verified thrombosis, 5 proximal and 27 distal, whereas 3 had non-fatal pulmonary embolism. Surgical treatment was performed in 49 patients, non-surgical in 46. There were no significant differences in DVT frequency between the two treatment groups. The incidence of asymptomatic and symptomatic deep venous thrombosis is high after Achilles tendon rupture and there is a need to define the possible benefit of thromboprophylaxis.
C1 [Nilsson-Helander, Katarina] Kungsbacka Hosp, Dept Orthopaed, S-43440 Kungsbacka, Sweden.
   [Karlsson, Jon; Eriksson, Bengt I.] Univ Gothenburg, Sahlgrenska Univ Hosp, Dept Orthopaed, Gothenburg, Sweden.
   [Thurin, Anders] Sahlgrens Univ Hosp, Dept Clin Physiol, Gothenburg, Sweden.
RP Nilsson-Helander, K (通讯作者)，Kungsbacka Hosp, Dept Orthopaed, S-43440 Kungsbacka, Sweden.
EM ina.nilsson@telia.com
RI Helander, Katarina Nilsson/AAE-1793-2020
OI Helander, Katarina Nilsson/0000-0002-1292-5102; Thurin,
   Anders/0000-0002-5044-6364
FU Swedish National Centre for Research in Sports (CIF); Research and
   Development Council of Halland, Sweden
FX The authors thank Gunnel Sandgren and Marie Magnusson for their help in
   interpreting the CDS results, Henry Eriksson for interpreting the
   diagnostic results of pulmonary embolism and the ultrasound technicians
   at the Department of Clinical Physiology for performing the ultrasound
   examinations. We also thank the Swedish National Centre for Research in
   Sports (CIF) and the local Research and Development Council of Halland,
   Sweden, for financial support.
CR DAVIDSON BL, 1992, ANN INTERN MED, V117, P735, DOI 10.7326/0003-4819-117-9-735
   ERIKSSON BI, 1991, J BONE JOINT SURG AM, V73A, P484, DOI 10.2106/00004623-199173040-00002
   HAVIG O, 1977, ACTA CHIR SCAND, P1
   Jorgensen PS, 2002, THROMB RES, V105, P477, DOI 10.1016/S0049-3848(02)00059-2
   Kassai B, 2004, THROMB HAEMOSTASIS, V91, P655, DOI 10.1160/TH03-11-0722
   Kearon C, 2001, SEMIN THROMB HEMOST, V27, P3, DOI 10.1055/s-2001-12842
   KUJATH P, 1993, HAEMOSTASIS, V23, P20
   Lapidus L, 2006, J THROMB HAEMOST, V4, P807, DOI 10.1111/j.1538-7836.2006.01832.x
   LAPIDUS L, 2007, THESIS KAROLINSKA I
   Lapidus LJ, 2007, J ORTHOP TRAUMA, V21, P52, DOI 10.1097/01.bot.0000250741.65003.14
   Lassen MR, 2002, NEW ENGL J MED, V347, P726, DOI 10.1056/NEJMoa011327
   Magnusson M, 1996, THROMB HAEMOSTASIS, V75, P242
   Mattos MA, 1996, J VASC SURG, V24, P738, DOI 10.1016/S0741-5214(96)70006-X
   Moller M, 2001, J BONE JOINT SURG BR, V83B, P843
   Schellong SM, 2007, J THROMB HAEMOST, V5, P1431, DOI 10.1111/j.1538-7836.2007.02570.x
   White RH, 1998, ARCH INTERN MED, V158, P1525, DOI 10.1001/archinte.158.14.1525
NR 16
TC 75
Z9 79
U1 0
U2 9
PU SPRINGER
PI NEW YORK
PA 233 SPRING ST, NEW YORK, NY 10013 USA
SN 0942-2056
EI 1433-7347
J9 KNEE SURG SPORT TR A
JI Knee Surg. Sports Traumatol. Arthrosc.
PD OCT
PY 2009
VL 17
IS 10
BP 1234
EP 1238
DI 10.1007/s00167-009-0727-y
PG 5
WC Orthopedics; Sport Sciences; Surgery
WE Science Citation Index Expanded (SCI-EXPANDED)
SC Orthopedics; Sport Sciences; Surgery
GA 501MH
UT WOS:000270385500017
PM 19238360
DA 2022-07-22
ER

PT J
AU Sarman, H
   Atmaca, H
   Cakir, O
   Muezzinoglu, US
   Anik, Y
   Memisoglu, K
   Baran, T
   Isik, C
AF Sarman, Hakan
   Atmaca, Halil
   Cakir, Ozgur
   Muezzinoglu, Umit Sefa
   Anik, Yonca
   Memisoglu, Kaya
   Baran, Tuncay
   Isik, Cengiz
TI Assessment of Postoperative Tendon Quality in Patients With Achilles
   Tendon Rupture Using Diffusion Tensor Imaging and Tendon Fiber Tracking
SO JOURNAL OF FOOT & ANKLE SURGERY
LA English
DT Article
DE Achilles tendon rupture; diffusion tensor imaging; fractional
   anisotropy; surgery; tendon fiber continuity; trauma
ID HUMAN SKELETAL-MUSCLE; HUMAN FOREARM; REPAIR; TRACTOGRAPHY;
   IMMOBILIZATION; MRI; EXERCISE; THERAPY; INJURY
AB Although pre- and postoperative imaging of Achilles tendon rupture (ATR) has been well documented, radiographic evaluations of postoperative intratendinous healing and microstructure are still lacking. Diffusion tensor imaging (DTI) is an innovative technique that offers a noninvasive method for describing the microstructure characteristics and organization of tissues. DTI was used in the present study for quantitative assessment of fiber continuity postoperatively in patients with acute ATR. The data from 16 patients with ATR from 2005 to 2012 were retrospectively analyzed. The microstructure of ART was evaluated using tendon fiber tracking, tendon continuity, fractional anisotropy, and apparent diffusion coefficient values by way of DTI. The distal and proximal portions were measured separately in both the ruptured and the healthy extremities of each patient. The mean patient age was 41.56 +/- 8.49 (range 26 to 56) years. The median duration of follow-up was 21 (range 6 to 80) months. The tendon fractional anisotropy values of the ruptured Achilles tendon were significantly lower statistically than those of the normal side (p - .001). However, none of the differences between the 2 groups with respect to the distal and proximal apparent diffusion coefficient were statistically significant (p = .358 and p = .899, respectively). In addition, the fractional anisotropy and apparent diffusion coefficient measurements were not significantly different in the proximal and distal regions of the ruptured tendons compared with the healthy tendons. The present study used DTI and fiber tracking to demonstrate the radiologic properties of postoperative Achilles tendons with respect to trajectory and tendinous fiber continuity. Quantifying DTI and fiber tractography offers an innovative and effective tool that might be able to detect microstructural abnormalities not appreciable using conventional radiologic techniques. (C) 2015 by the American College of Foot and Ankle Surgeons. All rights reserved.
C1 [Sarman, Hakan; Isik, Cengiz] Abant Izzet Baysal Univ, Sch Med, Dept Orthoped & Traumatol, TR-14280 Golkoy, Bolu, Turkey.
   [Atmaca, Halil] Akdeniz Univ, Sch Med, Dept Orthoped & Traumatol, TR-07058 Antalya, Turkey.
   [Cakir, Ozgur] Minist Hlth Batman Reg Hosp, Dept Radiol, Batman, Turkey.
   [Muezzinoglu, Umit Sefa; Memisoglu, Kaya; Baran, Tuncay] Kocaeli Univ, Sch Med, Dept Orthoped & Traumatol, Kocaeli, Turkey.
   [Anik, Yonca] Kocaeli Univ, Sch Med, Dept Radiol, Kocaeli, Turkey.
RP Sarman, H (通讯作者)，Abant Izzet Baysal Univ, Sch Med, Dept Orthoped & Traumatol, TR-14280 Golkoy, Bolu, Turkey.
EM hakansarman@yahoo.com
RI Sarman, Hakan MD/B-5281-2015; çakır, özgür/G-4427-2018; çakır,
   özgür/AAA-8756-2021; Atmaca, Halil/M-3550-2014; memişoğlu,
   kaya/AAK-3514-2020; ANIK, YONCA/AAA-5092-2021
OI Sarman, Hakan MD/0000-0003-2221-4731; çakır, özgür/0000-0001-6565-9488;
   çakır, özgür/0000-0001-6565-9488; Atmaca, Halil/0000-0001-5820-6456;
   ANIK, YONCA/0000-0002-6768-2574; Isik, cengiz/0000-0002-9750-9350
CR Bleakney RR, 2002, CLIN J SPORT MED, V12, P273, DOI 10.1097/00042752-200209000-00003
   BRADLEY JP, 1990, AM J SPORT MED, V18, P188, DOI 10.1177/036354659001800213
   Buchgraber A, 1997, CLIN ORTHOP RELAT R, P113
   Cermak NM, 2012, MUSCLE NERVE, V46, P42, DOI 10.1002/mus.23276
   Deux JF, 2008, EUR RADIOL, V18, P2303, DOI 10.1007/s00330-008-1012-z
   Froeling M, 2012, J MAGN RESON IMAGING, V36, P237, DOI 10.1002/jmri.23608
   Froeling M, 2010, MAGN RESON MED, V64, P1182, DOI 10.1002/mrm.22477
   Jallageas R, 2013, ORTHOP TRAUMATOL-SUR, V99, P577, DOI 10.1016/j.otsr.2013.03.024
   Kan JH, 2009, J MAGN RESON IMAGING, V29, P663, DOI 10.1002/jmri.21687
   Kannus P, 1997, SCAND J MED SCI SPOR, V7, P67
   Kermarrec E, 2010, AM J ROENTGENOL, V195, pW352, DOI 10.2214/AJR.09.3368
   Khan KM, 2003, BRIT J SPORT MED, V37, P149, DOI 10.1136/bjsm.37.2.149
   Leppilahti J, 1998, SPORTS MED, V25, P79, DOI 10.2165/00007256-199825020-00002
   Leppilahti J, 1998, CLIN ORTHOP RELAT R, P152
   Majewski M, 2003, UNFALLCHIRURG, V106, P556, DOI 10.1007/s00113-003-0623-8
   Min Y, 2013, ANN REHABIL MED-ARM, V37, P1, DOI 10.5535/arm.2013.37.1.1
   Moller M, 2002, KNEE SURG SPORT TR A, V10, P49, DOI 10.1007/s001670100245
   Muezzinoglu S, 2013, TECH FOOT ANKLE SURG, V12, P92, DOI 10.1097/BTF.0b013e318290ff37
   Nilsson-Helander K, 2007, AM J SPORT MED, V35, P421, DOI 10.1177/0363546506294856
   OBrien M, 1997, SCAND J MED SCI SPOR, V7, P55
   Ozkaya U, 2009, INJURY, V40, P669, DOI 10.1016/j.injury.2008.10.033
   Saupe N, 2009, AM J ROENTGENOL, V192, pW282, DOI 10.2214/AJR.08.1340
   Schepull T, 2013, KNEE SURG SPORTS TRA
   Schizas N, 2010, J ORTHOP RES, V28, P852, DOI 10.1002/jor.21066
   Sharma P, 2005, J BONE JOINT SURG AM, V87A, P187, DOI 10.2106/JBJS.D.01850
   Strauss EJ, 2007, INJURY, V38, P832, DOI 10.1016/j.injury.2006.06.005
   Thermann H, 2002, FOOT ANKLE INT, V23, P606, DOI 10.1177/107110070202300704
   White DW, 2007, AM J SPORT MED, V35, P1308, DOI 10.1177/0363546507301256
   Yanagisawa O, 2011, J MAGN RESON IMAGING, V34, P887, DOI 10.1002/jmri.22668
   Zaraiskaya T, 2006, J MAGN RESON IMAGING, V24, P402, DOI 10.1002/jmri.20651
   Zijta FM, 2013, CLIN ANAT, V26, P110, DOI 10.1002/ca.22184
NR 32
TC 12
Z9 13
U1 1
U2 10
PU ELSEVIER SCIENCE INC
PI NEW YORK
PA 360 PARK AVE SOUTH, NEW YORK, NY 10010-1710 USA
SN 1067-2516
EI 1542-2224
J9 J FOOT ANKLE SURG
JI J. Foot Ankle Surg.
PD SEP-OCT
PY 2015
VL 54
IS 5
BP 782
EP 786
DI 10.1053/j.jfas.2014.12.025
PG 5
WC Orthopedics; Surgery
WE Science Citation Index Expanded (SCI-EXPANDED)
SC Orthopedics; Surgery
GA CQ1QO
UT WOS:000360373600004
PM 25736446
DA 2022-07-22
ER

PT J
AU Jiang, X
   Qian, SL
   Chen, C
   Wu, HL
   Zhi, XS
   Xu, D
   Lian, JH
   Liu, XM
   Wei, SJ
   Xu, F
AF Jiang, Xiang
   Qian, Shenglong
   Chen, Cheng
   Wu, Helin
   Zhi, Xiaosong
   Xu, Dan
   Lian, Junhong
   Liu, Ximing
   Wei, Shijun
   Xu, Feng
TI Modified mini-incision "internal splinting" versus percutaneous repair
   technique of acute Achilles tendon rupture: five year retrospective
   case-controlled study
SO INTERNATIONAL ORTHOPAEDICS
LA English
DT Article
DE Achilles tendon rupture; Mini-incision; Internal Splinting;
   Percutaneous; Suture anchor
ID COMPLICATIONS; OUTCOMES
AB Purpose Several kinds of minimally invasive surgical techniques are applied to acute Achilles tendon rupture. The risks of sural never injury and re-ruptures are still major issues. The purpose of this study was to compare the middle-term results of two different minimally invasive repair techniques for acute Achille tendon rupture. Methods Twenty-four cases underwent the modified mini-incision "internal splinting" repair technique in group A, and 29 cases underwent a percutaneous repair technique in group B and were evaluated. The intra-operative data, complications, the time of recovery, and the post-operative magnetic resonance imaging were analyzed. At the final follow-up, the American Orthopedic Foot and Ankle Society (AOFAS) ankle-hindfoot score and Thermann score were evaluated. Results The mean follow-up time was 59.96 +/- 4.16 months (range 48-67). At the final follow-up, the AOFAS score and Thermann score in both groups had similar feedback. No nerve injury, infection, or re-rupture was encountered in group A, except two cases with anchor irritation. One case with sural nerve injury and one with a re-rupture were recorded in group B, respectively. The final MRI showed thicker regeneration of the tendon in both groups. Conclusions The middle-term results indicated the modified mini-incision "internal splinting" technique for acute Achilles tendon rupture is similar to the percutaneous repair technique. A lower risk of sural nerve injury and re-rupture may be advantages.
C1 [Jiang, Xiang; Qian, Shenglong; Chen, Cheng; Zhi, Xiaosong; Lian, Junhong; Liu, Ximing; Wei, Shijun; Xu, Feng] Gen Hosp Cent Theater Command, Wuhan Gen Hosp Guangzhou Command, Dept Orthopaed, Wuhan, Hubei, Peoples R China.
   [Wu, Helin; Liu, Ximing; Wei, Shijun; Xu, Feng] Southern Med Univ, Clin Med Sch 1, Guangzhou, Guangdong, Peoples R China.
   [Xu, Dan] Gen Hosp Cent Theater Command, Wuhan Gen Hosp Guangzhou Command, Dept Rehabil, Wuhan, Hubei, Peoples R China.
RP Lian, JH; Wei, SJ (通讯作者)，Gen Hosp Cent Theater Command, Wuhan Gen Hosp Guangzhou Command, Dept Orthopaed, Wuhan, Hubei, Peoples R China.; Wei, SJ (通讯作者)，Southern Med Univ, Clin Med Sch 1, Guangzhou, Guangdong, Peoples R China.
EM 1329161413@qq.com; wsj1974@yeah.net
CR Alcelik I, 2018, FOOT ANKLE SURG, V24, P427, DOI 10.1016/j.fas.2017.04.016
   Assal M, 2002, J BONE JOINT SURG AM, V84A, P161, DOI 10.2106/00004623-200202000-00001
   Chan JJ, 2020, INT ORTHOP, V44, P585, DOI 10.1007/s00264-019-04471-2
   Clanton T, 2020, ORTHOP CLIN N AM, V51, P391, DOI 10.1016/j.ocl.2020.02.005
   Cottom JM, 2017, J FOOT ANKLE SURG, V56, P423, DOI 10.1053/j.jfas.2016.10.012
   Fischer S, 2021, ARCH ORTHOP TRAUM SU, V141, P751, DOI 10.1007/s00402-020-03461-z
   Gatz M, 2021, ARCH ORTHOP TRAUM SU, V141, P383, DOI 10.1007/s00402-020-03437-z
   Heikkinen J, 2017, AM J SPORT MED, V45, P1395, DOI 10.1177/0363546517694610
   Hsu AR, 2015, FOOT ANKLE INT, V36, P1279, DOI 10.1177/1071100715589632
   Joannas G, 2020, FOOT ANKLE SURG, V26, P209, DOI 10.1016/j.fas.2019.02.005
   Kupcha Paul C, 2008, Am J Orthop (Belle Mead NJ), V37, P586
   Mavrodontidis A, 2015, ACTA ORTHOP TRAUMATO, V49, P661, DOI 10.3944/AOTT.2015.14.0196
   Meulenkamp B, 2021, CLIN ORTHOP RELAT R, V479, P2119, DOI 10.1097/CORR.0000000000001861
   Noback PC, 2017, INJURY, V48, P2342, DOI 10.1016/j.injury.2017.08.050
   Ochen Y, 2019, BMJ-BRIT MED J, V364, DOI 10.1136/bmj.k5120
   Sarman H, 2016, J FOOT ANKLE SURG, V55, P965, DOI 10.1053/j.jfas.2016.04.014
   Severyns M, 2019, ARTHROSC TEC, V8, pE489, DOI 10.1016/j.eats.2019.01.007
   Soubeyrand M, 2010, FOOT ANKLE INT, V31, P1069, DOI 10.3113/FAI.2010.1069
   Valentini V, 2002, FOOT ANKLE SURG, V8, P255, DOI [10.1046/j.1460-9584.2002.00334.x, DOI 10.1046/J.1460-9584.2002.00334.X]
   van Maele M, 2018, INJURY, V49, P1947, DOI 10.1016/j.injury.2018.08.012
   Wei SJ, 2021, ARCH ORTHOP TRAUM SU, V141, P1753, DOI 10.1007/s00402-021-03818-y
   Yang B, 2017, INT J SURG, V40, P178, DOI 10.1016/j.ijsu.2017.03.021
   Zappia M, 2018, FOOT ANKLE SURG, V24, P342, DOI 10.1016/j.fas.2017.03.006
NR 23
TC 0
Z9 0
U1 0
U2 1
PU SPRINGER
PI NEW YORK
PA ONE NEW YORK PLAZA, SUITE 4600, NEW YORK, NY, UNITED STATES
SN 0341-2695
EI 1432-5195
J9 INT ORTHOP
JI Int. Orthop.
PD DEC
PY 2021
VL 45
IS 12
BP 3243
EP 3251
DI 10.1007/s00264-021-05185-0
EA AUG 2021
PG 9
WC Orthopedics
WE Science Citation Index Expanded (SCI-EXPANDED)
SC Orthopedics
GA XD7MY
UT WOS:000684458700001
PM 34386884
DA 2022-07-22
ER

PT J
AU Maffulli, N
   D'Addona, A
   Gougoulias, N
   Oliva, F
   Maffulli, GD
AF Maffulli, Nicola
   D'Addona, Alessio
   Gougoulias, Nikolaos
   Oliva, Francesco
   Maffulli, Gayle D.
TI Ipsilateral free semitendinosus graft with interference screw fixation
   for surgical management of insertional acute Achilles tendon tears
SO INJURY-INTERNATIONAL JOURNAL OF THE CARE OF THE INJURED
LA English
DT Article
DE Acute rupture; Achilles tendon; Insertional avulsion; Semitendinosus
   graft; Minimally invasive
ID REGIONAL PROPHYLAXIS; SLEEVE AVULSION; OPEN REPAIR; RUPTURES;
   RECONSTRUCTION; TEICOPLANIN; ANATOMY
AB Introduction: Very distal tears of the Achilles tendon are uncommon, and poor quality tendinous tissue of the calcaneal stump can compromise healing. Little has been published about the characteristics and surgical management of such injuries. We present a surgical technique, developed by the senior author, to restore continuity of the gastrosoleus-Achilles tendon-calcaneus complex using a free ipsilateral semi-tendinosus graft, and clinical outcomes of a case series of 28 consecutive patients. Our hypothesis was that this is a safe technique, and patients can return to pre-injury occupation and athletic activities.
   Patients and Methods: A total of 28 patients (mean age 46 years) underwent minimally invasive reconstruction using a free ipsilateral semitendinosus graft for acute insertional rupture of tendo Achillis. The procedure required two small incisions along the course of the Achilles tendon, and one posteromedial incision at level of the ipsilateral knee to harvest the semitendinosus tendon. Patients were assessed at minimum 2 years (range, 2-2.5 years) following the index procedure.
   Results: The median Achilles tendon Rupture Score (ATRS) at the latest follow-up was 88. Two patients developed a superficial wound infection. All patients returned to their preinjury occupation, whilst 22 out of 28 patients (79%), returned to their preinjury level of physical activity at a mean of 6.7 months after surgery, reporting good or excellent overall satisfaction in 88.5% of cases.
   Conclusion: This minimally invasive technique was safe, and allowed most of patients to return to preinjury daily and sport activities within 9 months from surgery. (C) 2019 Elsevier Ltd. All rights reserved.
C1 [Maffulli, Nicola; Oliva, Francesco] Univ Salerno, Dept Musculoskeletal Disorders, Salerno, Italy.
   [Maffulli, Nicola] Mile End Hosp, Ctr Sports & Exercise Med, Barts & London Sch Med & Dent, 275 Bancroft Rd, London E1 4DG, England.
   [Maffulli, Nicola] Keele Univ, Sch Pharm & Bioengn, Fac Med, Thorn Burrow Dr, Stoke On Trent ST4, Staffs, England.
   [D'Addona, Alessio] Sch Med & Surg Federico II, Dept Publ Hlth, Sect Orthopaed & Trauma Surg, AOU Federico 2,Via S Pansini 5, I-80131 Naples, Italy.
   [Gougoulias, Nikolaos] Frimley Pk Hosp, Frimley Hlth NHS Fdn Trust, Portsmouth Rd, Surrey GU16 7UJ, England.
   [Gougoulias, Nikolaos] Foot Surg Private Practice, Thessaloniki, Greece.
   [Gougoulias, Nikolaos] Foot Surg Private Practice, Athens, Greece.
   [Maffulli, Gayle D.] Wholelife Clin, London, England.
RP Maffulli, N (通讯作者)，Univ Salerno, Dept Musculoskeletal Disorders, Salerno, Italy.
EM n.maffulli@qmul.ac.uk
RI Maffulli, Nicola/A-1839-2014
OI Maffulli, Nicola/0000-0002-5327-3702
FU Scuola di specializzazione in Ortopedia e Traumatologia, Catholic
   University, Rome
FX The paper is part of a Supplement supported by the Scuola di
   specializzazione in Ortopedia e Traumatologia, Catholic University,
   Rome.
CR Bibbo C, 2003, FOOT ANKLE INT, V24, P539
   Clain M R, 1992, Foot Ankle, V13, P482
   De Lalla F, 2001, J CHEMOTHERAPY, V13, P48, DOI 10.1179/joc.2001.13.Supplement-2.48
   de Lalla F, 2000, ANTIMICROB AGENTS CH, V44, P316, DOI 10.1128/AAC.44.2.316-319.2000
   Del Buono A, 2013, INT ORTHOP, V37, P715, DOI 10.1007/s00264-012-1743-y
   Diniz P, 2019, KNEE SURG SPORT TR A, V27, P1847, DOI 10.1007/s00167-019-05362-0
   Huh J, 2016, FOOT ANKLE INT, V37, P596, DOI 10.1177/1071100716629778
   Hulet C, 2019, KNEE SURG SPORT TR A, V27, P1754, DOI 10.1007/s00167-019-05440-3
   Lazzarini L, 2003, J ARTHROPLASTY, V18, P342, DOI 10.1054/arth.2003.50053
   Longo UG, 2010, BRIT MED BULL, V94, P165, DOI 10.1093/bmb/ldp051
   Maffulli N, 1999, J BONE JOINT SURG AM, V81A, P1019, DOI 10.2106/00004623-199907000-00017
   Maffulli N, 2003, AM J SPORT MED, V31, P692, DOI 10.1177/03635465030310051001
   Maffulli N, 2003, J SPORT MED PHYS FIT, V43, P367
   Maffulli Nicola, 2007, Foot Ankle Clin, V12, P583, DOI 10.1016/j.fcl.2007.07.007
   Maffulli N, 2018, FOOT ANKLE SURG, V24, P164, DOI 10.1016/j.fas.2017.02.002
   Maffulli N, 2008, BMC MUSCULOSKEL DIS, V9, DOI 10.1186/1471-2474-9-100
   Maffulli N, 2012, J BONE JOINT SURG AM, V94A, P906, DOI 10.2106/JBJS.K.00869
   Maffulli N, 2010, KNEE SURG SPORT TR A, V18, P269, DOI 10.1007/s00167-009-0968-9
   McClelland D, 2002, J ROY COLL SURG EDIN, V47, P613
   Movin Tomas, 2005, Foot Ankle Clin, V10, P331, DOI 10.1016/j.fcl.2005.01.003
   Oliva F, 2018, MLTJ-MUSCLE LIGAMENT, V8, P310, DOI 10.11138/mltj/2018.8.3.310
   Papalia R, 2015, ARTHROSCOPY, V31, P1169, DOI 10.1016/j.arthro.2014.11.015
   Papalia R, 2014, BRIT MED BULL, V109, P19, DOI 10.1093/bmb/ldt038
   Pavlou G, 2009, FOOT ANKLE INT, V30, P65, DOI 10.3113/FAI.2009.0065
   Perkins CA, 2019, AM J SPORT MED, V47, P1576, DOI 10.1177/0363546519849607
   Schipper ON, 2018, FOOT ANKLE INT, V39, P664, DOI 10.1177/1071100718758257
   Webb J, 2000, FOOT ANKLE INT, V21, P475, DOI 10.1177/107110070002100604
   WILLIAMS A, 1990, HEALTH POLICY, V16, P199
   Young SW, 2013, CLIN ORTHOP RELAT R, V471, P244, DOI 10.1007/s11999-012-2469-2
   Zhang H, 2018, J ORTHOP SURG RES, V13, DOI 10.1186/s13018-018-0895-x
NR 30
TC 6
Z9 6
U1 0
U2 0
PU ELSEVIER SCI LTD
PI OXFORD
PA THE BOULEVARD, LANGFORD LANE, KIDLINGTON, OXFORD OX5 1GB, OXON, ENGLAND
SN 0020-1383
EI 1879-0267
J9 INJURY
JI Injury-Int. J. Care Inj.
PD AUG
PY 2020
VL 51
SU 3
BP S73
EP S79
DI 10.1016/j.injury.2019.11.013
PG 7
WC Critical Care Medicine; Emergency Medicine; Orthopedics; Surgery
WE Science Citation Index Expanded (SCI-EXPANDED)
SC General & Internal Medicine; Emergency Medicine; Orthopedics; Surgery
GA OA1OX
UT WOS:000577565000015
PM 31761423
DA 2022-07-22
ER

PT J
AU Koltsov, JCB
   Gribbin, C
   Ellis, SJ
   Nwachukwu, BU
AF Koltsov, Jayme C. B.
   Gribbin, Caitlin
   Ellis, Scott J.
   Nwachukwu, Benedict U.
TI Cost-effectiveness of Operative Versus Non-operative Management of Acute
   Achilles Tendon Ruptures
SO HSS JOURNAL
LA English
DT Article
DE acute Achilles tendon ruptures; cost-effectiveness; cost utility
AB Background The management of acute Achilles tendon ruptures is controversial, and most injuries are treated with surgery in the USA. The cost utility of operative versus non-operative treatment of acute Achilles tendon injury is unclear. Questions/Purposes The purpose of this study was to compare the cost-effectiveness of operative versus functional non-operative treatment of acute Achilles tendon ruptures. Methods A Markov cost-utility analysis was conducted from the societal perspective using a 2-year time horizon. Hospital costs were derived from New York State billing data, and physician and rehabilitation costs were derived from the Medicare physician fee schedule. Indirect costs of missed work were calculated using estimates from the US Bureau of Labor Statistics. Rates of re-rupture, major and minor complications, and the associated costs were obtained from the literature. Effectiveness was expressed in quality-adjusted life years (QALYs). For the base-case analysis, operative and non-operative patients were assumed to have the same utilities (quality of life) following surgery. Deterministic and probabilistic sensitivity analyses were conducted to evaluate the robustness of model assumptions. Results In the base-case model, non-operative management of acute Achilles tendon ruptures dominated operative management, resulting in both lower costs and greater QALY gains. The differences in costs and effectiveness were relatively small. The benefit of non-operative treatment was 1.69 QALYs, and the benefit of operative treatment was 1.67 QALYs. Similarly, the total cost of operative and non-operative management was $13, 936 versus $13, 413, respectively. In sensitivity analyses, surgical costs and days of missed work were important drivers of cost-effectiveness. If hospitalization costs dropped below $2621 (compared with $3145) or the hourly wage rose above $29 (compared with $24), then operative treatment became a cost-effective strategy at the willingness-to-pay threshold of $50, 000/QALY. The model results were also highly sensitive to the relative utilities for operative versus non-operative treatment. If non-operative utilities decreased relative to operative utilities by just 2%, then operative management became the dominant treatment strategy. Conclusion For acute Achilles tendon ruptures, non-operative treatment provided greater benefits and lower costs than operative management in the base case; however, surgical costs and the economic impact associated with return to work are important determinants of the preferred cost-effective strategy.
C1 [Koltsov, Jayme C. B.] Stanford Univ, Sch Med, Dept Orthopaed Surg, 450 Broadway St,Pavil C,4th Floor,Mail Code 6342, Redwood City, CA 94063 USA.
   [Gribbin, Caitlin; Ellis, Scott J.; Nwachukwu, Benedict U.] Hosp Special Surg, Dept Orthopaed Surg, 535 East 70th St, New York, NY 10021 USA.
RP Nwachukwu, BU (通讯作者)，Hosp Special Surg, Dept Orthopaed Surg, 535 East 70th St, New York, NY 10021 USA.
EM nwachukwub@hss.edu
OI Nwachukwu, Benedict/0000-0002-6170-7769
CR Bhandari M, 2002, CLIN ORTHOP RELAT R, P190
   Centers for Medicare & Medicaid Services, 2014, PHYS FEE SCHED CY 20
   Claxton K, 2005, HEALTH ECON, V14, P339, DOI 10.1002/hec.985
   Cooper MT, 2015, CLIN SPORT MED, V34, P595, DOI 10.1016/j.csm.2015.06.001
   Costa ML, 2006, J BONE JOINT SURG BR, V88B, P69, DOI 10.1302/0301-620X.88B1.16549
   Del Buono A, 2014, BRIT MED BULL, V109, P45, DOI 10.1093/bmb/ldt029
   Healthcare Cost and Utilization Project, 2015, COST TO CHARG RAT FI
   Heikkinen J, 2017, AM J SPORT MED, V45, P1395, DOI 10.1177/0363546517694610
   Houshian S, 1998, INJURY, V29, P651, DOI 10.1016/S0020-1383(98)00147-8
   Huttunen TT, 2014, AM J SPORT MED, V42, P2419, DOI 10.1177/0363546514540599
   Keating JF, 2011, J BONE JOINT SURG BR, V93B, P1071, DOI 10.1302/0301-620X.93B8.25998
   Khan RJK, 2005, J BONE JOINT SURG AM, V87A, P2202, DOI 10.2106/JBJS.D.03049
   Kocher MS, 2002, AM J SPORT MED, V30, P783, DOI 10.1177/03635465020300060501
   Levi N, 1997, INJURY, V28, P311, DOI 10.1016/S0020-1383(96)00200-8
   Metz R, 2008, AM J SPORT MED, V36, P1688, DOI 10.1177/0363546508319312
   Moller A, 1996, ACTA ORTHOP SCAND, V67, P479, DOI 10.3109/17453679608996672
   Moller M, 2001, J BONE JOINT SURG BR, V83B, P843
   Neumann PJ, 2014, NEW ENGL J MED, V371, P796, DOI 10.1056/NEJMp1405158
   Nwachukwu BU, 2015, ORTHOPEDICS, V38, pE673, DOI 10.3928/01477447-20150804-53
   Nwachukwu BU, 2015, J ARTHROPLASTY, V30, P1118, DOI 10.1016/j.arth.2015.02.017
   Nwachukwu BU, 2015, AM J SPORT MED, V43, P1530, DOI 10.1177/0363546514544684
   Nwachukwu BU, 2015, CLIN ORTHOP RELAT R, V473, P1815, DOI 10.1007/s11999-014-3964-4
   Nwachukwu BU, 2015, SPINE, V40, P31, DOI 10.1097/BRS.0000000000000663
   Nwachukwu BU, 2013, JBJS REV, V1, DOI 10.2106/JBJS.RVW.M.00067
   Nyyssonen T, 2008, SCAND J SURG, V97, P272, DOI 10.1177/145749690809700312
   Olsson N, 2013, AM J SPORT MED, V41, P2867, DOI 10.1177/0363546513503282
   Porter ME, 2010, NEW ENGL J MED, V363, P2477, DOI 10.1056/NEJMp1011024
   Rebeccato A, 2001, J Foot Ankle Surg, V40, P188
   Riley GF, 2009, MED CARE, V47, pS51, DOI 10.1097/MLR.0b013e31819c95aa
   Russell LB, 1996, JAMA-J AM MED ASSOC, V276, P1172, DOI 10.1001/jama.276.14.1172
   Saltzman BM, 2016, AM J SPORT MED, V44, P1329, DOI 10.1177/0363546515581470
   Siegel JE, 1996, JAMA-J AM MED ASSOC, V276, P1339, DOI 10.1001/jama.276.16.1339
   SONNENBERG FA, 1993, MED DECIS MAKING, V13, P322, DOI 10.1177/0272989X9301300409
   Soroceanu A, 2012, J BONE JOINT SURG AM, V94A, P2136, DOI 10.2106/JBJS.K.00917
   Suchak AA, 2005, FOOT ANKLE INT, V26, P932, DOI 10.1177/107110070502601106
   Uquillas CA, 2015, J BONE JOINT SURG AM, V97A, P1187, DOI 10.2106/JBJS.O.00002
   US Bureau of Labor Statistics, 2014, CURR EMPL STAT ARCH
   van der Eng DM, 2013, J FOOT ANKLE SURG, V52, P622, DOI 10.1053/j.jfas.2013.03.027
   Weinstein MC, 1996, JAMA-J AM MED ASSOC, V276, P1253, DOI 10.1001/jama.276.15.1253
   Willits K, 2010, J BONE JOINT SURG AM, V92A, P2767, DOI 10.2106/JBJS.I.01401
NR 40
TC 2
Z9 2
U1 0
U2 0
PU SAGE PUBLICATIONS INC
PI THOUSAND OAKS
PA 2455 TELLER RD, THOUSAND OAKS, CA 91320 USA
SN 1556-3316
EI 1556-3324
J9 HSS J
JI HSS J.
PD FEB
PY 2020
VL 16
IS 1
BP 39
EP 45
DI 10.1007/s11420-019-09684-0
PG 7
WC Orthopedics; Surgery
WE Science Citation Index Expanded (SCI-EXPANDED)
SC Orthopedics; Surgery
GA VK8CZ
UT WOS:000755971800007
PM 32015739
OA Green Published, hybrid
DA 2022-07-22
ER

PT J
AU Tasatan, E
   Emre, TY
   Demircioglu, DT
   Demiralp, B
   Kirdemir, V
AF Tasatan, Ersin
   Emre, Tuluhan Yunus
   Demircioglu, Demet Tekdos
   Demiralp, Bahtiyar
   Kirdemir, Vecihi
TI Long-Term Results of Mini-Open Repair Technique in the Treatment of
   Acute Achilles Tendon Rupture: A Prospective Study
SO JOURNAL OF FOOT & ANKLE SURGERY
LA English
DT Article
DE Achilles tendon rupture; mini-open surgery; repeat rupture; wound
   healing
ID NONSURGICAL TREATMENT; PERCUTANEOUS REPAIR
AB An ideal surgical treatment of acute Achilles tendon rupture includes restoring the original length of the tendon, minimizing possible adhesions with the surrounding tissues, minimizing the risk of repeat rupture, alleviating wound problems, and providing an acceptable cosmetic outcome. In the mini-open repair technique, unlike the percutaneous repair technique, the quality of the tenodesis can be visualized without disturbing the healing potential of the surrounding tissues, thus minimizing wound problems. The purpose of the present study was to assess the long-term results of the mini-open repair technique in patients with acute Achilles tendon rupture. A total of 20 consecutive patients with acute Achilles tendon rupture, admitted to our inpatient clinic from October 2003 to March 2008, were included in the present study. The patients underwent Achilles tenodesis with the mini-open repair technique, and each patient was followed up for 5 years. The study was completed in April 2013. The surgical procedure was performed with the assistance of a device designed in our orthosis laboratories, similarly to that defined by Assal et al. Of the 20 patients, 18 were male and 2 were female. Their mean age was 39.3 (range 21 to 55) years. The Achilles tendon rupture was located on the left side in 15 patients (75%) and on the right side in 5 patients (25%). The mean follow-up duration was 58.5 (range 18 to 60) months and no complications occurred during the follow-up period, including repeat rupture, wound site infection, and sural nerve injury. The mean American Orthopaedic Foot and Ankle Society scale score for the patients was 99.2 (range 94 to 100) points at the final follow-up visit. All our patients were able to return to work and sporting activities. According to the Trillat scores, the outcome was excellent in 19 patients and good in 1 patient at the 18th postoperative month. No complaint, such as pain or loss of function, that might have a negative effect on the patients' business or social life was detected in 18 patients who were assessed at 5 years after surgery; 2 patients could not be reached at 5 years. In conclusion, as a technique combining percutaneous and open surgical techniques, mini-open repair of Achilles tendon rupture allows a satisfactory end-to-end approximation of the tendon just in the open surgery and provides the wound healing advantages of percutaneous surgery. (C) 2016 by the American College of Foot and Ankle Surgeons. All rights reserved.
C1 [Tasatan, Ersin] Kasimpasa Mil Hosp, Orthopaed & Traumatol Dept, Istanbul, Turkey.
   [Emre, Tuluhan Yunus] Mem Hizmet Hosp, Orthopaed & Traumatol Dept, Halkali Caddesi Yesilkoy,Toplu Konut Sitesi A 31, Istanbul, Turkey.
   [Demircioglu, Demet Tekdos] Mem Hizmet Hosp, Phys Med & Rehabil Dept, Istanbul, Turkey.
   [Demiralp, Bahtiyar] Medipol Univ Hosp, Orthopaed & Traumatol Dept, Istanbul, Turkey.
   [Kirdemir, Vecihi] Suleyman Demirel Univ Hosp, Orthopaed & Traumatol Dept, Isparta, Turkey.
RP Emre, TY (通讯作者)，Mem Hizmet Hosp, Orthopaed & Traumatol Dept, Halkali Caddesi Yesilkoy,Toplu Konut Sitesi A 31, Istanbul, Turkey.
EM drtuluhan@gmail.com
RI ; Tekdos Demircioglu, Demet/V-6713-2017
OI KIRDEMIR, VECIHI/0000-0003-1568-2828; Tekdos Demircioglu,
   Demet/0000-0001-5946-3542
CR Aktas S, 2009, FOOT ANKLE INT, V30, P391, DOI 10.3113/FAI.2009.0391
   Assal M, 2002, J BONE JOINT SURG AM, V84A, P161, DOI 10.2106/00004623-200202000-00001
   Bartel AFP, 2014, J FOOT ANKLE SURG, V53, P744, DOI 10.1053/j.jfas.2014.07.009
   CETTI R, 1993, AM J SPORT MED, V21, P791, DOI 10.1177/036354659302100606
   Cretnik Andrej, 2004, J Foot Ankle Surg, V43, P72, DOI 10.1053/j.jfas.2004.01.008
   Feldbrin Z, 2010, ISR MED ASSOC J, V12, P609
   Garrido Ignacio Martinez, 2010, Foot Ankle Surg, V16, P189, DOI 10.1016/j.fas.2009.10.014
   Guillo S, 2013, SURG-J R COLL SURG E, V11, P14, DOI 10.1016/j.surge.2011.12.002
   HART T J, 1988, Journal of Foot Surgery, V27, P30
   Henriquez H, 2012, CLIN ORTHOP RELAT R, V470, P998, DOI 10.1007/s11999-011-1830-1
   Horstmann T, 2012, INT J SPORTS MED, V33, P474, DOI 10.1055/s-0032-1301932
   INGLIS AE, 1981, CLIN ORTHOP RELAT R, P160
   Jiang N, 2012, INT ORTHOP, V36, P765, DOI 10.1007/s00264-011-1431-3
   KAKIUCHI M, 1995, J BONE JOINT SURG BR, V77B, P60, DOI 10.1302/0301-620X.77B1.7822398
   KELLAM JF, 1985, CLIN ORTHOP RELAT R, P80
   Khan RJK, 2010, COCHRANE DB SYST REV, DOI 10.1002/14651858.CD003674.pub4
   KITAOKA HB, 1994, FOOT ANKLE INT, V15, P349, DOI 10.1177/107110079401500701
   LEA RB, 1972, J BONE JOINT SURG AM, VA 54, P1398, DOI 10.2106/00004623-197254070-00002
   MA GWC, 1977, CLIN ORTHOP RELAT R, P247
   Maes Renaud, 2006, Acta Orthop Belg, V72, P179
   Maffulli N, 1999, J BONE JOINT SURG AM, V81A, P1019, DOI 10.2106/00004623-199907000-00017
   McMahon Samuel E, 2011, Foot Ankle Surg, V17, P211, DOI 10.1016/j.fas.2010.11.001
   NISTOR L, 1981, J BONE JOINT SURG AM, V63, P394, DOI 10.2106/00004623-198163030-00012
   oeuvres Les, 1633, LES OEUVRES
   Olsson N, 2011, KNEE SURG SPORT TR A, V19, P1385, DOI 10.1007/s00167-011-1511-3
   Taglialavoro G, 2011, STRATEG TRAUMA LIMB, V6, P147, DOI 10.1007/s11751-011-0124-1
   Trillat A, 1967, Lyon Chir, V63, P603
   Wilkins R, 2012, AM J SPORT MED, V40, P2154, DOI 10.1177/0363546512453293
NR 28
TC 10
Z9 13
U1 0
U2 8
PU ELSEVIER SCIENCE INC
PI NEW YORK
PA STE 800, 230 PARK AVE, NEW YORK, NY 10169 USA
SN 1067-2516
EI 1542-2224
J9 J FOOT ANKLE SURG
JI J. Foot Ankle Surg.
PD SEP-OCT
PY 2016
VL 55
IS 5
BP 971
EP 975
DI 10.1053/j.jfas.2016.04.016
PG 5
WC Orthopedics; Surgery
WE Science Citation Index Expanded (SCI-EXPANDED)
SC Orthopedics; Surgery
GA DU5MY
UT WOS:000382257000018
PM 27289216
DA 2022-07-22
ER

PT J
AU Okay, E
   Turgut, MC
   Tokyay, A
AF Okay, Erhan
   Turgut, Mehmet Cenk
   Tokyay, Abbas
TI Quadriceps reconstruction with suture anchor and Achilles allograft
   combination in quadriceps tendon re-rupture after primary surgical
   repair: A novel technique
SO JOINT DISEASES AND RELATED SURGERY
LA English
DT Article
DE Achilles allograft; quadriceps tendon re-rupture; suture anchor
ID TOTAL KNEE ARTHROPLASTY; RUPTURE
AB Quadriceps ruptures are one of the pathological conditions of the knee extensor mechanism, accounting for 3% of all tendon injuries. These injuries cause substantial disability of the extensor mechanism. Primary repair is the treatment of choice in acute presentation. In the setting of chronic conditions, the treatment becomes more challenging. Available surgical options include lengthening procedures, and reconstruction with autograft or allografts. The traditional Scuderi and Codivilla techniques are challenging to perform in degenerative or traumatic retracted ruptures. There is no standard effective treatment in these patients, which yields the best clinical and biomechanical outcomes. An 18-year-old male patient with quadriceps re-rupture after a primary repair was managed with allograft reconstruction using suture anchors. At six years of follow-up, the patient gained a full range of motion with excellent clinical outcomes. He returned to his previous work. In conclusion, quadriceps reconstruction using suture anchor and Achilles allograft combination is a feasible technique in neglected cases who present with quadriceps tendon re-rupture after primary surgical repair.
C1 [Okay, Erhan] Goztepe Prof Dr Suleyman Yalcin City Hosp, Dept Orthoped & Traumatol, Minist Hlth, Istanbul, Turkey.
   [Turgut, Mehmet Cenk] Erzurum City Hosp, Dept Orthoped & Traumatol, Minist Hlth, Erzurum, Turkey.
   [Tokyay, Abbas] Yuzuncu Yil Univ, Dept Orthoped & Traumatol, Fac Med, Van, Turkey.
RP Turgut, MC (通讯作者)，Erzurum City Hosp, Dept Orthoped & Traumatol, Minist Hlth, Erzurum, Turkey.
EM m.cenkturgut@hotmail.com
OI Okay, Erhan/0000-0003-2443-2505; turgut, m.cenk/0000-0002-8642-6824
CR Atik OS, 2019, EKLEM HAST CERRAHISI, V30, P69, DOI 10.5606/ehc.2019.003
   Forslund Johan, 2014, JBJS Case Connect, V4, pe42, DOI 10.2106/JBJS.CC.M.00230
   Jimenez A, 2020, KNEE, V27, P1446, DOI 10.1016/j.knee.2020.07.090
   Karas V, 2014, J KNEE SURG, V27, P489, DOI 10.1055/s-0034-1370899
   Kunze KN, 2019, ARTHROSC TEC, V8, pE1031, DOI 10.1016/j.eats.2019.05.015
   Lamberti Alfredo, 2019, JBJS Case Connect, V9, pe0058, DOI 10.2106/JBJS.CC.18.00058
   Langenhan R, 2012, KNEE SURG SPORT TR A, V20, P2275, DOI 10.1007/s00167-012-1887-8
   Maffulli N, 2017, SPORTS MED ARTHROSC, V25, P41, DOI 10.1097/JSA.0000000000000139
   Maffulli N, 2017, SPORTS MED ARTHROSC, V25, P51, DOI 10.1097/JSA.0000000000000138
   Nazarian DG, 1999, CLIN ORTHOP RELAT R, P123
   Paez CJ, 2020, ARTHROSC TEC, V9, pE1033, DOI 10.1016/j.eats.2020.03.021
   Pengas IP, 2016, INJURY, V47, P2065, DOI 10.1016/j.injury.2016.06.032
   Petri M, 2015, KNEE SURG SPORT TR A, V23, P1039, DOI 10.1007/s00167-014-2854-3
   de Faria JLR, 2019, ARTHROSC TEC, V8, pE1163, DOI 10.1016/j.eats.2019.06.006
   Wise BT, 2018, INT ORTHOP, V42, P2367, DOI 10.1007/s00264-018-3848-4
NR 15
TC 0
Z9 0
U1 2
U2 2
PU TURKISH JOINT DISEASES FOUNDATION
PI CANKARA
PA MUSTAFA KEMAL MAHALLESI, DUMLUPINAR BULVARI 274-2 C2 BLOK OFIS, 5,
   CANKARA, ANKARA, TURKEY
SN 2687-4784
EI 2687-4792
J9 JOINT DIS RELAT SURG
JI Joint Dis. Relat. Surg.
PY 2021
VL 32
IS 3
BP 798
EP 803
DI 10.52312/jdrs.2021.14
EA MAY 2021
PG 6
WC Orthopedics; Surgery
WE Science Citation Index Expanded (SCI-EXPANDED)
SC Orthopedics; Surgery
GA XC4JW
UT WOS:000719708900001
PM 34842117
OA gold, Green Published
DA 2022-07-22
ER

PT J
AU Longo, UG
   Forriol, F
   Campi, S
   Maffulli, N
   Denaro, V
AF Longo, Umile Giuseppe
   Forriol, Francisco
   Campi, Stefano
   Maffulli, Nicola
   Denaro, Vincenzo
TI A biomechanical comparison of the primary stability of two minimally
   invasive techniques for repair of ruptured Achilles tendon
SO KNEE SURGERY SPORTS TRAUMATOLOGY ARTHROSCOPY
LA English
DT Article
DE Achilles; Tendon; Rupture; Percutaneous; Sports; Athlete
ID PERCUTANEOUS REPAIR; SURGICAL REPAIR; IN-VITRO; FUNCTIONAL TREATMENT;
   WEIGHT-BEARING; SUTURE; INJURIES; STRENGTH; RECONSTRUCTION;
   WEIGHTBEARING
AB To compare the primary stability of two minimally invasive procedures of Achilles tendon (AT) repair, namely a modified percutaneous repair of ruptured AT and the Achillon suture configuration.
   Eighteen (nine matched pairs) frozen ovine ATs were tenotomized 5 cm from the calcaneal insertion. In each pair, one tendon was randomly allocated to one of the two techniques: a modified percutaneous repair group and the Achillon device suture configuration. Specimens were tested performing an unidirectional tensile load to failure using a servo-hydraulic testing device (MTS Systems, Eden Prairie, MN, USA), controlled by an electropulse e3000 INSTRON machine (Instron Ltd, Buckinghamshire, UK). The tendons were then loaded to failure at a rate of 10 mm/s.
   Two of the nine pairs of specimens were discarded because one specimen for each of the pair pulled out of the pneumatic clamp during mechanical testing. The remaining seven matched pairs were successfully tested. There were no differences in mean strength, mean maximum load, mean failure elongation, tension, stiffness and mode of failure between the two groups.
   The Achillon-like configuration and the modified percutaneous repair of ruptured AT provided similar biomechanical performance.
C1 [Maffulli, Nicola] Queen Mary Univ London, Mile End Hosp, Barts & London Sch Med & Dent, Ctr Sports & Exercise Med, London E1 4DG, England.
   [Forriol, Francisco] CEU Univ, Sch Med, Madrid, Spain.
   [Longo, Umile Giuseppe; Campi, Stefano; Denaro, Vincenzo] Campus Biomed Univ, Dept Orthopaed & Trauma Surg, I-00128 Rome, Italy.
RP Maffulli, N (通讯作者)，Queen Mary Univ London, Mile End Hosp, Barts & London Sch Med & Dent, Ctr Sports & Exercise Med, 275 Bancroft Rd, London E1 4DG, England.
EM n.maffulli@qmul.ac.uk
RI Maffulli, Nicola/A-1839-2014; Denaro, Vincenzo/K-9567-2016; Campi,
   Stefano/AAD-1108-2020; Longo, Umile Giuseppe/K-9147-2016
OI Maffulli, Nicola/0000-0002-5327-3702; Denaro,
   Vincenzo/0000-0003-4739-9795; Campi, Stefano/0000-0002-7544-7427; Longo,
   Umile Giuseppe/0000-0003-4063-9821; Forriol,
   Francisco/0000-0002-4307-2237
CR Aktas S, 2009, FOOT ANKLE INT, V30, P391, DOI 10.3113/FAI.2009.0391
   Amlang MH, 2010, UNFALLCHIRURG, V113, P712, DOI 10.1007/s00113-010-1809-5
   Assal M, 2002, J BONE JOINT SURG AM, V84A, P161, DOI 10.2106/00004623-200202000-00001
   Avina Valencia Jorge Arturo, 2009, Acta Ortop Mex, V23, P125
   Bhattacharyya M, 2009, INT ORTHOP, V33, P151, DOI 10.1007/s00264-008-0564-5
   Calder JDF, 2006, FOOT ANKLE INT, V27, P93, DOI 10.1177/107110070602700204
   Calder JDF, 2005, BRIT J SPORT MED, V39, P857, DOI 10.1136/bjsm.2004.017509
   Carmont MR, 2008, KNEE SURG SPORT TR A, V16, P199, DOI 10.1007/s00167-007-0411-z
   Chan S. K., 2008, Hong Kong Medical Journal, V14, P255
   Cretnik A, 2000, MED SCI SPORT EXER, V32, P16
   Deramo DM, 2008, ARTHROSCOPY, V24, P599, DOI 10.1016/j.arthro.2007.11.020
   Ebinesan AD, 2008, DISABIL REHABIL, V30, P1721, DOI 10.1080/09638280701786815
   Herbort M, 2008, ARCH ORTHOP TRAUM SU, V128, P1273, DOI 10.1007/s00402-008-0602-1
   HOCKENBURY RT, 1990, FOOT ANKLE, V11, P67, DOI 10.1177/107110079001100202
   Huffard B, 2008, CLIN BIOMECH, V23, P1158, DOI 10.1016/j.clinbiomech.2008.05.007
   Ismail M, 2008, FOOT ANKLE INT, V29, P808, DOI 10.3113/FAI.2008.0808
   Jassem M, 2001, AM J SPORT MED, V29, P734, DOI 10.1177/03635465010290061101
   Kangas J, 2003, J TRAUMA, V54, P1171, DOI 10.1097/01.TA.0000047945.20863.A2
   Khan RJK, 2010, COCHRANE DB SYST REV, DOI 10.1002/14651858.CD003674.pub4
   Kraemer Robert, 2009, J Orthop Surg Res, V4, P32, DOI 10.1186/1749-799X-4-32
   Lee SJ, 2009, AM J SPORT MED, V37, P786, DOI 10.1177/0363546508328595
   Longo UG, 2010, BR MED B, V98, P31
   Longo UG, 2011, SPORTS MED ARTHROSC, V19, P184, DOI 10.1097/JSA.0b013e318205470e
   Longo UG, 2010, BRIT MED BULL, V94, P165, DOI 10.1093/bmb/ldp051
   Longo UG, 2009, SPORTS MED ARTHROSC, V17, P112, DOI 10.1097/JSA.0b013e3181a3d625
   Longo UG, 2009, SPORTS MED ARTHROSC, V17, P127, DOI 10.1097/JSA.0b013e3181a3d767
   MA GWC, 1977, CLIN ORTHOP RELAT R, P247
   Maffulli N, 2003, AM J SPORT MED, V31, P692, DOI 10.1177/03635465030310051001
   Maffulli N, 2003, J SPORT MED PHYS FIT, V43, P367
   Maffulli N, 2010, UNFALLCHIRURG, V113, P721, DOI 10.1007/s00113-010-1834-4
   Maffulli N, 2010, BRIT J SPORT MED, V44, P21, DOI 10.1136/bjsm.2009.069526
   Maffulli Nicola, 2007, Foot Ankle Clin, V12, P583, DOI 10.1016/j.fcl.2007.07.007
   Maffulli N, 2011, BRIT MED BULL, V97, P47, DOI 10.1093/bmb/ldq026
   Maffulli N, 2011, FOOT ANKLE INT, V32, P9, DOI 10.3113/FAI.2011.0009
   Maffulli N, 2011, ARCH ORTHOP TRAUM SU, V131, P33, DOI 10.1007/s00402-010-1097-0
   Maffulli N, 2011, MED SPORT SCI, V56, P187, DOI 10.1159/000321078
   Maffulli N, 2011, KNEE SURG SPORT TR A, V19, P680, DOI 10.1007/s00167-010-1193-2
   Maffulli N, 2010, J BONE JOINT SURG AM, V92A, P2604, DOI 10.2106/JBJS.I.01744
   Maffulli N, 2010, AM J SPORT MED, V38, P2304, DOI 10.1177/0363546510376619
   Maffulli N, 2010, PHYSICIAN SPORTSMED, V38, P29, DOI 10.3810/psm.2010.06.1780
   Maffulli N, 2010, CLIN ORTHOP RELAT R, V468, P1039, DOI 10.1007/s11999-009-0944-1
   Maffulli N, 2010, KNEE SURG SPORT TR A, V18, P269, DOI 10.1007/s00167-009-0968-9
   Rouvillain JL, 2010, ACTA ORTHOP BELG, V76, P237
   Schlegel TF, 2007, J SHOULDER ELB SURG, V16, P115, DOI 10.1016/j.jse.2006.05.002
   Speck M, 1998, AM J SPORT MED, V26, P789, DOI 10.1177/03635465980260060901
   Suchak AA, 2008, J BONE JOINT SURG AM, V90A, P1876, DOI 10.2106/JBJS.G.01242
   WATSON TW, 1995, FOOT ANKLE INT, V16, P191, DOI 10.1177/107110079501600404
   Yildirim Y, 2002, FOOT ANKLE INT, V23, P1126
NR 48
TC 14
Z9 14
U1 0
U2 6
PU SPRINGER
PI NEW YORK
PA 233 SPRING ST, NEW YORK, NY 10013 USA
SN 0942-2056
EI 1433-7347
J9 KNEE SURG SPORT TR A
JI Knee Surg. Sports Traumatol. Arthrosc.
PD JUL
PY 2012
VL 20
IS 7
BP 1392
EP 1397
DI 10.1007/s00167-011-1868-3
PG 6
WC Orthopedics; Sport Sciences; Surgery
WE Science Citation Index Expanded (SCI-EXPANDED)
SC Orthopedics; Sport Sciences; Surgery
GA 962FP
UT WOS:000305527400022
PM 22228376
DA 2022-07-22
ER

PT J
AU McCartney, WT
   Robertson, I
   Kiss, K
AF McCartney, W. T.
   Robertson, I.
   Kiss, K.
TI Use of a Transarticular Circular Fixator Construct for Immobilisation of
   the Tarsocrural Joint Following Common Calcaneal Tenorraphy in Four Dogs
SO INTERNATIONAL JOURNAL OF APPLIED RESEARCH IN VETERINARY MEDICINE
LA English
DT Article
DE Achilles; calcaneal tendon; rupture; ring fixator
ID ACHILLES-TENDON RUPTURES; FASCIA LATA; REPAIR; METAANALYSIS
AB A transarticular circular fixator construct was used to immobilize the tarsocrural joint following common calcaneal tenorraphy in four dogs. The time from injury to surgery ranged from three to eight months. The circular fixator construct allowed for controlled transition from immobilisation to movement over a period of 8 weeks. No long lasting complications were associated with using the circular fixator and all dogs had a normal gait at final follow up.
C1 [McCartney, W. T.; Kiss, K.] Marie Louise Vet Hosp, Dublin, Ireland.
   [Robertson, I.] Royal Coll Surgeons Ireland, Dublin 2, Ireland.
RP McCartney, WT (通讯作者)，Marie Louise Vet Hosp, Dublin, Ireland.
CR Kangas J, 2007, AM J SPORT MED, V35, P59, DOI 10.1177/0363546506293255
   Khan RJK, 2005, J BONE JOINT SURG AM, V87A, P2202, DOI 10.2106/JBJS.D.03049
   Maffulli N, 2003, AM J SPORT MED, V31, P692, DOI 10.1177/03635465030310051001
   Maffulli N, 2008, J BONE JOINT SURG AM, V90A, P1348, DOI 10.2106/JBJS.G.01241
   MEUTSTEGE FJ, 1993, VET COMP ORTHOPAED, V6, P53
   Nielsen C, 2006, VET COMP ORTHOPAED, V19, P246, DOI 10.1055/s-0038-1633008
   Shani J, 2000, VET COMP ORTHOPAED, V13, P104
   Sivacolundhu RK, 2001, VET COMP ORTHOPAED, V14, P25
   SPRECHER H, 1998, VET COMP ORTHOPAED T, P205
   Suchak AA, 2006, CLIN ORTHOP RELAT R, P216, DOI 10.1097/01.blo.0000203458.05135.74
   Swiderski J, 2005, VET COMP ORTHOPAED, V18, P258
   Twaddle BC, 2007, AM J SPORT MED, V35, P2033, DOI 10.1177/0363546507307503
NR 12
TC 2
Z9 2
U1 0
U2 4
PU VETERINARY SOLUTIONS LLC
PI APOPKA
PA PO BOX 2083, APOPKA, FL 32704-2083 USA
SN 1542-2666
J9 INT J APPL RES VET M
JI Int. J. Appl. Res. Vet. Med.
PY 2009
VL 7
IS 3
BP 69
EP 72
PG 4
WC Veterinary Sciences
WE Science Citation Index Expanded (SCI-EXPANDED)
SC Veterinary Sciences
GA 684CG
UT WOS:000284524000002
DA 2022-07-22
ER

PT J
AU Karaaslan, F
   Mermerkaya, MU
   Cirakli, A
   Karaoglu, S
   Duygulu, F
AF Karaaslan, Fatih
   Mermerkaya, Musa Ugur
   Cirakli, Alper
   Karaoglu, Sinan
   Duygulu, Fuat
TI Surgical versus conservative treatment following acute rupture of the
   Achilles tendon: is there a pedobarographic difference?
SO THERAPEUTICS AND CLINICAL RISK MANAGEMENT
LA English
DT Article
DE Achilles tendon; acute rupture; pedobarographic analysis
ID PLATELET-RICH PLASMA; NONOPERATIVE TREATMENT; TRIALS; MUSCLE
AB Introduction: Controversy remains regarding the optimal treatment method and postoperative rehabilitation of acute Achilles tendon ruptures. In this study, pedobarographic assessments of surgical and conservative treatments were compared.
   Material and methods: A prospective assessment was made of 16 patients (eight surgical, eight conservative) and eight healthy controls using a plantar pressure measurement system. Biomechanical gait parameters were obtained using the Footscan dynamic gait analysis system. Kruskal-Wallis and Mann-Whitney U-tests were used for the evaluation of data.
   Results: Nineteen males and five females were assessed, with an average age of 42.0 +/- 11.9 years. Follow-up was completed in 16 patients. No statistically significant difference was determined between the two treatment groups with regard to the gait analysis, but a difference was observed with the control group (P<0.001). All patients were able to resume their prior activities after 6 months and regained normal ranges of motion, with a high rate of satisfaction. Most of the patients (75%) were able to return to their pre-injury level of activities.
   Conclusion: Satisfactory results were obtained through conservative treatment of acute ruptures of the Achilles tendon. No significant differences or complications were observed in the group managed conservatively versus the group treated surgically. Further studies including 3D gait analyses and tendon biomechanical research are required to further investigate this issue.
C1 [Karaaslan, Fatih; Mermerkaya, Musa Ugur] Bozok Univ, Fac Med, Dept Orthopaed & Traumatol, 18 Madrasa Adnan Menderes,Blvd, TR-66200 Yozgat, Turkey.
   [Cirakli, Alper; Duygulu, Fuat] Kayseri Training Hosp, Dept Orthopaed & Traumatol, Kayseri, Turkey.
   [Karaoglu, Sinan] Mem Kayseri Hosp, Dept Orthopaed & Traumatol, Kayseri, Turkey.
RP Karaaslan, F (通讯作者)，Bozok Univ, Fac Med, Dept Orthopaed & Traumatol, 18 Madrasa Adnan Menderes,Blvd, TR-66200 Yozgat, Turkey.
EM fkaraaslan@gmail.com
RI mermerkaya, musa uğur/AFQ-4593-2022
OI mermerkaya, musa uğur/0000-0001-5539-2775
CR Cary David V, 2009, JAAPA, V22, P39
   CETTI R, 1993, AM J SPORT MED, V21, P791, DOI 10.1177/036354659302100606
   Clayton RAE, 2008, INJURY, V39, P1338, DOI 10.1016/j.injury.2008.06.021
   Costa ML, 2005, J BONE JOINT SURG BR, V87B, P1085, DOI 10.1302/0301-620X.87B8.16540
   Don R, 2007, CLIN BIOMECH, V22, P211, DOI 10.1016/j.clinbiomech.2006.10.001
   Filardo G, 2015, KNEE SURG SPORTS TRA
   Follak Niels, 2002, Eur J Orthop Surg Traumatol, V12, P90, DOI 10.1007/s00590-002-0020-y
   Foster TE, 2009, AM J SPORT MED, V37, P2259, DOI 10.1177/0363546509349921
   Holm C, 2015, SCAND J MED SCI SPOR, V25, pE1, DOI 10.1111/sms.12209
   Jallageas R, 2013, ORTHOP TRAUMATOL-SUR, V99, P577, DOI 10.1016/j.otsr.2013.03.024
   Jiang N, 2012, INT ORTHOP, V36, P765, DOI 10.1007/s00264-011-1431-3
   Kawakami Y, 2008, J ORTHOP SPORT PHYS, V38, P269, DOI 10.2519/jospt.2008.2632
   Keating JF, 2011, J BONE JOINT SURG BR, V93B, P1071, DOI 10.1302/0301-620X.93B8.25998
   Leppilahti J, 1998, SPORTS MED, V25, P79, DOI 10.2165/00007256-199825020-00002
   Middleton Kellie K, 2012, Iowa Orthop J, V32, P150
   Mishra A, 2012, CURR PHARM BIOTECHNO, V13, P1185, DOI 10.2174/138920112800624283
   Myerson M S, 1999, Instr Course Lect, V48, P219
   Nyyssonen T, 2008, SCAND J SURG, V97, P272, DOI 10.1177/145749690809700312
   Raisbeck CC, 2000, J BONE JOINT SURG AM, V82A, P1804, DOI 10.2106/00004623-200012000-00016
   Soroceanu A, 2012, J BONE JOINT SURG AM, V94A, P2136, DOI 10.2106/JBJS.K.00917
   Suchak AA, 2005, FOOT ANKLE INT, V26, P932, DOI 10.1177/107110070502601106
   Wilkins R, 2012, AM J SPORT MED, V40, P2154, DOI 10.1177/0363546512453293
NR 22
TC 6
Z9 8
U1 0
U2 3
PU DOVE MEDICAL PRESS LTD
PI ALBANY
PA PO BOX 300-008, ALBANY, AUCKLAND 0752, NEW ZEALAND
SN 1178-203X
J9 THER CLIN RISK MANAG
JI Therap. Clin. Risk Manag.
PY 2016
VL 12
BP 1311
EP 1315
DI 10.2147/TCRM.S116385
PG 5
WC Health Care Sciences & Services
WE Science Citation Index Expanded (SCI-EXPANDED)
SC Health Care Sciences & Services
GA DU5AI
UT WOS:000382223800001
PM 27621640
OA Green Published, gold, Green Submitted
DA 2022-07-22
ER

PT J
AU Kastoft, R
   Bencke, J
   Speedtsberg, MB
   Penny, JO
   Barfod, K
AF Kastoft, Rasmus
   Bencke, Jesper
   Speedtsberg, Merete B.
   Penny, Jeannette O.
   Barfod, Kristoffer
TI Early weight-bearing in nonoperative treatment of acute Achilles tendon
   rupture did not influence mid-term outcome: a blinded, randomised
   controlled trial
SO KNEE SURGERY SPORTS TRAUMATOLOGY ARTHROSCOPY
LA English
DT Article
DE Achilles tendon rupture; Conservative; Nonoperative; Early
   weight-bearing; Heel raise work; Heel raise height; Achilles Tendon
   Total Rupture Score; ATRS
ID NONSURGICAL TREATMENT; DYNAMIC TREATMENT; EARLY MOTION; IMMOBILIZATION;
   WEIGHTBEARING; SURGERY; REPAIR
AB Purpose Acute Achilles tendon rupture leads to impaired outcome at short term, but little is known concerning medium-term results. The aim of the present study was to investigate the influence of early weight-bearing on clinical outcome 4.5 years after nonoperative treatment of acute Achilles tendon rupture. Method The study was performed as a medium-term follow-up on patients included in a randomised controlled trial. Both groups were treated with nonoperative management and controlled early motion. The intervention group was allowed full weight-bearing from day 1, and the control group was non-weight-bearing for 6 weeks. 60 patients were randomised 1:1. Of those, 56 were eligible for inclusion in the medium-term follow-up and 37 participated (18 control, 19 intervention). The outcomes were Achilles tendon Total Rupture Score (ATRS) and heel raise work performed at an average of 4.5 years after the initial injury. Statistical analysis was performed using paired t tests and linear regression. Results No differences were found between control and intervention groups at 4.5 years. ATRS scores consistently improved in both the intervention and the control groups from 0.5 to 1 year and from 1 to 4.5 years, ending up at 80.5 on average after 4.5 years. Heel raise height kept improving from 0.5 to 4.5 years, reaching a limb symmetry index of 82.4%. The heel raise work improvement from 0.5 to 1 year did not continue. No significant change was found from 1 to 4.5 years, ending at a limb symmetry index of 60%. Conclusion Early weight-bearing did not influence outcome 4.5 years after nonoperative treatment of acute Achilles tendon rupture. The calf muscle of the injured limb performs 40% less work, when compared to the healthy limb, though lifting height recovers better.
C1 [Kastoft, Rasmus; Penny, Jeannette O.; Barfod, Kristoffer] Copenhagen Univ Hosp, Dept Orthoped Surg, Hvidovre, Denmark.
   [Bencke, Jesper; Speedtsberg, Merete B.] Copenhagen Univ Hosp, Lab Human Movement Anal, Dept Orthoped Surg, Hvidovre, Denmark.
RP Kastoft, R (通讯作者)，Copenhagen Univ Hosp, Dept Orthoped Surg, Hvidovre, Denmark.
EM Rasmus-kastoft@dadlnet.dk; Jesper.Bencke@regionh.dk;
   merete.brink.speedtsberg@regionh.dk; jopn@regionsjaelland.dk;
   kbarfod@dadlnet.dk
RI Barfod, Kristoffer/AAY-3288-2021; Bencke, Jesper/AAE-7914-2022
OI Barfod, Kristoffer/0000-0003-2620-5891; Kastoft,
   Rasmus/0000-0002-1133-7325
FU Department of Orthopaedic Surgery, Copenhagen University Hospital,
   Hvidovre, Denmark
FX The study was funded by Department of Orthopaedic Surgery, Copenhagen
   University Hospital, Hvidovre, Denmark, with no external funding, or
   involvement.
CR Barfod KW, 2015, J FOOT ANKLE SURG, V54, P220, DOI 10.1053/j.jfas.2014.11.018
   Barfod KW, 2014, J BONE JOINT SURG AM, V96A, P1497, DOI 10.2106/JBJS.M.01273
   De la Fuente Carlos, 2016, Foot (Edinb), V26, P15, DOI 10.1016/j.foot.2015.10.003
   El-Akkawi AI, 2017, J FOOT ANKLE SURG
   Ellison P, 2017, J FOOT ANKLE SURG, V56, P960, DOI 10.1053/j.jfas.2017.06.017
   Erickson BJ, 2015, ORTHOP J SPORTS MED, V3, DOI 10.1177/2325967115579188
   Ganestam A, 2016, KNEE SURG SPORT TR A, V24, P3730, DOI 10.1007/s00167-015-3544-5
   Ganestam A, 2013, J FOOT ANKLE SURG, V52, P736, DOI 10.1053/j.jfas.2013.07.004
   Gelberman RH, 1989, J HAND THER, V2, P66, DOI DOI 10.1016/S0894-1130(89)80044-4
   Heikkinen J, 2017, AM J SPORT MED, V45, P1395, DOI 10.1177/0363546517694610
   Korkmaz M, 2015, J ORTHOP SCI, V20, P513, DOI 10.1007/s00776-015-0710-z
   Lantto I, 2016, AM J SPORT MED, V44, P2406, DOI 10.1177/0363546516651060
   Lantto I, 2015, AM J SPORT MED, V43, P2302, DOI 10.1177/0363546515591267
   Lawrence JE, 2017, BONE JOINT J, V99B, P87, DOI 10.1302/0301-620X.99B1.BJJ-2016-0452.R1
   Mark-Christensen Troels, 2016, Knee Surg Sports Traumatol Arthrosc, V24, P1852, DOI 10.1007/s00167-014-3180-5
   Matsumoto F, 2003, ARCH PHYS MED REHAB, V84, P662, DOI 10.1016/S0003-9993(03)04834-7
   Mattila VM, 2015, BRIT J SPORT MED, V49, P1084, DOI 10.1136/bjsports-2013-092756
   Moller M, 2001, J BONE JOINT SURG BR, V83B, P843
   Mortensen NHM, 1999, J BONE JOINT SURG AM, V81A, P983, DOI 10.2106/00004623-199907000-00011
   Nilsson-Helander K, 2007, AM J SPORT MED, V35, P421, DOI 10.1177/0363546506294856
   Nilsson-Helander K, 2010, AM J SPORT MED, V38, P2186, DOI 10.1177/0363546510376052
   NISTOR L, 1981, J BONE JOINT SURG AM, V63, P394, DOI 10.2106/00004623-198163030-00012
   Olsson N, 2013, AM J SPORT MED, V41, P2867, DOI 10.1177/0363546513503282
   Olsson N, 2011, KNEE SURG SPORT TR A, V19, P1385, DOI 10.1007/s00167-011-1511-3
   Rosso C, 2015, KNEE SURG SPORT TR A, V23, P890, DOI 10.1007/s00167-013-2726-2
   Rosso C, 2013, KNEE SURG SPORT TR A, V21, P1369, DOI 10.1007/s00167-013-2407-1
   Silbernagel KG, 2012, AM J SPORT MED, V40, P1564, DOI 10.1177/0363546512447926
   Soroceanu A, 2012, J BONE JOINT SURG AM, V94A, P2136, DOI 10.2106/JBJS.K.00917
   Tengman T, 2013, ORTHOP J SPORTS MED, V1, DOI 10.1177/2325967113504734
   Twaddle BC, 2007, AM J SPORT MED, V35, P2033, DOI 10.1177/0363546507307503
NR 30
TC 16
Z9 16
U1 0
U2 6
PU SPRINGER
PI NEW YORK
PA 233 SPRING ST, NEW YORK, NY 10013 USA
SN 0942-2056
EI 1433-7347
J9 KNEE SURG SPORT TR A
JI Knee Surg. Sports Traumatol. Arthrosc.
PD SEP
PY 2019
VL 27
IS 9
BP 2781
EP 2788
DI 10.1007/s00167-018-5058-4
PG 8
WC Orthopedics; Sport Sciences; Surgery
WE Science Citation Index Expanded (SCI-EXPANDED)
SC Orthopedics; Sport Sciences; Surgery
GA IT5OW
UT WOS:000482915100009
PM 30030579
DA 2022-07-22
ER

PT J
AU Barfod, KW
   Bencke, J
   Lauridsen, HB
   Dippmann, C
   Ebskov, L
   Troelsen, A
AF Barfod, Kristoffer Weisskirchner
   Bencke, Jesper
   Lauridsen, Hanne Bloch
   Dippmann, Christian
   Ebskov, Lars
   Troelsen, Anders
TI Nonoperative, Dynamic Treatment of Acute Achilles Tendon Rupture:
   Influence of Early Weightbearing on Biomechanical Properties of the
   Plantar Flexor Muscle-Tendon Complex- A Blinded, Randomized, Controlled
   Trial
SO JOURNAL OF FOOT & ANKLE SURGERY
LA English
DT Article
DE Achilles tendon rupture; biomechanical properties; nonoperative
   treatment; weightbearing
ID MOBILIZATION; VALIDATION; REPAIR; MODEL
AB Acute Achilles tendon rupture alters the biomechanical properties of the plantar flexor muscle-tendon complex that can affect functional performance and the risk of repeat injury. The purpose of the present study was to compare the biomechanical properties of the plantar flexor muscle-tendon complex in patients randomized to early weightbearing or non-weightbearing in the nonoperative treatment of Achilles tendon rupture. A total of 60 patients were randomized to full weightbearing from day 1 of treatment or non-weightbearing for 6 weeks. After 6 and 12 months, the peak passive torque at 20 degrees dorsiflexion, the stiffness during slow stretching, and the maximal strength were measured in both limbs. The stiffness of the plantar flexor muscle-tendon complex in the terminal part of dorsiflexion was significantly increased (p = .024) in the non-weightbearing group at 12 months. The peak passive torque was significantly lower for the affected limb at 6 months (91%; p = .01), and the stiffness was significantly lower for the affected limb during the early part of dorsiflexion at 6 (67%; p < .001) and 12 (77%; p < .001) months. In conclusion, an increased stiffness of the plantar flexor muscle-tendon complex in the terminal part of dorsiflexion was found in the non-weightbearing group. The altered stiffness and strength in the affected limb could affect the coordination of gait and running. (C) 2015 by the American College of Foot and Ankle Surgeons. All rights reserved.
C1 [Barfod, Kristoffer Weisskirchner; Troelsen, Anders] Univ Copenhagen, Hvidovre Hosp, Dept Orthoped, Clin Orthoped Res Hvidovre, DK-2650 Hvidovre, Denmark.
   [Bencke, Jesper; Lauridsen, Hanne Bloch] Univ Copenhagen, Hvidovre Hosp, Dept Orthoped, Gait Anal Lab, DK-2650 Hvidovre, Denmark.
   [Dippmann, Christian; Ebskov, Lars] Univ Copenhagen, Hvidovre Hosp, Dept Orthoped, DK-2650 Hvidovre, Denmark.
RP Barfod, KW (通讯作者)，Univ Copenhagen, Hvidovre Hosp, Dept Orthoped, Clin Orthoped Res Hvidovre, Kettegard Alle 30, DK-2650 Hvidovre, Denmark.
EM kbarfod@dadlnet.dk
RI Bencke, Jesper/AAE-7914-2022; Barfod, Kristoffer/AAY-3288-2021
OI Barfod, Kristoffer/0000-0003-2620-5891; Dippmann,
   Christian/0000-0002-4990-6802; Troelsen, Anders/0000-0003-0132-8182
FU DJO Nordic
FX Limited grant support was received for the research from DJO Nordic. The
   funder had no role in study design, data collection and analysis,
   decision to publish, or preparation of the report.
CR Aspenberg P, 2007, INT ORTHOP, V31, P783, DOI 10.1007/s00264-007-0398-6
   Barfod KW, 2013, J FOOT ANKLE SURG, V52, P629, DOI 10.1053/j.jfas.2013.04.012
   Barfod KW, 2014, J BONE JOINT SURG AM, V96A, P1497, DOI 10.2106/JBJS.M.01273
   Bressel E, 2004, CLIN BIOMECH, V19, P284, DOI 10.1016/j.clinbiomech.2003.12.008
   Bressel E, 2001, AM J SPORT MED, V29, P321, DOI 10.1177/03635465010290031201
   Cavagna G A, 1977, Exerc Sport Sci Rev, V5, P89
   CHARLSON ME, 1987, J CHRON DIS, V40, P373, DOI 10.1016/0021-9681(87)90171-8
   Don R, 2007, CLIN BIOMECH, V22, P211, DOI 10.1016/j.clinbiomech.2006.10.001
   Eriksen HA, 2002, J ORTHOPAED RES, V20, P1352, DOI 10.1016/S0736-0266(02)00064-5
   Houshian S, 1998, INJURY, V29, P651, DOI 10.1016/S0020-1383(98)00147-8
   Krapf D, 2012, ORTHOPEDICS, V35, pE1383, DOI 10.3928/01477447-20120822-26
   Levi N, 1997, INJURY, V28, P311, DOI 10.1016/S0020-1383(96)00200-8
   Lin TW, 2004, J BIOMECH, V37, P865, DOI 10.1016/j.jbiomech.2003.11.005
   Mattila VM, 2013, BR J SPORTS MED
   McNair P, 2013, J ORTHOP RES, V31, P1469, DOI 10.1002/jor.22381
   McNair PJ, 1996, BRIT J SPORT MED, V30, P243, DOI 10.1136/bjsm.30.3.243
   Moller A, 1996, ACTA ORTHOP SCAND, V67, P479, DOI 10.3109/17453679608996672
   Nigg B M, 2000, BIOMECHANICS BIOL MO
   Nilsson-Helander K, 2007, AM J SPORT MED, V35, P421, DOI 10.1177/0363546506294856
   Nilsson-Helander K, 2010, AM J SPORT MED, V38, P2186, DOI 10.1177/0363546510376052
   Palmes D, 2002, J ORTHOPAED RES, V20, P939, DOI 10.1016/S0736-0266(02)00032-3
   Silbernagel KG, 2012, AM J SPORT MED, V40, P1564, DOI 10.1177/0363546512447926
   Silbernagel KG, 2012, J ORTHOP SPORT PHYS, V42, P521, DOI 10.2519/jospt.2012.3913
   SIMMONDS F A, 1957, Practitioner, V179, P56
   Suydam SM, 2013, KNEE SURG SPORTS TRA
   Thermann H, 2002, FOOT ANKLE INT, V23, P606, DOI 10.1177/107110070202300704
   Thermann H, 2001, FOOT ANKLE INT, V22, P478, DOI 10.1177/107110070102200604
   THOMPSON T C, 1962, J Trauma, V2, P126, DOI 10.1097/00005373-196203000-00003
   TOFT E, 1991, J NEUROPHYSIOL, V65, P1402, DOI 10.1152/jn.1991.65.6.1402
   VOIGT M, 1995, J BIOMECH, V28, P281, DOI 10.1016/0021-9290(94)00071-B
   Willerslev-Olsen M, 2013, DEV MED CHILD NEUROL, V55, P617, DOI 10.1111/dmcn.12124
NR 31
TC 20
Z9 21
U1 0
U2 10
PU ELSEVIER SCIENCE INC
PI NEW YORK
PA 360 PARK AVE SOUTH, NEW YORK, NY 10010-1710 USA
SN 1067-2516
EI 1542-2224
J9 J FOOT ANKLE SURG
JI J. Foot Ankle Surg.
PD MAR-APR
PY 2015
VL 54
IS 2
BP 220
EP 226
DI 10.1053/j.jfas.2014.11.018
PG 7
WC Orthopedics; Surgery
WE Science Citation Index Expanded (SCI-EXPANDED)
SC Orthopedics; Surgery
GA CC2IA
UT WOS:000350167500015
PM 25618802
DA 2022-07-22
ER

PT J
AU Noback, PC
   Jang, ES
   Cuellar, DO
   Seetharaman, M
   Malagoli, E
   Greisberg, JK
   Vosseller, JT
AF Noback, Peter C.
   Jang, Eugene S.
   Cuellar, Derly O.
   Seetharaman, Mani
   Malagoli, Emiliano
   Greisberg, Justin K.
   Vosseller, J. Turner
TI Risk factors for achilles tendon rupture: A matched case control study
SO INJURY-INTERNATIONAL JOURNAL OF THE CARE OF THE INJURED
LA English
DT Article
DE Achilles tendon rupture; Body mass index; Risk factors; Activity;
   Epidemiology
ID BODY-MASS INDEX; OVERWEIGHT; EPIDEMIOLOGY; PREDICTORS; INJURIES; ADULTS
AB Purpose: The purpose of this study was to elucidate whether body mass index (BMI), activity level, and other risk factors predispose patients to Achilles tendon ruptures.
   Materials and methods: A retrospective review of 279 subjects was performed (93 with Achilles tendon rupture, matched 1: 2 with 186 age/sex matched controls with ankle sprains). Demographic variables and risk factors for rupture were tabulated and compared.
   Results: The rupture group mean BMI was 27.77 (95% CI, 26.94-28.49), and the control group mean BMI was 26.66 (95% CI, 26.06-27.27). These populations were found to be statistically equivalent (p = 0.047 and p < 0.001 by two one-sided t-test). A significantly higher proportion of those suffering ruptures reported regular athletic activity at baseline (74%) versus controls (59%, p = 0.013).
   Conclusion: There was no clinically significant difference found in BMI between patients with ruptures and controls. Furthermore, it was found that patients who sustained ruptures were also more likely to be active at baseline than their ankle sprain counterparts. (C) 2017 Elsevier Ltd. All rights reserved.
C1 [Noback, Peter C.; Jang, Eugene S.; Cuellar, Derly O.; Seetharaman, Mani; Malagoli, Emiliano; Greisberg, Justin K.; Vosseller, J. Turner] Columbia Univ, Med Ctr, Dept Orthopaed Surg, 622 West 168th St,PH-1164, New York, NY 10032 USA.
RP Vosseller, JT (通讯作者)，Columbia Univ, Med Ctr, Dept Orthopaed Surg, 622 West 168th St,PH-1164, New York, NY 10032 USA.
EM jtv2111@cumc.columbia.edu
RI Vosseller, J. Turner/S-2479-2019
OI Vosseller, J. Turner/0000-0002-9639-3739; Jang,
   Eugene/0000-0002-9808-2678
CR Claessen FMAP, 2014, SPORTS MED, V44, P1241, DOI 10.1007/s40279-014-0200-z
   Cretnik A, 2004, WIEN KLIN WOCHENSCHR, V116, P33
   Davis JJ, 1999, MIL MED, V164, P872, DOI 10.1093/milmed/164.12.872
   de Gonzalez AB, 2010, NEW ENGL J MED, V363, P2211, DOI 10.1056/NEJMoa1000367
   Flegal KM, 2005, JAMA-J AM MED ASSOC, V293, P1861, DOI 10.1001/jama.293.15.1861
   Flood L, 2009, MED J AUSTRALIA, V190, P87, DOI 10.5694/j.1326-5377.2009.tb02285.x
   Fryar Cheryl D, 2012, Vital Health Stat 11, P1
   Garras DN, 2012, CLIN ORTHOP RELAT R, V470, P2268, DOI 10.1007/s11999-012-2355-y
   Gwynne-Jones DP, 2011, FOOT ANKLE INT, V32, P337, DOI 10.3113/FAI.2011.0337
   Jarvinen Tero A H, 2005, Foot Ankle Clin, V10, P255, DOI 10.1016/j.fcl.2005.01.013
   JOZSA L, 1989, AM J SPORT MED, V17, P338, DOI 10.1177/036354658901700305
   Kannus P, 1997, SCAND J MED SCI SPOR, V7, P107
   Kuczmarski RJ, 1997, OBES RES, V5, P542, DOI 10.1002/j.1550-8528.1997.tb00575.x
   Kujala UM, 2005, CLIN J SPORT MED, V15, P133, DOI 10.1097/01.jsm.0000165347.55638.23
   Leppilahti J, 1996, ACTA ORTHOP SCAND, V67, P277, DOI 10.3109/17453679608994688
   Levi N, 1997, INJURY, V28, P311, DOI 10.1016/S0020-1383(96)00200-8
   Maffulli N, 1999, CLIN J SPORT MED, V9, P157, DOI 10.1097/00042752-199907000-00007
   Moller A, 1996, ACTA ORTHOP SCAND, V67, P479, DOI 10.3109/17453679608996672
   Nilsson-Helander K, 2007, AM J SPORT MED, V35, P421, DOI 10.1177/0363546506294856
   Olsson N, 2014, AM J SPORT MED, V42, P1448, DOI 10.1177/0363546514527409
   Posthumus M, 2009, J SCI MED SPORT, V12, P184, DOI 10.1016/j.jsams.2007.12.006
   Seeger JD, 2006, PHARMACOEPIDEM DR S, V15, P784, DOI 10.1002/pds.1214
   Suchak AA, 2005, FOOT ANKLE INT, V26, P932, DOI 10.1177/107110070502601106
   Tallon C, 2001, MED SCI SPORT EXER, V33, P1983, DOI 10.1097/00005768-200112000-00002
   Trinh A, 2013, PEDIATRICS, V131, pE470, DOI 10.1542/peds.2012-1092
   van der Linden PD, 2003, ARCH INTERN MED, V163, P1801, DOI 10.1001/archinte.163.15.1801
   Vosseller JT, 2013, FOOT ANKLE INT, V34, P49, DOI 10.1177/1071100712460223
NR 27
TC 14
Z9 16
U1 0
U2 5
PU ELSEVIER SCI LTD
PI OXFORD
PA THE BOULEVARD, LANGFORD LANE, KIDLINGTON, OXFORD OX5 1GB, OXON, ENGLAND
SN 0020-1383
EI 1879-0267
J9 INJURY
JI Injury-Int. J. Care Inj.
PD OCT
PY 2017
VL 48
IS 10
BP 2342
EP 2347
DI 10.1016/j.injury.2017.08.050
PG 6
WC Critical Care Medicine; Emergency Medicine; Orthopedics; Surgery
WE Science Citation Index Expanded (SCI-EXPANDED)
SC General & Internal Medicine; Emergency Medicine; Orthopedics; Surgery
GA FL4UR
UT WOS:000414227500051
PM 28859844
DA 2022-07-22
ER

PT J
AU Alcelik, I
   Saeed, ZM
   Haughton, BA
   Shahid, R
   Alcelik, JC
   Brogden, C
   Budgen, A
AF Alcelik, Ilhan
   Saeed, Zubair M.
   Haughton, Ben A.
   Shahid, Rizwan
   Alcelik, John C.
   Brogden, Craig
   Budgen, Adam
TI Achillon versus open surgery in acute Achilles tendon repair
SO FOOT AND ANKLE SURGERY
LA English
DT Article
DE Achilles tendon rupture; Achillon; Open repair; Minimally invasive
   technique; Complication rate
ID RUPTURE; MANAGEMENT; INJURY; COMPLICATIONS; METAANALYSIS
AB Background: Open Achilles tendon repairs (OATR) are associated with high complication rates. Minimally invasive surgery (MIS) techniques like the Achillon Achilles tendon repair (AATR) were developed to reduce this. We performed a systematic review and meta-analysis to compare OATR with AATR.
   Methods: We performed an extensive literature search including all studies that compared the two techniques. Outcomes assessed included overall complication rate, re-rupture rate, sural nerve injury, wound length, The American Orthopaedic Foot and Ankle Scores (AOFAS) scores and return to sports.
   Results: Eight studies were suitable for inclusion totalling 210 patients in the AATR group vs 233 patients in the OATR group. Total complication rates were significantly reduced in the Achillon patients with odd ratio of 0.14 (CI 95%, 0.08-0.27, P < 0.00001) in favour. There were no significant differences in re-rupture rate, sural nerve injury, return to sports and AOFAS scores following repair between the two groups.
   Conclusions: AATR has fewer overall complications compared with OATR. It should be considered as an alternative to open surgical repair. (C) 2017 European Foot and Ankle Society. Published by Elsevier Ltd. All rights reserved.
C1 [Alcelik, Ilhan; Saeed, Zubair M.; Haughton, Ben A.; Shahid, Rizwan; Brogden, Craig; Budgen, Adam] York Hosp, Dept Orthopaed, Wigginton Rd, York YO31 8HE, N Yorkshire, England.
   [Alcelik, John C.] All St RC Sch, Year 12,Mill Mt Lane, York YO24 1BJ, N Yorkshire, England.
RP Alcelik, I (通讯作者)，York Hosp, Dept Orthopaed, Wigginton Rd, York YO31 8HE, N Yorkshire, England.
EM ialcelik67@gmail.com; zubair.saeed@doctors.org.uk;
   b.a.haughton@gmail.com; rizwanshahid@gmail.com; jalcelik@gmail.com;
   brogden@ntlworld.com; adam.budgen@btinternet.com
CR Aibinder WR, 2013, FOOT ANKLE INT, V34, P870, DOI 10.1177/1071100712473097
   Aktas S, 2009, FOOT ANKLE INT, V30, P391, DOI 10.3113/FAI.2009.0391
   Assal M, 2002, J BONE JOINT SURG AM, V84A, P161, DOI 10.2106/00004623-200202000-00001
   Avina Valencia Jorge Arturo, 2009, Acta Ortop Mex, V23, P125
   Bartel AFP, 2014, J FOOT ANKLE SURG, V53, P744, DOI 10.1053/j.jfas.2014.07.009
   Bhattacharyya M, 2009, INT ORTHOP, V33, P151, DOI 10.1007/s00264-008-0564-5
   Chan Alexander Pak-Hin, 2011, Sports Med Arthrosc Rehabil Ther Technol, V3, P32, DOI 10.1186/1758-2555-3-32
   Clanton TO, 2015, AM J SPORT MED, V43, P1957, DOI 10.1177/0363546515587082
   Daghino W, 2016, INJURY, V47, P2591, DOI 10.1016/j.injury.2016.09.009
   Del Buono A, 2014, BRIT MED BULL, V109, P45, DOI 10.1093/bmb/ldt029
   Feldbrin Z, 2010, ISR MED ASSOC J, V12, P609
   Gulati V, 2015, WORLD J ORTHOP, V6, P380, DOI 10.5312/wjo.v6.i4.380
   Handoll MM, 2008, COCHRANE BONE JOINT
   Higgins JPT, 2006, COCHRANE LIB, V4
   Hutchison AM, 2015, BONE JOINT J, V97B, P510, DOI 10.1302/0301-620X.97B4.35314
   Huttunen TT, 2014, AM J SPORT MED, V42, P2419, DOI 10.1177/0363546514540599
   Khan RJK, 2010, COCHRANE DB SYST REV, DOI 10.1002/14651858.CD003674.pub4
   Khan RJK, 2005, J BONE JOINT SURG AM, V87A, P2202, DOI 10.2106/JBJS.D.03049
   Kolodziej L, 2013, INT ORTHOP, V37, P625, DOI 10.1007/s00264-012-1737-9
   MANTEL N, 1959, J NATL CANCER I, V22, P719
   McMahon Samuel E, 2011, Foot Ankle Surg, V17, P211, DOI 10.1016/j.fas.2010.11.001
   Moher D, 2009, PLOS MED, V6, DOI 10.1371/journal.pmed.1000097
   Obada B, 2014, ARS MED TOMITANA, V3, P135
   Porter KJ, 2014, FOOT ANKLE SURG, V20, P90, DOI 10.1016/j.fas.2013.11.005
   Soroceanu A, 2012, J BONE JOINT SURG AM, V94A, P2136, DOI 10.2106/JBJS.K.00917
   Wallace RGH, 2011, J BONE JOINT SURG BR, V93B, P1362, DOI 10.1302/0301-620X.93B10.26187
   Wilkins R, 2012, AM J SPORT MED, V40, P2154, DOI 10.1177/0363546512453293
NR 27
TC 10
Z9 13
U1 0
U2 2
PU ELSEVIER
PI AMSTERDAM
PA RADARWEG 29, 1043 NX AMSTERDAM, NETHERLANDS
SN 1268-7731
EI 1460-9584
J9 FOOT ANKLE SURG
JI Foot Ankle Surg.
PD OCT
PY 2018
VL 24
IS 5
BP 427
EP 434
DI 10.1016/j.fas.2017.04.016
PG 8
WC Orthopedics
WE Science Citation Index Expanded (SCI-EXPANDED)
SC Orthopedics
GA GX4DA
UT WOS:000447676100011
PM 29409202
DA 2022-07-22
ER

PT J
AU Thermann, H
   Frerichs, O
   Holch, M
   Biewener, A
AF Thermann, H
   Frerichs, O
   Holch, M
   Biewener, A
TI Healing of Achilles tendon, an experimental study: Part 2 -
   Histological, immunohistological and ultrasonographic analysis
SO FOOT & ANKLE INTERNATIONAL
LA English
DT Article
DE Achilles tendon rupture; histology, immunmohistology; ultrasonography
ID SURGICAL-TREATMENT; III COLLAGEN; RUPTURE; DIAGNOSIS; RABBIT; INJURY
AB In 105 rabbits the course of healing was examined at one, two, four, eight and 12 weeks (21 rabbits per group) after an experimental Achilles tendon rupture. The following treatment modalities were compared: A) primary functional treatment; B) operative functional treatment (resorbable suture); and C) operative functional treatment with fibrin glue. For the functional (after)-treatment a special orthosis was applied. A 7.5 MHz Ultrasound probe was used for ultrasonographic evaluation. The histological specimens were stained in Masson-Goldner and Azan technique. Collagen Type III was depicted immunhistologically. A semiquantitative fibrocyte count was performed. The histological results showed a smooth healing in the primary functional treatment group (A), reaching parallel orientation of collagen fibers at 12 weeks. In the suture group (B), a secondary gapping of the tendon stumps was detectable after one week as in all other groups. In the fibrin group (C), the fibrin was resorbed after four weeks without essential influence to the course of healing. At 12 weeks the histological evaluation in all groups showed approximately normal tendon pattern. lmmunohistochemically, all groups showed cell-associated positive reactions for type-III collagen after one week with a maximum after two weeks. The semiquantitative fibrocyte count in the primary functional group showed a maximal number after one week. In the fibrin glue and suture groups the maximal number could be found after two weeks. Sonographically an increase in tendon thickness was detectable up to the fourth week in all groups. The secondary gapping of the tendon stumps in the suture group could also be detected sonographically. The echogenicity of the tendon during the course of healing showed increasing homogeneity and parallelism in all groups. At 12 weeks the echogenicity was comparable in all groups. The experiment suggests the equivalence of primary functional treatment to a combination of operative and functional therapy in Achilles tendon rupture.
C1 GustavCarus Univ Dresden, Klin Unfall & Wiederherstellungschirurg, Dresden, Germany.
RP Thermann, H (通讯作者)，Atos Praxisklin, Bismarckstr 9-15, D-69115 Heidelberg, Germany.
RI Biewener, Andrew/O-2989-2016
OI Biewener, Andrew/0000-0003-3303-8737
CR BAILEY AJ, 1975, BIOCHEM BIOPH RES CO, V66, P1160, DOI 10.1016/0006-291X(75)90480-5
   BLEI CL, 1986, RADIOLOGY, V159, P765, DOI 10.1148/radiology.159.3.3517960
   BOSCH P, 1981, ARCH ORTHOP TRAUM SU, V98, P305, DOI 10.1007/BF00378887
   BROWN TD, 1981, J TRAUMA, V21, P951, DOI 10.1097/00005373-198111000-00007
   BURCHHARDT H, 1992, Z ORTHOP GRENZGEB, V130, P109, DOI 10.1055/s-2008-1040122
   DATE T, 1986, Journal of the Japanese Orthopaedic Association, V60, P449
   ENWEMEKA CS, 1988, AM J PHYS MED REHAB, V67, P264
   FLEISCHMAJER R, 1980, J INVEST DERMATOL, V75, P189, DOI 10.1111/1523-1747.ep12522644
   FORNAGE BD, 1986, RADIOLOGY, V159, P759, DOI 10.1148/radiology.159.3.3517959
   FRANK C, 1983, AM J SPORT MED, V11, P379, DOI 10.1177/036354658301100602
   HALBROCK HF, 1986, ORTHOPADIE, V1, P17
   KAINBERGER FM, 1990, AM J ROENTGENOL, V155, P1031, DOI 10.2214/ajr.155.5.2120931
   LIPSCOMP PR, 1961, STAFF M MAYO CLIN, V36, P277
   MATHIESON JR, 1988, AM J ROENTGENOL, V151, P127
   MILBRADT H, 1988, RADIOLOGE, V28, P330
   MOHR W, 1987, SPEZIELLE PATHOLOGIS, V19, P122
   MUNETA T, 1992, T ORTHOP RES SOC, V17, P672
   NISTOR L, 1981, J BONE JOINT SURG AM, V63, P394, DOI 10.2106/00004623-198163030-00012
   NYSTROM B, 1983, ACTA CHIR SCAND, V149, P669
   POSTACCHINI F, 1978, CELL TISSUE RES, V195, P81
   REINERT KH, 1987, ENVIRON TOXICOL CHEM, V6, P99
   ROBERTS JM, 1985, AM J SPORTS MED, V13, P242
   SCHATZKER J, 1969, ACTA ORTHOP SCAND S, P3
   SJOSTROM M, 1983, VIRCHOWS ARCH A, V399, P177, DOI 10.1007/BF00619578
   THERMANN H, 1989, UNFALLCHIRURG, V92, P266
   THERMANN H, 1995, UNFALLCHIRURG, V98, P21
   THERMANN H, 1995, STUDIE HEFTE UNFALLC, V248
   THERMANN H, 1992, FOOT ANKLE, V12, P321
   VIDLIK A, 1969, ACTA ORTHOP SCAND, V40, P261
   WILLIAMS IF, 1984, CONNECT TISSUE RES, V12, P211, DOI 10.3109/03008208409013684
NR 30
TC 23
Z9 25
U1 1
U2 4
PU AMER ORTHOPAEDIC FOOT & ANKLE SOC, INC
PI SEATTLE
PA 2517 EASTLAKE AVE EAST, STE 200, SEATTLE, WA 98102 USA
SN 1071-1007
J9 FOOT ANKLE INT
JI Foot Ankle Int.
PD JUL
PY 2002
VL 23
IS 7
BP 606
EP 613
DI 10.1177/107110070202300704
PG 8
WC Orthopedics
WE Science Citation Index Expanded (SCI-EXPANDED)
SC Orthopedics
GA 573XV
UT WOS:000176858500004
PM 12146770
DA 2022-07-22
ER

PT J
AU Freedman, BR
   Sarver, JJ
   Buckley, MR
   Voleti, PB
   Soslowsky, LJ
AF Freedman, Benjamin R.
   Sarver, Joseph J.
   Buckley, Mark R.
   Voleti, Pramod B.
   Soslowsky, Louis J.
TI Biomechanical and structural response of healing Achilles tendon to
   fatigue loading following acute injury
SO JOURNAL OF BIOMECHANICS
LA English
DT Article
DE Achilles tendon; Fatigue mechanics; Damage; Healing; Imaging; Foot and
   ankle; Injury
ID MECHANICAL-PROPERTIES; DAMAGE; LIGAMENT; RUPTURE; MODEL
AB Achilles tendon injuries affect both athletes and the general population, and their incidence is rising. In particular, the Achilles tendon is subject to dynamic loading at or near failure loads during activity, and fatigue induced damage is likely a contributing factor to ultimate tendon failure. Unfortunately, little is known about how injured Achilles tendons respond mechanically and structurally to fatigue loading during healing. Knowledge of these properties remains critical to best evaluate tendon damage induction and the ability of the tendon to maintain mechanical properties with repeated loading. Thus, this study investigated the mechanical and structural changes in healing mouse Achilles tendons during fatigue loading. Twenty four mice received bilateral full thickness, partial width excisional injuries to their Achilles tendons (IACUC approved) and twelve tendons from six uninjured mice were used as controls. Tendons were fatigue loaded to assess mechanical and structural properties simultaneously after 0, 1, 3, and 6 weeks of healing using an integrated polarized light system. Results showed that the number of cycles to failure decreased dramatically (37-fold, p < 0.005) due to injury, but increased throughout healing, ultimately recovering after 6 weeks. The tangent stiffness, hysteresis, and dynamic modulus did not improve with healing (p < 0.005). Linear regression analysis was used to determine relationships between mechanical and structural properties. Of tendon structural properties, the apparent birefringence was able to best predict dynamic modulus (R-2=0.88-0.92) throughout healing and fatigue life. This study reinforces the concept that fatigue loading is a sensitive metric to assess tendon healing and demonstrates potential structural metrics to predict mechanical properties. (C) 2013 Elsevier Ltd. All rights reserved.
C1 [Freedman, Benjamin R.; Sarver, Joseph J.; Buckley, Mark R.; Voleti, Pramod B.; Soslowsky, Louis J.] Univ Penn, McKay Orthopaed Res Lab, Philadelphia, PA 19104 USA.
   [Buckley, Mark R.] Univ Rochester, Dept Biomed Engn, Rochester, NY USA.
RP Soslowsky, LJ (通讯作者)，Univ Penn, McKay Orthopaed Res Lab, 424 Stemmler Hall,3450 Hamilton Walk, Philadelphia, PA 19104 USA.
EM soslowsk@upenn.edu
OI Buckley, Mark/0000-0002-6938-2110
FU NIH/NIAMS [P30 AR050950]; National Science Foundation Graduate Research
   Fellowship; NATIONAL INSTITUTE OF ARTHRITIS AND MUSCULOSKELETAL AND SKIN
   DISEASES [P30AR050950, R01AR064216] Funding Source: NIH RePORTER
FX This study was funded by an NIH/NIAMS supported Penn Center for
   Musculoskeletal Disorders (NIH, P30 AR050950) and a National Science
   Foundation Graduate Research Fellowship.
CR AAOS, 2009, GUID DIAGN TREATM AC
   Ahmadzadeh H, 2013, J BIOMECH, V46, P2497, DOI 10.1016/j.jbiomech.2013.07.008
   Andarawis-Puri N, 2012, J ORTHOP RES, V30, P1327, DOI 10.1002/jor.22059
   Beason D.P., 2012, J BIOMECH
   Buckley MR, 2013, J BIOMECH, V46, P2242, DOI 10.1016/j.jbiomech.2013.06.029
   Buckley MR, 2013, J BIOMECH ENG, V135
   Carpenter JE, 1998, J SHOULDER ELB SURG, V7, P599, DOI 10.1016/S1058-2746(98)90007-6
   Chiodo CP, 2010, J AM ACAD ORTHOP SUR, V18, P503, DOI 10.5435/00124635-201008000-00007
   Dourte LM, 2012, J BIOMECH ENG-T ASME, V134, DOI 10.1115/1.4006200
   Duenwald-Kuehl S, 2012, ANN BIOMED ENG, V40, P1692, DOI 10.1007/s10439-012-0538-z
   Dunkman AA, 2013, MATRIX BIOL, V32, P3, DOI 10.1016/j.matbio.2012.11.005
   Favata M., 2006, SCARLESS HEALING FET
   Fessel G, 2009, MATRIX BIOL, V28, P503, DOI 10.1016/j.matbio.2009.08.002
   FUKASHIRO S, 1995, EUR J APPL PHYSIOL O, V71, P453, DOI 10.1007/BF00635880
   Fung DT, 2010, J BIOMECH, V43, P274, DOI 10.1016/j.jbiomech.2009.08.039
   Fung DT, 2009, J ORTHOP RES, V27, P264, DOI 10.1002/jor.20722
   Gimbel JA, 2007, J BIOMECH ENG-T ASME, V129, P400, DOI 10.1115/1.2721075
   Glazer AM, 1996, P ROY SOC A-MATH PHY, V452, P2751, DOI 10.1098/rspa.1996.0145
   Ikoma K., 2013, J ORTHOP RES
   KOMI PV, 1992, CLIN SPORT MED, V11, P521
   Lake SP, 2009, J ORTHOP RES, V27, P1596, DOI 10.1002/jor.20938
   Legerlotz K, 2011, SCAND J MED SCI SPOR
   Maffulli N, 1999, CLIN J SPORT MED, V9, P157, DOI 10.1097/00042752-199907000-00007
   Miller KS, 2012, J BIOMECH, V45, P2061, DOI 10.1016/j.jbiomech.2012.06.006
   Nagase S, 2013, PLOS ONE, V8, DOI 10.1371/journal.pone.0058716
   Parent G, 2011, ANN BIOMED ENG, V39, P1535, DOI 10.1007/s10439-011-0254-0
   Provenzano PP, 2006, MATRIX BIOL, V25, P71, DOI 10.1016/j.matbio.2005.09.005
   Provenzano PP, 2002, J APPL PHYSIOL, V92, P362, DOI 10.1152/jappl.2002.92.1.362
   Quinn KP, 2008, J APPL PHYSIOL, V105, P1881, DOI 10.1152/japplphysiol.90792.2008
   Schechtman H, 2002, J BIOMECH, V35, P347, DOI 10.1016/S0021-9290(01)00177-4
   Schechtman H, 1997, J BIOMECH, V30, P829, DOI 10.1016/S0021-9290(97)00033-X
   Sereysky JB, 2012, J ORTHOP RES, V30, P965, DOI 10.1002/jor.22012
   Sereysky JB, 2010, J BIOMECH, V43, P2641, DOI 10.1016/j.jbiomech.2010.04.043
   Shepherd JH, 2013, INT J EXP PATHOL, V94, P260, DOI 10.1111/iep.12037
   Suchak AA, 2005, FOOT ANKLE INT, V26, P932, DOI 10.1177/107110070502601106
   Silva DFT, 2013, ANN BIOMED ENG, V41, P752, DOI 10.1007/s10439-012-0720-3
   Timoshenko S, 1969, THEORY ELASTICITY
   Veres SP, 2013, J ORTHOP RES, V31, P731, DOI 10.1002/jor.22292
   Veres SP, 2012, BIOPHYS J, V102, P2876, DOI 10.1016/j.bpj.2012.05.022
   WANG XT, 1995, J EXP BIOL, V198, P847
   Wren TAL, 2003, ANN BIOMED ENG, V31, P710, DOI 10.1114/1.1569267
   Yamanari M, 2012, PLOS ONE, V7, DOI 10.1371/journal.pone.0044026
NR 42
TC 47
Z9 48
U1 0
U2 24
PU ELSEVIER SCI LTD
PI OXFORD
PA THE BOULEVARD, LANGFORD LANE, KIDLINGTON, OXFORD OX5 1GB, OXON, ENGLAND
SN 0021-9290
EI 1873-2380
J9 J BIOMECH
JI J. Biomech.
PD JUN 27
PY 2014
VL 47
IS 9
SI SI
BP 2028
EP 2034
DI 10.1016/j.jbiomech.2013.10.054
PG 7
WC Biophysics; Engineering, Biomedical
WE Science Citation Index Expanded (SCI-EXPANDED)
SC Biophysics; Engineering
GA AK7QD
UT WOS:000338621900013
PM 24280564
OA Green Accepted
DA 2022-07-22
ER

PT J
AU Manegold, S
   Tsitsilonis, S
   Schumann, J
   Gehlen, T
   Agres, AN
   Keller, J
   Gesslein, M
   Wichlas, F
AF Manegold, Sebastian
   Tsitsilonis, Serafim
   Schumann, Jakob
   Gehlen, Tobias
   Agres, Alison N.
   Keller, Johannes
   Gesslein, Markus
   Wichlas, Florian
TI Functional outcome and complication rate after percutaneous suture of
   fresh Achilles tendon ruptures with the Dresden instrument
SO JOURNAL OF ORTHOPAEDICS AND TRAUMATOLOGY
LA English
DT Article
DE Achilles tendon; Rupture; Percutaneous; Minimal invasive
ID OPEN REPAIR; FOLLOW-UP; METAANALYSIS; STRENGTH; SURGERY
AB BackgroundThe aim of this study was to evaluate the outcome of patients with a rupture of the Achilles tendon (ATR) treated percutaneously with the Dresden instrument in the hands of surgeons others than its inventors.Materials and methods118 patients (FU rate: 77.1%) with an acute ATR treated with the Dresden instrument were retrospectively evaluated. The following data were evaluated: pain intensity, functional limitation, Hannover score, Achilles tendon total rupture score (ATRS), AOFAS ankle-hindfoot score, Tegner activity score, complications, maximum calf circumference (MCC) on both sides, and the Matles test for tendon lengthening. The effect of the time point of the surgery after trauma was examined.ResultsHannover scores and ATRSs were good; AOFAS scores were excellent. Almost all patients returned to sporting activities postoperatively, and 66.1% were able to return to their previous level. The Tegner activity score revealed a slight posttraumatic decrease (p=0.009) in the level of physical activity overall (pre-injury: 5.370.15; postoperatively: 4.77 +/- 0.15). The re-rupture rate was 2%. No sural nerve lesions and no infections were reported. Even after 3years, there was still a difference in MCC that was correlated with inferior clinical score and AT lengthening. Patients treated within the first 2days after ATR showed inferior clinical outcomes in terms of AOFAS score, ATRS, and functional limitations.ConclusionsPercutaneous ATR suture with the Dresden instrument is a safe and reliable method. Low complication and re-rupture rates, good clinical results, and a high rate of return to play support this fact. The time point of the operation may influence the outcome.
C1 [Manegold, Sebastian; Tsitsilonis, Serafim; Schumann, Jakob; Gehlen, Tobias; Keller, Johannes] Charite Univ Med Berlin, Ctr Musculoskeletal Surg, Campus Virchow Clin, Augustenburger Pl 1, D-13353 Berlin, Germany.
   [Agres, Alison N.] Charite Univ Med Berlin, Julius Wolff Inst, Augustenburger Pl 1, D-13353 Berlin, Germany.
   [Gesslein, Markus] Paracelsus Univ, Clin Orthopaed & Traumatol, Klinikum Nurnberg Sud, Breslauer Str 201, D-90471 Nurnberg, Germany.
   [Wichlas, Florian] Univ Salzburg, Clin Orthopaed & Traumatol, Mullner Hauptstr 48, Salzburg, Austria.
RP Tsitsilonis, S (通讯作者)，Charite Univ Med Berlin, Ctr Musculoskeletal Surg, Campus Virchow Clin, Augustenburger Pl 1, D-13353 Berlin, Germany.
EM sebastian.manegold@charite.de; serafeim.tsitsilonis@charite.de
RI Geßlein, Markus/O-4858-2019
OI Geßlein, Markus/0000-0003-3863-1580; Agres, Alison/0000-0002-9620-5689
FU German Research Foundation (DFG); Open Access Publication Fund of
   Charite-Universitatsmedizin Berlin
FX We acknowledge support from the German Research Foundation (DFG) and the
   Open Access Publication Fund of Charite-Universitatsmedizin Berlin.
CR Agres AN, 2015, SCAND J MED SCI SPOR, V25, P860, DOI 10.1111/sms.12456
   Amlang MH, 2010, UNFALLCHIRURG, V113, P712, DOI 10.1007/s00113-010-1809-5
   Amlang MH, 2005, UNFALLCHIRURG, V108, P529, DOI 10.1007/s00113-005-0938-8
   Amlang Michael H, 2006, Oper Orthop Traumatol, V18, P287, DOI 10.1007/s00064-006-1178-y
   Assal M, 2002, J BONE JOINT SURG AM, V84A, P161, DOI 10.2106/00004623-200202000-00001
   Bartel AFP, 2014, J FOOT ANKLE SURG, V53, P744, DOI 10.1053/j.jfas.2014.07.009
   Chmielnicki M, 2016, Z ORTHOP UNFALLCHIR, V154, P303, DOI 10.1055/s-0042-104513
   Davies H, 2017, INJURY, V48, P781, DOI 10.1016/j.injury.2017.01.018
   Del Buono A, 2014, BRIT MED BULL, V109, P45, DOI 10.1093/bmb/ldt029
   Don R, 2007, CLIN BIOMECH, V22, P211, DOI 10.1016/j.clinbiomech.2006.10.001
   Ebinesan AD, 2008, DISABIL REHABIL, V30, P1721, DOI 10.1080/09638280701786815
   Eliasson P, 2009, J APPL PHYSIOL, V107, P399, DOI 10.1152/japplphysiol.91563.2008
   Gerber C, 2007, J SHOULDER ELB SURG, V16, P691, DOI 10.1016/j.jse.2007.02.122
   Gigante A, 2008, KNEE SURG SPORT TR A, V16, P204, DOI 10.1007/s00167-007-0448-z
   Hunt Kenneth J, 2014, Foot Ankle Spec, V7, P199, DOI 10.1177/1938640014533478
   Huttunen TT, 2014, AM J SPORT MED, V42, P2419, DOI 10.1177/0363546514540599
   Jiang N, 2012, INT ORTHOP, V36, P765, DOI 10.1007/s00264-011-1431-3
   JOZSA L, 1989, AM J SPORT MED, V17, P338, DOI 10.1177/036354658901700305
   KAKIUCHI M, 1995, J BONE JOINT SURG BR, V77B, P60, DOI 10.1302/0301-620X.77B1.7822398
   Keller A, 2014, AM J SPORT MED, V42, P731, DOI 10.1177/0363546513511418
   Khan RJK, 2010, COCHRANE DB SYST REV, DOI 10.1002/14651858.CD003674.pub4
   Khan RJK, 2005, J BONE JOINT SURG AM, V87A, P2202, DOI 10.2106/JBJS.D.03049
   Lantto I, 2015, SCAND J MED SCI SPOR, V25, pE133, DOI 10.1111/sms.12253
   Leppilahti J, 2000, FOOT ANKLE INT, V21, P330, DOI 10.1177/107110070002100410
   Lim J, 2001, FOOT ANKLE INT, V22, P559, DOI 10.1177/107110070102200705
   MA GWC, 1977, CLIN ORTHOP RELAT R, P247
   Maffulli N, 2010, CLIN ORTHOP RELAT R, V468, P1039, DOI 10.1007/s11999-009-0944-1
   Mullaney MJ, 2006, AM J SPORT MED, V34, P1120, DOI 10.1177/0363546505284186
   Nilsson-Helander K, 2007, AM J SPORT MED, V35, P421, DOI 10.1177/0363546506294856
   Nilsson-Helander K, 2010, AM J SPORT MED, V38, P2186, DOI 10.1177/0363546510376052
   Olsson N, 2014, AM J SPORT MED, V42, P1448, DOI 10.1177/0363546514527409
   Olsson N, 2011, KNEE SURG SPORT TR A, V19, P1385, DOI 10.1007/s00167-011-1511-3
   Parekh Selene G, 2009, Foot Ankle Spec, V2, P283, DOI 10.1177/1938640009351138
   Rosso C, 2015, KNEE SURG SPORT TR A, V23, P890, DOI 10.1007/s00167-013-2726-2
   Rosso C, 2013, KNEE SURG SPORT TR A, V21, P1369, DOI 10.1007/s00167-013-2407-1
   Thermann H, 1999, ORTHOPADE, V28, P82, DOI 10.1007/s001320050325
   THERMANN H, 1995, UNFALLCHIRURG, V98, P21
   Valente Maurizio, 2012, Musculoskelet Surg, V96, P35, DOI 10.1007/s12306-011-0158-z
   Willits K, 2010, J BONE JOINT SURG AM, V92A, P2767, DOI 10.2106/JBJS.I.01401
   Zellers JA, 2016, BRIT J SPORT MED, V50, P1325, DOI 10.1136/bjsports-2016-096106
NR 41
TC 8
Z9 9
U1 0
U2 11
PU SPRINGER
PI NEW YORK
PA ONE NEW YORK PLAZA, SUITE 4600, NEW YORK, NY, UNITED STATES
SN 1590-9921
EI 1590-9999
J9 J ORTHOP TRAUMATOL
JI J. Orthop. Traumatol.
PD DEC
PY 2018
VL 19
IS 1
AR 19
DI 10.1186/s10195-018-0511-1
PG 7
WC Orthopedics
WE Science Citation Index Expanded (SCI-EXPANDED)
SC Orthopedics
GA GU0OB
UT WOS:000444949200001
PM 30229505
OA Green Published, gold
DA 2022-07-22
ER

PT J
AU Metineren, H
   Dulgeroglu, TC
   Metineren, MH
   Aydin, E
   Kocak, C
AF Metineren, Hasan
   Dulgeroglu, Turan Cihan
   Metineren, Mehmet Huseyin
   Aydin, Ekrem
   Kocak, Cengiz
TI Effectiveness of platelet-rich fibrin on tendon healing: experimental
   study in a rat model
SO INTERNATIONAL JOURNAL OF CLINICAL AND EXPERIMENTAL MEDICINE
LA English
DT Article
DE Platelet rich fibrin; Achilles tendon; platelet rich plasma; tendon
   injury; experimental model
ID ACHILLES-TENDON; NONOPERATIVE TREATMENT; PLASMA PRP; AUGMENTATION;
   TRANSMISSION; MEDICINE; RUPTURES; INJURY; REPAIR
AB Platelet Rich Fibrin (PRF) is a new biological augmentation material which is remarkable with its rich cellular components and growth factors. The aim of this study was to evaluate the effect of PRF on tendon healing in a rat model. PRF and control groups included eight rats. PRF was obtained with centrifuging blood collected from the rats and applied on the tendon repair site in Achilles tendon injury model created in rats. Effectiveness of PRF was evaluated with histological and immuno-histochemical methods. PRF improves formation of regular collagen structures (P<0.01) and also increases vascularity (P<0.05) of the tendon repair site. Cartilage formation occurred both in PRF and control groups. Gap formation was observed only in the control group. PRF is an autogenous biomaterial which enhances and support tendon healing.
C1 [Metineren, Hasan; Dulgeroglu, Turan Cihan; Aydin, Ekrem] Dumlupinar Univ, Fac Med, Dept Orthopaed & Traumatol, Kutahya, Turkey.
   [Metineren, Mehmet Huseyin; Kocak, Cengiz] Dumlupinar Univ, Fac Med, Dept Pathol, Kutahya, Turkey.
RP Metineren, H (通讯作者)，Dumlupinar Univ, Fac Med, Evliya Celebi Campus, TR-43100 Kutahya, Turkey.
EM drhmetin19@gmail.com
FU Dumlupinar University Scientific Research Project Supporting Fund
FX This project was supported by Dumlupinar University Scientific Research
   Project Supporting Fund.
CR Bielecki T, 2012, CURR PHARM BIOTECHNO, V13, P1121, DOI 10.2174/138920112800624292
   Choueka J, 2000, J HAND SURG-AM, V25A, P1127, DOI 10.1053/jhsu.2000.20155
   Choukroun J, 2006, ORAL SURG ORAL MED O, V101, P299, DOI 10.1016/j.tripleo.2005.07.012
   CONRAD EU, 1995, J BONE JOINT SURG AM, V77A, P214, DOI 10.2106/00004623-199502000-00007
   Dietrich F, 2015, WORLD J PLAST SURG, V4, P101
   Everts Peter A M, 2006, J Extra Corpor Technol, V38, P174
   George JN, 2000, LANCET, V355, P1531, DOI 10.1016/S0140-6736(00)02175-9
   Ghanaati S, 2014, J ORAL IMPLANTOL, V40, P679, DOI 10.1563/aaid-joi-D-14-00138
   Harry Lorraine E., 2003, Birth Defects Research, V69, P363, DOI 10.1002/bdrc.10024
   Khanna A, 2009, BRIT MED BULL, V90, P85, DOI 10.1093/bmb/ldp013
   Kim TH, 2014, ARCH ORAL BIOL, V59, P550, DOI 10.1016/j.archoralbio.2014.02.004
   Lemaire R, 2000, J BONE JOINT SURG BR, V82B, P313, DOI 10.1302/0301-620X.82B3.14194
   Lo IKY, 1997, CLIN J SPORT MED, V7, P207, DOI 10.1097/00042752-199707000-00010
   Longo UG, 2011, BRIT MED BULL, V98, P31, DOI 10.1093/bmb/ldq030
   Ma JJ, 2007, J BONE JOINT SURG AM, V89A, P2241, DOI 10.2106/JBJS.D.03054
   Maffulli N, 2010, EXPERT OPIN PHARMACO, V11, P2177, DOI 10.1517/14656566.2010.495715
   Molloy T, 2003, SPORTS MED, V33, P381, DOI 10.2165/00007256-200333050-00004
   Rodeo SA, 2012, AM J SPORT MED, V40, P1234, DOI 10.1177/0363546512442924
   Rosenbaum Andrew J, 2010, HSS J, V6, P164, DOI 10.1007/s11420-009-9152-5
   Sharma P, 2005, J BONE JOINT SURG AM, V87A, P187, DOI 10.2106/JBJS.D.01850
   Sharma P, 2005, SURG-J R COLL SURG E, V3, P309, DOI 10.1016/S1479-666X(05)80109-X
   Sharma Pankaj, 2005, Foot Ankle Clin, V10, P383, DOI 10.1016/j.fcl.2005.01.005
   Shim JW, 2006, BIOMECH MODEL MECHAN, V5, P247, DOI 10.1007/s10237-005-0013-y
   Tilak Raj TN., 2011, J DENT SCI RES, V2, P50
   Willits K, 2010, J BONE JOINT SURG AM, V92A, P2767, DOI 10.2106/JBJS.I.01401
   Zumstein MA, 2014, J SHOULDER ELB SURG, V23, P3, DOI 10.1016/j.jse.2013.08.017
   Zumstein MA, 2011, OPER TECHN SPORT MED, V19, P190, DOI 10.1053/j.otsm.2011.01.001
NR 27
TC 2
Z9 2
U1 0
U2 5
PU E-CENTURY PUBLISHING CORP
PI MADISON
PA 40 WHITE OAKS LN, MADISON, WI 53711 USA
SN 1940-5901
J9 INT J CLIN EXP MED
JI Int. J. Clin. Exp. Med.
PY 2016
VL 9
IS 7
BP 14260
EP 14265
PG 6
WC Medicine, Research & Experimental
WE Science Citation Index Expanded (SCI-EXPANDED)
SC Research & Experimental Medicine
GA EA2MT
UT WOS:000386428400223
DA 2022-07-22
ER

PT J
AU Del Buono, A
   Volpin, A
   Maffulli, N
AF Del Buono, Angelo
   Volpin, Andrea
   Maffulli, Nicola
TI Minimally invasive versus open surgery for acute Achilles tendon
   rupture: a systematic review
SO BRITISH MEDICAL BULLETIN
LA English
DT Article
DE Achilles tendon rupture; minimally invasive surgery; open repair surgery
ID NON-SURGICAL TREATMENT; PERCUTANEOUS REPAIR; NONOPERATIVE TREATMENT;
   NONSURGICAL TREATMENT; COMPLICATIONS; METAANALYSIS; MULTICENTER;
   MANAGEMENT
AB Introduction: This review provides a comprehensive description of clinical, functional outcomes, and complications after open and minimally invasive surgery for Achilles tendon ruptures.
   Sources of data: We systematically searched Medline (PubMED), EMBASE, CINHAL, Cochrane, Sports Discus and Google scholar databases using the combined keywords 'open repair', 'percutaneous surgery', 'minimally invasive surgery' 'Achilles tendon rupture', 'complications', 'infections', 'wound disorders' to identify articles published in English, Spanish, French and Italian.
   Areas of agreement: Twelve studies fulfilled our inclusion criteria. Six studies were retrospective, five were randomized controlled trials and one was a prospective investigation. Of a total of 781 patients, 375 underwent open repair and 406 percutaneous surgery. Different procedures were performed for open and minimally invasive repair.
   Areas of controversy: The range of motion was significantly greater after percutaneous repair than open surgery. The number of complications that occurred after open surgery was higher than after minimally invasive surgery.
   Growing points: Minimally invasive surgery is less expensive and less time demanding.
   Areas timely for developing research: Minimally invasive and open surgery of the Achilles tendon are grossly equivalent. However, iatrogenic neurological complications are more frequent after percutaneous repair. Novel percutaneous repairs have been proposed to minimize the risk of sural nerve injury.
C1 [Del Buono, Angelo] Campus Biomed Univ Rome, Dept Orthopaed & Trauma Surg, Rome, Italy.
   [Volpin, Andrea] Univ Padua, Dept Orthopaed & Trauma Surg, I-35128 Padua, Italy.
   [Volpin, Andrea] Addenbrookes Hosp, Dept Trauma & Orthopaed, Cambridge CB2 0QQ, England.
   [Maffulli, Nicola] Mile End Hosp, Barts & London Sch Med & Dent, Ctr Sports & Exercise Med, London E1 4DG, England.
   [Maffulli, Nicola] Univ Salerno, Chair Musculoskeletal Disorders, I-84100 Salerno, Italy.
RP Maffulli, N (通讯作者)，Mile End Hosp, Barts & London Sch Med & Dent, Ctr Sports & Exercise Med, 275 Bancroft Rd, London E1 4DG, England.
EM n.maffulli@qmul.ac.uk
RI Maffulli, Nicola/A-1839-2014
OI Maffulli, Nicola/0000-0002-5327-3702
CR Aktas S, 2009, FOOT ANKLE INT, V30, P391, DOI 10.3113/FAI.2009.0391
   Amlang Michael H, 2006, Oper Orthop Traumatol, V18, P287, DOI 10.1007/s00064-006-1178-y
   Assal M, 2002, J BONE JOINT SURG AM, V84A, P161, DOI 10.2106/00004623-200202000-00001
   Avina Valencia Jorge Arturo, 2009, Acta Ortop Mex, V23, P125
   CARR AJ, 1989, J BONE JOINT SURG BR, V71, P100, DOI 10.1302/0301-620X.71B1.2914976
   Chan Alexander Pak-Hin, 2011, Sports Med Arthrosc Rehabil Ther Technol, V3, P32, DOI 10.1186/1758-2555-3-32
   Coleman BD, 2000, SCAND J MED SCI SPOR, V10, P2, DOI 10.1034/j.1600-0838.2000.010001002.x
   Crawford J, 2004, COCHRANE DB SYST REV, DOI DOI 10.1002/14651858.CD003674.PUB2
   Cretnik A, 2005, AM J SPORT MED, V33, P1369, DOI 10.1177/0363546504271501
   Del Buono A, 2013, BRIT MED BULL, V106, P179, DOI 10.1093/bmb/lds039
   Del Buono A, 2013, KNEE SURG SPORT TR A, V21, P1050, DOI 10.1007/s00167-013-2418-y
   Del Buono A, 2012, BRIT MED BULL, V102, P115, DOI 10.1093/bmb/ldr038
   Ebinesan AD, 2008, DISABIL REHABIL, V30, P1721, DOI 10.1080/09638280701786815
   Gigante A, 2008, KNEE SURG SPORT TR A, V16, P204, DOI 10.1007/s00167-007-0448-z
   Grubor P, 2012, VOJNOSANIT PREGL, V69, P663, DOI 10.2298/VSP1208663G
   Guillo S, 2013, SURG-J R COLL SURG E, V11, P14, DOI 10.1016/j.surge.2011.12.002
   Haji A, 2004, FOOT ANKLE INT, V25, P215, DOI 10.1177/107110070402500404
   HATTRUP SJ, 1985, FOOT ANKLE, V6, P34, DOI 10.1177/107110078500600107
   Henriquez H, 2012, CLIN ORTHOP RELAT R, V470, P998, DOI 10.1007/s11999-011-1830-1
   INGLIS AE, 1976, J BONE JOINT SURG AM, V58, P990, DOI 10.2106/00004623-197658070-00015
   Kolodziej L, 2013, INT ORTHOP, V37, P625, DOI 10.1007/s00264-012-1737-9
   Leppilahti J, 1998, SPORTS MED, V25, P79, DOI 10.2165/00007256-199825020-00002
   Lim J, 2001, FOOT ANKLE INT, V22, P559, DOI 10.1177/107110070102200705
   MA GWC, 1977, CLIN ORTHOP RELAT R, P247
   Maffulli N, 1999, J BONE JOINT SURG AM, V81A, P1019, DOI 10.2106/00004623-199907000-00017
   Maffulli N, 1999, CLIN J SPORT MED, V9, P157, DOI 10.1097/00042752-199907000-00007
   Moller A, 1996, ACTA ORTHOP SCAND, V67, P479, DOI 10.3109/17453679608996672
   Moller M, 2001, J BONE JOINT SURG BR, V83B, P843
   Movin Tomas, 2005, Foot Ankle Clin, V10, P331, DOI 10.1016/j.fcl.2005.01.003
   NISTOR L, 1981, J BONE JOINT SURG AM, V63, P394, DOI 10.2106/00004623-198163030-00012
   Papalia R, 2011, BRIT MED BULL, V99, P89, DOI 10.1093/bmb/ldq043
   Papalia R, 2010, BRIT MED BULL, V96, P75, DOI 10.1093/bmb/ldq009
   Papalia R, 2010, BRIT MED BULL, V93, P201, DOI 10.1093/bmb/ldp044
   Rebeccato A, 2001, J Foot Ankle Surg, V40, P188
   Soroceanu A, 2012, J BONE JOINT SURG AM, V94A, P2136, DOI 10.2106/JBJS.K.00917
   Thevendran G, 2013, Musculoskelet Surg, V97, P9, DOI 10.1007/s12306-013-0251-6
   Willits K, 2010, J BONE JOINT SURG AM, V92A, P2767, DOI 10.2106/JBJS.I.01401
   WILLS CA, 1986, CLIN ORTHOP RELAT R, P156
   Winter E, 1998, ARCH ORTHOP TRAUM SU, V117, P364, DOI 10.1007/s004020050267
   Worth N, 2007, J Orthop Surg (Hong Kong), V15, P311
   Zhao HM, 2011, CHINESE MED J-PEKING, V124, P4050, DOI 10.3760/cma.j.issn.0366-6999.2011.23.038
NR 41
TC 50
Z9 50
U1 0
U2 8
PU OXFORD UNIV PRESS
PI OXFORD
PA GREAT CLARENDON ST, OXFORD OX2 6DP, ENGLAND
SN 0007-1420
EI 1471-8391
J9 BRIT MED BULL
JI Br. Med. Bull.
PD MAR
PY 2014
VL 109
IS 1
BP 45
EP 54
DI 10.1093/bmb/ldt029
PG 10
WC Medicine, General & Internal
WE Science Citation Index Expanded (SCI-EXPANDED)
SC General & Internal Medicine
GA AJ8RG
UT WOS:000337974000005
PM 24126569
OA Green Published, Bronze
DA 2022-07-22
ER

PT J
AU Meulenkamp, B
   Brillinger, J
   Fergusson, D
   Stacey, D
   Graham, ID
AF Meulenkamp, Brad
   Brillinger, Julia
   Fergusson, Dean
   Stacey, Dawn
   Graham, Ian D.
TI Development and field testing of a patient decision aid for management
   of acute Achilles tendon rupture: a study protocol
SO BMC MEDICAL INFORMATICS AND DECISION MAKING
LA English
DT Article
DE Achilles tendon rupture; Decision aid; Shared decision-making
ID SUPPORT FRAMEWORK; SAMPLE-SIZE; VALIDATION; CHALLENGES; CONFLICT;
   BENEFITS; SURGERY; WOMEN; SURE
AB Background Achilles tendon ruptures are common injuries in an otherwise healthy, active population. Several treatment options exist, with both surgical and non-surgical options. Each treatment option has a unique set of risks and harms, which may present patients with decisional conflict. The aim of the proposed study is to develop, alpha test and field test a patient decision aid for patients presenting with acute Achilles tendon ruptures. Methods This is a three-stage study protocol. First, we will assemble a multi-disciplinary steering group including patients, clinicians, educators, and researchers to develop the patient decision aid prototype using the Ottawa Decision Support Framework. Second, we will perform a mixed-methods alpha test of the decision aid prototype with patients and clinicians experienced in acute Achilles tendon ruptures. Outcomes measured will include acceptability and usability of the patient decision aid measured using validated outcome scales and semi-structured interviews. A minimum of three rounds of feedback will be obtained. Results will be analyzed using descriptive statistics, reviewed by the steering group, to guide revisions to decision aid prototype at each round. The third stage will be field testing the revised decision aid prototype in usual clinical care. A pre-/post-study will be performed with patients with acute Achilles tendon ruptures. Patients will be recruited from the emergency department and complete the pre-consultation decision aid prior to a one-week follow up with their surgeon. The primary outcome of field testing will be feasibility of implementing the decision aid in the clinical setting and will be measured with recruitment and completion metrics. Secondary outcomes include acceptability of the decision aid, knowledge, preparedness for decision making, and decisional conflict, measured using validated outcome measures. Statistical analysis will be performed using descriptive analysis for primary outcomes and a student t-test and Wilcoxon Rank-Sum test for secondary outcomes. Discussion This comprehensive study protocol outlines the development, alpha testing, and field testing of a patient decision aid for patients with acute Achilles tendon rupture. Systematic and transparent development and testing of patient decision aids is critical to improve decision aid quality. Trial registration Not Applicable.
C1 [Meulenkamp, Brad; Fergusson, Dean] Univ Ottawa, Dept Med, Ottawa, ON, Canada.
   [Brillinger, Julia; Fergusson, Dean] Ottawa Hosp Res Inst, Ottawa, ON, Canada.
   [Stacey, Dawn; Graham, Ian D.] Ottawa Hosp Res Inst, Ctr Practice Changing Res, Ottawa, ON, Canada.
   [Fergusson, Dean; Stacey, Dawn] Univ Ottawa, Fac Hlth Sci, Ottawa, ON, Canada.
   [Graham, Ian D.] Univ Ottawa, Sch Epidemiol & Publ Hlth, Ottawa, ON, Canada.
   [Meulenkamp, Brad] Ottawa Hosp, Orthopaed Trauma Foot & Ankle Surg, Ottawa, ON, Canada.
RP Meulenkamp, B (通讯作者)，Univ Ottawa, Dept Med, Ottawa, ON, Canada.; Meulenkamp, B (通讯作者)，Ottawa Hosp, Orthopaed Trauma Foot & Ankle Surg, Ottawa, ON, Canada.
EM bmeulenkamp@toh.ca
RI Graham, Ian D./ABD-5259-2021
OI Graham, Ian D./0000-0002-3669-1216; Stacey, Dawn/0000-0002-2681-741X;
   Brillinger, Julia/0000-0001-8430-1967
FU Canadian Orthopaedic Foundation, through the Canadian Orthopaedic
   Research Legacy (CORL) fund
FX This study has received funding from the Canadian Orthopaedic
   Foundation, through the Canadian Orthopaedic Research Legacy (CORL)
   fund. The threestage study protocol has undergone peer-review by this
   funding body. This organization was not involved in manuscript writing
   or the decision to submit the manuscript for publication.
CR [Anonymous], 2019, OTTAWA PATIENT DECIS
   Bangor A, 2008, INT J HUM-COMPUT INT, V24, P574, DOI 10.1080/10447310802205776
   Bangor A, 2009, J USABILITY STUD, V4, P114
   Bansback N, 2019, BMC NEUROL, V19, DOI 10.1186/s12883-019-1382-7
   Barry MJ, 2012, NEW ENGL J MED, V366, P780, DOI 10.1056/NEJMp1109283
   Bartunek Jean, 2003, J Health Serv Res Policy, V8 Suppl 2, P62, DOI 10.1258/135581903322405199
   Bennett C, 2010, PATIENT EDUC COUNS, V78, P130, DOI 10.1016/j.pec.2009.05.012
   Berger Ken, 2003, Med Law, V22, P743
   Boland L, 2019, MED DECIS MAKING, V39, P1010, DOI 10.1177/0272989X19884541
   Cargo M, 2008, ANNU REV PUBL HEALTH, V29, P325, DOI 10.1146/annurev.publhealth.29.091307.083824
   Carroll Sandra L, 2017, Pilot Feasibility Stud, V3, P49, DOI 10.1186/s40814-017-0189-9
   Charles C, 1999, SOC SCI MED, V49, P651, DOI 10.1016/S0277-9536(99)00145-8
   Cohen D, 2019, COA B, V125, P19
   Colorafi KJ, 2016, HERD-HEALTH ENV RES, V9, P16, DOI 10.1177/1937586715614171
   Coulter A, 2013, BMC MED INFORM DECIS, V13, DOI 10.1186/1472-6947-13-S2-S2
   Drake E R, 1999, J Genet Couns, V8, P217, DOI 10.1023/A:1022998415890
   Edwards A, 2003, PATIENT EDUC COUNS, V50, P33, DOI 10.1016/S0738-3991(03)00077-6
   Elwyn G, 2006, BMJ-BRIT MED J, V333, P417, DOI 10.1136/bmj.38926.629329.AE
   Erad MU, 2020, JBJS
   Faulkner L, 2003, BEHAV RES METH INS C, V35, P379, DOI 10.3758/BF03195514
   Fiset Valerie, 2000, Health Expect, V3, P125, DOI 10.1046/j.1369-6513.2000.00067.x
   Flesch R, 1948, J APPL PSYCHOL, V32, P221, DOI 10.1037/h0057532
   Garvelink MM, 2019, MED DECIS MAKING, V39, P301, DOI 10.1177/0272989X19851345
   Golden-Biddle Karen, 2003, J Health Serv Res Policy, V8 Suppl 2, P20, DOI 10.1258/135581903322405135
   Graham I., 1995, USER MANUAL PREPARAT
   Graneheim UH, 2017, NURS EDUC TODAY, V56, P29, DOI 10.1016/j.nedt.2017.06.002
   Hertzog MA, 2008, RES NURS HEALTH, V31, P180, DOI 10.1002/nur.20247
   Hoffman AS, 2020, GERONTOLOGIST, V60, P935, DOI 10.1093/geront/gnz141
   Hooiveld T, 2018, SAGE OPEN MED, V6, DOI 10.1177/2050312118809462
   Joseph-Williams N, 2014, MED DECIS MAKING, V34, P699, DOI 10.1177/0272989X13501721
   Jull J, 2017, IMPLEMENT SCI, V12, DOI 10.1186/s13012-017-0696-3
   Kadakia AR, 2017, J AM ACAD ORTHOP SUR, V25, P23, DOI 10.5435/JAAOS-D-15-00187
   Kujala S, 2003, BEHAV INFORM TECHNOL, V22, P1, DOI 10.1080/0144929021000055530
   Legare F, 2010, CAN FAM PHYSICIAN, V56, pE308
   ManSonHing M, 1996, ARCH INTERN MED, V156, P1841, DOI 10.1001/archinte.156.16.1841
   Mattila VM, 2015, BRIT J SPORT MED, V49, P1084, DOI 10.1136/bjsports-2013-092756
   O'Connor A M, 1999, J Natl Cancer Inst Monogr, P67
   O'Connor AM, 1998, PATIENT EDUC COUNS, V33, P267, DOI 10.1016/S0738-3991(98)00026-3
   O'Connor AM, 2000, USER MANUAL KNOWLEDG
   OCONNOR AM, 1995, MED DECIS MAKING, V15, P25, DOI 10.1177/0272989X9501500105
   OConnor AM, 1992, USER MANUAL ACCEPTAB
   Palinkas LA, 2015, ADM POLICY MENT HLTH, V42, P533, DOI 10.1007/s10488-013-0528-y
   Parayre AF, 2014, MED DECIS MAKING, V34, P54, DOI 10.1177/0272989X13491463
   Patel MS, 2019, FOOT ANKLE CLIN, V24, P399, DOI 10.1016/j.fcl.2019.05.002
   Rahman M. D., 2016, J ED LEARNING, V6, P102, DOI [10.5539/jel.v6n1p102, DOI 10.5539/JEL.V6N1P102]
   SANDELOWSKI M, 1995, RES NURS HEALTH, V18, P179, DOI 10.1002/nur.4770180211
   Sandelowski M, 2010, RES NURS HEALTH, V33, P77, DOI 10.1002/nur.20362
   Sauro J., 2011, PRACTICAL GUIDE SYST
   Scholl I, 2014, PLOS ONE, V9, DOI 10.1371/journal.pone.0107828
   Sepucha KR, 2018, BMJ QUAL SAF, V27, P380, DOI 10.1136/bmjqs-2017-006986
   Shay LA, 2015, MED DECIS MAKING, V35, P114, DOI 10.1177/0272989X14551638
   Sheth U, 2017, BONE JOINT J, V99B, P78, DOI [10.1302/0301-620X.99B1.BJJ-2016-0434.R1, 10.1302/0301-620x.99b1.bjj-2016-0434.r1]
   Sivell S, 2012, PATIENT EDUC COUNS, V86, P179, DOI 10.1016/j.pec.2011.04.014
   Soroceanu A, 2012, J BONE JOINT SURG AM, V94A, P2136, DOI 10.2106/JBJS.K.00917
   Stacey D, 2020, MED DECIS MAKING, V40, P379, DOI 10.1177/0272989X20911870
   Stacey D, 2017, STUD HEALTH TECHNOL, V240, P263, DOI 10.3233/978-1-61499-790-0-263
   Stacey D, 2017, COCHRANE DB SYST REV, DOI 10.1002/14651858.CD001431.pub5
   Trevena LJ, 2013, BMC MED INFORM DECIS, V13, DOI 10.1186/1472-6947-13-S2-S7
   Vaismoradi M, 2013, NURS HEALTH SCI, V15, P398, DOI 10.1111/nhs.12048
   Walter Isabel, 2003, J Health Serv Res Policy, V8 Suppl 2, P58
   Weston WW, 2001, CAN MED ASSOC J, V165, P438
NR 61
TC 0
Z9 0
U1 0
U2 1
PU BMC
PI LONDON
PA CAMPUS, 4 CRINAN ST, LONDON N1 9XW, ENGLAND
EI 1472-6947
J9 BMC MED INFORM DECIS
JI BMC Med. Inform. Decis. Mak.
PD JUL 24
PY 2021
VL 21
IS 1
AR 225
DI 10.1186/s12911-021-01589-5
PG 11
WC Medical Informatics
WE Science Citation Index Expanded (SCI-EXPANDED); Social Science Citation Index (SSCI)
SC Medical Informatics
GA TX6BH
UT WOS:000683173600002
PM 34303358
OA gold, Green Published
DA 2022-07-22
ER

PT J
AU Ko, PY
   Huang, MT
   Li, CL
   Su, WR
   Jou, IM
   Wu, PT
AF Ko, Po-Yen
   Huang, Ming-Tung
   Li, Chia-Lung
   Su, Wei-Ren
   Jou, I-Ming
   Wu, Po-Ting
TI Jigless knotless internal brace technique for acute Achilles tendon
   rupture: a case series study
SO JOURNAL OF ORTHOPAEDIC SURGERY AND RESEARCH
LA English
DT Article
DE Achilles tendon rupture; Minimally invasive; Knotless; Internal brace
ID NONSURGICAL TREATMENT; SURGICAL-TREATMENT; SURAL NERVE; REPAIR;
   REHABILITATION; METAANALYSIS; COMPLICATIONS; MANAGEMENT; SURGERY; INJURY
AB Purpose: To mitigate the risk of poor wound healing and of infection associated with the open repair of Achilles tendon midsubstance ruptures, minimally invasive techniques have been developed. We report our preliminary results after reviewing our "jigless knotless internal brace technique."
   Methods: Patients were placed in prone position and a transverse 3-cm incision was made proximal to the palpable ruptured end. The proximal ruptured end was pulled out, gently debrided, and sutured using Krackow locking loops. Percutaneous sutures were crisscrossed through the distal tendon stump and looped around the Krackow sutures over the proximal stump. The ipsilateral Krackow sutures and the contralateral crisscrossed sutures were subcutaneously passed through two mini-incisions over the posterior calcaneus tuberosity and seated at the tuberosity with two 4.5-mm knotless suture anchors. All patients underwent the same post-operative rehabilitation protocol and regular follow-ups for at least 1 year.
   Results: We recruited 10 patients (mean age, 37.3 years) who scored 100 points on the American Orthopaedic Foot and Ankle Society (AOFAS) scale, and who returned to their preoperative exercise levels 1-year postoperatively with no complications.
   Conclusion: Our method is simple, effective, and requires no special tools. It might be a reliable option for Achilles tendon repair.
C1 [Ko, Po-Yen; Wu, Po-Ting] Natl Cheng Kung Univ, Dept Biomed Engn, Tainan, Taiwan.
   [Ko, Po-Yen; Li, Chia-Lung; Wu, Po-Ting] Natl Cheng Kung Univ, Coll Med, Dou Liu Branch, Dept Orthoped,Natl Cheng Kung Univ Hosp, Tainan, Yunlin, Taiwan.
   [Ko, Po-Yen; Huang, Ming-Tung; Li, Chia-Lung; Su, Wei-Ren; Wu, Po-Ting] Natl Cheng Kung Univ, Natl Cheng Kung Univ Hosp, Coll Med, Dept Orthoped, Tainan, Taiwan.
   [Su, Wei-Ren; Wu, Po-Ting] Natl Cheng Kung Univ, Coll Med, Dept Orthoped, 1 Univ Rd, Tainan 701, Taiwan.
   [Jou, I-Ming] E Da Hosp, Dept Orthoped, Kaohsiung, Taiwan.
   [Jou, I-Ming] I Shou Univ, Coll Med, Sch Med, Kaohsiung, Taiwan.
   [Wu, Po-Ting] Natl Cheng Kung Univ, Med Device Innovat Ctr, Tainan, Taiwan.
RP Wu, PT (通讯作者)，Natl Cheng Kung Univ, Dept Biomed Engn, Tainan, Taiwan.; Wu, PT (通讯作者)，Natl Cheng Kung Univ, Coll Med, Dou Liu Branch, Dept Orthoped,Natl Cheng Kung Univ Hosp, Tainan, Yunlin, Taiwan.; Wu, PT (通讯作者)，Natl Cheng Kung Univ, Natl Cheng Kung Univ Hosp, Coll Med, Dept Orthoped, Tainan, Taiwan.; Wu, PT (通讯作者)，Natl Cheng Kung Univ, Coll Med, Dept Orthoped, 1 Univ Rd, Tainan 701, Taiwan.; Wu, PT (通讯作者)，Natl Cheng Kung Univ, Med Device Innovat Ctr, Tainan, Taiwan.
EM anotherme500@gmail.com
OI Wu, Po-Ting/0000-0001-5534-8993
FU Taiwan National Science Council [MOST 107-2314-B-006-065-MY3,
   MOST-107-2622-E-006-019-CC2, MOST 106-2314-B-006-004, MOST
   1082314-B-006-011-MY2]; National Cheng Kung University Hospital
   [NCKUH-107-02-024, NCKUH-106-03-030]
FX Taiwan National Science Council (grants: MOST 107-2314-B-006-065-MY3,
   MOST-107-2622-E-006-019-CC2, MOST 106-2314-B-006-004, and MOST
   1082314-B-006-011-MY2) and National Cheng Kung University Hospital
   (grants: NCKUH-107-02-024, NCKUH-106-03-030)
CR Avina Valencia Jorge Arturo, 2009, Acta Ortop Mex, V23, P125
   Barfod KW, 2015, J FOOT ANKLE SURG, V54, P220, DOI 10.1053/j.jfas.2014.11.018
   Bevoni R, 2014, INJURY, V45, P1268, DOI 10.1016/j.injury.2014.04.028
   Blackmon JA, 2013, J FOOT ANKLE SURG, V52, P42, DOI 10.1053/j.jfas.2012.09.010
   Carmont MR, 2011, BMC SPORTS SCI MED R, V3, DOI 10.1186/1758-2555-3-28
   Citak M, 2007, BRIT J SPORT MED, V41, P456, DOI 10.1136/bjsm.2006.031328
   Ganestam A, 2016, KNEE SURG SPORT TR A, V24, P3730, DOI 10.1007/s00167-015-3544-5
   Hsu AR, 2015, FOOT ANKLE INT, V36, P1279, DOI 10.1177/1071100715589632
   Huang JZ, 2015, AM J SPORT MED, V43, P1008, DOI 10.1177/0363546514531014
   Huttunen TT, 2014, AM J SPORT MED, V42, P2419, DOI 10.1177/0363546514540599
   Ismail M, 2008, FOOT ANKLE INT, V29, P808, DOI 10.3113/FAI.2008.0808
   Keating JF, 2011, J BONE JOINT SURG BR, V93B, P1071, DOI 10.1302/0301-620X.93B8.25998
   Khan RJK, 2005, J BONE JOINT SURG AM, V87A, P2202, DOI 10.2106/JBJS.D.03049
   KLEIN W, 1991, Chirurgia degli Organi di Movimento, V76, P223
   Lantto I, 2015, SCAND J MED SCI SPOR, V25, pE133, DOI 10.1111/sms.12253
   Liechti DJ, 2018, ARTHROSC TEC, V7, pE171, DOI 10.1016/j.eats.2017.08.065
   Maffulli N, 2009, ORTHOP CLIN N AM, V40, P491, DOI 10.1016/j.ocl.2009.05.006
   McCormack R, 2015, BRIT J SPORT MED, V49, P1329, DOI 10.1136/bjsports-2015-094935
   McMahon Samuel E, 2011, Foot Ankle Surg, V17, P211, DOI 10.1016/j.fas.2010.11.001
   McWilliam JR, 2016, FOOT ANKLE INT, V37, P794, DOI 10.1177/1071100716653373
   Olsson N, 2013, AM J SPORT MED, V41, P2867, DOI 10.1177/0363546513503282
   Paavola M, 2000, AM J SPORT MED, V28, P77, DOI 10.1177/03635465000280012501
   Sarman H, 2016, J FOOT ANKLE SURG, V55, P965, DOI 10.1053/j.jfas.2016.04.014
   Soroceanu A, 2012, J BONE JOINT SURG AM, V94A, P2136, DOI 10.2106/JBJS.K.00917
   Suchak AA, 2005, FOOT ANKLE INT, V26, P932, DOI 10.1177/107110070502601106
   Uquillas CA, 2015, J BONE JOINT SURG AM, V97A, P1187, DOI 10.2106/JBJS.O.00002
   Valkering KP, 2017, KNEE SURG SPORT TR A, V25, P1807, DOI 10.1007/s00167-016-4270-3
   Yepes H, 2010, J BONE JOINT SURG AM, V92A, P1215, DOI 10.2106/JBJS.I.00743
   Yin LJ, 2018, INJURY, V49, P726, DOI 10.1016/j.injury.2018.01.011
NR 29
TC 2
Z9 3
U1 0
U2 0
PU BMC
PI LONDON
PA CAMPUS, 4 CRINAN ST, LONDON N1 9XW, ENGLAND
SN 1749-799X
J9 J ORTHOP SURG RES
JI J. Orthop. Surg. Res.
PD DEC 5
PY 2019
VL 14
IS 1
AR 415
DI 10.1186/s13018-019-1471-8
PG 8
WC Orthopedics
WE Science Citation Index Expanded (SCI-EXPANDED)
SC Orthopedics
GA KD8RQ
UT WOS:000508130900003
PM 31806034
OA Green Published, gold
DA 2022-07-22
ER

PT J
AU Nilsson-Helander, K
   Silbernagel, KG
   Thomee, R
   Faxen, E
   Olsson, N
   Eriksson, BI
   Karlsson, J
AF Nilsson-Helander, Katarina
   Silbernagel, Karin Gravare
   Thomee, Roland
   Faxen, Eva
   Olsson, Nicklas
   Eriksson, Bengt I.
   Karlsson, Jon
TI Acute Achilles Tendon Rupture A Randomized, Controlled Study Comparing
   Surgical and Nonsurgical Treatments Using Validated Outcome Measures
SO AMERICAN JOURNAL OF SPORTS MEDICINE
LA English
DT Article
DE Achilles tendon rupture; movable brace; rerupture; ATRS; validated
   functional tests
ID FUNCTIONAL TREATMENT; NONOPERATIVE MANAGEMENT; EARLY MOTION; OPEN
   REPAIR; METAANALYSIS; ANKLE; MOBILIZATION; SURGERY; CAST
AB Background: There is no consensus regarding the optimal treatment for patients with acute Achilles tendon rupture. Few randomized controlled studies have compared outcomes after surgical or nonsurgical treatment with both groups receiving early mobilization.
   Purpose: This study was undertaken to compare outcomes of patients with acute Achilles tendon rupture treated with or without surgery using early mobilization and identical rehabilitation protocols.
   Study Design: Randomized, controlled trial; Level of evidence, 1.
   Methods: Ninety-seven patients (79 men, 18 women; mean age, 41 years) with acute Achilles tendon rupture were treated and followed for 1 year. The primary end point was rerupturing. Patients were evaluated using the Achilles tendon Total Rupture Score (ATRS), functional tests, and clinical examination at 6 and 12 months after injury.
   Results: There were 6 (12%) reruptures in the nonsurgical group and 2 (4%) in the surgical group (P = .377). The mean 6- and 12-month ATRS were 72 and 88 points in the surgical group and 71 and 86 points in the nonsurgical group, respectively. Improvements in ATRS between 6 and 12 months were significant for both groups, with no significant between-group differences. At the 6- month evaluation, the surgical group had better results compared with the nonsurgically treated group in some of the muscle function tests; however, at the 12-month evaluation there were no differences between the 2 groups except for the heel-rise work test in favor of the surgical group. At the 12-month follow-up, the level of function of the injured leg remained significantly lower than that of the uninjured leg in both groups.
   Conclusion: The results of this study did not demonstrate any statistically significant difference between surgical and nonsurgical treatment. Furthermore, the study suggests that early mobilization is beneficial for patients with acute Achilles tendon rupture whether they are treated surgically or nonsurgically. The preferred treatment strategy for patients with acute Achilles tendon rupture remains a subject of debate. Although the study met the sample size dictated by the authors' a priori power calculation, the difference in the rerupture rate might be considered clinically important by some.
C1 [Nilsson-Helander, Katarina] Kungsbacka Hosp, Dept Orthopaed, SE-43440 Kungsbacka, Sweden.
   [Nilsson-Helander, Katarina; Silbernagel, Karin Gravare; Thomee, Roland; Faxen, Eva; Olsson, Nicklas; Eriksson, Bengt I.; Karlsson, Jon] Univ Gothenburg, Dept Orthopaed, Inst Clin Sci, Sahlgrenska Acad,Sahlgrenska Univ Hosp, Gothenburg, Sweden.
   [Silbernagel, Karin Gravare] Univ Delaware, Newark, DE USA.
RP Nilsson-Helander, K (通讯作者)，Kungsbacka Hosp, Dept Orthopaed, SE-43440 Kungsbacka, Sweden.
EM ina.nilsson@telia.com
RI Helander, Katarina Nilsson/AAE-1793-2020
OI Helander, Katarina Nilsson/0000-0002-1292-5102; Gravare Silbernagel,
   Karin/0000-0001-7566-407X
FU Swedish National Centre for Research in Sports; Local Research and
   Development Council of Halland, Sweden
FX The authors thank Lotta Falkheden-Henning and Annelie Brorsson for their
   help treating and evaluating the patients. They also thank the Swedish
   National Centre for Research in Sports (CIF) and the local Research and
   Development Council of Halland, Sweden, for providing financial support.
CR Ajis A, 2007, FOOT ANKLE SURG, V13, P132
   Aspenberg P, 2007, INT ORTHOP, V31, P783, DOI 10.1007/s00264-007-0398-6
   Bhandari M, 2002, CLIN ORTHOP RELAT R, P190
   CETTI R, 1994, CLIN ORTHOP RELAT R, P155
   CETTI R, 1993, AM J SPORT MED, V21, P791, DOI 10.1177/036354659302100606
   Kangas J, 2003, J TRAUMA, V54, P1171, DOI 10.1097/01.TA.0000047945.20863.A2
   Kerkhoffs GMMJ, 2002, ARCH ORTHOP TRAUM SU, V122, P102, DOI 10.1007/s004020100312
   Kessler I, 1973, Hand, V5, P253, DOI 10.1016/0072-968X(73)90038-7
   Khan RJK, 2005, J BONE JOINT SURG AM, V87A, P2202, DOI 10.2106/JBJS.D.03049
   Kjaer M., 2005, Journal of Musculoskeletal & Neuronal Interactions, V5, P41
   Kocher MS, 2002, AM J SPORT MED, V30, P783, DOI 10.1177/03635465020300060501
   Lapidus L, 2006, J THROMB HAEMOST, V4, P807, DOI 10.1111/j.1538-7836.2006.01832.x
   Lapidus LJ, 2007, J ORTHOP TRAUMA, V21, P52, DOI 10.1097/01.bot.0000250741.65003.14
   Lim J, 2001, FOOT ANKLE INT, V22, P559, DOI 10.1177/107110070102200705
   Lo IKY, 1997, CLIN J SPORT MED, V7, P207, DOI 10.1097/00042752-199707000-00010
   Lynch Richard M, 2004, Accid Emerg Nurs, V12, P149, DOI 10.1016/j.aaen.2003.11.004
   Maffulli N, 2003, AM J SPORT MED, V31, P692, DOI 10.1177/03635465030310051001
   Moller M, 2001, J BONE JOINT SURG BR, V83B, P843
   Moller M, 2002, SCAND J MED SCI SPOR, V12, P9, DOI 10.1034/j.1600-0838.2002.120103.x
   Mortensen NHM, 1999, J BONE JOINT SURG AM, V81A, P983, DOI 10.2106/00004623-199907000-00011
   Mullaney MJ, 2006, AM J SPORT MED, V34, P1120, DOI 10.1177/0363546505284186
   Nilsson-Helander K, 2008, KNEE SURG SPORT TR A, V16, P614, DOI 10.1007/s00167-008-0492-3
   Nilsson-Helander K, 2007, AM J SPORT MED, V35, P421, DOI 10.1177/0363546506294856
   Nilsson-Helander K, 2009, KNEE SURG SPORT TR A, V17, P1234, DOI 10.1007/s00167-009-0727-y
   NISTOR L, 1981, J BONE JOINT SURG AM, V63, P394, DOI 10.2106/00004623-198163030-00012
   Pajala A, 2002, J BONE JOINT SURG AM, V84A, P2016, DOI 10.2106/00004623-200211000-00017
   Petersen Oleg Funck, 2002, Ugeskr Laeger, V164, P3852
   SALEH M, 1992, J BONE JOINT SURG BR, V74, P206, DOI 10.1302/0301-620X.74B2.1544953
   SALTIN B, 1968, CIRCULATION, V38, P1104, DOI 10.1161/01.CIR.38.6.1104
   Silbernagel KG, 2006, KNEE SURG SPORT TR A, V14, P1207, DOI 10.1007/s00167-006-0150-6
   Suchak AA, 2006, CLIN ORTHOP RELAT R, P216, DOI 10.1097/01.blo.0000203458.05135.74
   Thermann H, 1995, UNFALLCHIRURG, V98, P507
   THOMPSON T C, 1962, J Trauma, V2, P126, DOI 10.1097/00005373-196203000-00003
   Twaddle BC, 2007, AM J SPORT MED, V35, P2033, DOI 10.1177/0363546507307503
   Wong J, 2002, AM J SPORT MED, V30, P565, DOI 10.1177/03635465020300041701
NR 35
TC 244
Z9 262
U1 1
U2 27
PU SAGE PUBLICATIONS INC
PI THOUSAND OAKS
PA 2455 TELLER RD, THOUSAND OAKS, CA 91320 USA
SN 0363-5465
EI 1552-3365
J9 AM J SPORT MED
JI Am. J. Sports Med.
PD NOV
PY 2010
VL 38
IS 11
BP 2186
EP 2193
DI 10.1177/0363546510376052
PG 8
WC Orthopedics; Sport Sciences
WE Science Citation Index Expanded (SCI-EXPANDED)
SC Orthopedics; Sport Sciences
GA 669KT
UT WOS:000283348600009
PM 20802094
DA 2022-07-22
ER

PT J
AU Ozsoy, MH
   Cengiz, B
   Ozsoy, A
   Aksekili, MAE
   Yucel, M
   Fakioglu, O
   Dincel, VE
   Aydogan, NH
AF Ozsoy, Mehmet Hakan
   Cengiz, Bertan
   Ozsoy, Arzu
   Aksekili, Mehmet Atif Erol
   Yucel, Mehmet
   Fakioglu, Onur
   Dincel, Veysel Ercan
   Aydogan, Nevres Hurriyet
TI Minimally Invasive Achilles Tendon Repair: A Modification of the
   Achillon Technique
SO FOOT & ANKLE INTERNATIONAL
LA English
DT Article
DE Achilles tendon; tendon rupture; minimally invasive; sports; surgery
ID PERCUTANEOUS REPAIR; SURGICAL REPAIR; ACUTE RUPTURES; DEVICE
AB Background: The purpose of the study was to present the results of a new minimally invasive operative method for Achilles tendon (AT) ruptures that could reduce nerve injuries and add no extra cost.
   Methods: Thirteen patients with acute AT ruptures who were treated with minimally invasive surgery and followed for a minimum of 12 months were included. At the latest follow-up, American Orthopaedic Foot & Ankle Society (AOFAS) score, calf diameters, ability to walk on tiptoe, and ultrasound examination were evaluated. The average age was 42.8 years (range, 31-62 years). Average follow up was 24.5 months (range, 12-34 months).
   Results: AOFAS score was 92.5 (range, 85-100). Average calf diameters on the operated and nonoperated extremities were 38.9 cm (range, 36-44 cm) and 38.9 cm (range, 36-41 cm), respectively. On ultrasound examination, the site of the rupture was found to be 46.2 mm proximal from the calcaneal insertion, and the operated side was found to be significantly thicker than the nonoperated side (P = .008). There was 1 deep vein thrombosis, which recovered without sequelae. There were no wound problems, reruptures, or nerve injuries.
   Conclusions: This new minimally invasive operative method was successful, showing good functional results and low complication rates. In our experience, the use of 3 continuous polyester sutures was less irritable with 3 knots.
C1 [Ozsoy, Mehmet Hakan; Cengiz, Bertan; Aksekili, Mehmet Atif Erol; Yucel, Mehmet; Dincel, Veysel Ercan; Aydogan, Nevres Hurriyet] Ankara Numune Training & Res Hosp, Dept Orthoped & Traumatol, TR-06340 Ankara, Turkey.
   [Ozsoy, Arzu] Ankara Numune Training & Res Hosp, Dept Radiol, Ankara, Turkey.
   [Fakioglu, Onur] Dortyol State Hosp, Dept Orthoped & Traumatol, Antakya, Turkey.
RP Ozsoy, MH (通讯作者)，Ankara Numune Training & Res Hosp, Dept Orthoped & Traumatol, TR-06340 Ankara, Turkey.
EM hakanozsoy@rocketmail.com
RI Aksekili, Atif MAEA/D-2075-2016
CR Aibinder WR, 2013, FOOT ANKLE INT, V34, P870, DOI 10.1177/1071100712473097
   Aktas S, 2009, FOOT ANKLE INT, V30, P391, DOI 10.3113/FAI.2009.0391
   Assal M, 2002, J BONE JOINT SURG AM, V84A, P161, DOI 10.2106/00004623-200202000-00001
   Barwick TW, 2013, FOOT ANKLE SURG, V19, pE5, DOI 10.1016/j.fas.2012.11.007
   Bergqvist D, 2012, THROMB HAEMOSTASIS, V107, P280, DOI 10.1160/TH11-07-0464
   Bhattacharyya M, 2009, INT ORTHOP, V33, P151, DOI 10.1007/s00264-008-0564-5
   Bleakney RR, 2002, CLIN J SPORT MED, V12, P273, DOI 10.1097/00042752-200209000-00003
   Carmont MR, 2008, KNEE SURG SPORT TR A, V16, P199, DOI 10.1007/s00167-007-0411-z
   Cretnik A, 2005, AM J SPORT MED, V33, P1369, DOI 10.1177/0363546504271501
   Ebinesan AD, 2008, DISABIL REHABIL, V30, P1721, DOI 10.1080/09638280701786815
   Feldbrin Z, 2010, ISR MED ASSOC J, V12, P609
   Guillo S, 2013, SURG-J R COLL SURG E, V11, P14, DOI 10.1016/j.surge.2011.12.002
   HAGGMARK T, 1986, ORTHOPEDICS, V9, P160
   Huffard B, 2008, CLIN BIOMECH, V23, P1158, DOI 10.1016/j.clinbiomech.2008.05.007
   Ismail M, 2008, FOOT ANKLE INT, V29, P808, DOI 10.3113/FAI.2008.0808
   Khan RJK, 2005, J BONE JOINT SURG AM, V87A, P2202, DOI 10.2106/JBJS.D.03049
   Lansdaal JR, 2007, INJURY, V38, P839, DOI 10.1016/j.injury.2006.12.010
   Longo UG, 2012, KNEE SURG SPORT TR A, V20, P1392, DOI 10.1007/s00167-011-1868-3
   Longo UG, 2009, SPORTS MED ARTHROSC, V17, P127, DOI 10.1097/JSA.0b013e3181a3d767
   Maes Renaud, 2006, Acta Orthop Belg, V72, P179
   Maffulli N, 1999, J BONE JOINT SURG AM, V81A, P1019, DOI 10.2106/00004623-199907000-00017
   Majewski M, 2006, AM J SPORT MED, V34, P793, DOI 10.1177/0363546505283266
   McMahon Samuel E, 2011, Foot Ankle Surg, V17, P211, DOI 10.1016/j.fas.2010.11.001
   Metz R, 2011, AM J SPORT MED, V39, P820, DOI 10.1177/0363546510392012
   Moller M, 2002, KNEE SURG SPORT TR A, V10, P49, DOI 10.1007/s001670100245
   Nilsson-Helander K, 2010, AM J SPORT MED, V38, P2186, DOI 10.1177/0363546510376052
   Nilsson-Helander K, 2009, KNEE SURG SPORT TR A, V17, P1234, DOI 10.1007/s00167-009-0727-y
   Patel A, 2012, CLIN ORTHOP RELAT R, V470, P270, DOI 10.1007/s11999-011-2166-6
   RUPP S, 1995, BRIT J RADIOL, V68, P454, DOI 10.1259/0007-1285-68-809-454
   THERMANN H, 1989, J SPORT TRAUMATOL, V11, P9
   Valente Maurizio, 2012, Musculoskelet Surg, V96, P35, DOI 10.1007/s12306-011-0158-z
   Wong J, 2002, AM J SPORT MED, V30, P565, DOI 10.1177/03635465020300041701
NR 32
TC 9
Z9 11
U1 1
U2 7
PU SAGE PUBLICATIONS INC
PI THOUSAND OAKS
PA 2455 TELLER RD, THOUSAND OAKS, CA 91320 USA
SN 1071-1007
EI 1944-7876
J9 FOOT ANKLE INT
JI Foot Ankle Int.
PD DEC
PY 2013
VL 34
IS 12
BP 1683
EP 1688
DI 10.1177/1071100713505754
PG 6
WC Orthopedics
WE Science Citation Index Expanded (SCI-EXPANDED)
SC Orthopedics
GA 297YU
UT WOS:000330291800013
PM 24045854
DA 2022-07-22
ER

PT J
AU Li, Y
   Jiang, Q
   Chen, H
   Xin, HK
   He, Q
   Ruan, DK
AF Li, Yong
   Jiang, Qiang
   Chen, Hua
   Xin, Hongkui
   He, Qing
   Ruan, Dike
TI Comparison of mini-open repair system and percutaneous repair for acute
   Achilles tendon rupture
SO BMC MUSCULOSKELETAL DISORDERS
LA English
DT Article
DE Acute Achilles tendon rupture; Minimally invasive; Percutaneous;
   Mini-open
ID MINIMALLY INVASIVE REPAIR; CLINICAL-OUTCOMES; OPEN SURGERY; SUTURES
AB Background To reduce incision complications, minimally invasive operative approaches for treatment with acute Achilles tendon rupture have been developed, such as Mini-open repair and percutaneous repair. Which technique is the better surgical option? In the present study, we compared the two surgical procedures- modified Mini-open repair versus percutaneous repair-in the treatment of acute Achilles tendon rupture. Methods From January 2016 to November 2018, 68 matched patients with acute Achilles tendon rupture were divided into treatment group (Mini-open with modified Ma-Griffith technique) and control group (the Ma-Griffith technique). The patients were then treated with different surgical techniques and followed up for no less than 24 months, and the functional outcome scores and complications were retrospectively evaluated. Results The mean follow-up time in Mini-open repair group was 29.0 +/- 2.9 months, and that in control group was 27.9 +/- 2.9 months (P=0.147). The Mini-open repair group showed reliably higher American Orthopedic Foot and Ankle Society (AOFAS) Ankle-Hindfoot Score and Achilles tendon Total Rupture Score (ATRS) than the control group in functional assessment (95.0 +/- 3.8 vs. 92.3 +/- 5.3, P=0.000; 93.8 +/- 3.8 vs. 90.9 +/- 4.5,P=0.000). There was no cases of sural nerve injury in Mini-open repair group, whereas the percutaneous repair group had 5 cases of the same (P=0.027). No significant differences were found in the calf circumference (32.3 +/- 3.9 vs. 31.8 +/- 3.6) (P=0.564), range of motion of the ankle (51.3 +/- 4.8 vs. 50.5 +/- 4.2, P=0.362), or wound complications (34/0 vs. 34/0) (P=1.000) between the two groups at the end of the follow-up time. However, the percutaneous repair group had a shorter average operating time (23.1 +/- 5.2 min) than that of the Mini-open repair group (27.7 +/- 4.3 min) (P=0.000). Conclusions Acute Achilles tendon ruptures may be treated successfully with a new Mini-open repair system or percutaneous repair technique. However, the Mini-open repair system may represent a superior surgical option, since it offers advantages in terms of direct visual control of the repair, AOFAS Ankle-Hindfoot Score, Achilles tendon Total Rupture Score and risk of sural nerve palsy. Study design Case-control studies, Level of evidence, 3.
C1 [Li, Yong; Chen, Hua; Xin, Hongkui; He, Qing; Ruan, Dike] Chinese Peoples Liberat Army Gen Hosp, Med Ctr 6, Dept Orthoped Surg, 6 Fucheng Rd, Beijing 100048, Peoples R China.
   [Chen, Hua] Chinese Peoples Liberat Army Gen Hosp, Dept Orthoped, 28 Fuxing Rd, Beijing 100048, Peoples R China.
RP Li, Y (通讯作者)，Chinese Peoples Liberat Army Gen Hosp, Med Ctr 6, Dept Orthoped Surg, 6 Fucheng Rd, Beijing 100048, Peoples R China.
EM liyong807@163.com
FU new technology & new business projects of Navy General Hospital of
   Chinese PLA
FX This research was supported by the new technology & new business
   projects of Navy General Hospital of Chinese PLA.
CR Alcelik I, 2018, FOOT ANKLE SURG, V24, P427, DOI 10.1016/j.fas.2017.04.016
   Amlang Michael H, 2006, Oper Orthop Traumatol, V18, P287, DOI 10.1007/s00064-006-1178-y
   Assal M, 2002, J BONE JOINT SURG AM, V84A, P161, DOI 10.2106/00004623-200202000-00001
   Baltes TPA, 2017, KNEE SURG SPORT TR A, V25, P1817, DOI 10.1007/s00167-016-4062-9
   Baumfeld D, 2019, FOOT ANKLE SURG, V25, P503, DOI 10.1016/j.fas.2018.03.003
   Bekler HI, 2008, ACTA ORTHOP TRAUMATO, V42, P135
   Bhattacharyya M, 2009, INT ORTHOP, V33, P151, DOI 10.1007/s00264-008-0564-5
   Biz C, 2021, MEDICINA-LITHUANIA, V57, DOI 10.3390/medicina57101073
   Calder JDF, 2005, BRIT J SPORT MED, V39, P857, DOI 10.1136/bjsm.2004.017509
   Ceccarelli F, 2007, CLIN ORTHOP RELAT R, P188, DOI 10.1097/BLO.0b013e3180396f07
   Chen H, 2015, J ORTHOP SURG RES, V10, DOI 10.1186/s13018-015-0310-9
   Clanton T, 2020, ORTHOP CLIN N AM, V51, P391, DOI 10.1016/j.ocl.2020.02.005
   Dams OC, 2019, KNEE SURG SPORT TR A, V27, P2754, DOI 10.1007/s00167-018-5049-5
   Del Buono A, 2014, BRIT MED BULL, V109, P45, DOI 10.1093/bmb/ldt029
   Demetracopoulos CA, 2014, FOOT ANKLE INT, V35, P612, DOI 10.1177/1071100714524550
   Frosch S, 2020, PLOS ONE, V15, DOI 10.1371/journal.pone.0243306
   Gatz M, 2021, ARCH ORTHOP TRAUM SU, V141, P383, DOI 10.1007/s00402-020-03437-z
   Gigante A, 2008, KNEE SURG SPORT TR A, V16, P204, DOI 10.1007/s00167-007-0448-z
   Haji A, 2004, FOOT ANKLE INT, V25, P215, DOI 10.1177/107110070402500404
   He SK, 2022, J INVEST SURG, V35, P157, DOI 10.1080/08941939.2020.1824247
   Hsu AR, 2015, FOOT ANKLE INT, V36, P1279, DOI 10.1177/1071100715589632
   Ismail M, 2008, FOOT ANKLE INT, V29, P808, DOI 10.3113/FAI.2008.0808
   Jaakkola JI, 2001, FOOT ANKLE INT, V22, P979, DOI 10.1177/107110070102201210
   Jiang N, 2012, INT ORTHOP, V36, P765, DOI 10.1007/s00264-011-1431-3
   JOZSA L, 1989, AM J SPORT MED, V17, P338, DOI 10.1177/036354658901700305
   Kadakia AR, 2017, J AM ACAD ORTHOP SUR, V25, P23, DOI 10.5435/JAAOS-D-15-00187
   KAKIUCHI M, 1995, J BONE JOINT SURG BR, V77B, P60, DOI 10.1302/0301-620X.77B1.7822398
   Karabinas Panagiotis K, 2014, Eur J Orthop Surg Traumatol, V24, P607, DOI 10.1007/s00590-013-1350-7
   Keller A, 2014, AM J SPORT MED, V42, P731, DOI 10.1177/0363546513511418
   Khan RJK, 2005, J BONE JOINT SURG AM, V87A, P2202, DOI 10.2106/JBJS.D.03049
   Kupcha Paul C, 2008, Am J Orthop (Belle Mead NJ), V37, P586
   Lacoste S, 2014, ORTHOP TRAUMATOL-SUR, V100, P925, DOI 10.1016/j.otsr.2014.09.018
   Ling Samuel K K, 2017, Foot (Edinb), V33, P35, DOI 10.1016/j.foot.2017.06.002
   MA GWC, 1977, CLIN ORTHOP RELAT R, P247
   Maffulli N, 2020, LANCET, V395, P397, DOI 10.1016/S0140-6736(19)33133-2
   Majewski M, 2006, AM J SPORT MED, V34, P793, DOI 10.1177/0363546505283266
   Martinelli B, 2000, Bull Hosp Jt Dis, V59, P149
   Meulenkamp B, 2018, SYST REV-LONDON, V7, DOI 10.1186/s13643-018-0912-5
   Mukundan C, 2010, Foot Ankle Surg, V16, P122, DOI 10.1016/j.fas.2009.07.005
   Nilsson-Helander K, 2008, KNEE SURG SPORT TR A, V16, P614, DOI 10.1007/s00167-008-0492-3
   Nilsson-Helander K, 2010, AM J SPORT MED, V38, P2186, DOI 10.1177/0363546510376052
   Park CH, 2021, J FOOT ANKLE SURG, V60, P237, DOI 10.1053/j.jfas.2020.03.009
   Qi HZ, 2019, EXP THER MED, V17, P1426, DOI 10.3892/etm.2018.7075
   Rippstein Pascal F, 2002, Foot Ankle Clin, V7, P611, DOI 10.1016/S1083-7515(02)00040-2
   Sliwa Marek, 2014, Pol Orthop Traumatol, V79, P92
   Stecco C, 2013, J ANAT, V223, P665, DOI 10.1111/joa.12111
   Taglialavoro G, 2011, STRATEG TRAUMA LIMB, V6, P147, DOI 10.1007/s11751-011-0124-1
   Tejwani Nirmal C, 2014, Am J Orthop (Belle Mead NJ), V43, pE221
   Vadala A, 2012, J SPORT MED PHYS FIT, V52, P616
   Vadala Antonio, 2014, Muscles Ligaments Tendons J, V4, P177
NR 51
TC 0
Z9 0
U1 1
U2 2
PU BMC
PI LONDON
PA CAMPUS, 4 CRINAN ST, LONDON N1 9XW, ENGLAND
EI 1471-2474
J9 BMC MUSCULOSKEL DIS
JI BMC Musculoskelet. Disord.
PD OCT 30
PY 2021
VL 22
IS 1
AR 914
DI 10.1186/s12891-021-04802-8
PG 9
WC Orthopedics; Rheumatology
WE Science Citation Index Expanded (SCI-EXPANDED)
SC Orthopedics; Rheumatology
GA WP2IV
UT WOS:000712962400001
PM 34717595
OA gold, Green Published
DA 2022-07-22
ER

PT J
AU van Maele, M
   Misselyn, D
   Metsemakers, WJ
   Sermon, A
   Nijs, S
   Hoekstra, H
AF van Maele, Margaux
   Misselyn, Dominique
   Metsemakers, Willem-Jan
   Sermon, An
   Nijs, Stefaan
   Hoekstra, Harm
TI Is open acute Achilles tendon rupture repair still justified? A single
   center experience and critical appraisal of the literature
SO INJURY-INTERNATIONAL JOURNAL OF THE CARE OF THE INJURED
LA English
DT Article
DE Achilles tendon rupture; Functional outcome; Economics
ID SURGICAL REPAIR; ACCELERATED REHABILITATION; NONOPERATIVE TREATMENT;
   NONSURGICAL TREATMENT; COMPLICATIONS; MANAGEMENT; INFECTION; TRIAL
AB Introduction: Acute Achilles tendon ruptures are injuries with multiple treatment strategies with possibly far reaching consequences. Open repair is associated with a high complication rate, whereas percutaneous techniques are associated with higher re-rupture rates. The goal of this study was to evaluate the clinical outcome and economic burden of open surgical repair and define a medically and economically sound treatment protocol for acute Achilles tendon ruptures.
   Methods: Between June 2012 and December 2016 one hundred and five patients with an acute Achilles tendon rupture, treated in an open surgical manner, were studied retrospectively. All demographic, clinical and hospital-related costs were retrieved from the electronic patient database. ATRS questionnaires were sent to assess the functional outcome. A response rate of 70.5% was achieved.
   Results: We recorded a complication rate of 40%, respectively sural nerve hypoesthesia (14.3%), delayed wound healing (28.6%), infection (20.9%) and re-rupture (4.8%). Surgical resident, as primary operating surgeon was associated with a higher complication rate (p = 0.042). Overall, a median functional ATRS score of 17 (IQR 6.5-39.5) was recorded. Infection was associated with significantly higher total healthcare costs per patient as compared to re-rupture ((sic)17,435 vs. (sic)4,537, p = 0.013). The total cost for surgical debridement (n = 6) was approximately 5-times higher than for re-rupture (n = 5), (sic)108,382 vs. (sic)22,272. The median ATRS score for surgical debridement after infection and re-rupture did not differ significantly from the overall ATRS score, respectively 32 (IQR 21-63) and 28 (IQR 15-28). Nevertheless, a difference of 10 points is considered clinically relevant.
   Conclusion: The overall functional outcome of open repair of Achilles tendon ruptures is rather good, however associated with a high complication rate, mainly due to wound problems and infection. Although several risk factors were identified, only the operating surgeon is modifiable. Considering the high total costs for surgical debridement in the context of infection compared to re-rupture surgery, despite equal functional outcome,we decided to change clinical practice to reduce the complication rate and healthcare costs. The outcome and precise costs for percutaneous repair will be addressed. (C) 2018 Elsevier Ltd. All rights reserved.
C1 [van Maele, Margaux] Katholieke Univ Leuven, Univ Leuven, Fac Med, B-3000 Leuven, Belgium.
   [Misselyn, Dominique; Metsemakers, Willem-Jan; Sermon, An; Nijs, Stefaan; Hoekstra, Harm] Univ Hosp Leuven, Dept Trauma Surg, Herestr 49, B-3000 Leuven, Belgium.
   [Metsemakers, Willem-Jan; Sermon, An; Nijs, Stefaan; Hoekstra, Harm] Katholieke Univ Leuven, Univ Leuven, Dept Dev & Regenerat, B-3000 Leuven, Belgium.
RP Hoekstra, H (通讯作者)，Univ Hosp Leuven, Dept Trauma Surg, Herestr 49, B-3000 Leuven, Belgium.
EM harm.hoekstra@uzleuven.be
RI Misselyn, Dominique/AAE-2084-2019; Metsemakers, Willem-Jan/F-8647-2016
OI Metsemakers, Willem-Jan/0000-0002-4114-9093; Nijs,
   Stefaan/0000-0002-1119-4918; Hoekstra, Harm/0000-0001-8061-1060; Sermon,
   An/0000-0001-6295-9127
CR Alcelik I, 2017, FOOT ANKLE SURG, V4
   BRADLEY JP, 1990, AM J SPORT MED, V18, P188, DOI 10.1177/036354659001800213
   Carmont MR, 2013, KNEE SURG SPORT TR A, V21, P1361, DOI 10.1007/s00167-013-2423-1
   Cretnik A, 2005, AM J SPORT MED, V33, P1369, DOI 10.1177/0363546504271501
   Ganestam A, 2016, KNEE SURG SPORT TR A, V24, P3730, DOI 10.1007/s00167-015-3544-5
   Holm C, 2015, SCAND J MED SCI SPOR, V25, pE1, DOI 10.1111/sms.12209
   Khan RJK, 2005, J BONE JOINT SURG AM, V87A, P2202, DOI 10.2106/JBJS.D.03049
   Khan RJK, 2005, J BONE JT SURG, V87
   Leppilahti J, 1996, ACTA ORTHOP SCAND, V67, P277, DOI 10.3109/17453679608994688
   Lonzaric D, 2017, WIEN KLIN WOCHENSCHR, V26
   Metsemakers WJ, 2017, INJURY, V48, P1204, DOI 10.1016/j.injury.2017.03.030
   Metz R, 2008, AM J SPORT MED, V36, P1688, DOI 10.1177/0363546508319312
   Monteban P, 2017, EUR J TRAUMA EMERG S, V12, P1
   Nilsson-Helander K, 2007, AM J SPORT MED, V35, P421, DOI 10.1177/0363546506294856
   Nilsson-Helander K, 2010, AM J SPORT MED, V38, P2186, DOI 10.1177/0363546510376052
   Olsson N, 2013, AM J SPORT MED, V41, P2867, DOI 10.1177/0363546513503282
   Opdam KTM, 2016, KNEE SURG SPORT TR A, V26, P1
   Pajala A, 2002, J BONE JOINT SURG AM, V84A, P2016, DOI 10.2106/00004623-200211000-00017
   Porter MD, 2015, ANZ J SURG, V85, P373, DOI 10.1111/ans.12910
   Reul M, 2017, EUR J TRAUMA EMERG S, V30, P1
   Seeger JD, 2006, PHARMACOEPIDEM DR S, V15, P784, DOI 10.1002/pds.1214
   Smeets B, 2016, J FOOT ANKLE SURG, V55, P535, DOI 10.1053/j.jfas.2016.02.004
   Tejwani Nirmal C, 2014, Am J Orthop (Belle Mead NJ), V43, pE221
   Truntzer JN, 2017, J AM ACAD ORTHOP SUR, V25, P449, DOI 10.5435/JAAOS-D-16-00553
   Wang Ting, 2012, Zhongguo Xiu Fu Chong Jian Wai Ke Za Zhi, V26, P814
   Wilkins R, 2012, AM J SPORT MED, V40, P2154, DOI 10.1177/0363546512453293
   Willits K, 2010, J BONE JOINT SURG AM, V92A, P2767, DOI 10.2106/JBJS.I.01401
   Wu YH, 2016, INT J SURG, V36, P305, DOI 10.1016/j.ijsu.2016.11.014
   Yang B, 2017, INT J SURG, V40, P178, DOI 10.1016/j.ijsu.2017.03.021
   Zhang H, 2015, MEDICINE, V94, DOI 10.1097/MD.0000000000001951
NR 30
TC 9
Z9 9
U1 0
U2 5
PU ELSEVIER SCI LTD
PI OXFORD
PA THE BOULEVARD, LANGFORD LANE, KIDLINGTON, OXFORD OX5 1GB, OXON, ENGLAND
SN 0020-1383
EI 1879-0267
J9 INJURY
JI Injury-Int. J. Care Inj.
PD OCT
PY 2018
VL 49
IS 10
BP 1947
EP 1952
DI 10.1016/j.injury.2018.08.012
PG 6
WC Critical Care Medicine; Emergency Medicine; Orthopedics; Surgery
WE Science Citation Index Expanded (SCI-EXPANDED)
SC General & Internal Medicine; Emergency Medicine; Orthopedics; Surgery
GA GU0AB
UT WOS:000444908500035
PM 30144965
OA Green Accepted
DA 2022-07-22
ER

PT J
AU Chai, JH
   Bae, SW
AF Chai, Jung-Hoon
   Bae, Sang-Won
TI Evaluation of Calf Muscular Function During the Recovery Phase After the
   Repair of an Achilles Tendon Rupture
SO FRONTIERS IN SURGERY
LA English
DT Article
DE tensiomyography; Achilles tendon rupture repair; maximal displacement;
   muscle function; recovery process
ID SKIN; FAT
AB Background: During the recovery phase after the repair of an Achilles tendon rupture, measuring calf muscular function is important for predicting prognosis. Tensiomyography (TMG) is a selective and non-invasive diagnostic method for skeletal muscular contractile properties based on the displacement of the muscle belly.
   Case Presentation: Tensiomyography gives information about maximal displacement of the muscle belly (Dm), delay time, contraction time (Tc), sustain time, and relaxation time. Using Tensiomyography we evaluated a patient that had Achilles tendon rupture surgery. The contralateral normal side measurements were also performed for evaluation and comparison of the site of injury.
   Findings: In this study, the maximal displacement of the muscle belly changed significantly compared to other parameters. The maximal displacement of the muscle belly decreased after cast removal day and increased gradually during the early recovery phase and then slightly decreased again during the late recovery phase.
   Conclusions: These responses of the maximal displacement of muscle belly show a correlation with the recovery of muscular function.
C1 [Chai, Jung-Hoon] Soonchunhyang Univ, Dept Sports Med, Asan, South Korea.
   [Bae, Sang-Won] Sunsoochon Hosp, Dept Orthoped Surg, Seoul, South Korea.
RP Bae, SW (通讯作者)，Sunsoochon Hosp, Dept Orthoped Surg, Seoul, South Korea.
EM yodeo@naver.com
RI Chai, Jung-Hoon/AAQ-6641-2020
CR Alentorn-Geli E, 2015, KNEE SURG SPORT TR A, V23, P2502, DOI 10.1007/s00167-014-3007-4
   Garcia-Garcia O, 2017, J HUM KINET, V58, P23, DOI 10.1515/hukin-2017-0066
   Lieber RL., 2010, SKELETAL MUSCLE STRU
   Narici M, 1998, J GRAVIT PHYSL, V5, P73
   Petrofsky J, 2008, MED ENG PHYS, V30, P1168, DOI 10.1016/j.medengphy.2008.02.009
   Petrofsky JS, 2008, MED ENG PHYS, V30, P931, DOI 10.1016/j.medengphy.2007.12.007
   Pisot R, 2008, EUR J APPL PHYSIOL, V104, P409, DOI 10.1007/s00421-008-0698-6
   Simunic B, 2011, MED SCI SPORT EXER, V43, P1619, DOI 10.1249/MSS.0b013e31821522d0
   Tous-Fajardo J, 2010, J ELECTROMYOGR KINES, V20, P761, DOI 10.1016/j.jelekin.2010.02.008
   Zubac D, 2017, J STRENGTH COND RES, V31, P1610, DOI 10.1519/JSC.0000000000001626
NR 11
TC 2
Z9 2
U1 0
U2 4
PU FRONTIERS MEDIA SA
PI LAUSANNE
PA AVENUE DU TRIBUNAL FEDERAL 34, LAUSANNE, CH-1015, SWITZERLAND
SN 2296-875X
J9 FRONT SURG
JI Front. Surg.
PD SEP 24
PY 2019
VL 6
AR 57
DI 10.3389/fsurg.2019.00057
PG 5
WC Surgery
WE Science Citation Index Expanded (SCI-EXPANDED)
SC Surgery
GA JB1IR
UT WOS:000488314600001
PM 31608286
OA gold, Green Published
DA 2022-07-22
ER

PT J
AU Zhang, K
   Asai, S
   Hast, MW
   Liu, M
   Usami, Y
   Iwamoto, M
   Soslowsky, LJ
   Enomoto-Iwamoto, M
AF Zhang, Kairui
   Asai, Shuji
   Hast, Michael W.
   Liu, Min
   Usami, Yu
   Iwamoto, Masahiro
   Soslowsky, Louis J.
   Enomoto-Iwamoto, Motomi
TI Tendon mineralization is progressive and associated with deterioration
   of tendon biomechanical properties, and requires BMP-Smad signaling in
   the mouse Achilles tendon injury model
SO MATRIX BIOLOGY
LA English
DT Article
DE Achilles tendon; Injury; Ectopic mineralization; Biomechanics; Smad; BMP
ID FIBRODYSPLASIA OSSIFICANS PROGRESSIVA; HETEROTOPIC OSSIFICATION; CELLS;
   BONE; CALCIFICATIONS; IDENTIFICATION; TENDINOPATHY; INHIBITION;
   EXPRESSION
AB Ectopic tendon mineralization can develop following tendon rupture or trauma surgery. The pathogenesis of ectopic tendon mineralization and its clinical impact have not been fully elucidated yet. In this study, we utilized a mouse Achilles tendon injury model to determine whether ectopic tendon mineralization alters the biomechanical properties of the tendon and whether BMP signaling is involved in this condition. A complete transverse incision was made at the midpoint of the right Achilles tendon in 8-week-old CD1 mice and the gap was left open. Ectopic cartilaginous mass formation was found in the injured tendon by 4 weeks post-surgery and ectopic mineralization was detected at 8 to 10 weeks post-surgery. Ectopic mineralization grew over time and volume of the mineralized materials of 25-weeks samples was about 2.5 fold bigger than that of 10-weeks samples, indicating that injury-induced ectopic tendon mineralization is progressive. In vitro mechanical testing showed that max force, max stress and mid-substance modulus in the 25-weeks samples were significantly lower than the 10-weeks samples. We observed substantial increases in expression of bone morphogenetic protein family genes in injured tendons 1 week post-surgery. Immunohistochemical analysis showed that phosphorylation of both Smad1 and Smad3 was highly increased in injured tendons as early as 1 week post-injury and remained high in ectopic chondrogenic lesions 4-weeks post-injury. Treatment with the BMP receptor kinase inhibitor (LDN193189) significantly inhibited injury-induced tendon mineralization. These findings indicate that injury-induced ectopic tendon mineralization is progressive, involves BMP signaling and associated with deterioration of tendon biomechanical properties. (C) 2016 Elsevier B.V. All rights reserved.
C1 [Zhang, Kairui; Asai, Shuji; Liu, Min; Usami, Yu; Iwamoto, Masahiro; Enomoto-Iwamoto, Motomi] Childrens Hosp Philadelphia, Div Orthopaed Surg, 3615 Civ Ctr Blvd,ARC 902, Philadelphia, PA 19104 USA.
   [Zhang, Kairui] Southern Med Univ, Dept Orthopaed & Traumatol, Nanfang Hosp, Guangzhou, Guangdong, Peoples R China.
   [Asai, Shuji] Nagoya Univ, Grad Sch Med, Dept Orthopaed Surg, Nagoya, Aichi 4648601, Japan.
   [Hast, Michael W.; Soslowsky, Louis J.] Univ Penn, McKay Orthopaed Res Lab, Dept Orthopaed Surg, Philadelphia, PA 19104 USA.
RP Enomoto-Iwamoto, M (通讯作者)，Childrens Hosp Philadelphia, Div Orthopaed Surg, 3615 Civ Ctr Blvd,ARC 902, Philadelphia, PA 19104 USA.
EM iwamotom1@email.chop.edu
FU PCMD Pilot and Feasibility Grant (NIH/NIAMS) [P30AR050950]; NIH
   [R21AR062193]; Children's Hospital of Philadelphia; NATIONAL INSTITUTE
   OF ARTHRITIS AND MUSCULOSKELETAL AND SKIN DISEASES [P30AR050950,
   P30AR069619, R21AR062193] Funding Source: NIH RePORTER
FX We thank Miss L. Cantley, A. T. Gunawardena and N. Francois for the
   technical assistance. We also thank technical assistance for the
   Biomechanics Core of the Penn Center for Musculoskeletal Disorders
   (PCMD). This study was supported by the PCMD Pilot and Feasibility Grant
   (NIH/NIAMS P30AR050950), the NIH R21AR062193 Grant and the
   interdepartmental fund of the Children's Hospital of Philadelphia.
CR Agabalyan NA, 2013, J ANAT, V223, P262, DOI 10.1111/joa.12078
   Asai S., 2014, STEM CELLS
   Ateschrang A, 2008, ARCH ORTHOP TRAUM SU, V128, P1087, DOI 10.1007/s00402-007-0441-5
   Bi YM, 2007, NAT MED, V13, P1219, DOI 10.1038/nm1630
   Blankstein A, 2001, ISRAEL MED ASSOC J, V3, P575
   Cai J, 2012, FEBS LETT, V586, P1993, DOI 10.1016/j.febslet.2012.04.030
   Connizzo BK, 2013, J BIOMECH ENG-T ASME, V135, DOI 10.1115/1.4023234
   Cuny GD, 2008, BIOORG MED CHEM LETT, V18, P4388, DOI 10.1016/j.bmcl.2008.06.052
   Fenwick S, 2002, RHEUMATOLOGY, V41, pA474, DOI 10.1093/rheumatology/41.4.474
   Hatori M, 2002, ARCH ORTHOP TRAUM SU, V122, P414, DOI 10.1007/s00402-002-0412-9
   HATORI M, 1994, FOOT ANKLE, V15, P44, DOI 10.1177/107110079401500109
   Hatsell SJ, 2015, SCI TRANSL MED, V7, DOI 10.1126/scitranslmed.aac4358
   KANNUS P, 1991, J BONE JOINT SURG AM, V73A, P1507, DOI 10.2106/00004623-199173100-00009
   Kaplan FS, 2007, J BONE JOINT SURG AM, V89A, P347, DOI 10.2106/JBJS.F.00472
   Kaplan FS, 2012, DIS MODEL MECH, V5, P756, DOI 10.1242/dmm.010280
   Kraus R, 2004, FOOT ANKLE INT, V25, P827, DOI 10.1177/107110070402501113
   Lake SP, 2009, J ORTHOP RES, V27, P1596, DOI 10.1002/jor.20938
   Lin L, 2010, BONE, V46, P425, DOI 10.1016/j.bone.2009.08.057
   Lounev VY, 2009, J BONE JOINT SURG AM, V91A, P652, DOI 10.2106/JBJS.H.01177
   Lui PPY, 2011, J ORTHOP RES, V29, P816, DOI 10.1002/jor.21313
   Malhotra R, 2015, PLOS ONE, V10, DOI 10.1371/journal.pone.0117098
   O'Brien EJO, 2012, INT J EXP PATHOL, V93, P319, DOI 10.1111/j.1365-2613.2012.00829.x
   O'Brien EJO, 2013, J ORTHOP RES, V31, P1520, DOI 10.1002/jor.22404
   Oliva F, 2012, BMC MED, V10, DOI 10.1186/1741-7015-10-95
   Oliva F, 2011, SPORTS MED ARTHROSC, V19, P237, DOI 10.1097/JSA.0b013e318225bc5f
   Peterson JR, 2015, JOVE-J VIS EXP, DOI 10.3791/52880
   Richards PJ, 2008, DISABIL REHABIL, V30, P1651, DOI 10.1080/09638280701785866
   ROONEY P, 1992, MATRIX, V12, P274, DOI 10.1016/S0934-8832(11)80079-X
   Rui YF, 2012, KNEE SURG SPORT TR A, V20, P1409, DOI 10.1007/s00167-011-1685-8
   Salisbury E, 2011, J CELL BIOCHEM, V112, P2748, DOI 10.1002/jcb.23225
   Shimono K, 2011, NAT MED, V17, P454, DOI 10.1038/nm.2334
   Suda RK, 2009, STEM CELLS, V27, P2209, DOI 10.1002/stem.150
   Yu PB, 2008, NAT MED, V14, P1363, DOI 10.1038/nm.1888
NR 33
TC 26
Z9 27
U1 0
U2 14
PU ELSEVIER SCIENCE BV
PI AMSTERDAM
PA PO BOX 211, 1000 AE AMSTERDAM, NETHERLANDS
SN 0945-053X
EI 1569-1802
J9 MATRIX BIOL
JI Matrix Biol.
PD MAY-JUL
PY 2016
VL 52-54
SI SI
BP 315
EP 324
DI 10.1016/j.matbio.2016.01.015
PG 10
WC Biochemistry & Molecular Biology; Cell Biology
WE Science Citation Index Expanded (SCI-EXPANDED)
SC Biochemistry & Molecular Biology; Cell Biology
GA DP0PC
UT WOS:000378190700026
PM 26825318
OA Green Accepted
DA 2022-07-22
ER

PT J
AU Gwynne-Jones, DP
   Sims, M
   Handcock, D
AF Gwynne-Jones, David P.
   Sims, Martyn
   Handcock, Deb
TI Epidemiology and Outcomes of Acute Achilles Tendon Rupture with
   Operative or Nonoperative Treatment Using an Identical Functional
   Bracing Protocol
SO FOOT & ANKLE INTERNATIONAL
LA English
DT Article
DE Achilles Tendon; Rupture; Functional Rehabilitation; Operative Repair
ID NONSURGICAL TREATMENT; CONTROLLED-TRIALS; NETBALL INJURIES; EARLY
   MOTION; REPAIR; METAANALYSIS; RERUPTURE; REHABILITATION; SURGERY; RISK
AB Background: This study reports on the demographics of acute Achilles tendon rupture in our region and compares the results of a selective approach to operative and nonoperative treatment using an identical rehabilitation program with functional bracing. Materials and Methods: A consecutive series of 363 patients, aged 15 to 60 years, treated over 8.5 years by either open operative repair (143) or nonoperatively (220) were compared with respect to demographics, re-rupture rate, and major wound complication. Results: There was an almost equal number of males (159) and females (152) up to age 50 years but males comprised 73% of patients aged 51 to 60 years. Netball was the most common cause of injury and explains the relatively high incidence in females. In the 143 patients treated surgically there were two re-ruptures (1.4%) and two reoperations for wound complications (1.4%). In the 220 patients treated nonoperatively there were 19 re-ruptures (8.6%), 13 of 113 males (11.5%) and six of 107 females (5.6%). There was a significantly lower re-rupture rate, and reoperation rate in the surgical group (p < 0.05). In the nonoperative group there was a significantly lower rate of re-rupture in patients over 40 (six of 119) (4.1%) compared with those 40 years and under (13 of 99, 13.1%) and between females over 40 when compared with males 40 years and under. Conclusion: In our region there is a high incidence of Achilles tendon rupture among women due to netball and results in a younger age of injury than previously reported. Our results support surgery in patients less than 40 years, particularly males, if there are no contraindications. Functional bracing as part of nonoperative treatment can result in low re-rupture rates in patients over 40, especially in females.
C1 [Gwynne-Jones, David P.] Dunedin Publ Hosp, Dept Orthopaed Surg, Dunedin, New Zealand.
RP Gwynne-Jones, DP (通讯作者)，Dunedin Publ Hosp, Dept Orthopaed Surg, Dunedin, New Zealand.
EM david.gwynne-jones@otago.ac.nz
RI Jones, David Gwynne-/AAJ-7059-2020
OI Jones, David Gwynne-/0000-0001-8484-614X
CR Bhandari M, 2002, CLIN ORTHOP RELAT R, P190
   BRUGGEMAN NB, 2004, CLIN ORTHOP RELAT R, V427, P63
   CETTI R, 1993, AM J SPORT MED, V21, P791, DOI 10.1177/036354659302100606
   Chiodo CP, 2006, FOOT ANKLE INT, V27, P305, DOI 10.1177/107110070602700415
   Costa ML, 2006, J BONE JOINT SURG BR, V88B, P69, DOI 10.1302/0301-620X.88B1.16549
   Cretnik A, 2005, AM J SPORT MED, V33, P1369, DOI 10.1177/0363546504271501
   Flood L, 2009, MED J AUSTRALIA, V190, P87, DOI 10.5694/j.1326-5377.2009.tb02285.x
   Haji A, 2004, FOOT ANKLE INT, V25, P215, DOI 10.1177/107110070402500404
   Hopper D, 1995, BRIT J SPORT MED, V29, P223, DOI 10.1136/bjsm.29.4.223
   Houshian S, 1998, INJURY, V29, P651, DOI 10.1016/S0020-1383(98)00147-8
   Hufner TM, 2006, FOOT ANKLE INT, V27, P167, DOI 10.1177/107110070602700302
   Ingvar J, 2005, ACTA ORTHOP, V76, P597, DOI 10.1080/17453670510041619
   Jacob KM, 2007, ANZ J SURG, V77, P287, DOI 10.1111/j.1445-2197.2007.04035.x
   Khan RJK, 2005, J BONE JOINT SURG AM, V87A, P2202, DOI 10.2106/JBJS.D.03049
   Lansdaal JR, 2007, INJURY, V38, P839, DOI 10.1016/j.injury.2006.12.010
   Maffulli N, 1999, CLIN J SPORT MED, V9, P157, DOI 10.1097/00042752-199907000-00007
   Metz R, 2008, AM J SPORT MED, V36, P1688, DOI 10.1177/0363546508319312
   Moller M, 2001, J BONE JOINT SURG BR, V83B, P843
   Mortensen NHM, 1999, J BONE JOINT SURG AM, V81A, P983, DOI 10.2106/00004623-199907000-00011
   NISTOR L, 1981, J BONE JOINT SURG AM, V63, P394, DOI 10.2106/00004623-198163030-00012
   Nyyssonen T, 2008, SCAND J SURG, V97, P272, DOI 10.1177/145749690809700312
   Pajala A, 2002, J BONE JOINT SURG AM, V84A, P2016, DOI 10.2106/00004623-200211000-00017
   Rettig AC, 2005, AM J SPORT MED, V33, P119, DOI 10.1177/0363546504268720
   Strauss EJ, 2007, INJURY, V38, P832, DOI 10.1016/j.injury.2006.06.005
   Suchak AA, 2005, FOOT ANKLE INT, V26, P932, DOI 10.1177/107110070502601106
   Suchak AA, 2006, CLIN ORTHOP RELAT R, P216, DOI 10.1097/01.blo.0000203458.05135.74
   Twaddle BC, 2007, AM J SPORT MED, V35, P2033, DOI 10.1177/0363546507307503
   van der Linden-van der Zwaag HMJ, 2004, INT ORTHOP, V28, P370, DOI 10.1007/s00264-004-0575-9
   Wallace RGH, 2004, J BONE JOINT SURG AM, V86A, P1198, DOI 10.2106/00004623-200406000-00011
   Weber M, 2003, AM J SPORT MED, V31, P685, DOI 10.1177/03635465030310050901
   WILLS CA, 1986, CLIN ORTHOP RELAT R, V207, P156
NR 31
TC 73
Z9 75
U1 0
U2 6
PU SAGE PUBLICATIONS INC
PI THOUSAND OAKS
PA 2455 TELLER RD, THOUSAND OAKS, CA 91320 USA
SN 1071-1007
EI 1944-7876
J9 FOOT ANKLE INT
JI Foot Ankle Int.
PD APR
PY 2011
VL 32
IS 4
BP 337
EP 343
DI 10.3113/FAI.2011.0337
PG 7
WC Orthopedics
WE Science Citation Index Expanded (SCI-EXPANDED)
SC Orthopedics
GA 742XV
UT WOS:000288979500005
PM 21733434
DA 2022-07-22
ER

PT J
AU Schepull, T
   Kvist, J
   Aspenberg, P
AF Schepull, T.
   Kvist, J.
   Aspenberg, P.
TI Early E-modulus of healing Achilles tendons correlates with late
   function: Similar results with or without surgery
SO SCANDINAVIAN JOURNAL OF MEDICINE & SCIENCE IN SPORTS
LA English
DT Article
DE achilles tendon rupture; tendon healing; biomechanics; modulus of
   elasticity
ID ROENTGEN STEREOPHOTOGRAMMETRIC ANALYSIS; SURGICAL REPAIR; RUPTURE; RATS;
   METAANALYSIS; INJECTION; ENDURANCE
AB Non-operative treatment of Achilles tendon ruptures is associated with an increased risk of rerupture. We hypothesized that this is due to inferior mechanical properties during an early phase of healing, and performed a randomized trial, using a new method to measure the mechanical properties. Tantalum markers were inserted in the tendon stumps, and tendon strain at different loadings was measured by stereo-radiography (Roentgen stereophotogrammetric analysis) at 3, 7 and 19 weeks and 18 months after injury. Thirty patients were randomized to operative or non-operative treatment. The primary out-come variable was an estimate for the modulus of elasticity at 7 weeks. Strain per force, cross-sectional area and tendon elongation were also measured. The functional outcome variable was the heel-raise index after 18 months. There was no difference in the mean modulus of elasticity or other mechanical or functional variables between operative and non-operative treatments at any time-point, but strain per force at 7 and 19 weeks had a significantly larger variation in the non-operative group. This group, therefore, might contain more outliers with poor healing. The modulus of elasticity at 7 weeks correlated with the heel-raise index after 18 months in both treatment groups (r2=0.75; P=0.0001). This correlation is an intriguing finding.
C1 [Schepull, T.; Aspenberg, P.] Linkoping Univ Hosp, Fac Hlth Sci, Dept Neurosci & Locomot, Sect Orthopaed & Sports Med, S-58185 Linkoping, Sweden.
   [Kvist, J.] Dept Med & Hlth Sci, Sect Physiotherapy, Linkoping, Sweden.
RP Schepull, T (通讯作者)，Linkoping Univ Hosp, Fac Hlth Sci, Dept Neurosci & Locomot, Sect Orthopaed & Sports Med, S-58185 Linkoping, Sweden.
EM thorsten.schepull@lio.se
RI Kvist, Joanna/S-8023-2017
OI Kvist, Joanna/0000-0003-3527-5488
FU Swedish Medical Research council; Swedish Center for Sports Medicine
   Research; King Gustav V and Queen Victoria Free Mason fund
FX This study was supported by the Swedish Medical Research council, The
   Swedish Center for Sports Medicine Research, and the King Gustav V and
   Queen Victoria Free Mason fund.
CR Aktas S, 2009, FOOT ANKLE INT, V30, P391, DOI 10.3113/FAI.2009.0391
   Aspenberg P, 2004, ACTA ORTHOP SCAND, V75, P93, DOI 10.1080/00016470410001708190
   Bhandari M, 2002, CLIN ORTHOP RELAT R, P190
   ENWEMEKA CS, 1988, AM J PHYS MED REHAB, V67, P264
   Forslund C, 2003, AM J SPORT MED, V31, P555, DOI 10.1177/03635465030310041301
   Forslund C, 2003, ACTA ORTHOP SCAND, V74, P465, DOI 10.1080/00016470310017802
   Fujikawa A, 2007, AM J ROENTGENOL, V189, P1169, DOI 10.2214/AJR.07.2260
   Gigante A, 2008, KNEE SURG SPORT TR A, V16, P204, DOI 10.1007/s00167-007-0448-z
   Kangas J, 2007, AM J SPORT MED, V35, P59, DOI 10.1177/0363546506293255
   Khan RJK, 2005, J BONE JOINT SURG AM, V87A, P2202, DOI 10.2106/JBJS.D.03049
   Lo IKY, 1997, CLIN J SPORT MED, V7, P207, DOI 10.1097/00042752-199707000-00010
   Lynch Richard M, 2004, Accid Emerg Nurs, V12, P149, DOI 10.1016/j.aaen.2003.11.004
   Moller M, 2001, J BONE JOINT SURG BR, V83B, P843
   Moller M, 2005, KNEE SURG SPORT TR A, V13, P60, DOI 10.1007/s00167-003-0441-0
   Mortensen NHM, 1999, J BONE JOINT SURG AM, V81A, P983, DOI 10.2106/00004623-199907000-00011
   Nilsson-Helander K, 2007, AM J SPORT MED, V35, P421, DOI 10.1177/0363546506294856
   Nilsson-Helander K, 2009, KNEE SURG SPORT TR A, V17, P1234, DOI 10.1007/s00167-009-0727-y
   NISTOR L, 1981, J BONE JOINT SURG AM, V63, P394, DOI 10.2106/00004623-198163030-00012
   Pajala A, 2002, J BONE JOINT SURG AM, V84A, P2016, DOI 10.2106/00004623-200211000-00017
   Schepull T, 2007, BMC MUSCULOSKEL DIS, V8, DOI 10.1186/1471-2474-8-116
   SELVIK G, 1990, ACTA RADIOL, V31, P113, DOI 10.1177/028418519003100201
   Silbernagel KG, 2010, KNEE SURG SPORT TR A, V18, P258, DOI 10.1007/s00167-009-0889-7
   Suchak AA, 2008, J BONE JOINT SURG AM, V90A, P1876, DOI 10.2106/JBJS.G.01242
   Virchenko O, 2004, AM J SPORT MED, V32, P1743, DOI 10.1177/0363546504263403
NR 24
TC 52
Z9 54
U1 0
U2 3
PU WILEY
PI HOBOKEN
PA 111 RIVER ST, HOBOKEN 07030-5774, NJ USA
SN 0905-7188
EI 1600-0838
J9 SCAND J MED SCI SPOR
JI Scand. J. Med. Sci. Sports
PD FEB
PY 2012
VL 22
IS 1
BP 18
EP 23
DI 10.1111/j.1600-0838.2010.01154.x
PG 6
WC Sport Sciences
WE Science Citation Index Expanded (SCI-EXPANDED)
SC Sport Sciences
GA 874UT
UT WOS:000298984300004
PM 20673250
OA Green Submitted
DA 2022-07-22
ER

PT J
AU Sun, D
   Fekete, G
   Mei, QC
   Gu, YD
AF Sun, Dong
   Fekete, Gusztav
   Mei, Qichang
   Gu, Yaodong
TI Gait Abnormality and Asymmetry Analysis After 18-24 Months Surgical
   Repair of Unilateral Achilles Tendon Rupture
SO JOURNAL OF MEDICAL IMAGING AND HEALTH INFORMATICS
LA English
DT Article
DE Achilles Tendon Rupture; Gait Abnormality; Asymmetry; Lower Limbs
ID STRENGTH; MOMENTS; WALKING; INJURY; HIP
AB Incidence of Achilles tendon rupture (ATR) highly increased over the last years, which can alter tissue composition while having non-wanted effects on long-term tendon mechanics and lower limb functions during gait movement. 10 male athletes were recruited to follow up their rehabilitation 18-24 months after a percutaneous unilateral ATR surgical repair. Using ultrasonography, the Achilles tendon (AT) elongation of these athletes could be clearly detected. During the experiments, kinematics (200 Hz), ground reaction forces (1000 Hz) and subdivision plantar pressure (50 Hz) were measured bilaterally, while paired sample f-tests were used to evaluate differences between injured and uninjured limbs. According to the obtained results, the increased ankle dorsiflexion angle and decreased knee flexion may be the two governing compensation mechanisms for functional deficit of elongated AT. It is also suggested that due to the elongated AT the knee is overused, which results that injuries and tibia stress fracture risks may be increased as well. This idea is supported by the obtained results, since significantly greater loading rate, hip adduction angle and knee adduction moment, were found in the injured limb. It is worth mentioning that increased ankle inversion angle accompanied with laterally shifted planter pressure distribution indicate higher ankle sprain injury risk as well. Greater symmetry angle were found during walking. It is supposed that side to side gait variable differences, found between lower limbs, may be related to the AT elongation of surgical repaired side. Increased asymmetry angle found in walking of AT ruptured athletes should pay additional attention in the future studies.
C1 [Sun, Dong; Mei, Qichang; Gu, Yaodong] Ningbo Univ, Fac Sports Sci, Ningbo 315211, Zhejiang, Peoples R China.
   [Sun, Dong; Fekete, Gusztav] Univ Pannonia, Fac Engn, H-8200 Veszprem, Hungary.
   [Fekete, Gusztav] Eotvos Lorand Univ, Savaria Inst Technol, H-9700 Szombathely, Hungary.
   [Mei, Qichang] Univ Auckland, Auckland Bioengn Inst, Auckland 1010, New Zealand.
RP Gu, YD (通讯作者)，Ningbo Univ, Fac Sports Sci, Ningbo 315211, Zhejiang, Peoples R China.
RI Mei, Qichang/AAF-2467-2019; Gu, Yaodong/AAJ-4148-2020; Gu,
   Yaodong/AAH-7551-2020; Mei, Qichang/AAN-4052-2020; Fekete,
   Gusztáv/ABG-2914-2020
OI Mei, Qichang/0000-0003-1113-877X; Fekete, Gusztáv/0000-0002-6138-8382
FU National Natural Science Foundation of China [81772423]; K. C. Wong
   Magna Fund in Ningbo University; ANTA Sports Products Co., Ltd.
FX This study sponsored by National Natural Science Foundation of China
   (81772423), K. C. Wong Magna Fund in Ningbo University and ANTA Sports
   Products Co., Ltd.
CR Alexander LD, 2009, STROKE, V40, P537, DOI 10.1161/STROKEAHA.108.527374
   Brorsson A, 2017, AM J SPORT MED, V45, P3060, DOI 10.1177/0363546517717698
   Chang A, 2010, ARTHRITIS RHEUM-US, V62, P1403, DOI 10.1002/art.27377
   Costa ML, 2005, J BONE JOINT SURG BR, V87B, P1085, DOI 10.1302/0301-620X.87B8.16540
   Don R, 2007, CLIN BIOMECH, V22, P211, DOI 10.1016/j.clinbiomech.2006.10.001
   Farris DJ, 2011, J BIOMECH, V44, P822, DOI 10.1016/j.jbiomech.2010.12.015
   Geremia JM, 2015, CLIN BIOMECH, V30, P485, DOI 10.1016/j.clinbiomech.2015.03.005
   Jandacka Daniel, 2013, BMJ Case Rep, V2013, DOI 10.1136/bcr-2012-007370
   Kean CO, 2015, CLIN BIOMECH, V30, P226, DOI 10.1016/j.clinbiomech.2015.01.008
   KNAPIK JJ, 1991, AM J SPORT MED, V19, P76, DOI 10.1177/036354659101900113
   Kondo H, 2016, J PHYS THER SCI, V28, P319, DOI 10.1589/jpts.28.319
   Konradsen L, 2002, J Electromyogr Kinesiol, V12, P199, DOI 10.1016/S1050-6411(02)00021-4
   Kristianslund E, 2012, J BIOMECH, V45, P666, DOI 10.1016/j.jbiomech.2011.12.011
   Kujala UM, 2005, CLIN J SPORT MED, V15, P133, DOI 10.1097/01.jsm.0000165347.55638.23
   Kutzner I, 2013, PLOS ONE, V8, DOI 10.1371/journal.pone.0081036
   Lathrop-Lambach RL, 2014, GAIT POSTURE, V40, P526, DOI 10.1016/j.gaitpost.2014.06.010
   Maffulli N, 1999, J BONE JOINT SURG AM, V81A, P1019, DOI 10.2106/00004623-199907000-00017
   Maffulli N, 1998, AM J SPORT MED, V26, P266, DOI 10.1177/03635465980260021801
   Maquirriain Javier, 2011, Yale Journal of Biology and Medicine, V84, P289
   Mei QC, 2015, J MECH MED BIOL, V15, DOI 10.1142/S0219519415500141
   MUELLER MJ, 1995, PHYS THER, V75, P684, DOI 10.1093/ptj/75.8.684
   Naim Fadel, 2005, Acta Orthop Traumatol Turc, V39, P1
   Neptune RR, 2001, J BIOMECH, V34, P1387, DOI 10.1016/S0021-9290(01)00105-1
   Nigg BM, 2001, CLIN J SPORT MED, V11, P2, DOI 10.1097/00042752-200101000-00002
   Plotnik M, 2013, GAIT POSTURE, V38, P864, DOI 10.1016/j.gaitpost.2013.04.011
   Polk JD, 2017, GAIT POSTURE, V52, P140, DOI 10.1016/j.gaitpost.2016.11.028
   Radzak KN, 2017, GAIT POSTURE, V51, P268, DOI 10.1016/j.gaitpost.2016.11.005
   Sadeghi H, 1997, HUM MOVEMENT SCI, V16, P243, DOI 10.1016/S0167-9457(96)00054-1
   Sadeghi H, 2003, GAIT POSTURE, V17, P197, DOI 10.1016/S0966-6362(02)00089-9
   Shin CS, 2009, J BIOMECH, V42, P280, DOI 10.1016/j.jbiomech.2008.10.031
   Silbernagel KG, 2012, J ORTHOP SPORT PHYS, V42, P521, DOI 10.2519/jospt.2012.3913
   Stenroth L, 2012, J APPL PHYSIOL, V113, P1537, DOI 10.1152/japplphysiol.00782.2012
   Vincent KR, 2011, MED SCI SPORT EXER, V43, P60, DOI 10.1249/01.MSS.0000402851.25234.2b
   Zifchock RA, 2008, GAIT POSTURE, V27, P622, DOI 10.1016/j.gaitpost.2007.08.006
   Zifchock RA, 2008, HUM MOVEMENT SCI, V27, P888, DOI 10.1016/j.humov.2008.03.007
NR 35
TC 3
Z9 3
U1 2
U2 11
PU AMER SCIENTIFIC PUBLISHERS
PI VALENCIA
PA 26650 THE OLD RD, STE 208, VALENCIA, CA 91381-0751 USA
SN 2156-7018
EI 2156-7026
J9 J MED IMAG HEALTH IN
JI J. Med. Imaging Health Inform.
PD MAR
PY 2019
VL 9
IS 3
BP 552
EP 560
DI 10.1166/jmihi.2019.2630
PG 9
WC Mathematical & Computational Biology; Radiology, Nuclear Medicine &
   Medical Imaging
WE Science Citation Index Expanded (SCI-EXPANDED)
SC Mathematical & Computational Biology; Radiology, Nuclear Medicine &
   Medical Imaging
GA HO4VW
UT WOS:000460922300022
DA 2022-07-22
ER

PT J
AU Lemme, NJ
   Li, NY
   DeFroda, SF
   Kleiner, J
   Owens, BD
AF Lemme, Nicholas J.
   Li, Neill Y.
   DeFroda, Steven F.
   Kleiner, Justin
   Owens, Brett D.
TI Epidemiology of Achilles Tendon Ruptures in the United States: Athletic
   and Nonathletic Injuries From 2012 to 2016
SO ORTHOPAEDIC JOURNAL OF SPORTS MEDICINE
LA English
DT Article
DE Achilles tendon; tendon rupture; epidemiology; database; NEISS;
   basketball
ID EMERGENCY-DEPARTMENTS; OUTCOMES; REPAIR; RISK; AGE
AB Background: Achilles tendon (AT) ruptures are one of the most common tendon ruptures, but there have been no studies investigating these injuries in the United States (US) using data representative of the entire US population. Purpose/Hypothesis: The purpose of this study was to determine the incidence and risk factors for AT ruptures in the US. We hypothesized that male sex, older age, and sport participation would increase the risk for AT ruptures. Study Design: Descriptive epidemiology study. Methods: All patients presenting to an emergency department with ruptured AT in the US from 2012 through 2016 were selected from the National Electronic Injury Surveillance System (NEISS) database. Incidence was calculated for sex, race, and age. AT ruptures were characterized based on the mechanism of injury, with subanalyses performed on sport-related AT ruptures to examine sex-, race-, and age-related differences. Results: From 2012 to 2016, a significant increase in the incidence of AT ruptures was observed, from 1.8 per 100,000 person-years in 2012 to 2.5 per 100,000 person-years in 2016 (P < .01), for an overall incidence of 2.1 per 100,000 person-years. The majority of AT ruptures occurred in male compared with female patients, with an incidence rate ratio of 3.5 (P < .01). The largest overall incidence of AT ruptures occurred in those aged 20-39 years for male patients (5.6/100,000 person-years) and in those aged 40-59 years for female patients (1.2/100,000 person-years). The largest rise in the incidence of AT ruptures during the study period was observed in patients aged 40-59 years (78% increase). The most common injury mechanism was participation in a sport or recreational activity (81.9% of all injuries), with basketball being the most common overall cause of AT ruptures. Conclusion: While AT ruptures in the US most commonly occur in young male patients (20-39 years old), the largest rise in the incidence was observed in middle-aged patients (40-59 years old), with participation in recreational sports being the most likely mechanism. Recognizing high-risk patients can help physicians counsel them and recommend strategies for injury prevention.
C1 [Lemme, Nicholas J.; Li, Neill Y.; DeFroda, Steven F.; Kleiner, Justin; Owens, Brett D.] Brown Univ, Warren Alpert Med Sch, Dept Orthopaed Surg, 593 Eddy St, Providence, RI 02903 USA.
RP Lemme, NJ (通讯作者)，Brown Univ, Warren Alpert Med Sch, Dept Orthopaed Surg, 593 Eddy St, Providence, RI 02903 USA.
EM nicholas_lemme@brown.edu
OI Kleiner, Justin/0000-0003-4788-1522; Lemme, Nicholas/0000-0002-3136-7177
CR Amin NH, 2013, AM J SPORT MED, V41, P1864, DOI 10.1177/0363546513490659
   Burry HC, 1973, RHEUMATOL REHABIL, V12, P177, DOI DOI 10.1093/RHEUMATOLOGY/12.4.177
   Clayton RAE, 2008, INJURY, V39, P1338, DOI 10.1016/j.injury.2008.06.021
   Davis JJ, 1999, MIL MED, V164, P872, DOI 10.1093/milmed/164.12.872
   DeFroda SF, 2016, CURR SPORT MED REP, V15, P426, DOI 10.1249/JSR.0000000000000310
   Diamond PT, 2001, BRAIN INJURY, V15, P537, DOI 10.1080/02699050010007362
   Erickson BJ, 2014, ORTHOP J SPORTS MED, V2, DOI 10.1177/2325967114549948
   Fukashiro S, 2002, ACTA PHYSIOL SCAND, V175, P183, DOI 10.1046/j.1365-201X.2002.00985.x
   Gil JA, 2017, CLIN J SPORT MED, V27, P499, DOI 10.1097/JSM.0000000000000395
   Hallal PC, 2012, LANCET, V380, P247, DOI 10.1016/S0140-6736(12)60646-1
   Hoffmann A, 2011, EUR RADIOL, V21, P1996, DOI 10.1007/s00330-011-2136-0
   Hojjat H, 2016, ANN OTO RHINOL LARYN, V125, P652, DOI 10.1177/0003489416644617
   Houshian S, 1998, INJURY, V29, P651, DOI 10.1016/S0020-1383(98)00147-8
   Hsu AR, 2015, FOOT ANKLE INT, V36, P1279, DOI 10.1177/1071100715589632
   Huttunen TT, 2014, AM J SPORT MED, V42, P2419, DOI 10.1177/0363546514540599
   INHOFE PD, 1995, AM J SPORT MED, V23, P227, DOI 10.1177/036354659502300217
   Jarvinen Tero A H, 2005, Foot Ankle Clin, V10, P255, DOI 10.1016/j.fcl.2005.01.013
   Krolo I, 2007, COLLEGIUM ANTROPOL, V31, P275
   Krul AJ, 2011, EUR J PUBLIC HEALTH, V21, P414, DOI 10.1093/eurpub/ckp228
   Kujala UM, 2005, CLIN J SPORT MED, V15, P133, DOI 10.1097/01.jsm.0000165347.55638.23
   Lantto I, 2015, SCAND J MED SCI SPOR, V25, pE133, DOI 10.1111/sms.12253
   Leppilahti J, 1996, ACTA ORTHOP SCAND, V67, P277, DOI 10.3109/17453679608994688
   Minhas SV, 2016, AM J SPORT MED, V44, P1056, DOI 10.1177/0363546515623028
   Myers RJ, 2010, WEST J EMERG MED, V11, P442
   Nelson NG, 2011, AM J SPORT MED, V39, P404, DOI 10.1177/0363546510383478
   NILLIUS SA, 1976, ACTA ORTHOP SCAND, V47, P118, DOI 10.3109/17453677608998983
   OBRIEN M, 1992, CLIN SPORT MED, V11, P505
   Ootes Daan, 2012, Hand (N Y), V7, P18, DOI 10.1007/s11552-011-9383-z
   Owens B, 2007, INT J SPORTS MED, V28, P617, DOI 10.1055/s-2007-964837
   Pappas E, 2007, J SPORT SCI MED, V6, P58
   Parekh Selene G, 2009, Foot Ankle Spec, V2, P283, DOI 10.1177/1938640009351138
   Raikin SM, 2013, FOOT ANKLE INT, V34, P475, DOI 10.1177/1071100713477621
   Schepsis AA, 2002, AM J SPORT MED, V30, P287, DOI 10.1177/03635465020300022501
   van der Linden PD, 2003, ARCH INTERN MED, V163, P1801, DOI 10.1001/archinte.163.15.1801
   Van Tassel D, 2014, INT J SPORTS MED, V35, P83, DOI 10.1055/s-0033-1345127
   Vasileff WK, 2014, J FOOT ANKLE SURG, V53, P485, DOI 10.1053/j.jfas.2014.02.016
   Wang Y, 2007, EPIDEMIOL REV, V29, P6, DOI 10.1093/epirev/mxm007
   Waterman BR, 2010, J BONE JOINT SURG AM, V92A, P2279, DOI 10.2106/JBJS.I.01537
   Weinfeld SB, 2014, MED CLIN N AM, V98, P331, DOI 10.1016/j.mcna.2013.11.005
   White DW, 2007, AM J SPORT MED, V35, P1308, DOI 10.1177/0363546507301256
   Zellers JA, 2016, BRIT J SPORT MED, V50, P1325, DOI 10.1136/bjsports-2016-096106
NR 41
TC 58
Z9 60
U1 2
U2 9
PU SAGE PUBLICATIONS INC
PI THOUSAND OAKS
PA 2455 TELLER RD, THOUSAND OAKS, CA 91320 USA
EI 2325-9671
J9 ORTHOP J SPORTS MED
JI Orthop. J. Sports Med.
PD NOV 26
PY 2018
VL 6
IS 11
AR 2325967118808238
DI 10.1177/2325967118808238
PG 7
WC Orthopedics; Sport Sciences
WE Science Citation Index Expanded (SCI-EXPANDED)
SC Orthopedics; Sport Sciences
GA HB7MD
UT WOS:000451261200001
PM 30505872
OA Green Published, gold
DA 2022-07-22
ER

PT J
AU Maffulli, N
   Tallon, C
   Wong, J
   Lim, KP
   Bleakney, R
AF Maffulli, N
   Tallon, C
   Wong, J
   Lim, KP
   Bleakney, R
TI No adverse effect of early weight bearing following open repair of acute
   tears of the Achilles tendon
SO JOURNAL OF SPORTS MEDICINE AND PHYSICAL FITNESS
LA English
DT Article
DE Achilles tendon; tendeon injuries, surgery; rehabilitation; treatment,
   outcome
ID CALF MUSCLE ATROPHY; SURGICAL REPAIR; RUPTURE; IMMOBILIZATION; MOTION;
   REHABILITATION; WEIGHTBEARING; ANKLE; RATS
AB Aim. To study the effects of early weight bearing following acute repair of ruptured Achilles tendon.
   Methods. Using a comparative longitudinal study design, following repair of an Achilles tendon rupture, patients in Group 1 were immobilised with their ankle in gravity equinus, were encouraged to weight bear on the operated limb as soon as possible to full weight bearing, and received a single a cast change at 2 weeks, when the ankle was immobilised in a plantigrade position. Patients in Group 2 were immobilised with their ankle in full equinus, and received a cast change at 2 weeks, when the ankle was immobilised in mid equinus, and at 4 weeks, when the ankle was immobilised in a plantigrade position. They were advised to weight bear 4 weeks from the operation.
   Results. Patients in Group 1 attended less outpatient visits, completely discarded their crutches at an average of 2.5 weeks from the operation (Group 2: average of 5.7 weeks from the operation), (p=0.013), and a greater proportion of them were satisfied with the results of surgery. At ultrasound scan, the average thickness of the repaired tendon was 12.1 mm (SD 2), with no difference in the thickness of the ruptured tendon regardless of the method of postoperative management. There was no significant difference in isometric strength between the two groups of patients.
   Conclusion. Early weight bearing with the ankle plantigrade is not detrimental to the outcome of repair following rupture of the Achilles tendon.
C1 Univ Keele, Sch Med, Dept Traumat & Orthopaed Surg, N Staffordshire Hosp, Stoke On Trent ST4 7QB, Staffs, England.
   Univ Aberdeen, Sch Med, Dept Orthopaed Surg, Aberdeen AB9 2ZD, Scotland.
   Aberdeen Royal Infirm, Dept Radiol, Aberdeen, Scotland.
RP Maffulli, N (通讯作者)，Univ Keele, Sch Med, Dept Traumat & Orthopaed Surg, N Staffordshire Hosp, Thornburrow Dr, Stoke On Trent ST4 7QB, Staffs, England.
EM n.maffulli@keele.ac.uk
RI Maffulli, Nicola/A-1839-2014; Wong, Jason/AAQ-9239-2020
OI Maffulli, Nicola/0000-0002-5327-3702; Wong, Jason/0000-0003-2592-3226
CR AKIZUKI KH, 2001, AM AC ORTH SURG ANN
   Aoki M, 1998, AM J SPORT MED, V26, P794, DOI 10.1177/03635465980260061001
   BOYDEN EM, 1995, CLIN ORTHOP RELAT R, V317, P150
   Boyer MI, 2001, J BONE JOINT SURG AM, V83A, P891, DOI 10.2106/00004623-200106000-00011
   Cook JL, 1997, BRIT J SPORT MED, V31, P332, DOI 10.1136/bjsm.31.4.332
   ENWEMEKA CS, 1988, AM J PHYS MED REHAB, V67, P264
   Farizon F, 1997, REV CHIR ORTHOP, V83, P65
   Frost HM, 2000, J BONE MINER METAB, V18, P305, DOI 10.1007/s007740070001
   FUGLMEYER AR, 1979, ACTA PHYSIOL SCAND, V107, P47, DOI 10.1111/j.1748-1716.1979.tb06441.x
   GELBERMAN RH, 1980, CLIN ORTHOP RELAT R, V153, P283
   HAGGMARK T, 1986, ORTHOPEDICS, V9, P160
   Haggmark T, 1979, Am J Sports Med, V7, P121, DOI 10.1177/036354657900700208
   JESSING P, 1975, ACTA CHIR SCAND, V141, P370
   Leppilahti J, 1998, SPORTS MED, V25, P79, DOI 10.2165/00007256-199825020-00002
   Leppilahti J, 1996, INT J SPORTS MED, V17, P619, DOI 10.1055/s-2007-972905
   Lo IKY, 1997, CLIN J SPORT MED, V7, P207, DOI 10.1097/00042752-199707000-00010
   Maffulli N, 1999, J BONE JOINT SURG AM, V81A, P1019, DOI 10.2106/00004623-199907000-00017
   Maffulli N, 1998, Bull Hosp Jt Dis, V57, P152
   Maffulli N, 1999, CLIN J SPORT MED, V9, P157, DOI 10.1097/00042752-199907000-00007
   MAFFULLI N, 1990, INT J SPORTS MED, V11, P78, DOI 10.1055/s-2007-1024767
   MANDELBAUM BR, 1995, AM J SPORT MED, V23, P392, DOI 10.1177/036354659502300403
   MAXWELL LC, 1992, ANAT REC, V234, P55, DOI 10.1002/ar.1092340107
   Mortensen NHM, 1999, J BONE JOINT SURG AM, V81A, P983, DOI 10.2106/00004623-199907000-00011
   Motta P, 1997, AM J SPORT MED, V25, P172, DOI 10.1177/036354659702500205
   Picaud A J, 1966, Ann Chir, V20, P1361
   Pintore E, 2001, J TRAUMA, V50, P71, DOI 10.1097/00005373-200101000-00013
   Rantanen J, 1999, SCAND J MED SCI SPOR, V9, P57
   RANTANEN J, 1993, ACTA ORTHOP SCAND, V64, P333, DOI 10.3109/17453679308993638
   SOLVEBORN SA, 1994, AM J SPORT MED, V22, P607, DOI 10.1177/036354659402200507
   Speck M, 1998, AM J SPORT MED, V26, P789, DOI 10.1177/03635465980260060901
   Sutherland A, 1998, Oper Orthop Traumatol, V10, P50, DOI 10.1007/s00064-006-0044-2
   TALLON C, 2000, THESIS U ABERDEEN
   WILLS CA, 1986, CLIN ORTHOP RELAT R, V207, P156
NR 33
TC 56
Z9 63
U1 0
U2 3
PU EDIZIONI MINERVA MEDICA
PI TURIN
PA CORSO BRAMANTE 83-85 INT JOURNALS DEPT., 10126 TURIN, ITALY
SN 0022-4707
EI 1827-1928
J9 J SPORT MED PHYS FIT
JI J. Sports Med. Phys. Fit.
PD SEP
PY 2003
VL 43
IS 3
BP 367
EP 379
PG 13
WC Sport Sciences
WE Science Citation Index Expanded (SCI-EXPANDED)
SC Sport Sciences
GA 755NR
UT WOS:000187416100016
PM 14625519
DA 2022-07-22
ER

PT J
AU Chan, KOW
   Tong, HHY
   Ng, GYF
AF Chan, Karly O. W.
   Tong, Henry H. Y.
   Ng, Gabriel Y. F.
TI TOPICAL FISH OIL APPLICATION COUPLING WITH THERAPEUTIC ULTRASOUND
   IMPROVES TENDON HEALING
SO ULTRASOUND IN MEDICINE AND BIOLOGY
LA English
DT Article
DE Fish oil; Omega-3; Achilles tendon rupture; Therapeutic ultrasound;
   Repair
ID POLYUNSATURATED-FATTY-ACIDS; ACHILLES-TENDON; MECHANICAL-PROPERTIES;
   RHEUMATOID-ARTHRITIS; COLLAGEN FORMATION; REPAIR; SKIN; RATS; DELIVERY;
   RUPTURE
AB Fish oil has been shown to promote collagen synthesis, and hence, connective tissue healing. Therapeutic ultrasound is commonly used to treat soft tissue injuries. This study aimed to investigate the therapeutic effect of topical fish oil on the management of Achilles tendon rupture, comparing normal therapeutic ultrasound with a combination of ultrasound and fish oil. Eighty-five Sprague-Dawley rats underwent surgical hemitenotomy of the right medial Achilles tendon. The rats received daily treatment of either topical placebo ointment (control group [CON]), topical fish oil (FO), therapeutic ultrasound (US) or ultrasound with fish oil as the coupling medium (FU). The treatment started on post-surgical day 2 over a 2-wk or 4-wk period. On days 15 and 29, the rats were sacrificed and their Achilles tendons were tested for structural stiffness, ultimate tensile strength (UTS) and energy absorption capacity. At 2 wk, only US showed higher normalized UTS compared with CON (p < 0.05). At 4 wk, both US and FU demonstrated better UTS (p < 0.05), while both FO and FU had improved in structural stiffness (p < 0.05). Four wk of treatment with ultrasound using fish oil as coupling medium showed improvement in both structural stiffness and UTS (p < 0.05). (E-mail: cckarly@hkcc-polyu.edu.hk) (C) 2016 World Federation for Ultrasound in Medicine & Biology.
C1 [Chan, Karly O. W.; Ng, Gabriel Y. F.] Hong Kong Polytech Univ, Dept Rehabil Sci, 11 Yuk Choi Rd, Kowloon, Hong Kong, Peoples R China.
   [Tong, Henry H. Y.] Macao Polytech Inst, Sch Hlth Sci, Macau, Macau, Peoples R China.
RP Chan, KOW (通讯作者)，Hong Kong Polytech Univ, Dept Rehabil Sci, 11 Yuk Choi Rd, Kowloon, Hong Kong, Peoples R China.
EM cckarly@hkcc-polyu.edu.hk
OI Tong, Henry/0000-0003-2687-741X
CR Abate M, 2009, ARTHRITIS RES THER, V11, DOI 10.1186/ar2723
   Ammar H, 2007, ASIAN J PHARM SCI, V2, P96
   Berbert AA, 2005, NUTRITION, V21, P131, DOI 10.1016/j.nut.2004.03.023
   Bhandari M, 2002, CLIN ORTHOP RELAT R, P190
   Calder PC, 2006, AM J CLIN NUTR, V83, p1505S, DOI 10.1093/ajcn/83.6.1505S
   Cardoso CRB, 2004, WOUND REPAIR REGEN, V12, P235
   Caturla N, 2011, J ALTERN COMPLEM MED, V17, P1051, DOI 10.1089/acm.2010.0410
   Cleland LG, 2006, ARTHRITIS RES THER, V8, DOI 10.1186/ar1876
   Cleland LG, 2003, DRUGS, V63, P845, DOI 10.2165/00003495-200363090-00001
   Contri RV, 2011, NANOCOSMETICS AND NANOMEDICINES: NEW APPROACHES FOR SKIN CARE, P3
   Curtis CL, 2002, P NUTR SOC, V61, P381, DOI 10.1079/PNS2002174
   Ding WG, 2013, J FOOT ANKLE SURG, V52, P143, DOI 10.1053/j.jfas.2012.12.006
   Doral MN, 2010, KNEE SURG SPORT TR A, V18, P638, DOI 10.1007/s00167-010-1083-7
   Doroski DM, 2007, BIOMATERIALS, V28, P187, DOI 10.1016/j.biomaterials.2006.08.040
   Forslund C, 2003, ACTA ORTHOP SCAND, V74, P465, DOI 10.1080/00016470310017802
   Goldberg RJ, 2007, PAIN, V129, P210, DOI 10.1016/j.pain.2007.01.020
   Hankenson KD, 2000, P SOC EXP BIOL MED, V223, P88, DOI 10.1046/j.1525-1373.2000.22312.x
   Heard CM, 2003, INT J PHARM, V261, P165, DOI 10.1016/S0378-5173(03)00297-7
   JACKSON BA, 1991, MED SCI SPORT EXER, V23, P171
   Jia Y, 2004, EXP BIOL MED, V229, P676, DOI 10.1177/153537020422900712
   Jia Y, 2005, J NUTR BIOCHEM, V16, P500, DOI 10.1016/j.jnutbio.2005.01.016
   KANNUS P, 1991, J BONE JOINT SURG AM, V73A, P1507, DOI 10.2106/00004623-199173100-00009
   Khan RJK, 2005, J BONE JOINT SURG AM, V87A, P2202, DOI 10.2106/JBJS.D.03049
   Machet L, 2002, INT J PHARMACEUT, V243, P1, DOI 10.1016/S0378-5173(02)00299-5
   Maquirriain Javier, 2011, Yale Journal of Biology and Medicine, V84, P289
   Mavrogenis S, 2004, PHYS THER SPORT, V5, P194, DOI 10.1016/j.ptsp.2004.06.002
   Meidan Victor M, 2004, Am J Ther, V11, P312, DOI 10.1097/01.mjt.0000101826.94820.6b
   Mitragotri S, 2004, ADV DRUG DELIVER REV, V56, P589, DOI 10.1016/j.addr.2003.10.024
   Nandra RS, 2012, TRAUMA, V14, P67, DOI 10.1177/1460408611415909
   Ng COY, 2003, ULTRASOUND MED BIOL, V29, P1501, DOI 10.1016/S0301-5629(03)01018-4
   Ng GYF, 2008, PHOTOMED LASER SURG, V26, P137, DOI 10.1089/pho.2007.2145
   Ng GYF, 2007, ULTRASOUND MED BIOL, V33, P1750, DOI 10.1016/j.ultrasmedbio.2007.05.019
   Ng GYF, 2008, ULTRASOUND MED BIOL, V34, P1919, DOI 10.1016/j.ultrasmedbio.2008.04.017
   Ng GYF, 2004, ULTRASOUND MED BIOL, V30, P1539, DOI 10.1016/j.ultrasmedbio.2004.08.030
   Nino Massimiliano, 2010, Dermatol Online J, V16, P4
   Olsson N, 2011, KNEE SURG SPORT TR A, V19, P1385, DOI 10.1007/s00167-011-1511-3
   Otranto M, 2010, WOUND REPAIR REGEN, V18, P629, DOI 10.1111/j.1524-475X.2010.00617.x
   PARRY DAD, 1988, BIOPHYS CHEM, V29, P195, DOI 10.1016/0301-4622(88)87039-X
   Polat BE, 2011, J CONTROL RELEASE, V152, P330, DOI 10.1016/j.jconrel.2011.01.006
   Prilutsky BI, 1996, J BIOMECH, V29, P417, DOI 10.1016/0021-9290(95)00085-2
   Rees JD, 2006, RHEUMATOLOGY, V45, P508, DOI 10.1093/rheumatology/kel046
   Roeder BA, 2002, J BIOMECH ENG-T ASME, V124, P214, DOI 10.1115/1.1449904
   Russell LM, 2009, EXPERT OPIN DRUG DEL, V6, P355, DOI [10.1517/17425240902865561 , 10.1517/17425240902865561]
   Shingel KI, 2008, J TISSUE ENG REGEN M, V2, P383, DOI 10.1002/term.101
   Tallon C, 2001, MED SCI SPORT EXER, V33, P1983, DOI 10.1097/00005768-200112000-00002
   Tanojo H, 1997, PHARMACEUT RES, V14, P42, DOI 10.1023/A:1012099216060
   van Ravenzwaay B, 2004, HUM EXP TOXICOL, V23, P421, DOI 10.1191/0960327104ht471oa
   Virchenko O, 2004, AM J SPORT MED, V32, P1743, DOI 10.1177/0363546504263403
   Weber M, 2003, AM J SPORT MED, V31, P685, DOI 10.1177/03635465030310050901
   Yeung CK, 2006, J ORTHOP RES, V24, P193, DOI 10.1002/jor.20020
   Zhai H, 2002, SKIN RES TECHNOL, V8, P1, DOI 10.1046/j.0909-752x.2001.10311.x
   Zulfakar MH, 2010, INFLAMM RES, V59, P23, DOI 10.1007/s00011-009-0065-z
NR 52
TC 3
Z9 3
U1 1
U2 7
PU ELSEVIER SCIENCE INC
PI NEW YORK
PA 360 PARK AVE SOUTH, NEW YORK, NY 10010-1710 USA
SN 0301-5629
EI 1879-291X
J9 ULTRASOUND MED BIOL
JI Ultrasound Med. Biol.
PD DEC
PY 2016
VL 42
IS 12
BP 2983
EP 2989
DI 10.1016/j.ultrasmedbio.2016.08.018
PG 7
WC Acoustics; Radiology, Nuclear Medicine & Medical Imaging
WE Science Citation Index Expanded (SCI-EXPANDED)
SC Acoustics; Radiology, Nuclear Medicine & Medical Imaging
GA EE4CK
UT WOS:000389549300027
PM 27692307
DA 2022-07-22
ER

PT J
AU Brorsson, A
   Olsson, N
   Nilsson-Helander, K
   Karlsson, J
   Eriksson, BI
   Silbernagel, KG
AF Brorsson, A.
   Olsson, N.
   Nilsson-Helander, K.
   Karlsson, J.
   Eriksson, B. I.
   Silbernagel, K. G.
TI Recovery of calf muscle endurance 3 months after an Achilles tendon
   rupture
SO SCANDINAVIAN JOURNAL OF MEDICINE & SCIENCE IN SPORTS
LA English
DT Article
DE Achilles tendon Total Rupture Score (ATRS); clinical test; early
   exercise; functional performance
ID NONOPERATIVE TREATMENT; OUTCOME MEASURES; ANKLE-JOINT; REPAIR;
   REHABILITATION; RELIABILITY; ELONGATION; POSITIONS; RERUPTURE; DEFICITS
AB The purpose of this study was to evaluate calf muscle endurance in a seated position 3 months after an Achilles tendon rupture and to evaluate how the ability to perform standardized seated heel-rises correlated to the single-leg standing heel-rise test and to patient-reported symptoms evaluated with the Achilles tendon Total Rupture Score (ATRS) 3 and 6 months after the injury. Ninety-three patients were included from a cohort of 101 patients participating in a prospective, randomized controlled trial comparing surgical and nonsurgical treatment after Achilles tendon rupture. Forty-seven patients were treated surgically and 46 nonsurgically. Ninety-one patients out of 93 (98%) could perform the standardized seated heel-rises. At the 3-month follow-up, there was a significant difference (P<0.001) between the injured and the healthy side performing standardized seated heel-rises. There were also significant correlations (r=0.29-0.37, P=<0.05) between the standardized seated heel-rises and ATRS 3 and 6 months after injury in the group who could not perform single-leg standing heel-rises. There were no significant differences between the surgical and nonsurgical treatment groups. The evaluation of standardized seated heel-rises appears to be a useful tool to quantify progress and predict future functional performance and patient-reported symptoms.
C1 [Brorsson, A.; Olsson, N.; Nilsson-Helander, K.; Karlsson, J.; Eriksson, B. I.; Silbernagel, K. G.] Univ Gothenburg, Sahlgrenska Acad, Inst Clin Sci, Dept Orthopaed, Gothenburg, Sweden.
   [Brorsson, A.] IFK Kliniken Rehab, Gothenburg, Sweden.
   [Olsson, N.] Orthoctr IFK Kliniken, Gothenburg, Sweden.
   [Olsson, N.; Karlsson, J.; Eriksson, B. I.] Sahlgrens Univ Hosp, Molndal, Sweden.
   [Nilsson-Helander, K.] Hallands Sjukhus, Kungsbacka, Sweden.
   [Silbernagel, K. G.] Univ Delaware, Dept Phys Therapy, Newark, DE USA.
RP Brorsson, A (通讯作者)，Sahlgrenska Univ Sahlgrenska, Lundberg Lab Orthopaed Res, Grona Straket 12, S-41345 Gothenburg, Sweden.
EM annelie.brorsson@orthop.gu.se
RI Brorsson, Annelie/AAD-5986-2020; Helander, Katarina
   Nilsson/AAE-1793-2020
OI Helander, Katarina Nilsson/0000-0002-1292-5102; Gravare Silbernagel,
   Karin/0000-0001-7566-407X
FU Swedish National Centre for Research in Sports (CIF)
FX The authors thank Colleen O'Hara, Kristen Lutter, Alex Skacel och
   Jessica Zarco for their help in the evaluation of the healthy subjects.
   The Swedish National Centre for Research in Sports (CIF) supported this
   study.
CR Arampatzis A, 2006, J BIOMECH, V39, P1891, DOI 10.1016/j.jbiomech.2005.05.010
   Bostick GP, 2010, J ORTHOP SPORT PHYS, V40, P345, DOI 10.2519/jospt.2010.3204
   Carlsson U, 2001, CLIN PHYSIOL, V21, P1, DOI 10.1046/j.1365-2281.2001.00304.x
   Fleiss JL, 2011, J ROYAL STAT SOC SER, P73
   GRIMBY G, 1986, ACTA MED SCAND, P233
   Hebert-Losier K, 2013, J ELECTROMYOGR KINES, V23, P916, DOI 10.1016/j.jelekin.2013.03.012
   Hebert-Losier K, 2011, J ELECTROMYOGR KINES, V21, P819, DOI 10.1016/j.jelekin.2011.04.009
   Hebert-Losier K, 2009, PHYS THER SPORT, V10, P142, DOI 10.1016/j.ptsp.2009.07.001
   Holm C, 2015, SCAND J MED SCI SPOR, V25, pE1, DOI 10.1111/sms.12209
   Kangas J, 2007, AM J SPORT MED, V35, P59, DOI 10.1177/0363546506293255
   Kearney RS, 2012, BRIT J SPORT MED, V46, P1102, DOI 10.1136/bjsports-2011-090497
   Keating JF, 2011, J BONE JOINT SURG BR, V93B, P1071, DOI 10.1302/0301-620X.93B8.25998
   Moller M, 2005, KNEE SURG SPORT TR A, V13, P60, DOI 10.1007/s00167-003-0441-0
   Nilsson-Helander K, 2007, AM J SPORT MED, V35, P421, DOI 10.1177/0363546506294856
   Nilsson-Helander K, 2010, AM J SPORT MED, V38, P2186, DOI 10.1177/0363546510376052
   Olsson N, 2014, SCAND J MED SCI SPOR, V24, P152, DOI 10.1111/j.1600-0838.2012.01497.x
   Olsson N, 2013, AM J SPORT MED, V41, P2867, DOI 10.1177/0363546513503282
   Olsson N, 2011, KNEE SURG SPORT TR A, V19, P1385, DOI 10.1007/s00167-011-1511-3
   Pajala A, 2002, J BONE JOINT SURG AM, V84A, P2016, DOI 10.2106/00004623-200211000-00017
   Rettig AC, 2005, AM J SPORT MED, V33, P119, DOI 10.1177/0363546504268720
   Silbernagel KG, 2012, AM J SPORT MED, V40, P1564, DOI 10.1177/0363546512447926
   Silbernagel KG, 2006, KNEE SURG SPORT TR A, V14, P1207, DOI 10.1007/s00167-006-0150-6
   Silbernagel KG, 2010, KNEE SURG SPORT TR A, V18, P258, DOI 10.1007/s00167-009-0889-7
   Silbernagel KG, 2001, SCAND J MED SCI SPOR, V11, P197
   Uchiyama E, 2007, AM J SPORT MED, V35, P1739, DOI 10.1177/0363546507304492
   Wakahara T, 2007, J BIOMECH, V40, P2291, DOI 10.1016/j.jbiomech.2006.10.006
   Willits K, 2010, J BONE JOINT SURG AM, V92A, P2767, DOI 10.2106/JBJS.I.01401
NR 27
TC 18
Z9 18
U1 2
U2 12
PU WILEY
PI HOBOKEN
PA 111 RIVER ST, HOBOKEN 07030-5774, NJ USA
SN 0905-7188
EI 1600-0838
J9 SCAND J MED SCI SPOR
JI Scand. J. Med. Sci. Sports
PD JUL
PY 2016
VL 26
IS 7
BP 844
EP 853
DI 10.1111/sms.12533
PG 10
WC Sport Sciences
WE Science Citation Index Expanded (SCI-EXPANDED)
SC Sport Sciences
GA DR2UD
UT WOS:000379758500016
PM 26283647
DA 2022-07-22
ER

PT J
AU Maffulli, N
   Via, AG
   Oliva, F
AF Maffulli, Nicola
   Via, Alessio Giai
   Oliva, Francesco
TI Achilles Injuries in the Athlete: Noninsertional
SO OPERATIVE TECHNIQUES IN SPORTS MEDICINE
LA English
DT Article
DE Achilles tendon; tendinopathy; rupture; repair; minimally invasive
   surgery
ID IMAGE-GUIDED INJECTIONS; TENDON RUPTURE; CLINICAL-DIAGNOSIS; MAIN BODY;
   TENDINOPATHY; MANAGEMENT; DIAMETER; ESTROGEN; ETIOLOGY; THERAPY
AB Achilles tendinopathy is common in both athletic and nonathletic individuals, and the incidence has risen in the last few decades. Although Achilles tendinopathy has been extensively studied, there is a clear lack of properly conducted scientific research to clarify its cause, pathology, natural history, and optimal management. The treatment of Achilles tendinopathy lacks evidence-based support, and individuals having tendinopathy are at risk of long-term morbidity with unpredictable clinical outcome. Most patients respond to conservative treatments. When conservative management is unsuccessful, surgery is recommended. Similar results have been reported with both. open and minimally invasive techniques. However, minimally invasive techniques appear to reduce the risks of infection and provide better cosmetic results. An Achilles tendon rupture is frequent in young athletes and middle-aged people who practice recreational activities, and it is a serious injury. The management should take into account the age, occupation, and level of sporting activity. Open surgery provides good functional results and a lower rerupture rate, but it is frequently associated with a higher risk of superficial skin breakdown and wound problems. Percutaneous repair aims to provide good functional outcome while decreasing the problems associated with open surgery for wound healing and skin breakdown. Percutaneous repair followed by early functional rehabilitation is becoming increasingly common and should be considered in selected patients. (C) 2014 Elsevier Inc. All rights reserved.
C1 [Maffulli, Nicola] Univ Salerno, Sch Med & Surg, Dept Musculoskeletal Disorders, I-84100 Salerno, Italy.
   [Maffulli, Nicola] Mary Univ London, Mile End Hosp, Ctr Sports & Exercise Med, Bans & London Sch Med & Dent, London E1 4DG, England.
   [Via, Alessio Giai; Oliva, Francesco] Univ Roma Tor Vergata, Sch Med, Dept Orthopaed & Traumatol, Rome, Italy.
RP Maffulli, N (通讯作者)，Mary Univ London, Mile End Hosp, Ctr Sports & Exercise Med, Bans & London Sch Med & Dent, 275 Bancroft Rd, London E1 4DG, England.
EM n.maffulli@qmul.ac.uk
RI Maffulli, Nicola/A-1839-2014; Oliva, Francesco/AAC-2030-2019
OI Maffulli, Nicola/0000-0002-5327-3702; Oliva,
   Francesco/0000-0002-7473-0271; GIAI VIA, Alessio Davide
   Enrico/0000-0002-5943-081X
CR Abate Michele, 2012, Muscles Ligaments Tendons J, V2, P44
   Ahmed N, 2005, DIABETES RES CLIN PR, V67, P3, DOI 10.1016/j.diabres.2004.09.004
   Alfredson H, 1998, AM J SPORT MED, V26, P360, DOI 10.1177/03635465980260030301
   Alfredson H, 2007, BRIT J SPORT MED, V41, P211, DOI 10.1136/bjsm.2007.035543
   Astrom M, 1998, ACTA ORTHOP SCAND, V69, P404, DOI 10.3109/17453679808999056
   ASTROM M, 1992, ACTA ORTHOP SCAND, V63, P631
   Biancalana A, 2010, CONNECT TISSUE RES, V51, P171, DOI 10.3109/03008200903191312
   Bryant AL, 2008, J APPL PHYSIOL, V105, P1035, DOI 10.1152/japplphysiol.01281.2007
   Chan O, 2008, DISABIL REHABIL, V30, P1697, DOI 10.1080/09638280701788225
   Circi E, 2009, INT ORTHOP, V33, P1461, DOI 10.1007/s00264-009-0778-1
   Cook JL, 2007, SCAND J MED SCI SPOR, V17, P128
   De Felipe C, 1998, NATURE, V392, P394, DOI 10.1038/32904
   de Vos RJ, 2007, BRIT J SPORT MED, V41, DOI 10.1136/bjsm.2006.032532
   Dean BJF, 2013, CLIN ORTHOP RELAT R, V471, P3036, DOI 10.1007/s11999-013-3010-y
   Del Buono A, 2014, BRIT MED BULL, V109, P45, DOI 10.1093/bmb/ldt029
   Frairia R, 2012, MUSCLES LIGAMENTS TE, V1, P137
   Gaida JE, 2008, DISABIL REHABIL, V30, P1555, DOI 10.1080/09638280701786864
   Gautieri A, 2014, MATRIX BIOL, V34, P89, DOI 10.1016/j.matbio.2013.09.004
   Guillo S, 2013, SURG-J R COLL SURG E, V11, P14, DOI 10.1016/j.surge.2011.12.002
   HATTRUP SJ, 1985, FOOT ANKLE, V6, P34, DOI 10.1177/107110078500600107
   Humphrey J, 2010, J SCI MED SPORT, V13, P295, DOI 10.1016/j.jsams.2009.09.007
   Khan KM, 2000, SPORTS MED ARTHROSC, V8, P17, DOI 10.1097/00132585-200008010-00003
   Li YF, 2013, MATRIX BIOL, V32, P169, DOI 10.1016/j.matbio.2013.01.003
   Lohrer H, 2002, SPORTVERLETZ SPORTSC, V16, P108, DOI 10.1055/s-2002-34752
   Longo UG, 2013, FOOT ANKLE CLIN, V18, P319, DOI 10.1016/j.fcl.2013.02.009
   Longo UG, 2008, DISABIL REHABIL, V30, P1709, DOI 10.1080/09638280701786922
   Loppini M., 2011, MUSCLES LIGAMENTS TE, V1, P133
   Maffulli N, 1999, J BONE JOINT SURG AM, V81A, P1019, DOI 10.2106/00004623-199907000-00017
   Maffulli N, 2004, J ROY SOC MED, V97, P472, DOI 10.1258/jrsm.97.10.472
   Maffulli N, 2003, CLIN J SPORT MED, V13, P11, DOI 10.1097/00042752-200301000-00003
   Maffulli N, 1998, AM J SPORT MED, V26, P266, DOI 10.1177/03635465980260021801
   Maffulli N, 1999, CLIN J SPORT MED, V9, P157, DOI 10.1097/00042752-199907000-00007
   Maffulli N, 1999, AM J SPORT MED, V27, P747, DOI 10.1177/03635465990270061101
   Maffulli N, 2013, PHYS THER SPORT, V14, P163, DOI 10.1016/j.ptsp.2012.07.002
   Maffulli N, 2011, FOOT ANKLE INT, V32, P9, DOI 10.3113/FAI.2011.0009
   Maffulli N, 2008, DISABIL REHABIL, V30, P1677, DOI 10.1080/09638280701786427
   Magra M, 2005, BRIT J SPORT MED, V39, P789, DOI 10.1136/bjsm.2005.017855
   McCrory JL, 1999, MED SCI SPORT EXER, V31, P1374, DOI 10.1097/00005768-199910000-00003
   Nigg BM, 2001, CLIN J SPORT MED, V11, P2, DOI 10.1097/00042752-200101000-00002
   Peers K., 2003, EXTRACORPOREAL SHOCK, P61
   Rees JD, 2009, AM J SPORT MED, V37, P1855, DOI 10.1177/0363546508324283
   Rolf C, 1997, FOOT ANKLE INT, V18, P565, DOI 10.1177/107110079701800906
   Rompe JD, 2007, AM J SPORT MED, V35, P374, DOI 10.1177/0363546506295940
   Rompe JD, 2008, DISABIL REHABIL, V30, P1666, DOI 10.1080/09638280701785825
   Ronga M, 2009, ACHILLES TENDON, P39
   Roos EM, 2004, SCAND J MED SCI SPOR, V14, P286, DOI 10.1111/j.1600-0838.2004.378.x
   Sayana MK, 2007, J SCI MED SPORT, V10, P52, DOI 10.1016/j.jsams.2006.05.008
   Schubert TEO, 2005, ANN RHEUM DIS, V64, P1083, DOI 10.1136/ard.2004.029876
   SIMMONDS F A, 1957, Practitioner, V179, P56
   STANISH WD, 1986, CLIN ORTHOP RELAT R, P65
   Tallon C, 2001, AM J SPORT MED, V29, P315, DOI 10.1177/03635465010290031101
   Testa V, 2002, MED SCI SPORT EXER, V34, P573, DOI 10.1097/00005768-200204000-00002
   Torricelli P, 2013, AGE, V35, P2125, DOI 10.1007/s11357-012-9500-0
   Tsai WC, 2007, CONNECT TISSUE RES, V48, P46, DOI 10.1080/03008200601071295
   Via Alessio Giai, 2013, Muscles Ligaments Tendons J, V3, P70, DOI 10.11138/mltj/2013.3.2.070
   Waggett AD, 1998, MATRIX BIOL, V16, P457, DOI 10.1016/S0945-053X(98)90017-8
   Warrender WJ, 2011, J SHOULDER ELB SURG, V20, P961, DOI 10.1016/j.jse.2010.11.006
   Wearing SC, 2006, OBES REV, V7, P13, DOI 10.1111/j.1467-789X.2006.00215.x
   Wong J, 2002, AM J SPORT MED, V30, P565, DOI 10.1177/03635465020300041701
   Zanetti M, 2003, RADIOLOGY, V227, P556, DOI 10.1148/radiol.2272012069
NR 60
TC 3
Z9 3
U1 0
U2 11
PU W B SAUNDERS CO-ELSEVIER INC
PI PHILADELPHIA
PA 1600 JOHN F KENNEDY BOULEVARD, STE 1800, PHILADELPHIA, PA 19103-2899 USA
SN 1060-1872
EI 1557-9794
J9 OPER TECHN SPORT MED
JI Oper. Tech. Sports Med.
PD DEC
PY 2014
VL 22
IS 4
BP 321
EP 330
DI 10.1053/j.otsm.2014.09.001
PG 10
WC Sport Sciences; Surgery
WE Science Citation Index Expanded (SCI-EXPANDED)
SC Sport Sciences; Surgery
GA AW7QK
UT WOS:000346459500008
DA 2022-07-22
ER

PT J
AU Pollock, N
   Dijkstra, P
   Calder, J
   Chakraverty, R
AF Pollock, Noel
   Dijkstra, Paul
   Calder, James
   Chakraverty, Robin
TI Plantaris injuries in elite UK track and field athletes over a 4-year
   period: a retrospective cohort study
SO KNEE SURGERY SPORTS TRAUMATOLOGY ARTHROSCOPY
LA English
DT Article
DE Plantaris; Athletics; Injury; Achilles; Tendon
ID PORTION ACHILLES TENDINOPATHY; POTENTIAL ROLE; TENDON; MUSCLE;
   TENDINOSIS; RUPTURE; PAIN
AB The plantaris tendon is present in 98-100 % of people, and a potential relationship between the plantaris tendon and the development of Achilles tendinopathy has been postulated. There are no studies on the epidemiology of plantaris injuries in a sporting population. This retrospective cohort study presents the incidence, nature and outcome of plantaris injuries in elite British track and field athletes.
   All 214 elite athletes supported by the British Athletics Medical team from 2009 to 2013 were included in this cohort study. An injury was recorded if a plantaris injury was diagnosed and confirmed with imaging or surgical findings. Patient demographics, injury details and return to competitive elite track and field were recorded.
   There were 33 new plantaris injuries, with an annual plantaris injury incidence of 3.9-9.3 %. There were significantly more right-sided plantaris injuries in bend running sprinters (15 right-sided vs. 4 left-sided). 74 % of the athletes who had a plantaris injury also had Achilles tendinopathy at some point during the study period. Seventeen plantaris tendons were surgically removed from 13 athletes during the course of the study with 12 of the 13 athletes returning to the same level on the Tegner activity scale.
   This retrospective cohort study demonstrates that plantaris injuries are common in elite track and field athletes and may be underreported in the literature. There may be an association between the biomechanics bend sprinting and plantaris injury. Elite athletes may benefit from appropriate preventative and management strategies implemented by coaching and medical teams.
   IV.
C1 [Pollock, Noel] Hosp St John & St Elizabeth, British Athlet Team Doctor, Level 1 Outpatients,0 Grove End Rd, London NW8 9NH, England.
   [Dijkstra, Paul] Qatar Orthopaed & Sports Med Hosp, Sports Med Dept, Aspetar, POB 29222, Doha, Qatar.
   [Calder, James] Chelsea & Westminster Hosp NHS Fdn, London, England.
   [Chakraverty, Robin] Univ Loughborough, British Athlet Inst, Loughborough, Leics, England.
RP Pollock, N (通讯作者)，Hosp St John & St Elizabeth, British Athlet Team Doctor, Level 1 Outpatients,0 Grove End Rd, London NW8 9NH, England.
EM npollock@uka.org.uk
OI Dijkstra, H Paul/0000-0003-3166-1357
CR Alfredson H, 2011, BRIT J SPORT MED, V45, P1023, DOI 10.1136/bjsports-2011-090217
   Alfredson H, 2011, BRIT J SPORT MED, V45, P407, DOI 10.1136/bjsm.2010.081216
   Andersson G, 2007, KNEE SURG SPORT TR A, V15, P1272, DOI 10.1007/s00167-007-0364-2
   Bianchi S, 2011, SKELETAL RADIOL, V40, P891, DOI 10.1007/s00256-010-1076-0
   Chang YH, 2007, J EXP BIOL, V210, P971, DOI 10.1242/jeb.02728
   Cook JL, 2012, BRIT J SPORT MED, V46, P163, DOI 10.1136/bjsports-2011-090414
   Delgado GJ, 2002, RADIOLOGY, V224, P112, DOI 10.1148/radiol.2241011067
   Dijkstra HP, 2014, BRIT J SPORT MED, V48, P523, DOI 10.1136/bjsports-2013-093222
   Freeman AJ, 2008, CLIN ANAT, V21, P178, DOI 10.1002/ca.20594
   Gopinath TN, 2012, J CLIN IMAG SCI, V2, DOI 10.4103/2156-7514.95433
   Hamilton W, 1997, FOOT ANKLE INT, V18, P522, DOI 10.1177/107110079701800813
   Harmon KJ, 2006, CLIN J SPORT MED, V16, P361, DOI 10.1097/00042752-200607000-00015
   HARVEY FJ, 1983, J HAND SURG-AM, V8, P243, DOI 10.1016/S0363-5023(83)80151-8
   HELMS CA, 1995, RADIOLOGY, V195, P201, DOI 10.1148/radiology.195.1.7892469
   Humphrey J, 2010, J SCI MED SPORT, V13, P295, DOI 10.1016/j.jsams.2009.09.007
   Lintz F, 2011, Foot Ankle Surg, V17, P252, DOI 10.1016/j.fas.2010.08.004
   Miller W A, 1977, Am J Sports Med, V5, P191, DOI 10.1177/036354657700500505
   Nayak SR, 2010, CLIN TER, V161, P249
   Pearce Christopher J, 2012, Foot Ankle Surg, V18, P124, DOI 10.1016/j.fas.2011.04.008
   Rio E, 2014, SPORTS MED, V44, P9, DOI 10.1007/s40279-013-0096-z
   Saxena A, 2000, FOOT ANKLE INT, V21, P570, DOI 10.1177/107110070002100707
   Schubert TEO, 2005, ANN RHEUM DIS, V64, P1083, DOI 10.1136/ard.2004.029876
   SEVERANCE HW, 1982, J BONE JOINT SURG AM, V64, P1387, DOI 10.2106/00004623-198264090-00021
   SIMPSON SL, 1991, J HAND SURG-AM, V16A, P708
   Steenstra Ferry, 2006, Foot Ankle Clin, V11, P429, DOI 10.1016/j.fcl.2006.02.001
   Taunton JE, 2002, BRIT J SPORT MED, V36, P95, DOI 10.1136/bjsm.36.2.95
   TEGNER Y, 1985, CLIN ORTHOP RELAT R, P43
   van Sterkenburg MN, 2011, KNEE SURG SPORT TR A, V19, P1362, DOI 10.1007/s00167-011-1514-0
   van Sterkenburg MN, 2011, J ANAT, V218, P336, DOI 10.1111/j.1469-7580.2011.01335.x
NR 29
TC 10
Z9 10
U1 4
U2 16
PU SPRINGER
PI NEW YORK
PA 233 SPRING ST, NEW YORK, NY 10013 USA
SN 0942-2056
EI 1433-7347
J9 KNEE SURG SPORT TR A
JI Knee Surg. Sports Traumatol. Arthrosc.
PD JUL
PY 2016
VL 24
IS 7
BP 2287
EP 2292
DI 10.1007/s00167-014-3409-3
PG 6
WC Orthopedics; Sport Sciences; Surgery
WE Science Citation Index Expanded (SCI-EXPANDED)
SC Orthopedics; Sport Sciences; Surgery
GA DQ4HO
UT WOS:000379165000031
PM 25362249
DA 2022-07-22
ER

PT J
AU Doral, MN
   Bozkurt, M
   Turhan, E
   Ayvaz, M
   Atay, OA
   Uzumcugil, A
   Leblebicioglu, G
   Kaya, D
   Aydog, T
AF Doral, Mahmut Nedim
   Bozkurt, Murat
   Turhan, Egemen
   Ayvaz, Mehmet
   Atay, Oezguer Ahmet
   Uzumcugil, Akin
   Leblebicioglu, Guersel
   Kaya, Defne
   Aydog, Tolga
TI Percutaneous suturing of the ruptured Achilles tendon with endoscopic
   control
SO ARCHIVES OF ORTHOPAEDIC AND TRAUMA SURGERY
LA English
DT Article
DE Achilles tendon rupture; Surgical procedure; Percutaneous repair;
   Endoscopy control
ID NONOPERATIVE TREATMENT; OPERATIVE TREATMENT; OPEN REPAIR; NONSURGICAL
   TREATMENT; SURGICAL REPAIR; MANAGEMENT; ANKLE; IMMOBILIZATION;
   METAANALYSIS; MOTION
AB A prospective study of modified percutaneous Achilles tendon repair performed between 1999 and 2005 under local infiltration anesthesia is presented; the study evaluated the results of percutaneous repair technique by visualization of the synovia under endoscopic control, followed by early functional postoperative treatment for surgical intervention of acute Achilles tendon ruptures.
   Sixty-two patients (58 males, 4 females, mean age 32) were treated by percutaneous suturing with modified Bunnel technique under endoscopic control within 10 days after acute total rupture. Physiotherapy was initiated immediately after the operation and patients were encouraged to weight-bearing ambulation with a walking brace-moon boot as tolerated. Full weight-bearing was allowed minimum after 3 weeks postoperatively without brace.
   The procedure was tolerated in all patients. There were no significant ROM limitation was observed. Two patients experienced transient hypoesthesia in the region of sural nerve that spontaneously resolved in 6 months. Fifty-nine patients (95%) including professional athletes returned to their previous sportive activities, while 18 of them (29%) had some minor complaints. The interval from injury to return to regular work and rehabilitation training was 11.7 weeks (10-13 weeks). At the latest follow-up (mean: 46 months; range: 12-78 months), all the patients had satisfactory results with a mean American Orthopedic Foot and Ankle Society's ankle-hindfoot score of 94.6. No re-ruptures, deep venous thrombosis or wound problems occurred.
   The proposed method offers a reasonable treatment option for acute total Achilles tendon rupture with a low number of complications. The rerupture rate and return to preinjury activities are comparable to open and percutaneous without endoscopic control procedures.
C1 [Doral, Mahmut Nedim] Hacettepe Univ, Fac Med, Dept Orthopaed & Sports Med, TR-06100 Ankara, Turkey.
   [Bozkurt, Murat] Yildirim Beyazit Training & Res Hosp, Dept Orthopaed, Ankara, Turkey.
   [Turhan, Egemen] Zonguldak Karaelmas Univ, Dept Orthopaed, Fac Med, Zonguldak, Turkey.
   [Ayvaz, Mehmet; Atay, Oezguer Ahmet; Uzumcugil, Akin; Leblebicioglu, Guersel] Hacettepe Univ, Fac Med, Dept Orthopaed, TR-06100 Ankara, Turkey.
   [Kaya, Defne] Hacettepe Univ, Fac Phys Rehabil, TR-06100 Ankara, Turkey.
   [Kaya, Defne; Aydog, Tolga] Hacettepe Univ, Dept Sports Med, Fac Med, TR-06100 Ankara, Turkey.
RP Doral, MN (通讯作者)，Hacettepe Univ, Fac Med, Dept Orthopaed & Sports Med, TR-06100 Ankara, Turkey.
EM ndoral@hacettepe.edu.tr
RI Kaya, Defne/AAE-8707-2019; turhan, egemen/AAB-7310-2020
OI Kaya Utlu, Defne/0000-0003-0552-1190
CR Aoki M, 1998, AM J SPORT MED, V26, P794, DOI 10.1177/03635465980260061001
   Assal M, 2002, J BONE JOINT SURG AM, V84A, P161, DOI 10.2106/00004623-200202000-00001
   Bhandari M, 2002, CLIN ORTHOP RELAT R, P190
   Blankstein A, 2007, ISR MED ASSOC J, V9, P83
   BRADLEY JP, 1990, AM J SPORT MED, V18, P188, DOI 10.1177/036354659001800213
   Buchgraber A, 1997, CLIN ORTHOP RELAT R, P113
   CARDEN DG, 1987, J BONE JOINT SURG BR, V69, P416, DOI 10.1302/0301-620X.69B3.3294839
   Carmont MR, 2008, KNEE SURG SPORT TR A, V16, P199, DOI 10.1007/s00167-007-0411-z
   Ceccarelli F, 2007, CLIN ORTHOP RELAT R, P188, DOI 10.1097/BLO.0b013e3180396f07
   CETTI R, 1994, CLIN ORTHOP RELAT R, P155
   CETTI R, 1993, AM J SPORT MED, V21, P791, DOI 10.1177/036354659302100606
   CHILLEMI C, 2002, FOOT ANKLE SURG, V8, P267
   Chiodo CP, 2006, FOOT ANKLE INT, V27, P305, DOI 10.1177/107110070602700415
   Cretnik A, 2005, AM J SPORT MED, V33, P1369, DOI 10.1177/0363546504271501
   Doral Mahmut Nedim, 2002, Acta Orthop Traumatol Turc, V36 Suppl 1, P42
   FIERRO NL, 1995, POSTGRAD MED, V98, P145, DOI 10.1080/00325481.1995.11946048
   FITZGIBBONS RE, 1993, AM J SPORT MED, V21, P724, DOI 10.1177/036354659302100516
   Fortis AP, 2008, ARTHROSCOPY, V24, P683, DOI 10.1016/j.arthro.2008.02.018
   Gigante A, 2008, KNEE SURG SPORT TR A, V16, P204, DOI 10.1007/s00167-007-0448-z
   Goren D, 2005, FOOT ANKLE INT, V26, P286, DOI 10.1177/107110070502600404
   Gorschewsky O, 1999, INJURY, V30, P315, DOI 10.1016/S0020-1383(99)00089-3
   Haji A, 2004, FOOT ANKLE INT, V25, P215, DOI 10.1177/107110070402500404
   Halasi T, 2003, KNEE SURG SPORT TR A, V11, P409, DOI 10.1007/s00167-003-0397-0
   INGLIS AE, 1976, J BONE JOINT SURG AM, V58, P990, DOI 10.2106/00004623-197658070-00015
   Ingvar J, 2005, ACTA ORTHOP, V76, P597, DOI 10.1080/17453670510041619
   Kangas J, 2003, J TRAUMA, V54, P1171, DOI 10.1097/01.TA.0000047945.20863.A2
   Khan RJK, 2005, J BONE JOINT SURG AM, V87A, P2202, DOI 10.2106/JBJS.D.03049
   KITAOKA HB, 1994, FOOT ANKLE INT, V15, P349, DOI 10.1177/107110079401500701
   Kocher MS, 2002, AM J SPORT MED, V30, P783, DOI 10.1177/03635465020300060501
   Leppilahti J, 1998, SPORTS MED, V25, P79, DOI 10.2165/00007256-199825020-00002
   Leppilahti J, 1996, ACTA ORTHOP SCAND, V67, P277, DOI 10.3109/17453679608994688
   Li Jun, 2007, Zhongguo Xiu Fu Chong Jian Wai Ke Za Zhi, V21, P367
   LIEM MD, 1991, AM J ROENTGENOL, V156, P769, DOI 10.2214/ajr.156.4.2003444
   Lim J, 2001, FOOT ANKLE INT, V22, P559, DOI 10.1177/107110070102200705
   LUI TH, 2007, ARTHROSCOPY, V23
   MA GWC, 1977, CLIN ORTHOP RELAT R, P247
   Maffulli N, 1999, J BONE JOINT SURG AM, V81A, P1019, DOI 10.2106/00004623-199907000-00017
   Majewski M, 2000, ORTHOPADE, V29, P670
   Moller M, 2001, J BONE JOINT SURG BR, V83B, P843
   Moller M, 2002, KNEE SURG SPORT TR A, V10, P49, DOI 10.1007/s001670100245
   Mortensen NHM, 1999, J BONE JOINT SURG AM, V81A, P983, DOI 10.2106/00004623-199907000-00011
   Popovic N, 1999, Acta Orthop Belg, V65, P458
   Rebeccato A, 2001, J Foot Ankle Surg, V40, P188
   ROWLEY DI, 1982, INJURY, V14, P252, DOI 10.1016/0020-1383(82)90138-3
   Sauer ST, 2004, J BONE JOINT SURG AM, V86A, P878, DOI 10.2106/00004623-200404000-00045
   Strauss EJ, 2007, INJURY, V38, P832, DOI 10.1016/j.injury.2006.06.005
   Suchak AA, 2005, FOOT ANKLE INT, V26, P932, DOI 10.1177/107110070502601106
   Takao M, 2003, ARCH ORTHOP TRAUM SU, V123, P471, DOI 10.1007/s00402-002-0443-2
   Tang KL, 2007, AM J SPORT MED, V35, P589, DOI 10.1177/0363546506295080
   Thermann H, 2001, FOOT ANKLE INT, V22, P158, DOI 10.1177/107110070102200214
   Turgut A, 2002, KNEE SURG SPORT TR A, V10, P130, DOI 10.1007/s00167-001-0258-7
   Wagnon Raymond, 2005, J Foot Ankle Surg, V44, P437, DOI 10.1053/j.jfas.2005.07.015
   Webb JM, 1999, J BONE JOINT SURG BR, V81B, P877, DOI 10.1302/0301-620X.81B5.9784
   Weber M, 2003, AM J SPORT MED, V31, P685, DOI 10.1177/03635465030310050901
   WILLS CA, 1986, CLIN ORTHOP RELAT R, P156
   Winter E, 1998, ARCH ORTHOP TRAUM SU, V117, P364, DOI 10.1007/s004020050267
   Wong J, 2002, AM J SPORT MED, V30, P565, DOI 10.1177/03635465020300041701
NR 57
TC 34
Z9 36
U1 0
U2 8
PU SPRINGER
PI NEW YORK
PA 233 SPRING ST, NEW YORK, NY 10013 USA
SN 0936-8051
EI 1434-3916
J9 ARCH ORTHOP TRAUM SU
JI Arch. Orthop. Trauma Surg.
PD AUG
PY 2009
VL 129
IS 8
BP 1093
EP 1101
DI 10.1007/s00402-009-0880-2
PG 9
WC Orthopedics; Surgery
WE Science Citation Index Expanded (SCI-EXPANDED)
SC Orthopedics; Surgery
GA 462WX
UT WOS:000267390000015
PM 19404654
DA 2022-07-22
ER

PT J
AU Yuksel, S
   Adanir, O
   Gultekin, MZ
   Caglar, A
   Kucukyildirim, BO
   Gulec, MA
   Alagoz, E
AF Yuksel, Serdar
   Adanir, Oktay
   Gultekin, Muhammet Zeki
   Caglar, Aysel
   Kucukyildirim, Bedri Onur
   Gulec, Mehmet Akif
   Alagoz, Ender
TI Effect of platelet-rich plasma for treatment of Achilles tendons in
   free-moving rats after surgical incision and treatment
SO ACTA ORTHOPAEDICA ET TRAUMATOLOGICA TURCICA
LA English
DT Article
DE Achilles tendon injuries; biomechanics; histopathology; platelet-rich
   plasma; rat
ID RANDOMIZED CONTROLLED-TRIAL; HEALING IN-VITRO; GROWTH-FACTOR; INJECTION;
   MODEL; CONCENTRATE; INJURIES; REPAIR; VIVO; ACL
AB Objective: The purpose of this study is to investigate, both histopathologically and biomechanically, the effect of platelet-rich plasma (PRP) on surgically repaired Achilles tendon rupture recovery in rats.
   Methods: The study included 25 17-month-old male Sprague-Dawley rats with an average weight of 500-550 g. Five rats were used as donors, while 20 rats were separated as PRP group (n=10) and control group (n=10). The Achilles tendons of the rats were cut transversely, and PRP was administered to the PRP group, while the control group received serum physiologically to create the same surgical effect. In previous studies, it was shown that this serum does not affect tendon recovery. Fifteen and 30 days post-treatment, the rats were sacrificed, and their Achilles tendons were extracted and examined histopathologically and biomechanically.
   Results: Based on the obtained findings, it was observed that the histopathologic Movin and Bonar scores of PRP group on the 15th and 30th day post-treatment were significantly lower than those of the control group (p=0.019, p=0.017, respectively), while no significant difference was found in maximum force (Fmax) values in biomechanical measures on the 15th and 30th day post-treatment.
   Conclusion: It is thought that PRP use in Achilles tendon ruptures positively affects histopathological recovery in the early period, but that it does not produce the same biomechanical effect. We believe that PRP use for qualified tendon recovery is histopathologically beneficial.
C1 [Yuksel, Serdar] Trabzon Kanuni Training & Res Hosp, Dept Orthoped & Traumatol, Trabzon, Turkey.
   [Adanir, Oktay; Gulec, Mehmet Akif; Alagoz, Ender] Bagcilar Training & Res Hosp, Dept Orthoped & Traumatol, Istanbul, Turkey.
   [Gultekin, Muhammet Zeki] Sanliurfa Balikligol State Hosp, Dept Orthoped & Traumatol, Sanliurfa, Turkey.
   [Caglar, Aysel] Bagcilar Training & Res Hosp, Dept Pathol, Istanbul, Turkey.
   [Kucukyildirim, Bedri Onur] Yildiz Tech Univ, Dept Mech Engn, Istanbul, Turkey.
RP Yuksel, S (通讯作者)，Trabzon Kanuni Egitim & Arastirma Hastanesi, Ortopedi & Travmatoloji Klin, Trabzon, Turkey.
EM serdar84yuksel@gmail.com
RI Kucukyildirim, Bedri Onur/AAZ-6726-2020; Güleç, Akif/AAU-4848-2020;
   alagöz, ender/AAU-4953-2020; Adanır, Oktay/AAT-7040-2020
FU Bagcilar Training and Research Hospital Management; Bagcilar Training
   and Research Hospital Experimental Research and Skill Development Center
   (BADABEM)
FX For their contributions and support, we would like to thank the Bagcilar
   Training and Research Hospital Management and the Bagcilar Training and
   Research Hospital Experimental Research and Skill Development Center
   (BADABEM). For their help during the biomechanical laboratory studies,
   we would like to thank Yildiz Technical University Mechanical
   Engineering Department member Prof. Dr. Aysegul Akdogan Eker and
   Research Asst. Ali Avci.
CR Alissa R, 2010, EUR J ORAL IMPLANTOL, V3, P121
   Anitua E, 2006, TRENDS BIOTECHNOL, V24, P227, DOI 10.1016/j.tibtech.2006.02.010
   Aspenberg P, 2004, ACTA ORTHOP SCAND, V75, P93, DOI 10.1080/00016470410001708190
   Babbush Charles A, 2003, Implant Dent, V12, P24
   CETTI R, 1993, AM J SPORT MED, V21, P791, DOI 10.1177/036354659302100606
   Creaney L, 2008, BRIT J SPORT MED, V42, DOI 10.1136/bjsm.2007.040071
   de Vos RJ, 2010, JAMA-J AM MED ASSOC, V303, P144, DOI 10.1001/jama.2009.1986
   Freymiller EG, 2004, J ORAL MAXIL SURG, V62, P1046, DOI 10.1016/j.joms.2004.05.205
   Gardner MJ, 2007, INT ORTHOP, V31, P309, DOI 10.1007/s00264-006-0174-z
   Hapa O, 2013, ACTA ORTHOP TRAUMATO, V47, P201, DOI 10.3944/AOTT.2013.2928
   Karahan M, 2004, TURK ORTOPEDI VE TRA, V3, P1
   Kashiwagi K, 2004, SCAND J PLAST RECONS, V38, P193, DOI 10.1080/02844310410029110
   Kaux JF, 2012, WOUND REPAIR REGEN, V20, P748, DOI 10.1111/j.1524-475X.2012.00826.x
   KRACKOW KA, 1986, J BONE JOINT SURG AM, V68A, P764, DOI 10.2106/00004623-198668050-00020
   Majewski M, 2009, AM J SPORT MED, V37, P2117, DOI 10.1177/0363546509348047
   Marx R E, 2001, Implant Dent, V10, P225
   Marx RE, 1998, ORAL SURG ORAL MED O, V85, P638, DOI 10.1016/S1079-2104(98)90029-4
   Moller M, 2002, J BONE JOINT SURG AM, V84A, P324, DOI 10.2106/00004623-200202000-00031
   Morizaki Y, 2010, J HAND SURG-AM, V35A, P1833, DOI 10.1016/j.jhsa.2010.07.034
   Murray MM, 2009, J ORTHOP RES, V27, P639, DOI 10.1002/jor.20796
   Nurden AT, 2008, FRONT BIOSCI-LANDMRK, V13, DOI 10.2741/2947
   Orrego M, 2008, ARTHROSCOPY, V24, P1373, DOI 10.1016/j.arthro.2008.07.016
   Peerbooms JC, 2010, AM J SPORT MED, V38, P255, DOI 10.1177/0363546509355445
   Puddu G, 1976, Am J Sports Med, V4, P145, DOI 10.1177/036354657600400404
   Rickert M, 2001, GROWTH FACTORS, V19, P115, DOI 10.3109/08977190109001080
   ROBERTS AB, 1993, GROWTH FACTORS, V8, P1
   Rozman P, 2007, ACTA DERMATOVEN ALP, V16, P156
   Sadoghi P, 2013, J ORTHOP RES, V31, P111, DOI 10.1002/jor.22199
   Sanchez M, 2007, AM J SPORT MED, V35, P245, DOI 10.1177/0363546506294078
   Spindler KP, 2009, J ORTHOP RES, V27, P631, DOI 10.1002/jor.20785
   Suchak AA, 2005, FOOT ANKLE INT, V26, P932, DOI 10.1177/107110070502601106
   Taylor DW, 2011, CLIN J SPORT MED, V21, P344, DOI 10.1097/JSM.0b013e31821d0f65
   Virchenko O, 2006, ACTA ORTHOP, V77, P806, DOI 10.1080/17453670610013033
   Wang XT, 2004, J HAND SURG-AM, V29A, P884, DOI 10.1016/j.jhsa.2004.05.016
   Zhang F, 2003, PLAST RECONSTR SURG, V112, P1613, DOI 10.1097/01.PRS.0000086772.72535.A4
NR 35
TC 9
Z9 9
U1 0
U2 2
PU TURKISH ASSOC ORTHOPAEDICS TRAUMATOLOGY
PI ISTANBUL
PA SEHREMINI MAH KOYUNCU SK CIGDEM APT NO 4 D 5 FATIH, ISTANBUL, 00000,
   TURKEY
SN 1017-995X
J9 ACTA ORTHOP TRAUMATO
JI Acta Orthop. Traumatol. Turc.
PY 2015
VL 49
IS 5
BP 544
EP 551
DI 10.3944/AOTT.2015.15.0028
PG 8
WC Orthopedics
WE Science Citation Index Expanded (SCI-EXPANDED)
SC Orthopedics
GA CT7WK
UT WOS:000363025000014
PM 26422351
OA gold, Green Submitted
DA 2022-07-22
ER

PT J
AU Kerkhoffs, GMMJ
   Struijs, PAA
   Raaymakers, ELFB
   Marti, RK
AF Kerkhoffs, GMMJ
   Struijs, PAA
   Raaymakers, ELFB
   Marti, RK
TI Functional treatment after surgical repair of acute Achilles tendon
   rupture: wrap vs walking cast
SO ARCHIVES OF ORTHOPAEDIC AND TRAUMA SURGERY
LA English
DT Article
DE spontaneous Achilles tendon rupture; wrap; early mobilization; walking
   cast
ID WEIGHTBEARING; MOTION
AB The aim of this study was to compare the clinical outcome for patients treated with walking cast immobilization and wrap early mobilization after surgical repair of acute Achilles tendon ruptures. A total of 39 consecutive patients with complete ruptures of the Achilles tendon were identified, treated, and functionally rehabilitated with either a walking cast or a wrap. Because the randomization was quasi-random, chi-square and t-tests were performed to compare the baseline characteristics. A statistically significant difference was present only for the injured side (p<0.05). Therefore, groups were considered comparable for analysis of outcome. All patients were evaluated at an average follow-up of 6.7 years (range 5-8 years). Functional postoperative treatment with a wrap allowed a significantly shorter hospital stay (p<0.05) as well as a shorter period to return to pre-injury sports level (p<0.01) compared with treatment with a walking cast. According to the modified Rupp score, 91.3% of patients in the walking cast group had a good or excellent result, as did 93.8% in the wrap group (p=0.9). Slight atrophy of the calf muscles was reported in 3 patients in the walking cast group (13.0%) and in 4 in the wrap group (25.0%). One re-rupture was reported in the walking cast group (4.3%). Functional treatment after surgical Achilles tendon repair is safe, and there is no increased risk of re-rupture or wound healing problems. Functional treatment with a wrap is preferable to treatment with a walking cast with respect to hospitalization time and return to sports.
C1 Univ Amsterdam, Acad Med Ctr, Dept Orthopaed Surg, NL-1105 AZ Amsterdam, Netherlands.
   Univ Amsterdam, Acad Med Ctr, Dept Traumatol, NL-1105 AZ Amsterdam, Netherlands.
RP Kerkhoffs, GMMJ (通讯作者)，Univ Amsterdam, Acad Med Ctr, Dept Orthopaed Surg, POB 22700, NL-1105 AZ Amsterdam, Netherlands.
RI Struijs, Peter/AAH-5148-2019
CR Aoki M, 1998, AM J SPORT MED, V26, P794, DOI 10.1177/03635465980260061001
   ARNER O, 1959, Acta Chir Scand, V116, P484
   CETTI R, 1993, AM J SPORT MED, V21, P791, DOI 10.1177/036354659302100606
   FITZGIBBONS RE, 1993, AM J SPORT MED, V21, P724, DOI 10.1177/036354659302100516
   LAGERGREN C, 1959, Acta Chir Scand, V116, P491
   Leppilahti J, 1996, ACTA ORTHOP SCAND, V67, P277, DOI 10.3109/17453679608994688
   MANDELBAUM BR, 1995, AM J SPORT MED, V23, P392, DOI 10.1177/036354659502300403
   MARTI R, 1974, HELV CHIR ACTA, V41, P293
   MARTI RK, 1983, NETH J SURG, V35, P61
   Speck M, 1998, AM J SPORT MED, V26, P789, DOI 10.1177/03635465980260060901
NR 10
TC 50
Z9 54
U1 0
U2 7
PU SPRINGER-VERLAG
PI NEW YORK
PA 175 FIFTH AVE, NEW YORK, NY 10010 USA
SN 0936-8051
J9 ARCH ORTHOP TRAUM SU
JI Arch. Orthop. Trauma Surg.
PD MAR
PY 2002
VL 122
IS 2
BP 102
EP 105
DI 10.1007/s004020100312
PG 4
WC Orthopedics; Surgery
WE Science Citation Index Expanded (SCI-EXPANDED)
SC Orthopedics; Surgery
GA 538GK
UT WOS:000174806900009
PM 11880912
DA 2022-07-22
ER

PT J
AU Yu, H
   Wang, FY
   Xie, J
   Yao, YF
   Jing, JH
   Li, J
AF Yu, Hao
   Wang, Fangyuan
   Xie, Jia
   Yao, Yunfeng
   Jing, Juehua
   Li, Jun
TI The Reinforced Ma-Griffith Method Combined with Minimally Invasive
   Small-Incision Suture for Acute Achilles Tendon Rupture
SO ORTHOPAEDIC SURGERY
LA English
DT Article
DE Achilles tendon; Minimally invasive surgery; Reinforced Ma-Griffith
   method; Rupture; Small incision
ID OPEN REPAIR; PERCUTANEOUS REPAIR; ANKLE-HINDFOOT; MIDFOOT; HALLUX; SCORE
AB Objective To evaluate the treatment effects of the reinforced Ma-Griffith method combined with a minimally invasive small incision(M-G/MISI) in the treatment of acute Achilles tendon rupture. Methods From January 2012 to January 2020, a retrospective study was carried out on thirty-one patients with acute Achilles tendon ruptures that were treated using the M-G/MISI. Patient with acute Achilles tendon rupture was operated on in the prone position. The M-G/MISI begin with making a small incision to debride the stumps of ruptured tendon. Then M-G/MISI was used to suture the distal and proximal Achilles tendons with the help of a epidural puncture needle and polydioxanone synthetic absorbable suture (PDS) II line. Finally the stumps of ruptured tendon was reattached. After the surgery, the affected limb was fixed with either a plaster slab below the knee brace or a functional brace. Removal of plaster external fixation and partial weight-bearing with crutches five weeks after the operation; Complete weight-bearing nine weeks after the operation; jogging permitted 12 weeks after the operation; Patients were allowed to resume normal activities six months after the operation. Results All 31 patients in this study were male. Nineteen of these patients had Achilles tendon rupture on the right lower extremity, while 12 had ruptures on the left lower extremity. The patients had a mean age of 33.35 +/- 7.13 years (range, 18-52 years). The mean operation time was 79.58 +/- 22.67 minutes (range, 40-167 minutes). The mean time from injury to operation was 4.19 +/- 2.01 days (range, 1-8 days), and the mean hospital stay was 9.87 +/- 3.88 days (range, 5-22 days). The new technique had a small incision with a mean length of 3.94 +/- 1.82 cm (range, 2-6 cm). The mean intraoperative blood loss was 16.77 +/- 13.76 mL (range, 10-50 mL) and the mean follow-up time was 21.35 +/- 10.18 months (range, 6-50 months). No wound infection, fistula, skin necrosis, sural nerve damage, deep venous thrombosis or tendon re-rupture was found. One year after the surgery, all patients reported 97.00 (range, 93-100 points) AOFAS ankle-hindfoot score points and the mean ATRS was 97.39 (range, 91-100) points. Conclusion The reinforced Ma-Griffith method, combined with a minimally invasive small incision suture, is a simple, effective, minimally invasive technique and low-cost surgical method for the treatment of acute Achilles tendon rupture.
C1 [Yu, Hao; Wang, Fangyuan; Xie, Jia; Yao, Yunfeng; Jing, Juehua; Li, Jun] Anhui Med Univ, Affiliated Hosp 2, Dept Orthopaed, 678 Furong Ave, Hefei 230601, Anhui, Peoples R China.
RP Li, J (通讯作者)，Anhui Med Univ, Affiliated Hosp 2, Dept Orthopaed, 678 Furong Ave, Hefei 230601, Anhui, Peoples R China.
EM efylijunpaper@163.com
FU Youth Project of National Natural Science Foundation of China
   [81702656]; Clinical Research and Cultivation Program of the Second
   Affiliated Hospital of Anhui Medical University [2020LCZD20]
FX Youth Project of National Natural Science Foundation of China (No.:
   81702656); Clinical Research and Cultivation Program of the Second
   Affiliated Hospital of Anhui Medical University (No.: 2020LCZD20).
CR Ahmed IM, 1998, J ORTHOPAED RES, V16, P591, DOI 10.1002/jor.1100160511
   Alcelik I, 2018, FOOT ANKLE SURG, V24, P427, DOI 10.1016/j.fas.2017.04.016
   Ames PRJ, 2008, DISABIL REHABIL, V30, P1646, DOI 10.1080/09638280701785882
   Assal M, 2002, J BONE JOINT SURG AM, V84A, P161, DOI 10.2106/00004623-200202000-00001
   Beddy P, 2009, AM J ROENTGENOL, V192, pW79, DOI 10.2214/AJR.08.1654
   BRADLEY JP, 1990, AM J SPORT MED, V18, P188, DOI 10.1177/036354659001800213
   Carmont MR, 2008, KNEE SURG SPORT TR A, V16, P199, DOI 10.1007/s00167-007-0411-z
   Cretnik A, 2005, AM J SPORT MED, V33, P1369, DOI 10.1177/0363546504271501
   Gigante A, 2008, KNEE SURG SPORT TR A, V16, P204, DOI 10.1007/s00167-007-0448-z
   Hansen MS, 2016, KNEE SURG SPORT TR A, V24, P1365, DOI 10.1007/s00167-015-3974-0
   Jallageas R, 2013, ORTHOP TRAUMATOL-SUR, V99, P577, DOI 10.1016/j.otsr.2013.03.024
   KAKIUCHI M, 1995, J BONE JOINT SURG BR, V77B, P60, DOI 10.1302/0301-620X.77B1.7822398
   Kitaoka HB, 1997, FOOT ANKLE INT, V18, P187, DOI 10.1177/107110079701800315
   Lemme NJ, 2018, ORTHOP J SPORTS MED, V6, DOI 10.1177/2325967118808238
   Levi N, 1997, INJURY, V28, P311, DOI 10.1016/S0020-1383(96)00200-8
   MA GWC, 1977, CLIN ORTHOP RELAT R, P247
   Nilsson-Helander K, 2007, AM J SPORT MED, V35, P421, DOI 10.1177/0363546506294856
   Park YH, 2019, AM J SPORT MED, V47, P2374, DOI 10.1177/0363546519858990
   Rippstein Pascal F, 2002, Foot Ankle Clin, V7, P611, DOI 10.1016/S1083-7515(02)00040-2
   Schneider W, 2016, INT ORTHOP, V40, P301, DOI 10.1007/s00264-015-3066-2
   Valente Maurizio, 2012, Musculoskelet Surg, V96, P35, DOI 10.1007/s12306-011-0158-z
   Wilkins R, 2012, AM J SPORT MED, V40, P2154, DOI 10.1177/0363546512453293
   Yang B, 2017, INT J SURG, V40, P178, DOI 10.1016/j.ijsu.2017.03.021
   Zhou K, 2018, J FOOT ANKLE SURG, V57, P1191, DOI 10.1053/j.jfas.2018.05.007
NR 24
TC 0
Z9 0
U1 1
U2 1
PU WILEY
PI HOBOKEN
PA 111 RIVER ST, HOBOKEN 07030-5774, NJ USA
SN 1757-7853
EI 1757-7861
J9 ORTHOP SURG
JI Orthop. Surg.
PD FEB
PY 2022
VL 14
IS 2
BP 315
EP 322
DI 10.1111/os.13140
EA DEC 2021
PG 8
WC Orthopedics
WE Science Citation Index Expanded (SCI-EXPANDED)
SC Orthopedics
GA ZG5NW
UT WOS:000734132200001
PM 34939329
OA Green Published
DA 2022-07-22
ER

PT J
AU Amin, NH
   Old, AB
   Tabb, LP
   Garg, R
   Toossi, N
   Cerynik, DL
AF Amin, Nirav H.
   Old, Andrew B.
   Tabb, Loni P.
   Garg, Rohit
   Toossi, Nader
   Cerynik, Douglas L.
TI Performance Outcomes After Repair of Complete Achilles Tendon Ruptures
   in National Basketball Association Players
SO AMERICAN JOURNAL OF SPORTS MEDICINE
LA English
DT Article
DE Achilles tendon rupture; National Basketball Association; NBA players;
   athletic performance; outcome measure
ID NONOPERATIVE TREATMENT; ANKLE INJURIES; EPIDEMIOLOGY; MICROFRACTURE;
   RISK
AB Background: A complete rupture of the Achilles tendon is a devastating injury. Variables affecting return to competition and performance changes for National Basketball Association (NBA) players are not readily evident.
   Hypothesis: Players in the NBA who ruptured their Achilles tendons and who underwent surgical repair would have more experience in the league, and the performance of those who were able to return to competition would be decreased when compared with their performance before injury and with their control-matched peers.
   Study Design: Cohort study; Level of evidence, 3.
   Methods: Data for 18 basketball players with Achilles tendon repair over a 23-year period (1988-2011) were obtained from injury reports, press releases, and player profiles. Variables included age, body mass index (BMI), player position, and number of years playing in the league. Individual season statistics were obtained, and the NBA Player Efficiency Rating (PER) was calculated for 2 seasons before and after injury. Controls were matched by playing position, number of seasons played, and performance statistics. Univariate and multivariate analyses were performed to assess the effect of each factor.
   Results: At the time of injury, the average age was 29.7 years, average BMI was 25.6, and average playing experience was 7.6 years. Seven players never returned to play an NBA game, whereas 11 players returned to play 1 season, with 8 of those players returning for 2 seasons. Players who returned missed an average of 55.9 games. The PER was reduced by 4.57 (P = .003) in the first season and by 4.38 (P = .010) in the second season. When compared with controls, players demonstrated a significant decline in the PER the first season (P = .038) and second season (P = .081) after their return.
   Conclusion: The NBA players who returned to play after repair of complete Achilles tendon ruptures showed a significant decrease in playing time and performance. Thirty-nine percent of players never returned to play.
C1 [Amin, Nirav H.; Old, Andrew B.; Tabb, Loni P.; Garg, Rohit; Toossi, Nader; Cerynik, Douglas L.] Drexel Univ, Coll Med, Dept Orthopaed Surg, Philadelphia, PA 19102 USA.
RP Cerynik, DL (通讯作者)，Drexel Univ, Coll Med, Dept Orthopaed Surg, 245 N 15th St, Philadelphia, PA 19102 USA.
EM douglas.cerynik@drexelmed.edu
RI Toossi, Nader/AAD-8948-2019; Amin, Nirav/J-7177-2019
OI Toossi, Nader/0000-0002-4167-2976;
CR Cerynik DL, 2009, KNEE SURG SPORT TR A, V17, P1135, DOI 10.1007/s00167-009-0765-5
   Deitch JR, 2006, AM J SPORT MED, V34, P1077, DOI 10.1177/0363546505285383
   FIRTH D, 1993, BIOMETRIKA, V80, P27, DOI 10.1093/biomet/80.1.27
   Garrick James G, 2012, Clin J Sport Med, V22, P379, DOI 10.1097/JSM.0b013e3182603905
   Gwynne-Jones DP, 2011, FOOT ANKLE INT, V32, P337, DOI 10.3113/FAI.2011.0337
   Klein Erin E, 2013, Foot Ankle Spec, V6, P15, DOI 10.1177/1938640012463052
   Kujala UM, 2005, CLIN J SPORT MED, V15, P133, DOI 10.1097/01.jsm.0000165347.55638.23
   McKay GD, 2001, BRIT J SPORT MED, V35, P103, DOI 10.1136/bjsm.35.2.103
   Namdari S, 2009, AM J SPORT MED, V37, P943, DOI 10.1177/0363546508330150
   Nigg B M, 2001, Exerc Sport Sci Rev, V29, P37, DOI 10.1097/00003677-200101000-00008
   Parekh Selene G, 2009, Foot Ankle Spec, V2, P283, DOI 10.1177/1938640009351138
   Saltzman C L, 1998, J Am Acad Orthop Surg, V6, P316
   Sankey RA, 2008, AM J SPORT MED, V36, P2415, DOI 10.1177/0363546508322889
   Soroceanu A, 2012, J BONE JOINT SURG AM, V94A, P2136, DOI 10.2106/JBJS.K.00917
   Starkey C, 2000, J ATHL TRAINING, V35, P161
   Willits K, 2010, J BONE JOINT SURG AM, V92A, P2767, DOI 10.2106/JBJS.I.01401
   Yeh PC, 2012, AM J SPORT MED, V40, P589, DOI 10.1177/0363546511428601
   ZELISKO JA, 1982, AM J SPORT MED, V10, P297, DOI 10.1177/036354658201000507
NR 18
TC 103
Z9 103
U1 0
U2 22
PU SAGE PUBLICATIONS INC
PI THOUSAND OAKS
PA 2455 TELLER RD, THOUSAND OAKS, CA 91320 USA
SN 0363-5465
EI 1552-3365
J9 AM J SPORT MED
JI Am. J. Sports Med.
PD AUG
PY 2013
VL 41
IS 8
BP 1864
EP 1868
DI 10.1177/0363546513490659
PG 5
WC Orthopedics; Sport Sciences
WE Science Citation Index Expanded (SCI-EXPANDED)
SC Orthopedics; Sport Sciences
GA 235HN
UT WOS:000325708000021
PM 23733634
DA 2022-07-22
ER

PT J
AU Holzgrefe, RE
   McCarthy, TP
   Wilson, JM
   Bariteau, JT
   Labib, S
AF Holzgrefe, Russell E.
   McCarthy, Timothy P.
   Wilson, Jacob M.
   Bariteau, Jason T.
   Labib, Sameh
TI Association of Strength Following Achilles Tendon Repair With Return to
   Same Level of Play in High-Level Athletes
SO FOOT & ANKLE INTERNATIONAL
LA English
DT Article
DE Achilles tendon rupture; Achilles tendon repair; strength; return to
   play; return to sport
ID NONOPERATIVE TREATMENT; NONSURGICAL TREATMENTS; PERCUTANEOUS SUTURE;
   SURGICAL REPAIR; LYSHOLM SCORE; RUPTURES; PERFORMANCE; SURGERY;
   ENDURANCE; SPORT
AB Background: Rupture of the Achilles tendon is a common injury and the ability to return to the same level of sporting activity after treatment is an important outcome for patients. The objective of the current study was to examine the relationship between ankle strength and the ability to return to previous level of play following operative repair of an Achilles tendon rupture. Methods: Patients aged 18 to 50 years at a minimum 1 year postoperation from surgical repair of an Achilles tendon returned for a study visit. Patients reported both preinjury and current activity level using the 10-point Tegner Activity Level Scale. Isokinetic strength testing was performed and the Isokinetic Strength Score (ISS) was calculated. Logistic regression analysis was used to determine the relationship between ISS and return to play by Tegner level. A total of 36 patients (mean 35 years old, 72% male) completed the study protocol at a mean 1.8 years postoperatively. Results: Logistic regression revealed no association between ISS and return to play in the complete cohort. Subgroup analysis revealed that for 20 high-level athletes (preinjury Tegner >= 7), for every 16-point increase in ISS, the odds ratio (OR) for return to same level of play was 8.3 (P= .055) and the OR for return to within 1 Tegner level of play was 6.3 (P= .043). There was no association between ISS and return to previous activity in the 16 patients with lower preinjury levels of activity. Conclusion: Improved ankle strength was associated with return to previous level of activity in patients participating in high-level athletic activity, suggesting that these patients were more dependent on recovery of ankle strength in the postoperative time period in order to return to their previous high level of play. In contrast, regaining strength may be less important for returning to normal activities for less active patients.
C1 [Holzgrefe, Russell E.; Wilson, Jacob M.; Bariteau, Jason T.; Labib, Sameh] Emory Univ, Dept Orthopaed Surg, 59 Execut Pk S, Atlanta, GA 30324 USA.
   [McCarthy, Timothy P.] USAF, Landstuhl Reg Med Ctr, Washington, DC USA.
RP Holzgrefe, RE (通讯作者)，Emory Univ, Dept Orthopaed Surg, 59 Execut Pk S, Atlanta, GA 30324 USA.
EM rholzgr@emory.edu
CR Alcelik I, 2017, ACTA ORTHOP BELG, V83, P387
   Amin NH, 2013, AM J SPORT MED, V41, P1864, DOI 10.1177/0363546513490659
   Baumfeld D, 2019, FOOT ANKLE SURG, V25, P503, DOI 10.1016/j.fas.2018.03.003
   Bostick GP, 2010, J ORTHOP SPORT PHYS, V40, P345, DOI 10.2519/jospt.2010.3204
   Briggs KK, 2009, AM J SPORT MED, V37, P890, DOI 10.1177/0363546508330143
   Briggs KK, 2009, AM J SPORT MED, V37, P898, DOI 10.1177/0363546508330149
   Brorsson A, 2018, AM J SPORT MED, V46, P470, DOI 10.1177/0363546517737055
   Burland JP, 2018, J ATHL TRAINING, V53, P442, DOI 10.4085/1062-6050-302-16
   Carmont MR, 2020, ORTHOP J SPORTS MED, V8, DOI 10.1177/2325967120909556
   Czuppon S, 2014, BRIT J SPORT MED, V48, DOI 10.1136/bjsports-2012-091786
   Ganestam A, 2016, KNEE SURG SPORT TR A, V24, P3730, DOI 10.1007/s00167-015-3544-5
   Gigante A, 2008, KNEE SURG SPORT TR A, V16, P204, DOI 10.1007/s00167-007-0448-z
   Goren D, 2005, FOOT ANKLE INT, V26, P286, DOI 10.1177/107110070502600404
   Grassi A, 2019, BR J SPORTS MED, V54
   Henriquez H, 2012, CLIN ORTHOP RELAT R, V470, P998, DOI 10.1007/s11999-011-1830-1
   Ibrahim SAR, 2009, J FOOT ANKLE SURG, V48, P340, DOI 10.1053/j.jfas.2009.02.007
   Ithurburn MP, 2019, KNEE, V26, P355, DOI 10.1016/j.knee.2019.01.011
   Jack RA, 2017, FOOT ANKLE INT, V38, P1092, DOI 10.1177/1071100717718131
   Kadakia AR, 2017, J AM ACAD ORTHOP SUR, V25, P23, DOI 10.5435/JAAOS-D-15-00187
   Keating JF, 2011, J BONE JOINT SURG BR, V93B, P1071, DOI 10.1302/0301-620X.93B8.25998
   Kosanovic M, 2008, ARCH ORTHOP TRAUM SU, V128, P211, DOI 10.1007/s00402-007-0514-5
   Lantto I, 2016, AM J SPORT MED, V44, P2406, DOI 10.1177/0363546516651060
   Leppilahti J, 1998, CLIN ORTHOP RELAT R, P152
   Lever CJ, 2018, BONE JOINT J, V100B, P584, DOI 10.1302/0301-620X.100B5.BJJ-2017-1053.R1
   Manegold S, 2018, J ORTHOP TRAUMATOL, V19, DOI 10.1186/s10195-018-0511-1
   McCullough Kirk A, 2014, J Surg Orthop Adv, V23, P179
   Metz R, 2008, AM J SPORT MED, V36, P1688, DOI 10.1177/0363546508319312
   Miao XD, 2016, J FOOT ANKLE SURG, V55, P529, DOI 10.1053/j.jfas.2016.01.016
   Mullaney MJ, 2006, AM J SPORT MED, V34, P1120, DOI 10.1177/0363546505284186
   Nilsson-Helander K, 2010, AM J SPORT MED, V38, P2186, DOI 10.1177/0363546510376052
   Ochen Y, 2019, BMJ-BRIT MED J, V364, DOI 10.1136/bmj.k5120
   Olsson N, 2013, AM J SPORT MED, V41, P2867, DOI 10.1177/0363546513503282
   Philippot R, 2010, FOOT ANKLE INT, V31, P802, DOI 10.3113/FAI.2010.0802
   Pi YB, 2019, AM J SPORT MED, V47, P2993, DOI 10.1177/0363546519869952
   Song YJ, 2018, KNEE SURG SPORT TR A, V28, P1619
   Soroceanu A, 2012, J BONE JOINT SURG AM, V94A, P2136, DOI 10.2106/JBJS.K.00917
   TEGNER Y, 1985, CLIN ORTHOP RELAT R, P43
   Trofa DP, 2018, ORTHOP J SPORTS MED, V6, DOI 10.1177/2325967118810772
   Trofa DP, 2017, AM J SPORT MED, V45, P2864, DOI 10.1177/0363546517713001
   Twaddle BC, 2007, AM J SPORT MED, V35, P2033, DOI 10.1177/0363546507307503
   Willits K, 2010, J BONE JOINT SURG AM, V92A, P2767, DOI 10.2106/JBJS.I.01401
   Zwolski C, 2015, AM J SPORT MED, V43, P2242, DOI 10.1177/0363546515591258
NR 42
TC 1
Z9 1
U1 0
U2 3
PU SAGE PUBLICATIONS INC
PI THOUSAND OAKS
PA 2455 TELLER RD, THOUSAND OAKS, CA 91320 USA
SN 1071-1007
EI 1944-7876
J9 FOOT ANKLE INT
JI Foot Ankle Int.
PD SEP
PY 2020
VL 41
IS 9
BP 1041
EP 1048
AR 1071100720936276
DI 10.1177/1071100720936276
EA JUL 2020
PG 8
WC Orthopedics
WE Science Citation Index Expanded (SCI-EXPANDED)
SC Orthopedics
GA NN3QA
UT WOS:000549971500001
PM 32660267
DA 2022-07-22
ER

PT J
AU Olsson, N
   Silbernagel, KG
   Eriksson, BI
   Sansone, M
   Brorsson, A
   Nilsson-Helander, K
   Karlsson, J
AF Olsson, Nicklas
   Silbernagel, Karin Gravare
   Eriksson, Bengt I.
   Sansone, Mikael
   Brorsson, Annelie
   Nilsson-Helander, Katarina
   Karlsson, Jon
TI Stable Surgical Repair With Accelerated Rehabilitation Versus
   Nonsurgical Treatment for Acute Achilles Tendon Ruptures A Randomized
   Controlled Study
SO AMERICAN JOURNAL OF SPORTS MEDICINE
LA English
DT Article
DE Achilles tendon rupture; Achilles tendon Total Rupture Score; ATRS;
   functional evaluation; rerupture; heel-rise test; physical activity
   level; Foot and Ankle Outcome Score; FAOS; EQ-5D
ID NONOPERATIVE TREATMENT; IN-VITRO; WEIGHT-BEARING; FOLLOW-UP; STRENGTH;
   MUSCLE; SCORE; RESPONSIVENESS; WEIGHTBEARING; MOBILIZATION
AB Background: The optimal treatment for acute Achilles tendon ruptures is still a subject of debate. Early loading of the tendon is a factor that has been shown to be beneficial to recovery and to minimize complications. The main outcome of previous studies has been complications such as reruptures and deep infections, without focusing on the functional outcome relevant to the majority of patients who do not experience these complications.
   Purpose: To evaluate whether stable surgical repair and early loading of the tendon could improve patient-reported outcome and function after an acute Achilles tendon rupture.
   Study Design: Randomized controlled trial; Level of evidence, 1.
   Methods: A total of 100 patients (86 men, 14 women; mean age, 40 years) with an acute total Achilles tendon rupture were randomized to either surgical treatment, including an accelerated rehabilitation protocol, or nonsurgical treatment. The primary outcome was the Achilles tendon Total Rupture Score (ATRS). The patients were evaluated at 3, 6, and 12 months for symptoms, physical activity level, and function.
   Results: There were no significant differences between the groups in terms of symptoms, physical activity level, or quality of life. There was a trend toward improved function in surgically treated patients; the results were significantly superior when assessed by the drop countermovement jump (95% CI, 0.03-0.15; P = .003) and hopping (95% CI, 0.01-0.33; P = .040). No reruptures occurred in the surgical group, while there were 5 in the nonsurgical group (P = .06). There were 6 superficial infections in the surgically treated group; however, these superficial infections had no bearing on the final outcome. Symptoms, reduced quality of life, and functional deficits still existed 12 months after injury on the injured side in both groups.
   Conclusion: The results of the present study demonstrate that stable surgical repair with accelerated tendon loading could be performed in all (n = 49) patients without reruptures and major soft tissue-related complications. However, this treatment was not significantly superior to nonsurgical treatment in terms of functional results, physical activity, or quality of life.
C1 [Olsson, Nicklas; Silbernagel, Karin Gravare; Eriksson, Bengt I.; Sansone, Mikael; Brorsson, Annelie; Nilsson-Helander, Katarina; Karlsson, Jon] Univ Gothenburg, Sahlgrenska Univ Hosp, Inst Clin Sci, Sahlgrenska Acad, Molndal, Sweden.
RP Olsson, N (通讯作者)，Gothenburg Univ, Orthctr IFK Kliniken, Arvid Wallgrens Backe 4A, SE-41346 Gothenburg, Sweden.
EM nicklas.olsson@gu.se
RI Brorsson, Annelie/AAD-5986-2020; Helander, Katarina
   Nilsson/AAE-1793-2020
OI Helander, Katarina Nilsson/0000-0002-1292-5102; Gravare Silbernagel,
   Karin/0000-0001-7566-407X
CR Aspenberg P, 2007, INT ORTHOP, V31, P783, DOI 10.1007/s00264-007-0398-6
   Bergkvist D, 2012, J BONE JOINT SURG AM, V94A, P1229, DOI 10.2106/JBJS.J.01601
   Bhandari M, 2002, CLIN ORTHOP RELAT R, P190
   Bostick GP, 2010, J ORTHOP SPORT PHYS, V40, P345, DOI 10.2519/jospt.2010.3204
   Brooks R, 1996, HEALTH POLICY, V37, P53, DOI 10.1016/0168-8510(96)00822-6
   Carmont MR, 2013, KNEE SURG SPORT TR A, V21, P1356, DOI 10.1007/s00167-012-2146-8
   CETTI R, 1993, AM J SPORT MED, V21, P791, DOI 10.1177/036354659302100606
   Chiodo CP, 2010, J AM ACAD ORTHOP SUR, V18, P503, DOI 10.5435/00124635-201008000-00007
   Costa ML, 2006, J BONE JOINT SURG BR, V88B, P69, DOI 10.1302/0301-620X.88B1.16549
   Eliasson P, 2012, J ORTHOP RES, V30, P274, DOI 10.1002/jor.21511
   GRIMBY G, 1986, ACTA MED SCAND, P233
   Ingvar J, 2005, ACTA ORTHOP, V76, P597, DOI 10.1080/17453670510041619
   Jiang N, 2012, INT ORTHOP, V36, P765, DOI 10.1007/s00264-011-1431-3
   Kangas J, 2007, AM J SPORT MED, V35, P59, DOI 10.1177/0363546506293255
   Kearney RS, 2012, HEALTH QUAL LIFE OUT, V10, DOI 10.1186/1477-7525-10-24
   Keating JF, 2011, J BONE JOINT SURG BR, V93B, P1071, DOI 10.1302/0301-620X.93B8.25998
   Kessler I, 1973, Hand, V5, P253, DOI 10.1016/0072-968X(73)90038-7
   Khan RJK, 2005, J BONE JOINT SURG AM, V87A, P2202, DOI 10.2106/JBJS.D.03049
   Kjaer M, 2004, PHYSIOL REV, V84, P649, DOI 10.1152/physrev.00031.2003
   Kjaer M., 2005, Journal of Musculoskeletal & Neuronal Interactions, V5, P41
   Lister G D, 1977, J Hand Surg Am, V2, P441
   Maffulli N, 2003, AM J SPORT MED, V31, P692, DOI 10.1177/03635465030310051001
   Metz R, 2008, AM J SPORT MED, V36, P1688, DOI 10.1177/0363546508319312
   Moller M, 2001, J BONE JOINT SURG BR, V83B, P843
   Moller M, 2002, SCAND J MED SCI SPOR, V12, P9, DOI 10.1034/j.1600-0838.2002.120103.x
   MORTENSEN NHM, 1992, ORTHOPEDICS, V15, P899
   Mullaney MJ, 2006, AM J SPORT MED, V34, P1120, DOI 10.1177/0363546505284186
   Nilsson-Helander K, 2008, KNEE SURG SPORT TR A, V16, P614, DOI 10.1007/s00167-008-0492-3
   Nilsson-Helander K, 2007, AM J SPORT MED, V35, P421, DOI 10.1177/0363546506294856
   Nilsson-Helander K, 2010, AM J SPORT MED, V38, P2186, DOI 10.1177/0363546510376052
   Nilsson-Helander K, 2009, KNEE SURG SPORT TR A, V17, P1234, DOI 10.1007/s00167-009-0727-y
   Olsson N, 2011, KNEE SURG SPORT TR A, V19, P1385, DOI 10.1007/s00167-011-1511-3
   Roos EM, 2001, FOOT ANKLE INT, V22, P788, DOI 10.1177/107110070102201004
   Schepull T, 2007, BMC MUSCULOSKEL DIS, V8, DOI 10.1186/1471-2474-8-116
   Shaieb MD, 1997, J HAND SURG-BRIT EUR, V22B, P764, DOI 10.1016/S0266-7681(97)80444-5
   Shepard ME, 2007, FOOT ANKLE INT, V28, P1074, DOI 10.3113/FAI.2007.1074
   Silbernagel KG, 2012, AM J SPORT MED, V40, P1564, DOI 10.1177/0363546512447926
   Silbernagel KG, 2006, KNEE SURG SPORT TR A, V14, P1207, DOI 10.1007/s00167-006-0150-6
   Silbernagel KG, 2010, KNEE SURG SPORT TR A, V18, P258, DOI 10.1007/s00167-009-0889-7
   SILFVERSKIOLD KL, 1993, J HAND SURG-AM, V18A, P58, DOI 10.1016/0363-5023(93)90246-Y
   Suchak AA, 2008, J BONE JOINT SURG AM, V90A, P1876, DOI 10.2106/JBJS.G.01242
   THERMANN H, 1995, UNFALLCHIRURG, V98, P21
   THOMPSON T C, 1962, J Trauma, V2, P126, DOI 10.1097/00005373-196203000-00003
   Twaddle BC, 2007, AM J SPORT MED, V35, P2033, DOI 10.1177/0363546507307503
   WATSON TW, 1995, FOOT ANKLE INT, V16, P191, DOI 10.1177/107110079501600404
   WILLIAMS A, 1990, HEALTH POLICY, V16, P199
   Willits K, 2010, J BONE JOINT SURG AM, V92A, P2767, DOI 10.2106/JBJS.I.01401
   Yildirim Y, 2002, FOOT ANKLE INT, V23, P1126
NR 48
TC 180
Z9 181
U1 1
U2 16
PU SAGE PUBLICATIONS INC
PI THOUSAND OAKS
PA 2455 TELLER RD, THOUSAND OAKS, CA 91320 USA
SN 0363-5465
EI 1552-3365
J9 AM J SPORT MED
JI Am. J. Sports Med.
PD DEC
PY 2013
VL 41
IS 12
BP 2867
EP 2876
DI 10.1177/0363546513503282
PG 10
WC Orthopedics; Sport Sciences
WE Science Citation Index Expanded (SCI-EXPANDED)
SC Orthopedics; Sport Sciences
GA 258QH
UT WOS:000327473300020
PM 24013347
DA 2022-07-22
ER

PT J
AU Fu, CY
   Qu, W
AF Fu, Chongyang
   Qu, Wei
TI Acute Achilles tendon rupture: Mini-incision repair with double-Tsuge
   loop suture vs. open repair with modified Kessler suture
SO SURGEON-JOURNAL OF THE ROYAL COLLEGES OF SURGEONS OF EDINBURGH AND
   IRELAND
LA English
DT Article
DE Achilles tendon; Rupture; Repair; Double-Tsuge suture; Mini open repair
ID NONOPERATIVE TREATMENT; SURGICAL REPAIR; REHABILITATION; METAANALYSIS;
   MULTICENTER; INJURIES; TRIALS
AB Background and purpose: Achilles tendon rupture is a common injury of the foot and ankle. However, the optimal treatment strategy for Achilles tendon rupture is still not established. This study was Conducted to compare the efficacy and complications of mini-incision repair with double-Tsuge loop sutures and open repair with modified Kessler sutures.
   Methods: We evaluated data from 60 patients with acute closed Achilles tendon ruptures who underwent mini-incision repair with double-Tsuge loop sutures (n = 30) or open repair with modified Kessler sutures (n = 30) from 2006 to 2010 in an ongoing prospective study conducted by us and have finished at least 18-month follow-up or finished the study. The AOFAS Ankle-Hindfoot score, ATRS, maximal ankle range of motion and the time to achieve 20 continuous single heel raises after operation were recorded to compare the efficacy. The complications were also evaluated.
   Results: During a mean follow-up of 25 months after surgery, the time to achieve 20 continuous single heel raises after operation of patients in Group Mini was significantly shorter than patients in Group Open. Moreover, the mini-incision with double-Tsuge repair was associated with a significantly shorter operating time, smaller incision length, and lower rate of complications.
   Conclusion: The mini-incision with double-Tsuge suture method in our study was shown to provide earlier strength recovery, as well as shorter operation time, less complications and improved cosmetic appearance. (C) 2014 Royal College of Surgeons of Edinburgh (Scottish charity number SC005317) and Royal College of Surgeons in Ireland. Published by Elsevier Ltd. All rights reserved.
C1 [Fu, Chongyang; Qu, Wei] Dalian Med Univ, Affiliated Hosp 1, Dept Hand Surg & Microsurg, Dalian 116011, Liaoning Provin, Peoples R China.
RP Qu, W (通讯作者)，Dalian Med Univ, Affiliated Hosp 1, Dept Hand Surg & Microsurg, 222 Zhongshan Rd, Dalian 116011, Liaoning Provin, Peoples R China.
EM quwei333@aliyun.com
CR Ahluwalia R, 2013, FOOT ANKLE SURG, V19, pE7, DOI 10.1016/j.fas.2012.11.003
   Aibinder WR, 2013, FOOT ANKLE INT, V34, P870, DOI 10.1177/1071100712473097
   Assal M, 2002, J BONE JOINT SURG AM, V84A, P161, DOI 10.2106/00004623-200202000-00001
   Carmont MR, 2008, KNEE SURG SPORT TR A, V16, P199, DOI 10.1007/s00167-007-0411-z
   Fu Chongyang, 2011, Zhongguo Xiu Fu Chong Jian Wai Ke Za Zhi, V25, P1214
   HADDAD RJ, 1988, J HAND SURG-AM, V13A, P709, DOI 10.1016/S0363-5023(88)80130-8
   Haji A, 2004, FOOT ANKLE INT, V25, P215, DOI 10.1177/107110070402500404
   Hislop HJ, 1995, DANIELS WORTHINGTONS
   IKUTA Y, 1985, J HAND SURG-BRIT EUR, V10B, P67, DOI 10.1016/S0266-7681(85)80021-8
   Jarvinen Tero A H, 2005, Foot Ankle Clin, V10, P255, DOI 10.1016/j.fcl.2005.01.013
   Jiang N, 2012, INT ORTHOP, V36, P765, DOI 10.1007/s00264-011-1431-3
   Keating JF, 2011, J BONE JOINT SURG BR, V93B, P1071, DOI 10.1302/0301-620X.93B8.25998
   Khan RJK, 2010, COCHRANE DB SYST REV, DOI 10.1002/14651858.CD003674.pub4
   Khan RJK, 2005, J BONE JOINT SURG AM, V87A, P2202, DOI 10.2106/JBJS.D.03049
   KLEIN W, 1991, Chirurgia degli Organi di Movimento, V76, P223
   Lim J, 2001, FOOT ANKLE INT, V22, P559, DOI 10.1177/107110070102200705
   Maes Renaud, 2006, Acta Orthop Belg, V72, P179
   Maffulli N, 2003, AM J SPORT MED, V31, P692, DOI 10.1177/03635465030310051001
   Majewski M, 2006, AM J SPORT MED, V34, P793, DOI 10.1177/0363546505283266
   Maquirriain Javier, 2011, Yale Journal of Biology and Medicine, V84, P289
   Nilsson-Helander K, 2010, AM J SPORT MED, V38, P2186, DOI 10.1177/0363546510376052
   Petri M, 2012, ARCH ORTHOP TRAUM SU, V132, P649, DOI 10.1007/s00402-011-1452-9
   Saxena A, 2011, J FOOT ANKLE SURG, V50, P37, DOI 10.1053/j.jfas.2010.10.008
   Soroceanu A, 2012, J BONE JOINT SURG AM, V94A, P2136, DOI 10.2106/JBJS.K.00917
   Suchak AA, 2008, J BONE JOINT SURG AM, V90A, P1876, DOI 10.2106/JBJS.G.01242
   Suchak AA, 2006, CLIN ORTHOP RELAT R, P216, DOI 10.1097/01.blo.0000203458.05135.74
   TANG JB, 1994, J HAND SURG-BRIT EUR, V19B, P699, DOI 10.1016/0266-7681(94)90238-0
   Thevendran G, 2013, Musculoskelet Surg, V97, P9, DOI 10.1007/s12306-013-0251-6
   Tsuge K, 1977, J Hand Surg Am, V2, P436
   Uchiyama E, 2007, AM J SPORT MED, V35, P1739, DOI 10.1177/0363546507304492
   Veitch A, 2000, CLIN ORTHOP RELAT R, P228
   Willits K, 2010, J BONE JOINT SURG AM, V92A, P2767, DOI 10.2106/JBJS.I.01401
   Yildirim Yakup, 2006, Acta Orthop Traumatol Turc, V40, P164
   Yotsumoto T, 2010, AM J SPORT MED, V38, P287, DOI 10.1177/0363546509351557
NR 34
TC 6
Z9 8
U1 0
U2 3
PU ROYAL COLLEGE SURGEONS EDINBURGH
PI EDINBURGH
PA NICOLSON ST, EDINBURGH EH8 9DW, SCOTLAND
SN 1479-666X
J9 SURG-J R COLL SURG E
JI Surg. J. R. Coll. Surg. Edinb. Irel.
PD AUG
PY 2015
VL 13
IS 4
BP 207
EP 212
DI 10.1016/j.surge.2014.03.010
PG 6
WC Surgery
WE Science Citation Index Expanded (SCI-EXPANDED)
SC Surgery
GA CN9YK
UT WOS:000358806900005
PM 24784822
DA 2022-07-22
ER

PT J
AU Lacoste, S
   Feron, JM
   Cherrier, B
AF Lacoste, S.
   Feron, J. M.
   Cherrier, B.
TI Percutaneous Tenolig (R) repair under intra-operative ultrasonography
   guidance in acute Achilles tendon rupture
SO ORTHOPAEDICS & TRAUMATOLOGY-SURGERY & RESEARCH
LA English
DT Article
DE Achilles tendon rupture; Ultrasonography; Percutaneous treatment;
   Tenolig (R)
ID NONOPERATIVE TREATMENT; NONSURGICAL TREATMENT; SURGICAL REPAIR;
   TENORRAPHY
AB Background: Acute Achilles tendon rupture can be treated conservatively or surgically. Open surgery restores tendon continuity but carries a risk of skin complications. Tenolig (R) is a device designed for the percutaneous biological treatment of acute Achilles tendon rupture. Earlier studies found high rates of recurrent tears and nerve injury after Tenolig (R) repair. Hypothesis: We hypothesised that intra-operative ultrasonography during Tenolie repair would decrease the post-operative complication rate and improve functional outcomes.
   Materials and methods: We studied 75 consecutive patients with a mean age of 39.9 years. The injury was sports-related in 82.8% of cases. Mean distance from the calcaneal tendon attachment to the tear was 5 cm and mean time from injury to repair was 4.2 days. All patients underwent Tenolig (R) repair under ultrasound guidance followed by early rehabilitation therapy with partial weight bearing started after 3 weeks.
   Results: Mean follow-up was 20.7 months and no patient was lost to follow-up. A single patient (1.3%) experienced rerupture and none had permanent sural nerve damage. Mean time to sports resumption was 8.6 months, with two-thirds of patients returning to their previous level of sporting activities. The mean AOFAS functional score was 95 and the mean ATRS score was 91.3.
   Discussion: Our experience suggests that intra-operative ultrasonography, a non-invasive, widely available, and accurate tool, provided improved control of Tenoli:n suture position. Ultrasonography provided valuable guidance during this demanding procedure and allowed the very early initiation of rehabilitation therapy. Another crucial factor is patient education about the physical therapy programme. Attention to this point allowed us to obtain robust and reliable functional outcomes in a population predominantly composed of athletes. Level of evidence: Level IV. (C) 2014 Elsevier Masson SAS. All rights reserved.
C1 [Lacoste, S.; Feron, J. M.; Cherrier, B.] Hop Univ Est Parisien St Antoine, Serv Chirurg Orthoped & Traumatol B, F-75571 Paris, France.
   [Lacoste, S.; Feron, J. M.; Cherrier, B.] Univ Paris 04, UPMC, F-75230 Paris 05, France.
RP Lacoste, S (通讯作者)，Hop Univ Est Parisien St Antoine, Serv Chirurg Orthoped & Traumatol B, 184 Rue Faubourg St Antoine, F-75571 Paris, France.
EM lacoste.sophie@hotmail.fr
CR CETTI R, 1993, AM J SPORT MED, V21, P791, DOI 10.1177/036354659302100606
   DELPONTE P, 1992, REV CHIR ORTHOP, V78, P404
   Gigante A, 2008, KNEE SURG SPORT TR A, V16, P204, DOI 10.1007/s00167-007-0448-z
   Khan RJK, 2005, J BONE JOINT SURG AM, V87A, P2202, DOI 10.2106/JBJS.D.03049
   KITAOKA HB, 1994, FOOT ANKLE INT, V15, P349, DOI 10.1177/107110079401500701
   Lacoste S, 2012, EUR J ORTHOP SURG TR, V22, P585, DOI 10.1007/s00590-011-0867-x
   LEA RB, 1968, CLIN ORTHOP RELAT R, P115
   MA GWC, 1977, CLIN ORTHOP RELAT R, P247
   Maes Renaud, 2006, Acta Orthop Belg, V72, P179
   Maffulli N., 2007, ACHILLES TENDON
   Mertl P, 1999, REV CHIR ORTHOP, V85, P277
   Metz R, 2008, AM J SPORT MED, V36, P1688, DOI 10.1177/0363546508319312
   Nilsson-Helander K, 2007, AM J SPORT MED, V35, P421, DOI 10.1177/0363546506294856
   Olsson N, 2013, AM J SPORT MED, V41, P2867, DOI 10.1177/0363546513503282
   Soubeyrand M, 2010, FOOT ANKLE INT, V31, P1069, DOI 10.3113/FAI.2010.1069
   Suchak AA, 2008, J BONE JOINT SURG AM, V90A, P1876, DOI 10.2106/JBJS.G.01242
   Taglialavoro G, 2011, STRATEG TRAUMA LIMB, V6, P147, DOI 10.1007/s11751-011-0124-1
   Webb J, 2000, FOOT ANKLE INT, V21, P475, DOI 10.1177/107110070002100604
NR 18
TC 19
Z9 29
U1 0
U2 8
PU ELSEVIER MASSON
PI MILANO
PA VIA PALEOCAPA 7, 20121 MILANO, ITALY
SN 1877-0568
J9 ORTHOP TRAUMATOL-SUR
JI Orthop. Traumatol.-Surg. Res.
PD DEC
PY 2014
VL 100
IS 8
BP 925
EP 930
DI 10.1016/j.otsr.2014.09.018
PG 6
WC Orthopedics; Surgery
WE Science Citation Index Expanded (SCI-EXPANDED)
SC Orthopedics; Surgery
GA CB3TF
UT WOS:000349550900014
PM 25453930
OA Bronze
DA 2022-07-22
ER

PT J
AU She, GR
   Teng, Q
   Li, JR
   Zheng, XF
   Chen, L
   Hou, HG
AF She, Guorong
   Teng, Qiang
   Li, Jieruo
   Zheng, Xiaofei
   Chen, Lin
   Hou, Huige
TI Comparing Surgical and Conservative Treatment on Achilles Tendon
   Rupture: A Comprehensive Meta-Analysis of RCTs
SO FRONTIERS IN SURGERY
LA English
DT Article
DE Achilles tendon rupture; surgery; conservative; meta-analysis; clinical
   outcome
ID PROSPECTIVE RANDOMIZED-TRIAL; PRESSURE WOUND THERAPY; NONOPERATIVE
   TREATMENT; NONSURGICAL TREATMENT; OPEN RECONSTRUCTION; RISK; MANAGEMENT;
   REPAIR; REHABILITATION; THROMBOSIS
AB Background: The Achilles tendon is the strongest tendon in human and is frequently injured, mainly in the young to middle age active population. Increasing incidence of Achilles tendon rupture (ATR) is still reported in several studies. Surgical repair and conservative treatment are two major management strategies widely adopted in ATR patients, but the consensus of the optimal treatment strategy is still debated. We aimed at thoroughly reviewing the ATR topic with additional assessments and performed a most comprehensive meta-analysis of randomized controlled trials (RCTs).
   Method: We comprehensively searched PubMed, Embase, Cochrane, and and retrieved all RCTs comparing surgical and conservative treatment on ATR for further analysis. Two independent reviewers performed data extraction and random effect model was adopted when I-2 > 50%, with data presentation of risk ratio, risk difference, or mean difference and 95% confidence interval.
   Results: A total of 13 RCTs were included in this meta-analysis. A significant difference was observed in re-rupture, complication rate, adhesion to the underlying tendon, sural nerve injury, and superficial infection. A substantial reduction in re-rupture rate could be observed for surgical treatment while the complication rate was higher compared with conservative treatment.
   Conclusion: Surgical treatment revealed significance in reducing the re-rupture rate but was associated with a higher complication rate, while conservative treatment showed similar outcomes with a lower complication rate. Collectively, we recommend conservative treatment if patients' status and expectations are suitable, but surgeon and physician discretion is also crucial in decision making.
C1 [She, Guorong; Teng, Qiang; Li, Jieruo; Zheng, Xiaofei; Hou, Huige] Jinan Univ, Dept Sport Med, Affiliated Hosp 1, Guangzhou, Peoples R China.
   [Chen, Lin] Jinan Univ, Drug Clin Trial Inst, Affiliated Hosp 1, Guangzhou, Peoples R China.
RP Hou, HG (通讯作者)，Jinan Univ, Dept Sport Med, Affiliated Hosp 1, Guangzhou, Peoples R China.; Chen, L (通讯作者)，Jinan Univ, Drug Clin Trial Inst, Affiliated Hosp 1, Guangzhou, Peoples R China.
EM chenlinllu@163.com; drhouhuige@126.com
CR Ames PRJ, 2008, DISABIL REHABIL, V30, P1646, DOI 10.1080/09638280701785882
   Barnes GD, 2018, THROMB HAEMOSTASIS, V118, P1345, DOI 10.1055/s-0038-1667034
   Bhandari M, 2002, CLIN ORTHOP RELAT R, P190
   Calder JDF, 2016, KNEE SURG SPORT TR A, V24, P1409, DOI 10.1007/s00167-015-3976-y
   CETTI R, 1993, AM J SPORT MED, V21, P791, DOI 10.1177/036354659302100606
   Colegrave N, 2017, P ROY SOC B-BIOL SCI, V284, DOI 10.1098/rspb.2016.1850
   Costa ML, 2006, J BONE JOINT SURG BR, V88B, P69, DOI 10.1302/0301-620X.88B1.16549
   Deng SL, 2017, J FOOT ANKLE SURG, V56, P1236, DOI 10.1053/j.jfas.2017.05.036
   Domeij-Arverud E, 2015, BONE JOINT J, V97B, P675, DOI 10.1302/0301-620X.97B5.34581
   Egger AC, 2017, CURR REV MUSCULOSKE, V10, P72, DOI 10.1007/s12178-017-9386-7
   Fischer S, 2021, ARCH ORTHOP TRAUM SU, V141, P751, DOI 10.1007/s00402-020-03461-z
   Frankewycz B, 2017, ARCH ORTHOP TRAUM SU, V137, P333, DOI 10.1007/s00402-017-2627-9
   Ganestam A, 2016, KNEE SURG SPORT TR A, V24, P3730, DOI 10.1007/s00167-015-3544-5
   Grassi A, 2018, J BONE JOINT SURG AM, V100, P1969, DOI 10.2106/JBJS.17.01364
   Guorong SheQT., 2020, COMP SURG CONSERVATI, DOI [10.21203/rs.3.rs-62388/v1, DOI 10.21203/RS.3.RS-62388/V1]
   Higgins J, 2008, COCHRANE HDB SYSTEMA
   Higgins J, 2012, COCHRANE DB SYST REV, DOI 10.1002/14651858.ED000049
   Higgins Julian P T, 2011, BMJ, V343, pd5928, DOI 10.1136/bmj.d5928
   Higgins JPT, 2008, STAT MED, V27, P6072, DOI 10.1002/sim.3427
   Holm C, 2015, SCAND J MED SCI SPOR, V25, pE1, DOI 10.1111/sms.12209
   Hozo SP, 2005, BMC MED RES METHODOL, V5, P13, DOI 10.1186/1471-2288-5-13
   Keating JF, 2011, J BONE JOINT SURG BR, V93B, P1071, DOI 10.1302/0301-620X.93B8.25998
   Khan RJK, 2010, COCHRANE DB SYST REV, DOI 10.1002/14651858.CD003674.pub4
   Khan RJK, 2005, J BONE JOINT SURG AM, V87A, P2202, DOI 10.2106/JBJS.D.03049
   Lantto I, 2016, AM J SPORT MED, V44, P2406, DOI 10.1177/0363546516651060
   Liberati A, 2009, ANN INTERN MED, V151, pW65, DOI [10.1371/journal.pmed.1000100, 10.7326/0003-4819-151-4-200908180-00136, 10.1136/bmj.b2700]
   Lin LF, 2018, BIOMETRICS, V74, P785, DOI 10.1111/biom.12817
   Longo UG, 2009, AM J SPORT MED, V37, P1400, DOI 10.1177/0363546509332250
   Luo DH, 2018, STAT METHODS MED RES, V27, P1785, DOI 10.1177/0962280216669183
   Makhdom AM, 2013, J FOOT ANKLE SURG, V52, P584, DOI 10.1053/j.jfas.2013.03.001
   Makulavicius A, 2016, FOLIA MORPHOL, V75, P53, DOI 10.5603/FM.a2015.0067
   Manent A, 2019, J FOOT ANKLE SURG, V58, P1229, DOI 10.1053/j.jfas.2019.02.002
   Metz R, 2008, AM J SPORT MED, V36, P1688, DOI 10.1177/0363546508319312
   Moller M, 2001, J BONE JOINT SURG BR, V83B, P843
   Mosser P, 2015, J FOOT ANKLE SURG, V54, P2, DOI 10.1053/j.jfas.2014.09.040
   Nilsson-Helander K, 2007, AM J SPORT MED, V35, P421, DOI 10.1177/0363546506294856
   Nilsson-Helander K, 2010, AM J SPORT MED, V38, P2186, DOI 10.1177/0363546510376052
   NISTOR L, 1981, J BONE JOINT SURG AM, V63, P394, DOI 10.2106/00004623-198163030-00012
   OBRIEN T, 1984, J BONE JOINT SURG AM, V66A, P1099, DOI 10.2106/00004623-198466070-00018
   Ochen Y, 2019, BMJ-BRIT MED J, V364, DOI 10.1136/bmj.k5120
   Olsson N, 2013, AM J SPORT MED, V41, P2867, DOI 10.1177/0363546513503282
   Patel A, 2012, CLIN ORTHOP RELAT R, V470, P270, DOI 10.1007/s11999-011-2166-6
   Reda Y, 2020, FOOT ANKLE SURG, V26, P280, DOI 10.1016/j.fas.2019.03.010
   Saku I, 2017, INT J SURG CASE REP, V37, P106, DOI 10.1016/j.ijscr.2017.06.027
   Sode J, 2007, EUR J CLIN PHARMACOL, V63, P499, DOI 10.1007/s00228-007-0265-9
   Soroceanu A, 2012, J BONE JOINT SURG AM, V94A, P2136, DOI 10.2106/JBJS.K.00917
   Stehno-Bittel L, 1998, MED SCI SPORT EXER, V30, P788, DOI 10.1097/00005768-199806000-00002
   Stroup DF, 2000, JAMA-J AM MED ASSOC, V283, P2008, DOI 10.1001/jama.283.15.2008
   Suchak AA, 2006, CLIN ORTHOP RELAT R, P216, DOI 10.1097/01.blo.0000203458.05135.74
   Twaddle BC, 2007, AM J SPORT MED, V35, P2033, DOI 10.1177/0363546507307503
   Wan X, 2014, BMC MED RES METHODOL, V14, DOI 10.1186/1471-2288-14-135
   Willits K, 2010, J BONE JOINT SURG AM, V92A, P2767, DOI 10.2106/JBJS.I.01401
NR 52
TC 3
Z9 3
U1 5
U2 8
PU FRONTIERS MEDIA SA
PI LAUSANNE
PA AVENUE DU TRIBUNAL FEDERAL 34, LAUSANNE, CH-1015, SWITZERLAND
SN 2296-875X
J9 FRONT SURG
JI Front. Surg.
PD FEB 18
PY 2021
VL 8
AR 607743
DI 10.3389/fsurg.2021.607743
PG 11
WC Surgery
WE Science Citation Index Expanded (SCI-EXPANDED)
SC Surgery
GA QR5BD
UT WOS:000625230700001
PM 33681281
OA Green Published, gold, Green Submitted
DA 2022-07-22
ER

PT J
AU Farcic, TS
   Baldan, CS
   Machado, AFP
   Caffaro, LAM
   Masson, IFB
   Casarotto, RA
AF Farcic, Thiago Saikali
   Baldan, Cristiano Schiavinato
   Perez Machado, Aline Fernanda
   Massabki Caffaro, Leonardo Affonso
   Bordello Masson, Igor Fagioli
   Casarotto, Raquel Aparecida
TI Collagen Fibers in the Healing Process of Rat Achilles Tendon Rupture
   Using Different Times of Ultrasound Therapy
SO ADVANCES IN WOUND CARE
LA English
DT Article
DE birefringence; dosimetry; wound healing; Achilles tendon; ultrasonic
   therapy; tendon injuries
ID TISSUE-REPAIR; MORPHOLOGY; INTENSITY
AB Objective: The aim of this study was to evaluate the organization of collagen fibers in the healing process of rat Achilles tendon rupture using different times of ultrasound therapy (TUS).
   Approach: Forty Wistar rats were selected. Among these, 32 were submitted to total tenotomy of the calcaneous tendon and divided into 5 groups: control group (CG, n = 8), without tenotomy or any treatment; tenotomy group (n = 8), with tenotomy and without treatment; TUS groups-TUS3 (n = 8), TUS4 (n = 8), and TUS5 (n = 8)-submitted to tenotomy and treated with TUS for 3, 4, and 5 min per effective radiating area (ERA), respectively. The animals were sacrificed on the 12th postoperative day. The tendons were surgically removed for analysis of the collagen fiber organization using the birefringence technique (OR, optical retardation).
   Results: The collagen fibers exhibited better aggregation and organization in the UST3, TUS4, and TUS5 groups compared with CG (p < 0.05). The TUS5 group had better response rates in intergroup comparison.
   Innovation: The dose response of therapeutic TUS is influenced by many variables. The scientific evidence to support the dosimetry is insufficient. The application time is an important variable to be considered in TUS. In this study, the longer the application time, the better for organization and aggregation of collagen fibers in the rat tendon.
   Conclusion: TUS applied for 5min per ERA presented higher dose response to the organization of collagen fibers in the healing process of rat Achilles tendon rupture.
C1 [Farcic, Thiago Saikali; Baldan, Cristiano Schiavinato; Massabki Caffaro, Leonardo Affonso; Casarotto, Raquel Aparecida] Univ Sao Paulo FMUSP, Fac Med, Dept Phys Therapy, Sao Paulo, SP, Brazil.
   [Farcic, Thiago Saikali; Baldan, Cristiano Schiavinato; Perez Machado, Aline Fernanda; Bordello Masson, Igor Fagioli] Univ Paulista UNIP, Dept Phys Therapy, Sao Paulo, SP, Brazil.
   [Perez Machado, Aline Fernanda] Univ Cidade Sao Paulo UNICID, Masters & Doctoral Programs Phys Therapy, Sao Paulo, SP, Brazil.
RP Farcic, TS (通讯作者)，Univ Paulista UNIP, Dept Phys Therapy, Sao Paulo, SP, Brazil.; Farcic, TS (通讯作者)，Yojiro Takaoka Ave 3500, BR-06500000 Santana De Parnaiba, SP, Brazil.
EM thiagofarcic@hotmail.com
RI Machado, Aline Fernanda Perez/B-9036-2015; Casarotto, Raquel
   Aparecida/F-5582-2012
OI Caffaro, Leonardo/0000-0002-5004-9780; Casarotto, Raquel
   Aparecida/0000-0003-2852-2401
CR Aiyegbusi AI, 2012, CONNECT TISSUE RES, V53, P478, DOI 10.3109/03008207.2012.690793
   Alexander LD, 2010, PHYS THER, V90, P14, DOI 10.2522/ptj.20080272
   Ansell DM, 2012, EXP DERMATOL, V21, P581, DOI 10.1111/j.1600-0625.2012.01540.x
   Bertolini Gladson Ricardo Flor, 2012, Fisioter. Pesqui., V19, P242
   Carrinho PM, 2006, PHOTOMED LASER SURG, V24, P754, DOI 10.1089/pho.2006.24.754
   Casarotto RA, 2004, ARCH PHYS MED REHAB, V85, P162, DOI 10.1016/S0003-9993(03)00293-4
   Culav EM, 1999, PHYS THER, V79, P308, DOI 10.1093/ptj/79.3.308
   da Cunha A, 2001, ULTRASOUND MED BIOL, V27, P1691, DOI 10.1016/S0301-5629(01)00477-X
   Draper DO, 2014, EUR J PHYS REHAB MED, V50, P209
   Eckelman WC, 2007, NUCL MED BIOL, V34, P229, DOI 10.1016/j.nucmedbio.2007.01.005
   Enwemeka CS, 2000, LASER THER, V12, P22, DOI DOI 10.5978/ISLSM.12.22
   Farcic TS, 2013, BRAZ J PHYS THER, V17, P263, DOI 10.1590/S1413-35552012005000090
   FYFE MC, 1980, ULTRASOUND MED BIOL, V6, P107, DOI 10.1016/0301-5629(80)90038-1
   FYFE MC, 1984, BRIT J EXP PATHOL, V65, P671
   Hess Gregory William, 2010, Foot Ankle Spec, V3, P29, DOI 10.1177/1938640009355191
   Hoogland R, 1986, MANUAL TERAPIA ULTRA
   Jeremias SL, 2011, J ORTHOP SPORT PHYS, V41, P526, DOI 10.2519/jospt.2011.3468
   Kim U, 2017, INJURY, V48, P1710, DOI 10.1016/j.injury.2017.04.050
   Kitchen S, 2009, ELECTROTHERAPY EVIDE, P47
   Koeke PU, 2005, ULTRASOUND MED BIOL, V31, P345, DOI 10.1016/j.ultrasmedbio.2004.12.005
   Maffulli N, 2000, AM J SPORT MED, V28, P499, DOI 10.1177/03635465000280040901
   Maia ALM, 2010, ULTRASOUND MED BIOL, V36, P1682, DOI 10.1016/j.ultrasmedbio.2010.06.012
   Moura MD, 2014, LASER MED SCI, V29, P301, DOI 10.1007/s10103-013-1327-0
   Ng COY, 2003, ULTRASOUND MED BIOL, V29, P1501, DOI 10.1016/S0301-5629(03)01018-4
   Ng GYF, 2007, ULTRASOUND MED BIOL, V33, P1750, DOI 10.1016/j.ultrasmedbio.2007.05.019
   Ng GYF, 2011, CONNECT TISSUE RES, V52, P178, DOI 10.3109/03008207.2010.500752
   Oakley E M, 1978, Physiotherapy, V64, P169
   Prentice W., 2011, THERAPEUTIC MODALITI, V4, P433
   Reuter P, 2010, ULTRASOUND MED BIOL, V36, P1188, DOI 10.1016/j.ultrasmedbio.2010.04.013
   Robertson VJ, 2002, PHYS THER SPORT, V3, P124, DOI 10.1054/ptsp.2002.0107
   Tsai WC, 2011, AM J PHYS MED REHAB, V90, P1068, DOI 10.1097/PHM.0b013e31821a70be
   Watson T, 2008, ULTRASONICS, V48, P321, DOI 10.1016/j.ultras.2008.02.004
   Wood VT, 2010, LASER SURG MED, V42, P559, DOI 10.1002/lsm.20932
NR 33
TC 1
Z9 1
U1 0
U2 11
PU MARY ANN LIEBERT, INC
PI NEW ROCHELLE
PA 140 HUGUENOT STREET, 3RD FL, NEW ROCHELLE, NY 10801 USA
SN 2162-1918
EI 2162-1934
J9 ADV WOUND CARE
JI Adv. Wound Care
PD APR
PY 2018
VL 7
IS 4
BP 114
EP 120
DI 10.1089/wound.2017.0748
PG 7
WC Dermatology
WE Science Citation Index Expanded (SCI-EXPANDED)
SC Dermatology
GA GB6RV
UT WOS:000429201200002
PM 29696098
OA Green Published
DA 2022-07-22
ER

PT J
AU Yuksel, S
   Gulec, MA
   Gultekin, MZ
   Adanir, O
   Caglar, A
   Beytemur, O
   Kucukyildirim, BO
   Avci, A
   Subasi, C
   Inci, C
   Karaoz, E
AF Yuksel, Serdar
   Gulec, M. Akif
   Gultekin, M. Zeki
   Adanir, Oktay
   Caglar, Aysel
   Beytemur, Ozan
   Kucukyildirim, B. Onur
   Avci, Ali
   Subasi, Cansu
   Inci, Cigdem
   Karaoz, Erdal
TI Comparison of the early period effects of bone marrow-derived
   mesenchymal stem cells and platelet-rich plasma on the Achilles tendon
   ruptures in rats
SO CONNECTIVE TISSUE RESEARCH
LA English
DT Article
DE Achilles tendon rupture; bone marrow cells; mesenchymal stem cells;
   platelet-rich plasma; rats
ID ENDOTHELIAL GROWTH-FACTOR; REPAIR; ANGIOGENESIS; THERAPY; STIMULATION;
   INJECTION; INJURIES; IMPROVE; MODEL; PRP
AB Introduction: This study aims to histopathologically, biomechanically, and immunohistochemically compare the fourth-week efficiencies of local platelet-rich plasma (PRP) and bone marrow-derived mesenchymal stem cell (rBM-MSC) treatments of the Achilles tendon ruptures created surgically in rats. Materials and Methods: The study included 35 12-month-old male Sprague Dawley rats, with an average weight of 400-500 g. Five rats were used as donors for MSC and PRP, and 30 rats were separated into MSC, PRP, and control groups (n = 10). The Achilles tendons of the rats were cut transversely, the MSC from bone marrow was administered to the MSC group, the PRP group received PRP, and the control group received physiological saline to create the same surgical effect. In previous studies, it was shown that this physiological saline does not have any effect on tendon recovery. Thirty days after the treatment, the rats were sacrificed and their Achilles tendons were examined histopathologically, immunohistochemically, and biomechanically. Results: The use of rBM-MSC and PRP in the Achilles tendon ruptures when the tendon is in its weakest phase positively affected the recovery of the tendon in histopathologic, immunohistochemical, and biomechanical manners compared to the control group (p < 0.05). While the levels of pro-inflammatory cytokines TNF-, IFN, and IL 1 were significantly low, the levels of anti-inflammatory cytokines and growth factors playing key roles in tendon recovery, such as IL2, VEGF, transforming growth factor-beta, and HGF, were significantly higher in the MSC group than those of the PRP and control groups (p < 0.05). In the MSC group, the Delta IFmax (mm) value was significantly higher (p ? 0.05) than that in the PRP and control groups. Conclusion: rBM-MSC and PRP promote the recovery of the tendon and increase its structural strength. The use of PRP and MSC provides hope for the treatment of the Achilles tendon ruptures that limit human beings' functionalities and quality of life, particularly for athletes. It is thought that the use of MSC can be more effective for tendon healing; hence, more extensive and advanced studies are needed on this topic.
C1 [Yuksel, Serdar] Trabzon Kanuni Training & Res Hosp, Dept Orthopaed & Traumatol, Trabzon, Turkey.
   [Gulec, M. Akif; Gultekin, M. Zeki; Adanir, Oktay; Beytemur, Ozan] Bagcilar Training & Res Hosp, Dept Orthopaed & Traumatol, Istanbul, Turkey.
   [Caglar, Aysel] Bagcilar Training & Res Hosp, Dept Pathol, Istanbul, Turkey.
   [Kucukyildirim, B. Onur; Avci, Ali] Yildiz Tech Univ, Dept Mech Engn, Istanbul, Turkey.
   [Subasi, Cansu; Inci, Cigdem; Karaoz, Erdal] Ctr Regenerat Med & Stem Cell Res & Mfg LivMedCel, Istanbul, Turkey.
RP Yuksel, S (通讯作者)，Trabzon Kanuni Training & Res Hosp, Dept Orthopaed & Traumatol, Trabzon, Turkey.
EM Serdar84yuksel@gmail.com
RI Avci, Ali/AAI-3086-2021; Kucukyildirim, Bedri Onur/AAZ-6726-2020;
   Adanır, Oktay/AAT-7040-2020; Güleç, Akif/AAU-4848-2020; Karaoz,
   Erdal/AAO-1470-2020; beytemür, ozan/AAU-7362-2020; İnci Aydemir,
   Çiğdem/ABB-5706-2021
OI Avci, Ali/0000-0003-3901-6248; Akdogan Eker,
   Aysegul/0000-0003-0212-9230; Inci Aydemir, Cigdem/0000-0001-6814-0585
FU Bagcilar Training and Research Hospital Management, Bagcilar Training
   and Research Hospital Experimental Research and Skill Development Center
   (BADABEM)
FX For their contribution and support, we would like to thank Bagcilar
   Training and Research Hospital Management, Bagcilar Training and
   Research Hospital Experimental Research and Skill Development Center
   (BADABEM), and also for their help during the biomechanical laboratory
   studies to Yildiz Technical University, Mechanical Engineering
   Department member Prof. Dr. Aysegul Akdogan Eker.
CR Adas G, 2011, LANGENBECK ARCH SURG, V396, P115, DOI 10.1007/s00423-010-0717-z
   Anitua E, 2005, J ORTHOP RES, V23, P281, DOI 10.1016/j.orthres.2004.08.015
   [Anonymous], 2009, E909 ASTM INT
   Aspenberg P, 2004, ACTA ORTHOP SCAND, V75, P93, DOI 10.1080/00016470410001708190
   Aspenberg P, 2007, INT ORTHOP, V31, P783, DOI 10.1007/s00264-007-0398-6
   Awad HA, 2003, J ORTHOP RES, V21, P420, DOI 10.1016/S0736-0266(02)00163-8
   Babbush Charles A, 2003, Implant Dent, V12, P24
   BESKIN JL, 1987, AM J SPORT MED, V15, P1, DOI 10.1177/036354658701500101
   Bolt P, 2007, J BONE JOINT SURG AM, V89A, P1315, DOI 10.2106/JBJS.F.00257
   Carmona JU, 2013, REC PAT REG MED, V3, P148, DOI DOI 10.2174/2210296511303020005
   Chiodo CP, 2006, FOOT ANKLE INT, V27, P305, DOI 10.1177/107110070602700415
   Chong AKS, 2007, J BONE JOINT SURG AM, V89A, P74, DOI 10.2106/JBJS.E.01396
   Creaney L, 2008, BRIT J SPORT MED, V42, DOI 10.1136/bjsm.2007.040071
   Ehrenfest DMD, 2012, CURR PHARM BIOTECHNO, V13, P1131
   Folkman J, 2007, NAT REV DRUG DISCOV, V6, P273, DOI 10.1038/nrd2115
   Forslund C, 2003, ACTA ORTHOP SCAND, V74, P465, DOI 10.1080/00016470310017802
   Freymiller EG, 2004, J ORAL MAXIL SURG, V62, P1046, DOI 10.1016/j.joms.2004.05.205
   Fu SC, 2005, CLIN ORTHOP RELAT R, P226, DOI 10.1097/01.blo.0000145887.48534.6f
   Gigante A, 2008, KNEE SURG SPORT TR A, V16, P204, DOI 10.1007/s00167-007-0448-z
   Hung SC, 2007, STEM CELLS, V25, P2363, DOI 10.1634/stemcells.2006-0686
   Ichim TE, 2010, CELL IMMUNOL, V260, P75, DOI 10.1016/j.cellimm.2009.10.006
   JOZSA L, 1989, AM J SPORT MED, V17, P338, DOI 10.1177/036354658901700305
   Karaoz E, 2010, CELL DEATH DIS, V1, DOI 10.1038/cddis.2010.14
   Karaoz E, 2009, HISTOCHEM CELL BIOL, V132, P533, DOI 10.1007/s00418-009-0629-6
   Kaux JF, 2012, WOUND REPAIR REGEN, V20, P748, DOI 10.1111/j.1524-475X.2012.00826.x
   Kilic Emine, 2007, Acta Orthop Traumatol Turc, V41, P295
   KRACKOW KA, 1986, J BONE JOINT SURG AM, V68A, P764, DOI 10.2106/00004623-198668050-00020
   Maffulli N, 2008, CLIN ORTHOP RELAT R, V466, P1605, DOI 10.1007/s11999-008-0261-0
   Mahler F, 1992, Br J Sports Med, V26, P7
   Majewski M, 2009, AM J SPORT MED, V37, P2117, DOI 10.1177/0363546509348047
   Moller M, 2001, J BONE JOINT SURG BR, V83B, P843
   Molloy T, 2003, SPORTS MED, V33, P381, DOI 10.2165/00007256-200333050-00004
   Morizaki Y, 2010, J HAND SURG-AM, V35A, P1833, DOI 10.1016/j.jhsa.2010.07.034
   Murray MM, 2009, J ORTHOP RES, V27, P639, DOI 10.1002/jor.20796
   NILLIUS SA, 1976, ACTA ORTHOP SCAND, V47, P118, DOI 10.3109/17453677608998983
   Okamoto N, 2010, J BONE JOINT SURG AM, V92A, P2776, DOI 10.2106/JBJS.I.01325
   Pinedo HM, 1998, LANCET, V352, P1775, DOI 10.1016/S0140-6736(98)05095-8
   Pittenger MF, 1999, SCIENCE, V284, P143, DOI 10.1126/science.284.5411.143
   Prockop DJ, 1997, SCIENCE, V276, P71, DOI 10.1126/science.276.5309.71
   Rozman P, 2007, ACTA DERMATOVEN ALP, V16, P156
   Sanchez M, 2007, AM J SPORT MED, V35, P245, DOI 10.1177/0363546506294078
   Selek O, 2014, ACTA ORTHOP TRAUMATO, V48, P187, DOI 10.3944/AOTT.2014.2985
   Suchak AA, 2005, FOOT ANKLE INT, V26, P932, DOI 10.1177/107110070502601106
   Taylor DW, 2011, CLIN J SPORT MED, V21, P344, DOI 10.1097/JSM.0b013e31821d0f65
   Virchenko O, 2006, ACTA ORTHOP, V77, P806, DOI 10.1080/17453670610013033
   Wang QW, 2005, J BIOSCI BIOENG, V100, P418, DOI 10.1263/jbb.100.418
   Wang XJ, 2008, BIOCHEM BIOPH RES CO, V365, P548, DOI 10.1016/j.bbrc.2007.11.014
   Willits K, 2010, J BONE JOINT SURG AM, V92A, P2767, DOI 10.2106/JBJS.I.01401
   Xu YX, 2008, MED HYPOTHESES, V71, P390, DOI 10.1016/j.mehy.2008.03.046
   Zhang F, 2003, PLAST RECONSTR SURG, V112, P1613, DOI 10.1097/01.PRS.0000086772.72535.A4
NR 50
TC 19
Z9 20
U1 0
U2 14
PU TAYLOR & FRANCIS INC
PI PHILADELPHIA
PA 530 WALNUT STREET, STE 850, PHILADELPHIA, PA 19106 USA
SN 0300-8207
EI 1607-8438
J9 CONNECT TISSUE RES
JI Connect. Tissue Res.
PD SEP
PY 2016
VL 57
IS 5
BP 360
EP 373
DI 10.1080/03008207.2016.1189909
PG 14
WC Cell Biology; Orthopedics
WE Science Citation Index Expanded (SCI-EXPANDED)
SC Cell Biology; Orthopedics
GA DW2AF
UT WOS:000383444600004
PM 27191749
DA 2022-07-22
ER

PT J
AU Nyyssonen, T
   Luthje, P
AF Nyyssonen, T
   Luthje, P
TI Achilles tendon ruptures in South-East Finland between 1986-1996, with
   special reference to epidemiology, complications of surgery and hospital
   costs
SO ANNALES CHIRURGIAE ET GYNAECOLOGIAE
LA English
DT Article
DE achilles tendon; rupture; incidence; complications; costs
AB Background and Aims: The incidence of achilles tendon (AT) ruptures is increasing. The aim of the present study was to evaluate annual incidence, aetiology, operative complications and direct hospital costs of AT ruptures.
   Material and Methods: A retrospective study of 93 consecutive patients operated on for AT rupture from January 1986 to December 1996 at Kuusankoski District Hospital (area with 92 500 inhabitants) was performed. During the observation period no patient with an AT rupture was treated conservatively.
   Results: 95 AT ruptures were treated including one rerupture (1 %) and one patient with two ruptures. There were 7 (7 %) patients with an open AT rupture. The total annual incidence in the hospital area was 8.6 (+/- 4.3) and for closed AT ruptures 8.0 (+/- 3.8). The total incidence was 9.3 (+/- 4.6) /10(5) and for closed AT ruptures 8.6 (+/- 4.1) /10(5) inhabitants per year. Most of the injuries were sport related, the most frequent sport being volleyball. Patients operated for closed AT rupture had major surgical complications in 4.5 % of the cases and the total, complication rate was 11 %. The average direct hospital costs per patient was USD 1375.
   Conclusions: The incidence of AT ruptures is increasing in South-East Finland. The rate of major surgical complication was low (4.5 %) and comparable with earlier studies.
C1 Kuusankoski Dist Hosp, Dept Surg, FIN-45750 Kuusankoski, Finland.
RP Nyyssonen, T (通讯作者)，Kuusankoski Dist Hosp, Dept Surg, FIN-45750 Kuusankoski, Finland.
EM timo.nyyssonen@tiny.pp.fi
CR BARFRED T, 1973, ACTA ORTHOP SCAND, P1
   CARDEN DG, 1987, J BONE JOINT SURG BR, V69, P416, DOI 10.1302/0301-620X.69B3.3294839
   CETTI R, 1994, CLIN ORTHOP RELAT R, P155
   CETTI R, 1993, AM J SPORT MED, V21, P791, DOI 10.1177/036354659302100606
   HATTRUP SJ, 1985, FOOT ANKLE, V6, P34, DOI 10.1177/107110078500600107
   HOOKER CH, 1963, J BONE JOINT SURG BR, V45, P360, DOI 10.1302/0301-620X.45B2.360
   INGLIS AE, 1981, CLIN ORTHOP RELAT R, V156, P160
   JOZSA L, 1989, AM J SPORT MED, V17, P338, DOI 10.1177/036354658901700305
   JOZSA LG, 1997, HUMAN TENDONS ANATOM, P254
   KRUGERFRANKE M, 1993, UNFALLCHIRURG, V96, P524
   LEPPILAHTI, 1996, THESIS OULU U FINLAN
   Leppilahti J, 1998, SPORTS MED, V25, P79, DOI 10.2165/00007256-199825020-00002
   Leppilahti J, 1996, ACTA ORTHOP SCAND, V67, P277, DOI 10.3109/17453679608994688
   Levi N, 1997, INJURY, V28, P311, DOI 10.1016/S0020-1383(96)00200-8
   Moller A, 1996, ACTA ORTHOP SCAND, V67, P479, DOI 10.3109/17453679608996672
   NILLIUS SA, 1976, ACTA ORTHOP SCAND, V47, P118, DOI 10.3109/17453677608998983
   NISTOR L, 1981, J BONE JOINT SURG AM, V63, P394, DOI 10.2106/00004623-198163030-00012
   RANTANEN J, 1993, ACTA ORTHOP SCAND, V64, P333, DOI 10.3109/17453679308993638
   WAHLBY L, 1978, THESIS U UMEA SWEDEN
   WILLS CA, 1986, CLIN ORTHOP RELAT R, V207, P156
   WINTER E, 1995, UNFALLCHIRURG, V98, P468
   ZOLLINGER H, 1983, SPORTVERLETZ SPORTSC, P75
   ZWIPP H, 1989, UNFALLCHIRURG, V92, P554
NR 23
TC 19
Z9 20
U1 0
U2 3
PU FINNISH SURGICAL SOC
PI HELSINKI
PA MAKELANKATU 2 C, PO BOX 49, SF-00501 HELSINKI, FINLAND
SN 0355-9521
J9 ANN CHIR GYNAECOL
JI Ann. Chir. Gynaecol.
PY 2000
VL 89
IS 1
BP 53
EP 57
PG 5
WC Obstetrics & Gynecology; Surgery
WE Science Citation Index Expanded (SCI-EXPANDED)
SC Obstetrics & Gynecology; Surgery
GA 305UM
UT WOS:000086559000011
PM 10791646
DA 2022-07-22
ER

PT J
AU Olsson, N
   Nilsson-Helander, K
   Karlsson, J
   Eriksson, BI
   Thomee, R
   Faxen, E
   Silbernagel, KG
AF Olsson, Nicklas
   Nilsson-Helander, Katarina
   Karlsson, Jon
   Eriksson, Bengt I.
   Thomee, Roland
   Faxen, Eva
   Silbernagel, Karin Graevare
TI Major functional deficits persist 2 years after acute Achilles tendon
   rupture
SO KNEE SURGERY SPORTS TRAUMATOLOGY ARTHROSCOPY
LA English
DT Article
DE Achilles tendon rupture; Long-term results; Functional tests; Heel-rise
   work; Patient-reported outcome; ATRS
ID NONOPERATIVE TREATMENT; SURGICAL REPAIR; WEIGHTBEARING; METAANALYSIS;
   MULTICENTER; MANAGEMENT; SEPARATION; ENDURANCE; RECOVERY; SURGERY
AB The purpose of this prospective randomized controlled study was to evaluate the long-term results after an acute Achilles tendon rupture in patients treated surgically or non-surgically. The focus was to evaluate whether any improvements occurred between the one and 2-year evaluation.
   Eighty-one patients (67 men, 14 women) with a mean (SD) age of 42 (9.1) were included in this study. Forty-two patients were treated surgically, and 39 treated non-surgically otherwise the treatment was identical for the two groups. All patients were evaluated using the Achilles tendon Total Rupture Score (ATRS), the Physical Activity Scale (PAS) and validated functional tests one and 2 years after injury.
   There were significant functional deficits on the injured side compared with the contralateral side 2 years after Achilles tendon rupture, regardless of treatment. Only minor improvements, even though statistically significant, occurred between the 1- and 2-year evaluations. The physical activity level remained significantly reduced as compared with prior to injury, but the ATRS mean was relatively high in both groups (89 and 90).
   This long-term follow-up indicates that the majority of patients with an Achilles tendon rupture have not fully recovered (in regards to symptoms, physical activity level and function) 2 years after injury regardless of surgical or non-surgical treatment. Furthermore, only minor improvements occur between the 1- and 2-year evaluations. This indicates that to enhance the final outcome the focus should be on improvements in treatment within the first year. The patients appear to have adjusted to their impairments since the patient-reported outcome is relatively high in spite of functional deficits and lower activity level compared with pre-injury.
   Prospective randomized study, Level I.
C1 [Olsson, Nicklas; Karlsson, Jon; Eriksson, Bengt I.; Thomee, Roland; Faxen, Eva] Univ Gothenburg, Sahlgrenska Univ Hosp, Inst Clin Sci, Dept Orthopaed,Sahlgrenska Acad, SE-43180 Molndal, Sweden.
   [Nilsson-Helander, Katarina] Kungsbacka Hosp, Dept Orthoped, Kungsbacka, Sweden.
   [Silbernagel, Karin Graevare] Univ Delaware, Dept Mech Engn, Biomed Engn Res Ctr, Newark, DE 19716 USA.
RP Olsson, N (通讯作者)，Univ Gothenburg, Sahlgrenska Univ Hosp, Inst Clin Sci, Dept Orthopaed,Sahlgrenska Acad, SE-43180 Molndal, Sweden.
EM nicklas.olsson@gu.se
RI Helander, Katarina Nilsson/AAE-1793-2020
OI Helander, Katarina Nilsson/0000-0002-1292-5102; Gravare Silbernagel,
   Karin/0000-0001-7566-407X
FU Swedish National Centre for Research and Sports
FX The authors thank Lotta Falkheden-Henning and Annelie Brorsson for their
   help treating and evaluating the patients. We would also thank Toran
   MacLeod for manuscript review. This study was supported by the Swedish
   National Centre for Research and Sports (CIF).
CR BARBER SD, 1990, CLIN ORTHOP RELAT R, P204
   Bhandari M, 2002, CLIN ORTHOP RELAT R, P190
   Bostick GP, 2010, J ORTHOP SPORT PHYS, V40, P345, DOI 10.2519/jospt.2010.3204
   Fitzgerald GK, 2000, KNEE SURG SPORT TR A, V8, P76, DOI 10.1007/s001670050190
   GRIMBY G, 1986, ACTA MED SCAND, P233
   Gustavsson A, 2006, KNEE SURG SPORT TR A, V14, P778, DOI 10.1007/s00167-006-0045-6
   Kangas J, 2003, J TRAUMA, V54, P1171, DOI 10.1097/01.TA.0000047945.20863.A2
   Kangas J, 2007, AM J SPORT MED, V35, P59, DOI 10.1177/0363546506293255
   Kerkhoffs GMMJ, 2002, ARCH ORTHOP TRAUM SU, V122, P102, DOI 10.1007/s004020100312
   Khan RJK, 2005, J BONE JOINT SURG AM, V87A, P2202, DOI 10.2106/JBJS.D.03049
   Kocher MS, 2002, AM J SPORT MED, V30, P783, DOI 10.1177/03635465020300060501
   Maffulli N, 2003, AM J SPORT MED, V31, P692, DOI 10.1177/03635465030310051001
   Metz R, 2008, AM J SPORT MED, V36, P1688, DOI 10.1177/0363546508319312
   MIZNER RL, 2010, J ARTHROPLASTY
   Moller M, 2001, J BONE JOINT SURG BR, V83B, P843
   Moller M, 2002, SCAND J MED SCI SPOR, V12, P9, DOI 10.1034/j.1600-0838.2002.120103.x
   Mortensen NHM, 1999, J BONE JOINT SURG AM, V81A, P983, DOI 10.2106/00004623-199907000-00011
   MORTENSEN NHM, 1992, ORTHOPEDICS, V15, P899
   Neumayer F, 2010, ARCH ORTHOP TRAUM SU, V130, P363, DOI 10.1007/s00402-009-0865-1
   Nilsson-Helander K, 2008, KNEE SURG SPORT TR A, V16, P614, DOI 10.1007/s00167-008-0492-3
   Nilsson-Helander K, 2007, AM J SPORT MED, V35, P421, DOI 10.1177/0363546506294856
   Nilsson-Helander K, 2010, AM J SPORT MED, V38, P2186, DOI 10.1177/0363546510376052
   NISTOR L, 1981, J BONE JOINT SURG AM, V63, P394, DOI 10.2106/00004623-198163030-00012
   NYSTROM B, 1983, ACTA ORTHOP SCAND, V54, P620, DOI 10.3109/17453678308992899
   Parent E, 2002, ARCH PHYS MED REHAB, V83, P70, DOI 10.1053/apmr.2002.27337
   SALTIN B, 1968, CIRCULATION, V38, P1104, DOI 10.1161/01.CIR.38.6.1104
   Schepull T, 2007, BMC MUSCULOSKEL DIS, V8, DOI 10.1186/1471-2474-8-116
   Silbernagel KG, 2006, KNEE SURG SPORT TR A, V14, P1207, DOI 10.1007/s00167-006-0150-6
   Silbernagel KG, 2010, KNEE SURG SPORT TR A, V18, P258, DOI 10.1007/s00167-009-0889-7
   Suchak AA, 2008, J BONE JOINT SURG AM, V90A, P1876, DOI 10.2106/JBJS.G.01242
   Twaddle BC, 2007, AM J SPORT MED, V35, P2033, DOI 10.1177/0363546507307503
   Weber M, 2003, AM J SPORT MED, V31, P685, DOI 10.1177/03635465030310050901
   Willits K, 2010, J BONE JOINT SURG AM, V92A, P2767, DOI 10.2106/JBJS.I.01401
   Wong J, 2002, AM J SPORT MED, V30, P565, DOI 10.1177/03635465020300041701
NR 34
TC 141
Z9 143
U1 1
U2 16
PU SPRINGER
PI NEW YORK
PA 233 SPRING ST, NEW YORK, NY 10013 USA
SN 0942-2056
EI 1433-7347
J9 KNEE SURG SPORT TR A
JI Knee Surg. Sports Traumatol. Arthrosc.
PD AUG
PY 2011
VL 19
IS 8
BP 1385
EP 1393
DI 10.1007/s00167-011-1511-3
PG 9
WC Orthopedics; Sport Sciences; Surgery
WE Science Citation Index Expanded (SCI-EXPANDED)
SC Orthopedics; Sport Sciences; Surgery
GA 793JM
UT WOS:000292817600026
PM 21533539
DA 2022-07-22
ER

PT J
AU Maffulli, N
   Gougoulias, N
   Maffulli, GD
   Oliva, F
   Migliorini, F
AF Maffulli, Nicola
   Gougoulias, Nikolaos
   Maffulli, Gayle D.
   Oliva, Francesco
   Migliorini, Filippo
TI Slowed-Down Rehabilitation Following Percutaneous Repair of Achilles
   Tendon Rupture
SO FOOT & ANKLE INTERNATIONAL
LA English
DT Article
DE Achilles tendon rupture; surgery; rehabilitation; slowed down
ID GROWTH-FACTORS; RESTING ANGLE; SURGICAL-MANAGEMENT; ANIMAL-MODELS;
   STEM-CELLS; LENGTH; ELONGATION; EXPRESSION; INJURIES; DEFICITS
AB Background: Following percutaneous repair of acute Achilles tendon (AT) ruptures, early postoperative weightbearing is advocated; however, it is debatable how aggressive rehabilitation should be. We compared the clinical and functional outcomes in 2 groups of patients who followed either our "traditional" or a "slowed down" rehabilitation after percutaneous surgical repair. Methods: Sixty patients were prospectively recruited to a slowed down (29 patients) or a traditional (31 patients) rehabilitation program. Both groups were allowed immediate weightbearing postoperatively; a removable brace with 5 heel wedges was applied at 2 weeks. In the slowed-down group, 1 wedge was removed after 4 weeks. Gradual removal of the boot took place after 4 wedges were kept for 4 weeks. In the traditional group, 1 wedge was removed every 2 weeks, with removal of the boot after 2 wedges had been kept for 2 weeks. The AT Resting Angle (ATRA) evaluated tendon elongation. Patient reported functional outcomes were assessed using the AT Rupture Score (ATRS). Calf circumference difference and the isometric plantarflexion strength of the gastro-soleus complex were evaluated. Results: At the 12-month follow-up, both ATRA and ATRS were more favorable in the slowed-down group. The isometric strength and the calf circumference were more similar to the contralateral leg in the slowed-down group than in the traditional one. Conclusion: Following percutaneous repair of acute Achilles tendon patients undergoing slowed down rehabilitation performed better than the traditional one. These conclusions must be considered within the limitations of the present study.
C1 [Maffulli, Nicola; Oliva, Francesco] Univ Salerno, Dept Med Surg & Dent, Baronissi, SA, Italy.
   [Maffulli, Nicola] Keele Univ, Sch Pharm & Bioengn, Sch Med, Thornburrow Dr, Stoke On Trent, Staffs, England.
   [Maffulli, Nicola] Queen Mary Univ London, Mile End Hosp, Ctr Sports & Exercise Med, Barts & London Sch Med & Dent, London, England.
   [Gougoulias, Nikolaos] Gen Hosp Katerini, Katerini, Greece.
   [Gougoulias, Nikolaos] Frimley Pk Hosp, Frimley, Surrey, England.
   [Maffulli, Gayle D.] Wholelife Clin, London, England.
   [Migliorini, Filippo] RWTH Aachen Univ Clin, Univ Clin Aachen, Dept Orthopaed, Aachen, Germany.
RP Maffulli, N (通讯作者)，Queen Mary Univ London, Mile End Hosp, 275 Bancroft Rd, London E1 4DG, England.
EM n.maffulli@qmul.ac.uk
RI migliorini, filippo/AAZ-9529-2021; Maffulli, Nicola/A-1839-2014
OI migliorini, filippo/0000-0001-7220-1221; Gougoulias,
   Nikolaos/0000-0001-8455-0007; Maffulli, Nicola/0000-0002-5327-3702
CR Ahmad Z, 2012, ARTHROSCOPY, V28, P1018, DOI 10.1016/j.arthro.2011.12.009
   Baumfeld D, 2019, FOOT ANKLE SURG, V25, P503, DOI 10.1016/j.fas.2018.03.003
   Benjamin M, 1997, HISTOL HISTOPATHOL, V12, P1135
   Carmont MR, 2013, FOOT ANKLE SURG, V19, P245, DOI 10.1016/j.fas.2013.06.007
   Carmont MR, 2008, KNEE SURG SPORT TR A, V16, P199, DOI 10.1007/s00167-007-0411-z
   Carmont MR, 2020, ORTHOP J SPORTS MED, V8, DOI 10.1177/2325967120909556
   Carmont MR, 2013, ORTHOP J SPORTS MED, V1, DOI 10.1177/2325967113494584
   Carmont MR, 2015, ASIA-PAC J SPORT MED, V2, P49, DOI 10.1016/j.asmart.2014.12.002
   Carpenter JE, 1999, CLIN ORTHOP RELAT R, pS296
   Chianca V, 2020, J ULTRASOUND, V23, P387, DOI 10.1007/s40477-020-00479-2
   Clanton T, 2020, ORTHOP CLIN N AM, V51, P391, DOI 10.1016/j.ocl.2020.02.005
   Costa ML, 2006, FOOT ANKLE INT, V27, P414, DOI 10.1177/107110070602700605
   D'Hooghe P, 2020, J BONE JOINT SURG AM, V102, P1466, DOI 10.2106/JBJS.20.00993
   Dahlgren LA, 2005, J ORTHOP RES, V23, P84, DOI 10.1016/j.orthres.2004.05.007
   de Aro AA, 2018, CELLS-BASEL, V7, DOI 10.3390/cells7090127
   Faul F, 2007, BEHAV RES METHODS, V39, P175, DOI 10.3758/BF03193146
   Frizziero A, 2014, BRIT MED BULL, V110, P47, DOI 10.1093/bmb/ldu006
   GARNER WL, 1989, PLAST RECONSTR SURG, V83, P875, DOI 10.1097/00006534-198905000-00018
   Gatz M, 2021, ARCH ORTHOP TRAUM SU, V141, P383, DOI 10.1007/s00402-020-03437-z
   Heinemeier KM, 2013, FASEB J, V27, P2074, DOI 10.1096/fj.12-225599
   Hope Matthew, 2007, Foot Ankle Clin, V12, P553, DOI 10.1016/j.fcl.2007.07.003
   Horstmann T, 2012, INT J SPORTS MED, V33, P474, DOI 10.1055/s-0032-1301932
   James R, 2008, J HAND SURG-AM, V33A, P102, DOI 10.1016/j.jhsa.2007.09.007
   Kanazawa Tomonoshin, 2014, Muscles Ligaments Tendons J, V4, P201
   Kangas J, 2007, AM J SPORT MED, V35, P59, DOI 10.1177/0363546506293255
   Klein MB, 2002, J HAND SURG-AM, V27A, P615, DOI 10.1053/jhsu.2002.34004
   Lui Pauline Po Yee, 2012, Muscles Ligaments Tendons J, V2, P163
   Maffulli N, 2003, AM J SPORT MED, V31, P692, DOI 10.1177/03635465030310051001
   Maffulli N, 2020, ORTHOP J SPORTS MED, V8, DOI 10.1177/2325967120907985
   Maffulli N, 2020, AM J SPORT MED, V48, P1181, DOI 10.1177/0363546520908592
   Maffulli N, 2020, LANCET, V395, P397, DOI 10.1016/S0140-6736(19)33133-2
   Maffulli N, 2020, INJURY, V51, pS73, DOI 10.1016/j.injury.2019.11.013
   Maffulli N, 2018, FOOT ANKLE SURG, V24, P164, DOI 10.1016/j.fas.2017.02.002
   Maffulli Nicola, 2017, Open Orthop J, V11, P660, DOI 10.2174/1874325001711010660
   Maffulli N, 2015, INT ORTHOP, V39, P707, DOI 10.1007/s00264-015-2686-x
   Maffulli N, 2010, AM J SPORT MED, V38, P2304, DOI 10.1177/0363546510376619
   Makarewich CA, 2020, J PEDIATR ORTHOP B, V29, P359, DOI 10.1097/BPB.0000000000000735
   Migliorini F, 2020, EXPERT OPIN BIOL TH, V20, P1373, DOI 10.1080/14712598.2020.1786532
   Mikic B, 2009, J ORTHOP RES, V27, P1603, DOI 10.1002/jor.20916
   Moher David, 2010, BMJ, V340, pc869, DOI [10.1016/j.ijsu.2011.10.001, 10.1016/j.jclinepi.2010.03.004, 10.1136/bmj.c869]
   NYSTROM B, 1983, ACTA ORTHOP SCAND, V54, P620, DOI 10.3109/17453678308992899
   Olsson N, 2013, AM J SPORT MED, V41, P2867, DOI 10.1177/0363546513503282
   Saxena A, 2011, J FOOT ANKLE SURG, V50, P37, DOI 10.1053/j.jfas.2010.10.008
   Schepull T, 2012, SCAND J MED SCI SPOR, V22, P18, DOI 10.1111/j.1600-0838.2010.01154.x
   Schneider M, 2018, ADV DRUG DELIVER REV, V129, P352, DOI 10.1016/j.addr.2017.12.016
   Sharma P, 2005, SURG-J R COLL SURG E, V3, P309, DOI 10.1016/S1479-666X(05)80109-X
   Silbernagel KG, 2012, AM J SPORT MED, V40, P1564, DOI 10.1177/0363546512447926
   Suydam SM, 2015, KNEE SURG SPORT TR A, V23, P868, DOI 10.1007/s00167-013-2512-1
   Thomopoulos S, 2005, J HAND SURG-AM, V30A, P441, DOI 10.1016/j.jhsa.2004.12.006
   Thomopoulos S, 2015, J ORTHOP RES, V33, P832, DOI 10.1002/jor.22806
   Tian J, 2020, ARCH ORTHOP TRAUM SU, V140, P1775, DOI 10.1007/s00402-020-03535-y
   Voleti PB, 2012, ANNU REV BIOMED ENG, V14, P47, DOI 10.1146/annurev-bioeng-071811-150122
   Wagner P, 2020, FOOT ANKLE SURG, V26, P421, DOI 10.1016/j.fas.2019.05.008
   Warden SJ, 2007, BRIT J SPORT MED, V41, P232, DOI 10.1136/bjsm.2006.032342
   Willits K, 2010, J BONE JOINT SURG AM, V92A, P2767, DOI 10.2106/JBJS.I.01401
   Yang B, 2017, INT J SURG, V40, P178, DOI 10.1016/j.ijsu.2017.03.021
   Yang G, 2013, BIRTH DEFECTS RES C, V99, P203, DOI 10.1002/bdrc.21041
   Yu Y, 2007, ARTHROSCOPY, V23, P205, DOI 10.1016/j.arthro.2006.08.023
   Zellers JA, 2018, FOOT ANKLE INT, V39, P343, DOI 10.1177/1071100717742372
NR 59
TC 0
Z9 0
U1 1
U2 1
PU SAGE PUBLICATIONS INC
PI THOUSAND OAKS
PA 2455 TELLER RD, THOUSAND OAKS, CA 91320 USA
SN 1071-1007
EI 1944-7876
J9 FOOT ANKLE INT
JI Foot Ankle Int.
PD FEB
PY 2022
VL 43
IS 2
BP 244
EP 252
AR 10711007211038594
DI 10.1177/10711007211038594
EA SEP 2021
PG 9
WC Orthopedics
WE Science Citation Index Expanded (SCI-EXPANDED)
SC Orthopedics
GA YY5PC
UT WOS:000703239900001
PM 34581220
OA hybrid, Green Published, Green Accepted
DA 2022-07-22
ER

PT J
AU Josey, RA
   Marymont, JV
   Varner, KE
   Borom, A
   O'Connor, D
   Oates, JC
AF Josey, RA
   Marymont, JV
   Varner, KE
   Borom, A
   O'Connor, D
   Oates, JC
TI Immediate, full weightbearing cast treatment of acute Achilles tendon
   ruptures: A long-term follow-up study
SO FOOT & ANKLE INTERNATIONAL
LA English
DT Article
DE Achilles tendon; rupture; treatment
ID NON-SURGICAL TREATMENT; NONOPERATIVE TREATMENT
AB Thirty-two patients with 33 ruptures were available for comprehensive evaluation, which included a questionnaire, physical examination, and manual as well as objective (Kin-Com) strength testing. Data excluding Kin-Corn strength testing were available in an additional seven patients with seven ruptures; hence, the data are based on these 39 patients with 40 ruptures. Five patients with eight ruptures were excluded from strength testing but were included in our rerupture data, which totaled 44 patients with 48 ruptures. All patients were treated using a standard protocol by a single surgeon. All patients were treated within 11 days of injury, and the average followup was 55 months (range, 24-133). Ninety-five percent (38/39) of patients were completely satisfied with their treatment. The American Orthopaedic Foot and Ankle Society (AOFAS) ankle hindfoot scores were >80 in 97.4% of patients, and the rerupture rate was 6.25% (3/48). Calf circumference and ankle range of motion were not statistically different between the two legs. The injured leg tended to be slightly weaker than the uninjured leg, but the difference was not significant for most of the quantitative strength measurements. This study provides data for the Achilles rupture patient who asks, "Is there a treatment option other than surgery?" In addition, the standardized protocol can be utilized to obtain successful outcomes in those who choose nonoperative treatment or are poor surgical candidates.
C1 Baylor Coll Med, Dept Orthoped Surg, Houston, TX 77030 USA.
   Fdn Orthoped Athlet & Reconstruct Res, Houston, TX USA.
   Joe W King Orthoped Inst, Houston, TX USA.
   Texas Orthoped Hosp, Houston, TX USA.
RP Marymont, JV (通讯作者)，6560 Fannin,Ste 400, Houston, TX 77030 USA.
CR CARDEN DG, 1987, J BONE JOINT SURG BR, V69, P416, DOI 10.1302/0301-620X.69B3.3294839
   CETTI R, 1993, AM J SPORT MED, V21, P791, DOI 10.1177/036354659302100606
   HAGGMARK T, 1986, ORTHOPEDICS, V9, P160
   INGLIS AE, 1976, J BONE JOINT SURG AM, V58, P990, DOI 10.2106/00004623-197658070-00015
   Jacobs D, 1978, Am J Sports Med, V6, P107, DOI 10.1177/036354657800600302
   Kannus P., 1994, INT J SPORTS MED, V15, P11
   KITAOKA HB, 1994, FOOT ANKLE INT, V15, P349, DOI 10.1177/107110079401500701
   LEA RB, 1972, J BONE JOINT SURG AM, VA 54, P1398, DOI 10.2106/00004623-197254070-00002
   Lo IKY, 1997, CLIN J SPORT MED, V7, P207, DOI 10.1097/00042752-199707000-00010
   McComis GP, 1997, J BONE JOINT SURG AM, V79A, P1799, DOI 10.2106/00004623-199712000-00005
   SAPEGA AA, 1990, J BONE JOINT SURG AM, V72A, P1562, DOI 10.2106/00004623-199072100-00023
   THERMANN H, 1992, ORTHOP T, V16, P729
   THOMPSON T C, 1962, J Trauma, V2, P126, DOI 10.1097/00005373-196203000-00003
NR 13
TC 16
Z9 16
U1 0
U2 1
PU AMER ORTHOPAEDIC FOOT & ANKLE SOC, INC
PI SEATTLE
PA 2517 EASTLAKE AVE EAST, STE 200, SEATTLE, WA 98102 USA
SN 1071-1007
J9 FOOT ANKLE INT
JI Foot Ankle Int.
PD OCT
PY 2003
VL 24
IS 10
BP 775
EP 779
DI 10.1177/107110070302401007
PG 5
WC Orthopedics
WE Science Citation Index Expanded (SCI-EXPANDED)
SC Orthopedics
GA 731HC
UT WOS:000185877400007
PM 14587992
DA 2022-07-22
ER

PT J
AU Therbo, M
   Petersen, MM
   Nielsen, PK
   Lund, B
AF Therbo, M
   Petersen, MM
   Nielsen, PK
   Lund, B
TI Loss of bone mineral of the hip and proximal tibia following rupture of
   the Achilles tendon
SO SCANDINAVIAN JOURNAL OF MEDICINE & SCIENCE IN SPORTS
LA English
DT Article
DE bone loss; prospective; DEXA; BMD; injury; Achilles tendon
ID CRUCIATE LIGAMENT INJURY; X-RAY ABSORPTIOMETRY; PERMANENT OSTEOPOROSIS;
   LOWER-EXTREMITY; DENSITY; FRACTURE; FEMUR; SPINE; KNEE; SITES
AB In a prospective uncontrolled study 12 patients suffering from a rupture of the Achilles tendon treated operatively with surgical repair and post-operative immobilization in a short plaster cast for 6 weeks had bilateral measurements of bone mineral content (BMC) of the proximal tibia and bone mineral density (BMD) of the femoral neck and greater trochanter. The measurements were performed by dual energy X-ray absorptiometry (DEXA) and scans were performed post-operatively within 7 days after the operation and with follow up after 6 weeks, 3, 6, and 12 months. In the operated legs, BMC of the proximal tibia showed a progressive decrease reaching a total bone loss of 6.4% (95%-CL: -10.6%; -2.3%) 1 year after the injury. Bone mineral density at the hip of the operated legs also decreased significantly and 1 year after the injury BMD was 2.5% (95%-CL: -5.5%; 0.5%) and 6.8% (95%-CL: -9.8%; -3.7%) below the initial value in, respectively, the femoral neck and greater trochanter. Patients with a previous Achilles tendon rupture must be considered to be some years ahead in their natural osteoporotic process of the bones of the affected legs, and an increased risk of osteoporotic fractures must be considered not to be only theoretical .
C1 Natl Univ Hosp, Rigshosp, Dept Orthopaed Surg U2161, DK-2100 Copenhagen O, Denmark.
   Natl Univ Hosp, Rigshosp, Dept Nephrol P, DK-2100 Copenhagen, Denmark.
RP Therbo, M (通讯作者)，Natl Univ Hosp, Rigshosp, Dept Orthopaed Surg U2161, Blegdamsvej 9, DK-2100 Copenhagen O, Denmark.
OI Petersen, Michael Mork/0000-0002-2324-6420
CR Alfredson H, 1998, CALCIFIED TISSUE INT, V62, P166, DOI 10.1007/s002239900411
   ALOIA JF, 1990, METABOLISM, V39, P1144, DOI 10.1016/0026-0495(90)90086-R
   ANDERSSON SM, 1979, MED SCI SPORT EXER, V11, P351
   ANDERSSON SM, 1979, CLIN ORTHOP RELAT R, P226
   BOHR HH, 1987, ACTA ORTHOP SCAND, V58, P557, DOI 10.3109/17453678709146399
   CHECOVICH MM, 1989, CALCIFIED TISSUE INT, V45, P281, DOI 10.1007/BF02556019
   Clement EV, 1999, BONE, V24, P131
   COURTNEY AC, 1994, CALCIFIED TISSUE INT, V55, P53, DOI 10.1007/BF00310169
   CUMMINGS SR, 1993, LANCET, V341, P72, DOI 10.1016/0140-6736(93)92555-8
   ERIKSEN EF, 1986, ENDOCR REV, V7, P379, DOI 10.1210/edrv-7-4-379
   FINSEN V, 1989, CLIN ORTHOP RELAT R, P244
   KANNUS P, 1994, J BONE MINER RES, V9, P423
   KANNUS P, 1994, J BONE MINER RES, V9, P1729
   KANNUS P, 1995, J BONE MINER RES, V10, P1263
   KANNUS P, 1992, J BONE MINER RES, V7, P1429
   KARLSSON MK, 1993, ACTA ORTHOP SCAND, V64, P482, DOI 10.3109/17453679308993674
   KARLSSON MK, 1993, CLIN ORTHOP RELAT R, P87
   KARLSSON MK, 1993, ACTA ORTHOP SCAND, V64, P362
   Leppala J, 1999, CALCIFIED TISSUE INT, V64, P357, DOI 10.1007/s002239900632
   Lilley J, 1991, Osteoporos Int, V1, P141, DOI 10.1007/BF01625443
   Marshall D, 1996, BRIT MED J, V312, P1254, DOI 10.1136/bmj.312.7041.1254
   MELTON LJ, 1986, AM J EPIDEMIOL, V124, P254, DOI 10.1093/oxfordjournals.aje.a114383
   NILSSON B. E. R., 1966, ACTA ORTHOP SCAND, V37, P1
   NILSSON BE, 1969, CALC TISS RES, V4, P185, DOI 10.1007/BF02279120
   OBRANT KJ, 1984, CLIN ORTHOP RELAT R, P270
   Petersen M, 1992, EUR J EXP MUSCULOSKE, V1, P77
   Petersen MM, 1996, J ORTHOPAED RES, V14, P16, DOI 10.1002/jor.1100140105
   Petersen MM, 1997, BONE, V20, P491, DOI 10.1016/S8756-3282(97)00028-8
   PETERSEN MM, 1993, EUR J EXP MUSCULOSKE, V2, P121
   SCHAADT O, 1988, CALCIFIED TISSUE INT, V42, P71, DOI 10.1007/BF02556337
   SIEVANEN H, 1992, J NUCL MED, V33, P1137
   VANDERWIEL HE, 1994, J BONE JOINT SURG AM, V76A, P230, DOI 10.2106/00004623-199402000-00009
   WILLIAMSRUSSO P, 1995, J ORTHOPAED RES, V13, P250, DOI 10.1002/jor.1100130213
NR 33
TC 11
Z9 11
U1 0
U2 1
PU BLACKWELL MUNKSGAARD
PI COPENHAGEN
PA 35 NORRE SOGADE, PO BOX 2148, DK-1016 COPENHAGEN, DENMARK
SN 0905-7188
J9 SCAND J MED SCI SPOR
JI Scand. J. Med. Sci. Sports
PD JUN
PY 2003
VL 13
IS 3
BP 194
EP 199
DI 10.1034/j.1600-0838.2003.20205.x
PG 6
WC Sport Sciences
WE Science Citation Index Expanded (SCI-EXPANDED)
SC Sport Sciences
GA 675YH
UT WOS:000182724800009
PM 12753493
DA 2022-07-22
ER

PT J
AU Inceoglu, A
   Sahin, F
   Akkaya, N
   Ok, N
   Yorukoglu, C
   Mete, G
   Akgun, S
AF Inceoglu, Alperen
   Sahin, Fusun
   Akkaya, Nuray
   Ok, Nusret
   Yorukoglu, Cagdas
   Mete, Gulcin
   Akgun, Sakir
TI Effects of low-density pulsed ultrasound treatment on transforming
   growth factor-beta, collagen level, histology, biomechanics, and
   function in repaired rat tendons
SO TURKISH JOURNAL OF PHYSICAL MEDICINE AND REHABILITATION
LA English
DT Article
DE Achilles tendon; biomechanic; growth factor; histology; low-density
   therapeutic ultrasound; tendon injury
ID ACHILLES-TENDON; THERAPEUTIC ULTRASOUND; INTENSITY; RUPTURE
AB Objectives: The aim of this study is to compare the effects of low-density pulsed ultrasound (LIPUS) treatment on growth factors/collagen production, histological, biomechanical, and function of rats with Achilles tendon injury.
   Materials and methods: A total of 44 Wistar Albino rats were used in the study between April 2017 and June 2018. The rats were randomized to two treatment groups. Group 1 (n=6) received LIPUS treatment (0.3 Watt/cm(2); 1 MHz, 1:5 pulse mode) and Group 2 (n=6)received sham ultrasound (US) treatment following Achilles tendon surgery. Transforming growth factor-beta 1 (TGF-beta 1) and collagen gene expression levels were evaluated using polymerase chain reaction. The histological evaluation was performed with the Bonar scoring system. The tensile strength was measured by biomechanical testing and the function was evaluated with the Achilles Functional Index (AFI).
   Results: Although TGF-beta 1 expression and tensile strength evaluation showed a tendency to improve in favor of the LIPUS group, no statistically significant difference was found (p=0.065 and p=0.053, respectively). The COL3 gene expression in the LIPUS group and the COL1 expression in the sham US group were significantly higher. Bonar scores and AFI scores showed a statistically significant improvement in the LIPUS group, compared to the sham US group.
   Conclusion: Our study results show that LIPUS yields positive effects on tendon histology and functional status in repaired Achilles tendon in rats.
C1 [Inceoglu, Alperen] Osmaniye State Hosp, Dept Phys Med & Rehabil, Osmaniye, Turkey.
   [Sahin, Fusun; Akkaya, Nuray] Pamukkale Univ, Dept Phys Med & Rehabil, Fac Med, Denizli, Turkey.
   [Ok, Nusret; Yorukoglu, Cagdas] Pamukkale Univ, Dept Orthoped & Traumatol, Fac Med, Denizli, Turkey.
   [Mete, Gulcin] Pamukkale Univ, Dept Histol & Embryol, Fac Med, Denizli, Turkey.
   [Akgun, Sakir] Pamukkale Univ, ILTAM Lab, Fac Med, Denizli, Turkey.
RP Sahin, F (通讯作者)，Pamukkale Univ, Dept Phys Med & Rehabil, Fac Med, Denizli, Turkey.
EM drfusunsahin@yahoo.com.tr
OI AKKAYA, NURAY/0000-0002-9307-3342
FU "specialization thesis support project" fund of university "Scientific
   Research Project" [2019TIPF022]
FX The study was realized with supporting of "specialization thesis support
   project" fund of university "Scientific Research Project" (2019TIPF022).
CR Birch HL, 2007, INT J EXP PATHOL, V88, P241, DOI 10.1111/j.1365-2613.2007.00552.x
   Branford OA, 2014, J HAND SURG-EUR VOL, V39, P60, DOI 10.1177/1753193413509231
   Burgisser GM, 2016, BIOL OPEN, V5, P1324, DOI 10.1242/bio.020644
   Chen Q, 2014, INT J CLIN EXP PATHO, V7, P8462
   Cinar BM, 2013, ACTA ORTHOP TRAUMATO, V47, P266, DOI 10.3944/AOTT.2013.2784
   da Cunha A, 2001, ULTRASOUND MED BIOL, V27, P1691, DOI 10.1016/S0301-5629(01)00477-X
   Dabak TK, 2015, BIOMED RES INT, V2015, DOI 10.1155/2015/689314
   ENWEMEKA CS, 1990, ULTRASOUND MED BIOL, V16, P801, DOI 10.1016/0301-5629(90)90044-D
   Farcic TS, 2013, BRAZ J PHYS THER, V17, P263, DOI 10.1590/S1413-35552012005000090
   Farhat YM, 2012, PLOS ONE, V7, DOI 10.1371/journal.pone.0051411
   Fu SC, 2008, AM J SPORT MED, V36, P1742, DOI 10.1177/0363546508318193
   Heisterbach PE, 2012, KNEE SURG SPORT TR A, V20, P1903, DOI 10.1007/s00167-011-1772-x
   Herod TW, 2016, ACTA BIOMATER, V42, P296, DOI 10.1016/j.actbio.2016.06.017
   Jeremias SL, 2011, J ORTHOP SPORT PHYS, V41, P526, DOI 10.2519/jospt.2011.3468
   Kannus P, 2000, SCAND J MED SCI SPOR, V10, P312, DOI 10.1034/j.1600-0838.2000.010006312.x
   Liang JI, 2012, ANN BIOMED ENG, V40, P2532, DOI 10.1007/s10439-012-0619-z
   Loiselle AE, 2015, J ORTHOP RES, V33, P859, DOI 10.1002/jor.22890
   Maffulli N, 2002, J BONE JOINT SURG BR, V84B, P1, DOI 10.1302/0301-620X.84B1.12792
   MURRELL GAC, 1992, J ORTHOPAED RES, V10, P398, DOI 10.1002/jor.1100100313
   Ng COY, 2003, ULTRASOUND MED BIOL, V29, P1501, DOI 10.1016/S0301-5629(03)01018-4
   Ng GYF, 2007, ULTRASOUND MED BIOL, V33, P1750, DOI 10.1016/j.ultrasmedbio.2007.05.019
   Ng GYF, 2004, ULTRASOUND MED BIOL, V30, P1539, DOI 10.1016/j.ultrasmedbio.2004.08.030
   Quigley AS, 2018, SCI REP-UK, V8, DOI 10.1038/s41598-018-22741-8
   Sammer DM, 2014, WOUND REPAIR REGEN, V22, P25, DOI 10.1111/wrr.12161
   Sharma P., 2006, Journal of Musculoskeletal & Neuronal Interactions, V6, P181
   Tsai WC, 2006, J ORTHOP RES, V24, P1310, DOI 10.1002/jor.20130
   Xia C, 2010, ORTHOPEDICS, V33
   Yang G, 2013, BIRTH DEFECTS RES C, V99, P203, DOI 10.1002/bdrc.21041
   Yeung CK, 2006, J ORTHOP RES, V24, P193, DOI 10.1002/jor.20020
   Zhang C, 2017, ACTA CIR BRAS, V32, P1045, DOI 10.1590/s0102-865020170120000006
NR 30
TC 0
Z9 0
U1 2
U2 3
PU BAYCINAR MEDICAL PUBL-BAYCINAR TIBBI YAYINCILIK
PI ATASEHIR
PA ORNEK MH DR SUPHI EZGI SK SARAY APT NO 11 D 6, ATASEHIR, ISTANBUL 34704,
   TURKEY
SN 2587-0823
J9 TURK J PH MED REHAB
JI Turk. J. Phys. Med. Rehabil.
PY 2021
VL 67
IS 2
BP 167
EP 174
DI 10.5606/tftrd.2021.5118
PG 8
WC Rehabilitation
WE Science Citation Index Expanded (SCI-EXPANDED)
SC Rehabilitation
GA SK2NO
UT WOS:000656056900004
PM 34396067
OA Green Published, gold
DA 2022-07-22
ER

PT J
AU Van der Linden, PD
   van Puijenbroek, EP
   Feenstra, J
   in't Veld, BA
   Sturkenboom, MCJM
   Herings, RMC
   Leufkens, HGM
   Stricker, BHC
AF Van der Linden, PD
   van Puijenbroek, EP
   Feenstra, J
   in't Veld, BA
   Sturkenboom, MCJM
   Herings, RMC
   Leufkens, HGM
   Stricker, BHC
TI Tendon disorders attributed to fluoroquinolones: A study on 42
   spontaneous reports in the period 1988 to 1998
SO ARTHRITIS & RHEUMATISM-ARTHRITIS CARE & RESEARCH
LA English
DT Article
DE fluoroquinolones; Achilles tendon rupture; tendinitis; ofloxacin;
   ciprofloxacin; norfloxacin; pefloxacin
ID ACHILLES-TENDON; CLINICAL-PRACTICE; RUPTURE; CIPROFLOXACIN; ANTIBIOTICS;
   TENDINITIS; TENDINOPATHY; QUINOLONES; NORFLOXACIN; AGENTS
AB Objective. Fluoroquinolone antibiotics have been associated with tendinitis and tendon rupture. In this paper we report on the followup of 42 spontaneous reports of fluoroquinolone-associated tendon disorders.
   Methods, This study is based on cases of fluoroquinolone-associated tendon disorders reported to the Netherlands Pharmacovigilance Foundation Lareb and the Drug Safety Unit of the Inspectorate for Health Care between January 1, 1988, and January 1, 1998. By means of a mailed questionnaire, we collected information on the site of injury, onset of symptoms, treatment, and course of the tendon disorder as well as information on possible risk factors and concomitant medication.
   Results. Of 50 mailed questionnaires, 42 (84%) were returned. The data concerned 32 patients (76%) with tendinitis and 10 patients (24%) with a tendon rupture. Sixteen cases (38%) were attributed to ofloxacin, 13 (31%) to ciprofloxacin, 8 (19%) to norfloxacin, and 5 (12%) to pefloxacin. There was a male predominance, and the median age of the patients was 68 years. Most of the reports concerned the Achilles tendon, and 24 patients (57%) had bilateral tendinitis. The latency period between the start of treatment and the appearance of the first symptoms ranged from 1 to 510 days with a median of 6 days. Most patients recovered within 2 months after cessation of therapy, but 26% had not yet recovered at followup.
   Conclusion. These reports suggest that fluoroquinolone-associated tendon disorders are more common in patients over 60 years of age. Ofloxacin was implicated most frequently relative to the number of filled prescriptions in the Netherlands.
C1 Erasmus MC, Dept Epidemiol & Biostat, Pharmacoepidemiol Unit, NL-3000 DR Rotterdam, Netherlands.
   Erasmus MC, Dept Internal Med, NL-3000 DR Rotterdam, Netherlands.
   Utrecht Inst Pharmaceut Sci, Dept Pharmacoepidemiol & Pharmacotherapy, Utrecht, Netherlands.
   Netherlands Pharmacovigilance Fdn, sHertogenbosch, Netherlands.
   Inspectorate Hlth Care, Drug Safety Unit, The Hague, Netherlands.
RP Stricker, BHC (通讯作者)，Erasmus MC, Dept Epidemiol & Biostat, Pharmacoepidemiol Unit, POB 1738, NL-3000 DR Rotterdam, Netherlands.
RI Herings, Ron/AAE-4096-2021; van Puijenbroek, Eugene/AAI-3240-2020
OI Herings, Ron/0000-0002-2561-8734; van Puijenbroek,
   Eugene/0000-0002-2236-1398
CR AMTSUMOTO K, 1992, CLIN ORTHOPAEDICS, P235
   BAILEY RR, 1983, NEW ZEAL MED J, V96, P590
   Bernard-Beaubois K, 1998, CELL BIOL TOXICOL, V14, P283, DOI 10.1023/A:1007435025616
   CHASLERIE A, 1992, REV RHUM, V59, P297
   CRONIN ME, 1988, RHEUM DIS CLIN N AM, V14, P99
   Davey PG, 1996, BRIT MED J, V312, P613, DOI 10.1136/bmj.312.7031.613
   DONCK JB, 1994, TRANSPLANTATION, V58, P736, DOI 10.1097/00007890-199409270-00021
   FRANCK JL, 1991, REV RHUM, V58, P904
   Guerin B, 1996, ANN MED INTERNE, V147, P69
   HAYEM G, 1995, DRUG SAFETY, V13, P338, DOI 10.2165/00002018-199513060-00003
   HERINGS RMC, 1993, PHARMO RECORD LINKAG
   HOOPER DC, 1991, NEW ENGL J MED, V324, P384, DOI 10.1056/NEJM199102073240606
   HUSTON KA, 1994, NEW ENGL J MED, V331, P748, DOI 10.1056/NEJM199409153311116
   Jagose JT, 1996, NEW ZEAL MED J, V109, P471
   JORGENSEN C, 1991, REV RHUM, V58, P623
   JOZSA L, 1989, AM J SPORT MED, V17, P338, DOI 10.1177/036354658901700305
   KATO M, 1995, TOXICOL PATHOL, V23, P385, DOI 10.1177/019262339502300315
   LEE WT, 1992, AUST NZ J MED, V22, P500
   LEHUEC JC, 1995, J BONE JOINT SURG BR, V77B, P293, DOI 10.1302/0301-620X.77B2.7706350
   Levadoux M, 1997, Ann Chir Main Memb Super, V16, P130, DOI 10.1016/S0753-9053(97)80033-6
   Lietman P S, 1995, Drugs, V49 Suppl 2, P159
   Marti HP, 1998, BRIT J RHEUMATOL, V37, P343
   MCEWAN SR, 1988, LANCET, V2, P900
   McGarvey WC, 1996, FOOT ANKLE INT, V17, P496, DOI 10.1177/107110079601700811
   Meyboom RHBS, 1994, PHARMACOEPIDEMIOL DR, V3, P185
   Moellering RC, 1996, CHEMOTHERAPY, V42, P54
   Movin T, 1997, FOOT ANKLE INT, V18, P297, DOI 10.1177/107110079701800510
   *NORD COUNC MED, 1996, NORD STAT MED 1993 1
   PERROT S, 1991, PRESSE MED, V20, P1234
   Pierfitte C, 1996, THERAPIE, V51, P419
   RIBARD P, 1992, J RHEUMATOL, V19, P1479
   RIBARD P, 1991, BAILLIERE CLIN RHEUM, V5, P175, DOI 10.1016/S0950-3579(05)80301-2
   ROYER RJ, 1994, THERAPIE, V49, P75
   Shinohara YT, 1997, J RHEUMATOL, V24, P238
   SZARFMAN A, 1995, NEW ENGL J MED, V332, P193
   van der Linden PD, 1999, BRIT J CLIN PHARMACO, V48, P433
   Waterston SW, 1997, BRIT J SPORT MED, V31, P285, DOI 10.1136/bjsm.31.4.285
   West MB, 1998, NEW ZEAL MED J, V111, P18
   Wilton LV, 1996, BRIT J CLIN PHARMACO, V41, P277, DOI 10.1046/j.1365-2125.1996.03013.x
   Zabraniecki L, 1996, J RHEUMATOL, V23, P516
NR 40
TC 96
Z9 97
U1 0
U2 3
PU WILEY-LISS
PI HOBOKEN
PA DIV JOHN WILEY & SONS INC, 111 RIVER ST, HOBOKEN, NJ 07030 USA
SN 0004-3591
EI 1529-0131
J9 ARTHRIT RHEUM-ARTHR
JI Arthritis Rheum-Arthritis Care Res.
PD JUN
PY 2001
VL 45
IS 3
BP 235
EP 239
DI 10.1002/1529-0131(200106)45:3<235::AID-ART254>3.0.CO;2-7
PG 5
WC Rheumatology
WE Science Citation Index Expanded (SCI-EXPANDED)
SC Rheumatology
GA 487JT
UT WOS:000171872500005
PM 11409663
OA Bronze
DA 2022-07-22
ER

PT J
AU Yotsumoto, T
   Miyamoto, W
   Uchio, Y
AF Yotsumoto, Tadahiko
   Miyamoto, Wataru
   Uchio, Yuji
TI Novel Approach to Repair of Acute Achilles Tendon Rupture Early Recovery
   Without Postoperative Fixation or Orthosis
SO AMERICAN JOURNAL OF SPORTS MEDICINE
LA English
DT Article
DE Achilles tendon; rupture; operation; early motion; braided polyblend
   suture thread; side-locking loop technique
ID LOCKING LOOP TECHNIQUE; EARLY MOBILIZATION; SURGICAL REPAIR; FUNCTIONAL
   TREATMENT; WEIGHTBEARING; SURGERY; FLEXION; WALKING; MOTION; KNOT
AB Background: Immobilization or orthosis is required after conventional Achilles tendon surgery.
   Hypothesis: This new Achilles tendon repair approach enables early rehabilitation without any postoperative immobilization or orthosis.
   Study Design: Case series; Level of evidence, 4.
   Methods: Twenty consecutive patients (14 men and 6 women; mean age, 43.4 years; range, 16-70 years) who had acute subcutaneous Achilles tendon rupture were treated by the new method, with an average follow-up of 2.9 years (range, 2-4.8 years). Among them, 15 injuries were sports-related and 5 were work-related. The authors applied a side-locking loop technique of their own design for the core suture, using braided polyblend suture thread, with peripheral cross-stitches added. The patients started active and passive ankle mobilization from the next day, partial weightbearing walking from 1 week, full-load walking from 4 weeks, and double-legged heel raises from 6 weeks after surgery.
   Results: The range of motion recovery equal to the intact side averaged 3.2 weeks. Double-legged heel raises and 20 continuous single-legged heel raise exercises were possible at an average of 6.3 weeks and 9.9 weeks, respectively. T2-weighted magnetic resonance signal intensity recovered to equal that of the intact portion of the same tendon at 12 weeks. The patients resumed sports activities or heavy labor at an average of 14.4 weeks. The Achilles tendon rupture score averaged 98.3 at 24 weeks. There were no complications.
   Conclusion: This new Achilles tendon repair approach enables early mobilization exercise without costly specialized orthosis or immobilization and allows an early return to normal life and sports activities, reducing the physical and economic burden on patients.
C1 [Yotsumoto, Tadahiko; Miyamoto, Wataru; Uchio, Yuji] Shimane Univ, Sch Med, Dept Orthopaed Surg, Izumo, Shimane 6938501, Japan.
RP Yotsumoto, T (通讯作者)，Shimane Univ, Sch Med, Dept Orthopaed Surg, 89-1 Enya Cho, Izumo, Shimane 6938501, Japan.
EM sssyotsu@yahoo.co.jp
CR Aoki M, 1998, AM J SPORT MED, V26, P794, DOI 10.1177/03635465980260061001
   CETTI R, 1994, CLIN ORTHOP RELAT R, P155
   CETTI R, 1993, AM J SPORT MED, V21, P791, DOI 10.1177/036354659302100606
   Froberg A, 2009, AM J SPORT MED, V37, P1200, DOI 10.1177/0363546508330126
   GREGOR RJ, 1987, INT J SPORTS MED, V8, P9
   Hufner T, 2002, FOOT ANKLE INT, V23, P614, DOI 10.1177/107110070202300705
   Jaakkola JI, 2000, FOOT ANKLE INT, V21, P14, DOI 10.1177/107110070002100103
   Kangas J, 2007, AM J SPORT MED, V35, P59, DOI 10.1177/0363546506293255
   Karjalainen PT, 1997, AM J SPORT MED, V25, P164, DOI 10.1177/036354659702500204
   Kerkhoffs GMMJ, 2002, ARCH ORTHOP TRAUM SU, V122, P102, DOI 10.1007/s004020100312
   Komatsu F, 2007, CLIN BIOMECH, V22, P112, DOI 10.1016/j.clinbiomech.2006.06.006
   KOMI PV, 1992, CLIN SPORT MED, V11, P521
   Kuwata S, 2007, CLIN BIOMECH, V22, P1083, DOI 10.1016/j.clinbiomech.2007.08.016
   Leppilahti J, 1998, CLIN ORTHOP RELAT R, P152
   Maffulli N, 2003, AM J SPORT MED, V31, P692, DOI 10.1177/03635465030310051001
   Maffulli N, 2001, SCAND J MED SCI SPOR, V11, P156
   Moller M, 2001, J BONE JOINT SURG BR, V83B, P843
   Moller M, 2002, SCAND J MED SCI SPOR, V12, P9, DOI 10.1034/j.1600-0838.2002.120103.x
   Mortensen NHM, 1999, J BONE JOINT SURG AM, V81A, P983, DOI 10.2106/00004623-199907000-00011
   Mullaney MJ, 2006, AM J SPORT MED, V34, P1120, DOI 10.1177/0363546505284186
   NISTOR L, 1981, J BONE JOINT SURG AM, V63, P394, DOI 10.2106/00004623-198163030-00012
   Palmes D, 2002, J ORTHOPAED RES, V20, P939, DOI 10.1016/S0736-0266(02)00032-3
   Pneumaticos SG, 2000, FOOT ANKLE INT, V21, P551, DOI 10.1177/107110070002100704
   SILFVERSKIOLD KL, 1994, J HAND SURG-AM, V19A, P53, DOI 10.1016/0363-5023(94)90224-0
   Sorrenti SJ, 2006, FOOT ANKLE INT, V27, P407, DOI 10.1177/107110070602700603
   Speck M, 1998, AM J SPORT MED, V26, P789, DOI 10.1177/03635465980260060901
   Stehno-Bittel L, 1998, MED SCI SPORT EXER, V30, P788, DOI 10.1097/00005768-199806000-00002
   Twaddle BC, 2007, AM J SPORT MED, V35, P2033, DOI 10.1177/0363546507307503
   Uchiyama E, 2007, AM J SPORT MED, V35, P1739, DOI 10.1177/0363546507304492
   Yamagami N, 2006, J ORTHOP SCI, V11, P614, DOI 10.1007/s00776-006-1064-3
   Yotsumoto T, 2005, J ORTHOP SCI, V10, P515, DOI 10.1007/s00776-005-0929-1
NR 31
TC 23
Z9 27
U1 0
U2 2
PU SAGE PUBLICATIONS INC
PI THOUSAND OAKS
PA 2455 TELLER RD, THOUSAND OAKS, CA 91320 USA
SN 0363-5465
EI 1552-3365
J9 AM J SPORT MED
JI Am. J. Sports Med.
PD FEB
PY 2010
VL 38
IS 2
BP 287
EP 292
DI 10.1177/0363546509351557
PG 6
WC Orthopedics; Sport Sciences
WE Science Citation Index Expanded (SCI-EXPANDED)
SC Orthopedics; Sport Sciences
GA 550AB
UT WOS:000274096500009
PM 20044498
DA 2022-07-22
ER

PT J
AU Li, QB
   Wang, CY
   Huo, YQ
   Jia, ZW
   Wang, XQ
AF Li, Qingbo
   Wang, Chuanying
   Huo, Yanqing
   Jia, Zhiwei
   Wang, Xiqian
TI 被撤回的出版物: Minimally invasive versus open surgery for acute Achilles
   tendon rupture: a systematic review of overlapping meta-analyses
   (Retracted article. See vol. 13, art. 156, 2018)
SO JOURNAL OF ORTHOPAEDIC SURGERY AND RESEARCH
LA English
DT Article; Retracted Publication
DE Achilles tendon rupture; Minimally invasive surgery; Open surgery;
   Systematic review; Meta-analysis
ID METHODOLOGICAL QUALITY; MEASUREMENT TOOL; OPEN REPAIR; FRACTURES; AMSTAR
AB Background: A number of meta-analyses have been carried out to evaluate the effects of minimally invasive surgery (MIS) versus open surgery (OS) for acute Achilles tendon rupture. However, discordant findings were seen in these meta-analyses. The present study, performing a systematic review of overlapping meta-analyses regarding MIS versus OS of acute Achilles tendon rupture, aimed to assist decision-makers interpret and choose among conflicting meta-analyses, as well as to offer treatment recommendations based on current best evidence.
   Methods: The literature search was performed to identify systematic reviews comparing MIS with OS for Achilles tendon rupture. Meta-analyses only comprising randomized controlled trials (RCTs) were included. Two authors individually evaluated the quality of meta-analysis and extracted data. The Jadad decision algorithm was conducted to ascertain which meta-analysis offered the best evidence.
   Results: A total of four meta-analyses was included. All these meta-analyses comprised RCTs or quasi-RCTs and were determined as Level-II evidence. The scores of the Assessment of Multiple Systematic Reviews (AMSTAR) ranged from 7 to 10 (median 9.5). The Jadad algorithm indicated that the best meta-analysis should be chosen according to the search strategies and application of selection. A high-quality meta-analysis with more RCTs was chosen, which suggested that there was no statistically significant difference between MIS and OS regarding rerupture rate, tissue adhesion, sural nerve injury, deep infection, and deep vein thrombosis. However, MIS could decrease superficial infection rate, and had a better patient satisfaction for good to excellent outcomes in comparison to OS.
   Conclusions: Based on the best available evidence, MIS may be superior to OS for treating acute Achilles tendon rupture. However, due to some limitations, this should be cautiously interpreted, and further high-quality studies are needed.
C1 [Li, Qingbo; Huo, Yanqing; Wang, Xiqian] Shandong Univ, Hosp 2, Dept Orthopaed Trauma, Jinan 250100, Peoples R China.
   [Wang, Chuanying] Shandong Univ Tradit Chinese Med, Affiliated Hosp 2, Dept Rehabil, 1 Jingba Rd, Jinan 250001, Peoples R China.
   [Jia, Zhiwei] 306th Hosp Peoples Liberat Army, Dept Orthopaed, Beijing, Peoples R China.
RP Wang, CY (通讯作者)，Shandong Univ Tradit Chinese Med, Affiliated Hosp 2, Dept Rehabil, 1 Jingba Rd, Jinan 250001, Peoples R China.; Jia, ZW (通讯作者)，306th Hosp Peoples Liberat Army, Dept Orthopaed, Beijing, Peoples R China.
EM wangchuanyingjinan@163.com; jiazhiwei@139.com
CR Aktas S, 2009, FOOT ANKLE INT, V30, P391, DOI 10.3113/FAI.2009.0391
   Avina Valencia Jorge Arturo, 2009, Acta Ortop Mex, V23, P125
   Carmont MR, 2013, KNEE SURG SPORT TR A, V21, P1361, DOI 10.1007/s00167-013-2423-1
   Del Buono A, 2014, BRIT MED BULL, V109, P45, DOI 10.1093/bmb/ldt029
   Erickson BJ, 2015, ARTHROSCOPY, V31, P1207, DOI 10.1016/j.arthro.2014.11.040
   Gigante A, 2008, KNEE SURG SPORT TR A, V16, P204, DOI 10.1007/s00167-007-0448-z
   Gwynne-Jones DP, 2011, FOOT ANKLE INT, V32, P337, DOI 10.3113/FAI.2011.0337
   Jadad AR, 1997, CAN MED ASSOC J, V156, P1411
   Jones Matthew P, 2012, J Bone Joint Surg Am, V94, pe88, DOI 10.2106/JBJS.J.01829
   Karabinas Panagiotis K, 2014, Eur J Orthop Surg Traumatol, V24, P607, DOI 10.1007/s00590-013-1350-7
   Khan RJK, 2010, COCHRANE DB SYST REV, DOI 10.1002/14651858.CD003674.pub4
   Khan RJK, 2005, J BONE JOINT SURG AM, V87A, P2202, DOI 10.2106/JBJS.D.03049
   Leppilahti J, 1996, ACTA ORTHOP SCAND, V67, P277, DOI 10.3109/17453679608994688
   Liberati A, 2009, ANN INTERN MED, V151, pW65, DOI [10.1371/journal.pmed.1000100, 10.7326/0003-4819-151-4-200908180-00136, 10.1136/bmj.b2700]
   Lim J, 2001, FOOT ANKLE INT, V22, P559, DOI 10.1177/107110070102200705
   Maffulli N, 1999, J BONE JOINT SURG AM, V81A, P1019, DOI 10.2106/00004623-199907000-00017
   Maffulli N, 2003, AM J SPORT MED, V31, P692, DOI 10.1177/03635465030310051001
   Majewski M, 2000, ORTHOPADE, V29, P670
   Mascarenhas R, 2015, ARTHROSCOPY, V31, P561, DOI 10.1016/j.arthro.2014.11.011
   McMahon Samuel E, 2011, Foot Ankle Surg, V17, P211, DOI 10.1016/j.fas.2010.11.001
   Porter KJ, 2014, FOOT ANKLE SURG, V20, P90, DOI 10.1016/j.fas.2013.11.005
   Schroeder D, 1997, AM ORTHOP SOC SPORT, V21, P1228
   Shea BJ, 2007, BMC MED RES METHODOL, V7, DOI 10.1186/1471-2288-7-10
   Shea BJ, 2009, J CLIN EPIDEMIOL, V62, P1013, DOI 10.1016/j.jclinepi.2008.10.009
   Shea BJ, 2007, PLOS ONE, V2, DOI 10.1371/journal.pone.0001350
   Wilkins R, 2012, AM J SPORT MED, V40, P2154, DOI 10.1177/0363546512453293
   Worth N, 2007, J Orthop Surg (Hong Kong), V15, P311
   Wright JG, 2003, J BONE JOINT SURG AM, V85A, P1, DOI 10.2106/00004623-200301000-00001
   Zhao JG, 2015, MEDICINE, V94, DOI 10.1097/MD.0000000000001057
   Zhao JG, 2015, MEDICINE, V94, DOI 10.1097/MD.0000000000000599
NR 30
TC 18
Z9 24
U1 2
U2 11
PU BMC
PI LONDON
PA CAMPUS, 4 CRINAN ST, LONDON N1 9XW, ENGLAND
SN 1749-799X
J9 J ORTHOP SURG RES
JI J. Orthop. Surg. Res.
PD JUN 6
PY 2016
VL 11
AR 65
DI 10.1186/s13018-016-0401-2
PG 7
WC Orthopedics
WE Science Citation Index Expanded (SCI-EXPANDED)
SC Orthopedics
GA DN7WL
UT WOS:000377290800001
PM 27266275
OA Green Published, gold
DA 2022-07-22
ER

PT J
AU Horstmann, T
   Lukas, C
   Merk, J
   Brauner, T
   Mundermann, A
AF Horstmann, T.
   Lukas, C.
   Merk, J.
   Brauner, T.
   Muendermann, A.
TI Deficits 10-Years after Achilles Tendon Repair
SO INTERNATIONAL JOURNAL OF SPORTS MEDICINE
LA English
DT Article
DE muscle strength; muscle strength endurance; muscle activity; Achilles
   tendon rupture
ID FUNCTIONAL TREATMENT; SURGICAL REPAIR; MUSCLE FUNCTION; GAIT PATTERN;
   RUPTURE; IMMOBILIZATION; WEIGHTBEARING; THERAPY
AB The purpose of this study was to determine the long-term impact of surgical repair and subsequent 6-week immobilization of an Achilles tendon rupture on muscle strength, muscle strength endurance and muscle activity. 63 patients participated in this study on average 10.8+/-3.4 years after surgically repaired Achilles tendon rupture and short-term immobilization. Clinical function was assessed and muscle strength, strength endurance and muscle activity were measured using a dynamometer and electromyography. Ankle ROM, heel height during heel-raise tests and calf circumference were smaller on the injured than on the contralateral side. Ankle torques during the concentric dorsiflexion tasks at 60 degrees/sec and 180 degrees/sec and ankle torques during the eccentric plantarflexion task and during the concentric plantarflexion task at 60 degrees/sec for the injured leg were significantly lower than those for the contralateral leg. The total work during a plantarflexion exercise at 180 degrees/sec was 14.9% lower in the injured compared to the contralateral leg (p<0.001). Muscle activity for the gastrocnemius muscle during dorsiflexion tasks was significantly higher in the injured than in the contralateral limb. Limited ankle joint ROM and increased muscle activity in the injured leg suggest compensatory mechanisms to account for differences in muscle morphology and physiology caused by the injury.
C1 [Horstmann, T.; Lukas, C.] Med Pk Bad Wiessee St Hubertus, D-83707 Bad Wiessee, Germany.
   [Horstmann, T.] Tech Univ Munich, Fac Sport & Hlth Sci, D-8000 Munich, Germany.
   [Merk, J.] Ctr Rehabil HESS, Bietigheim Bissingen, Germany.
   [Brauner, T.] BG Unfallklin, Tubingen, Germany.
   [Muendermann, A.] Univ Konstanz, Constance, Germany.
RP Horstmann, T (通讯作者)，Med Pk Bad Wiessee St Hubertus, Sonnenfeldweg 29, D-83707 Bad Wiessee, Germany.
EM t.horstmann@medicalpark.de
RI Brauner, Torsten/O-5832-2016; Mündermann, Annegret/C-9451-2011
OI Brauner, Torsten/0000-0002-2583-1121; Mündermann,
   Annegret/0000-0002-6472-1689; Horstmann, Thomas/0000-0002-8981-5956
CR Aktas S, 2009, FOOT ANKLE INT, V30, P391, DOI 10.3113/FAI.2009.0391
   Ames PRJ, 2008, DISABIL REHABIL, V30, P1646, DOI 10.1080/09638280701785882
   Bamman MM, 2000, MED SCI SPORT EXER, V32, P1307, DOI 10.1097/00005768-200007000-00019
   Don R, 2007, CLIN BIOMECH, V22, P211, DOI 10.1016/j.clinbiomech.2006.10.001
   HAGGMARK T, 1986, ORTHOPEDICS, V9, P160
   Haggmark T, 1979, Am J Sports Med, V7, P121, DOI 10.1177/036354657900700208
   Harriss DJ, 2011, INT J SPORTS MED, V32, P819, DOI 10.1055/s-0031-1287829
   HORSTMANN GA, 1988, ELECTROEN CLIN NEURO, V70, P447, DOI 10.1016/0013-4694(88)90022-3
   Horstmann T, 1999, INT J SPORTS MED, V20, P362, DOI 10.1055/s-2007-971145
   Jarvinen Tero A H, 2005, Foot Ankle Clin, V10, P255, DOI 10.1016/j.fcl.2005.01.013
   KADABA MP, 1989, J ORTHOPAED RES, V7, P849, DOI 10.1002/jor.1100070611
   Kangas J, 2003, J TRAUMA, V54, P1171, DOI 10.1097/01.TA.0000047945.20863.A2
   Khan RJK, 2005, J BONE JOINT SURG AM, V87A, P2202, DOI 10.2106/JBJS.D.03049
   Leppilahti J, 1996, INT J SPORTS MED, V17, P619, DOI 10.1055/s-2007-972905
   Leveau B, 1992, DHHS NIOSH PUBLICATI, P70
   Lill H, 1996, CHIRURG, V67, P1160, DOI 10.1007/s001040050119
   Maffulli N, 2003, AM J SPORT MED, V31, P692, DOI 10.1177/03635465030310051001
   Maffulli N, 1999, CLIN J SPORT MED, V9, P157, DOI 10.1097/00042752-199907000-00007
   Magnus CRA, 2010, J APPL PHYSIOL, V109, P1887, DOI 10.1152/japplphysiol.00597.2010
   Majewski M, 2008, DISABIL REHABIL, V30, P1726, DOI 10.1080/09638280701786831
   Moller A, 1996, ACTA ORTHOP SCAND, V67, P479, DOI 10.3109/17453679608996672
   Moller M, 2002, SCAND J MED SCI SPOR, V12, P9, DOI 10.1034/j.1600-0838.2002.120103.x
   Naim Fadel, 2005, Acta Orthop Traumatol Turc, V39, P1
   Neumann D, 1997, FOOT ANKLE INT, V18, P339, DOI 10.1177/107110079701800605
   Nilsson-Helander K, 2010, AM J SPORT MED, V38, P2186, DOI 10.1177/0363546510376052
   Olsson N, 2011, KNEE SURG SPORT TR A, V19, P1385, DOI 10.1007/s00167-011-1511-3
   Quagliarella L, 2010, FOOT ANKLE INT, V31, P306, DOI 10.3113/FAI.2010.0306
   Rippstein Pascal F, 2002, Foot Ankle Clin, V7, P611, DOI 10.1016/S1083-7515(02)00040-2
   Speck M, 1998, AM J SPORT MED, V26, P789, DOI 10.1177/03635465980260060901
   Suchak AA, 2008, J BONE JOINT SURG AM, V90A, P1876, DOI 10.2106/JBJS.G.01242
   VELDHUIZEN JW, 1993, INT J SPORTS MED, V14, P283, DOI 10.1055/s-2007-1021178
   Willits K, 2010, J BONE JOINT SURG AM, V92A, P2767, DOI 10.2106/JBJS.I.01401
   WINTER E, 1995, UNFALLCHIRURG, V98, P468
NR 33
TC 48
Z9 49
U1 0
U2 17
PU GEORG THIEME VERLAG KG
PI STUTTGART
PA RUDIGERSTR 14, D-70469 STUTTGART, GERMANY
SN 0172-4622
EI 1439-3964
J9 INT J SPORTS MED
JI Int. J. Sports Med.
PD JUN
PY 2012
VL 33
IS 6
BP 474
EP 479
DI 10.1055/s-0032-1301932
PG 6
WC Sport Sciences
WE Science Citation Index Expanded (SCI-EXPANDED)
SC Sport Sciences
GA 950AC
UT WOS:000304619500011
PM 22499571
DA 2022-07-22
ER

PT J
AU Apaydin, N
   Bozkurt, M
   Loukas, M
   Vefali, H
   Tubbs, RS
   Esmer, AF
AF Apaydin, Nihal
   Bozkurt, Murat
   Loukas, Marios
   Vefali, Huseng
   Tubbs, R. Shane
   Esmer, A. Firat
TI Relationships of the sural nerve with the calcaneal tendon: an
   anatomical study with surgical and clinical implications
SO SURGICAL AND RADIOLOGIC ANATOMY
LA English
DT Article
DE Achilles (calcaneal) tendon; Percutaneous Achilles (calcaneal) repair;
   Sural nerve injury
ID RUPTURED ACHILLES-TENDON; PERCUTANEOUS REPAIR; FUNCTIONAL
   AFTERTREATMENT; MANAGEMENT; INJURIES; FOOT
AB The percutaneous repair of the calcaneal tendon (CT) places the sural nerve (SN) at high risk for injury up to 60%. The aim of our study, therefore, was to explore and describe the course of SN in relation to the CT and to provide an anatomical description of the area in which the SN resides in order to assist surgeons in avoiding iatrogenic injury during surgical procedures in the leg. Forty-four lower extremities of 22 adult cadavers were dissected and the course of the sural nerve investigated. The CT was divided into ten horizontal equal fractions. The widths of CT, and horizontal distances of the SN and small saphenous vein (SSV) to a vertical line connecting the midpoints of these fractions were measured. All the measurements were obtained using a computer-assisted image analysis system. In 95.5% of the specimens the sural nerve was medial to the lateral border of the CT proximally and was intersecting with the lateral border of the CT at the 55% of the mid-tendon line. The SN divided into its terminal branches at a mean of 90% of the mid-tendon line. Based on our results, the course of the sural nerve is quite variable and seems to have the highest risk of injury at its proximal portion. The sutures placed on the CT distal to the 55% of the mid-tendon line may decrease iatrogenic nerve injury.
C1 [Apaydin, Nihal; Esmer, A. Firat] Ankara Univ, Dept Anat, Sch Med, TR-06100 Ankara, Turkey.
   [Apaydin, Nihal; Loukas, Marios; Vefali, Huseng] St Georges Univ, Sch Med, Dept Anat Sci, St Georges, Grenada.
   [Bozkurt, Murat] Diskapi Yildirim Beyazid Training & Res Hosp, Orthopaed & Traumatol Clin 3, Ankara, Turkey.
   [Loukas, Marios] Harvard Univ, Sch Med, Dept Educ & Dev, Boston, MA USA.
   [Tubbs, R. Shane] Univ Alabama Birmingham, Dept Cell Biol, Birmingham, AL 35294 USA.
RP Apaydin, N (通讯作者)，Ankara Univ, Dept Anat, Sch Med, TR-06100 Ankara, Turkey.
EM napaydin@gmail.com
RI Esmer, Ali Firat/A-9167-2013; Apaydin, Nihal/AAP-7181-2020
OI Esmer, Ali Firat/0000-0003-2511-9879; Apaydin, Nihal/0000-0002-7680-1766
CR ALDAM CH, 1989, J BONE JOINT SURG BR, V71, P486, DOI 10.1302/0301-620X.71B3.2722945
   BRADLEY JP, 1990, AM J SPORT MED, V18, P188, DOI 10.1177/036354659001800213
   Citak M, 2007, BRIT J SPORT MED, V41, P456, DOI 10.1136/bjsm.2006.031328
   Cretnik A, 2005, AM J SPORT MED, V33, P1369, DOI 10.1177/0363546504271501
   Flavin R, 2007, INJURY, V38, P845, DOI 10.1016/j.injury.2006.10.016
   Halasi T, 2003, KNEE SURG SPORT TR A, V11, P409, DOI 10.1007/s00167-003-0397-0
   HOCKENBURY RT, 1990, FOOT ANKLE, V11, P67, DOI 10.1177/107110079001100202
   Jennings AG, 2004, ANN ROY COLL SURG, V86, P445, DOI 10.1308/147870804957
   Jung HG, 2008, FOOT ANKLE INT, V29, P803, DOI 10.3113/FAI.2008.0803
   KLEIN W, 1991, Chirurgia degli Organi di Movimento, V76, P223
   Lansdaal JR, 2007, INJURY, V38, P839, DOI 10.1016/j.injury.2006.12.010
   LAWRENCE SJ, 1994, FOOT ANKLE INT, V15, P490, DOI 10.1177/107110079401500906
   Lo IKY, 1997, CLIN J SPORT MED, V7, P207, DOI 10.1097/00042752-199707000-00010
   Loukas M, 2005, CLIN ANAT, V18, P357, DOI 10.1002/ca.20112
   MA GWC, 1977, CLIN ORTHOP RELAT R, P247
   Madhavi C, 2005, CLIN ANAT, V18, P206, DOI 10.1002/ca.20094
   Maes Renaud, 2006, Acta Orthop Belg, V72, P179
   Maffulli N, 1999, J BONE JOINT SURG AM, V81A, P1019, DOI 10.2106/00004623-199907000-00017
   Maffulli N, 1999, CLIN J SPORT MED, V9, P157, DOI 10.1097/00042752-199907000-00007
   Mahakkanukrauh P, 2002, CLIN ANAT, V15, P263, DOI 10.1002/ca.10016
   Majewski M, 2006, AM J SPORT MED, V34, P793, DOI 10.1177/0363546505283266
   McClelland D, 2002, J ROY COLL SURG EDIN, V47, P613
   Paavola M, 2000, AM J SPORT MED, V28, P77, DOI 10.1177/03635465000280012501
   Pavic R, 2008, INJURY, V39, P499, DOI 10.1016/j.injury.2007.11.423
   ROWLEY DI, 1982, INJURY, V14, P252, DOI 10.1016/0020-1383(82)90138-3
   Salamon ML, 2006, FOOT ANKLE INT, V27, P411, DOI 10.1177/107110070602700604
   Wallace RGH, 2004, J BONE JOINT SURG AM, V86A, P1198, DOI 10.2106/00004623-200406000-00011
   Webb J, 2000, FOOT ANKLE INT, V21, P475, DOI 10.1177/107110070002100604
   Webb JM, 1999, J BONE JOINT SURG BR, V81B, P877, DOI 10.1302/0301-620X.81B5.9784
   WILLIAMS A, 2005, GRAYS ANATOMY ANATOM, P1503
   Wong J, 2002, AM J SPORT MED, V30, P565, DOI 10.1177/03635465020300041701
NR 31
TC 16
Z9 17
U1 0
U2 5
PU SPRINGER FRANCE
PI PARIS
PA 22 RUE DE PALESTRO, PARIS, 75002, FRANCE
SN 0930-1038
EI 1279-8517
J9 SURG RADIOL ANAT
JI Surg. Radiol. Anat.
PD DEC
PY 2009
VL 31
IS 10
BP 775
EP 780
DI 10.1007/s00276-009-0520-0
PG 6
WC Anatomy & Morphology; Radiology, Nuclear Medicine & Medical Imaging;
   Surgery
WE Science Citation Index Expanded (SCI-EXPANDED)
SC Anatomy & Morphology; Radiology, Nuclear Medicine & Medical Imaging;
   Surgery
GA 535WE
UT WOS:000273001800006
PM 19479178
DA 2022-07-22
ER

PT J
AU Rosso, C
   Buckland, DM
   Polzer, C
   Sadoghi, P
   Schuh, R
   Weisskopf, L
   Vavken, P
   Valderrabano, V
AF Rosso, Claudio
   Buckland, Daniel M.
   Polzer, Caroline
   Sadoghi, Patrick
   Schuh, Reinhard
   Weisskopf, Lukas
   Vavken, Patrick
   Valderrabano, Victor
TI Long-term biomechanical outcomes after Achilles tendon ruptures
SO KNEE SURGERY SPORTS TRAUMATOLOGY ARTHROSCOPY
LA English
DT Article
DE Peak plantar flexion torque; COP; Achilles tendon rupture; Plantar
   pressure distribution (PPD)
ID SURGICAL REPAIR; NONOPERATIVE TREATMENT; FUNCTIONAL TREATMENT;
   FOLLOW-UP; STRENGTH; LENGTH; REHABILITATION; VOLUME
AB The ideal treatment for Achilles tendon ruptures is still unknown. Biomechanical were correlated to radiological and clinical parameters to study outcomes.
   In this retrospective, assessor-blinded multi-centre cohort study, 52 patients with unilateral Achilles tendon rupture were assessed, each at least 3 years after injury. Patients underwent open surgery, percutaneous surgery or non-surgical treatment of Achilles tendon rupture. Both legs underwent plantar pressure distribution and isokinetic measures. Demographic parameters, maximum calf circumference (MCC) and clinical scores (American Orthopaedic Foot and Ankle Society, Achilles tendon rupture score, Hannover) were also evaluated. Complications were not assessed.
   Peak plantar flexion torque (PPFT) was significantly weaker on the treated side compared to the untreated leg [80.4 +/- A 29.7 Nm (mean +/- A SD) vs. 92.1 +/- A 27.4 Nm, p < 0.0001]. PPFT and push-off force (POFF) were not different between treatment groups nor was there a leg difference in POFF alone. There was only a weak correlation of clinical scores and PPFT or POFF, respectively. MCC correlated significantly with both PPFT (R (2) = 0.21, p = 0.01) and POFF (R (2) = 0.29, p < 0.0001). POFF appeared to be a predictor of PPFT (R (2) = 0.31, p < 0.0001). Open surgery outperformed non-surgical treatment in terms of centre-of-pressure line (p = 0.007), torque per muscle volume (p = 0.04) and relative POFF per body weight (p = 0.02) and relative in side comparison (p = 0.03).
   Clinical scores do not predict biomechanical outcomes. Clinically measured MCC is a good predictor of PPFT and POFF and can easily be used in clinical practice. Relative POFF in side comparison as well as per body weight favours surgical treatment.
C1 [Rosso, Claudio; Buckland, Daniel M.; Polzer, Caroline; Vavken, Patrick; Valderrabano, Victor] Univ Basel, Univ Basel Hosp, Dept Orthopaed, CH-4031 Basel, Switzerland.
   [Sadoghi, Patrick] Med Univ Graz, Dept Orthopaed Surg, Graz, Austria.
   [Schuh, Reinhard] Med Univ Vienna, Vienna Gen Hosp, Dept Orthopaed, Vienna, Austria.
   [Weisskopf, Lukas] Rennbahn Clin, Muttenz, Switzerland.
   [Vavken, Patrick] Harvard Univ, Sch Publ Hlth, Harvard Ctr Populat & Dev Studies, Boston, MA 02115 USA.
   [Vavken, Patrick] Harvard Univ, Sch Med, Orthoped, Childrens Hosp Boston, Boston, MA USA.
RP Rosso, C (通讯作者)，Univ Basel, Univ Basel Hosp, Dept Orthopaed, Spitalstr 21, CH-4031 Basel, Switzerland.
EM Claudio.Rosso@unibas.ch
RI Buckland, Daniel/S-1266-2019
OI Buckland, Daniel/0000-0001-5274-3840
FU Swiss National Accident Insurance Company (SUVA), Lucerne, Switzerland;
   Whitaker International Program
FX We thank Xavier Kaelin MSc and Marlene Mauch PhD for their biomechanical
   studies and their steady effort in patient examinations. From the
   Department of Radiology, we would like to thank Dr. Ueli Studler for his
   help in the radiologic workup and Tanja Haas for her MRI examinations.
   We thank Professor Beat Hintermann, Dr. Marc Lottenbach and Dr. Martin
   Majewski, for their contribution in patient acquisition. The lead centre
   of this study was the University Hospital Basel, Basel, Switzerland.
   This multi-centre study was funded by a grant from the Swiss National
   Accident Insurance Company (SUVA), Lucerne, Switzerland. DMB is funded
   by a post-doctoral fellowship from the Whitaker International Program.
CR Atik OS, 2011, EKLEM HAST CERRAHISI, V22, P54
   CARTER TR, 1992, AM J SPORT MED, V20, P459, DOI 10.1177/036354659202000417
   CETTI R, 1994, CLIN ORTHOP RELAT R, P155
   Chan Alexander Pak-Hin, 2011, Sports Med Arthrosc Rehabil Ther Technol, V3, P32, DOI 10.1186/1758-2555-3-32
   FITZGIBBONS RE, 1993, AM J SPORT MED, V21, P724, DOI 10.1177/036354659302100516
   GILLIES H, 1970, J BONE JOINT SURG AM, VA 52, P337, DOI 10.2106/00004623-197052020-00015
   HAGGMARK T, 1986, ORTHOPEDICS, V9, P160
   INGLIS AE, 1976, J BONE JOINT SURG AM, V58, P990, DOI 10.2106/00004623-197658070-00015
   Jacobs D, 1978, Am J Sports Med, V6, P107, DOI 10.1177/036354657800600302
   Khan RJK, 2010, COCHRANE DB SYST REV, DOI 10.1002/14651858.CD003674.pub4
   Khan RJK, 2005, J BONE JOINT SURG AM, V87A, P2202, DOI 10.2106/JBJS.D.03049
   KITAOKA HB, 1994, FOOT ANKLE INT, V15, P349, DOI 10.1177/107110079401500701
   Klein P, 1996, J BIOMECH, V29, P21, DOI 10.1016/0021-9290(95)00025-9
   Krapf D, 2012, ORTHOPEDICS, V35, pE1383, DOI 10.3928/01477447-20120822-26
   Lephart SM, 2002, CLIN ORTHOP RELAT R, P162
   Leppilahti J, 2000, FOOT ANKLE INT, V21, P330, DOI 10.1177/107110070002100410
   Maganaris CN, 2008, DISABIL REHABIL, V30, P1542, DOI 10.1080/09638280701785494
   Magnusson SP, 2008, J PHYSIOL-LONDON, V586, P71, DOI 10.1113/jphysiol.2007.139105
   Maughan R J, 1984, Br J Sports Med, V18, P149
   McComis GP, 1997, J BONE JOINT SURG AM, V79A, P1799, DOI 10.2106/00004623-199712000-00005
   Metz Roderik, 2009, Foot Ankle Spec, V2, P219, DOI 10.1177/1938640009348338
   Mezzarobba S, 2012, Foot (Edinb), V22, P303, DOI 10.1016/j.foot.2012.09.001
   Moller M, 2001, J BONE JOINT SURG BR, V83B, P843
   Mortensen NHM, 1999, J BONE JOINT SURG AM, V81A, P983, DOI 10.2106/00004623-199907000-00011
   Mullaney MJ, 2006, INT J SPORTS MED, V27, P725, DOI 10.1055/s-2005-872870
   Mullaney MJ, 2006, AM J SPORT MED, V34, P1120, DOI 10.1177/0363546505284186
   Nilsson-Helander K, 2007, AM J SPORT MED, V35, P421, DOI 10.1177/0363546506294856
   Olsson N, 2013, AM J SPORT MED, V41, P2867, DOI 10.1177/0363546513503282
   Olsson N, 2011, KNEE SURG SPORT TR A, V19, P1385, DOI 10.1007/s00167-011-1511-3
   Rebeccato A, 2001, J Foot Ankle Surg, V40, P188
   Rettig AC, 2005, AM J SPORT MED, V33, P119, DOI 10.1177/0363546504268720
   Rosso C, 2013, KNEE SURG SPORT TR A, V21, P1369, DOI 10.1007/s00167-013-2407-1
   Rosso C, 2012, CLIN J SPORT MED, V22, P483, DOI 10.1097/JSM.0b013e3182639a3e
   Sadoghi P, 2012, INT ORTHOP, V36, P1947, DOI 10.1007/s00264-012-1533-6
   Shields C L Jr, 1978, Am J Sports Med, V6, P369, DOI 10.1177/036354657800600610
   Speck M, 1998, AM J SPORT MED, V26, P789, DOI 10.1177/03635465980260060901
   Starkey DB, 1996, MED SCI SPORT EXER, V28, P1311, DOI 10.1097/00005768-199610000-00016
   THERMANN H, 1995, UNFALLCHIRURG, V98, P21
   WASHBURN SD, 1992, CLIN ORTHOP RELAT R, P237
   Willits K, 2010, J BONE JOINT SURG AM, V92A, P2767, DOI 10.2106/JBJS.I.01401
NR 40
TC 29
Z9 32
U1 0
U2 15
PU SPRINGER
PI NEW YORK
PA 233 SPRING ST, NEW YORK, NY 10013 USA
SN 0942-2056
EI 1433-7347
J9 KNEE SURG SPORT TR A
JI Knee Surg. Sports Traumatol. Arthrosc.
PD MAR
PY 2015
VL 23
IS 3
BP 890
EP 898
DI 10.1007/s00167-013-2726-2
PG 9
WC Orthopedics; Sport Sciences; Surgery
WE Science Citation Index Expanded (SCI-EXPANDED)
SC Orthopedics; Sport Sciences; Surgery
GA CC2XU
UT WOS:000350209300031
PM 24162761
OA Green Submitted
DA 2022-07-22
ER

PT J
AU Karatekin, YS
   Karaismailoglu, B
   Kaynak, G
   Ogut, T
   Dikici, AS
   Esmerer, EU
   Aydingoz, O
   Botanlioglu, H
AF Karatekin, Yavuz Selim
   Karaismailoglu, Bedri
   Kaynak, Gokhan
   Ogut, Tahir
   Dikici, Atilla Suleyman
   Esmerer, Emel Ure
   Aydingoz, Onder
   Botanlioglu, Huseyin
TI Does elasticity of Achilles tendon change after suture applications?
   Evaluation of repair area by acoustic radiation force impulse
   elastography
SO JOURNAL OF ORTHOPAEDIC SURGERY AND RESEARCH
LA English
DT Article
DE Achilles tendon rupture; Surgical repair; Modified Kessler method;
   Krackow method; Acoustic radiation force impulse; Elastography;
   Elasticity; Shear wave velocity
ID RUPTURE; AGE
AB Background: Achilles tendon injuries are one of the most common tendon injuries. Surgical treatment is preferred in young and active patients. Although there are studies which evaluate the repair area with magnetic resonance imaging and ultrasonography after surgical treatment, there are very few studies which analyzes the elasticity of the tendon by quantitative methods. ARFI (acoustic radiation force impulse) elastography is a simple and non-invasive method that can quantitatively measure the elasticity of the soft tissues. Our study aims to evaluate the elasticity in the repair area of the surgically treated Achilles tendons, compare them to the non-injured side, and evaluate the effect of the suture method to the elasticity of the repaired tendons by using ARFI elastography.
   Methods: In our retrospectively designed study, 19 patients who underwent surgical treatment with Krackow and modified Kessler suture methods after the Achilles tendon rupture between 2006 and 2014 were included. Shear wave velocity (SWV) of the repaired and non-injured Achilles tendons were measured by ARFI elastography in four different positions of the ankle.
   Results: It was determined that SWV in the surgically repaired tendons were significantly higher in each four different position of the ankle, compared to the non-injured side (p < 0.01), indicating less elasticity in the repaired tendons. There was no statistically significant difference between the SWV of Krackow and modified Kessler suture method groups at four different positions of the ankle (p > 0.05). AOFAS Ankle-Hindfoot, VISA-A, VAS, and FAOS scores were not also statistically different between these two suture methods (p > 0.05).
   Conclusions: In the repaired Achilles tendon, there is a decrease in the elasticity compared to the non-injured side. The functional and elastographic results of Krackow and modified Kessler suture methods are similar in long-term follow-ups of the patients.
C1 [Karatekin, Yavuz Selim; Karaismailoglu, Bedri; Kaynak, Gokhan; Ogut, Tahir; Aydingoz, Onder; Botanlioglu, Huseyin] Istanbul Univ, Dept Orthopaed & Traumatol, Cerrahpasa Med Fac, Kocamustafapasa Cad 53, Istanbul, Turkey.
   [Dikici, Atilla Suleyman; Esmerer, Emel Ure] Istanbul Univ, Cerrahpasa Med Fac, Dept Radiol, Kocamustafapasa Cad 53, Istanbul, Turkey.
RP Botanlioglu, H (通讯作者)，Istanbul Univ, Dept Orthopaed & Traumatol, Cerrahpasa Med Fac, Kocamustafapasa Cad 53, Istanbul, Turkey.
EM huseyinbotanlioglu@gmail.com
RI Botanlioglu, Huseyin/AAH-1100-2019; kaynak, gokhan/C-8041-2019;
   AYDINGOZ, ONDER/AAH-1019-2019; Karatekin, Yavuz Selim/AAK-9271-2021;
   Karaismailoglu, Bedri/E-3333-2013
OI kaynak, gokhan/0000-0002-4369-9195; Karatekin, Yavuz
   Selim/0000-0002-2460-4648; Karaismailoglu, Bedri/0000-0002-4565-6383;
   AYDINGOZ, ONDER/0000-0002-0219-2232; Botanlioglu,
   Huseyin/0000-0001-7997-995X
CR Aktas S, 2009, FOOT ANKLE INT, V30, P391, DOI 10.3113/FAI.2009.0391
   Bhattacharyya M, 2009, INT ORTHOP, V33, P151, DOI 10.1007/s00264-008-0564-5
   Cash MP, 2012, ANN PLAS SURG, V68, P583, DOI 10.1097/SAP.0b013e31821b6a0b
   Cretnik A, 2005, AM J SPORT MED, V33, P1369, DOI 10.1177/0363546504271501
   Del Buono A, 2014, BRIT MED BULL, V109, P45, DOI 10.1093/bmb/ldt029
   Dirrichs T, 2017, ACAD RADIOL, P30400
   Fu S, 2016, J ULTRAS MED, V35, P1881, DOI 10.7863/ultra.16.01052
   Harvey L, 2000, J Foot Ankle Surg, V39, P232
   HOCKENBURY RT, 1990, FOOT ANKLE, V11, P67, DOI 10.1177/107110079001100202
   Hsu AR, 2015, FOOT ANKLE INT, V36, P1279, DOI 10.1177/1071100715589632
   JESSING P, 1975, ACTA CHIR SCAND, V141, P370
   JOZSA L, 1989, AM J SPORT MED, V17, P338, DOI 10.1177/036354658901700305
   Maffulli N, 2000, AM J SPORT MED, V28, P499, DOI 10.1177/03635465000280040901
   McCoy BW, 2010, FOOT ANKLE INT, V31, P701, DOI 10.3113/FAI.2010.0701
   Ruan ZM, 2015, INT J CLIN EXP MED, V8, P1043
   Sharma P, 2005, J BONE JOINT SURG AM, V87A, P187, DOI 10.2106/JBJS.D.01850
   Shinohara M, 2010, MUSCLE NERVE, V42, P438, DOI 10.1002/mus.21723
   Soroceanu A, 2012, J BONE JOINT SURG AM, V94A, P2136, DOI 10.2106/JBJS.K.00917
   Tavares WC, 2014, J ORTHOP SURG RES, V9, DOI 10.1186/s13018-014-0132-1
   Turan A, 2015, MED ULTRASON, V17, P58, DOI 10.11152/mu.2013.2066.171.ayt
   Yammine Kaissar, 2017, Foot (Edinb), V30, P13, DOI 10.1016/j.foot.2016.09.006
   Yang TH, 2015, J STRUCT BIOL, V192, P59, DOI 10.1016/j.jsb.2015.08.012
   Zhang LN, 2016, SKELETAL RADIOL, V17, P30
NR 23
TC 5
Z9 7
U1 0
U2 6
PU BIOMED CENTRAL LTD
PI LONDON
PA 236 GRAYS INN RD, FLOOR 6, LONDON WC1X 8HL, ENGLAND
SN 1749-799X
J9 J ORTHOP SURG RES
JI J. Orthop. Surg. Res.
PD MAR 2
PY 2018
VL 13
AR 45
DI 10.1186/s13018-018-0751-z
PG 7
WC Orthopedics
WE Science Citation Index Expanded (SCI-EXPANDED)
SC Orthopedics
GA FY3CY
UT WOS:000426696400004
PM 29499741
OA Green Published, gold
DA 2022-07-22
ER

PT J
AU Morimoto, S
   Iseki, T
   Nakayama, H
   Shimomura, K
   Nishikawa, T
   Nakamura, N
   Tachibana, T
AF Morimoto, Shota
   Iseki, Tomoya
   Nakayama, Hiroshi
   Shimomura, Kazunori
   Nishikawa, Tetsuo
   Nakamura, Norimasa
   Tachibana, Toshiya
TI Return to the original sport at only 3 months after an Achilles tendon
   rupture by a combination of intra-tissue injection of freeze-dried
   platelet-derived factor concentrate and excessively early rehabilitation
   after operative treatment in a male basketball player: A case report
SO REGENERATIVE THERAPY
LA English
DT Article
DE Achilles tendon rupture; Operative treatment; Early rehabilitation;
   Freeze-dried platelet-derived factor concentrate; Platelet-rich plasma;
   Platelet-derived growth factor
ID LOOP SUTURE TECHNIQUE; GROWTH-FACTORS; RICH PLASMA; PDGF-BB;
   METAANALYSIS; IMMOBILIZATION; PROLIFERATION; OPTIMIZATION; MOBILIZATION;
   MANAGEMENT
AB Background: Achilles tendon rupture is one of the most common serious injuries in athletes. Various studies to accelerate the healing process of the Achilles tendon have been performed as it takes a longer time to repair the tissue compared to other tendons. Here, we report a case of an acute Achilles tendon rupture in a male basketball player treated by a combination of an intra-tissue injection of freeze-dried platelet-derived factor concentrate, which included a platelet-derived growth factor with an early rehabilitation protocol after the operative treatment to facilitate the biological healing of the injured tendon tissue. To the best of our knowledge, this case is the first instance that enabled the athlete to return to original sport activity at only 3-months after the injury.
   Case report: A23-year-old male basketball player who belonged to a university basketball team sustained an Achilles tendon rupture during running in a training match. The remaining time period until the final tournament of the university league as a senior player was only 3 months. The patient received a combination o f an intra-tissue injection of freeze-dried platelet-derived factor concentrate and early rehabilitation protocol afteroperative treatment. Surgery was performed 4 days after the injury and the early rehabilitation protocols were applied postoperatively. A freeze-dried platelet-derived factor concentrate was injected into the ruptured site of the Achilles tendon under ultrasound guide at 4 weeks postoperatively. The patient could return to play at the pre-injury level without any symptoms and disfunctions at 3 months after surgery. At two years postoperatively, the patient could play basketball without symptoms or rerupture.
   Conclusions: We reported a case of an Achilles tendon rupture which was treated by a combination of intra-tissue injection of freeze-dried platelet-derived factor concentrate and an early rehabilitation protocol after the operative treatment. The patient could return to play basketball at the pre-injury activity level at only 3-months after the injury, suggesting that the role of applying excessively early rehabilitation of mechanical loading could facilitate tendon tissue healing when combined with an intratissue injection of freeze-dried platelet-derived factor concentrate. (C) 2021, The Japanese Society for Regenerative Medicine. Production and hosting by Elsevier B.V.
C1 [Morimoto, Shota; Iseki, Tomoya; Nakayama, Hiroshi; Tachibana, Toshiya] Hyogo Coll Med, Dept Orthopaed Surg, 1-1 Mukogawa Cho, Nishinomiya, Hyogo 6638501, Japan.
   [Shimomura, Kazunori; Nakamura, Norimasa] Osaka Univ, Dept Orthopaed Surg, Grad Sch Med, Osaka, Japan.
   [Nishikawa, Tetsuo] Nishikawa Orthopaed & Rehabil Clin, 1-1-4 Souhon Machi, Amagasaki, Hyogo 6610031, Japan.
   [Nakamura, Norimasa] Osaka Hlth Sci Univ, Inst Med Sci Sports, Kita Ku, 1-9-27 Tenma, Osaka, Osaka 5300043, Japan.
   [Nakamura, Norimasa] Osaka Univ, Global Ctr Med Engn & Informat, 2-2 Yamadaoka, Suita, Osaka 5650871, Japan.
RP Iseki, T (通讯作者)，1-1 Mukogawa Cho, Nishinomiya, Hyogo 6638501, Japan.
EM iseki@hyo-med.ac.jp
RI Iseki, Tomoya/AAK-2114-2021
CR Anitua E, 2005, J ORTHOP RES, V23, P281, DOI 10.1016/j.orthres.2004.08.015
   Araki J, 2012, TISSUE ENG PART C-ME, V18, P176, DOI [10.1089/ten.tec.2011.0308, 10.1089/ten.TEC.2011.0308]
   Banes AJ, 1995, J BIOMECH, V28, P1505, DOI 10.1016/0021-9290(95)00098-4
   Braunstein M, 2018, KNEE SURG SPORT TR A, V26, P846, DOI 10.1007/s00167-015-3795-1
   Brumann M, 2014, INJURY, V45, P1782, DOI 10.1016/j.injury.2014.06.022
   Chan BP, 2000, ACTA ORTHOP SCAND, V71, P513, DOI 10.1080/000164700317381234
   Costa MA, 2006, TISSUE ENG, V12, P1937, DOI 10.1089/ten.2006.12.1937
   De Carli A, 2016, KNEE SURG SPORT TR A, V24, P2231, DOI 10.1007/s00167-015-3580-1
   El-Akkawi AI, 2018, J FOOT ANKLE SURG, V57, P346, DOI 10.1053/j.jfas.2017.06.006
   Evrova O, 2017, EUR CELLS MATER, V34, P15, DOI 10.22203/eCM.v034a02
   Evrova O, 2020, INT J MOL SCI, V21, DOI 10.3390/ijms21020458
   Filardo G, 2018, KNEE SURG SPORT TR A, V26, P1984, DOI 10.1007/s00167-016-4261-4
   Frank C. B., 2004, Journal of Musculoskeletal & Neuronal Interactions, V4, P199
   Gulati V, 2015, WORLD J ORTHOP, V6, P380, DOI 10.5312/wjo.v6.i4.380
   Imade S, 2013, J FOOT ANKLE SURG, V52, P553, DOI 10.1053/j.jfas.2013.03.036
   Jiang N, 2012, INT ORTHOP, V36, P765, DOI 10.1007/s00264-011-1431-3
   Jones Matthew P, 2012, J Bone Joint Surg Am, V94, pe88, DOI 10.2106/JBJS.J.01829
   Kim SG, 2002, CELL STRUCT FUNCT, V27, P139, DOI 10.1247/csf.27.139
   Lantto I, 2015, SCAND J MED SCI SPOR, V25, pE133, DOI 10.1111/sms.12253
   Lyras DN, 2009, FOOT ANKLE INT, V30, P1101, DOI 10.3113/FAI.2009.1101
   Maffulli N, 2018, SPORTS MED ARTHROSC, V26, P41, DOI 10.1097/JSA.0000000000000186
   McCormack R, 2015, BRIT J SPORT MED, V49, P1329, DOI 10.1136/bjsports-2015-094935
   Miyamoto W, 2017, FOOT ANKLE INT, V38, P167, DOI 10.1177/1071100716673589
   Molloy T, 2003, SPORTS MED, V33, P381, DOI 10.2165/00007256-200333050-00004
   Palmes D, 2002, J ORTHOPAED RES, V20, P939, DOI 10.1016/S0736-0266(02)00032-3
   Pan L, 2016, AESTHET PLAST SURG, V40, P157, DOI 10.1007/s00266-015-0580-y
   Pneumaticos SG, 2000, FOOT ANKLE INT, V21, P551, DOI 10.1177/107110070002100704
   Qiu YW, 2014, J TISSUE ENG REGEN M, V8, P955, DOI 10.1002/term.1597
   Raghavan SS, 2012, PLAST RECONSTR SURG, V129, P479, DOI 10.1097/PRS.0b013e31823aeb94
   Sanchez M, 2007, AM J SPORT MED, V35, P245, DOI 10.1177/0363546506294078
   Schepull T, 2011, AM J SPORT MED, V39, P38, DOI 10.1177/0363546510383515
   Shi LY, 2019, BLOOD COAGUL FIBRIN, V30, P58, DOI 10.1097/MBC.0000000000000796
   Shirata T, 2019, REGEN THER, V11, P5, DOI 10.1016/j.reth.2019.03.005
   Soroceanu A, 2012, J BONE JOINT SURG AM, V94A, P2136, DOI 10.2106/JBJS.K.00917
   Virchenko O, 2006, ACTA ORTHOP, V77, P806, DOI 10.1080/17453670610013033
   Zellers JA, 2016, BRIT J SPORT MED, V50, P1325, DOI 10.1136/bjsports-2016-096106
NR 36
TC 1
Z9 1
U1 1
U2 15
PU ELSEVIER
PI AMSTERDAM
PA RADARWEG 29, 1043 NX AMSTERDAM, NETHERLANDS
SN 2352-3204
J9 REGEN THER
JI Regen. Ther.
PD DEC
PY 2021
VL 18
BP 112
EP 116
DI 10.1016/j.reth.2021.05.002
PG 5
WC Cell & Tissue Engineering; Engineering, Biomedical
WE Science Citation Index Expanded (SCI-EXPANDED)
SC Cell Biology; Engineering
GA WA7TW
UT WOS:000703088900016
PM 34141835
OA gold, Green Published
DA 2022-07-22
ER

PT J
AU Boesen, AP
   Boesen, MI
   Hansen, R
   Barfod, KW
   Lenskjold, A
   Malliaras, P
   Langberg, H
AF Boesen, Anders Ploug
   Boesen, Morten Ilum
   Hansen, Rudi
   Barfod, Kristoffer Weisskirchner
   Lenskjold, Anders
   Malliaras, Peter
   Langberg, Henning
TI Effect of Platelet-Rich Plasma on Nonsurgically Treated Acute Achilles
   Tendon Ruptures: A Randomized, Double-Blinded Prospective Study
SO AMERICAN JOURNAL OF SPORTS MEDICINE
LA English
DT Article
DE platelet-rich plasma; Achilles tendon rupture; ATRS; heel-rise test;
   tendon elongation
ID GROWTH-FACTOR-I; NONOPERATIVE TREATMENT; COLLAGEN-SYNTHESIS; BADMINTON
   PLAYERS; TENDINOPATHY; RELIABILITY; INJECTION; DEFICITS; VOLUME; ANKLE
AB Background:
   An acute Achilles tendon rupture (ATR) is a long-lasting and devastating injury. Possible biological augmentation to promote and strengthen tendon healing after an ATR would be desirable.
   Purpose:
   To determine whether the application of a platelet-rich plasma (PRP) injection in nonsurgically treated ATRs may promote healing and thereby improve functional outcomes.
   Study Design:
   Randomized controlled trial; Level of evidence, 2.
   Methods:
   A total of 40 men (aged 18-60 years) with an ATR incurred within 72 hours were included, and 38 were followed for 12 months. All patients were treated with an orthosis with 3 wedges for 8 weeks; full weightbearing from day 1 was allowed, combined with either 4 PRP or 4 placebo injections (a few drops of saline, <0.5 mL, under the skin) 14 days apart. All patients received the same instructions on an exercise program starting from week 9. Outcomes included the self-reported Achilles tendon Total Rupture Score (ATRS) as well as heel-rise work, heel-rise height, tendon elongation, calf circumference, and ankle dorsiflexion range of motion.
   Results:
   The mean ATRS score improved in both groups at all time points (P < .001), but there was no difference between the groups at any time points (12 months: 90.1 points in PRP group and 88.8 points in placebo group). No differences in all functional outcomes at any time points were seen between the groups. At 12 months, the injured leg did not reach normal functional values compared with the uninjured leg.
   Conclusion:
   The application of PRP in nonsurgically treated ATRs did not appear to show any superior clinical and functional improvement.
   Registration:
   NCT02417922 (ClinicalTrials.gov identifier)
C1 [Boesen, Anders Ploug; Boesen, Morten Ilum; Hansen, Rudi; Barfod, Kristoffer Weisskirchner; Lenskjold, Anders; Malliaras, Peter; Langberg, Henning] Bispebjerg Hosp, Inst Sports Med, Dept Orthopaed Surg M, Bispebjerg Bakke 23, DK-2400 Copenhagen, Denmark.
   [Boesen, Anders Ploug; Hansen, Rudi; Lenskjold, Anders] Univ Copenhagen, Fac Hlth & Med Sci, Ctr Hlth Aging, Copenhagen, Denmark.
   [Boesen, Anders Ploug; Barfod, Kristoffer Weisskirchner] Hvidovre Univ Hosp, Dept Orthopaed Surg, Hvidovre, Denmark.
   [Boesen, Morten Ilum] Koge Sygehus, Dept Orthopaed, Koge, Denmark.
   [Malliaras, Peter] Monash Univ, Fac Med Nursing & Hlth Sci, Fac Med, Sch Primary & Allied Hlth Care,Dept Physiotherapy, Melbourne, Vic, Australia.
   [Langberg, Henning] Univ Copenhagen, Fac Hlth & Med Sci, Dept Publ Hlth, CopenRehab,Sect Social Med, Copenhagen, Denmark.
RP Boesen, AP (通讯作者)，Bispebjerg Hosp, Inst Sports Med, Dept Orthopaed Surg M, Bispebjerg Bakke 23, DK-2400 Copenhagen, Denmark.
EM boesenanders@hotmail.com
RI Barfod, Kristoffer/AAY-3288-2021
OI Barfod, Kristoffer/0000-0003-2620-5891; Hansen, Rudi/0000-0002-2313-8745
CR Andia I, 2010, EXPERT OPIN BIOL TH, V10, P1415, DOI 10.1517/14712598.2010.514603
   Barfod KW, 2013, J FOOT ANKLE SURG, V52, P629, DOI 10.1053/j.jfas.2013.04.012
   Barfod KW, 2015, KNEE SURG SPORT TR A, V23, P3398, DOI 10.1007/s00167-014-3175-2
   Barfod KW, 2014, J BONE JOINT SURG AM, V96A, P1497, DOI 10.2106/JBJS.M.01273
   Boesen AP, 2017, AM J SPORT MED, V45, P2034, DOI 10.1177/0363546517702862
   Boesen AP, 2012, AM J SPORT MED, V40, P548, DOI 10.1177/0363546511435478
   Boesen AP, 2011, KNEE SURG SPORT TR A, V19, P30, DOI 10.1007/s00167-010-1208-z
   Boesen MI, 2006, AM J SPORT MED, V34, P2013, DOI 10.1177/0363546506290188
   de Vos RJ, 2010, JAMA-J AM MED ASSOC, V303, P144, DOI 10.1001/jama.2009.1986
   Eliasson P, 2018, AM J SPORT MED, V46, P2492, DOI 10.1177/0363546518781826
   Fitzpatrick J, 2017, ORTHOP J SPORTS MED, V5, DOI 10.1177/2325967116675272
   Ganestam A, 2013, J FOOT ANKLE SURG, V52, P736, DOI 10.1053/j.jfas.2013.07.004
   Hall MP, 2009, J AM ACAD ORTHOP SUR, V17, P602, DOI 10.5435/00124635-200910000-00002
   Hansen M, 2013, SCAND J MED SCI SPOR, V23, P614, DOI 10.1111/j.1600-0838.2011.01431.x
   Holm C, 2015, SCAND J MED SCI SPOR, V25, pE1, DOI 10.1111/sms.12209
   Jeong DU, 2014, BIOMED RES INT, V2014, DOI 10.1155/2014/249498
   Kampa RJ, 2010, INT J CLIN PRACT, V64, P1813, DOI 10.1111/j.1742-1241.2010.02432.x
   Kaniki N, 2014, ARTHROSCOPY, V30, P1139, DOI 10.1016/j.arthro.2014.04.086
   Khan RJK, 2010, COCHRANE DB SYST REV, DOI 10.1002/14651858.CD003674.pub4
   Kjaer M, 2009, SCAND J MED SCI SPOR, V19, P500, DOI 10.1111/j.1600-0838.2009.00986.x
   Konor MM, 2012, INT J SPORTS PHYS TH, V7, P279
   Lantto I, 2015, SCAND J MED SCI SPOR, V25, pE133, DOI 10.1111/sms.12253
   Le ADK, 2018, CURR REV MUSCULOSKE, V11, P624, DOI 10.1007/s12178-018-9527-7
   Maffulli N, 2017, AM J SPORTS MED, V26, P266
   Mishra A, 2009, CLIN SPORT MED, V28, P113, DOI 10.1016/j.csm.2008.08.007
   Moller M, 2002, SCAND J MED SCI SPOR, V12, P9, DOI 10.1034/j.1600-0838.2002.120103.x
   Mortensen NHM, 1999, J BONE JOINT SURG AM, V81A, P983, DOI 10.2106/00004623-199907000-00011
   Nilsson-Helander K, 2007, AM J SPORT MED, V35, P421, DOI 10.1177/0363546506294856
   Nilsson-Helander K, 2010, AM J SPORT MED, V38, P2186, DOI 10.1177/0363546510376052
   Olesen JL, 2006, CONNECT TISSUE RES, V47, P200, DOI 10.1080/03008200600809893
   Rosso C, 2013, KNEE SURG SPORT TR A, V21, P1369, DOI 10.1007/s00167-013-2407-1
   Sanchez M, 2007, AM J SPORT MED, V35, P245, DOI 10.1177/0363546506294078
   Schepull T, 2011, AM J SPORT MED, V39, P38, DOI 10.1177/0363546510383515
   Silbernagel KG, 2012, AM J SPORT MED, V40, P1564, DOI 10.1177/0363546512447926
   Silbernagel KG, 2006, KNEE SURG SPORT TR A, V14, P1207, DOI 10.1007/s00167-006-0150-6
   Silbernagel KG, 2010, KNEE SURG SPORT TR A, V18, P258, DOI 10.1007/s00167-009-0889-7
   Silbernagel KG, 2001, SCAND J MED SCI SPOR, V11, P197
   Soroceanu A, 2012, J BONE JOINT SURG AM, V94A, P2136, DOI 10.2106/JBJS.K.00917
   Willits K, 2010, J BONE JOINT SURG AM, V92A, P2767, DOI 10.2106/JBJS.I.01401
   Zou J, 2016, BIOMED RES INT, V2016, DOI 10.1155/2016/9364170
NR 40
TC 9
Z9 9
U1 1
U2 13
PU SAGE PUBLICATIONS INC
PI THOUSAND OAKS
PA 2455 TELLER RD, THOUSAND OAKS, CA 91320 USA
SN 0363-5465
EI 1552-3365
J9 AM J SPORT MED
JI Am. J. Sports Med.
PD JUL
PY 2020
VL 48
IS 9
BP 2268
EP 2276
AR 0363546520922541
DI 10.1177/0363546520922541
EA JUN 2020
PG 9
WC Orthopedics; Sport Sciences
WE Science Citation Index Expanded (SCI-EXPANDED)
SC Orthopedics; Sport Sciences
GA MQ3IC
UT WOS:000537496400001
PM 32485112
DA 2022-07-22
ER

PT J
AU Fischer, S
   Colcuc, C
   Gramlich, Y
   Stein, T
   Abdulazim, A
   von Welck, S
   Hoffmann, R
AF Fischer, Sebastian
   Colcuc, Christin
   Gramlich, Yves
   Stein, Thomas
   Abdulazim, Ahmed
   von Welck, Stephanie
   Hoffmann, Reinhard
TI Prospective randomized clinical trial of open operative, minimally
   invasive and conservative treatments of acute Achilles tendon tear
SO ARCHIVES OF ORTHOPAEDIC AND TRAUMA SURGERY
LA English
DT Article
DE Acute achilles tendon rupture; Re-rupture; Minimally invasive surgery;
   Isokinetic force measurement; Dynamometer
ID OPEN REPAIR; RUPTURE; METAANALYSIS; DIAGNOSIS; SCORE; RISK
AB Introduction The acute Achilles tendon rupture (AATR) is a common injury of great importance in an increasingly active society. When early functional treatment is established, recent literature shows comparable rates of re-rupture in conservative and surgical treatments of AATR. However, there is no study comparing the outcome using a dynamometer. The aim of this study is to evaluate the results of patients with AATR treated conservatively and surgically using a dynamometer. In addition, the data are compared to evaluation of the Achilles tendon with ultrasound. Materials and methods Between 2012 and 2015, 90 patients (mean age 41 years, male-to-female ratio 81:9) with AATR were enrolled in a prospective, randomized, and monocentric study. Thirty patients were assigned to each of the three different treatment groups. Group OPEN received a conventional open suture of the Achilles tendon, group MIN received a minimally invasive suture and patients in group CONS were treated conservatively. Follow-up treatment was the same for all patients regardless of the group they were assigned to. Plantar flexion force was assessed using a dynamometer (Biodex (R) System 3 Pro, Biodex Medical Systems). Further evaluation included a physical test and ultrasound of the Achilles tendon. Results Sixty-nine patients were available for a 24-month follow-up. In each of the OPEN and MIN groups, there was one re-rupture. In the CONS group, there were two re-ruptures. A positive correlation between the Biodex (R) dynamometer measurement and the American Orthopaedic Foot and Ankle Society Ankle-Hindfoot Score (AOFAS-AH) could be found in all groups. Nevertheless, there were no significant differences between the treatment groups after 2 years. On sonography, all patients showed isolated structure loosening and a significantly thickened cross-sectional area compared with the non-injured opposite side, without differences between the groups. There was no correlation between the Biodex (R) measurement and sonographic outcome. Conclusion At 24-month follow-up, no significant difference can be found in patients with AATR treated operatively or conservatively. It is, therefore, important to inform patients with AATR regarding the respective advantages and disadvantages of the individual treatment strategies.
C1 [Fischer, Sebastian] Berufsgenossenschaftliche Unfallklin Frankfurt Ma, Dept Foot & Ankle Surg, Friedberger Landstr 430, Frankfurt 60389, Germany.
   [Colcuc, Christin] Evangel Hosp Bethel Bielefeld, Dept Trauma & Orthopaed Surg, Bielefeld, Germany.
   [Gramlich, Yves; von Welck, Stephanie; Hoffmann, Reinhard] Berufsgenossenschaftliche Unfallklinik Frankfurt, Dept Trauma & Orthopaed Surg, Frankfurt, Germany.
   [Stein, Thomas] Berufsgenossenschaftliche Unfallklinik Frankfurt, Dept Sporttraumatol Knee & Shoulder Surg, Frankfurt, Germany.
   [Stein, Thomas] Goethe Univ Frankfurt, Dept Sports Med, Frankfurt, Germany.
   [Abdulazim, Ahmed] Univ Spital Basel, Dept Orthoped, Basel, Switzerland.
RP Fischer, S (通讯作者)，Berufsgenossenschaftliche Unfallklin Frankfurt Ma, Dept Foot & Ankle Surg, Friedberger Landstr 430, Frankfurt 60389, Germany.
EM dr.sebastian.fischer@me.com
CR Amlang MH, 2012, Z ORTHOP UNFALLCHIR, V150, P99, DOI 10.1055/s-0031-1298311
   Amlang Michael H, 2006, Oper Orthop Traumatol, V18, P287, DOI 10.1007/s00064-006-1178-y
   Bayliss AJ, 2016, J MUSCULOSKEL NEURON, V16, P105
   Bohm S, 2016, J BIOMECH, V49, P2932, DOI 10.1016/j.jbiomech.2016.07.002
   Brouwer EF, 2018, KNEE SURG SPORT TR A, V26, P2088, DOI 10.1007/s00167-017-4815-0
   Carmont Michael R, 2017, J Exp Orthop, V4, P26, DOI 10.1186/s40634-017-0101-9
   Ceccarelli F, 2014, J FOOT ANKLE SURG, V53, P16, DOI 10.1053/j.jfas.2013.09.005
   CHILLEMI C, 2002, FOOT ANKLE SURG, V8, P267
   Cretnik A, 2010, FOOT ANKLE INT, V31, P14, DOI 10.3113/FAI.2010.0014
   Dams OC, 2017, INJURY, V48, P2383, DOI 10.1016/j.injury.2017.09.013
   Deng SL, 2017, J FOOT ANKLE SURG, V56, P1236, DOI 10.1053/j.jfas.2017.05.036
   Ernat JJ, 2019, J FOOT ANKLE SURG, V58, P161, DOI 10.1053/j.jfas.2018.06.008
   Frankewycz B, 2017, ARCH ORTHOP TRAUM SU, V137, P333, DOI 10.1007/s00402-017-2627-9
   Freedman Benjamin R, 2014, Muscles Ligaments Tendons J, V4, P245
   FUKASHIRO S, 1995, EUR J APPL PHYSIOL O, V71, P453, DOI 10.1007/BF00635880
   Ganestam A, 2016, KNEE SURG SPORT TR A, V24, P3730, DOI 10.1007/s00167-015-3544-5
   Garras DN, 2012, CLIN ORTHOP RELAT R, V470, P2268, DOI 10.1007/s11999-012-2355-y
   Gitto S, 2016, J ULTRAS MED, V35, P2529, DOI 10.7863/ultra.16.01092
   Goren D, 2005, FOOT ANKLE INT, V26, P286, DOI 10.1177/107110070502600404
   Grassi A, 2018, J BONE JOINT SURG AM, V100, P1969, DOI 10.2106/JBJS.17.01364
   Haapasalo H, 2018, ARCH ORTHOP TRAUM SU, V138, P1089, DOI 10.1007/s00402-018-2940-y
   Huttunen TT, 2014, AM J SPORT MED, V42, P2419, DOI 10.1177/0363546514540599
   Khan RJK, 2005, J BONE JOINT SURG AM, V87A, P2202, DOI 10.2106/JBJS.D.03049
   Kostuj T, 2018, BMJ OPEN, V8, DOI 10.1136/bmjopen-2017-019872
   Lantto I, 2016, AM J SPORT MED, V44, P2406, DOI 10.1177/0363546516651060
   Manegold S, 2018, J ORTHOP TRAUMATOL, V19, DOI 10.1186/s10195-018-0511-1
   Moller M, 2002, SCAND J MED SCI SPOR, V12, P9, DOI 10.1034/j.1600-0838.2002.120103.x
   Noback PC, 2017, INJURY, V48, P2342, DOI 10.1016/j.injury.2017.08.050
   Ochen Y, 2019, BMJ-BRIT MED J, V364, DOI 10.1136/bmj.k5120
   Pagenstert G, 2010, ORTHOPADE, V39, P1135, DOI 10.1007/s00132-010-1691-4
   Richter M, 2006, FOOT ANKLE SURG, V12, P191, DOI [10.1016/j.fas.2006.04.001, DOI 10.1016/j.fas.2006.04.001, 10.1016/j.fas.2006.04.0012006/01/01/.]
   Romero-Morales C, 2019, PHYS THER SPORT, V35, P122, DOI 10.1016/j.ptsp.2018.12.003
   Spoendlin J, 2016, DRUG SAFETY, V39, P1229, DOI 10.1007/s40264-016-0462-5
   THERMANN H, 1989, UNFALLCHIRURG, V92, P266
   Thevendran G, 2013, Musculoskelet Surg, V97, P9, DOI 10.1007/s12306-013-0251-6
   Trost SG, 2014, BRIT J SPORT MED, V48, P169, DOI 10.1136/bjsports-2013-093372
   Westin O, 2016, ORTHOP J SPORTS MED, V4, DOI 10.1177/2325967116667920
   Wu YH, 2016, INT J SURG, V36, P305, DOI 10.1016/j.ijsu.2016.11.014
   Yang B, 2017, INT J SURG, V40, P178, DOI 10.1016/j.ijsu.2017.03.021
   Zellers JA, 2016, BRIT J SPORT MED, V50, P1325, DOI 10.1136/bjsports-2016-096106
   Zhou K, 2018, J FOOT ANKLE SURG, V57, P1191, DOI 10.1053/j.jfas.2018.05.007
NR 41
TC 13
Z9 13
U1 0
U2 0
PU SPRINGER
PI NEW YORK
PA ONE NEW YORK PLAZA, SUITE 4600, NEW YORK, NY, UNITED STATES
SN 0936-8051
EI 1434-3916
J9 ARCH ORTHOP TRAUM SU
JI Arch. Orthop. Trauma Surg.
PD MAY
PY 2021
VL 141
IS 5
BP 751
EP 760
DI 10.1007/s00402-020-03461-z
EA MAY 2020
PG 10
WC Orthopedics; Surgery
WE Science Citation Index Expanded (SCI-EXPANDED)
SC Orthopedics; Surgery
GA RN7HZ
UT WOS:000530240000003
PM 32367375
DA 2022-07-22
ER

PT J
AU MacMahon, A
   Deland, JT
   Do, H
   Soukup, DS
   Sofka, CM
   Demetracopolous, CA
   DeBlis, R
AF MacMahon, Aoife
   Deland, Jonathan T.
   Huong Do
   Soukup, Dylan S.
   Sofka, Carolyn M.
   Demetracopolous, Constantine A.
   DeBlis, Ryan
TI MRI Evaluation of Achilles Tendon Rotation and Sural Nerve Anatomy:
   Implications for Percutaneous and Limited-Open Achilles Tendon Repair
SO FOOT & ANKLE INTERNATIONAL
LA English
DT Article
DE tendon disorders; Achilles tendon; percutaneous repair; limited-open
   repair; rupture; sural nerve; anatomy; magnetic resonance imaging (MRI)
ID RUPTURES; TORSION; INJURY; DEVICE; MODEL; KNEE
AB Background: Limited-open and percutaneous Achilles tendon (AT) repair techniques have limited visibility, which may result in sural nerve violation and poor tendon targeting. The goal of this study was to assess the in vivo rotation of the AT and its distance to the sural nerve in ruptured and nonruptured ATs to develop guidelines to aid in limited-open and percutaneous repair techniques.
   Methods: A retrospective review was conducted to identify magnetic resonance imaging (MRI) studies of patients with ruptured and healthy (nonruptured) ATs. AT rotation and distance to the sural nerve in the anterior-posterior (A-P) and medial-lateral (M-L) planes were measured at the level of and proximal to the ankle.
   Results: The AT was externally rotated in both ruptured and nonruptured cohorts. Ruptured ATs showed greater external rotation than nonruptured ATs at the ankle (15.8 16.2 degrees vs 5.9 9.0 degrees, P = .008) but not at 10 cm proximal to the tendon's insertion (10.9 +/- 10.9 degrees vs 6.1 +/- 8.4 degrees, P = .139). Proximal AT rotation was negatively correlated with rupture height (r = -0.477, P = .029). At 4 cm proximal to the AT insertion, the sural nerve was closer anteriorly to and farther laterally from the AT in ruptures than in nonruptures (P < .001). At 10 cm proximal to the AT insertion, the sural nerve was farther posteriorly and laterally from the AT in ruptures than in nonruptures (P = .027 and P < .001, respectively).
   Conclusion: We found that the AT was more externally rotated in ruptured than in nonruptured tendons at the ankle and that its distance to the sural nerve differed between the 2 cohorts in the A-P and M-L planes, likely due to increased AT rotation and swelling with ruptures. To minimize sural nerve injury and improve tendon targeting, we suggest an external rotation of 11 degrees at the proximal end of the rupture and 16 degrees at the distal end when using percutaneous and limited-open AT repair devices to try to minimize sural nerve violation and increase tendon capture, which can decrease rates of complication and rerupture.
   Level of Evidence: Level III, retrospective comparative study.
C1 [MacMahon, Aoife; Deland, Jonathan T.; Soukup, Dylan S.; Demetracopolous, Constantine A.; DeBlis, Ryan] Hosp Special Surg, Dept Foot & Ankle Surg, 523 East 72nd St, New York, NY 10021 USA.
   [Huong Do] Hosp Special Surg, Dept Epidemiol & Biostat, 535 E 70th St, New York, NY 10021 USA.
   [Sofka, Carolyn M.] Hosp Special Surg, Dept Radiol & Imaging, 535 E 70th St, New York, NY 10021 USA.
RP DeBlis, R (通讯作者)，Hosp Special Surg, Dept Foot & Ankle Surg, 523 East 72nd St, New York, NY 10021 USA.
EM ryandeblis@gmail.com
OI Soukup, Dylan/0000-0003-0600-1250
CR Aibinder WR, 2013, FOOT ANKLE INT, V34, P870, DOI 10.1177/1071100712473097
   ARACIL J, 1992, FOOT ANKLE, V13, P350, DOI 10.1177/107110079201300610
   Blackmon JA, 2013, J FOOT ANKLE SURG, V52, P42, DOI 10.1053/j.jfas.2012.09.010
   Citak M, 2007, BRIT J SPORT MED, V41, P456, DOI 10.1136/bjsm.2006.031328
   CUMMINS EJ, 1946, SURG GYNECOL OBSTET, V83, P107
   Demetracopoulos CA, 2014, FOOT ANKLE INT, V35, P612, DOI 10.1177/1071100714524550
   Donovan A, 2010, RADIOGRAPHICS, V30, P1001, DOI 10.1148/rg.304095188
   Ellapparadja Pregash, 2014, Eur J Orthop Surg Traumatol, V24, P615, DOI 10.1007/s00590-013-1343-6
   FITZGIBBONS RE, 1993, AM J SPORT MED, V21, P724, DOI 10.1177/036354659302100516
   Flavin R, 2007, INJURY, V38, P845, DOI 10.1016/j.injury.2006.10.016
   Haji A, 2004, FOOT ANKLE INT, V25, P215, DOI 10.1177/107110070402500404
   Hoffman B, 2006, FOOT ANKLE INT, V27, P960, DOI 10.1177/107110070602701116
   Hsu AR, 2015, FOOT ANKLE INT, V36, P1279, DOI 10.1177/1071100715589632
   Huttunen TT, 2014, AM J SPORT MED, V42, P2419, DOI 10.1177/0363546514540599
   Kammar H, 2014, ORTHOPEDICS, V37, pE298, DOI 10.3928/01477447-20140225-64
   Kangas J, 2007, AM J SPORT MED, V35, P59, DOI 10.1177/0363546506293255
   Keser S, 2006, ARTHROSCOPY, V22, P656, DOI 10.1016/j.arthro.2006.04.076
   Lantto I, 2015, SCAND J MED SCI SPOR, V25, pE133, DOI 10.1111/sms.12253
   McCann PA, 2010, INJURY, V41, P1012, DOI 10.1016/j.injury.2010.02.031
   MORIMOTO I, 1968, Acta Anatomica Nipponica, V43, P295
   Obst SJ, 2014, J APPL PHYSIOL, V116, P376, DOI 10.1152/japplphysiol.01249.2013
   Porter KJ, 2014, FOOT ANKLE SURG, V20, P90, DOI 10.1016/j.fas.2013.11.005
   van Gils C C, 1996, J Foot Ankle Surg, V35, P41
   Webb J, 2000, FOOT ANKLE INT, V21, P475, DOI 10.1177/107110070002100604
   White JW, 1943, ARCH SURG-CHICAGO, V46, P784, DOI 10.1001/archsurg.1943.01220110200033
NR 25
TC 9
Z9 10
U1 0
U2 3
PU SAGE PUBLICATIONS INC
PI THOUSAND OAKS
PA 2455 TELLER RD, THOUSAND OAKS, CA 91320 USA
SN 1071-1007
EI 1944-7876
J9 FOOT ANKLE INT
JI Foot Ankle Int.
PD JUN
PY 2016
VL 37
IS 6
BP 636
EP 643
DI 10.1177/1071100716628915
PG 8
WC Orthopedics
WE Science Citation Index Expanded (SCI-EXPANDED)
SC Orthopedics
GA DP3WC
UT WOS:000378425600010
PM 26843545
DA 2022-07-22
ER

PT J
AU Guelfi, M
   Pantalone, A
   Vanni, D
   Rosati, D
   Guelfi, MGB
   Salini, V
AF Guelfi, Matteo
   Pantalone, Andrea
   Vanni, Daniele
   Rosati, Denise
   Guelfi, Marco G. B.
   Salini, Vincenzo
TI Bilateral traumatic rupture of Achilles tendons in absence of risk
   factors treated with percutaneous technique and platelet-rich plasma: A
   case report
SO FOOT AND ANKLE SURGERY
LA English
DT Article
DE Achilles tendon; Bilateral traumatic rupture; Absence of risk factors;
   PRP; Growth factors; Percutaneous tenorraphy
ID GROWTH-FACTORS; NONOPERATIVE MANAGEMENT; FIBRIN MATRICES; OPEN REPAIR
AB We present a clinical case of a 52-year-old man with bilateral traumatic rupture of the Achilles tendon (AT) in absence of risk factors. In medical history, the patient does not report pre-existing tendon diseases. AT ruptures occurred following a skiing injury in which the forward fall caused a severe stress and elongation of the AT. Associated with tendon injury there was a fracture of the right humeral greater tuberosity. The patient was subjected to percutaneous tenorraphy according to Maffulli's technique and subsequently topical injection of autologous platelet-rich plasma (PRP) 7 days after the injury. After surgery, the patient followed an accelerated rehabilitation protocol, allowing the weight bearing with guards (Rom-Walker) and crutches to four weeks then freely to 8 weeks. We performed a clinical (AOFAS ankle-hindfoot score) and ultrasonography follow-up at month 1, 3, 6 and 12, with excellent results in the end. To the best of our knowledge bilateral cases like this have not been described in the literature. (c) 2014 European Foot and Ankle Society. Published by Elsevier Ltd. All rights reserved.
C1 [Guelfi, Matteo; Pantalone, Andrea; Vanni, Daniele; Rosati, Denise; Salini, Vincenzo] Univ G DAnnunzio, Orthopaed & Traumatol Div, I-66013 Chieti, Italy.
   [Guelfi, Marco G. B.] Clin Montallegro, Orthopaed Div, I-16145 Genoa, Italy.
RP Guelfi, M (通讯作者)，Via Caprera 7-3, I-16146 Genoa, Italy.
EM guelfi.m@gmail.com; marco.guelfi@fastwebnet.it; v.salini@unich.it
RI Salini, Vincenzo/ABB-5842-2021; Vanni, Daniele/ABH-2080-2021
OI Salini, Vincenzo/0000-0002-8166-3062; Vanni,
   Daniele/0000-0002-0753-6030; Guelfi, Matteo/0000-0003-0671-4648
CR Ahmad J, 2013, FOOT ANKLE INT, V34, P1074, DOI 10.1177/1071100713483115
   Anitua E, 2006, J BIOMED MATER RES A, V77A, P285, DOI 10.1002/jbm.a.30585
   Anitua E, 2005, J ORTHOP RES, V23, P281, DOI 10.1016/j.orthres.2004.08.015
   Aroen A, 2004, SCAND J MED SCI SPOR, V14, P30, DOI 10.1111/j.1600-0838.2004.00344.x
   BARUAH DR, 1984, UNFALLHEILKUNDE, V87, P35
   Battista V, 2003, AM J SPORT MED, V31, P1007, DOI 10.1177/03635465030310060201
   CETTI R, 1993, AM J SPORT MED, V21, P791, DOI 10.1177/036354659302100606
   Chandrakant V, 2012, FOOT ANKLE INT, V33, P1093, DOI 10.3113/FAI.2012.1093
   Cretnik A, 2005, AM J SPORT MED, V33, P1369, DOI 10.1177/0363546504271501
   Goren D, 2005, FOOT ANKLE INT, V26, P286, DOI 10.1177/107110070502600404
   HABUSTA SF, 1995, CLIN ORTHOP RELAT R, P231
   Haji A, 2004, FOOT ANKLE INT, V25, P215, DOI 10.1177/107110070402500404
   Hashim Zaid, 2012, Foot Ankle Spec, V5, P68, DOI 10.1177/1938640011434415
   Hsu C, 2004, J HAND SURG-AM, V29A, P551, DOI 10.1016/j.jhsa.2004.04.020
   Jielile J, 2012, FOOT ANKLE INT, V33, P1119, DOI 10.3113/FAI.2012.1119
   KAO NL, 1992, THORAX, V47, P484, DOI 10.1136/thx.47.6.484-b
   Khan RJK, 2005, J BONE JOINT SURG AM, V87A, P2202, DOI 10.2106/JBJS.D.03049
   Kocher MS, 2002, AM J SPORT MED, V30, P783, DOI 10.1177/03635465020300060501
   Kotnis RA, 1999, J ACCID EMERG MED, V16, P378
   Kowatari K, 2004, J ORTHOP SCI, V9, P186, DOI 10.1007/s00776-003-0761-4
   LEE WT, 1992, AUST NZ J MED, V22, P500
   Miller D, 2005, SCOT MED J, V50, P160, DOI 10.1177/003693300505000408
   Mishra A, 2006, AM J SPORT MED, V34, P1774, DOI 10.1177/0363546506288850
   Molloy T, 2003, SPORTS MED, V33, P381, DOI 10.2165/00007256-200333050-00004
   Orava S, 1996, SCAND J MED SCI SPOR, V6, P309
   Pantalone A, 2011, INT J IMMUNOPATH PH, V24, P519, DOI 10.1177/039463201102400227
   Rao S K, 2005, J Orthop Surg (Hong Kong), V13, P178
   Sanchez M, 2007, AM J SPORT MED, V35, P245, DOI 10.1177/0363546506294078
   Soldatis JJ, 1997, AM J SPORT MED, V25, P90, DOI 10.1177/036354659702500118
   Twaddle BC, 2005, J BONE JOINT SURG BR, V87, P30
   Wagnon Raymond, 2005, J Foot Ankle Surg, V44, P437, DOI 10.1053/j.jfas.2005.07.015
   Wong J, 2002, AM J SPORT MED, V30, P565, DOI 10.1177/03635465020300041701
NR 32
TC 1
Z9 2
U1 0
U2 5
PU ELSEVIER SCIENCE BV
PI AMSTERDAM
PA PO BOX 211, 1000 AE AMSTERDAM, NETHERLANDS
SN 1268-7731
EI 1460-9584
J9 FOOT ANKLE SURG
JI Foot Ankle Surg.
PD SEP
PY 2014
VL 20
IS 3
BP E43
EP E46
DI 10.1016/j.fas.2014.05.005
PG 4
WC Orthopedics
WE Science Citation Index Expanded (SCI-EXPANDED)
SC Orthopedics
GA CI8GN
UT WOS:000355008700003
PM 25103716
DA 2022-07-22
ER

PT J
AU Ready, LV
   Li, NY
   Worobey, S
   Lemme, NJ
   Yang, DS
   Yang, J
   Krill, M
   Owens, BD
AF Ready, Lauren, V
   Li, Neill Y.
   Worobey, Samantha
   Lemme, Nicholas J.
   Yang, Daniel S.
   Yang, JaeWon
   Krill, Michael
   Owens, Brett D.
TI Influence of Preseason Versus In-Season Play on Achilles Tendon Injuries
   in the National Football League
SO ORTHOPAEDIC JOURNAL OF SPORTS MEDICINE
LA English
DT Article
DE Achilles tendon; sports medicine; tendon injury; ankle injury; lower
   extremity injury
AB Background: A ruptured Achilles tendon (AT) can sideline a player for 6 to 12 months and reduce their power rankings by more than 50%. Previous research has compared AT rupture rates in different game conditions. Purpose: To determine environmental and physiological risk factors for AT tears, given the minimal amount of research on AT ruptures in the National Football League (NFL). Study Design: Case series; Level of evidence, 4. Methods: NFL players with a diagnosed AT tear between 2009 and 2016 were selected as the study population for this retrospective analysis. Data on NFL injury were collected from an established database composed of publicly available information. Player profiles were employed to determine position, team, and game statistics at the time of injury. The proportion of NFL rookies was approximated by summing the number of draft picks and the number of signed, undrafted free agents and measured against the number of roster spots before the season. Results: Between 2009 and 2016, there were 101 documented AT tears. Of these, 64% (65/101) occurred before the official season, including preseason games. Of the 36 tears that occurred in-season, 34 were during games. Overall, 29% (19/65) of the preseason tears occurred in rookies and 100% (36/36) of the in-season tears affected nonrookies. Of the rookies with AT ruptures, 42.11% returned to play in the NFL, while 62.20% of the nonrookies came back to partake in future seasons. Despite an average age of 26.7 years, the tear distribution was bimodal with players aged 24 and 36 years exhibiting the highest rates of tear. Conclusion: In our review of AT tears in NFL athletes, a large percentage of the tears occurred in rookie players, especially during the preseason. We also found that tears during the season occurred in only nonrookies, suggesting that the preseason is when rookies experience the greatest risk for injury.
C1 [Ready, Lauren, V; Li, Neill Y.; Worobey, Samantha; Lemme, Nicholas J.; Yang, Daniel S.; Yang, JaeWon; Krill, Michael; Owens, Brett D.] Brown Univ, Providence, RI 02912 USA.
   [Ready, Lauren, V; Yang, Daniel S.; Yang, JaeWon] Brown Univ, Warren Alpert Med Sch, Providence, RI 02912 USA.
   [Li, Neill Y.; Lemme, Nicholas J.; Owens, Brett D.] Brown Univ, Dept Orthopaed Surg, Warren Alpert Med Sch, Providence, RI 02912 USA.
   [Worobey, Samantha] Wheaton Coll, Norton, MA 02766 USA.
   [Krill, Michael] Univ Iowa Sports Med, Dept Family Med, Iowa City, IA USA.
RP Ready, LV (通讯作者)，Warrant Alpert Med Sch, 222 Richmond St, Providence, RI 02903 USA.
EM lvready@gmail.com
RI Krill, Michael/AAF-7180-2020
OI Krill, Michael/0000-0002-5248-4786
FU Arthrex; DePuy Mitek; Musculoskeletal Transplant Foundation
FX N.Y.L. has received hospitality payments from Axogen and Zimmer. B.D.O.
   has received research support from Arthrex, DePuy Mitek, and
   Musculoskeletal Transplant Foundation; consulting fees from ConMed
   Linvatec, DePuy Mitek, Miach, Musculoskeletal Transplant Foundation; and
   Vericel; royalties from ConMed Linvatec, Saunders/Mosby-Elsevier, Slack,
   and Springer; has stock/stock options in Vivorte; and is a paid
   associate editor for The American Journal of Sports Medicine. AOSSM
   checks author disclosures against the Open Payments Database (OPD).
   AOSSM has not conducted an independent investigation on the OPD and
   disclaims any liability or responsibility relating thereto.
CR Breech J, 2017 UNDRAFTED FREE
   Dulac G, PITTSBURGH POSTGAZET
   Jarvinen Tero A H, 2005, Foot Ankle Clin, V10, P255, DOI 10.1016/j.fcl.2005.01.013
   Kraemer R, 2009, AM J SPORT MED, V37, P1384, DOI 10.1177/0363546509333012
   Krill MK, 2018, PHYSICIAN SPORTSMED, V46, P342, DOI 10.1080/00913847.2018.1482188
   Krill MK, 2017, PHYSICIAN SPORTSMED, V45, P259, DOI 10.1080/00913847.2017.1343652
   Lawrence DW, 2016, ORTHOP J SPORTS MED, V4, DOI 10.1177/2325967116639222
   Lawrence DW, 2015, ORTHOP J SPORTS MED, V3, DOI 10.1177/2325967115583653
   Lemme NJ, 2019, AM J SPORT MED, V47, P2360, DOI 10.1177/0363546519858609
   Milgrom C, 2003, FOOT ANKLE INT, V24, P398, DOI 10.1177/107110070302400504
   National Football League, 2017, INJ DAT
   Parekh Selene G, 2009, Foot Ankle Spec, V2, P283, DOI 10.1177/1938640009351138
   Peters JA, 2016, J SCI MED SPORT, V19, P205, DOI 10.1016/j.jsams.2015.03.008
   Robbins DW, 2011, J STRENGTH COND RES, V25, P2661, DOI 10.1519/JSC.0b013e318208ae3f
   Sahota S, 2020, SPORTS HEALTH, V12, P116, DOI 10.1177/1941738119885867
   Schepsis AA, 2002, AM J SPORT MED, V30, P287, DOI 10.1177/03635465020300022501
   Yang J, 2019, ORTHOP J SPORTS MED, V7, DOI 10.1177/2325967119830139
NR 17
TC 0
Z9 0
U1 0
U2 0
PU SAGE PUBLICATIONS INC
PI THOUSAND OAKS
PA 2455 TELLER RD, THOUSAND OAKS, CA 91320 USA
EI 2325-9671
J9 ORTHOP J SPORTS MED
JI Orthop. J. Sports Med.
PD DEC
PY 2021
VL 9
IS 12
AR 23259671211056083
DI 10.1177/23259671211056083
PG 6
WC Orthopedics; Sport Sciences
WE Science Citation Index Expanded (SCI-EXPANDED)
SC Orthopedics; Sport Sciences
GA XN4TT
UT WOS:000729500000001
PM 34901290
OA Green Published
DA 2022-07-22
ER

PT J
AU Notermans, T
   Hammerman, H
   Eliasson, P
   Isaksson, H
AF Notermans, T.
   Hammerman, H.
   Eliasson, P.
   Isaksson, H.
TI TENDON MECHANOBIOLOGY IN SMALL-ANIMAL EXPERIMENTS DURING POST-TRANS
   ECTION HEALING
SO EUROPEAN CELLS & MATERIALS
LA English
DT Article
DE Collagen; extracellular matrix; rupture; Achilles tendon; cells;
   unloading; immobilization; tissue differentiation; mechanical;
   heterogeneous
ID RAT ACHILLES-TENDON; PLATELET-RICH PLASMA; HETEROTOPIC OSSIFICATION;
   STEM/PROGENITOR CELLS; DIABETES IMPAIRS; GENE-EXPRESSION;
   BONE-FORMATION; INJURY; REPAIR; IMMOBILIZATION
AB Ruptures to tendons are common and costly, and no clinical consensus exists on the appropriate treatment and rehabilitation regimen to promote their healing as well as full recovery of functionality. Although mechanobiology is known to play an important role in tendon regeneration, the understanding of how mechano-regulated processes affect tendon healing needs further clarification. Many small-animal studies, particularly in rats and mice, have characterized the progression of healing in terms of geometrical, structural, compositional, mechanical, and cellular properties. Some of the properties are also studied under different mechanical loading regimens. The focus of this review is to summarize and generalize the information in the literature regarding spatial and temporal differentiation of tendon properties during rodent tendon healing following full-tendon transection, as well as how this is affected by altered in vivo loading regimens.
C1 [Notermans, T.; Hammerman, H.; Isaksson, H.] Lund Univ, Dept Biomed Engn, Box 118, S-22100 Lund, Sweden.
   [Hammerman, H.; Eliasson, P.] Linkoping Univ, Dept Biomed & Clin Sci, Div Cell Biol, Linkoping, Sweden.
RP Isaksson, H (通讯作者)，Lund Univ, Dept Biomed Engn, Box 118, S-22100 Lund, Sweden.
EM hanna.isaksson@bme.lth.se
RI Isaksson, Hanna/L-1340-2015
OI Isaksson, Hanna/0000-0002-9690-8907
FU European Union [713645]; Knut and Alice Wallenberg KAW Foundation
   (Wallenberg Academy) [2017.0221]; European Research Council (ERC) under
   the European Union [101002516]
FX This project has received funding from the European Union's Horizon 2020
   research and innovation program under the Marie Sklodowska-Curie grant
   agreement number 713645, the Knut and Alice Wallenberg KAW Foundation
   (Wallenberg Academy Fellows 2017.0221) and the European Research Council
   (ERC) under the European Union's Horizon 2020 research and innovation
   program (grant agreement number 101002516).
CR Ackerman JE, 2019, ELIFE, V8, DOI 10.7554/eLife.45342
   Ackerman JE, 2017, SCI REP-UK, V7, DOI 10.1038/s41598-017-09407-7
   Agarwal S, 2017, STEM CELLS, V35, P705, DOI 10.1002/stem.2515
   Ahmed AS, 2012, J APPL PHYSIOL, V113, P1784, DOI 10.1152/japplphysiol.00767.2012
   Andersson T, 2012, J APPL PHYSIOL, V113, P1398, DOI 10.1152/japplphysiol.00491.2012
   Andersson T, 2009, J APPL PHYSIOL, V107, P417, DOI 10.1152/japplphysiol.00414.2009
   Asai S, 2014, STEM CELLS, V32, P3266, DOI 10.1002/stem.1847
   Best KT, 2021, ELIFE, V10, DOI 10.7554/eLife.62203
   Best KT, 2019, SCI REP-UK, V9, DOI 10.1038/s41598-019-47461-5
   Best KT, 2019, FASEB J, V33, P8578, DOI 10.1096/fj.201900130RR
   BEST TM, 1993, J ORTHOP RES, V11, P897, DOI 10.1002/jor.1100110617
   Bi YM, 2007, NAT MED, V13, P1219, DOI 10.1038/nm1630
   Black David A, 2012, Biomed Sci Instrum, V48, P43
   Bolt P, 2007, J BONE JOINT SURG AM, V89A, P1315, DOI 10.2106/JBJS.F.00257
   Burssens P, 2005, FOOT ANKLE INT, V26, P832, DOI 10.1177/107110070502601008
   Carlsson O, 2011, SCAND J MED SCI SPOR, V21, P562, DOI 10.1111/j.1600-0838.2009.01080.x
   Chamberlain CS, 2013, ANN BIOMED ENG, V41, P477, DOI 10.1007/s10439-012-0689-y
   Chbinou N, 2004, AM J PHYSIOL-REG I, V286, pR952, DOI 10.1152/ajpregu.00536.2003
   Cheema AN, 2019, J ORTHOP RES, V37, P94, DOI 10.1002/jor.24167
   Chen GR, 2017, MOL MED REP, V16, P5901, DOI 10.3892/mmr.2017.7380
   Chen K, 2018, PLOS COMPUT BIOL, V14, DOI 10.1371/journal.pcbi.1006652
   Cury DP, 2019, AM J SPORT MED, V47, P901, DOI 10.1177/0363546518822836
   da Silva FS, 2021, KNEE SURG SPORT TR A, V29, P1990, DOI 10.1007/s00167-020-06239-3
   Devana SK, 2018, CLIN ORTHOP RELAT R, V476, P2091, DOI 10.1097/CORR.0000000000000461
   Dex S, 2017, EBIOMEDICINE, V20, P240, DOI 10.1016/j.ebiom.2017.05.003
   Dietrich F, 2015, WORLD J PLAST SURG, V4, P101
   Dyment NA, 2014, PLOS ONE, V9, DOI 10.1371/journal.pone.0096113
   Dyment NA, 2013, PLOS ONE, V8, DOI 10.1371/journal.pone.0059944
   Eliasson P, 2012, J APPL PHYSIOL, V112, P279, DOI 10.1152/japplphysiol.00858.2011
   Eliasson P, 2012, J ORTHOP RES, V30, P274, DOI 10.1002/jor.21511
   Eliasson P, 2009, J APPL PHYSIOL, V107, P399, DOI 10.1152/japplphysiol.91563.2008
   Freedman Benjamin R, 2014, Muscles Ligaments Tendons J, V4, P245
   Freedman BR, 2017, J AM ACAD ORTHOP SUR, V25, P635, DOI 10.5435/JAAOS-D-16-00620
   Freedman BR, 2017, J BIOMECH, V56, P55, DOI 10.1016/j.jbiomech.2017.02.030
   Freedman BR, 2016, J ORTHOP RES, V34, P2172, DOI 10.1002/jor.23253
   Fryhofer GW, 2016, J APPL PHYSIOL, V121, P1106, DOI 10.1152/japplphysiol.00620.2016
   Galatz LM, 2015, J ORTHOP RES, V33, P823, DOI 10.1002/jor.22853
   George NS, 2021, J ORTHOP RES, V39, P1344, DOI 10.1002/jor.24705
   Graham JG, 2019, CONNECT TISSUE RES, V60, P10, DOI 10.1080/03008207.2018.1512979
   Guo DS, 2020, BIOCHEM BIOPH RES CO, V521, P91, DOI 10.1016/j.bbrc.2019.10.082
   Hammerman M, 2018, PLOS ONE, V13, DOI 10.1371/journal.pone.0201211
   Hammerman M, 2014, J APPL PHYSIOL, V116, P54, DOI 10.1152/japplphysiol.00741.2013
   Harvey T, 2019, NAT CELL BIOL, V21, P1490, DOI 10.1038/s41556-019-0417-z
   Hillin CD, 2019, J ORTHOP RES, V37, P562, DOI 10.1002/jor.24241
   Holm C, 2015, SCAND J MED SCI SPOR, V25, pE1, DOI 10.1111/sms.12209
   Howell K, 2017, SCI REP-UK, V7, DOI 10.1038/srep45238
   Hsieh CF, 2016, NANOMEDICINE-UK, V11, P1153, DOI 10.2217/nnm.16.34
   Hsu JE, 2016, ORTHOP CLIN N AM, V47, P169, DOI 10.1016/j.ocl.2015.08.017
   Huber AK, 2020, J CLIN INVEST, V130, P5444, DOI 10.1172/JCI136142
   Huegel J, 2019, J BIOMECH, V88, P194, DOI 10.1016/j.jbiomech.2019.03.029
   Jelinsky SA, 2011, J ORTHOP RES, V29, P1604, DOI 10.1002/jor.21427
   Kashiwagi K, 2004, SCAND J PLAST RECONS, V38, P193, DOI 10.1080/02844310410029110
   Kaux JF, 2012, WOUND REPAIR REGEN, V20, P748, DOI 10.1111/j.1524-475X.2012.00826.x
   Kendal AR, 2020, SCI REP-UK, V10, DOI 10.1038/s41598-020-70786-5
   Khayyeri H, 2020, PLOS ONE, V15, DOI 10.1371/journal.pone.0236681
   Killian ML, 2012, J SHOULDER ELB SURG, V21, P228, DOI 10.1016/j.jse.2011.11.002
   Komatsu I, 2016, ACTA BIOMATER, V42, P136, DOI 10.1016/j.actbio.2016.06.026
   Korntner S, 2017, SCI REP-UK, V7, DOI 10.1038/s41598-017-00700-z
   Kueckelhaus Maximilian, 2014, Eplasty, V14, pe29
   Kurtz CA, 1999, AM J SPORT MED, V27, P363, DOI 10.1177/03635465990270031701
   Lin DS, 2017, CELL DEATH DIS, V8, DOI 10.1038/cddis.2017.510
   Lin L, 2010, BONE, V46, P425, DOI 10.1016/j.bone.2009.08.057
   Majewski M, 2008, GENE THER, V15, P1139, DOI 10.1038/gt.2008.48
   Majewski M, 2018, EUR CELLS MATER, V35, P318, DOI 10.22203/eCM.v035a22
   Majewski M, 2012, EUR CELLS MATER, V23, P94, DOI 10.22203/eCM.v023a07
   Majewski M, 2009, AM J SPORT MED, V37, P2117, DOI 10.1177/0363546509348047
   Misir A, 2020, KNEE SURG SPORT TR A, V28, P2788, DOI 10.1007/s00167-019-05536-w
   Muller SA, 2018, AM J SPORT MED, V46, P1214, DOI 10.1177/0363546518756093
   Muller SA, 2016, AM J SPORT MED, V44, P1998, DOI 10.1177/0363546516641942
   Muller SA, 2015, KNEE SURG SPORT TR A, V23, P2097, DOI 10.1007/s00167-013-2680-z
   Murrell GAC, 1997, INFLAMM RES, V46, P19, DOI 10.1007/s000110050027
   Murrell GAC, 2008, CLIN ORTHOP RELAT R, V466, P1618, DOI 10.1007/s11999-008-0271-y
   Nichols AEC, 2019, TRANSL RES, V209, P156, DOI 10.1016/j.trsl.2019.02.002
   Notermans T, 2021, PLOS COMPUT BIOL, V17, DOI 10.1371/journal.pcbi.1008636
   Nourissat G, 2015, NAT REV RHEUMATOL, V11, P223, DOI 10.1038/nrrheum.2015.26
   Omachi T, 2015, J ORTHOP SCI, V20, P380, DOI 10.1007/s00776-014-0684-2
   Palmes D, 2002, J ORTHOPAED RES, V20, P939, DOI 10.1016/S0736-0266(02)00032-3
   Richardson WJ, 2018, BIOMECH MODEL MECHAN, V17, P1569, DOI 10.1007/s10237-018-1044-5
   Riggin CN, 2019, ULTRASOUND MED BIOL, V45, P1841, DOI 10.1016/j.ultrasmedbio.2019.03.002
   Runesson E, 2015, BMC MUSCULOSKEL DIS, V16, DOI 10.1186/s12891-015-0658-3
   Sakabe T, 2018, J BIOL CHEM, V293, P5766, DOI 10.1074/jbc.RA118.001987
   Sasaki K, 2012, J ELECTRON MICROSC, V61, P327, DOI 10.1093/jmicro/dfs057
   Schizas N, 2010, J ORTHOP RES, V28, P852, DOI 10.1002/jor.21066
   Snedeker JG, 2017, ACTA BIOMATER, V63, P18, DOI 10.1016/j.actbio.2017.08.032
   Staresinic M, 2003, J ORTHOP RES, V21, P976, DOI 10.1016/S0736-0266(03)00110-4
   Stauber T, 2020, MATRIX BIOL, V85-86, P68, DOI 10.1016/j.matbio.2019.07.008
   Sugg KB, 2014, J ORTHOP RES, V32, P944, DOI 10.1002/jor.22624
   Svard A, 2020, FASEB J, V34, P13409, DOI 10.1096/fj.202001255R
   Tan Q, 2013, STEM CELLS DEV, V22, P3128, DOI 10.1089/scd.2013.0073
   Tarafder S, 2017, FASEB J, V31, P3991, DOI 10.1096/fj.201700071R
   Taye N, 2020, J ORTHOP RES, V38, P23, DOI 10.1002/jor.24440
   Tempfer H, 2018, CELL PHYSIOL BIOCHEM, V46, P1148, DOI 10.1159/000489057
   Tempfer H, 2015, FRONT PHYSIOL, V6, DOI 10.3389/fphys.2015.00330
   Thomopoulos S, 2015, J ORTHOP RES, V33, P832, DOI 10.1002/jor.22806
   Usman MA, 2015, J ORTHOP SCI, V20, P538, DOI 10.1007/s00776-015-0709-5
   Voleti PB, 2012, ANNU REV BIOMED ENG, V14, P47, DOI 10.1146/annurev-bioeng-071811-150122
   Wang JHC, 2006, J BIOMECH, V39, P1563, DOI 10.1016/j.jbiomech.2005.05.011
   Wang JHC, 2012, J HAND THER, V25, P133, DOI 10.1016/j.jht.2011.07.004
   Weng CJ, 2020, INT J NANOMED, V15, P125, DOI 10.2147/IJN.S217697
   Wieloch P, 2004, J BIOMECH, V37, P1719, DOI 10.1016/j.jbiomech.2004.01.027
   Zhang K, 2016, MATRIX BIOL, V52-54, P315, DOI 10.1016/j.matbio.2016.01.015
NR 101
TC 0
Z9 0
U1 1
U2 2
PU AO RESEARCH INSTITUTE DAVOS-ARI
PI DAVOS
PA CLAVADELERSTRASSE 8, DAVOS, CH 7270, SWITZERLAND
SN 1473-2262
J9 EUR CELLS MATER
JI Eur. Cells Mater.
PD JUL-DEC
PY 2021
VL 42
BP 375
EP 391
DI 10.22203/eCM.v042a23
PG 17
WC Cell & Tissue Engineering; Engineering, Biomedical; Materials Science,
   Biomaterials; Orthopedics
WE Science Citation Index Expanded (SCI-EXPANDED)
SC Cell Biology; Engineering; Materials Science; Orthopedics
GA WT9LB
UT WOS:000716177300001
PM 34725807
OA Green Submitted, Bronze
DA 2022-07-22
ER

PT J
AU Blackmon, JA
   Atsas, S
   Clarkson, MJ
   Fox, JN
   Daney, BT
   Dodson, SC
   Lambert, HW
AF Blackmon, Joseph A.
   Atsas, Stavros
   Clarkson, Mackenzie J.
   Fox, Jacob N.
   Daney, Blake T.
   Dodson, Sean C.
   Lambert, H. Wayne
TI Locating the Sural Nerve during Calcaneal (Achilles) Tendon Repair with
   Confidence: A Cadaveric Study with Clinical Applications
SO JOURNAL OF FOOT & ANKLE SURGERY
LA English
DT Article
DE calcaneal (Achilles) tendon; calcaneal tuberosity; iatrogenic injury;
   percutaneous surgical repair
ID INVASIVE SURGICAL TECHNIQUE; PERCUTANEOUS REPAIR; FUNCTIONAL
   AFTERTREATMENT; NONOPERATIVE TREATMENT; ENDOSCOPIC CONTROL; RUPTURE;
   FLAP; INJURIES; ANATOMY
AB The sural nerve is at risk of iatrogenic injury even during minimally invasive operative procedures to repair the calcaneal (Achilles) tendon. Through 107 cadaveric leg dissections, the data derived from the present study was used to develop a regression equation that will enable surgeons to estimate the intersection point at which the sural nerve crosses the lateral border of the Achilles tendon, an important surgical landmark. In most cases, the sural nerve crossed the lateral border of the Achilles tendon 8 to 10 cm proximal to the superior border of the calcaneal tuberosity. By simply measuring the leg length of the patient (from the base of the heel to the flexor crease of the popliteal fossa), surgeons can approximate the location of this intersection point with an interval length of 0.68 to 1.80 cm, with 90% confidence, or 0.82 to 2.15 cm, with 95% confidence. For example, for a patient with a lower leg length of 47.0 cm, the mean measurement in the present study, a surgeon can be 90% confident that the sural nerve will cross the lateral border of the Achilles tendon 8.28 to 8.96 cm (interval width of 0.68 cm) proximal to the calcaneal tuberosity. Currently, ultrasound and clinical techniques have been implemented to approximate the location of the sural nerve. The results of the present study offer surgeons another method, that is less intensive, to locate reliably and subsequently avoid damage to the sural nerve during calcaneal (Achilles) tendon repair and other procedures of the posterolateral leg and ankle. (C) 2013 by the American College of Foot and Ankle Surgeons. All rights reserved.
C1 [Atsas, Stavros; Clarkson, Mackenzie J.; Fox, Jacob N.; Daney, Blake T.; Dodson, Sean C.; Lambert, H. Wayne] W Virginia Univ, Dept Neurobiol & Anat, Sch Med, Robert C Byrd Hlth Sci Ctr, Morgantown, WV 26506 USA.
   [Blackmon, Joseph A.] Univ Kansas, Med Ctr, Dept Internal Med, Div Dermatol, Kansas City, KS 66103 USA.
   [Blackmon, Joseph A.; Atsas, Stavros; Lambert, H. Wayne] Univ Louisville, Hlth Sci Ctr, Dept Anat Sci & Neurobiol, Louisville, KY 40292 USA.
   [Daney, Blake T.] Akron Gen Med Ctr, Dept Orthopaed Surg, Akron, OH USA.
RP Lambert, HW (通讯作者)，W Virginia Univ, Dept Neurobiol & Anat, Sch Med, Robert C Byrd Hlth Sci Ctr, HSN 4052,POB 9128, Morgantown, WV 26506 USA.
EM hwlambert@hsc.wvu.edu
OI Lambert, H. Wayne/0000-0002-0202-6779
FU West Virginia Clinical and Translational Science Institute, West
   Virginia University
FX The authors thank Dr. Edward D. Tillett, Department of Orthopaedic
   Surgery, University of Louisville, for suggesting the pursuit of this
   research project according to his surgical expertise; Dr. Ferrell R.
   Campbell, Department of Anatomical Sciences and Neurobiology, University
   of Louisville, for his mentoring and insight; Michael J. Wells for
   helping with the sural nerve dissections; and the West Virginia Clinical
   and Translational Science Institute, West Virginia University for its
   support and expertise.
CR Apaydin N, 2009, SURG RADIOL ANAT, V31, P775, DOI 10.1007/s00276-009-0520-0
   BRADLEY JP, 1990, AM J SPORT MED, V18, P188, DOI 10.1177/036354659001800213
   Buchgraber A, 1997, CLIN ORTHOP RELAT R, P113
   Calder JDF, 2006, FOOT ANKLE INT, V27, P93, DOI 10.1177/107110070602700204
   CETTI R, 1993, AM J SPORT MED, V21, P791, DOI 10.1177/036354659302100606
   Citak M, 2007, BRIT J SPORT MED, V41, P456, DOI 10.1136/bjsm.2006.031328
   Connolly ES, 1996, NEUROSURGERY, P3243
   Cretnik A, 2005, AM J SPORT MED, V33, P1369, DOI 10.1177/0363546504271501
   Feldbrin Z, 2010, ISR MED ASSOC J, V12, P609
   FITZGIBBONS RE, 1993, AM J SPORT MED, V21, P724, DOI 10.1177/036354659302100516
   Flavin R, 2007, INJURY, V38, P845, DOI 10.1016/j.injury.2006.10.016
   Fortis AP, 2008, ARTHROSCOPY, V24, P683, DOI 10.1016/j.arthro.2008.02.018
   Garrido Ignacio Martinez, 2010, Foot Ankle Surg, V16, P189, DOI 10.1016/j.fas.2009.10.014
   Halasi T, 2003, KNEE SURG SPORT TR A, V11, P409, DOI 10.1007/s00167-003-0397-0
   HOCKENBURY RT, 1990, FOOT ANKLE, V11, P67, DOI 10.1177/107110079001100202
   HUELKE DF, 1957, AM J PHYS ANTHROPOL, V15, P137, DOI 10.1002/ajpa.1330150115
   Ikiz ZAA, 2005, FOOT ANKLE INT, V26, P560, DOI 10.1177/107110070502600712
   Jung HG, 2008, FOOT ANKLE INT, V29, P803, DOI 10.3113/FAI.2008.0803
   KAKIUCHI M, 1995, J BONE JOINT SURG BR, V77B, P60, DOI 10.1302/0301-620X.77B1.7822398
   Khan RJK, 2005, J BONE JOINT SURG AM, V87A, P2202, DOI 10.2106/JBJS.D.03049
   KLEIN W, 1991, Chirurgia degli Organi di Movimento, V76, P223
   Kosinski C, 1926, J ANAT, V60, P274
   Lansdaal JR, 2007, INJURY, V38, P839, DOI 10.1016/j.injury.2006.12.010
   LI Z, 1990, BRIT J PLAST SURG, V43, P546
   Lim J, 2001, FOOT ANKLE INT, V22, P559, DOI 10.1177/107110070102200705
   Lo IKY, 1997, CLIN J SPORT MED, V7, P207, DOI 10.1097/00042752-199707000-00010
   MA GWC, 1977, CLIN ORTHOP RELAT R, P247
   Maes Renaud, 2006, Acta Orthop Belg, V72, P179
   Maffulli N, 1999, J BONE JOINT SURG AM, V81A, P1019, DOI 10.2106/00004623-199907000-00017
   Maffulli N, 1999, CLIN J SPORT MED, V9, P157, DOI 10.1097/00042752-199907000-00007
   Mahakkanukrauh P, 2002, CLIN ANAT, V15, P263, DOI 10.1002/ca.10016
   Majewski M, 2006, AM J SPORT MED, V34, P793, DOI 10.1177/0363546505283266
   McClelland D, 2002, J ROY COLL SURG EDIN, V47, P613
   McGillicuddy JE, 1996, NEUROSURGERY, P3185
   Mestdagh H, 2001, SURG RADIOL ANAT, V23, P307, DOI 10.1007/s00276-001-0307-4
   Moller M, 2001, J BONE JOINT SURG BR, V83B, P843
   Moore KL, 2010, CLIN ORIENTED ANATOM, P619
   Olney RK, 1999, ELECTRODIAGNOSIS CLI, P708
   Ong B C, 2001, Am J Orthop (Belle Mead NJ), V30, P433
   P'an MT, 1939, AM J PHYS ANTHROPOL, V25, P311
   Paavola M, 2000, AM J SPORT MED, V28, P77, DOI 10.1177/03635465000280012501
   Pavic R, 2008, INJURY, V39, P499, DOI 10.1016/j.injury.2007.11.423
   Rebeccato A, 2001, FOOT ANKLE SURG, V40, P188
   Richardson EG, 1999, CAMPBELLS OPERATIVE, P862
   ROWLEY DI, 1982, INJURY, V14, P252, DOI 10.1016/0020-1383(82)90138-3
   Sarrafian SK, 1993, ANATOMY FOOT ANKLE D, P356
   Schaeffer JP, 1953, MORRIS HUMAN ANATOMY, P1167
   WALTON RL, 1984, PLAST RECONSTR SURG, V74, P76, DOI 10.1097/00006534-198407000-00011
   Webb J, 2000, FOOT ANKLE INT, V21, P475, DOI 10.1177/107110070002100604
   Webb JM, 1999, J BONE JOINT SURG BR, V81B, P877, DOI 10.1302/0301-620X.81B5.9784
   WEI FC, 1994, BRIT J PLAST SURG, V47, P544, DOI 10.1016/0007-1226(94)90137-6
   WILLIAMS DD, 1954, ANAT REC, V120, P533, DOI 10.1002/ar.1091200302
   Wong J, 2002, AM J SPORT MED, V30, P565, DOI 10.1177/03635465020300041701
NR 53
TC 19
Z9 19
U1 0
U2 2
PU ELSEVIER SCIENCE INC
PI NEW YORK
PA 360 PARK AVE SOUTH, NEW YORK, NY 10010-1710 USA
SN 1067-2516
EI 1542-2224
J9 J FOOT ANKLE SURG
JI J. Foot Ankle Surg.
PD JAN-FEB
PY 2013
VL 52
IS 1
BP 42
EP 47
DI 10.1053/j.jfas.2012.09.010
PG 6
WC Orthopedics; Surgery
WE Science Citation Index Expanded (SCI-EXPANDED)
SC Orthopedics; Surgery
GA 061ZU
UT WOS:000312889700010
PM 23099184
OA Green Accepted
DA 2022-07-22
ER

PT J
AU Brumann, M
   Baumbach, SF
   Mutschler, W
   Polzer, H
AF Brumann, Mareen
   Baumbach, Sebastian F.
   Mutschler, Wolf
   Polzer, Hans
TI Accelerated rehabilitation following Achilles tendon repair after acute
   rupture - Development of an evidence-based treatment protocol
SO INJURY-INTERNATIONAL JOURNAL OF THE CARE OF THE INJURED
LA English
DT Article
DE Achilles tendon rupture; Rehabilitation; Surgical repair;
   Evidence-based; Treatment protocol; Weight bearing; Ankle mobilization;
   Immobilization; Functional treatment
ID WEIGHT-BEARING MOBILIZATION; FUNCTIONAL TREATMENT; SURGICAL REPAIR;
   POSTOPERATIVE REHABILITATION; EARLY IMMOBILIZATION; TENSION;
   QUESTIONNAIRE; TENDINOPATHY; GUIDELINES; TEARS
AB The acute rupture of the Achilles tendon is a protracted injury. Surgery is only the beginning of a long rehabilitation period. Therefore, the rehabilitation protocol is an integral aspect to restore the pre-injury activity level. Despite several trials available comparing different treatment regimes, there is still no consensus regarding the optimal protocol. Consequently, the aim of our study was to systematically search the evidence available and define a precise rehabilitation programme after operative repair of acute Achilles tendon rupture based on the trials with the highest level of evidence. We performed a systematic literature search in Medline, Embase and Cochrane library.
   We identified twelve randomized controlled trials comparing different treatment regimes after operative repair of the Achilles tendon.
   Five trials compared full to non weight bearing, all applying immobilization in equinus. Immediate full weight bearing led to significant higher patient satisfaction, earlier ambulation and return to preinjury activity. Four trials compared early ankle mobilization to immobilization. All trials found mobilization to be superior as it shortens time to return to work and sports significantly. Three trials compared the combination of full weight bearing and early ankle mobilization to immobilization. This combination was most beneficial. Patients showed significantly higher satisfaction, less use of rehabilitation resources, earlier return to pre-injury activities and further demonstrated significantly increased calf muscle strength, reduced atrophy and tendon elongation. No study found an increased rerupture rate for the more progressive treatment.
   In conclusion, the rehabilitation protocol after Achilles tendon repair should allow immediate full weight bearing. After the second postoperative week controlled ankle mobilization by free plantar flexion and limited dorsiflexion at 08 should be applied. (C) 2014 Elsevier Ltd. All rights reserved.
C1 [Brumann, Mareen; Baumbach, Sebastian F.; Mutschler, Wolf; Polzer, Hans] Univ Munich, Munich Univ Hosp, Dept Trauma Surg, D-80336 Munich, Germany.
RP Polzer, H (通讯作者)，Univ Munich, Munich Univ Hosp, Dept Trauma Surg, Campus Innenstadt,Nussbaumstr 20, D-80336 Munich, Germany.
EM Hans.Polzer@med.uni-muenchen.de
RI Polzer, Hans/Q-3864-2019; Baumbach, Sebastian F./AAQ-3224-2021
OI Polzer, Hans/0000-0002-6713-4017; Baumbach,
   Sebastian/0000-0002-6287-6206
CR Barfod KW, 2013, J FOOT ANKLE SURG, V52, P629, DOI 10.1053/j.jfas.2013.04.012
   BOYDEN EM, 1995, CLIN ORTHOP RELAT R, P150
   Brooks R, 1996, HEALTH POLICY, V37, P53, DOI 10.1016/0168-8510(96)00822-6
   Calder JDF, 2005, BRIT J SPORT MED, V39, P857, DOI 10.1136/bjsm.2004.017509
   CETTI R, 1994, CLIN ORTHOP RELAT R, P155
   Chiodo CP, 2010, J BONE JOINT SURG AM, V92A, P2466
   Coleman BD, 2000, SCAND J MED SCI SPOR, V10, P2, DOI 10.1034/j.1600-0838.2000.010001002.x
   Cook JL, 1997, BRIT J SPORT MED, V31, P332, DOI 10.1136/bjsm.31.4.332
   Costa ML, 2003, INJURY, V34, P874, DOI 10.1016/S0020-1383(02)00205-X
   Costa ML, 2006, J BONE JOINT SURG BR, V88B, P69, DOI 10.1302/0301-620X.88B1.16549
   Hays RD, 2001, ANN MED, V33, P350, DOI 10.3109/07853890109002089
   Huang J, 2014, AM J SPORTS MED
   Kangas J, 2003, J TRAUMA, V54, P1171, DOI 10.1097/01.TA.0000047945.20863.A2
   Kangas J, 2007, AM J SPORT MED, V35, P59, DOI 10.1177/0363546506293255
   Kauranen K, 2002, FOOT ANKLE INT, V23, P600, DOI 10.1177/107110070202300703
   Kearney RS, 2012, J BONE JOINT SURG BR, V94B, P28, DOI 10.1302/0301-620X.94B1.28008
   Kearney RS, 2012, PHYSIOTHERAPY, V98, P24, DOI 10.1016/j.physio.2011.04.349
   Kerkhoffs GMMJ, 2002, ARCH ORTHOP TRAUM SU, V122, P102, DOI 10.1007/s004020100312
   Lansdaal JR, 2007, INJURY, V38, P839, DOI 10.1016/j.injury.2006.12.010
   Leppilahti J, 1998, CLIN ORTHOP RELAT R, P152
   Longo UG, 2012, KNEE SURG SPORT TR A, V20, P1392, DOI 10.1007/s00167-011-1868-3
   Maffulli N, 2003, AM J SPORT MED, V31, P692, DOI 10.1177/03635465030310051001
   Maffulli N, 2003, J SPORT MED PHYS FIT, V43, P367
   Majewski M, 2008, DISABIL REHABIL, V30, P1726, DOI 10.1080/09638280701786831
   McMahon Samuel E, 2011, Foot Ankle Surg, V17, P211, DOI 10.1016/j.fas.2010.11.001
   Mortensen NHM, 1999, J BONE JOINT SURG AM, V81A, P983, DOI 10.2106/00004623-199907000-00011
   Nilsson-Helander K, 2007, AM J SPORT MED, V35, P421, DOI 10.1177/0363546506294856
   Olsson N, 2013, AM J SPORT MED, V41, P2867, DOI 10.1177/0363546513503282
   Robinson JM, 2001, BRIT J SPORT MED, V35, P335, DOI 10.1136/bjsm.35.5.335
   Sadoghi P, 2012, INT ORTHOP, V36, P1947, DOI 10.1007/s00264-012-1533-6
   Schepull T, 2013, AM J SPORT MED, V41, P2550, DOI 10.1177/0363546513501785
   Suchak AA, 2008, J BONE JOINT SURG AM, V90A, P1876, DOI 10.2106/JBJS.G.01242
   Suchak AA, 2006, CLIN ORTHOP RELAT R, P216, DOI 10.1097/01.blo.0000203458.05135.74
   Thevendran G, 2013, Musculoskelet Surg, V97, P9, DOI 10.1007/s12306-013-0251-6
   Wright JG, 2003, J BONE JOINT SURG AM, V85A, P1, DOI 10.2106/00004623-200301000-00001
NR 35
TC 51
Z9 54
U1 2
U2 33
PU ELSEVIER SCI LTD
PI OXFORD
PA THE BOULEVARD, LANGFORD LANE, KIDLINGTON, OXFORD OX5 1GB, OXON, ENGLAND
SN 0020-1383
EI 1879-0267
J9 INJURY
JI Injury-Int. J. Care Inj.
PD NOV
PY 2014
VL 45
IS 11
BP 1782
EP 1790
DI 10.1016/j.injury.2014.06.022
PG 9
WC Critical Care Medicine; Emergency Medicine; Orthopedics; Surgery
WE Science Citation Index Expanded (SCI-EXPANDED)
SC General & Internal Medicine; Emergency Medicine; Orthopedics; Surgery
GA AR9ME
UT WOS:000343898000020
PM 25059505
DA 2022-07-22
ER

PT J
AU Westin, O
   Svedman, S
   Senorski, EH
   Svantesson, E
   Nilsson-Helander, K
   Karlsson, J
   Ackerman, P
   Samuelsson, K
AF Westin, Olof
   Svedman, Simon
   Senorski, Eric Hamrin
   Svantesson, Eleonor
   Nilsson-Helander, Katarina
   Karlsson, Jon
   Ackerman, Paul
   Samuelsson, Kristian
TI Older Age Predicts Worse Function 1 Year After an Acute Achilles Tendon
   Rupture: A Prognostic Multicenter Study on 391 Patients
SO ORTHOPAEDIC JOURNAL OF SPORTS MEDICINE
LA English
DT Article
DE Achilles tendon; rupture; treatment; predictor; ATRS; heel-rise height
ID DEEP-VEIN THROMBOSIS; SMOKING-CESSATION; COMPRESSION; ENDURANCE;
   DEFICITS; SURGERY
AB Background: There is limited evidence regarding the patient-related factors that influence treatment outcomes after an acute Achilles tendon rupture. Purpose/Hypothesis: The purpose of this study was to determine the predictors of functional and patient-reported outcomes 1 year after an acute Achilles tendon rupture using a multicenter cohort and to determine patient characteristics for reporting within the top and bottom 10% of the Achilles tendon Total Rupture Score (ATRS) and heel-rise height outcomes. The hypothesis was that older age, greater body mass index (BMI), and female sex would lead to inferior outcomes. Study Design: Cohort study; Level of evidence, 2. Methods: Patients were selected by combining 5 randomized controlled trials from 2 different centers in Sweden. Functional outcomes were assessed using validated heel-rise tests (height, number of repetitions, total work, and concentric power) for muscular endurance and strength, and the relationship between injured and uninjured legs was calculated as the limb symmetry index (LSI). Patient-reported outcomes were measured using the ATRS. All outcomes were collected at the 1-year follow-up. Independent predictors included were patient sex, smoking, BMI, age, and surgical versus nonsurgical treatment. Results: Of the 391 included patients, 307 (79%) were treated surgically. The LSI of heel-rise height at the 1-year follow-up decreased by approximately 4% for every 10-year increment in age (beta, -3.94 [95% CI, -6.19 to -1.69]; P = .0006). In addition, every 10-year increment in age resulted in a 1.79-fold increase in the odds of being in the lowest 10% of the LSI of heel-rise height. Moreover, a nonsignificant superior LSI of heel-rise height was found in patients treated surgically compared with nonsurgical treatment (beta, -4.49 [95% CI, -9.14 to 0.16]; P = .058). No significant predictor was related to the ATRS. Smoking, patient sex, and BMI did not significantly affect the 1-year results for the LSI of the heel-rise tests. Conclusion: Older age at the time of injury negatively affected heel-rise height 1 year after an Achilles tendon rupture. Irrespective of age, a nonsignificant relationship toward the superior recovery of heel-rise height was seen in patients treated surgically. None of the factors studied affected patient-reported outcomes.
C1 [Westin, Olof; Nilsson-Helander, Katarina; Karlsson, Jon; Samuelsson, Kristian] Sahlgrens Univ Hosp, Dept Orthopaed, Molndal, Sweden.
   [Westin, Olof; Svantesson, Eleonor; Nilsson-Helander, Katarina; Karlsson, Jon; Samuelsson, Kristian] Univ Gothenburg, Sahlgrenska Acad, Inst Clin Sci, Dept Orthopaed, Gothenburg, Sweden.
   [Svedman, Simon] Karolinska Inst, Dept Mol Med & Surg, Integrat Orthoped Lab, Stockholm, Sweden.
   [Senorski, Eric Hamrin] Univ Gothenburg, Sahlgrenska Acad, Inst Neurosci & Physiol, Dept Hlth & Rehabil, Gothenburg, Sweden.
   [Ackerman, Paul] Karolinska Univ Hosp, Dept Orthoped Surg, Stockholm, Sweden.
RP Westin, O (通讯作者)，Univ Gothenburg, Sahlgrenska Acad, Inst Clin Sci, Dept Orthopaed, S-43180 Molndal, Sweden.
EM olof.westin@gmail.com
RI Helander, Katarina Nilsson/AAE-1793-2020; W, Ackermann P/B-9714-2008;
   Senorski, Eric Hamrin/AAA-2913-2019
OI Helander, Katarina Nilsson/0000-0002-1292-5102; W, Ackermann
   P/0000-0002-5520-169X; Senorski, Eric Hamrin/0000-0002-9340-0147
CR Alim MA, 2016, BMJ OPEN SPORT EXERC, V2, P1
   Aujla R, 2018, FOOT ANKLE SURG, V24, P336, DOI 10.1016/j.fas.2017.03.007
   Birch HL, 2016, ADV EXP MED BIOL, V920, P247, DOI 10.1007/978-3-319-33943-6_24
   Bostick GP, 2010, J ORTHOP SPORT PHYS, V40, P345, DOI 10.2519/jospt.2010.3204
   Deng SL, 2017, J FOOT ANKLE SURG, V56, P1236, DOI 10.1053/j.jfas.2017.05.036
   Domeij-Arverud E, 2016, BONE JOINT J, V98B, P1635, DOI 10.1302/0301-620X.98B12.BJJ-2016-0008.R1
   Domeij-Arverud E, 2015, BONE JOINT J, V97B, P675, DOI 10.1302/0301-620X.97B5.34581
   Domeij-Arverud E, 2013, BONE JOINT J, V95B, P1227, DOI 10.1302/0301-620X.95B9.31162
   Ganestam A, 2016, KNEE SURG SPORT TR A, V24, P3730, DOI 10.1007/s00167-015-3544-5
   Ganestam A, 2013, J FOOT ANKLE SURG, V52, P736, DOI 10.1053/j.jfas.2013.07.004
   Glassman SD, 2000, SPINE, V25, P2608, DOI 10.1097/00007632-200010150-00011
   Hardin EC, 2004, MED SCI SPORT EXER, V36, P838, DOI 10.1249/01.MSS.0000126605.65966.40
   Horstmann T, 2012, INT J SPORTS MED, V33, P474, DOI 10.1055/s-0032-1301932
   Huttunen TT, 2014, AM J SPORT MED, V42, P2419, DOI 10.1177/0363546514540599
   Jackson KL, 2016, GLOB SPINE J, V6, P695, DOI 10.1055/s-0036-1571285
   Jandali Z, 2018, J RECONSTR MICROSURG, V34, P632, DOI 10.1055/s-0038-1660830
   Leppilahti J, 1996, INT J SPORTS MED, V17, P619, DOI 10.1055/s-2007-972905
   Levi N, 1997, INJURY, V28, P311, DOI 10.1016/S0020-1383(96)00200-8
   McNair P, 2013, J ORTHOP RES, V31, P1469, DOI 10.1002/jor.22381
   Mullaney MJ, 2006, AM J SPORT MED, V34, P1120, DOI 10.1177/0363546505284186
   Nilsson-Helander K, 2007, AM J SPORT MED, V35, P421, DOI 10.1177/0363546506294856
   Nilsson-Helander K, 2010, AM J SPORT MED, V38, P2186, DOI 10.1177/0363546510376052
   Olsson N, 2014, AM J SPORT MED, V42, P1448, DOI 10.1177/0363546514527409
   Olsson N, 2013, AM J SPORT MED, V41, P2867, DOI 10.1177/0363546513503282
   Olsson N, 2011, KNEE SURG SPORT TR A, V19, P1385, DOI 10.1007/s00167-011-1511-3
   Pauly F, 2013, J PROTEOME RES, V12, P5943, DOI 10.1021/pr4003245
   Santiago-Torres J, 2015, AM J SPORT MED, V43, P745, DOI 10.1177/0363546514533776
   Silbernagel KG, 2006, KNEE SURG SPORT TR A, V14, P1207, DOI 10.1007/s00167-006-0150-6
   Silbernagel KG, 2015, ORTHOP J SPORTS MED, V3, DOI 10.1177/2325967115586768
   Silbernagel KG, 2010, KNEE SURG SPORT TR A, V18, P258, DOI 10.1007/s00167-009-0889-7
   Svedman S, 2018, KNEE SURG SPORT TR A, V26, P2011, DOI 10.1007/s00167-017-4606-7
   Wang JHC, 2006, J BIOMECH, V39, P1563, DOI 10.1016/j.jbiomech.2005.05.011
   Zhang H, 2015, MEDICINE, V94, DOI 10.1097/MD.0000000000001951
NR 33
TC 5
Z9 5
U1 0
U2 4
PU SAGE PUBLICATIONS INC
PI THOUSAND OAKS
PA 2455 TELLER RD, THOUSAND OAKS, CA 91320 USA
EI 2325-9671
J9 ORTHOP J SPORTS MED
JI Orthop. J. Sports Med.
PD DEC 26
PY 2018
VL 6
IS 12
AR 2325967118813904
DI 10.1177/2325967118813904
PG 15
WC Orthopedics; Sport Sciences
WE Science Citation Index Expanded (SCI-EXPANDED)
SC Orthopedics; Sport Sciences
GA HF7BJ
UT WOS:000454392800001
PM 30627587
OA gold, Green Published
DA 2022-07-22
ER

PT J
AU Brorsson, A
   Willy, RW
   Tranberg, R
   Silbernagel, KG
AF Brorsson, Annelie
   Willy, Richard W.
   Tranberg, Roy
   Silbernagel, Karin Gravare
TI Heel-Rise Height Deficit 1 Year After Achilles Tendon Rupture Relates to
   Changes in Ankle Biomechanics 6 Years After Injury
SO AMERICAN JOURNAL OF SPORTS MEDICINE
LA English
DT Article
DE Achilles tendon rupture; ankle; biomechanics; heel-rise test
ID PHYSICAL-ACTIVITY; RANDOMIZED-TRIAL; SURGICAL REPAIR; RELIABILITY;
   LENGTH; JOINT; VALIDATION; ELONGATION; ENDURANCE; RECOVERY
AB Background: It is unknown whether the height of a heel-rise performed in the single-leg standing heel-rise test 1 year after an Achilles tendon rupture (ATR) correlates with ankle biomechanics during walking, jogging, and jumping in the long-term.
   Purpose: To explore the differences in ankle biomechanics, tendon length, calf muscle recovery, and patient-reported outcomes at a mean of 6 years after ATR between 2 groups that, at 1-year follow-up, had less than 15% versus greater than 30% differences in heel-rise height.
   Study Design: Cohort study;
   Methods: Seventeen patients with less than 15% (<15% group) and 17 patients with greater than 30% (>30% group) side-toside difference in heel-rise height at 1 year after ATR were evaluated at a mean (SD) 6.1 (2.0) years after their ATR. Ankle kinematics and kinetics were sampled via standard motion capture procedures during walking, jogging, and jumping. Patientreported outcome was evaluated with Achilles tendon Total Rupture Score (ATRS), Physical Activity Scale (PAS), and Foot and Ankle Outcome Score (FAOS). Tendon length was evaluated by ultrasonography. The Limb Symmetry Index (LSI = [Injured Side divided by Healthy Side x 3 100) was calculated for side differences.
   Results: The.30% group had significantly more deficits in ankle kinetics during all activities compared with patients in the <15% group at a mean of 6 years after ATR (LSI, 70%-149% and 84%-106%, respectively; P =.010-.024). The.30% group, compared with the <5% group, also had significantly lower values in heel-rise height (LSI, 72% and 95%, respectively; P<. 001) and heel-rise work (LSI, 58% and 91%, respectively; P<. 001) and significantly larger side-to-side difference in tendon length (114% and 106%, respectively; P =.012). Achilles tendon length correlated with ankle kinematic variables (r = 0.38-0.44; P =.015-.027) whereas heel-rise work correlated with kinetic variables (r = 20.57 to 0.56; P =.001-.047). LSI tendon length correlated negatively with LSI heel-rise height (r = 20.41; P =.018). No differences were found between groups in patient-reported outcome (P =.143-.852).
   Conclusion: Height obtained during the single-leg standing heel-rise test performed 1 year after ATR related to the long-term ability to regain normal ankle biomechanics. Minimizing tendon elongation and regaining heel-rise height may be important for the long-term recovery of ankle biomechanics, particularly during more demanding activities such as jumping.
C1 [Brorsson, Annelie; Willy, Richard W.; Tranberg, Roy; Silbernagel, Karin Gravare] Univ Gothenburg, Sahlgrenska Acad, Inst Clin Sci, Dept Orthopaed, Gothenburg, Sweden.
   [Willy, Richard W.] East Carolina Univ, Coll Allied Hlth Sci, Dept Phys Therapy, Greenville, NC USA.
   [Silbernagel, Karin Gravare] Univ Delaware, Dept Phys Therapy, Newark, DE USA.
   [Willy, Richard W.] Univ Montana, Sch Phys Therapy & Movement Sci, Missoula, MT 59812 USA.
RP Brorsson, A (通讯作者)，Univ Gothenburg, Sahlgrenska Acad, Inst Clin Sci, Dept Orthopaed, Gothenburg, Sweden.
EM annelie.brorsson@orthop.gu.se
RI Brorsson, Annelie/AAD-5986-2020; Tranberg, Roy/AAD-8901-2020;
   VARRECCHIA, TIWANA/AAJ-8712-2021
OI Willy, Richard/0000-0002-1249-228X; Gravare Silbernagel,
   Karin/0000-0001-7566-407X
CR Agres AN, 2015, SCAND J MED SCI SPOR, V25, P860, DOI 10.1111/sms.12456
   Brorsson A, 2016, SCAND J MED SCI SPOR, V26, P844, DOI 10.1111/sms.12533
   COHEN J, 1992, PSYCHOL BULL, V112, P155, DOI 10.1037/0033-2909.112.1.155
   DEMPSTER WT, 1959, AM J PHYS ANTHROPOL, V17, P289, DOI 10.1002/ajpa.1330170405
   Don R, 2007, CLIN BIOMECH, V22, P211, DOI 10.1016/j.clinbiomech.2006.10.001
   Ferber R, 2002, J ORTHOPAED RES, V20, P1139, DOI 10.1016/S0736-0266(02)00077-3
   FUKASHIRO S, 1995, EUR J APPL PHYSIOL O, V71, P453, DOI 10.1007/BF00635880
   Fukunaga T, 2001, P ROY SOC B-BIOL SCI, V268, P229, DOI 10.1098/rspb.2000.1361
   Grimby G, 2015, SCAND J MED SCI SPOR, V25, P119, DOI 10.1111/sms.12611
   GRIMBY G, 1986, ACTA MED SCAND, P233
   Holm C, 2015, SCAND J MED SCI SPOR, V25, pE1, DOI 10.1111/sms.12209
   Horstmann T, 2012, INT J SPORTS MED, V33, P474, DOI 10.1055/s-0032-1301932
   Kangas J, 2007, AM J SPORT MED, V35, P59, DOI 10.1177/0363546506293255
   Kristianslund E, 2012, J BIOMECH, V45, P666, DOI 10.1016/j.jbiomech.2011.12.011
   Kuo AD, 2005, EXERC SPORT SCI REV, V33, P88, DOI 10.1097/00003677-200504000-00006
   Lantto I, 2015, AM J SPORT MED, V43, P2302, DOI 10.1177/0363546515591267
   Mullaney MJ, 2006, AM J SPORT MED, V34, P1120, DOI 10.1177/0363546505284186
   Nilsson-Helander K, 2007, AM J SPORT MED, V35, P421, DOI 10.1177/0363546506294856
   Nilsson-Helander K, 2010, AM J SPORT MED, V38, P2186, DOI 10.1177/0363546510376052
   Noehren B, 2010, J ORTHOP RES, V28, P1405, DOI 10.1002/jor.21172
   Oda H, 2017, SPORTS MED, V47, P1221, DOI 10.1007/s40279-016-0629-3
   Olsson N, 2014, AM J SPORT MED, V42, P1448, DOI 10.1177/0363546514527409
   Olsson N, 2013, AM J SPORT MED, V41, P2867, DOI 10.1177/0363546513503282
   Olsson N, 2011, KNEE SURG SPORT TR A, V19, P1385, DOI 10.1007/s00167-011-1511-3
   Roos EM, 2001, FOOT ANKLE INT, V22, P788, DOI 10.1177/107110070102201004
   Rosso C, 2013, KNEE SURG SPORT TR A, V21, P1369, DOI 10.1007/s00167-013-2407-1
   SALTIN B, 1968, CIRCULATION, V38, P1104, DOI 10.1161/01.CIR.38.6.1104
   Schepull T, 2013, AM J SPORT MED, V41, P2550, DOI 10.1177/0363546513501785
   Schepull T, 2007, BMC MUSCULOSKEL DIS, V8, DOI 10.1186/1471-2474-8-116
   Silbernagel KG, 2012, AM J SPORT MED, V40, P1564, DOI 10.1177/0363546512447926
   Silbernagel KG, 2012, J ORTHOP SPORT PHYS, V42, P521, DOI 10.2519/jospt.2012.3913
   Silbernagel KG, 2006, KNEE SURG SPORT TR A, V14, P1207, DOI 10.1007/s00167-006-0150-6
   Silbernagel KG, 2016, MLTJ-MUSCLE LIGAMENT, V6, P104, DOI 10.11138/mltj/2016.6.1.104
   Silbernagel KG, 2010, KNEE SURG SPORT TR A, V18, P258, DOI 10.1007/s00167-009-0889-7
   SPOOR CW, 1990, J BIOMECH, V23, P1247, DOI 10.1016/0021-9290(90)90382-D
   Suydam SM, 2015, KNEE SURG SPORT TR A, V23, P868, DOI 10.1007/s00167-013-2512-1
   Tengman T, 2013, ORTHOP J SPORTS MED, V1, DOI 10.1177/2325967113504734
   Voleti PB, 2012, ANNU REV BIOMED ENG, V14, P47, DOI 10.1146/annurev-bioeng-071811-150122
   Willy RW, 2017, AM J SPORT MED, V45, P1124, DOI 10.1177/0363546516685055
   Willy RW, 2016, J ORTHOP SPORT PHYS, V46, P664, DOI 10.2519/jospt.2016.6494
   Zellers JA, 2016, BRIT J SPORT MED, V50, P1325, DOI 10.1136/bjsports-2016-096106
NR 41
TC 38
Z9 38
U1 1
U2 12
PU SAGE PUBLICATIONS INC
PI THOUSAND OAKS
PA 2455 TELLER RD, THOUSAND OAKS, CA 91320 USA
SN 0363-5465
EI 1552-3365
J9 AM J SPORT MED
JI Am. J. Sports Med.
PD NOV
PY 2017
VL 45
IS 13
BP 3060
EP 3068
DI 10.1177/0363546517717698
PG 9
WC Orthopedics; Sport Sciences
WE Science Citation Index Expanded (SCI-EXPANDED)
SC Orthopedics; Sport Sciences
GA FL3TK
UT WOS:000414146500022
PM 28783473
DA 2022-07-22
ER

PT J
AU Fox, G
   Gabbe, BJ
   Richardson, M
   Oppy, A
   Page, R
   Edwards, ER
   Hau, R
   Ekegren, CL
AF Fox, G.
   Gabbe, B. J.
   Richardson, M.
   Oppy, A.
   Page, R.
   Edwards, E. R.
   Hau, R.
   Ekegren, C. L.
TI Twelve-month outcomes following surgical repair of the Achilles tendon
SO INJURY-INTERNATIONAL JOURNAL OF THE CARE OF THE INJURED
LA English
DT Article
DE Achilles tendon; Injury; Surgery; Outcomes; Extended Glasgow Outcome
   Scale; Return to work; Quality of life
ID QUALITY-OF-LIFE; RUPTURES; REHABILITATION
AB Introduction: Incidence of Achilles tendon rupture (ATR) has increased over recent years, and debate regarding optimal management has been widely documented. Most papers have focused on surgical success, complications and short term region-specific outcomes. Inconsistent use of standardised outcome measures following surgical ATR repair has made it difficult to evaluate the impact of ATR on a patient's health status post-surgery, and to compare this to other injury types. This study aimed to report the frequency of surgical repairs of the Achilles tendon over a five-year period within an orthopaedic trauma registry, and to investigate return to work (RTW) status, health status and functional outcomes at 12 months post-surgical repair of the Achilles tendon.
   Methods: Two hundred and four adults registered by the Victorian Orthopaedic Trauma Outcomes Registry (VOTOR) who underwent surgical repair of the Achilles tendon between July 2009 and June 2014 were included in this prospective cohort study. The Extended Glasgow Outcome Scale (GOS-E), 3-level European Quality of Life 5 Dimension measure (EQ-5D-3L), and RTW status 12 months following surgical ATR repair were collected through structured telephone interviews conducted by trained interviewers.
   Results: At 12 months, 92% of patients were successfully followed up. Of those working prior to injury, 95% had returned to work. 42% of patients reported a full recovery on the GOS-E scale. The prevalence of problems on the EQ-5D-3L at 12 months was 0.5% for self-care, 11% for anxiety, 13% for mobility, 16% for activity, and 22% for pain. 16% of patients reported problems with more than one domain. The number of surgical repairs of the Achilles tendon within the VOTOR registry decreased by 68% over the five-year study period.
   Conclusions: Overall, patients recover well following surgical repair of the Achilles tendon. However, in this study, deficits in function persisted for over half of patients at 12 months post-injury. The decreased incidence of surgical Achilles tendon repair may reflect a change in practice at VOTOR hospitals whereby surgery may be becoming less favoured for initial ATR management. (C) 2016 Elsevier Ltd. All rights reserved.
C1 [Fox, G.; Gabbe, B. J.; Edwards, E. R.; Hau, R.; Ekegren, C. L.] Monash Univ, Dept Epidemiol & Prevent Med, Melbourne, Vic, Australia.
   [Richardson, M.] Epworth Med Fdn, Richmond, Australia.
   [Oppy, A.] Royal Melbourne Hosp, Dept Orthopaed Surg, Parkville, Vic, Australia.
   [Page, R.] Univ Hosp Geelong, Dept Orthopaed, Geelong, Vic, Australia.
   [Page, R.] Deakin Univ, Sch Med, Geelong, Vic, Australia.
   [Edwards, E. R.] Alfred Hosp, Dept Orthopaed Surg, Melbourne, Vic, Australia.
   [Hau, R.] Northern Hosp, Epping, NSW, Australia.
RP Ekegren, CL (通讯作者)，Monash Univ, Alfred Ctr, 99 Commercial Rd, Melbourne, Vic 3004, Australia.
EM christina.ekegren@monash.edu
RI Ekegren, Christina L./AAX-7887-2021; Richardson, Martin/H-2272-2018;
   Edwards, Elton R/AAP-4279-2020; Page, Richard/K-6327-2015
OI Ekegren, Christina L./0000-0002-7656-6209; Richardson,
   Martin/0000-0002-1280-3834; Edwards, Elton R/0000-0002-9455-8069; Page,
   Richard/0000-0002-2225-7144; Gabbe, Belinda/0000-0001-7096-7688
FU Transport Accident Commission via the Institute for Safety, Compensation
   and Recovery Research (ISCRR); National Health and Medical Research
   Council of Australia [GNT1106633, GNT1048731]
FX Melissa Hart, Sue McLellan, Adrian Buzgau, David Attwood and the
   Steering Committee of VOTOR are thanked for their assistance with this
   project. The Victorian Injury Surveillance Unit (VISU), Monash
   University is acknowledged for the provision and preliminary analysis of
   data from the Victorian Admitted Episodes Dataset (VAED). The Victorian
   Orthopaedic Trauma Outcomes Registry (VOTOR) is funded by the Transport
   Accident Commission via the Institute for Safety, Compensation and
   Recovery Research (ISCRR). Christina Ekegren is supported by a National
   Health and Medical Research Council of Australia Early Career Fellowship
   (GNT1106633). Belinda Gabbe was supported by a National Health and
   Medical Research Council of Australia Career Development Fellowship
   (GNT1048731).
CR Arslan A, 2014, ACTA ORTHOP TRAUMATO, V48, P563, DOI 10.3944/AOTT.2014.14.0132
   Bergkvist D, 2012, J BONE JOINT SURG AM, V94A, P1229, DOI 10.2106/JBJS.J.01601
   Bostick GP, 2010, J ORTHOP SPORT PHYS, V40, P345, DOI 10.2519/jospt.2010.3204
   Brukner P, 2007, CLIN SPORTS MED, P590
   Gabbe BJ, 2010, J TRAUMA, V69, P532, DOI 10.1097/TA.0b013e3181e5125b
   Holm C, 2015, SCAND J MED SCI SPOR, V25, pE1, DOI 10.1111/sms.12209
   Jackson G, 2013, ORTHOPEDICS, V36, pE1053, DOI 10.3928/01477447-20130724-23
   Kearney RS, 2012, BRIT J SPORT MED, V46, P1102, DOI 10.1136/bjsports-2011-090497
   Lantto I, 2015, SCAND J MED SCI SPOR, V25, pE133, DOI 10.1111/sms.12253
   Moller M, 2001, J BONE JOINT SURG BR, V83B, P843
   Olsson N, 2011, KNEE SURG SPORT TR A, V19, P1385, DOI 10.1007/s00167-011-1511-3
   Polinder S, 2010, INJURY PREV, V16, P147, DOI 10.1136/ip.2009.022418
   Rosso C, 2013, KNEE SURG SPORT TR A, V21, P1369, DOI 10.1007/s00167-013-2407-1
   Soroceanu A, 2012, J BONE JOINT SURG AM, V94A, P2136, DOI 10.2106/JBJS.K.00917
   Strauss EJ, 2007, INJURY, V38, P832, DOI 10.1016/j.injury.2006.06.005
   Twaddle BC, 2007, AM J SPORT MED, V35, P2033, DOI 10.1177/0363546507307503
   Willits K, 2010, J BONE JOINT SURG AM, V92A, P2767, DOI 10.2106/JBJS.I.01401
NR 17
TC 6
Z9 6
U1 0
U2 3
PU ELSEVIER SCI LTD
PI OXFORD
PA THE BOULEVARD, LANGFORD LANE, KIDLINGTON, OXFORD OX5 1GB, OXON, ENGLAND
SN 0020-1383
EI 1879-0267
J9 INJURY
JI Injury-Int. J. Care Inj.
PD OCT
PY 2016
VL 47
IS 10
BP 2370
EP 2374
DI 10.1016/j.injury.2016.07.013
PG 5
WC Critical Care Medicine; Emergency Medicine; Orthopedics; Surgery
WE Science Citation Index Expanded (SCI-EXPANDED)
SC General & Internal Medicine; Emergency Medicine; Orthopedics; Surgery
GA EC5IB
UT WOS:000388166500055
PM 27424531
DA 2022-07-22
ER

PT J
AU Jandacka, D
   Zahradnik, D
   Farana, R
   Uchytil, J
   Hamill, J
AF Jandacka, Daniel
   Zahradnik, David
   Farana, Roman
   Uchytil, Jaroslav
   Hamill, Joseph
TI Footfall patterns of a runner with an Achilles tendon rupture
SO JOURNAL OF SPORT AND HEALTH SCIENCE
LA English
DT Article
DE Achilles tendon; Calcaneus; Footfall patterns; Injury; Running; Tendon
   length
ID STRIKE PATTERNS; VALIDATION; INJURIES; BAREFOOT; LENGTH; SCORE
AB Purpose: This study aims to compare the load and the length of previously ruptured and healthy Achilles tendon (AT) of a recreational runner who used different footfall patterns on each limb during running.
   Methods: A 41-year-old recreational athlete with a ruptured AT participated in this report. Two force plates and a high-speed motion capture system were used to collect ground reaction force and kinematic data in shod and barefoot running conditions. AT length was measured using ultrasonography and an infrared camera system. AT force was estimated as the active plantar flexion moment divided by AT moment arm during stance phase.
   Results: The participant used a rearfoot pattern on the affected limb and a forefoot/midfoot pattern on the unaffected limb during shod running, and a forefoot/midfoot pattern during barefoot running. There was no difference between the length of the affected and the unaffected AT. During shod running, the maximal AT force and loading rate were lower in the affected AT versus the unaffected AT. During barefoot running, the affected maximal AT force and loading rate were greater than the unaffected AT.
   Conclusion: Footfall patterns can be an adaptation to reduce the loading on a previously injured AT. It appears that runners may consider using a rearfoot footfall pattern during running to reduce the stress on the AT. (c) 2017 Production and hosting by Elsevier B.V. on behalf of Shanghai University of Sport.
C1 [Jandacka, Daniel; Zahradnik, David; Farana, Roman; Uchytil, Jaroslav; Hamill, Joseph] Univ Ostrava, Human Mot Diagnost Ctr, Dept Human Movement Studies, Ostrava 70200, Czech Republic.
   [Hamill, Joseph] Univ Massachusetts, Dept Kinesiol, Amherst, MA 01003 USA.
RP Jandacka, D (通讯作者)，Univ Ostrava, Human Mot Diagnost Ctr, Dept Human Movement Studies, Ostrava 70200, Czech Republic.
EM daniel.jandacka@osu.cz
RI Jandacka, Daniel/B-6035-2011; Farana, Roman/K-5405-2019
OI Jandacka, Daniel/0000-0003-2653-2569; Farana, Roman/0000-0002-1451-5879
FU University of Ostrava, Czech Republic [SGS 6148]
FX This research has been supported by a grant from the University of
   Ostrava (SGS 6148), Czech Republic.
CR Bressel E, 2004, CLIN BIOMECH, V19, P284, DOI 10.1016/j.clinbiomech.2003.12.008
   Cohen J., 1988, STAT POWER ANAL BEHA, V2nd, DOI DOI 10.4324/9780203771587
   Daoud AI, 2012, MED SCI SPORT EXER, V44, P1325, DOI 10.1249/MSS.0b013e3182465115
   GRIMBY G, 1986, ACTA MED SCAND, P233
   Gruber AH, 2011, 2011 ANN M AM SOC BI
   Gruber AH, 2013, FOOTWEAR SCI, V5, P39, DOI 10.1080/19424280.2012.742141
   Hasegawa H, 2007, J STRENGTH COND RES, V21, P888, DOI 10.1519/R-22096.1
   Jandacka Daniel, 2013, BMJ Case Rep, V2013, DOI 10.1136/bcr-2012-007370
   Khan MH, 2005, CLIN J SPORT MED, V15, P27, DOI 10.1097/00042752-200501000-00006
   Larson P, 2011, J SPORT SCI, V29, P1665, DOI 10.1080/02640414.2011.610347
   Lieberman DE, 2010, NATURE, V463, P531, DOI 10.1038/nature08723
   Lopes AD, 2012, SPORTS MED, V42, P891, DOI [10.2165/11631170-000000000-00000, 10.1007/BF03262301]
   Moraux A, 2013, BMC MUSCULOSKEL DIS, V14, DOI 10.1186/1471-2474-14-104
   Nilsson-Helander K, 2007, AM J SPORT MED, V35, P421, DOI 10.1177/0363546506294856
   Rees JD, 2008, RHEUMATOLOGY, V47, P1493, DOI 10.1093/rheumatology/ken262
   Riener R, 1999, J BIOMECH, V32, P539, DOI 10.1016/S0021-9290(99)00009-3
   Roos EM, 2001, FOOT ANKLE INT, V22, P788, DOI 10.1177/107110070102201004
   Rosso C, 2013, KNEE SURG SPORT TR A, V21, P1369, DOI 10.1007/s00167-013-2407-1
   Saragiotto BT, 2014, SPORTS MED, V44, P1153, DOI 10.1007/s40279-014-0194-6
   Selbie W.S., 2013, RES METHODS BIOMECHA, Vsecond, P35
   Self BP, 2001, MED SCI SPORT EXER, V33, P1338, DOI 10.1097/00005768-200108000-00015
   Silbernagel KG, 2012, J ORTHOP SPORT PHYS, V42, P521, DOI 10.2519/jospt.2012.3913
   Sinclair J, 2014, CLIN BIOMECH, V29, P395, DOI 10.1016/j.clinbiomech.2014.02.004
   WILLIAMS KR, 1987, INT J SPORTS MED, V8, P107
   Zhang JY, 2010, J ORTHOP RES, V28, P198, DOI 10.1002/jor.20962
NR 25
TC 3
Z9 3
U1 0
U2 7
PU SHANGHAI UNIV SPORT
PI SHANGHAI
PA EDITORIAL BOARD, 650 QINGYUANHUAN RD, SHANGHAI, 200438, PEOPLES R CHINA
SN 2095-2546
EI 2213-2961
J9 J SPORT HEALTH SCI
JI J. Sport Health Sci.
PD DEC
PY 2017
VL 6
IS 4
BP 498
EP 502
DI 10.1016/j.jshs.2016.05.004
PG 5
WC Hospitality, Leisure, Sport & Tourism; Sport Sciences
WE Science Citation Index Expanded (SCI-EXPANDED); Social Science Citation Index (SSCI)
SC Social Sciences - Other Topics; Sport Sciences
GA FQ9RM
UT WOS:000418699400020
PM 30356660
OA Green Published, gold
DA 2022-07-22
ER

PT J
AU Borges, PRT
   Santos, TRT
   Procpio, PRS
   Chelidonopoulos, JHD
   Zambelli, R
   Ocarino, JM
AF Borges, Pollyana R. T.
   Santos, Thiago R. T.
   Procpio, Paula R. S.
   Chelidonopoulos, Jessica H. D.
   Zambelli, Roberto
   Ocarino, Juliana M.
TI Passive stiffness of the ankle and plantar flexor muscle performance
   after Achilles tendon repair: a cross-sectional study
SO BRAZILIAN JOURNAL OF PHYSICAL THERAPY
LA English
DT Article
DE Rehabilitation; Achilles tendon rupture; Surgical repair; Plantar
   flexors; Muscular strength; Joint stiffness
ID RUPTURE; RELIABILITY; INSTABILITY; RECOVERY; VALIDITY; WALKING; TORQUE;
   TESTS; JOINT
AB Background: Deficits in ankle muscle strength and ankle stiffness may be present in those subjects who underwent surgical treatment for an Achilles tendon rupture. The presence of these long-term deficits may contribute to a lower performance during daily activities and may be linked to future injuries.
   Objective: To compare the ankle passive stiffness and the plantar flexor muscle performance in patients who underwent unilateral surgical treatment of Achilles tendon rupture with nonsurgical subjects.
   Method: Twenty patients who underwent unilateral surgical treatment of Achilles tendon rupture [surgical (SU) group], and twenty nonsurgical subjects [non-surgical (NS) group] participated in this study. The ankle passive stiffness was evaluated using a clinical test. The concentric and eccentric plantar flexors performance (i.e. peak torque and work) was evaluated using an isokinetic dynamometer at 30 degrees /s.
   Results: The surgical ankle of the surgical group presented lower stiffness compared to the nonsurgical ankle (mean difference = 3.790; 95%CI = 1.23-6.35) and to the non-dominant ankle of the non-surgical group (mean difference = -3.860; 95%CI = -7.38 to -0.33). The surgical group had greater absolute asymmetry of ankle stiffness (mean difference = -2.630; 95%CI = -4.61 to -0.65) and greater absolute asymmetry of concentric (mean difference = -8.3%; 95%CI = -13.79 to -2.81) and eccentric (mean difference = -6.9%; 95%CI = -12.1 to -1.7) plantar flexor work compared to non-surgical group. There was no other difference in stiffness and plantar flexor performance.
   Conclusion: Patients who underwent surgical repair of the Achilles tendon presented with long-term (1 year or more) deficits of ankle stiffness and asymmetries of ankle stiffness and plantarflexor work in the affected ankle compared to the uninjured side in the surgical group and bothsides on the nonsurgical group. (C) 2017 Associacao Brasileira de Pesquisa e Pos-Graduacao em Fisioterapia. Published by Elsevier Editora Ltda. All rights reserved.
C1 [Borges, Pollyana R. T.; Santos, Thiago R. T.; Procpio, Paula R. S.; Chelidonopoulos, Jessica H. D.; Ocarino, Juliana M.] Univ Fed Minas Gerais, Dept Fisioterapia, EEFFTO, Belo Horizonte, MG, Brazil.
   [Zambelli, Roberto] Hosp Mater Dei, Belo Horizonte, MG, Brazil.
RP Ocarino, JM (通讯作者)，Univ Fed Minas Gerais, Escola Educ Fis Fisioterapia & Terapia Ocupac, Ave Presidente Antonio Carlos 6627, BR-31270901 Belo Horizonte, MG, Brazil.
EM julianaocarino@gmail.com
RI Santos, Thiago RT/N-7283-2015; Ocarino, Juliana M/O-2292-2013
OI Santos, Thiago RT/0000-0003-2395-2023; Ocarino, Juliana
   M/0000-0001-9404-1695
CR Araujo VL, 2011, BRAZ J PHYS THER, V15, P166, DOI 10.1590/S1413-35552011000200013
   Bressel E, 2001, AM J SPORT MED, V29, P321, DOI 10.1177/03635465010290031201
   Chleboun GS, 1997, CLIN BIOMECH, V12, P383, DOI 10.1016/S0268-0033(97)00027-2
   Christensen B, 2008, J APPL PHYSIOL, V105, P420, DOI 10.1152/japplphysiol.00201.2008
   Don R, 2007, CLIN BIOMECH, V22, P211, DOI 10.1016/j.clinbiomech.2006.10.001
   Drouin JM, 2004, EUR J APPL PHYSIOL, V91, P22, DOI 10.1007/s00421-003-0933-0
   Erickson BJ, 2015, ORTHOP J SPORTS MED, V3, DOI 10.1177/2325967115579188
   Faul F, 2007, BEHAV RES METHODS, V39, P175, DOI 10.3758/BF03193146
   Feger MA, 2015, J ATHL TRAINING, V50, P350, DOI 10.4085/1062-6050-50.2.06
   Ferris DP, 1999, J BIOMECH, V32, P787, DOI 10.1016/S0021-9290(99)00078-0
   Fleiss JL., 1999, RELIABILITY MEASUREM, DOI [10.1002/9781118032923, DOI 10.1002/9781118032923]
   Gajdosik RL, 2002, PERCEPT MOTOR SKILL, V95, P323
   Geremia JM, 2015, CLIN BIOMECH, V30, P485, DOI 10.1016/j.clinbiomech.2015.03.005
   Horstmann T, 2012, INT J SPORTS MED, V33, P474, DOI 10.1055/s-0032-1301932
   JOZSA L, 1989, AM J SPORT MED, V17, P338, DOI 10.1177/036354658901700305
   KANNUS P, 1994, INT J SPORTS MED, V15, pS11, DOI 10.1055/s-2007-1021104
   Keating JL, 1996, PHYS THER, V76, P866, DOI 10.1093/ptj/76.8.866a
   Khan RJK, 2010, COCHRANE DB SYST REV, DOI 10.1002/14651858.CD003674.pub4
   Lin CF, 2011, AM J SPORT MED, V39, P2007, DOI 10.1177/0363546511406868
   McGowan CP, 2008, J APPL PHYSIOL, V105, P486, DOI 10.1152/japplphysiol.90448.2008
   McNair PJ, 1996, BRIT J SPORT MED, V30, P243, DOI 10.1136/bjsm.30.3.243
   Menzel HJ, 2013, J STRENGTH COND RES, V27, P1370, DOI 10.1519/JSC.0b013e318265a3c8
   Moller M, 2005, KNEE SURG SPORT TR A, V13, P60, DOI 10.1007/s00167-003-0441-0
   Mullaney MJ, 2006, AM J SPORT MED, V34, P1120, DOI 10.1177/0363546505284186
   Perrin DH, 1993, ISOKINETIC EXERCISE, P212
   Soroceanu A, 2012, J BONE JOINT SURG AM, V94A, P2136, DOI 10.2106/JBJS.K.00917
   Webber SC, 2010, PHYS THER, V90, P1165, DOI 10.2522/ptj.20090394
   Wikstrom EA, 2006, SPORTS MED, V36, P393, DOI 10.2165/00007256-200636050-00003
NR 28
TC 8
Z9 8
U1 1
U2 3
PU ASSOCIACAO BRASILEIRA PESQUISA POS-GRADUACAO FISIOTERAPIA-ABRAPG-FT
PI SAO CARLOS SP
PA ROD WASHINGTON LUIS, KM 235, CAIXA POSTAL 676, SAO CARLOS SP,
   CEP13565-905, BRAZIL
SN 1413-3555
EI 1809-9246
J9 BRAZ J PHYS THER
JI Rev. Bras. Fisioter.
PD JAN-FEB
PY 2017
VL 21
IS 1
BP 51
EP 57
DI 10.1016/j.bjpt.2016.12.004
PG 7
WC Orthopedics; Rehabilitation
WE Science Citation Index Expanded (SCI-EXPANDED)
SC Orthopedics; Rehabilitation
GA EZ1RX
UT WOS:000404489600008
PM 28442075
OA Green Published
DA 2022-07-22
ER

PT J
AU Capone, G
   Svedman, S
   Juthberg, R
   Edman, G
   Ackermann, PW
AF Capone, Gianluigi
   Svedman, Simon
   Juthberg, Robin
   Edman, Gunnar
   Ackermann, Paul W.
TI Higher pyruvate levels after Achilles tendon rupture surgery could be
   used as a prognostic biomarker of an improved patient outcome
SO KNEE SURGERY SPORTS TRAUMATOLOGY ARTHROSCOPY
LA English
DT Article
DE Achilles tendon; Rupture; Biomarker; Healing; Lactate; Pyruvate;
   Patient-reported outcome measures; Pedometer; Early mobilization
ID DEEP-VEIN THROMBOSIS; BRAIN; COMPRESSION; INJURY; REPAIR; SCORE; FOOT
AB Purpose The primary aim of this study was to assess the relationship between the metabolites lactate and pyruvate in the healing tendon after Achilles tendon rupture (ATR) and patient-reported outcome at 6 and 12 months. A secondary aim was to evaluate which underlying factors regulate lactate and pyruvate concentrations. Methods Lactate and pyruvate concentrations were measured two weeks post-operatively in both the healing- and healthy Achilles tendon in 109 patients (90 men, 19 women; mean age 40 +/- 7.9 years). Patient demographics, degree of physical activity, timing of surgery, operation time, patient-reported loading and step counts were investigated in relation to metabolite concentrations. At 6 and 12 months, the Achilles tendon Total Rupture Score (ATRS) questionnaire was used to assess patient outcome. Results The mean number of steps taken during the post-operative days 1-10 was the only factor significantly related to the mean concentration of lactate (R-2 = 0.34, p = 0.038), and pyruvate (R-2 = 0.46, p = 0.006). Pyruvate was demonstrated as the only factor significantly associated with ATRS at both 6 months (R-2 = 0.32, p = 0.003) and at 12 months (R-2 = 0.37, p = 0.004) using multiple linear regression. Conclusion The mean concentration of pyruvate during early ATR healing may predict patient outcome at 6 and 12 months post-operatively and possibly be used as a biomarker of healing. Early mobilization with an increased number of steps taken is an important clinical strategy to improve the metabolite concentrations during healing.
C1 [Capone, Gianluigi; Svedman, Simon; Juthberg, Robin; Ackermann, Paul W.] Karolinska Inst, Dept Mol Med & Surg, Integrat Orthoped Lab, Stockholm, Sweden.
   [Capone, Gianluigi; Svedman, Simon; Ackermann, Paul W.] Karolinska Univ Hosp, Dept Orthoped Surg, Stockholm, Sweden.
   [Capone, Gianluigi] EOC, Osped Regionale Lugano, Orthopaed & Traumatol Unit, Lugano, Switzerland.
   [Capone, Gianluigi] Sapienza Univ Rome, Fac Pharm & Med, Rome, Italy.
   [Edman, Gunnar] Tiohundra AB, Dept Psychiat, Norrtalje, Sweden.
RP Capone, G (通讯作者)，Karolinska Inst, Dept Mol Med & Surg, Integrat Orthoped Lab, Stockholm, Sweden.; Capone, G (通讯作者)，Karolinska Univ Hosp, Dept Orthoped Surg, Stockholm, Sweden.; Capone, G (通讯作者)，EOC, Osped Regionale Lugano, Orthopaed & Traumatol Unit, Lugano, Switzerland.; Capone, G (通讯作者)，Sapienza Univ Rome, Fac Pharm & Med, Rome, Italy.
EM gianluigi.capone@eoc.ch; simon.svedman@gmail.com;
   robin.uthberg@outlook.com; gunnar.edman@gmail.com; paul.ackermann@ki.se
RI W, Ackermann P/B-9714-2008
OI W, Ackermann P/0000-0002-5520-169X; Capone,
   Gianluigi/0000-0003-0575-703X
FU Karolinska Institutet
FX Open access funding provided by Karolinska Institutet.
CR Ackermann PW, 2012, SPORTS HEALTH, V4, P193, DOI 10.1177/1941738112440957
   Addevico F, 2019, SCAND J MED SCI SPOR, V29, P1529, DOI 10.1111/sms.13469
   Aufwerber S, 2020, KNEE SURG SPORT TR A, V28, P312, DOI 10.1007/s00167-019-05767-x
   Aufwerber S, 2019, AM J SPORT MED, V47, P894, DOI 10.1177/0363546518824326
   Domeij-Arverud E, 2015, BONE JOINT J, V97B, P675, DOI 10.1302/0301-620X.97B5.34581
   Domeij-Arverud E, 2013, BONE JOINT J, V95B, P1227, DOI 10.1302/0301-620X.95B9.31162
   Fessel G, 2014, PLOS ONE, V9, DOI 10.1371/journal.pone.0110948
   Grassi A, 2020, BRIT J SPORT MED, V54, P480, DOI 10.1136/bjsports-2019-100556
   Greve K, 2012, SCAND J MED SCI SPOR, V22, pE55, DOI 10.1111/j.1600-0838.2012.01475.x
   Harvey SAK, 2010, INVEST OPHTH VIS SCI, V51, P2917, DOI 10.1167/iovs.09-4498
   Hsu AR, 2015, FOOT ANKLE INT, V36, P1279, DOI 10.1177/1071100715589632
   Kearney RS, 2012, HEALTH QUAL LIFE OUT, V10, DOI 10.1186/1477-7525-10-24
   KVIST M, 1994, SPORTS MED, V18, P173, DOI 10.2165/00007256-199418030-00004
   Magnoni S, 2003, J NEUROSURG, V98, P952, DOI 10.3171/jns.2003.98.5.0952
   Magnusson SP, 2020, CLIN ORTHOP RELAT R, V478, P1101, DOI 10.1097/CORR.0000000000001085
   Marom A, 2019, CLIN BIOMECH, V62, P96, DOI 10.1016/j.clinbiomech.2019.01.009
   Nilsson-Helander K, 2007, AM J SPORT MED, V35, P421, DOI 10.1177/0363546506294856
   Olsson N, 2011, KNEE SURG SPORT TR A, V19, P1385, DOI 10.1007/s00167-011-1511-3
   Peskind ER, 2015, JAMA NEUROL, V72, P1103, DOI 10.1001/jamaneurol.2015.1789
   Roos EM, 2001, FOOT ANKLE INT, V22, P788, DOI 10.1177/107110070102201004
   Schneider PL, 2004, MED SCI SPORT EXER, V36, P331, DOI 10.1249/01.MSS.0000113486.60548.E9
   Scott GF, 2017, EXP BIOL MED, V242, P1095, DOI 10.1177/1535370217703353
   Sharma R, 2012, CURR NEUROL NEUROSCI, V12, P560, DOI 10.1007/s11910-012-0301-8
   Stavenuiter XJR, 2019, FOOT ANKLE INT, V40, P679, DOI 10.1177/1071100719831371
   Svedman S, 2018, KNEE SURG SPORT TR A, V26, P2011, DOI 10.1007/s00167-017-4606-7
   Thermann H, 2019, CHIRURG, V90, P863, DOI 10.1007/s00104-019-01024-6
   Thermann H, 2020, CHIRURG, V91, P85, DOI 10.1007/s00104-019-01106-5
   Valkering KP, 2017, KNEE SURG SPORT TR A, V25, P1807, DOI 10.1007/s00167-016-4270-3
   Wang D, 2015, FOOT ANKLE SURG, V21, P250, DOI 10.1016/j.fas.2015.01.009
   Yalamanchi N, 2004, PLAST RECONSTR SURG, V113, P625, DOI 10.1097/01.PRS.0000101529.47062.34
   Zhang K, 2018, AM J SPORT MED, V46, P2222, DOI 10.1177/0363546518778789
   Zoremba N, 2014, ACTA VET SCAND, V56, DOI 10.1186/s13028-014-0072-0
NR 32
TC 0
Z9 0
U1 0
U2 1
PU SPRINGER
PI NEW YORK
PA ONE NEW YORK PLAZA, SUITE 4600, NEW YORK, NY, UNITED STATES
SN 0942-2056
EI 1433-7347
J9 KNEE SURG SPORT TR A
JI Knee Surg. Sports Traumatol. Arthrosc.
PD JAN
PY 2021
VL 29
IS 1
BP 300
EP 309
DI 10.1007/s00167-020-06037-x
EA MAY 2020
PG 10
WC Orthopedics; Sport Sciences; Surgery
WE Science Citation Index Expanded (SCI-EXPANDED)
SC Orthopedics; Sport Sciences; Surgery
GA QC5VL
UT WOS:000530777500001
PM 32377796
OA Green Published, hybrid
DA 2022-07-22
ER

PT J
AU Buddecke, D
AF Buddecke, Donald, Jr.
TI Acute Achilles Tendon Ruptures
SO CLINICS IN PODIATRIC MEDICINE AND SURGERY
LA English
DT Article
DE Achilles; Achilles tendon; Achilles tendon rupture
C1 [Buddecke, Donald, Jr.] Foot & Ankle Specialists, Foot & Ankle Surg, Private Practice, 18010 R Plaza, Omaha, NE 68135 USA.
RP Buddecke, D (通讯作者)，Foot & Ankle Specialists, Foot & Ankle Surg, Private Practice, 18010 R Plaza, Omaha, NE 68135 USA.
EM debuddecke@yahoo.com
CR Amlang MH, 2005, UNFALLCHIRURG, V108, P529, DOI 10.1007/s00113-005-0938-8
   Assal M, 2002, J BONE JOINT SURG AM, V84A, P161, DOI 10.2106/00004623-200202000-00001
   Backus JD, 2017, FOOT ANKLE INT, V38, P564, DOI 10.1177/1071100716687368
   Bhandari M, 2002, CLIN ORTHOP RELAT R, P190
   BRADLEY JP, 1990, AM J SPORT MED, V18, P188, DOI 10.1177/036354659001800213
   Bryant AL, 2008, J APPL PHYSIOL, V105, P1035, DOI 10.1152/japplphysiol.01281.2007
   Burgess KE, 2009, CLIN BIOMECH, V24, P504, DOI 10.1016/j.clinbiomech.2009.03.011
   Burgess KE, 2010, J STRENGTH COND RES, V24, P2088, DOI 10.1519/JSC.0b013e3181aeb12b
   Calder JDF, 2016, KNEE SURG SPORT TR A, V24, P1409, DOI 10.1007/s00167-015-3976-y
   Calleja M, 2010, SEMIN MUSCULOSKEL R, V14, P307, DOI 10.1055/s-0030-1254520
   CETTI R, 1994, CLIN ORTHOP RELAT R, P155
   CETTI R, 1993, AM J SPORT MED, V21, P791, DOI 10.1177/036354659302100606
   Clanton TO, 2015, AM J SPORT MED, V43, P1957, DOI 10.1177/0363546515587082
   Clayton RAE, 2008, INJURY, V39, P1338, DOI 10.1016/j.injury.2008.06.021
   Cottom JM, 2017, J FOOT ANKLE SURG, V56, P423, DOI 10.1053/j.jfas.2016.10.012
   Cretnik A, 2005, AM J SPORT MED, V33, P1369, DOI 10.1177/0363546504271501
   Cretnik A, 2019, J FOOT ANKLE SURG, V58, P828, DOI 10.1053/j.jfas.2018.11.029
   Cretnik Andrej, 2004, J Foot Ankle Surg, V43, P72, DOI 10.1053/j.jfas.2004.01.008
   Davis JJ, 1999, MIL MED, V164, P872, DOI 10.1093/milmed/164.12.872
   De la Fuente Carlos, 2016, Foot (Edinb), V26, P15, DOI 10.1016/j.foot.2015.10.003
   Demetracopoulos CA, 2014, FOOT ANKLE INT, V35, P612, DOI 10.1177/1071100714524550
   Deng SL, 2017, J FOOT ANKLE SURG, V56, P1236, DOI 10.1053/j.jfas.2017.05.036
   Erickson BJ, 2015, ORTHOP J SPORTS MED, V3, DOI 10.1177/2325967115579188
   Erickson BJ, 2014, ORTHOP J SPORTS MED, V2, DOI 10.1177/2325967114549948
   Ganestam A, 2016, KNEE SURG SPORT TR A, V24, P3730, DOI 10.1007/s00167-015-3544-5
   Gnandt RJ, 2016, ARTHROSCOPY, V32, P356, DOI 10.1016/j.arthro.2015.08.013
   Grassi A, 2018, J BONE JOINT SURG AM, V100, P1969, DOI 10.2106/JBJS.17.01364
   Groetelaers RPTGC, 2014, FOOT ANKLE INT, V35, P771, DOI 10.1177/1071100714536167
   Haji A, 2004, FOOT ANKLE INT, V25, P215, DOI 10.1177/107110070402500404
   Healy B, 2010, J BONE JOINT SURG BR, V92B, P646, DOI 10.1302/0301-620X.92B5.23241
   HOCKENBURY RT, 1990, FOOT ANKLE, V11, P67, DOI 10.1177/107110079001100202
   Houshian S, 1998, INJURY, V29, P651, DOI 10.1016/S0020-1383(98)00147-8
   Hsu AR, 2015, FOOT ANKLE INT, V36, P1279, DOI 10.1177/1071100715589632
   Jaakkola JI, 2000, FOOT ANKLE INT, V21, P14, DOI 10.1177/107110070002100103
   Jacob KM, 2007, ANZ J SURG, V77, P287, DOI 10.1111/j.1445-2197.2007.04035.x
   JONES BH, 1993, AM J SPORT MED, V21, P705, DOI 10.1177/036354659302100512
   Jones Matthew P, 2012, J Bone Joint Surg Am, V94, pe88, DOI 10.2106/JBJS.J.01829
   JOZSA L, 1989, AM J SPORT MED, V17, P338, DOI 10.1177/036354658901700305
   Khan RJK, 2005, J BONE JOINT SURG AM, V87A, P2202, DOI 10.2106/JBJS.D.03049
   KOBORI M, 1989, CLIN ORTHOP RELAT R, P306
   Labib SA, 2009, FOOT ANKLE INT, V30, P410, DOI 10.3113/FAI.2009.0410
   Lansdaal JR, 2007, INJURY, V38, P839, DOI 10.1016/j.injury.2006.12.010
   Lantto I, 2016, AM J SPORT MED, V44, P2406, DOI 10.1177/0363546516651060
   Leppilahti J, 1996, ACTA ORTHOP SCAND, V67, P277, DOI 10.3109/17453679608994688
   Levi N, 1997, INJURY, V28, P311, DOI 10.1016/S0020-1383(96)00200-8
   Lim J, 2001, FOOT ANKLE INT, V22, P559, DOI 10.1177/107110070102200705
   MA GWC, 1977, CLIN ORTHOP RELAT R, P247
   Maffulli N, 2003, AM J SPORT MED, V31, P692, DOI 10.1177/03635465030310051001
   Majewski M, 2006, AM J SPORT MED, V34, P793, DOI 10.1177/0363546505283266
   Manent A, 2019, J FOOT ANKLE SURG, V58, P1229, DOI 10.1053/j.jfas.2019.02.002
   Manent A, 2017, J FOOT ANKLE SURG, V56, P954, DOI 10.1053/j.jfas.2017.06.018
   McCoy BW, 2010, FOOT ANKLE INT, V31, P701, DOI 10.3113/FAI.2010.0701
   McMahon Samuel E, 2011, Foot Ankle Surg, V17, P211, DOI 10.1016/j.fas.2010.11.001
   Metz R, 2008, AM J SPORT MED, V36, P1688, DOI 10.1177/0363546508319312
   Moller A, 1996, ACTA ORTHOP SCAND, V67, P479, DOI 10.3109/17453679608996672
   Moller M, 2001, J BONE JOINT SURG BR, V83B, P843
   Netto CD, 2018, J FOOT ANKLE SURG, V57, P1272, DOI 10.1053/j.jfas.2018.04.002
   NILLIUS SA, 1976, ACTA ORTHOP SCAND, V47, P118, DOI 10.3109/17453677608998983
   Nilsson-Helander K, 2010, AM J SPORT MED, V38, P2186, DOI 10.1177/0363546510376052
   NISTOR L, 1981, J BONE JOINT SURG AM, V63, P394, DOI 10.2106/00004623-198163030-00012
   Nyyssonen T, 2008, SCAND J SURG, V97, P272, DOI 10.1177/145749690809700312
   Ollivere BJ, 2014, FOOT ANKLE SURG, V20, pE27, DOI 10.1016/j.fas.2014.01.006
   Olsson N, 2013, AM J SPORT MED, V41, P2867, DOI 10.1177/0363546513503282
   Osei DA, 2014, J HAND SURG-AM, V39, P262, DOI 10.1016/j.jhsa.2013.11.001
   Patel A, 2012, CLIN ORTHOP RELAT R, V470, P270, DOI 10.1007/s11999-011-2166-6
   Raikin SM, 2013, FOOT ANKLE INT, V34, P475, DOI 10.1177/1071100713477621
   Rozis M, 2018, FOOT ANKLE INT, V39, P689, DOI 10.1177/1071100718757971
   SALEH M, 1992, J BONE JOINT SURG BR, V74, P206, DOI 10.1302/0301-620X.74B2.1544953
   Schepull T, 2011, AM J SPORT MED, V39, P38, DOI 10.1177/0363546510383515
   Soldatis JJ, 1997, AM J SPORT MED, V25, P90, DOI 10.1177/036354659702500118
   Soroceanu A, 2012, J BONE JOINT SURG AM, V94A, P2136, DOI 10.2106/JBJS.K.00917
   Suchak AA, 2005, FOOT ANKLE INT, V26, P932, DOI 10.1177/107110070502601106
   Twaddle BC, 2007, AM J SPORT MED, V35, P2033, DOI 10.1177/0363546507307503
   Vosseller JT, 2013, FOOT ANKLE INT, V34, P49, DOI 10.1177/1071100712460223
   WATSON TW, 1995, FOOT ANKLE INT, V16, P191, DOI 10.1177/107110079501600404
   Wentorf FA, 2006, AM J SPORT MED, V34, P1948, DOI 10.1177/0363546506290060
   Wilkins R, 2012, AM J SPORT MED, V40, P2154, DOI 10.1177/0363546512453293
   Willits K, 2010, J BONE JOINT SURG AM, V92A, P2767, DOI 10.2106/JBJS.I.01401
   Wojtys EM, 2002, AM J SPORT MED, V30, P182, DOI 10.1177/03635465020300020601
   Wojtys EM, 1998, AM J SPORT MED, V26, P614, DOI 10.1177/03635465980260050301
   Yang B, 2017, INT J SURG, V40, P178, DOI 10.1016/j.ijsu.2017.03.021
   Zhou K, 2018, J FOOT ANKLE SURG, V57, P1191, DOI 10.1053/j.jfas.2018.05.007
NR 82
TC 0
Z9 0
U1 0
U2 2
PU W B SAUNDERS CO-ELSEVIER INC
PI PHILADELPHIA
PA 1600 JOHN F KENNEDY BOULEVARD, STE 1800, PHILADELPHIA, PA 19103-2899 USA
SN 0891-8422
EI 1558-2302
J9 CLIN PODIATR MED SUR
JI Clin. Podiatr. Med. Surg.
PD APR
PY 2021
VL 38
IS 2
SI SI
BP 201
EP 226
DI 10.1016/j.cpm.2020.12.006
EA MAR 2021
PG 26
WC Orthopedics
WE Science Citation Index Expanded (SCI-EXPANDED)
SC Orthopedics
GA RC3IQ
UT WOS:000632697700007
PM 33745652
DA 2022-07-22
ER

PT J
AU Sun, D
   Fekete, G
   Baker, JS
   Mei, QC
   Istvan, B
   Zhang, Y
   Gu, YD
AF Sun, Dong
   Fekete, Gusztav
   Baker, Julien S.
   Mei, Qichang
   Istvan, Biro
   Zhang, Yan
   Gu, Yaodong
TI A Pilot Study of Musculoskeletal Abnormalities in Patients in Recovery
   from a Unilateral Rupture-Repaired Achilles Tendon
SO INTERNATIONAL JOURNAL OF ENVIRONMENTAL RESEARCH AND PUBLIC HEALTH
LA English
DT Article
DE Achilles tendon rupture; musculoskeletal modeling; knee; ankle; gait
ID MUSCLE-TENDON; BIOMECHANICAL PROPERTIES; PEDIATRIC OBESITY; SURGICAL
   REPAIR; ANKLE KINETICS; STRENGTH; LENGTH; OUTCOMES; PATTERN; ATROPHY
AB The purpose of this study was to compare the inter-limb joint kinematics, joint moments, muscle forces, and joint reaction forces in patients after an Achilles tendon rupture (ATR) via subject-specific musculoskeletal modeling. Six patients recovering from a surgically repaired unilateral ATR were included in this study. The bilateral Achilles tendon (AT) lengths were evaluated using ultrasound imaging. The three-dimensional marker trajectories, ground reaction forces, and surface electromyography (sEMG) were collected on both sides during self-selected speed during walking, jogging and running. Subject-specific musculoskeletal models were developed to compute joint kinematics, joint moments, muscle forces and joint reaction forces. AT lengths were significantly longer in the involved side. The side-to-side triceps surae muscle strength deficits were combined with decreased plantarflexion angles and moments in the injured leg during walking, jogging and running. However, the increased knee extensor femur muscle forces were associated with greater knee extension degrees and moments in the involved limb during all tasks. Greater knee joint moments and joint reaction forces versus decreased ankle joint moments and joint reaction forces in the involved side indicate elevated knee joint loads compared with reduced ankle joint loads that are present during normal activities after an ATR. In the frontal plane, increased subtalar eversion angles and eversion moments in the involved side were demonstrated only during jogging and running, which were regarded as an indicator for greater medial knee joint loading. It seems after an ATR, the elongated AT accompanied by decreased plantarflexion degrees and calf muscle strength deficits indicates ankle joint function impairment in the injured leg. In addition, increased knee extensor muscle strength and knee joint loads may be a possible compensatory mechanism for decreased ankle function. These data suggest patients after an ATR may suffer from increased knee overuse injury risk.
C1 [Sun, Dong; Mei, Qichang; Zhang, Yan; Gu, Yaodong] Ningbo Univ, Fac Sports Sci, Ningbo 315211, Peoples R China.
   [Fekete, Gusztav] Eotvos Lorand Univ, Savaria Inst Technol, H-9700 Szombathely, Hungary.
   [Baker, Julien S.] Hong Kong Baptist Univ, Dept Sport & Phys Educ, Hong Kong 999077, Peoples R China.
   [Istvan, Biro] Univ Szeged, Dept Technol, Fac Engn, H-6727 Szeged, Hungary.
RP Gu, YD (通讯作者)，Ningbo Univ, Fac Sports Sci, Ningbo 315211, Peoples R China.
EM nbsundong@gmail.com; fg@inf.elte.hu; jsbaker@hkbu.edu.hk;
   meiqichang@aliyun.com; biro-i@mk.u-szeged.hu; champagne0906@hotmail.com;
   guyaodong@nbu.edu.cn
RI Fekete, Gusztáv/ABG-2914-2020
OI Fekete, Gusztáv/0000-0002-6138-8382; Mei, Qichang/0000-0003-1113-877X;
   gu, yaodong/0000-0003-2187-9440; Baker, Julien/0000-0002-9093-7897
FU Key Project of the National Social Science Foundation of China
   [19ZDA352]; National Natural Science Foundation of China [81772423];
   NSFC-RSE Joint Project [81911530253]; National Key R&D Program of China
   [2018YFF0300905]; K. C. Wong Magna Fund in Ningbo University
FX This study was funded by the by Key Project of the National Social
   Science Foundation of China (19ZDA352), National Natural Science
   Foundation of China (No. 81772423), NSFC-RSE Joint Project
   (81911530253), National Key R&D Program of China (2018YFF0300905), and
   K. C. Wong Magna Fund in Ningbo University.
CR Agres AN, 2015, SCAND J MED SCI SPOR, V25, P860, DOI 10.1111/sms.12456
   Almonroeder T, 2013, ANN BIOMED ENG, V41, P1758, DOI 10.1007/s10439-013-0819-1
   Barfod KW, 2015, KNEE SURG SPORT TR A, V23, P3398, DOI 10.1007/s00167-014-3175-2
   Barfod KW, 2015, J FOOT ANKLE SURG, V54, P220, DOI 10.1053/j.jfas.2014.11.018
   Baxter JR, 2018, J BIOMECH, V79, P238, DOI 10.1016/j.jbiomech.2018.08.016
   Bennett JE, 2001, J ORTHOP SPORT PHYS, V31, P504, DOI 10.2519/jospt.2001.31.9.504
   Chan APH, 2011, BMC SPORTS SCI MED R, V3, DOI 10.1186/1758-2555-3-32
   Chen C., 2019, PHYS ACTIVITY HLTH, V3, P63, DOI [10.5334/paah.37, DOI 10.5334/PAAH.37]
   Cooper A, 2012, PHYSIOTHER RES INT, V17, DOI 10.1002/pri.528
   Costa ML, 2006, FOOT ANKLE INT, V27, P414, DOI 10.1177/107110070602700605
   Delp SL, 2007, IEEE T BIO-MED ENG, V54, P1940, DOI 10.1109/TBME.2007.901024
   Don R, 2007, CLIN BIOMECH, V22, P211, DOI 10.1016/j.clinbiomech.2006.10.001
   Draper CE, 2012, J ORTHOP RES, V30, P209, DOI 10.1002/jor.21523
   Farrokhi S, 2011, OSTEOARTHR CARTILAGE, V19, P287, DOI 10.1016/j.joca.2010.12.001
   Fukuchi RK, 2017, PEERJ, V5, DOI 10.7717/peerj.3298
   Ganestam A, 2016, KNEE SURG SPORT TR A, V24, P3730, DOI 10.1007/s00167-015-3544-5
   Geremia JM, 2015, CLIN BIOMECH, V30, P485, DOI 10.1016/j.clinbiomech.2015.03.005
   Goren D, 2005, FOOT ANKLE INT, V26, P286, DOI 10.1177/107110070502600404
   Harris MD, 2017, J BIOMECH, V54, P80, DOI 10.1016/j.jbiomech.2017.01.040
   Hebenstreit F, 2015, HUM MOVEMENT SCI, V43, P118, DOI 10.1016/j.humov.2015.07.009
   Heikkinen J, 2017, J BONE JOINT SURG AM, V99, P1509, DOI 10.2106/JBJS.16.01491
   Heikkinen J, 2017, AM J SPORT MED, V45, P1395, DOI 10.1177/0363546517694610
   Jandacka D, 2018, ORTHOP J SPORTS MED, V6, DOI 10.1177/2325967118779862
   Jandacka Daniel, 2013, BMJ Case Rep, V2013, DOI 10.1136/bcr-2012-007370
   Kunimasa Y, 2014, SCAND J MED SCI SPOR, V24, pE269, DOI 10.1111/sms.12161
   Lantto I, 2015, SCAND J MED SCI SPOR, V25, pE133, DOI 10.1111/sms.12253
   Lerner ZF, 2016, J BIOMECH, V49, P1547, DOI 10.1016/j.jbiomech.2016.03.033
   Lerner ZF, 2016, J ORTHOP RES, V34, P97, DOI 10.1002/jor.23028
   Lerner ZF, 2015, J BIOMECH, V48, P644, DOI 10.1016/j.jbiomech.2014.12.049
   Lohman EB, 2011, PHYS THER SPORT, V12, P151, DOI 10.1016/j.ptsp.2011.09.004
   Mei QC, 2019, FRONT PHYSIOL, V10, DOI 10.3389/fphys.2019.00573
   Modenese L, 2018, J BIOMECH, V73, P108, DOI 10.1016/j.jbiomech.2018.03.039
   Moissenet F, 2019, FRONT NEUROROBOTICS, V13, DOI 10.3389/fnbot.2019.00048
   Mullaney MJ, 2006, AM J SPORT MED, V34, P1120, DOI 10.1177/0363546505284186
   Nilsson-Helander K, 2007, AM J SPORT MED, V35, P421, DOI 10.1177/0363546506294856
   Nilsson-Helander K, 2010, AM J SPORT MED, V38, P2186, DOI 10.1177/0363546510376052
   Olsson N, 2011, KNEE SURG SPORT TR A, V19, P1385, DOI 10.1007/s00167-011-1511-3
   Pataky TC, 2013, J BIOMECH, V46, P2394, DOI 10.1016/j.jbiomech.2013.07.031
   Peng WC, 2019, FOOT ANKLE INT, V40, P568, DOI 10.1177/1071100718822537
   Powell HC, 2018, J ORTHOP SPORT PHYS, V48, P34, DOI 10.2519/jospt.2018.7684
   Rosso C, 2015, KNEE SURG SPORT TR A, V23, P890, DOI 10.1007/s00167-013-2726-2
   Rosso C, 2013, KNEE SURG SPORT TR A, V21, P1369, DOI 10.1007/s00167-013-2407-1
   Seth A, 2018, PLOS COMPUT BIOL, V14, DOI 10.1371/journal.pcbi.1006223
   Silbernagel KG, 2012, J ORTHOP SPORT PHYS, V42, P521, DOI 10.2519/jospt.2012.3913
   Smale KB, 2019, J BIOMECH, V83, P9, DOI 10.1016/j.jbiomech.2018.11.016
   Speedtsberg MB, 2019, ORTHOP J SPORTS MED, V7, DOI 10.1177/2325967119854324
   Steele KM, 2012, GAIT POSTURE, V35, P556, DOI 10.1016/j.gaitpost.2011.11.023
   Strickland, 1995, J Am Acad Orthop Surg, V3, P55
   Sun D, 2019, J MED IMAG HEALTH IN, V9, P552, DOI 10.1166/jmihi.2019.2630
   Suydam SM, 2015, KNEE SURG SPORT TR A, V23, P868, DOI 10.1007/s00167-013-2512-1
   Wang HK, 2013, ARCH PHYS MED REHAB, V94, P1590, DOI 10.1016/j.apmr.2013.01.015
   Willy RW, 2017, AM J SPORT MED, V45, P1124, DOI 10.1177/0363546516685055
   Ye J, 2018, PHYS ACTIVITY HLTH, V2, P1, DOI [10.5334/paah.4, DOI 10.5334/paah.4, DOI 10.5334/PAAH.4]
   Zhou H., 2019, PHYS ACT HLTH, V3, P127, DOI [10.5334/paah.45, DOI 10.5334/PAAH.45]
NR 54
TC 5
Z9 5
U1 2
U2 8
PU MDPI
PI BASEL
PA ST ALBAN-ANLAGE 66, CH-4052 BASEL, SWITZERLAND
EI 1660-4601
J9 INT J ENV RES PUB HE
JI Int. J. Environ. Res. Public Health
PD JUL
PY 2020
VL 17
IS 13
AR 4642
DI 10.3390/ijerph17134642
PG 20
WC Environmental Sciences; Public, Environmental & Occupational Health
WE Science Citation Index Expanded (SCI-EXPANDED); Social Science Citation Index (SSCI)
SC Environmental Sciences & Ecology; Public, Environmental & Occupational
   Health
GA MM7EO
UT WOS:000550316700001
PM 32605170
OA Green Published
DA 2022-07-22
ER

PT J
AU Svedman, S
   Westin, O
   Aufwerber, S
   Edman, G
   Nilsson-Helander, K
   Carmont, MR
   Karlsson, J
   Ackermann, PW
AF Svedman, Simon
   Westin, Olof
   Aufwerber, Susanna
   Edman, Gunnar
   Nilsson-Helander, Katarina
   Carmont, Michael R.
   Karlsson, Jon
   Ackermann, Paul W.
TI Longer duration of operative time enhances healing metabolites and
   improves patient outcome after Achilles tendon rupture surgery
SO KNEE SURGERY SPORTS TRAUMATOLOGY ARTHROSCOPY
LA English
DT Article
DE Achilles tendon; Rupture; Operative time; Patient-reported outcomes;
   Physical activity; Pain; Post-operative complications; Microdialysis;
   Glutamate; Glycerol
ID INTERMITTENT PNEUMATIC COMPRESSION; LOWER-LIMB IMMOBILIZATION; DEEP-VEIN
   THROMBOSIS; RANDOMIZED-TRIAL; FUNCTIONAL REHABILITATION; NONOPERATIVE
   TREATMENT; GLUTAMATE RECEPTORS; MICROARRAY ANALYSIS; BARIATRIC SURGERY;
   REPAIR
AB The relationship between the duration of operative time (DOT), healing response and patient outcome has not been previously investigated. An enhanced healing response related to DOT may potentiate repair processes, especially in hypovascular and sparsely metabolized musculoskeletal tissues such as tendons. This study aimed to investigate the association between DOT and the metabolic healing response, patient-reported outcome and the rate of post-operative complications after acute Achilles tendon injury.
   Observational cohort, cross-sectional study with observers blinded to patient grouping. A total of two-hundred and fifty-six prospectively randomized patients (210 men, 46 women; mean age 41 years) with an acute total Achilles tendon rupture all operated on with uniform anaesthetic and surgical technique were retrospectively assessed. At 2 weeks post-operatively, six metabolites were quantified using microdialysis. At 3, 6 and 12 months, patient-reported pain, walking ability and physical activity were examined using self-reported questionnaires, Achilles tendon total rupture score, foot and ankle outcome score and physical activity scale. At 12 months, functional outcome was assessed using the heel-rise test. Complications, such as deep venous thrombosis, infections and re-operations, were recorded throughout the study.
   Patients who underwent longer DOT exhibited higher levels of glutamate (p = 0.026) and glycerol (p = 0.023) at 2 weeks. At the 1-year follow-up, longer DOT was associated with significantly less loss in physical activity (p = 0.003), less pain (p = 0.009), less walking limitations (p = 0.022) and better functional outcome (p = 0.014). DOT did not significantly correlate with the rate of adverse events, such as deep venous thrombosis, infections or re-ruptures. Higher glutamate levels were associated with less loss in physical activity (p = 0.017). All correlations were confirmed by multiple linear regressions taking confounding factors into consideration.
   The results from this study suggest a previously unknown mechanism, increased metabolic response associated with longer DOT, which may improve patient outcome after Achilles tendon rupture surgery. Allowing for a higher amount of traumatized tissue, as reflected by up-regulation of glycerol in patients with longer DOT, may prove to be an important surgical tip for stimulation of repair of hypometabolic soft tissue injuries, such as Achilles tendon ruptures.
   II.
C1 [Svedman, Simon; Aufwerber, Susanna; Ackermann, Paul W.] Karolinska Inst, Dept Mol Med & Surg, Integrat Orthoped Lab, Stockholm, Sweden.
   [Westin, Olof; Nilsson-Helander, Katarina; Carmont, Michael R.; Karlsson, Jon] Univ Gothenburg, Sahlgrenska Acad, Inst Clin Sci, Dept Orhopaed, Gothenburg, Sweden.
   [Westin, Olof; Karlsson, Jon] Sahlgrens Univ Hosp, Molndal, Sweden.
   [Edman, Gunnar] Tiohundra AB, Dept Psychiat, Norrtalje, Sweden.
   [Nilsson-Helander, Katarina] Hallands Sjukhus, Kungsbacka, Sweden.
   [Carmont, Michael R.] Princess Royal Hosp, Dept Orthopaed Surg, Telford, Shrops, England.
   [Ackermann, Paul W.] Karolinska Univ Hosp, Dept Orthoped Surg, Stockholm, Sweden.
RP Ackermann, PW (通讯作者)，Karolinska Inst, Dept Mol Med & Surg, Integrat Orthoped Lab, Stockholm, Sweden.; Ackermann, PW (通讯作者)，Karolinska Univ Hosp, Dept Orthoped Surg, Stockholm, Sweden.
EM simon.svedman@gmail.com; olof.westin@gmail.com;
   susanna.aufwerber@karolinska.se; gunnar.edman@gmail.com;
   ina.nilsson@telia.com; mcarmont@hotmail.com; jon.karlsson@telia.com;
   paul.ackermann@karolinska.se
RI Helander, Katarina Nilsson/AAE-1793-2020; W, Ackermann P/B-9714-2008
OI Helander, Katarina Nilsson/0000-0002-1292-5102; W, Ackermann
   P/0000-0002-5520-169X; Aufwerber, Susanna/0000-0001-8795-105X; Svedman,
   Simon/0000-0002-8872-7209
FU Swedish Research Council [2012-3510]; Stockholm County Council
   [SLL20130150]; Karolinska Institutet [SLL20130150]; American Orthopaedic
   Society for Sports Medicine's (AOSSM); Swedish National Centre for
   Sports Research [2013-0039]
FX This study was supported by the Swedish Research Council (Project No.
   2012-3510), the regional agreement on medical training and clinical
   research (ALF) between Stockholm County Council and Karolinska
   Institutet (Project No. SLL20130150), the American Orthopaedic Society
   for Sports Medicine's (AOSSM) and the Swedish National Centre for Sports
   Research (Project No. 2013-0039).
CR Acklin YP, 2011, INJURY, V42, P209, DOI 10.1016/j.injury.2010.09.039
   Alim MA, 2016, BMJ OPEN SPORT EXERC, V2, P1
   Chan MM, 2013, SURG OBES RELAT DIS, V9, P88, DOI 10.1016/j.soard.2011.09.019
   Chang Huan J, 2010, JAMA, V303, P188, DOI 10.1001/jama.303.2.188
   Chu D, 2008, AM J SURG, V196, P652, DOI 10.1016/j.amjsurg.2008.07.003
   Clericuzio M, 2014, J NAT MED-TOKYO, V68, P31, DOI 10.1007/s11418-013-0759-y
   Cordero-Ampuero J, 2010, CLIN ORTHOP RELAT R, V468, P3268, DOI 10.1007/s11999-010-1411-8
   de Vos RJ, 2010, JAMA-J AM MED ASSOC, V303, P144, DOI 10.1001/jama.2009.1986
   Domeij-Arverud E, 2015, BONE JOINT J, V97B, P675, DOI 10.1302/0301-620X.97B5.34581
   Domeij-Arverud E, 2013, BONE JOINT J, V95B, P1227, DOI 10.1302/0301-620X.95B9.31162
   Dunn AL, 1999, JAMA-J AM MED ASSOC, V281, P327, DOI 10.1001/jama.281.4.327
   Frankewycz B, 2017, ARCH ORTHOP TRAUM SU, V137, P333, DOI 10.1007/s00402-017-2627-9
   Frykholm P, 2001, J NEUROL NEUROSUR PS, V71, P455, DOI 10.1136/jnnp.71.4.455
   Greve K, 2012, SCAND J MED SCI SPOR, V22, pE55, DOI 10.1111/j.1600-0838.2012.01475.x
   Gupta R, 2013, AMINO ACIDS, V44, P757, DOI 10.1007/s00726-012-1400-1
   Horstmann T, 2012, INT J SPORTS MED, V33, P474, DOI 10.1055/s-0032-1301932
   KANNUS P, 1991, J BONE JOINT SURG AM, V73A, P1507, DOI 10.2106/00004623-199173100-00009
   Keating JF, 2011, J BONE JOINT SURG BR, V93B, P1071, DOI 10.1302/0301-620X.93B8.25998
   LANDIS JR, 1977, BIOMETRICS, V33, P159, DOI 10.2307/2529310
   Lorenzo Cedric S F, 2005, HPB (Oxford), V7, P226, DOI 10.1080/13651820510028864
   Marklund N, 1997, NEUROREPORT, V8, P1457, DOI 10.1097/00001756-199704140-00026
   Moller M, 2005, KNEE SURG SPORT TR A, V13, P60, DOI 10.1007/s00167-003-0441-0
   Molloy TJ, 2006, J APPL PHYSIOL, V101, P1702, DOI 10.1152/japplphysiol.00386.2006
   Molloy TJ, 2006, J ORTHOP RES, V24, P842, DOI 10.1002/jor.20093
   Mullaney MJ, 2006, AM J SPORT MED, V34, P1120, DOI 10.1177/0363546505284186
   Nilsson-Helander K, 2007, AM J SPORT MED, V35, P421, DOI 10.1177/0363546506294856
   Nilsson-Helander K, 2010, AM J SPORT MED, V38, P2186, DOI 10.1177/0363546510376052
   Olsson N, 2011, KNEE SURG SPORT TR A, V19, P1385, DOI 10.1007/s00167-011-1511-3
   Reames BN, 2015, SURG OBES RELAT DIS, V11, P207, DOI 10.1016/j.soard.2014.03.018
   Roos EM, 2001, FOOT ANKLE INT, V22, P788, DOI 10.1177/107110070102201004
   Schizas N, 2012, J ORTHOP RES, V30, P1447, DOI 10.1002/jor.22094
   Silbernagel KG, 2010, KNEE SURG SPORT TR A, V18, P258, DOI 10.1007/s00167-009-0889-7
   Tan TW, 2012, J AM COLL SURGEONS, V215, P512, DOI 10.1016/j.jamcollsurg.2012.06.007
   Valkering KP, 2017, KNEE SURG SPORT TR A, V25, P1807, DOI 10.1007/s00167-016-4270-3
   van der Linden W, 2011, ARCH SURG-CHICAGO, V146, P1198, DOI 10.1001/archsurg.2011.268
   Willits K, 2010, J BONE JOINT SURG AM, V92A, P2767, DOI 10.2106/JBJS.I.01401
NR 36
TC 12
Z9 12
U1 0
U2 4
PU SPRINGER
PI NEW YORK
PA 233 SPRING ST, NEW YORK, NY 10013 USA
SN 0942-2056
EI 1433-7347
J9 KNEE SURG SPORT TR A
JI Knee Surg. Sports Traumatol. Arthrosc.
PD JUL
PY 2018
VL 26
IS 7
SI SI
BP 2011
EP 2020
DI 10.1007/s00167-017-4606-7
PG 10
WC Orthopedics; Sport Sciences; Surgery
WE Science Citation Index Expanded (SCI-EXPANDED)
SC Orthopedics; Sport Sciences; Surgery
GA GL5XY
UT WOS:000437249800016
PM 28638971
OA hybrid, Green Published
DA 2022-07-22
ER

PT J
AU Genc, E
   Beytemur, O
   Yuksel, S
   Eren, Y
   Caglar, A
   Kucukyildirim, BO
   Gulec, MA
AF Genc, Erdinc
   Beytemur, Ozan
   Yuksel, Serdar
   Eren, Yilmaz
   Caglar, Aysel
   Kucukyildirim, Bedri Onur
   Gulec, Mehmet Akif
TI Investigation of the biomechanical and histopathological effects of
   autologous conditioned serum on healing of Achilles tendon
SO ACTA ORTHOPAEDICA ET TRAUMATOLOGICA TURCICA
LA English
DT Article
DE Autologous conditioned serum; Achilles tendon rupture; Rats; Animal
   model; Growth factors
ID GROWTH-FACTOR; SURGICAL REPAIR; RUPTURE
AB Objective: The aim of this study to evaluate the effects of autologous conditioned serum (ACS) on the healing of transected rat Achilles tendons via the assessment of biomechanical and histological parameters.
   Methods: The study was conducted on 45 male Sprague-Dawley rats. Five rats were used as donors for ACS preparation. Animals were randomly assigned to the experimental or control group. In both groups, the Achilles tendon was cut transversally and then sutured. In the placebo control and ACS-treated groups, saline or ACS, respectively, was injected into the repair zone three times after surgery. Ten rats from each group (ACS group, n = 20; control group, n = 20) were euthanized at days 15 and 30 after surgery for histopathological (n = 5) and biomechanical (n = 5) testing. The histopathological findings were interpreted using the Bonar and Movin scales. Tendon remodelling was evaluated via the immunohistochemical staining of collagen type 3. Biomechanical effects were assessed by tensile testing.
   Results: The Bonar and Movin scale scores were significantly better in the ACS-treated group on both day 15 (p = 0.003 and p = 0.003, respectively) and day 30 (p = 0.005 and p = 0.004, respectively). The immunohistochemical density of collagen type 3 was significantly lower in the ACS-treated group on day 30 (p = 0.018). The type 1/3 collagen ratios of the groups were similar on days 15 and 30, as determined by Sirius Red staining (p = 0.910 and p = 0.133, respectively). In the biomechanical assessment results, the ACS-treated group's maximum load to failure values were significantly higher on day 15 (p = 0.049).
   Conclusion: Injection of ACS had a positive effect on the histopathological healing of rat Achilles tendons on days 15 and 30 and on biomechanical healing on day 15. ACS treatment contributed to lowering the collagen type 3 density by day 30. According to our study, ACS may be favourable for the treatment of human Achilles tendon injuries and tendinopathies. (C) 2018 Turkish Association of Orthopaedics and Traumatology. Publishing services by Elsevier B.V.
C1 [Genc, Erdinc] Mengucek Gazi Training & Res Hosp, Dept Orthoped & Traumatol, Erzincan, Turkey.
   [Beytemur, Ozan; Yuksel, Serdar; Gulec, Mehmet Akif] Bagcilar Training & Res Hosp, Dept Orthoped & Traumatol, Istanbul, Turkey.
   [Eren, Yilmaz] Sisli Etfal Training & Res Hosp, Dept Orthoped & Traumatol, Istanbul, Turkey.
   [Caglar, Aysel] Bagcilar Training & Res Hosp, Dept Pathol, Istanbul, Turkey.
   [Kucukyildirim, Bedri Onur] Yildiz Tech Univ, Dept Mech Engn, Istanbul, Turkey.
RP Genc, E (通讯作者)，Basbaglar Mh Mengucek Gazi Egitim & Arastirma Has, Giris Kat Ortopedi Poliklin, TR-24000 Erzincan, Turkey.
EM erdincgenc@hotmail.com; beytemur@yahoo.com; Serdar84yuksel@gmail.com;
   Yilmazeren55@gmail.com; ayselkaracaglar@gmail.com;
   kucukyil@yildiz.edu.tr; akifgulec@yahoo.com
RI Kucukyildirim, Bedri Onur/AAZ-6726-2020; beytemür, ozan/AAU-7362-2020;
   Güleç, Akif/AAU-4848-2020
OI Genc, Erdinc/0000-0002-1260-6443
CR Aspenberg P, 2004, ACTA ORTHOP SCAND, V75, P93, DOI 10.1080/00016470410001708190
   Baltzer AWA, 2013, ORTHOP REV, V5, P59, DOI 10.4081/or.2013.e13
   Berkoff DJ, 2016, J ORTHOP RES, V34, P616, DOI 10.1002/jor.23057
   Daghino W, 2016, INJURY, V47, P2591, DOI 10.1016/j.injury.2016.09.009
   Darabos N, 2014, KNEE SURG SPORT TR A, V24, P1
   Drissi H, 1999, EXP CELL RES, V251, P275, DOI 10.1006/excr.1999.4539
   Evans CH, 2016, PHYS MED REH CLIN N, V27, P893, DOI 10.1016/j.pmr.2016.06.003
   Forslund C, 2003, J ORTHOPAED RES, V21, P617, DOI 10.1016/S0736-0266(03)00010-X
   Geburek F, 2015, STEM CELL RES THER, V6, DOI 10.1186/s13287-015-0115-0
   Heisterbach PE, 2012, KNEE SURG SPORT TR A, V20, P1903, DOI 10.1007/s00167-011-1772-x
   INGLIS AE, 1981, CLIN ORTHOP RELAT R, P160
   Longo UG, 2011, BRIT MED BULL, V98, P31, DOI 10.1093/bmb/ldq030
   Lopez-Najera D, 2015, KNEE SURG SPORT TR A, V24, P1
   Maffulli N, 1999, J BONE JOINT SURG AM, V81A, P1019, DOI 10.2106/00004623-199907000-00017
   Majewski M, 2009, AM J SPORT MED, V37, P2117, DOI 10.1177/0363546509348047
   Maquirriain Javier, 2011, Yale Journal of Biology and Medicine, V84, P289
   Rutgers M, 2010, ARTHRITIS RES THER, V12, P1
   Thomopoulos S, 2010, ANN BIOMED ENG, V38, P225, DOI 10.1007/s10439-009-9844-5
   Wang XT, 2004, J HAND SURG-AM, V29A, P884, DOI 10.1016/j.jhsa.2004.05.016
   Wright-Carpenter T, 2004, INT J SPORTS MED, V25, P588, DOI 10.1055/s-2004-821304
   Yang KGA, 2008, OSTEOARTHR CARTILAGE, V16, P498, DOI 10.1016/j.joca.2007.07.008
   Yuksel S, 2014, ACTA ORTHOP TRAUMATO, V49, P544
   Zhang F, 2003, PLAST RECONSTR SURG, V112, P1613, DOI 10.1097/01.PRS.0000086772.72535.A4
NR 23
TC 10
Z9 10
U1 0
U2 0
PU TURKISH ASSOC ORTHOPAEDICS TRAUMATOLOGY
PI ISTANBUL
PA SEHREMINI MAH KOYUNCU SK CIGDEM APT NO 4 D 5 FATIH, ISTANBUL, 00000,
   TURKEY
SN 1017-995X
J9 ACTA ORTHOP TRAUMATO
JI Acta Orthop. Traumatol. Turc.
PY 2018
VL 52
IS 3
BP 226
EP 231
DI 10.1016/j.aott.2018.01.005
PG 6
WC Orthopedics
WE Science Citation Index Expanded (SCI-EXPANDED)
SC Orthopedics
GA GL7ZQ
UT WOS:000437429400011
PM 29454565
OA Green Published, gold
DA 2022-07-22
ER

PT J
AU Yang, X
   Meng, HY
   Peng, J
   Xu, LJ
   Wang, Y
   Sun, X
   Zhao, YX
   Quan, Q
   Yu, W
   Chen, MX
   Shi, T
   Du, YA
   Lu, SB
   Wang, AY
AF Yang, Xuan
   Meng, Haoye
   Peng, Jiang
   Xu, Lijuan
   Wang, Yu
   Sun, Xun
   Zhao, Yanxu
   Quan, Qi
   Yu, Wen
   Chen, Mingxue
   Shi, Tong
   Du, Yanan
   Lu, Shibi
   Wang, Aiyuan
TI Construction of Microunits by Adipose-Derived Mesenchymal Stem Cells
   Laden with Porous Microcryogels for Repairing an Acute Achilles Tendon
   Rupture in a Rat Model
SO INTERNATIONAL JOURNAL OF NANOMEDICINE
LA English
DT Article
DE Achilles tendon rupture; tendon tissue engineering; adipose-derived
   mesenchymal stem cells; ASCs; injectable biomaterials
ID RISK-FACTORS; INJURY; REHABILITATION; PROLIFERATION; MICRONICHES;
   THERAPY; SURGERY
AB Objective: Tissue engineering approaches seem to be an attractive therapy for tendon rupture. Novel injectable porous gelatin microcryogels (GMs) can promote cell attachment and proliferation, thus facilitating the repair potential for target tissue regeneration. The research objectives of this study were to assess the efficacy of tissue-like microunits con-structed by multiple GMs laden with adipose-derived mesenchymal stem cells (ASCs) in accelerated tendon regeneration in a rat model.
   Methods: Through a series of experiments, such as isolation and identification of ASCs, scanning electron microscopy, mercury intrusion porosimetry (MIP), laser scanning con focal microscopy and the CCK-8 test, the biocompatibility of GMs was evaluated. In an in vivo study, 64 rat right transected Achilles tendons were randomly divided into four groups: the ASCs+GMs group (microunits aggregated by multiple ASC-laden GMs injected into the gap), the ASCs group (ASCs injected into the gap), the GMs group (GMs injected into the gap) and the blank defect group (non-treated). At 2 and 4 weeks postoperatively, the healing tissue was harvested to evaluate the gross observation and scoring, biomechanical testing, histological staining and quantitative scoring. Gait analysis was performed over time. The 64 rats were randomly assigned into 4 groups: (1) micro-unit group (ASCs+GMs) containing ASC (105)-loaded 120 GMs in 60 mu L DMEM; (2) cell control group (ASCs) containing 106 ASCs in 60 mu L DMEM; (3) GM control group (GMs) containing 120 blank GMs in 60 mu L DMEM; (4) blank defect group (Defect) containing 60 mu L DMEM, which were injected into the defect sites. All animals were sacrificed at 2 and 4 weeks postsurgery (Table 1).
   Results: In an in vitro study, GMs (from 126 mu m to 348 mu m) showed good porosities and a three-dimensional void structure with a good interpore connectivity of the micropores and exhibited excellent biocompatibility with ASCs. As the culture time elapsed, the extracellular matrix (ECM) secreted by ASCs encased the GMs, bound multiple microspheres together, and then formed active tendon tissue-engineering microunits. In animal experiments, the ASCs+GMs group and the ASCs group showed stimulatory effects on Achilles tendon healing. Moreover, the ASCs+GMs group was the best at improving the macroscopic appearance, histological morphology, Achilles functional index (AFI), and biomechanical properties of repair tissue without causing adverse immune reactions.
   Conclusion: Porous GMs were conducive to promoting cell proliferation and facilitating ECM secretion. The ASCs-GMs matrices showed an obvious therapeutic efficiency for Achilles tendon rupture in rats.
C1 [Yang, Xuan; Meng, Haoye; Peng, Jiang; Xu, Lijuan; Wang, Yu; Zhao, Yanxu; Quan, Qi; Yu, Wen; Chen, Mingxue; Lu, Shibi; Wang, Aiyuan] Chinese Peoples Liberat Army Gen Hosp, Beijing Key Lab Regenerat Med Orthoped, Dept Orthoped Surg, Key Lab Musculoskeletal Trauma & War Injuries PLA, Beijing 100853, Peoples R China.
   [Yang, Xuan; Shi, Tong] Shanxi Med Univ, Dept Orthoped, Hosp 1, YanHu Dist Branch, Yuncheng 044000, Shanxi, Peoples R China.
   [Sun, Xun] Tianjin Hosp, Dept Orthoped, Tianjin 300211, Peoples R China.
   [Du, Yanan] Tsinghua Univ, Sch Med, Dept Biomed Engn, Beijing 100084, Peoples R China.
RP Peng, J; Wang, AY (通讯作者)，Chinese Peoples Liberat Army Gen Hosp, Beijing Key Lab Regenerat Med Orthoped, Dept Orthoped Surg, Key Lab Musculoskeletal Trauma & War Injuries PLA, Beijing 100853, Peoples R China.
EM pengjiang301@126.com; aiyuanwang301@126.com
RI Sun, Xun/B-5575-2018
OI Sun, Xun/0000-0001-6172-4692; yang, xuan/0000-0003-0296-6725
FU Beijing Key Laboratory Innovation and development project [Z1611000050
   16059]; Key projects of Military Medical Innovation Engineering
   [16CXZ044]; military's 13th five-year priorities [BWS13C029]; National
   Natural Science Foundation of China [31640029]
FX This study was supported by the Beijing Key Laboratory Innovation and
   development project (Z1611000050 16059), Key projects of Military
   Medical Innovation Engineering (16CXZ044), The military's 13th five-year
   priorities (BWS13C029), National Natural Science Foundation of China
   (31640029).
CR Alviti F, 2017, J FOOT ANKLE SURG, V56, P581, DOI 10.1053/j.jfas.2017.01.039
   Assal M, 2002, J BONE JOINT SURG AM, V84A, P161, DOI 10.2106/00004623-200202000-00001
   BEST TM, 1993, J ORTHOP RES, V11, P897, DOI 10.1002/jor.1100110617
   DelaRosa O, 2009, TISSUE ENG PT A, V15, P2795, DOI [10.1089/ten.tea.2008.0630, 10.1089/ten.TEA.2008.0630]
   Docheva D, 2015, ADV DRUG DELIVER REV, V84, P222, DOI 10.1016/j.addr.2014.11.015
   Drury JL, 2003, BIOMATERIALS, V24, P4337, DOI 10.1016/S0142-9612(03)00340-5
   Ganestam A, 2016, KNEE SURG SPORT TR A, V24, P3730, DOI 10.1007/s00167-015-3544-5
   Gonzalez MA, 2009, ARTHRITIS RHEUM-US, V60, P1006, DOI 10.1002/art.24405
   Gonzalez MA, 2009, GASTROENTEROLOGY, V136, P978, DOI 10.1053/j.gastro.2008.11.041
   Hamdi H, 2011, CARDIOVASC RES, V91, P483, DOI 10.1093/cvr/cvr099
   Huttunen TT, 2014, AM J SPORT MED, V42, P2419, DOI 10.1177/0363546514540599
   Juncosa-Melvin N, 2006, TISSUE ENG, V12, P681, DOI 10.1089/ten.2006.12.681
   Knobe M, 2015, INT ORTHOP, V39, P535, DOI 10.1007/s00264-014-2615-4
   Komatsu I, 2016, ACTA BIOMATER, V42, P136, DOI 10.1016/j.actbio.2016.06.026
   Konerding MA, 2010, INT J MOL MED, V25, P217, DOI 10.3892/ijmm_00000333
   Kuraitis D, 2012, BIOMATERIALS, V33, P428, DOI 10.1016/j.biomaterials.2011.09.078
   Lantto I, 2015, SCAND J MED SCI SPOR, V25, pE133, DOI 10.1111/sms.12253
   Li YQ, 2014, P NATL ACAD SCI USA, V111, P13511, DOI 10.1073/pnas.1411295111
   Lin H, 2012, BIOMATERIALS, V33, P4480, DOI 10.1016/j.biomaterials.2012.03.012
   Liu W, 2014, ACTA BIOMATER, V10, P1864, DOI 10.1016/j.actbio.2013.12.008
   Liu XH, 2011, NAT MATER, V10, P398, DOI [10.1038/NMAT2999, 10.1038/nmat2999]
   MA GWC, 1977, CLIN ORTHOP RELAT R, P247
   Mahieu NN, 2006, AM J SPORT MED, V34, P226, DOI 10.1177/0363546505279918
   MURRELL GAC, 1992, J ORTHOPAED RES, V10, P398, DOI 10.1002/jor.1100100313
   Nilsson-Helander K, 2007, AM J SPORT MED, V35, P421, DOI 10.1177/0363546506294856
   Okamoto N, 2010, J BONE JOINT SURG AM, V92A, P2776, DOI 10.2106/JBJS.I.01325
   Olsson N, 2013, AM J SPORT MED, V41, P2867, DOI 10.1177/0363546513503282
   Piryaei A, 2011, STEM CELL REV REP, V7, P103, DOI 10.1007/s12015-010-9126-5
   Place ES, 2009, NAT MATER, V8, P457, DOI [10.1038/nmat2441, 10.1038/NMAT2441]
   Rickert M, 2008, J BONE JOINT SURG AM, V90A, P445
   Seeger JD, 2006, PHARMACOEPIDEM DR S, V15, P784, DOI 10.1002/pds.1214
   Seminatore C, 2010, STROKE, V41, P153, DOI 10.1161/STROKEAHA.109.563015
   Sheth U, 2017, BONE JOINT J, V99-b, P1629
   Silbernagel KG, 2012, AM J SPORT MED, V40, P1564, DOI 10.1177/0363546512447926
   Sivasubramaniyan K, 2018, AM J SPORT MED, V46, P3521, DOI 10.1177/0363546518804807
   Sorrenti SJ, 2006, FOOT ANKLE INT, V27, P407, DOI 10.1177/107110070602700603
   Stein BE, 2015, INT ORTHOP, V39, P901, DOI 10.1007/s00264-015-2725-7
   Stoll C, 2011, BIOMATERIALS, V32, P4806, DOI 10.1016/j.biomaterials.2011.03.026
   Thai HM, 2009, CELL TRANSPLANT, V18, P283, DOI 10.3727/096368909788535004
   Twaddle BC, 2007, AM J SPORT MED, V35, P2033, DOI 10.1177/0363546507307503
   Vadala G, 2012, J TISSUE ENG REGEN M, V6, P348, DOI 10.1002/term.433
   Vosseller JT, 2013, FOOT ANKLE INT, V34, P49, DOI 10.1177/1071100712460223
   Xiong Y, 2015, TISSUE ENG PT A, V21, P1886, DOI [10.1089/ten.tea.2014.0539, 10.1089/ten.TEA.2014.0539]
   Yang X, 2018, BONE JOINT RES, V7, P561, DOI 10.1302/2046-3758.710.BJR-2018-0004.R2
   Zhang L, 2013, STEM CELLS DEV, V22, P3074, DOI 10.1089/scd.2013.0142
   Zou J, 2016, BIOMED RES INT, V2016, DOI 10.1155/2016/9364170
NR 46
TC 3
Z9 3
U1 3
U2 9
PU DOVE MEDICAL PRESS LTD
PI ALBANY
PA PO BOX 300-008, ALBANY, AUCKLAND 0752, NEW ZEALAND
SN 1178-2013
J9 INT J NANOMED
JI Int. J. Nanomed.
PY 2020
VL 15
BP 7155
EP 7171
DI 10.2147/IJN.S238399
PG 17
WC Nanoscience & Nanotechnology; Pharmacology & Pharmacy
WE Science Citation Index Expanded (SCI-EXPANDED)
SC Science & Technology - Other Topics; Pharmacology & Pharmacy
GA OC1XX
UT WOS:000578956300002
PM 33061373
OA Green Published, gold
DA 2022-07-22
ER

PT J
AU Dundar, N
   Guneri, B
   Uzel, M
   Doganer, A
AF Dundar, Nuh
   Guneri, Bulent
   Uzel, Murat
   Doganer, Adem
TI Biomechanical comparison of Bunnell, modified Kessler, and Tsuge tendon
   repair techniques using two suture types
SO ACTA ORTHOPAEDICA ET TRAUMATOLOGICA TURCICA
LA English
DT Article
DE Suture techniques; Bunnell technique; Ankle injuries; Achilles tendon;
   Tsuge technique; Tendon injuries; Modified Kessler technique
ID ACHILLES-TENDON; IN-VITRO; RUPTURE; STRENGTH; CADAVER
AB Objective: The aim of this study was to compare the biomechanical properties of modified Kessler, Bunnell and Tsuge techniques in sheep Achilles tendon tear repaired using polyester and polydioxanone sutures which are also compared.
   Methods: Sixty sheep Achilles tendons were cut transversely as a substitute for rupture and repaired using modified Kessler, Bunnell and Tsuge techniques with No. 2 braided polyester and monofilament polydioxanone sutures. Specimens were loaded to failure. Four biomechanical parameters - ultimate strength (US), strength to 2 mm gap (S2G), strength to 5 mm gap (S5G) and Young's modulus (YM) - were recorded for statistical analysis.
   Results: The Tsuge-Polyester group demonstrated the highest results regarding S2G (21.24 +/- 4.75 N) (p=.002) and S5G (38.91 +/- 7.45 N) (p=.002). According to YM, the Bunnell-Polyester group was the most superior (1929.9 +/- 512.28 kilopascal) (p=.009). In the repairs with the polydioxanone suture, Bunnell technique achieved the best purchase with regard to S2G (18.14 +/- 6.86 N) (p=.006) and S5G (35.69 +/- 13.49 N) (p=.015). The difference between the three repair techniques with the polydioxanone suture was statistically insipidicant regarding US (p=.252) and YM (p=.338). Concerning the repairs with the polyester suture, the repair techniques demonstrated no statistically silmificant difference in terms of US (p=.195), S2G (p=.667), S5G (p=.689) and YM (p=.195). Regarding the mean S2G and S5G values, the polyester suture was significantly superior to the polydioxanone suture in modified Kessler repairs (S2G: p<.001, S5G: p=.001) and Tsuge repairs (S2G: p<.001, S5G: p=.009). The polyester suture was also significantly superior to the polydioxanone suture in the modified Kessler repairs with respect to YM (p=.003).
   Conclusion: This study supports the opinion that Tsuge technique is a promising procedure in Achilles tendon repair (ATR). For Tsuge and modified Kessler repairs, braided polyester suture use appears more advantageous compared to monofilament polydioxanone suture use in biomechanical terms.
C1 [Dundar, Nuh; Guneri, Bulent; Uzel, Murat] Kahramanmaras Sutcu Imam Univ, Sch Med, Dept Orthoped & Traumatol, Kahramanmaras, Turkey.
   [Doganer, Adem] Kahramanmaras Sutcu Imam Univ, Sch Med, Dept Biostat & Med Informat, Kahramanmaras, Turkey.
RP Guneri, B (通讯作者)，Kahramanmaras Sutcu Imam Univ, Sch Med, Dept Orthoped & Traumatol, Kahramanmaras, Turkey.
EM bulentguneri@yahoo.com
OI Doganer, Adem/0000-0002-0270-9350
CR Askeland DR., 2010, SCI ENG MAT
   Canale S. T., 2013, CAMPBELLS OPERATIVE, P2846
   Ding WG, 2014, FOOT ANKLE SURG, V20, P105, DOI 10.1016/j.fas.2013.12.007
   Doral MN, 2010, KNEE SURG SPORT TR A, V18, P638, DOI 10.1007/s00167-010-1083-7
   Fu CY, 2015, SURG-J R COLL SURG E, V13, P207, DOI 10.1016/j.surge.2014.03.010
   Fu Chongyang, 2011, Zhongguo Xiu Fu Chong Jian Wai Ke Za Zhi, V25, P1214
   Gorschewsky O, 1999, INJURY, V30, P315, DOI 10.1016/S0020-1383(99)00089-3
   Herbort M, 2008, ARCH ORTHOP TRAUM SU, V128, P1273, DOI 10.1007/s00402-008-0602-1
   Kadakia AR, 2017, J AM ACAD ORTHOP SUR, V25, P23, DOI 10.5435/JAAOS-D-15-00187
   Khan RJK, 2005, J BONE JOINT SURG AM, V87A, P2202, DOI 10.2106/JBJS.D.03049
   Kim JC, 2007, J MATER SCI-MATER M, V18, P2363, DOI 10.1007/s10856-007-3114-6
   Leppilahti J, 1996, ACTA ORTHOP SCAND, V67, P277, DOI 10.3109/17453679608994688
   Park HG, 2001, FOOT ANKLE INT, V22, P985, DOI 10.1177/107110070102201211
   Santrock RD, 2017, CLIN PODIATR MED SUR, V34, P245, DOI 10.1016/j.cpm.2016.10.010
   Shepard ME, 2007, FOOT ANKLE INT, V28, P1074, DOI 10.3113/FAI.2007.1074
   Stavrou M, 2013, J ORTHOP SURG-HONG K, V21, P232, DOI 10.1177/230949901302100224
   TSUGE K, 1974, HAND, V6, P255, DOI 10.1016/0072-968X(74)90036-9
   Wang D, 2015, FOOT ANKLE SURG, V21, P250, DOI 10.1016/j.fas.2015.01.009
   WATSON TW, 1995, FOOT ANKLE INT, V16, P191, DOI 10.1177/107110079501600404
   Yammine Kaissar, 2017, Foot (Edinb), V30, P13, DOI 10.1016/j.foot.2016.09.006
   Yildirim Y, 2002, FOOT ANKLE INT, V23, P1126
   Yildirim Yakup, 2006, Acta Orthop Traumatol Turc, V40, P164
   Zandbergen RA, 2005, ACTA ORTHOP, V76, P408, DOI 10.1080/17453670510041312
NR 23
TC 0
Z9 1
U1 0
U2 2
PU TURKISH ASSOC ORTHOPAEDICS TRAUMATOLOGY
PI ISTANBUL
PA SEHREMINI MAH KOYUNCU SK CIGDEM APT NO 4 D 5 FATIH, ISTANBUL, 00000,
   TURKEY
SN 1017-995X
J9 ACTA ORTHOP TRAUMATO
JI Acta Orthop. Traumatol. Turc.
PD JAN
PY 2020
VL 54
IS 1
BP 104
EP 112
DI 10.5152/j.aott.2020.01.411
PG 9
WC Orthopedics
WE Science Citation Index Expanded (SCI-EXPANDED)
SC Orthopedics
GA KT1UV
UT WOS:000518799000016
PM 32175904
OA gold, Green Published
DA 2022-07-22
ER

PT J
AU Maempel, JF
   Clement, ND
   Duckworth, AD
   Keenan, OJF
   White, TO
   Biant, LC
AF Maempel, Julian F.
   Clement, Nick D.
   Duckworth, Andrew D.
   Keenan, Oisin J. F.
   White, Tim O.
   Biant, Leela C.
TI A Randomized Controlled Trial Comparing Traditional Plaster Cast
   Rehabilitation With Functional Walking Boot Rehabilitation for Acute
   Achilles Tendon Ruptures
SO AMERICAN JOURNAL OF SPORTS MEDICINE
LA English
DT Article
DE Achilles tendon; rupture; clinical assessment; ankle
ID NONOPERATIVE TREATMENT; ACCELERATED REHABILITATION; NONSURGICAL
   TREATMENT; FULL WEIGHTBEARING; SURGICAL REPAIR; WEIGHT-BEARING; SURGERY;
   QUESTIONNAIRE; RELIABILITY; MANAGEMENT
AB Background: There has been a shift toward functional nonoperative rehabilitation in the treatment of Achilles tendon rupture (ATR) despite a shortage of studies directly comparing nonoperative functional rehabilitation with traditional nonoperative immobilization. Purpose: To compare patient-reported outcome measures and functional outcomes for nonoperatively treated ATR with traditional cast immobilization or functional rehabilitation in a walking boot. Study Design: Randomized controlled clinical trial; Level of evidence, 2. Methods: In a single-center nonblinded study, 140 patients were randomized to compare treatment for acute ATR in (1) an immobilizing cast in reducing degrees of equinus over a 10-week period with 8 weeks of nonweightbearing mobilization or (2) a walking boot for 8 weeks with reducing equinus and immediate full weightbearing. Exclusion criteria were delayed presentation >2 weeks after injury, tendon reruptures, and latex allergy. Analysis was undertaken on an intention-to-treat basis. Results: A total of 69 patients (median age, 41 years [interquartile range, 33-50.5 years]) were randomized to walking boot treatment and 71 patients (41 [32-49]) to cast treatment. At 6 months, patients treated in a walking boot reported better Short Musculoskeletal Function Assessment (SMFA) dysfunction index (6.62 [2.21-12.50] vs 10.66 [4.96-13.42];P= .050), SMFA bother index (7.29 [2.08-14.58] vs 10.42 [5.73-19.27];P= .04), Achilles Tendon Total Rupture Score (71.5 [53.50-84.25] vs 54.0 [37-76];P= .01), and Foot and Ankle Questionnaire core score (91 [81.89-97.55] vs 85 [78.25-92.09];P= .04). At 1 year, there was no difference in SMFA dysfunction index (2.21 [0.74-5.88] vs 2.94 [1.47-6.62];P= .25), SMFA bother index (2.08 [0-9.38] vs 5.21 [0.52-11.98];P= .25), Achilles Tendon Total Rupture Score (92 [72.50-96] vs 87.5 [66.0-94.75];P= .21), or Foot and Ankle Questionnaire core score (97.75 [89.46-99.00] vs 95.50 [90.88-97.50];P= .18). Rerupture occurred in 5 and 11 patients (P= .075) and venous thromboembolism in 2 and 3 patients (P= .67) in the boot and cast groups, respectively. Fifteen patients in the boot group but none in the cast group had skin problems (P< .001). Patients treated in a boot returned to driving at a median 12 weeks (vs 13 weeks for cast;P= .045), but there was no difference in time to return to work (P= .48). Conclusion: Functional rehabilitation with early weightbearing is a safe alternative to traditional immobilizing treatment for ATR, giving better early functional outcomes, albeit with a higher incidence of transient minor skin complications. Registration: NCT02598843 (ClinicalTrials.gov identifier).
C1 [Maempel, Julian F.; Clement, Nick D.; Duckworth, Andrew D.; Keenan, Oisin J. F.; White, Tim O.; Biant, Leela C.] Royal Infirm Edinburgh NHS Trust, Edinburgh Orthopaed Trauma, Edinburgh, Midlothian, Scotland.
   [Maempel, Julian F.] Royal Prince Alfred Hosp, 50 Missenden Rd, Sydney, NSW 2050, Australia.
   [Maempel, Julian F.] Univ Malta, Med Sch, Msida, Malta.
   [Clement, Nick D.; Duckworth, Andrew D.; Keenan, Oisin J. F.; White, Tim O.] Royal Infirm Edinburgh NHS Trust, Edinburgh, Midlothian, Scotland.
   [Duckworth, Andrew D.; White, Tim O.] Univ Edinburgh, Edinburgh, Midlothian, Scotland.
   [Biant, Leela C.] Univ Manchester, Med Sch, Manchester, Lancs, England.
   [Biant, Leela C.] Manchester Univ NHS Fdn Trust, Manchester Orthopaed Ctr, Manchester, Lancs, England.
RP Maempel, JF (通讯作者)，Royal Prince Alfred Hosp, 50 Missenden Rd, Sydney, NSW 2050, Australia.
EM julian.maempel@doctors.org.uk
OI Biant, Leela/0000-0002-5291-8898; White, Tim/0000-0002-6125-7112
CR Aujla RS, 2019, INJURY, V50, P995, DOI 10.1016/j.injury.2019.03.007
   Barfod KW, 2013, J FOOT ANKLE SURG, V52, P629, DOI 10.1053/j.jfas.2013.04.012
   Barfod KW, 2014, J BONE JOINT SURG AM, V96A, P1497, DOI 10.2106/JBJS.M.01273
   Bergkvist D, 2012, J BONE JOINT SURG AM, V94A, P1229, DOI 10.2106/JBJS.J.01601
   Black N., 2013, BMJ-BRIT MED J, V346, pF167, DOI [10.1136/bmj.f167, 10.1136/bmj, DOI 10.1136/BMJ.F167]
   Brumann M, 2014, INJURY, V45, P1782, DOI 10.1016/j.injury.2014.06.022
   Busse JW, 2009, J CLIN EPIDEMIOL, V62, P1210, DOI 10.1016/j.jclinepi.2009.01.014
   Carmont MR, 2013, KNEE SURG SPORT TR A, V21, P1356, DOI 10.1007/s00167-012-2146-8
   CETTI R, 1993, AM J SPORT MED, V21, P791, DOI 10.1177/036354659302100606
   Concato J, 2016, J INVEST MED, V64, P1166, DOI 10.1136/jim-2016-000206
   Costa ML, 2006, J BONE JOINT SURG BR, V88B, P69, DOI 10.1302/0301-620X.88B1.16549
   Ecker TM, 2016, AM J SPORT MED, V44, P1004, DOI 10.1177/0363546515623501
   Ganestam A, 2016, KNEE SURG SPORT TR A, V24, P3730, DOI 10.1007/s00167-015-3544-5
   Hutchison AM, 2015, BONE JOINT J, V97B, P510, DOI 10.1302/0301-620X.97B4.35314
   Huttunen TT, 2014, AM J SPORT MED, V42, P2419, DOI 10.1177/0363546514540599
   Jiang N, 2012, INT ORTHOP, V36, P765, DOI 10.1007/s00264-011-1431-3
   Johanson NA, 2004, J BONE JOINT SURG AM, V86A, P902, DOI 10.2106/00004623-200405000-00003
   Josey RA, 2003, FOOT ANKLE INT, V24, P775, DOI 10.1177/107110070302401007
   Keating JF, 2011, J BONE JOINT SURG BR, V93B, P1071, DOI 10.1302/0301-620X.93B8.25998
   Lantto I, 2016, AM J SPORT MED, V44, P2406, DOI 10.1177/0363546516651060
   Linnehan R, 2015, ORTHOPEDICS TODA NOV
   Mark-Christensen T, 2016, KNEE SURG SPORT TR A, V24, P1852, DOI 10.1007/s00167-014-3180-5
   Mattila VM, 2015, BRIT J SPORT MED, V49, P1084, DOI 10.1136/bjsports-2013-092756
   Metz R, 2008, AM J SPORT MED, V36, P1688, DOI 10.1177/0363546508319312
   Moller M, 2001, J BONE JOINT SURG BR, V83B, P843
   Nilsson-Helander K, 2007, AM J SPORT MED, V35, P421, DOI 10.1177/0363546506294856
   Nilsson-Helander K, 2010, AM J SPORT MED, V38, P2186, DOI 10.1177/0363546510376052
   Ochen Y, 2019, BMJ-BRIT MED J, V364, DOI 10.1136/bmj.k5120
   Olsson N, 2014, SCAND J MED SCI SPOR, V24, P152, DOI 10.1111/j.1600-0838.2012.01497.x
   Olsson N, 2013, AM J SPORT MED, V41, P2867, DOI 10.1177/0363546513503282
   Porter MD, 2015, ANZ J SURG, V85, P373, DOI 10.1111/ans.12910
   Reda Y, 2020, FOOT ANKLE SURG, V26, P280, DOI 10.1016/j.fas.2019.03.010
   Reito A, 2018, FOOT ANKLE INT, V39, P694, DOI 10.1177/1071100717754042
   Sheth U, 2017, BONE JOINT J, V99B, P78, DOI [10.1302/0301-620X.99B1.BJJ-2016-0434.R1, 10.1302/0301-620x.99b1.bjj-2016-0434.r1]
   Soroceanu A, 2012, J BONE JOINT SURG AM, V94A, P2136, DOI 10.2106/JBJS.K.00917
   Suchak AA, 2006, CLIN ORTHOP RELAT R, P216, DOI 10.1097/01.blo.0000203458.05135.74
   Swiontkowski MF, 1999, J BONE JOINT SURG AM, V81A, P1245, DOI 10.2106/00004623-199909000-00006
   Timmins N, 2008, BRIT MED J, V336, P1464, DOI 10.1136/bmj.39618.627951.80
   Twaddle BC, 2007, AM J SPORT MED, V35, P2033, DOI 10.1177/0363546507307503
   Wallace RGH, 2011, J BONE JOINT SURG BR, V93B, P1362, DOI 10.1302/0301-620X.93B10.26187
   Willits K, 2010, J BONE JOINT SURG AM, V92A, P2767, DOI 10.2106/JBJS.I.01401
NR 41
TC 6
Z9 6
U1 1
U2 12
PU SAGE PUBLICATIONS INC
PI THOUSAND OAKS
PA 2455 TELLER RD, THOUSAND OAKS, CA 91320 USA
SN 0363-5465
EI 1552-3365
J9 AM J SPORT MED
JI Am. J. Sports Med.
PD SEP
PY 2020
VL 48
IS 11
BP 2755
EP 2764
AR 0363546520944905
DI 10.1177/0363546520944905
EA AUG 2020
PG 10
WC Orthopedics; Sport Sciences
WE Science Citation Index Expanded (SCI-EXPANDED)
SC Orthopedics; Sport Sciences
GA NI3RK
UT WOS:000561604600001
PM 32816521
DA 2022-07-22
ER

PT J
AU Guerra, FD
   Vieira, CP
   Almeida, MS
   Oliveira, LP
   de Aro, AA
   Pimentel, ER
AF Guerra, Flavia Da Re
   Vieira, Cristiano Pedrozo
   Almeida, Marcos Santos
   Oliveira, Leticia Prado
   de Aro, Andrea Aparecida
   Pimentel, Edson Rosa
TI LLLT improves tendon healing through increase of MMP activity and
   collagen synthesis
SO LASERS IN MEDICAL SCIENCE
LA English
DT Article
DE Achilles tendon; Rupture; LLLT; Collagen; Matrix metalloproteinase 9;
   Matrix metalloproteinase 2
ID MATRIX-METALLOPROTEINASE ACTIVITY; LASER IRRADIATION; ACHILLES-TENDON;
   GROWTH-FACTORS; BIOLOGY; REPAIR; RAT; IMMOBILIZATION; EXPRESSION;
   RUPTURES
AB The Achilles tendon has a high incidence of rupture, and the healing process leads to a disorganized extracellular matrix (ECM) with a high rate of injury recurrence. To evaluate the effects of different conditions of low-level laser (LLL) application on partially tenotomized tendons, adult male rats were divided into the following groups: G1, intact; G2, injured; G3, injured + LLL therapy (LLLT; 4 J/cm(2) continuous); G4, injured + LLLT (4 J/cm(2), 20 Hz); G5, injured; G6, injured + LLLT (4 J/cm(2) continuous); and G7, injured + LLLT (4 J/cm(2), 20 Hz until the 7th day and 2 kHz from 8 to 14 days). G2, G3, and G4 were euthanized 8 days after injury, and G5, G6, and G7 were euthanized on the 15th day. The quantification of hydroxyproline (HOPro) and non-collagenous protein (NCP), zymography for matrix metalloproteinase (MMP)-2 and MMP-9, and Western blotting (WB) for collagen types I and III were performed. HOPro levels showed a significant decrease in all groups (except G7) when compared with G1. The NCP level increased in all transected groups. WB for collagen type I showed an increase in G4 and G7. For collagen type III, G4 presented a higher value than G2. Zymography for MMP-2 indicated high values in G4 and G7. MMP-9 increased in both treatment groups euthanized at 8 days, especially in G4. Our results indicate that the pulsed LLLT improved the remodeling of the ECM during the healing process in tendons through activation of MMP-2 and stimulation of collagen synthesis.
C1 [Guerra, Flavia Da Re; Vieira, Cristiano Pedrozo; Almeida, Marcos Santos; Oliveira, Leticia Prado; de Aro, Andrea Aparecida; Pimentel, Edson Rosa] Univ Campinas UNICAMP, Inst Biol, Dept Anat Cell Biol & Physiol & Biophys, BR-13083970 Sao Paulo, Brazil.
RP Guerra, FD (通讯作者)，Univ Campinas UNICAMP, Inst Biol, Dept Anat Cell Biol & Physiol & Biophys, CP 6109, BR-13083970 Sao Paulo, Brazil.
EM dgflavia@yahoo.com.br
RI Vieira, Cristiano Pedrozo/L-2517-2014; Guerra, Flavia D/N-6274-2014;
   Da+Ré+Guerra, Flávia/ABE-6224-2021; Pimentel, Edson/E-3805-2012; de Aro,
   Andrea Aparecida/E-3753-2012
OI Guerra, Flavia D/0000-0001-9142-9109; Pimentel,
   Edson/0000-0002-1049-8880; de Aro, Andrea Aparecida/0000-0003-2895-0000;
   Almeida, Marcos/0000-0002-2080-0609
CR ABRAHAMSSON SO, 1991, SCAND J PLAST RECONS, pUR1
   Ahtikoski AM, 2003, ACTA PHYSIOL SCAND, V177, P473, DOI 10.1046/j.1365-201X.2003.01061.x
   AMIEL D, 1983, CLIN ORTHOP RELAT R, P265
   Aro AE, 2008, CONNECT TISSUE RES, V49, P443, DOI 10.1080/03008200802325250
   Baranauskas V, 1998, APPL BIOCHEM BIOTECH, V69, P91, DOI 10.1007/BF02919391
   Benevides GP, 2004, CONNECT TISSUE RES, V45, P206, DOI 10.1080/03008200490522997
   Bosman FT, 2003, J PATHOL, V200, P423, DOI 10.1002/path.1437
   BRADFORD MM, 1976, ANAL BIOCHEM, V72, P248, DOI 10.1016/0003-2697(76)90527-3
   Chan HKF, 2007, J ORTHOP SPORT PHYS, V37, P399, DOI 10.2519/jospt.2007.2412
   Chen CH, 2009, J ORTHOP RES, V27, P646, DOI 10.1002/jor.20800
   Clutterbuck AL, 2010, VET J, V183, P27, DOI 10.1016/j.tvjl.2008.09.022
   de Aro AA, 2010, Retainer of rats for laser beam and biocompounds application, Patent No. 90006224
   de Aro AA, 2012, CONNECT TISSUE RES, V53, P29, DOI 10.3109/03008207.2011.608868
   Demir H, 2004, LASER SURG MED, V35, P84, DOI 10.1002/lsm.20046
   Elwakil TF, 2007, LASER MED SCI, V22, P53, DOI 10.1007/s10103-006-0423-9
   ENWEMEKA C S, 1991, Journal of Orthopaedic and Sports Physical Therapy, V14, P198
   Enwemeka CS, 2000, LASER THER, V12, P22, DOI DOI 10.5978/ISLSM.12.22
   Gavish L, 2006, LASER SURG MED, V38, P779, DOI 10.1002/lsm.20383
   Gillard JA, 2004, WOUND REPAIR REGEN, V12, P295, DOI 10.1111/j.1067-1927.2004.012314.x
   HEINEGARD D, 1987, METHOD ENZYMOL, V144, P319
   James R, 2008, J HAND SURG-AM, V33A, P102, DOI 10.1016/j.jhsa.2007.09.007
   Jarvinen Tero A H, 2005, Foot Ankle Clin, V10, P255, DOI 10.1016/j.fcl.2005.01.013
   Kannus P, 1997, SCAND J MED SCI SPOR, V7, P107
   Karousou E, 2008, CLIN ORTHOP RELAT R, V466, P1577, DOI 10.1007/s11999-008-0255-y
   Lesic A, 2004, CURR ORTHOPAED, V18, P63, DOI 10.1016/j.cuor.2003.10.002
   Marqueti RC, 2006, AM J SPORT MED, V34, P1274, DOI 10.1177/0363546506286867
   MURPHY PG, 1994, BIOCHEM CELL BIOL, V72, P403, DOI 10.1139/o94-054
   Ng GYF, 2004, LASER SURG MED, V35, P317, DOI 10.1002/lsm.20077
   Oakes B.W., 2003, REHABILITATION SPORT, P56
   OBrien M, 1997, SCAND J MED SCI SPOR, V7, P55
   Oliveira FS, 2009, LASER SURG MED, V41, P271, DOI 10.1002/lsm.20760
   Oshiro W, 2003, J HAND SURG-AM, V28A, P814, DOI 10.1016/S0363-5023(03)00366-6
   Pinfildi CE, 2005, LASER SURG MED, V37, P74, DOI 10.1002/lsm.20190
   Pugliese Livia Souza, 2003, Pesqui Odontol Bras, V17, P307
   Salate ACB, 2005, PHOTOMED LASER SURG, V23, P470, DOI 10.1089/pho.2005.23.470
   Sharma P., 2006, Journal of Musculoskeletal & Neuronal Interactions, V6, P181
   Sharma P, 2005, SURG-J R COLL SURG E, V3, P309, DOI 10.1016/S1479-666X(05)80109-X
   Snoek-van Beurden PAM, 2005, BIOTECHNIQUES, V38, P73, DOI 10.2144/05381RV01
   STEGEMAN.H, 1967, CLIN CHIM ACTA, V18, P267, DOI 10.1016/0009-8981(67)90167-2
   TOWBIN H, 1979, P NATL ACAD SCI USA, V76, P4350, DOI 10.1073/pnas.76.9.4350
   Vieira CP, 2012, CONNECT TISSUE RES, V53, P160, DOI 10.3109/03008207.2011.620189
   Wren TAL, 2001, CLIN BIOMECH, V16, P245, DOI 10.1016/S0268-0033(00)00089-9
   Yamaguchi M, 2010, EUR J ORTHODONT, V32, P131, DOI 10.1093/ejo/cjp078
   Yoon J. H., 2005, Journal of Musculoskeletal & Neuronal Interactions, V5, P22
NR 44
TC 50
Z9 50
U1 0
U2 13
PU SPRINGER LONDON LTD
PI LONDON
PA 236 GRAYS INN RD, 6TH FLOOR, LONDON WC1X 8HL, ENGLAND
SN 0268-8921
EI 1435-604X
J9 LASER MED SCI
JI Lasers Med. Sci.
PD SEP
PY 2013
VL 28
IS 5
BP 1281
EP 1288
DI 10.1007/s10103-012-1236-7
PG 8
WC Engineering, Biomedical; Surgery
WE Science Citation Index Expanded (SCI-EXPANDED)
SC Engineering; Surgery
GA 209GF
UT WOS:000323742200009
PM 23179310
DA 2022-07-22
ER

PT J
AU Ko, PY
   Hsu, CH
   Hong, CK
   Hung, MT
   Su, WR
   Wu, PT
   Jou, IM
   Su, FC
AF Ko, Po-Yen
   Hsu, Chieh-Hsiang
   Hong, Chih-Kai
   Hung, Ming-Tung
   Su, Wei-Ren
   Wu, Po-Ting
   Jou, I-Ming
   Su, Fong-Chin
TI Jigless knotless internal brace versus other open Achilles tendon
   repairs using a progressive rehabilitation protocol: a biomechanical
   study
SO BMC MUSCULOSKELETAL DISORDERS
LA English
DT Article
DE Achilles tendon rupture; Minimally invasive; Jigless knotless internal
   brace; Biomechanical study; Krachow suture; tipple bundle
ID MODIFIED PERCUTANEOUS REPAIR; IN-VITRO; SURAL NERVE; KRACKOW; SUTURE;
   RUPTURES; STRENGTH; MOBILIZATION; FLEXION; SYSTEM
AB Background The jigless knotless internal brace surgery (JKIB), a modified minimal invasive surgery (MIS) for acute Achilles tendon injury, has advantages of preventing sural-nerve injury in MIS and superficial wound infection in open surgery, as demonstrated in previous clinical research. However, to date, biomechanical testing has not yet been validated. Materials and methods Sixty fresh porcine Achilles tendons were used to compare the JKIB with other open surgery techniques, the four-stranded Krackow suture (4sK) and the triple-bundle suture (TBS) in biomechanical testing with cyclic loading set at 1 Hz. This approach simulated a progressive rehabilitation protocol where 20-100 N was applied in the first 1000 cycles, followed by 20-190 N in the second 1000 cycles, and then 20-369 N in the third 1000 cycles. The cycles leading to repair gaps of 2 mm, 5 mm, and 10 mm were recorded. The survival cycles were defined as repair gap of 10 mm. Results With respect to survival cycles, a significant difference was found among the three groups, in which the TBS was the most robust, followed by the JKIB and the 4sK, where the mean survived cycles were 2639.3 +/- 263.55, 2073.6 +/- 319.92, and 1425.25 +/- 268.96, respectively. Significant differences were verified via a post hoc analysis with the Mann-Whitney U test after the Bonferroni correction (p < 0.017). Conclusions The TBS was the strongest suture structure in acute Achilles tendon repair. However, the JKIB could be an option in acute Achilles tendon repair with the MIS technique due to it being more robust than the 4sK, which has been typically favored for use in open repair.
C1 [Ko, Po-Yen; Hsu, Chieh-Hsiang; Wu, Po-Ting; Su, Fong-Chin] Natl Cheng Kung Univ, Dept Biomed Engn, Tainan, Taiwan.
   [Ko, Po-Yen; Hong, Chih-Kai; Su, Wei-Ren; Wu, Po-Ting] Natl Cheng Kung Univ Hosp, Dept Orthoped, Tainan, Taiwan.
   [Jou, I-Ming] I Shou Univ, E Da Hosp, Dept Orthopaed Surg & Pathol, Kaohsiung, Taiwan.
   [Hung, Ming-Tung; Jou, I-Ming] GEG Orthoped Clin, Tainan, Taiwan.
RP Su, FC (通讯作者)，Natl Cheng Kung Univ, Dept Biomed Engn, Tainan, Taiwan.; Jou, IM (通讯作者)，I Shou Univ, E Da Hosp, Dept Orthopaed Surg & Pathol, Kaohsiung, Taiwan.; Jou, IM (通讯作者)，GEG Orthoped Clin, Tainan, Taiwan.
EM jming@mail.ncku.edu.tw; fcsu@ncku.edu.tw
OI Hsu, Chieh-Hsiang/0000-0001-9989-7003
FU National Science Council, Taiwan [MOST108-2314-B-006-011-MY2]; National
   Cheng Kung University Hospital, Tainan, Taiwan [NCKUH-10904016]
FX This study was supported by grant MOST108-2314-B-006-011-MY2 from the
   National Science Council, Taiwan, and grant NCKUH-10904016 from the
   National Cheng Kung University Hospital, Tainan, Taiwan.
CR Akizuki KH, 2001, BRIT J SPORT MED, V35, P329, DOI 10.1136/bjsm.35.5.329
   Assal M, 2002, J BONE JOINT SURG AM, V84A, P161, DOI 10.2106/00004623-200202000-00001
   Aufwerber S, 2020, AM J SPORT MED, V48, P3296, DOI 10.1177/0363546520956677
   Biz C, 2021, MEDICINA-LITHUANIA, V57, DOI 10.3390/medicina57101073
   Blackmon JA, 2013, J FOOT ANKLE SURG, V52, P42, DOI 10.1053/j.jfas.2012.09.010
   Carmont MR, 2013, KNEE SURG SPORT TR A, V21, P1361, DOI 10.1007/s00167-013-2423-1
   Carmont MR, 2008, KNEE SURG SPORT TR A, V16, P199, DOI 10.1007/s00167-007-0411-z
   Choi GW, 2017, KNEE SURG SPORT TR A, V25, P1878, DOI 10.1007/s00167-016-4265-0
   Clanton T, 2020, ORTHOP CLIN N AM, V51, P391, DOI 10.1016/j.ocl.2020.02.005
   Clanton TO, 2015, AM J SPORT MED, V43, P1957, DOI 10.1177/0363546515587082
   Cox JT, 2014, AM J SPORT MED, V42, P2727, DOI 10.1177/0363546514549004
   Cretnik A, 2005, AM J SPORT MED, V33, P1369, DOI 10.1177/0363546504271501
   Cretnik A, 2019, J FOOT ANKLE SURG, V58, P828, DOI 10.1053/j.jfas.2018.11.029
   Dai W, 2020, J SCI MED SPORT
   Demetracopoulos CA, 2014, FOOT ANKLE INT, V35, P612, DOI 10.1177/1071100714524550
   Diniz P, 2020, KNEE SURG SPORT TR A, V28, P4011, DOI 10.1007/s00167-020-06010-8
   Elton JP, 2010, FOOT ANKLE INT, V31, P914, DOI 10.3113/FAI.2010.0914
   FITZGIBBONS RE, 1993, AM J SPORT MED, V21, P724, DOI 10.1177/036354659302100516
   Ganestam A, 2016, KNEE SURG SPORT TR A, V24, P3730, DOI 10.1007/s00167-015-3544-5
   Haji A, 2004, FOOT ANKLE INT, V25, P215, DOI 10.1177/107110070402500404
   Heitman DE, 2011, FOOT ANKLE INT, V32, P879, DOI 10.3113/FAI.2011.0879
   Hong CK, 2018, J ORTHOP SURG-HONG K, V26, DOI 10.1177/2309499018799514
   Huffard B, 2008, CLIN BIOMECH, V23, P1158, DOI 10.1016/j.clinbiomech.2008.05.007
   Hurmeydan OM, 2020, FOOT ANKLE INT, V41, P140, DOI 10.1177/1071100719879659
   Huttunen TT, 2014, AM J SPORT MED, V42, P2419, DOI 10.1177/0363546514540599
   Jaakkola JI, 2000, FOOT ANKLE INT, V21, P14, DOI 10.1177/107110070002100103
   KAKIUCHI M, 1995, J BONE JOINT SURG BR, V77B, P60, DOI 10.1302/0301-620X.77B1.7822398
   Kearney RS, 2012, PHYSIOTHERAPY, V98, P24, DOI 10.1016/j.physio.2011.04.349
   Ko PY, 2019, J ORTHOP SURG RES, V14, DOI 10.1186/s13018-019-1471-8
   KRACKOW KA, 1986, J BONE JOINT SURG AM, V68A, P764, DOI 10.2106/00004623-198668050-00020
   Kujala UM, 2005, CLIN J SPORT MED, V15, P133, DOI 10.1097/01.jsm.0000165347.55638.23
   Labib SA, 2009, FOOT ANKLE INT, V30, P410, DOI 10.3113/FAI.2009.0410
   Lee SJ, 2008, FOOT ANKLE INT, V29, P427, DOI 10.3113/FAI.2008.0427
   Lee SJ, 2009, AM J SPORT MED, V37, P786, DOI 10.1177/0363546508328595
   Leigheb Massimiliano, 2017, Acta Biomed, V88, P50, DOI 10.23750/abm.v88i4 -S.6794
   Lim J, 2001, FOOT ANKLE INT, V22, P559, DOI 10.1177/107110070102200705
   Lu J, 2019, J FOOT ANKLE SURG, V58, P938, DOI 10.1053/j.jfas.2018.12.014
   MA GWC, 1977, CLIN ORTHOP RELAT R, P247
   Maes Renaud, 2006, Acta Orthop Belg, V72, P179
   Maffulli N, 2011, FOOT ANKLE INT, V32, P9, DOI 10.3113/FAI.2011.0009
   McMahon Samuel E, 2011, Foot Ankle Surg, V17, P211, DOI 10.1016/j.fas.2010.11.001
   Mullaney MJ, 2006, AM J SPORT MED, V34, P1120, DOI 10.1177/0363546505284186
   Nyyssonen T, 2008, SCAND J SURG, V97, P272, DOI 10.1177/145749690809700312
   O'Donnell Seth W, 2019, Foot Ankle Spec, V12, P563, DOI 10.1177/1938640019863268
   Orishimo KF, 2008, J FOOT ANKLE SURG, V47, P34, DOI 10.1053/j.jfas.2007.10.008
   Ostrander RV, 2016, ARTHROSCOPY, V32, P1384, DOI 10.1016/j.arthro.2016.01.054
   Palmes D, 2002, J ORTHOPAED RES, V20, P939, DOI 10.1016/S0736-0266(02)00032-3
   Pneumaticos SG, 2000, FOOT ANKLE INT, V21, P551, DOI 10.1177/107110070002100704
   Porter KJ, 2014, FOOT ANKLE SURG, V20, P90, DOI 10.1016/j.fas.2013.11.005
   Saarensilta IA, 2020, KNEE SURG SPORT TR A, V28, P3978, DOI 10.1007/s00167-020-05982-x
   Shepard ME, 2007, FOOT ANKLE INT, V28, P1074, DOI 10.3113/FAI.2007.1074
   WATSON TW, 1995, FOOT ANKLE INT, V16, P191, DOI 10.1177/107110079501600404
   Wu YH, 2019, AM J SPORT MED, V47, P2251, DOI 10.1177/0363546518824601
NR 53
TC 0
Z9 1
U1 0
U2 1
PU BMC
PI LONDON
PA CAMPUS, 4 CRINAN ST, LONDON N1 9XW, ENGLAND
EI 1471-2474
J9 BMC MUSCULOSKEL DIS
JI BMC Musculoskelet. Disord.
PD OCT 28
PY 2021
VL 22
IS 1
AR 908
DI 10.1186/s12891-021-04809-1
PG 8
WC Orthopedics; Rheumatology
WE Science Citation Index Expanded (SCI-EXPANDED)
SC Orthopedics; Rheumatology
GA WO4DC
UT WOS:000712405700001
PM 34711221
OA Green Published, gold
DA 2022-07-22
ER

PT J
AU Konerding, MA
   Arlt, F
   Wellmann, A
   Li, V
   Li, W
AF Konerding, Moritz A.
   Arlt, Friederike
   Wellmann, Axel
   Li, Vincent
   Li, William
TI Impact of combinatory growth factor application on rabbit Achilles
   tendon injury with operative versus conservative treatment: A pilot
   study
SO INTERNATIONAL JOURNAL OF MOLECULAR MEDICINE
LA English
DT Article
DE Achilles tendon injury; growth factors; operative treatment;
   conservative treatment; rabbit
ID GENE-THERAPY; FACTOR-BB; IN-VITRO; COLLAGEN; RUPTURES
AB Acute Achilles tendon (AT) rupture is a common injury with a comparatively high complication rate. Presently, surgical treatments compete with nonoperative treatment modalities. The aim of this study was to elucidate the possible beneficial effects of short-term combinatory application of growth factors on tendon healing during operative or conservative treatment. In this controlled laboratory study, the left ATs of 40 adult New Zealand White rabbits were transected and either sutured or treated conservatively. Half of the animals from each treatment modality group repetitively received a mixture of VEGF165, bFGF, and rPDGF which was administered peritendineally. The left legs were immobilized with external fixateurs for 6 weeks. The ATs were harvested 3 months after intervention. Tensile strength tests revealed no significant differences between operative and conservative treatments. Compared to the normal right ATs, 60% of the average breaking strength was reached 3 months after surgery. Growth factor application did not result in significant improvements. Only a tendency towards higher blood vessel densities was noted in the groups treated with the factors. Collagen type I/III ratios also displayed no significant differences. This study indicates that there is no difference in the biomechanical outcome of conservative versus operative AT rupture treatment and only a marginal impact of short-term combinatory growth and angiogenesis factor application.
C1 [Konerding, Moritz A.; Arlt, Friederike] Johannes Gutenberg Univ Mainz, Inst Anat & Cell Biol, Univ Med Ctr, D-55099 Mainz, Germany.
   [Wellmann, Axel] Inst Pathol Celle, D-29223 Celle, Germany.
   [Li, Vincent] Brigham & Womens Hosp, Wound Healing Clin, Boston, MA 02115 USA.
   [Li, William] Angiogenesis Fdn, Boston, MA USA.
RP Konerding, MA (通讯作者)，Johannes Gutenberg Univ Mainz, Inst Anat & Cell Biol, Univ Med Ctr, Saarstr 13-18, D-55099 Mainz, Germany.
EM konerdin@uni-mainz.de
FU German Army; The Angiogenesis Foundation
FX This study was made possible by a grant of the German Army (Inspection
   of the Sanitary Staff; M-SAB-005) to M.K. and A.W. We greatly appreciate
   the additional support of The Angiogenesis Foundation (Boston, MA, USA).
   The authors thank Dr G. Spatkowski for surgery and animal care. We
   further thank our technicians Kerstin Bahr and Gisela Reifenberg for
   their skillful and patient assistance. This study contains parts of the
   doctoral thesis of F.A.
CR Aspenberg P, 2007, INT ORTHOP, V31, P783, DOI 10.1007/s00264-007-0398-6
   Batten ML, 1996, J ORTHOPAED RES, V14, P736, DOI 10.1002/jor.1100140509
   Bishop JR, 2007, NATURE, V446, P1030, DOI 10.1038/nature05817
   Bolt P, 2007, J BONE JOINT SURG AM, V89A, P1315, DOI 10.2106/JBJS.F.00257
   Broughton G, 2006, PLAST RECONSTR SURG, V117, p6S, DOI 10.1097/01.prs.0000225429.76355.dd
   Cornelison DDW, 2004, GENE DEV, V18, P2231, DOI 10.1101/gad.1214204
   Forslund C, 2003, AM J SPORT MED, V31, P555, DOI 10.1177/03635465030310041301
   Geer DJ, 2005, AM J PATHOL, V167, P1575, DOI 10.1016/S0002-9440(10)61242-4
   GREENHALGH DG, 1990, AM J PATHOL, V136, P1235
   Hildebrand KA, 1998, AM J SPORT MED, V26, P549, DOI 10.1177/03635465980260041401
   Ilhami K, 2004, ARCH ORTHOP TRAUM SU, V124, P608, DOI 10.1007/s00402-003-0626-5
   JUNQUEIRA LCU, 1979, HISTOCHEM J, V11, P447, DOI 10.1007/BF01002772
   Khan RJK, 2005, J BONE JOINT SURG AM, V87A, P2202, DOI 10.2106/JBJS.D.03049
   Kurtz CA, 1999, AM J SPORT MED, V27, P363, DOI 10.1177/03635465990270031701
   Leppilahti J, 1996, ACTA ORTHOP SCAND, V67, P277, DOI 10.3109/17453679608994688
   Lindahl U, 2007, THROMB HAEMOSTASIS, V98, P109, DOI 10.1160/Th07-04-0310
   Liu PY, 2005, DNA CELL BIOL, V24, P751, DOI 10.1089/dna.2005.24.751
   Lohrer H, 2008, CLIN ORTHOP RELAT R, V466, P2230, DOI 10.1007/s11999-008-0298-0
   Metz R, 2008, AM J SPORT MED, V36, P1688, DOI 10.1177/0363546508319312
   PIERCE GF, 1988, J EXP MED, V167, P974, DOI 10.1084/jem.167.3.974
   PIERCE GF, 1991, AM J PATHOL, V138, P629
   Reddy GK, 1999, WOUND REPAIR REGEN, V7, P518, DOI 10.1046/j.1524-475X.1999.00518.x
   SCHLOSSER MJ, 1987, LIFE SCI, V41, P1345, DOI 10.1016/0024-3205(87)90608-4
   Stepp MA, 2002, J CELL SCI, V115, P4517, DOI 10.1242/jcs.00128
   Tallon C, 2001, MED SCI SPORT EXER, V33, P1983, DOI 10.1097/00005768-200112000-00002
   Weibel E.R., 1979, STEREOLOGICAL METHOD, V1
   WHITTAKER P, 1994, BASIC RES CARDIOL, V89, P397, DOI 10.1007/BF00788278
   Wolloscheck T, 2004, Hernia, V8, P233
   Zhang F, 2003, PLAST RECONSTR SURG, V112, P1613, DOI 10.1097/01.PRS.0000086772.72535.A4
   Zhao YN, 2005, AM J PATHOL, V167, P1519, DOI 10.1016/S0002-9440(10)61238-2
   Zhou ZJ, 2004, CANCER RES, V64, P4699, DOI 10.1158/0008-5472.CAN-04-0810
NR 31
TC 6
Z9 6
U1 0
U2 2
PU SPANDIDOS PUBL LTD
PI ATHENS
PA POB 18179, ATHENS, 116 10, GREECE
SN 1107-3756
EI 1791-244X
J9 INT J MOL MED
JI Int. J. Mol. Med.
PD FEB
PY 2010
VL 25
IS 2
BP 217
EP 224
DI 10.3892/ijmm_00000333
PG 8
WC Medicine, Research & Experimental
WE Science Citation Index Expanded (SCI-EXPANDED)
SC Research & Experimental Medicine
GA 547CE
UT WOS:000273862300005
PM 20043130
OA Bronze
DA 2022-07-22
ER

PT J
AU Wenning, M
   Mauch, M
   Heitner, A
   Lienhard, J
   Ritzmann, R
   Paul, J
AF Wenning, Markus
   Mauch, Marlene
   Heitner, Albrecht
   Lienhard, Johannes
   Ritzmann, Ramona
   Paul, Jochen
TI Neuromechanical activation of triceps surae muscle remains altered at
   3.5 years following open surgical repair of acute Achilles tendon
   rupture
SO KNEE SURGERY SPORTS TRAUMATOLOGY ARTHROSCOPY
LA English
DT Article
DE Achilles tendon rupture; EMG; Neuromuscular activation; Functional
   performance testing
AB Purpose To assess whether the neuromuscular activation pattern following Achilles tendon rupture and repair may contributes to the observable functional deficits in this severe and increasingly frequent injury. Methods In this study, the neuromuscular activation using surface EMG of n = 52 patients was assessed during a battery of functional performance tasks to assess potential alterations of muscular activation and recruitment. We analyzed the injured leg vs. the contralateral healthy leg at a mean of 3.5 years following open surgical repair. The testing battery included isokinetic strength testing, bipedal and single-legged heel-rise testing as well as gait analysis. Results During isokinetic testing, we observed a higher activation integral for all triceps surae muscles of the injured side during active dorsiflexion, e.g., eccentric loading on the injured leg, while concentric plantarflexion showed no significant difference. Dynamic heel-rise testing showed a higher activation in concentric and eccentric loading for all posterior muscles on the injured side (not significant); while static heel-rise for 10 sec. revealed a significantly higher activation. Further analysis of frequency of fast Fourier-transformed EMG revealed a significantly higher median frequency in the injured leg. Gait analysis revealed a higher pre-activation of the tibialis anterior before ground contact, while medial and lateral gastrocnemius muscles of the injured leg showed a significantly higher activation during push-off phase. Conclusions The results of this study provide evidence on the neuromuscular changes 3.5 years following open surgical Achilles tendon repair. These complex neuromuscular changes are manifested to produce the maximum force output whilst protecting the previously injured tendon. The observed alterations may be related to an increased recruitment of type II muscle fibers which could make the muscles prone to fatigue.
C1 [Wenning, Markus; Mauch, Marlene; Heitner, Albrecht; Ritzmann, Ramona; Paul, Jochen] Rennbahnklinik, Basel, Switzerland.
   [Wenning, Markus] Albert Ludwigs Univ Freiburg, Univ Med Ctr Freiburg, Dept Orthoped & Trauma Surg, Fac Med, Freiburg, Germany.
   [Lienhard, Johannes] Albert Ludwigs Univ Freiburg, Dept Sport & Sport Sci, Biomech & Motor Control, Freiburg, Germany.
RP Wenning, M (通讯作者)，Rennbahnklinik, Basel, Switzerland.; Wenning, M (通讯作者)，Albert Ludwigs Univ Freiburg, Univ Med Ctr Freiburg, Dept Orthoped & Trauma Surg, Fac Med, Freiburg, Germany.
EM research@rennbahnklinik.ch
OI Wenning, Markus/0000-0002-3929-2317
FU Projekt DEAL
FX Open Access funding enabled and organized by Projekt DEAL.
CR Agres AN, 2015, SCAND J MED SCI SPOR, V25, P860, DOI 10.1111/sms.12456
   Arslan A, 2014, ACTA ORTHOP TRAUMATO, V48, P563, DOI 10.3944/AOTT.2014.14.0132
   Bilodeau M, 2003, J ELECTROMYOGR KINES, V13, P83, DOI 10.1016/S1050-6411(02)00050-0
   Bohm S, 2019, P ROY SOC B-BIOL SCI, V286, DOI 10.1098/rspb.2019.2560
   Burden A, 2010, J ELECTROMYOGR KINES, V20, P1023, DOI 10.1016/j.jelekin.2010.07.004
   Chowdhury SK, 2015, J ELECTROMYOGR KINES, V25, P205, DOI 10.1016/j.jelekin.2014.11.005
   De la Fuente C, 2022, FOOT ANKLE SURG, V28, P37, DOI 10.1016/j.fas.2021.01.007
   Diniz P, 2020, KNEE SURG SPORT TR A, V28, P4011, DOI 10.1007/s00167-020-06010-8
   Don R, 2007, CLIN BIOMECH, V22, P211, DOI 10.1016/j.clinbiomech.2006.10.001
   Drechsler WI, 2006, EUR J APPL PHYSIOL, V98, P613, DOI 10.1007/s00421-006-0311-9
   Henriksen M, 2009, J BIOMECH, V42, P2702, DOI 10.1016/j.jbiomech.2009.08.009
   Hermens HJ, 2000, J ELECTROMYOGR KINES, V10, P361, DOI 10.1016/S1050-6411(00)00027-4
   Ishikawa M, 2005, J APPL PHYSIOL, V99, P603, DOI 10.1152/japplphysiol.00189.2005
   Kastoft R, 2019, KNEE SURG SPORT TR A, V27, P2781, DOI 10.1007/s00167-018-5058-4
   Laurent D, 2020, SCI REP-UK, V10, DOI 10.1038/s41598-020-77691-x
   McHugh MP, 2019, ORTHOP J SPORTS MED, V7, DOI 10.1177/2325967119883357
   Nilsson-Helander K, 2010, AM J SPORT MED, V38, P2186, DOI 10.1177/0363546510376052
   Novacheck TF, 1998, GAIT POSTURE, V7, P77, DOI 10.1016/S0966-6362(97)00038-6
   Oda H, 2017, SPORTS MED, V47, P1221, DOI 10.1007/s40279-016-0629-3
   Okoroha KR, 2020, AM J SPORT MED, V48, P1720, DOI 10.1177/0363546520909389
   Paavo V.Komi, 2003, STRENGTH POWER SPORT
   Sharma P, 2005, J BONE JOINT SURG AM, V87A, P187, DOI 10.2106/JBJS.D.01850
   Silbernagel KG, 2012, AM J SPORT MED, V40, P1564, DOI 10.1177/0363546512447926
   Souissi H, 2017, J BIOMECH, V57, P141, DOI 10.1016/j.jbiomech.2017.03.029
   Speedtsberg MB, 2019, ORTHOP J SPORTS MED, V7, DOI 10.1177/2325967119854324
   Suydam SM, 2015, KNEE SURG SPORT TR A, V23, P868, DOI 10.1007/s00167-013-2512-1
   Tengman T, 2013, ORTHOP J SPORTS MED, V1, DOI 10.1177/2325967113504734
   Trofa DP, 2018, ORTHOP J SPORTS MED, V6, DOI 10.1177/2325967118810772
   Trofa DP, 2017, AM J SPORT MED, V45, P2864, DOI 10.1177/0363546517713001
   Von Tscharner V, 2006, J ELECTROMYOGR KINES, V16, P188, DOI 10.1016/j.jelekin.2005.07.004
   Warren GL, 2000, MED SCI SPORT EXER, V32, P820, DOI 10.1097/00005768-200004000-00015
   Wenning M, 2022, ARCH ORTHOP TRAUM SU, V142, P1337, DOI 10.1007/s00402-020-03746-3
   Winter D. A., 2009, BIOMECHANICS MOTOR C, V4th
   Zellers JA, 2019, J ORTHOP RES, V37, P933, DOI 10.1002/jor.24260
NR 34
TC 2
Z9 2
U1 0
U2 2
PU SPRINGER
PI NEW YORK
PA ONE NEW YORK PLAZA, SUITE 4600, NEW YORK, NY, UNITED STATES
SN 0942-2056
EI 1433-7347
J9 KNEE SURG SPORT TR A
JI Knee Surg. Sports Traumatol. Arthrosc.
PD AUG
PY 2021
VL 29
IS 8
BP 2517
EP 2527
DI 10.1007/s00167-021-06512-z
EA MAR 2021
PG 11
WC Orthopedics; Sport Sciences; Surgery
WE Science Citation Index Expanded (SCI-EXPANDED)
SC Orthopedics; Sport Sciences; Surgery
GA TR3RI
UT WOS:000625087000002
PM 33660053
OA Green Published, hybrid
DA 2022-07-22
ER

PT J
AU Westin, O
   Svensson, M
   Helander, KN
   Samuelsson, K
   Silbernagel, KG
   Olsson, N
   Karlsson, J
   Olofsson, EH
AF Westin, Olof
   Svensson, Mikael
   Helander, Katarina Nilsson
   Samuelsson, Kristian
   Silbernagel, Karin Gravare
   Olsson, Nicklas
   Karlsson, Jon
   Olofsson, Elisabeth Hansson
TI Cost-effectiveness analysis of surgical versus non-surgical management
   of acute Achilles tendon ruptures
SO KNEE SURGERY SPORTS TRAUMATOLOGY ARTHROSCOPY
LA English
DT Article
DE Achilles tendon rupture; HRQoL; ICER; QALY; Sensitivity analyses;
   Surgical; Non-surgical; Treatment
ID REPAIR; METAANALYSIS
AB Purpose An Achilles tendon rupture is a common injury that typically affects people in the middle of their working lives. The injury has a negative impact in terms of both morbidity for the individual and the risk of substantial sick leave. The aim of this study was to investigate the cost-effectiveness of surgical compared with non-surgical management in patients with an acute Achilles tendon rupture.
   Methods One hundred patients (86 men, 14 women; mean age, 40 years) with an acute Achilles tendon rupture were randomised (1:1) to either surgical treatment or non-surgical treatment, both with an accelerated rehabilitation protocol (surgical n = 49, non-surgical n = 51). One of the surgical patients was excluded due to a partial re-rupture and five surgical patients were lost to the 1-year economic follow-up. One patient was excluded due to incorrect inclusion and one was lost to the 1-year follow-up in the non-surgical group. The cost was divided into direct and indirect costs. The direct cost is the actual cost of health care, whereas the indirect cost is the production loss related to the impact of the patient's injury in terms of lost ability to work. The health benefits were assessed using quality-adjusted life years (QALYs). Sampling uncertainty was assessed by means of non-parametric boot-strapping.
   Results Pre-injury, the groups were comparable in terms of demographic data and health-related quality of life (HRQoL). The mean cost of surgical management was (sic)7332 compared with (sic)6008 for non-surgical management (p = 0.024). The mean number of QALYs during the 1-year time period was 0.89 and 0.86 in the surgical and non-surgical groups respectively. The (incremental) cost-effectiveness ratio was (sic)45,855. Based on bootstrapping, the cost-effectiveness acceptability curve shows that the surgical treatment is 57% likely to be cost-effective at a threshold value of (sic)50,000 per QALY.
   Conclusions Surgical treatment was more expensive compared with non-surgical management. The cost-effectiveness results give a weak support (57% likelihood) for the surgical treatment to be cost-effective at a willingness to pay per QALY threshold of (sic)50,000. This is support for surgical treatment; however, additionally cost-effectiveness studies alongside RCTs are important to clarify which treatment option is preferred from a cost-effectiveness perspective.
C1 [Westin, Olof; Helander, Katarina Nilsson; Samuelsson, Kristian; Silbernagel, Karin Gravare; Olsson, Nicklas; Karlsson, Jon; Olofsson, Elisabeth Hansson] Univ Gothenburg, Sahlgrenska Acad, Inst Clin Sci, Dept Orthopaed, Gothenburg, Sweden.
   [Westin, Olof; Helander, Katarina Nilsson; Samuelsson, Kristian; Olsson, Nicklas; Karlsson, Jon] Sahlgrens Univ Hosp, Molndal, Sweden.
   [Silbernagel, Karin Gravare] Univ Delaware, Dept Phys Therapy, Newark, DE USA.
   [Svensson, Mikael] Univ Gothenburg, Sahlgrenska Acad, Hlth Metr, Gothenburg, Sweden.
   [Olofsson, Elisabeth Hansson] Sahlgrens Acad, Inst Hlth & Care Sci, Gothenburg, Sweden.
RP Westin, O (通讯作者)，Univ Gothenburg, Sahlgrenska Acad, Inst Clin Sci, Dept Orthopaed, Gothenburg, Sweden.; Westin, O (通讯作者)，Sahlgrens Univ Hosp, Molndal, Sweden.
EM olof.westin@gmail.com
RI Svensson, Mikael/P-2108-2014
OI Svensson, Mikael/0000-0003-1113-7478
CR Bonsell S, 2000, Proc (Bayl Univ Med Cent), V13, P327
   Brazier JE, 1999, RHEUMATOLOGY, V38, P870, DOI 10.1093/rheumatology/38.9.870
   Brooks R, 1996, HEALTH POLICY, V37, P53, DOI 10.1016/0168-8510(96)00822-6
   Carmont MR, 2013, KNEE SURG SPORT TR A, V21, P1361, DOI 10.1007/s00167-013-2423-1
   Coughlin MT, 2003, CRIT CARE MED, V31, pS7, DOI 10.1097/00003246-200301001-00002
   Deng SL, 2017, J FOOT ANKLE SURG, V56, P1236, DOI 10.1053/j.jfas.2017.05.036
   Ebinesan AD, 2008, DISABIL REHABIL, V30, P1721, DOI 10.1080/09638280701786815
   Ganestam A, 2016, KNEE SURG SPORT TR A, V24, P3730, DOI 10.1007/s00167-015-3544-5
   Hamid KS, 2013, ARTHROSCOPY, V29, P1900, DOI 10.1016/j.arthro.2013.10.001
   Huttunen TT, 2014, AM J SPORT MED, V42, P2419, DOI 10.1177/0363546514540599
   Khan RJK, 2010, COCHRANE DB SYST REV, DOI 10.1002/14651858.CD003674.pub4
   Levi N, 1997, INJURY, V28, P311, DOI 10.1016/S0020-1383(96)00200-8
   Moller M, 2001, J BONE JOINT SURG BR, V83B, P843
   Nilsson-Helander K, 2010, AM J SPORT MED, V38, P2186, DOI 10.1177/0363546510376052
   Nwachukwu BU, 2015, AM J SPORT MED, V43, P1530, DOI 10.1177/0363546514544684
   Olsson N, 2013, AM J SPORT MED, V41, P2867, DOI 10.1177/0363546513503282
   Pajala A, 2002, J BONE JOINT SURG AM, V84A, P2016, DOI 10.2106/00004623-200211000-00017
   Pot JH, 2014, FOOT ANKLE ONLINE J, V7, P5, DOI DOI 10.3827/FAOJ.2014.0703.0005
   Sheth U, 2017, BONE JOINT J, V99B, P1629, DOI 10.1302/0301-620X.99B12.BJJ-2017-0465.R1
   Sheth U, 2017, BONE JOINT J, V99B, P78, DOI [10.1302/0301-620X.99B1.BJJ-2016-0434.R1, 10.1302/0301-620x.99b1.bjj-2016-0434.r1]
   SILFVERSKIOLD KL, 1993, J HAND SURG-AM, V18A, P58, DOI 10.1016/0363-5023(93)90246-Y
   Soroceanu A, 2012, J BONE JOINT SURG AM, V94A, P2136, DOI 10.2106/JBJS.K.00917
   Truntzer JN, 2017, J AM ACAD ORTHOP SUR, V25, P449, DOI 10.5435/JAAOS-D-16-00553
   Willits K, 2010, J BONE JOINT SURG AM, V92A, P2767, DOI 10.2106/JBJS.I.01401
   Zhang H, 2015, MEDICINE, V94, DOI 10.1097/MD.0000000000001951
NR 25
TC 20
Z9 22
U1 1
U2 3
PU SPRINGER
PI NEW YORK
PA 233 SPRING ST, NEW YORK, NY 10013 USA
SN 0942-2056
EI 1433-7347
J9 KNEE SURG SPORT TR A
JI Knee Surg. Sports Traumatol. Arthrosc.
PD OCT
PY 2018
VL 26
IS 10
BP 3074
EP 3082
DI 10.1007/s00167-018-4953-z
PG 9
WC Orthopedics; Sport Sciences; Surgery
WE Science Citation Index Expanded (SCI-EXPANDED)
SC Orthopedics; Sport Sciences; Surgery
GA GT9AR
UT WOS:000444832000026
PM 29696317
OA Green Published, hybrid
DA 2022-07-22
ER

PT J
AU Chamberlain, CS
   Clements, AEB
   Kink, JA
   Choi, U
   Baer, GS
   Halanski, MA
   Hematti, P
   Vanderby, R
AF Chamberlain, Connie S.
   Clements, Anna E. B.
   Kink, John A.
   Choi, Ugeun
   Baer, Geoffrey S.
   Halanski, Matthew A.
   Hematti, Peiman
   Vanderby, Ray
TI Extracellular Vesicle-Educated Macrophages Promote Early Achilles Tendon
   Healing
SO STEM CELLS
LA English
DT Article
DE Macrophages; Mesenchymal stromal cells; Achilles tendon injury;
   Extracellular vesicles; Tendon healing
ID MESENCHYMAL STEM-CELLS; STROMAL CELLS; REPAIR; ANGIOGENESIS; MODEL;
   DIFFERENTIATION; POLARIZATION; REGENERATION; EXPRESSION; PLASTICITY
AB Tendon healing follows a complex series of coordinated events, which ultimately produces a mechanically inferior tissue more scar-like than native tendon. More regenerative healing occurs when anti-inflammatory M2 macrophages play a more dominant role. Mesenchymal stromal/stem cells (MSCs) are able to polarize macrophages to an M2 immunophenotype via paracrine mechanisms. We previously reported that coculture of CD14+ macrophages (MQs) with MSCs resulted in a unique M2-like macrophage. More recently, we generated M2-like macrophages using only extracellular vesicles (EVs) isolated from MSCs creating "EV-educated macrophages" (also called exosome-educated macrophages [EEMs]), thereby foregoing direct use of MSCs. For the current study, we hypothesized that cell therapy with EEMs would improve in vivo tendon healing by modulating tissue inflammation and endogenous macrophage immunophenotypes. We evaluated effects of EEMs using a mouse Achilles tendon rupture model and compared results to normal tendon healing (without any biologic intervention), MSCs, MQs, or EVs. We found that exogenous administration of EEMs directly into the wound promoted a healing response that was significantly more functional and more regenerative. Injured tendons treated with exogenous EEMs exhibited (a) improved mechanical properties, (b) reduced inflammation, and (c) earlier angiogenesis. Treatment with MSC-derived EVs alone were less effective functionally but stimulated a biological response as evidenced by an increased number of endothelial cells and decreased M1/M2 ratio. Because of their regenerative and immunomodulatory effects, EEM treament could provide a novel strategy to promote wound healing in this and various other musculoskeletal injuries or pathologies where inflammation and inadequate healing is problematic.
C1 [Chamberlain, Connie S.; Clements, Anna E. B.; Choi, Ugeun; Baer, Geoffrey S.; Halanski, Matthew A.; Vanderby, Ray] Univ Wisconsin, Dept Orthoped & Rehabil, 1111 Highland Ave,5059 WIMR, Madison, WI 53705 USA.
   [Kink, John A.; Hematti, Peiman] Univ Wisconsin, Dept Med, 1111 Highland Ave,4033 WIMR, Madison, WI 53705 USA.
   [Kink, John A.; Hematti, Peiman] Univ Wisconsin, Carbone Canc Ctr, Madison, WI 53705 USA.
   [Choi, Ugeun; Vanderby, Ray] Univ Wisconsin, Dept Biomed Engn, Madison, WI 53705 USA.
RP Vanderby, R (通讯作者)，Univ Wisconsin, Dept Orthoped & Rehabil, 1111 Highland Ave,5059 WIMR, Madison, WI 53705 USA.; Hematti, P (通讯作者)，Univ Wisconsin, Dept Med, 1111 Highland Ave,4033 WIMR, Madison, WI 53705 USA.
EM pxh@medicine.wisc.edu; vanderby@ortho.wisc.edu
OI Chamberlain, Connie/0000-0002-4980-4347
FU Orthopedic Research and Education Foundation [MSN180250, MSN197479]
FX We acknowledge immunohistochemistry and/or cryosectioning work by Amy
   Ticknor, Allison P. Geoke, and Lorenzo Manalo. This work was supported
   by the Orthopedic Research and Education Foundation Award numbers
   MSN180250 and MSN197479. The content is solely the responsibility of the
   authors and does not necessarily represent the official views of the
   OREF.
CR Abumaree MH, 2013, STEM CELL REV REP, V9, P620, DOI 10.1007/s12015-013-9455-2
   Ahmed IM, 1998, J ORTHOPAED RES, V16, P591, DOI 10.1002/jor.1100160511
   Akhavani MA, 2008, J PLAST RECONSTR AES, V61, P1425, DOI 10.1016/j.bjps.2008.05.041
   Aktas E, 2017, J ORTHOP RES, V35, P269, DOI 10.1002/jor.23258
   Anderson JD, 2016, STEM CELLS, V34, P601, DOI 10.1002/stem.2298
   Aslam M, 2009, AM J RESP CRIT CARE, V180, P1122, DOI 10.1164/rccm.200902-0242OC
   Battiwalla M, 2009, CYTOTHERAPY, V11, P503, DOI 10.1080/14653240903193806
   Benoit M, 2008, J IMMUNOL, V181, P3733, DOI 10.4049/jimmunol.181.6.3733
   Bouchlaka MN, 2017, BIOL BLOOD MARROW TR, V23, P897, DOI 10.1016/j.bbmt.2017.02.018
   Bruno S, 2009, J AM SOC NEPHROL, V20, P1053, DOI 10.1681/ASN.2008070798
   Cantu DA, 2012, STEM CELL TRANSL MED, V1, P740, DOI 10.5966/sctm.2012-0061
   Chamberlain Connie S, 2017, J Cytokine Biol, V2
   Chamberlain CS, 2015, TISSUE ENG PT A, V21, P916, DOI [10.1089/ten.tea.2014.0001, 10.1089/ten.TEA.2014.0001]
   Chamberlain CS, 2014, CONNECT TISSUE RES, V55, P177, DOI 10.3109/03008207.2014.906408
   Chamberlain CS, 2013, PLOS ONE, V8, DOI 10.1371/journal.pone.0071631
   Chamberlain CS, 2011, WOUND REPAIR REGEN, V19, P426, DOI 10.1111/j.1524-475X.2011.00682.x
   Chamberlain CS, 2011, CONNECT TISSUE RES, V52, P203, DOI 10.3109/03008207.2010.511355
   Chamberlain CS, 2009, WOUND REPAIR REGEN, V17, P206, DOI 10.1111/j.1524-475X.2009.00465.x
   da Silva CL, 2005, EXP HEMATOL, V33, P828, DOI 10.1016/j.exphem.2005.03.017
   Dachir S, 2014, CUTAN OCUL TOXICOL, V33, P317, DOI 10.3109/15569527.2013.877023
   Danon D, 1997, EXP GERONTOL, V32, P633, DOI 10.1016/S0531-5565(97)00094-6
   Dayan V, 2011, BASIC RES CARDIOL, V106, P1299, DOI 10.1007/s00395-011-0221-9
   Deng W, 2015, CLIN IMMUNOL, V161, P209, DOI 10.1016/j.clim.2015.07.011
   Depres-tremblay G, 2016, J SHOULDER ELB SURG, V25, P2078, DOI 10.1016/j.jse.2016.06.009
   Dominici M, 2006, CYTOTHERAPY, V8, P315, DOI 10.1080/14653240600855905
   Eslani M, 2018, STEM CELLS, V36, P775, DOI 10.1002/stem.2781
   Falanga V, 2004, WOUND REPAIR REGEN, V12, P320, DOI 10.1111/j.1067-1927.2004.012316.x
   Frisch KE, 2014, J BIOMECH ENG-T ASME, V136, DOI 10.1115/1.4026643
   Frisch KE, 2012, ACTA HISTOCHEM, V114, P140, DOI 10.1016/j.acthis.2011.03.010
   Galatz LM, 2015, J ORTHOP RES, V33, P823, DOI 10.1002/jor.22853
   Gnecchi M, 2005, NAT MED, V11, P367, DOI 10.1038/nm0405-367
   Godwin JW, 2013, P NATL ACAD SCI USA, V110, P9415, DOI 10.1073/pnas.1300290110
   Gomez-Aristizabal A, 2016, PLOS ONE, V11, DOI 10.1371/journal.pone.0147868
   Goolaerts A, 2014, AM J PHYSIOL-LUNG C, V306, pL975, DOI 10.1152/ajplung.00242.2013
   Goren I, 2009, AM J PATHOL, V175, P132, DOI 10.2353/ajpath.2009.081002
   Hanson SE, 2011, TISSUE ENG PT A, V17, P2463, DOI [10.1089/ten.TEA.2010.0716, 10.1089/ten.tea.2010.0716]
   Hu Y, 2016, MEDIAT INFLAMM, V2016, DOI DOI 10.1155/2016/3735452
   Jetten N, 2014, PLOS ONE, V9, DOI 10.1371/journal.pone.0102994
   Kholia S, 2016, VASC PHARMACOL, V86, P64, DOI 10.1016/j.vph.2016.03.005
   Kim J, 2009, EXP HEMATOL, V37, P1445, DOI 10.1016/j.exphem.2009.09.004
   King SN, 2014, J BIOMED MATER RES A, V102, P890, DOI 10.1002/jbm.a.34746
   Kourembanas S, 2015, ANNU REV PHYSIOL, V77, P13, DOI 10.1146/annurev-physiol-021014-071641
   Lai RC, 2010, STEM CELL RES, V4, P214, DOI 10.1016/j.scr.2009.12.003
   Leong DJ, 2016, ANN NY ACAD SCI, V1383, P88, DOI 10.1111/nyas.13262
   Lucas T, 2010, J IMMUNOL, V184, P3964, DOI 10.4049/jimmunol.0903356
   Maggini J, 2010, PLOS ONE, V5, DOI 10.1371/journal.pone.0009252
   Mantovani A, 2013, J PATHOL, V229, P176, DOI 10.1002/path.4133
   Martinez FO, 2009, ANNU REV IMMUNOL, V27, P451, DOI 10.1146/annurev.immunol.021908.132532
   Mirza R, 2009, AM J PATHOL, V175, P2454, DOI 10.2353/ajpath.2009.090248
   Moisy F, BOXCOUNT FRACTAL DIM
   Muir P, 2016, PLOS ONE, V11, DOI 10.1371/journal.pone.0159095
   Murray PJ, 2017, ANNU REV PHYSIOL, V79, P541, DOI 10.1146/annurev-physiol-022516-034339
   Nemeth K, 2009, NAT MED, V15, P42, DOI 10.1038/nm.1905
   Qi X, 2016, INT J BIOL SCI, V12, P836, DOI 10.7150/ijbs.14809
   Saether EE, 2016, STEM CELL REV REP, V12, P42, DOI 10.1007/s12015-015-9633-5
   Selleri S, 2016, ONCOTARGET, V7, P30193, DOI 10.18632/oncotarget.8623
   Shen H, 2016, STEM CELL RES THER, V7, DOI 10.1186/s13287-016-0406-0
   Sindrilaru A, 2011, J CLIN INVEST, V121, P985, DOI 10.1172/JCI44490
   Thery C., 2006, CURR PROTOC CELL BIO, V30
   Torihashi S, 2015, J BIOL CHEM, V290, P22771, DOI 10.1074/jbc.M114.629774
   Trivedi P, 2008, EXP HEMATOL, V36, P350, DOI 10.1016/j.exphem.2007.10.007
   Vasandan AB, 2016, SCI REP-UK, V6, DOI 10.1038/srep38308
   Weisberg SP, 2003, J CLIN INVEST, V112, P1796, DOI 10.1172/JCI200319246
   Yin F, 2014, STEM CELLS, V32, P1278, DOI 10.1002/stem.1638
   Zhang S, 2016, OSTEOARTHR CARTILAGE, V24, P2135, DOI 10.1016/j.joca.2016.06.022
   Zuloff-Shani A, 2004, TRANSFUS APHER SCI, V30, P163, DOI 10.1016/j.transci.2003.11.007
NR 66
TC 65
Z9 68
U1 1
U2 1
PU OXFORD UNIV PRESS
PI OXFORD
PA GREAT CLARENDON ST, OXFORD OX2 6DP, ENGLAND
SN 1066-5099
EI 1549-4918
J9 STEM CELLS
JI Stem Cells
PD MAY
PY 2019
VL 37
IS 5
BP 652
EP 662
DI 10.1002/stem.2988
PG 11
WC Cell & Tissue Engineering; Biotechnology & Applied Microbiology;
   Oncology; Cell Biology; Hematology
WE Science Citation Index Expanded (SCI-EXPANDED)
SC Cell Biology; Biotechnology & Applied Microbiology; Oncology; Hematology
GA HW8PP
UT WOS:000466952000013
PM 30720911
OA Green Published, hybrid
DA 2022-07-22
ER

PT J
AU Plate, JF
   Wiggins, WF
   Haubruck, P
   Scott, AT
   Smith, TL
   Saul, KR
   Mannava, S
AF Plate, Johannes F.
   Wiggins, Walter F.
   Haubruck, Patrick
   Scott, Aaron T.
   Smith, Thomas L.
   Saul, Katherine R.
   Mannava, Sandeep
TI Normal aging alters in vivo passive biomechanical response of the rat
   gastrocnemius-Achilles muscle-tendon unit
SO JOURNAL OF BIOMECHANICS
LA English
DT Article
DE Achilles tendon rupture; Passive biomechanical properties; Quasilinear
   viscoelastic; Fung QLV; Aging
ID VISCOELASTIC PROPERTIES; SKELETAL-MUSCLE; BROWN-NORWAY; MODEL; AGE;
   EXERCISE; FISCHER-344; MECHANISMS; RUPTURES; FORCE
AB Predisposition to Achilles tendon (AT) ruptures in middle-aged individuals may be associated with age-related changes to inherent passive biomechanical properties of the gastrocnemius-Achilles (GC-AT) muscle-tendon unit, due to known muscle-tendon structural changes in normal aging. The goal of this study was to determine whether the passive biomechanical response of the GC-AT muscle-tendon unit was altered with age in 6 young (8 months) and 6 middle-aged (24 months) F344xBN hybrid rats from the National Institute on Aging colony. Fung's quasilinear viscoelastic (QLV) model was used to determine in vivo history and time-dependent load-relaxation response of the GC-AT. Effective stiffness and modulus were also estimated using linear regression analysis. Fung's QLV revealed a significantly decreased magnitude of the relaxation response (parameter C, p=0.026) in middle-aged animals compared to young animals (0.108 +/- 0.007 vs. 0.144 +/- 0.015), with similar time-dependent viscous GC-AT properties (tau(1), tau(2)). The product of elastic parameters (A*B), which represents the initial slope of the elastic response, was significantly increased by 50% in middle-aged rats (p=0.014). Estimated GC-AT stiffness increased 28% at peak tensions in middle-aged rats (2.7 +/- 0.2 N/mm) compared to young rats (1.9 +/- 0.2 N/mm; p=0.036). While the limitations of this animal model must be considered, the changes we describe could be associated with the observation that GC-AT pathology and injury is more common in middle-aged individuals. Further studies are necessary to characterize the load-to-failure behavior of AT in middle-aged compared to young animals. (C) 2012 Elsevier Ltd. All rights reserved.
C1 [Plate, Johannes F.; Scott, Aaron T.; Smith, Thomas L.; Saul, Katherine R.; Mannava, Sandeep] Wake Forest Sch Med, Dept Orthopaed Surg, Winston Salem, NC 27157 USA.
   [Plate, Johannes F.; Wiggins, Walter F.; Mannava, Sandeep] Wake Forest Univ, Grad Sch Arts & Sci, Neurosci Program, Winston Salem, NC 27109 USA.
   [Haubruck, Patrick] Univ Klinikum Heidelberg, Stiftung Orthopad Univ Klin, Heidelberg, Germany.
   [Saul, Katherine R.] Wake Forest Sch Med, Dept Biomed Engn, Winston Salem, NC 27157 USA.
   [Saul, Katherine R.] Wake Forest Univ, Sch Biomed Engn & Sci, Virginia Tech, Winston Salem, NC 27109 USA.
RP Plate, JF (通讯作者)，Wake Forest Sch Med, Dept Orthopaed Surg, Med Ctr Blvd, Winston Salem, NC 27157 USA.
EM jplate@wakehealth.edu
RI Wiggins, Walter/AAT-4620-2020; Saul, Katherine/C-6805-2011; Plate,
   Johannes/AAK-8474-2021; Haubruck, Patrick/U-4442-2019
OI Saul, Katherine/0000-0002-8439-7951; Plate,
   Johannes/0000-0002-2735-2589; Haubruck, Patrick/0000-0002-7045-0387;
   Wiggins, Walter/0000-0002-0258-2708
FU Orthopaedic Research and Education Foundation
FX This research was funded in part by the Orthopaedic Research and
   Education Foundation. This project is taken in part from a dissertation
   submitted to the Neuroscience Program, Wake Forest University Graduate
   School of Arts and Sciences, in partial fulfillment of the requirements
   for the degree of Doctor of Philosophy.
CR Abramowitch SD, 2004, ANN BIOMED ENG, V32, P329, DOI 10.1023/B:ABME.0000017539.85245.6a
   Abramowitch SD, 2010, CLIN BIOMECH, V25, P325, DOI 10.1016/j.clinbiomech.2009.12.007
   Betik AC, 2008, EXP PHYSIOL, V93, P863, DOI 10.1113/expphysiol.2008.042069
   Calvo B, 2010, J BIOMECH, V43, P318, DOI 10.1016/j.jbiomech.2009.08.032
   Delbono O, 2002, BIOGERONTOLOGY, V3, P265, DOI 10.1023/A:1020189627325
   DORTMANS LJMG, 1984, J BIOMECH ENG-T ASME, V106, P198, DOI 10.1115/1.3138483
   Fung Y.C.B, 1972, BIOMECHANICS ITS FDN, P181
   FUNG YCB, 1967, AM J PHYSIOL, V213, P1532, DOI 10.1152/ajplegacy.1967.213.6.1532
   Gibbon WW, 1999, BRIT J SPORT MED, V33, P129, DOI 10.1136/bjsm.33.2.129
   Hagen JL, 2004, J GERONTOL A-BIOL, V59, P1099, DOI 10.1093/gerona/59.11.1099
   Hill A.V., 1938, P ROYAL SOC LONDON B
   Holmes JW, 2006, ADV PHYSIOL EDUC, V30, P67, DOI 10.1152/advan.00072.2005
   Jarvinen TAH, 2001, CURR OPIN RHEUMATOL, V13, P150
   Karamanidis K, 2006, J BIOMECH, V39, P406, DOI 10.1016/j.jbiomech.2004.12.017
   Kay AD, 2009, J APPL PHYSIOL, V106, P1249, DOI 10.1152/japplphysiol.91476.2008
   Kearney RS, 2012, J BONE JOINT SURG BR, V94B, P28, DOI 10.1302/0301-620X.94B1.28008
   KIM AW, 1995, ARCH PHYS MED REHAB, V76, P750, DOI 10.1016/S0003-9993(95)80530-3
   Kubo K, 2001, J APPL PHYSIOL, V90, P520, DOI 10.1152/jappl.2001.90.2.520
   Kubo K, 2007, MED SCI SPORT EXER, V39, P541, DOI 10.1249/01.mss.0000247006.24965.74
   KVIST M, 1994, SPORTS MED, V18, P173, DOI 10.2165/00007256-199418030-00004
   Larsson L, 2000, DRUG AGING, V17, P303, DOI 10.2165/00002512-200017040-00006
   Leppilahti J, 1996, ACTA ORTHOP SCAND, V67, P277, DOI 10.3109/17453679608994688
   Lin RM, 1999, CLIN BIOMECH, V14, P412, DOI 10.1016/S0268-0033(98)00108-9
   Lipman RD, 1996, J GERONTOL A-BIOL, V51, pB54, DOI 10.1093/gerona/51A.1.B54
   Longo UG, 2009, SPORTS MED ARTHROSC, V17, P127, DOI 10.1097/JSA.0b013e3181a3d767
   MacIntosh BR, 2011, JOVE-J VIS EXP, DOI 10.3791/3167
   Maffulli N, 2000, AM J SPORT MED, V28, P499, DOI 10.1177/03635465000280040901
   Magnusson S. P., 1995, Scandinavian Journal of Medicine and Science in Sports, V5, P342
   Mannava S, 2011, ANN BIOMED ENG, V39, P1914, DOI 10.1007/s10439-011-0301-x
   Oh JH, 2010, AM J SPORT MED, V38, P672, DOI 10.1177/0363546509352460
   Onambele GL, 2006, J APPL PHYSIOL, V100, P2048, DOI 10.1152/japplphysiol.01442.2005
   Peltz CD, 2010, J ORTHOP RES, V28, P841, DOI 10.1002/jor.21059
   Quinn R, 2005, NUTRITION, V21, P775, DOI 10.1016/j.nut.2005.04.002
   Raj IS, 2010, EXP GERONTOL, V45, P81, DOI 10.1016/j.exger.2009.10.013
   Reeves ND, 2006, EXP PHYSIOL, V91, P483, DOI 10.1113/expphysiol.2005.032896
   Rice KM, 2005, BIOGERONTOLOGY, V6, P335, DOI 10.1007/s10522-005-4808-0
   SPROTT RL, 1991, NEUROBIOL AGING, V12, P635, DOI 10.1016/0197-4580(91)90113-X
   STOLOV WALTER C., 1966, ARCH PHYS MED REHABIL, V47, P612
   Suchak AA, 2005, FOOT ANKLE INT, V26, P932, DOI 10.1177/107110070502601106
   TAYLOR DC, 1990, AM J SPORT MED, V18, P300, DOI 10.1177/036354659001800314
NR 40
TC 19
Z9 19
U1 0
U2 15
PU ELSEVIER SCI LTD
PI OXFORD
PA THE BOULEVARD, LANGFORD LANE, KIDLINGTON, OXFORD OX5 1GB, OXON, ENGLAND
SN 0021-9290
EI 1873-2380
J9 J BIOMECH
JI J. Biomech.
PD FEB 1
PY 2013
VL 46
IS 3
BP 450
EP 455
DI 10.1016/j.jbiomech.2012.11.007
PG 6
WC Biophysics; Engineering, Biomedical
WE Science Citation Index Expanded (SCI-EXPANDED)
SC Biophysics; Engineering
GA 095EI
UT WOS:000315317100003
PM 23245562
DA 2022-07-22
ER

PT J
AU Ceccarelli, F
   Calderazzi, F
   Pedrazzi, G
AF Ceccarelli, Francesco
   Calderazzi, Filippo
   Pedrazzi, Giuseppe
TI Is There a Relation between AOFAS Ankle-Hindfoot Score and SF-36 in
   Evaluation of Achilles Ruptures Treated by Percutaneous Technique?
SO JOURNAL OF FOOT & ANKLE SURGERY
LA English
DT Article
DE Achilles tendon; ankle; injury; Medical Outcomes Study; questionnaire;
   sural nerve
ID CLINICAL RATING SYSTEMS; TOTAL HIP-ARTHROPLASTY; HEALTH-STATUS MEASURES;
   FOOT FUNCTION INDEX; TENDON RUPTURES; KNEE ARTHROPLASTY; REPAIR;
   RESPONSIVENESS; FRACTURES; SHOULDER
AB The percutaneous technique of Achilles tendon repair seems to offer satisfactory clinical and functional results, although these results have been evaluated mainly using objective rating scales. Recently, some "subjective" rating scales have been combined to evaluate the results of various surgical treatments. The purpose of the present study was to compare the results of a percutaneous Achilles tendon repair evaluated objectively using the American Orthopaedic Foot and Ankle Society (AOFAS) ankle-hindfoot score and subjectively using the Medical Outcomes Study, short-form, 36-item questionnaire (SF-36) questionnaire. A total of 17 consecutive patients were treated for acute Achilles tendon rupture using the modified percutaneous Ma and Griffith technique. We reviewed all patients with a follow-up of 24 to 64 months (mean 45.5). At the final follow-up visit, the AOFAS ankle-hindfoot score of each patient was compared with each 1 of the 8 domains of the SF-36 questionnaire, using the parametric Pearson correlation coefficient and the equivalent nonparametric Spearman rho correlation coefficient. The relation between the objective (AOFAS) and subjective (SF-36) results showed a significant correlation (Pearson's correlation coefficient) between the physical functioning (r = 0.597, p = .011) and bodily pain (r = 0.663, p = .004) SF-36 domains, and a nonstatistically significant correlation with the other SF-36 domains. Very similar results were found using the nonparametric Spearman rho correlation coefficient. These results suggest that regarding pain and function, the AOFAS ankle-hindfoot score and SF-36 provide complementary information; therefore, we believe that the SF-36 questionnaire should be used with the AOFAS ankle-hindfoot score for a more complete evaluation of the outcome. (C) 2014 by the American College of Foot and Ankle Surgeons. All rights reserved.
C1 [Ceccarelli, Francesco; Calderazzi, Filippo] Univ Parma, Osped Maggiore, Dept Surg Sci, Clin Ortoped Operat Unit, I-43100 Parma, Italy.
   [Pedrazzi, Giuseppe] Univ Parma, Dept Publ Hlth, Phys Unit, I-43100 Parma, Italy.
RP Calderazzi, F (通讯作者)，Univ Parma, Osped Maggiore, Dept Surg Sci, Clin Ortoped Operat Unit, Via Gramsci 14, I-43100 Parma, Italy.
EM filippo.calderazzi@tin.it
RI Calderazzi, Filippo/G-9912-2015
OI Calderazzi, Filippo/0000-0002-9087-5147; Pedrazzi,
   Giuseppe/0000-0002-5971-2040; Ceccarelli, Francesco/0000-0002-4457-0457
CR Apolone G, 1998, J CLIN EPIDEMIOL, V51, P1025, DOI 10.1016/S0895-4356(98)00094-8
   Assal M, 2002, J BONE JOINT SURG AM, V84A, P161, DOI 10.2106/00004623-200202000-00001
   Beaton DE, 1996, J BONE JOINT SURG AM, V78A, P882, DOI 10.2106/00004623-199606000-00011
   Beaton DE, 1997, J CLIN EPIDEMIOL, V50, P79, DOI 10.1016/S0895-4356(96)00296-X
   Bessette L, 1998, MED CARE, V36, P491, DOI 10.1097/00005650-199804000-00005
   Boardman DL, 2000, J ARTHROPLASTY, V15, P200, DOI 10.1016/S0883-5403(00)90242-0
   BUDIMANMAK E, 1991, J CLIN EPIDEMIOL, V44, P561, DOI 10.1016/0895-4356(91)90220-4
   Bunnell S, 1928, J BONE JOINT SURG, V10, P1
   Button G, 2004, FOOT ANKLE INT, V25, P521, DOI 10.1177/107110070402500802
   Carmont MR, 2008, KNEE SURG SPORT TR A, V16, P199, DOI 10.1007/s00167-007-0411-z
   Ceccarelli F, 2009, EUR MUSCULOSKEL REV, V4, P83
   Ceccarelli F, 2007, CLIN ORTHOP RELAT R, P188, DOI 10.1097/BLO.0b013e3180396f07
   CONSTANT CR, 1987, CLIN ORTHOP RELAT R, P160
   Dawson J, 2012, ORTHOP TRAUMATOL-SUR, V98, P652, DOI 10.1016/j.otsr.2012.05.011
   Dawson J, 1998, J BONE JOINT SURG BR, V80B, P63, DOI 10.1302/0301-620X.80B1.7859
   Farrugia P, 2011, INJURY, V42, P276, DOI 10.1016/j.injury.2010.11.051
   Garrido Ignacio Martinez, 2010, Foot Ankle Surg, V16, P189, DOI 10.1016/j.fas.2009.10.014
   Ghanem E, 2010, J BONE JOINT SURG AM, V92A, P2445, DOI 10.2106/JBJS.I.00252
   Gorschewsky O, 1999, INJURY, V30, P315, DOI 10.1016/S0020-1383(99)00089-3
   Guyton GP, 2001, FOOT ANKLE INT, V22, P779, DOI 10.1177/107110070102201003
   HARRIS WH, 1969, J BONE JOINT SURG AM, VA 51, P737, DOI 10.2106/00004623-196951040-00012
   Henriquez H, 2012, CLIN ORTHOP RELAT R, V470, P998, DOI 10.1007/s11999-011-1830-1
   Hudak PL, 1996, AM J IND MED, V29, P602, DOI 10.1002/(SICI)1097-0274(199606)29:6<602::AID-AJIM4>3.0.CO;2-L
   Ibrahim Talal, 2007, J Foot Ankle Surg, V46, P65, DOI 10.1053/j.jfas.2006.12.002
   INSALL JN, 1989, CLIN ORTHOP RELAT R, P13
   Jung HG, 2008, FOOT ANKLE INT, V29, P803, DOI 10.3113/FAI.2008.0803
   KITAOKA HB, 1994, FOOT ANKLE INT, V15, P349, DOI 10.1177/107110079401500701
   Klassbo M, 2003, SCAND J RHEUMATOL, V32, P46, DOI 10.1080/03009740310000409
   KLEIN W, 1991, Chirurgia degli Organi di Movimento, V76, P223
   KOSANOVIC M, 1994, ARCH ORTHOP TRAUM SU, V113, P177, DOI 10.1007/BF00441827
   Lingard EA, 2001, J BONE JOINT SURG AM, V83A, P1856, DOI 10.2106/00004623-200112000-00014
   MA GWC, 1977, CLIN ORTHOP RELAT R, P247
   Malviya A, 2007, FOOT ANKLE INT, V28, P494, DOI 10.3113/FAI.2007.0494
   Mertl P, 1999, REV CHIR ORTHOP, V85, P277
   Morrey B., 2000, ELBOW ITS DISORDERS, V3, P74
   Reuling EMBP, 2012, ARCH ORTHOP TRAUM SU, V132, P249, DOI 10.1007/s00402-011-1424-0
   Robertsson O, 2001, J ARTHROPLASTY, V16, P476, DOI 10.1054/arth.2001.22395a
   Rocourt MHH, 2008, J SHOULDER ELB SURG, V17, P364, DOI 10.1016/j.jse.2007.06.024
   Rouvillain JL, 2010, ACTA ORTHOP BELG, V76, P237
   SANDERS R, 1993, CLIN ORTHOP RELAT R, P87
   SooHoo NF, 2006, FOOT ANKLE INT, V27, P930, DOI 10.1177/107110070602701111
   SooHoo NF, 2007, J ARTHROPLASTY, V22, P1168, DOI 10.1016/j.arth.2006.10.006
   SooHoo NF, 2003, FOOT ANKLE INT, V24, P50, DOI 10.1177/107110070302400108
   TEGNER Y, 1985, CLIN ORTHOP RELAT R, P43
   Wamper KE, 2010, ACTA ORTHOP, V81, P703, DOI 10.3109/17453674.2010.537808
   Wang Ting, 2012, Zhongguo Xiu Fu Chong Jian Wai Ke Za Zhi, V26, P814
   WARE JE, 1992, MED CARE, V30, P473, DOI 10.1097/00005650-199206000-00002
   Webb J, 2000, FOOT ANKLE INT, V21, P475, DOI 10.1177/107110070002100604
   Webb JM, 1999, J BONE JOINT SURG BR, V81B, P877, DOI 10.1302/0301-620X.81B5.9784
   Westphal T, 2004, ACTA ORTHOP SCAND, V75, P750, DOI 10.1080/00016470410004148
NR 50
TC 33
Z9 33
U1 0
U2 1
PU ELSEVIER SCIENCE INC
PI NEW YORK
PA 360 PARK AVE SOUTH, NEW YORK, NY 10010-1710 USA
SN 1067-2516
EI 1542-2224
J9 J FOOT ANKLE SURG
JI J. Foot Ankle Surg.
PD JAN-FEB
PY 2014
VL 53
IS 1
BP 16
EP 21
DI 10.1053/j.jfas.2013.09.005
PG 6
WC Orthopedics; Surgery
WE Science Citation Index Expanded (SCI-EXPANDED)
SC Orthopedics; Surgery
GA 282IN
UT WOS:000329164600006
PM 24239424
DA 2022-07-22
ER

PT J
AU Olsson, N
   Karlsson, J
   Eriksson, BI
   Brorsson, A
   Lundberg, M
   Silbernagel, KG
AF Olsson, N.
   Karlsson, J.
   Eriksson, B. I.
   Brorsson, A.
   Lundberg, M.
   Silbernagel, K. G.
TI Ability to perform a single heel-rise is significantly related to
   patient-reported outcome after Achilles tendon rupture
SO SCANDINAVIAN JOURNAL OF MEDICINE & SCIENCE IN SPORTS
LA English
DT Article
DE Achilles Tendon Total Rupture Score (ATRS); Foot and Ankle Outcome Score
   (FAOS); EQ-5D; kinesiophobia; calf muscle strength
ID NONOPERATIVE TREATMENT; WEIGHT-BEARING; MUSCLE; REPAIR; IMMOBILIZATION;
   MOBILIZATION; ENDURANCE; EXERCISE; SURGERY; SCORE
AB This study evaluated the short-term recovery of function after an acute Achilles tendon rupture, measured by a single-legged heel-rise test, with main emphasis on the relation to the patient-reported outcomes and fear of physical activity and movement (kinesiophobia). Eighty-one patients treated surgically or non-surgically with early active rehabilitation after Achilles tendon rupture were included in the study. Patient's ability to perform a single-legged heel-rise, physical activity level, patient-reported symptoms, general health, and kinesiophobia was evaluated 12 weeks after the injury. The heel-rise test showed that 40 out of 81 (49%) patients were unable to perform a single heel-rise 12 weeks after the injury. We found that patients who were able to perform a heel-rise were significantly younger, more often of male gender, reported a lesser degree of symptoms, and also had a higher degree of physical activity at 12 weeks. There was also a significant negative correlation between kinesiophobia and all the patient-reported outcomes and the physical activity level. The heel-rise ability appears to be an important early achievement and reflects the general level of healing, which influences patient-reported outcome and physical activity. Future treatment protocols focusing on regaining strength early after the injury therefore seem to be of great importance. Kinesiophobia needs to be addressed early during the rehabilitation process.
C1 [Olsson, N.; Karlsson, J.; Eriksson, B. I.; Brorsson, A.; Lundberg, M.] Univ Gothenburg, Sahlgrenska Univ Hosp, Dept Orthopaed, Inst Clin Sci,Sahlgrenska Acad, Molndal, Sweden.
   [Silbernagel, K. G.] Univ Sci, Dept Phys Therapy, Philadelphia, PA USA.
RP Olsson, N (通讯作者)，Orthoctr IFK Kliniken, Arvid Wallgrens Backe 4A, SE-41346 Gothenburg, Sweden.
EM nicklas.olsson@gu.se
RI Brorsson, Annelie/AAD-5986-2020
OI Lundberg, Mari/0000-0001-7123-0362; Gravare Silbernagel,
   Karin/0000-0001-7566-407X
FU Swedish National Centre for Research in Sports (CIF)
FX This study was supported by the Swedish National Centre for Research in
   Sports (CIF).
CR Aspenberg P, 2007, INT ORTHOP, V31, P783, DOI 10.1007/s00264-007-0398-6
   Bostick GP, 2010, J ORTHOP SPORT PHYS, V40, P345, DOI 10.2519/jospt.2010.3204
   Brooks R, 1996, HEALTH POLICY, V37, P53, DOI 10.1016/0168-8510(96)00822-6
   Burstrom K, 2001, QUAL LIFE RES, V10, P621, DOI 10.1023/A:1013171831202
   Costa ML, 2006, J BONE JOINT SURG BR, V88B, P69, DOI 10.1302/0301-620X.88B1.16549
   Eliasson P, 2012, J ORTHOP RES, V30, P274, DOI 10.1002/jor.21511
   GRIMBY G, 1986, ACTA MED SCAND, P233
   Jarvinen M, 1997, SCAND J MED SCI SPOR, V7, P86
   Kangas J, 2003, J TRAUMA, V54, P1171, DOI 10.1097/01.TA.0000047945.20863.A2
   Kannus P, 1997, SCAND J MED SCI SPOR, V7, P67
   Keating JF, 2011, J BONE JOINT SURG BR, V93B, P1071, DOI 10.1302/0301-620X.93B8.25998
   Kessler I, 1973, Hand, V5, P253, DOI 10.1016/0072-968X(73)90038-7
   Kjaer M, 2004, PHYSIOL REV, V84, P649, DOI 10.1152/physrev.00031.2003
   Kjaer M., 2005, Journal of Musculoskeletal & Neuronal Interactions, V5, P41
   Kvist J, 2005, KNEE SURG SPORT TR A, V13, P393, DOI 10.1007/s00167-004-0591-8
   Leppilahti J, 1996, ACTA ORTHOP SCAND, V67, P277, DOI 10.3109/17453679608994688
   Lundberg Mari, 2009, Physiotherapy Theory and Practice, V25, P495, DOI [10.1080/09593980802662160, 10.3109/09593980802662160]
   Lundberg Mari K. E., 2004, Physiotherapy Theory and Practice, V20, P121, DOI 10.1080/09593980490453002
   Maffulli N, 2003, AM J SPORT MED, V31, P692, DOI 10.1177/03635465030310051001
   Maffulli N, 1999, CLIN J SPORT MED, V9, P157, DOI 10.1097/00042752-199907000-00007
   Maffulli N, 2011, FOOT ANKLE INT, V32, P9, DOI 10.3113/FAI.2011.0009
   Magnusson SP, 2007, INT J EXP PATHOL, V88, P237, DOI 10.1111/j.1365-2613.2007.00551.x
   Miller BF, 2007, J APPL PHYSIOL, V102, P541, DOI 10.1152/japplphysiol.00797.2006
   Moller M, 2001, J BONE JOINT SURG BR, V83B, P843
   Moller M, 2005, KNEE SURG SPORT TR A, V13, P60, DOI 10.1007/s00167-003-0441-0
   Moller M, 2002, SCAND J MED SCI SPOR, V12, P9, DOI 10.1034/j.1600-0838.2002.120103.x
   Nilsson-Helander K, 2007, AM J SPORT MED, V35, P421, DOI 10.1177/0363546506294856
   Nilsson-Helander K, 2010, AM J SPORT MED, V38, P2186, DOI 10.1177/0363546510376052
   Olsson N, 2011, KNEE SURG SPORT TR A, V19, P1385, DOI 10.1007/s00167-011-1511-3
   Rantanen J, 1999, SCAND J MED SCI SPOR, V9, P57
   Roos EM, 2001, FOOT ANKLE INT, V22, P788, DOI 10.1177/107110070102201004
   Saxena A, 2011, J FOOT ANKLE SURG, V50, P37, DOI 10.1053/j.jfas.2010.10.008
   Silbernagel KG, 2010, KNEE SURG SPORT TR A, V18, P258, DOI 10.1007/s00167-009-0889-7
   SILFVERSKIOLD KL, 1994, J HAND SURG-AM, V19A, P53, DOI 10.1016/0363-5023(94)90224-0
   Suchak AA, 2008, J BONE JOINT SURG AM, V90A, P1876, DOI 10.2106/JBJS.G.01242
   Suchak AA, 2006, CLIN ORTHOP RELAT R, P216, DOI 10.1097/01.blo.0000203458.05135.74
   Twaddle BC, 2007, AM J SPORT MED, V35, P2033, DOI 10.1177/0363546507307503
   Uchiyama E, 2007, AM J SPORT MED, V35, P1739, DOI 10.1177/0363546507304492
   van Sterkenburg MN, 2008, ACHILLES TENDON RUPT, P108
   Weber M, 2003, AM J SPORT MED, V31, P685, DOI 10.1177/03635465030310050901
   Westh E, 2008, SCAND J MED SCI SPOR, V18, P23, DOI 10.1111/j.1600-0838.2007.00638.x
   WILLIAMS A, 1990, HEALTH POLICY, V16, P199
   Willits K, 2010, J BONE JOINT SURG AM, V92A, P2767, DOI 10.2106/JBJS.I.01401
NR 43
TC 49
Z9 51
U1 3
U2 15
PU WILEY
PI HOBOKEN
PA 111 RIVER ST, HOBOKEN 07030-5774, NJ USA
SN 0905-7188
EI 1600-0838
J9 SCAND J MED SCI SPOR
JI Scand. J. Med. Sci. Sports
PD FEB
PY 2014
VL 24
IS 1
BP 152
EP 158
DI 10.1111/j.1600-0838.2012.01497.x
PG 7
WC Sport Sciences
WE Science Citation Index Expanded (SCI-EXPANDED)
SC Sport Sciences
GA 292PU
UT WOS:000329915200026
PM 22716232
DA 2022-07-22
ER

PT J
AU Smigielski, R
AF Smigielski, Robert
TI Management of partial tears of the gastro-soleus complex
SO CLINICS IN SPORTS MEDICINE
LA English
DT Article
ID ACHILLES-TENDON RUPTURE; HUMAN TRICEPS SURAE; SPORTS; FORCE
AB Although Achilles tendon is the strongest tendon in the human body, it is one of the most commonly injured tendons. Most Achilles ruptures are associated with sports activity. Up to one-fourth of all Achilles tendon pathologies are partial ruptures. Increased recognizability of partial rupture of Achilles tendon is connected with the development of ultrasound and MRI techniques. Partial ruptures of Achilles tendon are a clinical demonstration of asymmetric loading of Achilles tendon caused by its special anatomic structure. To describe those partial injuries, the author created histologic and anatomic classification. In this article detailed treatment options has been proposed.
C1 Carolina Med Ctr, PL-01876 Warsaw, Poland.
RP Smigielski, R (通讯作者)，Carolina Med Ctr, Broniewskiego 89, PL-01876 Warsaw, Poland.
EM robert.smigielski@carolina.pl
CR AMENDOLA A, 2003, ACHILLES PATHOLOGY C, P31
   Arndt A, 1999, FOOT ANKLE INT, V20, P444, DOI 10.1177/107110079902000709
   Arndt A, 1999, FOOT ANKLE INT, V20, P450, DOI 10.1177/107110079902000710
   Arndt AN, 1998, CLIN BIOMECH, V13, P532, DOI 10.1016/S0268-0033(98)00032-1
   CETTI R, 1993, AM J SPORT MED, V21, P791, DOI 10.1177/036354659302100606
   CUMMINS EJ, 1946, SURG GYNECOL OBSTET, V83, P107
   CZYRNY Z, 2003, ACTA CLIN, V3, P216
   Denstad T F, 1979, Am J Sports Med, V7, P15, DOI 10.1177/036354657900700104
   Hartgerink P, 2001, RADIOLOGY, V220, P406, DOI 10.1148/radiology.220.2.r01au41406
   JOZSA L, 1989, AM J SPORT MED, V17, P338, DOI 10.1177/036354658901700305
   Kainberger F, 1997, EUR J RADIOL, V25, P209, DOI 10.1016/S0720-048X(97)00058-2
   KANNUS P, 1991, J BONE JOINT SURG AM, V73A, P1507, DOI 10.2106/00004623-199173100-00009
   Kannus P, 1997, SCAND J MED SCI SPOR, V7, P107
   Knobloch K, 2008, J FINN ORTHOP TRAUMA, V3, P18
   KONN K, 1967, HEFTE UNFALLHEILKD, V91, P255
   KRAHL H, 2003, BIOMECHANICAL FACTOR, P13
   LAGERGREN C, 1959, Acta Chir Scand, V116, P491
   LANG J, 1960, Z Anat Entwicklungsgesch, V122, P197, DOI 10.1007/BF00521798
   LIPENSKA M, 1979, Folia Morphologica (Prague), V27, P326
   Ljungqvist R., 1968, ACTA ORTHOP SCAND S, V39, P1, DOI 10.3109/ort.1968.39.suppl-113.01
   LYNN TA, 1966, J BONE JOINT SURG AM, VA 48, P268, DOI 10.2106/00004623-196648020-00005
   ORAVA S, 1980, EXERTION INJURIES DU, P1
   Paavola M, 2000, AM J SPORT MED, V28, P77, DOI 10.1177/03635465000280012501
   Renstrom P, 1991, SPORTS MED, P307
   Schepsis AA, 2002, AM J SPORT MED, V30, P287, DOI 10.1177/03635465020300022501
   SCHEPSIS AA, 1994, AM J SPORT MED, V22, P611, DOI 10.1177/036354659402200508
   SCHONBAUER HR, 1986, WIEN KLIN WSCHR S168, V98, P1
   SEGESSER B, 1995, ORTHOPADE, V24, P252
   SMIGIELSKI R, 2003, ACTA CLIN, V3, P225
   WILLIAMS JGP, 1986, SPORTS MED, V3, P114, DOI 10.2165/00007256-198603020-00003
NR 30
TC 22
Z9 22
U1 0
U2 7
PU W B SAUNDERS CO-ELSEVIER INC
PI PHILADELPHIA
PA 1600 JOHN F KENNEDY BOULEVARD, STE 1800, PHILADELPHIA, PA 19103-2899 USA
SN 0278-5919
J9 CLIN SPORT MED
JI Clin. Sports Med.
PD JAN
PY 2008
VL 27
IS 1
BP 219
EP +
DI 10.1016/j.csm.2007.10.005
PG 12
WC Sport Sciences
WE Science Citation Index Expanded (SCI-EXPANDED)
SC Sport Sciences
GA 265YP
UT WOS:000253398200013
PM 18206576
DA 2022-07-22
ER

PT J
AU Wood, VT
   Pinfildi, CE
   Neves, MAI
   Parizoto, NA
   Hochman, B
   Ferreira, LM
AF Wood, Viviane T.
   Pinfildi, Carlos E.
   Neves, Marco A. I.
   Parizoto, Nivaldo A.
   Hochman, Bernardo
   Ferreira, Lydia M.
TI Collagen Changes and Realignment Induced by Low-Level Laser Therapy and
   Low-Intensity Ultrasound in the Calcaneal Tendon
SO LASERS IN SURGERY AND MEDICINE
LA English
DT Article
DE achilles tendon; birefringence; diode laser; physical therapy;
   picrosirius red; tendon injuries; ultrasonography
ID MEDIAL COLLATERAL LIGAMENT; HUMAN ACHILLES-TENDON; TISSUE-REPAIR;
   ELECTRICAL-STIMULATION; III COLLAGEN; ULTRASTRUCTURAL MORPHOLOGY;
   IN-VITRO; RAT; RUPTURE; REGENERATION
AB Background and Objective: The treatment of calcaneal tendon injuries requires long-term rehabilitation Ultrasound (US) and low-level laser therapy (LLLT) are the most used and studied physical agents in the treatment of tendon injuries, however, only a few studies examined the effects of the combination of US and LLLT. Therefore, the purpose of this study was to investigate which treatment (the exclusive or combined use of US and LLLT) most effectively contribute to tendon healing.
   Study Design/Materials and Methods: This was a controlled laboratory study with 50 rats whose Achilles tendon was injured by direct trauma. The rats were randomly divided into five groups and treated for 5 consecutive days, as follows: group 1 (control) received no treatment; group 2 was treated with US alone, group 3 was treated with LLLT alone, group 4 was treated first with US followed by LLLT, and group 5 was treated first with LLLT followed by US On the sixth post-injury day, the tendons were removed and examined by polarized light microscopy. The organization of collagen fibers was assessed by birefringence measurements Picrosirius-stained sections were examined for the presence of types I and Ill collagen.
   Results: There was a significantly higher organization of collagen fibers in group 2 (US) than in the control group (P=0.03). The amount of type I collagen found in groups 2 (US), 3 (LLLT), and 5 (LLLT+US) was significantly higher than that in the control group (P <= 0.01), but no significant differences were found between treatment groups. There were no differences in the amount of type III collagen between groups.
   Conclusion: Ultrasound, LLLT, and the combined use of LLLT and US resulted in greater synthesis of type I collagen; US was also effective in increasing collagen organization in the early stages of the healing process Lasers Surg. Med. 42 559-565, 2010 (C) 2010 Wiley-Liss, Inc.
C1 [Wood, Viviane T.] Univ Fed Sao Paulo, Div Plast Surg, BR-04023002 Sao Paulo, Brazil.
   [Parizoto, Nivaldo A.] Univ Fed Sao Carlos, BR-13565905 Sao Carlos, SP, Brazil.
RP Wood, VT (通讯作者)，Univ Fed Sao Paulo, Div Plast Surg, Napoleao Barros 715,4 Andar, BR-04023002 Sao Paulo, Brazil.
RI Ferreira, Lydia Masako/B-7511-2012; Pinfildi, Carlos E/D-3966-2012
FU Division of Plastic Surgery of the Federal University of Sao Paulo
   (UNIFESP); Laboratory of Vitreous Materials (LaMaV); Department of
   Materials of the Federal University of Sao Carlos (UFSCar); Bioset
   Industria de Tecnologia Eletronica Ltda; Coordination for the
   Improvement of Higher Education Personnel (Capes)
FX We thank the Division of Plastic Surgery of the Federal University of
   Sao Paulo (UNIFESP), the Laboratory of Vitreous Materials (LaMaV),
   Department of Materials of the Federal University of Sao Carlos
   (UFSCar), the Bioset Industria de Tecnologia Eletronica Ltda, and the
   Coordination for the Improvement of Higher Education Personnel (Capes)
   for supporting this study
CR ARRUDA ERB, 2007, REV BRAS FISIOTER, V11, P283, DOI DOI 10.1590/S1413-35552007000400007
   BASFORD JR, 1989, LASER SURG MED, V9, P1, DOI 10.1002/lsm.1900090103
   Bossini PS, 2009, LASER MED SCI, V24, P209, DOI 10.1007/s10103-008-0551-5
   Carrinho PM, 2006, PHOTOMED LASER SURG, V24, P754, DOI 10.1089/pho.2006.24.754
   COOMBS RRH, 1980, J BONE JOINT SURG BR, V62, P258
   da Cunha A, 2001, ULTRASOUND MED BIOL, V27, P1691, DOI 10.1016/S0301-5629(01)00477-X
   de Oliveira RF, 2008, PHOTOMED LASER SURG, V26, P6, DOI 10.1089/pho.2007.2112
   Demir H, 2004, LASER SURG MED, V35, P84, DOI 10.1002/lsm.20046
   Draper David O., 1995, Journal of Athletic Training, V30, P304
   DYSON M, 1968, CLIN SCI, V35, P273
   Enwemeka CS, 2004, PHOTOMED LASER SURG, V22, P323, DOI 10.1089/1549541041797841
   ENWEMEKA CS, 1989, AM J PHYS MED REHAB, V68, P283, DOI 10.1097/00002060-198912000-00005
   Eriksen HA, 2002, J ORTHOPAED RES, V20, P1352, DOI 10.1016/S0736-0266(02)00064-5
   Fillipin LI, 2005, LASER SURG MED, V37, P293, DOI 10.1002/lsm.20225
   FRIEDMANN H, 1991, J PHOTOCH PHOTOBIO B, V11, P87, DOI 10.1016/1011-1344(91)80271-I
   Fung DTC, 2003, LASER SURG MED, V32, P286, DOI 10.1002/lsm.10161
   Fung DTC, 2002, LASER SURG MED, V31, P91, DOI 10.1002/lsm.10083
   GASQUE K.C.S., 2008, CIENC ODONTOL BRAS, V11, P58
   Greenough CG, 1996, J HAND SURG-BRIT EUR, V21B, P808, DOI 10.1016/S0266-7681(96)80198-7
   Gum SL, 1997, AM J PHYS MED REHAB, V76, P288, DOI 10.1097/00002060-199707000-00007
   Karu T, 1998, SCI LOW POWER LASER
   Koeke PU, 2005, ULTRASOUND MED BIOL, V31, P345, DOI 10.1016/j.ultrasmedbio.2004.12.005
   Maffulli N, 2000, AM J SPORT MED, V28, P499, DOI 10.1177/03635465000280040901
   NESSLER JP, 1987, CLIN ORTHOP RELAT R, P303
   Ng GYF, 2007, ULTRASOUND MED BIOL, V33, P1750, DOI 10.1016/j.ultrasmedbio.2007.05.019
   Oliveira FS, 2009, LASER SURG MED, V41, P271, DOI 10.1002/lsm.20760
   Pinfildi CE, 2009, PHOTOMED LASER SURG, V27, P337, DOI 10.1089/pho.2008.2295
   Pinfildi CE, 2005, LASER SURG MED, V37, P74, DOI 10.1002/lsm.20190
   Reddy GK, 1998, MED SCI SPORT EXER, V30, P794, DOI 10.1097/00005768-199806000-00003
   Riley G, 2008, NAT CLIN PRACT RHEUM, V4, P82, DOI 10.1038/ncprheum0700
   Salate ACB, 2005, PHOTOMED LASER SURG, V23, P470, DOI 10.1089/pho.2005.23.470
   Schindl A, 2000, J INVEST MED, V48, P312
   Tsai WC, 2006, J ORTHOP RES, V24, P1310, DOI 10.1002/jor.20130
   Vidal BD, 2003, MICRON, V34, P423, DOI 10.1016/S0968-4328(03)00039-8
   Yeung CK, 2006, J ORTHOP RES, V24, P193, DOI 10.1002/jor.20020
   Zantop T, 2003, ARCH ORTHOP TRAUM SU, V123, P501, DOI 10.1007/s00402-003-0491-2
NR 36
TC 35
Z9 37
U1 2
U2 15
PU WILEY
PI HOBOKEN
PA 111 RIVER ST, HOBOKEN 07030-5774, NJ USA
SN 0196-8092
EI 1096-9101
J9 LASER SURG MED
JI Lasers Surg. Med.
PD AUG
PY 2010
VL 42
IS 6
BP 559
EP 565
DI 10.1002/lsm.20932
PG 7
WC Dermatology; Surgery
WE Science Citation Index Expanded (SCI-EXPANDED)
SC Dermatology; Surgery
GA 639XC
UT WOS:000281009400015
PM 20662033
DA 2022-07-22
ER

PT J
AU Marican, MM
   Fook-Chong, SMC
   Rikhraj, IS
AF Marican, Mohd Mizan
   Fook-Chong, Stephanie Man Chung
   Rikhraj, Inderjeet Singh
TI Incidence of postoperative wound infections after open tendo Achilles
   repairs
SO SINGAPORE MEDICAL JOURNAL
LA English
DT Article
DE Achilles tendon rupture; risk factors; wound infection
ID SUTURE MATERIALS; SURGICAL REPAIR; PROPHYLAXIS; RUPTURE; ANGIOSOMES;
   VANCOMYCIN; RISK; MANAGEMENT; OUTCOMES; REDUCE
AB INTRODUCTION Tendo Achilles (TA), which is the confluence of the gastrocnemius and soleus muscles, is one of the most commonly injured tendons. The surgical repair of TA ruptures is associated with a significant risk of infection. This study examined several factors (i.e. gender, age, body mass index, history of diabetes mellitus, steroid use, acute or chronic TA injuries, type of surgical incision and type of sutures used) that may be associated with postoperative wound infection after open TA repair.
   METHODS This was a retrospective study involving 60 patients who underwent open TA repair over an 18-month period. Patients who had prior TA surgery or open TA injuries, or who needed soft tissues flaps were excluded.
   RESULTS Among the patients, 7 (11.7%) developed superficial wound infections that were successfully treated with oral antibiotics, while 3 (5.0%) developed deep wound infections that required at least one debridement procedure. No significant association was found between the risk of postoperative wound infection and gender, age, the presence of diabetes mellitus, acute or chronic ruptures, site of surgical incision and type of deep or superficial sutures used.
   CONCLUSION While diabetes mellitus and age did not appear to be associated with postoperative wound infections after open TA repair, obese patients were found to be two times more likely to develop a wound infection than normal-weight patients. The incidence of superficial wound infections in this study was similar to previously published results (11.7% vs. 8.2%-14.6%), but the incidence of deep infections was higher (5% vs. 1%-2%).
C1 [Marican, Mohd Mizan; Rikhraj, Inderjeet Singh] Singapore Gen Hosp, Dept Orthopaed Surg, Singapore 169856, Singapore.
   [Fook-Chong, Stephanie Man Chung] Singapore Gen Hosp, Div Res, Singapore 169856, Singapore.
RP Marican, MM (通讯作者)，Singapore Gen Hosp, Dept Orthopaed Surg, 20 Coll Rd,Acad Level 4, Singapore 169856, Singapore.
EM mohd.mizan.marican@singhealth.com.sg
CR Alexander JW, 2011, ANN SURG, V253, P1082, DOI 10.1097/SLA.0b013e31821175f8
   ALEXANDER JW, 1967, ANN SURG, V165, P192, DOI 10.1097/00000658-196702000-00005
   Anderson DJ, 2014, INFECT CONT HOSP EP, V35, P605, DOI [10.1017/S0899823X00193869, 10.1086/676022]
   Attinger CE, 2006, PLAST RECONSTR SURG, V117, p261S, DOI 10.1097/01.prs.0000222582.84385.54
   Bhattacharyya M, 2009, INT ORTHOP, V33, P151, DOI 10.1007/s00264-008-0564-5
   Bratzler DW, 2013, SURG INFECT, V14, P73, DOI 10.1089/sur.2013.9999
   Bull AL, 2012, ANN SURG, V256, P1089, DOI 10.1097/SLA.0b013e31825fa398
   Chiodo CP, 2010, J BONE JOINT SURG AM, V92A, P2466
   CHU CC, 1984, AM J SURG, V147, P197, DOI 10.1016/0002-9610(84)90088-6
   Costa ML, 2006, J BONE JOINT SURG BR, V88B, P69, DOI 10.1302/0301-620X.88B1.16549
   Edmiston CE, 2006, J AM COLL SURGEONS, V203, P481, DOI 10.1016/j.jamcollsurg.2006.06.026
   Finkelstein R, 2002, J THORAC CARDIOV SUR, V123, P326, DOI 10.1067/mtc.2002.119698
   Garrick James G, 2012, Clin J Sport Med, V22, P379, DOI 10.1097/JSM.0b013e3182603905
   Gilbert Peter, 2002, Surg Infect (Larchmt), V3 Suppl 1, pS55, DOI 10.1089/sur.2002.3.s1-55
   Gupta K, 2011, INFECT CONT HOSP EP, V32, P791, DOI 10.1086/660362
   Highlander Peter, 2011, Foot Ankle Spec, V4, P361, DOI 10.1177/1938640011418488
   Kanj WW, 2013, ORTHOPEDICS, V36, P138, DOI 10.3928/01477447-20130122-10
   Khan RJK, 2005, J BONE JOINT SURG AM, V87A, P2202, DOI 10.2106/JBJS.D.03049
   Ming Xintian, 2007, Surg Infect (Larchmt), V8, P209, DOI 10.1089/sur.2006.004
   Ming XT, 2008, SURG INFECT, V9, P451, DOI 10.1089/sur.2007.061
   Nilsson-Helander K, 2010, AM J SPORT MED, V38, P2186, DOI 10.1177/0363546510376052
   Olsson N, 2013, AM J SPORT MED, V41, P2867, DOI 10.1177/0363546513503282
   OSTERBERG B, 1983, ACTA CHIR SCAND, V149, P663
   Pajala A, 2002, J BONE JOINT SURG AM, V84A, P2016, DOI 10.2106/00004623-200211000-00017
   SHARP WV, 1982, SURGERY, V91, P61
   Speck M, 1998, AM J SPORT MED, V26, P789, DOI 10.1177/03635465980260060901
   Tallon C, 2001, MED SCI SPORT EXER, V33, P1983, DOI 10.1097/00005768-200112000-00002
   TAYLOR GI, 1987, BRIT J PLAST SURG, V40, P113, DOI 10.1016/0007-1226(87)90185-8
   Taylor GI, 1998, PLAST RECONSTR SURG, V102, P599, DOI 10.1097/00006534-199809030-00001
   Thevendran G, 2013, Musculoskelet Surg, V97, P9, DOI 10.1007/s12306-013-0251-6
   van der Linden PD, 2003, ARCH INTERN MED, V163, P1801, DOI 10.1001/archinte.163.15.1801
   VARMA S, 1974, J SURG RES, V17, P165, DOI 10.1016/0022-4804(74)90103-6
   Wallace RGH, 2011, J BONE JOINT SURG BR, V93B, P1362, DOI 10.1302/0301-620X.93B10.26187
   Wong J, 2002, AM J SPORT MED, V30, P565, DOI 10.1177/03635465020300041701
   Yepes H, 2010, J BONE JOINT SURG AM, V92A, P1215, DOI 10.2106/JBJS.I.00743
   Zgonis Thomas, 2004, J Foot Ankle Surg, V43, P97, DOI 10.1053/j.jfas.2004.01.003
   Zhao HM, 2011, CHINESE MED J-PEKING, V124, P4050, DOI 10.3760/cma.j.issn.0366-6999.2011.23.038
NR 37
TC 11
Z9 15
U1 0
U2 3
PU SINGAPORE MEDICAL ASSOC
PI SINGAPORE
PA 2985 JALAN BUKIT MERAH, #02-2C, SMF BUILDING, SINGAPORE, SINGAPORE
SN 0037-5675
J9 SINGAP MED J
JI Singap. Med. J.
PD OCT
PY 2015
VL 56
IS 10
BP 549
EP 554
PG 6
WC Medicine, General & Internal
WE Science Citation Index Expanded (SCI-EXPANDED)
SC General & Internal Medicine
GA CY8GK
UT WOS:000366646600004
PM 26512146
OA Green Published, Bronze
DA 2022-07-22
ER

PT J
AU Aibinder, WR
   Patel, A
   Arnouk, J
   El-Gendi, H
   Korshunov, Y
   Mitgang, J
   Uribe, J
AF Aibinder, William R.
   Patel, Ashish
   Arnouk, Johnny
   El-Gendi, Hebah
   Korshunov, Yevgeniy
   Mitgang, Joshua
   Uribe, Jaime
TI The Rate of Sural Nerve Violation Using the Achillon Device: A Cadaveric
   Study
SO FOOT & ANKLE INTERNATIONAL
LA English
DT Article
DE Achillon; Achilles tendon rupture; sural nerve; limited open repair
ID NON-SURGICAL TREATMENT; TENDON RUPTURE; OPEN REPAIR; NONOPERATIVE
   TREATMENT
AB Background: Management of acute Achilles tendon rupture remains controversial. Open repair results in lower rerupture rates but is associated with complications from wound healing. Percutaneous and limited open repairs minimize soft tissue complications; however, there is a theoretical increased risk of sural nerve injury. We evaluated the risk of sural nerve violation with the Achillon limited open repair device in cadavers.
   Methods: This cadaveric study consisted of 2 parts. The first part evaluated the frequency and location of sural nerve violation using the standard Achillon protocol in 18 intact embalmed left lower limbs. The second part involved device manipulation with 30 degrees of either internal or external rotation in 13 paired, intact embalmed lower limbs.
   Results: In the first part, 5 of 18 specimens had at least 1 suture passing through the sural nerve. Eight of the 54 needle passes (14.8%) directly pierced the substance of the sural nerve. With internal rotation, 8 of 13 specimens had at least 1 violation of the nerve. Ten of the 39 needle passes (25.6%) directly pierced the substance of the sural nerve. With external rotation, no nerves were violated, significantly decreasing the risk of sural nerve violation versus neutral (P = .038) and internal rotation (P = .001). When sural nerve violation was evaluated in regard to instrument outlet, there was a higher risk in the proximal needle passer (outlet no. 3) compared with the distal needle passer (outlet no. 1) with both neutral testing and internal rotation (P = .027).
   Conclusion: This study demonstrated the potential risk for sural nerve injury using the Achillon device.
   Clinical Relevance: External rotation of the Achillon decreased the rate of sural nerve violation. This may optimize its use in Achilles tendon repairs.
C1 [Aibinder, William R.; Patel, Ashish; Arnouk, Johnny; El-Gendi, Hebah; Korshunov, Yevgeniy; Mitgang, Joshua; Uribe, Jaime] Suny Downstate Med Ctr, Dept Orthopaed Surg & Rehabil Med, Brooklyn, NY 11203 USA.
RP Aibinder, WR (通讯作者)，Suny Downstate Med Ctr, Dept Orthopaed Surg & Rehabil Med, 450 Clarkson Ave,Box 30, Brooklyn, NY 11203 USA.
EM william.aibinder@downstate.edu
RI Aibinder, William/AAC-8590-2019
OI Aibinder, William/0000-0001-7620-426X
CR ARACIL J, 1992, FOOT ANKLE, V13, P350, DOI 10.1177/107110079201300610
   Assal M, 2002, J BONE JOINT SURG AM, V84A, P161, DOI 10.2106/00004623-200202000-00001
   Bhandari M, 2002, CLIN ORTHOP RELAT R, P190
   Bruggeman NB, 2004, CLIN ORTHOP RELAT R, P63, DOI 10.1097/01.blo.0000144475.05543.e7
   Calder JDF, 2006, FOOT ANKLE INT, V27, P93, DOI 10.1177/107110070602700204
   Ceccarelli F, 2007, CLIN ORTHOP RELAT R, P188, DOI 10.1097/BLO.0b013e3180396f07
   CETTI R, 1993, AM J SPORT MED, V21, P791, DOI 10.1177/036354659302100606
   Cretnik A, 2004, WIEN KLIN WOCHENSCHR, V116, P33
   FITZGIBBONS RE, 1993, AM J SPORT MED, V21, P724, DOI 10.1177/036354659302100516
   GILLIES H, 1970, J BONE JOINT SURG AM, VA 52, P337, DOI 10.2106/00004623-197052020-00015
   Haji A, 2004, FOOT ANKLE INT, V25, P215, DOI 10.1177/107110070402500404
   HATTRUP SJ, 1985, FOOT ANKLE, V6, P34, DOI 10.1177/107110078500600107
   INGLIS AE, 1981, CLIN ORTHOP RELAT R, V156, P160
   Jarvinen Tero A H, 2005, Foot Ankle Clin, V10, P255, DOI 10.1016/j.fcl.2005.01.013
   Jung HG, 2008, FOOT ANKLE INT, V29, P803, DOI 10.3113/FAI.2008.0803
   KAKIUCHI M, 1995, J BONE JOINT SURG BR, V77B, P60, DOI 10.1302/0301-620X.77B1.7822398
   Keating JF, 2011, J BONE JOINT SURG BR, V93B, P1071, DOI 10.1302/0301-620X.93B8.25998
   KELLAM JF, 1985, CLIN ORTHOP RELAT R, V201, P80
   KLEIN W, 1991, Chirurgia degli Organi di Movimento, V76, P223
   LEA RB, 1972, J BONE JOINT SURG AM, VA 54, P1398, DOI 10.2106/00004623-197254070-00002
   Leppilahti J, 1998, SPORTS MED, V25, P79, DOI 10.2165/00007256-199825020-00002
   Leppilahti J, 1996, ACTA ORTHOP SCAND, V67, P277, DOI 10.3109/17453679608994688
   Lim J, 2001, FOOT ANKLE INT, V22, P559, DOI 10.1177/107110070102200705
   MA GWC, 1977, CLIN ORTHOP RELAT R, P247
   Maffulli N, 1999, J BONE JOINT SURG AM, V81A, P1019, DOI 10.2106/00004623-199907000-00017
   Maffulli N, 1999, CLIN J SPORT MED, V9, P157, DOI 10.1097/00042752-199907000-00007
   NISTOR L, 1981, J BONE JOINT SURG AM, V63, P394, DOI 10.2106/00004623-198163030-00012
   Nyyssonen T, 2008, SCAND J SURG, V97, P272, DOI 10.1177/145749690809700312
   Poynton AR, 2001, FOOT ANKLE INT, V22, P572, DOI 10.1177/107110070102200707
   Rebeccato A, 2001, J Foot Ankle Surg, V40, P188
   Shields C L Jr, 1978, Am J Sports Med, V6, P369, DOI 10.1177/036354657800600610
   Waterston SW, 1997, BRIT J SPORT MED, V31, P285, DOI 10.1136/bjsm.31.4.285
   Webb J, 2000, FOOT ANKLE INT, V21, P475, DOI 10.1177/107110070002100604
   WILLS CA, 1986, CLIN ORTHOP RELAT R, P156
NR 34
TC 28
Z9 35
U1 0
U2 2
PU SAGE PUBLICATIONS INC
PI THOUSAND OAKS
PA 2455 TELLER RD, THOUSAND OAKS, CA 91320 USA
SN 1071-1007
EI 1944-7876
J9 FOOT ANKLE INT
JI Foot Ankle Int.
PD JUN
PY 2013
VL 34
IS 6
BP 870
EP 875
DI 10.1177/1071100712473097
PG 6
WC Orthopedics
WE Science Citation Index Expanded (SCI-EXPANDED)
SC Orthopedics
GA 298FI
UT WOS:000330309100015
PM 23696186
DA 2022-07-22
ER

PT J
AU Liu, YS
   Peng, YB
   Fang, Y
   Yao, M
   Redmond, RW
   Ni, T
AF Liu, Yushu
   Peng, Yinbo
   Fang, Yong
   Yao, Min
   Redmond, Robert W.
   Ni, Tao
TI No midterm advantages in the middle term using small intestinal
   submucosa and human amniotic membrane in Achilles tendon transverse
   tenotomy
SO JOURNAL OF ORTHOPAEDIC SURGERY AND RESEARCH
LA English
DT Article
DE Small intestinal submucosa; Human amniotic membrane; Achilles tendon
   injury
ID NONOPERATIVE MANAGEMENT; REPAIR; RUPTURES; MODEL
AB Background: The study was aimed to compare the effects of small intestinal submucosa (SIS) and human amniotic membrane (HAM) on Achilles tendon healing.
   Methods: A total of 48 New Zealand white rabbits were divided into two groups. A full-thickness transverse tenotomy was made at the right leg of the rabbits. Then, the laceration site was wrapped with HAM (P/A group) or SIS (P/S group). The ultimate stress (US) and Young's modulus (E) of the tendons were detected for biomechanical analysis. Histological evaluation was performed using hematoxylin and eosin, immunohistochemical, and immunofluorescent stain. Expression of collagen I was detected by western blot analysis, and levels of inflammatory cytokines IL-1 beta,IL-6, and TNF-alpha were measured. Finally, adhesion formation was evaluated.
   Results: There were no significant differences in filamentous adhesion, cross-sectional areas of the laceration sites, levels of inflammatory response, and collagen type I expression between the P/A and P/S groups (p > 0.05). Compared with the P/A group, the US and E values were significantly higher in the P/S group at day 7 (p < 0.05) and at day 14 (p < 0.05). In addition, vascularity was significantly higher in the P/S group than that in the P/A group at day 3 (p < 0.05), day 7 (p < 0.01), and day 9 (p < 0.05).
   Conclusions: SIS showed superior biomechanical properties and neovascularization over HAM in treatment of Achilles tendon injury in the early stage of healing.
C1 [Liu, Yushu; Peng, Yinbo; Fang, Yong; Yao, Min; Ni, Tao] Shanghai Jiao Tong Univ, Sch Med, Shanghai Peoples Hosp 9, Dept Burns & Plast Surg, Mohe Rd 280, Shanghai 201900, Peoples R China.
   [Yao, Min; Redmond, Robert W.] Harvard Med Sch, Massachusetts Gen Hosp, Wellman Ctr Photomed, Boston, MA 02114 USA.
RP Ni, T (通讯作者)，Shanghai Jiao Tong Univ, Sch Med, Shanghai Peoples Hosp 9, Dept Burns & Plast Surg, Mohe Rd 280, Shanghai 201900, Peoples R China.
EM nitao6666@yeah.net
FU Science and Technology Commission of Shanghai Municipality
   [134119b1300]; National Natural Science Fund for Young Scholars of China
   [81401585]
FX This work was supported by Science and Technology Commission of Shanghai
   Municipality (134119b1300) and the National Natural Science Fund for
   Young Scholars of China (No. 81401585).
CR Assal M, 2002, J BONE JOINT SURG AM, V84A, P161, DOI 10.2106/00004623-200202000-00001
   Badylak S, 1999, CLIN ORTHOP RELAT R, pS333, DOI 10.1097/00003086-199910001-00032
   Badylak S, J SURG RES
   BADYLAK SF, 1995, J BIOMED MATER RES, V29, P977, DOI 10.1002/jbm.820290809
   BENNETT NT, 1993, AM J SURG, V165, P728, DOI 10.1016/S0002-9610(05)80797-4
   CAPLAN AI, 1994, CLIN PLAST SURG, V21, P429
   Chang J, 2000, PLAST RECONSTR SURG, V105, P148, DOI 10.1097/00006534-200001000-00025
   Cooper LJ, 2005, CORNEA, V24, P722, DOI 10.1097/01.ico.0000154237.49112.29
   Dejardin LM, 2001, AM J SPORT MED, V29, P175, DOI 10.1177/03635465010290021001
   Gilbert TW, 2007, J BONE JOINT SURG AM, V89A, P621, DOI 10.2106/JBJS.F.00742
   Gomes Jose A P, 2005, Curr Opin Ophthalmol, V16, P233, DOI 10.1097/01.icu.0000172827.31985.3a
   Kocher MS, 2002, AM J SPORT MED, V30, P783, DOI 10.1177/03635465020300060501
   Maffulli N., 1999, J BONE JOINT SURG, V75, P751
   Moller A, 1996, ACTA ORTHOP SCAND, V67, P479, DOI 10.3109/17453679608996672
   Ozgenel GY, 2004, J BONE JOINT SURG BR, V86B, P301, DOI 10.1302/0301-620X.86B2.14435
   Rippstein Pascal F, 2002, Foot Ankle Clin, V7, P611, DOI 10.1016/S1083-7515(02)00040-2
   Suchak AA, 2005, FOOT ANKLE INT, V26, P932, DOI 10.1177/107110070502601106
   Suckow MA, 2007, J MATER SCI-MATER M, V18, P1105, DOI 10.1007/s10856-007-0158-6
   Winter E, 1998, ARCH ORTHOP TRAUM SU, V117, P364, DOI 10.1007/s004020050267
   Wong J, 2002, AM J SPORT MED, V30, P565, DOI 10.1177/03635465020300041701
   Yang JJ, 2010, TISSUE ENG REGEN MED, V7, P323
   Yun TM, 2009, BIOMATERIALS, V30, P3234
   Zalavras CG, J SHOULDER ELBOW SUR
NR 23
TC 4
Z9 4
U1 0
U2 4
PU BIOMED CENTRAL LTD
PI LONDON
PA 236 GRAYS INN RD, FLOOR 6, LONDON WC1X 8HL, ENGLAND
SN 1749-799X
J9 J ORTHOP SURG RES
JI J. Orthop. Surg. Res.
PD NOV 24
PY 2016
VL 11
AR 125
DI 10.1186/s13018-016-0463-1
PG 8
WC Orthopedics
WE Science Citation Index Expanded (SCI-EXPANDED)
SC Orthopedics
GA FH6XC
UT WOS:000411322900001
PM 27881176
OA Green Published, gold
DA 2022-07-22
ER

PT J
AU Shalabi, A
   Kristoffersen-Wiberg, M
   Aspelin, P
   Movin, T
AF Shalabi, A
   Kristoffersen-Wiberg, M
   Aspelin, P
   Movin, T
TI MR evaluation of chronic achilles tendinosis - A longitudinal study of
   15 patients preoperatively and two years postoperatively
SO ACTA RADIOLOGICA
LA English
DT Article
DE achilles tendon, tendinitis; athletic injuries; prospective studies; MR
   imaging, gadolinium
ID MAGNETIC-RESONANCE; SURGICAL-TREATMENT; TENDON RUPTURES;
   ULTRASONOGRAPHY; ACHILLODYNIA; TENDINITIS; ULTRASOUND; ETIOLOGY; BIOPSY;
   TIME
AB Purpose: To evaluate surgically treated patients with chronic Achilles tendinosis by MR.
   Material and Methods: Gd-contrast-enhanced (CME) T1-, precontrast T1-, PD- and T2-weighted images were obtained preoperatively and 2 years following surgical treatment on 15 middle-aged patients with severe symptoms of chronic Achilles tendinosis. MR evaluation included the depiction of intratendinous signal alterations and their volume, and also measurement of tendon diameter. A questionnaire and clinical examination evaluated the clinical outcome.
   Results: The most sensitive sequence to depict an intratendinous lesion was the CME T1-WI. There was marked regress of the estimated volume of the intratendinous signal alteration from a median of 1.2 cm(3) preoperatively to 0.0 cm(3) postoperatively on CME T1-WI. CME T1-WI showed a regress in intratendinous signal abnormality from 13 out of 15 patients preoperatively to 4 of 15 patients 2 years postoperatively. The a.p. dimension was 9 mm at both MR occasions. The clinical outcome was excellent in 8, good in 5, fair in 1 and poor in 1 patient.
   Conclusion: Surgical treatment of chronic Achilles tendinosis and its healing resulted in a decrease or elimination of the intratendinous signal alteration correlating to an improved clinical outcome 2 years postoperatively.
C1 Huddinge Univ Hosp, Karolinska Inst, Dept Radiol, SE-14186 Huddinge, Sweden.
   Huddinge Univ Hosp, Karolinska Inst, Dept Orthoped Surg, SE-14186 Huddinge, Sweden.
RP Shalabi, A (通讯作者)，Huddinge Univ Hosp, Karolinska Inst, Dept Radiol, SE-14186 Huddinge, Sweden.
OI Kristoffersen Wiberg, Maria/0000-0001-6577-8217
CR Archambault JM, 1998, J CLIN ULTRASOUND, V26, P335, DOI 10.1002/(SICI)1097-0096(199809)26:7<335::AID-JCU1>3.0.CO;2-A
   Astrom M, 1996, SKELETAL RADIOL, V25, P615, DOI 10.1007/s002560050146
   ASTROM M, 1995, CLIN ORTHOP RELAT R, P151
   Bashir A, 1996, MAGNET RESON MED, V36, P665, DOI 10.1002/mrm.1910360504
   CLEMENT DB, 1984, AM J SPORT MED, V12, P179, DOI 10.1177/036354658401200301
   CURWIN S, 1984, TENDINITIS ETIOLOGY
   James S L, 1978, Am J Sports Med, V6, P40, DOI 10.1177/036354657800600202
   Karjalainen PT, 1996, ACTA RADIOL, V37, P639
   Karjalainen PT, 1997, AM J SPORT MED, V25, P164, DOI 10.1177/036354659702500204
   Karjalainen PT, 2000, AM J ROENTGENOL, V175, P251, DOI 10.2214/ajr.175.1.1750251
   Khan KM, 1996, RADIOLOGY, V200, P821, DOI 10.1148/radiology.200.3.8756939
   KOBLIK PD, 1993, INVEST RADIOL, V28, P1095, DOI 10.1097/00004424-199312000-00003
   KVIST H, 1980, J BONE JOINT SURG BR, V62, P353, DOI 10.1302/0301-620X.62B3.7410468
   KVIST M, 1991, ACHILLES TENDON OVER
   LEADBETTER WB, 1992, CLIN SPORT MED, V11, P679
   Maffulli N, 1998, ARTHROSCOPY, V14, P840, DOI 10.1016/S0749-8063(98)70021-0
   Matles A L, 1975, Bull Hosp Joint Dis, V36, P48
   Movin T, 1998, FOOT ANKLE INT, V19, P311, DOI 10.1177/107110079801900508
   Movin T, 1997, SCAND J MED SCI SPOR, V7, P244
   Movin T, 1997, ACTA ORTHOP SCAND, V68, P170, DOI 10.3109/17453679709004002
   MOVIN T, 1998, ASPECTS AETIOLOGY PA
   NELEN G, 1989, AM J SPORT MED, V17, P754, DOI 10.1177/036354658901700605
   NEUHOLD A, 1992, EUR J RADIOL, V14, P213, DOI 10.1016/0720-048X(92)90090-V
   REIFF DB, 1995, SKELETAL RADIOL, V24, P123
   Rolf C, 1997, FOOT ANKLE INT, V18, P565, DOI 10.1177/107110079701800906
   Rominger MB, 1998, ROFO-FORTSCHR RONTG, V168, P27, DOI 10.1055/s-2007-1015178
   Seddio C, 1997, Radiol Med, V94, P47
   SIMMONDS F A, 1957, Practitioner, V179, P56
   Soila K, 1999, AM J ROENTGENOL, V173, P323, DOI 10.2214/ajr.173.2.10430128
   TEITZ CC, 1997, J BONE JOINT SURG AM, V46, P569
NR 30
TC 43
Z9 43
U1 0
U2 6
PU MUNKSGAARD INT PUBL LTD
PI COPENHAGEN
PA 35 NORRE SOGADE, PO BOX 2148, DK-1016 COPENHAGEN, DENMARK
SN 0284-1851
J9 ACTA RADIOL
JI Acta Radiol.
PD MAY
PY 2001
VL 42
IS 3
BP 269
EP 276
DI 10.1080/028418501127346819
PG 8
WC Radiology, Nuclear Medicine & Medical Imaging
WE Science Citation Index Expanded (SCI-EXPANDED)
SC Radiology, Nuclear Medicine & Medical Imaging
GA 435WZ
UT WOS:000168906300004
PM 11350284
DA 2022-07-22
ER

PT J
AU Schipper, O
   Cohen, B
AF Schipper, Oliver
   Cohen, Bruce
TI The Acute Injury of the Achilles Surgical Options (Open Treatment, and,
   Minimally Invasive Surgery)
SO FOOT AND ANKLE CLINICS
LA English
DT Article
DE Achilles rupture; Achilles repair; Achilles tendinitis
ID TENDON RUPTURES; OPEN REPAIR; NONOPERATIVE TREATMENT; EPIDEMIOLOGY;
   MULTICENTER; OUTCOMES
AB Achilles tendon rupture is a common lower extremity injury seen in the active population. Although rerupture rates have improved with nonoperative functional management, surgical treatment is still preferred by the authors. Minimally invasive techniques allow optimal Achilles tendon rupture apposition and tensioning, with a reduced risk of soft tissue complications associated with the traditional open repair.
C1 [Schipper, Oliver; Cohen, Bruce] OrthoCarolina Foot & Ankle Inst, 2001 Vail Ave,Suite 200B, Charlotte, NC 28207 USA.
   [Schipper, Oliver] Anderson Orthopaed Clin, 2445 Army Navy Dr, Arlington, VA 22206 USA.
RP Schipper, O (通讯作者)，Anderson Orthopaed Clin, 2445 Army Navy Dr, Arlington, VA 22206 USA.
EM o.schipper@gmail.com
CR Ahmad J, 2013, FOOT ANKLE INT, V34, P1074, DOI 10.1177/1071100713483115
   Assal M, 2002, J BONE JOINT SURG AM, V84A, P161, DOI 10.2106/00004623-200202000-00001
   Bhandari M, 2002, CLIN ORTHOP RELAT R, P190
   Cretnik A, 2005, AM J SPORT MED, V33, P1369, DOI 10.1177/0363546504271501
   Cretnik A, 2000, MED SCI SPORT EXER, V32, P16
   Griffin MJ, 2017, FOOT ANKLE INT, V38, P36, DOI 10.1177/1071100716669983
   Gwynne-Jones DP, 2011, FOOT ANKLE INT, V32, P337, DOI 10.3113/FAI.2011.0337
   Houshian S, 1998, INJURY, V29, P651, DOI 10.1016/S0020-1383(98)00147-8
   Hsu AR, 2015, FOOT ANKLE INT, V36, P1279, DOI 10.1177/1071100715589632
   Huh J, 2016, FOOT ANKLE INT, V37, P596, DOI 10.1177/1071100716629778
   Jaakkola JI, 2000, FOOT ANKLE INT, V21, P14, DOI 10.1177/107110070002100103
   KAKIUCHI M, 1995, J BONE JOINT SURG BR, V77B, P60, DOI 10.1302/0301-620X.77B1.7822398
   Labib SA, 2009, FOOT ANKLE INT, V30, P410, DOI 10.3113/FAI.2009.0410
   Lim J, 2001, FOOT ANKLE INT, V22, P559, DOI 10.1177/107110070102200705
   MA GWC, 1977, CLIN ORTHOP RELAT R, P247
   Maffulli N, 1998, AM J SPORT MED, V26, P266, DOI 10.1177/03635465980260021801
   Maffulli N, 1999, CLIN J SPORT MED, V9, P157, DOI 10.1097/00042752-199907000-00007
   McWilliam JR, 2016, FOOT ANKLE INT, V37, P794, DOI 10.1177/1071100716653373
   Willits K, 2010, J BONE JOINT SURG AM, V92A, P2767, DOI 10.2106/JBJS.I.01401
   Yepes H, 2010, J BONE JOINT SURG AM, V92A, P1215, DOI 10.2106/JBJS.I.00743
NR 20
TC 7
Z9 8
U1 1
U2 6
PU W B SAUNDERS CO-ELSEVIER INC
PI PHILADELPHIA
PA 1600 JOHN F KENNEDY BOULEVARD, STE 1800, PHILADELPHIA, PA 19103-2899 USA
SN 1083-7515
EI 1558-1934
J9 FOOT ANKLE CLIN
JI Foot Ankle Clin.
PD DEC
PY 2017
VL 22
IS 4
BP 689
EP +
DI 10.1016/j.fcl.2017.07.003
PG 27
WC Orthopedics
WE Science Citation Index Expanded (SCI-EXPANDED)
SC Orthopedics
GA FN7MO
UT WOS:000416202500005
PM 29078823
DA 2022-07-22
ER

PT J
AU Kosaka, T
   Yamamoto, K
AF Kosaka, T.
   Yamamoto, K.
TI Long-term Effects of Chronic Achilles Tendon Rupture Treatment, Using
   Reconstruction with Peroneus Brevis Transfer, on Sports Activities
SO WEST INDIAN MEDICAL JOURNAL
LA English
DT Article
DE Chronic Achilles tendon rupture; long-term effects; peroneus brevis
   transfer; sports activities
ID FLAP
AB Objective: This study reports long-term effects of chronic Achilles tendon rupture treatment, using reconstruction with peroneus brevis transfer (PBT), on sports activities based on an approximate 10-year follow-up study.
   Methods: Twenty patients (6 women and 14 men; mean age, 43 +/- 12.85 years at the time of operation) underwent chronic Achilles tendon repair with an average follow-up of 164.05 +/- 5.07 months. Seven were involved in competitive sports, 10 participated in recreational activities and three were not involved in any sporting activities. All patients were Asians. Results were assessed using Cybex strength testing and the American Othopaedic Foot and Ankle Society (AOFAS) Score, the muscle manual test (MMT), sports activities and comprehensive satisfaction assessment.
   Results: Cybex strength testing resulted in an average gain of 87.05 +/- 14.83% in dorsiflexion strength (range 65-110%) and 98.05 +/- 9.02% in plantar flexion strength (range 85%-120%). The AOFAS score average was 86.9 +/- 7.27. There were no postoperative re-ruptures, no recurrences and no wound complications. Plantar flexion strength and the AOFAS score were negatively correlated with the age at the time of operation (r = -0.566, r = -0.669, respectively). Seventeen patients (85%) were level five of MMT in eversion strength. Following treatment, six patients (30%) returned to competitive sports, while 10 (50%) who, prior to the injury and surgery, were involved in recreational activities, returned to similar activities. The relatively younger group tended to continue sport activities as competitive athletes (p < 0.05). Significant differences were observed in age at the operation between non-satisfaction group and excellent group (p < 0.05). The under 40-year age group tended to show a poor value.
   Conclusion: Recreational athletes and non-athletes could return to their sports activities satisfactorily, while young competitive athletes found difficulties in certain actions, especially related to eversion.
C1 [Kosaka, T.] Tokyo Med Univ, Dept Orthopaed Surg, Shinjuku Ku, Tokyo 1600023, Japan.
RP Kosaka, T (通讯作者)，Tokyo Med Univ, Dept Orthopaed Surg, Shinjuku Ku, 6-7-1 Nishi Shinjuku, Tokyo 1600023, Japan.
EM tikosaka@h6.dion.ne.jp
CR ABRAHAM E, 1975, J BONE JOINT SURG AM, VA 57, P253, DOI 10.2106/00004623-197557020-00019
   ARNER O, 1959, Acta Chir Scand Suppl, V116, P1
   CHRISTENSEN I, 1953, ACTA CHIR SCAND, V106, P50
   Elias I, 2007, FOOT ANKLE INT, V28, P1238, DOI 10.3113/FAI.2007.1238
   Imakiire A, 2004, JPN J ORTHOP SPORTS, V23, P292
   KATO YP, 1991, J BONE JOINT SURG AM, V73A, P561, DOI 10.2106/00004623-199173040-00013
   LIDMAN D, 1987, SCAND J PLAST RECONS, V21, P213, DOI 10.3109/02844318709078102
   Maffulli Nicola, 2007, Foot Ankle Clin, V12, P583, DOI 10.1016/j.fcl.2007.07.007
   McClelland D, 2004, SURG-J R COLL SURG E, V2, P209, DOI 10.1016/S1479-666X(04)80002-7
   Miskulin Mladen, 2005, J Foot Ankle Surg, V44, P49, DOI 10.1053/j.jfas.2004.11.003
   PEREZTEUFFER A, 1974, ORTHOP CLIN N AM, V5, P89
   Sebastian Hermann, 2007, J Foot Ankle Surg, V46, P424, DOI 10.1053/j.jfas.2007.07.003
   TURCO VJ, 1987, FOOT ANKLE, V7, P253, DOI 10.1177/107110078700700407
   Wegrzyn J, 2010, INT ORTHOP, V34, P1187, DOI 10.1007/s00264-009-0859-1
   WHITE RK, 1959, SURG GYNECOL OBSTET, V108, P117
   Wilcox DK, 2000, FOOT ANKLE INT, V21, P1004, DOI 10.1177/107110070002101204
NR 16
TC 4
Z9 7
U1 0
U2 3
PU UNIV WEST INDIES FACULTY MEDICAL SCIENCES
PI KINGSTON
PA MONA CAMPUS, KINGSTON 7, JAMAICA
SN 0043-3144
J9 W INDIAN MED J
JI West Ind. Med. J.
PD DEC
PY 2011
VL 60
IS 6
BP 628
EP 635
PG 8
WC Medicine, General & Internal
WE Science Citation Index Expanded (SCI-EXPANDED)
SC General & Internal Medicine
GA 920GQ
UT WOS:000302392700006
PM 22512219
DA 2022-07-22
ER

PT J
AU Park, YH
   Kim, TJ
   Choi, GW
   Kim, HJ
AF Park, Young Hwan
   Kim, Tae Jin
   Choi, Gi Won
   Kim, Hak Jun
TI Age is a risk factor for contralateral tendon rupture in patients with
   acute Achilles tendon rupture
SO KNEE SURGERY SPORTS TRAUMATOLOGY ARTHROSCOPY
LA English
DT Article
DE Achilles tendon rupture; Contralateral tendon rupture; Age; Risk factor
ID NONOPERATIVE TREATMENT; INCREASING INCIDENCE; GRAFT RUPTURE;
   EPIDEMIOLOGY; YOUNGER
AB Purpose Rupture of the contralateral Achilles tendon following Achilles tendon rupture can lead to devastating outcomes. However, despite the clinical importance, the risk factors and incidence of contralateral Achilles tendon rupture have not been well studied. This study aimed to determine the incidence of contralateral tendon rupture after Achilles tendon rupture and to identify associated patient characteristics. Methods Medical records for 226 consecutive patients with Achilles tendon rupture were retrospectively reviewed. The occurrence of contralateral Achilles tendon rupture and patient characteristics were determined through review of medical records and telephone surveys. Results The cumulative incidences of contralateral Achilles tendon rupture at 1, 3, 5, and 7 years after Achilles tendon rupture were 0.4%, 1.8%, 3.4%, and 5.1%, respectively. The only statistically significant risk factor was age between 30 and 39 years at the time of initial Achilles tendon rupture (hazard ratio = 4.9). Conclusions Patients who sustain Achilles tendon rupture in their 30 s have significantly increased risk for contralateral tendon rupture.
C1 [Park, Young Hwan; Kim, Tae Jin; Kim, Hak Jun] Korea Univ, Guro Hosp, Dept Orthopaed Surg, 148 Gurodong Ro, Seoul 08308, South Korea.
   [Choi, Gi Won] Korea Univ, Ansan Hosp, Dept Orthopaed Surg, 123 Jeokgeum Ro, Ansan 15355, South Korea.
RP Kim, HJ (通讯作者)，Korea Univ, Guro Hosp, Dept Orthopaed Surg, 148 Gurodong Ro, Seoul 08308, South Korea.
EM ospark1982@gmail.com; owlrocker@gmail.com; manbalian@gmail.com;
   hjunkimos@gmail.com
RI Choi, Gi Won/AAX-7187-2020; Kim, Hak Jun/AAR-5929-2020
OI Kim, Hak Jun/0000-0003-3633-6174
CR Aroen A, 2004, SCAND J MED SCI SPOR, V14, P30, DOI 10.1111/j.1600-0838.2004.00344.x
   El Khoury L, 2016, META GENE, V9, P52, DOI 10.1016/j.mgene.2016.03.007
   Ganestam A, 2016, KNEE SURG SPORT TR A, V24, P3730, DOI 10.1007/s00167-015-3544-5
   Gwynne-Jones DP, 2011, FOOT ANKLE INT, V32, P337, DOI 10.3113/FAI.2011.0337
   Halasi T, 2004, AM J SPORT MED, V32, P899, DOI 10.1177/0363546503262181
   Houshian S, 1998, INJURY, V29, P651, DOI 10.1016/S0020-1383(98)00147-8
   KANNUS P, 1991, J BONE JOINT SURG AM, V73A, P1507, DOI 10.2106/00004623-199173100-00009
   Khan RJK, 2005, J BONE JOINT SURG AM, V87A, P2202, DOI 10.2106/JBJS.D.03049
   Kim SK, 2017, PLOS ONE, V12, DOI 10.1371/journal.pone.0170422
   Maffulli N, 2000, AM J SPORT MED, V28, P499, DOI 10.1177/03635465000280040901
   Maffulli N, 1999, CLIN J SPORT MED, V9, P157, DOI 10.1097/00042752-199907000-00007
   Moller A, 1996, ACTA ORTHOP SCAND, V67, P479, DOI 10.3109/17453679608996672
   Nilsson-Helander K, 2010, AM J SPORT MED, V38, P2186, DOI 10.1177/0363546510376052
   Nyyssonen T, 2008, SCAND J SURG, V97, P272, DOI 10.1177/145749690809700312
   Park YH, 2017, INJURY, V48, P776, DOI 10.1016/j.injury.2017.01.020
   Raikin SM, 2013, FOOT ANKLE INT, V34, P475, DOI 10.1177/1071100713477621
   Reito A, 2018, FOOT ANKLE INT, DOI [10.1177/10711007177540421071100717754042, DOI 10.1177/10711007177540421071100717754042]
   Rettig AC, 2005, AM J SPORT MED, V33, P119, DOI 10.1177/0363546504268720
   Salmon L, 2005, ARTHROSCOPY, V21, P948, DOI 10.1016/j.arthro.2005.04.110
   Webster KE, 2014, AM J SPORT MED, V42, P641, DOI 10.1177/0363546513517540
   Willits K, 2010, J BONE JOINT SURG AM, V92A, P2767, DOI 10.2106/JBJS.I.01401
   Yasui Y, 2017, BIOMED RES INT, V2017, DOI 10.1155/2017/7021862
   Young SW, 2014, J BONE JOINT SURG AM, V96A, P1073, DOI 10.2106/JBJS.M.00248
NR 23
TC 2
Z9 3
U1 0
U2 5
PU SPRINGER
PI NEW YORK
PA ONE NEW YORK PLAZA, SUITE 4600, NEW YORK, NY, UNITED STATES
SN 0942-2056
EI 1433-7347
J9 KNEE SURG SPORT TR A
JI Knee Surg. Sports Traumatol. Arthrosc.
PD MAY
PY 2020
VL 28
IS 5
BP 1625
EP 1630
DI 10.1007/s00167-019-05380-y
PG 6
WC Orthopedics; Sport Sciences; Surgery
WE Science Citation Index Expanded (SCI-EXPANDED)
SC Orthopedics; Sport Sciences; Surgery
GA LH8KZ
UT WOS:000529032900037
PM 30778628
DA 2022-07-22
ER

PT J
AU Heckman, DS
   Gluck, GS
   Parekh, SG
AF Heckman, Daniel S.
   Gluck, George S.
   Parekh, Selene G.
TI Tendon Disorders of the Foot and Ankle, Part 2 Achilles Tendon Disorders
SO AMERICAN JOURNAL OF SPORTS MEDICINE
LA English
DT Article
DE Achilles; ruptures; insertional tendinitis; Haglund; bursitis
ID SURGICAL-TREATMENT; RETROCALCANEAL BURSITIS; OPEN REPAIR; PROSPECTIVE
   MULTICENTER; DIFFERENTIAL-DIAGNOSIS; NONOPERATIVE TREATMENT;
   PERCUTANEOUS REPAIR; HAGLUND DEFORMITY; OVERUSE INJURIES; CALCANEAL
   TENDON
AB Disorders of the Achilles tendon include both acute and chronic ruptures as well as a spectrum of chronic overuse injuries involving inflammatory and degenerative changes within the tendon and surrounding tissues. These injuries are relatively common in athletes as well as among the general population. There is no consensus on the optimal treatment of Achilles tendon disorders. The goals of this review are to develop a current understanding of the anatomy and diagnostic evaluation of the Achilles tendon, and to present current treatment options and the authors' preferred surgical techniques for operative management of Achilles tendon disorders.
C1 [Heckman, Daniel S.; Gluck, George S.] Univ N Carolina, Sch Med, Dept Orthopaed Surg, Chapel Hill, NC USA.
   [Parekh, Selene G.] Duke Univ, Div Orthopaed Surg, Durham, NC USA.
   [Parekh, Selene G.] Duke Univ, Fuqua Sch Business, Durham, NC 27706 USA.
RP Parekh, SG (通讯作者)，N Carolina Orthopaed Clin, 3609 SW Durham Dr, Durham, NC 27707 USA.
EM selene.parekh@gmail.com
OI Parekh, Selene/0000-0002-1308-9038
CR ABRAHAM E, 1975, J BONE JOINT SURG AM, V57, P871
   Alfredson H, 1998, AM J SPORT MED, V26, P360, DOI 10.1177/03635465980260030301
   Assal M, 2002, J BONE JOINT SURG AM, V84A, P161, DOI 10.2106/00004623-200202000-00001
   Bhandari M, 2002, CLIN ORTHOP RELAT R, P190
   BUGG EI, 1968, CLIN ORTHOP RELAT R, P73
   Calder JDF, 2003, FOOT ANKLE INT, V24, P119, DOI 10.1177/107110070302400203
   CARR AJ, 1989, J BONE JOINT SURG BR, V71, P100, DOI 10.1302/0301-620X.71B1.2914976
   CETTI R, 1983, CLIN ORTHOP RELAT R, P204
   CETTI R, 1993, AM J SPORT MED, V21, P791, DOI 10.1177/036354659302100606
   CHRISTENSEN I, 1953, ACTA CHIR SCAND, V106, P50
   Coughlin MJ, 1999, SURG FOOT ANKLE, P835
   Cretnik A, 2005, AM J SPORT MED, V33, P1369, DOI 10.1177/0363546504271501
   Den Hartog BD, 2003, FOOT ANKLE INT, V24, P233, DOI 10.1177/107110070302400306
   DeOrio MJ, 2008, FOOT ANKLE INT, V29, P542, DOI 10.3113/FAI.2008.0542
   Fahlstrom M, 2003, KNEE SURG SPORT TR A, V11, P327, DOI 10.1007/s00167-003-0418-z
   FITZGIBBONS RE, 1993, AM J SPORT MED, V21, P724, DOI 10.1177/036354659302100516
   Gorschewsky O, 2004, FOOT ANKLE INT, V25, P219, DOI 10.1177/107110070402500405
   Halasi T, 2003, KNEE SURG SPORT TR A, V11, P409, DOI 10.1007/s00167-003-0397-0
   Hoffman B, 2006, FOOT ANKLE INT, V27, P960, DOI 10.1177/107110070602701116
   HOWARD CB, 1984, J BONE JOINT SURG BR, V66, P206, DOI 10.1302/0301-620X.66B2.6707056
   Huffard B, 2008, CLIN BIOMECH, V23, P1158, DOI 10.1016/j.clinbiomech.2008.05.007
   Hufner TM, 2006, FOOT ANKLE INT, V27, P167, DOI 10.1177/107110070602700302
   Jennings AG, 2002, J BONE JOINT SURG BR, V84B, P361, DOI 10.1302/0301-620X.84B3.11559
   Johnston E, 1997, FOOT ANKLE INT, V18, P570, DOI 10.1177/107110079701800907
   JONES DC, 1984, AM J SPORT MED, V12, P72, DOI 10.1177/036354658401200111
   Jonsson P, 2008, BRIT J SPORT MED, V42, P746, DOI 10.1136/bjsm.2007.039545
   KAKIUCHI M, 1995, J BONE JOINT SURG BR, V77B, P60, DOI 10.1302/0301-620X.77B1.7822398
   KANNUS P, 1991, J BONE JOINT SURG AM, V73A, P1507, DOI 10.2106/00004623-199173100-00009
   Kaufman KR, 1999, AM J SPORT MED, V27, P585, DOI 10.1177/03635465990270050701
   Khan RJ, 2004, COCHRANE DB SYST REV, V3, DOI DOI 10.1002/14651858.CD003674.PUB2
   Kolodziej P, 1999, FOOT ANKLE INT, V20, P433, DOI 10.1177/107110079902000707
   Kotnis R, 2006, AM J SPORT MED, V34, P1395, DOI 10.1177/0363546506288678
   Krishna Sayana Murali, 2005, Foot Ankle Clin, V10, P309
   KVIST H, 1980, J BONE JOINT SURG BR, V62, P353, DOI 10.1302/0301-620X.62B3.7410468
   KVIST M, 1994, SPORTS MED, V18, P173, DOI 10.2165/00007256-199418030-00004
   LEITNER A, 1992, FOOT ANKLE, V13, P176, DOI 10.1177/107110079201300402
   Leitze Z, 2003, J BONE JOINT SURG AM, V85A, P1488, DOI 10.2106/00004623-200308000-00009
   LYNN TA, 1966, J BONE JOINT SURG AM, VA 48, P268, DOI 10.2106/00004623-196648020-00005
   LYSHOLM J, 1987, AM J SPORT MED, V15, P168, DOI 10.1177/036354658701500213
   MA GWC, 1977, CLIN ORTHOP RELAT R, P247
   Maffulli N, 1999, J BONE JOINT SURG AM, V81A, P1019, DOI 10.2106/00004623-199907000-00017
   Maffulli N, 1997, AM J SPORT MED, V25, P835, DOI 10.1177/036354659702500618
   Maffulli N, 2005, CLIN J SPORT MED, V15, P56, DOI 10.1097/01.jsm.0000152714.05097.ef
   Maffulli N, 2004, AM J SPORT MED, V32, P174, DOI 10.1177/0363546503258923
   Maffulli N, 1996, BRIT J SPORT MED, V30, P250, DOI 10.1136/bjsm.30.3.250
   Maffulli Nicola, 2007, Foot Ankle Clin, V12, P583, DOI 10.1016/j.fcl.2007.07.007
   Mafi N, 2001, KNEE SURG SPORT TR A, V9, P42, DOI 10.1007/s001670000148
   Mahler F, 1992, Br J Sports Med, V26, P7
   MANDELBAUM BR, 1995, AM J SPORT MED, V23, P392, DOI 10.1177/036354659502300403
   MANN RA, 1991, J BONE JOINT SURG AM, V73A, P214, DOI 10.2106/00004623-199173020-00009
   Maquirriain J, 2002, ARTHROSCOPY, V18, P298, DOI 10.1053/jars.2002.30065b
   McGarvey WC, 1996, FOOT ANKLE INT, V17, P496, DOI 10.1177/107110079601700811
   McGarvey WC, 2002, FOOT ANKLE INT, V23, P19, DOI 10.1177/107110070202300104
   NELEN G, 1989, AM J SPORT MED, V17, P754, DOI 10.1177/036354658901700605
   Nellas Z J, 1996, J Foot Ankle Surg, V35, P144
   Nigg BM, 2001, CLIN J SPORT MED, V11, P2, DOI 10.1097/00042752-200101000-00002
   O'Brien Moira, 2005, Foot Ankle Clin, V10, P225, DOI 10.1016/j.fcl.2005.01.011
   Ohberg L, 2003, KNEE SURG SPORT TR A, V11, P339, DOI 10.1007/s00167-003-0402-7
   Ortmann FW, 2007, FOOT ANKLE INT, V28, P149, DOI 10.3113/FAI.2007.0149
   OZAKI J, 1989, CLIN ORTHOP RELAT R, P204
   Paavola M, 1998, ACTA RADIOL, V39, P612, DOI 10.3109/02841859809175485
   Paavola M, 2002, J BONE JOINT SURG AM, V84A, P2062, DOI 10.2106/00004623-200211000-00024
   Paoloni JA, 2004, J BONE JOINT SURG AM, V86A, P916, DOI 10.2106/00004623-200405000-00005
   PARSONS JR, 1984, FOOT ANKLE, V5, P49, DOI 10.1177/107110078400500202
   PAVLOV H, 1982, RADIOLOGY, V144, P83, DOI 10.1148/radiology.144.1.7089270
   PEREZTEUFFER A, 1974, ORTHOP CLIN N AM, V5, P89
   Porter DA, 1997, FOOT ANKLE INT, V18, P557, DOI 10.1177/107110079701800905
   Puddu G, 1976, Am J Sports Med, V4, P145, DOI 10.1177/036354657600400404
   Saltzman C L, 1998, J Am Acad Orthop Surg, V6, P316
   Saxena A, 2003, J AM PODIAT MED ASSN, V93, P283, DOI 10.7547/87507315-93-4-283
   Schepsis AA, 2002, AM J SPORT MED, V30, P287, DOI 10.1177/03635465020300022501
   SCHEPSIS AA, 1994, AM J SPORT MED, V22, P611, DOI 10.1177/036354659402200508
   Sella EJ, 1998, J FOOT ANKLE SURG, V37, P173
   STEPHENS MM, 1994, ORTHOP CLIN N AM, V25, P41
   Thermann H, 2001, FOOT ANKLE INT, V22, P158, DOI 10.1177/107110070102200214
   Turco V, 1988, Orthop Rev, V17, P822
   van Dijk CN, 2001, AM J SPORT MED, V29, P185
   Wagner E, 2006, FOOT ANKLE INT, V27, P677, DOI 10.1177/107110070602700904
   Wapner K L, 1993, Foot Ankle, V14, P443
   WATSON TW, 1995, FOOT ANKLE INT, V16, P191, DOI 10.1177/107110079501600404
   Wilcox DK, 2000, FOOT ANKLE INT, V21, P1004, DOI 10.1177/107110070002101204
   Wong J, 2002, AM J SPORT MED, V30, P565, DOI 10.1177/03635465020300041701
NR 82
TC 44
Z9 46
U1 0
U2 13
PU SAGE PUBLICATIONS INC
PI THOUSAND OAKS
PA 2455 TELLER RD, THOUSAND OAKS, CA 91320 USA
SN 0363-5465
EI 1552-3365
J9 AM J SPORT MED
JI Am. J. Sports Med.
PD JUN
PY 2009
VL 37
IS 6
BP 1223
EP 1234
DI 10.1177/0363546509335947
PG 12
WC Orthopedics; Sport Sciences
WE Science Citation Index Expanded (SCI-EXPANDED)
SC Orthopedics; Sport Sciences
GA 449KH
UT WOS:000266329000023
PM 19417123
DA 2022-07-22
ER

PT J
AU Zhao, HM
   Yu, GR
   Yang, YF
   Zhou, JQ
   Aubeeluck, A
AF Zhao Hong-mou
   Yu Guang-rong
   Yang Yun-feng
   Zhou Jia-qian
   Aubeeluck, Ashwin
TI Outcomes and complications of operative versus non-operative treatment
   of acute Achilles tendon rupture: a meta-analysis
SO CHINESE MEDICAL JOURNAL
LA English
DT Article
DE Achilles tendon injury; operation; conservative; complication;
   meta-analysis
ID SURGICAL-TREATMENT; PROSPECTIVE TRIAL; EARLY MOTION; SURGERY
AB Background There is lack of consensus regarding the best option for the treatment of acute Achilles tendon rupture operation or non-operation. The purpose of this meta-analysis was to identify and summarize the randomized controlled trials comparing the operative and non-operative lines of treatment of acute Achilles tendon ruptures.
   Methods We searched multiple databases in English (including EMBASE, PubMed, and OVID) and in Chinese (including CNKI, WANFANG, and VIP), as well as reference lists of articles and main orthopaedic and sports medical journals. Two reviewers independently screened the studies for eligibility, evaluated the quality and extracted data from eligible studies, with confirmation by cross-checking. The major results and conclusions were concluded, and the different complication rates and functional outcomes were compared. Meta-analysis was processed by Rev Man 5.0 software.
   Results Eight randomized controlled trials (RCTs) involving 777 patients met the inclusion criteria. The rerupture rate in non-operative group was significantly higher (Z=3.33, P <0.01). However, the moderate (Z=4.27, P <0.01) and minor (Z=5.59, P <0.01) complication rate in the operative group were significantly higher. No significant difference in comparing the major and total complication rates. The return to work time in the operative group was shorter (Z=2.65, P <0.01). The inability to return to previous level sporting rate and ankle joint decreased range of motion (ROM) rate showed no significant difference in the two groups. Other functional outcomes were similar in the two groups.
   Conclusions Operation could significantly reduce the risk of rerupture; however, it was associated with a higher risk of other complications. The functional outcomes were similar in two treatment methods except an earlier return to work in patients treated operatively. Thus operative treatment is preferable for patients with good physical condition. Non-operative treatment is an acceptable alternative especially for the older and patients with lower sporting requirements. Chin Med J 2011;124(23):4050-4055
C1 [Zhao Hong-mou; Yu Guang-rong; Yang Yun-feng; Zhou Jia-qian; Aubeeluck, Ashwin] Tongji Univ, Tongji Hosp, Dept Orthopaed Surg, Shanghai 200065, Peoples R China.
RP Yu, GR (通讯作者)，Tongji Univ, Tongji Hosp, Dept Orthopaed Surg, Shanghai 200065, Peoples R China.
EM yuguangrong2002@yahoo.com.cn
FU National Natural Science Foundation of China [30801163/H0610]
FX This study was supported by a grant from the National Natural Science
   Foundation of China (No. 30801163/H0610).
CR Bhandari M, 2002, CLIN ORTHOP RELAT R, P190
   CETTI R, 1993, AM J SPORT MED, V21, P791, DOI 10.1177/036354659302100606
   COOMBS RRH, 1981, J BONE JOINT SURG BR, V63, P288
   HATTRUP SJ, 1985, FOOT ANKLE, V6, P34, DOI 10.1177/107110078500600107
   Jadad AR, 1996, CONTROL CLIN TRIALS, V17, P1, DOI 10.1016/0197-2456(95)00134-4
   Kangas J, 2007, AM J SPORT MED, V35, P59, DOI 10.1177/0363546506293255
   Keating JF, 2011, J BONE JOINT SURG BR, V93B, P1071, DOI 10.1302/0301-620X.93B8.25998
   KELLAM JF, 1985, CLIN ORTHOP RELAT R, P80
   Khan RJK, 2005, J BONE JOINT SURG AM, V87A, P2202, DOI 10.2106/JBJS.D.03049
   Leppilahti J, 1998, SPORTS MED, V25, P79, DOI 10.2165/00007256-199825020-00002
   Leppilahti J, 1996, ACTA ORTHOP SCAND, V67, P277, DOI 10.3109/17453679608994688
   Lo IKY, 1997, CLIN J SPORT MED, V7, P207, DOI 10.1097/00042752-199707000-00010
   Maffulli N, 1999, J BONE JOINT SURG AM, V81A, P1019, DOI 10.2106/00004623-199907000-00017
   Maffulli N, 1999, CLIN J SPORT MED, V9, P157, DOI 10.1097/00042752-199907000-00007
   Majewski M, 2000, ORTHOPADE, V29, P670
   Metz R, 2008, AM J SPORT MED, V36, P1688, DOI 10.1177/0363546508319312
   Moller M, 2001, J BONE JOINT SURG BR, V83B, P843
   Mortensen NHM, 1999, J BONE JOINT SURG AM, V81A, P983, DOI 10.2106/00004623-199907000-00011
   Nilsson-Helander K, 2010, AM J SPORT MED, V38, P2186, DOI 10.1177/0363546510376052
   Nilsson-Helander K, 2009, KNEE SURG SPORT TR A, V17, P1234, DOI 10.1007/s00167-009-0727-y
   NISTOR L, 1981, J BONE JOINT SURG AM, V63, P394, DOI 10.2106/00004623-198163030-00012
   Pajala A, 2002, J BONE JOINT SURG AM, V84A, P2016, DOI 10.2106/00004623-200211000-00017
   THERMANN H, 1995, UNFALLCHIRURG, V98, P21
   Twaddle BC, 2007, AM J SPORT MED, V35, P2033, DOI 10.1177/0363546507307503
   Willits K, 2010, J BONE JOINT SURG AM, V92A, P2767, DOI 10.2106/JBJS.I.01401
   WILLS CA, 1986, CLIN ORTHOP RELAT R, P156
NR 26
TC 20
Z9 22
U1 1
U2 10
PU WOLTERS KLUWER MEDKNOW PUBLICATIONS
PI MUMBAI
PA WOLTERS KLUWER INDIA PVT LTD , A-202, 2ND FLR, QUBE, C T S  NO 1498A-2
   VILLAGE MAROL, ANDHERI EAST, MUMBAI, 400059, INDIA
SN 0366-6999
J9 CHINESE MED J-PEKING
JI Chin. Med. J.
PD DEC 5
PY 2011
VL 124
IS 23
BP 4050
EP 4055
DI 10.3760/cma.j.issn.0366-6999.2011.23.038
PG 6
WC Medicine, General & Internal
WE Science Citation Index Expanded (SCI-EXPANDED)
SC General & Internal Medicine
GA 875US
UT WOS:000299061300038
PM 22340341
DA 2022-07-22
ER

PT J
AU McClelland, D
   Maffulli, N
AF McClelland, D
   Maffulli, N
TI Percutaneous repair of ruptured Achilles tendon
SO JOURNAL OF THE ROYAL COLLEGE OF SURGEONS OF EDINBURGH
LA English
DT Article
DE Achilles tendon; percutaneous repair
ID BILATERAL RUPTURE
AB Percutaneous repair of a ruptured Achilles tendon is a safe and reliable method of treating such injuries in patients with low sporting requirements. It has a lower incidence of wound complications compared with open techniques but a slightly higher incidence of re-rupture. Sural nerve injury is avoidable by careful placement of the incisions.
C1 N Staffordshire Royal Infirm, Stoke On Trent ST4 7LN, Staffs, England.
RP Maffulli, N (通讯作者)，Keele Univ, Sch Med, Dept Trauma & Orthopaed Surg, Hartshill,Thornburrow Dr, Stoke On Trent ST4 7QB, Staffs, England.
RI Maffulli, Nicola/A-1839-2014
OI Maffulli, Nicola/0000-0002-5327-3702
CR BRADLEY JP, 1990, AM J SPORT MED, V18, P188, DOI 10.1177/036354659001800213
   Canale ST, CAMPBELLS OPERATIVE
   CARLSTEDT CA, 1987, ACTA ORTHOP SCAND, V58, P7
   Cretnik A, 2000, MED SCI SPORT EXER, V32, P16
   Gorschewsky O, 1999, INJURY, V30, P315, DOI 10.1016/S0020-1383(99)00089-3
   HAGGMARK T, 1986, ORTHOPEDICS, V9, P160
   HAINES JF, 1983, ANN RHEUM DIS, V42, P652, DOI 10.1136/ard.42.6.652
   HESTIN D, 1993, NEPHRON, V65, P491, DOI 10.1159/000187546
   HOCKENBURY RT, 1990, FOOT ANKLE, V11, P67, DOI 10.1177/107110079001100202
   Kauranen KJ, 2001, INT J SPORTS MED, V22, P154, DOI 10.1055/s-2001-11345
   LANGERGREN C, 1958, ACTA CHIR SCAND, V116, P491
   Lim J, 2001, FOOT ANKLE INT, V22, P559, DOI 10.1177/107110070102200705
   MA GWC, 1977, CLIN ORTHOP RELAT R, P247
   Martinelli B, 2000, Bull Hosp Jt Dis, V59, P149
   Matles A L, 1975, Bull Hosp Joint Dis, V36, P48
   Mcmaster PE, 1933, J BONE JOINT SURG, V15, P705
   OBRIEN T, 1984, J BONE JOINT SURG AM, V66A, P1099, DOI 10.2106/00004623-198466070-00018
   Poon CCH, 1997, MED J AUSTRALIA, V166, P665, DOI 10.5694/j.1326-5377.1997.tb123308.x
   RAKS MA, 1978, JAMA-J AM MED ASSOC, V239, P435
   SIMMONDS F A, 1957, Practitioner, V179, P56
   Tallon C, 2001, MED SCI SPORT EXER, V33, P1983, DOI 10.1097/00005768-200112000-00002
   VIIDIK A, 1962, ACTA ORTHOP SCAND, V10, P261
   Webb J, 2000, FOOT ANKLE INT, V21, P475, DOI 10.1177/107110070002100604
   Webb JM, 1999, J BONE JOINT SURG BR, V81B, P877, DOI 10.1302/0301-620X.81B5.9784
   WILLIAMS IF, 1985, CELLULAR BIOCH COMPO, V3, P43
   WILLIAMS PL, 1989, GRAYS ABATOMY
NR 26
TC 35
Z9 36
U1 0
U2 0
PU ROYAL COLLEGE SURGEONS EDINBURGH
PI EDINBURGH
PA NICOLSON ST, EDINBURGH EH8 9DW, SCOTLAND
SN 0035-8835
J9 J ROY COLL SURG EDIN
JI J. R. Coll. Surg. Edinb.
PD AUG
PY 2002
VL 47
IS 4
BP 613
EP 618
PG 6
WC Surgery
WE Science Citation Index Expanded (SCI-EXPANDED)
SC Surgery
GA 600HN
UT WOS:000178384700004
PM 12363186
DA 2022-07-22
ER

PT J
AU Touzell, A
AF Touzell, Amy
TI The Achilles tendon Management of acute and chronic conditions
SO AUSTRALIAN JOURNAL OF GENERAL PRACTICE
LA English
DT Article
ID SURGICAL REPAIR; RUPTURE; TENDINOPATHY
AB Background
   The prevalence of acute and chronic conditions of the Achilles tendon is increasing among an ageing, active population. These conditions are a common cause of presentation to general practitioners and allied health practitioners. Achilles tendon injuries have a bimodal demographical presentation, with acute injuries commonly occurring in younger people and chronic conditions presenting in patients who are elderly.
   Objective
   The aims of this article are to discuss management of acute Achilles tendon ruptures in the primary care setting, explain the risks associated with calcaneal tuberosity fracture and discuss non-operative and surgical management of acute and chronic overload conditions of the Achilles tendon.
   Discussion
   Achilles tendon injuries can be divided into acute ruptures and chronic overuse injuries. Both can be debilitating, with significant morbidity for patients; fortunately, both types of injuries respond well to non-operative interventions, with only a small proportion requiring surgery. Management of acute Achilles tendon rupture has evolved, with increasing evidence that non-operative management is appropriate providing patients participate in a functional rehabilitation protocol. Chronic conditions such as the sequalae of an untreated rupture or Achilles tendinopathy can be debilitating but often respond well to non-operative management.
C1 [Touzell, Amy] Monash Univ, Peninsula Clin Sch, Melbourne, Vic, Australia.
   [Touzell, Amy] Peninsula Hlth, Frankston, Vic, Australia.
   [Touzell, Amy] Holmesglen Private Hosp, Moorabbin, Vic, Australia.
   [Touzell, Amy] Cabrini Private Hosp, Malvern, Vic, Australia.
   [Touzell, Amy] Frankston Private Hosp, Frankston, Vic, Australia.
RP Touzell, A (通讯作者)，Monash Univ, Peninsula Clin Sch, Melbourne, Vic, Australia.; Touzell, A (通讯作者)，Peninsula Hlth, Frankston, Vic, Australia.; Touzell, A (通讯作者)，Holmesglen Private Hosp, Moorabbin, Vic, Australia.; Touzell, A (通讯作者)，Cabrini Private Hosp, Malvern, Vic, Australia.; Touzell, A (通讯作者)，Frankston Private Hosp, Frankston, Vic, Australia.
EM Vic.admin@southeastortho.com.au
CR Alfredson H, 1998, AM J SPORT MED, V26, P360, DOI 10.1177/03635465980260030301
   Bullock Mark J, 2017, Foot Ankle Spec, V10, P415, DOI 10.1177/1938640016679706
   Cook JL, 2014, BRIT J SPORT MED, V48, P506, DOI 10.1136/bjsports-2012-092078
   de Vos RJ, 2011, BRIT J SPORT MED, V45, P387, DOI 10.1136/bjsm.2010.076398
   GABEL S, 1994, FOOT ANKLE INT, V15, P512, DOI 10.1177/107110079401500912
   Gillissen S, 2021, EUR J TRAUMA EMERG S, V47, P2049, DOI 10.1007/s00068-020-01376-3
   Hreljac A, 2004, MED SCI SPORT EXER, V36, P845, DOI 10.1249/01.MSS.0000126803.66636.DD
   INGLIS AE, 1981, CLIN ORTHOP RELAT R, P160
   Jarvinen Tero A H, 2005, Foot Ankle Clin, V10, P255, DOI 10.1016/j.fcl.2005.01.013
   Jones Matthew P, 2012, J Bone Joint Surg Am, V94, pe88, DOI 10.2106/JBJS.J.01829
   Levin A, 2002, J ECONOMETRICS, V108, P1, DOI 10.1016/S0304-4076(01)00098-7
   Maffulli N, 1999, J BONE JOINT SURG AM, V81A, P1019, DOI 10.2106/00004623-199907000-00017
   Maffulli N, 1998, AM J SPORT MED, V26, P266, DOI 10.1177/03635465980260021801
   MAFFULLI N, 1990, INT J SPORTS MED, V11, P78, DOI 10.1055/s-2007-1024767
   Molloy A, 2009, FOOT ANKLE CLIN, V14, P745, DOI 10.1016/j.fcl.2009.07.004
   Nilsson-Helander K, 2010, AM J SPORT MED, V38, P2186, DOI 10.1177/0363546510376052
   Park SH, 2020, CLIN ORTHOP SURG, V12, P1, DOI 10.4055/cios.2020.12.1.1
   Patel A, 2012, CLIN ORTHOP RELAT R, V470, P270, DOI 10.1007/s11999-011-2166-6
   Rauer T, 2018, J FOOT ANKLE SURG, V57, P191, DOI 10.1053/j.jfas.2017.07.016
   Reddy SS, 2009, J AM ACAD ORTHOP SUR, V17, P3, DOI 10.5435/00124635-200901000-00002
   Saltzman C L, 1998, J Am Acad Orthop Surg, V6, P316
   Sharma P, 2005, J BONE JOINT SURG AM, V87A, P187, DOI 10.2106/JBJS.D.01850
   Singh D, 2017, BRIT J SPORT MED, V51, P1158, DOI 10.1136/bjsports-2016-h4722rep
   Suchak AA, 2008, J BONE JOINT SURG AM, V90A, P1876, DOI 10.2106/JBJS.G.01242
   Willits K, 2010, J BONE JOINT SURG AM, V92A, P2767, DOI 10.2106/JBJS.I.01401
   Wu YH, 2019, AM J SPORT MED, V47, P2251, DOI 10.1177/0363546518824601
   Young J. S., 2007, ACHILLES TENDON, P39
NR 27
TC 2
Z9 2
U1 0
U2 0
PU ROYAL AUSTRALIAN COLLEGE GENERAL PRACTITIONERS
PI SOUTH MELBOURNE
PA 1 PALMERSTON CRESCENT, SOUTH MELBOURNE, VICTORIA 3205, AUSTRALIA
SN 2208-794X
EI 2208-7958
J9 AUST J GEN PRACT
JI Aust. J. Gen. Pract.
PD NOV
PY 2020
VL 49
IS 11
BP 715
EP 719
PG 5
WC Medicine, General & Internal
WE Science Citation Index Expanded (SCI-EXPANDED)
SC General & Internal Medicine
GA PE8YB
UT WOS:000598646200009
PM 33123713
OA Bronze
DA 2022-07-22
ER

PT J
AU Gross, CE
   Nunley, JA
AF Gross, Christopher E.
   Nunley, James A.
TI Treatment of Neglected Achilles Tendon Ruptures with Interpositional
   Allograft
SO FOOT AND ANKLE CLINICS
LA English
DT Article
DE Chronic Achilles tear; Achilles allograft; Achilles reconstruction
ID RECONSTRUCTION; DIAGNOSIS; REPAIR
AB Although most astute clinicians can diagnose Achilles tendon ruptures by physical examination alone, more than 20% are not accurately diagnosed in a timely fashion. The definition of a "chronic" Achilles tendon rupture in the foot and ankle literature varies widely: from 4 to 10 weeks status after injury. Neglected or chronic Achilles tendon ruptures can be significantly disabling to patients if the muscle-tendon unit is stretched beyond its normal passive limit. There are a variety of treatment options that all have valid uses but have not been proved to be superior to one another.
C1 [Gross, Christopher E.] Med Univ South Carolina, Dept Orthopaed, 96 Jonathan Lucas Dr,CSB 708,MSC 622, Charleston, SC 29425 USA.
   [Nunley, James A.] Duke Univ, Dept Orthopaed Surg, Med Ctr, 4709 Creekstone Dr, Durham, NC 27703 USA.
RP Gross, CE (通讯作者)，Med Univ South Carolina, Dept Orthopaed, 96 Jonathan Lucas Dr,CSB 708,MSC 622, Charleston, SC 29425 USA.
EM cgross144@gmail.com
CR Catanzariti AR, 2016, CLIN PODIATR MED SUR, V33, P125, DOI 10.1016/j.cpm.2015.07.003
   Chiodo CP, 2010, J AM ACAD ORTHOP SUR, V18, P503, DOI 10.5435/00124635-201008000-00007
   Cienfuegos A, 2013, J FOOT ANKLE SURG, V52, P95, DOI 10.1053/j.jfas.2012.06.006
   Deese J Melvin, 2015, J Surg Orthop Adv, V24, P75
   DISTEFANO VJ, 1972, J TRAUM, V12, P671
   GILLIES H, 1970, J BONE JOINT SURG AM, VA 52, P337, DOI 10.2106/00004623-197052020-00015
   Hanna T, 2014, FOOT ANKLE INT, V35, P168, DOI 10.1177/1071100713514037
   Hansen U, 2010, J FOOT ANKLE SURG, V49, DOI 10.1053/j.jfas.2009.08.006
   Haraguchi N, 2005, TECH FOOT ANKLE SURG, V4, P154, DOI 10.1097/01.btf.0000177228.45213.5d
   Hollawell S, 2015, J FOOT ANKLE SURG, V54, P1146, DOI 10.1053/j.jfas.2014.09.006
   Horstmann T, 2012, INT J SPORTS MED, V33, P474, DOI 10.1055/s-0032-1301932
   KUWADA G T, 1990, Journal of Foot Surgery, V29, P361
   Lepow Gary M, 2006, J Foot Ankle Surg, V45, P351, DOI 10.1053/j.jfas.2006.06.004
   Leppilahti J, 2000, FOOT ANKLE INT, V21, P330, DOI 10.1177/107110070002100410
   Maffulli N, 1999, J BONE JOINT SURG AM, V81A, P1019, DOI 10.2106/00004623-199907000-00017
   Maffulli N, 2005, CLIN J SPORT MED, V15, P56, DOI 10.1097/01.jsm.0000152714.05097.ef
   Maffulli N, 1996, BRIT J SPORT MED, V30, P250, DOI 10.1136/bjsm.30.3.250
   Maffulli N, 1998, AM J SPORT MED, V26, P266, DOI 10.1177/03635465980260021801
   Maffulli Nicola, 2007, Foot Ankle Clin, V12, P583, DOI 10.1016/j.fcl.2007.07.007
   Mook WR, 2013, FOOT ANKLE INT, V34, P1212, DOI 10.1177/1071100713487527
   Myerson M S, 1999, Instr Course Lect, V48, P219
   Nellas ZJ, 1996, J FOOT ANKLE SURG, V35, P190
   Ofili KP, 2016, J FOOT ANKLE SURG, V55, P1245, DOI 10.1053/j.jfas.2016.01.001
   Parodi Pier Camillo, 2006, Ann Ital Chir, V77, P361
   Porter DA, 1997, FOOT ANKLE INT, V18, P557, DOI 10.1177/107110079701800905
   Saxena V, 2013, INDIAN J ORTHOP, V47, P634, DOI 10.4103/0019-5413.121600
   Yuen JC, 2001, PLAST RECONSTR SURG, V107, P1807, DOI 10.1097/00006534-200106000-00028
NR 27
TC 7
Z9 8
U1 0
U2 0
PU W B SAUNDERS CO-ELSEVIER INC
PI PHILADELPHIA
PA 1600 JOHN F KENNEDY BOULEVARD, STE 1800, PHILADELPHIA, PA 19103-2899 USA
SN 1083-7515
EI 1558-1934
J9 FOOT ANKLE CLIN
JI Foot Ankle Clin.
PD DEC
PY 2017
VL 22
IS 4
BP 735
EP +
DI 10.1016/j.fcl.2017.07.010
PG 10
WC Orthopedics
WE Science Citation Index Expanded (SCI-EXPANDED)
SC Orthopedics
GA FN7MO
UT WOS:000416202500007
PM 29078825
DA 2022-07-22
ER

PT J
AU Maffulli, N
   Richards, PJ
AF Maffulli, Nicola
   Richards, Paula J.
TI Subcutaneous rupture of the Achilles tendon and ipsilateral fracture of
   the medial malleolus
SO BMC MUSCULOSKELETAL DISORDERS
LA English
DT Article
ID INJURY
AB Background: Although ankle fractures and an Achilles tendon rupture are relatively frequent in isolation, their association in the same injury is uncommon.
   Case presentation: A 38 year old male tree surgeon fell six meters from a tree, sustaining a subcutaneous rupture of the Achilles tendon and an ipsilateral closed fracture of the medial malleolus. The injuries were diagnosed following clinical examination and imaging.
   Conclusion: This injury combination is infrequent, and management of the Achilles tendon rupture should take into account the necessity not to secondarily displace the fracture of the medial malleollus.
C1 Keele Univ, Sch Med, Dept Traumat & Orthopaed Surg, Stoke On Trent, Staffs, England.
   Univ Hosp N Staffordshire, Dept Imaging, Stoke On Trent, Staffs, England.
RP Maffulli, N (通讯作者)，Keele Univ, Sch Med, Dept Traumat & Orthopaed Surg, Stoke On Trent, Staffs, England.
EM osa14@keele.ac.uk; doctor.p.richards@btinternet.com
RI Maffulli, Nicola/A-1839-2014
OI Maffulli, Nicola/0000-0002-5327-3702
CR Assal M, 2002, J ORTHOP TRAUMA, V16, P358, DOI 10.1097/00005131-200205000-00013
   BARRON JL, 1993, AM J SPORT MED, V21, P629, DOI 10.1177/036354659302100426
   CLAYTON M L, 1962, Clin Orthop, V23, P52
   Funk JR, 2002, J BIOMECH ENG-T ASME, V124, P750, DOI 10.1115/1.1514675
   Jensen SL, 1998, ACTA ORTHOP SCAND, V69, P48, DOI 10.3109/17453679809002356
   LEITSCHUH PH, 1995, FOOT ANKLE INT, V16, P232, DOI 10.1177/107110079501600413
   Lubin J W, 2000, Am J Orthop (Belle Mead NJ), V29, P707
   LUGGER LJ, 1977, ZBL CHIR, V102, P1320
   Maffulli N, 2003, AM J SPORT MED, V31, P692, DOI 10.1177/03635465030310051001
   Maffulli N, 1999, CLIN J SPORT MED, V9, P157, DOI 10.1097/00042752-199907000-00007
   MARTIN JW, 1986, CLIN ORTHOP RELAT R, P216
   Pieper HG, 1998, INT J SPORTS MED, V19, P68, DOI 10.1055/s-2007-971883
   Sell S, 1996, ARCH ORTHOP TRAUM SU, V115, P28, DOI 10.1007/BF00453213
   SIMMONDS F A, 1957, Practitioner, V179, P56
NR 14
TC 5
Z9 5
U1 0
U2 2
PU BMC
PI LONDON
PA CAMPUS, 4 CRINAN ST, LONDON N1 9XW, ENGLAND
EI 1471-2474
J9 BMC MUSCULOSKEL DIS
JI BMC Musculoskelet. Disord.
PD JUL 27
PY 2006
VL 7
AR 59
DI 10.1186/1471-2474-7-59
PG 5
WC Orthopedics; Rheumatology
WE Science Citation Index Expanded (SCI-EXPANDED)
SC Orthopedics; Rheumatology
GA 078PV
UT WOS:000240116600001
PM 16872521
OA gold, Green Published, Green Accepted
DA 2022-07-22
ER

PT J
AU Dams, OC
   Reininga, IHF
   Zwerver, J
   Diercks, RL
   van den Akker-Scheek, I
AF Dams, Olivier C.
   Reininga, Inge H. F.
   Zwerver, Johannes
   Diercks, Ronald L.
   van den Akker-Scheek, Inge
TI The Achilles tendon Total Rupture Score is a responsive primary outcome
   measure: an evaluation of the Dutch version including minimally
   important change
SO KNEE SURGERY SPORTS TRAUMATOLOGY ARTHROSCOPY
LA English
DT Article
DE Achilles tendon rupture; PROM; Questionnaire; Epidemiology; ATRS; Dutch;
   Responsiveness; Minimally important change; Follow-up
ID CROSS-CULTURAL ADAPTATION; QUALITY-OF-LIFE; HEALTH-STATUS;
   CLINICAL-SIGNIFICANCE; VALIDATION; RELIABILITY; INSTRUMENTS; VALIDITY;
   COSMIN
AB Purpose Aim of this study was to evaluate the responsiveness of the Dutch version of the Achilles tendon Total Rupture Score (ATRS-NL). Methods Patients (N = 47) completed the ATRS-NL at 3 and 6 months after Achilles tendon rupture (ATR). Additionally, they filled out the Euroqol-5D-5L (EQ-5D-5L) and Global Rating of Change Score (GRoC). Effect sizes (ES) and standardized response means (SRM) were calculated. The anchor-based method for determining the minimally important change (MIC) was used. GRoC and improvement on the itemsmobilityandusual activitieson the EQ-5D-5L served as external criteria. The scores on these anchors were used to categorize patients' physical functioning asimprovedorunchangedbetween 3 and 6 months after ATR. Receiver operating curve (ROC) analysis was performed, with the calculation of the area under the ROC curve (AUC) and the estimation of MIC values using the optimal cut-off points. Results There was a large change (ES: 1.58) and good responsiveness (SRM: 1.19) of the ATRS-NL between 3 and 6 months after ATR. Using ROC analysis, the MIC values ranged from 13.5 to 28.5 for reporting improvement on EQ-5D-5Lmobilityand GRoC, respectively. The AUC of improvement on mobility and improvement on GRoC were > 0.70. Conclusion The ATRS-NL showed good responsiveness in ATR patients between 3 and 6 months after injury. Use of this questionnaire is recommended in clinical follow-up and longitudinal research of ATR patients. MIC values of 13.5 and 28.5 are recommended to consider ATR patients as improved and greatly improved between 3 and 6 months after ATR.
C1 [Dams, Olivier C.; Diercks, Ronald L.; van den Akker-Scheek, Inge] Univ Groningen, Univ Med Ctr Groningen, Dept Orthopaed Surg, Hanzepl 1, NL-9713 GZ Groningen, Netherlands.
   [Reininga, Inge H. F.] Univ Groningen, Univ Med Ctr Groningen, Dept Trauma Surg, Hanzepl 1, NL-9713 GZ Groningen, Netherlands.
   [Zwerver, Johannes] Univ Groningen, Univ Med Ctr Groningen, Dept Human Movement Sci, Hanzepl 1, NL-9713 GZ Groningen, Netherlands.
   [Zwerver, Johannes] Gelderse Vallei Hosp, Sports Valley, Willy Brandtlaan 10, NL-6716 RP Ede, Netherlands.
RP Dams, OC (通讯作者)，Univ Groningen, Univ Med Ctr Groningen, Dept Orthopaed Surg, Hanzepl 1, NL-9713 GZ Groningen, Netherlands.
EM O.C.Dams@umcg.nl
OI van den Akker-Scheek, Inge/0000-0002-1614-8419; Reininga,
   Inge/0000-0003-1341-845X; Diercks, Ronald/0000-0001-9873-208X; Zwerver,
   Johannes/0000-0002-8499-2806
CR Ansari NN, 2016, KNEE SURG SPORT TR A, V24, P1372, DOI 10.1007/s00167-015-3977-x
   Bakowski P, 2018, KNEE SURG SPORT TR A, V26, P2074, DOI 10.1007/s00167-017-4764-7
   Black N, 2013, BMJ-BRIT MED J, V346, DOI 10.1136/bmj.f167
   Buckinx F, 2020, FOOT ANKLE SURG, V26, P662, DOI 10.1016/j.fas.2019.08.010
   CAMPBELL DT, 1959, PSYCHOL BULL, V56, P81, DOI 10.1037/h0046016
   Carmont MR, 2013, KNEE SURG SPORT TR A, V21, P1356, DOI 10.1007/s00167-012-2146-8
   Cohen L, 1988, STAT POWER ANAL BEHA, V2, P1
   Crosby RD, 2003, J CLIN EPIDEMIOL, V56, P395, DOI 10.1016/S0895-4356(03)00044-1
   Cui J, 2017, HEALTH QUAL LIFE OUT, V15, DOI 10.1186/s12955-016-0574-8
   Dams OC, 2019, BMC MUSCULOSKEL DIS, V20, DOI 10.1186/s12891-019-2437-z
   Dams OC, 2019, KNEE SURG SPORT TR A, V27, P2754, DOI 10.1007/s00167-018-5049-5
   de Vet HC, 2006, HEALTH QUAL LIFE OUT, V4, DOI 10.1186/1477-7525-4-54
   de Vet HCW, 2007, QUAL LIFE RES, V16, P131, DOI 10.1007/s11136-006-9109-9
   de Vet HCW, 2010, J CLIN EPIDEMIOL, V63, P37, DOI 10.1016/j.jclinepi.2009.03.011
   DEYO RA, 1986, J CHRON DIS, V39, P897, DOI 10.1016/0021-9681(86)90038-X
   FLETCHER A, 1995, BRIT J CLIN PHARMACO, V39, P217, DOI 10.1111/j.1365-2125.1995.tb04439.x
   Gagnier JJ, 2017, J ORTHOP RES, V35, P2098, DOI 10.1002/jor.23604
   Ganestam A, 2013, J FOOT ANKLE SURG, V52, P736, DOI 10.1053/j.jfas.2013.07.004
   GUYATT G, 1987, J CHRON DIS, V40, P171, DOI 10.1016/0021-9681(87)90069-5
   Guyatt GH, 2002, MAYO CLIN PROC, V77, P371, DOI 10.4065/77.4.371
   Husted JA, 2000, J CLIN EPIDEMIOL, V53, P459, DOI 10.1016/S0895-4356(99)00206-1
   JAESCHKE R, 1989, CONTROL CLIN TRIALS, V10, P407, DOI 10.1016/0197-2456(89)90005-6
   Janssen MF, 2018, PHARMACOECONOMICS, V36, P675, DOI 10.1007/s40273-018-0623-8
   Jia YX, 2017, QUAL LIFE RES, V26, P1969, DOI 10.1007/s11136-017-1542-4
   JUNIPER EF, 1994, J CLIN EPIDEMIOL, V47, P81, DOI 10.1016/0895-4356(94)90036-1
   Kearney RS, 2012, BRIT J SPORT MED, V46, P1102, DOI 10.1136/bjsports-2011-090497
   Kearney RS, 2012, HEALTH QUAL LIFE OUT, V10, DOI 10.1186/1477-7525-10-24
   KIRSHNER B, 1985, J CHRON DIS, V38, P27, DOI 10.1016/0021-9681(85)90005-0
   Lamers LM, 2005, NED TIJDSCHR GENEES, V149, P1574
   LIANG MH, 1990, MED CARE, V28, P632, DOI 10.1097/00005650-199007000-00008
   Mokkink LB., 2019, COSMIN STUDY DESIGN
   Mokkink LB, 2016, BRAZ J PHYS THER, V20, P105, DOI 10.1590/bjpt-rbf.2014.0143
   Mokkink LB, 2010, J CLIN EPIDEMIOL, V63, P737, DOI 10.1016/j.jclinepi.2010.02.006
   Mokkink LB, 2010, QUAL LIFE RES, V19, P539, DOI 10.1007/s11136-010-9606-8
   Mutlu EK, 2015, KNEE SURG SPORT TR A, V23, P2427, DOI 10.1007/s00167-014-3042-1
   Myhrvold SB, 2018, KNEE SURG SPORT TR A, V26, P2045, DOI 10.1007/s00167-017-4689-1
   Nilsson-Helander K, 2007, AM J SPORT MED, V35, P421, DOI 10.1177/0363546506294856
   Norman GR, 2007, QUAL LIFE RES, V16, P815, DOI 10.1007/s11136-007-9180-x
   Norman GR, 2003, MED CARE, V41, P582, DOI 10.1097/00005650-200305000-00004
   Norman GR, 1997, J CLIN EPIDEMIOL, V50, P869, DOI 10.1016/S0895-4356(97)00097-8
   Ochen Y, 2019, BMJ-BRIT MED J, V364, DOI 10.1136/bmj.k5120
   Olsson N, 2011, KNEE SURG SPORT TR A, V19, P1385, DOI 10.1007/s00167-011-1511-3
   Opdam KTM, 2018, KNEE SURG SPORT TR A, V26, P862, DOI 10.1007/s00167-016-4242-7
   Revicki D, 2008, J CLIN EPIDEMIOL, V61, P102, DOI 10.1016/j.jclinepi.2007.03.012
   Revicki DA, 2006, HEALTH QUAL LIFE OUT, V4, DOI 10.1186/1477-7525-4-70
   Shultz S, 2013, INT J SPORTS PHYS TH, V8, P838
   Sierevelt IN, 2018, KNEE SURG SPORT TR A, V26, P2059, DOI 10.1007/s00167-017-4748-7
   Simundic Ana-Maria, 2009, EJIFCC, V19, P203
   Sloan JA, 2005, J CLIN EPIDEMIOL, V58, P1217, DOI 10.1016/j.jclinepi.2005.07.009
   STEWART AL, 1990, FRONT PR CA, P3
   Streiner D, 2014, HLTH MEASUREMENT SCA, P258
   Terwee C.B., 2011, MEASUREMENT MED, P202, DOI 10.1017/CBO9780511996214.011
   Terwee CB, 2007, J CLIN EPIDEMIOL, V60, P34, DOI 10.1016/j.jclinepi.2006.03.012
   Touzopoulos P, 2019, FOOT ANKLE SURG, V25, P8, DOI 10.1016/j.fas.2017.07.560
   Turner D, 2010, J CLIN EPIDEMIOL, V63, P28, DOI 10.1016/j.jclinepi.2009.01.024
   Vascellari A, 2018, KNEE SURG SPORT TR A, V26, P854, DOI 10.1007/s00167-016-4152-8
   Weel Hanneke, 2015, Ned Tijdschr Geneeskd, V159, pA8831
   Wyrwich KW, 2005, QUAL LIFE RES, V14, P285, DOI 10.1007/s11136-004-0705-2
   Zambelli R, 2016, BMC SPORTS SCI MED R, V8, DOI 10.1186/s13102-016-0034-0
   Zellers JA, 2016, BRIT J SPORT MED, V50, P1325, DOI 10.1136/bjsports-2016-096106
NR 60
TC 2
Z9 2
U1 0
U2 2
PU SPRINGER
PI NEW YORK
PA ONE NEW YORK PLAZA, SUITE 4600, NEW YORK, NY, UNITED STATES
SN 0942-2056
EI 1433-7347
J9 KNEE SURG SPORT TR A
JI Knee Surg. Sports Traumatol. Arthrosc.
PD OCT
PY 2020
VL 28
IS 10
BP 3330
EP 3338
DI 10.1007/s00167-020-05924-7
EA MAR 2020
PG 9
WC Orthopedics; Sport Sciences; Surgery
WE Science Citation Index Expanded (SCI-EXPANDED); Social Science Citation Index (SSCI)
SC Orthopedics; Sport Sciences; Surgery
GA NS2SG
UT WOS:000562485400001
PM 32146524
OA hybrid, Green Published
DA 2022-07-22
ER

PT J
AU Patel, MS
   Kadakia, AR
AF Patel, Milap S.
   Kadakia, Anish R.
TI Minimally Invasive Treatments of Acute Achilles Tendon Ruptures
SO FOOT AND ANKLE CLINICS
LA English
DT Article
DE Achilles rupture; Achilles repair; Minimally invasive Achilles repair;
   Percutaneous Achilles repair
ID NONOPERATIVE TREATMENT; NONSURGICAL TREATMENT; SURGICAL REPAIR; SURAL
   NERVE; PASSIVE MOBILIZATION; INCREASING INCIDENCE; PERCUTANEOUS REPAIR;
   PROSPECTIVE TRIAL; CALCANEAL TENDON; WEIGHT-BEARING
AB Achilles tendon rupture is a common injury to the lower extremity that requires appropriate treatment to minimize functional deficit. Available treatments of Achilles tendon ruptures include nonoperative, open surgical repair, percutaneous repair, and minimally invasive repair. Open surgical repair obtains favorable functional outcomes with significant potential for deep soft tissue complications, calling into question the value of open repair. Percutaneous repair is an alternative option with comparable functional results and minimal soft tissue complications; however, sural nerve injury is a complication. Minimally invasive Achilles repair offers optimal results with superior functional outcomes with minimal soft tissue complications and sural nerve injury.
C1 [Patel, Milap S.] Northwestern Univ, Feinberg Sch Med, Northwestern Mem Hosp, 259 East Erie,13th Floor, Chicago, IL 60611 USA.
   [Kadakia, Anish R.] Northwestern Univ, Feinberg Sch Med, Northwestern Mem Hosp, Dept Orthopaed Surg, 259 East Erie,13th Floor, Chicago, IL 60611 USA.
RP Kadakia, AR (通讯作者)，Northwestern Univ, Feinberg Sch Med, Northwestern Mem Hosp, Dept Orthopaed Surg, 259 East Erie,13th Floor, Chicago, IL 60611 USA.
EM kadak259@gmail.com
CR Aktas S, 2009, FOOT ANKLE INT, V30, P391, DOI 10.3113/FAI.2009.0391
   Alcelik I, 2018, FOOT ANKLE SURG, V24, P427, DOI 10.1016/j.fas.2017.04.016
   Amlang Michael H, 2006, Oper Orthop Traumatol, V18, P287, DOI 10.1007/s00064-006-1178-y
   Assal M, 2002, J BONE JOINT SURG AM, V84A, P161, DOI 10.2106/00004623-200202000-00001
   Avina Valencia Jorge Arturo, 2009, Acta Ortop Mex, V23, P125
   Barfod KW, 2015, J FOOT ANKLE SURG, V54, P220, DOI 10.1053/j.jfas.2014.11.018
   Bhandari M, 2002, CLIN ORTHOP RELAT R, P190
   Bhattacharyya M, 2009, INT ORTHOP, V33, P151, DOI 10.1007/s00264-008-0564-5
   Calder JDF, 2005, BRIT J SPORT MED, V39, P857, DOI 10.1136/bjsm.2004.017509
   CARDEN DG, 1987, J BONE JOINT SURG BR, V69, P416, DOI 10.1302/0301-620X.69B3.3294839
   Carmont M R, 2011, Foot Ankle Surg, V17, P186, DOI 10.1016/j.fas.2010.06.003
   CARR AJ, 1989, J BONE JOINT SURG BR, V71, P100, DOI 10.1302/0301-620X.71B1.2914976
   CETTI R, 1993, AM J SPORT MED, V21, P791, DOI 10.1177/036354659302100606
   Chen TM, 2009, CLIN ANAT, V22, P377, DOI 10.1002/ca.20758
   COOMBS RRH, 1981, J BONE JOINT SURG BR, V63, P288
   Costa ML, 2006, J BONE JOINT SURG BR, V88B, P69, DOI 10.1302/0301-620X.88B1.16549
   Cretnik A, 2000, MED SCI SPORT EXER, V32, P16
   Dekker RG, 2017, FOOT ANKLE ORTHOPAED, V2, P1, DOI DOI 10.1177/2473011417715431
   DELPONTE P, 1992, REV CHIR ORTHOP, V78, P404
   Demetracopoulos CA, 2014, FOOT ANKLE INT, V35, P612, DOI 10.1177/1071100714524550
   Eliasson P, 2012, J ORTHOP RES, V30, P274, DOI 10.1002/jor.21511
   ENWEMEKA CS, 1988, AM J PHYS MED REHAB, V67, P264
   GELBERMAN RH, 1982, J HAND SURG-AM, V7, P170, DOI 10.1016/S0363-5023(82)80083-X
   Goren D, 2005, FOOT ANKLE INT, V26, P286, DOI 10.1177/107110070502600404
   Guillo S, 2013, SURG-J R COLL SURG E, V11, P14, DOI 10.1016/j.surge.2011.12.002
   Gwynne-Jones DP, 2011, FOOT ANKLE INT, V32, P337, DOI 10.3113/FAI.2011.0337
   Haji A, 2004, FOOT ANKLE INT, V25, P215, DOI 10.1177/107110070402500404
   Hammerman M, 2014, J APPL PHYSIOL, V116, P54, DOI 10.1152/japplphysiol.00741.2013
   Heitman DE, 2011, FOOT ANKLE INT, V32, P879, DOI 10.3113/FAI.2011.0879
   HOCKENBURY RT, 1990, FOOT ANKLE, V11, P67, DOI 10.1177/107110079001100202
   Hsu AR, 2015, FOOT ANKLE INT, V36, P1279, DOI 10.1177/1071100715589632
   Huffard B, 2008, CLIN BIOMECH, V23, P1158, DOI 10.1016/j.clinbiomech.2008.05.007
   Hufner TM, 2006, FOOT ANKLE INT, V27, P167, DOI 10.1177/107110070602700302
   Huttunen TT, 2014, AM J SPORT MED, V42, P2419, DOI 10.1177/0363546514540599
   Ismail M, 2008, FOOT ANKLE INT, V29, P808, DOI 10.3113/FAI.2008.0808
   Jiang N, 2012, INT ORTHOP, V36, P765, DOI 10.1007/s00264-011-1431-3
   Jones Matthew P, 2012, J Bone Joint Surg Am, V94, pe88, DOI 10.2106/JBJS.J.01829
   JOZSA L, 1989, AM J SPORT MED, V17, P338, DOI 10.1177/036354658901700305
   KAKIUCHI M, 1995, J BONE JOINT SURG BR, V77B, P60, DOI 10.1302/0301-620X.77B1.7822398
   Keller A, 2014, AM J SPORT MED, V42, P731, DOI 10.1177/0363546513511418
   Khan RJK, 2005, J BONE JOINT SURG AM, V87A, P2202, DOI 10.2106/JBJS.D.03049
   KLEIN W, 1991, Chirurgia degli Organi di Movimento, V76, P223
   Kolodziej L, 2013, INT ORTHOP, V37, P625, DOI 10.1007/s00264-012-1737-9
   Kotnis R, 2006, AM J SPORT MED, V34, P1395, DOI 10.1177/0363546506288678
   Leppilahti J, 1996, ACTA ORTHOP SCAND, V67, P277, DOI 10.3109/17453679608994688
   Lo IKY, 1997, CLIN J SPORT MED, V7, P207, DOI 10.1097/00042752-199707000-00010
   MA GWC, 1977, CLIN ORTHOP RELAT R, P247
   Maes Renaud, 2006, Acta Orthop Belg, V72, P179
   Maffulli N, 1999, CLIN J SPORT MED, V9, P157, DOI 10.1097/00042752-199907000-00007
   Majewski M, 2006, AM J SPORT MED, V34, P793, DOI 10.1177/0363546505283266
   Majewski M, 2000, ORTHOPADE, V29, P670
   Mattila VM, 2015, BRIT J SPORT MED, V49, P1084, DOI 10.1136/bjsports-2013-092756
   McComis GP, 1997, J BONE JOINT SURG AM, V79A, P1799, DOI 10.2106/00004623-199712000-00005
   Metz R, 2008, AM J SPORT MED, V36, P1688, DOI 10.1177/0363546508319312
   MICHNA H, 1989, INT ORTHOP, V13, P161, DOI 10.1007/BF00268040
   Moller A, 1996, ACTA ORTHOP SCAND, V67, P479, DOI 10.3109/17453679608996672
   Moller M, 2001, J BONE JOINT SURG BR, V83B, P843
   Mortensen NHM, 1999, J BONE JOINT SURG AM, V81A, P983, DOI 10.2106/00004623-199907000-00011
   MOSLER E, 1985, J MOL BIOL, V182, P589, DOI 10.1016/0022-2836(85)90244-X
   Muller SA, 2018, AM J SPORT MED, V46, P1214, DOI 10.1177/0363546518756093
   Mullaney MJ, 2006, AM J SPORT MED, V34, P1120, DOI 10.1177/0363546505284186
   Nilsson-Helander K, 2010, AM J SPORT MED, V38, P2186, DOI 10.1177/0363546510376052
   NISTOR L, 1981, J BONE JOINT SURG AM, V63, P394, DOI 10.2106/00004623-198163030-00012
   Nyyssonen T, 2008, SCAND J SURG, V97, P272, DOI 10.1177/145749690809700312
   Olsson N, 2013, AM J SPORT MED, V41, P2867, DOI 10.1177/0363546513503282
   Porter KJ, 2014, FOOT ANKLE SURG, V20, P90, DOI 10.1016/j.fas.2013.11.005
   Poynton AR, 2001, FOOT ANKLE INT, V22, P572, DOI 10.1177/107110070102200707
   Rebeccato A, 2001, J Foot Ankle Surg, V40, P188
   ROWLEY DI, 1982, INJURY, V14, P252, DOI 10.1016/0020-1383(82)90138-3
   Rozis M, 2018, FOOT ANKLE INT, V39, P689, DOI 10.1177/1071100718757971
   SALEH M, 1992, J BONE JOINT SURG BR, V74, P206, DOI 10.1302/0301-620X.74B2.1544953
   SALTER RB, 1980, J BONE JOINT SURG AM, V62, P1232, DOI 10.2106/00004623-198062080-00002
   Schepull T, 2013, AM J SPORT MED, V41, P2550, DOI 10.1177/0363546513501785
   Soroceanu A, 2012, J BONE JOINT SURG AM, V94A, P2136, DOI 10.2106/JBJS.K.00917
   Twaddle BC, 2007, AM J SPORT MED, V35, P2033, DOI 10.1177/0363546507307503
   Wallace RGH, 2011, J BONE JOINT SURG BR, V93B, P1362, DOI 10.1302/0301-620X.93B10.26187
   Webb J, 2000, FOOT ANKLE INT, V21, P475, DOI 10.1177/107110070002100604
   Wilkins R, 2012, AM J SPORT MED, V40, P2154, DOI 10.1177/0363546512453293
   Willits K, 2010, J BONE JOINT SURG AM, V92A, P2767, DOI 10.2106/JBJS.I.01401
   WOO SLY, 1981, ACTA ORTHOP SCAND, V52, P615, DOI 10.3109/17453678108992156
   Yepes H, 2010, J BONE JOINT SURG AM, V92A, P1215, DOI 10.2106/JBJS.I.00743
   Young SW, 2014, J BONE JOINT SURG AM, V96A, P1073, DOI 10.2106/JBJS.M.00248
   Zhao HM, 2011, CHINESE MED J-PEKING, V124, P4050, DOI 10.3760/cma.j.issn.0366-6999.2011.23.038
NR 83
TC 12
Z9 14
U1 3
U2 5
PU W B SAUNDERS CO-ELSEVIER INC
PI PHILADELPHIA
PA 1600 JOHN F KENNEDY BOULEVARD, STE 1800, PHILADELPHIA, PA 19103-2899 USA
SN 1083-7515
EI 1558-1934
J9 FOOT ANKLE CLIN
JI Foot Ankle Clin.
PD SEP
PY 2019
VL 24
IS 3
BP 399
EP +
DI 10.1016/j.fcl.2019.05.002
PG 27
WC Orthopedics
WE Science Citation Index Expanded (SCI-EXPANDED)
SC Orthopedics
GA IX7JC
UT WOS:000485858600004
PM 31370993
DA 2022-07-22
ER

PT J
AU So, E
   Consul, D
   Holmes, T
AF So, Eric
   Consul, Devon
   Holmes, Timothy
TI Achilles Tendon Reconstruction With Bone Block Allograft: Long-Term
   Follow-Up of Two Cases
SO JOURNAL OF FOOT & ANKLE SURGERY
LA English
DT Article
DE cadaver; calcaneus; chronic injury; flexor hallucis longus; neglected
   rupture; triceps surae
ID RUPTURES
AB The treatment of neglected or chronically ruptured Achilles tendon is challenging. Various treatments for large defects associated with chronic Achilles ruptures have been described. Many surgeons recommend the use of a tendon transfer, turndown rotational flap, advancement flap, or reconstruction with Achilles tendon allograft with calcaneal bone block. Long-term outcomes of these procedures are unknown. We present 2 cases with the use of an Achilles tendon with calcaneus bone block allograft. At >8-year follow-up duration, both patients are afforded satisfactory levels of activity and are without pain or gait disturbance. This procedure is a viable option for Achilles ruptures with large defects, ruptures with small intact distal tendon portions, or re-ruptures of previously repaired Achilles tendons. The long-term outcomes of these case reports suggest that Achilles tendon reconstruction with bone block allograft is a viable option. (C) 2018 by the American College of Foot and Ankle Surgeons. All rights reserved.
C1 [So, Eric] Core Inst, Phoenix, AZ USA.
   [Consul, Devon; Holmes, Timothy] Grant Med Ctr, Columbus, OH USA.
RP So, E (通讯作者)，285 East State St,Suite 670, Columbus, OH 43215 USA.
EM encso70@gmail.com
CR Beals TC, 2010, J ORTHOP TRAUMA, V24, pE78, DOI 10.1097/BOT.0b013e3181c80a87
   Cetti R, 1995, AM J SPORTS MED, V23, P571
   Duhamel P, 2010, J BONE JOINT SURG AM, V92A, P2598, DOI 10.2106/JBJS.I.01578
   Hahn F, 2008, FOOT ANKLE INT, V29, P794, DOI 10.3113/FAI.2008.0794
   Hanna T, 2014, FOOT ANKLE INT, V35, P168, DOI 10.1177/1071100713514037
   Hansen U, 2010, J FOOT ANKLE SURG, V49, DOI 10.1053/j.jfas.2009.08.006
   INGLIS AE, 1976, J BONE JOINT SURG AM, V58, P990, DOI 10.2106/00004623-197658070-00015
   Kocabey Yavuz, 2006, J Foot Ankle Surg, V45, P42, DOI 10.1053/j.jfas.2005.10.009
   KUWADA G T, 1990, Journal of Foot Surgery, V29, P361
   Lepow Gary M, 2006, J Foot Ankle Surg, V45, P351, DOI 10.1053/j.jfas.2006.06.004
   Maffulli N, 2008, J BONE JOINT SURG AM, V90A, P1348, DOI 10.2106/JBJS.G.01241
   Myerson M S, 1999, Instr Course Lect, V48, P219
   Nellas Z J, 1996, J Foot Ankle Surg, V35, P144
   SILVER RL, 1985, J BONE JOINT SURG BR, V67, P432, DOI 10.1302/0301-620X.67B3.3997956
   Wegrzyn J, 2010, INT ORTHOP, V34, P1187, DOI 10.1007/s00264-009-0859-1
   Yuen JC, 2001, PLAST RECONSTR SURG, V107, P1807, DOI 10.1097/00006534-200106000-00028
NR 16
TC 2
Z9 2
U1 0
U2 0
PU ELSEVIER SCIENCE INC
PI NEW YORK
PA STE 800, 230 PARK AVE, NEW YORK, NY 10169 USA
SN 1067-2516
EI 1542-2224
J9 J FOOT ANKLE SURG
JI J. Foot Ankle Surg.
PD JUL
PY 2019
VL 58
IS 4
BP 779
EP 784
DI 10.1053/j.jfas.2018.11.023
PG 6
WC Orthopedics; Surgery
WE Science Citation Index Expanded (SCI-EXPANDED)
SC Orthopedics; Surgery
GA IF3UQ
UT WOS:000473009000032
PM 31010771
DA 2022-07-22
ER

PT J
AU Bevilacqua, NJ
AF Bevilacqua, Nicholas J.
TI Treatment of the Neglected Achilles Tendon Rupture
SO CLINICS IN PODIATRIC MEDICINE AND SURGERY
LA English
DT Article
DE Achilles; Neglected; Chronic rupture; FHL transfer
ID REPAIR; RECONSTRUCTION
AB Achilles tendon ruptures are best managed acutely. Neglected Achilles tendon ruptures are debilitating injuries and the increased complexity of the situation must be appreciated. Surgical management is recommended, and only in the poorest surgical candidate is conservative treatment entertained. Numerous treatment algorithms and surgical techniques have been described. A V-Y advancement flap and flexor halluces longus tendon transfer have been found to be reliable and achieve good clinical outcomes for defects ranging from 2 cm to 8 cm. This article focuses on the treatment options for the neglected Achilles tendon rupture.
C1 N Jersey Orthopaed Specialists, Teaneck, NJ 07666 USA.
RP Bevilacqua, NJ (通讯作者)，N Jersey Orthopaed Specialists, 730 Palisade Ave, Teaneck, NJ 07666 USA.
EM Nicholas.bevilacgua@gmail.com
CR ABRAHAM E, 1975, J BONE JOINT SURG AM, VA 57, P253, DOI 10.2106/00004623-197557020-00019
   BOSWORTH DM, 1956, J BONE JOINT SURG AM, V38, P111, DOI 10.2106/00004623-195638010-00011
   Cottom James M, 2008, Foot Ankle Spec, V1, P280, DOI 10.1177/1938640008322690
   Den Hartog BD, 2008, FOOT ANKLE INT, V29, P456, DOI 10.3113/FAI.2008.0456
   DENT CM, 1991, INJURY, V22, P239, DOI 10.1016/0020-1383(91)90054-I
   Elias I, 2007, FOOT ANKLE INT, V28, P1238, DOI 10.3113/FAI.2007.1238
   GABEL S, 1994, FOOT ANKLE INT, V15, P512, DOI 10.1177/107110079401500912
   Gebauer M, 2007, ARCH ORTHOP TRAUM SU, V127, P795, DOI 10.1007/s00402-007-0325-8
   HAINES JF, 1983, ANN RHEUM DIS, V42, P652, DOI 10.1136/ard.42.6.652
   Herbst SA, 2006, FOOT ANKLE INT, V27, P639, DOI 10.1177/107110070602700814
   Jennings AG, 2002, J BONE JOINT SURG BR, V84B, P361, DOI 10.1302/0301-620X.84B3.11559
   KLEINMAN M, 1983, J BONE JOINT SURG AM, V65, P1345, DOI 10.2106/00004623-198365090-00019
   KUWADA G T, 1990, Journal of Foot Surgery, V29, P361
   Leslie Hamish D H, 2005, Foot Ankle Clin, V10, P357, DOI 10.1016/j.fcl.2005.01.009
   Lin JL, 2009, FOOT ANKLE CLIN, V14, P729, DOI 10.1016/j.fcl.2009.08.007
   Mahajan Ravindra H, 2009, J Orthop Surg (Hong Kong), V17, P194
   MAHONEY PG, 1981, ANN RHEUM DIS, V40, P416, DOI 10.1136/ard.40.4.416
   MANN RA, 1991, J BONE JOINT SURG AM, V73A, P214, DOI 10.2106/00004623-199173020-00009
   Matles A L, 1975, Bull Hosp Joint Dis, V36, P48
   Padanilam TG, 2009, FOOT ANKLE CLIN, V14, P711, DOI 10.1016/j.fcl.2009.08.001
   Pintore E, 2001, J TRAUMA, V50, P71, DOI 10.1097/00005373-200101000-00013
   Porter DA, 1997, FOOT ANKLE INT, V18, P557, DOI 10.1177/107110079701800905
   RASK MR, 1978, JAMA-J AM MED ASSOC, V239, P435, DOI 10.1001/jama.239.5.435
   Saltzman C L, 1998, J Am Acad Orthop Surg, V6, P316
   SCHELLER AD, 1980, ORTHOP CLIN N AM, V11, P801
   Schuberth J, 1996, FOOT ANKLE TRAUMA AC
   SOMA CA, 1994, CLIN SPORT MED, V13, P811
   STROCCHI R, 1991, FOOT ANKLE, V12, P100, DOI 10.1177/107110079101200207
   Takao M, 2003, ARCH ORTHOP TRAUM SU, V123, P471, DOI 10.1007/s00402-002-0443-2
   Thompson J, 2011, CLIN PODIATR MED SUR, V28, P117, DOI 10.1016/j.cpm.2010.10.002
   THOMPSON T C, 1962, J Trauma, V2, P126, DOI 10.1097/00005373-196203000-00003
   Us AK, 1997, ARCH ORTHOP TRAUM SU, V116, P408
NR 32
TC 9
Z9 9
U1 0
U2 2
PU W B SAUNDERS CO-ELSEVIER INC
PI PHILADELPHIA
PA 1600 JOHN F KENNEDY BOULEVARD, STE 1800, PHILADELPHIA, PA 19103-2899 USA
SN 0891-8422
EI 1558-2302
J9 CLIN PODIATR MED SUR
JI Clin. Podiatr. Med. Surg.
PD APR
PY 2012
VL 29
IS 2
BP 291
EP +
DI 10.1016/j.cpm.2012.01.004
PG 10
WC Orthopedics
WE Science Citation Index Expanded (SCI-EXPANDED)
SC Orthopedics
GA 044VC
UT WOS:000311651200009
PM 22424489
DA 2022-07-22
ER

PT J
AU Kearney, RS
   Costa, ML
AF Kearney, Rebecca S.
   Costa, Matthew L.
TI Collagen-Matrix Allograft Augmentation of Bilateral Rupture of the
   Achilles Tendon
SO FOOT & ANKLE INTERNATIONAL
LA English
DT Article
DE Achilles Tendon; Rupture; Rehabilitation
ID REPAIR
C1 [Kearney, Rebecca S.] Univ Warwick, Warwick Med Sch, Clin Sci Res Inst, Coventry CV2 2DX, W Midlands, England.
RP Kearney, RS (通讯作者)，Univ Warwick, Warwick Med Sch, Clin Sci Res Inst, Clifford Bridge Rd, Coventry CV2 2DX, W Midlands, England.
EM R.S.Kearney@Warwick.ac.uk
OI Kearney, Rebecca/0000-0002-8010-164X
CR ABRAHAM E, 1975, J BONE JOINT SURG AM, VA 57, P253, DOI 10.2106/00004623-197557020-00019
   Barnett S, 1998, DIABETIC FOOT, V1, P137
   Beniker D, 2003, ORTHOPEDICS, V26, pS591
   Costa ML, 2005, J BONE JOINT SURG BR, V87B, P1085, DOI 10.1302/0301-620X.87B8.16540
   Hahn F, 2008, CLIN BIOMECH, V23, P109, DOI 10.1016/j.clinbiomech.2007.08.015
   HOWARD CB, 1984, J BONE JOINT SURG BR, V66, P206, DOI 10.1302/0301-620X.66B2.6707056
   Jennings AG, 2002, J BONE JOINT SURG BR, V84B, P361, DOI 10.1302/0301-620X.84B3.11559
   LEE DK, 2007, J FOOT ANKLE SURG, P451
   Maffulli N, 2005, CLIN J SPORT MED, V15, P56, DOI 10.1097/01.jsm.0000152714.05097.ef
   MANN RA, 1991, J BONE JOINT SURG AM, V73A, P214, DOI 10.2106/00004623-199173020-00009
   MISKULIN M, 2005, J FOOT ANKLE SURG, P49
   Muermans S, 1998, J HAND SURG-BRIT EUR, V23B, P64, DOI 10.1016/S0266-7681(98)80222-2
   OZAKI J, 1989, CLIN ORTHOP RELAT R, P204
   SALEN BA, 1994, J CLIN EPIDEMIOL, V47, P1423, DOI 10.1016/0895-4356(94)90086-8
   Takao M, 2003, ARCH ORTHOP TRAUM SU, V123, P471, DOI 10.1007/s00402-002-0443-2
   WAPNER KL, 1993, FOOT ANKLE, P443
   WILLIAMS A, 1990, HEALTH POLICY, V16, P199
NR 17
TC 4
Z9 5
U1 0
U2 2
PU SAGE PUBLICATIONS INC
PI THOUSAND OAKS
PA 2455 TELLER RD, THOUSAND OAKS, CA 91320 USA
SN 1071-1007
EI 1944-7876
J9 FOOT ANKLE INT
JI Foot Ankle Int.
PD JUN
PY 2010
VL 31
IS 6
BP 556
EP 559
DI 10.3113/FAI.2010.0556
PG 4
WC Orthopedics
WE Science Citation Index Expanded (SCI-EXPANDED)
SC Orthopedics
GA 608RN
UT WOS:000278603800018
PM 20557825
DA 2022-07-22
ER

PT J
AU Li, CG
   Li, B
   Yang, YF
AF Li, Chun-Guang
   Li, Bing
   Yang, Yun-Feng
TI Management of acute Achilles tendon rupture with tendon-bundle technique
SO JOURNAL OF INTERNATIONAL MEDICAL RESEARCH
LA English
DT Article
DE Achilles tendon; rupture; tendon-bundle
ID OPEN REPAIR; SURGICAL REPAIR; SUTURE; AUGMENTATION; PROTOCOL; FLAP
AB Objective: To explore tendon-bundle technique for treating Achilles tendon rupture with no defects.
   Methods: Patients with full unilateral Achilles tendon rupture with no defects were included. The Achilles tendon medial edge surgical repair approach was used, revealing horsetail-like rupture bundles. Tendon bundles were anatomically realigned and repaired end-to-end using 5-0 sutures. Patients were followed-up for 1 year, and assessed for differences between the repaired versus healthy limb.
   Results: Out of 24 patients ( 18 male, 6 female; aged 19-56 years) at 1 year following surgery, mean American Orthopaedic Foot and Ankle Society score was 92.4 +/- 5.9; mean differences between the surgically repaired versus contralateral side in dorsiflexion and plantarflexion angle were 3.5 +/- 2.3 degrees and 5.6 +/- 3.2 degrees, respectively; mean difference in calf circumference between the two sides was 0.9 +/- 0.5 cm; and mean increase in Achilles tendon width versus the healthy side was 0.8 +/- 0.2 cm. By 1 year post-surgery, there were no significant between-side differences in dorsiflexion and plantarflexion angle, or calf circumference.
   Conclusions: Tendon-bundle surgery resulted in good ankle function restoration and low complication rates. Tendon-bundle surgery may reduce blood supply destruction and maximally preserve Achilles tendon length, and may be effective for treating Achilles tendon rupture with no defects.
C1 [Li, Chun-Guang; Li, Bing; Yang, Yun-Feng] Tongji Univ, Sch Med, Tongji Hosp, Dept Orthopaed, 389 Xincun Rd, Shanghai 200065, Peoples R China.
RP Yang, YF (通讯作者)，Tongji Univ, Sch Med, Tongji Hosp, Dept Orthopaed, 389 Xincun Rd, Shanghai 200065, Peoples R China.
EM 15216719674@163.com
FU National Natural Science Foundation of China [81472144]
FX This study was supported by the National Natural Science Foundation of
   China (81472144).
CR Assal M, 2002, J BONE JOINT SURG AM, V84A, P161, DOI 10.2106/00004623-200202000-00001
   Barber FA, 2008, FOOT ANKLE INT, V29, P329, DOI 10.3113/FAI.2008.0329
   Bevoni R, 2014, INJURY, V45, P1268, DOI 10.1016/j.injury.2014.04.028
   CARR AJ, 1989, J BONE JOINT SURG BR, V71, P100, DOI 10.1302/0301-620X.71B1.2914976
   Ceccarelli F, 2007, CLIN ORTHOP RELAT R, P188, DOI 10.1097/BLO.0b013e3180396f07
   Chiodo CP, 2010, J BONE JOINT SURG AM, V92A, P2466
   Chiodo CP, 2010, J AM ACAD ORTHOP SUR, V18, P503, DOI 10.5435/00124635-201008000-00007
   Corradino B, 2015, INJURY, V46, P1637, DOI 10.1016/j.injury.2015.05.014
   Gigante A, 2008, KNEE SURG SPORT TR A, V16, P204, DOI 10.1007/s00167-007-0448-z
   Halasi T, 2003, KNEE SURG SPORT TR A, V11, P409, DOI 10.1007/s00167-003-0397-0
   Holm C, 2015, SCAND J MED SCI SPOR, V25, pE1, DOI 10.1111/sms.12209
   Jaakkola JI, 2000, FOOT ANKLE INT, V21, P14, DOI 10.1177/107110070002100103
   Jones Matthew P, 2012, J Bone Joint Surg Am, V94, pe88, DOI 10.2106/JBJS.J.01829
   KITAOKA HB, 1994, FOOT ANKLE INT, V15, P349, DOI 10.1177/107110079401500701
   Lansdaal JR, 2007, INJURY, V38, P839, DOI 10.1016/j.injury.2006.12.010
   Lim J, 2001, FOOT ANKLE INT, V22, P559, DOI 10.1177/107110070102200705
   MA GWC, 1977, CLIN ORTHOP RELAT R, P247
   McMahon Samuel E, 2011, Foot Ankle Surg, V17, P211, DOI 10.1016/j.fas.2010.11.001
   Moller M, 2001, KNEE SURG SPORT TR A, V9, P228, DOI 10.1007/s001670000185
   Nilsson-Helander K, 2010, AM J SPORT MED, V38, P2186, DOI 10.1177/0363546510376052
   NISTOR L, 1981, J BONE JOINT SURG AM, V63, P394, DOI 10.2106/00004623-198163030-00012
   Nyyssonen T, 2003, ACTA ORTHOP SCAND, V74, P206, DOI 10.1080/00016470310013978
   Ozer H, 2016, J FOOT ANKLE SURG, V55, P1180, DOI 10.1053/j.jfas.2016.07.005
   Rebeccato A, 2001, J Foot Ankle Surg, V40, P188
   Reiter M, 2004, INT J SPORTS MED, V25, P301, DOI 10.1055/s-2004-815828
   Riedl S, 2002, CHIRURG, V73, P607, DOI 10.1007/s00104-002-0452-2
   Rippstein Pascal F, 2002, Foot Ankle Clin, V7, P611, DOI 10.1016/S1083-7515(02)00040-2
   Rosenzweig S, 2009, FOOT ANKLE CLIN, V14, P699, DOI 10.1016/j.fcl.2009.07.002
   Schatzker J, 1969, Acta Orthop Scand Suppl, V126, P1
   Smith DW, 2013, WIRES SYST BIOL MED, V5, P523, DOI 10.1002/wsbm.1229
   Stein BE, 2015, INT ORTHOP, V39, P901, DOI 10.1007/s00264-015-2725-7
   Thermann H, 2001, FOOT ANKLE INT, V22, P158, DOI 10.1177/107110070102200214
   Twaddle BC, 2007, AM J SPORT MED, V35, P2033, DOI 10.1177/0363546507307503
   Weber M, 2003, AM J SPORT MED, V31, P685, DOI 10.1177/03635465030310050901
NR 34
TC 5
Z9 6
U1 0
U2 12
PU SAGE PUBLICATIONS LTD
PI LONDON
PA 1 OLIVERS YARD, 55 CITY ROAD, LONDON EC1Y 1SP, ENGLAND
SN 0300-0605
EI 1473-2300
J9 J INT MED RES
JI J. Int. Med. Res.
PD FEB
PY 2017
VL 45
IS 1
BP 310
EP 319
DI 10.1177/0300060516677928
PG 10
WC Medicine, Research & Experimental; Pharmacology & Pharmacy
WE Science Citation Index Expanded (SCI-EXPANDED)
SC Research & Experimental Medicine; Pharmacology & Pharmacy
GA EL3KF
UT WOS:000394517300030
PM 28222622
OA gold, Green Published
DA 2022-07-22
ER

PT J
AU Dams, OC
   van den Akker-Scheek, I
   Diercks, RL
   Wendt, KW
   Bosma, E
   van Raaij, TM
   Munzebrock, AV
   Zijlstra, WP
   Zwerver, J
   Reininga, IHF
AF Dams, Olivier C.
   van den Akker-Scheek, Inge
   Diercks, Ron L.
   Wendt, Klaus W.
   Bosma, Eelke
   van Raaij, Tom M.
   Munzebrock, Arvid V.
   Zijlstra, Wierd P.
   Zwerver, Johannes
   Reininga, Inge H. F.
TI The recovery after Achilles tendon rupture: a protocol for a multicenter
   prospective cohort study
SO BMC MUSCULOSKELETAL DISORDERS
LA English
DT Article
DE Achilles tendon rupture; PROM; Ultrasound tissue characterization;
   Prospective cohort; Multicenter; Rehabilitation; Economic;
   Shared-decision making
ID SURGICAL REPAIR; PSYCHOLOGICAL READINESS; INCREASING INCIDENCE; RETURN;
   REHABILITATION; MANAGEMENT; INJURIES; COMPLICATIONS; EPIDEMIOLOGY;
   METAANALYSIS
AB BackgroundAchilles tendon rupture (ATR) is a common sports injury, with a rising incidence and significant impairments. Due to the lack of treatment guidelines, there is no consensus about diagnostic methods, primary treatment (non-surgical or surgical) and rehabilitation. It is hypothesized that this lack of consensus and guidelines leads to sub-optimal recovery and higher societal costs.The primary aim of this study is to give a broad insight into the recovery after ATR. Secondarily this study aims to explore factors contributing to recovery and gain insight into the cost-effectiveness of ATR management.MethodsThis multicenter prospective cohort study will include all adult ( 18years) patients with an ATR treated at the three main hospitals in the Northern Netherlands: University Medical Center Groningen, Martini Hospital Groningen and Medical Center Leeuwarden. All subjects will be invited for three visits at 3, 6 and 12months post-injury. The following data will be collected: patient-reported outcome measures (PROMs), physical tests, imaging and economic questionnaires. At 3months post-injury personal, injury, and treatment data will be collected through a baseline questionnaire and assessment of the medical file. The PROMs concern the Dutch version of the Achilles Tendon Total Rupture Score, EQ-5D-5L, Oslo Sport Trauma Research Center Overuse Injury Questionnaire, Injury Psychological Readiness Return to Sport Scale, Tampa Scale of Kinesiophobia, Expectations, Motivation and Satisfaction questionnaire and a ranking of reasons for not returning to sport. The administered physical tests are the heel-rise test, standing dorsiflexion range of motion, resting tendon length and single leg hop for distance. Ultrasound Tissue Characterization will be used for imaging. Finally, economic data will be collected using the Productivity Cost Questionnaire and Medical Consumption Questionnaire.DiscussionThis prospective cohort study will contribute to optimal decision making in the primary treatment and rehabilitation of ATRs by providing insight into (1) ATR recovery (2) novel imaging for monitoring recovery (3) (barriers to) return to sport and (4) cost-effectiveness of management. The analysis of these data strives to give a broad insight into the recovery after ATR as well as provide data on novel imaging and costs, contributing to individualized ATR management.Trial registrationTrialregister.nl. NTR6484. 20/06/2017. 20/07/2017.
C1 [Dams, Olivier C.; Zwerver, Johannes] Univ Groningen, Univ Med Ctr Groningen, Dept Sport & Exercise Med, Groningen, Netherlands.
   [Dams, Olivier C.; van den Akker-Scheek, Inge; Diercks, Ron L.] Univ Groningen, Univ Med Ctr Groningen, Dept Orthoped, Groningen, Netherlands.
   [Wendt, Klaus W.] Univ Groningen, Univ Med Ctr Groningen, Dept Trauma Surg, Groningen, Netherlands.
   [Bosma, Eelke; Reininga, Inge H. F.] Martini Hosp, Dept Surg, Groningen, Netherlands.
   [van Raaij, Tom M.] Martini Hosp, Dept Orthopaed Surg, Groningen, Netherlands.
   [Munzebrock, Arvid V.] Med Ctr Leeuwarden, Dept Surg, Leeuwarden, Netherlands.
   [Zijlstra, Wierd P.] Med Ctr Leeuwarden, Dept Orthopaed Surg, Leeuwarden, Netherlands.
RP Dams, OC (通讯作者)，Univ Groningen, Univ Med Ctr Groningen, Dept Sport & Exercise Med, Groningen, Netherlands.; Dams, OC (通讯作者)，Univ Groningen, Univ Med Ctr Groningen, Dept Orthoped, Groningen, Netherlands.
EM o.c.dams@umcg.nl
RI Zijlstra, Wierd/ABB-9018-2020; van+Raaij, Tom/AFS-4556-2022; Zijlstra,
   Wierd/AFM-2737-2022; Reininga, Inge H.F./H-4250-2013
OI Reininga, Inge H.F./0000-0003-1341-845X; Diercks,
   Ronald/0000-0001-9873-208X; Zwerver, Johannes/0000-0002-8499-2806; van
   den Akker-Scheek, Inge/0000-0002-1614-8419
CR Ardern CL, 2014, BRIT J SPORT MED, V48, P1613, DOI 10.1136/bjsports-2014-093842
   Beaton DE, 2000, SPINE, V25, P3186, DOI 10.1097/00007632-200012150-00014
   Bolgla LA, 1997, J ORTHOP SPORT PHYS, V26, P138, DOI 10.2519/jospt.1997.26.3.138
   Bouwmans C, 2015, VALUE HEALTH, V18, P753, DOI 10.1016/j.jval.2015.05.009
   Brumann M, 2014, INJURY, V45, P1782, DOI 10.1016/j.injury.2014.06.022
   Carmont MR, 2017, ORTHOP J SPORTS MED, V5, DOI 10.1177/2325967117723347
   Carmont MR, 2013, ORTHOP J SPORTS MED, V1, DOI 10.1177/2325967113494584
   Carmont MR, 2015, ASIA-PAC J SPORT MED, V2, P49, DOI 10.1016/j.asmart.2014.12.002
   Chiodo CP, 2010, J BONE JOINT SURG AM, V92A, P2466
   Claessen FMAP, 2014, SPORTS MED, V44, P1241, DOI 10.1007/s40279-014-0200-z
   Clarsen B, 2013, BRIT J SPORT MED, V47, P495, DOI 10.1136/bjsports-2012-091524
   Dams OC, 2019, KNEE SURG SPORT TR A, V27, P2754, DOI 10.1007/s00167-018-5049-5
   Dams OC, 2017, INJURY, V48, P2383, DOI 10.1016/j.injury.2017.09.013
   Ecker TM, 2016, AM J SPORT MED, V44, P1004, DOI 10.1177/0363546515623501
   EKSTRAND J, 1982, ARCH PHYS MED REHAB, V63, P171
   Erickson BJ, 2014, ORTHOP J SPORTS MED, V2, DOI 10.1177/2325967114549948
   Fox G, 2016, INJURY, V47, P2370, DOI 10.1016/j.injury.2016.07.013
   Freedman Benjamin R, 2014, Muscles Ligaments Tendons J, V4, P245
   Ganestam A, 2016, KNEE SURG SPORT TR A, V24, P3730, DOI 10.1007/s00167-015-3544-5
   Glazer DD, 2009, J ATHL TRAINING, V44, P185, DOI 10.4085/1062-6050-44.2.185
   HARRELL FE, 1984, STAT MED, V3, P143, DOI 10.1002/sim.4780030207
   Harrell FE, 1996, STAT MED, V15, P361, DOI 10.1002/(SICI)1097-0258(19960229)15:4<361::AID-SIM168>3.0.CO;2-4
   Ho G, 2017, INJURY, V48, P1701, DOI 10.1016/j.injury.2017.04.007
   Holm C, 2015, SCAND J MED SCI SPOR, V25, pE1, DOI 10.1111/sms.12209
   Horstmann T, 2012, INT J SPORTS MED, V33, P474, DOI 10.1055/s-0032-1301932
   Houshian S, 1998, INJURY, V29, P651, DOI 10.1016/S0020-1383(98)00147-8
   Huang JZ, 2015, AM J SPORT MED, V43, P1008, DOI 10.1177/0363546514531014
   Hutchison AM, 2015, BONE JOINT J, V97B, P510, DOI 10.1302/0301-620X.97B4.35314
   Huttunen TT, 2014, AM J SPORT MED, V42, P2419, DOI 10.1177/0363546514540599
   Kori SH., 1990, PAIN MANAG, V3, P35, DOI [10.1007/978-3-540-29805-2_4377, DOI 10.1007/978-3-540-29805-2_4377]
   KVIST M, 1994, SPORTS MED, V18, P173, DOI 10.2165/00007256-199418030-00004
   Lamers LM, 2005, NEDERLANDSE TIJDSCHR, V149, P5
   Lantto I, 2015, AM J SPORTS MED
   Lawrence JE, 2017, BONE JOINT J, V99B, P87, DOI 10.1302/0301-620X.99B1.BJJ-2016-0452.R1
   Leppilahti J, 1996, ACTA ORTHOP SCAND, V67, P277, DOI 10.3109/17453679608994688
   Maffulli N, 1999, J BONE JOINT SURG AM, V81A, P1019, DOI 10.2106/00004623-199907000-00017
   Maffulli N, 1999, CLIN J SPORT MED, V9, P157, DOI 10.1097/00042752-199907000-00007
   Maffulli N, 2008, J BONE JOINT SURG AM, V90A, P1348, DOI 10.2106/JBJS.G.01241
   Maffulli N, 2006, AUST J PHYSIOTHER, V52, P225, DOI 10.1016/S0004-9514(06)70033-2
   Mark-Christensen T, 2016, KNEE SURG SPORT TR A, V24, P1852, DOI 10.1007/s00167-014-3180-5
   McCormack R, 2015, BRIT J SPORT MED, V49, P1329, DOI 10.1136/bjsports-2015-094935
   Metz R, 2011, AM J SPORT MED, V39, P820, DOI 10.1177/0363546510392012
   Moller A, 1996, ACTA ORTHOP SCAND, V67, P479, DOI 10.3109/17453679608996672
   Moller M, 2005, KNEE SURG SPORT TR A, V13, P60, DOI 10.1007/s00167-003-0441-0
   Nilsson-Helander K, 2007, AM J SPORT MED, V35, P421, DOI 10.1177/0363546506294856
   Nyyssonen T, 2008, SCAND J SURG, V97, P272, DOI 10.1177/145749690809700312
   Opdam KTM, 2016, KNEE SURG SPORT TR A, V26, P1
   Pajala A, 2009, J BONE JOINT SURG AM, V91A, P1092, DOI 10.2106/JBJS.G.01089
   Peduzzi P, 1996, J CLIN EPIDEMIOL, V49, P1373, DOI 10.1016/S0895-4356(96)00236-3
   Raikin SM, 2013, FOOT ANKLE INT, V34, P475, DOI 10.1177/1071100713477621
   Silbernagel KG, 2010, KNEE SURG SPORT TR A, V18, P258, DOI 10.1007/s00167-009-0889-7
   Slagers AJ, 2019, J SPORT SCI, V37, P1038, DOI 10.1080/02640414.2018.1540101
   Sonesson S, 2016, KNEE SURG SPORT TR A, P1
   Trofa DP, 2017, AM J SPORT MED, V45, P2864, DOI 10.1177/0363546517713001
   van Ark M, 2016, SCAND J MED SCI SPOR, V26, P189, DOI 10.1111/sms.12426
   van Schie HTM, 2010, BRIT J SPORT MED, V44, P1153, DOI 10.1136/bjsm.2009.061010
   VLAEYEN JWS, 1995, PAIN, V62, P363, DOI 10.1016/0304-3959(94)00279-N
   Wang KC, 2017, OPER TECHN SPORT MED, V25, P214, DOI 10.1053/j.otsm.2017.07.009
   Zellers JA, 2016, BRIT J SPORT MED, V50, P1325, DOI 10.1136/bjsports-2016-096106
NR 59
TC 7
Z9 7
U1 1
U2 8
PU BMC
PI LONDON
PA CAMPUS, 4 CRINAN ST, LONDON N1 9XW, ENGLAND
SN 1471-2474
J9 BMC MUSCULOSKEL DIS
JI BMC Musculoskelet. Disord.
PD FEB 11
PY 2019
VL 20
AR 69
DI 10.1186/s12891-019-2437-z
PG 8
WC Orthopedics; Rheumatology
WE Science Citation Index Expanded (SCI-EXPANDED)
SC Orthopedics; Rheumatology
GA HL0MP
UT WOS:000458388900001
PM 30744626
OA Green Published, gold
DA 2022-07-22
ER

PT J
AU Longo, UG
   Salvatore, G
   Ambrogioni, LR
   Cella, E
   Candela, V
   Carnevale, A
   Schena, E
   Ciccozzi, M
   Maffulli, N
   Denaro, V
AF Longo, U. G.
   Salvatore, Giuseppe
   Risi Ambrogioni, Laura
   Cella, Eleonora
   Candela, Vincenzo
   Carnevale, Arianna
   Schena, Emiliano
   Ciccozzi, Massimo
   Maffulli, Nicola
   Denaro, Vincenzo
TI Epidemiology of Achilles tendon surgery in Italy: a nationwide registry
   study, from 2001 through 2015
SO BMC MUSCULOSKELETAL DISORDERS
LA English
DT Article
DE Achilles tendon; Rupture; Registry; Surgery; Epidemiology; Prevalence
ID INVASIVE RECONSTRUCTION; RUPTURES; TENDINOPATHY; MANAGEMENT; GRAFT
AB Background This study aims (1) to estimate the yearly number of Achilles tendon (AT) surgeries in Italy from 2001 to 2015 based on official hospitalization records; (2) to investigate the eventual presence of geographical variation in equity in access to AT surgery between three macroregions of Italy (North, Center and South); (3) to perform statistical projections of the number of AT procedure volumes and rates based on these data. Methods We analysed the National Hospital Discharge records (SDO) maintained at the Italian Ministry of Health for a 15-year period, from 2001 through 2015. These data are anonymous and include the patient's age (evaluated in the class of age), sex, census region, the region of hospitalization, length of the hospitalization, public or private reimbursement and diagnosis. Results During the 15-year study period, 118,652 AT repair were performed in Italy, whose peak of incidence was in 2010. More than half of AT repairs was performed in the North of Italy (52.1%), while 27.2% was performed in the South of Italy and 20.6% Center of Italy. The projection model predicted a slight growth of 2.65% in 2025 in comparison with 2015. Conclusion The current study provides detailed information about the national population-weighted incidence of AT surgery, distribution and projection. The peak of average age was 35-45 year. The majority of AT procedures was performed in the North of Italy. The projection model predicts a slight growth of AT surgery by 2025. Furthermore, this 15-year nationwide registry study shows that the age of incidence of AT injuries shifted from 30 to 40 to 35-45 years compared to the available literature. The higher prevalence of AT surgery was found in men during the working age. Moreover, a low rate of procedures in pediatric and elder age classes was observed.
C1 [Longo, U. G.; Salvatore, Giuseppe; Risi Ambrogioni, Laura; Denaro, Vincenzo] Campus Biomed Univ, Dept Orthopaed & Trauma Surg, Via Alvaro del Portillo 200, I-00128 Rome, Italy.
   [Cella, Eleonora; Candela, Vincenzo; Ciccozzi, Massimo] Campus Biomed Univ, Med Stat & Mol Epidemiol, Via Alvaro del Portillo 200, I-00128 Rome, Italy.
   [Carnevale, Arianna; Schena, Emiliano] Campus Biomed Univ Rome, Unit Measurements & Biomed Instrumentat, Rome, Italy.
   [Maffulli, Nicola] Mile End Hosp, Barts & London Sch Med & Dent, Ctr Sports & Exercise Med, 275 Bancroft Rd, London E1 4DG, England.
   [Maffulli, Nicola] Univ Salerno, Sch Med Surg & Dent, Dept Musculoskeletal Surg, I-84121 Salerno, Italy.
RP Longo, UG (通讯作者)，Campus Biomed Univ, Dept Orthopaed & Trauma Surg, Via Alvaro del Portillo 200, I-00128 Rome, Italy.
EM g.longo@unicampus.it
RI Ciccozzi, Massimo/AAC-3271-2022; Salvatore, Giuseppe/AAC-5947-2022;
   Schena, Emiliano/J-5619-2018; Carnevale, Arianna/AAB-5328-2022; Longo,
   Umile Giuseppe/K-9147-2016; Maffulli, Nicola/A-1839-2014
OI Ciccozzi, Massimo/0000-0003-3866-9239; Schena,
   Emiliano/0000-0002-9696-1265; Carnevale, Arianna/0000-0003-4543-1149;
   Salvatore, Giuseppe/0000-0003-1937-3272; Longo, Umile
   Giuseppe/0000-0003-4063-9821; Maffulli, Nicola/0000-0002-5327-3702
CR Amram O, 2016, BMJ OPEN, V6, DOI 10.1136/bmjopen-2015-010274
   Berto P, 2012, ISPOR ITALY MED DEVI
   Bini SA, 2011, J ARTHROPLASTY, V26, P124, DOI 10.1016/j.arth.2011.03.043
   Bozic KJ, 2010, CLIN ORTHOP RELAT R, V468, P45, DOI 10.1007/s11999-009-0945-0
   Brenna E, 2011, HEALTH POLICY, V103, P209, DOI 10.1016/j.healthpol.2011.10.003
   Fauci AS, 2018, NEW ENGL J MED, V378, P314, DOI 10.1056/NEJMp1714773
   France G, 2005, J HEALTH POLIT POLIC, V30, P169, DOI 10.1215/03616878-30-1-2-169
   Ganestam A, 2016, KNEE SURG SPORT TR A, V24, P3730, DOI 10.1007/s00167-015-3544-5
   Guerriero C, 2016, HOSP BUDGET CONSTRAI
   Huttunen TT, 2014, AM J SPORT MED, V42, P2419, DOI 10.1177/0363546514540599
   ISTAT, 2010, POP DEC 31 2009
   Jarvinen Tero A H, 2005, Foot Ankle Clin, V10, P255, DOI 10.1016/j.fcl.2005.01.013
   Longo UG, 2017, ARCH ORTHOP TRAUM SU, V137, P217, DOI 10.1007/s00402-016-2610-x
   Longo UG, 2013, FOOT ANKLE CLIN, V18, P319, DOI 10.1016/j.fcl.2013.02.009
   Maffulli N, 1999, J BONE JOINT SURG AM, V81A, P1019, DOI 10.2106/00004623-199907000-00017
   Maffulli N, 2008, J BONE JOINT SURG AM, V90A, P1348, DOI 10.2106/JBJS.G.01241
   Maffulli Nicola, 2017, Open Orthop J, V11, P660, DOI 10.2174/1874325001711010660
   Maffulli N, 2013, PHYS THER SPORT, V14, P163, DOI 10.1016/j.ptsp.2012.07.002
   Maffulli N, 2013, AM J SPORT MED, V41, P1100, DOI 10.1177/0363546513479017
   Maffulli N, 2012, J BONE JOINT SURG AM, V94A, P906, DOI 10.2106/JBJS.K.00869
   Maffulli N, 2011, ARCH ORTHOP TRAUM SU, V131, P33, DOI 10.1007/s00402-010-1097-0
   Maffulli N, 2010, AM J SPORT MED, V38, P2304, DOI 10.1177/0363546510376619
   Maffulli N, 2009, ORTHOP CLIN N AM, V40, P491, DOI 10.1016/j.ocl.2009.05.006
   Maffulli N, 2008, DISABIL REHABIL, V30, P1714, DOI 10.1080/09638280701786765
   Mattila VM, 2015, BRIT J SPORT MED, V49, P1084, DOI 10.1136/bjsports-2013-092756
   Moller A, 1996, ACTA ORTHOP SCAND, V67, P479, DOI 10.3109/17453679608996672
   Ochen Y, 2019, BMJ-BRIT MED J, V364, DOI 10.1136/bmj.k5120
   PRICE AE, 1986, CLIN ORTHOP RELAT R, P249
   Schepsis AA, 2002, AM J SPORT MED, V30, P287, DOI 10.1177/03635465020300022501
NR 29
TC 3
Z9 3
U1 0
U2 0
PU BMC
PI LONDON
PA CAMPUS, 4 CRINAN ST, LONDON N1 9XW, ENGLAND
EI 1471-2474
J9 BMC MUSCULOSKEL DIS
JI BMC Musculoskelet. Disord.
PD OCT 17
PY 2020
VL 21
IS 1
AR 687
DI 10.1186/s12891-020-03688-2
PG 12
WC Orthopedics; Rheumatology
WE Science Citation Index Expanded (SCI-EXPANDED); Social Science Citation Index (SSCI)
SC Orthopedics; Rheumatology
GA OH6YF
UT WOS:000582740900001
PM 33069229
OA Green Published, gold
DA 2022-07-22
ER

PT J
AU Toyooka, S
   Takeda, H
   Nakajima, K
   Masujima, A
   Miyamoto, W
   Pagliazzi, G
   Nakagawa, T
   Kawano, H
AF Toyooka, Seikai
   Takeda, Hideki
   Nakajima, Kohei
   Masujima, Atsushi
   Miyamoto, Wataru
   Pagliazzi, Gherardo
   Nakagawa, Takumi
   Kawano, Hirotaka
TI Correlation Between Recovery of Triceps Surae Muscle Strength and Level
   of Activity After Open Repair of Acute Achilles Tendon Rupture
SO FOOT & ANKLE INTERNATIONAL
LA English
DT Article
DE Achilles tendon; retrospective study; tendon injuries; heel rise;
   triceps surae
ID RANDOMIZED CONTROLLED-TRIALS; CONSERVATIVE TREATMENT; NONSURGICAL
   TREATMENT; ELECTROMYOGRAM; MOBILIZATION; MANAGEMENT; TEARS
AB Background: This study aimed to determine whether physical findings reflecting triceps surae strength recovery could predict return to activities such as jogging and sports and whether patients' age and sex would influence recovery of triceps surae strength postoperatively.
   Methods: Between 2009 and 2013, 96 consecutive cases of postoperative acute Achilles tendon rupture were reviewed. The postoperative triceps surae strength recovery rate was investigated in all patients by using half body weight 1-time heel rise, full body weight (FBW) 1-time heel rise, FBW 20-time heel rise, jogging, and full return to sports activities. Influence of age and sex on triceps surae strength recovery was also investigated.
   Results: FBW 1-time heel rise and jogging were achieved at an average of 14 weeks (range, 6-24 weeks) and 15 weeks (range, 8-25 weeks) postoperatively, respectively. FBW 20-time heel rise and full return to sports activities were achieved at a mean of 21 weeks (range, 12-29 weeks) and 22 weeks (range, 13-29 weeks) postoperatively, respectively. Ability to perform FBW 1-time heel rise was directly related to resilience of jogging capability (R-2 = 0.317, P < 0.001), and ability to perform FBW 20-time heel rise was related to full return to sports activities (R-2 = 0.508, P < 0.001). Time to heel rise was not correlated with patient age or sex.
   Conclusion: Postoperative ability to perform FBW 1-time heel rise in patients postoperatively was directly related to resilience of jogging, and ability to perform FBW 20-time heel rise was directly related to full return to sports activities.
   Level of Evidence: Level IV, case series.
C1 [Toyooka, Seikai; Miyamoto, Wataru; Nakagawa, Takumi; Kawano, Hirotaka] Teikyo Univ, Dept Orthopaed Surg, Sch Med, Tokyo, Japan.
   [Takeda, Hideki; Nakajima, Kohei; Masujima, Atsushi] Toshiba Gen Hosp, Dept Orthopaed Surg, Tokyo, Japan.
   [Pagliazzi, Gherardo] Rizzoli Orthopaed Inst, Dept Orthopaed Surg, Bologna, Italy.
RP Toyooka, S (通讯作者)，Teikyo Univ, Dept Orthopaed Surg, Sch Med, Itabashi Ku, 2-11-1 Kaga, Tokyo 1738605, Japan.
EM blueocean1092@hotmail.com
CR BUNNELL S, 1953, Ind Med Surg, V22, P251
   Costa ML, 2006, J BONE JOINT SURG BR, V88B, P69, DOI 10.1302/0301-620X.88B1.16549
   Fujisawa H, 2015, J PHYS THER SCI, V27, P1523, DOI 10.1589/jpts.27.1523
   Hislop HJ, 1995, DANIELS WORTHINGHAMS
   INGLIS AE, 1976, J BONE JOINT SURG AM, V58, P990, DOI 10.2106/00004623-197658070-00015
   Kangas J, 2007, AM J SPORT MED, V35, P59, DOI 10.1177/0363546506293255
   Karaaslan F, 2016, THER CLIN RISK MANAG, V12, P1311, DOI 10.2147/TCRM.S116385
   Maffulli N, 1999, J BONE JOINT SURG AM, V81A, P1019, DOI 10.2106/00004623-199907000-00017
   Maffulli N, 2003, AM J SPORT MED, V31, P692, DOI 10.1177/03635465030310051001
   Maffulli N, 2003, J SPORT MED PHYS FIT, V43, P367
   Moller M, 2002, SCAND J MED SCI SPOR, V12, P9, DOI 10.1034/j.1600-0838.2002.120103.x
   Mullaney M, 2011, SPORTS HEALTH, V3, P543, DOI 10.1177/1941738111416911
   Osarumwense D, 2013, J ORTHOP SURG-HONG K, V21, P44, DOI 10.1177/230949901302100112
   Peng WC, 2017, CLIN BIOMECH, V43, P50, DOI 10.1016/j.clinbiomech.2017.02.002
   Stavrou M, 2013, J ORTHOP SURG-HONG K, V21, P232, DOI 10.1177/230949901302100224
   STORPER IS, 1992, OTOLARYNG HEAD NECK, V107, P527, DOI 10.1177/019459989210700404
   Sveen TM, 2015, DAN MED J, V62
   Tamaki H, 1997, EUR J APPL PHYSIOL O, V75, P1
   Tejwani Nirmal C, 2014, Am J Orthop (Belle Mead NJ), V43, pE221
   Todorov A, 2015, MLTJ-MUSCLE LIGAMENT, V5, P68, DOI 10.11138/mltj/2015.5.2.068
   TSUGE K, 1974, HAND, V6, P255, DOI 10.1016/0072-968X(74)90036-9
   Uchiyama E, 2007, AM J SPORT MED, V35, P1739, DOI 10.1177/0363546507304492
   Wang D, 2015, FOOT ANKLE SURG, V21, P250, DOI 10.1016/j.fas.2015.01.009
   Wilkins R, 2012, AM J SPORT MED, V40, P2154, DOI 10.1177/0363546512453293
   Wu YH, 2016, INT J SURG, V36, P305, DOI 10.1016/j.ijsu.2016.11.014
NR 25
TC 2
Z9 2
U1 0
U2 1
PU SAGE PUBLICATIONS INC
PI THOUSAND OAKS
PA 2455 TELLER RD, THOUSAND OAKS, CA 91320 USA
SN 1071-1007
EI 1944-7876
J9 FOOT ANKLE INT
JI Foot Ankle Int.
PD DEC
PY 2017
VL 38
IS 12
BP 1324
EP 1330
DI 10.1177/1071100717728686
PG 7
WC Orthopedics
WE Science Citation Index Expanded (SCI-EXPANDED)
SC Orthopedics
GA FN8JA
UT WOS:000416268700005
PM 28891314
DA 2022-07-22
ER

PT J
AU Kumar, A
   Rutherford-Davies, J
   Thorpe, P
   Newson, A
AF Kumar, Abhijeet
   Rutherford-Davies, Jill
   Thorpe, Philippa
   Newson, Annalisa
TI Combined quadriceps lengthening (using an external ring fixator) and
   patellar tendon reconstruction (using a tendoachilles allograft) in a
   case of chronic patellar tendon rupture: A case report
SO KNEE
LA English
DT Article
DE Patella alta; Extensor mechanism reconstruction; Quadriceps contracture;
   Achilles tendon allograft; Chronic patellar tendon rupture; External
   fixator
ID EXTENSOR MECHANISM; KNEE; DISRUPTION; LIGAMENT
AB Background: Patellar tendon rupture is an infrequent and debilitating condition, which if left untreated, is complicated by quadriceps contracture and patella alta. This results in reduced function of the knee extensor mechanism including extension lag, reduced range of motion (ROM), chronic knee pain, and frequent falls. Early primary repair has good results in most cases and is performed by opposing and suturing the tendon ends. However, when there is a delay between rupture and repair or in case of a re-rupture, primary repair techniques may not work due to retraction of the extensor mechanism. Several treatment modalities have been proposed in such cases, but there is no clear consensus.
   Methodology: We present the case of a 39-year-old male with chronic rupture of patellar tendon that was reconstructed with a two-stage technique. In the first stage, a ring-fixator was applied to stretch the quadriceps muscle and the second stage consisted of reconstruction of the patellar tendon with a tendoachilles allograft. This procedure was performed in two stages five weeks apart and almost four years after the initial injury.
   Results: Results were excellent at three years' follow-up with the patient achieving full extension, a ROM of 0-110 degrees and 85% quadriceps strength. There was significant improvement in his pain, mobility and quadriceps bulk.
   Conclusion: To our knowledge, this treatment has not previously been described in the literature. We present this case as a proven treatment option for treatment of chronic patellar tendon injuries with extensor mechanism shortening. (C) 2019 Elsevier B.V. All rights reserved.
C1 [Kumar, Abhijeet; Rutherford-Davies, Jill; Newson, Annalisa] Salford Royal NHS Fdn Trust, Salford, Lancs, England.
   [Thorpe, Philippa] Royal Liverpool & Broadgreen NHS Trust, Liverpool, Merseyside, England.
RP Kumar, A (通讯作者)，Salford Royal NHS Fdn Trust, Salford, Lancs, England.
EM abhijeet232@gmail.com
CR Aarimaa V, 2008, SCAND J MED SCI SPOR, V18, P442, DOI 10.1111/j.1600-0838.2007.00730.x
   [Anonymous], [No title captured]
   Barrack RL, 2003, CLIN ORTHOP RELAT R, P98, DOI 10.1097/01.blo.0000092993.90435.69
   Bermudez CA, 2007, J TRAUMA, V63, P211, DOI 10.1097/01.ta.0000241293.03907.ae
   BLACKBURNE JS, 1977, J BONE JOINT SURG BR, V59, P241, DOI 10.1302/0301-620X.59B2.873986
   Conrad BP, 2013, CELL TISSUE BANK, V14, P359, DOI 10.1007/s10561-012-9340-2
   Crossett LS, 2002, J BONE JOINT SURG AM, V84A, P1354, DOI 10.2106/00004623-200208000-00011
   DEJOUR H, 1992, REV CHIR ORTHOP, V78, P58
   ECKER ML, 1979, J BONE JOINT SURG AM, V61, P884, DOI 10.2106/00004623-197961060-00013
   Falconiero RP, 1996, ARTHROSCOPY, V12, P623, DOI 10.1016/S0749-8063(96)90204-2
   Gilmore JH, 2015, KNEE, V22, P148, DOI 10.1016/j.knee.2014.10.007
   Greis PE, 2005, ORTHOPEDICS, V28, P765, DOI 10.3928/0147-7447-20050801-13
   Jarvela T, 2005, KNEE, V12, P139, DOI 10.1016/j.knee.2004.05.005
   Kannus P, 1997, SCAND J MED SCI SPOR, V7, P78
   Katz LM, 2008, ARTHROSCOPY, V24, P1330, DOI 10.1016/j.arthro.2008.07.015
   Lewis P, 2010, J KNEE SURG, V21, P130, DOI [10.1055/s-0030-1247807., DOI 10.1055/S-0030-1247807]
   MANDELBAUM BR, 1988, CLIN ORTHOP RELAT R, P268
   McNally PD, 1998, ARTHROSCOPY, V14, P340, DOI 10.1016/S0749-8063(98)70154-9
   Naguib AM, 2006, INJURY EXTRA, V37, P379
NR 19
TC 3
Z9 3
U1 0
U2 2
PU ELSEVIER
PI AMSTERDAM
PA RADARWEG 29, 1043 NX AMSTERDAM, NETHERLANDS
SN 0968-0160
EI 1873-5800
J9 KNEE
JI Knee
PD MAR
PY 2020
VL 27
IS 2
BP 598
EP 606
DI 10.1016/j.knee.2019.12.014
PG 9
WC Orthopedics; Sport Sciences; Surgery
WE Science Citation Index Expanded (SCI-EXPANDED)
SC Orthopedics; Sport Sciences; Surgery
GA LJ3OS
UT WOS:000530078000040
PM 32014413
DA 2022-07-22
ER

PT J
AU Padanilam, TG
AF Padanilam, Thomas G.
TI Chronic Achilles Tendon Ruptures
SO FOOT AND ANKLE CLINICS
LA English
DT Article
DE Chronic Achilles tendon ruptures; Neglected Achilles tendon ruptures;
   Achilles; Tendon transfers for Achilles; Treatment of chronic Achilles
   rupture
AB Chronic Achilles tendon ruptures can be challenging to treat. Most of these are a result of failure to make the initial diagnosis. Although pain is not a prominent symptom, these patients have significant functional deficits that can interfere with activities of daily living. Nonoperative treatment options are limited and probably best reserved for patients with low functional demands or those with significant medical contraindications for surgical treatment. Surgical treatment of chronic rupture often involves the need to restore continuity to tendon ends that may have retracted leading to irreducible gaps. A variety of techniques have been described to address this situation. Most of the reports have shown improvement in patient outcomes with surgical intervention; however, most patients continue to show some strength deficits in comparison to their contralateral limb. The small number of patients in these studies combined with variations in patient selection, postoperative regimens, and outcome measurements make meaningful comparisons difficult. Restoration of the physiologic tension of the gastrocnemius-soleus complex is the goal of surgical intervention and controlled studies are needed to determine if one method is superior to others in the achievement of this goal.
C1 [Padanilam, Thomas G.] Univ Toledo, Toledo, OH 43615 USA.
   [Padanilam, Thomas G.] Toledo Orthopaed Surg, Toledo, OH 43615 USA.
RP Padanilam, TG (通讯作者)，Univ Toledo, Toledo, OH 43615 USA.
EM tgpadanilam@msn.com
CR ABRAHAM E, 1975, J BONE JOINT SURG AM, VA 57, P253, DOI 10.2106/00004623-197557020-00019
   ARNER O, 1959, ACTA CHIR SCAND    S, V239, P1
   BARNES MJ, 1986, J BONE JOINT SURG BR, V68, P121, DOI 10.1302/0301-620X.68B1.3941128
   BOSWORTH DM, 1956, J BONE JOINT SURG AM, V38, P111, DOI 10.2106/00004623-195638010-00011
   BOYDEN EM, 1995, CLIN ORTHOP RELAT R, P150
   BUGG EI, 1968, CLIN ORTHOP RELAT R, P73
   CARDEN DG, 1987, J BONE JOINT SURG BR, V69, P416, DOI 10.1302/0301-620X.69B3.3294839
   CHRISTENSEN I, 1953, ACTA CHIR SCAND, V106, P50
   COPELAND SA, 1990, ANN ROY COLL SURG, V72, P270
   Coull R, 2003, FOOT ANKLE INT, V24, P931, DOI 10.1177/107110070302401211
   Dalal RB, 2003, ANN ROY COLL SURG, V85, P283, DOI 10.1308/003588403766275088
   Den Hartog BD, 2008, FOOT ANKLE INT, V29, P456, DOI 10.3113/FAI.2008.0456
   ELFTMAN H, 1966, J BONE JOINT SURG AM, VA 48, P363, DOI 10.2106/00004623-196648020-00017
   FRENETTE JP, 1977, J BONE JOINT SURG AM, V59, P673, DOI 10.2106/00004623-197759050-00015
   GABEL S, 1994, FOOT ANKLE INT, V15, P512, DOI 10.1177/107110079401500912
   Haraguchi N, 2005, TECH FOOT ANKLE SURG, V4, P154, DOI 10.1097/01.btf.0000177228.45213.5d
   HOWARD CB, 1984, J BONE JOINT SURG BR, V66, P206, DOI 10.1302/0301-620X.66B2.6707056
   INGLIS AE, 1976, J BONE JOINT SURG AM, V58, P990, DOI 10.2106/00004623-197658070-00015
   Jennings AG, 2002, J BONE JOINT SURG BR, V84B, P361, DOI 10.1302/0301-620X.84B3.11559
   Kangas J, 2007, AM J SPORT MED, V35, P59, DOI 10.1177/0363546506293255
   Karjalainen PT, 1997, AM J SPORT MED, V25, P164, DOI 10.1177/036354659702500204
   KRACKOW KA, 1988, ORTHOPEDICS, V11, P909, DOI 10.3928/0147-7447-19880601-11
   KUWADA G T, 1990, Journal of Foot Surgery, V29, P361
   LEITNER A, 1992, FOOT ANKLE, V13, P176, DOI 10.1177/107110079201300402
   Leslie Hamish D H, 2005, Foot Ankle Clin, V10, P357, DOI 10.1016/j.fcl.2005.01.009
   Maffulli N, 2005, CLIN J SPORT MED, V15, P56, DOI 10.1097/01.jsm.0000152714.05097.ef
   Maffulli N, 1998, AM J SPORT MED, V26, P266, DOI 10.1177/03635465980260021801
   Maffulli N, 2008, J BONE JOINT SURG AM, V90A, P1348, DOI 10.2106/JBJS.G.01241
   Maffulli Nicola, 2007, Foot Ankle Clin, V12, P583, DOI 10.1016/j.fcl.2007.07.007
   MANDELBAUM BR, 1995, AM J SPORT MED, V23, P392, DOI 10.1177/036354659502300403
   MANN RA, 1991, J BONE JOINT SURG AM, V73A, P214, DOI 10.2106/00004623-199173020-00009
   Matles A L, 1975, Bull Hosp Joint Dis, V36, P48
   Mulier T, 2007, FOOT ANKLE INT, V28, P910, DOI 10.3113/FAI.2007.0910
   Myerson M S, 1999, Instr Course Lect, V48, P219
   Nellas Z J, 1996, J Foot Ankle Surg, V35, P144
   Nestorson J, 2000, ACTA ORTHOP SCAND, V71, P64, DOI 10.1080/00016470052943928
   OBRIEN T, 1984, J BONE JOINT SURG AM, V66A, P1099, DOI 10.2106/00004623-198466070-00018
   OZAKI J, 1989, CLIN ORTHOP RELAT R, P204
   PARKER RG, 1979, J AM POD ASSOC, V69, P548, DOI 10.7547/87507315-69-9-548
   PEREZTEUFFER A, 1974, ORTHOP CLIN N AM, V5, P89
   Pintore E, 2001, J TRAUMA, V50, P71, DOI 10.1097/00005373-200101000-00013
   Porter DA, 1997, FOOT ANKLE INT, V18, P557, DOI 10.1177/107110079701800905
   RUSH JH, 1980, AUST NZ J SURG, V50, P420, DOI 10.1111/j.1445-2197.1980.tb04156.x
   SCHELLER AD, 1980, ORTHOP CLIN N AM, V11, P801
   SILVER RL, 1985, J BONE JOINT SURG BR, V67, P432, DOI 10.1302/0301-620X.67B3.3997956
   SIMMONDS F A, 1957, Practitioner, V179, P56
   Tashjian RZ, 2003, FOOT ANKLE INT, V24, P673, DOI 10.1177/107110070302400903
   THOMPSON T C, 1962, J Trauma, V2, P126, DOI 10.1097/00005373-196203000-00003
   TOBIN W J, 1953, Am Surg, V19, P514
   TURCO VJ, 1987, FOOT ANKLE, V7, P253, DOI 10.1177/107110078700700407
   Us AK, 1997, ARCH ORTHOP TRAUM SU, V116, P408
   Wapner K L, 1993, Foot Ankle, V14, P443
   Wilcox DK, 2000, FOOT ANKLE INT, V21, P1004, DOI 10.1177/107110070002101204
   Yasuda T, 2007, AM J SPORT MED, V35, P582, DOI 10.1177/0363546506295939
   Zadek I, 1940, J BONE JOINT SURG, V22, P1070
NR 55
TC 40
Z9 48
U1 0
U2 2
PU W B SAUNDERS CO-ELSEVIER INC
PI PHILADELPHIA
PA 1600 JOHN F KENNEDY BOULEVARD, STE 1800, PHILADELPHIA, PA 19103-2899 USA
SN 1083-7515
EI 1558-1934
J9 FOOT ANKLE CLIN
JI Foot Ankle Clin.
PD DEC
PY 2009
VL 14
IS 4
BP 711
EP 728
DI 10.1016/j.fcl.2009.08.001
PG 18
WC Orthopedics
WE Science Citation Index Expanded (SCI-EXPANDED)
SC Orthopedics
GA V16PP
UT WOS:000207881800011
PM 19857844
DA 2022-07-22
ER

PT J
AU Gossage, W
   Kohls-Gatzoulis, J
   Solan, M
AF Gossage, William
   Kohls-Gatzoulis, Julie
   Solan, Matthew
TI Endoscopic Assisted Repair of Chronic Achilles Tendon Rupture with
   Flexor Hallucis Longus Augmentation
SO FOOT & ANKLE INTERNATIONAL
LA English
DT Article
DE Chronic Rupture; Achilles Tendon; FHL Tendon
ID MANAGEMENT; ANKLE
C1 [Solan, Matthew] Royal Surrey Cty Hosp NHS Trust, Guildford GU2 7XX, Surrey, England.
RP Solan, M (通讯作者)，Royal Surrey Cty Hosp NHS Trust, Egerton Rd, Guildford GU2 7XX, Surrey, England.
EM matthewsolan1@aol.com
CR ABRAHAM E, 1975, J BONE JOINT SURG AM, VA 57, P253, DOI 10.2106/00004623-197557020-00019
   BRUGGEMAN NB, 2004, CLIN ORTHOP RELAT R, V427, P63
   CHRISTENSEN I, 1953, ACTA CHIR SCAND, V106, P50
   Kissel C G, 1994, J Foot Ankle Surg, V33, P46
   Leslie Hamish D H, 2005, Foot Ankle Clin, V10, P357, DOI 10.1016/j.fcl.2005.01.009
   Lui TH, 2007, KNEE SURG SPORT TR A, V15, P1163, DOI 10.1007/s00167-007-0352-6
   Maffulli N, 2008, J BONE JOINT SURG AM, V90A, P1348, DOI 10.2106/JBJS.G.01241
   MANN RA, 1991, J BONE JOINT SURG AM, V73A, P214, DOI 10.2106/00004623-199173020-00009
   Miller D, 2005, SCOT MED J, V50, P160, DOI 10.1177/003693300505000408
   Myerson M S, 1999, Instr Course Lect, V48, P219
   Nellas Z J, 1996, J Foot Ankle Surg, V35, P144
   PARSONS JR, 1989, FOOT ANKLE, V9, P179, DOI 10.1177/107110078900900406
   PEREZTEUFFER A, 1974, ORTHOP CLIN N AM, V5, P89
   Scholten PE, 2008, J BONE JOINT SURG AM, V90A, P2665, DOI 10.2106/JBJS.F.00188
   SILVER RL, 1985, J BONE JOINT SURG BR, V67, P432, DOI 10.1302/0301-620X.67B3.3997956
   Wapner K L, 1993, Foot Ankle, V14, P443
NR 16
TC 9
Z9 10
U1 0
U2 0
PU SAGE PUBLICATIONS INC
PI THOUSAND OAKS
PA 2455 TELLER RD, THOUSAND OAKS, CA 91320 USA
SN 1071-1007
EI 1944-7876
J9 FOOT ANKLE INT
JI Foot Ankle Int.
PD APR
PY 2010
VL 31
IS 4
BP 343
EP 347
DI 10.3113/FAI.2010.0343
PG 5
WC Orthopedics
WE Science Citation Index Expanded (SCI-EXPANDED)
SC Orthopedics
GA 576IN
UT WOS:000276137900013
PM 20371024
DA 2022-07-22
ER

PT J
AU Aroen, A
   Helgo, D
   Granlund, OG
   Bahr, R
AF Aroen, A
   Helgo, D
   Granlund, OG
   Bahr, R
TI Contralateral tendon rupture risk is increased in individuals with a
   previous Achilles tendon rupture
SO SCANDINAVIAN JOURNAL OF MEDICINE & SCIENCE IN SPORTS
LA English
DT Article
DE surgical treatment; risk factor; prevention; post-injury problems
ID SURGICAL-TREATMENT; BLOOD-GROUPS; INJURIES; BADMINTON; REPAIR; MODEL
AB The purpose of this study was to examine the outcome after surgery for an acute Achilles tendon rupture. In particular, we wanted to study whether persons who have suffered an Achilles tendon rupture are at greater risk of a contralateral tendon rupture. From September 1990 to April 1997, 168 acute Achilles tendon ruptures in 26 women and 142 men were treated operatively. In August 1998 (median: 4.2 years post injury), 154 of these patients (92%) responded to a follow-up questionnaire. Local symptoms (pain, decreased strength and/or reduced range of motion) were reported preinjury by 25% of the patients; at follow-up this had increased to 52%. Ten patients (6%) experienced a rupture on the contralateral side during the follow-up period (OR: 176 [170-282] vs. the expected rate based on the general population risk of this injury, P<0.001). Thus, this study suggests that patients with an Achilles tendon rupture are at significantly increased risk of a contralateral tendon rupture, as well. Also, as many as half of the patients suffered from post-injury problems at long-term follow-up.
C1 Univ Oslo, Akershus Univ Hosp, Dept Orthoped Surg, NO-1474 Nordbyhagen, Norway.
   Norwegian Univ Sport & Phys Educ, Dept Sports Med, Oslo Sports Trauma Res Ctr, Oslo, Norway.
RP Aroen, A (通讯作者)，Univ Oslo, Akershus Univ Hosp, Dept Orthoped Surg, NO-1474 Nordbyhagen, Norway.
EM asben@online.no
RI Årøen, Asbjørn/AAP-4533-2020; Bahr, Roald/B-6964-2014
OI Bahr, Roald/0000-0001-5725-4237
CR Archambault JM, 2001, CONNECT TISSUE RES, V42, P13, DOI 10.3109/03008200109014245
   BRUKNER P, 2001, CLIN SPORTS MED, P481
   Cetti R, 2003, ACTA ORTHOP SCAND, V74, P78, DOI 10.1080/00016470310013707
   CETTI R, 1983, CLIN ORTHOP RELAT R, P204
   CETTI R, 1993, AM J SPORT MED, V21, P791, DOI 10.1177/036354659302100606
   HELGELAND J, 1997, TIDSKR NOR LAEGEFORE, V117, P1733
   Herbert RD, 2002, BRIT MED J, V325, P468, DOI 10.1136/bmj.325.7362.468
   JORGENSEN U, 1990, SPORTS MED, V10, P59, DOI 10.2165/00007256-199010010-00006
   Jozsa L, 1997, SCAND J MED SCI SPOR, V7, P113
   JOZSA L, 1989, J BONE JOINT SURG BR, V71, P272, DOI 10.1302/0301-620X.71B2.2494187
   JOZSA L, 1989, AM J SPORT MED, V17, P338, DOI 10.1177/036354658901700305
   KANNUS P, 1991, J BONE JOINT SURG AM, V73A, P1507, DOI 10.2106/00004623-199173100-00009
   Khan KM, 1999, SPORTS MED, V27, P393, DOI 10.2165/00007256-199927060-00004
   Kluger R, 1999, SPORTVERLETZ SPORTSC, V13, P96, DOI 10.1055/s-2007-993323
   Leppilahti J, 1996, ANN CHIR GYNAECOL FE, V85, P369
   Maffulli N, 1999, J BONE JOINT SURG AM, V81A, P1019, DOI 10.2106/00004623-199907000-00017
   Maffulli N, 2000, AM J SPORT MED, V28, P499, DOI 10.1177/03635465000280040901
   Maffulli N, 2002, MED SCI SPORT EXER, V34, P1057, DOI 10.1097/00005768-200207000-00001
   Maffulli N, 2000, CLIN J SPORT MED, V10, P269, DOI 10.1097/00042752-200010000-00008
   Mathiak G, 1999, ARCH ORTHOP TRAUM SU, V119, P280, DOI 10.1007/s004020050410
   Messner K, 1999, CELLS TISSUES ORGANS, V165, P30, DOI 10.1159/000016671
   Moller A, 1996, ACTA ORTHOP SCAND, V67, P479, DOI 10.3109/17453679608996672
   MOLLER M, 2001, THESIS GOTEBERG U GO
   NISTOR L, 1981, J BONE JOINT SURG AM, V63, P394, DOI 10.2106/00004623-198163030-00012
   Selvanetti A, 1997, OPER TECHN SPORT MED, V5, P110, DOI 10.1016/S1060-1872(97)80031-7
   Soldatis JJ, 1997, AM J SPORT MED, V25, P90, DOI 10.1177/036354659702500118
   Speck M, 1998, AM J SPORT MED, V26, P789, DOI 10.1177/03635465980260060901
   Tallon C, 2001, MED SCI SPORT EXER, V33, P1983, DOI 10.1097/00005768-200112000-00002
NR 28
TC 48
Z9 49
U1 0
U2 4
PU WILEY
PI HOBOKEN
PA 111 RIVER ST, HOBOKEN 07030-5774, NJ USA
SN 0905-7188
EI 1600-0838
J9 SCAND J MED SCI SPOR
JI Scand. J. Med. Sci. Sports
PD FEB
PY 2004
VL 14
IS 1
BP 30
EP 33
DI 10.1111/j.1600-0838.2004.00344.x
PG 4
WC Sport Sciences
WE Science Citation Index Expanded (SCI-EXPANDED)
SC Sport Sciences
GA 801JB
UT WOS:000220091100005
PM 14723785
DA 2022-07-22
ER

PT J
AU Nakajima, K
   Taketomi, S
   Inui, H
   Nakamura, K
   Sanada, T
   Tanaka, S
AF Nakajima, Koji
   Taketomi, Shuji
   Inui, Hiroshi
   Nakamura, Kensuke
   Sanada, Takaki
   Tanaka, Sakae
TI Missed Medial Malleolar Fracture Associated With Achilles Tendon
   Rupture: A Case Report and Literature Review
SO JOURNAL OF FOOT & ANKLE SURGERY
LA English
DT Article
DE ankle; diagnosis; injury; post-traumatic arthrosis
ID ANKLE
AB A 45-year-old man sustained an Achilles tendon rupture while playing futsal. A concomitant medial malleolar fracture was not diagnosed until the patient underwent an operation for Achilles tendon repair. A routine postoperative radiograph showed a minimally displaced medial malleolar fracture. Conservative treatment was chosen for the fracture. The function of the Achilles tendon recovered well, and the fracture was united. A medial malleolar fracture can be missed when an Achilles tendon rupture occurs simultaneously. Thus, surgeons should consider the possibility of medial malleolar fracture associated with an Achilles tendon rupture. (C) 2016 by the American College of Foot and Ankle Surgeons. All rights reserved.
C1 [Nakajima, Koji; Taketomi, Shuji; Inui, Hiroshi; Nakamura, Kensuke; Sanada, Takaki; Tanaka, Sakae] Univ Tokyo, Fac Med, Dept Orthopaed Surg, Tokyo 1130033, Japan.
RP Taketomi, S (通讯作者)，Univ Tokyo, Fac Med, Dept Orthopaed Surg, Bunkyo Ku, 7-3-1 Hongo, Tokyo 1130033, Japan.
EM takeos-tky@umin.ac.jp
RI Tanaka, Sakae/Y-3061-2019
OI Tanaka, Sakae/0000-0001-9210-9414
CR Assal M, 2002, J ORTHOP TRAUMA, V16, P358, DOI 10.1097/00005131-200205000-00013
   BARRON JL, 1993, AM J SPORT MED, V21, P629, DOI 10.1177/036354659302100426
   Bunnell S, 1941, J BONE JOINT SURG, V23, P240
   Garneti N., 2005, FOOT ANKLE SURG, V11, P219, DOI [10.1016/j.fas.2005.06.003, DOI 10.1016/J.FAS.2005.06.003]
   Lubin J W, 2000, Am J Orthop (Belle Mead NJ), V29, P707
   Maffulli N, 2006, BMC MUSCULOSKEL DIS, V7, DOI 10.1186/1471-2474-7-59
   MARTIN JW, 1986, CLIN ORTHOP RELAT R, P216
   Pieper HG, 1998, INT J SPORTS MED, V19, P68, DOI 10.1055/s-2007-971883
   THOMPSON T C, 1962, J Trauma, V2, P126, DOI 10.1097/00005373-196203000-00003
   TSUGE K, 1974, HAND, V6, P255, DOI 10.1016/0072-968X(74)90036-9
   Uchiyama E, 2007, AM J SPORT MED, V35, P1739, DOI 10.1177/0363546507304492
NR 11
TC 2
Z9 2
U1 0
U2 3
PU ELSEVIER SCIENCE INC
PI NEW YORK
PA 360 PARK AVE SOUTH, NEW YORK, NY 10010-1710 USA
SN 1067-2516
EI 1542-2224
J9 J FOOT ANKLE SURG
JI J. Foot Ankle Surg.
PD JAN-FEB
PY 2016
VL 55
IS 1
BP 169
EP 172
DI 10.1053/j.jfas.2014.09.027
PG 4
WC Orthopedics; Surgery
WE Science Citation Index Expanded (SCI-EXPANDED)
SC Orthopedics; Surgery
GA CZ4KF
UT WOS:000367071200038
PM 25441273
DA 2022-07-22
ER

PT J
AU Bonanno, J
   Cheng, J
   Tilley, D
   Abutalib, Z
   Casey, E
AF Bonanno, Joseph
   Cheng, Jennifer
   Tilley, Dave
   Abutalib, Zafir
   Casey, Ellen
TI Factors Associated With Achilles Tendon Rupture in Women's Collegiate
   Gymnastics
SO SPORTS HEALTH-A MULTIDISCIPLINARY APPROACH
LA English
DT Article
DE gymnastics; female athlete; Achilles tendon
ID ABO BLOOD-GROUPS; SPORT SPECIALIZATION; TRICEPS SURAE; INJURY
AB Background: Achilles injury risk in women's collegiate gymnastics is 10-fold higher than in all other collegiate sports. This study aims to identify risk factors for Achilles tendon ruptures in collegiate female gymnasts. Hypothesis: Gymnasts with Achilles tendon ruptures will be more likely to report early gymnastics specialization, elite-level training before college, and performance of high-difficulty skills on floor and vault.
   Study Design: Cross-sectional study.
   Methods: Anonymous surveys were distributed to current and former collegiate female gymnasts, aged 18 to 30 years, via coaches, athletic trainers, Twitter, and ResearchMatch. Information about Achilles tendon ruptures, gymnastics-related injuries, sport specialization, event/skills participation, and medication use were collected.
   Results: A total of 581 gymnasts were included. One hundred gymnasts (17.2%; 95% CI: 14.1%-20.3%) reported Achilles tendon ruptures during collegiate training or competition. Most ruptures (91%) occurred on floor exercise; 85.7% of these occurred during back tumbling-take-off. Compared with gymnasts without ruptures during college, a greater percentage of gymnasts with ruptures competed at a Division I program, trained elite, competed difficult vaults and floor passes before and during college, competed in all 4 events during college, identified as Black/African American, and used retinoid medications.
   Conclusion: Achilles tendon ruptures are more common in women's collegiate gymnastics than other sports. Competing at the elite level, performing difficult floor and vault skills, and competing in all 4 events may increase the risk for an Achilles tendon rupture. Potential nontraining risk factors include retinoid exposure and Black/African American race. Future studies regarding the mechanisms of Achilles tendon ruptures in female collegiate gymnasts are warranted.
C1 [Bonanno, Joseph; Cheng, Jennifer; Casey, Ellen] Hosp Special Surg, Dept Physiatry, 535 E 70th St, New York, NY 10021 USA.
   [Tilley, Dave] Champ PT & Performance, Waltham, MA USA.
   [Abutalib, Zafir] Hosp Special Surg, Healthcare Res Inst, 535 E 70th St, New York, NY 10021 USA.
RP Casey, E (通讯作者)，Hosp Special Surg, 535 East 70th St, New York, NY 10021 USA.
EM caseye@hss.edu
FU National Center for Advancing Translational Sciences (NCATS) of the
   National Institutes of Health (NIH) [UL1 TR 002384]
FX This work was supported by grant number UL1 TR 002384 from the National
   Center for Advancing Translational Sciences (NCATS) of the National
   Institutes of Health (NIH) for REDCap use.
CR Arampatzis A, 2007, J BIOMECH, V40, P1946, DOI 10.1016/j.jbiomech.2006.09.005
   Bell DR, 2018, PEDIATRICS, V142, DOI 10.1542/peds.2018-0657
   Beytemur O, 2018, ACTA ORTHOP TRAUMATO, V52, P387, DOI 10.1016/j.aott.2018.06.006
   Bohm E, 1990, Sportverletz Sportschaden, V4, P22, DOI 10.1055/s-2007-993593
   Borms J., 2003, MED SPORTS SCI, V45
   Campbell R, 2019, BRIT J SPORT MED, V53, P1056, DOI 10.1136/bjsports-2018-099547
   Chan JJ, 2020, INT ORTHOP, V44, P585, DOI 10.1007/s00264-019-04471-2
   College Gym News, COLL GYM NEWS
   Dakin SG, 2018, BRIT J SPORT MED, V52, P359, DOI 10.1136/bjsports-2017-098161
   Davis JJ, 1999, MIL MED, V164, P872, DOI 10.1093/milmed/164.12.872
   Emerson C, 2010, MANUAL THER, V15, P37, DOI 10.1016/j.math.2009.05.008
   Fukashiro S, 2002, ACTA PHYSIOL SCAND, V175, P183, DOI 10.1046/j.1365-201X.2002.00985.x
   Ganestam A, 2016, KNEE SURG SPORT TR A, V24, P3730, DOI 10.1007/s00167-015-3544-5
   Garratty G, 2004, TRANSFUSION, V44, P703, DOI 10.1111/j.1537-2995.2004.03338.x
   Hart E, 2018, CURR SPORT MED REP, V17, P366, DOI 10.1249/JSR.0000000000000536
   Hicks KM, 2017, EUR J APPL PHYSIOL, V117, P1393, DOI 10.1007/s00421-017-3629-6
   Jemni M, 2017, SCI GYMNASTICS ADV C
   Johns W, 2021, FOOT ANKLE INT, V42, P495, DOI 10.1177/1071100720969633
   JOZSA L, 1989, J BONE JOINT SURG BR, V71, P272, DOI 10.1302/0301-620X.71B2.2494187
   Klatte-Schulz F, 2018, INT J MOL SCI, V19, DOI 10.3390/ijms19020404
   KUJALA UM, 1992, INJURY, V23, P131, DOI 10.1016/0020-1383(92)90050-3
   LEA RB, 1972, J BONE JOINT SURG AM, VA 54, P1398, DOI 10.2106/00004623-197254070-00002
   Leglise G, S SED TECHN, V1999, P17
   Lemme NJ, 2018, ORTHOP J SPORTS MED, V6, DOI 10.1177/2325967118808238
   Leppilahti J, 1998, SPORTS MED, V25, P79, DOI 10.2165/00007256-199825020-00002
   Leppilahti J, 1996, ANN CHIR GYNAECOL FE, V85, P369
   Ling D, 2020, PM&R, V12, P43, DOI 10.1002/pmrj.12184
   Luthi F, 2012, JOINT BONE SPINE, V79, P314, DOI 10.1016/j.jbspin.2011.11.001
   Maffulli N, 1998, Bull Hosp Jt Dis, V57, P152
   Maffulli N, 2000, CLIN J SPORT MED, V10, P269, DOI 10.1097/00042752-200010000-00008
   McGowan J, 2020, J SCI MED SPORT, V23, P139, DOI 10.1016/j.jsams.2019.09.002
   Myer GD, 2015, SPORTS HEALTH, V7, P437, DOI 10.1177/1941738115598747
   National Collegiate Athletic Association, DEF COUNT ATHL REL A
   National Institutes of Health, RAC ETHN CAT DEF NIH
   Nattiv Aurelia, 2007, Med Sci Sports Exerc, V39, P1867
   Nilsson O, 2016, HORM METAB RES, V48, P737, DOI 10.1055/s-0042-114038
   Owens B, 2007, INT J SPORTS MED, V28, P617, DOI 10.1055/s-2007-964837
   Park YH, 2019, KNEE SURG SPORT TR A, V27, P3297, DOI 10.1007/s00167-018-5172-3
   Peterson JG, 2021, J ORTHOP, V23, P46, DOI 10.1016/j.jor.2020.12.010
   Post EG, 2017, AM J SPORT MED, V45, P1405, DOI 10.1177/0363546517690848
   Scheid Jennifer L, 2019, J Funct Morphol Kinesiol, V4, DOI 10.3390/jfmk4030052
   Splittgerber LE, 2019, CURR SPORT MED REP, V18, P192, DOI 10.1249/JSR.0000000000000600
   Sweeney E, GYMNASTICS MED, V2020, P55
   Sweeney E, 2021, PHYSICIAN SPORTSMED, V49, P438, DOI 10.1080/00913847.2020.1850150
   Thomas R, 2018, PALGR CRIT UNIV STUD, P1, DOI [10.1007/978-3-319-95723-4_1, 10.1080/00913847.2018.1527646]
   Wertz J, 2013, SPORTS HEALTH, V5, P407, DOI 10.1177/1941738112472165
   White DW, 2007, AM J SPORT MED, V35, P1308, DOI 10.1177/0363546507301256
   Winnicki K, 2020, ANN ANAT, V229, DOI 10.1016/j.aanat.2020.151461
NR 48
TC 1
Z9 1
U1 3
U2 4
PU SAGE PUBLICATIONS INC
PI THOUSAND OAKS
PA 2455 TELLER RD, THOUSAND OAKS, CA 91320 USA
SN 1941-7381
EI 1941-0921
J9 SPORTS HEALTH
JI Sports Health
PD MAY
PY 2022
VL 14
IS 3
BP 358
EP 368
AR 19417381211034510
DI 10.1177/19417381211034510
EA JUL 2021
PG 11
WC Sport Sciences
WE Science Citation Index Expanded (SCI-EXPANDED)
SC Sport Sciences
GA 1G7CF
UT WOS:000679709100001
PM 34338076
DA 2022-07-22
ER

PT J
AU Naldo, J
   Agnew, P
   Brucato, M
   Dayton, P
   Shane, A
AF Naldo, Jason
   Agnew, Patrick
   Brucato, Maryellen
   Dayton, Paul
   Shane, Amber
TI ACFAS Clinical Consensus Statement: Acute Achilles Tendon Pathology
SO JOURNAL OF FOOT & ANKLE SURGERY
LA English
DT Article
DE Achilles; Delphi method; nonoperative; operative; percutaneous; rupture
ID NONAUGMENTED SURGICAL REPAIR; PROSPECTIVE RANDOMIZED-TRIAL; NONOPERATIVE
   TREATMENT; BLOOD-FLOW; NONSURGICAL TREATMENT; OPERATIVE TREATMENT; ACUTE
   RUPTURES; EARLY MOTION; MANAGEMENT; COMPLICATIONS
AB Injuries to the Achilles tendon are a challenge to the foot and ankle surgeon. In recent years, research has led to a relative change in the way that many surgeons view acute Achilles tendon rupture. In an effort to fully evaluate these trends, as well as to evaluate all aspects of care for acute Achilles tendon rupture, the American College of Foot and Ankle Surgeons convened a panel of experts to create a clinical consensus statement to address selected aspects of care of the acute Achilles tendon injury. (c) 2020 by the American College of Foot and Ankle Surgeons. All rights reserved.
C1 [Naldo, Jason] ACFAS Acute Achilles Tendon Pathol Clin Consensus, Chicago, IL USA.
   [Naldo, Jason] Virginia Tech Carilion Sch Med, Dept Orthopaed, Roanoke, VA USA.
   [Naldo, Jason] Carilion Clin, Inst Orthopaed & Neurosci, Roanoke, VA USA.
   [Agnew, Patrick] Eastern Virginia Med Sch, Podiatr Med & Surg Educ, Norfolk, VA 23501 USA.
   [Agnew, Patrick] Eastern Virginia Med Sch, Norfolk, VA 23501 USA.
   [Brucato, Maryellen] Brucato Foot & Ankle Surg, Clifton, NJ USA.
   [Dayton, Paul] Foot & Ankle Ctr Iowa, Ankeny, IA USA.
   [Shane, Amber] Advent Hlth Syst, Dept Podiatr Surg, Orlando, FL USA.
   [Shane, Amber] Advent Hlth East Orlando Podiatr Surg Residency, Orlando, FL USA.
RP Naldo, J (通讯作者)，2900 Lamb Circle Suite L-760, Christiansburg, VA 24073 USA.
EM jnaldo@gmail.com
OI Naldo, Jason/0000-0002-2796-9968
CR Abubeih H, 2018, INT ORTHOP, V42, P2699, DOI 10.1007/s00264-018-3976-x
   Ahmad J, 2017, J AM ACAD ORTHOP SUR, V25, P773, DOI 10.5435/JAAOS-D-16-00306
   Aktas Seref, 2007, J Foot Ankle Surg, V46, P336, DOI 10.1053/j.jfas.2007.06.006
   Alhammoud A, 2017, INT ORTHOP, V41, P309, DOI 10.1007/s00264-016-3318-9
   ALLENMARK C, 1992, CLIN SPORT MED, V11, P759
   ARACIL J, 1992, FOOT ANKLE, V13, P350, DOI 10.1177/107110079201300610
   Aspenberg P, 2007, INT ORTHOP, V31, P783, DOI 10.1007/s00264-007-0398-6
   ASTROM M, 1994, J ORTHOP RES, V12, P246, DOI 10.1002/jor.1100120214
   Astrom M, 2000, SCAND J MED SCI SPOR, V10, P365, DOI 10.1034/j.1600-0838.2000.010006365.x
   Avina Valencia Jorge Arturo, 2009, Acta Ortop Mex, V23, P125
   Bartel AFP, 2014, J FOOT ANKLE SURG, V53, P744, DOI 10.1053/j.jfas.2014.07.009
   Basiglini L, 2010, KNEE SURG SPORT TR A, V18, P644, DOI 10.1007/s00167-010-1085-5
   Becher C, 2018, FOOT ANKLE SURG, V24, P110, DOI 10.1016/j.fas.2016.12.003
   Berlet GC, 2014, J FOOT ANKLE SURG, V53, P298, DOI 10.1053/j.jfas.2014.02.001
   Bhandari M, 2002, CLIN ORTHOP RELAT R, P190
   Boushel R, 2000, J PHYSIOL-LONDON, V524, P305, DOI 10.1111/j.1469-7793.2000.t01-2-00305.x
   BOYDEN EM, 1995, CLIN ORTHOP RELAT R, P150
   BRADLEY JP, 1990, AM J SPORT MED, V18, P188, DOI 10.1177/036354659001800213
   Braunstein M, 2018, KNEE SURG SPORT TR A, V26, P846, DOI 10.1007/s00167-015-3795-1
   Bruggeman NB, 2004, CLIN ORTHOP RELAT R, P63, DOI 10.1097/01.blo.0000144475.05543.e7
   Bullock Mark J, 2017, Foot Ankle Spec, V10, P415, DOI 10.1177/1938640016679706
   Burrus M Tyrrell, 2016, Foot Ankle Spec, V9, P208, DOI 10.1177/1938640015609983
   Carmont MR, 2013, KNEE SURG SPORT TR A, V21, P1361, DOI 10.1007/s00167-013-2423-1
   CARR AJ, 1989, J BONE JOINT SURG BR, V71, P100, DOI 10.1302/0301-620X.71B1.2914976
   CETTI R, 1993, AM J SPORT MED, V21, P791, DOI 10.1177/036354659302100606
   Costa ML, 2006, J BONE JOINT SURG BR, V88B, P69, DOI 10.1302/0301-620X.88B1.16549
   Cretnik A, 2005, AM J SPORT MED, V33, P1369, DOI 10.1177/0363546504271501
   Cretnik Andrej, 2004, J Foot Ankle Surg, V43, P72, DOI 10.1053/j.jfas.2004.01.008
   CUMMINS EJ, 1946, SURG GYNECOL OBSTET, V83, P107
   DALKEY N, 1963, MANAGE SCI, V9, P458, DOI 10.1287/mnsc.9.3.458
   Del Buono A, 2014, BRIT MED BULL, V109, P45, DOI 10.1093/bmb/ldt029
   Deng SL, 2017, J FOOT ANKLE SURG, V56, P1236, DOI 10.1053/j.jfas.2017.05.036
   Eid A., 2014, INT SCH RES NOTICES
   Garras DN, 2012, CLIN ORTHOP RELAT R, V470, P2268, DOI 10.1007/s11999-012-2355-y
   Gigante A, 2008, KNEE SURG SPORT TR A, V16, P204, DOI 10.1007/s00167-007-0448-z
   Goren D, 2005, FOOT ANKLE INT, V26, P286, DOI 10.1177/107110070502600404
   Grassi A, 2018, J BONE JOINT SURG AM, V100, P1969, DOI 10.2106/JBJS.17.01364
   Haapasalo H, 2018, ARCH ORTHOP TRAUM SU, V138, P1089, DOI 10.1007/s00402-018-2940-y
   HASTAD K, 1959, Acta Chir Scand, V116, P251
   Heikkinen J, 2016, J BONE JOINT SURG AM, V98, P85, DOI 10.2106/JBJS.O.00496
   Highlander Peter, 2011, Foot Ankle Spec, V4, P361, DOI 10.1177/1938640011418488
   Hsu AR, 2015, FOOT ANKLE INT, V36, P1279, DOI 10.1177/1071100715589632
   Jiang N, 2012, INT ORTHOP, V36, P765, DOI 10.1007/s00264-011-1431-3
   Karabinas Panagiotis K, 2014, Eur J Orthop Surg Traumatol, V24, P607, DOI 10.1007/s00590-013-1350-7
   Keating JF, 2011, J BONE JOINT SURG BR, V93B, P1071, DOI 10.1302/0301-620X.93B8.25998
   Ketterer D J, 1991, Clin Podiatr Med Surg, V8, P401
   Khan RJK, 2005, J BONE JOINT SURG AM, V87A, P2202, DOI 10.2106/JBJS.D.03049
   Klos K, 2018, J ORTHOP SURG RES, V13, DOI 10.1186/s13018-018-0850-x
   Korntner S, 2017, SCI REP-UK, V7, DOI 10.1038/s41598-017-00700-z
   Kou J, 2010, J AM ACAD ORTHOP SUR, V18, P511, DOI 10.5435/00124635-201008000-00008
   Kubo K, 2008, ACTA PHYSIOL, V193, P257, DOI 10.1111/j.1748-1716.2008.01841.x
   Kumar A, 2018, INT J SCI INVENT TOD, V7, P687
   LAGERGREN C, 1958, ACTA RADIOL, V49, P441, DOI 10.3109/00016925809171014
   Langberg H, 1998, ACTA PHYSIOL SCAND, V163, P149, DOI 10.1046/j.1365-201X.1998.00361.x
   Langberg H, 2001, EUR J APPL PHYSIOL, V84, P246, DOI 10.1007/s004210170013
   Lantto I, 2016, AM J SPORT MED, V44, P2406, DOI 10.1177/0363546516651060
   Lawrence JE, 2017, BONE JOINT J, V99B, P87, DOI 10.1302/0301-620X.99B1.BJJ-2016-0452.R1
   Lee Daniel K, 2007, J Foot Ankle Surg, V46, P451, DOI 10.1053/j.jfas.2007.05.007
   Lim CS, 2017, FOOT ANKLE INT, V38, P1331, DOI 10.1177/1071100717728687
   Lim J, 2001, FOOT ANKLE INT, V22, P559, DOI 10.1177/107110070102200705
   Lin TW, 2004, J BIOMECH, V37, P865, DOI 10.1016/j.jbiomech.2003.11.005
   Maffulli G, 2017, MLTJ-MUSCLE LIGAMENT, V7, P46, DOI 10.11138/mltj/2017.7.1.046
   Maffulli N, 1998, AM J SPORT MED, V26, P266, DOI 10.1177/03635465980260021801
   Maffulli N, 2008, J BONE JOINT SURG AM, V90A, P1348, DOI 10.2106/JBJS.G.01241
   Maffulli N, 2011, ARCH ORTHOP TRAUM SU, V131, P33, DOI 10.1007/s00402-010-1097-0
   Magnussen RA, 2011, AM J SPORT MED, V39, P1522, DOI 10.1177/0363546510397815
   Marican MM, 2015, SINGAP MED J, V56, P549, DOI 10.11622/smedj.2015150
   Matles A L, 1975, Bull Hosp Joint Dis, V36, P48
   McMahon Samuel E, 2011, Foot Ankle Surg, V17, P211, DOI 10.1016/j.fas.2010.11.001
   Metz R, 2008, AM J SPORT MED, V36, P1688, DOI 10.1177/0363546508319312
   Moller M, 2001, J BONE JOINT SURG BR, V83B, P843
   Mortensen NHM, 1999, J BONE JOINT SURG AM, V81A, P983, DOI 10.2106/00004623-199907000-00011
   Nilsson-Helander K, 2010, AM J SPORT MED, V38, P2186, DOI 10.1177/0363546510376052
   NISTOR L, 1981, J BONE JOINT SURG AM, V63, P394, DOI 10.2106/00004623-198163030-00012
   Ofili KP, 2016, J FOOT ANKLE SURG, V55, P1245, DOI 10.1053/j.jfas.2016.01.001
   Olsson N, 2013, AM J SPORT MED, V41, P2867, DOI 10.1177/0363546513503282
   Ozer H, 2018, J FOOT ANKLE SURG, V57, P1042, DOI 10.1053/j.jfas.2018.03.005
   Pajala A, 2002, J BONE JOINT SURG AM, V84A, P2016, DOI 10.2106/00004623-200211000-00017
   Pajala A, 2009, J BONE JOINT SURG AM, V91A, P1092, DOI 10.2106/JBJS.G.01089
   PARK RE, 1986, AM J PUBLIC HEALTH, V76, P766, DOI 10.2105/AJPH.76.7.766
   Park YH, 2017, INJURY, V48, P776, DOI 10.1016/j.injury.2017.01.020
   Patel A, 2012, CLIN ORTHOP RELAT R, V470, P270, DOI 10.1007/s11999-011-2166-6
   Pean CA, 2018, J ORTHOP, V15, P226, DOI 10.1016/j.jor.2018.01.017
   Porter KJ, 2014, FOOT ANKLE SURG, V20, P90, DOI 10.1016/j.fas.2013.11.005
   Reiman M, 2014, J ATHL TRAINING, V49, P820, DOI 10.4085/1062-6050-49.3.36
   Rosenbaum Andrew J, 2010, HSS J, V6, P164, DOI 10.1007/s11420-009-9152-5
   Rozis M, 2018, FOOT ANKLE INT, V39, P689, DOI 10.1177/1071100718757971
   Sharma P, 2005, J BONE JOINT SURG AM, V87A, P187, DOI 10.2106/JBJS.D.01850
   Sheth U, 2017, BONE JOINT J, V99B, P1629, DOI 10.1302/0301-620X.99B12.BJJ-2017-0465.R1
   Shoaib A, 2017, FOOT ANKLE SURG, V23, P179, DOI 10.1016/j.fas.2016.04.006
   SIMMONDS F A, 1957, Practitioner, V179, P56
   Smoot MK, 2014, CLIN J SPORT MED, V24, P438, DOI 10.1097/JSM.0000000000000057
   Soroceanu A, 2012, J BONE JOINT SURG AM, V94A, P2136, DOI 10.2106/JBJS.K.00917
   Sorrenti SJ, 2006, FOOT ANKLE INT, V27, P407, DOI 10.1177/107110070602700603
   Spoendlin J, 2016, J DIABETES COMPLICAT, V30, P903, DOI 10.1016/j.jdiacomp.2016.02.017
   Steinbrueck K, 1997, ORTHOP T, V21, P1228
   Tejwani NC, 2014, AM J ORTHOPSYCHIAT, V43, P221
   THOMPSON T C, 1962, Acta Orthop Scand, V32, P461, DOI 10.3109/17453676208989608
   Twaddle BC, 2007, AM J SPORT MED, V35, P2033, DOI 10.1177/0363546507307503
   van der Eng DM, 2013, J FOOT ANKLE SURG, V52, P622, DOI 10.1053/j.jfas.2013.03.027
   van Maele M, 2018, INJURY, V49, P1947, DOI 10.1016/j.injury.2018.08.012
   Wallace RGH, 2011, J BONE JOINT SURG BR, V93B, P1362, DOI 10.1302/0301-620X.93B10.26187
   Wang D, 2015, FOOT ANKLE SURG, V21, P250, DOI 10.1016/j.fas.2015.01.009
   Wilkins R, 2012, AM J SPORT MED, V40, P2154, DOI 10.1177/0363546512453293
   Willits K, 2010, J BONE JOINT SURG AM, V92A, P2767, DOI 10.2106/JBJS.I.01401
   Wisbeck JM, 2012, ORTHOPEDICS, V35, pE331, DOI 10.3928/01477447-20120222-41
   Woo SLY, 1999, CLIN ORTHOP RELAT R, pS312
   Yang B, 2017, INT J SURG, V40, P178, DOI 10.1016/j.ijsu.2017.03.021
   Yilmaz G, 2014, ULUS TRAVMA ACIL CER, V20, P311, DOI 10.5505/tjtes.2014.42716
   Yotsumoto T, 2010, AM J SPORT MED, V38, P287, DOI 10.1177/0363546509351557
   Zhang YJ, 2018, AM J SPORT MED, V46, P1767, DOI 10.1177/0363546517702872
NR 111
TC 2
Z9 2
U1 4
U2 5
PU ELSEVIER SCIENCE INC
PI NEW YORK
PA STE 800, 230 PARK AVE, NEW YORK, NY 10169 USA
SN 1067-2516
EI 1542-2224
J9 J FOOT ANKLE SURG
JI J. Foot Ankle Surg.
PD JAN-FEB
PY 2021
VL 60
IS 1
BP 93
EP 101
DI 10.1053/j.jfas.2020.02.006
PG 9
WC Orthopedics; Surgery
WE Science Citation Index Expanded (SCI-EXPANDED); Social Science Citation Index (SSCI)
SC Orthopedics; Surgery
GA TF3YD
UT WOS:000670651700020
PM 33199168
OA Bronze
DA 2022-07-22
ER

PT J
AU Addevico, F
   Svedman, S
   Edman, G
   Ackermann, PW
AF Addevico, Francesco
   Svedman, Simon
   Edman, Gunnar
   Ackermann, Paul W.
TI Pyruvate and lactate as local prognostic biomarkers of patient outcome
   after achilles tendon rupture
SO SCANDINAVIAN JOURNAL OF MEDICINE & SCIENCE IN SPORTS
LA English
DT Article
DE achilles tendon; biomarker; healing; lactate; lactate-pyruvate ratio;
   patient-reported outcome measures; pyruvate; rupture
ID HEALING IN-VITRO; MICRODIALYSIS; BRAIN; DISEASE; INJURY; REPAIR; TISSUE;
   SCORE; BETA
AB Background: Acute Achilles tendon rupture (ATR) is a frequently disabling injury, which exhibits unclear variability in long-term functional and patient-reported outcomes. Biomarkers from early healing, which have been shown to be prognostic of long-term outcome would facilitate the development of improved treatment methods.
   Hypothesis/Purpose: The aim of this study was to assess essential metabolites pyruvate and its product lactate, as early biomarkers in relation to long-term functionaland patient-reported outcome after ATR.
   Study design: Prospective cohort study.
   Methods: A total of 124 patients (103 men, 21 women; mean age 40 +/- 7 years) with ATR, treated with uniform anesthetic and surgical technique, were prospectively assessed. At two weeks post-injury pyruvate and lactate concentrations were assessed in both the injured and uninjured limbs using microdialysis followed by enzymatic quantification. The ratios of the concentration in the injured versus uninjured limb of pyruvate (pyruvate-r) and lactate (lactate-r) were calculated as well as the lactate/pyruvate ratios (L/P-r). At 12 months, patient-reported outcome was examined using self-reported questionnaires; Achilles tendon Total Rupture Score (ATRS), Foot and Ankle Outcome Score (FAOS), and physical activity score. At 12 months, functional outcome was studied using the validated heel-rise test.
   Results: Elevated pyruvate-r, at two weeks, was significantly associated with total ATRS (R = 0.254, P = 0.028), less loss in physical activity (R = 0.241, P = 0.039), less experience of pain in FAOS (R = 0.275, P = 0.032), and a higher number of heel-rise repetitions on injured side (R = 0.230, P = 0.040) at 12 months. Increased lactate-r was related with less strength limitations in the calf (R = 0.283, P = 0.011), while the elevated lactate-pyruvate ratio, notably, was related to more limitations in walking on uneven surface (R = -0,243, P = 0.027). The findings were verified by multiple linear regression taking confounding factors into consideration.
   Conclusion: This study established that the metabolite pyruvate is a good potential biomarker, prognostic of patient outcome at the one-year follow-up after ATR surgery. These novel findings suggest that local biomarkers could be developed at an early-stage screen for new ATR treatments.
C1 [Addevico, Francesco] Univ Pisa, Orthopaed & Traumatol, Via Paradisa 2, I-56125 Pisa, Italy.
   [Addevico, Francesco; Svedman, Simon; Ackermann, Paul W.] Karolinska Univ Hosp, S-17176 Stockholm, Sweden.
   [Svedman, Simon; Edman, Gunnar; Ackermann, Paul W.] Karolinska Inst, Dept Mol Med & Surg, Stockholm, Sweden.
RP Addevico, F (通讯作者)，Univ Pisa, Orthopaed & Traumatol, Via Paradisa 2, I-56125 Pisa, Italy.; Addevico, F (通讯作者)，Karolinska Univ Hosp, S-17176 Stockholm, Sweden.
EM francescoaddevico@gmail.com
RI Addevico, Francesco/AAC-4692-2020; W, Ackermann P/B-9714-2008
OI Addevico, Francesco/0000-0002-9071-9881; W, Ackermann
   P/0000-0002-5520-169X
CR Ackermann PW, 2016, ADV EXP MED BIOL, V920, P293, DOI 10.1007/978-3-319-33943-6_28
   Alim MA, 2016, BMJ OPEN SPORT EXERC, V2, P1
   BORDER WA, 1992, J CLIN INVEST, V90, P1, DOI 10.1172/JCI115821
   de la Pena A, 2000, ADV DRUG DELIVER REV, V45, P189, DOI 10.1016/S0169-409X(00)00106-X
   Domeij-Arverud E, 2015, BONE JOINT J, V97B, P675, DOI 10.1302/0301-620X.97B5.34581
   Fessel G, 2014, PLOS ONE, V9, DOI 10.1371/journal.pone.0110948
   Greve K, 2012, SCAND J MED SCI SPOR, V22, pE55, DOI 10.1111/j.1600-0838.2012.01475.x
   Harvey SAK, 2010, INVEST OPHTH VIS SCI, V51, P2917, DOI 10.1167/iovs.09-4498
   Hsu AR, 2015, FOOT ANKLE INT, V36, P1279, DOI 10.1177/1071100715589632
   Kannus P, 1997, SCAND J MED SCI SPOR, V7, P53
   Klein MB, 2001, J HAND SURG-AM, V26A, P847, DOI 10.1053/jhsu.2001.26185
   Langberg H, 2002, ACTA PHYSIOL SCAND, V174, P377, DOI 10.1046/j.1365-201x.2002.00964.x
   Magnoni S, 2003, J NEUROSURG, V98, P952, DOI 10.3171/jns.2003.98.5.0952
   Moller M, 2005, KNEE SURG SPORT TR A, V13, P60, DOI 10.1007/s00167-003-0441-0
   Nilsson-Helander K, 2007, AM J SPORT MED, V35, P421, DOI 10.1177/0363546506294856
   Olesen JL, 2006, SCAND J RHEUMATOL, V35, P312, DOI 10.1080/03009740600556373
   Oliva F, 2016, BRIT MED BULL, V117, P39, DOI 10.1093/bmb/ldv054
   Olsson N, 2011, KNEE SURG SPORT TR A, V19, P1385, DOI 10.1007/s00167-011-1511-3
   Peskind ER, 2015, JAMA NEUROL, V72, P1103, DOI 10.1001/jamaneurol.2015.1789
   Roos EM, 2001, FOOT ANKLE INT, V22, P788, DOI 10.1177/107110070102201004
   Scott GF, 2017, EXP BIOL MED, V242, P1095, DOI 10.1177/1535370217703353
   Sharma R, 2012, CURR NEUROL NEUROSCI, V12, P560, DOI 10.1007/s11910-012-0301-8
   Silbernagel KG, 2010, KNEE SURG SPORT TR A, V18, P258, DOI 10.1007/s00167-009-0889-7
   Svedman S, 2018, KNEE SURG SPORT TR A, V26, P2011, DOI 10.1007/s00167-017-4606-7
   Vacanti NM, 2014, MOL CELL, V56, P425, DOI 10.1016/j.molcel.2014.09.024
   Valkering KP, 2017, KNEE SURG SPORT TR A, V25, P1807, DOI 10.1007/s00167-016-4270-3
   Wang D, 2015, FOOT ANKLE SURG, V21, P250, DOI 10.1016/j.fas.2015.01.009
   Yalamanchi N, 2004, PLAST RECONSTR SURG, V113, P625, DOI 10.1097/01.PRS.0000101529.47062.34
   Zhang K, 2018, AM J SPORT MED, V46, P2222, DOI 10.1177/0363546518778789
   Zoremba N, 2014, ACTA VET SCAND, V56, DOI 10.1186/s13028-014-0072-0
NR 30
TC 2
Z9 3
U1 0
U2 3
PU WILEY
PI HOBOKEN
PA 111 RIVER ST, HOBOKEN 07030-5774, NJ USA
SN 0905-7188
EI 1600-0838
J9 SCAND J MED SCI SPOR
JI Scand. J. Med. Sci. Sports
PD OCT
PY 2019
VL 29
IS 10
BP 1529
EP 1536
DI 10.1111/sms.13469
PG 8
WC Sport Sciences
WE Science Citation Index Expanded (SCI-EXPANDED)
SC Sport Sciences
GA JB5PE
UT WOS:000488616400009
PM 31102560
DA 2022-07-22
ER

PT J
AU Leahy, TP
   Nuss, CA
   Evans, MK
   Fung, AK
   Shetye, SS
   Soslowsky, LJ
AF Leahy, Thomas P.
   Nuss, Courtney A.
   Evans, Mary Kate
   Fung, Ashley K.
   Shetye, Snehal S.
   Soslowsky, Louis J.
TI Achilles Tendon Ruptures in Middle-Aged Rats Heal Poorly Compared With
   Those in Young and Old Rats
SO AMERICAN JOURNAL OF SPORTS MEDICINE
LA English
DT Article
DE Achilles tendon; biomechanics of tendon; biology of tendon; ankle
ID SPATIAL-FREQUENCY PARAMETERS; ROTATOR CUFF TEARS; MECHANICAL-PROPERTIES;
   TENDINOPATHY; INFERIOR; INJURY; EPIDEMIOLOGY; ETIOLOGY
AB Background: Achilles tendon ruptures are painful and debilitating injuries and are most common in middle-aged patients. There is a lack of understanding of the underlying causes for increased rupture rates in middle-aged patients and how healing outcomes after a rupture might be affected by patient age. Therefore, the objective of this study was to define age-specific Achilles tendon healing by assessing ankle functional outcomes and Achilles tendon mechanical and histological properties after a rupture using a rat model.
   Hypothesis: Rats representing the middle-aged patient population would demonstrate reduced healing capability after an Achilles tendon rupture, as demonstrated by a slower return to baseline ankle functional properties and inferior biomechanical and histological tendon properties.
   Study Design: Controlled laboratory study.
   Methods: Fischer 344 rats were categorized by age to represent young, middle-aged, and old patients, and Achilles tendon ruptures were induced in the right hindlimb. Animals were allowed to heal and were euthanized at 3 or 6 weeks after the injury. In vivo functional assays and ultrasound imaging were performed throughout the healing period, and ex vivo tendon mechanical and histological properties were assessed after euthanasia.
   Results: Rats representing middle-aged patients displayed reduced healing potential compared with the other age groups, as they demonstrated decreased recovery of in vivo functional and ultrasound assessment parameters and inferior mechanical and histological properties after an Achilles tendon rupture.
   Conclusion: These findings may help explain the increased rupture rate observed clinically in middle-aged patients by suggesting that there may be altered tendon responses to daily trauma.
C1 [Leahy, Thomas P.; Nuss, Courtney A.; Evans, Mary Kate; Fung, Ashley K.; Shetye, Snehal S.; Soslowsky, Louis J.] Univ Penn, Philadelphia, PA 19104 USA.
   [Leahy, Thomas P.; Nuss, Courtney A.; Evans, Mary Kate; Fung, Ashley K.; Shetye, Snehal S.; Soslowsky, Louis J.] Univ Penn, McKay Orthopaed Res Lab, 3450 Hamilton Walk,307A Stemmler Hall, Philadelphia, PA 19104 USA.
   [Leahy, Thomas P.; Evans, Mary Kate; Fung, Ashley K.; Soslowsky, Louis J.] Univ Penn, Dept Bioengn, Philadelphia, PA 19104 USA.
RP Soslowsky, LJ (通讯作者)，Univ Penn, McKay Orthopaed Res Lab, 3450 Hamilton Walk,307A Stemmler Hall, Philadelphia, PA 19104 USA.
EM soslowsk@upenn.edu
RI Shetye, Snehal/AAJ-3805-2020
OI Leahy, Thomas/0000-0002-2749-6021; Shetye, Snehal/0000-0001-7469-3039
FU NIH/NIAMS [T32AR007132]; Penn Center for Musculoskeletal Disorders
   [P30AR069619]; AOSSM
FX One or more of the authors has declared the following potential conflict
   of interest or source of funding: This study was supported by the
   NIH/NIAMS (T32AR007132) and the Penn Center for Musculoskeletal
   Disorders (P30AR069619). AOSSM checks author disclosures against the
   Open Payments Database (OPD). AOSSM has not conducted an independent
   investigation on the OPD and disclaims any liability or responsibility
   relating thereto.
CR Ackerman JE, 2019, J ORTHOP RES, V37, P2476, DOI 10.1002/jor.24397
   Ackerman JE, 2017, J ORTHOP RES, V35, P2716, DOI 10.1002/jor.23580
   Bashford GR, 2008, IEEE T MED IMAGING, V27, P608, DOI 10.1109/TMI.2007.912389
   Chang KV, 2012, J ULTRAS MED, V31, P737
   Cheema AN, 2019, J ORTHOP RES, V37, P94, DOI 10.1002/jor.24167
   Chen MC, 2020, J BIOMECH ENG-T ASME, V142, DOI 10.1115/1.4046864
   Clayton RAE, 2008, INJURY, V39, P1338, DOI 10.1016/j.injury.2008.06.021
   Connizzo BK, 2013, J BIOMECH ENG-T ASME, V135, DOI 10.1115/1.4023234
   Deuis JR, 2017, FRONT MOL NEUROSCI, V10, DOI 10.3389/fnmol.2017.00284
   Dunkman AA, 2013, MATRIX BIOL, V32, P3, DOI 10.1016/j.matbio.2012.11.005
   Ensey JS, 2009, BIOMED ENG ONLINE, V8, DOI 10.1186/1475-925X-8-12
   Fredberg U, 2008, SCAND J MED SCI SPOR, V18, P3, DOI 10.1111/j.1600-0838.2007.00746.x
   Freedman BR, 2017, J AM ACAD ORTHOP SUR, V25, P635, DOI 10.5435/JAAOS-D-16-00620
   Freedman BR, 2017, J BIOMECH, V56, P55, DOI 10.1016/j.jbiomech.2017.02.030
   Freedman BR, 2016, J ORTHOP RES, V34, P2172, DOI 10.1002/jor.23253
   Fryhofer GW, 2016, J APPL PHYSIOL, V121, P1106, DOI 10.1152/japplphysiol.00620.2016
   Galatz LM, 2015, J ORTHOP RES, V33, P823, DOI 10.1002/jor.22853
   Gibbon WW, 1999, BRIT J SPORT MED, V33, P129, DOI 10.1136/bjsm.33.2.129
   Gulati V, 2015, WORLD J ORTHOP, V6, P380, DOI 10.5312/wjo.v6.i4.380
   Gwynne-Jones DP, 2011, FOOT ANKLE INT, V32, P337, DOI 10.3113/FAI.2011.0337
   Hillin CD, 2019, J ORTHOP RES, V37, P562, DOI 10.1002/jor.24241
   Huegel J, 2020, J ORTHOP RES, V38, P70, DOI 10.1002/jor.24487
   Huegel J, 2019, J BIOMECH, V88, P194, DOI 10.1016/j.jbiomech.2019.03.029
   Huttunen TT, 2014, AM J SPORT MED, V42, P2419, DOI 10.1177/0363546514540599
   Ishigaki T, 2020, CLIN BIOMECH, V71, P5, DOI 10.1016/j.clinbiomech.2019.10.003
   KANNUS P, 1991, J BONE JOINT SURG AM, V73A, P1507, DOI 10.2106/00004623-199173100-00009
   Kubo K, 2007, MED SCI SPORT EXER, V39, P541, DOI 10.1249/01.mss.0000247006.24965.74
   Kulig K, 2016, ULTRASOUND MED BIOL, V42, P664, DOI 10.1016/j.ultrasmedbio.2015.11.013
   Maffulli N, 2004, J ROY SOC MED, V97, P472, DOI 10.1258/jrsm.97.10.472
   Maffulli N, 1999, CLIN J SPORT MED, V9, P157, DOI 10.1097/00042752-199907000-00007
   Mian OS, 2007, ACTA PHYSIOL, V189, P57, DOI 10.1111/j.1748-1716.2006.01634.x
   Minagawa H, 2013, J ORTHOP, V10, P8, DOI 10.1016/j.jor.2013.01.008
   Morse CI, 2005, J APPL PHYSIOL, V98, P221, DOI 10.1152/japplphysiol.00774.2004
   Needles A, 2010, ULTRASOUND MED BIOL, V36, P2097, DOI 10.1016/j.ultrasmedbio.2010.08.012
   Nielsen HM, 1998, MECH AGEING DEV, V100, P243, DOI 10.1016/S0047-6374(97)00147-4
   Onambele GL, 2006, J APPL PHYSIOL, V100, P2048, DOI 10.1152/japplphysiol.01442.2005
   Pardes AM, 2017, J BIOMECH, V60, P30, DOI 10.1016/j.jbiomech.2017.06.008
   Pardes AM, 2016, ANN BIOMED ENG, V44, P2901, DOI 10.1007/s10439-016-1635-1
   Pearson SJ, 2017, J BIOMECH, V57, P136, DOI 10.1016/j.jbiomech.2017.03.017
   Peltz CD, 2009, J ORTHOP RES, V27, P416, DOI 10.1002/jor.20770
   Plate JF, 2013, J BIOMECH, V46, P450, DOI 10.1016/j.jbiomech.2012.11.007
   Raikin SM, 2013, FOOT ANKLE INT, V34, P475, DOI 10.1177/1071100713477621
   Riggin CN, 2021, J ORTHOP RES, V39, P2000, DOI 10.1002/jor.24861
   Riggin CN, 2019, ULTRASOUND MED BIOL, V45, P1841, DOI 10.1016/j.ultrasmedbio.2019.03.002
   Sarver JJ, 2010, J BIOMECH, V43, P778, DOI 10.1016/j.jbiomech.2009.10.031
   Sengupta P, 2013, INT J PREVENTIVE MED, V4, P624
   Stenroth L, 2012, J APPL PHYSIOL, V113, P1537, DOI 10.1152/japplphysiol.00782.2012
   Tempfer H, 2015, FRONT PHYSIOL, V6, DOI 10.3389/fphys.2015.00330
   Tucker JJ, 2016, J ORTHOP RES, V34, P2009, DOI 10.1002/jor.23225
   Turan A, 2015, MED ULTRASON, V17, P58, DOI 10.11152/mu.2013.2066.171.ayt
   Wood LK, 2011, J APPL PHYSIOL, V111, P999, DOI 10.1152/japplphysiol.00460.2011
   Wunderli SL, 2020, MATRIX BIOL, V89, P11, DOI 10.1016/j.matbio.2019.12.003
NR 52
TC 0
Z9 0
U1 3
U2 4
PU SAGE PUBLICATIONS INC
PI THOUSAND OAKS
PA 2455 TELLER RD, THOUSAND OAKS, CA 91320 USA
SN 0363-5465
EI 1552-3365
J9 AM J SPORT MED
JI Am. J. Sports Med.
PD JAN
PY 2022
VL 50
IS 1
BP 170
EP 181
DI 10.1177/03635465211055476
EA DEC 2021
PG 12
WC Orthopedics; Sport Sciences
WE Science Citation Index Expanded (SCI-EXPANDED)
SC Orthopedics; Sport Sciences
GA YC1WV
UT WOS:000727780800001
PM 34851182
DA 2022-07-22
ER

PT J
AU Ngai, WYH
   Chan, SCF
AF Ngai, William Y. H.
   Chan, Samson C. F.
TI An Uncomplicated Method for Minimally Invasive Achilles Tendon Repair
SO JOURNAL OF FOOT & ANKLE SURGERY
LA English
DT Article
DE rupture; sponge forceps; surgery; trauma
ID RUPTURE
AB The Achilles tendon is the largest palpable tendon in the human body, and rupture of this tendon is not an uncommon injury encountered by foot and ankle surgeons. A number of different minimally invasive methods have been described for repair of the ruptured Achilles tendon. In this article, we describe a relatively simple, minimally invasive technique of Achilles tendon repair that does not require special instrumentation, the key requirement being that of a sponge forceps. (C) 2010 by the American College of Foot and Ankle Surgeons. Published by Elsevier Inc. All rights reserved.
C1 [Ngai, William Y. H.; Chan, Samson C. F.] Tseung Kwan O Hosp, Dept Orthopaed & Traumatol, Tseung Kwan O, Hong Kong, Peoples R China.
RP Ngai, WYH (通讯作者)，Tseung Kwan O Hosp, Dept Orthopaed & Traumatol, Tseung Kwan O, Hong Kong, Peoples R China.
EM drwilliamngai@gmail.com
CR Aktas Seref, 2007, J Foot Ankle Surg, V46, P336, DOI 10.1053/j.jfas.2007.06.006
   Aktas S, 2009, FOOT ANKLE INT, V30, P391, DOI 10.3113/FAI.2009.0391
   Atinga M, 2008, FOOT ANKLE INT, V29, P814, DOI 10.3113/FAI.2008.0814
   Cretnik Andrej, 2004, J Foot Ankle Surg, V43, P72, DOI 10.1053/j.jfas.2004.01.008
   Gorschewsky O, 1999, INJURY, V30, P315, DOI 10.1016/S0020-1383(99)00089-3
   Hohendorff B, 2008, J FOOT ANKLE SURG, V47, P392, DOI 10.1053/j.jfas.2008.05.006
   Kupcha Paul C, 2008, Am J Orthop (Belle Mead NJ), V37, P586
   Lee DK, 2008, J FOOT ANKLE SURG, V47, P8, DOI 10.1053/j.jfas.2007.08.015
   MA GWC, 1977, CLIN ORTHOP RELAT R, P247
   Maffulli N, 1999, J BONE JOINT SURG AM, V81A, P1019, DOI 10.2106/00004623-199907000-00017
   Metz R, 2008, AM J SPORT MED, V36, P1688, DOI 10.1177/0363546508319312
   Ng E S, 2006, J Orthop Surg (Hong Kong), V14, P142
   Rebeccato A, 2001, J Foot Ankle Surg, V40, P188
   Sebastian Hermann, 2007, J Foot Ankle Surg, V46, P424, DOI 10.1053/j.jfas.2007.07.003
NR 14
TC 4
Z9 6
U1 0
U2 0
PU ELSEVIER SCIENCE INC
PI NEW YORK
PA 360 PARK AVE SOUTH, NEW YORK, NY 10010-1710 USA
SN 1067-2516
EI 1542-2224
J9 J FOOT ANKLE SURG
JI J. Foot Ankle Surg.
PD MAR-APR
PY 2010
VL 49
IS 2
BP 208
EP 211
DI 10.1053/j.jfas.2009.12.011
PG 4
WC Orthopedics; Surgery
WE Science Citation Index Expanded (SCI-EXPANDED)
SC Orthopedics; Surgery
GA 561EH
UT WOS:000274958100028
PM 20188285
DA 2022-07-22
ER

PT J
AU Huang, TF
   Perry, SM
   Soslowsky, LJ
AF Huang, TF
   Perry, SM
   Soslowsky, LJ
TI The effect of overuse activity on Achilles tendon in an animal model: A
   biomechanical study
SO ANNALS OF BIOMEDICAL ENGINEERING
LA English
DT Article
DE exercise; injury; mechanical testing; rat; rotator cuff; soft tissue
   mechanics; supraspinatus
ID ROTATOR CUFF TENDINOSIS; SUPRASPINATUS TENDON; INJURIES; RUPTURE; RAT;
   EXERCISE
AB Musculoskeletal Soft tissue injuries from athletic activities are common in the rotator cuff tendons, lateral epicondyle of the elbow, the patella tendon, and the Achilles tendon. Despite the fact that the Achilles tendon is the largest and strongest tendon in the human body, it is frequently injured in the athletic setting. To study the etiology and pathogenesis of Achilles tendon injuries, our goal was to develop a model of Achilles tendon overuse by evaluating the Achilles tendons from animals subjected to the exercise protocol previously described as overuse for the supraspinatus tendon. We hypothesized that the same exercise protocol would produce injuries to the Achilles tendon as demonstrated by changes in the cross-sectional area and biomechanical properties. While a significant injury was induced into the supraspinatus tendon, we found no changes in the Achilles tendons of these exercised animals based on gross observation, geometric measurements, and mechanical testing analyses. Although surprising, there are many possible explanations for these findings including differences in potential injury mechanisms, functional capabilities of the differing tendons, and other factors.
C1 Univ Penn, McKay Orthopaed Res Lab, Philadelphia, PA 19104 USA.
   Taipei Vet Gen Hosp, Dept Orthopaed & Traumatol, Taipei, Taiwan.
   Natl Yang Ming Univ, Sch Med, Taipei 112, Taiwan.
RP Soslowsky, LJ (通讯作者)，Univ Penn, McKay Orthopaed Res Lab, 424 Stemmler Hall,3450 Hamilton Walk, Philadelphia, PA 19104 USA.
EM soslowsk@mail.med.upenn.edu
CR Ahmed IM, 1998, J ORTHOPAED RES, V16, P591, DOI 10.1002/jor.1100160511
   Archambault JM, 2001, CONNECT TISSUE RES, V42, P13, DOI 10.3109/03008200109014245
   ARMSTRONG RB, 1983, J APPL PHYSIOL, V54, P80, DOI 10.1152/jappl.1983.54.1.80
   Buchanan CI, 2001, J APPL PHYSIOL, V90, P164, DOI 10.1152/jappl.2001.90.1.164
   Carpenter JE, 1998, AM J SPORT MED, V26, P801, DOI 10.1177/03635465980260061101
   CODMAN EA, 1990, CLIN ORTHOP RELAT R, V254, P3
   GALLOWAY MT, 1992, CLIN SPORT MED, V11, P771
   IANNOTTI J, 1991, PATHOGENESIS ROTATOR
   Jarvinen TAH, 2001, CURR OPIN RHEUMATOL, V13, P150
   Kannus P, 1997, SCAND J MED SCI SPOR, V7, P107
   LEPPILAHTI J, 1991, ANN CHIR GYNAECOL FE, V80, P202
   Leppilahti J, 1998, SPORTS MED, V25, P79, DOI 10.2165/00007256-199825020-00002
   Leppilahti J, 1996, ACTA ORTHOP SCAND, V67, P277, DOI 10.3109/17453679608994688
   LOHR JF, 1990, CLIN ORTHOP RELAT R, P35
   Maffulli N, 2000, AM J SPORT MED, V28, P499, DOI 10.1177/03635465000280040901
   Magnusson SP, 2003, EUR J APPL PHYSIOL, V90, P549, DOI 10.1007/s00421-003-0865-8
   Mazzone MF, 2002, AM FAM PHYSICIAN, V65, P1805
   Messner K, 1999, CELLS TISSUES ORGANS, V165, P30, DOI 10.1159/000016671
   Pyne D B, 1997, Aust J Sci Med Sport, V29, P77
   Schepsis AA, 2002, AM J SPORT MED, V30, P287, DOI 10.1177/03635465020300022501
   SOMMER HM, 1987, INT ORTHOP, V11, P71, DOI 10.1007/BF00266061
   Soslowsky L J, 1996, J Shoulder Elbow Surg, V5, P383
   Soslowsky LJ, 2002, ANN BIOMED ENG, V30, P1057, DOI 10.1114/1.1509765
   Soslowsky LJ, 2000, J SHOULDER ELB SURG, V9, P79, DOI 10.1067/mse.2000.101962
   Tomczak R L, 2001, Clin Podiatr Med Surg, V18, P255
NR 25
TC 59
Z9 63
U1 1
U2 8
PU SPRINGER
PI NEW YORK
PA 233 SPRING ST, NEW YORK, NY 10013 USA
SN 0090-6964
EI 1573-9686
J9 ANN BIOMED ENG
JI Ann. Biomed. Eng.
PD MAR
PY 2004
VL 32
IS 3
BP 336
EP 341
DI 10.1023/B:ABME.0000017537.26426.76
PG 6
WC Engineering, Biomedical
WE Science Citation Index Expanded (SCI-EXPANDED)
SC Engineering
GA 835FB
UT WOS:000222465100004
PM 15095808
DA 2022-07-22
ER

PT J
AU Noh, JH
   Roh, YH
   Lee, K
   Lee, JS
AF Noh, Jung Ho
   Roh, Young Hak
   Lee, Kang
   Lee, Jun Suk
TI Achilles tendon allograft with its bony attachment to repair rupture and
   extensive degeneration of the heel cord
SO ACTA ORTHOPAEDICA BELGICA
LA English
DT Article
DE Achilles tendon; rupture; degeneration; allograft
AB An Achilles tendon allograft with its bony calcaneal attachment was successfully used to repair a spontaneous rupture of a severely degenerative and irreparable Achilles tendon. After 18 months of follow-up, the patient is satisfied and the Achilles tendon power has returned to a normal level.
C1 [Noh, Jung Ho; Roh, Young Hak; Lee, Kang; Lee, Jun Suk] Kangwon Natl Univ Hosp, Chuncheon Si, South Korea.
RP Roh, YH (通讯作者)，Gachon Univ, Sch Med, Gil Med Ctr, Dept Orthopaed Surg, 1198 Guwol Dong, Inchon 405760, South Korea.
EM ryhak@hanmail.net
CR Battaglia TC, 2006, ORTHOPEDICS, V29, P453, DOI 10.3928/01477447-20060501-11
   Mady F, 2000, FOOT ANKLE INT, V21, P1015, DOI 10.1177/107110070002101206
   Postacchini F, 1983, Ital J Orthop Traumatol, V9, P529
   WESELEY M S, 1976, Bulletin of the Hospital for Joint Diseases, V37, P159
NR 4
TC 3
Z9 3
U1 0
U2 0
PU ACTA MEDICA BELGICA
PI BRUSSELS
PA AVENUE WINSTON CHURCHILL, WINSTON CHURCHILL-LAAN, 11 BOX 30, B-1180
   BRUSSELS, BELGIUM
SN 0001-6462
J9 ACTA ORTHOP BELG
JI Acta Orthop. Belg.
PD OCT
PY 2012
VL 78
IS 5
BP 678
EP 680
PG 3
WC Orthopedics
WE Science Citation Index Expanded (SCI-EXPANDED)
SC Orthopedics
GA 036UW
UT WOS:000311058700018
PM 23162967
DA 2022-07-22
ER

PT J
AU Carmont, MR
   Maffulli, N
AF Carmont, Michael R.
   Maffulli, Nicola
TI Modified percutaneous repair of ruptured Achilles tendon
SO KNEE SURGERY SPORTS TRAUMATOLOGY ARTHROSCOPY
LA English
DT Article
DE percutaneous; Achilles; tendon; repair
ID SURAL NERVE; SUTURE; IMMOBILIZATION
AB Percutaneous repair of the Achilles tendon is now well established. Sural nerve injury is one of the commonest complications following this method of repair. We present our current method of percutaneous repair which permits a less invasive approach to the tendon, accurate opposition of the tendon ends, and minimises the chance of sural nerve injury.
C1 [Carmont, Michael R.; Maffulli, Nicola] Keele Univ, Sch Med, Univ Hosp N Staffordshire, Hartshill Orthopaed Surg Unit,Dept Trauma & Ortho, Stoke On Trent ST7 4QG, Staffs, England.
RP Carmont, MR (通讯作者)，Keele Univ, Sch Med, Univ Hosp N Staffordshire, Hartshill Orthopaed Surg Unit,Dept Trauma & Ortho, Stoke On Trent ST7 4QG, Staffs, England.
EM mcarmont@hotmail.com; n.maffulli@orth.keele.ac.uk
RI Maffulli, Nicola/A-1839-2014
OI Maffulli, Nicola/0000-0002-5327-3702
CR Barrie KA, 2000, J HAND SURG-AM, V25A, P714, DOI 10.1053/jhsu.2000.9414
   Buchgraber A, 1997, CLIN ORTHOP RELAT R, P113
   Costa ML, 2006, J BONE JOINT SURG AM, V88A, P1894, DOI 10.2106/JBJS.8808.ebo2
   Cretnik Andrej, 2004, J Foot Ankle Surg, V43, P72, DOI 10.1053/j.jfas.2004.01.008
   FLAVIN R, 2006, BRIT ORTH FOOT ANKL
   KESSLER I, 1969, ACTA ORTHOP SCAND, V40, P587, DOI 10.3109/17453676908989524
   MA GWC, 1977, CLIN ORTHOP RELAT R, P247
   Maes Renaud, 2006, Acta Orthop Belg, V72, P179
   Majewski M, 2006, AM J SPORT MED, V34, P793, DOI 10.1177/0363546505283266
   Matles A L, 1975, Bull Hosp Joint Dis, V36, P48
   McClelland D, 2002, J ROY COLL SURG EDIN, V47, P613
   NEWPORT ML, 1992, J HAND SURG-AM, V17A, P1117, DOI 10.1016/S0363-5023(09)91077-2
   SIMMONDS F A, 1957, Practitioner, V179, P56
   Tang KL, 2007, AM J SPORT MED, V35, P589, DOI 10.1177/0363546506295080
   Thermann H, 2001, FOOT ANKLE INT, V22, P158, DOI 10.1177/107110070102200214
   Waldram M, 2003, TRAUMA, V5, P79, DOI DOI 10.1191/1460408603TA2370A
   Webb J, 2000, FOOT ANKLE INT, V21, P475, DOI 10.1177/107110070002100604
   Webb JM, 1999, J BONE JOINT SURG BR, V81B, P877, DOI 10.1302/0301-620X.81B5.9784
NR 18
TC 66
Z9 70
U1 1
U2 3
PU SPRINGER
PI NEW YORK
PA 233 SPRING ST, NEW YORK, NY 10013 USA
SN 0942-2056
EI 1433-7347
J9 KNEE SURG SPORT TR A
JI Knee Surg. Sports Traumatol. Arthrosc.
PD FEB
PY 2008
VL 16
IS 2
BP 199
EP 203
DI 10.1007/s00167-007-0411-z
PG 5
WC Orthopedics; Sport Sciences; Surgery
WE Science Citation Index Expanded (SCI-EXPANDED)
SC Orthopedics; Sport Sciences; Surgery
GA 258NS
UT WOS:000252875200019
PM 17874233
DA 2022-07-22
ER

PT J
AU Guerra, FD
   Vieira, CP
   de Almeida, MD
   Oliveira, LP
   Claro, ACF
   Simoes, GF
   de Oliveira, ALR
   Pimentel, ER
AF Guerra, Flavia Da Re
   Vieira, Cristiano Pedrozo
   de Almeida, Marcos dos Santos
   Oliveira, Leticia Prado
   Ferreira Claro, Ana Carolina
   Simoes, Gustavo Ferreira
   Rodrigues de Oliveira, Alexandre Leite
   Pimentel, Edson Rosa
TI Pulsed LLLT improves tendon healing in rats: a biochemical,
   organizational, and functional evaluation
SO LASERS IN MEDICAL SCIENCE
LA English
DT Article
DE Achilles tendon; Gait analysis; Rehabilitation program; Soft tissue
   injuries; Collagen
ID HUMAN ACHILLES-TENDON; LASER IRRADIATION; COLLAGENS; INJURY;
   GLYCOSAMINOGLYCANS; TENDINOPATHY; BEHAVIOR; RUPTURES; REPAIR
AB In the last decades, the tendon injuries have increased substantially. Previous results suggested that low-level laser treatment (LLLT) promotes synthesis of extracellular matrix and improves the functional properties of the tendon. The aim of this study was to evaluate the effects of different protocols of LLLT on partially tenotomized tendons. Adult male rats were divided into the following: G1-intact, G2-injured, G3-injured + LLLT (4 J/cm(2) continuous), G4-injured + LLLT (4 J/cm(2) at 20 Hz). G2, G3, and G4 were euthanized 8 days after injury. G5-injured, G6-injured + LLLT (4 J/cm(2) continuous), and G7-injured + LLL (4 J/cm(2) at 20 Hz until the seventh day and 2 kHz from 8 to 14 days). G5, G6, and G7 were euthanized on the 15th day. Glycosaminoglycan (GAG) level was quantified by dimethylmethylene blue method and analyzed on agarose gel. Toluidine blue (TB) stain was used to observe metachromasy. CatWalk system was used to evaluate gait recovery. Collagen organization was analyzed by polarization microscopy. The GAG level increased in all transected groups, except G5. In G6 and G7, there was a significant increase in GAG in relation to G5. In G3 and G4, the presence of dermatan sulfate band was more prominent than G2. TB stains showed intense metachromasy in the treated groups. Birefringence analysis showed improvement in collagen organization in G7. The gait was significantly improved in G7. In conclusion, pulsed LLLT leads to increased organization of collagen bundles and improved gait recovery.
C1 [Guerra, Flavia Da Re; Vieira, Cristiano Pedrozo; de Almeida, Marcos dos Santos; Oliveira, Leticia Prado; Ferreira Claro, Ana Carolina; Simoes, Gustavo Ferreira; Rodrigues de Oliveira, Alexandre Leite; Pimentel, Edson Rosa] Univ Campinas UNICAMP, Inst Biol, Dept Struct & Funct Biol, BR-13083970 Campinas, SP, Brazil.
RP Guerra, FD (通讯作者)，Univ Campinas UNICAMP, Inst Biol, Dept Struct & Funct Biol, CP 6109, BR-13083970 Campinas, SP, Brazil.
EM dgflavia@yahoo.com.br
RI Oliveira, Alexandre L. R./C-4436-2012; Vieira, Cristiano
   Pedrozo/L-2517-2014; Da+Ré+Guerra, Flávia/ABE-6224-2021; Guerra, Flavia
   D/N-6274-2014; Oliveira, Alexandre/AAI-6815-2020
OI Oliveira, Alexandre L. R./0000-0003-4224-4575; Guerra, Flavia
   D/0000-0001-9142-9109; Oliveira, Alexandre/0000-0003-4224-4575; Almeida,
   Marcos/0000-0002-2080-0609; , Gustavo/0000-0001-5362-4085
FU FAPESP [2010/52383-2]; CNPq
FX The authors thank FAPESP for the financial support (2010/52383-2). F. D.
   Guerra was the recipient of a CNPq fellowship. We also thank Francisco
   A. Malatesta for his expert technical assistance and IBRAMED for the LLL
   equipment.
CR Aro AA, 2013, INJURY, V44, P884, DOI 10.1016/j.injury.2012.08.055
   Bosch U, 1998, AM J SPORT MED, V26, P103, DOI 10.1177/03635465980260010201
   Bosman FT, 2003, J PATHOL, V200, P423, DOI 10.1002/path.1437
   Chan HKF, 2007, J ORTHOP SPORT PHYS, V37, P399, DOI 10.2519/jospt.2007.2412
   Chen CH, 2009, J ORTHOP RES, V27, P646, DOI 10.1002/jor.20800
   Chen ML, 2008, J MOL NEUROSCI, V34, P89, DOI 10.1007/s12031-007-9005-y
   Comper WD, 1996, EXTRACELLULAR MATRIX, P200
   de Almeida MD, 2012, CONNECT TISSUE RES, V53, P542, DOI 10.3109/03008207.2012.710671
   Demir H, 2004, LASER SURG MED, V35, P84, DOI 10.1002/lsm.20046
   DIETRICH CP, 1976, ANAL BIOCHEM, V70, P645, DOI 10.1016/0003-2697(76)90496-6
   ENWEMEKA C S, 1991, Journal of Orthopaedic and Sports Physical Therapy, V14, P198
   Enwemeka CS, 2000, LASER THER, V12, P22, DOI DOI 10.5978/ISLSM.12.22
   FARNDALE RW, 1986, BIOCHIM BIOPHYS ACTA, V883, P173, DOI 10.1016/0304-4165(86)90306-5
   Gelse K, 2003, ADV DRUG DELIVER REV, V55, P1531, DOI 10.1016/j.addr.2003.08.002
   Guerra FD, 2013, LASER MED SCI, V28, P1281, DOI 10.1007/s10103-012-1236-7
   Jarvinen Tero A H, 2005, Foot Ankle Clin, V10, P255, DOI 10.1016/j.fcl.2005.01.013
   Karousou E, 2008, CLIN ORTHOP RELAT R, V466, P1577, DOI 10.1007/s11999-008-0255-y
   Kongsgaard M, 2005, J APPL PHYSIOL, V99, P1965, DOI 10.1152/japplphysiol.00384.2005
   Maffulli N, 1999, J BONE JOINT SURG AM, V81A, P1019, DOI 10.2106/00004623-199907000-00017
   Maffulli N, 2003, CLIN SPORT MED, V22, P675, DOI 10.1016/S0278-5919(03)00004-8
   MURPHY PG, 1994, BIOCHEM CELL BIOL, V72, P403, DOI 10.1139/o94-054
   Oakes B.W., 2003, REHABILITATION SPORT, P56
   Oliveira FS, 2009, LASER SURG MED, V41, P271, DOI 10.1002/lsm.20760
   POSTACCHINI F, 1976, INT SURG, V61, P14
   Pugliese Livia Souza, 2003, Pesqui Odontol Bras, V17, P307
   REID T, 1974, J EMBRYOL EXP MORPH, V31, P489
   Request Privilege of Utility Model in Brazil titled, RETAINER RATS LASER
   Salate ACB, 2005, PHOTOMED LASER SURG, V23, P470, DOI 10.1089/pho.2005.23.470
   SEIBERT K, 1994, P NATL ACAD SCI USA, V91, P12013, DOI 10.1073/pnas.91.25.12013
   Sharma P., 2006, Journal of Musculoskeletal & Neuronal Interactions, V6, P181
   Sharma P, 2005, J BONE JOINT SURG AM, V87A, P187, DOI 10.2106/JBJS.D.01850
   Silver FH, 2002, J THEOR BIOL, V216, P243, DOI 10.1006/jtbi.2002.2542
   Sommer AP, 2011, ARTIF CELL BLOOD SUB, V39, P169, DOI 10.3109/10731199.2010.516262
   Szaro P, 2009, ANN ANAT, V191, P586, DOI 10.1016/j.aanat.2009.07.006
   Tanaka E, 2003, CRIT REV ORAL BIOL M, V14, P138, DOI 10.1177/154411130301400207
   Thompson J, 2011, CLIN PODIATR MED SUR, V28, P117, DOI 10.1016/j.cpm.2010.10.002
   Vidal BC, 2005, BRAZ J MORPHOL SCI, V22, P49
   Vidal BD, 2010, ACTA HISTOCHEM, V112, P53, DOI 10.1016/j.acthis.2008.07.005
   VOGEL KG, 1989, INT REV CYTOL, V115, P267
   Wren TAL, 2001, CLIN BIOMECH, V16, P245, DOI 10.1016/S0268-0033(00)00089-9
   Yamaguchi M, 2010, EUR J ORTHODONT, V32, P131, DOI 10.1093/ejo/cjp078
NR 41
TC 25
Z9 24
U1 1
U2 11
PU SPRINGER LONDON LTD
PI LONDON
PA 236 GRAYS INN RD, 6TH FLOOR, LONDON WC1X 8HL, ENGLAND
SN 0268-8921
EI 1435-604X
J9 LASER MED SCI
JI Lasers Med. Sci.
PD MAR
PY 2014
VL 29
IS 2
SI SI
BP 805
EP 811
DI 10.1007/s10103-013-1406-2
PG 7
WC Engineering, Biomedical; Surgery
WE Science Citation Index Expanded (SCI-EXPANDED)
SC Engineering; Surgery
GA AD2GH
UT WOS:000333051700052
PM 23982719
DA 2022-07-22
ER

PT J
AU Pierre-Jerome, C
   Moncayo, V
   Terk, MR
AF Pierre-Jerome, Claude
   Moncayo, Valeria
   Terk, Michael R.
TI MRI of the Achilles tendon: A comprehensive review of the anatomy,
   biomechanics, and imaging of overuse tendinopathies
SO ACTA RADIOLOGICA
LA English
DT Article
ID CLINICAL-RELEVANCE; CALCANEAL TENDON; RUPTURE; DISORDERS; INJURIES;
   ABNORMALITIES; TENDINITIS; ANKLE; MANAGEMENT; WIIITIS
AB The Achilles tendon is the largest tendon in the body; it plays an important role in the biomechanics of the lower extremity. It can withstand great forces, especially during sporting exercises and pivoting. The pathologies related to the Achilles tendon are diverse and many carry undesirable consequences. We retrospectively analyzed the images of patients who underwent examinations of the ankle/foot region to review the anatomy of the Achilles tendon and its surroundings and to search for pathologies consistent with overuse injuries. The anatomy of the tendon is described from origin to insertion. The imaging characteristics of the Achilles tendon including pitfalls are reviewed. We also describe the Achilles overuse injuries: paratenonitis, tendinosis, tendon tear, atypical tear, tendon re-tear, retrocalcaneal bursitis, retro-Achilles bursitis, Haglund's deformity, and tendon calcification. We present other entities like tendon ossification and failed transplanted Achilles tendon, with emphasis on MRI.</.
C1 [Pierre-Jerome, Claude; Moncayo, Valeria; Terk, Michael R.] Emory Univ, Dept Radiol, Orthopaed & Spine Ctr, Atlanta, GA 30329 USA.
   [Pierre-Jerome, Claude] Sahlgrenska Molndal Univ Hosp, Gothenburg, Sweden.
RP Pierre-Jerome, C (通讯作者)，Emory Univ, Dept Radiol, Orthopaed & Spine Ctr, 59 Execut Pk S, Atlanta, GA 30329 USA.
EM cpierr3@emory.edu
CR Alfredson H, 1998, AM J SPORT MED, V26, P166, DOI 10.1177/03635465980260020301
   ALLENMARK C, 1992, CLIN SPORT MED, V11, P759
   Arndt A, 1999, FOOT ANKLE INT, V20, P450, DOI 10.1177/107110079902000710
   ASTROM M, 1994, J ORTHOP RES, V12, P246, DOI 10.1002/jor.1100120214
   ASTROM M, 1995, CLIN ORTHOP RELAT R, P151
   Beddy P, 2009, AM J ROENTGENOL, V192, pW79, DOI 10.2214/AJR.08.1654
   Bonis J, 2007, NEW ENGL J MED, V356, P2431, DOI 10.1056/NEJMc070670
   Bottger BA, 1998, AM J ROENTGENOL, V170, P1239, DOI 10.2214/ajr.170.5.9574592
   Brewer BJ, 1960, AM ACAD ORTHOP SURG, V17, P354
   BUCHBINDER MR, 1979, J AM POD ASSOC, V69, P159, DOI 10.7547/87507315-69-2-159
   CARDEN DG, 1987, J BONE JOINT SURG BR, V69, P416, DOI 10.1302/0301-620X.69B3.3294839
   CARR AJ, 1989, J BONE JOINT SURG BR, V71, P100, DOI 10.1302/0301-620X.71B1.2914976
   CHEUNG Y, 1992, RADIOGRAPHICS, V12, P429, DOI 10.1148/radiographics.12.3.1609136
   Clain M R, 1992, Foot Ankle, V13, P482
   CLEMENT DB, 1984, AM J SPORT MED, V12, P179, DOI 10.1177/036354658401200301
   CUMMINS EJ, 1946, SURG GYNECOL OBSTET, V83, P107
   ERICKSON SJ, 1993, RADIOLOGY, V188, P23, DOI 10.1148/radiology.188.1.7685531
   FORNAGE BD, 1986, RADIOLOGY, V159, P759, DOI 10.1148/radiology.159.3.3517959
   FOX JM, 1997, CLIN PODIATR MED SUR, V107, P221
   GABEL S, 1994, FOOT ANKLE INT, V15, P512, DOI 10.1177/107110079401500912
   Harris C A, 2006, Australas Radiol, V50, P513, DOI 10.1111/j.1440-1673.2006.01622.x
   HESS GP, 1989, SPORTS MED, V8, P371, DOI 10.2165/00007256-198908060-00005
   Houshian S, 1998, INJURY, V29, P651, DOI 10.1016/S0020-1383(98)00147-8
   James S L, 1978, Am J Sports Med, V6, P40, DOI 10.1177/036354657800600202
   JOZSA L, 1989, AM J SPORT MED, V17, P338, DOI 10.1177/036354658901700305
   JOZSA L, 1984, ARCH ORTHOP TRAUM SU, V103, P47, DOI 10.1007/BF00451318
   KANNUS P, 1991, J BONE JOINT SURG AM, V73A, P1507, DOI 10.2106/00004623-199173100-00009
   Koivunen-Niemela T, 1995, SURG RADIOL ANAT, V17, P263, DOI 10.1007/BF01795061
   KOMI PV, 1992, CLIN SPORT MED, V11, P521
   KUJALA UM, 1992, INJURY, V23, P131, DOI 10.1016/0020-1383(92)90050-3
   KVIST M, 1980, MED SPORT, V20, P375
   LEACH RE, 1981, AM J SPORT MED, V9, P93, DOI 10.1177/036354658100900204
   Leppilahti J, 1998, FOOT ANKLE INT, V19, P683, DOI 10.1177/107110079801901006
   Leppilahti J, 1996, ACTA ORTHOP SCAND, V67, P277, DOI 10.3109/17453679608994688
   Ly JQ, 2004, AM J ROENTGENOL, V182, P147, DOI 10.2214/ajr.182.1.1820147
   Maffulli N, 2000, AM J SPORT MED, V28, P499, DOI 10.1177/03635465000280040901
   Mahler F, 1992, Br J Sports Med, V26, P7
   Mann RA, 1993, SURG FOOT ANKLE
   Mantel D, 1996, J RADIOL, V77, P261
   McGarvey WC, 1996, FOOT ANKLE INT, V17, P496, DOI 10.1177/107110079601700811
   Millar A P, 1979, Am J Sports Med, V7, P172, DOI 10.1177/036354657900700306
   Miller W A, 1977, Am J Sports Med, V5, P191, DOI 10.1177/036354657700500505
   Moller A, 1996, ACTA ORTHOP SCAND, V67, P479, DOI 10.3109/17453679608996672
   Movin T, 1997, ACTA ORTHOP SCAND, V68, P170, DOI 10.3109/17453679709004002
   Myerson M S, 1999, Instr Course Lect, V48, P211
   Narvaez JA, 2000, RADIOGRAPHICS, V20, P333, DOI 10.1148/radiographics.20.2.g00mc09333
   Ohashi K, 2004, AM J ROENTGENOL, V182, P161, DOI 10.2214/ajr.182.1.1820161
   Olivieri I, 2001, ANN RHEUM DIS, V60, P1160, DOI 10.1136/ard.60.12.1160
   OSTLERE S, 2003, IMAGING, V15, P242
   Paavola M, 1998, ACTA RADIOL, V39, P612, DOI 10.3109/02841859809175485
   Paavola M, 2002, J BONE JOINT SURG AM, V84A, P2062, DOI 10.2106/00004623-200211000-00024
   PALASTANGA N, 1989, ANATOMY HUMAN MOVEME, P360
   PERRY J, 1983, CLIN ORTHOP RELAT R, V177, P9
   Read JW, 2000, SPORTS MED ARTHROSC, V8, P32, DOI 10.1097/00132585-200008010-00004
   RESNICK D, 1977, RADIOLOGY, V125, P355, DOI 10.1148/125.2.355
   RESNIK CS, 1990, J CAN ASSOC RADIOL, V41, P153
   Robinson RJ, 2008, EMERG RADIOL, V15, P255, DOI 10.1007/s10140-008-0712-7
   Saltzman C L, 1998, J Am Acad Orthop Surg, V6, P316
   Sandelin J, 1985, Br J Sports Med, V19, P103
   SARTORIS D J, 1989, Journal of Foot Surgery, V28, P370
   SCHEPSIS AA, 1987, AM J SPORT MED, V15, P308, DOI 10.1177/036354658701500403
   Schepsis AA, 2002, AM J SPORT MED, V30, P287, DOI 10.1177/03635465020300022501
   Schweitzer ME, 2000, AM J ROENTGENOL, V175, P613, DOI 10.2214/ajr.175.3.1750613
   Sella E J, 1998, J Foot Ankle Surg, V37, P110
   SNOW SW, 1995, FOOT ANKLE INT, V16, P418, DOI 10.1177/107110079501600707
   STAUBER WT, 1989, EXERCISE SPORT SCI R, V17, P157
   STROCCHI R, 1991, FOOT ANKLE, V12, P100, DOI 10.1177/107110079101200207
   SUN YS, 1977, ZHONGHUA YIXUE ZAZHI, V57, P94
   SUTHERLAND DH, 1966, J BONE JOINT SURG AM, VA 48, P66, DOI 10.2106/00004623-196648010-00005
   Taunton JE, 2003, BRIT J SPORT MED, V37, P239, DOI 10.1136/bjsm.37.3.239
   Teitz C C, 1997, Instr Course Lect, V46, P569
   Turlik M A, 1990, Clin Podiatr Med Surg, V7, P369
   WHITTAKER P, 1991, MATRIX, V11, P56, DOI 10.1016/S0934-8832(11)80227-1
   Zanetti M, 2003, RADIOLOGY, V227, P556, DOI 10.1148/radiol.2272012069
NR 74
TC 68
Z9 69
U1 3
U2 33
PU SAGE PUBLICATIONS LTD
PI LONDON
PA 1 OLIVERS YARD, 55 CITY ROAD, LONDON EC1Y 1SP, ENGLAND
SN 0284-1851
EI 1600-0455
J9 ACTA RADIOL
JI Acta Radiol.
PD MAY
PY 2010
VL 51
IS 4
BP 438
EP 454
DI 10.3109/02841851003627809
PG 17
WC Radiology, Nuclear Medicine & Medical Imaging
WE Science Citation Index Expanded (SCI-EXPANDED)
SC Radiology, Nuclear Medicine & Medical Imaging
GA 581JP
UT WOS:000276519000014
PM 20380605
DA 2022-07-22
ER

PT J
AU Dinato, MCME
   Freitas, MD
   D'Elia, CO
   Bitar, AC
   Goncalves, FMR
AF Mattos e Dinato, Mauro Cesar
   Freitas, Marcio de Farias
   D'Elia, Caio Oliveira
   Bitar, Alexandre Carneiro
   Rodrigues Goncalves, Fabio Minutti
TI Acute Calcaneus Tendon Rupture Associated with Ipsilateral Malleolar
   Fracture: Case Report and Literature Review
SO JOURNAL OF FOOT & ANKLE SURGERY
LA English
DT Article
DE Achilles tendon; ankle; fracture; surgery; tibia; ultrasound
ID ACHILLES-TENDON
AB Malleolar fractures are rarely associated with lesions of the adjacent tendons or neurovascular structures. The association of ankle fractures with Achilles tendon rupture is even more infrequent, although both of these injuries are very common in and of themselves. To our knowledge, fracture of the lateral malleolus in association with an acute rupture of the ipsilateral calcaneus tendon has not been previously described. In this article, we describe a female patient who sustained an acute rupture of the Achilles tendon in conjunction with fracture of the ipsilateral lateral malleolus. (C) 2010 by the American College of Foot and Ankle Surgeons. All rights reserved.
C1 [Mattos e Dinato, Mauro Cesar; Freitas, Marcio de Farias; D'Elia, Caio Oliveira; Bitar, Alexandre Carneiro; Rodrigues Goncalves, Fabio Minutti] Vita Care, Sao Paulo, Brazil.
RP Dinato, MCME (通讯作者)，Vita Care, Rua Mato Grosso 306,1 Andar Higienopolis, Sao Paulo, Brazil.
EM mauro@vita.org.br
CR Assal M, 2002, J ORTHOP TRAUMA, V16, P358, DOI 10.1097/00005131-200205000-00013
   BARRON JL, 1993, AM J SPORT MED, V21, P629, DOI 10.1177/036354659302100426
   CETTI R, 1993, CLIN ORTHOP RELAT R, P215
   Chiodo CP, 2006, FOOT ANKLE INT, V27, P305, DOI 10.1177/107110070602700415
   Komarasamy Baskaran, 2007, J Foot Ankle Surg, V46, P188, DOI 10.1053/j.jfas.2006.12.006
   Lubin J W, 2000, Am J Orthop (Belle Mead NJ), V29, P707
   Maffulli N, 2006, BMC MUSCULOSKEL DIS, V7, DOI 10.1186/1471-2474-7-59
   MARTIN JW, 1986, CLIN ORTHOP RELAT R, P216
   Pieper HG, 1998, INT J SPORTS MED, V19, P68, DOI 10.1055/s-2007-971883
   Sugimoto K, 2003, J ORTHOP TRAUMA, V17, P534, DOI 10.1097/00005131-200308000-00012
NR 10
TC 2
Z9 2
U1 0
U2 1
PU ELSEVIER SCIENCE INC
PI NEW YORK
PA STE 800, 230 PARK AVE, NEW YORK, NY 10169 USA
SN 1067-2516
EI 1542-2224
J9 J FOOT ANKLE SURG
JI J. Foot Ankle Surg.
PD NOV-DEC
PY 2010
VL 49
IS 6
DI 10.1053/j.jfas.2010.06.014
PG 4
WC Orthopedics; Surgery
WE Science Citation Index Expanded (SCI-EXPANDED)
SC Orthopedics; Surgery
GA 672LS
UT WOS:000283585400001
PM 20797880
DA 2022-07-22
ER

PT J
AU Humbyrd, CJ
   Bae, S
   Kucirka, LM
   Segev, DL
AF Humbyrd, Casey Jo
   Bae, Sunjae
   Kucirka, Lauren M.
   Segev, Dorry L.
TI Incidence, Risk Factors, and Treatment of Achilles Tendon Rupture in
   Patients With End-Stage Renal Disease
SO FOOT & ANKLE INTERNATIONAL
LA English
DT Article
DE Achilles tendon rupture; dialysis; end-stage renal disease; kidney
   transplant
ID TRANSPLANT RECIPIENT; INCREASING INCIDENCE; UNITED-STATES; EPIDEMIOLOGY;
   POPULATION; DIALYSIS; SURGERY; INJURY
AB Background: Dialysis-dependent patients and kidney transplant recipients may be at increased risk for Achilles tendon rupture (ATR).
   Methods: We studied Medicare patients with end-stage renal disease (ESRD) from 1999 through 2013. Patients were categorized as waitlisted for a transplant, not waitlisted, or received a transplant. We performed multivariate negative binomial regression using demographic characteristics, comorbidities, and year of study entry to estimate adjusted incidence rate ratios (aIRRs), identify ATR risk factors, and determine treatment patterns and outcomes.
   Results: We identified 1091 ATRs (incidence, 3.80/10 000 person-years; 95% confidence interval [CI], 3.58-4.03). Compared with transplant recipients, nonwaitlisted patients had a lower incidence (aIRR, 0.44; 95% CI, 0.37-0.53), and waitlisted patients had a similar incidence (aIRR, 0.94; 95% CI, 0.78-1.12) of ATR. ATR incidence was higher among patients taking fluoroquinolones (aIRR, 1.65; 95% CI, 1.32-1.84) and corticosteroids (aIRR, 1.72; 95% CI, 1.44-2.05) compared with those who did not. Patients with ATR were younger, had higher mean body mass index, and had fewer comorbidities than patients without ATR. Seventeen percent of patients received operative treatment within 14 days of ATR diagnosis. The 30-day cumulative incidence of operative site infections was 6.5%.
   Conclusion: The incidence of ATR was higher among transplant recipients and waitlisted patients compared with nonwaitlisted patients. Younger age, higher body mass index, fewer comorbidities, fluoroquinolone use, and corticosteroid use were risk factors for ATR. Patients were more likely to receive nonoperative than operative treatment for ATR. Those who underwent operative treatment had a low incidence of operative site infection.
   Level of Evidence: Prognostic level III, comparative study.
C1 [Humbyrd, Casey Jo] Johns Hopkins Sch Med, Dept Orthopaed Surg, Baltimore, MD USA.
   [Bae, Sunjae; Kucirka, Lauren M.; Segev, Dorry L.] Johns Hopkins Sch Med, Dept Surg, Baltimore, MD USA.
   [Bae, Sunjae; Segev, Dorry L.] Johns Hopkins Sch Publ Hlth, Dept Epidemiol, Baltimore, MD USA.
RP Humbyrd, CJ (通讯作者)，Johns Hopkins Univ, Johns Hopkins Bayview Med Ctr, Dept Orthopaed Surg, 4940 Eastern Ave,Suite A660B, Baltimore, MD 21224 USA.
EM casey.humbyrd@jhu.edu
RI Humbyrd, Casey/ABF-2192-2021; Humbyrd, Casey/L-3287-2019; Bae,
   Sunjae/D-4205-2018
OI Humbyrd, Casey/0000-0001-9623-4212; Bae, Sunjae/0000-0003-0098-8816
FU National Institute of Diabetes and Digestive and Kidney Diseases
   [K24DK101828, F30DK095545]; NATIONAL INSTITUTE OF DIABETES AND DIGESTIVE
   AND KIDNEY DISEASES [F30DK095545, K24DK101828] Funding Source: NIH
   RePORTER
FX The author(s) disclosed receipt of the following financial support for
   the research, authorship, and/or publication of this article: This work
   was supported by the National Institute of Diabetes and Digestive and
   Kidney Diseases (grants K24DK101828 to DLS and F30DK095545). The data
   reported here were supplied by the US Renal Data System. The
   interpretation and reporting of these data are the responsibility of the
   author(s) and in no way should be seen as an official policy or
   interpretation of the US government.
CR American Medical Association, 2016, CURR PROC TERM CPT 2
   Aucella F, 2014, KIDNEY BLOOD PRESS R, V39, P97, DOI 10.1159/000355783
   Basic-Jukic N, 2009, KIDNEY BLOOD PRESS R, V32, P32, DOI 10.1159/000201792
   BRADLOW A, 1984, BRIT MED J, V288, P364, DOI 10.1136/bmj.288.6414.364-a
   Claessen FMAP, 2014, SPORTS MED, V44, P1241, DOI 10.1007/s40279-014-0200-z
   Davis JJ, 1999, MIL MED, V164, P872, DOI 10.1093/milmed/164.12.872
   Dombrowski ME, 2016, AOFAS ANN M JUL 20 2
   Erickson BJ, 2014, ORTHOP J SPORTS MED, V2, DOI 10.1177/2325967114549948
   Grams ME, 2013, AM J KIDNEY DIS, V62, P245, DOI 10.1053/j.ajkd.2013.03.009
   Hart A, 2017, AM J TRANSPLANT, V17, DOI 10.1111/ajt.14124
   HESTIN D, 1993, NEPHRON, V65, P491, DOI 10.1159/000187546
   Houshian S, 1998, INJURY, V29, P651, DOI 10.1016/S0020-1383(98)00147-8
   Huttunen TT, 2014, AM J SPORT MED, V42, P2419, DOI 10.1177/0363546514540599
   Jarvinen Tero A H, 2005, Foot Ankle Clin, V10, P255, DOI 10.1016/j.fcl.2005.01.013
   Jones N, 1996, AM J KIDNEY DIS, V28, P861, DOI 10.1016/S0272-6386(96)90386-8
   JOZSA L, 1989, AM J SPORT MED, V17, P338, DOI 10.1177/036354658901700305
   Kato A, 2011, CLIN EXP NEPHROL, V15, P318, DOI 10.1007/s10157-011-0417-1
   Lantto I, 2015, SCAND J MED SCI SPOR, V25, pE133, DOI 10.1111/sms.12253
   Leppilahti J, 1996, ACTA ORTHOP SCAND, V67, P277, DOI 10.3109/17453679608994688
   Levi N, 1997, INJURY, V28, P311, DOI 10.1016/S0020-1383(96)00200-8
   Lui PPY, 2017, SCAND J MED SCI SPOR, V27, P776, DOI 10.1111/sms.12824
   Maffulli N, 1999, CLIN J SPORT MED, V9, P157, DOI 10.1097/00042752-199907000-00007
   Mattila VM, 2015, BRIT J SPORT MED, V49, P1084, DOI 10.1136/bjsports-2013-092756
   Moller A, 1996, ACTA ORTHOP SCAND, V67, P479, DOI 10.3109/17453679608996672
   National Center for Health Statistics Centers for Medicare and Medicaid Services, 2010, ICD 9 CM INT CLASS D
   National Kidney Foundation, KIDN TRANSPL WAITL W
   Partridge L, 2017, CURR DIABETES REV, V13, P424, DOI 10.2174/1573399812666160620100616
   Raikin SM, 2013, FOOT ANKLE INT, V34, P475, DOI 10.1177/1071100713477621
   Saran R, 2017, AM J KIDNEY DIS, V69, DOI 10.1053/j.ajkd.2016.12.004
   Sode J, 2007, EUR J CLIN PHARMACOL, V63, P499, DOI 10.1007/s00228-007-0265-9
   Soroceanu A, 2012, J BONE JOINT SURG AM, V94A, P2136, DOI 10.2106/JBJS.K.00917
   SPENCER JD, 1988, INJURY, V19, P86, DOI 10.1016/0020-1383(88)90079-4
   Suchak AA, 2005, FOOT ANKLE INT, V26, P932, DOI 10.1177/107110070502601106
   White DW, 2007, AM J SPORT MED, V35, P1308, DOI 10.1177/0363546507301256
   YASUI Y, 2017, BIOMED RES INT, V2017, DOI DOI 10.1155/2017/7021862
NR 35
TC 5
Z9 5
U1 0
U2 2
PU SAGE PUBLICATIONS INC
PI THOUSAND OAKS
PA 2455 TELLER RD, THOUSAND OAKS, CA 91320 USA
SN 1071-1007
EI 1944-7876
J9 FOOT ANKLE INT
JI Foot Ankle Int.
PD JUL
PY 2018
VL 39
IS 7
BP 821
EP 828
DI 10.1177/1071100718762089
PG 8
WC Orthopedics
WE Science Citation Index Expanded (SCI-EXPANDED)
SC Orthopedics
GA GM9LT
UT WOS:000438570700009
PM 29582683
OA Green Accepted
DA 2022-07-22
ER

PT J
AU Lesic, A
   Bumbasirevic, M
AF Lesic, A
   Bumbasirevic, M
TI Disorders of the Achilles tendon
SO CURRENT ORTHOPAEDICS
LA English
DT Article
DE Achilles tendon; disorders; treatment
ID SURGICAL-TREATMENT; NONSURGICAL TREATMENT; CALCANEAL TENDON; RUPTURES;
   PATHOLOGY; REPAIR; ULTRASONOGRAPHY; DIAGNOSIS; HEEL
AB The Achilles tendon is susceptible to various types of acute and chronic injuries as a consequence of its large size, enormous continuous functional demands during gait, and poor blood supply. Also, the occurrence of overuse syndromes of the Achilles tendon is increasing due to the modern physical life style. In this article the different forms of tendinitis (insertional and non-insertional), Haglund's deformity and bursitis are discussed. Also, acute and chronic Achilles tendon ruptures are considered, paying particular attention to non-operative vs operative treatment, where opinion is stilt controversial. There are many reports about the disadvantages and complications of operative treatment of closed Achilles tendon rupture, but nowadays a more aggressive approach to this entity is common. (C) 2004 Elsevier Ltd. All rights reserved.
C1 Inst Orthpaed Surg & Traumatol, YU-11000 Belgrade, Serbia.
RP Lesic, A (通讯作者)，Inst Orthpaed Surg & Traumatol, Visegradska 26, YU-11000 Belgrade, Serbia.
CR ABRAHAM E, 1975, J BONE JOINT SURG AM, VA 57, P253, DOI 10.2106/00004623-197557020-00019
   Alfredson H, 2000, SPORTS MED, V29, P135, DOI 10.2165/00007256-200029020-00005
   ARMAGAN O, 2000, FOOT ANKLE DISORDERS, P942
   ARNER O, 1959, Acta Chir Scand, V116, P484
   ARNER O, 1959, ACTA CHIR SCAND    S, V239, P1
   Astrom M, 1996, SKELETAL RADIOL, V25, P615, DOI 10.1007/s002560050146
   ASTROM M, 1995, CLIN ORTHOP RELAT R, V361, P151
   BARFRED T, 1973, ACTA ORTHOP SCAND, P1
   BAXTER DE, 1999, CLIN SPORTS MD, V13, P683
   BOOTH FW, 1987, CLIN ORTHOP RELAT R, P15
   BOYDEN EM, 1995, CLIN ORTHOP RELAT R, V317, P150
   BUGG EI, 1968, CLIN ORTHOP RELAT R, P73
   Bunnell S., 1940, AM J SURG, DOI [10.1016/S0002- 9610(40)90160- X, DOI 10.1016/S0002-9610(40)90160-X]
   CALHOUN JN, 2002, FOOT ANKLE, P311
   CARR AJ, 1989, J BONE JOINT SURG BR, V71, P100, DOI 10.1302/0301-620X.71B1.2914976
   CETTI R, 1994, CLIN ORTHOP RELAT R, P155
   CHAUVEAUX D, 1991, SURG RADIOL ANAT, V13, P39, DOI 10.1007/BF01623140
   Clain M R, 1992, Foot Ankle, V13, P482
   Clancy W G Jr, 1976, Am J Sports Med, V4, P46, DOI 10.1177/036354657600400202
   Clement D B, 1981, Phys Sportsmed, V9, P47, DOI 10.1080/00913847.1981.11711077
   DEMAIO M, 1995, ORTHOPEDICS, V18, P195
   FIAMENGO SA, 1982, CLIN ORTHOPAEDICS, V167, P203
   FITZGIBBONS RE, 1993, AM J SPORT MED, V21, P724, DOI 10.1177/036354659302100516
   Fowler A, 1945, BRIT J SURG, V32, P494, DOI 10.1002/bjs.18003212812
   FOX JM, 1975, CLIN ORTHOP RELAT R, P221
   GILLIES H, 1970, J BONE JOINT SURG AM, VA 52, P337, DOI 10.2106/00004623-197052020-00015
   Haglund P, 1928, ASCHR ORTHOP CHIR, V49, P49
   HANSEN ST, 2000, HEEL CORD INJURIES F, P39
   HELAL B, 1989, CLIN SPORTS MED, V1, P17
   HELAL B, 1993, FOOT, V3, P13
   HENEGHAN MA, 1984, CLIN ORTHOP RELAT R, V187, P228
   HOWARD CB, 1984, J BONE JOINT SURG BR, V66, P206, DOI 10.1302/0301-620X.66B2.6707056
   INGLIS AE, 1976, J BONE JOINT SURG AM, V58, P990, DOI 10.2106/00004623-197658070-00015
   INGLIS AE, 1981, CLIN ORTHOP RELAT R, V156, P160
   Jacobs D, 1978, Am J Sports Med, V6, P107, DOI 10.1177/036354657800600302
   Karjalainen PT, 1996, ACTA RADIOL, V37, P639
   KECK SW, 1965, J BONE JOINT SURG AM, VA 47, P267, DOI 10.2106/00004623-196547020-00003
   KELIKIAN H, 1985, DISORDERS ANKLE, P792
   Kessler I, 1973, Hand, V5, P253, DOI 10.1016/0072-968X(73)90038-7
   KRUEGERFRANKE M, 1995, BRIT J SPORT MED, V29, P121, DOI 10.1136/bjsm.29.2.121
   KVIST H, 1980, J BONE JOINT SURG BR, V62, P353, DOI 10.1302/0301-620X.62B3.7410468
   KVIST M, 1992, INT ORTHOP, V16, P377
   LAGERGREN C, 1959, Acta Chir Scand, V116, P491
   LASETER JT, 1991, MED SCI SPORT EXER, V23, P1
   LEA RB, 1972, J BONE JOINT SURG AM, VA 54, P1398, DOI 10.2106/00004623-197254070-00002
   LEACH RE, 1981, AM J SPORT MED, V9, P93, DOI 10.1177/036354658100900204
   LEHTO MU, 1994, KNEE SURG SPORTS ART, V1, P182
   LEMM M, 1992, J AM PODIAT MED ASSN, V82, P482, DOI 10.7547/87507315-82-9-482
   Leppilahti J, 1996, CLIN ORTHOP RELAT R, P171
   Leppilahti J, 1996, ACTA ORTHOP SCAND, V67, P277, DOI 10.3109/17453679608994688
   LIPSCOMB P R, 1956, Minn Med, V39, P731
   LUNQUIST R, 1968, ACTA ORTHO SCAND S, V113, P1
   LYNN TA, 1966, J BONE JOINT SURG AM, VA 48, P268, DOI 10.2106/00004623-196648020-00005
   MA GWC, 1977, CLIN ORTHOP RELAT R, P247
   Maffulli N, 2000, AM J SPORT MED, V28, P499, DOI 10.1177/03635465000280040901
   Maffulli N, 1999, CLIN J SPORT MED, V9, P157, DOI 10.1097/00042752-199907000-00007
   MAYERSON MS, 2000, FOOT ANKLE DISORDERS, P1367
   McCrory JL, 1999, MED SCI SPORT EXER, V31, P1374, DOI 10.1097/00005768-199910000-00003
   McGarvey WC, 1996, FOOT ANKLE INT, V17, P496, DOI 10.1177/107110079601700811
   Moller M, 2001, J BONE JOINT SURG BR, V83B, P843
   Movin T, 1997, FOOT ANKLE INT, V18, P297, DOI 10.1177/107110079701800510
   NELEN G, 1989, AM J SPORT MED, V17, P754, DOI 10.1177/036354658901700605
   NESSE E, 1994, ACTA ORTHOP SCAND, V65, P107, DOI 10.3109/17453679408993732
   NISTOR L, 1976, ACTA ORTHOP SCAND, V47, P459, DOI 10.3109/17453677608988720
   NISTOR L, 1981, J BONE JOINT SURG AM, V63, P394, DOI 10.2106/00004623-198163030-00012
   NOYES ER, 1982, J BONE JOINT SURG AM, V57, P876
   OREILLY MAR, 1993, CLIN RADIOL, V48, P202, DOI 10.1016/S0009-9260(05)80284-3
   PRIESKORN D, 1996, SURG DISORDERS FOOT, P369
   PUDDU G, 1980, AM J SPORT MED, V8, P402, DOI 10.1177/036354658000800603
   Qenu J S., 1929, REV CHIRURG, V67, P647
   QUIGLEY TB, 1980, AM J SPORT MED, V8, P244, DOI 10.1177/036354658000800406
   RAED MT, 1999, BRIT J SPORT MED, V33, P134
   RUCH J, 1974, AM POD ASS, V64, P1000
   SALTER RB, 1980, J BONE JOINT SURG AM, V62, P1232, DOI 10.2106/00004623-198062080-00002
   Schepsis AA, 2002, AM J SPORT MED, V30, P287, DOI 10.1177/03635465020300022501
   SCHMIDTROHLFING B, 1992, INT ORTHOP, V16, P29
   SCIOLI MW, 1994, ORTHOP CLIN N AM, V25, P177
   SCOTT WN, 1979, CLIN ORTHOP RELAT R, P175
   SNYDER M, 2003, EUR INSTR COURS LECT, V6, P62
   STEPHENS MM, 1994, ORTHOP CLIN N AM, V25, P41
   STUCKE K, 1950, ARCH KLIN CHIR, V26, P579
   THOMPSON T C, 1962, J Trauma, V2, P126, DOI 10.1097/00005373-196203000-00003
   Wapner K L, 1993, Foot Ankle, V14, P443
   Wapner KL, 1999, OPERATIVE TREATMENT OF THE FOOT AND ANKLE, P369
   WEINSTABL R, 1991, J BONE JOINT SURG BR, V73, P683, DOI 10.1302/0301-620X.73B4.2071660
   White JW, 1943, ARCH SURG-CHICAGO, V46, P784, DOI 10.1001/archsurg.1943.01220110200033
   Zadek I, 1939, AM J SURG, V43, P542
NR 87
TC 15
Z9 16
U1 2
U2 10
PU CHURCHILL LIVINGSTONE
PI EDINBURGH
PA JOURNAL PRODUCTION DEPT, ROBERT STEVENSON HOUSE, 1-3 BAXTERS PLACE,
   LEITH WALK, EDINBURGH EH1 3AF, MIDLOTHIAN, SCOTLAND
SN 0268-0890
J9 CURR ORTHOPAED
JI Curr. Orthop.
PD FEB
PY 2004
VL 18
IS 1
BP 63
EP 75
DI 10.1016/j.cuor.2003.10.002
PG 13
WC Orthopedics
WE Science Citation Index Expanded (SCI-EXPANDED)
SC Orthopedics
GA 819IL
UT WOS:000221308500007
DA 2022-07-22
ER

PT J
AU Luthje, P
   Nurmi, I
   Nyyssonen, T
AF Luthje, P
   Nurmi, I
   Nyyssonen, T
TI Missed Achilles tendon rupture due to oral levofloxacin: surgical
   treatment and result
SO ARCHIVES OF ORTHOPAEDIC AND TRAUMA SURGERY
LA English
DT Article
DE fluoroquinolones; Achilles tendon rupture
ID CALCANEAL TENDON; FLUOROQUINOLONES; TENDINOPATHY; CIPROFLOXACIN;
   PEFLOXACIN; REPAIR; NORFLOXACIN; TOXICITY; RISK
AB We describe a case of a 60-year-old male patient who was treated with oral levofloxacin. The patient sustained a total subcutaneous rupture of the left Achilles tendon which was not diagnosed for at least 5 months. Surgical treatment was successful.
C1 Kuusankoski Reg Hosp, Dept Orthopaed & Traumatol, Kuusankoski 45750, Finland.
   Univ Helsinki, Dept Gen Practice & Primary Hlth Care, FIN-00014 Helsinki, Finland.
   Kuopio Univ Hosp, Dept Orthopaed & Traumatol, Kuopio 70211, Finland.
RP Luthje, P (通讯作者)，Sairaalankuja 2, Kuusankoski 45750, Finland.
EM peter.luthje@pp.inet.fi
CR BAILEY RR, 1983, NEW ZEAL MED J, V96, P590
   Beuchard J, 1996, PRESSE MED, V25, P1083
   CARDEN DG, 1987, J BONE JOINT SURG BR, V69, P416, DOI 10.1302/0301-620X.69B3.3294839
   Casparian JM, 2000, SOUTHERN MED J, V93, P488, DOI 10.1097/00007611-200005000-00008
   INGLIS AE, 1976, J BONE JOINT SURG AM, V58, P990, DOI 10.2106/00004623-197658070-00015
   Jagose JT, 1996, NEW ZEAL MED J, V109, P471
   JORGENSEN C, 1991, REV RHUM, V58, P623
   Kahn MF, 1997, REV RHUM, V64, P437
   Kashida Y, 1997, ANTIMICROB AGENTS CH, V41, P2389, DOI 10.1128/AAC.41.11.2389
   Khaliq Y, 2003, CLIN INFECT DIS, V36, P1404, DOI 10.1086/375078
   Lafon M, 1993, CONCOURS MED, V115, P819
   LEHUEC JC, 1995, J BONE JOINT SURG BR, V77B, P293, DOI 10.1302/0301-620X.77B2.7706350
   LEPPILAHTI J, 1998, CLIN ORTHOP RELAT R, V346, P152
   LINDHOLM A, 1959, Acta Chir Scand, V117, P261
   MCEWAN SR, 1988, LANCET, V2, P900
   NADA A, 1985, J BONE JOINT SURG BR, V67, P449, DOI 10.1302/0301-620X.67B3.3997958
   Nyyssonen T, 2003, ACTA ORTHOP SCAND, V74, P206, DOI 10.1080/00016470310013978
   Simonin MA, 2000, ANTIMICROB AGENTS CH, V44, P867, DOI 10.1128/AAC.44.4.867-872.2000
   Stahlmann R, 2003, DRUG AGING, V20, P289, DOI 10.2165/00002512-200320040-00005
   Takao M, 2003, ARCH ORTHOP TRAUM SU, V123, P471, DOI 10.1007/s00402-002-0443-2
   THOMPSON T C, 1962, J Trauma, V2, P126, DOI 10.1097/00005373-196203000-00003
   van der Linden PD, 1999, BRIT J CLIN PHARMACO, V48, P433
   van der Linden PD, 2003, ARCH INTERN MED, V163, P1801, DOI 10.1001/archinte.163.15.1801
   van der Linden PD, 2002, BRIT MED J, V324, P1306, DOI 10.1136/bmj.324.7349.1306
   Wilton LV, 1996, BRIT J CLIN PHARMACO, V41, P277, DOI 10.1046/j.1365-2125.1996.03013.x
   Zabraniecki L, 1996, J RHEUMATOL, V23, P516
NR 26
TC 4
Z9 4
U1 0
U2 0
PU SPRINGER
PI NEW YORK
PA 233 SPRING ST, NEW YORK, NY 10013 USA
SN 0936-8051
EI 1434-3916
J9 ARCH ORTHOP TRAUM SU
JI Arch. Orthop. Trauma Surg.
PD MAR
PY 2005
VL 125
IS 2
BP 124
EP 126
DI 10.1007/s00402-004-0784-0
PG 3
WC Orthopedics; Surgery
WE Science Citation Index Expanded (SCI-EXPANDED)
SC Orthopedics; Surgery
GA 907WQ
UT WOS:000227749200008
PM 15645269
DA 2022-07-22
ER

PT J
AU Werd, MB
AF Werd, Matthew B.
TI Achilles tendon sports injuries - A review of classification and
   treatment
SO JOURNAL OF THE AMERICAN PODIATRIC MEDICAL ASSOCIATION
LA English
DT Article
ID NONOPERATIVE TREATMENT; OPEN REPAIR; TENDINOPATHY; RUPTURE; ANKLE;
   TENDINITIS; MANAGEMENT; SURGERY; FORCES
AB Achilles tendon injuries are among the three most frequent sports-related injuries of the foot and ankle. Proper function of the Achilles tendon is critical to performance in sports. A thorough knowledge of the anatomy and biomechanical function of this tendon is essential to the effective treatment of these injuries. Distinguishing among the various pathologies of the Achilles tendon is an important first step toward successful treatment and return of the athlete to sports activity. The term Achilles tendinitis is a nonspecific diagnosis that does not accurately describe an actual injury. This review is intended to provide the sports medicine physician with a means of classifying Achilles tendon injuries and, thus, arriving at an accurate diagnosis and treatment plan.
C1 Lakeland Reg Med Ctr, Lakeland, FL 33803 USA.
RP Werd, MB (通讯作者)，Lakeland Reg Med Ctr, 2939 S Florida Ave, Lakeland, FL 33803 USA.
CR Alfredson H, 1998, AM J SPORT MED, V26, P360, DOI 10.1177/03635465980260030301
   Alfredson H, 2000, SPORTS MED, V29, P135, DOI 10.2165/00007256-200029020-00005
   Aroen A, 2004, SCAND J MED SCI SPOR, V14, P30, DOI 10.1111/j.1600-0838.2004.00344.x
   Aspenberg P, 1999, ACTA ORTHOP SCAND, V70, P51, DOI 10.3109/17453679909000958
   Benazzo F, 2001, MED SCI SPORT EXER, V33, P696
   Bhandari M, 2002, CLIN ORTHOP RELAT R, P190
   BOUCHE R, 2005, MASTER TECHNIQUES PO, V1, P266
   BRUNET ME, 1990, J SPORT MED PHYS FIT, V30, P307
   BURDETT RG, 1982, MED SCI SPORT EXER, V14, P308, DOI 10.1249/00005768-198204000-00010
   CETTI R, 1993, AM J SPORT MED, V21, P791, DOI 10.1177/036354659302100606
   Clement D B, 1981, Phys Sportsmed, V9, P47, DOI 10.1080/00913847.1981.11711077
   CLEMENT DB, 1984, AM J SPORT MED, V12, P179, DOI 10.1177/036354658401200301
   Cohen Ronald S, 2003, Curr Sports Med Rep, V2, P251
   DEMAIO M, 1995, ORTHOPEDICS, V18, P195
   Forslund C, 2001, MED SCI SPORT EXER, V33, P685
   Gould N, 2002, FOOT ANKLE INT, V23, P595, DOI 10.1177/107110070202300702
   Hersh BL, 2002, J AM PODIAT MED ASSN, V92, P355, DOI 10.7547/87507315-92-6-355
   Hootman JM, 2002, MED SCI SPORT EXER, V34, P838, DOI 10.1097/00005768-200205000-00017
   Hunte Garth, 2005, Clin J Sport Med, V15, P116, DOI 10.1097/01.jsm.0000151866.75684.3d
   Kauranen K, 2002, FOOT ANKLE INT, V23, P600, DOI 10.1177/107110070202300703
   Khan RJ, 2004, COCHRANE DB SYST REV, V3, DOI DOI 10.1002/14651858.CD003674.PUB2
   KRISSOFF WB, 1979, PHYSICIAN SPORTSMED, V7, P55
   Kurtz CA, 1999, AM J SPORT MED, V27, P363, DOI 10.1177/03635465990270031701
   KVIST M, 1994, SPORTS MED, V18, P173, DOI 10.2165/00007256-199418030-00004
   Lim J, 2001, FOOT ANKLE INT, V22, P559, DOI 10.1177/107110070102200705
   Maffulli N, 2003, AM J SPORT MED, V31, P692, DOI 10.1177/03635465030310051001
   Maffulli N, 2004, AM J SPORT MED, V32, P174, DOI 10.1177/0363546503258923
   Maffulli N, 1999, AM J SPORT MED, V27, P747, DOI 10.1177/03635465990270061101
   Mafi N, 2001, KNEE SURG SPORT TR A, V9, P42, DOI 10.1007/s001670000148
   McCrory JL, 1999, MED SCI SPORT EXER, V31, P1374, DOI 10.1097/00005768-199910000-00003
   MORRIS K L, 1990, Journal of Foot Surgery, V29, P533
   MOSELY JB, 1995, FOOT ANKLE SPORTS
   NELEN G, 1989, AM J SPORT MED, V17, P754, DOI 10.1177/036354658901700605
   Puddu G, 1976, Am J Sports Med, V4, P145, DOI 10.1177/036354657600400404
   Reddy GK, 1998, LASER SURG MED, V22, P281, DOI 10.1002/(SICI)1096-9101(1998)22:5<281::AID-LSM4>3.0.CO;2-L
   SAMMARCO V, 2003, TECH FOOT ANKLE SURG, V2, P51
   Saxena A, 2003, FOOT ANKLE INT, V24, P712, DOI 10.1177/107110070302400911
   Saxena A, 2003, J AM PODIAT MED ASSN, V93, P312, DOI 10.7547/87507315-93-4-312
   Saxena A, 2001, J Foot Ankle Surg, V40, P132
   SAXENA A, 2000, FOOT ANKLE Q, V13, P58
   Schepsis AA, 2002, AM J SPORT MED, V30, P287, DOI 10.1177/03635465020300022501
   SCOTT SH, 1990, MED SCI SPORT EXER, V22, P357
   Shalabi A, 2004, AM J SPORT MED, V32, P1286, DOI 10.1177/0363546504263148
   Tallon C, 2001, AM J SPORT MED, V29, P315, DOI 10.1177/03635465010290031101
   Testa V, 2002, MED SCI SPORT EXER, V34, P573, DOI 10.1097/00005768-200204000-00002
   Thermann H, 2002, FOOT ANKLE INT, V23, P606, DOI 10.1177/107110070202300704
   Thermann H, 2001, FOOT ANKLE INT, V22, P478, DOI 10.1177/107110070102200604
   Vanek D, 2003, J AM PODIAT MED ASSN, V93, P333, DOI 10.7547/87507315-93-4-333
   Wallace RGH, 2004, J BONE JOINT SURG AM, V86A, P1198, DOI 10.2106/00004623-200406000-00011
   Weber M, 2003, AM J SPORT MED, V31, P685, DOI 10.1177/03635465030310050901
   Yinger Kent, 2002, Clin Podiatr Med Surg, V19, P231, DOI 10.1016/S0891-8422(02)00002-2
   Young RG, 1998, J ORTHOPAED RES, V16, P406, DOI 10.1002/jor.1100160403
NR 52
TC 7
Z9 7
U1 1
U2 13
PU AMER PODIATRIC MED ASSOC
PI BETHESDA
PA 9312 OLD GEORGETOWN ROAD, BETHESDA, MD 20814-1621 USA
SN 8750-7315
EI 1930-8264
J9 J AM PODIAT MED ASSN
JI J. Am. Podiatr. Med. Assoc.
PD JAN-FEB
PY 2007
VL 97
IS 1
BP 37
EP 48
DI 10.7547/0970037
PG 12
WC Orthopedics
WE Science Citation Index Expanded (SCI-EXPANDED)
SC Orthopedics
GA 130BO
UT WOS:000243774300005
PM 17218624
DA 2022-07-22
ER

PT J
AU Forlenza, EM
   Lavoie-Gagne, OZ
   Lu, YN
   Diaz, CC
   Chahla, J
   Forsythe, B
AF Forlenza, Enrico M.
   Lavoie-Gagne, Ophelie Z.
   Lu, Yining
   Diaz, Connor C.
   Chahla, Jorge
   Forsythe, Brian
TI Return to Play and Player Performance After Achilles Tendon Rupture in
   UEFA Professional Soccer Players: A Matched-Cohort Analysis of Players
   From 1999 to 2018
SO ORTHOPAEDIC JOURNAL OF SPORTS MEDICINE
LA English
DT Article
DE soccer; player performance; return to play; Achilles tendon rupture;
   Union of European Football Associations
ID LIGAMENT RECONSTRUCTION; EPIDEMIOLOGY; INJURIES; SURGERY; REPAIR
AB Background: Achilles tendon rupture (ATR) is a potentially career-ending injury in professional athletes. Limited information exists regarding return to play (RTP) in professional soccer players after this injury. Purpose: To determine the RTP rate and time in professional soccer players after ATR and to evaluate player performance relative to matched controls. Study Design: Cohort study; Level of evidence, 3. Methods: We evaluated 132 professional soccer players who suffered an ATR between 1999 and 2018. These athletes were matched 2:1 to uninjured controls by position, age, season of injury, seasons played, and height. We collected information on the date of injury, the date of RTP, and player performance metrics (minutes played, games played, goals scored, assists made, and points per game) from official team websites, public injury reports, and press releases. Changes in performance metrics for the 4 years after the season of injury were compared with metrics 1 season before injury. Univariate comparisons were performed using independent-sample, 2-group t tests and Wilcoxon rank-sum tests when normality of distributions was violated. Results: The mean age at ATR was 27.49 +/- 4.06 years, and the mean time to RTP was 5.07 +/- 2.61 months (18.19 +/- 10.96 games). The RTP rate was 71% for the season after injury and 78% for return at any timepoint. Overall, 9% of the injured players experienced a rerupture during the study period. Compared with controls, the injured players played significantly less (-6.77 vs -1.81 games [P < .001] and -560.17 vs -171.17 minutes [P < .05]) and recorded fewer goals (-1.06 vs -0.29 [P < .05]) and assists (-0.76 vs -0.02 [P < .05]) during the season of their Achilles rupture. With the exception of midfielders, there were no significant differences in play time or performance metrics between injured and uninjured players at any postinjury timepoint. Conclusion: Soccer players who suffered an ATR had a 78% RTP rate, with a mean RTP time of 5 months. Injured players played less and demonstrated inferior performance during the season of injury. With the exception of midfielders, players displayed no significant differences in play time or performance during any of the 4 postinjury seasons.
C1 [Forlenza, Enrico M.; Lavoie-Gagne, Ophelie Z.; Lu, Yining; Diaz, Connor C.; Chahla, Jorge; Forsythe, Brian] Midwest Orthopaed Rush, Div Sports Med, 1611 West Harrison St, Chicago, IL 60612 USA.
   [Forlenza, Enrico M.; Lavoie-Gagne, Ophelie Z.; Lu, Yining; Diaz, Connor C.; Chahla, Jorge; Forsythe, Brian] Rush Univ, Med Ctr, Dept Orthopaed Surg, Chicago, IL 60612 USA.
RP Forsythe, B (通讯作者)，Midwest Orthopaed Rush, Div Sports Med, 1611 West Harrison St, Chicago, IL 60612 USA.
EM brian.forsythe@rushortho.com
CR Amin Nirav H, 2016, Clin Res Foot Ankle, V4, DOI 10.4172/2329-910X.1000205
   Amin NH, 2013, AM J SPORT MED, V41, P1864, DOI 10.1177/0363546513490659
   Dendir S., 2016, J SPORT ANAL, V2, P89, DOI [DOI 10.3233/JSA-160021, 10.3233/JSA-160021]
   Egger AC, 2017, CURR REV MUSCULOSKE, V10, P72, DOI 10.1007/s12178-017-9386-7
   Eliasson P, 2018, AM J SPORT MED, V46, P2492, DOI 10.1177/0363546518781826
   Erickson BJ, 2014, ORTHOPEDICS, V37, DOI 10.3928/01477447-20140728-59
   Farber J, 2014, ORTHOP J SPORTS MED, V2, DOI 10.1177/2325967114559892
   Frankewycz B, 2018, KNEE SURG SPORT TR A, V26, P2080, DOI 10.1007/s00167-017-4791-4
   Fredberg U, 2008, AM J SPORT MED, V36, P451, DOI 10.1177/0363546507310073
   Gajhede-Knudsen M, 2013, BRIT J SPORT MED, V47, P763, DOI 10.1136/bjsports-2013-092271
   Grassi A, 2020, BRIT J SPORT MED, V54, P480, DOI 10.1136/bjsports-2019-100556
   Ho G, 2017, INJURY, V48, P1701, DOI 10.1016/j.injury.2017.04.007
   Houshian S, 1998, INJURY, V29, P651, DOI 10.1016/S0020-1383(98)00147-8
   Jandacka D, 2017, J FOOT ANKLE RES, V10, DOI 10.1186/s13047-017-0235-0
   Kalen A, 2019, FRONT PSYCHOL, V10, DOI 10.3389/fpsyg.2019.00076
   King G, 2006, POLIT ANAL, V14, P131, DOI 10.1093/pan/mpj004
   King G, 2017, AM J POLIT SCI, V61, P473, DOI 10.1111/ajps.12272
   LeBrun DG, 2019, CLIN ORTHOP RELAT R, V477, P655, DOI 10.1097/CORR.0000000000000612
   Lemme NJ, 2019, AM J SPORT MED, V47, P2360, DOI 10.1177/0363546519858609
   Leppilahti J, 1996, ACTA ORTHOP SCAND, V67, P277, DOI 10.3109/17453679608994688
   Lu YN, 2020, J SHOULDER ELB SURG, V29, P50, DOI 10.1016/j.jse.2019.05.035
   Maffulli N, 1999, CLIN J SPORT MED, V9, P157, DOI 10.1097/00042752-199907000-00007
   Maffulli N, 2011, FOOT ANKLE INT, V32, P9, DOI 10.3113/FAI.2011.0009
   Makhni EC, 2014, AM J SPORT MED, V42, P1323, DOI 10.1177/0363546514528864
   Mansournia MA, 2018, EUR J EPIDEMIOL, V33, P5, DOI 10.1007/s10654-017-0325-0
   Marshall NE, 2015, AM J SPORT MED, V43, P1051, DOI 10.1177/0363546515579636
   Minhas SV, 2016, AM J SPORT MED, V44, P1056, DOI 10.1177/0363546515623028
   Niederer D, 2018, SCAND J MED SCI SPOR, V28, P2226, DOI 10.1111/sms.13245
   Okoroha KR, 2019, ARTHROSCOPY, V35, P158, DOI 10.1016/j.arthro.2018.08.007
   Okoroha KR, 2018, J SHOULDER ELB SURG, V27, P17, DOI 10.1016/j.jse.2017.07.027
   Okoroha KR, 2017, ORTHOP J SPORTS MED, V5, DOI 10.1177/2325967117698788
   Parekh Selene G, 2009, Foot Ankle Spec, V2, P283, DOI 10.1177/1938640009351138
   Sankey RA, 2008, AM J SPORT MED, V36, P2415, DOI 10.1177/0363546508322889
   Schiffner E, 2019, J SPORT MED PHYS FIT, V59, P110, DOI 10.23736/S0022-4707.17.07901-4
   Trofa DP, 2018, ORTHOP J SPORTS MED, V6, DOI 10.1177/2325967118810772
   Trofa DP, 2017, AM J SPORT MED, V45, P2864, DOI 10.1177/0363546517713001
NR 36
TC 0
Z9 0
U1 0
U2 1
PU SAGE PUBLICATIONS INC
PI THOUSAND OAKS
PA 2455 TELLER RD, THOUSAND OAKS, CA 91320 USA
EI 2325-9671
J9 ORTHOP J SPORTS MED
JI Orthop. J. Sports Med.
PD SEP
PY 2021
VL 9
IS 9
AR 23259671211024199
DI 10.1177/23259671211024199
PG 8
WC Orthopedics; Sport Sciences
WE Science Citation Index Expanded (SCI-EXPANDED)
SC Orthopedics; Sport Sciences
GA WA8IQ
UT WOS:000703127400001
PM 35146029
OA gold, Green Published
DA 2022-07-22
ER

PT J
AU Carmont, MR
   Maffulli, N
AF Carmont, Michael R.
   Maffulli, Nicola
TI Achilles tendon rupture following surgical management for tendinopathy:
   a case report
SO BMC MUSCULOSKELETAL DISORDERS
LA English
DT Article
ID SURGERY; TENDINOSIS; INJURY; REPAIR
AB Background: Achilles tendinopathy is understood to be a failed healing response. Operative management is utilised following the failure of non-operative methods.
   Case Presentation: We present a case of Achilles tendon rupture, sustained whilst isometrically loading the Achilles tendon during an eccentric loading exercise programme. Conclusion: Bilateral surgical exploration and debridement had previously been performed after conservative management of bilateral Achilles tendinopathy had been unsuccessful.
C1 Keele Univ, Sch Med, Univ Hosp N Staffordshire, Dept Trauma & Orthopaed Surg, Stoke On Trent ST4 6QG, Staffs, England.
RP Carmont, MR (通讯作者)，Keele Univ, Sch Med, Univ Hosp N Staffordshire, Dept Trauma & Orthopaed Surg, Stoke On Trent ST4 6QG, Staffs, England.
EM mcarmont@hotmail.com; n.maffulli@orth.keele.ac.uk
RI Maffulli, Nicola/A-1839-2014
OI Maffulli, Nicola/0000-0002-5327-3702
CR Alfredson Hakan, 2005, Foot Ankle Clin, V10, P321, DOI 10.1016/j.fcl.2005.01.002
   Houglum PA, 1992, J SPORT REHABIL, V1, P19, DOI DOI 10.1123/jsr.1.1.19
   JOZSA L, 1997, HUMAN TENDON ANATOMY
   KADER D, 2002, BRIT J SPORT MED, V36, P29
   Maffulli N, 2000, AM J SPORT MED, V28, P499, DOI 10.1177/03635465000280040901
   Maffulli N, 2006, CLIN J SPORT MED, V16, P123, DOI 10.1097/00042752-200603000-00007
   Maffulli N, 1998, Bull Hosp Jt Dis, V57, P152
   Mafi N, 2001, KNEE SURG SPORT TR A, V9, P42, DOI 10.1007/s001670000148
   Matles A L, 1975, Bull Hosp Joint Dis, V36, P48
   McClelland D, 2002, J ROY COLL SURG EDIN, V47, P613
   Ohberg L, 2004, BRIT J SPORT MED, V38, P8, DOI 10.1136/bjsm.2001.000284
   Paavola M, 2000, AM J SPORT MED, V28, P77, DOI 10.1177/03635465000280012501
   Paavola M, 2002, BRIT J SPORT MED, V36, P178, DOI 10.1136/bjsm.36.3.178
   Roos EM, 2004, SCAND J MED SCI SPOR, V14, P286, DOI 10.1111/j.1600-0838.2004.378.x
   Saxena A, 2003, J AM PODIAT MED ASSN, V93, P283, DOI 10.7547/87507315-93-4-283
   SAYANA MK, 2006, IN PRESS J SCI  0705
   Shalabi A, 2004, AM J SPORT MED, V32, P1286, DOI 10.1177/0363546504263148
   Sharma P, 2005, J BONE JOINT SURG AM, V87A, P187, DOI 10.2106/JBJS.D.01850
   SIMMONDS F A, 1957, Practitioner, V179, P56
   Sorosky B, 2004, CLIN J SPORT MED, V14, P40, DOI 10.1097/00042752-200401000-00007
   Tallon C, 2001, AM J SPORT MED, V29, P315, DOI 10.1177/03635465010290031101
   Tallon C, 2001, MED SCI SPORT EXER, V33, P1983, DOI 10.1097/00005768-200112000-00002
   Vora Anand M, 2005, Foot Ankle Clin, V10, P293, DOI 10.1016/j.fcl.2005.01.007
   Vulpiani MC, 2003, INT ORTHOP, V27, P307, DOI 10.1007/s00264-003-0472-7
   Young Jonathan S, 2005, Foot Ankle Clin, V10, P371, DOI 10.1016/j.fcl.2005.01.004
NR 25
TC 5
Z9 5
U1 0
U2 3
PU BIOMED CENTRAL LTD
PI LONDON
PA MIDDLESEX HOUSE, 34-42 CLEVELAND ST, LONDON W1T 4LB, ENGLAND
SN 1471-2474
J9 BMC MUSCULOSKEL DIS
JI BMC Musculoskelet. Disord.
PD FEB 27
PY 2007
VL 8
AR 19
DI 10.1186/1471-2474-8-19
PG 4
WC Orthopedics; Rheumatology
WE Science Citation Index Expanded (SCI-EXPANDED)
SC Orthopedics; Rheumatology
GA 142PJ
UT WOS:000244661700001
PM 17326834
OA Green Accepted, gold, Green Published
DA 2022-07-22
ER

PT J
AU Lui, TH
AF Lui, Tun Hing
TI Endoscopic Achilles Tenolysis for Management of Heel Cord Pain after
   Repair of Acute Rupture of Achilles Tendon
SO JOURNAL OF FOOT & ANKLE SURGERY
LA English
DT Article
DE calcaneus; endoscopy; injury; pain; rupture; tendoscopy
ID TENDINOSIS
AB Tendon pain after repair of an acute Achilles tendon rupture can result from suture granuloma formation, modification of the threshold of the pain receptors inside the tendon by scar tissue, expansion of the paratenon by tendon enlargement with secondary stimulation of mechanoreceptors, or underlying tendon degeneration. In the present technique report, an endoscopic technique of Achilles tenolysis for denervation and debulking is described that might be applicable in cases in which conservative treatment fails to alleviate the pain. (C) 2013 by the American College of Foot and Ankle Surgeons. All rights reserved.
C1 N Dist Hosp, Dept Orthopaed & Traumatol, Sheung Shui, Hong Kong, Peoples R China.
RP Lui, TH (通讯作者)，N Dist Hosp, Dept Orthopaed & Traumatol, 9 Po Kin Rd, Sheung Shui, Hong Kong, Peoples R China.
EM luithderek@yahoo.co.uk
CR Alfredson H, 2011, BRIT J SPORT MED, V45, P1023, DOI 10.1136/bjsports-2011-090217
   Andersson G, 2007, KNEE SURG SPORT TR A, V15, P1272, DOI 10.1007/s00167-007-0364-2
   Bjur D, 2005, CELL TISSUE RES, V320, P201, DOI 10.1007/s00441-004-1014-3
   Maffulli N, 2010, OPEN ACCESS J SPORTS, V1, P29
   Maquirriain J, 2002, ARTHROSCOPY, V18, P298, DOI 10.1053/jars.2002.30065b
   Ohberg L, 2001, KNEE SURG SPORT TR A, V9, P233, DOI 10.1007/s001670000189
   Osadnik R, 2010, KNEE SURG SPORT TR A, V18, P977, DOI 10.1007/s00167-009-0958-y
   Phisitkul P, 2012, CURR REV MUSCULOSKE, V5, P156, DOI 10.1007/s12178-012-9115-1
   Rebeccato A, 2001, J Foot Ankle Surg, V40, P188
   Schubert TEO, 2005, ANN RHEUM DIS, V64, P1083, DOI 10.1136/ard.2004.029876
   Steenstra F, 2006, FOOT ANKLE CLIN, V11, p[428, viii]
   Steenstra Ferry, 2006, Foot Ankle Clin, V11, P429, DOI 10.1016/j.fcl.2006.02.001
   Strauss EJ, 2007, INJURY, V38, P832, DOI 10.1016/j.injury.2006.06.005
   Thermann H, 2009, KNEE SURG SPORT TR A, V17, P1264, DOI 10.1007/s00167-009-0751-y
   van Sterkenburg MN, 2011, KNEE SURG SPORT TR A, V19, P1367, DOI 10.1007/s00167-011-1535-8
NR 15
TC 5
Z9 7
U1 0
U2 1
PU ELSEVIER SCIENCE INC
PI NEW YORK
PA 360 PARK AVE SOUTH, NEW YORK, NY 10010-1710 USA
SN 1067-2516
J9 J FOOT ANKLE SURG
JI J. Foot Ankle Surg.
PD JAN-FEB
PY 2013
VL 52
IS 1
BP 125
EP 127
DI 10.1053/j.jfas.2012.09.007
PG 3
WC Orthopedics; Surgery
WE Science Citation Index Expanded (SCI-EXPANDED)
SC Orthopedics; Surgery
GA 061ZU
UT WOS:000312889700028
PM 23085384
DA 2022-07-22
ER

PT J
AU Cook, JL
   Khan, KM
   Purdam, C
AF Cook, JL
   Khan, KM
   Purdam, C
TI Achilles tendinopathy
SO MANUAL THERAPY
LA English
DT Article
ID TENDON RUPTURE; PATELLAR TENDINITIS; CLINICAL-DIAGNOSIS; TENDINOSIS;
   IMMOBILIZATION; REMOBILIZATION; INJURIES; LESIONS; MOTION; PAIN
AB Achilles tendon injury (tendinopathy) and pain occur inactive individuals, when the tendon is subject to high or unusual load. Achilles tendinopathy can be resistant to treatment, and symptoms may persist despite both conservative and surgical intervention. The pathology of overuse tendinopathy is non-inflammatory, with a degenerative or failed healing tendon response. The diagnosis of Achilles tendinopathy requires excellent differential diagnosis and an understanding of the role of tendon imaging. Conservative treatment must include exercise, with a bias to eccentric contractions. Surgical treatment is effective after complete tendon rupture, but may not assist recovery from overuse tendinopathy. Further research into the clinical aspects of Achilles tendinopathy is required. (C) 2002 Elsevier Science Ltd. All rights reserved.
C1 La Trobe Univ, Musculoskeletal Res Ctr, Bundoora, Vic 3086, Australia.
   Univ British Columbia, Sch Human Kinet, Allan McGavin Sports Med Ctr, Dept Family Practice, Vancouver, BC V5Z 1M9, Canada.
   Australian Inst Sport, Canberra, ACT, Australia.
RP Cook, JL (通讯作者)，La Trobe Univ, Musculoskeletal Res Ctr, Bundoora, Vic 3086, Australia.
EM j.cook@latrobe.edu.au
CR Ahmed IM, 1998, J ORTHOPAED RES, V16, P591, DOI 10.1002/jor.1100160511
   Alfredson H, 1998, AM J SPORT MED, V26, P360, DOI 10.1177/03635465980260030301
   ALMEKINDERS LC, 1993, MED SCI SPORT EXER, V25, P603
   ASTROM M, 1994, J ORTHOP RES, V12, P246, DOI 10.1002/jor.1100120214
   ASTROM M, 1995, CLIN ORTHOP RELAT R, P151
   ASTROM M, 1997, NATURE ETIOLOGY CHRO
   Benazzo Francesco, 1996, Bulletin Hospital for Joint Diseases, V54, P236
   Bressel E, 2001, AM J SPORT MED, V29, P321, DOI 10.1177/03635465010290031201
   CAMERON DM, 1994, J ORTHOP SPORT PHYS, V19, P168, DOI 10.2519/jospt.1994.19.3.168
   Clancy W., 1989, SPORTS INDUCED INFLA
   CLEMENT DB, 1984, AM J SPORT MED, V12, P179, DOI 10.1177/036354658401200301
   Cook JL, 2001, BRIT J SPORT MED, V35, P65, DOI 10.1136/bjsm.35.1.65
   Curwin S, 1984, TENDINITIS ITS ETIOL
   DAVIES SG, 1991, CLIN RADIOL, V43, P52, DOI 10.1016/S0009-9260(05)80355-1
   Frank C, 1999, J Sci Med Sport, V2, P190, DOI 10.1016/S1440-2440(99)80173-X
   Gibbon WW, 1999, BRIT J SPORT MED, V33, P129, DOI 10.1136/bjsm.33.2.129
   Gibbon WW, 2000, J CLIN ULTRASOUND, V28, P61, DOI 10.1002/(SICI)1097-0096(200002)28:2<61::AID-JCU1>3.0.CO;2-R
   Haims AH, 2000, SKELETAL RADIOL, V29, P640, DOI 10.1007/s002560000273
   Horstmann T, 2000, ISOKINET EXERC SCI, V8, P141, DOI 10.3233/IES-2000-0044
   Hunter G, 2000, PHYS THER SPORT, V1, P6
   JOZSA L, 1991, ACTA ANAT, V142, P306
   JOZSA L, 1990, ARCH ORTHOP TRAUM SU, V110, P15, DOI 10.1007/BF00431359
   Kannus P, 1997, SCAND J MED SCI SPOR, V7, P67
   KANNUS P, 1991, J BONE JOINT SURG AM, V73A, P1507, DOI 10.2106/00004623-199173100-00009
   Kaufman KR, 1999, AM J SPORT MED, V27, P585, DOI 10.1177/03635465990270050701
   Khan KM, 1999, SPORTS MED, V27, P393, DOI 10.2165/00007256-199927060-00004
   Khan KM, 1996, RADIOLOGY, V200, P821, DOI 10.1148/radiology.200.3.8756939
   Khan KM, 2000, SPORTS MED ARTHROSC, V8, P17, DOI 10.1097/00132585-200008010-00003
   Kraushaar BS, 1999, J BONE JOINT SURG AM, V81A, P259, DOI 10.2106/00004623-199902000-00014
   KVIST M, 1992, INT ORTHOP, V16, P377
   LANG J, 1960, Z Anat Entwicklungsgesch, V122, P197, DOI 10.1007/BF00521798
   LEADBETTER WB, 1992, CLIN SPORT MED, V11, P533
   Maffulli N, 1998, AM J SPORT MED, V26, P266, DOI 10.1177/03635465980260021801
   Maffulli N, 2000, AM J SPORT MED, V28, P857, DOI 10.1177/03635465000280061401
   Maffulli N, 1999, CLIN J SPORT MED, V9, P157, DOI 10.1097/00042752-199907000-00007
   Maffulli N, 1998, BRIT J SPORT MED, V32, P174, DOI 10.1136/bjsm.32.2.174
   Maffulli N, 1999, AM J SPORT MED, V27, P747, DOI 10.1177/03635465990270061101
   Mafi N, 2001, KNEE SURG SPORT TR A, V9, P42, DOI 10.1007/s001670000148
   Moller M, 2001, J BONE JOINT SURG BR, V83B, P843
   OBRIEN M, 1992, CLIN SPORT MED, V11, P505
   Ohberg L, 2001, KNEE SURG SPORT TR A, V9, P233, DOI 10.1007/s001670000189
   Paavola M, 2000, AM J SPORT MED, V28, P634, DOI 10.1177/03635465000280050301
   RAIS O, 1961, ACTA CHIRURG SCAND S, V268
   Robinson JM, 2001, BRIT J SPORT MED, V35, P335, DOI 10.1136/bjsm.35.5.335
   RUFAI A, 1995, J ORTHOP RES, V13, P585, DOI 10.1002/jor.1100130414
   Silbernagel KG, 2001, SCAND J MED SCI SPOR, V11, P197
   Tallon C, 2001, AM J SPORT MED, V29, P315, DOI 10.1177/03635465010290031101
   Tuite DJ, 1997, SCAND J MED SCI SPOR, V7, P72
   WILLIAMS JGP, 1986, SPORTS MED, V3, P114, DOI 10.2165/00007256-198603020-00003
   Wilson JK, 2000, J SPORT REHABIL, V9, P304, DOI 10.1123/jsr.9.4.304
   Wren TAL, 2000, J REHABIL RES DEV, V37, P217
NR 51
TC 103
Z9 103
U1 1
U2 30
PU CHURCHILL LIVINGSTONE
PI EDINBURGH
PA JOURNAL PRODUCTION DEPT, ROBERT STEVENSON HOUSE, 1-3 BAXTERS PLACE,
   LEITH WALK, EDINBURGH EH1 3AF, MIDLOTHIAN, SCOTLAND
SN 1356-689X
EI 1532-2769
J9 MANUAL THER
JI Man. Ther.
PD AUG
PY 2002
VL 7
IS 3
BP 121
EP 130
DI 10.1054/math.2002.0458
PG 10
WC Rehabilitation
WE Science Citation Index Expanded (SCI-EXPANDED)
SC Rehabilitation
GA 613GR
UT WOS:000179125000002
PM 12372309
DA 2022-07-22
ER

PT J
AU Miller, D
   Waterston, S
   Reaper, J
   Barrass, V
   Maffulli, N
AF Miller, D
   Waterston, S
   Reaper, J
   Barrass, V
   Maffulli, N
TI Conservative management, percutaneous or open repair of acute Achilles
   tendon rupture: A retrospective study
SO SCOTTISH MEDICAL JOURNAL
LA English
DT Article
DE Achilles tendon; rupture; repair
ID NONOPERATIVE TREATMENT; NONSURGICAL TREATMENT; SURGICAL REPAIR
AB Background and Aims: To ascertain the differences inpatients' perceived outcomes between conservative, percutaneous or open repair of Achilles tendon ruptures. Methods: We studied 111 patients who bad been managed for a unilateral closed Achilles tendon rupture. We excluded patients with open Achilles tendon lesions, patients whose tear bad occurred more than seven days from operation, patients with diabetes, inflammatory disease, systemic corticosteroids or fluoroquinolones. Patients were contacted by telephone and asked to answer a questionnaire. Results: There was no difference in effects on working life, patient satisfaction, sports performance, muscle strength, swelling and fear of re-rupture. Pain on weight hearing and cramps were significantly more frequent in the patients managed conservatively. Re-rupture and complication rates were comparable to published rates. Conclusion: Treatment should be individualised according to the demands and health of the patient.
C1 Keele Univ, Sch Med, Dept Trauma & Orthopaed Surg, Stoke On Trent ST4 7QB, Staffs, England.
   Univ Aberdeen, Sch Med, Dept Orthopaed Surg, Aberdeen, Scotland.
RP Maffulli, N (通讯作者)，Keele Univ, Sch Med, Dept Trauma & Orthopaed Surg, Hartshill,Thornburrow Dr, Stoke On Trent ST4 7QB, Staffs, England.
EM n.maffulli@keele.ac.uk
RI Maffulli, Nicola/A-1839-2014
OI Maffulli, Nicola/0000-0002-5327-3702
CR [Anonymous], [No title captured]
   ARACIL J, 1992, FOOT ANKLE, V13, P350, DOI 10.1177/107110079201300610
   BESKIN JL, 1987, AM J SPORT MED, V15, P1, DOI 10.1177/036354658701500101
   BRADLEY JP, 1990, AM J SPORT MED, V18, P188, DOI 10.1177/036354659001800213
   CARDEN DG, 1984, J BONE JOINT SURG BR, V66, P286
   Casteleyn P P, 1980, Acta Orthop Belg, V46, P310
   CETTI R, 1993, AM J SPORT MED, V21, P791, DOI 10.1177/036354659302100606
   Cretnik A, 2005, AM J SPORT MED, V33, P1369, DOI 10.1177/0363546504271501
   Cretnik Andrej, 2004, J Foot Ankle Surg, V43, P72, DOI 10.1053/j.jfas.2004.01.008
   EDNA TH, 1980, ACTA ORTHOP SCAND, V51, P991, DOI 10.3109/17453678008990905
   FRUENSGAARD S, 1992, INT ORTHOP, V16, P33
   GILLIES H, 1970, J BONE JOINT SURG AM, VA 52, P337, DOI 10.2106/00004623-197052020-00015
   HATTRUP SJ, 1985, FOOT ANKLE, V6, P34, DOI 10.1177/107110078500600107
   HOCKENBURY RT, 1990, FOOT ANKLE, V11, P67, DOI 10.1177/107110079001100202
   JOZSA L, 1989, AM J SPORT MED, V17, P338, DOI 10.1177/036354658901700305
   Kader D, 2005, TENDON INJURIES: BASIC SCIENCE AND CLINICAL MEDICINE, P187, DOI 10.1007/1-84628-050-8_20
   Khan RJ, 2004, COCHRANE DB SYST REV, V3, DOI DOI 10.1002/14651858.CD003674.PUB2
   KLEIN W, 1991, CHIR ORG MOVIMENTO, V76, P229
   KRUEGERFRANKE M, 1995, BRIT J SPORT MED, V29, P121, DOI 10.1136/bjsm.29.2.121
   KVISTKRISTIANSE.J, 1972, J TRAUMA, V12, P794
   Lea R B, 1968, Clin Orthop Relat Res, V60, P115
   Lim J, 2001, FOOT ANKLE INT, V22, P559, DOI 10.1177/107110070102200705
   Lo IKY, 1997, CLIN J SPORT MED, V7, P207, DOI 10.1097/00042752-199707000-00010
   MA GWC, 1977, CLIN ORTHOP RELAT R, P247
   Maffulli N, 1999, J BONE JOINT SURG AM, V81A, P1019, DOI 10.2106/00004623-199907000-00017
   Maffulli N, 1999, J ORTHOP TRAUMA, V13, P395, DOI 10.1097/00005131-199906000-00029
   Maffulli N, 1998, AM J SPORT MED, V26, P266, DOI 10.1177/03635465980260021801
   McClelland D, 2002, J ROY COLL SURG EDIN, V47, P613
   McComis GP, 1997, J BONE JOINT SURG AM, V79A, P1799, DOI 10.2106/00004623-199712000-00005
   Moller M, 2001, J BONE JOINT SURG BR, V83B, P843
   NISTOR L, 1981, J BONE JOINT SURG AM, V63, P394, DOI 10.2106/00004623-198163030-00012
   QUIGLEY TB, 1980, AM J SPORT MED, V8, P244, DOI 10.1177/036354658000800406
   RALSTON EL, 1971, J TRAUM, V11, P15, DOI 10.1097/00005373-197101000-00001
   Roberts C, 1989, Orthop Rev, V18, P513
   Sutherland A, 1998, Oper Orthop Traumatol, V10, P50, DOI 10.1007/s00064-006-0044-2
   Wallace RGH, 2004, J BONE JOINT SURG AM, V86A, P1198, DOI 10.2106/00004623-200406000-00011
   WATERSTON SW, 1997, THESIS U ABERDEEN AB, P1
   Webb JM, 1999, J BONE JOINT SURG BR, V81B, P877, DOI 10.1302/0301-620X.81B5.9784
   WILLS CA, 1986, CLIN ORTHOP RELAT R, V207, P156
   Wong J, 2002, AM J SPORT MED, V30, P565, DOI 10.1177/03635465020300041701
NR 40
TC 20
Z9 22
U1 0
U2 4
PU SAGE PUBLICATIONS LTD
PI LONDON
PA 1 OLIVERS YARD, 55 CITY ROAD, LONDON EC1Y 1SP, ENGLAND
SN 0036-9330
EI 2045-6441
J9 SCOT MED J
JI Scott. Med. J.
PD NOV
PY 2005
VL 50
IS 4
BP 160
EP 165
DI 10.1177/003693300505000408
PG 6
WC Medicine, General & Internal
WE Science Citation Index Expanded (SCI-EXPANDED)
SC General & Internal Medicine
GA 994JV
UT WOS:000234028900008
PM 16374980
DA 2022-07-22
ER

PT J
AU September, AV
   Posthumus, M
   van der Merwe, L
   Schwellnus, M
   Noakes, TD
   Collins, M
AF September, A. V.
   Posthumus, M.
   van der Merwe, L.
   Schwellnus, M.
   Noakes, T. D.
   Collins, M.
TI The COL12A1 and COL14A1 genes and Achilles tendon injuries
SO INTERNATIONAL JOURNAL OF SPORTS MEDICINE
LA English
DT Article
DE tendinopathy; rupture; FACIT; collagen genes
ID XIV COLLAGEN; RISK-FACTORS; TENASCIN-C; EXPRESSION; FIBRILLOGENESIS;
   PERSPECTIVE; FIBRILS; UNDULIN; DISEASE; FAMILY
AB Genes encoding for tenascin C and a subunit of type V collagen have previously been reported to be associated with Achilles tendon injuries. Types XII and XIV collagen may be involved in similar biological processes as these proteins in tendons. The aim of this study was therefore to test the association between polymorphisms within COL12A1 and COL14A1 and Achilles tendon injuries. Restriction fragment length polymorphism (RFLP) analysis was used to identify the relative frequencies of two polymorphisms within each of the COL12A1 and COL14A1 genes within 137 subjects with clinical symptoms of Achilles tendon injuries, consisting of 93 with Achilles tendinopathy and 44 with Achilles tendon rupture, and 131 asymptomatic control subjects. No statistically significant differences were identified in the genotype, allele or haplotype distributions between the affected and control subjects. The findings from this study suggest that although COL12A1 and COL14A1 are involved in similar biological processes as TNC and COL5A1, the polymorphisms tested are not associated with clinical symptoms of Achilles tendon injury within the investigated population.
C1 [September, A. V.; Posthumus, M.; Schwellnus, M.; Noakes, T. D.; Collins, M.] Univ Cape Town, Dept Human Biol, MRC, Res Unit Exercise Sci & Sports Med,UCT, ZA-7925 Cape Town, South Africa.
   [van der Merwe, L.] S African MRC, Biostat Res Unit, Cape Town, South Africa.
   [Collins, M.] S African MRC, Exercise Sci & Sports Med Res Unit, Cape Town, South Africa.
RP Collins, M (通讯作者)，S African MRC, Exercise Sci & Sports Med Res Unit, POB 115, ZA-7725 Newlands, South Africa.
EM malcolm.collins@mrc.ac.za
RI van der Merwe, Lize/D-3148-2012; Collins, Malcolm/G-4046-2011; Noakes,
   Tim/AAD-3162-2020; Noakes, Timothy D./E-7253-2011; Schwellnus,
   Martin/E-6042-2010
OI van der Merwe, Lize/0000-0001-9705-8126; Collins,
   Malcolm/0000-0002-2564-0480; Noakes, Tim/0000-0001-7244-2375; Noakes,
   Timothy D./0000-0001-7244-2375; Schwellnus, Martin/0000-0003-3647-0429;
   September, Alison V/0000-0003-0950-286X
CR Akutsu N, 1999, EXP DERMATOL, V8, P17, DOI 10.1111/j.1600-0625.1999.tb00343.x
   Ala-Kokko L, 2002, ANN MED, V34, P42, DOI 10.1080/078538902317338634
   BIRK DE, 1990, J CELL SCI, V95, P649
   Chiquet M, 1999, MATRIX BIOL, V18, P417, DOI 10.1016/S0945-053X(99)00039-6
   DUBLET B, 1991, J BIOL CHEM, V266, P6853
   Gerecke DR, 1997, GENOMICS, V41, P236, DOI 10.1006/geno.1997.4638
   GORDON MK, 1987, P NATL ACAD SCI USA, V84, P6040, DOI 10.1073/pnas.84.17.6040
   Jarvinen TAH, 2003, J CELL SCI, V116, P857, DOI 10.1242/jcs.00303
   Jarvinen Tero A H, 2005, Foot Ankle Clin, V10, P255, DOI 10.1016/j.fcl.2005.01.013
   Jones FS, 2000, DEV DYNAM, V218, P235, DOI 10.1002/(SICI)1097-0177(200006)218:2<235::AID-DVDY2>3.0.CO;2-G
   KEENE DR, 1991, J CELL BIOL, V113, P971, DOI 10.1083/jcb.113.4.971
   KVIST M, 1994, SPORTS MED, V18, P173, DOI 10.2165/00007256-199418030-00004
   LAHIRI DK, 1991, NUCLEIC ACIDS RES, V19, P5444, DOI 10.1093/nar/19.19.5444
   Mazzone MF, 2002, AM FAM PHYSICIAN, V65, P1805
   Mokone GG, 2006, SCAND J MED SCI SPOR, V16, P19, DOI 10.1111/j.1600-0838.2005.00439.x
   Mokone GG, 2005, AM J SPORT MED, V33, P1016, DOI 10.1177/0363546504271986
   NISHIYAMA T, 1994, J BIOL CHEM, V269, P28193
   RICH SS, 1990, DIABETES, V39, P1315, DOI 10.2337/diabetes.39.11.1315
   Riley G, 2004, RHEUMATOLOGY, V43, P131, DOI 10.1093/rheumatology/keg448
   Risch N, 2001, CANCER EPIDEM BIOMAR, V10, P733
   SCHNITTGER S, 1995, CYTOGENET CELL GENET, V68, P233, DOI 10.1159/000133920
   SCHUPPAN D, 1990, J BIOL CHEM, V265, P8823
   September AV, 2006, INT SPORTMED J, V7, P201
   SHAW LM, 1991, TRENDS BIOCHEM SCI, V16, P191, DOI 10.1016/0968-0004(91)90074-6
   Thomopoulos S, 2002, J ORTHOP RES, V20, P454, DOI 10.1016/S0736-0266(01)00144-9
   Todd JA, 2001, NATURE, V411, P537, DOI 10.1038/35079223
   WALCHLI C, 1994, J CELL SCI, V107, P669
   WATT SL, 1992, J BIOL CHEM, V267, P20093
   Young BB, 2002, J CELL BIOCHEM, V87, P208, DOI 10.1002/jcb.10290
   Zhang GY, 2003, J ANAT, V202, P411, DOI 10.1046/j.1469-7580.2003.00174.x
NR 30
TC 38
Z9 40
U1 0
U2 5
PU GEORG THIEME VERLAG KG
PI STUTTGART
PA RUDIGERSTR 14, D-70469 STUTTGART, GERMANY
SN 0172-4622
J9 INT J SPORTS MED
JI Int. J. Sports Med.
PD MAR
PY 2008
VL 29
IS 3
BP 257
EP 263
DI 10.1055/s-2007-965127
PG 7
WC Sport Sciences
WE Science Citation Index Expanded (SCI-EXPANDED)
SC Sport Sciences
GA 275OV
UT WOS:000254082400014
PM 17960519
DA 2022-07-22
ER

PT J
AU Svedman, S
   Juthberg, R
   Edman, G
   Ackermann, PW
AF Svedman, Simon
   Juthberg, Robin
   Edman, Gunnar
   Ackermann, Paul W.
TI Reduced Time to Surgery Improves Patient-Reported Outcome After Achilles
   Tendon Rupture
SO AMERICAN JOURNAL OF SPORTS MEDICINE
LA English
DT Article
DE foot; Achilles tendon; rupture; time to treatment; patient-reported
   outcome measures; postoperative complications; venous thrombosis
ID ANTERIOR CRUCIATE LIGAMENT; DEEP-VEIN THROMBOSIS; FRACTURE SURGERY;
   DELAY; MORTALITY; INJURY; RECONSTRUCTION; EPIDEMIOLOGY; 30-DAY; TRIAL
AB Background: Patient outcome after an acute Achilles tendon rupture (ATR) continues to be suboptimal and heterogeneous. Thus, prognostic factors are called for to optimize evidence-based ATR treatment protocols; however, the influence of delayed time from injury to surgery (TTS) on patient outcome after ATR remains largely unknown.
   Purpose: To determine whether patient outcomes and adverse events after surgical repair of acute ATR are related to delayed TTS.
   Study Design: Cohort study; Level of evidence, 3.
   Methods: Two hundred twenty-eight patients with ATR who were treated with uniform anesthetic and surgical techniques, within 10 days after injury, were retrospectively assessed. TTS depended on availability of the operating theater, and neither surgeon nor patient could influence the TTS. Patients were assigned to 1 of 3 groups: short TTS (<48 hours), intermediate TTS (48-72 hours), and long TTS (>72 hours). Patient-reported outcome at 1 year was assessed with the validated Achilles tendon Total Rupture Score, with scores higher than 80 on a 0- to 100-point scale indicating an overall good outcome. The incidences of adverse events (perioperative and postoperative) and deep venous thrombosis were assessed.
   Results: Short TTS was significantly associated with increased rate of good outcome and reduced risk of adverse events. Seventy-one percent (95% CI, 60%-83%) of the patients with short TTS attained a good outcome compared with 44% (95% CI, 33%-56%) of the patients with long TTS (P = .002), with the intermediate TTS group in between, 63% (95% CI, 47%-78%). The incidence of adverse events was significantly reduced among patients with short TTS, 1.4% (95% CI, 1%-4%) compared with those with intermediate TTS, 11% (95% CI, 2%-21%) (P = .035) and those with long TTS, 14.8% (95% CI, 7%-23%) (P = .003). The risk of sustaining a deep venous thrombosis was not statistically significantly different among the 3 groups (P = .15).
   Conclusion: Patients with acute ATR undergoing operation within 48 hours after injury had better outcomes and a lower number of adverse events compared with patients undergoing operation after 72 hours. These results align with evidence-based recommendations from other surgical disciplines and should be used as guidelines for optimizing ATR treatment protocols.
C1 [Svedman, Simon; Juthberg, Robin; Edman, Gunnar; Ackermann, Paul W.] Karolinska Univ Hosp, Stockholm, Sweden.
   [Svedman, Simon; Juthberg, Robin; Ackermann, Paul W.] Karolinska Inst, Dept Mol Med & Surg, Integrat Orthoped Lab, Stockholm, Sweden.
   [Edman, Gunnar] Tiohundra AB, Dept Psychiat, Norrtalje, Sweden.
   [Ackermann, Paul W.] Karolinska Univ Hosp, Dept Orthoped Surg, Stockholm, Sweden.
RP Ackermann, PW (通讯作者)，Karolinska Univ Hosp, Orthoped Dept, S-17176 Stockholm, Sweden.; Ackermann, PW (通讯作者)，Karolinska Inst, Dept Mol Med & Surg, Integrat Orthoped Lab, Stockholm, Sweden.; Ackermann, PW (通讯作者)，Karolinska Univ Hosp, Dept Orthoped Surg, Stockholm, Sweden.
EM paul.ackermann@karolinska.se
RI W, Ackermann P/B-9714-2008
OI W, Ackermann P/0000-0002-5520-169X
CR Ackermann PW, 2017, SURG ATLAS ACHILLES, P135
   Alim MA, 2018, KNEE SURG SPORT TR A, V26, P2021, DOI 10.1007/s00167-017-4621-8
   Anderson AF, 2015, AM J SPORT MED, V43, P275, DOI 10.1177/0363546514559912
   Brorsson A, 2017, AM J SPORT MED, V45, P3060, DOI 10.1177/0363546517717698
   Domeij-Arverud E, 2016, BONE JOINT J, V98B, P1635, DOI 10.1302/0301-620X.98B12.BJJ-2016-0008.R1
   Domeij-Arverud E, 2015, BONE JOINT J, V97B, P675, DOI 10.1302/0301-620X.97B5.34581
   Hantes ME, 2011, KNEE SURG SPORT TR A, V19, P1766, DOI 10.1007/s00167-011-1396-1
   Houshian S, 1998, INJURY, V29, P651, DOI 10.1016/S0020-1383(98)00147-8
   Huttunen TT, 2014, AM J SPORT MED, V42, P2419, DOI 10.1177/0363546514540599
   Inokuchi T, 2017, AM J SPORT MED, V45, P1359, DOI 10.1177/0363546517689871
   Lapidus L, 2006, J THROMB HAEMOST, V4, P807, DOI 10.1111/j.1538-7836.2006.01832.x
   Madhu R, 2006, J BONE JOINT SURG BR, V88B, P1197, DOI 10.1302/0301-620X.88B9.17588
   Magarian EM, 2010, AM J SPORT MED, V38, P2528, DOI 10.1177/0363546510377416
   National Institute for Health and Clinical Excellence, 2011, MAN HIP FRACT AD
   Nilsson-Helander K, 2007, AM J SPORT MED, V35, P421, DOI 10.1177/0363546506294856
   Nyholm AM, 2015, J BONE JOINT SURG AM, V97A, P1333, DOI 10.2106/JBJS.O.00029
   Park YH, 2017, INJURY, V48, P776, DOI 10.1016/j.injury.2017.01.020
   Pincus D, 2017, JAMA-J AM MED ASSOC, V318, P1994, DOI 10.1001/jama.2017.17606
   Sheth U, 2017, BONE JOINT J, V99B, P78, DOI [10.1302/0301-620X.99B1.BJJ-2016-0434.R1, 10.1302/0301-620x.99b1.bjj-2016-0434.r1]
   Siegmeth AW, 2005, J BONE JOINT SURG BR, V87B, P1123, DOI 10.1302/0301-620X.87B8
   Simunovic N, 2010, CAN MED ASSOC J, V182, P1609, DOI 10.1503/cmaj.092220
   Svedman S, 2018, KNEE SURG SPORT TR A, V26, P2011, DOI 10.1007/s00167-017-4606-7
   Valkering KP, 2017, KNEE SURG SPORT TR A, V25, P1807, DOI 10.1007/s00167-016-4270-3
   Zafar SN, 2015, JAMA SURG, V150, P129, DOI 10.1001/jamasurg.2014.2339
   ZUCKERMAN JD, 1995, J BONE JOINT SURG AM, V77, P1551, DOI 10.2106/00004623-199510000-00010
NR 25
TC 15
Z9 17
U1 0
U2 6
PU SAGE PUBLICATIONS INC
PI THOUSAND OAKS
PA 2455 TELLER RD, THOUSAND OAKS, CA 91320 USA
SN 0363-5465
EI 1552-3365
J9 AM J SPORT MED
JI Am. J. Sports Med.
PD OCT
PY 2018
VL 46
IS 12
BP 2929
EP 2934
DI 10.1177/0363546518793655
PG 6
WC Orthopedics; Sport Sciences
WE Science Citation Index Expanded (SCI-EXPANDED)
SC Orthopedics; Sport Sciences
GA GV7XJ
UT WOS:000446347200019
PM 30169112
OA Green Accepted
DA 2022-07-22
ER

PT J
AU Backer, HC
   Wong, TT
   Vosseller, JT
AF Backer, Henrik C.
   Wong, Tony T.
   Vosseller, J. Turner
TI MRI Assessment of Degeneration of the Tendon in Achilles Tendon Ruptures
SO FOOT & ANKLE INTERNATIONAL
LA English
DT Article
DE Achilles tendon; Achilles tendon rupture; tendinosis; tendinopathy
ID LONG-TERM; REPAIR; ATROPHY; TRIAL
AB Background: The extent of tendinosis along the entire tendon in Achilles tendon ruptures (ATRs) has not previously been assessed. MR images of acute ATRs were evaluated to assess this extent, even in areas distant from the rupture. Methods: Patients who had MR images for an ATR were reviewed. Each tendon was divided into a proximal, middle, and distal segment with the full tendon length defined as the length measured from the myotendinous junction to the insertion. The site of tendon rupture, presence of tendinosis and/or additional tearing, and largest anteroposterior (AP) diameter in the remaining segments were noted. In total, 45 MR iamges were included in our study. Results: The mean total tendon length was 9.93 cm, while the length from the insertion to the tear was 5.86 cm. Of the 45 ruptures, 20 (44.4%) were in the proximal third, 19 (42.2%) in the middle third, and 6 (13.3%) in the distal third. In all remaining segments where no ruptured tendon was observed, tendinosis was seen. Further, of the 90 segments of a tendon without rupture, 87 (96.7%) had an AP diameter greater than 6 mm. Of those 90 segments without rupture, 24 (26.7%) had a secondary partial-thickness tear in another third. Conclusion: Tendinosis is a known precursor of an ATR that in the current study was not limited to the area of rupture. The finding of extensive tendon degeneration may have implications for the optimal surgical treatment.
C1 [Backer, Henrik C.; Vosseller, J. Turner] Columbia Univ, Dept Orthopaed Surg, Med Ctr, New York, NY 10032 USA.
   [Wong, Tony T.] Columbia Univ, Med Ctr, Dept Radiol, New York, NY 10032 USA.
RP Vosseller, JT (通讯作者)，Columbia Univ, Med Ctr, Dept Orthopaed Surg, New York Presbyterian Hosp, 622 West 168th St PH-11, New York, NY 10032 USA.
EM jtv2111@cumc.columbia.edu
RI Vosseller, J. Turner/S-2479-2019
OI Vosseller, J. Turner/0000-0002-9639-3739; Backer,
   Henrik/0000-0002-4265-477X
CR Backus JD, 2017, FOOT ANKLE INT, V38, P564, DOI 10.1177/1071100716687368
   Cottom JM, 2017, J FOOT ANKLE SURG, V56, P423, DOI 10.1053/j.jfas.2016.10.012
   Demetracopoulos CA, 2014, FOOT ANKLE INT, V35, P612, DOI 10.1177/1071100714524550
   Eliasson P, 2018, AM J SPORT MED, V46, P2492, DOI 10.1177/0363546518781826
   Frankewycz B, 2018, KNEE SURG SPORT TR A, V26, P2080, DOI 10.1007/s00167-017-4791-4
   Heikkinen J, 2017, J BONE JOINT SURG AM, V99, P1509, DOI 10.2106/JBJS.16.01491
   Heikkinen J, 2017, AM J SPORT MED, V45, P1395, DOI 10.1177/0363546517694610
   Herbort M, 2008, ARCH ORTHOP TRAUM SU, V128, P1273, DOI 10.1007/s00402-008-0602-1
   KANNUS P, 1991, J BONE JOINT SURG AM, V73A, P1507, DOI 10.2106/00004623-199173100-00009
   Lantto I, 2015, AM J SPORT MED, V43, P2302, DOI 10.1177/0363546515591267
   Maffulli N, 2003, CLIN SPORT MED, V22, P675, DOI 10.1016/S0278-5919(03)00004-8
   Noback PC, 2018, FOOT ANKLE INT, V39, P1205, DOI 10.1177/1071100718778592
   Olsson N, 2014, SCAND J MED SCI SPOR, V24, P152, DOI 10.1111/j.1600-0838.2012.01497.x
   Schweitzer ME, 2000, AM J ROENTGENOL, V175, P613, DOI 10.2214/ajr.175.3.1750613
   Shalabi A, 2004, ACTA RADIOL, V45, P1, DOI 10.1080/03655950410009742
   Tallon C, 2001, MED SCI SPORT EXER, V33, P1983, DOI 10.1097/00005768-200112000-00002
NR 16
TC 3
Z9 3
U1 1
U2 3
PU SAGE PUBLICATIONS INC
PI THOUSAND OAKS
PA 2455 TELLER RD, THOUSAND OAKS, CA 91320 USA
SN 1071-1007
EI 1944-7876
J9 FOOT ANKLE INT
JI Foot Ankle Int.
PD AUG
PY 2019
VL 40
IS 8
BP 895
EP 899
DI 10.1177/1071100719845016
PG 5
WC Orthopedics
WE Science Citation Index Expanded (SCI-EXPANDED)
SC Orthopedics
GA IN3SO
UT WOS:000478596800004
PM 31006268
DA 2022-07-22
ER

PT J
AU Giannini, S
   Ceccarelli, F
AF Giannini, S
   Ceccarelli, F
TI Achilles tendon injuries in footballers
SO JOURNAL OF SPORTS TRAUMATOLOGY AND RELATED RESEARCH
LA English
DT Article; Proceedings Paper
CT 9th National Congress of the Italian-Sports-Tramatology-Society
CY OCT   16, 1999
CL TURIN, ITALY
SP Italian Sports Tramatol Soc
DE Achilles tendon; tendinopathies; sport; football
ID NON-SURGICAL TREATMENT; RUPTURES; REPAIR
AB Achilles tendon disorders in football are frequent in Italy owing to the large number of players and the features of the specific and basic movements required, the type of ground and the type of footwear. The cause, origin and treatment of such disorders are the subject of conflicting opinions. This paper reviews the relevant literature and illustrates personal preferences in the treatment of Achilles tendon disorders and subcutaneous ruptures.
C1 Univ Bologna, Ist Ortoped Rizzoli, Clin Ortoped, I-40136 Bologna, Italy.
RP Giannini, S (通讯作者)，Univ Bologna, Ist Ortoped Rizzoli, Clin Ortoped, Via GC Pupilli 1, I-40136 Bologna, Italy.
OI Ceccarelli, Francesco/0000-0002-4457-0457
CR ARNER O, 1959, ACTA CHIR SCAND    S, V239, P1
   BARFRED T, 1973, ACTA ORTHOP SCAND, V152, P12
   BESKIN JL, 1987, AM J SPORT MED, V15, P1, DOI 10.1177/036354658701500101
   BIEDERT R, 1991, UNFALLCHIRURG, V94, P531
   BONNEVIALLE P, 1992, LESIONS TRAUMATIQUES, P114
   CARDEN DG, 1987, J BONE JOINT SURG BR, V69, P416, DOI 10.1302/0301-620X.69B3.3294839
   Crenshaw A.H., 1992, CAMPBELLS OPERATIVE
   FREY C, 1994, FOOT ANKLE MASTER TE, P341
   GIANNINI S, 1994, FOOT ANKLE INT, V15, P372
   GIANNINI S, 1986, CHIR PIEDE, V10, P273
   GIANNINI S, 1983, CHIR PIEDE, V7, P421
   HAYASHI M, 1994, OPERATIVE FOOT SURG, P515
   INGLIS AE, 1976, J BONE JOINT SURG AM, V58, P990, DOI 10.2106/00004623-197658070-00015
   JOZSA L, 1989, AM J SPORT MED, V17, P338, DOI 10.1177/036354658901700305
   KANNUS P, 1991, J BONE JOINT SURG AM, V73A, P1507, DOI 10.2106/00004623-199173100-00009
   Kannus P, 1997, SCAND J MED SCI SPOR, V7, P107
   KOUVALCHOUK JF, 1990, REV CHIR ORTHOP  S1, V76, P147
   LANZETTA, 1981, J SPORTS TRAUMATOL, V3, P113
   MA GWC, 1976, CLIN ORTHOPAEDICS, V128, P247
   NISTOR L, 1981, J BONE JOINT SURG AM, V63, P394, DOI 10.2106/00004623-198163030-00012
   OBRIEN T, 1984, J BONE JOINT SURG AM, V66A, P1099, DOI 10.2106/00004623-198466070-00018
   ODEN RR, 1987, CLIN ORTHOP RELAT R, P63
   PERUGIA L, 1981, MANNON MILANO, P117
   Puddu G, 1976, Am J Sports Med, V4, P145, DOI 10.1177/036354657600400404
   ROOKS MD, 1994, OPERATIVE FOOT SURG, P515
   TESTUT L, 1959, TORINO, V2, P418
   THERMANN H, 1995, UNFALLCHIRURG, V98, P21
   THOMPSON T C, 1962, J Trauma, V2, P126, DOI 10.1097/00005373-196203000-00003
   WAPNER KL, 1994, FOOT ANKLE MASTER TE, P299
NR 29
TC 2
Z9 2
U1 0
U2 5
PU EDITRICE KURTIS S R L
PI MILAN
PA VIA LUIGI ZOJA 30, 20153 MILAN, ITALY
SN 1120-3137
J9 J SPORT TRAUMATOL
JI J. Sports Traumatol. Relat. Res.
PD MAR
PY 2000
VL 22
IS 1
BP 24
EP 34
PG 11
WC Sport Sciences
WE Conference Proceedings Citation Index - Science (CPCI-S); Science Citation Index Expanded (SCI-EXPANDED)
SC Sport Sciences
GA 367FN
UT WOS:000090051200005
DA 2022-07-22
ER

PT J
AU Hollawell, S
   Baione, W
AF Hollawell, Shane
   Baione, William
TI Chronic Achilles Tendon Rupture Reconstructed With Achilles Tendon
   Allograft and Xenograft Combination
SO JOURNAL OF FOOT & ANKLE SURGERY
LA English
DT Article
DE allograft; ankle; biotransplantation; injury; tendon repair; xenograft
ID AUGMENTATION; REPAIR; MANAGEMENT
AB More than 20% of acute Achilles tendon injuries are misdiagnosed, leading to chronic or neglected ruptures. Some controversy exists regarding how to best manage an acute Achilles tendon rupture. However, a general consensus has been reached that chronic rupture with >= 3 cm of separation is associated with functional morbidity and, therefore, should be managed operatively. It has been demonstrated that the functional outcomes of surgically treated Achilles ruptures are superior to the nonoperative outcomes in a chronic setting. In the present report, we reviewed 4 patients with chronic Achilles tendon ruptures that were successfully treated with an Achilles tendon interposition allograft and simultaneous augmentation with a xenograft. The median duration of rupture was 11 (range 8 to 16) weeks, the median gap between the proximal and distal segments of the tendon was 4.75 (range 3.5 to 6) cm, and the patients were able to return pain-free to all preinjury activities at a median of 14.5 (range 13.8 to 15.5) weeks, without the need for tendon transfer, lengthening, or additional intervention. The median duration of follow up was 37.25 (range 15.25 to 51.5) months, at which point the mean Foot and Ankle Outcomes Instrument core scale score was 97 +/- 1 (mean normative score 53 +/- 1), and the Foot and Ankle Outcomes Instrument shoe comfort core scale score was 100 +/- 0 (mean normative score 59 +/- 0). The combined Achilles allograft plus xenograft augmentation technique appears to be a reasonable option for the surgical treatment of chronic Achilles tendon rupture. (C) 2015 by the American College of Foot and Ankle Surgeons. All rights reserved.
C1 [Hollawell, Shane] Rutgers Robert Wood Johnson Med Sch, New Brunswick, NJ 08750 USA.
   [Hollawell, Shane] Orthoped Inst Cent Jersey, Manasquan, NJ USA.
   [Baione, William] Rutgers Robert Wood Johnson Med Sch, New Brunswick, NJ 08750 USA.
RP Hollawell, S (通讯作者)，Rutgers Robert Wood Johnson Med Sch, Orthoped Inst Cent Jersey, New Brunswick, NJ 08750 USA.
EM shollawell@hotmail.com
CR American Academy of Orthopedic Surgeons, 2005, FOOT ANKL OUTC INSTR
   ASSELMEIER MA, 1993, AM J SPORT MED, V21, P170, DOI 10.1177/036354659302100202
   Barber FA, 2008, FOOT ANKLE INT, V29, P329, DOI 10.3113/FAI.2008.0329
   Barber FA, 2010, ARTHROSCOPY, V26, P488, DOI 10.1016/j.arthro.2009.08.022
   Berlet GC, 2014, J FOOT ANKLE SURG, V53, P298, DOI 10.1053/j.jfas.2014.02.001
   BUCK BE, 1989, CLIN ORTHOP RELAT R, P129
   Chen JM, 2009, EXPERT REV MED DEVIC, V6, P61, DOI 10.1586/17434440.6.1.61
   DODDS WN, 1984, INJURY, V16, P94, DOI 10.1016/S0020-1383(84)80006-6
   Ibrahim SAR, 2009, J FOOT ANKLE SURG, V48, P340, DOI 10.1053/j.jfas.2009.02.007
   Kennedy J C, 1976, Am J Sports Med, V4, P11, DOI 10.1177/036354657600400103
   Kummer FJ, 2005, J BIOMED MATER RES B, V74B, P789, DOI 10.1002/jbm.b.30296
   Labib S, 2007, FOOT ANKLE INT, V28, P478, DOI 10.3113/FAI.2007.0478
   Lee DK, 2008, J FOOT ANKLE SURG, V47, P8, DOI 10.1053/j.jfas.2007.08.015
   Lee Daniel K, 2007, J Foot Ankle Surg, V46, P451, DOI 10.1053/j.jfas.2007.05.007
   Lepow Gary M, 2006, J Foot Ankle Surg, V45, P351, DOI 10.1053/j.jfas.2006.06.004
   Maffulli N, 2008, J BONE JOINT SURG AM, V90A, P1348, DOI 10.2106/JBJS.G.01241
   Magnussen RA, 2011, AM J SPORT MED, V39, P1522, DOI 10.1177/0363546510397815
   Molloy A, 2009, FOOT ANKLE CLIN, V14, P745, DOI 10.1016/j.fcl.2009.07.004
   Myerson M S, 1999, Instr Course Lect, V48, P219
   Nellas Z J, 1996, J Foot Ankle Surg, V35, P144
   O'Brien M, CLIN SPORTS MED
   Popovic N, 1999, Acta Orthop Belg, V65, P458
   ROYER RJ, 1994, THERAPIE, V49, P75
   SCHMIDTROHLFING B, 1992, INT ORTHOP, V16, P29
   Weber M, 2003, AM J SPORT MED, V31, P685, DOI 10.1177/03635465030310050901
   Wong J, 2002, AM J SPORT MED, V30, P565, DOI 10.1177/03635465020300041701
NR 26
TC 17
Z9 19
U1 1
U2 5
PU ELSEVIER SCIENCE INC
PI NEW YORK
PA STE 800, 230 PARK AVE, NEW YORK, NY 10169 USA
SN 1067-2516
EI 1542-2224
J9 J FOOT ANKLE SURG
JI J. Foot Ankle Surg.
PD NOV-DEC
PY 2015
VL 54
IS 6
BP 1146
EP 1150
DI 10.1053/j.jfas.2014.09.006
PG 5
WC Orthopedics; Surgery
WE Science Citation Index Expanded (SCI-EXPANDED)
SC Orthopedics; Surgery
GA CU8WX
UT WOS:000363825900030
PM 25488191
DA 2022-07-22
ER

PT J
AU Liu, W
   Zhuang, HY
   Shao, DZ
   Wang, LL
   Shi, M
AF Liu, Wen
   Zhuang, Haiying
   Shao, Dazhang
   Wang, Liangliang
   Shi, Miao
TI High-Frequency Color Doppler Ultrasound in Diagnosis, Treatment, and
   Rehabilitation of Achilles Tendon Injury
SO MEDICAL SCIENCE MONITOR
LA English
DT Article
DE Diagnosis; Rehabilitation; Ultrasonography; Doppler
ID RUPTURE; SONOGRAPHY
AB Background: This study evaluated the clinical application of high-frequency color Doppler ultrasonography (HFCDU) in the diagnosis, treatment, and rehabilitation of Achilles tendon injury.
   Material/Methods: A total of 68 patients with suspected Achilles tendon injury were examined by HFCDU. There were 42 Achilles tendon injury patients who underwent surgery, and they were randomly divided into a routine treatment group (n= 21) and a rehabilitation group (n= 21). HFCDU was performed at weeks 1, 3, 6, and 9, and months 3, 6, 9, 12, and 24 after the operation. The thickness of the injured Achilles tendon, echo, blood flow, and tissue adhesion were compared to those of the uninjured side.
   Results: Of the 68 patients, 14 had normal ultrasound presentation with strong echo; 7 had laceration, presenting as swelling and decreased echo; 26 had partial tear, presenting as discontinued rupture with no or low echo; 19 had complete rupture, presenting as discontinued fibers with hypoechoic hemorrhage and hyperechoic fat tissue between ends; 1 had plantar tendon rupture and periapical hemorrhage; and 1 had acute Achilles tendonitis. The postoperative tendon thickness decreased over time, echo turned homogeneous, blood flow and adhesions decreased, and these results were consistent with clinical symptoms. Patient condition and ultrasound recovery were better in the rehabilitation group, and the diameter of the injured tendon increased compared with the uninjured side.
   Conclusions: HFCDU can determine the types and extent of tendon injuries, and can help in the diagnosis, treatment, and rehabilitation of Achilles tendon injury.
C1 [Liu, Wen] Shandong Med Imaging Res Inst, Dept Ultrasound, Jinan, Shandong, Peoples R China.
   [Zhuang, Haiying] Liaocheng Peoples Hosp, Dept Rehabil, Liaocheng, Shandong, Peoples R China.
   [Shao, Dazhang; Wang, Liangliang; Shi, Miao] Liaocheng Peoples Hosp, Dept Ultrasound, Liaocheng, Shandong, Peoples R China.
RP Shi, M (通讯作者)，Liaocheng Peoples Hosp, Dept Ultrasound, Liaocheng, Shandong, Peoples R China.
EM shimiaoemail@163.com
CR Badalihan A, 2015, J FOOT ANKLE SURG, V54, P153, DOI 10.1053/j.jfas.2014.12.002
   Bleakney RR, 2002, CLIN J SPORT MED, V12, P273, DOI 10.1097/00042752-200209000-00003
   Donaldson Peter R, 2012, Clin J Sport Med, V22, P169, DOI 10.1097/JSM.0b013e31824c2b2c
   Esenyel CZ, 2014, J AM PODIAT MED ASSN, V104, P434, DOI 10.7547/0003-0538-104.5.434
   KAINBERGER FM, 1990, AM J ROENTGENOL, V155, P1031, DOI 10.2214/ajr.155.5.2120931
   Khoury V, 2007, AM J ROENTGENOL, V188, pW63, DOI 10.2214/AJR.06.0579
   KUWADA G T, 1990, Journal of Foot Surgery, V29, P361
   Lo IKY, 1997, CLIN J SPORT MED, V7, P207, DOI 10.1097/00042752-199707000-00010
   Maffulli N, 1998, AM J SPORT MED, V26, P266, DOI 10.1177/03635465980260021801
   Maquirriain Javier, 2011, Yale Journal of Biology and Medicine, V84, P289
   Martinelli B, 2000, Bull Hosp Jt Dis, V59, P149
   Popovic N, 1999, Acta Orthop Belg, V65, P458
   Tay D, 2010, ANN ACAD MED SINGAP, V39, P58
   Wong J, 2002, AM J SPORT MED, V30, P565, DOI 10.1177/03635465020300041701
   Yinger Kent, 2002, Clin Podiatr Med Surg, V19, P231, DOI 10.1016/S0891-8422(02)00002-2
   Zhang QG, 2002, ZHONG GUO XIU FU CHO, V16, P365
NR 16
TC 4
Z9 7
U1 0
U2 1
PU INT SCIENTIFIC LITERATURE, INC
PI SMITHTOWN
PA 361 FOREST LANE, SMITHTOWN, NY 11787 USA
SN 1643-3750
J9 MED SCI MONITOR
JI Med. Sci. Monitor
PD DEC 4
PY 2017
VL 23
BP 5752
EP 5759
DI 10.12659/MSM.904186
PG 8
WC Medicine, Research & Experimental
WE Science Citation Index Expanded (SCI-EXPANDED)
SC Research & Experimental Medicine
GA FO9HF
UT WOS:000417197900001
PM 29199267
OA Green Published, Green Submitted
DA 2022-07-22
ER

PT J
AU Ando, W
   Sakai, T
   Kudawara, I
   Ieguchi, M
   Miyamoto, T
   Ohzono, K
AF Ando, W
   Sakai, T
   Kudawara, I
   Ieguchi, M
   Miyamoto, T
   Ohzono, K
TI Bilateral Achilles, tendon ruptures in a patient with ochronosis
SO CLINICAL ORTHOPAEDICS AND RELATED RESEARCH
LA English
DT Article
ID ARTHROPATHY
AB We report the case of a 67-year-old man with ochronosis who had bilateral Achilles tendon ruptures. We reconstructed the Achilles tendon using pull-out wiring for the right side and an anchoring system for the left side, and reinforced the repair site using the peroneus brevis tendon for both sides. He could walk without a cane at 3 months postoperatively. Tendon ruptures in patients with ochronosis should be treated as pathologic ruptures because histologic examination reveals that both ends of the ruptured tendon and the insertion site at the calcaneus have extensive black pigment depositions where homogentisic acid and its metabolites have accumulated, and there are no normal collagen bundles present. Even if an Achilles tendon rupture is clinically diagnosed as an acute injury, the ruptured Achilles tendon should be primarily repaired and reinforced with autologous tissue because there are a few viable cells at the ruptured site, and because the tendon ruptures mainly at the insertion site of the calcaneus. Although this is a preliminary report, the short-term result is good and the reconstructed sites have showed no rerupture.
C1 Osaka Natl Hosp, Dept Orthopaed Surg, Chuo Ku, Osaka 5400006, Japan.
RP Sakai, T (通讯作者)，Osaka Natl Hosp, Dept Orthopaed Surg, Chuo Ku, 2-1-14,Hoenzaka, Osaka 5400006, Japan.
EM tsakai-osk@umin.ac.jp
CR Aynaci O, 2000, CLIN RHEUMATOL, V19, P150, DOI 10.1007/s100670050035
   Hamdi N, 1999, INT ORTHOP, V23, P122, DOI 10.1007/s002640050325
   Jarvinen M, 1997, SCAND J MED SCI SPOR, V7, P86
   MACKENZIE CR, 1982, J RHEUMATOL, V9, P634
   Nas K, 2002, CLIN RHEUMATOL, V21, P170, DOI 10.1007/s10067-002-8277-4
   Phornphutkul C, 2002, NEW ENGL J MED, V347, P2111, DOI 10.1056/NEJMoa021736
   SERADGE H, 1981, J HAND SURG-AM, V6, P359, DOI 10.1016/S0363-5023(81)80042-1
   WHITE RK, 1959, SURG GYNECOL OBSTET, V108, P117
NR 8
TC 5
Z9 6
U1 0
U2 0
PU LIPPINCOTT WILLIAMS & WILKINS
PI PHILADELPHIA
PA TWO COMMERCE SQ, 2001 MARKET ST, PHILADELPHIA, PA 19103 USA
SN 0009-921X
EI 1528-1132
J9 CLIN ORTHOP RELAT R
JI Clin. Orthop. Rel. Res.
PD JUL
PY 2004
IS 424
BP 180
EP 182
DI 10.1097/01.blo.0000128288.36202.e4
PG 3
WC Orthopedics; Surgery
WE Science Citation Index Expanded (SCI-EXPANDED)
SC Orthopedics; Surgery
GA 836FP
UT WOS:000222541400027
PM 15241162
DA 2022-07-22
ER

PT J
AU Williamson, PM
   Pennings, JPH
   Harlow, E
   Hanna, P
   Lechtig, A
   Okajima, S
   Biggane, P
   Nasr, M
   Zurakowski, D
   Duggal, N
   Nazarian, A
AF Williamson, Patrick M.
   Pennings, Jan P. H.
   Harlow, Ethan
   Hanna, Philip
   Lechtig, Aron
   Okajima, Stephen
   Biggane, Peter
   Nasr, Michael
   Zurakowski, David
   Duggal, Naven
   Nazarian, Ara
TI Tendon lengthening after achilles tendon rupture-passive effects on the
   ankle joint in a cadaveric pilot study simulating weight bearing
SO INJURY-INTERNATIONAL JOURNAL OF THE CARE OF THE INJURED
LA English
DT Article
DE Tendon lengthening; Achilles tendon; Cadaver; Cadaveric; Lower leg;
   Kinetics
ID OF-THE-LITERATURE; NONSURGICAL TREATMENT; NONOPERATIVE TREATMENT;
   PRESSURE; REPAIR; SEPARATION; COLLAGEN; INJURY; COMMON; ENDS
AB Background: In recent years, primary Achilles tendon ruptures have increased due to the aging population's participation in physically demanding activities. These injuries commonly occur during recreational sports and frequently lead to a long-term reduction in activity despite treatment. Non-operative methods of treatment for Achilles tendon ruptures may result in the Achilles healing in a lengthened position compared to the pre-injury state. This study uses a cadaveric model that simulates static weight bearing to explore the effect of a lengthened Achilles tendon on ankle joint load distribution.
   Methods: Five lower limb cadaveric specimens were placed on a custom jig, where a 334N (75 lb) load was applied at the femoral head, and the foot was supported against a plate to simulate static double-leg stance. A pressure mapping sensor was inserted into the ankle joint. A percutaneous triple hemiresection tendo-Achilles lengthening procedure (Hoke procedure) was performed on each specimen to simulate tendon lengthening after conservative treatment. Contact pressure, peak pressure, and center-of-pressure were measured for native and tendon-lengthened conditions.
   Results: Tendon rupture did not significantly alter average contact pressure, peak contact pressures, or center-of-pressure in the ankle joint compared with native tendon.
   Conclusion: Achilles lengthening does not significantly change contact pressures of the ankle joint in this model . This result suggests that the passive restraint on ankle joint translation imposed by the Achilles tendon is minimal without muscle activation. (C) 2019 Elsevier Ltd. All rights reserved.
C1 [Williamson, Patrick M.] Boston Univ, Mech Engn Dept, Boston, MA 02215 USA.
   [Williamson, Patrick M.; Pennings, Jan P. H.; Hanna, Philip; Lechtig, Aron; Okajima, Stephen; Biggane, Peter; Nasr, Michael; Nazarian, Ara] Harvard Med Sch, Ctr Adv Orthopaed Studies, Beth Israel Deaconess Med Ctr, 99 Brookline Ave,Off RN-123a, Boston, MA 02115 USA.
   [Harlow, Ethan] Univ Hosp Cleveland, Med Ctr, Dept Orthoped Surg, Cleveland, OH 44106 USA.
   [Duggal, Naven] Syracuse Orthopaed Specialists, Foot & Ankle Div, Dept Gen Orthoped & Trauma, Syracuse, NY USA.
   [Zurakowski, David] Harvard Med Sch, Boston Childrens Hosp, Dept Anesthesiol Crit Care & Pain Med, Boston, MA 02115 USA.
   [Nazarian, Ara] Yerevan State Med Univ, Dept Orthopaed Surg, Yerevan, Armenia.
RP Nazarian, A (通讯作者)，Harvard Med Sch, Ctr Adv Orthopaed Studies, Beth Israel Deaconess Med Ctr, 99 Brookline Ave,Off RN-123a, Boston, MA 02115 USA.
EM anazaria@bidmc.harvard.edu
FU American Orthopaedic Foot Ankle Society; Orthopaedic Foot & Ankle
   Foundation
FX The authors disclosed receipt of the following financial support for
   research, authorship, and/or publication of this article: Support by a
   grant from the American Orthopaedic Foot & Ankle Society with funding
   from the Orthopaedic Foot & Ankle Foundation.
CR Amlang MH, 2010, UNFALLCHIRURG, V113, P712, DOI 10.1007/s00113-010-1809-5
   Anderson DD, 2006, BIOMECH MODEL MECHAN, V5, P82, DOI 10.1007/s10237-006-0025-2
   Ballas MT, 1998, AM FAM PHYSICIAN, V57, P267
   Braunstein M, 2017, UNFALLCHIRURG, V120, P1007, DOI 10.1007/s00113-017-0420-4
   Brorsson A, 2017, AM J SPORT MED, V45, P3060, DOI 10.1177/0363546517717698
   Chen L, 2017, J FOOT ANKLE SURG, V56, P271, DOI 10.1053/j.jfas.2016.11.007
   Davitt JS, 2001, FOOT ANKLE INT, V22, P885, DOI 10.1177/107110070102201105
   Dayton P, 2017, CLIN PODIATR MED SUR, V34, P107, DOI 10.1016/j.cpm.2016.10.002
   Deng SL, 2017, J FOOT ANKLE SURG, V56, P1236, DOI 10.1053/j.jfas.2017.05.036
   Egger AC, 2017, CURR REV MUSCULOSKE, V10, P72, DOI 10.1007/s12178-017-9386-7
   Firth GB, 2013, J BONE JOINT SURG AM, V95A, P1489, DOI 10.2106/JBJS.K.01638
   Fulton J, 2014, INT J SPORTS PHYS TH, V9, P583
   FYFE I, 1992, CLIN SPORT MED, V11, P601
   HANG DW, 1995, PHYSICIAN SPORTSMED, V23, P57, DOI 10.1080/00913847.1995.11947748
   Heikkinen J, 2017, AM J SPORT MED, V45, P1395, DOI 10.1177/0363546517694610
   Hess Gregory William, 2010, Foot Ankle Spec, V3, P29, DOI 10.1177/1938640009355191
   Hoefnagels EM, 2007, ACTA ORTHOP, V78, P808, DOI 10.1080/17453670710014590
   Hufner T, 2010, UNFALLCHIRURG, V113, P699, DOI 10.1007/s00113-010-1833-5
   Huttunen TT, 2014, AM J SPORT MED, V42, P2419, DOI 10.1177/0363546514540599
   JOZSA L, 1989, AM J SPORT MED, V17, P338, DOI 10.1177/036354658901700305
   Kangas J, 2007, AM J SPORT MED, V35, P59, DOI 10.1177/0363546506293255
   Keating JF, 2011, J BONE JOINT SURG BR, V93B, P1071, DOI 10.1302/0301-620X.93B8.25998
   Killian ML, 2012, J SHOULDER ELB SURG, V21, P228, DOI 10.1016/j.jse.2011.11.002
   KIMIZUKA M, 1980, ARCH ORTHOP TRAUM SU, V96, P45, DOI 10.1007/BF01246141
   Krause F, 2007, J BONE JOINT SURG BR, V89B, P1660, DOI 10.1302/0301-620X.89B12.19849
   Krause FG, 2010, FOOT ANKLE INT, V31, P741, DOI 10.3113/FAI.2010.0741
   KVIST M, 1994, SPORTS MED, V18, P173, DOI 10.2165/00007256-199418030-00004
   Lantto I, 2016, AM J SPORT MED, V44, P2406, DOI 10.1177/0363546516651060
   LEA RB, 1972, J BONE JOINT SURG AM, VA 54, P1398, DOI 10.2106/00004623-197254070-00002
   Leppilahti J, 2000, FOOT ANKLE INT, V21, P330, DOI 10.1177/107110070002100410
   Lill H, 1996, CHIRURG, V67, P1160, DOI 10.1007/s001040050119
   LIU SH, 1995, CLIN ORTHOP RELAT R, P265
   Mazzone MF, 2002, AM FAM PHYSICIAN, V65, P1805
   MORTENSEN NHM, 1992, ORTHOPEDICS, V15, P899
   Nilsson-Helander K, 2010, AM J SPORT MED, V38, P2186, DOI 10.1177/0363546510376052
   NYSTROM B, 1983, ACTA ORTHOP SCAND, V54, P620, DOI 10.3109/17453678308992899
   Olsson N, 2013, AM J SPORT MED, V41, P2867, DOI 10.1177/0363546513503282
   Olsson N, 2011, KNEE SURG SPORT TR A, V19, P1385, DOI 10.1007/s00167-011-1511-3
   Pagenstert G, 2010, ORTHOPADE, V39, P1135, DOI 10.1007/s00132-010-1691-4
   Phillips S, 2018, FOOT ANKLE INT, V39, P500, DOI 10.1177/1071100717745559
   Salamon ML, 2006, FOOT ANKLE INT, V27, P411, DOI 10.1177/107110070602700604
   Sasaki N, 1996, J BIOMECH, V29, P655, DOI 10.1016/0021-9290(95)00110-7
   Schepull T, 2007, BMC MUSCULOSKEL DIS, V8, DOI 10.1186/1471-2474-8-116
   Suckel A, 2010, KNEE SURG SPORT TR A, V18, P664, DOI 10.1007/s00167-009-1040-5
   Waterston SW, 1997, BRIT J SPORT MED, V31, P285, DOI 10.1136/bjsm.31.4.285
   Williamson P, 2018, FOOT ANKLE INT
   Willits K, 2010, J BONE JOINT SURG AM, V92A, P2767, DOI 10.2106/JBJS.I.01401
   WILLS CA, 1986, CLIN ORTHOP RELAT R, P156
   Willy RW, 2017, AM J SPORT MED, V45, P1124, DOI 10.1177/0363546516685055
   Younger ASE, 2005, J AM ACAD ORTHOP SUR, V13, P302, DOI 10.5435/00124635-200509000-00004
NR 50
TC 1
Z9 1
U1 1
U2 2
PU ELSEVIER SCI LTD
PI OXFORD
PA THE BOULEVARD, LANGFORD LANE, KIDLINGTON, OXFORD OX5 1GB, OXON, ENGLAND
SN 0020-1383
EI 1879-0267
J9 INJURY
JI Injury-Int. J. Care Inj.
PD FEB
PY 2020
VL 51
IS 2
BP 532
EP 536
DI 10.1016/j.injury.2019.10.024
PG 5
WC Critical Care Medicine; Emergency Medicine; Orthopedics; Surgery
WE Science Citation Index Expanded (SCI-EXPANDED)
SC General & Internal Medicine; Emergency Medicine; Orthopedics; Surgery
GA KH4KX
UT WOS:000510616700065
PM 31685206
DA 2022-07-22
ER

PT J
AU Zappia, M
   Berritto, D
   Oliva, F
   Maffulli, N
AF Zappia, Marcello
   Berritto, Daniela
   Oliva, Francesco
   Maffulli, Nicola
TI High resolution real time ultrasonography of the sural nerve after
   percutaneous repair of the Achilles tendon
SO FOOT AND ANKLE SURGERY
LA English
DT Article
DE Ultrasonography; Sural nerve; Achilles tendon; Orthopedics; Surgery
ID MINIMALLY INVASIVE SURGERY; ANATOMY; RUPTURE; COMPLICATIONS; INJURIES;
   MODEL
AB Background: Percutaneous Achilles tendon repair has been developed to minimise soft tissue complications following treatment of tendon ruptures. However, there are concerns because of the risk of sural nerve injury. Few studies have investigated the relationship between the Achilles tendon, the sural nerve and its several anatomical course variants.
   Methods: We studied 7 cadaveric limbs (7 Achilles tendons) in which a percutaneous repair of the Achilles tendon was performed. On each tendon, high resolution real time ultrasonography examination was performed by an experienced musculoskeletal radiologist before and after the procedure, with the surgeons blind to the results of the scan both before and after surgery.
   Results: In two instances, high resolution real time ultrasonography examination revealed nerve entrapment at the level of most proximal lateral suture.
   Conclusions: Since the sural nerve can be easily visualised using high-frequency high resolution real time ultrasonography, intraoperative ultrasound can be of assistance during percutaneous repair of Achilles tendon rupture. (C) 2017 European Foot and Ankle Society. Published by Elsevier Ltd. All rights reserved.
C1 [Zappia, Marcello] Univ Molise, Dipartimento Med & Sci Salute, Via De Sanctis 1, I-86100 Campobasso, Italy.
   [Berritto, Daniela] Private Hosp Villa Fiori Srl Accredited Natl Hlth, Dept Radiol, Acerra, NA, Italy.
   [Oliva, Francesco] Univ Roma Tor Vergata, Dept Orthopaed & Traumatol, Rome, Italy.
   [Maffulli, Nicola] Univ Salerno, Azienda Osped San Giovanni Dio & Ruggi Aragona, Dept Orthopaed & Traumatol, Fisciano, Italy.
   [Maffulli, Nicola] Queen Mary Univ London, Barts & London Sch Med & Dent, Ctr Sports & Exercise Med, Mile End Hosp, London, England.
RP Berritto, D (通讯作者)，Corso Italia 157-80011, Acerra, NA, Italy.
EM berritto.daniela@gmail.com
RI Maffulli, Nicola/A-1839-2014; Oliva, Francesco/AAC-2030-2019
OI Maffulli, Nicola/0000-0002-5327-3702; Oliva,
   Francesco/0000-0002-7473-0271
CR Apaydin N, 2009, SURG RADIOL ANAT, V31, P775, DOI 10.1007/s00276-009-0520-0
   Blackmon JA, 2013, J FOOT ANKLE SURG, V52, P42, DOI 10.1053/j.jfas.2012.09.010
   Carmont MR, 2013, KNEE SURG SPORT TR A, V21, P1361, DOI 10.1007/s00167-013-2423-1
   Carmont M R, 2011, Foot Ankle Surg, V17, P186, DOI 10.1016/j.fas.2010.06.003
   Carmont MR, 2008, KNEE SURG SPORT TR A, V16, P199, DOI 10.1007/s00167-007-0411-z
   CETTI R, 1993, AM J SPORT MED, V21, P791, DOI 10.1177/036354659302100606
   Chen H, 2015, J ORTHOP SURG RES, V10, DOI 10.1186/s13018-015-0310-9
   Citak M, 2007, BRIT J SPORT MED, V41, P456, DOI 10.1136/bjsm.2006.031328
   Cretnik A, 2005, AM J SPORT MED, V33, P1369, DOI 10.1177/0363546504271501
   Ebinesan AD, 2008, DISABIL REHABIL, V30, P1721, DOI 10.1080/09638280701786815
   Flavin R, 2007, INJURY, V38, P845, DOI 10.1016/j.injury.2006.10.016
   Freedman Benjamin R, 2014, Muscles Ligaments Tendons J, V4, P245
   Galletti S, 2015, MLTJ-MUSCLE LIGAMENT, V5, P325, DOI 10.11138/mltj/2015.5.4.325
   Giannetti S, 2014, ORTHOPEDICS, V37, P820, DOI 10.3928/01477447-20141124-04
   Henriquez H, 2012, CLIN ORTHOP RELAT R, V470, P998, DOI 10.1007/s11999-011-1830-1
   Hsu AR, 2015, FOOT ANKLE INT, V36, P1279, DOI 10.1177/1071100715589632
   Ikiz ZAA, 2005, FOOT ANKLE INT, V26, P560, DOI 10.1177/107110070502600712
   Jones Matthew P, 2012, J Bone Joint Surg Am, V94, pe88, DOI 10.2106/JBJS.J.01829
   Kammar H, 2014, ORTHOPEDICS, V37, pE298, DOI 10.3928/01477447-20140225-64
   KLEIN W, 1991, Chirurgia degli Organi di Movimento, V76, P223
   Lacoste S, 2014, ORTHOP TRAUMATOL-SUR, V100, P925, DOI 10.1016/j.otsr.2014.09.018
   Li QB, 2016, J ORTHOP SURG RES, V11, DOI 10.1186/s13018-016-0401-2
   Loppini Mattia, 2011, Muscles Ligaments Tendons J, V1, P134
   Ma GW, 2016, CLIN ORTHOP RELAT R, V1977, P247
   MacMahon A, 2016, FOOT ANKLE INT, V37, P636, DOI 10.1177/1071100716628915
   Maffulli N, 2010, CLIN ORTHOP RELAT R, V468, P1039, DOI 10.1007/s11999-009-0944-1
   Maffulli N, 2009, ORTHOP CLIN N AM, V40, P491, DOI 10.1016/j.ocl.2009.05.006
   Mahakkanukrauh P, 2002, CLIN ANAT, V15, P263, DOI 10.1002/ca.10016
   Majewski M, 2006, AM J SPORT MED, V34, P793, DOI 10.1177/0363546505283266
   Mavrodontidis A, 2015, ACTA ORTHOP TRAUMATO, V49, P661, DOI 10.3944/AOTT.2015.14.0196
   McClelland D, 2002, J ROY COLL SURG EDIN, V47, P613
   Paavola M, 2000, AM J SPORT MED, V28, P77, DOI 10.1177/03635465000280012501
   Parafioriti A, 2011, MLTJ-MUSCLE LIGAMENT, V1, P41
   Porter KJ, 2014, FOOT ANKLE SURG, V20, P90, DOI 10.1016/j.fas.2013.11.005
   Pyun SB, 2008, AM J PHYS MED REHAB, V87, P438, DOI 10.1097/PHM.0b013e318174e569
   Ricci S, 2010, EUR J VASC ENDOVASC, V39, P636, DOI 10.1016/j.ejvs.2009.11.024
   Sadek AF, 2015, J ORTHOP SURG RES, V10, DOI 10.1186/s13018-015-0256-y
   Vadala Antonio, 2014, Muscles Ligaments Tendons J, V4, P177
   Wang CC, 2012, FOOT ANKLE INT, V33, P582, DOI [10.3113/FAI.2012.0582, 10.3113/FAI.2012.0001]
   Webb J, 2000, FOOT ANKLE INT, V21, P475, DOI 10.1177/107110070002100604
   Wong J, 2002, AM J SPORT MED, V30, P565, DOI 10.1177/03635465020300041701
NR 41
TC 9
Z9 9
U1 0
U2 3
PU ELSEVIER SCIENCE BV
PI AMSTERDAM
PA PO BOX 211, 1000 AE AMSTERDAM, NETHERLANDS
SN 1268-7731
EI 1460-9584
J9 FOOT ANKLE SURG
JI Foot Ankle Surg.
PD AUG
PY 2018
VL 24
IS 4
BP 342
EP 346
DI 10.1016/j.fas.2017.03.006
PG 5
WC Orthopedics
WE Science Citation Index Expanded (SCI-EXPANDED)
SC Orthopedics
GA GR5RM
UT WOS:000442696300013
PM 29409243
DA 2022-07-22
ER

PT J
AU Lin, JL
AF Lin, Johnny L.
TI Tendon Transfers for Achilles Reconstruction
SO FOOT AND ANKLE CLINICS
LA English
DT Article
DE Achilles tendinosis; Chronic Achilles rupture; Tendon transfer
ID FLEXOR HALLUCIS LONGUS; GLYCERYL TRINITRATE TREATMENT; ANTERIOR TIBIAL
   TENDON; SURGICAL-TREATMENT; CALCANEUS DEFORMITY; SURAL NERVE; RUPTURE;
   TENDINOPATHY; TENDINOSIS; REPAIR
AB Tendon transfers have been used in the treatment of tendon dysfunction for many years. Their use in Achilles tendon pathology is well accepted for the treatment of neglected Achilles ruptures, advanced tendinosis, and calcaneal deformities. Although satisfactory results have been obtained for many different types of transfers for neglected ruptures and tendinosis of the Achilles tendon, the flexor hallucis longus tendon has gained the most approval because of its superior donor characteristics and good clinical results. In calcaneal deformities secondary to poliomyelitis and myelomeningocoele, the use of the tibialis anterior tendon transfer remains one of the cornerstones of a reconstructive procedure to prevent progression of deformity and improve quality of life. By understanding the basic principles of tendon transfers and choosing the appropriate donor, these difficult clinical problems can be treated with the optimal technique to ensure the best possible result.
C1 [Lin, Johnny L.] Rush Univ, Dept Orthopaed Surg, Med Ctr, Chicago, IL 60612 USA.
RP Lin, JL (通讯作者)，Rush Univ, Dept Orthopaed Surg, Med Ctr, 1725W Harrison St,Ste 1063, Chicago, IL 60612 USA.
EM jlin@rushortho.com
CR ABRAHAM E, 1975, J BONE JOINT SURG AM, VA 57, P253, DOI 10.2106/00004623-197557020-00019
   Ahmed IM, 1998, J ORTHOPAED RES, V16, P591, DOI 10.1002/jor.1100160511
   Alfredson H, 2006, KNEE SURG SPORT TR A, V14, P399, DOI 10.1007/s00167-006-0720-7
   Anagnostakos K, 2006, FOOT ANKLE INT, V27, P330, DOI 10.1177/107110070602700504
   Angermann P, 1999, FOOT ANKLE INT, V20, P304, DOI 10.1177/107110079902000507
   ARNER O, 1959, Acta Chir Scand, V116, P484
   ASTROM M, 1994, J ORTHOP RES, V12, P246, DOI 10.1002/jor.1100120214
   BLISS DG, 1986, J BONE JOINT SURG AM, V68A, P1258, DOI 10.2106/00004623-198668080-00017
   Boesen MI, 2006, BRIT J SPORT MED, V40, P761, DOI 10.1136/bjsm.2006.027334
   BROUGHTON NS, 2001, CHAPMANS ORTHOPAEDIC, P4567
   BUGG EI, 1968, CLIN ORTHOP RELAT R, P73
   CHRISTENSEN I, 1953, ACTA CHIR SCAND, V106, P50
   Costa ML, 2005, CLIN ORTHOP RELAT R, P199, DOI 10.1097/01.blo.0000180451.03425.48
   Coughlin MJ., 2007, SURG FOOT ANKLE, P1149
   Coull R, 2003, FOOT ANKLE INT, V24, P931, DOI 10.1177/107110070302401211
   Den Hartog BD, 2003, FOOT ANKLE INT, V24, P233, DOI 10.1177/107110070302400306
   Dhillon M S, 2000, Foot Ankle Clin, V5, P327
   Elias I, 2007, FOOT ANKLE INT, V28, P1238, DOI 10.3113/FAI.2007.1238
   FERNANDEZFELIBERTI R, 1992, J BONE JOINT SURG AM, V74A, P1038, DOI 10.2106/00004623-199274070-00010
   FRASER RK, 1991, J BONE JOINT SURG BR, V73, P994, DOI 10.1302/0301-620X.73B6.1955450
   FRENETTE JP, 1977, J BONE JOINT SURG AM, V59, P673, DOI 10.2106/00004623-197759050-00015
   Gallant G G, 1995, Am J Orthop (Belle Mead NJ), V24, P257
   GEORGIADIS GM, 1990, J BONE JOINT SURG AM, V72A, P392, DOI 10.2106/00004623-199072030-00011
   GREEN DP, 1999, GREENS OPERATIVE HAN, V2, P1481
   Hahn F, 2008, FOOT ANKLE INT, V29, P794, DOI 10.3113/FAI.2008.0794
   Hansen Jr ST, 1991, DISORDERS FOOT ANKLE, V3, P2355
   Haraguchi N, 2005, TECH FOOT ANKLE SURG, V4, P154, DOI 10.1097/01.btf.0000177228.45213.5d
   HAVEL PE, 1988, FOOT ANKLE, V9, P117, DOI 10.1177/107110078800900304
   Herbst SA, 2006, FOOT ANKLE INT, V27, P639, DOI 10.1177/107110070602700814
   HERNDON CH, 1956, J BONE JOINT SURG AM, V38, P751, DOI 10.2106/00004623-195638040-00004
   Ikiz ZAA, 2005, FOOT ANKLE INT, V26, P560, DOI 10.1177/107110070502600712
   LAGERGREN C, 1959, Acta Chir Scand, V116, P491
   LEITNER A, 1992, FOOT ANKLE, V13, P176, DOI 10.1177/107110079201300402
   MANN RA, 1991, J BONE JOINT SURG AM, V73A, P214, DOI 10.2106/00004623-199173020-00009
   Martin RL, 2005, FOOT ANKLE INT, V26, P691, DOI 10.1177/107110070502600905
   McGarvey WC, 2002, FOOT ANKLE INT, V23, P19, DOI 10.1177/107110070202300104
   Mulier T, 2007, FOOT ANKLE INT, V28, P910, DOI 10.3113/FAI.2007.0910
   Myerson M S, 1999, Instr Course Lect, V48, P219
   Myerson MS, 1996, ORTHOPEDICS, V19, P383
   Nellas Z J, 1996, J Foot Ankle Surg, V35, P144
   NETTER FH, 1999, ATLAS HUMAN ANATOMY, P481
   NOONAN KJ, 2006, LOVELL WINTERS PEDIA, V1, P631
   Ohberg L, 2001, SCAND J MED SCI SPOR, V11, P207, DOI 10.1034/j.1600-0838.2001.110403.x
   Paoloni JA, 2004, J BONE JOINT SURG AM, V86A, P916, DOI 10.2106/00004623-200405000-00005
   Paoloni JA, 2007, FOOT ANKLE INT, V28, P1064, DOI 10.3113/FAI.2007.1064
   Park KB, 2008, J BONE JOINT SURG AM, V90A, P2149, DOI 10.2106/JBJS.G.00729
   PARKER RG, 1979, J AM POD ASSOC, V69, P548, DOI 10.7547/87507315-69-9-548
   PEABODY CW, 1949, AM ACADEMY ORTHOPEDI, V6, P178
   PEREZTEUFFER A, 1974, ORTHOP CLIN N AM, V5, P89
   Pintore E, 2001, J TRAUMA, V50, P71, DOI 10.1097/00005373-200101000-00013
   Qu Jia-fu, 2008, Zhongguo Gu Shang, V21, P297
   Rompe JD, 2006, CLIN ORTHOP RELAT R, P276, DOI 10.1097/01.blo.0000203488.44077.ff
   Sarrafian SK, 1993, ANATOMY FOOT ANKLE D, P474
   SILVER RL, 1985, J BONE JOINT SURG BR, V67, P432, DOI 10.1302/0301-620X.67B3.3997956
   Tashjian RZ, 2003, FOOT ANKLE INT, V24, P673, DOI 10.1177/107110070302400903
   TURCO VJ, 1987, FOOT ANKLE, V7, P253, DOI 10.1177/107110078700700407
   TURNER JW, 1971, CLIN ORTHOP RELAT R, P71
   Us AK, 1997, ARCH ORTHOP TRAUM SU, V116, P408
   Wapner K L, 1993, Foot Ankle, V14, P443
   WAPNER KL, 1994, FOOT ANKLE INT, V15, P420, DOI 10.1177/107110079401500803
   Webb J, 2000, FOOT ANKLE INT, V21, P475, DOI 10.1177/107110070002100604
   WHITE RK, 1959, SURG GYNECOL OBSTET, V108, P117
   Wilcox DK, 2000, FOOT ANKLE INT, V21, P1004, DOI 10.1177/107110070002101204
   Zadek I, 1940, J BONE JOINT SURG, V22, P1070
   Zantop T, 2003, ARCH ORTHOP TRAUM SU, V123, P501, DOI 10.1007/s00402-003-0491-2
NR 65
TC 17
Z9 17
U1 0
U2 2
PU W B SAUNDERS CO-ELSEVIER INC
PI PHILADELPHIA
PA 1600 JOHN F KENNEDY BOULEVARD, STE 1800, PHILADELPHIA, PA 19103-2899 USA
SN 1083-7515
EI 1558-1934
J9 FOOT ANKLE CLIN
JI Foot Ankle Clin.
PD DEC
PY 2009
VL 14
IS 4
BP 729
EP 744
DI 10.1016/j.fcl.2009.08.007
PG 16
WC Orthopedics
WE Science Citation Index Expanded (SCI-EXPANDED)
SC Orthopedics
GA V16PP
UT WOS:000207881800012
PM 19857845
DA 2022-07-22
ER

PT J
AU Bravo-Sanchez, A
   Abian, P
   Jimenez, F
   Abian-Vicen, J
AF Bravo-Sanchez, Alfredo
   Abian, Pablo
   Jimenez, Fernando
   Abian-Vicen, Javier
TI Structural and mechanical properties of the Achilles tendon in senior
   badminton players: Operated vs. non-injured tendons
SO CLINICAL BIOMECHANICS
LA English
DT Article
DE Badminton; Elastography; Achilles tendon; Myotonometer; Ultrasound
ID REAL-TIME SONOELASTOGRAPHY; STIFFNESS; RUPTURES; REPAIR; REHABILITATION;
   EPIDEMIOLOGY; RELIABILITY
AB Background: The aim of this study was to describe the differences in structural and mechanical properties between operated and non-injured Achilles tendons in senior badminton players who had had Achilles tendon surgery and had returned to play.
   Methods: Eighteen players (age = 48.9(10.0)years), assigned to the unilateral Achilles tendon rupture group and 177 non-injured players (age = 55.4(9.4)years), assigned to the control group. A Logiq (R) S8 ultrasound was used to study tendon structure and elastography index values and a Myoton (R) PRO hand-held myotonometer was used to record the stiffness of the Achilles tendon.
   Findings: In Achilles tendon rupture group, operated tendons showed higher values than non-injured ones in thickness (Operated = 9.03(2.67)mm vs. non-injured = 5.88(0.88)mm; P < 0.001), width (Operated = 18.44 (3.20)mm vs. non-injured = 16.80(1.97)mm; P = 0.039), cross sectional area (Operated = 140.33(60.29)mm(2) vs. non-injured = 74.40(17.09)mm(2); P < 0.001) and elastography index (Operated = 2.05(1.35)A.U. vs. non-injured = 1.47(0.62)A.U.; P = 0.025). The bilateral differences shown by the Achilles tendon rupture group were greater than the bilateral differences shown by the control group for thickness (P < 0.001), width (P = 0.001), cross sectional area (P < 0.001), tone (P = 0.006) and dynamic stiffness (Achilles tendon rupture group = 10.85 (23.90)N.m(-1). vs. control group = 0.18(18.83)N.m(-1); P = 0.031).
   Interpretation: Surgery on the Achilles tendon and adaptation to the mobilisation and strength training during rehabilitation could provoke structural and mechanical differences compared to the non-injured tendon. Furthermore, the differences between both Achilles tendons in the Achilles tendon rupture group was higher than the asymmetry observed between dominant and non-dominant Achilles tendons in the control group. In addition, the higher logarithmic decrement values showed by non-injured tendons in the Achilles tendon rupture group could be a tendinous injury risk factor.
C1 [Bravo-Sanchez, Alfredo; Jimenez, Fernando; Abian-Vicen, Javier] Univ Castilla La Mancha, Fac Sport Sci, Performance & Sport Rehabil Lab, Avda Carlos 3 S-N, Toledo 45071, Spain.
   [Abian, Pablo] Comillas Pontifical Univ, Fac Humanities & Social Sci, Madrid, Spain.
RP Abian-Vicen, J (通讯作者)，Univ Castilla La Mancha, Fac Sport Sci, Performance & Sport Rehabil Lab, Avda Carlos 3 S-N, Toledo 45071, Spain.
EM javier.abian@uclm.es
RI Bravo Sánchez, Alfredo/AAZ-5835-2021; ABIAN, PABLO/AAZ-8278-2021;
   Abián-Vicén, Javier/I-8509-2012
OI ABIAN, PABLO/0000-0002-6976-0740; Abián-Vicén,
   Javier/0000-0001-9635-3289
CR Abian-Vicen J, 2013, INT J PERF ANAL SPOR, V13, P310, DOI 10.1080/24748668.2013.11868650
   Aird L, 2012, ARCH GERONTOL GERIAT, V55, pE31, DOI 10.1016/j.archger.2012.03.005
   Amin NH, 2013, AM J SPORT MED, V41, P1864, DOI 10.1177/0363546513490659
   Boesen AP, 2011, KNEE SURG SPORT TR A, V19, P30, DOI 10.1007/s00167-010-1208-z
   Couppe C, 2013, SCAND J MED SCI SPOR, V23, pe89, DOI 10.1111/sms.12023
   Cristi-Sanchez I, 2019, SPORTS HEALTH, V11, P157, DOI 10.1177/1941738118820517
   Ditroilo M, 2011, SPORTS MED, V41, P1019, DOI 10.2165/11591470-000000000-00000
   Drakonaki EE, 2009, CLIN RADIOL, V64, P1196, DOI 10.1016/j.crad.2009.08.006
   Eliasson P, 2018, AM J SPORT MED, V46, P2492, DOI 10.1177/0363546518781826
   Fahlstrom M, 2002, KNEE SURG SPORT TR A, V10, P57, DOI 10.1007/s00167-001-0255-x
   Feng YN, 2018, SCI REP-UK, V8, DOI 10.1038/s41598-018-34719-7
   Fletcher JR, 2010, EUR J APPL PHYSIOL, V110, P1037, DOI 10.1007/s00421-010-1582-8
   Gavronski G, 2007, PHYSIOL MEAS, V28, P625, DOI 10.1088/0967-3334/28/6/002
   Geremia JM, 2015, CLIN BIOMECH, V30, P485, DOI 10.1016/j.clinbiomech.2015.03.005
   Gervasi M, 2017, EUR J APPL PHYSIOL, V117, P1739, DOI 10.1007/s00421-017-3668-z
   Herbaut A, 2018, SCI SPORT, V33, P145, DOI 10.1016/j.scispo.2018.02.001
   Houshian S, 1998, INJURY, V29, P651, DOI 10.1016/S0020-1383(98)00147-8
   Huang JZ, 2015, AM J SPORT MED, V43, P1008, DOI 10.1177/0363546514531014
   Jarvinen Tero A H, 2005, Foot Ankle Clin, V10, P255, DOI 10.1016/j.fcl.2005.01.013
   Jimenez-Sanchez C, 2018, PHYSIOL MEAS, V39, DOI 10.1088/1361-6579/aae9aa
   JORGENSEN U, 1987, INT J SPORTS MED, V8, P379, DOI 10.1055/s-2008-1025689
   Kannus P, 1997, SCAND J MED SCI SPOR, V7, P67
   Klauser AS, 2013, RADIOLOGY, V267, P837, DOI 10.1148/radiol.13121936
   LERNER RM, 1990, ULTRASOUND MED BIOL, V16, P231, DOI 10.1016/0301-5629(90)90002-T
   Longo UG, 2009, AM J SPORT MED, V37, P1400, DOI 10.1177/0363546509332250
   Maffulli N, 2003, AM J SPORT MED, V31, P692, DOI 10.1177/03635465030310051001
   Morgan GE, 2018, BMJ OPEN SPORT EXERC, V4, DOI 10.1136/bmjsem-2018-000446
   Murphy AJ, 2003, PHYS THER SPORT, V4, P175, DOI 10.1016/S1466-853X(03)00077-4
   Niemelainen R, 2011, SPINE, V36, P2152, DOI 10.1097/BRS.0b013e318204b05a
   Olsson N, 2013, AM J SPORT MED, V41, P2867, DOI 10.1177/0363546513503282
   Park GY, 2011, AM J PHYS MED REHAB, V90, P875, DOI 10.1097/PHM.0b013e31821a6f8d
   Pruyn EC, 2016, J SPORT HEALTH SCI, V5, P476, DOI 10.1016/j.jshs.2015.12.001
   Rasmussen OS, 2000, SCAND J MED SCI SPOR, V10, P360, DOI 10.1034/j.1600-0838.2000.010006360.x
   Reeves J, 2015, SPORTS, V3, P77, DOI 10.3390/sports3020077
   Schneebeli A, 2020, BMJ OPEN SPORT EXERC, V6, DOI 10.1136/bmjsem-2019-000726
   Tan S, 2012, SKELETAL RADIOL, V41, P1067, DOI 10.1007/s00256-011-1339-4
   Turan A, 2013, SKELETAL RADIOL, V42, P1113, DOI 10.1007/s00256-013-1637-0
   Wang Joong-San, 2017, J Phys Ther Sci, V29, P144, DOI 10.1589/jpts.29.144
   Zellers JA, 2016, BRIT J SPORT MED, V50, P1325, DOI 10.1136/bjsports-2016-096106
NR 39
TC 3
Z9 3
U1 2
U2 3
PU ELSEVIER SCI LTD
PI OXFORD
PA THE BOULEVARD, LANGFORD LANE, KIDLINGTON, OXFORD OX5 1GB, OXON, ENGLAND
SN 0268-0033
EI 1879-1271
J9 CLIN BIOMECH
JI Clin. Biomech.
PD MAY
PY 2021
VL 85
AR 105366
DI 10.1016/j.clinbiomech.2021.105366
EA APR 2021
PG 6
WC Engineering, Biomedical; Orthopedics; Sport Sciences
WE Science Citation Index Expanded (SCI-EXPANDED)
SC Engineering; Orthopedics; Sport Sciences
GA SD4ZC
UT WOS:000651381300003
PM 33915493
OA hybrid
DA 2022-07-22
ER

PT J
AU Dal-Bo, ID
   Ferrigno, CRA
   Macedo, AS
   Paes, F
   dos Santos, JF
   Marinho, PVT
   Bregadioli, T
   Galeazzi, VS
AF Dal-Bo, Isis dos Santos
   Auada Ferrigno, Cassio Ricardo
   Macedo, Aline Schafrum
   Paes, Fernanda
   dos Santos, Jaqueline Franca
   Tertuliano Marinho, Paulo Vinicius
   Bregadioli, Thales
   Galeazzi, Viviane Sanchez
TI Tenorrhaphy of the Common Calcaneal Tendon in Dogs and Cats
SO ACTA SCIENTIAE VETERINARIAE
LA English
DT Article
DE Achilles tendon; rupture; calcaneo-tibial screw; locking-loop suture
   pattern
ID SURGICAL REPAIR; ACHILLES; IMMOBILIZATION; RUPTURE
AB Background: Common calcaneal tendon (CCT) is usually affected by injuries in dogs and less frequently in cats. The Achilles mechanism consists of the convergence of three distinct structures: the tendons of the gastrocnemius muscle, superficial digital flexor muscle and the common tendons of the biceps femoris, gracilis, and semitendinosus muscles. Classification of lesions is based upon their location, severity and chronicity. Clinical signs include lameness, plantigrade stance and various degrees of tarsal hyperflexion according to the injury's severity. The purpose of this study is to report four cases of reconstruction of the common calcaneus in three dogs and a cat.
   Cases: Case 1. A 10-year-old male canine, Brazilian Terrier, 7.7 kg of body weight, with a 6-month history of nonweight-bearing lameness of the right hind limb attended consultation. Orthopedic examination showed plantigrade stance, hyperflexion of the right tibial-tarsal joint (TTJ) and swelling with fibrous thickening of the distal portion of the CCT. Surgical tendon repair was achieved through insertion of a locking screw on the calcaneo-tibial, tenorrhaphy and suture of a titanium plate on the caudal aspect of the CCT. Case 2. A 2-year-old female mongrel canine, with 13.3 kg of body weight was presented with the complaint of chronic left hind leg lameness. The owner described trauma at the area of the hock six months prior to consultation. On orthopedic exam, severe lameness was noted, swelling with fibrous thickening of the distal portion of the common calcaneal tendon and hyperflexion of the left TTJ (plantigrade position) were visible. Tendon repair was performed in the same manner as in case 1, except that in this case a bovine pericardium graft preserved in glycerin 98% was sutured over the tendon/plate conjunction. Case 3. A 6-year-old female mongrel canine, with 18.6 kg of body weight was attended with a 22-day history of right hind limb lameness. On physical exam intermittent lameness during gait performance and non-weight-bearing position in stance were noted. The distal portion of the limb was internally rotated with plantigrade stance and discontinuity of the CCT was palpable. Tenorrhaphy was performed similarly to case 2. Case 4. A 6 kg, 3-year-old, mixed breed male feline was admitted with a wound on the CCT region for 7 days. The wound was originated after animal interaction with a stray feline. On clinical examination the animal was non-weight bearing on the right hind limb, with plantigrade posture and with a 1 cm wound on the caudal portion of the CCT with tendon exposure. Surgical repair was performed by tenorrhaphy with placement of a surgical steel plate on the caudal aspect of the CCT. A bovine pericardium graft preserved in glycerin 98% was sutured on top of the plate. The TTJ was immobilized with a type II transarticular external fixator.
   Discussion: Ultrasonography is the test of choice to monitor tendon healing after tenorrhaphy. At this report, this exam was crucial to determine when the calcaneal-tibial screw could be removed. Two dogs and the cat from this report received an association of plate and bovine pericardium preserved in glycerin 98%. The purpose of this association was to increase the strength of the tenorrhaphy, and the pericardium graft had also the function of reducing the formation of peritendinous adhesions. Immobilization of the TTJ has the purpose of opposing muscle forces to avoid stress at tenorrhaphy suture increasing the strength of the healed tendon allowing early weight bear while minimizing the recurrence risk and avoiding limb contracture. We chose to perform the procedure with hyperextension of the TTJ in order to promote greater relaxation of the tendon since there was need to remove large portions of calcaneal fibrous tissues of the dogs and devitalized tissue in the cat from this report. Surgical treatment of injuries of the Achilles mechanism has good prognosis in complete closed lesions treated by tenorrhaphy associated with suture of a plate, suture of bovine pericardial graft preserved in 98% glycerin, calcaneal-tibial locking screw and splint for six weeks.
C1 [Dal-Bo, Isis dos Santos; Auada Ferrigno, Cassio Ricardo; Macedo, Aline Schafrum; Paes, Fernanda; dos Santos, Jaqueline Franca; Tertuliano Marinho, Paulo Vinicius; Bregadioli, Thales; Galeazzi, Viviane Sanchez] Univ Sao Paulo, FMVZ, Dept Cirurgia VCI, Lab Ortopedia & Traumatol Vet LOTC, Sao Paulo, SP, Brazil.
RP Dal-Bo, ID (通讯作者)，Univ Sao Paulo, Fac Med Vet & Zootecnia, Ave Prof Dr Orlando Marques de Paiva 87, BR-05508270 Sao Paulo, SP, Brazil.
EM idb_vet@yahoo.com.br
RI Macedo, Aline/ABD-1763-2020
OI Macedo, Aline/0000-0001-6725-7028
CR Cervi M, 2010, VET COMP ORTHOPAED, V23, P348, DOI 10.3415/VCOT-09-10-0109
   Gilbert PJ, 2010, VET COMP ORTHOPAED, V23, P81, DOI 10.3415/VCOT-09-05-0057
   Guerin S., 1998, VET COMP ORTHOPAED, V11, P47
   MEUTSTEGE FJ, 1993, VET COMP ORTHOPAED, V6, P53
   Nielsen C, 2006, VET COMP ORTHOPAED, V19, P246, DOI 10.1055/s-0038-1633008
   Norton JC, 2009, VET COMP ORTHOPAED, V22, P430, DOI 10.3415/VCOT-08-08-0072
   Pratesi A, 2012, VET COMP ORTHOPAED, V25, P246, DOI 10.3415/VCOT-11-07-0099
   Raiser A. G., 2001, Ciencia Rural, V31, P89, DOI 10.1590/S0103-84782001000100014
   Raiser A. G., 2001, Ciencia Rural, V31, P351, DOI 10.1590/S0103-84782001000200027
   Schulz K, 2008, CIRURGIA PEQUENOS AN, P1316
   Sivacolundhu RK, 2001, VET COMP ORTHOPAED, V14, P25
   Swiderski J, 2005, VET COMP ORTHOPAED, V18, P258
   Worth AJ, 2004, NEW ZEAL VET J, V52, P109, DOI 10.1080/00480169.2004.36415
NR 13
TC 2
Z9 2
U1 1
U2 10
PU UNIV FED RIO GRANDE DO SUL
PI PORTO ALEGRE RS
PA FAC VET, CAIXA POSTAL 15017, PORTO ALEGRE RS, 91501-570, BRAZIL
SN 1678-0345
EI 1679-9216
J9 ACTA SCI VET
JI Acta Sci. Vet.
PY 2016
VL 44
SU 1
AR 144
PG 7
WC Veterinary Sciences
WE Science Citation Index Expanded (SCI-EXPANDED)
SC Veterinary Sciences
GA FG9KR
UT WOS:000410758300035
DA 2022-07-22
ER

PT J
AU Patil, SSD
   Patil, VSD
   Basa, VR
   Dombale, AB
AF Patil, Sampat Shivajirao Dumbre
   Patil, Vaishali Sampat Dumbre
   Basa, Vikas Rajeshwarrao
   Dombale, Ajay Birappa
TI Semitendinosus Tendon Autograft for Reconstruction of Large Defects in
   Chronic Achilles Tendon Ruptures
SO FOOT & ANKLE INTERNATIONAL
LA English
DT Article
DE Achilles tendon reconstruction; chronic Achilles rupture; semitendinosus
   tendon
ID NEGLECTED RUPTURE; REPAIR; MANAGEMENT
AB Background: Chronic Achilles tendon ruptures are associated with considerable functional morbidity. When treated operatively, debridement of degenerated tendon ends may create large defects. Various procedures to reconstruct large defects have been described. We present a simple technique in which an autologous semitendinosus tendon graft is used to reconstruct defects larger than 5 cm in chronic Achilles tendon ruptures. The purpose of this study was to describe our operative technique and its functional outcome.
   Methods: Achilles ruptures of more than 6 weeks duration were considered for the study. We treated 35 patients (20 males, 15 females) with symptomatic chronic Achilles tendon ruptures. The mean age was 47.4 years (range, 30 to 59). The smallest defect that we had reconstructed was 5 cm, and the largest was 9 cm in length. The average follow-up duration was 30.7 months (range, 20 to 42). Postoperatively, the strength of gastrocsoleus was measured by manual muscle testing (MMT) in non-weight-bearing and weight-bearing positions.
   Results: All operated patients showed satisfactory functional outcome, good soft tissue healing, and no reruptures. The preoperative weight-bearing MMT of 2/5 improved to 4/5 or 5/5 postoperatively. In all patients, postoperative non-weight-bearing MMT was 5/5. All patients returned to their prerupture daily activity.
   Conclusion: We present a technique that is simple, with low morbidity. We believe it is a valuable option especially when allografts are not available. It is inexpensive as suture anchors or tenodesis screws are not used. This can be a useful option if other tendons (flexor hallucis longus, peroneus brevis, etc) are not available for transfer.
C1 [Patil, Sampat Shivajirao Dumbre] Noble Hosp, Dept Orthopaed, Pune 411013, Maharashtra, India.
   [Patil, Vaishali Sampat Dumbre] Noble Hosp, Pune 411013, Maharashtra, India.
   [Basa, Vikas Rajeshwarrao; Dombale, Ajay Birappa] Shri Prayag Dham Trust Charitable Hosp, Pune, Maharashtra, India.
RP Patil, SSD (通讯作者)，Noble Hosp, Dept Orthopaed, 153 Magarpatta, Pune 411013, Maharashtra, India.
EM sampatdumbre@gmail.com
RI Patil, Sampat Dumbre/AAN-9116-2020
OI Patil, Sampat Dumbre/0000-0003-3656-4943
CR ABRAHAM E, 1975, J BONE JOINT SURG AM, VA 57, P253, DOI 10.2106/00004623-197557020-00019
   BOSWORTH DM, 1956, J BONE JOINT SURG AM, V38, P111, DOI 10.2106/00004623-195638010-00011
   BOYDEN EM, 1995, CLIN ORTHOP RELAT R, P150
   CARDEN DG, 1987, J BONE JOINT SURG BR, V69, P416, DOI 10.1302/0301-620X.69B3.3294839
   CHRISTENSEN I, 1953, ACTA CHIR SCAND, V106, P50
   Dalal RB, 2003, ANN ROY COLL SURG, V85, P283, DOI 10.1308/003588403766275088
   GABEL S, 1994, FOOT ANKLE INT, V15, P512, DOI 10.1177/107110079401500912
   GILLESPIE HS, 1969, J TRAUM, V9, P247, DOI 10.1097/00005373-196903000-00007
   Hansen ST, 1991, DISORDERS FOOT ANKLE, P2355
   HATTRUP SJ, 1985, FOOT ANKLE, V6, P34, DOI 10.1177/107110078500600107
   Hislop HJ, 2002, DANIELS WORTHINGHAM
   INGLIS AE, 1976, J BONE JOINT SURG AM, V58, P990, DOI 10.2106/00004623-197658070-00015
   Jennings AG, 2002, J BONE JOINT SURG BR, V84B, P361, DOI 10.1302/0301-620X.84B3.11559
   Ji JH, 2007, FOOT ANKLE INT, V28, P1100, DOI 10.3113/FAI.2007.1100
   Maffulli N, 2008, J BONE JOINT SURG AM, V90A, P1348, DOI 10.2106/JBJS.G.01241
   Maffulli N, 2008, BMC MUSCULOSKEL DIS, V9, DOI 10.1186/1471-2474-9-100
   MANN RA, 1991, J BONE JOINT SURG AM, V73A, P214, DOI 10.2106/00004623-199173020-00009
   PEREZTEUFFER A, 1974, ORTHOP CLIN N AM, V5, P89
   Puddu G, 1976, Am J Sports Med, V4, P145, DOI 10.1177/036354657600400404
   Wilcox DK, 2000, FOOT ANKLE INT, V21, P1004, DOI 10.1177/107110070002101204
NR 20
TC 23
Z9 23
U1 1
U2 4
PU SAGE PUBLICATIONS INC
PI THOUSAND OAKS
PA 2455 TELLER RD, THOUSAND OAKS, CA 91320 USA
SN 1071-1007
EI 1944-7876
J9 FOOT ANKLE INT
JI Foot Ankle Int.
PD JUL
PY 2014
VL 35
IS 7
BP 699
EP 705
DI 10.1177/1071100714531228
PG 7
WC Orthopedics
WE Science Citation Index Expanded (SCI-EXPANDED)
SC Orthopedics
GA AL7QS
UT WOS:000339330400008
PM 24722009
DA 2022-07-22
ER

PT J
AU Pufe, T
   Petersen, W
   Tillmann, B
   Mentlein, R
AF Pufe, T
   Petersen, W
   Tillmann, B
   Mentlein, R
TI The angiogenic peptide vascular endothelial growth factor is expressed
   in foetal and ruptured tendons
SO VIRCHOWS ARCHIV-AN INTERNATIONAL JOURNAL OF PATHOLOGY
LA English
DT Article
DE Achilles tendon; angiogenesis; hypoxia; vascular endothelial growth
   factor (VEGF) VEGF receptor; epidermal growth factor (EGF)
ID ACHILLES-TENDON; FACTOR GENE; VEGF; TENDINOSIS; KINASE; MODEL; CELLS
AB The Achilles tendon is one of the most common sites of injury and rupture. Evidence suggests that local vascularisation is involved in this aetiology. We investigated the expression of one important angiogenic factor, the vascular endothelial growth factor (VEGF), in normal and pathologic human Achilles tendons using immunohistochemical, biochemical, molecular and cell biology methods. VEGF could be immunostained in tenocytes of ruptured and foetal Achilles tendons, but not in normal adult ones. In microvessels, the VEGF receptor VEGFR-1 (flt-1) could be visualised as well. High VEGF levels were measured in homogenates from ruptured adult, lower ones in foetal and negligible concentrations in normal adult Achilles tendons using enzyme-linked immunoassay (ELISA) and Western blot experiments. Reverse transcriptase-polymerase chain reaction (RT-PCR) showed that the splice variants VEGF(121) and VEGF(165) are exclusively expressed. In tenocytes cultivated from newborn rat Achilles tendons, hypoxia or epidermal growth factor (EGF) raised VEGF production moderately whereas their combination resulted in a strong, synergistic induction. These results prove the presence of an angiogenic peptide and vascularisation in ruptured and foetal tendons and support the view that microtrauma or degeneration in the Achilles tendon precedes its rupture.
C1 Univ Kiel, Dept Anat, D-24098 Kiel, Germany.
RP Mentlein, R (通讯作者)，Univ Kiel, Dept Anat, Olshaussenstr 40, D-24098 Kiel, Germany.
RI Mentlein, Rolf/B-1613-2010
OI Mentlein, Rolf/0000-0002-6350-3911
CR Ahmed IM, 1998, J ORTHOPAED RES, V16, P591, DOI 10.1002/jor.1100160511
   ASTROM M, 1994, CLIN ORTHOP RELAT R, P166
   ASTROM M, 1995, CLIN ORTHOP RELAT R, P151
   BACKMAN C, 1990, J ORTHOPAED RES, V8, P541, DOI 10.1002/jor.1100080410
   BACKMAN C, 1991, ACTA ORTHOP SCAND, V62, P386, DOI 10.3109/17453679108994477
   Bidder M, 2000, J ORTHOPAED RES, V18, P247, DOI 10.1002/jor.1100180212
   Callard RE, 1994, CYTOKINE FACTS BOOK
   Carmeliet P, 1996, NATURE, V380, P435, DOI 10.1038/380435a0
   CARR AJ, 1989, J BONE JOINT SURG BR, V71, P100, DOI 10.1302/0301-620X.71B1.2914976
   Chang J, 1998, J HAND SURG-AM, V23A, P1052, DOI 10.1016/S0363-5023(98)80015-4
   Eckhart L, 1999, J BIOL CHEM, V274, P2613, DOI 10.1074/jbc.274.5.2613
   FAVA RA, 1994, J EXP MED, V180, P341, DOI 10.1084/jem.180.1.341
   FEINDT J, 1995, J NEUROCHEM, V65, P1997
   Ferrara N, 1999, J MOL MED, V77, P527, DOI 10.1007/s001099900019
   Finkenzeller G, 1997, ONCOGENE, V15, P669, DOI 10.1038/sj.onc.1201219
   FONG GH, 1995, NATURE, V376, P66, DOI 10.1038/376066a0
   Gerber HP, 1999, NAT MED, V5, P623, DOI 10.1038/9467
   Held-Feindt J, 1999, MOL BRAIN RES, V64, P101, DOI 10.1016/S0169-328X(98)00312-X
   Jarvinen M, 1997, SCAND J MED SCI SPOR, V7, P86
   Jozsa L G., 1997, HUMAN TENDONS ANATOM
   KANNUS P, 1991, J BONE JOINT SURG AM, V73A, P1507, DOI 10.2106/00004623-199173100-00009
   KOCH S, 1995, ANN ANAT, V177, P467, DOI 10.1016/S0940-9602(11)80155-X
   LANG J, 1963, ACTA ANAT, V54, P273
   LANG J, 1960, Z Anat Entwicklungsgesch, V122, P197, DOI 10.1007/BF00521798
   Messner K, 1999, CELLS TISSUES ORGANS, V165, P30, DOI 10.1159/000016671
   Milanini J, 1998, J BIOL CHEM, V273, P18165, DOI 10.1074/jbc.273.29.18165
   Movin T, 1997, ACTA ORTHOP SCAND, V68, P170, DOI 10.3109/17453679709004002
   Neufeld G, 1999, FASEB J, V13, P9, DOI 10.1096/fasebj.13.1.9
   Petersen W, 1999, UNFALLCHIRURG, V102, P543, DOI 10.1007/s001130050448
   Petersen W, 1999, ARCH ORTHOP TRAUM SU, V119, P371
   Petersen W, 2000, ACTA ORTHOP SCAND, V71, P168, DOI 10.1080/000164700317413148
   Ryuto M, 1996, J BIOL CHEM, V271, P28220, DOI 10.1074/jbc.271.45.28220
   SHALABY F, 1995, NATURE, V376, P62, DOI 10.1038/376062a0
   STAVRI GT, 1995, CIRCULATION, V92, P11, DOI 10.1161/01.CIR.92.1.11
NR 34
TC 94
Z9 96
U1 0
U2 3
PU SPRINGER-VERLAG
PI NEW YORK
PA 175 FIFTH AVE, NEW YORK, NY 10010 USA
SN 0945-6317
J9 VIRCHOWS ARCH
JI Virchows Arch. Int. J. Pathol.
PD OCT
PY 2001
VL 439
IS 4
BP 579
EP 585
DI 10.1007/s004280100422
PG 7
WC Pathology
WE Science Citation Index Expanded (SCI-EXPANDED)
SC Pathology
GA 483AD
UT WOS:000171610800011
PM 11710646
DA 2022-07-22
ER

PT J
AU Frankewycz, B
   Penz, A
   Weber, J
   da Silva, NP
   Freimoser, F
   Bell, R
   Nerlich, M
   Jung, EM
   Docheva, D
   Pfeifer, CG
AF Frankewycz, B.
   Penz, A.
   Weber, J.
   da Silva, N. P.
   Freimoser, F.
   Bell, R.
   Nerlich, M.
   Jung, E. M.
   Docheva, D.
   Pfeifer, C. G.
TI Achilles tendon elastic properties remain decreased in long term after
   rupture
SO KNEE SURGERY SPORTS TRAUMATOLOGY ARTHROSCOPY
LA English
DT Article
DE Achilles tendon; Achilles tendon rupture; Shear wave elastography;
   Elasticproperties; Biomechanical properties; Tendon biomechanics
ID STIFFNESS; REPAIR; LIVER
AB Rupture of the Achilles tendon results in inferior scar tissue formation. Elastography allows a feasible in vivo investigation of biomechanical properties of the Achilles tendon. The purpose of this study is to investigate the biomechanical properties of healed Achilles tendons in the long term.
   Patients who suffered from Achilles tendon rupture were recruited for an elastographic evaluation. Unilateral Achilles tendon ruptures were included and scanned in the mid-substance and calcaneal insertion at least 2 years after rupture using shear wave elastography. Results were compared to patients' contralateral non-injured Achilles tendons and additionally to a healthy population. Descriptive statistics, reliability analysis, and correlation analysis with clinical scores were performed.
   Forty-one patients were included in the study with a mean follow-up-time of 74 +/- 30; [26-138] months after rupture. Significant differences were identified in shear wave elastography in the mid-substance of healed tendons (shear wave velocity 1.2 +/- 1.5 m/s) compared to both control groups [2.5 +/- 1.5 m/s (p < 0.01) and 2.8 +/- 1.6 m/s (p < 0.0001) contralateral and healthy population, respectively]. There was no correlation between the measurements and the clinical outcome.
   This study shows that the healed Achilles tendon after rupture has inferior elastic properties even after a long-term healing phase. Differences in elastic properties after rupture mainly originate from the mid-substance of the Achilles tendon, in which most of the ruptures occur. Elastographic results do not correspond with subjective perception. Clinically, sonoelastographical measurements of biomechanical properties can be useful to provide objective insights in tendon recovery.
C1 [Frankewycz, B.; Penz, A.; Weber, J.; Freimoser, F.; Nerlich, M.; Docheva, D.; Pfeifer, C. G.] Regensburg Univ, Med Ctr, Dept Trauma Surg, Franz Josef Strauss Allee 11, D-93053 Regensburg, Germany.
   [Frankewycz, B.; Penz, A.; Weber, J.; Freimoser, F.; Nerlich, M.; Docheva, D.; Pfeifer, C. G.] Regensburg Univ, Med Ctr, Lab Expt Trauma Surg, Franz Josef Strauss Allee 11, D-93053 Regensburg, Germany.
   [da Silva, N. P.; Jung, E. M.] Regensburg Univ, Med Ctr, Dept Radiol, Franz Josef Strauss Allee 11, D-93053 Regensburg, Germany.
   [Frankewycz, B.; Bell, R.] Cornell Univ, Sibley Sch Mech & Aerosp Engn, 341 Upson Hall, Ithaca, NY 14853 USA.
RP Frankewycz, B (通讯作者)，Regensburg Univ, Med Ctr, Dept Trauma Surg, Franz Josef Strauss Allee 11, D-93053 Regensburg, Germany.; Frankewycz, B (通讯作者)，Regensburg Univ, Med Ctr, Lab Expt Trauma Surg, Franz Josef Strauss Allee 11, D-93053 Regensburg, Germany.; Frankewycz, B (通讯作者)，Cornell Univ, Sibley Sch Mech & Aerosp Engn, 341 Upson Hall, Ithaca, NY 14853 USA.
EM borys.frankewycz@ukr.de; andrea.penz@stud.uni-regensburg.de;
   johannes1.weber@ukr.de; natascha.platz-batista-da-silva@ukr.de;
   florian.freimoser@ukr.de; rb622@cornell.edu; michael.nerlich@ukr.de;
   ernst-michael.jung@ukr.de; denitsa.docheva@ukr.de;
   christian.pfeifer@ukr.de
RI Docheva, Denitsa/R-1259-2018
OI Docheva, Denitsa/0000-0002-7588-1290
CR Arya S, 2010, J APPL PHYSIOL, V108, P670, DOI 10.1152/japplphysiol.00259.2009
   Attia D, 2015, ULTRASCHALL MED, V36, P603, DOI 10.1055/s-0041-107971
   Aubry S, 2015, RADIOLOGY, V274, P821, DOI 10.1148/radiol.14140434
   Aubry S, 2013, SKELETAL RADIOL, V42, P1143, DOI 10.1007/s00256-013-1649-9
   Bamber J, 2013, ULTRASCHALL MED, V34, P169, DOI 10.1055/s-0033-1335205
   Bleakney RR, 2002, CLIN J SPORT MED, V12, P273, DOI 10.1097/00042752-200209000-00003
   Bressel E, 2001, AM J SPORT MED, V29, P321, DOI 10.1177/03635465010290031201
   Chen TM, 2009, CLIN ANAT, V22, P377, DOI 10.1002/ca.20758
   Chen XM, 2013, J ULTRAS MED, V32, P449, DOI 10.7863/jum.2013.32.3.449
   DeWall RJ, 2014, J BIOMECH, V47, P2685, DOI 10.1016/j.jbiomech.2014.05.008
   Dillman JR, 2015, PEDIATR RADIOL, V45, P376, DOI 10.1007/s00247-014-3150-6
   Docheva D, 2015, ADV DRUG DELIVER REV, V84, P222, DOI 10.1016/j.addr.2014.11.015
   Eliasson P, 2016, EUR J NUCL MED MOL I, V43, P1868, DOI 10.1007/s00259-016-3379-4
   Frank C, 1997, CONNECT TISSUE RES, V36, P261, DOI 10.3109/03008209709160226
   Fu S, 2016, J ULTRAS MED, V35, P1881, DOI 10.7863/ultra.16.01052
   Ganestam A, 2016, KNEE SURG SPORT TR A, V24, P3730, DOI 10.1007/s00167-015-3544-5
   Geremia JM, 2015, CLIN BIOMECH, V30, P485, DOI 10.1016/j.clinbiomech.2015.03.005
   LaCroix AS, 2013, J APPL PHYSIOL, V115, P43, DOI 10.1152/japplphysiol.01449.2012
   Lohrer H, 2009, BMC MUSCULOSKEL DIS, V10, DOI 10.1186/1471-2474-10-134
   Longo UG, 2009, SPORTS MED ARTHROSC, V17, P127, DOI 10.1097/JSA.0b013e3181a3d767
   Maganaris CN, 2008, DISABIL REHABIL, V30, P1542, DOI 10.1080/09638280701785494
   Mark-Christensen T, 2016, KNEE SURG SPORT TR A, V24, P1852, DOI 10.1007/s00167-014-3180-5
   Nierhoff J, 2013, EUR RADIOL, V23, P3040, DOI 10.1007/s00330-013-2927-6
   Palmes D, 2002, J ORTHOPAED RES, V20, P939, DOI 10.1016/S0736-0266(02)00032-3
   Pedowitz D, 2013, CURR REV MUSCULOSKE, V6, P285, DOI 10.1007/s12178-013-9185-8
   Raikin SM, 2013, FOOT ANKLE INT, V34, P475, DOI 10.1177/1071100713477621
   Rettig AC, 2005, AM J SPORT MED, V33, P119, DOI 10.1177/0363546504268720
   Ruan ZM, 2015, INT J CLIN EXP MED, V8, P1043
   Thiele M, 2017, ULTRASCHALL MED, V38, P648, DOI 10.1055/s-0042-108431
   van Bergen CJA, 2014, ARCH ORTHOP TRAUM SU, V134, P897, DOI 10.1007/s00402-014-1994-8
   Wang JHC, 2006, J BIOMECH, V39, P1563, DOI 10.1016/j.jbiomech.2005.05.011
   WILLIAMS IF, 1980, RES VET SCI, V28, P302, DOI 10.1016/S0034-5288(18)32713-9
   Wu CH, 2012, J MED ULTRASOUND, V20, P79, DOI 10.1016/j.jmu.2012.04.006
   Ying M, 2003, ULTRASOUND MED BIOL, V29, P637, DOI 10.1016/S0301-5629(03)00008-5
   Zhang LN, 2016, MED SCI MONITOR, V22, P1186, DOI 10.12659/MSM.895674
NR 35
TC 21
Z9 23
U1 1
U2 10
PU SPRINGER
PI NEW YORK
PA 233 SPRING ST, NEW YORK, NY 10013 USA
SN 0942-2056
EI 1433-7347
J9 KNEE SURG SPORT TR A
JI Knee Surg. Sports Traumatol. Arthrosc.
PD JUL
PY 2018
VL 26
IS 7
SI SI
BP 2080
EP 2087
DI 10.1007/s00167-017-4791-4
PG 8
WC Orthopedics; Sport Sciences; Surgery
WE Science Citation Index Expanded (SCI-EXPANDED)
SC Orthopedics; Sport Sciences; Surgery
GA GL5XY
UT WOS:000437249800024
PM 29147741
DA 2022-07-22
ER

PT J
AU Kowatari, K
   Nakashima, K
   Ono, A
   Yoshihara, M
   Amano, M
   Toh, S
AF Kowatari, K
   Nakashima, K
   Ono, A
   Yoshihara, M
   Amano, M
   Toh, S
TI Levofloxacin-induced bilateral Achilles tendon rupture: a case report
   and review of the literature
SO JOURNAL OF ORTHOPAEDIC SCIENCE
LA English
DT Article
DE Levofloxacin; Achilles tendon rupture; side effect
ID FLUOROQUINOLONE ANTIBIOTICS; TENDINOPATHY; TENDINITIS; CIPROFLOXACIN;
   DISORDERS; THERAPY
AB We present a rare case of spontaneous bilateral Achilles tendon rupture induced by levofloxacin, one of the fluoroquinolone antibiotics. A 76-year-old man was diagnosed with acute appendicitis and was commenced on oral levofloxacin 300mg/day for 2 weeks. Seven days afterward he developed pain in both Achilles tendons, and at 14 days he developed swelling in the tendons. Four days later he felt a sharp, painful snap in both Achilles tendons while changing trousers. Both Achilles tendons ruptured completely at the mid-portion. There was no obvious underlying disease or pathophysiological factor causing fragility of his Achilles tendons. Despite the relatively large volume of case-based evidence, the pathophysiology of fluoroquinolone-induced tendinitis and tendon rupture is unclear. When tendinitis develops during fluoroquinolone therapy, the physician should consider the possible association between the fluoroquinolone and tendon rupture.
C1 Aomori Rosai Hosp, Dept Orthopaed Surg, Hachinohe 0318551, Japan.
   Hirosaki Univ, Sch Med, Dept Orthopaed Surg, Hirosaki, Aomori 036, Japan.
RP Kowatari, K (通讯作者)，Aomori Rosai Hosp, Dept Orthopaed Surg, 1 Minamigaoka,Shirogane Machi, Hachinohe 0318551, Japan.
RI Toh, Satoshi/K-5737-2013
CR BAILEY RR, 1983, NEW ZEAL MED J, V96, P590
   BURKHARDT JE, 1992, TOXICOL PATHOL, V20, P246, DOI 10.1177/019262339202000211
   COWAN MA, 1961, BRIT MED J, V1, P1658, DOI 10.1136/bmj.1.5240.1658
   Davey PG, 1996, BRIT MED J, V312, P613, DOI 10.1136/bmj.312.7031.613
   DONCK JB, 1994, TRANSPLANTATION, V58, P736, DOI 10.1097/00007890-199409270-00021
   HAINES JF, 1983, ANN RHEUM DIS, V42, P652, DOI 10.1136/ard.42.6.652
   JORGENSEN C, 1991, REV RHUM, V58, P623
   LEE MLH, 1961, BRIT MED J, V1, P1829, DOI 10.1136/bmj.1.5242.1829-b
   LEHUEC JC, 1995, J BONE JOINT SURG BR, V77B, P293, DOI 10.1302/0301-620X.77B2.7706350
   MAYER JH, 1961, BRIT MED J, V1, P1830, DOI 10.1136/bmj.1.5242.1830
   McGarvey WC, 1996, FOOT ANKLE INT, V17, P496, DOI 10.1177/107110079601700811
   Meyboom RHBS, 1994, PHARMACOEPIDEMIOL DR, V3, P185
   Movin T, 1997, FOOT ANKLE INT, V18, P297, DOI 10.1177/107110079701800510
   *NORD COUNC MED, 1996, NORD STAT MED 1993 1
   PERROT S, 1991, PRESSE MED, V20, P1234
   PIERFITTE C, 1995, NEW ENGL J MED, V332, P193
   Pierfitte C, 1996, THERAPIE, V51, P419
   PRESTON ET, 1972, J AMER MED ASSOC, V221, P406, DOI 10.1001/jama.221.4.406
   PRICE AE, 1986, CLIN ORTHOP RELAT R, P249
   RIBARD P, 1992, J RHEUMATOL, V19, P1479
   ROYER RJ, 1994, THERAPIE, V49, P75
   SMAILL GB, 1961, BRIT MED J, V1, P1657, DOI 10.1136/bmj.1.5240.1657
   SZARFMAN A, 1995, NEW ENGL J MED, V332, P193
   van der Linden PD, 1999, BRIT J CLIN PHARMACO, V48, P433
   West MB, 1998, NEW ZEAL MED J, V111, P18
   Zabraniecki L, 1996, J RHEUMATOL, V23, P516
NR 26
TC 21
Z9 21
U1 0
U2 6
PU SPRINGER TOKYO
PI TOKYO
PA 3-3-13, HONGO, BUNKYO-KU, TOKYO, 113-0033, JAPAN
SN 0949-2658
J9 J ORTHOP SCI
JI J. Orthop. Sci.
PY 2004
VL 9
IS 2
BP 186
EP 190
DI 10.1007/s00776-003-0761-4
PG 5
WC Orthopedics
WE Science Citation Index Expanded (SCI-EXPANDED)
SC Orthopedics
GA 872HU
UT WOS:000225199100015
PM 15045551
DA 2022-07-22
ER

PT J
AU Ilhami, K
   Gokhan, M
   Ulukan, I
   Eray, BM
   Levent, A
   Cigdem, T
AF Ilhami, K
   Gokhan, M
   Ulukan, I
   Eray, BM
   Levent, A
   Cigdem, T
TI Biomechanical and histologic comparison of Achilles tendon ruptures
   reinforced with intratendinous and peritendinous plantaris tendon grafts
   in rabbits: an experimental study
SO ARCHIVES OF ORTHOPAEDIC AND TRAUMA SURGERY
LA English
DT Article
DE Achilles tendon rupture; Achilles tendon augmentation; tendo Achillis;
   Achilles tendon biomechanics; Achilles tendon in rabbit
ID PERCUTANEOUS REPAIR
AB Introduction: We hypothesized that the closer the reinforcing graft was to the repair zone, the more strength the healed tendon would achieved. Therefore, we compared the ruptured rabbit Achilles tendons reinforced with intratendinous and peritendinous plantaris grafts. Materials and methods: The experimental study was performed on Achilles tendons of 20 rabbits. First, they were divided into two groups: group I (n=10) underwent intratendinous graft and end-to-end tenorraphy, and group P (n=10) were repaired end-to-end and then reinforced with a peritendinous plantaris graft. An above-knee cast was applied during 6 weeks postoperatively. The two groups were compared to each other biomechanically and histologically. Seven randomly selected rabbits from each group were used for biomechanical evaluation. The remaining six rabbits (three from each group) were used for histologic comparison. Non-operated sides (n=20) served as the control group. Results: The mean maximum load at rupture of the repaired and control groups was 159.9+31 N, 83 +/- 7.5 N, and 207.5 +/- 35 N for group I, group P, and the control group, respectively. Values between groups were significantly different considering maximum load and absorbed energy to rupture. There was no significant difference between groups I and P in respect to strain. Control group tendons (groups I-C and P-C) had significantly more lengthening capability than operated tendons. Macroscopically, group I tendons were thicker and stiffer than group P tendons. Histologically, differences between the group I and group P specimens revealed that the healing process was faster in tendons augmented intratendinously. Conclusions: In reinforcing Achilles tendon repair, the site of the tendon graft affected the result. When the graft was used intratendinously, the healed tendon was more similar biomechanically to normal tendon and had more graft-tendon orientation histologically than the tendon augmented peritendinously.
C1 Kocatepe Univ, Fac Med, Orthoped & Traumatol Dept, Afyon, Turkey.
   Osmangazi Univ, Fac Med, Orthoped & Traumatol Dept, Eskisehir, Turkey.
   Kocatepe Univ, Fac Med, Dept Pathol, Afyon, Turkey.
RP Ilhami, K (通讯作者)，Kocatepe Univ, Fac Med, Orthoped & Traumatol Dept, Afyon, Turkey.
EM ikuru@isnet.net.tr
CR BRADLEY JP, 1990, AM J SPORT MED, V18, P188, DOI 10.1177/036354659001800213
   BROWN TD, 1981, J TRAUMA, V21, P951, DOI 10.1097/00005373-198111000-00007
   COOMBS RRH, 1980, J BONE JOINT SURG BR, V62, P258
   HOCKENBURY RT, 1990, FOOT ANKLE, V11, P67, DOI 10.1177/107110079001100202
   Lim J, 2001, FOOT ANKLE INT, V22, P559, DOI 10.1177/107110070102200705
   LYNN TA, 1966, J BONE JOINT SURG AM, VA 48, P268, DOI 10.2106/00004623-196648020-00005
   Maffulli N, 1999, J BONE JOINT SURG AM, V81A, P1019, DOI 10.2106/00004623-199907000-00017
   MILES JW, 1992, J BONE JOINT SURG AM, V74A, P411, DOI 10.2106/00004623-199274030-00013
   NISTOR L, 1981, J BONE JOINT SURG AM, V63, P394, DOI 10.2106/00004623-198163030-00012
   NYSTROM B, 1983, ACTA CHIR SCAND, V149, P669
   OBRIEN M, 1992, CLIN SPORT MED, V11, P505
   PEREZ TA, 1974, ORTHOP CLIN N AM, V5, P89
   ROBERTS JM, 1983, CLIN ORTHOP RELAT R, P244
   RODKEY WG, 1985, AM J SPORT MED, V13, P242, DOI 10.1177/036354658501300405
   SOMA CA, 1995, ORTHOP CLIN N AM, V26, P239
   Thermann H, 2001, FOOT ANKLE INT, V22, P478, DOI 10.1177/107110070102200604
   VIDIIK A, 1969, ACTA ORTHOP SCAND, V40, P261
   Webb JM, 1999, J BONE JOINT SURG BR, V81B, P877, DOI 10.1302/0301-620X.81B5.9784
   Winter E, 1998, ARCH ORTHOP TRAUM SU, V117, P364, DOI 10.1007/s004020050267
NR 19
TC 8
Z9 9
U1 0
U2 4
PU SPRINGER
PI NEW YORK
PA 233 SPRING ST, NEW YORK, NY 10013 USA
SN 0936-8051
EI 1434-3916
J9 ARCH ORTHOP TRAUM SU
JI Arch. Orthop. Trauma Surg.
PD NOV
PY 2004
VL 124
IS 9
BP 608
EP 613
DI 10.1007/s00402-003-0626-5
PG 6
WC Orthopedics; Surgery
WE Science Citation Index Expanded (SCI-EXPANDED)
SC Orthopedics; Surgery
GA 881AS
UT WOS:000225836400006
PM 14991383
DA 2022-07-22
ER

PT J
AU Cebrian, P
   Manjon, P
   Caba, P
AF Cebrian, P
   Manjon, P
   Caba, P
TI Ultrasonography of non-traumatic rupture of the Achilles tendon
   secondary to levofloxacin
SO FOOT & ANKLE INTERNATIONAL
LA English
DT Article
DE tendinosis; levofloxacin; rupture; Achilles tendon; ultrasonography
ID FLUOROQUINOLONE ANTIBIOTICS; TENDINITIS
AB Rupture of Achilles tendon (AT) is an uncommon complication of treatment with fluoroquinolones. We describe a case of bilateral tendinosis and rupture of the right AT in a patient who began levofloxacin treatment for community acquired pneumonia. Sonography showed thickening and hypoecogenicity of both AT and complete rupture and separation of the right Achilles tendon.
C1 Hosp Univ 12 Octubre, Dept Radiodiagnost, Madrid 28041, Spain.
   Hosp Univ 12 Octubre, Serv Traumatol 2, Madrid 28041, Spain.
RP Cebrian, P (通讯作者)，Hosp Univ 12 Octubre, Dept Radiodiagnost, Carretera de Andalucia,Km 5,400, Madrid 28041, Spain.
EM pcvbb@yahoo.es
CR Biundo J J Jr, 1997, Curr Opin Rheumatol, V9, P151, DOI 10.1097/00002281-199703000-00012
   Burgos EC, 2000, MED CLIN-BARCELONA, V114, P319
   CHASLERIE A, 1992, REV RHUM, V59, P297
   Estrada VAD, 1997, ARCH MED RES, V28, P429
   Gibbon WW, 2000, J CLIN ULTRASOUND, V28, P61, DOI 10.1002/(SICI)1097-0096(200002)28:2<61::AID-JCU1>3.0.CO;2-R
   Kirschenbaum S E, 1980, J Foot Surg, V19, P4
   Lewis JR, 1999, ANN PHARMACOTHER, V33, P792, DOI 10.1345/aph.18298
   Lin J, 2000, AM J ROENTGENOL, V175, P637, DOI 10.2214/ajr.175.3.1750637
   LINDHOLM A, 1959, Acta Chir Scand, V117, P261
   McGarvey WC, 1996, FOOT ANKLE INT, V17, P496, DOI 10.1177/107110079601700811
   Meyboom RHBS, 1994, PHARMACOEPIDEMIOL DR, V3, P185
   RIBARD P, 1992, J RHEUMATOL, V19, P1479
   van der Linden PD, 1999, BRIT J CLIN PHARMACO, V48, P433
   1996, FDA MED B, V3, P26
NR 14
TC 6
Z9 6
U1 0
U2 1
PU SAGE PUBLICATIONS INC
PI THOUSAND OAKS
PA 2455 TELLER RD, THOUSAND OAKS, CA 91320 USA
SN 1071-1007
EI 1944-7876
J9 FOOT ANKLE INT
JI Foot Ankle Int.
PD FEB
PY 2003
VL 24
IS 2
BP 122
EP 124
DI 10.1177/107110070302400204
PG 3
WC Orthopedics
WE Science Citation Index Expanded (SCI-EXPANDED)
SC Orthopedics
GA 644WQ
UT WOS:000180943700005
PM 12627618
DA 2022-07-22
ER

PT J
AU Wang, BC
   Feng, XN
   Yan, M
   Wang, H
   Li, Y
AF Wang, Baocang
   Feng, Xiaona
   Yan, Ming
   Wang, Hui
   Li, Yong
TI Application of lariat lock catch knot suture in the achilles tendon
   rupture
SO INTERNATIONAL JOURNAL OF CLINICAL AND EXPERIMENTAL MEDICINE
LA English
DT Article
DE Achilles tendon; lariat lock catch knot; tendon suture; achilles tendon
   lengthening
ID NONSURGICAL TREATMENT; REPAIR; RECONSTRUCTION; INJURY
AB The aim of this study was to summarize the clinical experience of repairing the Achilles tendon rupture by lariat lock catch knot suture. Between January 2011 and February, 2014, 32 cases of the Achilles tendon rupture were treated by lariat lock catch knot suture. There were 26 males and 6 females, with the average age of 39 years (range 17-53 years), including 13 left knees and 19 right knees. 29 wounds healed by first intention, and 3 cases who were performed local flap transfer due to necrosis of skin were healed by second intention. Thirty-two cases were followed up 10-25 months (13 months on average). No re-rupture of Achilles tendon or deep infection occurred during follow-up period. According to Arner-Lindholm assessment standard, the results were excellent in 19 cases and good in 13 cases, the excellent and good rate was 100%. Lariat lock catch knot suture is a safe and effective method for repairing Achilles tendon.
C1 [Wang, Baocang; Yan, Ming; Wang, Hui; Li, Yong] Second Hosp Tangshan, Dept Orthoped, Tangshan 063000, Peoples R China.
   [Feng, Xiaona] Second Hosp Tangshan, Dept Pediat Orthoped, 21 North Jianshe Rd, Tangshan 063000, Peoples R China.
RP Feng, XN (通讯作者)，Second Hosp Tangshan, Dept Pediat Orthoped, 21 North Jianshe Rd, Tangshan 063000, Peoples R China.
EM xiaonafengcn@126.com
CR Appy-Fedida B, 2015, ORTHOP TRAUMATOL-SUR, V101, P93, DOI 10.1016/j.otsr.2014.10.019
   Barfod KW, 2014, DAN MED J, V61
   Del Buono A, 2014, BRIT MED BULL, V109, P45, DOI 10.1093/bmb/ldt029
   Demirel M, 2011, INDIAN J ORTHOP, V45, P45, DOI 10.4103/0019-5413.73654
   Dhillon Mandeep S, 2010, Foot (Edinb), V20, P154, DOI 10.1016/j.foot.2010.09.008
   Ding WG, 2014, FOOT ANKLE SURG, V20, P105, DOI 10.1016/j.fas.2013.12.007
   Flint JH, 2014, AM J SPORT MED, V42, P235, DOI 10.1177/0363546513490656
   Franz JR, 2015, GAIT POSTURE, V41, P192, DOI 10.1016/j.gaitpost.2014.10.001
   Jaakkola JI, 2001, FOOT ANKLE INT, V22, P979, DOI 10.1177/107110070102201210
   Kilicoglu Onder, 2014, Eur J Orthop Surg Traumatol, V24, P117, DOI 10.1007/s00590-012-1129-2
   Li CJ, 2014, GENET MOL RES, V13, P5405, DOI 10.4238/2014.July.24.20
   Longo UG, 2013, FOOT ANKLE CLIN, V18, P319, DOI 10.1016/j.fcl.2013.02.009
   Moller M, 2001, J BONE JOINT SURG BR, V83B, P843
   Olsson N, 2013, AM J SPORT MED, V41, P2867, DOI 10.1177/0363546513503282
   Ozsoy MH, 2013, FOOT ANKLE INT, V34, P1683, DOI 10.1177/1071100713505754
   Peterson KS, 2014, J FOOT ANKLE SURG, V53, P664, DOI 10.1053/j.jfas.2013.10.001
   Ponnapula P, 2010, J FOOT ANKLE SURG, V49, P310, DOI 10.1053/j.jfas.2010.02.021
   Singh R, 2014, BMJ CASE REP, V2014
   Sliwa Marek, 2014, Pol Orthop Traumatol, V79, P92
   Soroceanu A, 2012, J BONE JOINT SURG AM, V94A, P2136, DOI 10.2106/JBJS.K.00917
   Stavrou M, 2013, J ORTHOP SURG-HONG K, V21, P232, DOI 10.1177/230949901302100224
   Stickles SP, 2015, WEST J EMERG MED, V16, P161, DOI 10.5811/westjem.2014.10.24127
   Suchak AA, 2005, FOOT ANKLE INT, V26, P932, DOI 10.1177/107110070502601106
   Tang KL, 2007, AM J SPORT MED, V35, P589, DOI 10.1177/0363546506295080
   Thomopoulos S, 2015, J ORTHOP RES, V33, P832, DOI 10.1002/jor.22806
   Weinfeld SB, 2014, MED CLIN N AM, V98, P331, DOI 10.1016/j.mcna.2013.11.005
   Wisbeck JM, 2012, ORTHOPEDICS, V35, pE331, DOI 10.3928/01477447-20120222-41
NR 27
TC 0
Z9 0
U1 1
U2 1
PU E-CENTURY PUBLISHING CORP
PI MADISON
PA 40 WHITE OAKS LN, MADISON, WI 53711 USA
SN 1940-5901
J9 INT J CLIN EXP MED
JI Int. J. Clin. Exp. Med.
PY 2015
VL 8
IS 10
BP 19571
EP 19575
PG 5
WC Medicine, Research & Experimental
WE Science Citation Index Expanded (SCI-EXPANDED)
SC Research & Experimental Medicine
GA DA3BB
UT WOS:000367669800320
PM 26770612
DA 2022-07-22
ER

PT J
AU Alfredson, H
   Masci, L
   Ohberg, L
AF Alfredson, Hakan
   Masci, Lorenzo
   Ohberg, Lars
TI Partial mid-portion Achilles tendon ruptures: new sonographic findings
   helpful for diagnosis
SO BRITISH JOURNAL OF SPORTS MEDICINE
LA English
DT Article
ID SURGICAL FINDINGS; ULTRASONOGRAPHY; TENDINOPATHY; TENDINOSIS; INJURIES
AB Background Partial Achilles tendon ruptures are not always easy to diagnose. A history including a sudden onset of pain and/or relative weakness in plantar flexion force is an indicator. The most loaded side of the Achilles tendon is the dorsal side (skin side).
   Objective The aim of this study was to evaluate the ultrasound (US) and colour Doppler (CD) findings in patients with a suspected partial rupture in the Achilles tendon.
   Materials and Methods Seventeen patients (16 men and 1 woman) with a mean age of 36 years (range 23-71 years) were examined clinically and by US+CD because of mid-portion Achilles tendon pain. There was an acute onset in 14 of 17 patients, and all had painful weakness during tendon loading activity.
   Results In all patients, the US examination showed a partial Achilles tendon rupture, presented as a disrupted dorsal (skin side) tendon line and an irregular tendon structure mainly located in the dorsal and mid-tendon. The size of the rupture varied from 1/3 to 2/3 of the tendon thickness. In the dorsal part of the tendon, corresponding to the region with disrupted tendon line and irregular structure, CD examination showed high blood flow-most often of a longitudinal character. Six of the patients were surgically treated, and macroscopical examination verified the US findings showing disruption on the dorsal side and a partial rupture in the dorsal and mid-tendon.
   Conclusions US and Doppler examination can be helpful tools to diagnose partial mid-portion Achilles tendon ruptures. The characteristic findings of a disrupted dorsal tendon line and high blood flow in the structurally abnormal dorsal tendon indicate a partial rupture.
C1 [Alfredson, Hakan] Umea Univ, Sports Med Unit, Dept Surg & Perioperat Sci Sports Med, S-90187 Umea, Sweden.
   [Alfredson, Hakan; Ohberg, Lars] Umea Univ, Dept Radiat Sci, S-90187 Umea, Sweden.
   [Masci, Lorenzo] Pure Sports Med Clin, London, England.
RP Alfredson, H (通讯作者)，Umea Univ, Sports Med Unit, Dept Surg & Perioperat Sci Sports Med, S-90187 Umea, Sweden.
EM hakan.alfredson@idrott.umu.se
CR Alfredson H, 2005, KNEE SURG SPORT TR A, V13, P338, DOI 10.1007/s00167-004-0585-6
   ALMEKINDERS LC, 2002, P 10 C EUR SOC SPORT
   Astrom M, 1996, SKELETAL RADIOL, V25, P615, DOI 10.1007/s002560050146
   Fahlstrom M, 1998, AM J SPORT MED, V26, P467, DOI 10.1177/03635465980260032201
   GABEL S, 1994, FOOT ANKLE INT, V15, P512, DOI 10.1177/107110079401500912
   KALEBO P, 1992, AM J SPORT MED, V20, P378, DOI 10.1177/036354659202000402
   KANGAS J, 2007, THESIS OULU U HOSP
   Kayser R, 2005, BRIT J SPORT MED, V39, P838, DOI 10.1136/bjsm.2005.018416
   KVIST M, 1994, SPORTS MED, V18, P173, DOI 10.2165/00007256-199418030-00004
   Maganaris CN, 2008, DISABIL REHABIL, V30, P1542, DOI 10.1080/09638280701785494
   Ohberg L, 2001, KNEE SURG SPORT TR A, V9, P233, DOI 10.1007/s001670000189
   Paavola M, 1998, ACTA RADIOL, V39, P612, DOI 10.3109/02841859809175485
   Terslev L, 2001, Eur J Ultrasound, V13, P183, DOI 10.1016/S0929-8266(01)00130-6
   Weinberg EP, 1998, AM J ROENTGENOL, V171, P743, DOI 10.2214/ajr.171.3.9725308
NR 14
TC 17
Z9 18
U1 0
U2 8
PU B M J PUBLISHING GROUP
PI LONDON
PA BRITISH MED ASSOC HOUSE, TAVISTOCK SQUARE, LONDON WC1H 9JR, ENGLAND
SN 0306-3674
J9 BRIT J SPORT MED
JI Br. J. Sports Med.
PD APR
PY 2011
VL 45
IS 5
BP 429
EP U59
DI 10.1136/bjsm.2009.067298
PG 4
WC Sport Sciences
WE Science Citation Index Expanded (SCI-EXPANDED)
SC Sport Sciences
GA 732PL
UT WOS:000288199900010
PM 19945976
DA 2022-07-22
ER

PT J
AU Chen, TM
   Rozen, WM
   Pan, WR
   Ashton, MW
   Richardson, MD
   Taylor, GI
AF Chen, Tony M.
   Rozen, Warren M.
   Pan, Wei-Ren
   Ashton, Mark W.
   Richardson, Martin D.
   Taylor, G. Ian
TI The Arterial Anatomy of the Achilles Tendon: Anatomical Study and
   Clinical Implications
SO CLINICAL ANATOMY
LA English
DT Article
DE Achilles tendon; anatomy; vascular; orthopaedic surgery; leg; heel
ID QUANTITATIVE ASSESSMENT; CALCANEAL TENDON; BLOOD-FLOW; ANGIOSOMES;
   VASCULARIZATION; MANAGEMENT; RUPTURE; CADAVER
AB The Achilles tendon is the most frequently ruptured tendon in the lower limb and accounts for almost 20% of all large tendon injuries. Despite numerous published studies describing its blood supply, there has been no uniformity in describing its topography. The current study comprises a detailed anatomical study of both the intrinsic and extrinsic arterial supply of the Achilles tendon, providing the detail sought from studies calling for improved planning of surgical procedures where damage to the vascularity of the Achilles tendon is likely. A dissection, microdissection, histological, and angiographic study was undertaken on 20 cadaveric lower limbs from 16 fresh and four embalmed cadavers. The Achilles tendon is supplied by two arteries, the posterior tibial and peroneal arteries. Three vascular territories were identified, with the midsection supplied by the peroneal artery, and the proximal and distal sections supplied by the posterior tibial artery. The midsection of the Achilles tendon was markedly more hypovascular that the rest of the tendon. The Achilles tendon is at highest risk of rupture and surgical complications at its midsection. Individuals with particularly poor supply of the miclsection may be at increased risk of tendon rupture, and approaches to the tendon operatively should consider the route of supply by the peroneal artery to this susceptible part of the tendon. Clin. Anat. 22:377-385, 2009. (C) 2009 Wiley-Liss, Inc.
C1 [Chen, Tony M.; Rozen, Warren M.; Pan, Wei-Ren; Ashton, Mark W.; Richardson, Martin D.; Taylor, G. Ian] Univ Melbourne, Dept Anat & Cell Biol, Jack Brockhoff Reconstruct Plast Surg Res Unit, Parkville, Vic 3050, Australia.
RP Richardson, MD (通讯作者)，Univ Melbourne, Dept Anat & Cell Biol, Jack Brockhoff Reconstruct Plast Surg Res Unit, Room E533,Grattan St, Parkville, Vic 3050, Australia.
EM pdodwell@unimelb.edu.au
RI Rozen, Warren Matthew/AAE-6296-2022; Richardson, Martin/H-2272-2018
OI Rozen, Warren Matthew/0000-0002-4092-182X; Richardson,
   Martin/0000-0002-1280-3834; Ashton, Mark W/0000-0002-5104-480X
CR Ahmed IM, 1998, J ORTHOPAED RES, V16, P591, DOI 10.1002/jor.1100160511
   ASTROM M, 1994, J ORTHOP RES, V12, P246, DOI 10.1002/jor.1100120214
   Attinger CE, 2006, PLAST RECONSTR SURG, V117, p261S, DOI 10.1097/01.prs.0000222582.84385.54
   CAMPBELL P, 1993, BRIT J HOSP MED, V50, P321
   CARR AJ, 1989, J BONE JOINT SURG BR, V71, P100, DOI 10.1302/0301-620X.71B1.2914976
   Dietz Frederick R, 2006, Iowa Orthop J, V26, P27
   EDWARDS DAW, 1946, J ANAT, V80, P147
   FREY C, 1990, J BONE JOINT SURG AM, V72A, P884, DOI 10.2106/00004623-199072060-00014
   GILLIES H, 1970, J BONE JOINT SURG AM, VA 52, P337, DOI 10.2106/00004623-197052020-00015
   Goldstein Murray, 2001, Developmental Medicine and Child Neurology, V43, P563, DOI 10.1017/S0012162201001025
   GRAHAM HK, 1988, J BONE JOINT SURG BR, V70, P472, DOI 10.1302/0301-620X.70B3.3372574
   Green WT, 1942, J AMER MED ASSOC, V118, P434, DOI 10.1001/jama.1942.02830060014004
   Karcz M J, 1996, Folia Morphol (Warsz), V55, P306
   KOLTS I, 1994, ANN ANAT, V176, P75, DOI 10.1016/S0940-9602(11)80420-6
   LAGERGREN C, 1959, Acta Chir Scand, V116, P491
   LAINE V A, 1955, Acta Orthop Scand, V24, P250
   Langberg H, 1998, ACTA PHYSIOL SCAND, V163, P149, DOI 10.1046/j.1365-201X.1998.00361.x
   LANGBERG H, 1972, J BONE JOINT SURG AM, V54, P1398
   LEA RB, 1972, J BONE JOINT SURG AM, VA 54, P1398, DOI 10.2106/00004623-197254070-00002
   LOHR JF, 1990, CLIN ORTHOP RELAT R, P35
   Majewski M, 2006, AM J SPORT MED, V34, P793, DOI 10.1177/0363546505283266
   Melhus Asa, 2005, Expert Opin Drug Saf, V4, P299, DOI 10.1517/14740338.4.2.299
   MORIMOTO I, 1968, ACTA ANAT NIPPON, V43, P295
   Ng E S, 2006, J Orthop Surg (Hong Kong), V14, P142
   Niculescu V, 1988, Morphol Embryol (Bucur), V34, P5
   PEACOCK EE, 1959, ANN SURG, V149, P415, DOI 10.1097/00000658-195903000-00011
   Petersen W, 2000, J ANAT, V197, P617, DOI 10.1046/j.1469-7580.2000.19740617.x
   Pufe T, 2005, SCAND J MED SCI SPOR, V15, P211, DOI 10.1111/j.1600-0838.2005.00465.x
   REES MJW, 1986, PLAST RECONSTR SURG, V77, P141, DOI 10.1097/00006534-198601000-00023
   REINHERZ R P, 1991, Journal of Foot Surgery, V30, P117
   SanzHospital FJ, 1997, FOOT ANKLE INT, V18, P506, DOI 10.1177/107110079701800809
   SCHMIDTROHLFING B, 1992, INT ORTHOP, V16, P29
   SMITH JW, 1965, AM J SURG, V109, P272, DOI 10.1016/S0002-9610(65)80073-3
   Stein V, 2000, ACTA ORTHOP SCAND, V71, P60, DOI 10.1080/00016470052943919
   TAYLOR GI, 1987, BRIT J PLAST SURG, V40, P113, DOI 10.1016/0007-1226(87)90185-8
   TAYLOR GI, 1992, PLAST RECONSTR SURG, V89, P408, DOI 10.1097/00006534-199203000-00002
   Taylor GI, 1998, PLAST RECONSTR SURG, V102, P599, DOI 10.1097/00006534-199809030-00001
   Theobald P, 2006, J ANAT, V208, P91, DOI 10.1111/j.1469-7580.2006.00510.x
   Theobald P, 2005, INJURY, V36, P1267, DOI 10.1016/j.injury.2005.02.012
   Yu C, 2005, Australas Radiol, V49, P407, DOI 10.1111/j.1440-1673.2005.01470.x
   Zantop T, 2003, ARCH ORTHOP TRAUM SU, V123, P501, DOI 10.1007/s00402-003-0491-2
NR 41
TC 107
Z9 118
U1 1
U2 26
PU WILEY
PI HOBOKEN
PA 111 RIVER ST, HOBOKEN 07030-5774, NJ USA
SN 0897-3806
EI 1098-2353
J9 CLIN ANAT
JI Clin. Anat.
PD APR
PY 2009
VL 22
IS 3
BP 377
EP 385
DI 10.1002/ca.20758
PG 9
WC Anatomy & Morphology
WE Science Citation Index Expanded (SCI-EXPANDED)
SC Anatomy & Morphology
GA 426IZ
UT WOS:000264702500015
PM 19173244
DA 2022-07-22
ER

PT J
AU Patel, KA
   O'Malley, MJ
AF Patel, Karan A.
   O'Malley, Martin J.
TI Management of Achilles Tendon Injuries in the Elite Athlete
SO ORTHOPEDIC CLINICS OF NORTH AMERICA
LA English
DT Article
DE Achilles rupture; High-level athletes; Elite athletes; Surgical
   intervention
ID OPEN REPAIR; NONOPERATIVE TREATMENT; RUPTURE; COMPLICATIONS; THROMBOSIS;
   THERAPY
C1 [Patel, Karan A.] Mayo Clin Arizona, 5777 East Mayo Blvd, Phoenix, AZ 85054 USA.
   [O'Malley, Martin J.] Hosp Special Surg, 420 East 72 St, New York, NY 10021 USA.
RP O'Malley, MJ (通讯作者)，Hosp Special Surg, 420 East 72 St, New York, NY 10021 USA.
EM OMalleyM@hss.edu
OI Patel, Karan/0000-0003-1107-5094
CR Aktas S, 2009, FOOT ANKLE INT, V30, P391, DOI 10.3113/FAI.2009.0391
   Alves AN, 2014, AM J PHYS MED REHAB, V93, P1073, DOI 10.1097/PHM.0000000000000158
   Avina Valencia Jorge Arturo, 2009, Acta Ortop Mex, V23, P125
   CETTI R, 1993, AM J SPORT MED, V21, P791, DOI 10.1177/036354659302100606
   Coalk I, 2020, J AM PODIAT MED ASSN, V110
   Ferraresi Cleber, 2012, Photonics Lasers Med, V1, P267
   Grassi A, 2018, J BONE JOINT SURG AM, V100, P1969, DOI 10.2106/JBJS.17.01364
   Jiang N, 2012, INT ORTHOP, V36, P765, DOI 10.1007/s00264-011-1431-3
   Karabinas Panagiotis K, 2014, Eur J Orthop Surg Traumatol, V24, P607, DOI 10.1007/s00590-013-1350-7
   Kearon C, 2012, CHEST, V141, pE419S, DOI 10.1378/chest.11-2301
   Kolodziej L, 2013, INT ORTHOP, V37, P625, DOI 10.1007/s00264-012-1737-9
   Lantto I, 2016, AM J SPORT MED, V44, P2406, DOI 10.1177/0363546516651060
   Lemme NJ, 2019, AM J SPORT MED, V47, P2360, DOI 10.1177/0363546519858609
   Lemme NJ, 2018, ORTHOP J SPORTS MED, V6, DOI 10.1177/2325967118808238
   Leppilahti J, 1998, SPORTS MED, V25, P79, DOI 10.2165/00007256-199825020-00002
   Lim J, 2001, FOOT ANKLE INT, V22, P559, DOI 10.1177/107110070102200705
   Maffulli N, 1999, J BONE JOINT SURG AM, V81A, P1019, DOI 10.2106/00004623-199907000-00017
   Maffulli N, 1999, CLIN J SPORT MED, V9, P157, DOI 10.1097/00042752-199907000-00007
   Majewski M, 2000, ORTHOPADE, V29, P670
   Makhdom AM, 2013, J FOOT ANKLE SURG, V52, P584, DOI 10.1053/j.jfas.2013.03.001
   Nicodemo MD, 2017, ACTA CIR BRAS, V32, P125, DOI 10.1590/s0102-865020170205
   Ochen Y, 2019, BMJ-BRIT MED J, V364, DOI 10.1136/bmj.k5120
   Pedowitz D, 2013, CURR REV MUSCULOSKE, V6, P285, DOI 10.1007/s12178-013-9185-8
   Perneby C, 2006, THROMB HAEMOSTASIS, V95, P652, DOI 10.1160/TH05-10-0653
   Trofa DP, 2017, AM J SPORT MED, V45, P2864, DOI 10.1177/0363546517713001
   Trofa DP, 2018, ORTHOP J SPORTS MED, V6
   van Maele M, 2018, INJURY, V49, P1947, DOI 10.1016/j.injury.2018.08.012
   Wertz J, 2013, SPORTS HEALTH, V5, P407, DOI 10.1177/1941738112472165
   Wilkins R, 2012, AM J SPORT MED, V40, P2154, DOI 10.1177/0363546512453293
   Willits K, 2010, J BONE JOINT SURG AM, V92A, P2767, DOI 10.2106/JBJS.I.01401
   Wu YH, 2019, AM J SPORT MED, V47, P2251, DOI 10.1177/0363546518824601
   Yang B, 2017, INT J SURG, V40, P178, DOI 10.1016/j.ijsu.2017.03.021
   Yow BG, 2018, J FOOT ANKLE SURG, V57, P635, DOI 10.1053/j.jfas.2017.11.008
   Zhou K, 2018, J FOOT ANKLE SURG, V57, P1191, DOI 10.1053/j.jfas.2018.05.007
NR 34
TC 1
Z9 1
U1 2
U2 4
PU W B SAUNDERS CO-ELSEVIER INC
PI PHILADELPHIA
PA 1600 JOHN F KENNEDY BOULEVARD, STE 1800, PHILADELPHIA, PA 19103-2899 USA
SN 0030-5898
EI 1558-1373
J9 ORTHOP CLIN N AM
JI Orthop. Clin. North Am.
PD OCT
PY 2020
VL 51
IS 4
SI SI
BP 533
EP 539
DI 10.1016/j.ocl.2020.06.009
PG 7
WC Orthopedics
WE Science Citation Index Expanded (SCI-EXPANDED)
SC Orthopedics
GA NU4PZ
UT WOS:000573626700013
PM 32950223
DA 2022-07-22
ER

PT J
AU Patel, A
   Ogawa, B
   Charlton, T
   Thordarson, D
AF Patel, Arush
   Ogawa, Brent
   Charlton, Timothy
   Thordarson, David
TI Incidence of Deep Vein Thrombosis and Pulmonary Embolism after Achilles
   Tendon Rupture
SO CLINICAL ORTHOPAEDICS AND RELATED RESEARCH
LA English
DT Article
ID MOLECULAR-WEIGHT HEPARIN; VENOUS THROMBOEMBOLISM; ANKLE SURGERY;
   PLASTER-CAST; RISK-FACTORS; PROLONGED THROMBOPROPHYLAXIS;
   IMMOBILIZATION; OUTPATIENTS; FOOT; INJURY
AB Background The use of venous thromboembolism prophylaxis after an Achilles rupture is controversial. The rates of reported deep vein thrombosis (DVT) range from 6.3% to 34%. There is no agreement regarding prophylactic therapy after an Achilles tendon rupture.
   Questions/purposes We determined the overall risk of DVT and pulmonary embolism (PE) after an Achilles tendon rupture and identified potential risk factors including surgery.
   Patients and Methods We retrospectively reviewed a large healthcare management organization database and identified 1172 patients who had Achilles tendon ruptures. None of the patients routinely received anticoagulation. Patients were stratified into surgical versus nonsurgical group, age older than 40 years, history of congestive heart failure, previous history of DVT or PE, and BMI greater than 30. A patient was considered to have symptomatic DVT or PE related to the Achilles tendon rupture if diagnosed within 3 months from the injury or surgery. We used a multivariable analysis to identify risk factors.
   Results The overall rates for DVT and PE after Achilles tendon ruptures were 0.43% and 0.34%, respectively. Age older than 40 years, congestive heart failure, history of DVT or PE, obesity, and whether a patient had surgery did not predict occurrence of DVT or PE.
   Conclusion We found the overall incidence of symptomatic DVT and PE to be low after an Achilles tendon rupture and believe routine use of anticoagulation might be unwarranted.
C1 [Patel, Arush; Ogawa, Brent; Charlton, Timothy; Thordarson, David] Univ So Calif, Keck Sch Med, Dept Orthopaed Surg, Los Angeles, CA 90033 USA.
   [Ogawa, Brent] Kaiser Permanente, Harbor City, CA USA.
RP Thordarson, D (通讯作者)，Univ So Calif, Keck Sch Med, Dept Orthopaed Surg, 1520 San Pablo St,Suite 2000, Los Angeles, CA 90033 USA.
EM thordars@usc.edu
FU NATIONAL CENTER FOR ADVANCING TRANSLATIONAL SCIENCES [UL1TR000130]
   Funding Source: NIH RePORTER; NATIONAL CENTER FOR RESEARCH RESOURCES
   [UL1RR031986] Funding Source: NIH RePORTER; NCATS NIH HHS [UL1 TR000130]
   Funding Source: Medline; NCRR NIH HHS [UL1 RR031986] Funding Source:
   Medline
CR [Anonymous], INT CLASSIFICATION D
   Bergqvist D, 2002, ARCH INTERN MED, V162, P2173, DOI 10.1001/archinte.162.19.2173
   Di Nisio M, 2011, J GERONTOL A-BIOL, V66, P320, DOI 10.1093/gerona/glq207
   Felcher AH, 2009, CHEST, V135, P917, DOI 10.1378/chest.08-1631
   Geerts WH, 2008, CHEST, V133, p381S, DOI 10.1378/chest.08-0656
   Hanslow SS, 2006, FOOT ANKLE INT, V27, P693, DOI 10.1177/107110070602700907
   Healy B, 2010, J BONE JOINT SURG BR, V92B, P646, DOI 10.1302/0301-620X.92B5.23241
   Heit JA, 2000, ARCH INTERN MED, V160, P809, DOI 10.1001/archinte.160.6.809
   HOFMANN AA, 1985, J BONE JOINT SURG AM, V67A, P633, DOI 10.2106/00004623-198567040-00020
   Houshian S, 1998, INJURY, V29, P651, DOI 10.1016/S0020-1383(98)00147-8
   Howell MD, 2001, J CLIN EPIDEMIOL, V54, P810, DOI 10.1016/S0895-4356(00)00373-5
   Jorgensen PS, 2002, THROMB RES, V105, P477, DOI 10.1016/S0049-3848(02)00059-2
   KOCK HJ, 1995, LANCET, V346, P459, DOI 10.1016/S0140-6736(95)91320-3
   KUJATH P, 1993, HAEMOSTASIS, V23, P20
   Lapidus LJ, 2007, ACTA ORTHOP, V78, P528, DOI 10.1080/17453670710014185
   Lapidus LJ, 2007, J ORTHOP TRAUMA, V21, P52, DOI 10.1097/01.bot.0000250741.65003.14
   Lassen MR, 2002, NEW ENGL J MED, V347, P726, DOI 10.1056/NEJMoa011327
   Mizel MS, 1998, CLIN ORTHOP RELAT R, P180
   Nilsson-Helander K, 2009, KNEE SURG SPORT TR A, V17, P1234, DOI 10.1007/s00167-009-0727-y
   Patil S, 2007, J BONE JOINT SURG BR, V89B, P1340, DOI 10.1302/0301-620X.89B10.19241
   Samama MM, 2000, ARCH INTERN MED, V160, P3415, DOI 10.1001/archinte.160.22.3415
   Solis G, 2002, FOOT ANKLE INT, V23, P411, DOI 10.1177/107110070202300507
   SooHoo NF, 2009, J BONE JOINT SURG AM, V91A, P1042, DOI 10.2106/JBJS.H.00653
   Wukich DK, 2008, J FOOT ANKLE SURG, V47, P243, DOI 10.1053/j.jfas.2008.02.003
   Wysowski DK, 2007, ARCH INTERN MED, V167, P1414, DOI 10.1001/archinte.167.13.1414
NR 25
TC 49
Z9 49
U1 0
U2 6
PU SPRINGER
PI NEW YORK
PA 233 SPRING ST, NEW YORK, NY 10013 USA
SN 0009-921X
J9 CLIN ORTHOP RELAT R
JI Clin. Orthop. Rel. Res.
PD JAN
PY 2012
VL 470
IS 1
BP 270
EP 274
DI 10.1007/s11999-011-2166-6
PG 5
WC Orthopedics; Surgery
WE Science Citation Index Expanded (SCI-EXPANDED)
SC Orthopedics; Surgery
GA 862OR
UT WOS:000298103100034
PM 22045072
OA Green Published
DA 2022-07-22
ER

PT J
AU Kerr, R
AF Kerr, R
TI Magnetic resonance imaging of the foot and ankle
SO SEMINARS IN ROENTGENOLOGY
LA English
DT Article
ID MORTON NEUROMA; TENDON RUPTURES; ACHILLES-TENDON; BREVIS TENDON;
   MR-IMAGES; JOINT; OSTEOMYELITIS; IMPINGEMENT; FEATURES; INJURIES
C1 Orthoped Hosp, Dept Radiol, Los Angeles, CA 90007 USA.
RP Kerr, R (通讯作者)，Orthoped Hosp, Dept Radiol, 2400 S Flower St, Los Angeles, CA 90007 USA.
CR ALEXANDER AH, 1980, J BONE JOINT SURG AM, V62, P646, DOI 10.2106/00004623-198062040-00020
   ANDERSON IF, 1989, J BONE JOINT SURG AM, V71A, P1143, DOI 10.2106/00004623-198971080-00004
   Anzilotti K, 1996, RADIOLOGY, V201, P515, DOI 10.1148/radiology.201.2.8888251
   BERKOWITZ JF, 1991, RADIOLOGY, V179, P665, DOI 10.1148/radiology.179.3.2027971
   CHANDNANI VP, 1994, RADIOLOGY, V192, P189, DOI 10.1148/radiology.192.1.8208935
   CONTI S, 1992, FOOT ANKLE, V13, P208, DOI 10.1177/107110079201300408
   Craig JG, 1997, RADIOLOGY, V203, P849, DOI 10.1148/radiology.203.3.9169715
   DAFFNER RH, 1992, AM J ROENTGENOL, V159, P245, DOI 10.2214/ajr.159.2.1632335
   DeSmet AA, 1996, SKELETAL RADIOL, V25, P159
   DUSSAULT RG, 1995, AM J ROENTGENOL, V164, P403, DOI 10.2214/ajr.164.2.7839978
   Farooki S, 1998, RADIOLOGY, V207, P357, DOI 10.1148/radiology.207.2.9577480
   Karjalainen PT, 1997, AM J SPORT MED, V25, P164, DOI 10.1177/036354659702500204
   KERR R, 1991, J COMPUT ASSIST TOMO, V15, P280, DOI 10.1097/00004728-199103000-00018
   Khoury NJ, 1996, RADIOLOGY, V200, P833, DOI 10.1148/radiology.200.3.8756941
   KLEIN MA, 1993, RADIOLOGY, V186, P233, DOI 10.1148/radiology.186.1.8416571
   LEE MS, 1989, RADIOLOGY, V172, P635, DOI 10.1148/radiology.172.3.2772169
   Magee TH, 1998, AM J ROENTGENOL, V170, P1227, DOI 10.2214/ajr.170.5.9574590
   MILLER TT, 1995, RADIOLOGY, V195, P849, DOI 10.1148/radiology.195.3.7754020
   MINK JH, 1993, MRI FOOT ANKLE, P135
   Morrison WB, 1998, RADIOLOGY, V207, P625, DOI 10.1148/radiology.207.3.9609883
   MORRISON WB, 1994, RADIOLOGY, V193, P841, DOI 10.1148/radiology.193.3.7972835
   PFEIFFER WH, 1994, J BONE JOINT SURG AM, V76A, P1222, DOI 10.2106/00004623-199408000-00012
   REDD RA, 1989, RADIOLOGY, V171, P415, DOI 10.1148/radiology.171.2.2649918
   RODEO SA, 1990, AM J SPORT MED, V18, P280, DOI 10.1177/036354659001800311
   Rosenberg ZS, 1997, AM J ROENTGENOL, V168, P141, DOI 10.2214/ajr.168.1.8976937
   Rubin DA, 1996, AM J ROENTGENOL, V166, P1079, DOI 10.2214/ajr.166.5.8615247
   Rubin DA, 1997, AM J ROENTGENOL, V169, P829, DOI 10.2214/ajr.169.3.9275907
   Yao L, 1996, FOOT ANKLE INT, V17, P33, DOI 10.1177/107110079601700107
   Zanetti M, 1997, SKELETAL RADIOL, V26, P82, DOI 10.1007/s002560050198
   Zanetti M, 1999, RADIOLOGY, V213, P583, DOI 10.1148/radiology.213.2.r99nv06583
   Zanetti M, 1997, RADIOLOGY, V203, P516, DOI 10.1148/radiology.203.2.9114115
   Zanetti M, 1997, AM J ROENTGENOL, V168, P529, DOI 10.2214/ajr.168.2.9016241
NR 32
TC 3
Z9 3
U1 0
U2 2
PU W B SAUNDERS CO
PI PHILADELPHIA
PA INDEPENDENCE SQUARE WEST CURTIS CENTER, STE 300, PHILADELPHIA, PA
   19106-3399 USA
SN 0037-198X
J9 SEMIN ROENTGENOL
JI Semin. Roentgenology
PD JUL
PY 2000
VL 35
IS 3
BP 306
EP 318
PG 13
WC Radiology, Nuclear Medicine & Medical Imaging
WE Science Citation Index Expanded (SCI-EXPANDED)
SC Radiology, Nuclear Medicine & Medical Imaging
GA 339ZB
UT WOS:000088506800012
PM 10939132
DA 2022-07-22
ER

PT J
AU Hansen, MS
   Barfod, KW
   Kristensen, MT
AF Hansen, Maria Swennergren
   Barfod, Kristoffer Weisskirchner
   Kristensen, Morten Tange
TI Development and reliability of the Achilles Tendon Length Measure and
   comparison with the Achilles Tendon Resting Angle on patients with an
   Achilles tendon rupture
SO FOOT AND ANKLE SURGERY
LA English
DT Article
DE Achilles Tendon Length Measure; Achilles Tendon Resting Angle;
   Reliability; Achilles tendon rupture; Elongation
ID ELONGATION
AB Background: There is a need for a valid, reliable, and easily applicable clinical measure of the length of the Achilles tendon (AT) after rupture. This study examines the reliability of a new ruler based measurement, the Achilles Tendon Length Measure (ATLM) in comparison with the goniometer-based Achilles Tendon Resting Angle (ATRA).
   Methods: Measurements were performed by two independent physiotherapists eight weeks after AT rupture on 28 patients treated non-operatively.
   Results: The mean (SD) injured ATLM was 56.5 (2.3) cm, ICC2.1 0.91(CI [0.72-0.97]), SEM 0.7 cm (SEM% 1.2), MDC 1.9 cm (MDC% 3.4). Corresponding data for the injured ATRA was mean 64.4 degrees (3.9 degrees), ICC2.1 0.84 (CI [0.68-0-92]), SEM 1.5 degrees (SEM% 2.4), MDC 4.3 degrees (MDC% 6.6).
   Conclusion: Both ATLM and ATRA showed excellent inter-rater reliability with low measurement error. Both measurements seem easy to use in clinical practice and potentially providing an indirect measure of the length of the AT after rupture. (C) 2016 European Foot and Ankle Society. Published by Elsevier Ltd. All rights reserved.
C1 [Hansen, Maria Swennergren; Kristensen, Morten Tange] Copenhagen Univ Hosp, Phys Med & Rehabil Res Copenhagen PMR C, Kettegard Alle 30, DK-2650 Hvidovre, Denmark.
   [Hansen, Maria Swennergren; Kristensen, Morten Tange] Copenhagen Univ Hosp, Dept Physiotherapy, Kettegard Alle 30, DK-2650 Hvidovre, Denmark.
   [Barfod, Kristoffer Weisskirchner; Kristensen, Morten Tange] Clin Orthopaed Res Hvidovre, Dept Orthopaed Surg, Kettegard Alle 30, DK-2650 Hvidovre, Denmark.
RP Hansen, MS (通讯作者)，Copenhagen Univ Hosp, Phys Med & Rehabil Res Copenhagen PMR C, Kettegard Alle 30, DK-2650 Hvidovre, Denmark.
EM maria_swennergren@hotmail.com; kristoffer.barfod@gmail.com;
   mortentange@hotmail.com
RI Kristensen, Morten/L-3527-2019; Barfod, Kristoffer/AAY-3288-2021
OI Kristensen, Morten/0000-0001-5868-4677; Barfod,
   Kristoffer/0000-0003-2620-5891
CR Beckerman H, 2001, QUAL LIFE RES, V10, P571, DOI 10.1023/A:1013138911638
   BLAND JM, 1986, LANCET, V1, P307, DOI 10.1016/s0140-6736(86)90837-8
   Carmont MR, 2013, FOOT ANKLE SURG, V19, P245, DOI 10.1016/j.fas.2013.06.007
   Carmont MR, 2015, ASIA PACIFIC J SPORT
   Costa ML, 2006, FOOT ANKLE INT, V27, P414, DOI 10.1177/107110070602700605
   Flansbjer UB, 2005, CLIN REHABIL, V19, P514, DOI 10.1191/0269215505cr854oa
   Fleiss JL., 1999, RELIABILITY MEASUREM, DOI [10.1002/9781118032923, DOI 10.1002/9781118032923]
   Ganestam A., 2015, KNEE SURG SPORTS TRA
   Harvill LM, 1991, ED MEASUREMENT ISSUE, V10, P33, DOI DOI 10.1111/J.1745-3992.1991.TB00195.X
   Huang XH, 2014, SCI WORLD J, DOI 10.1155/2014/954849
   Kangas J, 2007, AM J SPORT MED, V35, P59, DOI 10.1177/0363546506293255
   Kristensen MT, 2014, GAIT POSTURE, V39, P424, DOI 10.1016/j.gaitpost.2013.08.017
   Lantto I, 2015, SCAND J MED SCI SPOR, V25, pE133, DOI 10.1111/sms.12253
   Matles A L, 1975, Bull Hosp Joint Dis, V36, P48
   Mayich DJ, 2009, CAN J EMERG MED, V11, P242
   Mortensen NHM, 1999, J BONE JOINT SURG AM, V81A, P983, DOI 10.2106/00004623-199907000-00011
   Nilsson-Helander K, 2010, AM J SPORT MED, V38, P2186, DOI 10.1177/0363546510376052
   Rosso C, 2013, KNEE SURG SPORT TR A, V21, P1369, DOI 10.1007/s00167-013-2407-1
   Schepull T, 2012, SCAND J MED SCI SPOR, V22, P18, DOI 10.1111/j.1600-0838.2010.01154.x
   Silbernagel KG, 2012, AM J SPORT MED, V40, P1564, DOI 10.1177/0363546512447926
   Suydam SM, 2016, KNEE SURG SPORT TR A, V2013, P1
   Twaddle BC, 2007, AM J SPORT MED, V35, P2033, DOI 10.1177/0363546507307503
   Vandenbroucke JP, 2007, EPIDEMIOLOGY, V18, P805, DOI [10.1371/journal.pmed.0040297, 10.1097/EDE.0b013e3181577511]
   Walter SD, 1998, STAT MED, V17, P101, DOI 10.1002/(SICI)1097-0258(19980115)17:1<101::AID-SIM727>3.0.CO;2-E
   Weir JP, 2005, J STRENGTH COND RES, V19, P231, DOI 10.1519/15184.1
   Willits K, 2010, J BONE JOINT SURG AM, V92A, P2767, DOI 10.2106/JBJS.I.01401
NR 26
TC 16
Z9 16
U1 0
U2 4
PU ELSEVIER
PI AMSTERDAM
PA RADARWEG 29, 1043 NX AMSTERDAM, NETHERLANDS
SN 1268-7731
EI 1460-9584
J9 FOOT ANKLE SURG
JI Foot Ankle Surg.
PD DEC
PY 2017
VL 23
IS 4
BP 275
EP 280
DI 10.1016/j.fas.2016.08.002
PG 6
WC Orthopedics
WE Science Citation Index Expanded (SCI-EXPANDED)
SC Orthopedics
GA FO9ZV
UT WOS:000417258500017
PM 29202987
DA 2022-07-22
ER

PT J
AU Gorschewsky, O
   Pitzl, M
   Putz, A
   Klakow, A
   Neumann, W
AF Gorschewsky, O
   Pitzl, M
   Putz, A
   Klakow, A
   Neumann, W
TI Percutaneous repair of acute Achilles tendon rupture
SO FOOT & ANKLE INTERNATIONAL
LA English
DT Article
DE Achilles; percutaneous; rupture; tendon
ID OPEN SURGICAL REPAIR; OPERATIVE TREATMENT; MANAGEMENT; SUTURE
AB Various studies have shown that the operative treatment of a freshly ruptured Achilles tendon is generally considered to be more appropriate than a nonoperative regimen. However, complications in open reconstructions are reported to occur in 11-29%. The method used in this study reduced the risk of complications arising from operation, but simultaneously allowed early postoperative mobilization and functional treatment. It was a percutaneous repair of the Achilles tendon, using two Lengemann extension wires for coadaptation of the ruptured tendon. To fix the rupture site, the authors used a fibrin sealant. The spikes of the wire were hooked in at the fascia of the soleus muscle. Via a big, curved needle, the wire was placed in the distal stumps of the ruptured tendon and guided out laterally and medially above the calcaneus. After blocking the wires distally, the fibrin sealant was applied at the rupture site. The current report describes this method of treatment in 66 patients. The postoperative observation period was 1 year. Sixty-four patients were male and two were female. Their average age was 42 years. The Achilles tendon ruptures occurred during sporting activities and were treated by operation within 22 hours on average. The outcome was very good in 98%. One patient (2%) suffered a rerupture due to trauma. There were no other complications.
C1 Klin Sonnenhof, Sportothopad, CH-3006 Bern, Switzerland.
   Univ Magdeburg, Orthopad Klin, D-39106 Magdeburg, Germany.
RP Gorschewsky, O (通讯作者)，Klin Sonnenhof, Sportothopad, Buchserstr 30, CH-3006 Bern, Switzerland.
EM ottmargorschewsky@sonnenhof.ch
CR ARNDT KH, 1976, ACHILLESSEHNENRUPTUR, V10
   BLUME M, 1987, Beitraege zur Orthopaedie und Traumatologie, V34, P309
   BRADLEY JP, 1990, AM J SPORT MED, V18, P188, DOI 10.1177/036354659001800213
   CARDEN DG, 1987, J BONE JOINT SURG BR, V69, P416, DOI 10.1302/0301-620X.69B3.3294839
   CARTER TR, 1992, AM J SPORT MED, V20, P459, DOI 10.1177/036354659202000417
   CETTI R, 1993, AM J SPORT MED, V21, P791, DOI 10.1177/036354659302100606
   CHILLEMI C, 2002, FOOT ANKLE SURG, V8, P267
   DEDERICH R, 1988, UNFALLCHIRURG, V91, P259
   DELPONTE P, 1992, REV CHIR ORTHOP, V78, P404
   DELPONTE P, 1995, MED HYG, V53, P1549
   Dugan Dirk H., 1994, Journal of Athletic Training, V29, P349
   EDNA TH, 1980, ACTA ORTHOP SCAND, V51, P991, DOI 10.3109/17453678008990905
   EYB R, 1981, UNFALLHEILKUNDE, V84, P427
   FITZGIBBONS RE, 1993, AM J SPORT MED, V21, P724, DOI 10.1177/036354659302100516
   FRINGS H, 1969, ARCH ORTHOPAD UNFALL, V67, P64, DOI 10.1007/BF00417140
   GIGANTE K, LECT SICOT C 1996 AM
   GILLIES H, 1970, J BONE JOINT SURG AM, VA 52, P337, DOI 10.2106/00004623-197052020-00015
   Gorschewsky O, 1999, INJURY, V30, P315, DOI 10.1016/S0020-1383(99)00089-3
   HAAS F, 1987, ZBL CHIR, V112, P99
   INGLIS AE, 1976, J BONE JOINT SURG AM, V58, P990, DOI 10.2106/00004623-197658070-00015
   Jacobs D, 1978, Am J Sports Med, V6, P107, DOI 10.1177/036354657800600302
   KAKIUCHI M, 1995, J BONE JOINT SURG BR, V77B, P60, DOI 10.1302/0301-620X.77B1.7822398
   KOSANOVIC M, 1994, ARCH ORTHOP TRAUM SU, V113, P177, DOI 10.1007/BF00441827
   Lea R B, 1968, Clin Orthop Relat Res, V60, P115
   Lim J, 2001, FOOT ANKLE INT, V22, P559, DOI 10.1177/107110070102200705
   Lusardi D A, 1994, J Foot Ankle Surg, V33, P443
   MA GWC, 1977, CLIN ORTHOP RELAT R, P247
   MANDELBAUM BR, 1995, AM J SPORT MED, V23, P392, DOI 10.1177/036354659502300403
   MARINELLI B, 2000, B HOSP JOINT DIS, V59, P149
   PAAR O, 1984, FORTSCHR MED, V102, P1106
   PERCY EC, 1987, AM J SPORTS MED, V6, P132
   REDAELLI C, 1992, CHIRURG, V63, P572
   Riedl S, 2002, CHIRURG, V73, P607, DOI 10.1007/s00104-002-0452-2
   Scarfi G, 2002, FOOT ANKLE SURG, V8, P105
   SCHLAG G, 1988, CLIN ORTHOP RELAT R, P269
   Schwarz B, 1984, Aktuelle Traumatol, V14, P8
   STEIN SR, 1976, ORTHOP CLIN N AM, V7, P241
   WEINER AD, 1956, MINN MED, V39, P713
   ZWIPP H, 1989, UNFALLCHIRURG, V92, P554
NR 39
TC 24
Z9 24
U1 0
U2 0
PU SAGE PUBLICATIONS INC
PI THOUSAND OAKS
PA 2455 TELLER RD, THOUSAND OAKS, CA 91320 USA
SN 1071-1007
EI 1944-7876
J9 FOOT ANKLE INT
JI Foot Ankle Int.
PD APR
PY 2004
VL 25
IS 4
BP 219
EP 224
DI 10.1177/107110070402500405
PG 6
WC Orthopedics
WE Science Citation Index Expanded (SCI-EXPANDED)
SC Orthopedics
GA 854FC
UT WOS:000223885700005
PM 15132929
DA 2022-07-22
ER

PT J
AU Song, YJ
   Chen, G
   Jia, SH
   Xu, WB
   Hua, YH
AF Song, Yu-Jie
   Chen, Gang
   Jia, Shao-Hua
   Xu, Wei-Bin
   Hua, Ying-Hui
TI Good outcomes at mid-term following the reconstruction of chronic
   Achilles tendon rupture with semitendinosus allograft
SO KNEE SURGERY SPORTS TRAUMATOLOGY ARTHROSCOPY
LA English
DT Article
DE Achilles tendon; Chronic rupture; Reconstruction; Semitendinosus tendon
   allograft
ID RATING SYSTEMS; AUGMENTATION; AUTOGRAFT
AB Purpose Chronic Achilles tendon rupture is commonly treated surgically, yet there are limited available data on tendon allografts for Achilles tendon reconstruction. The purpose of this study was to evaluate the patient-reported outcomes of chronic Achilles tendon rupture reconstructed with semitendinosus allograft. Methods A total of 34 patients (30 males and 4 females, average age 36.1 +/- 6.8 years ranging from 25 to 50 years) who underwent Achilles tendon reconstruction with semitendinosus allograft from 2011 to 2015 were identified for this retrospective study. The American Orthopedic Foot and Ankle Society (AOFAS) score, Victorian Institute of Sports Assessment-Achilles (VISA-A) score, Achilles tendon total rupture score (ATRS), and Tegner score were evaluated preoperatively and at final follow-up. Results A total of 33 patients (97.1%) were followed up for a median time of 53 (range 24-80) months. The median AOFAS score increased from 50 (5-75) to 100 (86-100), the median VISA-A score increased from 23 (5-59) to 94 (52-100), the median ATRS score increased from 22.5 (6-67) to 99 (84-100), and the median Tegner score increased from 1 (0-3) to 4 (3-9). Conclusions Reconstruction of chronic-ruptured Achilles tendon with semitendinosus allograft offers satisfactory patient-reported results with low risk of re-rupture and complications. This technique could be considered an effective alternative for chronic ruptures of the Achilles tendon.
C1 [Song, Yu-Jie; Hua, Ying-Hui] Fudan Univ, Huashan Hosp, Dept Sports Med, Shanghai 200040, Peoples R China.
   [Chen, Gang; Jia, Shao-Hua; Xu, Wei-Bin] Jiaxing Univ, Affiliated Hosp 2, Dept Orthoped, Jiaxing 314000, Zhejiang, Peoples R China.
RP Hua, YH (通讯作者)，Fudan Univ, Huashan Hosp, Dept Sports Med, Shanghai 200040, Peoples R China.
EM 16211220058@fudan.edu.cn; adcyy@aliyun.com; jiashaohuajx@163.com;
   bingwuzhe@sina.com; hua_cosm@aliyun.com
RI Song, Yujie/AAG-6819-2019
CR ABRAHAM E, 1975, J BONE JOINT SURG AM, VA 57, P253, DOI 10.2106/00004623-197557020-00019
   ASSELMEIER MA, 1993, AM J SPORT MED, V21, P170, DOI 10.1177/036354659302100202
   Aynardi MC, 2017, CLIN ORTHOP RELAT R, V475, P2588, DOI 10.1007/s11999-017-5410-x
   BUCK BE, 1990, CLIN ORTHOP RELAT R, P249
   CARDEN DG, 1987, J BONE JOINT SURG BR, V69, P416, DOI 10.1302/0301-620X.69B3.3294839
   Dierckman BD, 2015, AM J SPORT MED, V43, P1941, DOI 10.1177/0363546515593942
   Guclu B, 2016, FOOT ANKLE INT, V37, P737, DOI 10.1177/1071100716642753
   Hua YH, 2012, INT ORTHOP, V36, P2027, DOI 10.1007/s00264-012-1577-7
   Ibrahim SAR, 2009, J FOOT ANKLE SURG, V48, P340, DOI 10.1053/j.jfas.2009.02.007
   Ibrahim Talal, 2007, J Foot Ankle Surg, V46, P65, DOI 10.1053/j.jfas.2006.12.002
   JACKSON DW, 1993, AM J SPORT MED, V21, P176, DOI 10.1177/036354659302100203
   Ji JH, 2007, FOOT ANKLE INT, V28, P1100, DOI 10.3113/FAI.2007.1100
   Kearney RS, 2010, FOOT ANKLE INT, V31, P556, DOI 10.3113/FAI.2010.0556
   KITAOKA HB, 1994, FOOT ANKLE INT, V15, P349, DOI 10.1177/107110079401500701
   Li QR, 2018, INT ORTHOP, V42, P551, DOI 10.1007/s00264-018-3802-5
   Maffulli N, 2008, J BONE JOINT SURG AM, V90A, P1348, DOI 10.2106/JBJS.G.01241
   Maffulli N, 2008, BMC MUSCULOSKEL DIS, V9, DOI 10.1186/1471-2474-9-100
   Malinin TI, 2002, ARTHROSCOPY, V18, P163, DOI 10.1053/jars.2002.30485
   Muller SA, 2018, AM J SPORT MED, V46, P1214, DOI 10.1177/0363546518756093
   Nilsson-Helander K, 2007, AM J SPORT MED, V35, P421, DOI 10.1177/0363546506294856
   Patil SSD, 2014, FOOT ANKLE INT, V35, P699, DOI 10.1177/1071100714531228
   Porter DA, 1997, FOOT ANKLE INT, V18, P557, DOI 10.1177/107110079701800905
   Robinson JM, 2001, BRIT J SPORT MED, V35, P335, DOI 10.1136/bjsm.35.5.335
   Sarzaeem MM, 2012, KNEE SURG SPORT TR A, V20, P1386, DOI 10.1007/s00167-011-1703-x
   Song YJ, 2019, FOOT ANKLE SURG, V25, P252, DOI 10.1016/j.fas.2018.02.002
   Su EP, 2003, CLIN ORTHOP RELAT R, P232, DOI 10.1097/01.blo.0000092976.12414.b0
   Takeuchi M, 2014, J MED INVESTIG, V61, P417, DOI 10.2152/jmi.61.417
   TEGNER Y, 1985, CLIN ORTHOP RELAT R, P43
   Wu ZY, 2015, KNEE SURG SPORT TR A, V23, P3756, DOI 10.1007/s00167-014-3176-1
NR 29
TC 4
Z9 5
U1 0
U2 3
PU SPRINGER
PI NEW YORK
PA ONE NEW YORK PLAZA, SUITE 4600, NEW YORK, NY, UNITED STATES
SN 0942-2056
EI 1433-7347
J9 KNEE SURG SPORT TR A
JI Knee Surg. Sports Traumatol. Arthrosc.
PD MAY
PY 2020
VL 28
IS 5
BP 1619
EP 1624
DI 10.1007/s00167-018-5113-1
PG 6
WC Orthopedics; Sport Sciences; Surgery
WE Science Citation Index Expanded (SCI-EXPANDED)
SC Orthopedics; Sport Sciences; Surgery
GA LH8KZ
UT WOS:000529032900036
PM 30128686
DA 2022-07-22
ER

PT J
AU Park, DY
   Rubenson, J
   Carr, A
   Mattson, J
   Besier, T
   Chou, LB
AF Park, Don Young
   Rubenson, Jonas
   Carr, Amelia
   Mattson, James
   Besier, Thor
   Chou, Loretta B.
TI Influence of Stretching and Warm-Up on Achilles Tendon Material
   Properties
SO FOOT & ANKLE INTERNATIONAL
LA English
DT Article
DE Achilles Tendon Injuries; Prevention Of Achilles Tendon Injuries;
   Stretching Achilles Tendon; Warm Up Achilles Tendon
ID STRUCTURES IN-VIVO; HUMAN GASTROCNEMIUS-MUSCLE; OF-THE-LITERATURE;
   VISCOELASTIC PROPERTIES; MECHANICAL-PROPERTIES; INJURY PREVENTION;
   MUSCULAR INJURY; LOWER-EXTREMITY; RISK; EXERCISES
AB Background: Controversy exists on stretching and warm-up in injury prevention. We hypothesized that warm up has a greater effect on Achilles tendon biomechanics than static stretching. This study investigated static stretching and warm-up on Achilles tendon biomechanics in recreational athletes, in vivo. Materials and Methods: Ten active, healthy subjects, 5 males, 5 females, With a mean age of 22.9 years with no previous Achilles tendon injuries were recruited. Typical stretching and warm-up routines were created. Testing was performed in a randomized cross-over design. A custom-built dynamometer was utilized to perform controlled isometric plantarflexion. A low profile ultrasound probe was utilized to visualize the musculotendinous Junction of the medial gastrocnemius. An eight-camera motion capture system was used to capture ankle motion. Custom software calculated Achilles tendon biomechanics. Results: Achilles tendon force production was consistent. No statistically significant differences were detected in stretch, stiffness, and strain between pre-, post-stretching, and post-warm-up interventions. Conclusion: Stretching or warm-up alone, and combined did not demonstrate statistically significant differences. Stretching and warm-up may have an equivalent effect on Achilles tendon biomechanics. Prolonged and more intense protocols may be required for changes to occur. Clinical Relevance: Stretching and warm-up of the Achilles before exercise are commonly practiced. Investigating the effect of stretching and warm-up may shed light on potential injury prevention.
C1 [Chou, Loretta B.] Stanford Univ, Med Ctr, Dept Orthopaed Surg, Redwood City, CA 94063 USA.
   [Rubenson, Jonas] Univ Western Australia, Dept Sport Sci Exercise & Hlth, Perth, WA 6009, Australia.
   [Carr, Amelia; Mattson, James] Stanford Univ, Human Performance Lab, Stanford, CA 94305 USA.
RP Chou, LB (通讯作者)，Stanford Univ, Med Ctr, Dept Orthopaed Surg, 450 Broadway St MC 6342, Redwood City, CA 94063 USA.
EM lchou@stanford.edu
RI Besier, Thor F/P-6425-2019
OI Besier, Thor/0000-0003-0818-7554; Rubenson, Jonas/0000-0003-2854-1776
CR Alfredson H, 2000, SPORTS MED, V29, P135, DOI 10.2165/00007256-200029020-00005
   Amako M, 2003, MIL MED, V168, P442, DOI 10.1093/milmed/168.6.442
   CARR AJ, 1989, J BONE JOINT SURG BR, V71, P100, DOI 10.1302/0301-620X.71B1.2914976
   Chen TM, 2009, CLIN ANAT, V22, P377, DOI 10.1002/ca.20758
   Delp SL, 2007, IEEE T BIO-MED ENG, V54, P1940, DOI 10.1109/TBME.2007.901024
   Fradkin AJ, 2006, J SCI MED SPORT, V9, P214, DOI 10.1016/j.jsams.2006.03.026
   GALLOWAY MT, 1992, CLIN SPORT MED, V11, P771
   Hedrick TL, 2008, BIOINSPIR BIOMIM, V3, DOI 10.1088/1748-3182/3/3/034001
   Hoang PD, 2007, J EXP BIOL, V210, P4159, DOI 10.1242/jeb.002204
   KOMI PV, 1992, CLIN SPORT MED, V11, P521
   Kubo K, 2005, J SPORT SCI, V23, P851, DOI 10.1080/02640410400022029
   Kubo K, 2002, J PHYSIOL-LONDON, V538, P219, DOI 10.1113/jphysiol.2001.012703
   Kubo K, 2002, J APPL PHYSIOL, V92, P595, DOI 10.1152/japplphysiol.00658.2001
   Kubo K, 2001, J APPL PHYSIOL, V90, P520, DOI 10.1152/jappl.2001.90.2.520
   Lichtwark GA, 2005, J EXP BIOL, V208, P4715, DOI 10.1242/jeb.01950
   Maganaris CN, 2003, CLIN ANAT, V16, P215, DOI 10.1002/ca.10064
   Mahieu NN, 2007, MED SCI SPORT EXER, V39, P494, DOI 10.1249/01.mss.0000247004.40212.f7
   Mahieu NN, 2006, AM J SPORT MED, V34, P226, DOI 10.1177/0363546505279918
   McCrory JL, 1999, MED SCI SPORT EXER, V31, P1374, DOI 10.1097/00005768-199910000-00003
   Park DY, 2006, FOOT ANKLE INT, V27, P1086, DOI 10.1177/107110070602701215
   Pope Rodney, 1998, Aust J Physiother, V44, P165
   Pope RP, 2000, MED SCI SPORT EXER, V32, P271, DOI 10.1097/00005768-200002000-00004
   Rosenbaum Dieter, 1995, Journal of Sports Sciences, V13, P481, DOI 10.1080/02640419508732265
   SAFRAN MR, 1988, AM J SPORT MED, V16, P123, DOI 10.1177/036354658801600206
   Shrier I, 1999, CLIN J SPORT MED, V9, P221, DOI 10.1097/00042752-199910000-00007
   Silder A, 2007, J BIOMECH, V40, P2628, DOI 10.1016/j.jbiomech.2006.12.017
   Small Katie, 2008, Res Sports Med, V16, P213, DOI 10.1080/15438620802310784
   STRICKLER T, 1990, AM J SPORT MED, V18, P141, DOI 10.1177/036354659001800206
   Thacker SB, 2004, MED SCI SPORT EXER, V36, P371, DOI 10.1249/01.MSS.0000117134.83018.F7
   VANMECHELEN W, 1993, AM J SPORT MED, V21, P711, DOI 10.1177/036354659302100513
   WIKTORSSONMOLLER M, 1983, AM J SPORT MED, V11, P249, DOI 10.1177/036354658301100412
   WILLIFORD HN, 1986, AM J SPORT MED, V14, P316, DOI 10.1177/036354658601400413
   Witvrouw E, 2004, SPORTS MED, V34, P443, DOI 10.2165/00007256-200434070-00003
   Woods K, 2007, SPORTS MED, V37, P1089, DOI 10.2165/00007256-200737120-00006
   Wren TAL, 2001, CLIN BIOMECH, V16, P245, DOI 10.1016/S0268-0033(00)00089-9
NR 35
TC 18
Z9 18
U1 1
U2 29
PU SAGE PUBLICATIONS INC
PI THOUSAND OAKS
PA 2455 TELLER RD, THOUSAND OAKS, CA 91320 USA
SN 1071-1007
EI 1944-7876
J9 FOOT ANKLE INT
JI Foot Ankle Int.
PD APR
PY 2011
VL 32
IS 4
BP 407
EP 413
DI 10.3113/FAI.2011.0407
PG 7
WC Orthopedics
WE Science Citation Index Expanded (SCI-EXPANDED)
SC Orthopedics
GA 742XV
UT WOS:000288979500015
PM 21733444
DA 2022-07-22
ER

PT J
AU Powell, HC
   Silbernagel, KG
   Brorsson, A
   Tranberg, R
   Willy, RW
AF Powell, Hayley C.
   Silbernagel, Karin Gravare
   Brorsson, Annelie
   Tranberg, Roy
   Willy, Richard W.
TI Individuals Post Achilles Tendon Rupture Exhibit Asymmetrical Knee and
   Ankle Kinetics and Loading Rates During a Drop Countermovement Jump
SO JOURNAL OF ORTHOPAEDIC & SPORTS PHYSICAL THERAPY
LA English
DT Article
DE ankle; biomechanics; jumping; knee; tendon
ID CRUCIATE LIGAMENT INJURY; HEEL-RISE HEIGHT; PATELLAR-TENDINOPATHY;
   MUSCLE-TENDON; OUTCOMES; REPAIR; STRATEGIES; STIFFNESS; ENDURANCE;
   DYNAMICS
AB STUDY DESIGN: Cross-sectional laboratory study.
   BACKGROUND: Asymmetrical knee loading during jogging and hopping has been reported in individuals who have ruptured their Achilles tendon. No studies have examined knee loads in individuals post Achilles tendon rupture during high-demand tasks, such as single-limb landings.
   OBJECTIVES: We sought to determine whether individuals post Achilles tendon rupture demonstrated asymmetrical knee loads and impact forces during drop countermovement jumps (CMJs).
   METHODS: Achilles tendon length and the single-leg heel-rise test for endurance were assessed in 34 individuals (31 male) 6.1 +/- 2.0 years post Achilles tendon rupture. Movement patterns were assessed during a drop CMJ. Data were analyzed via repeated-measures analyses of variance, with comparisons between limbs and prior treatment history (surgery versus nonsurgery).
   RESULTS: An 8.6% longer Achilles tendon (P<. 001) was found in the involved limb. During the single-leg heel-rise test, the involved limb demonstrated 22.4% less endurance and 14.6% lower heel-rise height (all, P<. 001). During the landing phase of the drop CMJ, the involved limb exhibited 39.6% greater loading rate (P<. 001), 16.8% greater eccentric knee power (P =.048), but 21.6% lower eccentric ankle power (P<. 001). During the take-off phase, the involved limb exhibited 12.1% lower jump height and 19.9% lower concentric ankle power (both, P<. 001).
   CONCLUSION: Elevated eccentric knee joint power and higher loading rates during a drop CMJ in individuals who experienced Achilles tendon rupture several years earlier may be a compensation pattern for reduced plantar flexor function. This movement pattern may place individuals who have had an Achilles tendon rupture at greater risk for knee injuries.
C1 [Powell, Hayley C.] BreakThrough Phys Therapy, Winston Salem, NC USA.
   [Silbernagel, Karin Gravare; Brorsson, Annelie; Tranberg, Roy] Univ Gothenburg, Sahlgrenska Acad, Inst Clin Sci, Dept Orthopaed, Gothenburg, Sweden.
   [Silbernagel, Karin Gravare] Univ Delaware, Dept Phys Therapy, Newark, DE USA.
   [Willy, Richard W.] Univ Montana, Sch Phys Therapy & Rehabil Sci, Missoula, MT 59812 USA.
RP Willy, RW (通讯作者)，Univ Montana, Coll Hlth Profess & Biomed Sci, Sch Phys Therapy & Rehabil Sci, 32 Campus Dr,Skaggs Bldg 108, Missoula, MT 59812 USA.
EM rich.willy@umontana.edu
RI Tranberg, Roy/AAD-8901-2020; Brorsson, Annelie/AAD-5986-2020
OI Gravare Silbernagel, Karin/0000-0001-7566-407X; Willy,
   Richard/0000-0002-1249-228X
FU Swedish Research Council for Sport Science
FX Prior to initiation of this study, the research protocol (058-14) was
   approved by the Regional Ethical Review Board in Gothenburg, Sweden.
   Funding for this study was provided by the Swedish Research Council for
   Sport Science. The authors certify that they have no affiliations with
   or financial involvement in any organization or entity with a direct
   financial interest in the subject matter or materials discussed in the
   article. Address correspondence to Dr Richard Willy, School of Physical
   Therapy and Rehabilitation Science, College of Health Professions and
   Biomedical Sciences, 32 Campus Drive, Skaggs Building 108, University of
   Montana, Missoula, MT 59812. E-mail: rich. willy@umontana.edu circle dot
   Copyright (c) 2018 Journal of Orthopaedic & Sports Physical Therapy (R)
CR Amin NH, 2013, AM J SPORT MED, V41, P1864, DOI 10.1177/0363546513490659
   ARNER O, 1959, Acta Chir Scand Suppl, V116, P1
   Benjaminse A, 2015, J ORTHOP SPORT PHYS, V45, P170, DOI 10.2519/jospt.2015.4986
   Bisseling RW, 2008, BRIT J SPORT MED, V42, P483, DOI 10.1136/bjsm.2007.044057
   Bisseling RW, 2007, BRIT J SPORT MED, V41, DOI 10.1136/bjsm.2006.032565
   Blackburn JT, 2009, J ATHL TRAINING, V44, P174, DOI 10.4085/1062-6050-44.2.174
   Blackmore T, 2016, J BIOMECH, V49, P479, DOI 10.1016/j.jbiomech.2015.12.019
   Bostick GP, 2010, J ORTHOP SPORT PHYS, V40, P345, DOI 10.2519/jospt.2010.3204
   Brorsson A, 2017, AM J SPORT MED, V45, P3060, DOI 10.1177/0363546517717698
   Chiu LZF, 2010, J APPL BIOMECH, V26, P508, DOI 10.1123/jab.26.4.508
   Crawford J, 2004, COCHRANE DB SYST REV, DOI DOI 10.1002/14651858.CD003674.PUB2
   DAVIS RB, 1991, HUM MOVEMENT SCI, V10, P575, DOI 10.1016/0167-9457(91)90046-Z
   DEMPSTER WT, 1959, AM J PHYS ANTHROPOL, V17, P289, DOI 10.1002/ajpa.1330170405
   Farley CT, 1998, J APPL PHYSIOL, V85, P1044, DOI 10.1152/jappl.1998.85.3.1044
   Ganestam A, 2016, KNEE SURG SPORT TR A, V24, P3730, DOI 10.1007/s00167-015-3544-5
   Geremia JM, 2015, CLIN BIOMECH, V30, P485, DOI 10.1016/j.clinbiomech.2015.03.005
   Grau S, 2008, J BIOMECH, V41, P2042, DOI 10.1016/j.jbiomech.2008.03.005
   Houshian S, 1998, INJURY, V29, P651, DOI 10.1016/S0020-1383(98)00147-8
   Jandacka D, 2013, BMJ CASE REP, V2013, P1
   Kristianslund E, 2012, J BIOMECH, V45, P666, DOI 10.1016/j.jbiomech.2011.12.011
   Malliaras P, 2015, J ORTHOP SPORT PHYS, V45, P887, DOI 10.2519/jospt.2015.5987
   McNair P, 2013, J ORTHOP RES, V31, P1469, DOI 10.1002/jor.22381
   Mundermann A, 2005, ARTHRITIS RHEUM-US, V52, P2835, DOI 10.1002/art.21262
   Newberry WN, 1997, J ORTHOP RES, V15, P450, DOI 10.1002/jor.1100150319
   Nilsson-Helander K, 2007, AM J SPORT MED, V35, P421, DOI 10.1177/0363546506294856
   Nilsson-Helander K, 2010, AM J SPORT MED, V38, P2186, DOI 10.1177/0363546510376052
   Noehren B, 2013, MED SCI SPORT EXER, V45, P1340, DOI 10.1249/MSS.0b013e318285c6b6
   Olsson N, 2014, SCAND J MED SCI SPOR, V24, P152, DOI 10.1111/j.1600-0838.2012.01497.x
   Olsson N, 2013, AM J SPORT MED, V41, P2867, DOI 10.1177/0363546513503282
   Olsson N, 2011, KNEE SURG SPORT TR A, V19, P1385, DOI 10.1007/s00167-011-1511-3
   Parekh Selene G, 2009, Foot Ankle Spec, V2, P283, DOI 10.1177/1938640009351138
   Paterno MV, 2007, CLIN J SPORT MED, V17, P258, DOI 10.1097/JSM.0b013e31804c77ea
   Paterno MV, 2010, AM J SPORT MED, V38, P1968, DOI 10.1177/0363546510376053
   Petit DJ, 2014, J APPL BIOMECH, V30, P173, DOI 10.1123/jab.2012-0147
   Porter DA, 2014, FOOT ANKLE INT, V35, P123, DOI 10.1177/1071100713514228
   Radin EL, 2004, J RHEUMATOL, V31, P10
   Riley PO, 2008, MED SCI SPORT EXER, V40, P1093, DOI 10.1249/MSS.0b013e3181677530
   Roos EM, 2001, FOOT ANKLE INT, V22, P788, DOI 10.1177/107110070102201004
   Schepull T, 2007, BMC MUSCULOSKEL DIS, V8, DOI 10.1186/1471-2474-8-116
   Schmitt LC, 2007, ARTHRIT RHEUM-ARTHR, V57, P1018, DOI 10.1002/art.22889
   Silbernagel KG, 2012, AM J SPORT MED, V40, P1564, DOI 10.1177/0363546512447926
   Silbernagel KG, 2012, J ORTHOP SPORT PHYS, V42, P521, DOI 10.2519/jospt.2012.3913
   Silbernagel KG, 2006, KNEE SURG SPORT TR A, V14, P1207, DOI 10.1007/s00167-006-0150-6
   Silbernagel KG, 2016, MLTJ-MUSCLE LIGAMENT, V6, P104, DOI 10.11138/mltj/2016.6.1.104
   Silbernagel KG, 2010, KNEE SURG SPORT TR A, V18, P258, DOI 10.1007/s00167-009-0889-7
   Suydam SM, 2015, KNEE SURG SPORT TR A, V23, P868, DOI 10.1007/s00167-013-2512-1
   Tengman T, 2013, ORTHOP J SPORTS MED, V1, DOI 10.1177/2325967113504734
   Wang HK, 2013, ARCH PHYS MED REHAB, V94, P1590, DOI 10.1016/j.apmr.2013.01.015
   Willits K, 2010, J BONE JOINT SURG AM, V92A, P2767, DOI 10.2106/JBJS.I.01401
   Willy RW, 2017, AM J SPORT MED, V45, P1124, DOI 10.1177/0363546516685055
   Woodard CM, 1999, J APPL BIOMECH, V15, P404, DOI 10.1123/jab.15.4.404
   Zellers JA, 2016, BRIT J SPORT MED, V50, P1325, DOI 10.1136/bjsports-2016-096106
NR 52
TC 10
Z9 10
U1 4
U2 11
PU J O S P T
PI ALEXANDRIA
PA 1111 NORTH FAIRFAX ST, STE 100, ALEXANDRIA, VA 22314-1436 USA
SN 0190-6011
EI 1938-1344
J9 J ORTHOP SPORT PHYS
JI J. Orthop. Sports Phys. Ther.
PD JAN
PY 2018
VL 48
IS 1
BP 34
EP 43
DI 10.2519/jospt.2018.7684
PG 10
WC Orthopedics; Rehabilitation; Sport Sciences
WE Science Citation Index Expanded (SCI-EXPANDED)
SC Orthopedics; Rehabilitation; Sport Sciences
GA FY1VJ
UT WOS:000426601200008
PM 29073841
OA Bronze
DA 2022-07-22
ER

PT J
AU Kongsgaard, M
   Nielsen, CH
   Hegnsvad, S
   Aagaard, P
   Magnusson, SP
AF Kongsgaard, M.
   Nielsen, C. H.
   Hegnsvad, S.
   Aagaard, P.
   Magnusson, S. P.
TI Mechanical properties of the human Achilles tendon, in vivo
SO CLINICAL BIOMECHANICS
LA English
DT Article
DE Achilles tendon; Ultrasonography; Mechanical properties; Reproducibility
ID LOAD-DISPLACEMENT PROPERTIES; TRICEPS SURAE APONEUROSIS; MEDIAL
   GASTROCNEMIUS; ELASTIC PROPERTIES; PATELLAR TENDON; HUMAN SOLEUS;
   MUSCLE; STRAIN; BEHAVIOR; RUPTURES
AB Background: Ultrasonography has been widely applied for in vivo measurements of tendon mechanical properties. Assessments of human Achilles tendon mechanical properties have received great interest. Achilles tendon injuries predominantly occur in the tendon region between the Achilles-soleus myotendinous junction and Achilles-calcaneus osteotendinous junction i.e. in the free Achilles tendon. However, there has been no adequate ultrasound based method for quantifying the mechanical properties of the free human Achilles tendon. This study aimed to: 1) examine the mechanical properties of the free human Achilles tendon in vivo by the use of ultrasonography and 2) assess the between-day reproducibility of these measurements.
   Methods: Ten male subjects had the Achilles tendon moment arm length. Achilles tendon cross sectional area and free Achilles tendon length determined. All subjects performed isometric plantarflexion ramp contractions to assess between-day reproducibility on two separate days. Simultaneous ultrasonography based measurements of Achilles-soleus myotendinous junction and Achilles-calcaneus osteotendinous junction displacement together with Achilles tendon force estimates yielded free Achilles tendon mechanical properties.
   Findings: Free Achilles tendon maximal force, deformation and stiffness were 1924 (SD 229) N. 2.2 (SD 0.6) mm and 2622 (SD 534) N/mm on day 1. For between-day reproducibility there were no significant differences between days for free Achilles tendon mechanical properties. The between-day correlation coefficient and typical error percent were 0.81 and 5.3% for maximal Achilles tendon force, 0.85 and 11.8% for maximal Achilles tendon deformation and 0.84 and 8.8% for Achilles tendon stiffness respectively. Last, osteotendinous junction proximal displacement on average contributed with 71 (SD 12) % of proximal myotendinous junction displacement.
   Interpretation: This study, for the first time, presents an ultrasonography based in vivo method for measurement of free AT mechanical properties. The method is applicable for evaluation of free human Achilles tendon mechanical properties in relation to training, injury and rehabilitation. (C) 2011 Elsevier Ltd. All rights reserved.
C1 [Kongsgaard, M.; Nielsen, C. H.; Hegnsvad, S.; Magnusson, S. P.] Bispebjerg Hosp, Dept Othoped Surg M, Inst Sports Med, DK-2400 Copenhagen, Denmark.
   [Magnusson, S. P.] Bispebjerg Hosp, Dept Physiotherapy, DK-2400 Copenhagen, Denmark.
   [Kongsgaard, M.; Nielsen, C. H.; Hegnsvad, S.; Magnusson, S. P.] Univ Copenhagen, Fac Hlth Sci, Ctr Healthy Aging, DK-1168 Copenhagen, Denmark.
   [Aagaard, P.] Univ So Denmark, Inst Sports Exercise & Clin Biomech, Odense, Denmark.
RP Kongsgaard, M (通讯作者)，Bispebjerg Hosp, Dept Othoped Surg M, Inst Sports Med, DK-2400 Copenhagen, Denmark.
EM mads.kongsgaard@gmail.com
RI Magnusson, S. Peter/AAD-5903-2022; Magnusson, Peter/AAN-1631-2020;
   CHASSAGNE, Fanette/B-7212-2012
OI Magnusson, S. Peter/0000-0002-4308-5484; Aagaard,
   Per/0000-0002-9773-7361
FU Danish medical Research Counsil; Lundbeck Foundation; Novo Nordisk
   Foundation
FX This work was funded by grants from the Danish medical Research Counsil,
   The Lundbeck Foundation and the Novo Nordisk Foundation. The study
   sponsors had no involvement in study design, study management, data
   collection, data analysis or publication.
CR Arampatzis A, 2007, J BIOMECH, V40, P1946, DOI 10.1016/j.jbiomech.2006.09.005
   Arampatzis A, 2010, J BIOMECH, V43, P3073, DOI 10.1016/j.jbiomech.2010.08.014
   Arya S, 2010, J APPL PHYSIOL, V108, P670, DOI 10.1152/japplphysiol.00259.2009
   Bojsen-Moller J, 2004, J APPL PHYSIOL, V97, P1908, DOI 10.1152/japplphysiol.00084.2004
   Bryant AL, 2008, J APPL PHYSIOL, V105, P1035, DOI 10.1152/japplphysiol.01281.2007
   Calleja M, 2010, SEMIN MUSCULOSKEL R, V14, P307, DOI 10.1055/s-0030-1254520
   Carroll CC, 2008, J APPL PHYSIOL, V105, P1907, DOI 10.1152/japplphysiol.00059.2008
   Child S, 2010, AM J SPORT MED, V38, P1885, DOI 10.1177/0363546510366234
   CLEMENT DB, 1984, AM J SPORT MED, V12, P179, DOI 10.1177/036354658401200301
   Cook JL, 2002, MANUAL THER, V7, P121, DOI 10.1054/math.2002.0458
   Csapo R, 2010, J EXP BIOL, V213, P2582, DOI 10.1242/jeb.044271
   Finni T, 2003, J APPL PHYSIOL, V95, P829, DOI 10.1152/japplphysiol.00775.2002
   Foure A, 2010, J APPL PHYSIOL, V109, P849, DOI 10.1152/japplphysiol.01150.2009
   Hansen P, 2006, CLIN BIOMECH, V21, P54, DOI 10.1016/j.clinbiomech.2005.07.008
   Hansen P, 2003, J APPL PHYSIOL, V95, P2375, DOI 10.1152/japplphysiol.00503.2003
   Hopkins WG, 2000, SPORTS MED, V30, P1, DOI 10.2165/00007256-200030010-00001
   KER RF, 1988, J ZOOL, V216, P309, DOI 10.1111/j.1469-7998.1988.tb02432.x
   Kinugasa R, 2008, J APPL PHYSIOL, V105, P1312, DOI 10.1152/japplphysiol.90440.2008
   Kinugasa R, 2010, J APPL PHYSIOL, V109, P870, DOI 10.1152/japplphysiol.00384.2010
   KOMI PV, 1992, CLIN SPORT MED, V11, P521
   KOMI PV, 1987, INT J SPORTS MED, V8, P1
   Kongsgaard M, 2007, ACTA PHYSIOL, V191, P111, DOI 10.1111/j.1748-1716.2007.01714.x
   Kubo K, 2000, EUR J APPL PHYSIOL O, V81, P181, DOI 10.1007/s004210050028
   Lichtwark GA, 2005, J EXP BIOL, V208, P4715, DOI 10.1242/jeb.01950
   LIEBER RL, 1991, AM J PHYSIOL, V261, pC86, DOI 10.1152/ajpcell.1991.261.1.C86
   Longo UG, 2009, SPORTS MED ARTHROSC, V17, P127, DOI 10.1097/JSA.0b013e3181a3d767
   Maganaris CN, 2000, J BIOMECH, V33, P1453, DOI 10.1016/S0021-9290(00)00099-3
   Maganaris CN, 2002, J BIOMECH, V35, P1639, DOI 10.1016/S0021-9290(02)00240-3
   Maganaris CN, 2005, J BIOMECH, V38, P9, DOI 10.1016/j.jbiomech.2004.03.024
   Magnusson SP, 2003, ACTA PHYSIOL SCAND, V177, P185, DOI 10.1046/j.1365-201X.2003.01048.x
   Magnusson SP, 2001, J PHYSIOL-LONDON, V531, P277, DOI 10.1111/j.1469-7793.2001.0277j.x
   Muraoka T, 2005, J APPL PHYSIOL, V99, P665, DOI 10.1152/japplphysiol.00624.2004
   Rosager S, 2002, SCAND J MED SCI SPOR, V12, P90, DOI 10.1034/j.1600-0838.2002.120205.x
   Sandmeier R, 1997, SCAND J MED SCI SPOR, V7, P96
   Schepull T, 2012, SCAND J MED SCI SPOR, V22, P18, DOI 10.1111/j.1600-0838.2010.01154.x
   Schepull T, 2007, BMC MUSCULOSKEL DIS, V8, DOI 10.1186/1471-2474-8-116
   Scholz MN, 2008, J EXP BIOL, V211, P3266, DOI 10.1242/jeb.018812
   SCOTT SH, 1990, MED SCI SPORT EXER, V22, P357
   Shin D, 2008, J APPL PHYSIOL, V105, P1179, DOI 10.1152/japplphysiol.90699.2008
   Zhao H, 2009, J APPL PHYSIOL, V106, P843, DOI 10.1152/japplphysiol.91212.2008
   ZUURBIER CJ, 1994, J BIOMECH, V27, P445, DOI 10.1016/0021-9290(94)90020-5
NR 41
TC 79
Z9 79
U1 4
U2 72
PU ELSEVIER SCI LTD
PI OXFORD
PA THE BOULEVARD, LANGFORD LANE, KIDLINGTON, OXFORD OX5 1GB, OXON, ENGLAND
SN 0268-0033
EI 1879-1271
J9 CLIN BIOMECH
JI Clin. Biomech.
PD AUG
PY 2011
VL 26
IS 7
BP 772
EP 777
DI 10.1016/j.clinbiomech.2011.02.011
PG 6
WC Engineering, Biomedical; Orthopedics; Sport Sciences
WE Science Citation Index Expanded (SCI-EXPANDED)
SC Engineering; Orthopedics; Sport Sciences
GA 801GE
UT WOS:000293425700013
PM 21392868
DA 2022-07-22
ER

PT J
AU Bunch, TJ
   Welsh, GA
   Miller, DV
   Swaroop, VS
AF Bunch, TJ
   Welsh, GA
   Miller, DV
   Swaroop, VS
TI Acute spontaneous Achilles tendon rupture in a patient with giant cell
   arteritis
SO ANNALS OF CLINICAL AND LABORATORY SCIENCE
LA English
DT Article
DE temporal arteritis; Achilles tendon; ruptured tendon; tendonitis;
   prednisone
ID BILATERAL RUPTURE
AB We report a case of a 69-yr-old previously healthy man with acute spontaneous Achilles tendon rupture and severe tendonitis, which occurred after 2 weeks of steroid therapy for newly diagnosed giant cell arteritis. The Achilles tendon rupture was treated conservatively and the tendonitis resolved incrementally with steroid dose reduction. The patient made a complete recovery. In view of the widespread use of steroids in practice, this novel case presentation has important clinical implications. The tendon rupture early in the course of high-dose steroid therapy expands the understanding of this adverse reaction, which was previously reported only with long-term steroid therapy. The severe tendonitis responded to steroid therapy reduction suggesting a dose correlation. This report adds to a sole previous report of a spontaneous Achilles tendon rupture associated with giant cell arteritis.
C1 Mayo Clin & Mayo Fdn, Dept Internal Med, Rochester, MN 55905 USA.
   Mayo Clin & Mayo Fdn, Div Anat Pathol, Rochester, MN 55905 USA.
RP Swaroop, VS (通讯作者)，Mayo Clin & Mayo Fdn, Dept Internal Med, 200 1st St SW, Rochester, MN 55905 USA.
CR ASBOEHANSEN G, 1976, DERMATOLOGICA, V152, P127, DOI 10.1159/000257873
   FEST T, 1989, LANCET, V14, P918
   HAINES JF, 1983, ANN RHEUM DIS, V42, P652, DOI 10.1136/ard.42.6.652
   Khurana R, 2002, RESPIROLOGY, V7, P161, DOI 10.1046/j.1440-1843.2002.00381.x
   Kotnis RA, 1999, J ACCID EMERG MED, V16, P378
   NEWNHAM DM, 1991, THORAX, V46, P853, DOI 10.1136/thx.46.11.853
   POTASMAN I, 1984, ANN RHEUM DIS, V43, P347, DOI 10.1136/ard.43.2.347
   1989, LANCET, V24, P1427
NR 8
TC 6
Z9 6
U1 0
U2 0
PU INST CLINICAL SCIENCE INC
PI PHILADELPHIA
PA 1833 DELANCEY PLACE, PHILADELPHIA, PA 19103 USA
SN 0091-7370
J9 ANN CLIN LAB SCI
JI Ann. Clin. Lab. Sci.
PD SUM
PY 2003
VL 33
IS 3
BP 326
EP 328
PG 3
WC Medical Laboratory Technology
WE Science Citation Index Expanded (SCI-EXPANDED)
SC Medical Laboratory Technology
GA 714GN
UT WOS:000184904700014
PM 12956450
DA 2022-07-22
ER

PT J
AU Chen, Z
   Wang, J
   Wei, JS
   Hou, ZY
   Li, M
   Chen, QX
AF Chen, Zhe
   Wang, Jun
   Wei, Jia-Sen
   Hou, Zhao-Yang
   Li, Min
   Chen, Qi-Xin
TI Biomechanical evaluation of tendon connection with novel suture
   techniques
SO ACTA OF BIOENGINEERING AND BIOMECHANICS
LA English
DT Article
DE internal fixation with limited loop; Achilles tendon; biomechanics
ID CRUCIATE LIGAMENT RECONSTRUCTION; ACHILLES-TENDON; SURGICAL TECHNIQUE;
   RUPTURE; REPAIR; FIXATION; TENSION; GRAFTS
AB Purpose: Achilles tendon rupture is a severe injury with poor curative effect due to its anatomical characteristic and mechanical peculiarity . Internal fixation of limited loop (IFLL) with steel-wire has been applied on patients with tendon rupture to fix the broken ends before physical rehabilitation. The purpose of this study is to investigate the biomechanical property and radiological characteristic of such suture technique for the repairment of tendon rupture. Methods: Tendons of pigs' hint feet were separated for the biomechanical study Suture surgery was performed according to the protocol of IFLL. Biomechanical Testing Machine was adopted to conduct the biomechanical tensile load examination. The maximal load, elastic modulus and tendon stiffness of the stitched tendons with or without remforcement were examined. Results: The maximum tensile load of the stitched tendons using IFLL reached 1/4 of the uninjured tendon's maximum tensile load, indicating that such suture technique is capable of providing enough tension for the ruptured tendon. Surprisingly, tendons fixed with titanium wire showed the highest load tension, which was comparable to the undamaged tendon. Therefore, we found the biomechanical basis of using IFLL in effectively connecting the rupture ends of tendons. Conclusions: In conclusion, we provide biomechanical evidence for the use of IFLL in treatment of Achilles tendon rupture, by providing enough strength for the ankle function. Such suture technique could help the patients with better rehabilitation and reduced m-hospital stay after Achilles tendon injury.
C1 [Chen, Zhe; Wang, Jun; Wei, Jia-Sen; Hou, Zhao-Yang] Zhejiang Chinese Med Univ, Dept Orthoped, Affiliated Hosp 2, Hangzhou 310005, Zhejiang, Peoples R China.
   [Li, Min] Zhejiang Chinese Med Univ, Biomech Lab, Hangzhou, Zhejiang, Peoples R China.
   [Chen, Qi-Xin] Zhejiang Univ, Dept Orthoped, Affiliated Hosp 2, Hangzhou, Zhejiang, Peoples R China.
RP Chen, Z (通讯作者)，Zhejiang Chinese Med Univ, Dept Orthoped, Affiliated Hosp 2, Hangzhou 310005, Zhejiang, Peoples R China.
EM 375523808@qq.com
FU Scientific Research Planned Project of Taizhou Science and Technology
   Bureau in Zhejiang Province [031212]
FX This work was supported by Scientific Research Planned Project of
   Taizhou Science and Technology Bureau in Zhejiang Province (031212).
CR Andrzejewski T, 2017, ACTA BIOENG BIOMECH, V19, P167, DOI 10.5277/ABB-00521-2015-03
   Badalihan A, 2015, J FOOT ANKLE SURG, V54, P153, DOI 10.1053/j.jfas.2014.12.002
   Bukvic N, 2013, J PEDIATR ORTHOP B, V22, P372, DOI 10.1097/BPB.0b013e328360f8df
   Chen Z, 2013, ASIAN PAC J TROP MED, V6, P902, DOI 10.1016/S1995-7645(13)60161-3
   Ciccone WJ, 2006, J BONE JOINT SURG AM, V88A, P1071, DOI 10.2106/JBJS.E.00576
   Erdemir A, 2004, J BONE JOINT SURG AM, V86A, P546, DOI 10.2106/00004623-200403000-00013
   Gigante A, 2008, KNEE SURG SPORT TR A, V16, P204, DOI 10.1007/s00167-007-0448-z
   Groetelaers RPTGC, 2014, FOOT ANKLE INT, V35, P771, DOI 10.1177/1071100714536167
   Han XS, 2014, J BIOMECH, V47, P2406, DOI 10.1016/j.jbiomech.2014.04.022
   Ingvar J, 2005, ACTA ORTHOP, V76, P597, DOI 10.1080/17453670510041619
   Jallageas R, 2013, ORTHOP TRAUMATOL-SUR, V99, P577, DOI 10.1016/j.otsr.2013.03.024
   陈世益, 2004, [中国运动医学杂志, Chinese Journal of Sports Medicine], V23, P283
   Jielile J, 2012, FOOT ANKLE INT, V33, P1119, DOI 10.3113/FAI.2012.1119
   Kanz Brian N, 2014, Foot Ankle Spec, V7, P176, DOI 10.1177/1938640014528041
   Lichtwark GA, 2005, J EXP BIOL, V208, P4715, DOI 10.1242/jeb.01950
   Lohan A, 2013, CONNECT TISSUE RES, V54, P306, DOI 10.3109/03008207.2013.820283
   Nagasawa K, 2008, CLIN BIOMECH, V23, P832, DOI 10.1016/j.clinbiomech.2008.02.002
   Nurmi JT, 2004, AM J SPORT MED, V32, P418, DOI 10.1177/0363546503261703
   Soroceanu A, 2012, J BONE JOINT SURG AM, V94A, P2136, DOI 10.2106/JBJS.K.00917
   Strickland, 1995, J Am Acad Orthop Surg, V3, P55
   Tucker JJ, 2017, J SHOULDER ELB SURG, V26, P529, DOI 10.1016/j.jse.2016.09.006
   Tudisco C, 2012, J ORTHOP TRAUMATOL, V13, P163, DOI 10.1007/s10195-012-0178-y
   Wang G, 2015, PLOS ONE, V10, DOI 10.1371/journal.pone.0132046
NR 23
TC 2
Z9 2
U1 1
U2 9
PU WROCLAW UNIV TECHNOLOGY
PI WROCLAW
PA WROCLAW UNIV TECHNOLOGY, MECHANICAL DEPT, WYBRZEZE WYSPIANSKIEGO 27,
   WROCLAW, 50-370, POLAND
SN 1509-409X
J9 ACTA BIOENG BIOMECH
JI Acta Bioeng. Biomech.
PY 2018
VL 20
IS 1
BP 135
EP 141
DI 10.5277/ABB-01024-2017-03
PG 7
WC Biophysics; Engineering, Biomedical
WE Science Citation Index Expanded (SCI-EXPANDED)
SC Biophysics; Engineering
GA GG3XV
UT WOS:000432627900014
PM 29658519
DA 2022-07-22
ER

PT J
AU Yamaguchi, S
   Kimura, S
   Akagi, R
   Yoshimura, K
   Kawasaki, Y
   Shiko, Y
   Sasho, T
   Ohtori, S
AF Yamaguchi, Satoshi
   Kimura, Seiji
   Akagi, Ryuichiro
   Yoshimura, Kensuke
   Kawasaki, Yohei
   Shiko, Yuki
   Sasho, Takahisa
   Ohtori, Seiji
TI Increase in Achilles Tendon Rupture Surgery in Japan: Results From a
   Nationwide Health Care Database
SO ORTHOPAEDIC JOURNAL OF SPORTS MEDICINE
LA English
DT Article
DE Achilles tendon; Achilles tendon rupture; Achilles tendon rupture
   incidence; Achilles tendon surgery; epidemiology; foot; national
   database; tendon rupture
ID NONOPERATIVE TREATMENT; NONSURGICAL TREATMENT; DISTAL RADIUS;
   EPIDEMIOLOGY; MANAGEMENT; FRACTURES; TRENDS
AB Background: Nationwide epidemiologic studies in Scandinavian countries have shown that the incidence of Achilles tendon ruptures (ATRs) has increased, and the rate of surgical treatment has declined markedly in the past decade. However, there is a lack of national-level data on the trend of ATRs and surgical procedures in other regions. Purpose: To clarify the trend in the incidence of ATRs and the proportion of surgery using the nationwide health care database in Japan. Study Design: Descriptive epidemiology study. Methods: Age- and sex-stratified data on the annual number of ATRs and surgical procedures between 2010 and 2017 were obtained from the Japanese national health care database, which includes almost all inpatient and outpatient medical claims nationwide. The Japanese population data were also obtained from the population census. The change in the annual incidence of ATRs per 100,000 people was assessed using a Poisson regression analysis. The trend in the annual proportion of surgeries relative to the occurrence of tendon ruptures was determined using a linear regression analysis. Results: A total of 112,601 ATRs, with men accounting for 67%, were identified over 8 years. Patients aged >= 60 years accounted for 27,106 (24%), while those aged 20 to 39 years and 40 to 59 years accounted for 36,164 (32%) and 49,331 (44%), respectively. The annual incidence of ATR ranged from 12.8/100,000 to 13.9/100,000 (women, 8.2-8.9/100,000; men, 17.2-19.5/100,000), which did not change over the study period (P = .82). Moreover, the annual incidences did not change across sexes and age categories. The annual proportion of surgery increased significantly, from 67% in 2010 to 72% in 2017 (P = .003). The annual proportions increased across sexes and age categories except for women aged 40 to 59 years. Conclusion: The incidence of ATR did not change between 2010 and 2017, according to the Japanese nationwide health care database. Furthermore, the proportion of surgical treatment increased during the study period. Overall, 70% of patients underwent surgical treatment. This study suggested that the trend in ATR and surgery differed across regions.
C1 [Yamaguchi, Satoshi; Kimura, Seiji; Akagi, Ryuichiro; Yoshimura, Kensuke; Kawasaki, Yohei; Shiko, Yuki; Sasho, Takahisa; Ohtori, Seiji] Chiba Univ, Grad Sch Med & Pharmaceut Sci, Dept Orthopaed Surg, Chiba, Japan.
   [Yamaguchi, Satoshi] Chiba Univ, Grad Sch Global & Transdisciplinary Studies, Chiba, Japan.
   [Yoshimura, Kensuke] Chiba Univ Hosp, Ctr Next Generat Community Hlth, Chiba, Japan.
   [Kawasaki, Yohei; Shiko, Yuki] Chiba Univ Hosp, Clin Res Ctr, Biostat Sect, Chiba, Japan.
   [Sasho, Takahisa] Chiba Univ, Ctr Prevent Med Sci, Chiba, Japan.
RP Yamaguchi, S (通讯作者)，Chiba Univ, Grad Sch Med & Pharmaceut Sci, Dept Orthopaed Surg, Chuo Ku, 1-8-1 Inohana, Chiba, Chiba 2608670, Japan.
EM y-satoshi@mvb.biglobe.ne.jp
OI Shiko, Yuki/0000-0002-3959-9343
CR [Anonymous], Survey of medical institutions 2017
   [Anonymous], Eurobarometer 412: sport and physical activity
   [Anonymous], NATL HLTH NUTR SURVE
   [Anonymous], 2018, SURV SPORTS PART
   [Anonymous], POP CENS
   Chooi YC, 2019, METABOLISM, V92, P6, DOI 10.1016/j.metabol.2018.09.005
   Chung CP, 2013, VACCINE, V31, pK41, DOI 10.1016/j.vaccine.2013.03.075
   Claessen FMAP, 2014, SPORTS MED, V44, P1241, DOI 10.1007/s40279-014-0200-z
   Clayton RAE, 2008, INJURY, V39, P1338, DOI 10.1016/j.injury.2008.06.021
   Costa ML, 2016, BONE JOINT J, V98B, P410, DOI 10.1302/0301-620X.98B3.36730
   Erickson BJ, 2015, ORTHOP J SPORTS MED, V3, DOI 10.1177/2325967115579188
   Erickson BJ, 2014, ORTHOP J SPORTS MED, V2, DOI 10.1177/2325967114549948
   Ganestam A, 2016, KNEE SURG SPORT TR A, V24, P3730, DOI 10.1007/s00167-015-3544-5
   Hayashi S, 2019, BONE, V120, P512, DOI 10.1016/j.bone.2018.12.014
   Ho G, 2017, INJURY, V48, P1701, DOI 10.1016/j.injury.2017.04.007
   Houshian S, 1998, INJURY, V29, P651, DOI 10.1016/S0020-1383(98)00147-8
   Hudson M, 2013, J CLIN EPIDEMIOL, V66, P278, DOI 10.1016/j.jclinepi.2012.10.004
   Hulteen RM, 2017, PREV MED, V95, P14, DOI 10.1016/j.ypmed.2016.11.027
   Huttunen TT, 2014, AM J SPORT MED, V42, P2419, DOI 10.1177/0363546514540599
   Katano H, 2018, J ORTHOP SCI, V23, P676, DOI 10.1016/j.jos.2018.04.003
   Lantto I, 2015, SCAND J MED SCI SPOR, V25, pE133, DOI 10.1111/sms.12253
   Lemme NJ, 2018, ORTHOP J SPORTS MED, V6, DOI 10.1177/2325967118808238
   Levi N, 1997, INJURY, V28, P311, DOI 10.1016/S0020-1383(96)00200-8
   Longo UG, 2020, BMC MUSCULOSKEL DIS, V21, DOI 10.1186/s12891-020-03688-2
   Maffulli N, 1999, CLIN J SPORT MED, V9, P157, DOI 10.1097/00042752-199907000-00007
   Mattila VM, 2015, BRIT J SPORT MED, V49, P1084, DOI 10.1136/bjsports-2013-092756
   Mc Colgan R, 2019, BONE JOINT J, V101B, P1550, DOI 10.1302/0301-620X.101B12.BJJ-2018-1615.R3
   Moller A, 1996, ACTA ORTHOP SCAND, V67, P479, DOI 10.3109/17453679608996672
   Nilsson-Helander K, 2010, AM J SPORT MED, V38, P2186, DOI 10.1177/0363546510376052
   Noback PC, 2017, INJURY, V48, P2342, DOI 10.1016/j.injury.2017.08.050
   Nyyssonen T, 2008, SCAND J SURG, V97, P272, DOI 10.1177/145749690809700312
   Ochen Y, 2019, BMJ-BRIT MED J, V364, DOI 10.1136/bmj.k5120
   Olsson N, 2013, AM J SPORT MED, V41, P2867, DOI 10.1177/0363546513503282
   Sheth U, 2017, BONE JOINT J, V99B, P78, DOI [10.1302/0301-620X.99B1.BJJ-2016-0434.R1, 10.1302/0301-620x.99b1.bjj-2016-0434.r1]
   Soroceanu A, 2012, J BONE JOINT SURG AM, V94A, P2136, DOI 10.2106/JBJS.K.00917
   Suchak AA, 2005, FOOT ANKLE INT, V26, P932, DOI 10.1177/107110070502601106
   Tamaki J, 2019, OSTEOPOROSIS INT, V30, P975, DOI 10.1007/s00198-019-04844-8
   Terris DD, 2007, J CLIN EPIDEMIOL, V60, P734, DOI 10.1016/j.jclinepi.2006.08.012
   Tikkanen R, 2020, INT PROFILES HLTH CA
   van Walraven C, 2011, J CLIN EPIDEMIOL, V64, P1054, DOI 10.1016/j.jclinepi.2011.01.001
   Wang D, 2015, FOOT ANKLE SURG, V21, P250, DOI 10.1016/j.fas.2015.01.009
   Willits K, 2010, J BONE JOINT SURG AM, V92A, P2767, DOI 10.2106/JBJS.I.01401
   Zhang H, 2015, MEDICINE, V94, DOI 10.1097/MD.0000000000001951
NR 43
TC 1
Z9 1
U1 0
U2 0
PU SAGE PUBLICATIONS INC
PI THOUSAND OAKS
PA 2455 TELLER RD, THOUSAND OAKS, CA 91320 USA
EI 2325-9671
J9 ORTHOP J SPORTS MED
JI Orthop. J. Sports Med.
PD OCT
PY 2021
VL 9
IS 10
AR 23259671211034128
DI 10.1177/23259671211034128
PG 7
WC Orthopedics; Sport Sciences
WE Science Citation Index Expanded (SCI-EXPANDED)
SC Orthopedics; Sport Sciences
GA WM0MS
UT WOS:000710790000001
PM 34708136
OA Green Published, gold
DA 2022-07-22
ER

PT J
AU Heinzelmann, AD
   Barrett, GR
AF Heinzelmann, Andrew D.
   Barrett, Gene R.
TI Posterior Cruciate Ligament Reconstruction: Achilles Tendon Allograft,
   Double Bundle
SO CLINICS IN SPORTS MEDICINE
LA English
DT Article
DE Posterior cruciate ligament; Allograft; Achilles tendon
ID POSTEROLATERAL ROTATORY INSTABILITY; TISSUE REGULATION; TIBIAL-INLAY;
   KNEE-JOINT; INJURIES; ANTERIOR; PROCUREMENT; RUPTURE
AB Allograft usage for cruciate ligament reconstruction has gained in popularity. Many techniques are described for posterior cruciate reconstruction with both autograft and allograft tendons. Achilles tendon allograft is a versatile and effective graft that can be used for a transtibial, double femoral bundle posterior cruciate reconstruction.
C1 [Heinzelmann, Andrew D.; Barrett, Gene R.] Mississippi Sports Med & Orthopaed Ctr, Jackson, MS USA.
RP Barrett, GR (通讯作者)，Mississippi Sports Med & Orthopaed Ctr, Jackson, MS USA.
EM gbarrett@msmoc.com
CR Ahn JH, 2005, ARTHROSCOPY, V21, P965, DOI 10.1016/j.arthro.2005.05.004
   Amis AA, 2006, KNEE SURG SPORT TR A, V14, P257, DOI 10.1007/s00167-005-0686-x
   ASSELMEIER MA, 1993, AM J SPORT MED, V21, P170, DOI 10.1177/036354659302100202
   Battaglia T.C., 2006, CLIN SPORTS MED, P547
   BERG EE, 1995, ARTHROSCOPY, V11, P69, DOI 10.1016/0749-8063(95)90091-8
   BERGFELD JA, 1990, INSTR COURSE LECT AA, V208
   BULLIS DW, 1994, CLIN SPORT MED, V13, P581
   CLANCY WG, 1983, MED SCI SPORT EXER, V15, P9
   CLANCY WG, 1983, J BONE JOINT SURG AM, V65, P310, DOI 10.2106/00004623-198365030-00004
   Clancy WG, 1988, OPERATIVE ORTHOPAEDI, P1651
   Cohen SB, 2007, CLIN SPORT MED, V26, P597, DOI 10.1016/j.csm.2007.06.003
   Cosgarea A J, 2001, J Am Acad Orthop Surg, V9, P297
   CROSS MJ, 1984, AM J SPORT MED, V12, P292, DOI 10.1177/036354658401200409
   DeBerardino TM, 2008, AM J SPORT MED, V36, P142, DOI 10.1177/0363546507307393
   DELEE JC, 1983, AM J SPORT MED, V11, P199, DOI 10.1177/036354658301100403
   Dennis Michael G, 2004, J Knee Surg, V17, P133
   Edwards A, 2007, ARTHROSCOPY, V23, P284, DOI 10.1016/j.arthro.2006.11.005
   Fanelli GC, 2004, ARTHROSCOPY, V20, P339, DOI 10.1016/j.arthro.2004.01.034
   FANELLI GC, 1993, ARTHROSCOPY, V9, P291, DOI 10.1016/S0749-8063(05)80424-4
   Fanelli GC, 1996, ARTHROSCOPY, V12, P521, DOI 10.1016/S0749-8063(96)90189-9
   FANELLI GC, 1995, ARTHROSCOPY, V11, P526, DOI 10.1016/0749-8063(95)90127-2
   FANELLI GC, 1994, ARTHROSCOPY, V10, P673, DOI 10.1016/S0749-8063(05)80067-2
   Fanelli GC, 2008, ARTHROSCOPY, V24, P234, DOI 10.1016/j.arthro.2007.09.009
   FOWLER PJ, 1987, AM J SPORT MED, V15, P553, DOI 10.1177/036354658701500606
   Fox RJ, 1998, AM J SPORT MED, V26, P395, DOI 10.1177/03635465980260030901
   Gill TJ, 2004, AM J SPORT MED, V32, P109, DOI 10.1177/0095399703258794
   Harner CD, 2000, AM J SPORT MED, V28, P144, DOI 10.1177/03635465000280020201
   Harner CD, 2001, KNEE SURG SPORT TR A, V9, P330, DOI 10.1007/s001670100230
   Harner CD, 1999, ARTHROSCOPY, V15, P741, DOI 10.1016/S0749-8063(99)70006-X
   Hey Groves EW, 1917, LANCET, V2, P674
   Huang TW, 2003, ARTHROSCOPY, V19, P712, DOI 10.1016/S0749-8063(03)00394-3
   HUGHSTON JC, 1985, J BONE JOINT SURG AM, V67A, P351, DOI 10.2106/00004623-198567030-00001
   JOHNSON JC, 1990, AM J KNEE SURG, V3, P143
   Kim SJ, 2005, ARTHROSCOPY, V21, P1271, DOI 10.1016/j.arthro.2005.08.004
   LOOS WC, 1981, AM J SPORT MED, V9, P86, DOI 10.1177/036354658100900203
   Makris CA, 2000, ARTHROSCOPY, V16, P627, DOI 10.1053/jars.2000.9238
   Mariani PP, 2006, KNEE SURG SPORT TR A, V14, P1038, DOI 10.1007/s00167-006-0086-x
   MARKS PH, 1993, ORTHOPADE, V22, P386
   McAllister DR, 2007, AM J SPORT MED, V35, P2148, DOI 10.1177/0363546507308936
   Miyasaka K, 1991, AM J KNEE SURG, V4, P3
   Morgan CD, 1997, ARTHROSCOPY, V13, P325, DOI 10.1016/S0749-8063(97)90029-3
   Noyes F R, 1996, Am J Knee Surg, V9, P200
   NOYES FR, 1994, ARTHROSCOPY, V10, P371, DOI 10.1016/S0749-8063(05)80187-2
   PAROLIE JM, 1986, AM J SPORT MED, V14, P35, DOI 10.1177/036354658601400107
   PINKOWSKI JL, 1989, AM J SPORT MED, V17, P595, DOI 10.1177/036354658901700501
   Sekiya JK, 2005, ARTHROSCOPY, V21, P1042, DOI 10.1016/j.arthro.2005.05.023
   Shelbourne KD, 2005, ARTHROSCOPY, V21, P457, DOI 10.1016/j.arthro.2004.11.013
   Shelbourne KD, 1999, AM J SPORT MED, V27, P276, DOI 10.1177/03635465990270030201
   SHINO K, 1995, AM J SPORT MED, V23, P203, DOI 10.1177/036354659502300213
   Takahashi M, 2006, KNEE SURG SPORT TR A, V14, P1055, DOI 10.1007/s00167-006-0192-9
   TORG JS, 1989, CLIN ORTHOP RELAT R, P208
   TRICKEY EL, 1980, CLIN ORTHOP RELAT R, V147, P76
   Vangsness CT, 2003, AM J SPORT MED, V31, P474, DOI 10.1177/03635465030310032701
   Veltri, 1993, J Am Acad Orthop Surg, V1, P67
   WATANABE Y, 1993, ARTHROSCOPY, V9, P57, DOI 10.1016/S0749-8063(05)80344-5
   Wind WM, 2004, AM J SPORT MED, V32, P1765, DOI 10.1177/0363546504270481
   Yoon KH, 2005, ARTHROSCOPY, V21, P1436, DOI 10.1016/j.arthro.2005.09.002
NR 57
TC 8
Z9 11
U1 0
U2 0
PU W B SAUNDERS CO-ELSEVIER INC
PI PHILADELPHIA
PA 1600 JOHN F KENNEDY BOULEVARD, STE 1800, PHILADELPHIA, PA 19103-2899 USA
SN 0278-5919
EI 1556-228X
J9 CLIN SPORT MED
JI Clin. Sports Med.
PD APR
PY 2009
VL 28
IS 2
BP 245
EP +
DI 10.1016/j.csm.2008.10.013
PG 14
WC Sport Sciences
WE Science Citation Index Expanded (SCI-EXPANDED)
SC Sport Sciences
GA 435NA
UT WOS:000265349400008
PM 19306733
DA 2022-07-22
ER

PT J
AU Mohsin, M
   Zargar, HR
   Bhat, TA
   Bhat, HA
   Mir, FA
   Wani, AH
AF Mohsin, Mir
   Zargar, Haroon Rashid
   Bhat, Tanveer Ahmed
   Bhat, Hilal Ahmed
   Mir, Farooq Ahmed
   Wani, Adil Hafeez
TI Open tendoachilles injuries due to squatting type of toilet seats:
   Five-year prospective observational study from a tertiary care center in
   India
SO INJURY-INTERNATIONAL JOURNAL OF THE CARE OF THE INJURED
LA English
DT Article
DE Commode; Achilles tendon; Wounds; Ankle injury; Toilet; Microvascular;
   Free tissue flap; Tibial nerve
ID ACHILLES-TENDON RUPTURE; RECONSTRUCTION; TISSUE; DEFECTS; FLAP
AB Background: Open traumatic tendoachilles injuries due to toilet seats are least reported. The exact mechanism of such injuries is debatable. None of the studies have reported associated neurovascular injuries and the need for microvascular tissue transfer.
   Methods: It is a 5-year prospective observational study from Sep 2013 - Aug 2018 at a tertiary care center on 26 patients who had sustained foot injuries due to squatting type toilet seats. All the patients were managed by thorough wound irrigation and debridement followed by repair of cut tendoachilles, other tendons and neurovascular structures. All the complications and secondary procedures required were recorded. Functional outcome was assessed by Boyden clinical outcome score. Follow up ranged from 1 to 5 years.
   Results: All the 26 patients reported a particular mechanism of injury. Complete transection of tendoachilles was seen in 23 (88.5%) patients and partial transection in three (11.5%) patients. Microvascular repair of cut posterior tibial artery was undertaken in three and posterior tibial nerve in two cases and microvascular parascapular flap in one case for soft tissue reconstruction. Twenty-three (88.5%) patients had good to excellent Boyden score while three patients (11.5%) had fair to poor score at 1 year. Such severe injuries due to toilet seats have never been reported in literature.
   Conclusions: Squatting toilet seats can cause devastating foot injuries involving tendons and neurovascular structures and may require microvascular tissue transfer for definitive wound management. The risk of such injuries will continue unless some modifications are undertaken in the design of the seat. (C) 2020 Elsevier Ltd. All rights reserved.
C1 [Mohsin, Mir; Zargar, Haroon Rashid; Bhat, Tanveer Ahmed; Bhat, Hilal Ahmed; Mir, Farooq Ahmed; Wani, Adil Hafeez] Sheri Kashmir Inst Med Sci, Dept Plast & Reconstruct Surg, Srinagar, J&K, India.
RP Mohsin, M (通讯作者)，SKIMS, Dept Plast & Reconstruct Surg, Srinagar, India.
EM m_mohsin@rediffmail.com
RI Mohsin, Mir/AAA-1246-2022
OI Mohsin, Mir/0000-0001-8503-2543
CR Acland RD, 2008, PRACTICE MANUAL MICR
   ANGER DM, 1995, J BONE JOINT SURG AM, V77A, P719, DOI 10.2106/00004623-199505000-00008
   Awe A A, 2015, J West Afr Coll Surg, V5, P30
   Awe OO, 2016, E AFR ORTHOP J, V10, P11
   Boopalan P R J V C, 2008, Foot Ankle Surg, V14, P96, DOI 10.1016/j.fas.2007.11.002
   BOYDEN EM, 1995, CLIN ORTHOP RELAT R, P150
   Chatterjee SS, 2006, INDIAN J PLAST SURG, V39, P29, DOI 10.4103/0970-0358.26900
   Cretnik A, 2004, WIEN KLIN WOCHENSCHR, V116, P33
   Dalton GP, 2001, CLIN ORTHOP RELAT R, P133
   Dar TA, 2013, FOOT ANKLE SURG, V19, P65, DOI 10.1016/j.fas.2012.06.005
   Dar Tahir Ahmed, 2011, Foot Ankle Surg, V17, P284, DOI 10.1016/j.fas.2010.11.002
   Doty Jesse, 2019, Foot Ankle Spec, V12, P278, DOI 10.1177/1938640019856804
   Glass AS, 2013, BJU INT, V112, P398, DOI 10.1111/bju.12173
   GORDON L, 1982, PLAST RECONSTR SURG, V70, P173, DOI 10.1097/00006534-198208000-00008
   GORMAN R L, 1989, Pediatric Emergency Care, V5, P108, DOI 10.1097/00006565-198906000-00010
   Holme SA, 2005, PEDIATR DERMATOL, V22, P344, DOI 10.1111/j.1525-1470.2005.22413.x
   INOUE T, 1990, BRIT J PLAST SURG, V43, P728, DOI 10.1016/0007-1226(90)90199-A
   Jepegnanam TS, 2009, J TRAUMA, V66, P774, DOI 10.1097/TA.0b013e31817c96c7
   JOZSA L, 1989, AM J SPORT MED, V17, P338, DOI 10.1177/036354658901700305
   Langer V, 2013, FOOT ANKLE SURG, V19, P65, DOI 10.1016/j.fas.2012.06.002
   Lee HB, 1999, PLAST RECONSTR SURG, V104, P111, DOI 10.1097/00006534-199907000-00017
   Lee JW, 2000, BRIT J PLAST SURG, V53, P574, DOI 10.1054/bjps.2000.3407
   Litvinov IV, 2010, PEDIATRICS, V125, pE419, DOI 10.1542/peds.2009-2430
   Malik M, 2015, J CLIN ORTHOP TRAUMA, V6, P63, DOI [10.1016/j.jcot.2015.01.025., DOI 10.1016/J.JCOT.2015.01.025]
   Mine R, 2000, PLAST RECONSTR SURG, V106, P1501, DOI 10.1097/00006534-200012000-00009
   Mishra B, 2015, J EVID BASED MED HEA, V2, P5090, DOI [10.18410/jebmh/2015/710., DOI 10.18410/JEBMH/2015/710]
   Said M Nader, 2015, Eur J Orthop Surg Traumatol, V25, P591, DOI 10.1007/s00590-014-1554-5
   Saltzman C L, 1998, J Am Acad Orthop Surg, V6, P316
   Sauerbier M, 1998, EUR J PLAST SURG, V21, P333, DOI 10.1007/s002380050111
   Strauss EJ, 2007, INJURY, V38, P832, DOI 10.1016/j.injury.2006.06.005
   WEI FC, 1988, PLAST RECONSTR SURG, V81, P579, DOI 10.1097/00006534-198804000-00015
   Zhu Yueliang, 2009, Zhongguo Xiu Fu Chong Jian Wai Ke Za Zhi, V23, P1180
NR 32
TC 2
Z9 2
U1 1
U2 2
PU ELSEVIER SCI LTD
PI OXFORD
PA THE BOULEVARD, LANGFORD LANE, KIDLINGTON, OXFORD OX5 1GB, OXON, ENGLAND
SN 0020-1383
EI 1879-0267
J9 INJURY
JI Injury-Int. J. Care Inj.
PD OCT
PY 2020
VL 51
IS 10
BP 2316
EP 2321
DI 10.1016/j.injury.2020.06.051
PG 6
WC Critical Care Medicine; Emergency Medicine; Orthopedics; Surgery
WE Science Citation Index Expanded (SCI-EXPANDED)
SC General & Internal Medicine; Emergency Medicine; Orthopedics; Surgery
GA NU3MW
UT WOS:000573547300033
PM 32620331
DA 2022-07-22
ER

PT J
AU Geremia, JM
   Bobbert, MF
   Nova, MC
   Ott, RD
   Lemos, FD
   Lupion, RD
   Frasson, VB
   Vaz, MA
AF Geremia, Jeam Marcel
   Bobbert, Maarten Frank
   Nova, Mayra Casa
   Ott, Rafael Duvelius
   Lemos, Fernando de Aguiar
   Lupion, Raquel de Oliveira
   Frasson, Viviane Bortoluzzi
   Vaz, Marco Aurelio
TI The structural and mechanical properties of the Achilles tendon 2 years
   after surgical repair
SO CLINICAL BIOMECHANICS
LA English
DT Article
DE Stress-strain relation; Achilles tendon rupture; Immobilization; Tendon
   healing; Ultrasound
ID IN-VIVO; MUSCLE ARCHITECTURE; ELASTIC PROPERTIES; RUPTURES; STRENGTH;
   BEHAVIOR; COMPLEX; SURGERY; TEARS; REST
AB Background: Acute ruptures of the Achilles tendon affect the tendon's structural and mechanical properties. The long-term effects of surgical repair on these properties remain unclear.
   Purpose: To evaluate effects of early mobilization versus traditional immobilization rehabilitation programs 2 years after surgical Achilles tendon repair, by comparing force-elongation and stress-strain relationships of the injured tendon to those of the uninjured tendon.
   Methods: A group of males with previous Achilles tendon rupture (n = 18) and a group of healthy male controls (n = 9) participated. Achilles tendon rupture group consisted of patients that had received early mobilization (n = 9) and patients that had received traditional immobilization with a plaster cast (n = 9).Comparisons of tendon structural and mechanical properties were made between Achilles tendon rupture and healthy control groups, and between the uninjured and injured sides of the two rehabilitation groups in Achilles tendon rupture group. Ultrasound was used to determine bilaterally tendon cross-sectional area, tendon resting length, and tendon elongation as a function of torque during maximal voluntary plantar flexion. From these data, Achilles tendon force-elongation and stress-strain relationships were determined.
   Findings: The Achilles tendon rupture group uninjured side was not different from healthy control group. Structural and mechanical parameters of the injured side were not different between the Achilles tendon rupture early mobilization and the immobilization groups. Compared to the uninjured side, the injured side showed a reduction in stress at maximal voluntary force, in Young's modulus and in stiffness.
   Interpretation: Two years post-surgical repair, the Achilles tendon mechanical properties had not returned to the uninjured contralateral tendon values. (C) 2015 Elsevier Ltd. All rights reserved.
C1 [Geremia, Jeam Marcel; Nova, Mayra Casa; Lemos, Fernando de Aguiar; Lupion, Raquel de Oliveira; Vaz, Marco Aurelio] Univ Fed Rio Grande do Sul, Sch Phys Educ, Exercise Res Lab, Porto Alegre, RS, Brazil.
   [Geremia, Jeam Marcel] Fac Phys Educ Sogipa, Porto Alegre, RS, Brazil.
   [Bobbert, Maarten Frank] Vrije Univ Amsterdam, Move Res Inst Amsterdam, Fac Human Movement Sci, Amsterdam, Netherlands.
   [Ott, Rafael Duvelius] Pontificia Univ Catolica Rio Grande do Sul, Sao Lucas Hosp, Porto Alegre, RS, Brazil.
   [Frasson, Viviane Bortoluzzi] Phys Physiotherapy Ctr, Porto Alegre, RS, Brazil.
RP Geremia, JM (通讯作者)，Fed Univ Rio Grande Sul UFRGS, Exercise Res Lab LAPEX, 750 Bairro Jardim Bot, BR-90690200 Porto Alegre, RS, Brazil.
EM jeam_geremia@hotmail.com
RI Geremia, Jeam M/O-5489-2016; Lemos, Fernando/R-4967-2019; Vaz, Marco
   Aurelio/H-8037-2012
OI Lemos, Fernando/0000-0003-1734-9324; Vaz, Marco
   Aurelio/0000-0001-6741-3502; Geremia, Jeam Marcel/0000-0001-8918-806X
FU Coordenacao de Aperfeicoamento de Pessoal de Nivel Superior (CAPES);
   Conselho Nacional de Desenvolvimento Cientifico e Tecnologico (CNPq);
   Programa Ciencias Sem Fronteiras (CSF) e Financiadora de Estudos e
   Projetos (FINEP) from Brazil
FX The authors would like to acknowledge Coordenacao de Aperfeicoamento de
   Pessoal de Nivel Superior (CAPES), Conselho Nacional de Desenvolvimento
   Cientifico e Tecnologico (CNPq), Programa Ciencias Sem Fronteiras (CSF)
   e Financiadora de Estudos e Projetos (FINEP) from Brazil for financial
   support, and Valdirene Gambarra da Silva for technical help during
   patient recruitment.
CR Arya S, 2010, J APPL PHYSIOL, V108, P670, DOI 10.1152/japplphysiol.00259.2009
   Barone R, 2009, SCAND J MED SCI SPOR, V19, P782, DOI 10.1111/j.1600-0838.2008.00863.x
   Bressel E, 2001, AM J SPORT MED, V29, P321, DOI 10.1177/03635465010290031201
   Del Buono A, 2014, BRIT MED BULL, V109, P45, DOI 10.1093/bmb/ldt029
   Elias LJ, 1998, NEUROPSYCHOLOGIA, V36, P37, DOI 10.1016/S0028-3932(97)00107-3
   Foure A, 2013, J APPL PHYSIOL, V114, P523, DOI 10.1152/japplphysiol.01313.2011
   Huttunen TT, 2014, AM J SPORT MED, V42, P2419, DOI 10.1177/0363546514540599
   INGLIS AE, 1976, J BONE JOINT SURG AM, V58, P990, DOI 10.2106/00004623-197658070-00015
   Kangas J., 2007, THESIS OULU U FINLAN
   Kannus P, 1997, SCAND J MED SCI SPOR, V7, P67
   Karamanidis K, 2006, J BIOMECH, V39, P406, DOI 10.1016/j.jbiomech.2004.12.017
   Kubo K, 2005, CLIN BIOMECH, V20, P291, DOI 10.1016/j.clinbiomech.2004.11.005
   Kubo K, 2000, EUR J APPL PHYSIOL, V83, P463, DOI 10.1007/s004210000309
   Kubo K, 2012, EUR J APPL PHYSIOL, V112, P2679, DOI 10.1007/s00421-011-2248-x
   Lantto I, 2015, SCAND J MED SCI SPOR, V25, pE133, DOI 10.1111/sms.12253
   Mademli L, 2005, EUR J APPL PHYSIOL, V94, P611, DOI 10.1007/s00421-005-1366-8
   Maffulli N, 2003, AM J SPORT MED, V31, P692, DOI 10.1177/03635465030310051001
   Maffulli N, 2003, J SPORT MED PHYS FIT, V43, P367
   Maffulli N, 2000, AM J SPORT MED, V28, P499, DOI 10.1177/03635465000280040901
   Maffulli N, 2001, SCAND J MED SCI SPOR, V11, P156
   Maffulli N., 2007, THE ACHILLES TENDON
   Maganaris CN, 1998, J PHYSIOL-LONDON, V510, P977, DOI 10.1111/j.1469-7793.1998.977bj.x
   Maganaris CN, 2008, DISABIL REHABIL, V30, P1542, DOI 10.1080/09638280701785494
   Magnusson SP, 2001, J PHYSIOL-LONDON, V531, P277, DOI 10.1111/j.1469-7793.2001.0277j.x
   McNair P, 2013, J ORTHOP RES, V31, P1469, DOI 10.1002/jor.22381
   Moller M, 2002, KNEE SURG SPORT TR A, V10, P49, DOI 10.1007/s001670100245
   Muraoka T, 2005, J APPL PHYSIOL, V99, P665, DOI 10.1152/japplphysiol.00624.2004
   Olsson N, 2011, KNEE SURG SPORT TR A, V19, P1385, DOI 10.1007/s00167-011-1511-3
   Rosenzweig S, 2009, FOOT ANKLE CLIN, V14, P699, DOI 10.1016/j.fcl.2009.07.002
   Rosso C, 2013, KNEE SURG SPORT TR A, V21, P1369, DOI 10.1007/s00167-013-2407-1
   Stenroth L, 2012, J APPL PHYSIOL, V113, P1537, DOI 10.1152/japplphysiol.00782.2012
   Urlando A, 2007, MED SCI SPORT EXER, V39, P1147, DOI 10.1249/mss.0b013e31805371d1
   Waugh CM, 2012, J ANAT, V220, P144, DOI 10.1111/j.1469-7580.2011.01461.x
NR 33
TC 42
Z9 43
U1 0
U2 13
PU ELSEVIER SCI LTD
PI OXFORD
PA THE BOULEVARD, LANGFORD LANE, KIDLINGTON, OXFORD OX5 1GB, OXON, ENGLAND
SN 0268-0033
EI 1879-1271
J9 CLIN BIOMECH
JI Clin. Biomech.
PD JUN
PY 2015
VL 30
IS 5
BP 485
EP 492
DI 10.1016/j.clinbiomech.2015.03.005
PG 8
WC Engineering, Biomedical; Orthopedics; Sport Sciences
WE Science Citation Index Expanded (SCI-EXPANDED)
SC Engineering; Orthopedics; Sport Sciences
GA CJ3EJ
UT WOS:000355365900015
PM 25828432
DA 2022-07-22
ER

PT J
AU Weinfeld, SB
AF Weinfeld, Steven B.
TI Achilles Tendon Disorders
SO MEDICAL CLINICS OF NORTH AMERICA
LA English
DT Article
DE Achilles tendon; Retrocalcaneal bursitis; Achilles rupture; Tendinitis;
   Achilles tendinosis; Haglund deformity; Paratenonitis
ID SURGICAL-TREATMENT; RUPTURES; AUGMENTATION; TENDINOPATHY; TENDINOSIS;
   ALLOGRAFT
AB Achilles tendon disorders include tendinosis, paratenonitis, insertional tendinitis, retrocalcaneal bursitis, and frank rupture. Patients present with pain and swelling in the posterior aspect of the ankle. Magnetic resonance imaging and ultrasound are helpful in confirming the diagnosis and guiding treatment. Nonsurgical management of Achilles tendon disorders includes nonsteroidal anti-inflammatory drugs, physical therapy, bracing, and footwear modification. Surgical treatment includes debridement of the diseased area of the tendon with direct repair. Tendon transfer may be necessary to augment the strength of the Achilles tendon.
C1 Icahn Sch Med Mt Sinai, Foot & Ankle Serv, New York, NY 10029 USA.
RP Weinfeld, SB (通讯作者)，Icahn Sch Med Mt Sinai, Foot & Ankle Serv, New York, NY 10029 USA.
EM steven.weinfeld@mountsinai.org
CR Chen TM, 2009, CLIN ANAT, V22, P377, DOI 10.1002/ca.20758
   Coughlin MJ., 2007, SURG FOOT ANKLE, P1149
   Den Hartog BD, 2003, FOOT ANKLE INT, V24, P233, DOI 10.1177/107110070302400306
   Garras DN, 2012, CLIN ORTHOP RELAT R, V470, P2268, DOI 10.1007/s11999-012-2355-y
   Goodwin D W, 2000, Foot Ankle Clin, V5, P135
   Henriquez H, 2012, CLIN ORTHOP RELAT R, V470, P998, DOI 10.1007/s11999-011-1830-1
   Holmes GB, 2006, FOOT ANKLE INT, V27, P952, DOI 10.1177/107110070602701115
   Johnston E, 1997, FOOT ANKLE INT, V18, P570, DOI 10.1177/107110079701800907
   Kearney RS, 2010, FOOT ANKLE INT, V31, P556, DOI 10.3113/FAI.2010.0556
   Keating JF, 2011, J BONE JOINT SURG BR, V93B, P1071, DOI 10.1302/0301-620X.93B8.25998
   KVIST MH, 1988, AM J SPORT MED, V16, P616, DOI 10.1177/036354658801600611
   Lykoudis EG, 2010, FOOT ANKLE INT, V31, P634, DOI 10.3113/FAI.2010.0634
   McGarvey WC, 2002, FOOT ANKLE INT, V23, P19, DOI 10.1177/107110070202300104
   Metcalfe D, 2009, FOOT ANKLE INT, V30, P661, DOI 10.3113/FAI.2009.0661
   Monto RR, 2012, FOOT ANKLE INT, V33, P379, DOI 10.3113/FAI.2012.0379
   Ortmann FW, 2007, FOOT ANKLE INT, V28, P149, DOI 10.3113/FAI.2007.0149
   Paavola M, 1998, ACTA RADIOL, V39, P612, DOI 10.3109/02841859809175485
   Paavola M, 2002, J BONE JOINT SURG AM, V84A, P2062, DOI 10.2106/00004623-200211000-00024
   Pannar C, 2007, FOOT ANKLE INT, V28, P1287, DOI 10.3113/FAI.2007.1287
   Patel A, 2012, CLIN ORTHOP RELAT R, V470, P270, DOI 10.1007/s11999-011-2166-6
   Porter DA, 1997, FOOT ANKLE INT, V18, P557, DOI 10.1177/107110079701800905
   Rahm S, 2013, FOOT ANKLE INT, V34, P1100, DOI 10.1177/1071100713487725
   Reddy SS, 2009, J AM ACAD ORTHOP SUR, V17, P3, DOI 10.5435/00124635-200901000-00002
   Schepsis AA, 2002, AM J SPORT MED, V30, P287, DOI 10.1177/03635465020300022501
   Wapner K L, 1993, Foot Ankle, V14, P443
NR 25
TC 33
Z9 34
U1 4
U2 20
PU W B SAUNDERS CO-ELSEVIER INC
PI PHILADELPHIA
PA 1600 JOHN F KENNEDY BOULEVARD, STE 1800, PHILADELPHIA, PA 19103-2899 USA
SN 0025-7125
EI 1557-9859
J9 MED CLIN N AM
JI Med. Clin. N. Am.
PD MAR
PY 2014
VL 98
IS 2
BP 331
EP +
DI 10.1016/j.mcna.2013.11.005
PG 9
WC Medicine, General & Internal
WE Science Citation Index Expanded (SCI-EXPANDED)
SC General & Internal Medicine
GA AE1GZ
UT WOS:000333719100013
PM 24559878
DA 2022-07-22
ER

PT J
AU Ivanac, G
   Lemac, D
   Kosovic, V
   Bojanic, K
   Cengic, T
   Dumic-Cule, I
   Pecina, M
   Brkljacic, B
AF Ivanac, Gordana
   Lemac, Domagoj
   Kosovic, Vilma
   Bojanic, Kristina
   Cengic, Tomislav
   Dumic-Cule, Ivo
   Pecina, Marko
   Brkljacic, Boris
TI Importance of shear-wave elastography in prediction of Achilles tendon
   rupture
SO INTERNATIONAL ORTHOPAEDICS
LA English
DT Article
DE Achilles tendon; Shear wave elastography; Rupture; Prevention
ID REPRODUCIBILITY
AB Purpose It was demonstrated that about 6% of patients with a ruptured Achilles tendon experience the rupture of contralateral tendon in the future; the aim of this study was to estimate the risk for rupture of contralateral tendon in patients who underwent surgical reconstruction of ruptured Achilles tendon by using subjective questionnaires and shear-wave elastography. Methods Twenty-four patients who underwent surgical repair of the ruptured Achilles tendon and twelve age-matched healthy controls were examined with ultrasound SWE. Functional outcomes were assessed with American Orthopedic Foot and Ankle Society (AOFAS) scoring system and subjective rating system which we introduced and validated. Results The elasticity of injured tendon was markedly decreased (by 42%) compared to the contralateral tendon of the patient, as expected. Both AOFAS score and our novel subjective assessment scale positively correlate with ultrasound SWE values in ruptured Achilles tendons. The elasticity of contralateral Achilles tendons in patients was 23% lower than among healthy individuals. Conclusion Irrespective of the lack of difference in the subjective feeling assessed by AOFAS, the contralateral tendon in the patients with reconstructed Achilles tendon has significantly lower stiffness than healthy individuals. Therefore, contralateral tendons in patients who suffered from rupture are more prone to future ruptures.
C1 [Ivanac, Gordana; Dumic-Cule, Ivo; Brkljacic, Boris] Univ Hosp Dubrava, Dept Diagnost & Intervent Radiol, Av Gojka Suska 6, Zagreb 10000, Croatia.
   [Ivanac, Gordana; Dumic-Cule, Ivo; Pecina, Marko; Brkljacic, Boris] Univ Zagreb, Sch Med, Salata 3, Zagreb 10000, Croatia.
   [Lemac, Domagoj] Univ Hosp Dubrava, Dept Orthopaed & Trauma Surg, Av Gojka Suska 6, Zagreb 10000, Croatia.
   [Kosovic, Vilma] Gen Hosp Dubrovnik, Dept Radiol, Dubrovnik, Croatia.
   [Bojanic, Kristina] JJ Strossmayer Univ Osijek, Fac Med Osijek, Dept Biophys & Radiol, Osijek 31000, Croatia.
   [Cengic, Tomislav] Univ Hosp Ctr Sisters Mercy, Dept Orthopaed & Trauma Surg, Draskoviceva 19, Zagreb 10000, Croatia.
RP Dumic-Cule, I (通讯作者)，Univ Hosp Dubrava, Dept Diagnost & Intervent Radiol, Av Gojka Suska 6, Zagreb 10000, Croatia.; Dumic-Cule, I (通讯作者)，Univ Zagreb, Sch Med, Salata 3, Zagreb 10000, Croatia.
EM ivodc1@gmail.com
RI /AFW-0958-2022; /ABC-8179-2021
OI /0000-0002-2005-6402; /0000-0002-2005-6402; Bojanic,
   Kristina/0000-0003-4473-5706; Pecina, Marko/0000-0002-8537-1761
CR Alhammoud A, 2017, INT ORTHOP, V41, P309, DOI 10.1007/s00264-016-3318-9
   Aroen A, 2004, SCAND J MED SCI SPOR, V14, P30, DOI 10.1111/j.1600-0838.2004.00344.x
   Chan JJ, 2020, INT ORTHOP, V44, P585, DOI 10.1007/s00264-019-04471-2
   Chen XM, 2013, J ULTRAS MED, V32, P449, DOI 10.7863/jum.2013.32.3.449
   Corrigan P, 2019, ULTRASOUND MED BIOL, V45, P1574, DOI 10.1016/j.ultrasmedbio.2019.03.015
   Dirrichs T, 2019, ACAD RADIOL, V26, P1345, DOI 10.1016/j.acra.2018.12.014
   Dirrichs T, 2018, ACAD RADIOL, V25, P265, DOI 10.1016/j.acra.2017.09.011
   Grgurevic I, 2019, EUR RADIOL, V29, P2448, DOI 10.1007/s00330-018-5831-2
   Hari S, 2018, DIAGN INTERV IMAG, V99, P699, DOI 10.1016/j.diii.2018.06.002
   Holmes GB, 2006, FOOT ANKLE INT, V27, P952, DOI 10.1177/107110070602701115
   Huttunen TT, 2014, AM J SPORT MED, V42, P2419, DOI 10.1177/0363546514540599
   JOZSA L, 1989, MATRIX, V9, P140, DOI 10.1016/S0934-8832(89)80032-0
   Jozsa L, 1997, SCAND J MED SCI SPOR, V7, P113
   KOMI PV, 1992, CLIN SPORT MED, V11, P521
   Leppilahti J, 1998, SPORTS MED, V25, P79, DOI 10.2165/00007256-199825020-00002
   Leppilahti J, 1996, ANN CHIR GYNAECOL FE, V85, P369
   Li QR, 2018, BIOMED RES INT, V2018, DOI 10.1155/2018/2367615
   Maffulli N, 2000, CLIN J SPORT MED, V10, P269, DOI 10.1097/00042752-200010000-00008
   Meulenkamp B, 2018, SYST REV-LONDON, V7, DOI 10.1186/s13643-018-0912-5
   Moller A, 1996, ACTA ORTHOP SCAND, V67, P479, DOI 10.3109/17453679608996672
   Payne C, 2018, SKELETAL RADIOL, V47, P779, DOI 10.1007/s00256-017-2846-8
   Payne C, 2017, SKELETAL RADIOL, V46, P889, DOI 10.1007/s00256-017-2629-2
   Peina M, 2004, OVERUSE INJURIES MUS, P263
   Sheth U, 2017, BONE JOINT J, V99B, P78, DOI [10.1302/0301-620X.99B1.BJJ-2016-0434.R1, 10.1302/0301-620x.99b1.bjj-2016-0434.r1]
   Snoj Z, 2020, SEMIN MUSCULOSKEL R, V24, P156, DOI 10.1055/s-0039-3402746
   Suchak AA, 2005, FOOT ANKLE INT, V26, P932, DOI 10.1177/107110070502601106
   Zhang L, 2018, EUR J RADIOL, V109, P1, DOI 10.1016/j.ejrad.2018.10.003
NR 27
TC 3
Z9 3
U1 1
U2 7
PU SPRINGER
PI NEW YORK
PA ONE NEW YORK PLAZA, SUITE 4600, NEW YORK, NY, UNITED STATES
SN 0341-2695
EI 1432-5195
J9 INT ORTHOP
JI Int. Orthop.
PD APR
PY 2021
VL 45
IS 4
SI SI
BP 1043
EP 1047
DI 10.1007/s00264-020-04670-2
EA JUN 2020
PG 5
WC Orthopedics
WE Science Citation Index Expanded (SCI-EXPANDED)
SC Orthopedics
GA RO1GS
UT WOS:000544847300001
PM 32613301
DA 2022-07-22
ER

PT J
AU Makulavicius, A
   Oliva, XM
   Mazarevicius, G
   Klinga, M
   Uvarovas, V
   Porvaneckas, N
   Planella, MM
   Mazurek, T
AF Makulavicius, A.
   Oliva, X. Martin
   Mazarevicius, G.
   Klinga, M.
   Uvarovas, V.
   Porvaneckas, N.
   Planella, M. Monzo
   Mazurek, T.
TI Comparative anatomical study of standard percutaneous and modified
   medialised percutaneous Bunnell type repair for artificial Achilles
   tendon rupture: positive effect of medialisation of the stitches with
   lower risk of sural nerve injury
SO FOLIA MORPHOLOGICA
LA English
DT Article
DE Achilles repair; percutaneous; medialisation
ID NONOPERATIVE MANAGEMENT; CONTROLLED-TRIALS; SURGICAL ANATOMY;
   METAANALYSIS; DEVICE
AB Background: Less invasive percutaneous acute Achilles tendon rupture (AATR) repair techniques gain popularity because of lower risk of surgical wound complications. But these approaches have an increased risk of sural nerve iatrogenic injury as this sensory nerve is usually not visualised during minimally invasive operative procedures. We compared standard percutaneous Bunnell type and our proposed modified-medialised percutaneous technique in a cadaver study to evaluate potential advantages.
   Materials and methods: Ten pairs of fresh frozen specimens were divided into two groups for comparative anatomical study. Tenotomies of Achilles tendons were made and wounds sutured. Ten standard and 10 modified-medialised repairs were applied for artificially performed ruptures. All sutured tendons were dissected meticulously. We carefully looked at repaired Achilles tendon end-to-end contact and adaptation, distance from Achilles insertion in calcaneal tubercle to place where sural nerve crosses lateral border of the Achilles tendon and possible sural nerve and vein entrapment. Groups were compared using Fisher's exact and Student-T tests.
   Results: All ends of sharply dissected tendons in both groups were in sufficient contact. No measurable diastasis between tendon ends was found in all cases. No entrapment of sural nerve or vein was found in modified percutaneous Bunnell suture technique group, whereas 7 of 10 sural nerves and 9 small saphenous veins were entrapped when using standard percutaneous Bunnell type technique. Average distance from Achilles tendon insertion in tuber calcanei to sural nerve crossing the lateral border of Achilles was 93 mm.
   Conclusions: Medialisation of percutaneous suture in AATR repair shows clear advantages compared to standard non medialised technique ensuring a possible lower incidence of sural nerve entrapment injury. Our modified percutaneous Bunnell type technique allows sufficient adaptation of ruptured Achilles tendon.
C1 [Makulavicius, A.; Mazarevicius, G.; Klinga, M.; Uvarovas, V.; Porvaneckas, N.] Vilnius Univ, Fac Med, Republican Vilnius Univ Hosp, Ctr Orthopaed & Traumatol, Vilnius, Lithuania.
   [Oliva, X. Martin; Planella, M. Monzo] Univ Barcelona, Fac Med, Dept Embryol & Human Anat, E-08007 Barcelona, Spain.
   [Mazurek, T.] Med Univ Gdansk, Dept Orthopaed, Gdansk, Poland.
RP Makulavicius, A (通讯作者)，Vilnius Univ, Fac Med, Republican Vilnius Univ Hosp, Ctr Orthopaed & Traumatol, Vilnius, Lithuania.
EM makulaviciusa@gmail.com
CR Aktas S, 2009, FOOT ANKLE INT, V30, P391, DOI 10.3113/FAI.2009.0391
   Bhandari M, 2002, CLIN ORTHOP RELAT R, P190
   Bruggeman NB, 2004, CLIN ORTHOP RELAT R, P63, DOI 10.1097/01.blo.0000144475.05543.e7
   Carmont MR, 2013, KNEE SURG SPORT TR A, V21, P1361, DOI 10.1007/s00167-013-2423-1
   Ceccarelli F, 2007, CLIN ORTHOP RELAT R, P188, DOI 10.1097/BLO.0b013e3180396f07
   Citak M, 2007, BRIT J SPORT MED, V41, P456, DOI 10.1136/bjsm.2006.031328
   Flavin R, 2007, INJURY, V38, P845, DOI 10.1016/j.injury.2006.10.016
   Gigante A, 2008, KNEE SURG SPORT TR A, V16, P204, DOI 10.1007/s00167-007-0448-z
   Haji A, 2004, FOOT ANKLE INT, V25, P215, DOI 10.1177/107110070402500404
   Hamilton PD, 2009, FOOT ANKLE INT, V30, P1202, DOI 10.3113/FAI.2009.1202
   Henriquez H, 2012, CLIN ORTHOP RELAT R, V470, P998, DOI 10.1007/s11999-011-1830-1
   HOCKENBURY RT, 1990, FOOT ANKLE, V11, P67, DOI 10.1177/107110079001100202
   HUELKE DF, 1958, ANAT REC, V132, P81, DOI 10.1002/ar.1091320106
   Jiang N, 2012, INT ORTHOP, V36, P765, DOI 10.1007/s00264-011-1431-3
   KAKIUCHI M, 1995, J BONE JOINT SURG BR, V77B, P60, DOI 10.1302/0301-620X.77B1.7822398
   Kammar H, 2014, ORTHOPEDICS, V37, pE298, DOI 10.3928/01477447-20140225-64
   Keating JF, 2011, J BONE JOINT SURG BR, V93B, P1071, DOI 10.1302/0301-620X.93B8.25998
   Khan RJK, 2005, J BONE JOINT SURG AM, V87A, P2202, DOI 10.2106/JBJS.D.03049
   KLEIN W, 1991, Chirurgia degli Organi di Movimento, V76, P223
   Kocher MS, 2002, AM J SPORT MED, V30, P783, DOI 10.1177/03635465020300060501
   LAWRENCE SJ, 1994, FOOT ANKLE INT, V15, P490, DOI 10.1177/107110079401500906
   Lim J, 2001, FOOT ANKLE INT, V22, P559, DOI 10.1177/107110070102200705
   MA GWC, 1977, CLIN ORTHOP RELAT R, P247
   Maes Renaud, 2006, Acta Orthop Belg, V72, P179
   Majewski M, 2006, AM J SPORT MED, V34, P793, DOI 10.1177/0363546505283266
   Mazur L, 2014, WULFENIA, V21, P341
   McMahon Samuel E, 2011, Foot Ankle Surg, V17, P211, DOI 10.1016/j.fas.2010.11.001
   Metz R, 2008, AM J SPORT MED, V36, P1688, DOI 10.1177/0363546508319312
   Metz Roderik, 2009, Foot Ankle Spec, V2, P219, DOI 10.1177/1938640009348338
   Mizia E, 2014, FOLIA MORPHOL, V73, P292, DOI 10.5603/FM.2014.0046
   Moller M, 2001, J BONE JOINT SURG BR, V83B, P843
   Nilsson-Helander K, 2010, AM J SPORT MED, V38, P2186, DOI 10.1177/0363546510376052
   ORTIGUELA ME, 1987, J HAND SURG-AM, V12A, P1119, DOI 10.1016/S0363-5023(87)80129-6
   Porter KJ, 2014, FOOT ANKLE SURG, V20, P90, DOI 10.1016/j.fas.2013.11.005
   ROWLEY DI, 1982, INJURY, V14, P252, DOI 10.1016/0020-1383(82)90138-3
   Solomon LB, 2001, J ANAT, V199, P717, DOI 10.1046/j.1469-7580.2001.19960717.x
   Wagnon Raymond, 2005, J Foot Ankle Surg, V44, P437, DOI 10.1053/j.jfas.2005.07.015
   Webb J, 2000, FOOT ANKLE INT, V21, P475, DOI 10.1177/107110070002100604
   Webb JM, 1999, J BONE JOINT SURG BR, V81B, P877, DOI 10.1302/0301-620X.81B5.9784
   Wilkins R, 2012, AM J SPORT MED, V40, P2154, DOI 10.1177/0363546512453293
   Willits K, 2010, J BONE JOINT SURG AM, V92A, P2767, DOI 10.2106/JBJS.I.01401
   Wong J, 2002, AM J SPORT MED, V30, P565, DOI 10.1177/03635465020300041701
NR 42
TC 3
Z9 4
U1 0
U2 4
PU VIA MEDICA
PI GDANSK
PA UL SWIETOKRZYSKA 73, 80-180 GDANSK, POLAND
SN 0015-5659
EI 1644-3284
J9 FOLIA MORPHOL
JI Folia Morphol.
PY 2016
VL 75
IS 1
BP 53
EP 59
DI 10.5603/FM.a2015.0067
PG 7
WC Anatomy & Morphology
WE Science Citation Index Expanded (SCI-EXPANDED)
SC Anatomy & Morphology
GA DN2IY
UT WOS:000376888500008
PM 26365861
OA gold
DA 2022-07-22
ER

PT J
AU Jielile, J
   Bai, JP
   Sabirhazi, G
   Redat, D
   Yilihamu, T
   Xinlin, B
   Hu, GY
   Tang, B
   Liang, B
   Sun, Q
AF Jielile, Jiasharete
   Bai, Jing Ping
   Sabirhazi, Gulnur
   Redat, Darebai
   Yilihamu, Tuoheti
   Xinlin, Baoltri
   Hu, Geyang
   Tang, Bin
   Liang, Bing
   Sun, Qi
TI Factors influencing the tensile strength of repaired Achilles tendon: A
   biomechanical experiment study
SO CLINICAL BIOMECHANICS
LA English
DT Article
DE Achilles tendon rupture; Tendon healing; Kinesitherapy; Mechanobiology
ID FULL WEIGHTBEARING; SURGICAL REPAIR; IN-VITRO; RUPTURE; SUTURE;
   MOBILIZATION; MANAGEMENT; SYSTEM; MOTION; ANKLE
AB Background: Operative treatment has been advocated as the method of choice to repair Achilles tendon rupture as surgery results in reduced re-rupture rate and faster rehabilitation. Many surgical techniques have been introduced allowing for postoperative early motion of the ankle joint. However, it is currently very difficult for surgeons to determine the optimal treatment conditions for ruptured Achilles tendon with an increasing number of end-to-end suture methods, suture materials, and epitenon suture techniques.
   Methods: In the present biomechanical experiment study based on an orthogonal design, thirty-two New Zealand white rabbits received Achilles tendon tenotomy and subsequent operative treatment to repair the tendon employing four end-to-end suture methods, four suture materials, and four epitenon suture techniques. The tensile strength of the repaired Achilles tendon was investigated at four rehabilitation periods, and in comparison with the results of another sixteen rabbits with normal Achilles tendons.
   Findings: The end-to-end suture method contributed most to the final Achilles tendon tensile strength in addition to rehabilitation period, with the highest values occurring with the use of the parachute-like ("Pa" bone) suture method. The other two factors, namely, suture material and epitenon suture technique, had relatively little influence on the results.
   Interpretation: The parachute-like ("Pa" bone) surgical technique is superior to the other three end-to-end suture methods, with enhanced tensile strength of the repaired tendon. This method allows for postoperative early kinesitherapy of the ankle and knee joints. Therefore, this technique is highly recommended in clinical situations for treatment of ruptured Achilles tendon. (C) 2010 Elsevier Ltd. All rights reserved.
C1 [Bai, Jing Ping; Redat, Darebai; Xinlin, Baoltri; Tang, Bin; Liang, Bing] Xinjiang Med Univ, Teaching Hosp 3, Dept Orthoped, Urumqi 830011, Peoples R China.
   [Jielile, Jiasharete] Xinjiang Med Univ, Teaching Hosp 1, Inst Orthoped, Dept Microsurg Repair & Bone Tumor, Urumqi 830054, Peoples R China.
   [Sabirhazi, Gulnur] Chinese Acad Sci, Xinjiang Inst Ecol & Geog, Urumqi 830028, Peoples R China.
   [Yilihamu, Tuoheti] Xinjiang Med Univ, Teaching Hosp 2, Dept Orthoped, Urumqi 830028, Peoples R China.
   [Hu, Geyang; Sun, Qi] Xinjiang Med Univ, Teaching Hosp 1, Dept Med Res Ctr, Urumqi 830054, Peoples R China.
RP Bai, JP (通讯作者)，Xinjiang Med Univ, Teaching Hosp 3, Dept Orthoped, 30 Beijing S Rd, Urumqi 830011, Peoples R China.
EM jingping-bai@163.com
OI Jielile, Jiasharete/0000-0002-1809-1356
FU National Natural Science Foundation of China (NSFC); Xinjiang Medical
   University
FX The authors would like to thank National Natural Science Foundation of
   China (NSFC) and Xinjiang Medical University for their financial
   support. Many thanks are due to Nijiati Turson, Chief Physician of
   Department of Stomatology, the Second Teaching Hospital of Xinjiang
   Medical University, who provided the schematic drawing of Fig. 1. We
   also thank Professor Yijin Wang and Yongfeng Li, Department of
   Biomechanics, Shanghai University, for their intensive guidance and the
   help with the biomechanical tests.
CR Aoki M, 1998, AM J SPORT MED, V26, P794, DOI 10.1177/03635465980260061001
   Ballas MT, 1998, AM FAM PHYSICIAN, V57, P267
   CARDEN DG, 1987, J BONE JOINT SURG BR, V69, P416, DOI 10.1302/0301-620X.69B3.3294839
   Chiodo CP, 2006, FOOT ANKLE INT, V27, P305, DOI 10.1177/107110070602700415
   GELBERMAN RH, 1982, J HAND SURG-AM, V7, P170, DOI 10.1016/S0363-5023(82)80083-X
   Huffard B, 2008, CLIN BIOMECH, V23, P1158, DOI 10.1016/j.clinbiomech.2008.05.007
   JIASHARETE J, 2008, CHIN J CLIN REHABIL, V11, P17
   JIASHARETE J, 2004, CHIN J MED BIOMECH, V19, P108
   JIASHARETE J, 2007, CHINESE J CLIN REHAB, V11, P1419
   Jielile J., 2003, CHIN J GEN PRAC, V2, P49
   KAKIUCHI M, 1995, J BONE JOINT SURG BR, V77B, P60, DOI 10.1302/0301-620X.77B1.7822398
   Komatsu F, 2006, J ORTHOP SCI, V11, P70, DOI 10.1007/s00776-005-0973-x
   KRUEGERFRANKE M, 1995, BRIT J SPORT MED, V29, P121, DOI 10.1136/bjsm.29.2.121
   Lee Daniel K, 2007, J Foot Ankle Surg, V46, P451, DOI 10.1053/j.jfas.2007.05.007
   Lee SJ, 2008, FOOT ANKLE INT, V29, P427, DOI 10.3113/FAI.2008.0427
   Lewis N, 2003, FOOT ANKLE INT, V24, P857, DOI 10.1177/107110070302401109
   Maffulli N, 1999, J BONE JOINT SURG AM, V81A, P1019, DOI 10.2106/00004623-199907000-00017
   Maffulli N, 1999, CLIN J SPORT MED, V9, P157, DOI 10.1097/00042752-199907000-00007
   Maffulli N, 2008, J BONE JOINT SURG AM, V90A, P1348, DOI 10.2106/JBJS.G.01241
   MARTI R, 1974, HELV CHIR ACTA, V41, P293
   Metz R, 2008, AM J SPORT MED, V36, P1688, DOI 10.1177/0363546508319312
   Moller M, 2001, J BONE JOINT SURG BR, V83B, P843
   Mortensen NHM, 1999, J BONE JOINT SURG AM, V81A, P983, DOI 10.2106/00004623-199907000-00011
   Motta P, 1997, AM J SPORT MED, V25, P172, DOI 10.1177/036354659702500205
   Reddy GK, 1998, MED SCI SPORT EXER, V30, P794, DOI 10.1097/00005768-199806000-00003
   SILFVERSKIOLD KL, 1994, J HAND SURG-AM, V19A, P53, DOI 10.1016/0363-5023(94)90224-0
   SOMA CA, 1995, ORTHOP CLIN N AM, V26, P239
   Speck M, 1998, AM J SPORT MED, V26, P789, DOI 10.1177/03635465980260060901
   Suchak AA, 2005, FOOT ANKLE INT, V26, P932, DOI 10.1177/107110070502601106
   TRAIL IA, 1992, J HAND SURG-BRIT EUR, V17B, P544, DOI 10.1016/S0266-7681(05)80239-6
   Uchiyama E, 2007, AM J SPORT MED, V35, P1739, DOI 10.1177/0363546507304492
   Wang JHC, 2006, BIOMECH MODEL MECHAN, V5, P1, DOI 10.1007/s10237-005-0012-z
   WATSON TW, 1995, FOOT ANKLE INT, V16, P191, DOI 10.1177/107110079501600404
NR 33
TC 12
Z9 13
U1 0
U2 7
PU ELSEVIER SCI LTD
PI OXFORD
PA THE BOULEVARD, LANGFORD LANE, KIDLINGTON, OXFORD OX5 1GB, OXON, ENGLAND
SN 0268-0033
EI 1879-1271
J9 CLIN BIOMECH
JI Clin. Biomech.
PD OCT
PY 2010
VL 25
IS 8
BP 789
EP 795
DI 10.1016/j.clinbiomech.2010.05.005
PG 7
WC Engineering, Biomedical; Orthopedics; Sport Sciences
WE Science Citation Index Expanded (SCI-EXPANDED)
SC Engineering; Orthopedics; Sport Sciences
GA 643XT
UT WOS:000281334700007
PM 20696369
DA 2022-07-22
ER

PT J
AU Maffulli, N
   Via, AG
   Oliva, F
AF Maffulli, Nicola
   Via, Alessio Giai
   Oliva, Francesco
TI Chronic Achilles Tendon Disorders Tendinopathy and Chronic Rupture
SO CLINICS IN SPORTS MEDICINE
LA English
DT Article
DE Achilles tendinopathy; Chronic Achilles tendon rupture; Neglected
   ruptures; Mini-invasive surgery
ID RANDOMIZED CONTROLLED-TRIAL; IMAGE-GUIDED INJECTIONS; MAIN BODY;
   MANAGEMENT; GLYCATION; RECONSTRUCTION; EXPRESSION; PHENOTYPE; PATHOLOGY;
   TENOTOMY
AB Tendinopathy of the Achilles tendon involves clinical conditions in and around the tendon and it is the result of a failure of a chronic healing response. Although several conservative therapeutic options have been proposed, few of them are supported by randomized controlled trials. The management is primarily conservative and many patients respond well to conservative measures. If clinical conditions do not improve after 6 months of conservative management, surgery is recommended. The management of chronic ruptures is different from that of acute ruptures. The optimal surgical procedure is still debated. In this article chronic Achilles tendon disorders are debated and evidence-based medicine treatment strategies are discussed.
C1 [Maffulli, Nicola] Univ Salerno, Sch Med & Surg, Dept Musculoskeletal Disorders, I-84081 Salerno, Italy.
   [Maffulli, Nicola] Mary Univ London, Barts & London Sch Med & Dent, Mile End Hosp, Dept Musculoskeletal Disorders,Ctr Sports & Exerc, London E1 4DG, England.
   [Via, Alessio Giai; Oliva, Francesco] Univ Roma Tor Vergata, Sch Med, Dept Orthopaed & Traumatol, I-00133 Rome, Italy.
RP Maffulli, N (通讯作者)，Univ Salerno, Sch Med & Surg, Dept Musculoskeletal Disorders, Via Allende, I-84081 Salerno, Italy.
EM n.maffulli@qmul.ac.uk
RI Maffulli, Nicola/A-1839-2014; Oliva, Francesco/AAC-2030-2019
OI Maffulli, Nicola/0000-0002-5327-3702; Oliva,
   Francesco/0000-0002-7473-0271; GIAI VIA, Alessio Davide
   Enrico/0000-0002-5943-081X
CR Ahmed N, 2005, DIABETES RES CLIN PR, V67, P3, DOI 10.1016/j.diabres.2004.09.004
   Alfredson H, 2003, KNEE SURG SPORT TR A, V11, P334, DOI 10.1007/s00167-003-0391-6
   Ames PRJ, 2008, DISABIL REHABIL, V30, P1646, DOI 10.1080/09638280701785882
   Carlsson O, 2011, SCAND J MED SCI SPOR, V21, P562, DOI 10.1111/j.1600-0838.2009.01080.x
   Chan O, 2008, DISABIL REHABIL, V30, P1697, DOI 10.1080/09638280701788225
   Choi HR, 2002, J ORTHOP RES, V20, P927, DOI 10.1016/S0736-0266(02)00016-5
   DALTON S, 1995, ANN RHEUM DIS, V54, P571, DOI 10.1136/ard.54.7.571
   de Oliveira RR, 2011, DIABETIC MED, V28, P886, DOI 10.1111/j.1464-5491.2010.03197.x
   de Vos RJ, 2010, JAMA-J AM MED ASSOC, V303, P144, DOI 10.1001/jama.2009.1986
   Dean BJF, 2013, CLIN ORTHOP RELAT R, V471, P3036, DOI 10.1007/s11999-013-3010-y
   Duncan WS, 1928, J AMER MED ASSOC, V91, P1779, DOI 10.1001/jama.1928.02700230019006
   Frairia Roberto, 2011, Muscles Ligaments Tendons J, V1, P138
   Freemont AJ, 1997, LANCET, V350, P178, DOI 10.1016/S0140-6736(97)02135-1
   Ghilardi JR, 2012, ARTHRITIS RHEUM-US, V64, P2223, DOI 10.1002/art.34385
   Hadi M, 2013, BRIT MED BULL, V108, P95, DOI 10.1093/bmb/ldt019
   Hutchison AM, 2013, FOOT ANKLE SURG, V19, P112, DOI 10.1016/j.fas.2012.12.006
   Kreiner F, 2011, PAIN, V152, P1127, DOI 10.1016/j.pain.2011.01.032
   Li YF, 2013, MATRIX BIOL, V32, P169, DOI 10.1016/j.matbio.2013.01.003
   Lian O, 2006, AM J SPORT MED, V34, P1801, DOI 10.1177/0363546506289169
   Longo UG, 2008, DISABIL REHABIL, V30, P1709, DOI 10.1080/09638280701786922
   Loppini M, 2012, MUSCLES LIGAMENTS TE, V1, P134
   Maffulli N, 1998, ARTHROSCOPY, V14, P840, DOI 10.1016/S0749-8063(98)70021-0
   Maffulli N, 2003, CLIN SPORT MED, V22, P675, DOI 10.1016/S0278-5919(03)00004-8
   Maffulli N, 1997, AM J SPORT MED, V25, P835, DOI 10.1177/036354659702500618
   Maffulli N, 2006, CLIN J SPORT MED, V16, P123, DOI 10.1097/00042752-200603000-00007
   Maffulli N, 2004, J ROY SOC MED, V97, P472, DOI 10.1258/jrsm.97.10.472
   Maffulli N, 1996, BRIT J SPORT MED, V30, P250, DOI 10.1136/bjsm.30.3.250
   Maffulli N, 1999, AM J SPORT MED, V27, P747, DOI 10.1177/03635465990270061101
   Maffulli Nicola, 2007, Foot Ankle Clin, V12, P583, DOI 10.1016/j.fcl.2007.07.007
   Maffulli N, 2013, PHYS THER SPORT, V14, P163, DOI 10.1016/j.ptsp.2012.07.002
   Maffulli N, 2013, AM J SPORT MED, V41, P865, DOI 10.1177/0363546512473819
   Maffulli N, 2010, AM J SPORT MED, V38, P2304, DOI 10.1177/0363546510376619
   Mahajan Ravindra H, 2009, J Orthop Surg (Hong Kong), V17, P194
   Matles A L, 1975, Bull Hosp Joint Dis, V36, P48
   Modesti Andrea, 2013, Muscles Ligaments Tendons J, V3, P1, DOI 10.11138/mltj/2013.3.1.001
   Neufeld SK, 2014, FOOT ANKLE CLIN, V19, P73, DOI 10.1016/j.fcl.2013.10.005
   Oksanen MM, 2014, FOOT ANKLE SURG, V20, P253, DOI 10.1016/j.fas.2014.06.003
   Oliva F, 2013, CELL DEATH DIS, V4, DOI 10.1038/cddis.2013.229
   Oliva Francesco, 2013, Muscles Ligaments Tendons J, V3, P201
   Oliva F, 2012, BMC MED, V10, DOI 10.1186/1741-7015-10-95
   Oliva F, 2009, BIOCHEM BIOPH RES CO, V379, P887, DOI 10.1016/j.bbrc.2008.12.188
   Paavola M, 2000, AM J SPORT MED, V28, P634, DOI 10.1177/03635465000280050301
   Pannar C, 2007, FOOT ANKLE INT, V28, P1287, DOI 10.3113/FAI.2007.1287
   Reigle KL, 2008, J CELL BIOCHEM, V104, P1684, DOI 10.1002/jcb.21735
   Riley GP, 2002, MATRIX BIOL, V21, P185, DOI 10.1016/S0945-053X(01)00196-2
   Rompe JD, 2007, AM J SPORT MED, V35, P374, DOI 10.1177/0363546506295940
   Rompe JD, 2008, DISABIL REHABIL, V30, P1666, DOI 10.1080/09638280701785825
   Sayana MK, 2007, J SCI MED SPORT, V10, P52, DOI 10.1016/j.jsams.2006.05.008
   Schizas N, 2012, J ORTHOP RES, V30, P1447, DOI 10.1002/jor.22094
   Screen HRC, 2008, J MECH BEHAV BIOMED, V1, P51, DOI 10.1016/j.jmbbm.2007.03.002
   SIMMONDS F A, 1957, Practitioner, V179, P56
   Tallon C, 2001, AM J SPORT MED, V29, P315, DOI 10.1177/03635465010290031101
   Tarantino U, 2009, AMINO ACIDS, V36, P679, DOI 10.1007/s00726-008-0133-7
   Testa V, 2002, MED SCI SPORT EXER, V34, P573, DOI 10.1097/00005768-200204000-00002
   Xu YH, 2011, SPORTS MED ARTHROSC, V19, P354, DOI 10.1097/JSA.0b013e318229d7e3
   Young J, 2005, TECH FOOT ANKLE SURG, V4, P148, DOI 10.1097/01.btf.0000177225.35803.e2
   Young JS, 2005, TECH FOOT ANKLE SURG, V4, P143, DOI 10.1097/01.btf.0000177227.37589.d2
NR 57
TC 44
Z9 52
U1 2
U2 23
PU W B SAUNDERS CO-ELSEVIER INC
PI PHILADELPHIA
PA 1600 JOHN F KENNEDY BOULEVARD, STE 1800, PHILADELPHIA, PA 19103-2899 USA
SN 0278-5919
EI 1556-228X
J9 CLIN SPORT MED
JI Clin. Sports Med.
PD OCT
PY 2015
VL 34
IS 4
BP 607
EP +
DI 10.1016/j.csm.2015.06.010
PG 19
WC Sport Sciences
WE Science Citation Index Expanded (SCI-EXPANDED)
SC Sport Sciences
GA CU3OM
UT WOS:000363434700004
PM 26409586
DA 2022-07-22
ER

PT J
AU Piontek, T
   Bakowski, P
   Ciemniewska-Gorzela, K
   Grygorowicz, M
AF Piontek, Tomasz
   Bakowski, Pawel
   Ciemniewska-Gorzela, Kinga
   Grygorowicz, Monika
TI Minimally invasive, endoscopic Achilles tendon reconstruction using
   semitendinosus and gracilis tendons with Endobutton stabilization
SO BMC MUSCULOSKELETAL DISORDERS
LA English
DT Article
DE Achilles tendon; Neglected rupture; Achilles endoscopy; Achilles
   reconstruction
ID CHRONIC TEARS; RUPTURES; GRAFT; COMPLICATIONS; DIAGNOSIS
AB Background: Plantaris tendon, peronus brevis tendon and flexor hallucis longus tendon augmentation, commonly used in Achilles tendon rupture, often lead to weakening of injured foot and they require the immobilization after the surgery. It is essential to develop the technique, which gives no such limitation and allows for immediate functional improvement.
   Methods: We present our method of minimally invasive, endoscopic Achilles tendon reconstruction using semitendinosus and gracilis tendons with Endobutton stabilization.
   Results: Posterolateral and posteromedial portals were made approximately 3 cm above the posterosuperior part of the calcaneus to clean the area of the Achilles tendon endoscopically. Then the hamstrings are harvested and prepared for the "Endobutton" system. A midline incision of the skin is performed approximately 1 cm above the posterosuperior part of the calcaneus to approach to the posterosuperior part of the calcaneus. Then under fluoroscopy the calcaneus was drilled through using K-wire. The distal end of the graft equipped with an Endobutton loop was entered into the drilled tunnel in the calcaneus. Later, 8 consecutive skin incisions are performed. Proximal ends of the graft were brought out through the native Achilles tendon reaching medial and lateral skin incisions. The final step was to transfer and tie the graft ends through the most proximal skin incision.
   Conclusions: This minimally invasive, endoscopic technique allows reconstruction of the Achilles tendon using semitendinosus and gracilis tendons with Endobutton stabilization and can be used in so-called "difficult", resistant cases as a "salvage procedure".
C1 [Piontek, Tomasz; Bakowski, Pawel; Ciemniewska-Gorzela, Kinga] Rehasport Clin, Dept Orthoped, Gorecka 30, PL-60201 Poznan, Poland.
   [Grygorowicz, Monika] Rehasport Clin, Dept Res & Dev, Gorecka 30, PL-60201 Poznan, Poland.
RP Bakowski, P (通讯作者)，Rehasport Clin, Dept Orthoped, Gorecka 30, PL-60201 Poznan, Poland.
EM pawel.bakowski@rehasport.pl
RI Grygorowicz, Monika/W-2959-2019; Grygorowicz, Monika
   Małgorzata/A-1357-2012
OI Grygorowicz, Monika/0000-0002-9575-2074; Grygorowicz, Monika
   Małgorzata/0000-0002-9575-2074; Piontek, Tomasz/0000-0002-8604-4682;
   Bakowski, Pawel/0000-0001-8661-7754
CR Ahmad J, 2016, TREATMENT CHRONIC AC
   Bertelli R, 2009, Foot Ankle Surg, V15, P169, DOI 10.1016/j.fas.2008.12.003
   Den Hartog BD, 2008, FOOT ANKLE INT, V29, P456, DOI 10.3113/FAI.2008.0456
   Gulati V, 2015, WORLD J ORTHOP, V6, P380, DOI 10.5312/wjo.v6.i4.380
   Jennings AG, 2002, J BONE JOINT SURG BR, V84B, P361, DOI 10.1302/0301-620X.84B3.11559
   Knobe M, 2015, INT ORTHOP, V39, P535, DOI 10.1007/s00264-014-2615-4
   Lapidus LJ, 2012, INT ORTHOP, V36, P1629, DOI 10.1007/s00264-012-1521-x
   Lee KB, 2009, KNEE SURG SPORT TR A, V17, P316, DOI 10.1007/s00167-008-0693-9
   Longo UG, 2009, SPORTS MED ARTHROSC, V17, P127, DOI 10.1097/JSA.0b013e3181a3d767
   Maffulli N, 2005, CLIN J SPORT MED, V15, P56, DOI 10.1097/01.jsm.0000152714.05097.ef
   Maffulli Nicola, 2007, Foot Ankle Clin, V12, P583, DOI 10.1016/j.fcl.2007.07.007
   Maffulli N, 2008, BMC MUSCULOSKEL DIS, V9, DOI 10.1186/1471-2474-9-100
   Maffulli N, 2010, OPEN ACCESS J SPORTS, V1, P95
   Maffulli N, 2012, J BONE JOINT SURG AM, V94A, P906, DOI 10.2106/JBJS.K.00869
   Maffulli N, 2010, AM J SPORT MED, V38, P2304, DOI 10.1177/0363546510376619
   Mahajan Ravindra H, 2009, J Orthop Surg (Hong Kong), V17, P194
   McClelland D, 2004, SURG-J R COLL SURG E, V2, P209, DOI 10.1016/S1479-666X(04)80002-7
   Molloy A, 2009, FOOT ANKLE CLIN, V14, P745, DOI 10.1016/j.fcl.2009.07.004
   Peffers MJ, 2015, ARTHRITIS RES THER, V17, DOI 10.1186/s13075-015-0544-2
   Peterson KS, 2014, J FOOT ANKLE SURG, V53, P664, DOI 10.1053/j.jfas.2013.10.001
   Piontek T, 2013, PLOS ONE, V8, DOI 10.1371/journal.pone.0082462
   Reiman M, 2014, J ATHL TRAINING, V49, P820, DOI 10.4085/1062-6050-49.3.36
   Sadoghi P, 2012, INT ORTHOP, V36, P1947, DOI 10.1007/s00264-012-1533-6
   Saxena A, 2008, J AM PODIAT MED ASSN, V98, P95
   Singh A, 2014, J ORTHOP SURG-HONG K, V22, P52, DOI 10.1177/230949901402200114
   Takeuchi M, 2014, J MED INVESTIG, V61, P417, DOI 10.2152/jmi.61.417
NR 26
TC 9
Z9 10
U1 0
U2 8
PU BMC
PI LONDON
PA CAMPUS, 4 CRINAN ST, LONDON N1 9XW, ENGLAND
SN 1471-2474
J9 BMC MUSCULOSKEL DIS
JI BMC Musculoskelet. Disord.
PD JUN 3
PY 2016
VL 17
AR 247
DI 10.1186/s12891-016-1099-3
PG 8
WC Orthopedics; Rheumatology
WE Science Citation Index Expanded (SCI-EXPANDED)
SC Orthopedics; Rheumatology
GA DN7IM
UT WOS:000377248700001
PM 27256340
OA gold, Green Published
DA 2022-07-22
ER

PT J
AU Kramer, M
   Gerwing, M
   Michele, U
   Schimke, E
   Kindler, S
AF Kramer, M
   Gerwing, M
   Michele, U
   Schimke, E
   Kindler, S
TI Ultrasonographic examination of injuries to the Achilles tendon in dogs
   and cats
SO JOURNAL OF SMALL ANIMAL PRACTICE
LA English
DT Article
ID SONOGRAPHY; DIAGNOSIS; DISEASES; LESIONS
AB The value of ultrasonography in the diagnosis of lesions of the Achilles tendon was Investigated In 42 dogs and seven cats. A standardised four-part ultrasonographic examination was established. Linear transducers with a frequency of more than 7.5 MHz were used. Ultrasonography allowed identification and differentiation of total ruptures and the differentiation of partial ruptures into deep or superficial ruptures, or those comprising muscular tears. The healing process could be monitored and imaged using this technique. Suture material was readily visible in surgically treated cases. Displacement of the superficial digital flexor tendon could be identified. In such cases, the tendon tissue was seen either medially or laterally to the calcaneus. The healing process of the Achilles tendon could also be documented using ultrasonography. However, it was not possible to determine the age of the injury and the exact end of the healing process. Ultrasonography therefore proved to be an excellent diagnostic method for Imaging lesions of the Achilles tendon and associated Injuries in dogs and cats. After physical examination, the technique should be the next logical step in the evaluation of a suspected Injury to the Achilles tendon.
C1 Univ Giessen, Dept Vet Surg, D-35392 Giessen, Germany.
   Clin Small Anim Dr Stefan Kindler, D-63201 Wiesbaden, Germany.
RP Kramer, M (通讯作者)，State Univ Ghent, Fac Med Vet, Dept Small Anim Med, Salisburylaan 133, B-9820 Merelbeke, Belgium.
CR BLEI CL, 1986, RADIOLOGY, V159, P765, DOI 10.1148/radiology.159.3.3517960
   BLOOMBERG M, 1995, SMALL ANIMAL ORTHOPE, P473
   BURK RL, 1991, LEHRBUCH ATLAS KLEIN, P28
   ENGELKE A, 1996, ATLAS LEHRBUCH ULTRA, P329
   FORNAGE BD, 1986, RADIOLOGY, V159, P759, DOI 10.1148/radiology.159.3.3517959
   FORNAGE BD, 1987, BRIT VET J, V143, P278, DOI 10.1016/0007-1935(87)90091-1
   Hermanson J. W., 1993, Miller's anatomy of the dog., P258
   Kramer M, 1997, VET RADIOL ULTRASOUN, V38, P139, DOI 10.1111/j.1740-8261.1997.tb00829.x
   KRAMER M, 1993, KLEINTIERPRAXIS, V38, P703
   Kramer M, 1998, TIERARZTL PRAX K H, V26, P238
   Lill H, 1996, CHIRURG, V67, P1160, DOI 10.1007/s001040050119
   MAYRHOFER E, 1991, P 16 WSAVA WELTK WIE, P354
   MERK H, 1989, ULTRASCHALL MED, V10, P192, DOI 10.1055/s-2007-1005989
   MEUTSTEGE FJ, 1993, VET COMP ORTHOPAED, V6, P53
   MORGAN DO, 1994, CURR OPIN CELL BIOL, V6, P239, DOI 10.1016/0955-0674(94)90142-2
   MUGHANNAM A, 1994, J AM ANIM HOSP ASSOC, V30, P550
   Owens JM, 1989, RONTGENBILDINTERPRET
   Rivers B. J., 1997, Veterinary and Comparative Orthopaedics and Traumatology, V10, P45
   Vaughan L. C., 1980, California Veterinarian, V34, P15
NR 19
TC 16
Z9 16
U1 0
U2 9
PU BRITISH VETERINARY ASSOC
PI LONDON
PA 7 MANSFIELD ST, LONDON W1M 0AT, ENGLAND
SN 0022-4510
J9 J SMALL ANIM PRACT
JI J. Small Anim. Pract.
PD NOV
PY 2001
VL 42
IS 11
BP 531
EP 535
DI 10.1111/j.1748-5827.2001.tb06022.x
PG 5
WC Veterinary Sciences
WE Science Citation Index Expanded (SCI-EXPANDED)
SC Veterinary Sciences
GA 492UV
UT WOS:000172186700002
PM 11721980
DA 2022-07-22
ER

PT J
AU Posthumus, M
   September, AV
   Schwellnus, MP
   Collins, M
AF Posthumus, Michael
   September, Alison V.
   Schwellnus, Martin P.
   Collins, Malcolm
TI Investigation of the Sp1-binding site polymorphism within the COL1A1
   gene in participants with Achilles tendon injuries and controls
SO JOURNAL OF SCIENCE AND MEDICINE IN SPORT
LA English
DT Article
DE Tendinopathy; Rupture; Overuse injuries; Type I collagen
ID BONE-MINERAL DENSITY; OSTEOGENESIS IMPERFECTA; OSTEOPOROTIC FRACTURE;
   COLLAGEN; WOMEN; TENDINOPATHY; DISORDERS; TISSUE; RISK
AB Sequence variants within the type V collagen (COL5A1) and tenascin C (TNC) genes have to date been shown to be associated with chronic Achilles tendinopathies and/or spontaneous Achilles tendon ruptures. Type V collagen and tenascin C are quantitatively minor components of tendon, while type I collagen is the major structural component. There is increased expression of the COL1A1 gene, which encodes for the oil chain of type I collagen, in the painful Achilles tendon. A functional Sp1-binding site polymorphism (SNP rs1800012; IVS1 + 1023G > T) within this gene has been shown to be associated with several connective tissue disorders. The aim of this study was to determine whether the Sp1-binding site polymorphism within the COL1A1 gene is associated with chronic Achilles tendinopathies and/or spontaneous Achilles tendon ruptures. Achilles tendinopathy (n = 85), Achilles rupture (n = 41) and asymptomatic control (n = 125) participants were genotyped for the COL1A1 Sp1-binding site polymorphism. There were no observed statistical differences in the genotype (p=0.602) or allele (p=0.694) distributions between the groups. In conclusion, this study has shown that there is no association between the Sp1-binding site polymorphism within the first intron of COL1A1 and Achilles tendinopathy or Achilles tendon rupture within the population studied. (C) 2007 Sports Medicine Australia. Published by Elsevier Ltd. All rights reserved.
C1 [Posthumus, Michael; September, Alison V.; Schwellnus, Martin P.; Collins, Malcolm] Univ Cape Town, UCT MRC Res Unit Exercise Sci & Sports Med, ZA-7725 Newlands, South Africa.
   [Posthumus, Michael; September, Alison V.; Schwellnus, Martin P.; Collins, Malcolm] Univ Cape Town, Dept Human Biol, Fac Hlth Sci, ZA-7725 Newlands, South Africa.
   [Collins, Malcolm] S African MRC, Cape Town, South Africa.
RP Collins, M (通讯作者)，Univ Cape Town, UCT MRC Res Unit Exercise Sci & Sports Med, POB 115, ZA-7725 Newlands, South Africa.
EM malcolm.collins@uct.ac.za
RI Collins, Malcolm/G-4046-2011; Schwellnus, Martin/E-6042-2010
OI Collins, Malcolm/0000-0002-2564-0480; Schwellnus,
   Martin/0000-0003-3647-0429; September, Alison V/0000-0003-0950-286X
FU National Research Foundation (NRF) of South African [FA2005021700015];
   University of Cape Town; South African Medical Research Council (MRC)
FX This study was supported in part by funds from the National Research
   Foundation (NRF) of South African (grant no.: FA2005021700015),
   University of Cape Town and South African Medical Research Council
   (MRC). AS was supported by the post-doctoral innovation award from the
   NRF.
CR Birk DE, 2001, MICRON, V32, P223, DOI 10.1016/S0968-4328(00)00043-3
   Canty EG, 2002, COMP BIOCHEM PHYS A, V133, P979, DOI 10.1016/S1095-6433(02)00212-X
   Chagnon YC, 2003, OBES RES, V11, P313, DOI 10.1038/oby.2003.47
   de Mos M, 2007, AM J SPORT MED, V35, P1549, DOI 10.1177/0363546507301885
   DEAK SB, 1985, COLLAGEN REL RES, V5, P305
   Garcia-Giralt N, 2002, J BONE MINER RES, V17, P384, DOI 10.1359/jbmr.2002.17.3.384
   HUERRE C, 1982, P NATL ACAD SCI-BIOL, V79, P6627, DOI 10.1073/pnas.79.21.6627
   Ireland D, 2001, MATRIX BIOL, V20, P159, DOI 10.1016/S0945-053X(01)00128-7
   Jones FS, 2000, DEV DYNAM, V218, P235, DOI 10.1002/(SICI)1097-0177(200006)218:2<235::AID-DVDY2>3.0.CO;2-G
   JUNIEN C, 1982, AM J HUM GENET, V34, P381
   Kannus P, 2000, SCAND J MED SCI SPOR, V10, P312, DOI 10.1034/j.1600-0838.2000.010006312.x
   LAHIRI DK, 1991, NUCLEIC ACIDS RES, V19, P5444, DOI 10.1093/nar/19.19.5444
   Lian K, 2005, ARTHRITIS RHEUM-US, V52, P1431, DOI 10.1002/art.21011
   Mann V, 2001, J CLIN INVEST, V107, P899, DOI 10.1172/JCI10347
   Mao JR, 2001, J CLIN INVEST, V107, P1063, DOI 10.1172/JCI12881
   McGuigan FEA, 2000, OSTEOPOROSIS INT, V11, P338, DOI 10.1007/s001980070123
   Mokone GG, 2006, SCAND J MED SCI SPOR, V16, P19, DOI 10.1111/j.1600-0838.2005.00439.x
   Mokone GG, 2005, AM J SPORT MED, V33, P1016, DOI 10.1177/0363546504271986
   PROCKOP DJ, 1985, ANN NY ACAD SCI, V460, P289, DOI 10.1111/j.1749-6632.1985.tb51176.x
   Rees JD, 2006, RHEUMATOLOGY, V45, P508, DOI 10.1093/rheumatology/kel046
   Riley G, 2004, RHEUMATOLOGY, V43, P131, DOI 10.1093/rheumatology/keg448
   Schepsis AA, 2002, AM J SPORT MED, V30, P287, DOI 10.1177/03635465020300022501
   September AV, 2007, BRIT J SPORT MED, V41, P241, DOI 10.1136/bjsm.2006.033035
   September AV, 2006, INT SPORTMED J, V7, P201
   Skorupski P, 2006, AM J OBSTET GYNECOL, V194, P346, DOI 10.1016/j.ajog.2005.07.034
   Speer G, 2006, ACTA CARDIOL, V61, P321, DOI 10.2143/AC.61.3.2014835
   Stewart TL, 2006, J CLIN ENDOCR METAB, V91, P3575, DOI 10.1210/jc.2005-2651
   Tilkeridis C, 2005, J MED GENET, V42, DOI 10.1136/jmg.2005.033225
NR 28
TC 45
Z9 48
U1 0
U2 6
PU ELSEVIER SCI LTD
PI OXFORD
PA THE BOULEVARD, LANGFORD LANE, KIDLINGTON, OXFORD OX5 1GB, OXON, ENGLAND
SN 1440-2440
EI 1878-1861
J9 J SCI MED SPORT
JI J. Sci. Med. Sport
PD JAN
PY 2009
VL 12
IS 1
BP 184
EP 189
DI 10.1016/j.jsams.2007.12.006
PG 6
WC Sport Sciences
WE Science Citation Index Expanded (SCI-EXPANDED)
SC Sport Sciences
GA 407UV
UT WOS:000263390700031
PM 18353721
DA 2022-07-22
ER

PT J
AU Maffulli, N
   Longo, UG
   Maffulli, GD
   Rabitti, C
   Khanna, A
   Denaro, V
AF Maffulli, Nicola
   Longo, Umile Giuseppe
   Maffulli, Gayle D.
   Rabitti, Carla
   Khanna, Anil
   Denaro, Vincenzo
TI Marked pathological changes proximal and distal to the site of rupture
   in acute Achilles tendon ruptures
SO KNEE SURGERY SPORTS TRAUMATOLOGY ARTHROSCOPY
LA English
DT Article
DE Achilles tendon; Rupture; Histology; Athletes; Sports
ID PERCUTANEOUS REPAIR; PATELLAR TENDONS; TENDINOPATHY; MANAGEMENT;
   HISTOLOGY; COLLAGEN; SURGERY
AB A laboratory study was performed to evaluate the histopathological features of the macroscopically intact portion of the Achilles tendon in patients undergoing surgery for an acute rupture of the Achilles tendon. Tendon samples were harvested from 29 individuals (21 men, 8 women; mean age: 46 +/- A 12) who underwent repair of an Achilles tendon tear tear, and from 11 male patients who died of cardiovascular events (mean age: 61). Three pieces of tendon were harvested: at the rupture site, 4 cm proximal to the site of rupture, 1 cm proximal to the insertion of the Achilles tendon on the calcaneum. Slides were assessed using a semiquantitative grading scale assessing fiber structure and arrangement, rounding of the nuclei, regional variations in cellularity, increased vascularity, decreased collagen stainability, and hyalinization. Intra-observer reliability of the subscore readings was calculated. The pathological features were significantly more pronounced in the samples taken from the site of rupture than in the samples taken proximally and distal to it (0.008 < P < 0.01). There were no significant differences in the mean pathologic sum-scores in the samples taken proximally and distal to the site of rupture. Unruptured Achilles tendons, even at an advanced age, and ruptured Achilles tendons are clearly part of two distinct populations, with the latter demonstrating histopathological evidence of failed healing response even in areas macroscopically normal.
C1 [Maffulli, Nicola; Maffulli, Gayle D.; Khanna, Anil] Mile End Hosp, Barts & London Sch Med & Dent, Ctr Sports & Exercise Med, London E1 4DG, England.
   [Longo, Umile Giuseppe; Denaro, Vincenzo] Campus Biomed Univ, Dept Orthopaed & Trauma Surg, I-00128 Rome, Italy.
   [Rabitti, Carla] Campus Biomed Univ, Dept Surg Pathol, I-00128 Rome, Italy.
RP Maffulli, N (通讯作者)，Mile End Hosp, Barts & London Sch Med & Dent, Ctr Sports & Exercise Med, 275 Bancroft Rd, London E1 4DG, England.
EM n.maffulli@qmul.ac.uk
RI Denaro, Vincenzo/K-9567-2016; Longo, Umile Giuseppe/K-9147-2016;
   Maffulli, Nicola/A-1839-2014
OI Denaro, Vincenzo/0000-0003-4739-9795; Longo, Umile
   Giuseppe/0000-0003-4063-9821; Maffulli, Nicola/0000-0002-5327-3702
CR Ames PRJ, 2008, DISABIL REHABIL, V30, P1646, DOI 10.1080/09638280701785882
   ARNER O, 1959, Acta Chir Scand Suppl, V116, P1
   ASTROMM RA, 1995, CLIN ORTHOP RELAT R, V316, P151
   Cetti R, 2003, ACTA ORTHOP SCAND, V74, P78, DOI 10.1080/00016470310013707
   Chhajed PN, 2002, EUR RESPIR J, V19, P469, DOI 10.1183/09031936.02.00257202
   Cook JL, 2004, J ORTHOP RES, V22, P334, DOI 10.1016/j.orthres.2003.08.005
   Denaro V, 2010, KNEE SURG SPORT TR A, V18, P971, DOI 10.1007/s00167-009-0953-3
   EBINESAN AD, 2008, DISABIL REHABIL, P1
   Forriol F, 2009, INJURY, V40, P44
   Longo UG, 2009, SPORTS MED ARTHROSC, V17, P112, DOI 10.1097/JSA.0b013e3181a3d625
   Longo UG, 2009, SPORTS MED ARTHROSC, V17, P127, DOI 10.1097/JSA.0b013e3181a3d767
   Maffulli N, 2008, RHEUMATOLOGY, V47, P1444, DOI 10.1093/rheumatology/ken337
   Maffulli N, 2008, RHEUMATOLOGY, V47, P390, DOI 10.1093/rheumatology/ken011
   Maffulli N, 1997, AM J SPORT MED, V25, P835, DOI 10.1177/036354659702500618
   Maffulli N, 2000, AM J SPORT MED, V28, P499, DOI 10.1177/03635465000280040901
   Maffulli N, 2004, MED SCI SPORT EXER, V36, P1470, DOI 10.1249/01.MSS.0000139895.94846.8D
   Maffulli N, 2003, CLIN SPORT MED, V22, P761, DOI 10.1016/S0278-5919(03)00009-7
   Maffulli N, 2000, AM J SPORT MED, V28, P857, DOI 10.1177/03635465000280061401
   Maffulli N, 2008, J BONE JOINT SURG AM, V90A, P1348, DOI 10.2106/JBJS.G.01241
   Maffulli Nicola, 2007, Foot Ankle Clin, V12, P583, DOI 10.1016/j.fcl.2007.07.007
   Maffulli N, 2008, CLIN ORTHOP RELAT R, V466, P1605, DOI 10.1007/s11999-008-0261-0
   Maffulli N, 2011, ARCH ORTHOP TRAUM SU, V131, P33, DOI 10.1007/s00402-010-1097-0
   Maffulli Nicola, 2009, Clin J Sport Med, V19, P441, DOI 10.1097/01.jsm.0000360783.69873.4f
   Maffulli N, 2010, CLIN ORTHOP RELAT R, V468, P1039, DOI 10.1007/s11999-009-0944-1
   Maffulli N, 2009, ORTHOP CLIN N AM, V40, P491, DOI 10.1016/j.ocl.2009.05.006
   Matthews TJW, 2006, J BONE JOINT SURG BR, V88B, P489, DOI 10.1302/0301-620X.88B4.16845
   McClelland D, 2002, J ROY COLL SURG EDIN, V47, P613
   Movin T, 1997, ACTA ORTHOP SCAND, V68, P170, DOI 10.3109/17453679709004002
   Murrell GAC, 2007, J SHOULDER ELB SURG, V16, p208S, DOI 10.1016/j.jse.2007.03.007
   Murrell GAC, 2007, BRIT J SPORT MED, V41, P227, DOI 10.1136/bjsm.2006.034447
   NEWNHAM DM, 1991, THORAX, V46, P853, DOI 10.1136/thx.46.11.853
   Nyyssonen T, 2000, ANN CHIR GYNAECOL, V89, P53
   ROYER RJ, 1994, THERAPIE, V49, P75
   Scherb Michael B, 2009, Orthopedics, V32, P26
   Shrier I, 1996, CLIN J SPORT MED, V6, P245, DOI 10.1097/00042752-199610000-00007
   Tallon C, 2001, MED SCI SPORT EXER, V33, P1983, DOI 10.1097/00005768-200112000-00002
NR 36
TC 50
Z9 50
U1 0
U2 9
PU SPRINGER
PI NEW YORK
PA 233 SPRING ST, NEW YORK, NY 10013 USA
SN 0942-2056
J9 KNEE SURG SPORT TR A
JI Knee Surg. Sports Traumatol. Arthrosc.
PD APR
PY 2011
VL 19
IS 4
BP 680
EP 687
DI 10.1007/s00167-010-1193-2
PG 8
WC Orthopedics; Sport Sciences; Surgery
WE Science Citation Index Expanded (SCI-EXPANDED)
SC Orthopedics; Sport Sciences; Surgery
GA 738UT
UT WOS:000288668700026
PM 20563556
DA 2022-07-22
ER

PT J
AU Stein, BE
   Stroh, DA
   Schon, LC
AF Stein, Benjamin E.
   Stroh, David Alex
   Schon, Lew C.
TI Outcomes of acute Achilles tendon rupture repair with bone marrow
   aspirate concentrate augmentation
SO INTERNATIONAL ORTHOPAEDICS
LA English
DT Article
DE Achilles; Tendon; Rupture; Repair; Biologic
ID PLATELET-RICH PLASMA; MESENCHYMAL STEM-CELLS; ROTATOR CUFF REPAIR;
   NONOPERATIVE MANAGEMENT; NONSURGICAL TREATMENT; INCREASING INCIDENCE;
   SURGICAL REPAIR; RAT MODEL; EFFICACY
AB Purpose Optimal treatment of acute Achilles tendon ruptures remains controversial. Positive results using stem-cell-bearing concentrates have been reported with other soft-tissue repairs, but no studies exist on outcomes of bone marrow aspirate concentrate (BMAC) augmentation in primary Achilles tendon repair.
   Methods We reviewed patients with sport-related Achilles tendon ruptures treated via open repair augmented with BMAC injection from 2009 to 2011. Data on operative complications, strength, range of motion, rerupture, calf circumference and functional improvement through progressive return to sport and the Achilles tendon Total Rupture Score (ATRS) were analysed.
   Results A total of 27 patients (28 tendons) treated with open repair and BMAC injection were identified (mean age 38.3 +/- 9.6 years). At mean follow-up of 29.7 +/- 6.1 months, there were no reruptures. Walking without a boot was at 1.8 +/- 0.7 months, participation in light activity was at 3.4 +/- 1.8 months and 92 % (25 of 27) of patients returned to their sport at 5.9 +/- 1.8 months. Mean ATRS at final follow-up was 91 (range 72-100) points. One case of superficial wound dehiscence healed with local wound care. No soft-tissue masses, bone formation or tumors were observed in the operative extremity.
   Conclusions Excellent results, including no re-ruptures and early mobilisation, were observed in this small cohort with open Achilles tendon repair augmented by BMAC. No adverse outcomes of biologic treatment were observed with this protocol. The efficacy of BMAC in the operative repair of acute Achilles tendon ruptures warrants further study.
   Level of Evidence: IV - Therapeutic.
C1 [Stein, Benjamin E.; Stroh, David Alex; Schon, Lew C.] MedStar Union Mem Hosp, Dept Orthopaed Surg, Baltimore, MD 21218 USA.
RP Schon, LC (通讯作者)，MedStar Union Mem Hosp, Dept Orthopaed Surg, 3333 North Calvert St,Suite 400, Baltimore, MD 21218 USA.
EM lewschon@comcast.net
CR Adams SB, 2014, FOOT ANKLE INT, V35, P293, DOI 10.1177/1071100713519078
   Castricini R, 2011, AM J SPORT MED, V39, P258, DOI 10.1177/0363546510390780
   Courneya JP, 2010, FIBROGENESIS TISSUE, V3, DOI 10.1186/1755-1536-3-9
   Del Buono A, 2013, INT ORTHOP, V37, P715, DOI 10.1007/s00264-012-1743-y
   Jia XF, 2011, OPER TECHN SPORT MED, V19, P177, DOI 10.1053/j.otsm.2011.04.003
   Jones Matthew P, 2012, J Bone Joint Surg Am, V94, pe88, DOI 10.2106/JBJS.J.01829
   Knobe M, 2015, INT ORTHOP, V39, P535, DOI 10.1007/s00264-014-2615-4
   Kocher MS, 2002, AM J SPORT MED, V30, P783, DOI 10.1177/03635465020300060501
   Kolodziej L, 2013, INT ORTHOP, V37, P625, DOI 10.1007/s00264-012-1737-9
   Maffulli N, 1998, Bull Hosp Jt Dis, V57, P152
   McCarrel T, 2009, J ORTHOP RES, V27, P1033, DOI 10.1002/jor.20853
   Moller A, 1996, ACTA ORTHOP SCAND, V67, P479, DOI 10.3109/17453679608996672
   Nilsson-Helander K, 2007, AM J SPORT MED, V35, P421, DOI 10.1177/0363546506294856
   Nyyssonen T, 2008, SCAND J SURG, V97, P272, DOI 10.1177/145749690809700312
   Olsson N, 2013, AM J SPORT MED, V41, P2867, DOI 10.1177/0363546513503282
   Randelli P, 2011, J SHOULDER ELB SURG, V20, P518, DOI 10.1016/j.jse.2011.02.008
   Rodeo SA, 2012, AM J SPORT MED, V40, P1234, DOI 10.1177/0363546512442924
   Sanchez M, 2007, AM J SPORT MED, V35, P245, DOI 10.1177/0363546506294078
   Schepull T, 2011, AM J SPORT MED, V39, P38, DOI 10.1177/0363546510383515
   Schon LC, 2014, J TRANSL MED, V12, DOI 10.1186/1479-5876-12-110
   Soroceanu A, 2012, J BONE JOINT SURG AM, V94A, P2136, DOI 10.2106/JBJS.K.00917
   Suchak AA, 2005, FOOT ANKLE INT, V26, P932, DOI 10.1177/107110070502601106
   Tallon C, 2001, MED SCI SPORT EXER, V33, P1983, DOI 10.1097/00005768-200112000-00002
   Urdzikova LM, 2014, BIOMED ENG ONLINE, V13, DOI 10.1186/1475-925X-13-42
   Winter E, 1998, ARCH ORTHOP TRAUM SU, V117, P364, DOI 10.1007/s004020050267
   Wong J, 2002, AM J SPORT MED, V30, P565, DOI 10.1177/03635465020300041701
   Yao J, 2012, J HAND SURG-AM, V37A, P1639, DOI 10.1016/j.jhsa.2012.04.038
   Yokoya S, 2012, AM J SPORT MED, V40, P1259, DOI 10.1177/0363546512442343
NR 28
TC 31
Z9 33
U1 1
U2 12
PU SPRINGER
PI NEW YORK
PA 233 SPRING ST, NEW YORK, NY 10013 USA
SN 0341-2695
EI 1432-5195
J9 INT ORTHOP
JI Int. Orthop.
PD MAY
PY 2015
VL 39
IS 5
BP 901
EP 905
DI 10.1007/s00264-015-2725-7
PG 5
WC Orthopedics
WE Science Citation Index Expanded (SCI-EXPANDED)
SC Orthopedics
GA CG5RD
UT WOS:000353350700012
PM 25795246
DA 2022-07-22
ER

PT J
AU Blankstein, A
   Israeli, A
   Dudkiewicz, I
   Chechik, A
   Ganel, A
AF Blankstein, Alexander
   Israeli, Amnon
   Dudkiewicz, Israel
   Chechik, Aharon
   Ganel, Avraham
TI Percutaneous Achilles tendon repair combined with real-time sonography
SO ISRAEL MEDICAL ASSOCIATION JOURNAL
LA English
DT Article
DE sonography; ultrasound; Achilles tendon; repair; ruptures
ID NON-SURGICAL TREATMENT; RUPTURES; DIAGNOSIS; ULTRASONOGRAPHY
AB Background: When encountering complaints of pain in the area of Achilles tendon with a suspected lesion, the clinician seldom reaches a precise diagnosis based only on X-ray and clinical examination. Ultrasonography is useful for evaluating the pathology and treatment.
   Objectives: To assess the relative contribution of real-time intraoperative ultrasound examination and immediate postoperative ultrasound in patients with acute rupture of the Achilles tendon treated by percutaneous suture method. The combination of both procedures provides a unique advantage that could facilitate better results.
   Methods: Ultrasound examination was used in 20 patients with acute rupture of the Achilles tendon who were treated surgically. performed in 5 patients while 15 patients underwent an immediate postoperative ultrasound.
   Results: Ultrasound pathologies were found in all patients. Percutaneous surgical correction of ruptured Achilles tendon with accurate positioning of the foot using real-time sonography was successful in all the patients.
   Conclusion: As in many other soft tissue lesions, ultrasonography is a useful tool for evaluating the pathology and for planning the surgical correction of ruptures in the Achilles tendon.
C1 Chaim Sheba Med Ctr, Dept Orthoped Surg & Diagnost Imaging, IL-52621 Tel Hashomer, Israel.
   Chaim Sheba Med Ctr, Dept Diagnost Imaging, IL-52621 Tel Hashomer, Israel.
RP Blankstein, A (通讯作者)，Chaim Sheba Med Ctr, Dept Orthoped Surg & Diagnost Imaging, IL-52621 Tel Hashomer, Israel.
EM blankali@zahav.net.il
CR BRADLEY JP, 1990, AM J SPORT MED, V18, P188, DOI 10.1177/036354659001800213
   Buchgraber A, 1997, CLIN ORTHOP RELAT R, P113
   FITZGIBBONS RE, 1993, AM J SPORT MED, V21, P724, DOI 10.1177/036354659302100516
   HAGGMARK T, 1986, ORTHOPEDICS, V9, P160
   INGLIS AE, 1976, J BONE JOINT SURG AM, V58, P990, DOI 10.2106/00004623-197658070-00015
   Jacobs D, 1978, Am J Sports Med, V6, P107, DOI 10.1177/036354657800600302
   KAINBERGER FM, 1990, AM J ROENTGENOL, V155, P1031, DOI 10.2214/ajr.155.5.2120931
   Karjalainen PT, 1996, ACTA RADIOL, V37, P639
   KLEIN W, 1991, Chirurgia degli Organi di Movimento, V76, P223
   MA GWC, 1977, CLIN ORTHOP RELAT R, P247
   Mertl P, 1999, REV CHIR ORTHOP, V85, P277
   Nehrer S, 1997, ARCH ORTHOP TRAUM SU, V116, P14, DOI 10.1007/BF00434093
   NISTOR L, 1981, J BONE JOINT SURG AM, V63, P394, DOI 10.2106/00004623-198163030-00012
   OREILLY MAR, 1993, CLIN RADIOL, V48, P202, DOI 10.1016/S0009-9260(05)80284-3
   Paavola M, 1998, ACTA RADIOL, V39, P612, DOI 10.3109/02841859809175485
   WILLS CA, 1986, CLIN ORTHOP RELAT R, V207, P156
NR 16
TC 16
Z9 16
U1 0
U2 2
PU ISRAEL MEDICAL ASSOC JOURNAL
PI RAMAT GAN
PA 2 TWIN TOWERS, 11TH FL, 35 JABOTINSKY ST, PO BOX 3604, RAMAT GAN 52136,
   ISRAEL
SN 1565-1088
J9 ISR MED ASSOC J
JI Isr. Med. Assoc. J.
PD FEB
PY 2007
VL 9
IS 2
BP 83
EP 85
PG 3
WC Medicine, General & Internal
WE Science Citation Index Expanded (SCI-EXPANDED)
SC General & Internal Medicine
GA 147CF
UT WOS:000244980300005
PM 17348477
DA 2022-07-22
ER

PT J
AU De la Fuente, C
   Ramirez-Campillo, R
   Gallardo-Fuentes, F
   Alvarez, C
   Bustamante, C
   Henriquez, H
   Carpes, FP
AF De la Fuente, Carlos
   Ramirez-Campillo, Rodrigo
   Gallardo-Fuentes, Francisco
   Alvarez, Cristian
   Bustamante, Carlos
   Henriquez, Hugo
   Carpes, Felipe P.
TI Pattern analysis of a complete Achilles tendon rupture suffered during
   high jump preparation in an official national-level athletic competition
SO SPORTS BIOMECHANICS
LA English
DT Article
DE Kinematics; sports medicine; image analysis biomechanics; athlete;
   injury
ID QUADRICEPS; FATIGUE; REPAIR; RETURN; PLAY
AB Elite athletes are subject to injuries like the Achilles tendon rupture. This injury requires a long recovery process, with no guarantee of returning to the pre-injury level. When a rupture happens during natural life, movement analysis can provide useful insights concerning patterns of rupture to understand and prevent Achilles tendon injuries. Here we determined the pattern of rupture of an Achilles tendon (AT) in an elite high-jumper athlete who ruptured an AT during the straight line running phase in preparation for a high-jump attempt. This study is a novel case report regarding a national-level elite athlete. The main outcomes were kinematics parameters obtained from video analysis. The pattern of the rupture was determined by pixel intensity and outlier analysis. The rupture occurred at 44% of the single stance. The injured leg showed a higher ankle dorsal flexion and knee-ankle ratio, and a lower knee flexion compared to contralateral leg. An eccentric pattern of rupture occurred during the transition from the mid to terminal stance phases of running. The lower knee flexion and the increased ankle dorsal flexion during the stance suggest a loss of knee-ankle coordination. This might have favoured a major elongation of Achilles tendon causing the rupture.
C1 [De la Fuente, Carlos] Univ Andes, Sch Kinesiol, Integrat Lab Biomech & Efft Physiol, Santiago, Chile.
   [De la Fuente, Carlos; Henriquez, Hugo] Santa Maria Clin, Ctr Sport Hlth, Santiago, Chile.
   [De la Fuente, Carlos] Fed Univ Pampa, Lab Neuromech, Uruguaiana, Brazil.
   [Ramirez-Campillo, Rodrigo; Gallardo-Fuentes, Francisco; Alvarez, Cristian] Pontificia Univ Catolica Chile, Fac Med, Dept Hlth Sci, Kinesiol Carrer, Santiago, Chile.
   [Bustamante, Carlos] Univ Lakes, Dept Phys Act Sci, Qual Life & Wellness Grp, Lab Human Performance, Osorno, Chile.
   [Henriquez, Hugo] Integral Ctr Med & Exercise, Puerto Montt, Chile.
   [Carpes, Felipe P.] Univ Chile, Fac Med, Traumatol & Orthopaed, Santiago, Chile.
RP Carpes, FP (通讯作者)，Univ Chile, Fac Med, Traumatol & Orthopaed, Santiago, Chile.
EM carpes@unipampa.edu.br
RI Carpes, Felipe P./A-1069-2010; ALvarez, Cristian/S-8150-2019;
   Ramirez-Campillo, Rodrigo/M-1258-2016
OI Carpes, Felipe P./0000-0001-8923-4855; ALvarez,
   Cristian/0000-0003-4783-9981; Ramirez-Campillo,
   Rodrigo/0000-0003-2035-3279; Gallardo-Fuentes,
   Francisco/0000-0002-3985-157X; De la Fuente, Carlos/0000-0002-2429-837X
FU National Council for Scientific and Technological Development (CNPq)
   from Brazil
FX Felipe P Carpes is supported by the National Council for Scientific and
   Technological Development (CNPq) from Brazil.
CR Barbieri FA, 2014, ERGONOMICS, V57, P934, DOI 10.1080/00140139.2014.903302
   BARFRED T, 1971, ACTA ORTHOP SCAND, V42, P406, DOI 10.3109/17453677108989062
   Chiodo CP, 2006, FOOT ANKLE INT, V27, P305, DOI 10.1177/107110070602700415
   Cohn TA, 2013, WATER RESOUR RES, V49, P5047, DOI 10.1002/wrcr.20392
   Cooper MT, 2015, CLIN SPORT MED, V34, P595, DOI 10.1016/j.csm.2015.06.001
   Gooyers CE, 2013, CLIN BIOMECH, V28, P436, DOI 10.1016/j.clinbiomech.2013.02.002
   Hess Gregory William, 2010, Foot Ankle Spec, V3, P29, DOI 10.1177/1938640009355191
   INGLIS AE, 1981, CLIN ORTHOP RELAT R, P160
   Jandacka Daniel, 2013, BMJ Case Rep, V2013, DOI 10.1136/bcr-2012-007370
   Kim SM, 2019, J SPORT SCI, V37, P2331, DOI 10.1080/02640414.2019.1632604
   Maffulli N, 1998, Bull Hosp Jt Dis, V57, P152
   Munro BH, 2005, STAT METHOD HLTH CAR
   Oliveira RB, 2016, COMPUT METH PROG BIO, V131, P127, DOI 10.1016/j.cmpb.2016.03.032
   Shim VB, 2014, J BIOMECH, V47, P3598, DOI 10.1016/j.jbiomech.2014.10.001
   Singh R, 2014, ASHRAE TRAN, V120
   Souza RB, 2016, PHYS MED REH CLIN N, V27, P217, DOI 10.1016/j.pmr.2015.08.006
   Thomas AC, 2010, J APPL BIOMECH, V26, P159, DOI 10.1123/jab.26.2.159
   Thomopoulos S, 2015, J ORTHOP RES, V33, P832, DOI 10.1002/jor.22806
   Trofa DP, 2017, AM J SPORT MED, V45, P2864, DOI 10.1177/0363546517713001
   Winter D. A., 2009, BIOMECHANICS MOTOR C, V4th
   Zellers JA, 2016, INT J SPORTS PHYS TH, V11, P1150
NR 21
TC 2
Z9 2
U1 1
U2 7
PU ROUTLEDGE JOURNALS, TAYLOR & FRANCIS LTD
PI ABINGDON
PA 2-4 PARK SQUARE, MILTON PARK, ABINGDON OX14 4RN, OXON, ENGLAND
SN 1476-3141
EI 1752-6116
J9 SPORT BIOMECH
JI Sport. Biomech.
PD MAR 16
PY 2022
VL 21
IS 3
BP 312
EP 322
DI 10.1080/14763141.2019.1651897
EA AUG 2019
PG 11
WC Engineering, Biomedical; Sport Sciences
WE Science Citation Index Expanded (SCI-EXPANDED)
SC Engineering; Sport Sciences
GA YZ2TR
UT WOS:000484491300001
PM 31462176
DA 2022-07-22
ER

PT J
AU Peterson, KS
   Hentges, MJ
   Catanzariti, AR
   Mendicino, MR
   Mendicino, RW
AF Peterson, Kyle S.
   Hentges, Matthew J.
   Catanzariti, Alan R.
   Mendicino, Michael R.
   Mendicino, Robert W.
TI Surgical Considerations for the Neglected or Chronic Achilles Tendon
   Rupture: A Combined Technique for Reconstruction
SO JOURNAL OF FOOT & ANKLE SURGERY
LA English
DT Article
DE ankle; flexor hallucis longus; injury; leg; popliteal block; tendon
   transfer; triceps surae
ID REPAIR; FLAP
AB The Achilles tendon is among the most commonly injured tendons in the human body. The most common reason for delayed treatment is a missed diagnosis or a deficiency in presentation. The neglected or chronically ruptured Achilles tendon presents a unique treatment challenge. The surgical approach varies greatly depending on the extent of degeneration and the resultant gap between the opposing tendon ends. Most surgeons have recommended the use of a tendon transfer or augmentation to strengthen the Achilles tendon repair. The following technique uses a flexor hallucis longus tendon transfer with gastrocnemius aponeurosis turndown flap augmentation. This technique has been commonly performed by us with success. (C) 2014 by the American College of Foot and Ankle Surgeons. All rights reserved.
C1 [Peterson, Kyle S.; Hentges, Matthew J.] Western Penn Hosp, Div Foot & Ankle Surg, Pittsburgh, PA 15224 USA.
   [Catanzariti, Alan R.; Mendicino, Robert W.] Western Penn Hosp, Pittsburgh, PA 15224 USA.
   [Mendicino, Michael R.] Washington & Jefferson Coll, Washington, PA USA.
   [Mendicino, Robert W.] OhioHlth Orthoped Surg, Hilliard, OH 43026 USA.
   [Mendicino, Robert W.] Grant Med Ctr, Columbus, OH USA.
RP Mendicino, RW (通讯作者)，OhioHlth Orthoped Surg, 3663 Ridge Mill Dr, Hilliard, OH 43026 USA.
EM rmendicino@faiwp.com
CR ABRAHAM E, 1975, J BONE JOINT SURG AM, VA 57, P253, DOI 10.2106/00004623-197557020-00019
   CHRISTENSEN I, 1953, ACTA CHIR SCAND, V106, P50
   Coull R, 2003, FOOT ANKLE INT, V24, P931, DOI 10.1177/107110070302401211
   Den Hartog BD, 2003, FOOT ANKLE INT, V24, P233, DOI 10.1177/107110070302400306
   Den Hartog BD, 2008, FOOT ANKLE INT, V29, P456, DOI 10.3113/FAI.2008.0456
   DENHARTOG BD, 2002, TECH FOOT ANKLE SURG, V1, P145
   Elias I, 2007, FOOT ANKLE INT, V28, P1238, DOI 10.3113/FAI.2007.1238
   GERDES MH, 1992, CLIN ORTHOP RELAT R, P241
   Gerhardt K., 1937, ARCH KLIN CHIR, V189, P681
   Gilcreest E, 1953, JAMA-J AM MED ASSOC, V100, P153
   KRUEGERFRANKE M, 1995, BRIT J SPORT MED, V29, P121, DOI 10.1136/bjsm.29.2.121
   LAGERGREN C, 1959, Acta Chir Scand, V116, P491
   Leslie Hamish D H, 2005, Foot Ankle Clin, V10, P357, DOI 10.1016/j.fcl.2005.01.009
   Maffulli N, 1999, J BONE JOINT SURG AM, V81A, P1019, DOI 10.2106/00004623-199907000-00017
   Maffulli N, 1996, BRIT J SPORT MED, V30, P250, DOI 10.1136/bjsm.30.3.250
   Maffulli Nicola, 2007, Foot Ankle Clin, V12, P583, DOI 10.1016/j.fcl.2007.07.007
   MANN RA, 1991, J BONE JOINT SURG AM, V73A, P214, DOI 10.2106/00004623-199173020-00009
   Padanilam TG, 2009, FOOT ANKLE CLIN, V14, P711, DOI 10.1016/j.fcl.2009.08.001
   PEREZTEUFFER A, 1974, ORTHOP CLIN N AM, V5, P89
   Platt H, 1931, BRIT MED J, V1931, P611, DOI 10.1136/bmj.1.3666.611
   Ponnapula P, 2010, J FOOT ANKLE SURG, V49, P310, DOI 10.1053/j.jfas.2010.02.021
   RUSH JH, 1980, AUST NZ J SURG, V50, P420, DOI 10.1111/j.1445-2197.1980.tb04156.x
   SCHELLER AD, 1980, ORTHOP CLIN N AM, V11, P801
   Silfverskiold F., 1941, ACTA CHIR SCAND, V84, P393
   Wapner K L, 1993, Foot Ankle, V14, P443
   Zadek I, 1940, J BONE JOINT SURG, V22, P1070
NR 26
TC 15
Z9 16
U1 0
U2 5
PU ELSEVIER SCIENCE INC
PI NEW YORK
PA 360 PARK AVE SOUTH, NEW YORK, NY 10010-1710 USA
SN 1067-2516
EI 1542-2224
J9 J FOOT ANKLE SURG
JI J. Foot Ankle Surg.
PD SEP-OCT
PY 2014
VL 53
IS 5
BP 664
EP 671
DI 10.1053/j.jfas.2013.10.001
PG 8
WC Orthopedics; Surgery
WE Science Citation Index Expanded (SCI-EXPANDED)
SC Orthopedics; Surgery
GA AO2MW
UT WOS:000341158500029
PM 24269103
DA 2022-07-22
ER

PT J
AU Carmont, MR
   Highland, AM
   Blundell, CM
   Davies, MB
AF Carmont, Michael R.
   Highland, Adrian M.
   Blundell, Christopher M.
   Davies, Mark B.
TI Simultaneous bilateral Achilles tendon ruptures associated with statin
   medication despite regular rock climbing exercise
SO PHYSICAL THERAPY IN SPORT
LA English
DT Article
DE Bilateral; Achilles tendon; Rupture; Rock climbing; Statin
AB Introduction: Ruptures of the Achilles tendon are common however simultaneous ruptures occur less frequently. Eccentric loading exercise programmes have been used to successfully treat Achilles tendinopathy.
   Case report. We report a case of simultaneous bilateral Achilles tendon rupture in a patient predisposed to rupture due to longstanding raised serum lipoprotein and recently introduced therapeutic statin medication. The patient was also a keen rock climber and had regularly undertaken loading exercise.
   Conclusion: This case illustrates that the therapeutic effect of mixed loading exercises for the Achilles tendon may not be adequate to overcome the predisposition to rupture caused by hyperlipidaemia and statin medication. (C) 2009 Elsevier Ltd. All rights reserved.
C1 [Carmont, Michael R.] No Gen Hosp, Dept Orthopaed Surg, Foot & Ankle Off, Sheffield Foot & Ankle Unit, Sheffield S5 7AU, S Yorkshire, England.
   [Highland, Adrian M.] No Gen Hosp, Dept Radiol, Sheffield S5 7AU, S Yorkshire, England.
RP Carmont, MR (通讯作者)，No Gen Hosp, Dept Orthopaed Surg, Foot & Ankle Off, Sheffield Foot & Ankle Unit, Sorby Bldg,Herries Rd, Sheffield S5 7AU, S Yorkshire, England.
EM mcarmont@hotmail.com
CR Calder JDF, 2006, FOOT ANKLE INT, V27, P93, DOI 10.1177/107110070602700204
   Chiodo CP, 2006, FOOT ANKLE INT, V27, P305, DOI 10.1177/107110070602700415
   Karousou E, 2008, CLIN ORTHOP RELAT R, V466, P1577, DOI 10.1007/s11999-008-0255-y
   Khan RJK, 2005, J BONE JOINT SURG AM, V87A, P2202, DOI 10.2106/JBJS.D.03049
   MAFFULLI N, 1989, J SPORT MED PHYS FIT, V29, P365
   Marie I, 2008, ARTHRIT RHEUM-ARTHR, V59, P367, DOI 10.1002/art.23309
   Mathiak G, 1999, ARCH ORTHOP TRAUM SU, V119, P280, DOI 10.1007/s004020050410
   Mihaylova B, 2006, BMJ-BRIT MED J, V333, P1145, DOI 10.1136/bmj.38993.731725.BE
   Ohberg L, 2004, BRIT J SPORT MED, V38, P8, DOI 10.1136/bjsm.2001.000284
   Pullatt RC, 2007, AM J CARDIOL, V100, P152, DOI 10.1016/j.amjcard.2007.02.068
   Schweizer A, 2005, BRIT J SPORT MED, V39, P429, DOI 10.1136/bjsm.2002.001016
NR 11
TC 20
Z9 20
U1 0
U2 11
PU CHURCHILL LIVINGSTONE
PI EDINBURGH
PA JOURNAL PRODUCTION DEPT, ROBERT STEVENSON HOUSE, 1-3 BAXTERS PLACE,
   LEITH WALK, EDINBURGH EH1 3AF, MIDLOTHIAN, SCOTLAND
SN 1466-853X
J9 PHYS THER SPORT
JI Phys. Ther. Sport
PD NOV
PY 2009
VL 10
IS 4
BP 150
EP 152
DI 10.1016/j.ptsp.2009.01.003
PG 3
WC Rehabilitation; Sport Sciences
WE Science Citation Index Expanded (SCI-EXPANDED)
SC Rehabilitation; Sport Sciences
GA 526RU
UT WOS:000272311600007
PM 19897169
DA 2022-07-22
ER

PT J
AU Dobke, M
   Suliman, A
   Mackert, GA
   Herrera, FA
   Singer, R
   Nelson, J
AF Dobke, Marek
   Suliman, Ahmed
   Mackert, Gina A.
   Herrera, Fernando A.
   Singer, Robert
   Nelson, Jeffrey
TI Single-Stage Reconstruction of Achilles Tendon and Overlying Tissue With
   the Extended Temporoparietal Fasciagaleal Flap-23-Year Follow-Up and the
   Review of the Literature
SO ANNALS OF PLASTIC SURGERY
LA English
DT Article; Proceedings Paper
CT 65th Annual Meeting of the California-Society-of-Plastic-Surgeons (CSPS)
CY MAY 22-25, 2015
CL Monterey, CA
SP Calif Soc Plast Surg
DE Achilles tendon; Achilles tendon defect; functional reconstruction;
   tendon contracture; lower extremity reconstruction; surgical flaps
ID FUNCTIONAL RECONSTRUCTION; UNIT LENGTH; DEFECT; RUPTURE; INJURY; BONE;
   FLAP
AB In the absence of an established "gold standard" for complex Achilles tendon and regional soft tissue defect reconstruction, many techniques have been advocated. Two cases describing a novel technique of successful repair with the review of literature are presented. The underlying problem consisted of Achilles tendon necrosis with local inflammation in the first case and tendon contracture with foot malposition due to a burn injury in the other. Each patient, upon debridement, had a 6-cm Achilles tendon defect with associated overlying soft tissue deficits reconstructed with an extended temporoparietal fasciagaleal flap and a split thickness skin graft. Both cases highlight the successful functional and aesthetic quality as well as the durability of concurrent vascularized tendon and soft tissue replacement and coverage in 2 distinct clinical scenarios.
C1 [Dobke, Marek; Suliman, Ahmed; Mackert, Gina A.; Herrera, Fernando A.; Singer, Robert; Nelson, Jeffrey] Univ Calif San Diego, Dept Surg, Div Plast Surg, San Diego, CA 92103 USA.
RP Dobke, M (通讯作者)，Univ Calif San Diego, Div Plast Surg, 200 West Arbor Dr,MC 8890, San Diego, CA 92103 USA.
EM mdobke@ucsd.edu
OI Herrera, Fernando/0000-0001-6957-0413
CR Abrams RA, 2000, MUSCLE NERVE, V23, P707, DOI 10.1002/(SICI)1097-4598(200005)23:5<707::AID-MUS7>3.0.CO;2-T
   Berthe JV, 2000, ADV UPPER LOWER EXTR, P140
   Bohnsack M, 2000, Z ORTHOP GRENZGEB, V138, P501, DOI 10.1055/s-2000-9591
   BRENT B, 1985, PLAST RECONSTR SURG, V76, P177, DOI 10.1097/00006534-198508000-00001
   Chung CY, 2015, J PEDIATR ORTHOPED, V35, P419, DOI 10.1097/BPO.0000000000000278
   DeFazio MV, 2014, ARCH PLAST SURG-APS, V41, P285, DOI 10.5999/aps.2014.41.3.285
   FEIBEL RJ, 1993, J RECONSTR MICROSURG, V9, P321, DOI 10.1055/s-2007-1006737
   Grove JR, 2008, FOOT ANKLE ONLINE J, V1, P1
   GUAN WX, 1985, ANN PLAS SURG, V15, P515, DOI 10.1097/00000637-198512000-00010
   HARSHAI Y, 1992, PLAST RECONSTR SURG, V89, P64, DOI 10.1097/00006534-199289010-00012
   Haynes DF, 2007, PLAST RECONSTR SURG, V120, P1090, DOI 10.1097/01.prs.0000278222.30739.1b
   Iorio ML, 2015, ANN PLAS SURG, V74, P121, DOI 10.1097/SAP.0b013e31828bb353
   Iwanuma S, 2011, J BIOMECH, V44, P2579, DOI 10.1016/j.jbiomech.2011.07.003
   Kuo YR, 2003, ANN PLAS SURG, V50, P149, DOI 10.1097/01.SAP.0000037270.95257.B9
   Lee HB, 1999, PLAST RECONSTR SURG, V104, P111, DOI 10.1097/00006534-199907000-00017
   Lee JW, 2000, BRIT J PLAST SURG, V53, P574, DOI 10.1054/bjps.2000.3407
   Liu TY, 2009, BURNS, V35, P852, DOI 10.1016/j.burns.2008.09.008
   Maquirriain Javier, 2011, Yale Journal of Biology and Medicine, V84, P289
   MARKHEDE G, 1981, ACTA ORTHOP SCAND, V52, P373, DOI 10.3109/17453678109050118
   Mastacaneanu M, 2010, TIMISOARA MED J, V60, P154
   MOFFETT TR, 1992, PLAST RECONSTR SURG, V89, P259, DOI 10.1097/00006534-199202000-00010
   Naganawa T, 2009, J Orthop Surg (Hong Kong), V17, P116
   Nazerali RS, 2013, ANN PLAS SURG, V70, P416, DOI 10.1097/SAP.0b013e3182853d6c
   Ronel DN, 2004, MICROSURG, V24, P18, DOI 10.1002/micr.10191
   Ronel DN, 2007, THE ACHILLES TENDON, P208
   Rosso C, 2012, CLIN J SPORT MED, V22, P483, DOI 10.1097/JSM.0b013e3182639a3e
   Sabapathy S Raja, 2013, Indian J Plast Surg, V46, P121, DOI 10.4103/0970-0358.113729
   Sharma P, 2005, J BONE JOINT SURG AM, V87A, P187, DOI 10.2106/JBJS.D.01850
   Soons J, 2015, BONE JOINT J, V97B, P215, DOI 10.1302/0301-620X.97B2.34521
   SUOMINEN E, 1992, ACTA ORTHOP SCAND, V63, P482, DOI 10.3109/17453679209154719
   Upadhyaya DN, 2012, INDIAN J PLAST SURG, V45, P572, DOI 10.4103/0970-0358.105983
   WEI FC, 1988, PLAST RECONSTR SURG, V81, P579, DOI 10.1097/00006534-198804000-00015
   Wei ZR, 2014, ANN PLAS SURG, V73, P325, DOI 10.1097/SAP.0b013e31827a3007
   Yuen TJ, 2006, GAIT POSTURE, V23, P112, DOI 10.1016/j.gaitpost.2004.12.007
   Zhu YL, 2014, J RECONSTR MICROSURG, V30, P569, DOI 10.1055/s-0033-1358787
NR 35
TC 3
Z9 5
U1 0
U2 6
PU LIPPINCOTT WILLIAMS & WILKINS
PI PHILADELPHIA
PA TWO COMMERCE SQ, 2001 MARKET ST, PHILADELPHIA, PA 19103 USA
SN 0148-7043
EI 1536-3708
J9 ANN PLAS SURG
JI Ann. Plast. Surg.
PD MAY
PY 2016
VL 76
SU 3
BP S165
EP S170
DI 10.1097/SAP.0000000000000789
PG 6
WC Surgery
WE Science Citation Index Expanded (SCI-EXPANDED); Conference Proceedings Citation Index - Science (CPCI-S)
SC Surgery
GA DK6UO
UT WOS:000375061200006
PM 27070674
DA 2022-07-22
ER

PT J
AU Zantop, T
   Tillmann, B
   Petersen, W
AF Zantop, T
   Tillmann, B
   Petersen, W
TI Quantitative assessment of blood vessels of the human Achilles tendon:
   an immunohistochemical cadaver study
SO ARCHIVES OF ORTHOPAEDIC AND TRAUMA SURGERY
LA English
DT Article
DE Achilles tendon; vascularity; tendon degeneration; spontaneous rupture;
   laminin
ID RUPTURE
AB Background. The pathogenesis of Achilles tendon rupture remains unclear, but vascular patterns may play an important role. Hypoxia is considered to be an important factor in the aetiology of tendon degeneration. Methods. Statements from the literature regarding the vascularization of the Achilles tendon are controversial. We determined the vascular density of the Achilles tendon using a new method involving antibodies against laminin, a component of the basement membrane. Results. The blood supply of the Achilles tendon mainly arose from the anterior paratenon of the tendon from which vessels run into the tendon. The proximal part of the tendon was supplied by a recurrent branch of the posterior tibial artery, while the distal part of the tendon was vascularized by the rete arteriosum calcaneare, supplied by the fibular and posterior tibial arteries. An avascular area could be seen close to the insertion of the tendon to the calcaneus. Three regions with different vascular density could be determined in the Achilles tendon. The distal part of the Achilles tendon had a vascular density of 56.6 vessels/cm(2). In the middle part of the tendon, the vascular density was much lower: 28.2 vessels/cm(2). The proximal part of the tendon had a vascular density of 73.4 vessels/cm(2). Conclusion. The reduced vascularization in the tendon waist may be a predisposing factor for degeneration and spontaneous rupture of the human Achilles tendon.
C1 Univ Kiel, Dept Orthopaed Surg, D-24105 Kiel, Germany.
   Univ Kiel, Dept Anat, D-24098 Kiel, Germany.
RP Petersen, W (通讯作者)，Univ Kiel, Dept Orthopaed Surg, Michaelisstr 1, D-24105 Kiel, Germany.
CR Ahmed IM, 1998, J ORTHOPAED RES, V16, P591, DOI 10.1002/jor.1100160511
   ASTROM M, 1994, J ORTHOP RES, V12, P246, DOI 10.1002/jor.1100120214
   CARR AJ, 1989, J BONE JOINT SURG BR, V71, P100, DOI 10.1302/0301-620X.71B1.2914976
   GRAF J, 1990, Handchirurgie Mikrochirurgie Plastische Chirurgie, V22, P163
   HASTAD K, 1958, ACTA CHIR SCAND, V116, P251
   HOLZ J, 1980, FORTSCHR MED, V98, P1517
   Kannus P, 1997, SCAND J MED SCI SPOR, V7, P107
   KANNUS P, 1997, HUMAN TENDONS
   KOLTS I, 1994, ANN ANAT, V176, P75, DOI 10.1016/S0940-9602(11)80420-6
   LAGERGREN C, 1959, Acta Chir Scand, V116, P491
   Petersen W, 1999, ARCH ORTHOP TRAUM SU, V119, P371
   PLOTZ E, 1938, Z ORTHOP, V67, P212
   RUDERT M, 1993, ACTA ORTHOP SCAND, V64, P37, DOI 10.3109/17453679308994524
   SCHMIDTROHLFING B, 1992, INT ORTHOP, V16, P29
   SEEGESSER B, 1995, ORTHOPADE, V24, P252
   SPALTEHOLZ KW, 1914, DURCHSICHTIGMACHEN M
   Stein V, 2000, ACTA ORTHOP SCAND, V181, P313
   Thermann H, 1999, ORTHOPADE, V28, P82, DOI 10.1007/s001320050325
   TILLMANN B, 1991, ANAT ANZ S, V168, P23
   TIMPL R, 1979, J BIOL CHEM, V254, P9933
   WLADIMIROV B, 1971, ANAT ANZEIGER, V133, P12
   WOOD JONES F, 1944, FOOT
   ZWIPP H, 1989, UNFALLCHIRURG, V92, P554
NR 23
TC 46
Z9 49
U1 0
U2 3
PU SPRINGER-VERLAG
PI NEW YORK
PA 175 FIFTH AVE, NEW YORK, NY 10010 USA
SN 0936-8051
J9 ARCH ORTHOP TRAUM SU
JI Arch. Orthop. Trauma Surg.
PD NOV
PY 2003
VL 123
IS 9
BP 501
EP 504
DI 10.1007/s00402-003-0491-2
PG 4
WC Orthopedics; Surgery
WE Science Citation Index Expanded (SCI-EXPANDED)
SC Orthopedics; Surgery
GA 742WP
UT WOS:000186541300012
PM 12712360
DA 2022-07-22
ER

PT J
AU Latino, F
   D'Andria, A
   Tafuri, D
AF Latino, Francesca
   D'Andria, Antonio
   Tafuri, Domenico
TI BADMINTON PLAYER REATHLETIZATION AFTER ACHILLES TENDON RUPTURE
SO ACTA MEDICA MEDITERRANEA
LA English
DT Article
DE Training Program; Achilles Tendon rupture; Badminton
ID MUSCLE FUNCTION; MANAGEMENT
AB Introduction: The purpose of this study is to introduce an example of a criteria-driven progression through the return-to-sport phase of rehabilitation,following Achilles Tendon reconstruction.
   Case presentation: In this study the Authors present the case of a 28-years-old man, professional Badminton players, who had Achilles Tendon rupture during practice. So, the Authors describe recovery program necessary to get back to competitive sport.
   Conclusion: To ensure a successful return to sport following Achilles Tendon re-constructionis necessary to formulate an accurate training program. The training program should be personalised, gradual and editable.
C1 [Latino, Francesca; D'Andria, Antonio; Tafuri, Domenico] Parthenope Univ, Via Medina 40, Naples, Italy.
RP Tafuri, D (通讯作者)，Parthenope Univ Naples, Naples, Italy.
EM domenicotafuri@inwind.it
OI Latino, Francesca/0000-0003-0302-6145
CR Ascione A, 2018, ACTA MEDICA MEDITERR, V34, P1261, DOI 10.19193/0393-6384_2018_5_194
   Belfiore P, 2018, ACTA MEDICA MEDITERR, V34, P1257, DOI 10.19193/0393-6384_2018_5_193
   Carbonetto G, 2008, DA OLIMPIA A PECHINO
   Di Palma D, 2018, ACTA MEDICA MEDITERR, V34, P1253, DOI 10.19193/0393-6384_2018_5_192
   Doral MN, 2010, OPEN ACCESS J SPORTS, V1, P233, DOI 10.2147/OAJSM.S10670
   GILLIES H, 1970, J BONE JOINT SURG AM, VA 52, P337, DOI 10.2106/00004623-197052020-00015
   Gulati V, 2015, WORLD J ORTHOP, V6, P380, DOI 10.5312/wjo.v6.i4.380
   HAGGMARK T, 1986, ORTHOPEDICS, V9, P160
   Jafari A., 2014, International Journal of Basic Sciences and Applied Research, V3, P40
   Jafari A, 2014, INT J BASIC SCI APPL, V3, P40
   KENDAL FP, 2005, MUSCLES TESTING FUNC
   Lanzetta A, 1996, MALATTIE ORTOPEDICHE
   Leppilahti J, 1996, INT J SPORTS MED, V17, P619, DOI 10.1055/s-2007-972905
   LIPSCOMB P R, 1956, Minn Med, V39, P731
   Mahmudieh B, 2013, GLOBAL J SCI ENG TEC
   Martini F, 2012, HUMAN ANATOMY
   Moller M, 2002, SCAND J MED SCI SPOR, V12, P9, DOI 10.1034/j.1600-0838.2002.120103.x
   Myerson M S, 1999, Instr Course Lect, V48, P219
   Ooi CH, 2009, J SPORT SCI, V27, P1591, DOI 10.1080/02640410903352907
   Orio F, 2016, MINERVA ENDOCRINOL, V41, P509
   Palma D. di, 2018, Journal of Physical Education and Sport, V18, P473
   Palma D. di, 2017, Sport Science, V10, P36
   Palma D. di, 2017, Sport Science, V10, P18
   Palma D. di, 2016, Journal of Physical Education and Sport, V16, P785
   Palma D. di, 2016, Sport Science, V9, P24
   Perrone V, 2009, GIOT, V35, P129
   Raiola G, 2017, SPORT SCI, V10, P51
   Raiola G., 2016, SPORT SCI, V9, P49
   Tafuri D., 2017, SPORT SCI, V10, P70
   WREDMARK T, 1992, Scandinavian Journal of Medicine and Science in Sports, V2, P139
   Yung Patrick Shu-Hang, 2007, Res Sports Med, V15, P133, DOI 10.1080/15438620701405263
NR 31
TC 0
Z9 0
U1 1
U2 6
PU CARBONE EDITORE
PI PALERMO
PA VIA QUINTINO SELLA, 68, PALERMO, 90139, ITALY
SN 0393-6384
EI 2283-9720
J9 ACTA MEDICA MEDITERR
JI Acta Medica Mediterr.
PY 2018
VL 34
SI 3
BP 1493
EP 1497
DI 10.19193/0393-6384_2018_3s_227
PG 5
WC Medicine, General & Internal
WE Science Citation Index Expanded (SCI-EXPANDED)
SC General & Internal Medicine
GA GP7NG
UT WOS:000441088400004
DA 2022-07-22
ER

PT J
AU Li, PH
   Liu, SY
   Huang, JX
   Peng, J
   Lu, SB
   Guo, QY
AF Li, Penghao
   Liu, Shuyun
   Huang, Jingxiang
   Peng, Jiang
   Lu, Shibi
   Guo, Quanyi
TI In vitro preparation and characterization of dual biomimetic electrospun
   Wharton's Jelly-derived extra cellular matrix/polycaprolactone
   sub-micron fibrous band-aid for superior Achilles tendon recovery
SO INTERNATIONAL JOURNAL OF CLINICAL AND EXPERIMENTAL MEDICINE
LA English
DT Article
DE Achilles tendon rupture; Wharton's Jelly-derived extra cellular matrix;
   electrospinning; in vitro; ingredient and microstructure simulation
ID NANOFIBROUS MEMBRANES; SILVER NANOPARTICLES; ANTIADHESION; PREVENTION;
   ADHESION; RUPTURE; REHABILITATION; MATRIX; SHEATH; DIFFERENTIATION
AB Achilles tendon rupture (ATR) is a common sports injury, and complications usually occur. Promoting self-healing and suppressing the formation of scars and adhesions are priorities when treating ATR. The dual biomimetic electrospun Wharton's Jelly (WJ)-derived extra cellular matrix (ECM)/polycaprolactone (PCL) sub-micron fibrous band-aid was developed for this purpose. Each electrospun band-aid was prepared into two layers: the outer layer was made of ordered PCL fibers and the inner layer was made of ordered PCL and 0%, 25%, 50% or 75% WJ-derived ECM mixed fibers. The ingredients, microstructure, hydrophilicity, and cell adherence and survival were tested. Histological and immunohistochemical staining showed that WJ-derived ECM comprised type I collagen, and the cell nucleus was removed by a special decellularizing process. Fourier transform-infrared spectroscopy (FT-IR) verified the presence of protein in the electrospun band-aids. The electrospun PCL layer had high hydrophobicity and parallel, compact micron-size fibers that decreased viability of L929 cells and inhibited adherence. Electrospun PCL with different ratios of the WJ-derived ECM layer had good hydrophilicity and parallel, loose micron-size fibers. The ability to promote cell adherence and proliferation of the internal surface was 50% ECM > 0% ECM > 25% ECM > 75% ECM. Based on these results, the electrospun band-aid with 50% WJ-derived ECM might eliminate complications and result in superior recovery after ATR.
C1 [Li, Penghao; Liu, Shuyun; Huang, Jingxiang; Peng, Jiang; Lu, Shibi; Guo, Quanyi] Chinese Peoples Liberat Army Gen Hosp, Key Lab Musculoskeletal Trauma & War Injuries, Beijing Key Lab Regenerat Med Orthopaed, PLA,Inst Orthopaed, 28 Fuxing Rd, Beijing 100853, Peoples R China.
   [Li, Penghao] Nankai Univ, Coll Med, Tianjin, Peoples R China.
RP Guo, QY (通讯作者)，Chinese Peoples Liberat Army Gen Hosp, Key Lab Musculoskeletal Trauma & War Injuries, Beijing Key Lab Regenerat Med Orthopaed, PLA,Inst Orthopaed, 28 Fuxing Rd, Beijing 100853, Peoples R China.
EM doc-torguo_301@163.com
FU National Natural Science Foundation of China [21134004]; High Technology
   Research and Development Program of China [2012AA020502, 2015AA0-20303];
   Beijing Science and Technology Development [Z161100005016059]
FX This study was funded by the National Natural Science Foundation of
   China (21134004); High Technology Research and Development Program of
   China (2012AA020502, 2015AA0-20303), and Beijing Science and Technology
   Development (Z161100005016059). The authors are indebted to the pregnant
   women in the obstetrics department at Chinese PLA General Hospital for
   kindly providing the umbilical cords.
CR Badalihan A, 2015, J FOOT ANKLE SURG, V54, P153, DOI 10.1053/j.jfas.2014.12.002
   Chen CH, 2015, ACTA BIOMATER, V26, P225, DOI 10.1016/j.actbio.2015.07.041
   Chen CH, 2015, COLLOID SURFACE B, V133, P221, DOI 10.1016/j.colsurfb.2015.06.012
   Chen Q, 2014, INT J CLIN EXP PATHO, V7, P8462
   Chen SH, 2014, INT J NANOMED, V9, P4079, DOI 10.2147/IJN.S67931
   Chen S, 2014, INT J MOL SCI, V15, P14014, DOI 10.3390/ijms150814014
   Dankers PYW, 2011, BIOMATERIALS, V32, P723, DOI 10.1016/j.biomaterials.2010.09.020
   De la Fuente CI, 2016, J ATHL TRAINING, V51, P1013, DOI 10.4085/1062-6050-51.12.23
   Deepthi S, 2016, CARBOHYD POLYM, V153, P492, DOI 10.1016/j.carbpol.2016.07.124
   Franc S, 1998, PLACENTA, V19, P95, DOI 10.1016/S0143-4004(98)90104-7
   Groetelaers RPTGC, 2014, FOOT ANKLE INT, V35, P771, DOI 10.1177/1071100714536167
   Groetelaers RP, 2013, ARCH BONE JT SURG, V1, P86
   Guerquin MJ, 2013, J CLIN INVEST, V123, P3564, DOI 10.1172/JCI67521
   Inui A, 2010, INT ORTHOP, V34, P1327, DOI 10.1007/s00264-009-0917-8
   Ishiyama N, 2010, BIOMATERIALS, V31, P4009, DOI 10.1016/j.biomaterials.2010.01.100
   Jadalannagari S., 2017, PLOS ONE, V12, DOI DOI 10.1371/JOURNAL.PONE.0172098
   Jiang SC, 2015, INT J MOL SCI, V16, P6932, DOI 10.3390/ijms16046932
   Jiang SC, 2014, BIOMATERIALS, V35, P9920, DOI 10.1016/j.biomaterials.2014.08.028
   Jielile J, 2016, KNEE SURG SPORT TR A, V24, P2148, DOI 10.1007/s00167-015-3598-4
   Kauwe M, 2017, CLIN PODIATR MED SUR, V34, P229, DOI 10.1016/j.cpm.2016.10.009
   Kearney RS, 2012, PHYSIOTHERAPY, V98, P24, DOI 10.1016/j.physio.2011.04.349
   Liu S, 2013, MAT SCI ENG C-MATER, V33, P1176, DOI 10.1016/j.msec.2012.12.008
   Liu S, 2012, BIOMACROMOLECULES, V13, P3611, DOI 10.1021/bm301022p
   MacMahon A, 2016, FOOT ANKLE INT, V37, P636, DOI 10.1177/1071100716628915
   Maffulli N, 2015, INT ORTHOP, V39, P707, DOI 10.1007/s00264-015-2686-x
   Makulavicius A, 2016, FOLIA MORPHOL, V75, P53, DOI 10.5603/FM.a2015.0067
   Mardani-Kivi M, 2013, ARCH BONE JT SURG-AB, V1, P18
   Martinez C, 2017, J BIOMED MATER RES A, V105, P1034, DOI 10.1002/jbm.a.35976
   Metz R, 2007, BMC MUSCULOSKEL DIS, V8, DOI 10.1186/1471-2474-8-108
   Miyamoto W, 2017, FOOT ANKLE INT, V38, P167, DOI 10.1177/1071100716673589
   Qian YZ, 2016, INT J NANOMED, V11, P4157, DOI 10.2147/IJN.S110577
   Tosun HB, 2016, BALK MED J, V33, P258, DOI 10.5152/balkanmedj.2016.140172
   Uysal CA, 2012, J PLAST RECONSTR AES, V65, P1712, DOI 10.1016/j.bjps.2012.06.011
   Xia CS, 2012, ORTHOPEDICS, V35, pE1056, DOI 10.3928/01477447-20120621-21
   Yin HY, 2016, ACTA BIOMATER, V33, P96, DOI 10.1016/j.actbio.2016.01.024
   Yuan F, 2016, J BIOMED MATER RES A, V104, P1175, DOI 10.1002/jbm.a.35653
   Yuan ZG, 2016, BIOMATERIALS, V111, P13, DOI 10.1016/j.biomaterials.2016.09.017
   Zhang WX, 2016, TISSUE ENG PART B-RE, V22, P193, DOI [10.1089/ten.teb.2015.0290, 10.1089/ten.TEB.2015.0290]
   Zhao X, 2015, BIOMATERIALS, V61, P61, DOI 10.1016/j.biomaterials.2015.05.012
NR 39
TC 2
Z9 2
U1 2
U2 13
PU E-CENTURY PUBLISHING CORP
PI MADISON
PA 40 WHITE OAKS LN, MADISON, WI 53711 USA
SN 1940-5901
J9 INT J CLIN EXP MED
JI Int. J. Clin. Exp. Med.
PY 2017
VL 10
IS 8
BP 11563
EP 11573
PG 11
WC Medicine, Research & Experimental
WE Science Citation Index Expanded (SCI-EXPANDED)
SC Research & Experimental Medicine
GA FF1EX
UT WOS:000408643700027
DA 2022-07-22
ER

PT J
AU Belyea, CM
   Krul, KP
   Lause, G
   Magee, A
   Anderson, CD
   Ryan, PM
AF Belyea, Christopher M.
   Krul, Kevin P.
   Lause, Gregory
   Magee, Anthony
   Anderson, Claude D.
   Ryan, Paul M.
TI The Reliability of Kager's Triangle in Detecting Acute Achilles Tendon
   Ruptures
SO ORTHOPEDICS
LA English
DT Article
ID CLINICAL-PRACTICE GUIDELINE; ANATOMY
AB The Achilles tendon is a common site of anatomical injury among athletes and those participating in recreational sporting activities. Acute Achilles tendon ruptures are often misdiagnosed as a sprained ankle and are more common in the male population. Mechanism of injury is often a noncontact injury, resulting from sudden forced plantar flexion or violent dorsiflexion in a plantar flexed foot. Delays in diagnosis and treatment may complicate the clinical outcome. Because findings may be subtle on history and physical examination, the use of readily available adjunctive studies is important for practitioners. One method is the analysis of Kager's triangle on lateral ankle radiographs. Obscuration of Kager's triangle has been described as a radiographic indicator of Achilles tendon rupture, but the sensitivity and specificity of this finding have been poorly reported.
C1 [Belyea, Christopher M.; Krul, Kevin P.; Lause, Gregory; Magee, Anthony; Anderson, Claude D.; Ryan, Paul M.] Tripler Army Med Ctr, Dept Orthopaed Surg, 1 Jarrett White Rd, Honolulu, HI 96859 USA.
RP Belyea, CM (通讯作者)，Tripler Army Med Ctr, Dept Orthopaed Surg, 1 Jarrett White Rd, Honolulu, HI 96859 USA.
EM cmbelyea@gmail.com
CR Assal M, 2002, J ORTHOP TRAUMA, V16, P358, DOI 10.1097/00005131-200205000-00013
   CETTI R, 1993, CLIN ORTHOP RELAT R, P215
   Chiodo CP, 2010, J BONE JOINT SURG AM, V92A, P2466
   COMBALIA A, 1995, J ACCID EMERG MED, V12, P230
   Garras DN, 2012, CLIN ORTHOP RELAT R, V470, P2268, DOI 10.1007/s11999-012-2355-y
   Kou J, 2010, J AM ACAD ORTHOP SUR, V18, P511, DOI 10.5435/00124635-201008000-00008
   Ly JQ, 2004, AM J ROENTGENOL, V182, P147, DOI 10.2214/ajr.182.1.1820147
   Maffulli N, 1998, AM J SPORT MED, V26, P266, DOI 10.1177/03635465980260021801
   Pingel J, 2015, PLOS ONE, V10, DOI 10.1371/journal.pone.0127811
   Renninger LTCH, 2016, FOOT ANKLE INT, V37, P269, DOI 10.1177/1071100715615322
   Theobald P, 2006, J ANAT, V208, P91, DOI 10.1111/j.1469-7580.2006.00510.x
   Thevendran G, 2013, Musculoskelet Surg, V97, P9, DOI 10.1007/s12306-013-0251-6
   Tonarelli JM, 2011, J ORTHOP SPORT PHYS, V41, P904, DOI 10.2519/jospt.2011.0422
   Wiegerinck Johannes I, 2012, J Bone Joint Surg Am, V94, pe67, DOI 10.2106/JBJS.K.01074
NR 14
TC 0
Z9 0
U1 0
U2 1
PU SLACK INC
PI THOROFARE
PA 6900 GROVE RD, THOROFARE, NJ 08086 USA
SN 0147-7447
EI 1938-2367
J9 ORTHOPEDICS
JI Orthopedics
PD MAR-APR
PY 2020
VL 43
IS 2
BP E91
EP E94
DI 10.3928/01477447-20191212-05
PG 4
WC Orthopedics
WE Science Citation Index Expanded (SCI-EXPANDED)
SC Orthopedics
GA KW0WH
UT WOS:000520893400005
PM 31841605
DA 2022-07-22
ER

PT J
AU Neufeld, SK
   Farber, DC
AF Neufeld, Steven K.
   Farber, Daniel C.
TI Tendon Transfers in the Treatment of Achilles' Tendon Disorders
SO FOOT AND ANKLE CLINICS
LA English
DT Article
DE Tendon transfer; Achilles tendon rupture; Achilles tendinosis; V-Y
   advancement; Turndown; Flexor hallucis longus tendon
ID HALLUCIS LONGUS TRANSFER; MAGNETIC-RESONANCE; RELATIVE STRENGTHS;
   CHRONIC TEARS; TENDINOPATHY; RECONSTRUCTION; TENDINOSIS; RUPTURES;
   MUSCLES; REPAIR
AB The Achilles tendon is the strongest tendon in the human body and, as such, has its share of problems. Although many conditions affecting this tendon can be treated nonoperatively, surgical intervention is often necessary. Local, regional, distant, and allograft tendon can be used to supplement or enhance reconstruction or repair of the Achilles tendon. Specific techniques are explored and described, and the published results from the literature summarized. This article explores the use of tendon transfers and supplementation in the treatment of insertional and noninsertional Achilles tendinosis as well as in cases of neglected or chronic ruptures of the tendoachilles.
C1 [Neufeld, Steven K.] Orthopaed Foot & Ankle Ctr Washington, Falls Church, VA 22042 USA.
   [Neufeld, Steven K.] Virginia Commonwealth Univ, Dept Orthopaed Surg, Richmond, VA 23298 USA.
   [Farber, Daniel C.] Univ Maryland, Sch Med, Orthopaed & Rehabil Inst, Foot & Ankle Serv,Dept Orthopaed,Med Ctr, Baltimore, MD 21207 USA.
RP Farber, DC (通讯作者)，Univ Penn, Farm Journal Bldg,5th Floor,230 W Washington Sq, Philadelphia, PA 19106 USA.
EM dcfarber@gmail.com
RI Neufeld, Steven/AAM-5929-2020
CR ABRAHAM E, 1975, J BONE JOINT SURG AM, VA 57, P253, DOI 10.2106/00004623-197557020-00019
   Astrom M, 1996, SKELETAL RADIOL, V25, P615, DOI 10.1007/s002560050146
   Bruggeman NB, 2004, CLIN ORTHOP RELAT R, P63, DOI 10.1097/01.blo.0000144475.05543.e7
   Char JY, 2013, FOOT ANKLE INT, V34, P898
   Chen TM, 2009, CLIN ANAT, V22, P377, DOI 10.1002/ca.20758
   CLEMENT DB, 1984, AM J SPORT MED, V12, P179, DOI 10.1177/036354658401200301
   Cottom James M, 2008, Foot Ankle Spec, V1, P280, DOI 10.1177/1938640008322690
   Coull R, 2003, FOOT ANKLE INT, V24, P931, DOI 10.1177/107110070302401211
   Den Hartog BD, 2003, FOOT ANKLE INT, V24, P233, DOI 10.1177/107110070302400306
   Den Hartog BD, 2009, FOOT ANKLE CLIN, V14, P639, DOI 10.1016/j.fcl.2009.08.005
   DeOrio MJ, 2008, FOOT ANKLE INT, V29, P542, DOI 10.3113/FAI.2008.0542
   Elias I, 2009, FOOT ANKLE INT, V30, P197, DOI 10.3113/FAI.2009.0197
   Gurdezi S, 2013, FOOT ANKLE INT, V34, P1364, DOI 10.1177/1071100713488763
   Hahn F, 2008, FOOT ANKLE INT, V29, P794, DOI 10.3113/FAI.2008.0794
   Holmes GB, 2006, FOOT ANKLE INT, V27, P952, DOI 10.1177/107110070602701115
   Jeng CL, 2012, FOOT ANKLE INT, V33, P394, DOI 10.3113/FAI.2012.0394
   Jones, 1993, J Am Acad Orthop Surg, V1, P87
   Kiewiet NJ, 2013, FOOT ANKLE INT, V34, P481, DOI 10.1177/1071100713477620
   Maffulli N, 1997, AM J SPORT MED, V25, P835, DOI 10.1177/036354659702500618
   Maffulli N, 2004, AM J SPORT MED, V32, P174, DOI 10.1177/0363546503258923
   Maffulli N, 2011, J BONE JOINT SURG BR, V93B, P1503, DOI 10.1302/0301-620X.93B11.27379
   Maffulli N, 2012, J BONE JOINT SURG AM, V94A, P906, DOI 10.2106/JBJS.K.00869
   Maffulli N, 2012, J BONE JOINT SURG AM, V94A, P901, DOI 10.2106/JBJS.K.00200
   Martin RL, 2005, FOOT ANKLE INT, V26, P691, DOI 10.1177/107110070502600905
   Rahm S, 2013, FOOT ANKLE INT, V34, P1100, DOI 10.1177/1071100713487725
   Saxena A, 2000, FOOT ANKLE INT, V21, P570, DOI 10.1177/107110070002100707
   Schon LC, 2013, J BONE JOINT SURG AM, V95A, P54, DOI 10.2106/JBJS.K.00970
   Silbernagel KG, 2001, SCAND J MED SCI SPOR, V11, P197
   SILVER RL, 1985, J BONE JOINT SURG BR, V67, P432, DOI 10.1302/0301-620X.67B3.3997956
   Sorosky B, 2004, CLIN J SPORT MED, V14, P40, DOI 10.1097/00042752-200401000-00007
   Tashjian RZ, 2003, FOOT ANKLE INT, V24, P673, DOI 10.1177/107110070302400903
   Villarreal A D, 2012, Am J Orthop (Belle Mead NJ), V41, P126
   Waldecker Ute, 2012, Foot Ankle Surg, V18, P119, DOI 10.1016/j.fas.2011.04.007
   Wapner K L, 1993, Foot Ankle, V14, P443
   Will RE, 2009, FOOT ANKLE INT, V30, P315, DOI 10.3113/FAI.2009.0315
   Yeoman Thomas F M, 2012, Foot (Edinb), V22, P219, DOI 10.1016/j.foot.2012.05.004
NR 36
TC 15
Z9 17
U1 1
U2 5
PU W B SAUNDERS CO-ELSEVIER INC
PI PHILADELPHIA
PA 1600 JOHN F KENNEDY BOULEVARD, STE 1800, PHILADELPHIA, PA 19103-2899 USA
SN 1083-7515
EI 1558-1934
J9 FOOT ANKLE CLIN
JI Foot Ankle Clin.
PD MAR
PY 2014
VL 19
IS 1
BP 73
EP +
DI 10.1016/j.fcl.2013.10.005
PG 15
WC Orthopedics
WE Science Citation Index Expanded (SCI-EXPANDED)
SC Orthopedics
GA AC3MM
UT WOS:000332425900008
PM 24548511
DA 2022-07-22
ER

PT J
AU Kayser, R
   Mahlfeld, K
   Heyde, CE
AF Kayser, R
   Mahlfeld, K
   Heyde, CE
TI Partial rupture of the proximal Achilles tendon: a differential
   diagnostic problem in ultrasound imaging
SO BRITISH JOURNAL OF SPORTS MEDICINE
LA English
DT Article
ID MAGNETIC-RESONANCE; SONOGRAPHY; ULTRASONOGRAPHY; LESIONS
AB Objectives: The aim of this study was to determine whether ultrasound can correctly visualise partial ruptures of the proximal Achilles tendon.
   Method: This was a prospective study in which all chronic Achilles tendon injury patients seen at three centres in Germany from 1998 to 2003 were screened. All patients with clinical and/or sonographic signs of abnormalities in the region of the proximal third of the Achilles tendon and tendomuscular junction were included in the analysis. Each of these cases was evaluated by ultrasound following an assessment protocol. Patients with ambiguous ultrasound findings and/or clinical signs were additionally assessed by magnetic resonance imaging (MRI).
   Results: Sonomorphologic changes suggestive of an abnormality in the proximal third of the Achilles tendon were detected in 13 out of 320 patients (4.2%) with recurring Achilles tendon complaints. Thirteen patients had clinical signs but no sonographic changes in the tendon. The sonographic diagnosis was correct in 19 cases. In six of the 26 cases studied, MRI was needed to establish the correct diagnosis of partial intratendinous rupture of the proximal Achilles tendon. Sensitivity was 0.5, specificity was 0.81, and the overall agreement of the ultrasound examination was 61.5%. All patients were asymptomatic at follow up at a mean of 14 months ( range 12 - 17 months) after surgery.
   Conclusions: Ultrasound is a useful tool for evaluation of proximal Achilles tendon complaints. However, ultrasound is not sufficiently reliable for diagnosis of all pathologies, especially partial ruptures of the Achilles tendon. Thus, the definitive diagnosis must be established by MRI.
C1 Charite Univ Med Berlin, D-12200 Berlin, Germany.
   Univ Magdeburg, Orthoped Clin, D-39106 Magdeburg, Germany.
   Cent Hosp Bozen, Bolzano, Italy.
RP Kayser, R (通讯作者)，Charite Univ Med Berlin, Campus Benjamin Franklin,Hindenburgdamm 30, D-12200 Berlin, Germany.
EM ralph.kayser-berlin@t-online.de
RI Heyde, Christoph - E./AAV-9440-2021
CR BRANDSER EA, 1995, CAN ASSOC RADIOL J, V46, P9
   Gibbon WW, 1999, BRIT J SPORT MED, V33, P129, DOI 10.1136/bjsm.33.2.129
   Jacobson JA, 1999, RADIOL CLIN N AM, V37, P713, DOI 10.1016/S0033-8389(05)70125-1
   KAINBERGER F, 1992, ULTRASCHALL MED, V13, P28, DOI 10.1055/s-2007-1005271
   Kainberger F, 1996, RADIOLOGE, V36, P38, DOI 10.1007/s001170050037
   KALEBO P, 1992, AM J SPORT MED, V20, P378, DOI 10.1177/036354659202000402
   KALEBO P, 1990, ACTA RADIOL, V31, P565, DOI 10.3109/02841859009173098
   Khan KM, 2003, BRIT J SPORT MED, V37, P149, DOI 10.1136/bjsm.37.2.149
   LEHTINEN A, 1994, ANN CHIR GYNAECOL FE, V83, P322
   Mahlfeld K, 2001, ULTRASCHALL MED, V22, P87, DOI 10.1055/s-2001-12854
   Majewski M, 2003, UNFALLCHIRURG, V106, P556, DOI 10.1007/s00113-003-0623-8
   MERK H, 1989, ULTRASCHALL MED, V10, P192, DOI 10.1055/s-2007-1005989
   Movin T, 1998, FOOT ANKLE INT, V19, P311, DOI 10.1177/107110079801900508
   Movin T, 1998, ACTA RADIOL, V39, P126
   Nehrer S, 1997, ARCH ORTHOP TRAUM SU, V116, P14, DOI 10.1007/BF00434093
   Paavola M, 2000, AM J SPORT MED, V28, P634, DOI 10.1177/03635465000280050301
   REICHER MA, 1985, AM J ROENTGENOL, V145, P903, DOI 10.2214/ajr.145.5.903
   RUPP S, 1995, BRIT J RADIOL, V68, P454, DOI 10.1259/0007-1285-68-809-454
   SEGESSER B, 1995, ORTHOPADE, V24, P252
   Sell S, 1996, ARCH ORTHOP TRAUM SU, V115, P28, DOI 10.1007/BF00453213
   WILLIAMS JGP, 1986, SPORTS MED, V3, P114, DOI 10.2165/00007256-198603020-00003
NR 21
TC 47
Z9 49
U1 0
U2 1
PU B M J PUBLISHING GROUP
PI LONDON
PA BRITISH MED ASSOC HOUSE, TAVISTOCK SQUARE, LONDON WC1H 9JR, ENGLAND
SN 0306-3674
J9 BRIT J SPORT MED
JI Br. J. Sports Med.
PD NOV
PY 2005
VL 39
IS 11
BP 838
EP 842
DI 10.1136/bjsm.2005.018416
PG 5
WC Sport Sciences
WE Science Citation Index Expanded (SCI-EXPANDED)
SC Sport Sciences
GA 976HJ
UT WOS:000232723600016
PM 16244194
OA Bronze, Green Published
DA 2022-07-22
ER

PT J
AU Pedowitz, D
   Beck, D
AF Pedowitz, David
   Beck, David
TI Presentation, Diagnosis, and Nonsurgical Treatment Options of the
   Anterior Tibial Tendon, Posterior Tibial Tendon, Peroneals, and Achilles
SO FOOT AND ANKLE CLINICS
LA English
DT Article
DE Anterior tibial tendon; Achilles; Peroneals; Posterior tibial tendon
ID NONOPERATIVE TREATMENT; RUPTURE; ULTRASOUND; ANKLE; FRACTURE; MRI;
   CLASSIFICATION; TENDINOPATHY; MANAGEMENT; INJURIES
AB Disorders of the anterior tibial tendon (ATT) are rare, and relatively few series have been described in the literature. Ruptures of the ATT are more common than tendinopathies of the ATT. For those patients with a tendinopathy, initial treatment may include activity and shoe-wear modifications.
C1 [Pedowitz, David; Beck, David] Rothman Inst, 825 Old Lancaster Rd,Suite 202, Bryn Mawr, PA 19010 USA.
RP Pedowitz, D (通讯作者)，Rothman Inst, 825 Old Lancaster Rd,Suite 202, Bryn Mawr, PA 19010 USA.
EM drpedowitz@yahoo.com
CR Abousayed MM, 2016, CLIN ORTHOP RELAT R, V474, P588, DOI 10.1007/s11999-015-4581-6
   Al-Abbad H, 2013, FOOT ANKLE INT, V34, P33, DOI 10.1177/1071100712464354
   Anagnostakos K, 2006, FOOT ANKLE INT, V27, P330, DOI 10.1177/107110070602700504
   Augustin Jeffrey F, 2003, Foot Ankle Clin, V8, P491, DOI 10.1016/S1083-7515(03)00036-6
   Bare A A, 2001, Foot Ankle Clin, V6, P37
   Barfod KW, 2014, J BONE JOINT SURG AM, V96A, P1497, DOI 10.2106/JBJS.M.01273
   Baumhauer JF, 2004, CLIN SPORT MED, V23, P21, DOI 10.1016/S0278-5919(03)00088-7
   Beals T C, 1999, J Am Acad Orthop Surg, V7, P112
   Beischer AD, 2009, FOOT ANKLE INT, V30, P1053, DOI 10.3113/FAI.2009.1053
   Bernstein DT, 2016, ORTHOPEDICS, V39, pE970, DOI 10.3928/01477447-20160526-04
   Bianchi S, 2010, SEMIN MUSCULOSKEL R, V14, P292, DOI 10.1055/s-0030-1254519
   Bluman Eric M, 2007, Foot Ankle Clin, V12, P341, DOI 10.1016/j.fcl.2007.03.004
   Bohay Donald R, 2003, Foot Ankle Clin, V8, P619, DOI 10.1016/S1083-7515(03)00013-5
   BRADLEY JP, 1990, AM J SPORT MED, V18, P188, DOI 10.1177/036354659001800213
   Carroll M, 2015, SEMIN ARTHRITIS RHEU, V45, P103, DOI 10.1016/j.semarthrit.2015.03.001
   Chao W, 1996, FOOT ANKLE INT, V17, P736, DOI 10.1177/107110079601701204
   Claessen FMAP, 2014, SPORTS MED, V44, P1241, DOI 10.1007/s40279-014-0200-z
   Daftary A, 2009, ULTRASOUND Q, V25, P103, DOI 10.1097/RUQ.0b013e3181b738a6
   Dallaudiere B, 2014, J VASC INTERV RADIOL, V25, P717, DOI 10.1016/j.jvir.2014.01.026
   de Vos RJ, 2007, BRIT J SPORT MED, V41, DOI 10.1136/bjsm.2006.032532
   DeMill Shyler L, 2015, Foot Ankle Spec, V8, P360, DOI 10.1177/1938640015583308
   DiGiovanni BF, 2000, FOOT ANKLE INT, V21, P809, DOI 10.1177/107110070002101003
   Dombek Michael F, 2003, J Foot Ankle Surg, V42, P250, DOI 10.1016/S1067-2516(03)00314-4
   Feighan J, 1999, CLIN ORTHOP RELAT R, P23
   FORD LT, 1979, SOUTH MED J, V72, P827, DOI 10.1097/00007611-197907000-00019
   FUNK DA, 1986, J BONE JOINT SURG AM, V68A, P95, DOI 10.2106/00004623-198668010-00012
   Ganestam A, 2016, KNEE SURG SPORT TR A, V24, P3730, DOI 10.1007/s00167-015-3544-5
   Geideman WM, 2000, J ORTHOP SPORT PHYS, V30, P68, DOI 10.2519/jospt.2000.30.2.68
   Giza Eric, 2007, Foot Ankle Clin, V12, P251, DOI 10.1016/j.fcl.2007.03.008
   Grundy JRB, 2010, FOOT ANKLE INT, V31, P212, DOI 10.3113/FAI.2010.0212
   Gwynne-Jones D, 2009, FOOT ANKLE INT, V30, P758, DOI 10.3113/FAI.2009.0758
   Gwynne-Jones DP, 2011, FOOT ANKLE INT, V32, P337, DOI 10.3113/FAI.2011.0337
   Huttunen TT, 2014, AM J SPORT MED, V42, P2419, DOI 10.1177/0363546514540599
   Irwin TA, 2010, FOOT ANKLE INT, V31, P933, DOI 10.3113/FAI.2010.0933
   Jarvinen Tero A H, 2005, Foot Ankle Clin, V10, P255, DOI 10.1016/j.fcl.2005.01.013
   Jerome J Terrence Jose, 2008, Foot Ankle Surg, V14, P166, DOI 10.1016/j.fas.2007.12.001
   KIER R, 1991, J COMPUT ASSIST TOMO, V15, P477, DOI 10.1097/00004728-199105000-00026
   Lee SJ, 2013, SKELETAL RADIOL, V42, P1191, DOI 10.1007/s00256-013-1631-6
   Longo UG, 2013, FOOT ANKLE CLIN, V18, P319, DOI 10.1016/j.fcl.2013.02.009
   Longo UG, 2009, SPORTS MED ARTHROSC, V17, P112, DOI 10.1097/JSA.0b013e3181a3d625
   Markarian GG, 1998, FOOT ANKLE INT, V19, P792, DOI 10.1177/107110079801901202
   Martinelli N, 2014, J AM PODIAT MED ASSN, V104, P298, DOI 10.7547/0003-0538-104.3.298
   McLauchlan G, 2011, COCHRANE DB SYST REV, DOI 10.1002/14651858.CD000232.pub2
   MCLENNAN JG, 1980, AM J SPORT MED, V8, P432, DOI 10.1177/036354658000800609
   Mechrefe AP, 2006, FOOT ANKLE INT, V27, P645, DOI 10.1177/107110070602700816
   Meehan Robert E, 2003, Foot Ankle Clin, V8, P431, DOI 10.1016/S1083-7515(03)00019-6
   Mengiardi B, 2005, RADIOLOGY, V235, P977, DOI 10.1148/radiol.2353040617
   MIRZA MA, 1984, ORTHOPEDICS, V7, P1329, DOI 10.3928/0147-7447-19840801-16
   Nielsen MD, 2011, J FOOT ANKLE SURG, V50, P311, DOI 10.1053/j.jfas.2011.02.002
   Noll K H, 2001, Foot Ankle Clin, V6, P25, DOI 10.1016/S1083-7515(03)00077-9
   OUZOUNIAN TJ, 1995, FOOT ANKLE INT, V16, P406, DOI 10.1177/107110079501600705
   Paavola M, 1998, ACTA RADIOL, V39, P612, DOI 10.3109/02841859809175485
   Panageas E, 1990, Orthop Rev, V19, P975
   Patten A, 2000, FOOT ANKLE INT, V21, P697, DOI 10.1177/107110070002100814
   Peduto Anthony J, 2010, Top Magn Reson Imaging, V21, P25, DOI 10.1097/RMR.0b013e31820ef505
   Peetrons P, 1999, JBR-BTR, V82, P157
   Pesquer L, 2016, BRIT J RADIOL, V89, DOI 10.1259/bjr.20150958
   Philbin TM, 2009, J AM ACAD ORTHOP SUR, V17, P306, DOI 10.5435/00124635-200905000-00005
   Pierre-Jerome C, 2010, ACTA RADIOL, V51, P438, DOI 10.3109/02841851003627809
   Premkumar A, 2002, AM J ROENTGENOL, V178, P223, DOI 10.2214/ajr.178.1.1780223
   Raikin SM, 2012, FOOT ANKLE CLIN, V17, P169, DOI 10.1016/j.fcl.2012.03.002
   Renninger LTCH, 2016, FOOT ANKLE INT, V37, P269, DOI 10.1177/1071100715615322
   Roster B, 2015, CLIN SPORT MED, V34, P625, DOI 10.1016/j.csm.2015.06.003
   SCHWEITZER ME, 1994, SEMIN ULTRASOUND CT, V15, P410, DOI 10.1016/S0887-2171(05)80007-1
   Sensiba PR, 2010, FOOT ANKLE INT, V31, P141, DOI 10.3113/FAI.2010.0141
   Wapner KL, 1999, CLIN ORTHOP RELAT R, P39, DOI 10.1097/00003086-199908000-00005
   Weinfeld SB, 2014, MED CLIN N AM, V98, P331, DOI 10.1016/j.mcna.2013.11.005
   Willits K, 2010, J BONE JOINT SURG AM, V92A, P2767, DOI 10.2106/JBJS.I.01401
   Younger AS, 2005, FOOT ANKLE INT, V26, P820, DOI 10.1177/107110070502601006
NR 69
TC 3
Z9 3
U1 1
U2 8
PU W B SAUNDERS CO-ELSEVIER INC
PI PHILADELPHIA
PA 1600 JOHN F KENNEDY BOULEVARD, STE 1800, PHILADELPHIA, PA 19103-2899 USA
SN 1083-7515
EI 1558-1934
J9 FOOT ANKLE CLIN
JI Foot Ankle Clin.
PD DEC
PY 2017
VL 22
IS 4
BP 677
EP +
DI 10.1016/j.fcl.2017.07.012
PG 12
WC Orthopedics
WE Science Citation Index Expanded (SCI-EXPANDED)
SC Orthopedics
GA FN7MO
UT WOS:000416202500004
PM 29078822
DA 2022-07-22
ER

PT J
AU Richardson, LC
   Reitman, R
   Wilson, M
AF Richardson, LC
   Reitman, R
   Wilson, M
TI Achilles tendon ruptures: Functional outcome of surgical repair with a
   "pull-out" wire
SO FOOT & ANKLE INTERNATIONAL
LA English
DT Article
DE Achilles tendon; Achilles ruptures
ID NONOPERATIVE TREATMENT
AB This is a retrospective review of 30 consecutive patients with acute Achilles tendon ruptures treated surgically by a single surgeon between 1992 and 1999. Repair was effected with a 26-gauge "pull-out" wire technique. A compressive dressing reinforced with plaster was employed to immobilize the ankle in neutral for 6 weeks. The hardware was removed 6 weeks postoperatively under local anesthesia. Intensive physical therapy was employed until ankle motion was symmetric and single heel raise was possible. Average follow-up was 4 years (range, 1-8 years). Nearly all patients (29/30) report satisfaction with their outcome. Most patients (29/30) had AOFAS Ankle-Hindfoot Scores greater than 90. Twenty-three (77%) returned to preoperative levels of athletic activities. Isometric testing documented 81% plantarflexion strength compared to the normal ankle at a mean of 32 months postoperatively. Five of eight women reported shoe wear difficulties related to the incision. There were no reruptures or deep infections. The "pull-out" wire technique is recommended for patients who wish to return to an active lifestyle. This method provides rigid approximation of tendon length, minimizes strangulation of tissue, repairs large gaps, and avoids late foreign-body reactions.
C1 Faulkner Hosp, Brigham Foot & Ankle Ctr, Jamaica Plain, MA 02150 USA.
RP Wilson, M (通讯作者)，Faulkner Hosp, Brigham Foot & Ankle Ctr, 1153 Ctr St, Jamaica Plain, MA 02150 USA.
CR BRADLEY JP, 1990, AM J SPORT MED, V18, P188, DOI 10.1177/036354659001800213
   Bunnell S., 1940, AM J SURG, DOI [10.1016/S0002- 9610(40)90160- X, DOI 10.1016/S0002-9610(40)90160-X]
   CETTI R, 1993, AM J SPORT MED, V21, P791, DOI 10.1177/036354659302100606
   INGLIS AE, 1976, J BONE JOINT SURG AM, V58, P990, DOI 10.2106/00004623-197658070-00015
   KELLAM JF, 1985, CLIN ORTHOP RELAT R, V201, P80
   KITAOKA HB, 1994, FOOT ANKLE INT, V15, P349, DOI 10.1177/107110079401500701
   KLEIN W, 1991, Chirurgia degli Organi di Movimento, V76, P223
   LEA RB, 1972, J BONE JOINT SURG AM, VA 54, P1398, DOI 10.2106/00004623-197254070-00002
   LEPPILAHTI J, 1998, CLIN ORTHOP RELAT R, V346, P152
   Lo IKY, 1997, CLIN J SPORT MED, V7, P207, DOI 10.1097/00042752-199707000-00010
   MA GWC, 1977, CLIN ORTHOP RELAT R, P247
   PERSSON A, 1979, INT ORTHOP, V3, P149
   QUIGLEY TB, 1980, AM J SPORT MED, V8, P244, DOI 10.1177/036354658000800406
   Soldatis JJ, 1997, AM J SPORT MED, V25, P90, DOI 10.1177/036354659702500118
   SOMA CA, 1995, ORTHOP CLIN N AM, V26, P239
   THOMPSON T C, 1962, J Trauma, V2, P126, DOI 10.1097/00005373-196203000-00003
NR 16
TC 13
Z9 15
U1 1
U2 1
PU SAGE PUBLICATIONS INC
PI THOUSAND OAKS
PA 2455 TELLER RD, THOUSAND OAKS, CA 91320 USA
SN 1071-1007
EI 1944-7876
J9 FOOT ANKLE INT
JI Foot Ankle Int.
PD MAY
PY 2003
VL 24
IS 5
BP 439
EP 443
DI 10.1177/107110070302400512
PG 5
WC Orthopedics
WE Science Citation Index Expanded (SCI-EXPANDED)
SC Orthopedics
GA 682WN
UT WOS:000183115500012
PM 12801203
DA 2022-07-22
ER

PT J
AU Tan, S
   Kudas, S
   Ozcan, AS
   Ipek, A
   Karaoglanoglu, M
   Arslan, H
   Bozkurt, M
AF Tan, Sinan
   Kudas, Savas
   Ozcan, Aysenur Sirin
   Ipek, Ali
   Karaoglanoglu, Mustafa
   Arslan, Halil
   Bozkurt, Murat
TI Real-time sonoelastography of the Achilles tendon: pattern description
   in healthy subjects and patients with surgically repaired complete
   ruptures
SO SKELETAL RADIOLOGY
LA English
DT Article
DE Achilles tendon rupture; Elastography; Sonoelastography; Sports medicine
ID ULTRASONOGRAPHY; SONOGRAPHY; ULTRASOUND
AB The purpose of this study is to describe the elastographic appearance of the Achilles tendon in healthy subjects and patients with surgically repaired complete ruptures.
   Nineteen Achilles tendons of 16 amateur footballers with surgically repaired complete ruptures and their contralateral asymptomatic Achilles tendons were assessed with ultrasound and real-time sonoelastography. Additionally, 40 asymptomatic Achilles tendons of 20 healthy amateur footballers were assessed. The Achilles tendons were divided into the distal, middle, and proximal thirds for elastographic image evaluation. Tendons were classified into three main types according to the elasticity features: type 1, blue (hardest tissue); type 2, blue/green (hard tissue); or type 3, green (intermediate tissue). In addition, three subtypes were determined: homogeneous, relatively homogeneous, and heterogeneous.
   Most of the Achilles tendons of the patients with surgically repaired complete ruptures were detected to have type 2 elasticity (64.9%), and the remaining had type 1 (35.1%). In contrast, most of the healthy tendons had type 2 (64.2%), and the remaining had either a type 3 (20.8%) or a type 1 (15%) elastographic pattern. All of the ruptured tendons had a heterogeneous structure, whereas all of the healthy Achilles tendons had a homogeneous or relatively homogeneous structure.
   In sonoelastography, the recognition of normal tendon structure will be useful in assessing pathologies of the Achilles tendon. Additionally, in patients with excellent American Orthopedic Foot and Ankle Society (AOFAS) scores and surgically repaired complete ruptures, a hard and heterogeneous pattern of tendon structure may be a natural consequence of tendon healing.
C1 [Tan, Sinan; Ozcan, Aysenur Sirin; Ipek, Ali; Karaoglanoglu, Mustafa; Arslan, Halil] Ankara Ataturk Educ & Res Hosp, Dept Radiol, TR-06800 Ankara, Turkey.
   [Kudas, Savas] Ankara Ataturk Educ & Res Hosp, Dept Sports Med, TR-06800 Ankara, Turkey.
   [Bozkurt, Murat] Ankara Ataturk Educ & Res Hosp, Dept Orthopaed, TR-06800 Ankara, Turkey.
RP Tan, S (通讯作者)，Ankara Ataturk Educ & Res Hosp, Dept Radiol, Eskisehir Yolu 8,Km 3 Bilkent, TR-06800 Ankara, Turkey.
EM drsinantan@gmail.com; savaskudas@hotmail.com;
   aysenursirinozcan@gmail.com; aliipek1112@gmail.com;
   mustafakaraoglanoglu@hotmail.com; drhalilarslan@hotmail.com;
   nmbozkurt@yahoo.com
RI Arslan, Halil/AAC-5244-2020; Şirin Özcan, Ayşe Nur/AAY-8110-2021
OI Arslan, Halil/0000-0002-6552-1275; Şirin Özcan, Ayşe
   Nur/0000-0003-0019-8515
CR Allison SJ, 2010, AM J ROENTGENOL, V194, pW514, DOI 10.2214/AJR.09.4067
   Archambault JM, 1998, J CLIN ULTRASOUND, V26, P335, DOI 10.1002/(SICI)1097-0096(199809)26:7<335::AID-JCU1>3.0.CO;2-A
   Astrom M, 1996, SKELETAL RADIOL, V25, P615, DOI 10.1007/s002560050146
   Babic J, 2004, EUR J APPL PHYSIOL, V92, P477, DOI 10.1007/s00421-004-1107-4
   Botar-Jid C, 2010, MED ULTRASON, V12, P120
   De Zordo T, 2010, ULTRASCHALL MED, V31, P394, DOI 10.1055/s-0028-1109809
   De Zordo T, 2009, AM J ROENTGENOL, V193, pW134, DOI 10.2214/AJR.08.1843
   De Zordo T, 2009, AM J ROENTGENOL, V193, P180, DOI 10.2214/AJR.08.2020
   Dong Qian, 2009, AJR Am J Roentgenol, V193, pW173, DOI 10.2214/AJR.09.3111
   Doral MN, 2010, KNEE SURG SPORT TR A, V18, P638, DOI 10.1007/s00167-010-1083-7
   Doral MN, 2009, ARCH ORTHOP TRAUM SU, V129, P1093, DOI 10.1007/s00402-009-0880-2
   Drakonaki EE, 2009, CLIN RADIOL, V64, P1196, DOI 10.1016/j.crad.2009.08.006
   Holmes GB, 2006, FOOT ANKLE INT, V27, P952, DOI 10.1177/107110070602701115
   Jamadar DA, 2002, AM J ROENTGENOL, V179, DOI 10.2214/ajr.179.3.1790709
   Karjalainen PT, 1996, ACTA RADIOL, V37, P639
   KITAOKA HB, 1994, FOOT ANKLE INT, V15, P349, DOI 10.1177/107110079401500701
   Klauser AS, 2010, SKELETAL RADIOL, V39, P1061, DOI 10.1007/s00256-009-0782-y
   Klauser AS, 2010, SEMIN MUSCULOSKEL R, V14, P323, DOI 10.1055/s-0030-1254521
   LAINE HR, 1987, J ULTRAS MED, V6, P351, DOI 10.7863/jum.1987.6.7.351
   LEADBETTER WB, 1992, CLIN SPORT MED, V11, P533
   Li YF, 2011, SKELETAL RADIOL, V40, P389, DOI 10.1007/s00256-010-0918-0
   LIU SH, 1995, CLIN ORTHOP RELAT R, P265
   Maffulli N, 2000, AM J SPORT MED, V28, P499, DOI 10.1177/03635465000280040901
   Pang BSF, 2006, J ULTRAS MED, V25, P1291, DOI 10.7863/jum.2006.25.10.1291
   Richards P, 2005, SKELETAL RADIOL, V34, P336, DOI 10.1007/s00256-004-0834-2
   Sconfienza LM, 2010, CLIN EXP RHEUMATOL, V28, P373
   Wu CH, 2011, RADIOLOGY, V259, P502, DOI 10.1148/radiol.11101665
NR 27
TC 69
Z9 71
U1 1
U2 14
PU SPRINGER
PI NEW YORK
PA 233 SPRING ST, NEW YORK, NY 10013 USA
SN 0364-2348
EI 1432-2161
J9 SKELETAL RADIOL
JI Skeletal Radiol.
PD SEP
PY 2012
VL 41
IS 9
BP 1067
EP 1072
DI 10.1007/s00256-011-1339-4
PG 6
WC Orthopedics; Radiology, Nuclear Medicine & Medical Imaging
WE Science Citation Index Expanded (SCI-EXPANDED)
SC Orthopedics; Radiology, Nuclear Medicine & Medical Imaging
GA 991SB
UT WOS:000307720700006
PM 22167230
DA 2022-07-22
ER

PT J
AU Hansen, MS
   Helander, KN
   Karlsson, J
   Barfod, KW
AF Hansen, Maria Swennergren
   Helander, Katarina Nilsson
   Karlsson, Jon
   Barfod, Kristoffer Weisskirchner
TI Performance of the Achilles Tendon Total Rupture Score Over Time in a
   Large National Database: Development of an Instruction Manual for
   Accurate Use
SO AMERICAN JOURNAL OF SPORTS MEDICINE
LA English
DT Article
DE Achilles tendon rupture; Achilles tendon Total Rupture Score
ID CROSS-CULTURAL ADAPTATION; ACCELERATED REHABILITATION; SURGICAL REPAIR;
   VALIDATION; RELIABILITY; VERSION; PROTOCOL
AB Background:
   The Achilles tendon Total Rupture Score (ATRS) is a commonly used patient-reported outcome measure for patients with an acute Achilles tendon rupture. The score consists of 10 questions, the last 3 of which include activities that some patients cannot or do not do. No instruction manual has been developed for the ATRS.
   Hypothesis/Purpose:
   The purpose was to evaluate the ATRS at 4, 12, and 24 months after a rupture. The hypothesis was that the results at 4 months would be inconsistent when compared with the results at 1 year and 2 years. We also aimed to develop a manual that explains how to use the ATRS.
   Study Design:
   Cohort study (Diagnosis); Level of evidence, 3.
   Methods:
   This was a mixed-methods study. The first section was a registry study, where prospectively collected data were analyzed. Data were collected 4, 12, and 24 months after rupture. The original score based on 10 items was compared with a score based on the first 7 items adjusted to the same scale as the original score. Density plots and scatterplots were made and differences between the scores were tested using the Mann-Whitney U test. The second section of the study consisted of discussions among the authors, which resulted in a manual for the ATRS.
   Results:
   In total, 2790 complete ATRSs were included. The 7-item score significantly overestimated the value of the 10-item score at all time points (P < .001), but only at 4 months was the difference clinically relevant (9.7 points).
   Conclusion:
   When the ATRS is used for short-term evaluation, there is a risk of results being inconsistent because of the last 3 questions. A manual explaining how to use the ATRS was therefore developed. We recommend that the full ATRS together with the manual should be used in future research rather than eliminating the 3 last questions.
C1 [Hansen, Maria Swennergren; Helander, Katarina Nilsson; Karlsson, Jon; Barfod, Kristoffer Weisskirchner] Copenhagen Univ Hosp Amager Hvidovre, Hvidovre, Denmark.
   [Hansen, Maria Swennergren] Copenhagen Univ Hosp Amager Hvidovre, Dept Phys & Occupat Therapy, Phys Med & Rehabil Res Copenhagen PMR C, Kettegard Alle 30, DK-2650 Hvidovre, Denmark.
   [Hansen, Maria Swennergren; Barfod, Kristoffer Weisskirchner] Copenhagen Univ Hosp Amager Hvidovre, Dept Orthoped Surg, SORC C, Hvidovre, Denmark.
   [Helander, Katarina Nilsson; Karlsson, Jon] Sahlgrens Univ Hosp, Dept Orthopaed, Gothenburg, Sweden.
RP Hansen, MS (通讯作者)，Copenhagen Univ Hosp Amager Hvidovre, Dept Phys & Occupat Therapy, Phys Med & Rehabil Res Copenhagen PMR C, Kettegard Alle 30, DK-2650 Hvidovre, Denmark.
EM maria_swennergren@hotmail.com
RI Barfod, Kristoffer/AAY-3288-2021
OI Barfod, Kristoffer/0000-0003-2620-5891
CR Ansari NN, 2016, KNEE SURG SPORT TR A, V24, P1372, DOI 10.1007/s00167-015-3977-x
   Bakowski P, 2018, KNEE SURG SPORT TR A, V26, P2074, DOI 10.1007/s00167-017-4764-7
   Barfod KW, 2016, TRIALS, V17, DOI 10.1186/s13063-016-1697-2
   Barfod KW, 2014, J BONE JOINT SURG AM, V96A, P1497, DOI 10.2106/JBJS.M.01273
   Brorsson A, 2016, SCAND J MED SCI SPOR, V26, P844, DOI 10.1111/sms.12533
   Buckinx F, 2020, FOOT ANKLE SURG, V26, P662, DOI 10.1016/j.fas.2019.08.010
   Cui J, 2017, HEALTH QUAL LIFE OUT, V15, DOI 10.1186/s12955-016-0574-8
   Elo S, 2008, J ADV NURS, V62, P107, DOI 10.1111/j.1365-2648.2007.04569.x
   Ganestam A, 2013, J FOOT ANKLE SURG, V52, P736, DOI 10.1053/j.jfas.2013.07.004
   Hansen MS, 2016, KNEE SURG SPORT TR A, V24, P1365, DOI 10.1007/s00167-015-3974-0
   Hardon A, 2001, APPL HLTH RES MANUAL
   Kearney RS, 2012, BRIT J SPORT MED, V46, P1102, DOI 10.1136/bjsports-2011-090497
   Mutlu EK, 2015, KNEE SURG SPORT TR A, V23, P2427, DOI 10.1007/s00167-014-3042-1
   Myhrvold SB, 2018, KNEE SURG SPORT TR A, V26, P2045, DOI 10.1007/s00167-017-4689-1
   Nilsson-Helander K, 2007, AM J SPORT MED, V35, P421, DOI 10.1177/0363546506294856
   Ochen Y, 2019, BMJ-BRIT MED J, V364, DOI 10.1136/bmj.k5120
   Olsson N, 2014, SCAND J MED SCI SPOR, V24, P152, DOI 10.1111/j.1600-0838.2012.01497.x
   Olsson N, 2014, AM J SPORT MED, V42, P1448, DOI 10.1177/0363546514527409
   Olsson N, 2013, AM J SPORT MED, V41, P2867, DOI 10.1177/0363546513503282
   Opdam KTM, 2018, KNEE SURG SPORT TR A, V26, P862, DOI 10.1007/s00167-016-4242-7
   Porter MD, 2015, ANZ J SURG, V85, P373, DOI 10.1111/ans.12910
   Svedman S, 2018, AM J SPORT MED, V46, P2929, DOI 10.1177/0363546518793655
   Touzopoulos P, 2019, FOOT ANKLE SURG, V25, P8, DOI 10.1016/j.fas.2017.07.560
   Vascellari A, 2018, KNEE SURG SPORT TR A, V26, P854, DOI 10.1007/s00167-016-4152-8
   Zambelli R, 2016, BMC SPORTS SCI MED R, V8, DOI 10.1186/s13102-016-0034-0
NR 25
TC 4
Z9 5
U1 0
U2 1
PU SAGE PUBLICATIONS INC
PI THOUSAND OAKS
PA 2455 TELLER RD, THOUSAND OAKS, CA 91320 USA
SN 0363-5465
EI 1552-3365
J9 AM J SPORT MED
JI Am. J. Sports Med.
PD MAY
PY 2020
VL 48
IS 6
BP 1423
EP 1429
AR 0363546520912222
DI 10.1177/0363546520912222
EA APR 2020
PG 7
WC Orthopedics; Sport Sciences
WE Science Citation Index Expanded (SCI-EXPANDED)
SC Orthopedics; Sport Sciences
GA LM7BR
UT WOS:000527139500001
PM 32255669
DA 2022-07-22
ER

PT J
AU Lee, JK
   Kang, C
   Hwang, DS
   Kang, DH
   Lee, GS
   Hwang, JM
   Song, JH
   Lee, CW
AF Lee, Jeong-Kil
   Kang, Chan
   Hwang, Deuk-Soo
   Kang, Dong-Hun
   Lee, Gi-Soo
   Hwang, Jung-Mo
   Song, Jae-Hwang
   Lee, Cheol-Won
TI A comparative study of innovative percutaneous repair and open repair
   for acute Achilles tendon rupture: Innovative usage of intraoperative
   ultrasonography
SO JOURNAL OF ORTHOPAEDIC SURGERY
LA English
DT Article
DE absorbable suture; Achilles tendon; percutaneous repair; sural nerve;
   ultrasonography
ID SUTURE; COMPLICATIONS; METAANALYSIS; OUTCOMES; VICRYL
AB Purpose:
   There is no definitive consensus on the optimal treatment of Achilles tendon rupture. We comparatively analyzed the clinical outcomes of two types of repair surgeries in treating Achilles tendon rupture.
   Methods:
   This retrospective study included 12 patients of Achilles tendon rupture (group A) treated with ultrasound-guided percutaneous repair and 18 patients (group B) treated with open repair. Clinical evaluation was performed using the Arner-Lindholm scale, American Orthopedic Foot and Ankle Society (AOFAS) ankle-hindfoot score, Achilles Tendon Total Rupture score (ATRS), visual analog scale, time to single heel raise, bilateral calf circumferences, recovery of athletic ability, and other complications.
   Results:
   While the Arner-Lindholm scale, AOFAS ankle-hindfoot score, ATRS, time point when single heel raise was possible, differences in bilateral calf circumference, and recovery of athletic ability compared to pre-rupture level were not significantly different between the two groups (p = 0.999, 0.235, 0.357, 0.645, 0.497, and 0.881, respectively), overall and aesthetic satisfaction levels were higher in the group treated with percutaneous repair under ultrasonography guidance (p = 0.035 and 0.001, respectively). Overall, there were no cases involving sural nerve injury in either group.
   Conclusion:
   Innovative percutaneous repair provides not only similar clinical outcomes but also greater overall and aesthetic satisfaction levels of operative outcomes and minimal complications (i.e. sural nerve injury) compared to open repair surgeries. Therefore, percutaneous repair may be a useful technique in the treatment of Achilles tendon rupture.
C1 [Lee, Jeong-Kil; Kang, Chan; Hwang, Deuk-Soo; Lee, Gi-Soo; Hwang, Jung-Mo; Lee, Cheol-Won] Chungnam Natl Univ, Dept Orthpaed Surg, Sch Med, 282 Munwha Ro, Daejeon 35015, South Korea.
   [Kang, Dong-Hun] Daejeon Centum Hosp, Dept Orthpaed Surg, Daejeon, South Korea.
   [Song, Jae-Hwang] Konyang Univ Hosp, Dept Orthpaed Surg, Daejeon, South Korea.
RP Kang, C (通讯作者)，Chungnam Natl Univ, Dept Orthpaed Surg, Sch Med, 282 Munwha Ro, Daejeon 35015, South Korea.
EM faschan@daum.net
RI Song, Jaehwang/AAX-9140-2021
OI Song, Jaehwang/0000-0001-5192-4965; HWANG, Jungmo/0000-0002-0548-8485
FU Research Fund of Chungnam National University in Daejeon, Korea
FX The author(s) disclosed receipt of the following financial support for
   the research, authorship, and/or publication of this article: This work
   was supported by Research Fund of Chungnam National University in
   Daejeon, Korea.
CR Ahmad S, 2014, J GASTROINTEST SURG, V18, P15
   ARNER O, 1959, Acta Chir Scand Suppl, V116, P1
   Ashammakhi N., 2004, EUR J PLAST SURG, V26, P383
   Assal M, 2002, J BONE JOINT SURG AM, V84A, P161, DOI 10.2106/00004623-200202000-00001
   Baig M N, 2017, Ir Med J, V110, P566
   Blankstein A, 2007, ISR MED ASSOC J, V9, P83
   BOURNE RB, 1988, CAN J SURG, V31, P43
   Clanton TO, 2015, AM J SPORT MED, V43, P1957, DOI 10.1177/0363546515587082
   Giannetti S, 2014, ORTHOPEDICS, V37, P820, DOI 10.3928/01477447-20141124-04
   Gigante A, 2008, KNEE SURG SPORT TR A, V16, P204, DOI 10.1007/s00167-007-0448-z
   Hsu AR, 2015, FOOT ANKLE INT, V36, P1279, DOI 10.1177/1071100715589632
   Jacobs D, 1978, Am J Sports Med, V6, P107, DOI 10.1177/036354657800600302
   Khan RJK, 2005, J BONE JOINT SURG AM, V87A, P2202, DOI 10.2106/JBJS.D.03049
```

Kim Jung-Han, 2017, [Journal of the Korean Orthopaedic Association, 대한정형외과학회지], V52, P249, DOI 10.4055/jkoa.2017.52.3.249

```
   KITAOKA HB, 1994, FOOT ANKLE INT, V15, P349, DOI 10.1177/107110079401500701
   KLEIN W, 1991, Chirurgia degli Organi di Movimento, V76, P223
   Kocaoglu B, 2015, FOOT ANKLE INT, V36, P691, DOI 10.1177/1071100714568868
   Lacoste S, 2014, ORTHOP TRAUMATOL-SUR, V100, P925, DOI 10.1016/j.otsr.2014.09.018
   MA GWC, 1977, CLIN ORTHOP RELAT R, P247
   McCoy BW, 2010, FOOT ANKLE INT, V31, P701, DOI 10.3113/FAI.2010.0701
   Nilsson-Helander K, 2007, AM J SPORT MED, V35, P421, DOI 10.1177/0363546506294856
   Porter KJ, 2014, FOOT ANKLE SURG, V20, P90, DOI 10.1016/j.fas.2013.11.005
   Singer AJ, 1998, ACAD EMERG MED, V5, P1007, DOI 10.1111/j.1553-2712.1998.tb02781.x
   Soroceanu A, 2012, J BONE JOINT SURG AM, V94A, P2136, DOI 10.2106/JBJS.K.00917
   Soubeyrand M, 2010, FOOT ANKLE INT, V31, P1069, DOI 10.3113/FAI.2010.1069
   Sutherland A., 1999, ORTHOP TRAUMATOL, V7, P288, DOI [10.1007/BF03180949, DOI 10.1007/BF03180949]
   Wilkins R, 2012, AM J SPORT MED, V40, P2154, DOI 10.1177/0363546512453293
   Yang B, 2017, INT J SURG, V40, P178, DOI 10.1016/j.ijsu.2017.03.021
   Zhou YW, 2014, J ROBOT SURG, V8, P201, DOI 10.1007/s11701-014-0451-6
NR 29
TC 4
Z9 4
U1 0
U2 0
PU SAGE PUBLICATIONS LTD
PI LONDON
PA 1 OLIVERS YARD, 55 CITY ROAD, LONDON EC1Y 1SP, ENGLAND
SN 1022-5536
EI 2309-4990
J9 J ORTHOP SURG-HONG K
JI J. Orthop. Surg.
PD JAN
PY 2020
VL 28
IS 1
AR 2309499020910274
DI 10.1177/2309499020910274
PG 8
WC Orthopedics; Surgery
WE Science Citation Index Expanded (SCI-EXPANDED)
SC Orthopedics; Surgery
GA LB0LN
UT WOS:000524329700001
PM 32186233
OA gold
DA 2022-07-22
ER

PT J
AU Song, JH
   Kang, C
   Han, SY
   Park, WH
   Kim, MH
   Moon, JH
   Tae, JY
   Park, HY
   Yoo, IH
   Park, JH
   Yeo, YH
   Kim, DY
AF Song, Jae H.
   Kang, Chan
   Han, Seung Y.
   Park, Won H.
   Kim, Min H.
   Moon, Ji H.
   Tae, Jung Y.
   Park, Hyeon Y.
   Yoo, In H.
   Park, Jong H.
   Yeo, Yong H.
   Kim, Dae Y.
TI Comparative analysis of Achilles tendon healing outcomes after open
   tenotomy versus percutaneous tenotomy: An experimental study in rats
SO JOURNAL OF ORTHOPAEDIC RESEARCH
LA English
DT Article
DE Achilles tendon; animal model; open tenotomy; orthopedics; percutaneous
   tenotomy
ID GROWTH-FACTOR; REPAIR; RUPTURES; INJURY; MECHANISMS; MORPHOLOGY
AB There is growing interest in conservative treatment of Achilles tendon rupture. However, the majority of experimental studies of Achilles tendon have been performed by open tenotomy. More appropriate model of conservative treatment of Achilles tendon rupture is required. We performed an experimental study to evaluate whether outcomes differ between open tenotomy and percutaneous tenotomy of the Achilles tendon in rats. The Achilles tendons of 48 rats were transected. The animals were divided into two groups according to surgical technique: open tenotomy or microscopy-assisted percutaneous tenotomy. After 1, 2, and 4 weeks, functional, biomechanical, and histological analyses were performed. Western blot was performed for quantitative molecular analysis at 1 week. The Achilles functional index was superior in the percutaneous tenotomy group, compared with the open tenotomy group, at 1 week. The cross-sectional area was significantly larger in the percutaneous tenotomy group than in the open tenotomy group at 4 weeks. Relative to the native tendons, load to failure and stiffness yielded comparable results at 2 weeks in the percutaneous tenotomy group and at 4 weeks in the open tenotomy group. The histological score was significantly better in the percutaneous tenotomy group than in the open tenotomy group at 1 week. At 1 week, interleukin-1 beta expression in the open tenotomy group was higher than in the percutaneous tenotomy group. In summary, Achilles tendon healing was substantially affected by the tenotomy method. We presume that our percutaneous tenotomy method might constitute a useful experimental animal model for conservative treatment of Achilles tendon rupture.
C1 [Song, Jae H.; Kim, Dae Y.] Konyang Univ Hosp, Dept Orthoped Surg, Daejeon, South Korea.
   [Kang, Chan] Chungnam Natl Univ Hosp, Dept Orthoped Surg, Reg Rheumatoid & Degenerat Arthrit Ctr, Daejeon, South Korea.
   [Han, Seung Y.; Moon, Ji H.] Konyang Univ, Dept Anat, Coll Med, 158 Gwanjeodong Ro, Daejeon 35365, South Korea.
   [Park, Won H.; Yeo, Yong H.] Chungnam Natl Univ, Dept Adv Organ Mat & Text Engn Syst, Daejeon, South Korea.
   [Kim, Min H.] Indiana Univ Purdue Univ, Purdue Sch Engn & Technol, Dept Biomed Engn, Indianapolis, IN 46202 USA.
   [Tae, Jung Y.] Konyang Univ, Dept Med, Coll Med, Daejeon, South Korea.
   [Park, Hyeon Y.; Yoo, In H.] Konyang Univ, Dept Occupat Therapy, Daejeon, South Korea.
   [Park, Jong H.] Konyang Univ, Dept Biomed Mat, Coll Med Engn, Daejeon, South Korea.
RP Han, SY (通讯作者)，Konyang Univ, Dept Anat, Coll Med, 158 Gwanjeodong Ro, Daejeon 35365, South Korea.; Kang, C (通讯作者)，Chungnam Natl Univ, Sch Med, Dept Orthoped Surg, 282 Munwha Ro, Daejeon 35015, South Korea.
EM faschan@daum.net; jjzzy@konyang.ac.kr
RI Song, Jaehwang/AAX-9140-2021
OI Song, Jaehwang/0000-0001-5192-4965
FU National Research Foundation of Korea (NRF) grant - Korea Government
   (MSIT) [2019R1G1A1097704]; Korea Medical Device Development Fund grant -
   Korea Government (the Ministry of Science and ICT) [202011C18-02]; Korea
   Medical Device Development Fund grant - Korea Government (Ministry of
   Trade, Industry and Energy) [202011C18-02]; Korea Medical Device
   Development Fund grant - Korea Government (Ministry of Health & Welfare,
   Republic of Korea) [202011C18-02]; Korea Medical Device Development Fund
   grant - Korea Government (Ministry of Food and Drug Safety)
   [202011C18-02]
FX National Research Foundation of Korea (NRF) grant funded by the Korea
   Government (MSIT), Grant/Award Number: No.2019R1G1A1097704; Korea
   Medical Device Development Fund grant funded by the Korea Government
   (the Ministry of Science and ICT, the Ministry of Trade, Industry and
   Energy, the Ministry of Health & Welfare, Republic of Korea, the
   Ministry of Food and Drug Safety), Grant/Award Number: 202011C18-02
CR Aspenberg P, 2004, ACTA ORTHOP SCAND, V75, P93, DOI 10.1080/00016470410001708190
   BEST TM, 1993, J ORTHOP RES, V11, P897, DOI 10.1002/jor.1100110617
   Bhandari M, 2002, CLIN ORTHOP RELAT R, P190
   Bolt P, 2007, J BONE JOINT SURG AM, V89A, P1315, DOI 10.2106/JBJS.F.00257
   BROWN TD, 1981, J TRAUMA, V21, P951, DOI 10.1097/00005373-198111000-00007
   CETTI R, 1993, AM J SPORT MED, V21, P791, DOI 10.1177/036354659302100606
   Chen TM, 2009, CLIN ANAT, V22, P377, DOI 10.1002/ca.20758
   Cook JL, 2004, J ORTHOP RES, V22, P334, DOI 10.1016/j.orthres.2003.08.005
   da Costa J., 2017, OPEN J ANIM SCI, V8, P1
   Dimmen S, 2009, KNEE SURG SPORT TR A, V17, P835, DOI 10.1007/s00167-009-0763-7
   Fearon A, 2014, J SCI MED SPORT, V17, P346, DOI 10.1016/j.jsams.2013.07.008
   Forslund C, 2003, J ORTHOPAED RES, V21, P617, DOI 10.1016/S0736-0266(03)00010-X
   Gabler C, 2018, BIOMED RES INT, V2018, DOI 10.1155/2018/6432742
   Heitman DE, 2011, FOOT ANKLE INT, V32, P879, DOI 10.3113/FAI.2011.0879
   Huegel J, 2020, J ORTHOP RES, V38, P70, DOI 10.1002/jor.24487
   Hwang D-S., 2020, J ORTHOP SURG-HONG K, V28
   Karousou E, 2008, CLIN ORTHOP RELAT R, V466, P1577, DOI 10.1007/s11999-008-0255-y
   Keller A, 2014, AM J SPORT MED, V42, P731, DOI 10.1177/0363546513511418
   Khan RJK, 2005, J BONE JOINT SURG AM, V87A, P2202, DOI 10.2106/JBJS.D.03049
   Kim YS., 2020, ORTHOP J SPORTS MED, V8
   Kurtz CA, 1999, AM J SPORT MED, V27, P363, DOI 10.1177/03635465990270031701
   Lee SY, 2017, AM J SPORT MED, V45, P1429, DOI 10.1177/0363546517689874
   Leppilahti J, 1996, ACTA ORTHOP SCAND, V67, P277, DOI 10.3109/17453679608994688
   Liang JI, 2012, ANN BIOMED ENG, V40, P2532, DOI 10.1007/s10439-012-0619-z
   Maffulli N, 2000, AM J SPORT MED, V28, P857, DOI 10.1177/03635465000280061401
   Moller M, 2001, J BONE JOINT SURG BR, V83B, P843
   Muller SA, 2016, AM J SPORT MED, V44, P1998, DOI 10.1177/0363546516641942
   Murrell G A, 1993, Foot Ankle, V14, P400
   MURRELL GAC, 1992, J ORTHOPAED RES, V10, P398, DOI 10.1002/jor.1100100313
   National Research Council (US) Institute for Laboratory Animal Research, 1996, GUIDE CARE USE LAB A
   Netto CD, 2018, PLOS ONE, V13, DOI 10.1371/journal.pone.0192769
   Noguchi Masahiko, 2002, J Orthop Sci, V7, P247, DOI 10.1007/s007760200041
   Okamoto N, 2010, J BONE JOINT SURG AM, V92A, P2776, DOI 10.2106/JBJS.I.01325
   Parker R, 2004, COCHRANE DB SYST REV, V8
   Sharma P., 2006, Journal of Musculoskeletal & Neuronal Interactions, V6, P181
   Suh DS, 2017, AM J SPORT MED, V45, P2019, DOI 10.1177/0363546517703336
   Takamura M, 2017, ACTA ORTHOP TRAUMATO, V51, P65, DOI 10.1016/j.aott.2016.12.001
   Thomopoulos S, 2015, J ORTHOP RES, V33, P832, DOI 10.1002/jor.22806
   Uquillas CA, 2015, J BONE JOINT SURG AM, V97A, P1187, DOI 10.2106/JBJS.O.00002
   Vadala Antonio, 2014, Muscles Ligaments Tendons J, V4, P177
   Virchenko O, 2004, AM J SPORT MED, V32, P1743, DOI 10.1177/0363546504263403
   Waterston SW, 1997, BRIT J SPORT MED, V31, P285, DOI 10.1136/bjsm.31.4.285
   Webb WR, 2013, BIOMATERIALS, V34, P6683, DOI 10.1016/j.biomaterials.2013.05.041
   WILLIAMS IF, 1980, RES VET SCI, V28, P302, DOI 10.1016/S0034-5288(18)32713-9
   Yang B, 2017, INT J SURG, V40, P178, DOI 10.1016/j.ijsu.2017.03.021
   Zhang F, 2003, PLAST RECONSTR SURG, V112, P1613, DOI 10.1097/01.PRS.0000086772.72535.A4
NR 46
TC 2
Z9 1
U1 0
U2 1
PU WILEY
PI HOBOKEN
PA 111 RIVER ST, HOBOKEN 07030-5774, NJ USA
SN 0736-0266
EI 1554-527X
J9 J ORTHOP RES
JI J. Orthop. Res.
PD JUN
PY 2022
VL 40
IS 6
BP 1446
EP 1456
DI 10.1002/jor.25159
EA AUG 2021
PG 11
WC Orthopedics
WE Science Citation Index Expanded (SCI-EXPANDED)
SC Orthopedics
GA 1D7IF
UT WOS:000686925100001
PM 34370341
DA 2022-07-22
ER

PT J
AU Pardes, AM
   Beach, ZM
   Raja, H
   Rodriguez, AB
   Freedman, BR
   Soslowsky, LJ
AF Pardes, A. M.
   Beach, Z. M.
   Raja, H.
   Rodriguez, A. B.
   Freedman, B. R.
   Soslowsky, L. J.
TI Aging leads to inferior Achilles tendon mechanics and altered ankle
   function in rodents
SO JOURNAL OF BIOMECHANICS
LA English
DT Article
DE Biomechanics; Injury; Fatigue; Skeletal muscle; Elasticity;
   Orthopaedics; Age
ID MIDDLE-AGED ADULTS; OLDER-ADULTS; MUSCLE; EXERCISE; RAT; STRENGTH;
   RUPTURES; WALKING; MODEL; FOOT
AB Spontaneous rupture of the Achilles tendon is increasingly common in the middle aged population. However, the cause for the particularly high incidence of injury in this age group is not well understood. Therefore, the objective of this study was to identify age-specific differences in the Achilles tendon muscle complex using an animal model. Functional measures were performed in vivo and tissues were harvested following euthanasia for mechanical, structural, and histological analysis from young, middle aged, and old rats. Numerous alterations in tendon properties were detected across age groups, including inferior material properties (maximum stress, modulus) with increasing age. Differences in function were also observed, as older animals exhibited increased ankle joint passive stiffness and decreased propulsion force during locomotion. Macroscale differences in tendon organization were not observed, although cell density and nuclear shape did vary between age groups. Muscle fiber size and type distribution were not notably affected by age, indicating that other factors may be more responsible for age-specific Achilles tendon rupture rates. This study improves our understanding of the role of aging in Achilles tendon biomechanics and ankle function, and helps provide a potential explanation for the disparate incidence of Achilles tendon ruptures in varying age groups. (C) 2017 Elsevier Ltd. All rights reserved.
C1 [Pardes, A. M.; Beach, Z. M.; Raja, H.; Rodriguez, A. B.; Freedman, B. R.; Soslowsky, L. J.] Univ Penn, McKay Orthopaed Lab, 424 Stemmler Hall,3450 Hamilton Walk, Philadelphia, PA 19104 USA.
RP Soslowsky, LJ (通讯作者)，Univ Penn, McKay Orthopaed Lab, 424 Stemmler Hall,3450 Hamilton Walk, Philadelphia, PA 19104 USA.
EM soslowsk@upenn.edu
FU NIH/NIAMS [R01AR06421652, P30AR050950]; NSF GRFP; NATIONAL INSTITUTE OF
   ARTHRITIS AND MUSCULOSKELETAL AND SKIN DISEASES [T32AR007132,
   P30AR050950, R01AR064216] Funding Source: NIH RePORTER
FX This study was funded by NIH/NIAMS (R01AR06421652, P30AR050950) and the
   NSF GRFP.
CR Cury DP, 2016, PLOS ONE, V11, DOI 10.1371/journal.pone.0153568
   Danos N., 2016, J APPL PHYSL
   Devita P, 2016, MED SCI SPORT EXER, V48, P98, DOI 10.1249/MSS.0000000000000744
   Doherty TJ, 2003, J APPL PHYSIOL, V95, P1717, DOI 10.1152/japplphysiol.00347.2003
   Dudhia J, 2007, AGING CELL, V6, P547, DOI 10.1111/j.1474-9726.2007.00307.x
   Franz JR, 2016, EXERC SPORT SCI REV, V44, P129, DOI 10.1249/JES.0000000000000086
   Freedman B. R., 2016, J ORTHOP RES
   Fryhofer G. W., 2016, J APPL PHYSL
   Huttunen TT, 2014, AM J SPORT MED, V42, P2419, DOI 10.1177/0363546514540599
   Joseph MF, 2014, J STRENGTH COND RES, V28, P1181, DOI 10.1519/JSC.0000000000000361
   Karamanidis K, 2006, J BIOMECH, V39, P406, DOI 10.1016/j.jbiomech.2004.12.017
   Ko SU, 2012, ARCH GERONTOL GERIAT, V55, P474, DOI 10.1016/j.archger.2012.04.004
   Kostrominova TY, 2013, AGE, V35, P2203, DOI 10.1007/s11357-013-9514-2
   Kubo K, 2007, MED SCI SPORT EXER, V39, P541, DOI 10.1249/01.mss.0000247006.24965.74
   Langberg H, 2001, EUR J APPL PHYSIOL, V84, P246, DOI 10.1007/s004210170013
   Lantto I, 2015, SCAND J MED SCI SPOR, V25, pE133, DOI 10.1111/sms.12253
   LARSSON L, 1979, J APPL PHYSIOL, V46, P451, DOI 10.1152/jappl.1979.46.3.451
   Lenskjold A, 2015, SCAND J MED SCI SPOR, V25, P25, DOI 10.1111/sms.12143
   Leppilahti J, 1996, ACTA ORTHOP SCAND, V67, P277, DOI 10.3109/17453679608994688
   Maffulli N, 2000, AM J SPORT MED, V28, P499, DOI 10.1177/03635465000280040901
   Mannava S, 2011, J BONE JOINT SURG AM, V93A, P1702, DOI [10.2106/JBJSJ.00184, 10.2106/JBJS.J.00184]
   Menz HB, 2005, J GERONTOL A-BIOL, V60, P1546, DOI 10.1093/gerona/60.12.1546
   Menz HB, 2015, GERONTOLOGY, V61, P381, DOI 10.1159/000368357
   Mian OS, 2007, ACTA PHYSIOL, V189, P57, DOI 10.1111/j.1748-1716.2006.01634.x
   Mian OS, 2006, ACTA PHYSIOL, V186, P127, DOI 10.1111/j.1748-1716.2006.01522.x
   Ng N, 2011, GLOBAL HEALTH ACTION, V4, DOI 10.3402/gha.v4i0.6347
   Onambele GL, 2006, J APPL PHYSIOL, V100, P2048, DOI 10.1152/japplphysiol.01442.2005
   Pardes AM, 2016, ANN BIOMED ENG, V44, P2901, DOI 10.1007/s10439-016-1635-1
   Peffers MJ, 2015, ARTHRITIS RES THER, V17, DOI 10.1186/s13075-015-0544-2
   Plate JF, 2013, J BIOMECH, V46, P450, DOI 10.1016/j.jbiomech.2012.11.007
   Quinn R, 2005, NUTRITION, V21, P775, DOI 10.1016/j.nut.2005.04.002
   Riggin CN, 2014, J BIOMECH ENG-T ASME, V136, DOI 10.1115/1.4026285
   Rooney SI, 2016, AM J SPORT MED, V44, P2237, DOI 10.1177/0363546516646377
   Schmitz A, 2009, J ELECTROMYOGR KINES, V19, P1085, DOI 10.1016/j.jelekin.2008.10.008
   Slane LC, 2017, EUR RADIOL, V27, P474, DOI 10.1007/s00330-016-4409-0
   Slane LC, 2015, MED ENG PHYS, V37, P712, DOI 10.1016/j.medengphy.2015.04.004
   Slane LC, 2015, PHYSIOL MEAS, V36, P1485, DOI 10.1088/0967-3334/36/7/1485
   Smith LR, 2014, SKELET MUSCLE, V4, DOI 10.1186/2044-5040-4-21
   Smith RKW, 2002, COMP BIOCHEM PHYS A, V133, P1039, DOI 10.1016/S1095-6433(02)00148-4
   Spink MJ, 2011, ARCH PHYS MED REHAB, V92, P68, DOI 10.1016/j.apmr.2010.09.024
   Stenroth L, 2012, J APPL PHYSIOL, V113, P1537, DOI 10.1152/japplphysiol.00782.2012
   Suchak AA, 2005, FOOT ANKLE INT, V26, P932, DOI 10.1177/107110070502601106
   Suydam SM, 2015, KNEE SURG SPORT TR A, V23, P868, DOI 10.1007/s00167-013-2512-1
   Svensson RB, 1985, J APPL PHYSL, P2016
   Thornton GM, 2015, J BIOMECH, V48, P3306, DOI 10.1016/j.jbiomech.2015.06.005
   Thorpe CT, 2016, SCI REP-UK, V6, DOI 10.1038/srep20455
   Wood LK, 2011, J APPL PHYSIOL, V111, P999, DOI 10.1152/japplphysiol.00460.2011
   Yu TY, 2013, BMC MUSCULOSKEL DIS, V14, DOI 10.1186/1471-2474-14-2
   Zaseck LW, 2016, J GERONTOL A-BIOL, V71, P858, DOI 10.1093/gerona/glv307
NR 49
TC 30
Z9 29
U1 0
U2 15
PU ELSEVIER SCI LTD
PI OXFORD
PA THE BOULEVARD, LANGFORD LANE, KIDLINGTON, OXFORD OX5 1GB, OXON, ENGLAND
SN 0021-9290
EI 1873-2380
J9 J BIOMECH
JI J. Biomech.
PD JUL 26
PY 2017
VL 60
BP 30
EP 38
DI 10.1016/j.jbiomech.2017.06.008
PG 9
WC Biophysics; Engineering, Biomedical
WE Science Citation Index Expanded (SCI-EXPANDED)
SC Biophysics; Engineering
GA FE5YN
UT WOS:000408287300005
PM 28683928
OA Green Accepted
DA 2022-07-22
ER

PT J
AU Saxena, A
   Hofer, D
AF Saxena, Amol
   Hofer, Deann
TI Triple Achilles Tendon Rupture: Case Report
SO JOURNAL OF FOOT & ANKLE SURGERY
LA English
DT Article
DE Achilles rupture; athlete; football; running; soccer
ID HYPERTHERMIA
AB We present a case report with 1-year follow-up data of a 57-year-old male soccer referee who had sustained an acute triple Achilles tendon rupture injury during a game. His triple Achilles tendon rupture consisted of a rupture of the proximal watershed region, a rupture of the main body (mid-watershed area), and an avulsion-type rupture of insertional calcific tendinosis. The patient was treated surgically with primary repair of the tendon, including tenodesis with anchors. Postoperative treatment included non-weightbearing for 4 weeks and protected weightbearing until 10 weeks postoperative, followed by formal physical therapy, which incorporated an "antigravity" treadmill. The patient was able to return to full activity after 26 weeks, including running and refereeing, without limitations. (C) 2017 by the American College of Foot and Ankle Surgeons. All rights reserved.
C1 [Saxena, Amol; Hofer, Deann] Sutter Palo Alto, Dept Sports Med, Palo Alto, CA USA.
RP Saxena, A (通讯作者)，Palo Alto Med Ctr, Clark Bldg,Level 3,795 El Camino Real, Palo Alto, CA 94301 USA.
EM heysax@aol.com
CR Birch HL, 1997, J EXP BIOL, V200, P1703
   Casparian JM, 2000, SOUTHERN MED J, V93, P488, DOI 10.1097/00007611-200005000-00008
   Cottom James M, 2008, Foot Ankle Spec, V1, P280, DOI 10.1177/1938640008322690
   Gwynne-Jones DP, 2011, FOOT ANKLE INT, V32, P337, DOI 10.3113/FAI.2011.0337
   HATTRUP SJ, 1985, FOOT ANKLE, V6, P34, DOI 10.1177/107110078500600107
   Hersh BL, 2002, J AM PODIAT MED ASSN, V92, P355, DOI 10.7547/87507315-92-6-355
   JOZSA L, 1989, AM J SPORT MED, V17, P338, DOI 10.1177/036354658901700305
   Khan RJK, 2010, COCHRANE DB SYST REV, DOI 10.1002/14651858.CD003674.pub4
   Khan RJK, 2005, J BONE JOINT SURG AM, V87A, P2202, DOI 10.2106/JBJS.D.03049
   Kotian SR, 2013, ANAT SCI INT, V88, P239, DOI 10.1007/s12565-013-0184-z
   Maffulli N, 2012, J BONE JOINT SURG AM, V94A, P901, DOI 10.2106/JBJS.K.00200
   Matles A L, 1975, Bull Hosp Joint Dis, V36, P48
   McCullough Kirk A, 2014, J Surg Orthop Adv, V23, P179
   Porsch M, 1998, Z ORTHOP GRENZGEB, V136, P568, DOI 10.1055/s-2008-1045188
   Saxena A, 2001, J Foot Ankle Surg, V40, P132
   Saxena A, 2011, J FOOT ANKLE SURG, V50, P558, DOI 10.1053/j.jfas.2011.04.045
   Saxena A, 2011, J FOOT ANKLE SURG, V50, P37, DOI 10.1053/j.jfas.2010.10.008
   SIMMONDS F A, 1957, Practitioner, V179, P56
   STANISH WD, 1984, MED SCI SPORT EXER, V16, P1
   Tallon C, 2001, MED SCI SPORT EXER, V33, P1983, DOI 10.1097/00005768-200112000-00002
   Vanek D, 2003, J AM PODIAT MED ASSN, V93, P333, DOI 10.7547/87507315-93-4-333
   Ververidis AN, 2016, J ORTHOP, V13, P57, DOI 10.1016/j.jor.2015.09.004
   WILSON AM, 1994, J BIOMECH, V27, P899, DOI 10.1016/0021-9290(94)90262-3
NR 23
TC 2
Z9 2
U1 1
U2 6
PU ELSEVIER SCIENCE INC
PI NEW YORK
PA STE 800, 230 PARK AVE, NEW YORK, NY 10169 USA
SN 1067-2516
EI 1542-2224
J9 J FOOT ANKLE SURG
JI J. Foot Ankle Surg.
PD MAR-APR
PY 2018
VL 57
IS 2
BP 404
EP 408
DI 10.1053/j.jfas.2017.08.023
PG 5
WC Orthopedics; Surgery
WE Science Citation Index Expanded (SCI-EXPANDED)
SC Orthopedics; Surgery
GA FX8OS
UT WOS:000426355000035
PM 29154089
DA 2022-07-22
ER

PT J
AU Cuttica, DJ
   Hyer, CF
   Berlet, GC
AF Cuttica, Daniel J.
   Hyer, Christopher F.
   Berlet, Gregory C.
TI Intraoperative Value of the Thompson Test
SO JOURNAL OF FOOT & ANKLE SURGERY
LA English
DT Article
DE Achilles; rupture; tendon; Thompson test
ID ACHILLES-TENDON
AB The purpose of the present study was to assess the validity of the Thompson sign and determine whether the deep flexors of the foot can produce a falsely intact Achilles tendon. Ten unmatched above-the-knee lower extremity cadaveric specimens were studied. In group 1, the Achilles tendon was sectioned into 25% increments. The Thompson maneuver was performed after each sequential sectioning of the Achilles tendon, including after it had been completely sectioned. If the Thompson sign was still intact after complete release of the Achilles tendon, we proceeded to release the tendon, and tendon flexor hallucis longus, flexor digitorum longus, and posterior tibial tendons. The Thompson test was performed after the release of each tendon. In group 2, the tendon releases were performed in a reverse order to that of group 1, with the Thompson test performed after each release. In group 1, the Thompson sign remained intact in all specimens after sectioning of 25%, 50%, and 75% of the tendon. After complete (100%) release of the tendon, the Thompson sign was absent in all specimens. In group 2, the Thompson sign remained intact after sectioning of the posterior tibial, flexor digitorum longus, and flexor hallucis longus tendons in all specimens. The Thompson sign remained intact in all specimens after sectioning of 25%, 50%, and 75% of the Achilles tendon. After complete release of the tendon, the Thompson sign was absent in all specimens. The Thompson test is an accurate clinical test for diagnosing complete Achilles tendon ruptures. However, it might not be a useful test for diagnosing partial Achilles tendon ruptures. Our findings also call into question the usefulness of the Thompson test in the intraoperative setting. (C) 2015 by the American College of Foot and Ankle Surgeons. All rights reserved.
C1 [Cuttica, Daniel J.] Orthopaed Foot & Ankle Ctr, Washington, DC USA.
   [Hyer, Christopher F.; Berlet, Gregory C.] Orthoped Foot & Ankle Ctr, Westerville, OH 43082 USA.
RP Hyer, CF (通讯作者)，Orthoped Foot & Ankle Ctr, 300 Polaris Pkwy,Suite 2000, Westerville, OH 43082 USA.
EM ofacresearch@orthofootankle.com
CR Kolodziej P, 1999, FOOT ANKLE INT, V20, P433, DOI 10.1177/107110079902000707
   Lagergren C, 1959, ACTA CHIR SCAND, V116, P491
   RALSTON EL, 1971, J TRAUM, V11, P15, DOI 10.1097/00005373-197101000-00001
   SIMMONDS F A, 1957, Practitioner, V179, P56
   THOMPSON T C, 1962, J Trauma, V2, P126, DOI 10.1097/00005373-196203000-00003
   THOMPSON T C, 1962, Acta Orthop Scand, V32, P461, DOI 10.3109/17453676208989608
   1973, LANCET, V1, P189
NR 7
TC 3
Z9 4
U1 0
U2 3
PU ELSEVIER SCIENCE INC
PI NEW YORK
PA 360 PARK AVE SOUTH, NEW YORK, NY 10010-1710 USA
SN 1067-2516
EI 1542-2224
J9 J FOOT ANKLE SURG
JI J. Foot Ankle Surg.
PD JAN-FEB
PY 2015
VL 54
IS 1
BP 99
EP 101
DI 10.1053/j.jfas.2014.09.014
PG 3
WC Orthopedics; Surgery
WE Science Citation Index Expanded (SCI-EXPANDED)
SC Orthopedics; Surgery
GA AW7CH
UT WOS:000346421600020
PM 25441265
DA 2022-07-22
ER

PT J
AU Jielile, J
   Aibai, M
   Sabirhazi, G
   Shawutali, N
   Tangkejie, W
   Badelhan, A
   Nuerduola, Y
   Satewalede, T
   Buranbai, D
   Hunapia, B
   Jialihasi, A
   Bai, JP
   Kizaibek, M
AF Jielile, Jiasharete
   Aibai, Minawa
   Sabirhazi, Gulnur
   Shawutali, Nuerai
   Tangkejie, Wulanbai
   Badelhan, Aynaz
   Nuerduola, Yeermike
   Satewalede, Turde
   Buranbai, Darehan
   Hunapia, Beicen
   Jialihasi, Ayidaer
   Bai, Jingping
   Kizaibek, Murat
TI Active Achilles tendon kinesitherapy accelerates Achilles tendon repair
   by promoting neurite regeneration
SO NEURAL REGENERATION RESEARCH
LA English
DT Article
DE achilles tendon rupture; early; motion; functional exercise; exercise;
   Achilles tendon; healing; proteomics; marker; tissue repair
ID SURGICAL REPAIR; FUNCTIONAL TREATMENT; RUPTURE; PROTEINS; GELSOLIN;
   CRMP-2; REHABILITATION; IMMOBILIZATION; WEIGHTBEARING; PROTEOMICS
AB Active Achilles tendon kinesitherapy facilitates the functional recovery of a ruptured Achilles tendon. However, protein expression during the healing process remains a controversial issue. New Zealand rabbits, aged 14 weeks, underwent tenotomy followed immediately by Achilles tendon microsurgery to repair the Achilles tendon rupture. The tendon was then immobilized or subjected to postoperative early motion treatment (kinesitherapy). Mass spectrography results showed that after 14 days of motion treatment, 18 protein spots were differentially expressed, among which, 12 were up-regulated, consisting of gelsolin isoform b and neurite growth-related protein collapsing response mediator protein 2. Western blot analysis showed that gelsolin isoform b was up-regulated at days 7-21 of motion treatment. These findings suggest that active Achilles tendon kinesitherapy promotes the neurite regeneration of a ruptured Achilles tendon and gelsolin isoform b can be used as a biomarker for Achilles tendon healing after kinesitherapy.
C1 [Jielile, Jiasharete; Shawutali, Nuerai; Tangkejie, Wulanbai; Badelhan, Aynaz; Nuerduola, Yeermike; Satewalede, Turde; Buranbai, Darehan; Hunapia, Beicen; Jialihasi, Ayidaer] Xinjiang Med Univ, Teaching Hosp 1, Dept Microsurg Repair & Reconstruct, Urumqi 830054, Xinjiang Uyghur, Peoples R China.
   [Jielile, Jiasharete; Shawutali, Nuerai; Tangkejie, Wulanbai; Badelhan, Aynaz; Nuerduola, Yeermike; Satewalede, Turde; Buranbai, Darehan; Hunapia, Beicen; Jialihasi, Ayidaer] Res Inst Orthoped Xinjiang Uyghur Autonomous Reg, Sports Med Res Ctr, Urumqi 830054, Xinjiang Uyghur, Peoples R China.
   [Aibai, Minawa] Urumqi Ctr Dis Control & Prevent, Urumqi 830026, Xinjiang Uyghur, Peoples R China.
   [Sabirhazi, Gulnur] Chinese Acad Sci, Xinjiang Inst Ecol & Geog, Urumqi 830011, Xinjiang Uyghur, Peoples R China.
   [Bai, Jingping] Xinjiang Med Univ, Teaching Hosp 3, Dept Orthoped, Urumqi 830011, Xinjiang Uyghur, Peoples R China.
   [Kizaibek, Murat] Res Inst Kazakh Tradit Med Ili Kazakh Autonomous, Yining 835000, Ili Kazakh Auto, Peoples R China.
RP Bai, JP (通讯作者)，Xinjiang Med Univ, Teaching Hosp 3, Dept Orthoped, 30 Beijing S Rd, Urumqi 830011, Xinjiang Uyghur, Peoples R China.
EM jingping_bai@163.com
OI Kizaibek, Murat/0000-0002-5861-3809
FU National Natural Science Foundation of China [30760256]
FX This study was financially supported by the National Natural Science
   Foundation of China, No. 30760256.
CR Aoki M, 1998, AM J SPORT MED, V26, P794, DOI 10.1177/03635465980260061001
   Arimura N, 2005, NEURON, V48, P881, DOI 10.1016/j.neuron.2005.11.007
   Arimura N, 2009, J NEUROCHEM, V111, P380, DOI 10.1111/j.1471-4159.2009.06317.x
   Bring D, 2010, SCAND J MED SCI SPOR, V20, P411, DOI 10.1111/j.1600-0838.2009.00954.x
   CETTI R, 1994, CLIN ORTHOP RELAT R, P155
   Chae YC, 2009, CELL SIGNAL, V21, P1818, DOI 10.1016/j.cellsig.2009.07.017
   Chen Xue-Song, 2012, Zhonghua Zheng Xing Wai Ke Za Zhi, V28, P22
   Cretnik A, 2010, FOOT ANKLE INT, V31, P14, DOI 10.3113/FAI.2010.0014
   Fukata Y, 2002, NAT CELL BIOL, V4, P583, DOI 10.1038/ncb825
   Gharahdaghi F, 1999, ELECTROPHORESIS, V20, P601, DOI 10.1002/(SICI)1522-2683(19990301)20:3<601::AID-ELPS601>3.3.CO;2-Y
   Grewal R, 2006, CLIN BIOMECH, V21, P804, DOI 10.1016/j.clinbiomech.2006.05.002
   Hamajima N, 1996, GENE, V180, P157, DOI 10.1016/S0378-1119(96)00445-3
   Hess Gregory William, 2010, Foot Ankle Spec, V3, P29, DOI 10.1177/1938640009355191
   James R, 2008, J HAND SURG-AM, V33A, P102, DOI 10.1016/j.jhsa.2007.09.007
   [加莎热特·杰力勒 Jia Sharetejielilei], 2004, [医用生物力学, Journal of medical biomechanics], V19, P108
   [加莎热特·杰力勒 Jiasharete.Jielile], 2008, [中国组织工程研究与临床康复, Journal of Clinical Rehabilitative Tissue Engineering Research], V12, P8352
   [加莎热特·杰力勒 Jielile J], 2007, [中国组织工程研究与临床康复, Journal of Clinical Rehabilitative Tissue Engineering Research], V11, P1419
   Jielile J, 2003, CHIN J GEN PRACT, V12, P49
   Jielile J, 2004, CHIN GEN PRACT, V3, P823
   Jielile J, 2003, CHIN J MED BIOMECH, V18, P62
   Jielile J, 2010, CLIN BIOMECH, V25, P789, DOI 10.1016/j.clinbiomech.2010.05.005
   Kawano Y, 2005, MOL CELL BIOL, V25, P9920, DOI 10.1128/MCB.25.22.9920-9935.2005
   Kerkhoffs GMMJ, 2002, ARCH ORTHOP TRAUM SU, V122, P102, DOI 10.1007/s004020100312
   Kimura T, 2005, J NEUROCHEM, V93, P1371, DOI 10.1111/j.1471-4159.2005.03063.x
   LEVY M, 1984, CLIN ORTHOP RELAT R, P199
   LIEBERMAN JR, 1988, CLIN ORTHOP RELAT R, P204
   Litwin M, 2009, ACTA BIOCHIM POL, V56, P739
   Lynch Richard M, 2004, Accid Emerg Nurs, V12, P149, DOI 10.1016/j.aaen.2003.11.004
   Maffulli N, 1999, CLIN J SPORT MED, V9, P157, DOI 10.1097/00042752-199907000-00007
   Maffulli Nicola, 2007, Foot Ankle Clin, V12, P583, DOI 10.1016/j.fcl.2007.07.007
   Maffulli N, 2008, BMC MUSCULOSKEL DIS, V9, DOI 10.1186/1471-2474-9-100
   MARTI R, 1974, HELV CHIR ACTA, V41, P293
   Molloy A, 2009, FOOT ANKLE CLIN, V14, P745, DOI 10.1016/j.fcl.2009.07.004
   Motta P, 1997, AM J SPORT MED, V25, P172, DOI 10.1177/036354659702500205
   Ohnishi M, 2009, PATHOL INT, V59, P797, DOI 10.1111/j.1440-1827.2009.02447.x
   Parekh Selene G, 2009, Foot Ankle Spec, V2, P283, DOI 10.1177/1938640009351138
   Peter K, 2010, BLOOD, V115, P4008, DOI 10.1182/blood-2010-02-270298
   Sankey RA, 2008, AM J SPORT MED, V36, P2415, DOI 10.1177/0363546508322889
   Shoja MM, 2009, AM J MED SCI, V337, P370, DOI 10.1097/MAJ.0b013e3181a4199c
   Silacci P, 2004, CELL MOL LIFE SCI, V61, P2614, DOI 10.1007/s00018-004-4225-6
   Solomon JP, 2009, BIOCHEMISTRY-US, V48, P11370, DOI 10.1021/bi901368e
   Sorrenti SJ, 2006, FOOT ANKLE INT, V27, P407, DOI 10.1177/107110070602700603
   Speck M, 1998, AM J SPORT MED, V26, P789, DOI 10.1177/03635465980260060901
   Sun QJ, 2008, J CELL BIOCHEM, V105, P176, DOI 10.1002/jcb.21811
   Tang B, 2009, J XINJIANG MED U, V32, P463
   Tang B, 2010, J CHIN PHYS, V12, P19
   TROOP RL, 1995, FOOT ANKLE INT, V16, P705, DOI 10.1177/107110079501601106
   Wang JHC, 2006, J BIOMECH, V39, P1563, DOI 10.1016/j.jbiomech.2005.05.011
   Welch GR, 2010, AM J PHYSIOL-CELL PH, V298, pC1280, DOI 10.1152/ajpcell.00016.2010
   Wu YF, 2009, CHIN J MED POSTGRAD, V22, P758
   Yotsumoto T, 2010, AM J SPORT MED, V38, P287, DOI 10.1177/0363546509351557
NR 51
TC 6
Z9 8
U1 0
U2 19
PU WOLTERS KLUWER MEDKNOW PUBLICATIONS
PI MUMBAI
PA WOLTERS KLUWER INDIA PVT LTD , A-202, 2ND FLR, QUBE, C T S  NO 1498A-2
   VILLAGE MAROL, ANDHERI EAST, MUMBAI, 400059, INDIA
SN 1673-5374
EI 1876-7958
J9 NEURAL REGEN RES
JI Neural Regen. Res.
PD DEC 15
PY 2012
VL 7
IS 35
BP 2801
EP 2810
DI 10.3969/j.issn.1673-5374.2012.35.008
PG 10
WC Cell Biology; Neurosciences
WE Science Citation Index Expanded (SCI-EXPANDED)
SC Cell Biology; Neurosciences & Neurology
GA 065KV
UT WOS:000313148100008
PM 25317130
DA 2022-07-22
ER

PT J
AU Davies, H
   Agrawal, Y
   Blundell, C
   Davies, MB
AF Davies, Howard
   Agrawal, Yuvraj
   Blundell, Chris
   Davies, Mark B.
TI Outcome following use of the Achillon jig for the repair of acutely
   ruptured Achilles tendons
SO INJURY-INTERNATIONAL JOURNAL OF THE CARE OF THE INJURED
LA English
DT Article
DE Achilles; Tendon; Repair; Achillon; Sports; Percutaneous
ID PERCUTANEOUS REPAIR; REHABILITATION; MANAGEMENT
AB We report a series of 143 patients who underwent limited open Achilles tendon repair using the Achillon device at a mean follow-up of 25 months.
   All patients attending our institution with a diagnosis of acute Achilles tendon rupture were considered for operative repair using the Achillon jig unless they declined surgery or met the exclusion criteria. Following surgery patients were contacted and asked to complete an Achilles Tendon Rupture Score (ATRS). The clinical records were reviewed for evidence of complications, demographic information and evidence of re-rupture. Statistical analysis of subgroups including age at presentation, delay to surgery and patients with complications was carried out using the Kruskal Wallis non-parametric test.
   We report no re-ruptures at a mean of 25 months (minimum 12 months) following surgery. The mean ATRS score was 84/100 (range 15-100). There was no statistical significance between any of the subgroups analyzed.
   In conclusion, acute Achilles repair using the Achillon jig is safe, with a low re-rupture rate, excellent ATRS scores at a minimum of 12 months post-operatively and low complications. The incidence of sural nerve injury is much lower than published series of percutaneous Achilles tendon repair without the use of a jig.
C1 [Davies, Howard; Agrawal, Yuvraj; Blundell, Chris; Davies, Mark B.] Northern Gen Hosp, Dept Trauma & Orthopaed Surg, Sheffield Foot & Ankle Unit, Herries Rd, Sheffield S5 7AU, S Yorkshire, England.
RP Davies, H (通讯作者)，Northern Gen Hosp, Dept Trauma & Orthopaed Surg, Sheffield Foot & Ankle Unit, Herries Rd, Sheffield S5 7AU, S Yorkshire, England.
EM howard.davies@doctors.org.uk
RI Agrawal, Yuvraj/AGN-7662-2022
OI Agrawal, Yuvraj/0000-0001-6944-2841
CR Aibinder WR, 2013, FOOT ANKLE INT, V34, P870, DOI 10.1177/1071100712473097
   Atinga M, 2008, FOOT ANKLE INT, V29, P814, DOI 10.3113/FAI.2008.0814
   Calder JDF, 2005, BRIT J SPORT MED, V39, P857, DOI 10.1136/bjsm.2004.017509
   Carmont M R, 2011, Foot Ankle Surg, V17, P186, DOI 10.1016/j.fas.2010.06.003
   Ganestam A, 2015, KNEE SURG SPORTS TRA, V20
   Gigante A, 2008, KNEE SURG SPORT TR A, V16, P204, DOI 10.1007/s00167-007-0448-z
   Guillo S, 2013, SURG-J R COLL SURG E, V11, P14, DOI 10.1016/j.surge.2011.12.002
   Heitman DE, 2011, FOOT ANKLE INT, V32, P879, DOI 10.3113/FAI.2011.0879
   Heyes GJ, 2015, EUR J TRAUMA EMERG S, V41, P273, DOI 10.1007/s00068-014-0408-5
   Hutchison AM, 2015, BONE JOINT J, V97B, P510, DOI 10.1302/0301-620X.97B4.35314
   Kearney RS, 2012, J BONE JOINT SURG BR, V94B, P28, DOI 10.1302/0301-620X.94B1.28008
   Kearney RS, 2012, HEALTH QUAL LIFE OUT, V10, DOI 10.1186/1477-7525-10-24
   KLEIN W, 1991, Chirurgia degli Organi di Movimento, V76, P223
   Leppilahti J, 1998, SPORTS MED, V25, P79, DOI 10.2165/00007256-199825020-00002
   Leppilahti J, 1996, ACTA ORTHOP SCAND, V67, P277, DOI 10.3109/17453679608994688
   MA GWC, 1977, CLIN ORTHOP RELAT R, P247
   Maes Renaud, 2006, Acta Orthop Belg, V72, P179
   Soroceanu A, 2012, J BONE JOINT SURG AM, V94A, P2136, DOI 10.2106/JBJS.K.00917
   Strauss EJ, 2007, INJURY, V38, P832, DOI 10.1016/j.injury.2006.06.005
   Tejwani Nirmal C, 2014, Am J Orthop (Belle Mead NJ), V43, pE221
   Valente Maurizio, 2012, Musculoskelet Surg, V96, P35, DOI 10.1007/s12306-011-0158-z
   Van der Eng DM, 2013, J FOOT ANKLE SURG
   Wilkins R, 2012, AM J SPORT MED, V40, P2154, DOI 10.1177/0363546512453293
   Willits K, 2010, J BONE JOINT SURG AM, V92A, P2767, DOI 10.2106/JBJS.I.01401
NR 24
TC 4
Z9 5
U1 0
U2 1
PU ELSEVIER SCI LTD
PI OXFORD
PA THE BOULEVARD, LANGFORD LANE, KIDLINGTON, OXFORD OX5 1GB, OXON, ENGLAND
SN 0020-1383
EI 1879-0267
J9 INJURY
JI Injury-Int. J. Care Inj.
PD MAR
PY 2017
VL 48
IS 3
BP 781
EP 783
DI 10.1016/j.injury.2017.01.018
PG 3
WC Critical Care Medicine; Emergency Medicine; Orthopedics; Surgery
WE Science Citation Index Expanded (SCI-EXPANDED)
SC General & Internal Medicine; Emergency Medicine; Orthopedics; Surgery
GA EQ5CA
UT WOS:000398097700033
PM 28088379
DA 2022-07-22
ER

PT J
AU Hullfish, TJ
   O'Connor, KM
   Baxter, JR
AF Hullfish, Todd J.
   O'Connor, Kathryn M.
   Baxter, Josh R.
TI Medial gastrocnemius muscle remodeling correlates with reduced
   plantarflexor kinetics 14 weeks following Achilles tendon rupture
SO JOURNAL OF APPLIED PHYSIOLOGY
LA English
DT Article
DE clinical research; fascicle; isokinetic dynamometry; ultrasound
ID HEEL-RISE HEIGHT; ELONGATION; REPAIR; LENGTH
AB Deficits in plantarflexor kinetics are associated with poor outcomes in patients following Achilles tendon rupture. In this longitudinal study, we analyzed the fascicle length and pennation angle of the medial gastrocnemius muscle and the length of the Achilles tendon using ultrasound imaging. To determine the relationship between muscle remodeling and deficits in plantarflexor kinetics measured at 14 wk after injury, we correlated the reduction in fascicle length and increase in pennation angle with peak torque measured during isometric and isokinetic plantarflexor contractions. We found that the medial gastrocnemius underwent an immediate change in structure, characterized by decreased length and increased pennation of the muscle fascicles. This decrease in fascicle length was coupled with an increase in tendon length. These changes in muscle-tendon structure persisted throughout the first 14 wk following rupture. Deficits in peak plantarflexor torque were moderately correlated with decreased fascicle length at 120 degrees per second (R-2 = 0.424, P = 0.057) and strongly correlated with decreased fascicle length at 210 degrees per second (R-2 = 0.737, P = 0.003). However, increases in pennation angle did not explain functional deficits. These findings suggest that muscle-tendon structure is detrimentally affected following Achilles tendon rupture. Plantarflexor power deficits are positively correlated with the magnitude of reductions in fascicle length. Preserving muscle structure following Achilles tendon rupture should be a clinical priority to maintain plantarflexor kinetics.
   NEW & NOTEWORTHY In our study, we found that when the Achilles tendon ruptures due to excessive biomechanical loading, the neighboring skeletal muscle undergoes rapid changes in its configuration. The magnitude of this muscle remodeling explains the amount of ankle power loss demonstrated by these patients once their Achilles tendons are fully healed. These findings highlight the interconnected relationship between muscle and tendon. Isolated injuries to the tendon stimulate detrimental changes to the muscle, thereby limiting joint-level function.
C1 [Hullfish, Todd J.; O'Connor, Kathryn M.; Baxter, Josh R.] Univ Penn, Dept Orthopaed Surg, 3737 Market St,Suite 702, Philadelphia, PA 19104 USA.
RP Baxter, JR (通讯作者)，Univ Penn, Dept Orthopaed Surg, 3737 Market St,Suite 702, Philadelphia, PA 19104 USA.
EM josh.baxter@pennmedicine.upenn.edu
RI Baxter, Josh/N-4020-2013
OI Baxter, Josh/0000-0003-0269-2170
FU Thomas B. McCabe and Jeannette E. Laws McCabe Fund
FX This work was supported by The Thomas B. McCabe and Jeannette E. Laws
   McCabe Fund.
CR Baxter JR, 2019, J BIOMECH, V86, P27, DOI 10.1016/j.jbiomech.2019.01.035
   Baxter JR, 2018, J BIOMECH, V79, P238, DOI 10.1016/j.jbiomech.2018.08.016
   Bolsterlee B, 2016, J BIOMECH, V49, P1002, DOI 10.1016/j.jbiomech.2016.02.014
   Brorsson A, 2017, AM J SPORT MED, V45, P3060, DOI 10.1177/0363546517717698
   Bruggeman NB, 2004, CLIN ORTHOP RELAT R, P63, DOI 10.1097/01.blo.0000144475.05543.e7
   Cohen J., 1988, STAT POWER ANAL BEHA, VSecond second, DOI DOI 10.4324/9780203771587
   Crouzier M, 2018, J EXP BIOL, V221, DOI 10.1242/jeb.187260
   Drazan JF, 2019, BIORXIV, DOI [10.1101/547042, DOI 10.1101/547042]
   Drazan JF, 2019, PEERJ, V7, DOI 10.7717/peerj.7120
   Froberg A, 2009, AM J SPORT MED, V37, P1200, DOI 10.1177/0363546508330126
   Heroux ME, 2016, PHYSIOL REP, V4, DOI 10.14814/phy2.12947
   Hillin CD, 2019, J ORTHOP RES, V37, P562, DOI 10.1002/jor.24241
   Hullfish TJ, 2019, PEERJ, V7, DOI 10.7717/peerj.6788
   Huttunen TT, 2014, AM J SPORT MED, V42, P2419, DOI 10.1177/0363546514540599
   Kadel NJ, 2004, FOOT ANKLE INT, V25, P406, DOI 10.1177/107110070402500607
   Kangas J, 2007, AM J SPORT MED, V35, P59, DOI 10.1177/0363546506293255
   Lantto I, 2015, SCAND J MED SCI SPOR, V25, pE133, DOI 10.1111/sms.12253
   Morton RF, 2005, STUDY GUIDE EPIDEMIO
   NYSTROM B, 1983, ACTA ORTHOP SCAND, V54, P620, DOI 10.3109/17453678308992899
   Ochen Y, 2019, BMJ-BRIT MED J, V364, DOI 10.1136/bmj.k5120
   Peng WC, 2019, FOOT ANKLE INT, V40, P568, DOI 10.1177/1071100718822537
   Peng WC, 2017, CLIN BIOMECH, V43, P50, DOI 10.1016/j.clinbiomech.2017.02.002
   Renninger LTCH, 2016, FOOT ANKLE INT, V37, P269, DOI 10.1177/1071100715615322
   Salzano MQ, 2018, J BIOMECH, V80, P1, DOI 10.1016/j.jbiomech.2018.09.001
   Silbernagel KG, 2012, AM J SPORT MED, V40, P1564, DOI 10.1177/0363546512447926
   Tengman T, 2013, ORTHOP J SPORTS MED, V1, DOI 10.1177/2325967113504734
   WILLIAMS PE, 1978, J ANAT, V127, P459
   Willits K, 2010, J BONE JOINT SURG AM, V92A, P2767, DOI 10.2106/JBJS.I.01401
   Zellers JA, 2019, GAIT POSTURE, V70, P59, DOI 10.1016/j.gaitpost.2019.02.027
   Zellers JA, 2019, J ORTHOP RES, V37, P933, DOI 10.1002/jor.24260
   Zellers JA, 2018, FOOT ANKLE INT, V39, P343, DOI 10.1177/1071100717742372
NR 31
TC 7
Z9 7
U1 1
U2 4
PU AMER PHYSIOLOGICAL SOC
PI BETHESDA
PA 9650 ROCKVILLE PIKE, BETHESDA, MD 20814 USA
SN 8750-7587
EI 1522-1601
J9 J APPL PHYSIOL
JI J. Appl. Physiol.
PD OCT
PY 2019
VL 127
IS 4
BP 1005
EP 1011
DI 10.1152/japplphysiol.00255.2019
PG 7
WC Physiology; Sport Sciences
WE Science Citation Index Expanded (SCI-EXPANDED)
SC Physiology; Sport Sciences
GA JE7GQ
UT WOS:000490858000011
PM 31589091
OA Bronze
DA 2022-07-22
ER

PT J
AU Chen, XM
   Cui, LG
   He, P
   Shen, WW
   Qian, YJ
   Wang, JR
AF Chen, Xiang-Mei
   Cui, Li-Gang
   He, Ping
   Shen, Wei-Wei
   Qian, Ya-Jun
   Wang, Jin-Rui
TI Shear Wave Elastographic Characterization of Normal and Torn Achilles
   Tendons A Pilot Study
SO JOURNAL OF ULTRASOUND IN MEDICINE
LA English
DT Article
DE Achilles tendon; rupture; shear wave elastography; sonography
ID ULTRASOUND ELASTOGRAPHY; SONOELASTOGRAPHY; ELASTICITY; DIAGNOSIS
AB Objectives-The purpose of this study was to investigate the feasibility of using quantitative shear wave elastography for assessing the functional integrity of the Achilles tendon and to summarize the changes in elasticity of ruptured Achilles tendons in comparison with normal controls.
   Methods-Thirty-six normal and 14 ruptured Achilles tendons were examined with shear wave elastography coupled with a linear array transducer (4-15 MHz). The elasticity value value of each Achilles tendon in a longitudinal view was measured.
   Results-The mean elasticity value +/- SD for the normal Achilles tendons was 291.91 +/- 4.38kPa (note that there are saturated measurement phenomena for the normal Achilles tendon, so the actual value will be >300 kPa), whereas the ruptured Achilles tendons had an elasticity value of 56.48 +/- 68.59 kPa. A statistically significant difference was found in relation to the findings in healthy volunteers (P = .006).
   Conclusions-Our results suggest that shear wave elastography is a valuable tool that can provide complementary biomechanical information for evaluating the function of the Achilles tendon.
C1 [Chen, Xiang-Mei; Cui, Li-Gang; He, Ping; Shen, Wei-Wei; Qian, Ya-Jun; Wang, Jin-Rui] Peking Univ, Hosp 3, Dept Ultrasonog, Beijing 100191, Peoples R China.
RP Cui, LG (通讯作者)，Peking Univ, Hosp 3, Dept Ultrasonog, Beijing 100191, Peoples R China.
EM cuijuegang@126.com
CR Alam F, 2008, AM J ROENTGENOL, V191, P604, DOI 10.2214/AJR.07.3401
   Arda K, 2011, AM J ROENTGENOL, V197, P532, DOI 10.2214/AJR.10.5449
   Athanasiou A, 2010, RADIOLOGY, V256, P297, DOI 10.1148/radiol.10090385
   BAVU E, 2010, J HEPATOL S1, V52, pS166
   Bleakney RR, 2002, CLIN J SPORT MED, V12, P273, DOI 10.1097/00042752-200209000-00003
   De Zordo T, 2010, ULTRASCHALL MED, V31, P394, DOI 10.1055/s-0028-1109809
   Drakonaki EE, 2009, CLIN RADIOL, V64, P1196, DOI 10.1016/j.crad.2009.08.006
   Garra Brian Stephen, 2007, Ultrasound Q, V23, P255, DOI 10.1097/ruq.0b013e31815b7ed6
   Hoffmeister BK, 1996, J ACOUST SOC AM, V100, P3933, DOI 10.1121/1.417246
   Klauser AS, 2010, SEMIN MUSCULOSKEL R, V14, P323, DOI 10.1055/s-0030-1254521
   Kuo PL, 1999, ULTRASOUND MED BIOL, V25, P1241, DOI 10.1016/S0301-5629(99)00078-2
   Kuo PL, 2001, ULTRASOUND MED BIOL, V27, P1275, DOI 10.1016/S0301-5629(01)00442-2
   Lyshchik A, 2005, RADIOLOGY, V237, P202, DOI 10.1148/radiol.2363041248
   Maganaris CN, 1999, J PHYSIOL-LONDON, V521, P307, DOI 10.1111/j.1469-7793.1999.00307.x
   Maganaris CN, 2002, J BIOMECH, V35, P1639, DOI 10.1016/S0021-9290(02)00240-3
   Moller M, 2002, KNEE SURG SPORT TR A, V10, P49, DOI 10.1007/s001670100245
   OPHIR J, 1991, ULTRASONIC IMAGING, V13, P111, DOI 10.1016/0161-7346(91)90079-W
   Pallwein L, 2008, EUR J RADIOL, V65, P304, DOI 10.1016/j.ejrad.2007.03.032
   Sconfienza LM, 2010, CLIN EXP RHEUMATOL, V28, P373
   Wang JHC, 2006, J BIOMECH, V39, P1563, DOI 10.1016/j.jbiomech.2005.05.011
   Wojcinski S, 2010, ULTRASCHALL MED, V31, P484, DOI 10.1055/s-0029-1245282
NR 21
TC 102
Z9 112
U1 0
U2 26
PU AMER INST ULTRASOUND MEDICINE
PI LAUREL
PA SUBSCRIPTION DEPT, 14750 SWEITZER LANE, STE 100, LAUREL, MD 20707-5906
   USA
SN 0278-4297
J9 J ULTRAS MED
JI J. Ultrasound Med.
PD MAR
PY 2013
VL 32
IS 3
BP 449
EP 455
DI 10.7863/jum.2013.32.3.449
PG 7
WC Acoustics; Radiology, Nuclear Medicine & Medical Imaging
WE Science Citation Index Expanded (SCI-EXPANDED)
SC Acoustics; Radiology, Nuclear Medicine & Medical Imaging
GA 102HO
UT WOS:000315835900009
PM 23443185
DA 2022-07-22
ER

PT J
AU Mattila, VM
   Huttunen, TT
   Haapasalo, H
   Sillanpaa, P
   Malmivaara, A
   Pihlajamaki, H
AF Mattila, Ville M.
   Huttunen, Tuomas T.
   Haapasalo, Heidi
   Sillanpaa, Petri
   Malmivaara, Antti
   Pihlajamaki, Harri
TI Declining incidence of surgery for Achilles tendon rupture follows
   publication of major RCTs: evidence-influenced change evident using the
   Finnish registry study
SO BRITISH JOURNAL OF SPORTS MEDICINE
LA English
DT Article
ID NONOPERATIVE TREATMENT; METAANALYSIS; TRIALS
AB Objectives Acute Achilles tendon ruptures are common among highly active people. Recently published studies have provided increasing evidence to support non-surgical treatment. This study aimed to assess the incidence trends of surgically treated, acute Achilles tendon ruptures. Our hypothesis, based on the recent literature showing no difference in functional results between surgical and non-surgical treatment, was that the incidence of surgery would be declining.
   Methods We conducted a nationwide hospital register-based study. All patients 18 years of age or older with a diagnosis of acute Achilles tendon injury, and treated with Achilles tendon repair from 1987 to 2011 in Finland were included in the study.
   Results During the 25-year study period in Finland, a total of 15 252 patients received surgical treatment for an acute Achilles tendon rupture. The incidence of surgical treatment of acute Achilles tendon rupture in men was 11.1/100 000 person-years in 1987 and 20.5/100 000 person-years in 2011. The corresponding figures in women were 2.5/100 000 person-years in 1987 and 4.2/100 000 person-years in 2011. The highest rates occurred in 2008 in men and 2007 in women, and since then the decrease has been 42% in men and 55% in women.
   Conclusions During the past few years, the rate of surgically treated acute Achilles tendon ruptures has declined remarkably. The findings of the present study indicate that orthopaedic surgeons have chosen more often non-surgical treatment option for acute Achilles ruptures. This can be considered as an example, how high-quality scientific evidence can lead to a rapid change in clinical practice.
C1 [Mattila, Ville M.; Huttunen, Tuomas T.; Haapasalo, Heidi; Sillanpaa, Petri] Tampere Univ Hosp, Dept Orthopaed & Trauma Surg, Tampere 12838, Finland.
   [Mattila, Ville M.] Karolinska Inst, Dept Clin Sci Intervent & Technol, Stockholm, Sweden.
   [Mattila, Ville M.] Karolinska Inst, Div Orthoped & Biotechnol, Stockholm, Sweden.
   [Mattila, Ville M.] Karolinska Univ, Huddinge Hosp, Dept Orthoped, Stockholm, Sweden.
   [Huttunen, Tuomas T.] Valkeakoski Reg Hosp, Dept Anesthesia & Perioperat Care, Valkeakoski, Finland.
   [Malmivaara, Antti] Natl Inst Hlth & Welf, Ctr Hlth & Social Econ, Helsinki, Finland.
   [Pihlajamaki, Harri] Seinajoki Cent Hosp, Dept Orthopaed & Trauma Surg, Seinajoki, Finland.
   [Pihlajamaki, Harri] Univ Tampere, Seinajoki, Finland.
RP Mattila, VM (通讯作者)，Tampere Univ Hosp, Dept Orthopaed & Trauma Surg, Tampere 12838, Finland.
EM ville.mattila@uta.fi
OI Pihlajamaki, Harri/0000-0002-2998-0773; Huttunen,
   Tuomas/0000-0001-7884-7533
CR CETTI R, 1993, AM J SPORT MED, V21, P791, DOI 10.1177/036354659302100606
   Crawford J, 2004, COCHRANE DB SYST REV, DOI DOI 10.1002/14651858.CD003674.PUB2
   Houshian S, 1998, INJURY, V29, P651, DOI 10.1016/S0020-1383(98)00147-8
   Keskimaki I., 1991, INT J HLTH SCI, V2, P15
   Khan RJK, 2005, J BONE JOINT SURG AM, V87A, P2202, DOI 10.2106/JBJS.D.03049
   Lantto I, 2011, SOT, V34, P177
   Leppilahti J, 1996, ACTA ORTHOP SCAND, V67, P277, DOI 10.3109/17453679608994688
   Levi N, 1997, INJURY, V28, P311, DOI 10.1016/S0020-1383(96)00200-8
   Maffulli N, 2011, FOOT ANKLE INT, V32, P9, DOI 10.3113/FAI.2011.0009
   Mattila VM, 2008, INJURY, V39, P1373, DOI 10.1016/j.injury.2008.05.007
   Metz R, 2008, AM J SPORT MED, V36, P1688, DOI 10.1177/0363546508319312
   Moller M, 2001, J BONE JOINT SURG BR, V83B, P843
   Nilsson-Helander K, 2010, AM J SPORT MED, V38, P2186, DOI 10.1177/0363546510376052
   NISTOR L, 1981, J BONE JOINT SURG AM, V63, P394, DOI 10.2106/00004623-198163030-00012
   Nyyssonen T, 2008, SCAND J SURG, V97, P272, DOI 10.1177/145749690809700312
   Salmela R, 1987, HOSPITAL, V49, P480
   Soroceanu A, 2012, J BONE JOINT SURG AM, V94A, P2136, DOI 10.2106/JBJS.K.00917
   Suchak AA, 2008, J BONE JOINT SURG AM, V90A, P1876, DOI 10.2106/JBJS.G.01242
   Wallace RGH, 2011, J BONE JOINT SURG BR, V93B, P1362, DOI 10.1302/0301-620X.93B10.26187
   WHO, 1994, 25 NORDIC SEMINAR DE, V10th
   Willits K, 2010, J BONE JOINT SURG AM, V92A, P2767, DOI 10.2106/JBJS.I.01401
NR 21
TC 49
Z9 50
U1 0
U2 8
PU BMJ PUBLISHING GROUP
PI LONDON
PA BRITISH MED ASSOC HOUSE, TAVISTOCK SQUARE, LONDON WC1H 9JR, ENGLAND
SN 0306-3674
EI 1473-0480
J9 BRIT J SPORT MED
JI Br. J. Sports Med.
PD AUG
PY 2015
VL 49
IS 16
BP 1084
EP U83
DI 10.1136/bjsports-2013-092756
PG 4
WC Sport Sciences
WE Science Citation Index Expanded (SCI-EXPANDED)
SC Sport Sciences
GA CN8WO
UT WOS:000358726600013
PM 24128757
DA 2022-07-22
ER

PT J
AU Counihan, M
   Leahy, T
   Nuss, C
   Newton, J
   Mohanty, S
   Soslowsky, LJ
   Farber, D
AF Counihan, Matthew
   Leahy, Thomas
   Nuss, Courtney
   Newton, Joseph
   Mohanty, Sarthak
   Soslowsky, Louis J.
   Farber, Daniel
TI Limited Scar Resection for Chronic Achilles Tendon Repair Use of a Rat
   Model
SO AMERICAN JOURNAL OF SPORTS MEDICINE
LA English
DT Article
DE Achilles tendon; biomechanics of tendon; biology of tendon; ankle
ID NEGLECTED RUPTURE; RECONSTRUCTION; INFERIOR; TENDINOPATHY; MANAGEMENT;
   INJURY; TISSUE
AB Background: Achilles tendon rupture diagnosis is frequently missed, leading to the development of a chronic rupture that requires surgical intervention to remove scar tissue and return the elongated Achilles tendon to appropriate functional length. The limited scar resection (LSR) intervention strategy may provide an advantage over other techniques, as it is less invasive and nondestructive to other tissues, although there is little evidence comparing outcomes between intervention strategies.
   Hypothesis: The LSR technique would be a viable treatment option for chronic Achilles tendon ruptures and would perform comparably with a more clinically accepted procedure, the gastrocnemius fascial turndown (GFT), in postintervention functional outcome measures and tendon mechanical and histological properties.
   Study Design: Controlled laboratory study.
   Methods: Chronic Achilles tendon ruptures were induced in the right hindlimb of Sprague-Dawley rats by Achilles tendon transection without repair, immobilization in dorsiflexion, and 5 weeks of cage activity. Animals were randomly divided between the intervention strategy groups (LSR and GFT), received 1 week of immobilization in plantarflexion, and were sacrificed at 3 or 6 weeks postintervention. In vivo functional outcome measures (gait kinetics, passive joint function, tendon vascular perfusion) were quantified during healing, and tendon mechanical and histological properties were assessed postsacrifice.
   Results: When compared with the GFT, the LSR technique elicited a faster return to baseline in gait kinetics, although there were few differences between groups or with healing time in other functional outcome measures (passive joint function and vascular perfusion). Quasi-static mechanical properties were improved with healing in both surgical intervention groups, although only the LSR group showed an improvement in fatigue properties between 3 and 6 weeks postintervention. Histological properties were similar between intervention strategies, except for decreased cellularity in the LSR group at 6 weeks postintervention.
   Conclusion: The LSR technique is a viable surgical intervention strategy for a chronic Achilles tendon rupture in a rodent model, and it performs similarly, if not better, when directly compared with a more clinically accepted surgery, the GFT. Clinical Relevance: This study supports the increased clinical use of the LSR technique for treating chronic Achilles tendon rupture cases.
C1 [Counihan, Matthew; Leahy, Thomas; Nuss, Courtney; Newton, Joseph; Mohanty, Sarthak; Soslowsky, Louis J.; Farber, Daniel] Univ Penn, Philadelphia, PA 19104 USA.
   [Counihan, Matthew; Leahy, Thomas; Nuss, Courtney; Newton, Joseph; Mohanty, Sarthak; Soslowsky, Louis J.; Farber, Daniel] Univ Penn, McKay Orthopaed Lab, Philadelphia, PA 19104 USA.
   [Leahy, Thomas; Newton, Joseph; Soslowsky, Louis J.] Univ Penn, Dept Bioengn, Philadelphia, PA 19104 USA.
RP Soslowsky, LJ (通讯作者)，Univ Penn, McKay Orthopaed Res Lab, 307A Stemmler Hall,3450 Hamilton Walk, Philadelphia, PA 19104 USA.
EM soslowsk@upenn.edu
CR [Anonymous], VEVO CQ SOFTWARE FUJ
   BARNES MJ, 1986, J BONE JOINT SURG BR, V68, P121, DOI 10.1302/0301-620X.68B1.3941128
   Chang KV, 2012, J ULTRAS MED, V31, P737
   Cheema AN, 2019, J ORTHOP RES, V37, P94, DOI 10.1002/jor.24167
   Chen MC, 2020, J BIOMECH ENG-T ASME, V142, DOI 10.1115/1.4046864
   Freedman BR, 2017, J AM ACAD ORTHOP SUR, V25, P635, DOI 10.5435/JAAOS-D-16-00620
   Freedman BR, 2017, J BIOMECH, V56, P55, DOI 10.1016/j.jbiomech.2017.02.030
   Freedman BR, 2016, J ORTHOP RES, V34, P2172, DOI 10.1002/jor.23253
   Fryhofer GW, 2016, J APPL PHYSIOL, V121, P1106, DOI 10.1152/japplphysiol.00620.2016
   Gittings Daniel, 2019, Foot Ankle Orthop, V4, DOI 10.1177/2473011419842529
   Gulati V, 2015, WORLD J ORTHOP, V6, P380, DOI 10.5312/wjo.v6.i4.380
   Gwynne-Jones DP, 2011, FOOT ANKLE INT, V32, P337, DOI 10.3113/FAI.2011.0337
   Hillin CD, 2019, J ORTHOP RES, V37, P562, DOI 10.1002/jor.24241
   HOWARD CB, 1984, J BONE JOINT SURG BR, V66, P206, DOI 10.1302/0301-620X.66B2.6707056
   Huegel J, 2020, J ORTHOP RES, V38, P70, DOI 10.1002/jor.24487
   Jennings AG, 2002, J BONE JOINT SURG BR, V84B, P361, DOI 10.1302/0301-620X.84B3.11559
   Maffulli N, 2004, J ROY SOC MED, V97, P472, DOI 10.1258/jrsm.97.10.472
   Maffulli N, 1998, AM J SPORT MED, V26, P266, DOI 10.1177/03635465980260021801
   Maffulli N, 1999, CLIN J SPORT MED, V9, P157, DOI 10.1097/00042752-199907000-00007
   Maffulli Nicola, 2007, Foot Ankle Clin, V12, P583, DOI 10.1016/j.fcl.2007.07.007
   MANN RA, 1991, J BONE JOINT SURG AM, V73A, P214, DOI 10.2106/00004623-199173020-00009
   Needles A, 2010, ULTRASOUND MED BIOL, V36, P2097, DOI 10.1016/j.ultrasmedbio.2010.08.012
   OZAKI J, 1989, CLIN ORTHOP RELAT R, P204
   Pardes AM, 2017, J BIOMECH, V60, P30, DOI 10.1016/j.jbiomech.2017.06.008
   Pardes AM, 2016, ANN BIOMED ENG, V44, P2901, DOI 10.1007/s10439-016-1635-1
   PARKER RG, 1979, J AM POD ASSOC, V69, P548, DOI 10.7547/87507315-69-9-548
   PARSONS JR, 1989, FOOT ANKLE, V9, P179, DOI 10.1177/107110078900900406
   Peltz CD, 2009, J ORTHOP RES, V27, P416, DOI 10.1002/jor.20770
   Porter DA, 1997, FOOT ANKLE INT, V18, P557, DOI 10.1177/107110079701800905
   Raikin SM, 2013, FOOT ANKLE INT, V34, P475, DOI 10.1177/1071100713477621
   RUSH JH, 1980, AUST NZ J SURG, V50, P420, DOI 10.1111/j.1445-2197.1980.tb04156.x
   Sarver JJ, 2008, J SHOULDER ELB SURG, V17, p108S, DOI 10.1016/j.jse.2007.08.004
   Sarver JJ, 2010, J BIOMECH, V43, P778, DOI 10.1016/j.jbiomech.2009.10.031
   Sengupta P, 2013, INT J PREVENTIVE MED, V4, P624
   Tucker JJ, 2016, J ORTHOP RES, V34, P2009, DOI 10.1002/jor.23225
   TURCO VJ, 1987, FOOT ANKLE, V7, P253, DOI 10.1177/107110078700700407
   Wapner KL, 1994, OPER TECH ORTHOP, V4, P132, DOI [10.1016/S1048-6666(06)80123-0, DOI 10.1016/S1048-6666(06)80123-0]
   Wilcox DK, 2000, FOOT ANKLE INT, V21, P1004, DOI 10.1177/107110070002101204
   Yasuda T, 2007, AM J SPORT MED, V35, P582, DOI 10.1177/0363546506295939
   Yasuda T, 2016, J BONE JOINT SURG AM, V98, P1168, DOI 10.2106/JBJS.15.00865
NR 40
TC 1
Z9 1
U1 0
U2 1
PU SAGE PUBLICATIONS INC
PI THOUSAND OAKS
PA 2455 TELLER RD, THOUSAND OAKS, CA 91320 USA
SN 0363-5465
EI 1552-3365
J9 AM J SPORT MED
JI Am. J. Sports Med.
PD AUG
PY 2021
VL 49
IS 10
BP 2707
EP 2715
DI 10.1177/03635465211023096
PG 9
WC Orthopedics; Sport Sciences
WE Science Citation Index Expanded (SCI-EXPANDED)
SC Orthopedics; Sport Sciences
GA TY5RC
UT WOS:000683840500020
PM 34197235
DA 2022-07-22
ER

PT J
AU Shane, AM
   Reeves, CL
   Nguyen, GB
   Sebag, JA
AF Shane, Amber M.
   Reeves, Christopher L.
   Nguyen, Garrett B.
   Sebag, Joshua A.
TI Revision Surgery for the Achilles Tendon
SO CLINICS IN PODIATRIC MEDICINE AND SURGERY
LA English
DT Article
DE Revisional Achilles tendon; Reconstruction; Augmentation; Gastrocnemius
   lengthening; Turndown flap; Tendon transfer; Autografts; Allografts
ID FLEXOR HALLUCIS LONGUS; BIOMECHANICAL EVALUATION; NONOPERATIVE
   TREATMENT; CHRONIC RUPTURES; RECONSTRUCTION; RERUPTURE; REPAIR;
   TENDINOPATHY; TENDINOSIS; ALLOGRAFT
AB Treatment of Achilles tendon ruptures may be surgical or nonsurgical depending on health, history, age, acuity, and severity of the injury. With chronic or revisional injuries, the best method often requires an open repair with reconstructive soft tissue procedures. Revision surgery can be challenging because of the complexity involving tendinous deficits with nonviable and friable tissue. Surgical treatment is based on tendon approximation, size of the defect, tendon integrity, and functional demands. The goal is to restore anatomic and physiologic tension, provide adequate strength for proper ambulation, optimize functional return to activity, decrease pain, and decrease complications.
C1 [Shane, Amber M.; Reeves, Christopher L.] Upperline Hlth, Advent Hlth East Orlando Podiatr Surg Residency, Orlando Foot & Ankle Clin, 250 North Alafaya Trail Suite 115, Orlando, FL 32828 USA.
   [Nguyen, Garrett B.; Sebag, Joshua A.] Advent Hlth East Orlando Podiatr Surg Residency, Dept Podiatr Surg, 250 North Alafaya Trail Suite 1115, Orlando, FL 32828 USA.
RP Shane, AM (通讯作者)，Upperline Hlth, Advent Hlth East Orlando Podiatr Surg Residency, Orlando Foot & Ankle Clin, 250 North Alafaya Trail Suite 115, Orlando, FL 32828 USA.
EM Ashane@orlandofoot.com
CR ABRAHAM E, 1975, J BONE JOINT SURG AM, VA 57, P253, DOI 10.2106/00004623-197557020-00019
   Ahmad CS, 2004, AM J SPORT MED, V32, P635, DOI 10.1177/0363546503261714
   Akgun Umut, 2006, Acta Orthop Traumatol Turc, V40, P228
   BOSWORTH DM, 1956, J BONE JOINT SURG AM, V38, P111, DOI 10.2106/00004623-195638010-00011
   CETTI R, 1983, CLIN ORTHOP RELAT R, P204
   Chan JY, 2013, FOOT ANKLE INT, V34, P898, DOI 10.1177/1071100712473273
   Cienfuegos A, 2013, J FOOT ANKLE SURG, V52, P95, DOI 10.1053/j.jfas.2012.06.006
   COPELAND SA, 1990, ANN ROY COLL SURG, V72, P270
   Coull R, 2003, FOOT ANKLE INT, V24, P931, DOI 10.1177/107110070302401211
   Deese J Melvin, 2015, J Surg Orthop Adv, V24, P75
   Doral MN, 2010, KNEE SURG SPORT TR A, V18, P638, DOI 10.1007/s00167-010-1083-7
   Elias I, 2007, FOOT ANKLE INT, V28, P1238, DOI 10.3113/FAI.2007.1238
   Elias I, 2009, FOOT ANKLE INT, V30, P197, DOI 10.3113/FAI.2009.0197
   FRENETTE JP, 1977, J BONE JOINT SURG AM, V59, P673, DOI 10.2106/00004623-197759050-00015
   Gallant G G, 1995, Am J Orthop (Belle Mead NJ), V24, P257
   Goss DA, 2019, FOOT ANKLE INT, V40, P1214, DOI 10.1177/1071100719858074
   Guclu B, 2016, FOOT ANKLE INT, V37, P737, DOI 10.1177/1071100716642753
   Hahn F, 2008, FOOT ANKLE INT, V29, P794, DOI 10.3113/FAI.2008.0794
   Hanada M, 2011, CLINICS PRACT, V1, P299, DOI 10.4081/cp.2011.e134
   Hersh BL, 2002, J AM PODIAT MED ASSN, V92, P355, DOI 10.7547/87507315-92-6-355
   Herzenberg JE, 2007, FOOT ANKLE INT, V28, P1154, DOI 10.3113/FAI.2007.1154
   Hong CK, 2018, J ORTHOP SURG-HONG K, V26, DOI 10.1177/2309499018799514
   Ingvar J, 2005, ACTA ORTHOP, V76, P597, DOI 10.1080/17453670510041619
   Jain P, 2014, ADV ORTHOP SURG, V1, P1
   Jildeh TR, 2018, ORTHOP J SPORTS MED, V6, DOI 10.1177/2325967118774302
   Kelahmetoglu O, 2018, J HAND MICROSURG, V10, P49, DOI 10.1055/s-0037-1608745
   Khiami F, 2013, ORTHOP TRAUMATOL-SUR, V99, P585, DOI 10.1016/j.otsr.2013.03.021
   Kissel C G, 1994, J Foot Ankle Surg, V33, P46
   Krahe MA, 2009, FOOT ANKLE CLIN, V14, P247, DOI 10.1016/j.fcl.2009.04.003
   Lee YS, 2005, ORTHOPEDICS, V28, P647, DOI 10.3928/0147-7447-20050701-10
   Lepow Gary M, 2006, J Foot Ankle Surg, V45, P351, DOI 10.1053/j.jfas.2006.06.004
   Lin JL, 2009, FOOT ANKLE CLIN, V14, P729, DOI 10.1016/j.fcl.2009.08.007
   LINDHOLM A, 1959, Acta Chir Scand, V117, P261
   Maffulli N, 2000, AM J SPORT MED, V28, P499, DOI 10.1177/03635465000280040901
   Maffulli N, 2004, J ROY SOC MED, V97, P472, DOI 10.1258/jrsm.97.10.472
   Maffulli N, 2014, OPER ORTHOP TRAUMATO, V26, P513, DOI 10.1007/s00064-012-0228-x
   Maganaris CN, 2008, DISABIL REHABIL, V30, P1542, DOI 10.1080/09638280701785494
   MAHONEY PG, 1981, ANN RHEUM DIS, V40, P416, DOI 10.1136/ard.40.4.416
   MANN RA, 1991, J BONE JOINT SURG AM, V73A, P214, DOI 10.2106/00004623-199173020-00009
   Matles A L, 1975, Bull Hosp Joint Dis, V36, P48
   Mazzocca AD, 2007, AM J SPORT MED, V35, P252, DOI 10.1177/0363546506294854
   McKeon BP, 2006, ARTHROSCOPY, V22, P33, DOI 10.1016/j.arthro.2005.10.008
   Myerson MS, 1999, INISTR COURSE LECT, V48, P226
   Nagakiran KV, 2019, INT J RES ORTHOPAEDI, V5, P264
   Nilsson-Helander K, 2008, KNEE SURG SPORT TR A, V16, P614, DOI 10.1007/s00167-008-0492-3
   Nyyssonen T, 2018, SCAND J MED SCI SPOR, V28, P2625, DOI 10.1111/sms.13281
   O'Brien Moira, 2005, Foot Ankle Clin, V10, P225, DOI 10.1016/j.fcl.2005.01.011
   OBRIEN T, 1984, J BONE JOINT SURG AM, V66A, P1099, DOI 10.2106/00004623-198466070-00018
   Ofili KP, 2016, J FOOT ANKLE SURG, V55, P1245, DOI 10.1053/j.jfas.2016.01.001
   Ozan Firat, 2017, J Clin Med Res, V9, P573, DOI 10.14740/jocmr2943w
   Padanilam TG, 2009, FOOT ANKLE CLIN, V14, P711, DOI 10.1016/j.fcl.2009.08.001
   Patil SSD, 2014, FOOT ANKLE INT, V35, P699, DOI 10.1177/1071100714531228
   Petersen W, 2004, CLIN ORTHOP RELAT R, P286, DOI 10.1097/01.blo.0000126228.78639.76
   Pintore E, 2001, J TRAUMA, V50, P71, DOI 10.1097/00005373-200101000-00013
   Reito A, 2018, FOOT ANKLE INT, V39, P694, DOI 10.1177/1071100717754042
   Sadek AF, 2015, J ORTHOP SURG RES, V10, DOI 10.1186/s13018-015-0256-y
   SILVER RL, 1985, J BONE JOINT SURG BR, V67, P432, DOI 10.1302/0301-620X.67B3.3997956
   STROCCHI R, 1991, FOOT ANKLE, V12, P100, DOI 10.1177/107110079101200207
   Sutton KM, 2010, J AM ACAD ORTHOP SUR, V18, P139, DOI 10.5435/00124635-201003000-00003
   Tallon C, 2001, MED SCI SPORT EXER, V33, P1983, DOI 10.1097/00005768-200112000-00002
   Thevendran G, 2013, Musculoskelet Surg, V97, P9, DOI 10.1007/s12306-013-0251-6
   THOMPSON T C, 1962, Acta Orthop Scand, V32, P461, DOI 10.3109/17453676208989608
   Us AK, 1997, ARCH ORTHOP TRAUM SU, V116, P408
   Vega J, 2018, FOOT ANKLE INT, V39, P1464, DOI 10.1177/1071100718793172
   Wallace RGH, 2011, J BONE JOINT SURG BR, V93B, P1362, DOI 10.1302/0301-620X.93B10.26187
   Wang CC, 2009, J TRAUMA, V67, P1109, DOI 10.1097/TA.0b013e3181a73f02
   Wapner K L, 1993, Foot Ankle, V14, P443
   White KL, 2010, ARTHROSCOPY, V26, P1662, DOI 10.1016/j.arthro.2010.05.013
   Wijdicks CA, 2010, KNEE SURG SPORT TR A, V18, P1532, DOI 10.1007/s00167-010-1127-z
   Yasuda T, 2016, J BONE JOINT SURG AM, V98, P1168, DOI 10.2106/JBJS.15.00865
   Zayda AI, 2018, BIOMED J SCI TECH RE, V2, P2912
NR 71
TC 0
Z9 0
U1 2
U2 3
PU W B SAUNDERS CO-ELSEVIER INC
PI PHILADELPHIA
PA 1600 JOHN F KENNEDY BOULEVARD, STE 1800, PHILADELPHIA, PA 19103-2899 USA
SN 0891-8422
EI 1558-2302
J9 CLIN PODIATR MED SUR
JI Clin. Podiatr. Med. Surg.
PD JUL
PY 2020
VL 37
IS 3
BP 553
EP +
DI 10.1016/j.cpm.2020.03.005
PG 17
WC Orthopedics
WE Science Citation Index Expanded (SCI-EXPANDED)
SC Orthopedics
GA LT6KF
UT WOS:000537176900012
PM 32471618
DA 2022-07-22
ER

PT J
AU Miadlikowski, M
   Kaluza, K
   Domzalski, M
AF Miadlikowski, Michel
   Kaluza, Konrad
   Domzalski, Marcin
TI Snapping Plantaris Tendon as Cause of Pain in an Amateur Runner A Case
   Report
SO JOURNAL OF THE AMERICAN PODIATRIC MEDICAL ASSOCIATION
LA English
DT Article
ID RUPTURE
AB A rare case of painful snapping around the Achilles tendon without reported injury in a healthy 30-year-old male amateur runner is reported. The plantaris tendon was firmly attached to the Achilles tendon by adhesions, and its movement was restricted and impaired. All adhesions were removed and the plantaris tendon was liberated during surgery, with an excellent outcome. This case showed that previously described removal of the snapping tendon is not necessary because liberation of the plantaris tendon restored function of both the plantaris and the Achilles tendons.
C1 [Miadlikowski, Michel; Kaluza, Konrad] Med Univ Lodz, Lodz, Poland.
   [Domzalski, Marcin] Med Univ Lodz, Vet Mem Teaching Hosp, Dept Orthoped & Trauma, Zeromskiego Str 113, PL-90549 Lodz, Poland.
RP Domzalski, M (通讯作者)，Med Univ Lodz, Vet Mem Teaching Hosp, Dept Orthoped & Trauma, Zeromskiego Str 113, PL-90549 Lodz, Poland.
EM marcin.domzalski@umed.lodz.pl
CR Alfredson H, 2011, BRIT J SPORT MED, V45, P1023, DOI 10.1136/bjsports-2011-090217
   [Anonymous], 2014, CLIN ORIENTED ANATOM
   Bianchi S, 2011, SKELETAL RADIOL, V40, P891, DOI 10.1007/s00256-010-1076-0
   Calder JDF, 2015, BRIT J SPORT MED, V49, P1532, DOI 10.1136/bjsports-2014-093827
   Cook JL, 2012, BRIT J SPORT MED, V46, P163, DOI 10.1136/bjsports-2011-090414
   Delgado GJ, 2002, RADIOLOGY, V224, P112, DOI 10.1148/radiol.2241011067
   Han FC, 2014, FOOT ANKLE INT, V35, P1358, DOI 10.1177/1071100714549048
   Harmon KJ, 2006, CLIN J SPORT MED, V16, P361, DOI 10.1097/00042752-200607000-00015
   JAYASREE K, 2016, J EVOLUTION MED DENT, V5, P2792
   ROHILLA S, 2013, BMJ CASE REP
   SEVERANCE HW, 1982, J BONE JOINT SURG AM, V64, P1387, DOI 10.2106/00004623-198264090-00021
   Smith J, 2017, PM&R, V9, P691, DOI 10.1016/j.pmrj.2016.10.013
   Steenstra Ferry, 2006, Foot Ankle Clin, V11, P429, DOI 10.1016/j.fcl.2006.02.001
NR 13
TC 1
Z9 1
U1 0
U2 0
PU AMER PODIATRIC MED ASSOC
PI BETHESDA
PA 9312 OLD GEORGETOWN ROAD, BETHESDA, MD 20814-1621 USA
SN 8750-7315
EI 1930-8264
J9 J AM PODIAT MED ASSN
JI J. Am. Podiatr. Med. Assoc.
PD NOV-DEC
PY 2020
VL 110
IS 6
PG 4
WC Orthopedics
WE Science Citation Index Expanded (SCI-EXPANDED)
SC Orthopedics
GA RP8PP
UT WOS:000641985500020
DA 2022-07-22
ER

PT J
AU Wang, ZM
   Wang, QA
   Tang, H
   Kang, YF
AF Wang, Zimin
   Wang, Qian
   Tang, Hao
   Kang, Yifan
TI Extensor mechanism repair and reconstruction using Achilles tendon
   allograft after bilateral patellar tendon rupture in a patient with
   rheumatoid arthritis
SO KNEE SURGERY SPORTS TRAUMATOLOGY ARTHROSCOPY
LA English
DT Article
DE Patellar tendon rupture; Allograft Achilles tendon; Rheumatoid arthritis
ID FIXATION
AB Here we report spontaneous rupture of the bilateral patellar tendons in a 49-year-old man with rheumatoid arthritis. The ruptured tendons were sutured via bone tunnels using Krackow stitch, and the extensor mechanism of the knee was reinforced with three bundles of allograft Achilles tendons. The patient recovered well after operation. He could actively flex, extend the knees and carry out full-weight-bearing activities, and he resumed the former employment 6 months after the operation. Follow-up showed that the patient had satisfactory range of motion of the knees 1 year later.
C1 [Wang, Zimin; Wang, Qian; Tang, Hao; Kang, Yifan] Second Mil Med Univ, Changhai Hosp, Dept Orthopaed Surg, Shanghai 200433, Peoples R China.
RP Kang, YF (通讯作者)，Second Mil Med Univ, Changhai Hosp, Dept Orthopaed Surg, Shanghai 200433, Peoples R China.
EM DrKangyifan@gmail.com
CR CARSON WG, 1985, CLIN SPORT MED, V4, P231
   Crossett LS, 2002, J BONE JOINT SURG AM, V84A, P1354, DOI 10.2106/00004623-200208000-00011
   EMERSON RH, 1990, CLIN ORTHOP RELAT R, P154
   EMERSON RH, 1994, CLIN ORTHOP RELAT R, P79
   Falconiero RP, 1996, ARTHROSCOPY, V12, P623, DOI 10.1016/S0749-8063(96)90204-2
   Ho HM, 2003, KNEE SURG SPORT TR A, V11, P105, DOI 10.1007/s00167-002-0332-9
   INSALL J, 1971, RADIOLOGY, V101, P101, DOI 10.1148/101.1.101
   Jarvela T, 2005, KNEE, V12, P139, DOI 10.1016/j.knee.2004.05.005
   Kothari P, 1998, ANN ROY COLL SURG, V80, P416
   KRACKOW KA, 1986, J BONE JOINT SURG AM, V68A, P764, DOI 10.2106/00004623-198668050-00020
   Miroslav MZ, 2008, KNEE, V15, P419, DOI 10.1016/j.knee.2008.05.009
   Muratli H, 2005, KNEE SURG SPORT TR A, V13, P677, DOI 10.1007/s00167-005-0620-2
   PEIRO A, 1975, ACTA ORTHOP SCAND, V46, P700, DOI 10.3109/17453677508989253
   Rose PS, 2001, J BONE JOINT SURG AM, V83A, P1382, DOI 10.2106/00004623-200109000-00014
NR 14
TC 5
Z9 5
U1 0
U2 2
PU SPRINGER
PI NEW YORK
PA 233 SPRING ST, NEW YORK, NY 10013 USA
SN 0942-2056
EI 1433-7347
J9 KNEE SURG SPORT TR A
JI Knee Surg. Sports Traumatol. Arthrosc.
PD AUG
PY 2010
VL 18
IS 8
BP 1113
EP 1115
DI 10.1007/s00167-009-1021-8
PG 3
WC Orthopedics; Sport Sciences; Surgery
WE Science Citation Index Expanded (SCI-EXPANDED)
SC Orthopedics; Sport Sciences; Surgery
GA 628RA
UT WOS:000280137100015
PM 20033671
DA 2022-07-22
ER

PT J
AU Labib, SA
   Rolf, R
   Dacus, R
   Hutton, WC
AF Labib, Sameh A.
   Rolf, Robert
   Dacus, Rashard
   Hutton, William C.
TI The "Giftbox" Repair of the Achilles Tendon: A Modification of the
   Krackow Technique
SO FOOT & ANKLE INTERNATIONAL
LA English
DT Article
DE Krackow; Achilles Tendon; Giftbox; Rupture; Tensile Testing
ID TRIPLE BUNDLE TECHNIQUE; OPERATIVE REPAIR; SURGICAL REPAIR; RUPTURES;
   STRENGTH; METAANALYSIS; CADAVER; MOTION
AB Background: The Krackow locking loop technique has been used for Achilles tendon repair with documented success in allowing early range of motion with stable fixation. Previous studies documented failure at the rupture site by knot failure. We propose a modification of the traditional Krackow technique where the knots of the suture are tied away from the rupture site (aka the Giftbox technique). We compared the tensile strength of Achilles tendons repaired using the traditional Krackow technique with those repaired using the Giftbox technique. Materials and Methods: Thirteen pairs of fresh frozen cadaveric Achilles tendons were harvested. An Achilles tendon rupture,was created 4 cm from the calcaneal insertion. Thirteen Achilles ruptures were repaired using the traditional Krackow technique and 13 pairs were repaired using the Giftbox technique. The Achilles tendons were then tested to failure as defined as a gap of I cm. Results: The mean force to failure for the tendons using the Giftbox technique was 168 N, whereas the mean for the traditional Krackow technique was 81 N (p < 0.0001). Conclusion: Based on our biomechanical study, Achilles tendons repaired using the Giftbox technique are more than twice as strong as those repaired using the traditional Krackow technique. Clinical Relevance: We recommend the Giftbox modification to minimize gap formation and improve the strength of the repair of a ruptured Achilles tendon.
C1 [Labib, Sameh A.; Rolf, Robert; Dacus, Rashard; Hutton, William C.] Emory Univ, Sch Med, Dept Orthopaed Surg, Atlanta, GA USA.
   [Hutton, William C.] Atlanta VA Med Ctr, Atlanta, GA USA.
RP Hutton, WC (通讯作者)，Emory Spine Ctr, 59 Execut Pk S, Atlanta, GA 30329 USA.
EM William_Hutton@emoryhealthcare.org
CR Amlang MH, 2005, UNFALLCHIRURG, V108, P529, DOI 10.1007/s00113-005-0938-8
   Assal M, 2002, J BONE JOINT SURG AM, V84A, P161, DOI 10.2106/00004623-200202000-00001
   BESKIN JL, 1987, AM J SPORT MED, V15, P1, DOI 10.1177/036354658701500101
   Bhandari M, 2002, CLIN ORTHOP RELAT R, P190
   Boorman RS, 1998, J ORTHOP RES, V16, P682, DOI 10.1002/jor.1100160609
   Bruggeman NB, 2004, CLIN ORTHOP RELAT R, P63, DOI 10.1097/01.blo.0000144475.05543.e7
   CETTI R, 1993, AM J SPORT MED, V21, P791, DOI 10.1177/036354659302100606
   Cretnik A, 2000, MED SCI SPORT EXER, V32, P16
   Cretnik Andrej, 2004, J Foot Ankle Surg, V43, P72, DOI 10.1053/j.jfas.2004.01.008
   Haji A, 2004, FOOT ANKLE INT, V25, P215, DOI 10.1177/107110070402500404
   Jaakkola JI, 2001, FOOT ANKLE INT, V22, P979, DOI 10.1177/107110070102201210
   Jaakkola JI, 2000, FOOT ANKLE INT, V21, P14, DOI 10.1177/107110070002100103
   Khan RJK, 2005, J BONE JOINT SURG AM, V87A, P2202, DOI 10.2106/JBJS.D.03049
   KRAKOW KA, 1986, J BONE JOINT SURG AM, V68, P764
   KRAKOW KA, 1988, ORTHOPEDICS, V11, P909
   LABIB S, 2009, OPEN REPAIR IN PRESS
   Labib S, 2007, FOOT ANKLE INT, V28, P478, DOI 10.3113/FAI.2007.0478
   Lim J, 2001, FOOT ANKLE INT, V22, P559, DOI 10.1177/107110070102200705
   Maffulli N, 1999, J BONE JOINT SURG AM, V81A, P1019, DOI 10.2106/00004623-199907000-00017
   MANDELBAUM BR, 1995, AM J SPORT MED, V23, P392, DOI 10.1177/036354659502300403
   McKeon BP, 2006, ARTHROSCOPY, V22, P33, DOI 10.1016/j.arthro.2005.10.008
   MORTENSEN N H M, 1990, Ugeskrift for Laeger, V152, P3248
   Richardson LC, 2003, FOOT ANKLE INT, V24, P439, DOI 10.1177/107110070302400512
   Soldatis JJ, 1997, AM J SPORT MED, V25, P90, DOI 10.1177/036354659702500118
   WATSON TW, 1995, FOOT ANKLE INT, V16, P191, DOI 10.1177/107110079501600404
   Weinraub G M, 1998, J Foot Ankle Surg, V37, P86
   Winters SC, 1998, J HAND SURG-AM, V23A, P97, DOI 10.1016/S0363-5023(98)80096-8
NR 27
TC 33
Z9 36
U1 0
U2 6
PU SAGE PUBLICATIONS INC
PI THOUSAND OAKS
PA 2455 TELLER RD, THOUSAND OAKS, CA 91320 USA
SN 1071-1007
EI 1944-7876
J9 FOOT ANKLE INT
JI Foot Ankle Int.
PD MAY
PY 2009
VL 30
IS 5
BP 410
EP 414
DI 10.3113/FAI.2009.0410
PG 5
WC Orthopedics
WE Science Citation Index Expanded (SCI-EXPANDED)
SC Orthopedics
GA 440YO
UT WOS:000265735800005
PM 19439140
DA 2022-07-22
ER

PT J
AU Masood, T
   Kalliokoski, K
   Bojsen-Moller, J
   Finni, T
AF Masood, Tahir
   Kalliokoski, Kari
   Bojsen-Moller, Jens
   Finni, Taija
TI Muscle-tendon glucose uptake in Achilles tendon rupture and tendinopathy
   before and after eccentric rehabilitation: Comparative case reports
SO PHYSICAL THERAPY IN SPORT
LA English
DT Article
DE Glucose uptake; Positron emission tomography; Immobilization;
   Biomechanics
ID SKELETAL-MUSCLE; EXERCISE; PET; MECHANISMS; TENDINOSIS; HEALTHY;
   ATROPHY; HUMANS; ANKLE; PAIN
AB Achilles tendon rupture (ATR) is the most common tendon rupture injury. The consequences of ATR on metabolic activity of the Achilles tendon and ankle plantarflexors are unknown. Furthermore, the effects of eccentric rehabilitation on metabolic activity patterns of Achilles tendon and ankle plantarflexors in ATR patients have not been reported thus far. We present a case study demonstrating glucose uptake (GU) in the Achilles tendon, the triceps surae, and the flexor hallucis longus of a post-surgical ATR patient before and after a 5-month eccentric rehabilitation. At baseline, three months post-surgery, all muscles and Achilles tendon displayed much higher GU in the ATR patient compared to a healthy individual despite lower plantarflexion force. After the rehabilitation, plantarflexion force increased in the operated leg while muscle GU was considerably reduced. The triceps surae muscles showed similar values to the healthy control. When compared to the healthy or a matched patient with Achilles tendon pain after 12 weeks of rehabilitation, Achilles. tendon GU levels of ATR patient remained greater after the rehabilitation. Past studies have shown a shift in the metabolic fuel utilization towards glycolysis due to immobilization. Further research, combined with immuno-histological investigation, is needed to fully understand the mechanism behind excessive glucose uptake in ATR cases. (C) 2015 Elsevier Ltd. All rights reserved.
C1 [Masood, Tahir; Finni, Taija] Univ Jyvaskyla, Dept Biol Phys Act, Neuromuscular Res Ctr, SF-40351 Jyvaskyla, Finland.
   [Kalliokoski, Kari] Turku PET Ctr, Turku, Finland.
   [Bojsen-Moller, Jens] Norwegian Sch Sport Sci, Dept Phys Performance, Oslo, Norway.
   [Masood, Tahir] Isra Univ, Isra Inst Rehabil Sci, Islamabad Campus, Islamabad, Pakistan.
RP Masood, T (通讯作者)，Univ Jyvaskyla, Fac Sport & Hlth Sci, Dept Biol Phys Act, Neuromuscular Res Ctr NMRC, POB 35 VIV, Jyvaskyla 40014, Finland.
EM tahirphysio@gmail.com; Kari.Kalliokoski@tyks.fi;
   jens.bojsen-moller@nih.no; taija.finni@jyu.fi
RI Finni, Taija/AAV-9073-2020; Finni, Taija/F-6544-2014; Masood,
   Tahir/X-2473-2019
OI Finni, Taija/0000-0002-7697-2813; bojsen-moller,
   jens/0000-0001-6737-446X; Kalliokoski, Kari/0000-0002-8893-7126
FU Academy of Finland; Ministry of Education and Culture; Finnish Cultural
   Foundation
FX The study was funded by Academy of Finland, Ministry of Education and
   Culture (Taija Finni) and Finnish Cultural Foundation (Kari
   Kalliokoski).
CR Alfredson H, 1998, AM J SPORT MED, V26, P360, DOI 10.1177/03635465980260030301
   Aroen A, 2004, SCAND J MED SCI SPOR, V14, P30, DOI 10.1111/j.1600-0838.2004.00344.x
   Bojsen-Moller J, 2006, J APPL PHYSIOL, V101, P196, DOI 10.1152/japplphysiol.00004.2006
   Bojsen-Moller J, 2010, J APPL PHYSIOL, V109, P1895, DOI 10.1152/japplphysiol.00671.2010
   Cetti R, 2003, ACTA ORTHOP SCAND, V74, P78, DOI 10.1080/00016470310013707
   Finni T, 2006, CLIN BIOMECH, V21, P67, DOI 10.1016/j.clinbiomech.2005.08.007
   Foure A, 2013, J APPL PHYSIOL, V114, P523, DOI 10.1152/japplphysiol.01313.2011
   Fujimoto T, 2003, MED SCI SPORT EXER, V35, P777, DOI 10.1249/01.MSS.0000065070.49295.C0
   Goldsmith SJ, 2009, SEMIN NUCL MED, V39, P2, DOI 10.1053/j.semnuclmed.2008.08.002
   Heinonen I, 2012, PLOS ONE, V7, DOI 10.1371/journal.pone.0052191
   Hunter RB, 2002, FASEB J, V16, P529, DOI 10.1096/fj.01-0866com
   Kader D., 2007, EVIDENCE BASED SPORT, P562
   Kalliokoski KK, 2007, CLIN PHYSIOL FUNCT I, V27, P239, DOI 10.1111/j.1475-097X.2007.00744.x
   Kemppainen J, 2002, J PHYSIOL-LONDON, V542, P403, DOI 10.1113/jphysiol.2002.018135
   Kjaer M, 2014, J APPL PHYSIOL, V116, P1435, DOI 10.1152/japplphysiol.01044.2013
   Lawrence T, 2009, CSH PERSPECT BIOL, V1, DOI 10.1101/cshperspect.a001651
   Leppilahti J, 1998, SPORTS MED, V25, P79, DOI 10.2165/00007256-199825020-00002
   Leppilahti J, 1996, ACTA ORTHOP SCAND, V67, P277, DOI 10.3109/17453679608994688
   Lesic A, 2004, CURR ORTHOPAED, V18, P63, DOI 10.1016/j.cuor.2003.10.002
   Masood T, 2014, J APPL PHYSIOL, V117, P105, DOI 10.1152/japplphysiol.00113.2014
   Masood T, 2014, CLIN BIOMECH, V29, P564, DOI 10.1016/j.clinbiomech.2014.03.003
   Masood T, 2014, J ELECTROMYOGR KINES, V24, P367, DOI 10.1016/j.jelekin.2014.03.002
   Moller A, 1996, ACTA ORTHOP SCAND, V67, P479, DOI 10.3109/17453679608996672
   Mortensen NHM, 1999, J BONE JOINT SURG AM, V81A, P983, DOI 10.2106/00004623-199907000-00011
   Ohberg L, 2004, KNEE SURG SPORT TR A, V12, P465, DOI 10.1007/s00167-004-0494-8
   Peltoniemi P, 2000, AM J PHYSIOL-ENDOC M, V279, pE1122, DOI 10.1152/ajpendo.2000.279.5.E1122
   Rio E, 2015, BRIT J SPORT MED, V49, DOI 10.1136/bjsports-2014-094386
   Rose AJ, 2005, PHYSIOLOGY, V20, P260, DOI 10.1152/physiol.00012.2005
   Snyder W. S., 1975, ANN ICRP, V23, P273
   Stein TP, 2005, J NUTR, V135, p1824S, DOI 10.1093/jn/135.7.1824S
   Sussmilch-Leitch SP, 2012, J FOOT ANKLE RES, V5, DOI 10.1186/1757-1146-5-15
   van der Plas A, 2012, BRIT J SPORT MED, V46, P214, DOI 10.1136/bjsports-2011-090035
   Zhang P, 2007, MED HYPOTHESES, V69, P310, DOI 10.1016/j.mehy.2006.11.043
NR 33
TC 6
Z9 6
U1 1
U2 10
PU CHURCHILL LIVINGSTONE
PI EDINBURGH
PA JOURNAL PRODUCTION DEPT, ROBERT STEVENSON HOUSE, 1-3 BAXTERS PLACE,
   LEITH WALK, EDINBURGH EH1 3AF, MIDLOTHIAN, SCOTLAND
SN 1466-853X
J9 PHYS THER SPORT
JI Phys. Ther. Sport
PD SEP
PY 2016
VL 21
BP 14
EP 19
DI 10.1016/j.ptsp.2015.11.003
PG 6
WC Rehabilitation; Sport Sciences
WE Science Citation Index Expanded (SCI-EXPANDED)
SC Rehabilitation; Sport Sciences
GA DW0FF
UT WOS:000383315600003
PM 27428528
DA 2022-07-22
ER

PT J
AU Maffulli, N
   Leadbetter, WB
AF Maffulli, N
   Leadbetter, WB
TI Free gracilis tendon graft in neglected tears of the Achilles tendon
SO CLINICAL JOURNAL OF SPORT MEDICINE
LA English
DT Article
DE neglected Achilles tendon rupture; reconstruction; tendon autograft
ID CRUCIATE LIGAMENT RECONSTRUCTION; SEMITENDINOSUS TENDON; SUBCUTANEOUS
   RUPTURE; PLANTARIS TENDON; REPAIR; IMMOBILIZATION; MOBILIZATION;
   REGENERATION; MANAGEMENT; DIAGNOSIS
AB Objective: To report the results of a longitudinal study on reconstruction of neglected Achilles tendon rupture using a free autologous gracilis tendon graft.
   Design: Cohort study.
   Participants: Twenty-one patients underwent surgery for a neglected rupture of the Achilles tendon occurring between 65 days and 9 months before the operation.
   Methods: All participants were prospectively followed up for 2 years, and final review was performed at 28.4 +/- 3.5 months from the operation. Functional (anthropometric measurements, isometric strength, return to activities) and clinical assessment was performed.
   Results: No patients experienced any problems in the wound used to harvest the tendon of gracilis. Five patients were managed con servatively following a superficial infection of the Achilles tendon surgical wound. No patients developed a deep vein thrombosis or sustained a rerupture. All patients were able to walk on tiptoes, and no patient used a heel lift or walked with a visible limp. The maximum calf circumference remained significantly decreased in the operated leg at final review. The operated limb was significantly less strong than the nonoperated one.
   Conclusions: The management of neglected tears of the Achilles tendon by free gracilis tendon grafting is safe but technically demanding. It affords good recovery, even in patients with a neglected rupture of a duration of 9 months. These patients should be warned that they are at risk for postoperative complications, and that their ankle plantar flexion strength can remain reduced.
C1 Keele Univ, Sch Med, Dept Trauma & Orthopaed Surg, N Staffordshire Hosp, Stoke On Trent ST4 7QB, Staffs, England.
   Orthopaed Ctr PA, Rockville, MD USA.
RP Maffulli, N (通讯作者)，Keele Univ, Sch Med, Dept Trauma & Orthopaed Surg, N Staffordshire Hosp, Thornburrow Dr, Stoke On Trent ST4 7QB, Staffs, England.
EM n.maffulli@keele.ac.uk
RI Maffulli, Nicola/A-1839-2014
OI Maffulli, Nicola/0000-0002-5327-3702
CR Alfredson H, 1998, AM J SPORT MED, V26, P166, DOI 10.1177/03635465980260020301
   Bleakney RR, 2002, CLIN J SPORT MED, V12, P273, DOI 10.1097/00042752-200209000-00003
   BOSWORTH DM, 1956, J BONE JOINT SURG AM, V38, P111, DOI 10.2106/00004623-195638010-00011
   BOYDEN EM, 1995, CLIN ORTHOP RELAT R, V317, P150
   Bridgman Stephen, 2002, BMJ, V325, P658
   Coughlin MJ, 2002, ORTHOPEDICS, V25, P31
   Deehan DJ, 2002, J ROY COLL SURG EDIN, V47, P428
   DISTEFANO VJ, 1972, J TRAUM, V12, P671
   Ferretti A, 2002, AM J SPORT MED, V30, P204, DOI 10.1177/03635465020300021001
   INCAVO SJ, 1987, FOOT ANKLE, V8, P110, DOI 10.1177/107110078700800207
   Jennings AG, 2002, J BONE JOINT SURG BR, V84B, P361, DOI 10.1302/0301-620X.84B3.11559
   LINDHOLM A, 1959, Acta Chir Scand, V117, P261
   LYNN TA, 1966, J BONE JOINT SURG AM, VA 48, P268, DOI 10.2106/00004623-196648020-00005
   Maffulli N, 1999, J BONE JOINT SURG AM, V81A, P1019, DOI 10.2106/00004623-199907000-00017
   Maffulli N, 2003, AM J SPORT MED, V31, P692, DOI 10.1177/03635465030310051001
   MAFFULLI N, 1991, Journal of Foot Surgery, V30, P529
   Maffulli N, 1996, BRIT J SPORT MED, V30, P250, DOI 10.1136/bjsm.30.3.250
   Maffulli N, 1998, AM J SPORT MED, V26, P266, DOI 10.1177/03635465980260021801
   Maffulli N, 1999, CLIN J SPORT MED, V9, P157, DOI 10.1097/00042752-199907000-00007
   MAFFULLI N, 1989, J SPORT MED PHYS FIT, V29, P365
   MANDELBAUM BR, 1995, AM J SPORT MED, V23, P392, DOI 10.1177/036354659502300403
   OZAKI J, 1989, CLIN ORTHOP RELAT R, V238, P204
   Papandrea P, 2000, AM J SPORT MED, V28, P556, DOI 10.1177/03635465000280041901
   PEREZTEUFFER A, 1974, ORTHOP CLIN N AM, V5, P89
   Pintore E, 2001, J TRAUMA, V50, P71, DOI 10.1097/00005373-200101000-00013
   RANTANEN J, 1993, ACTA ORTHOP SCAND, V64, P333, DOI 10.3109/17453679308993638
   SAW Y, 1993, INJURY, V24, P479, DOI 10.1016/0020-1383(93)90155-Y
   Schepsis AA, 2002, AM J SPORT MED, V30, P287, DOI 10.1177/03635465020300022501
   Trillat A, 1967, Lyon Chir, V63, P603
   TURCO VJ, 1987, FOOT ANKLE, V7, P253, DOI 10.1177/107110078700700407
   Wapner K L, 1993, Foot Ankle, V14, P443
   WAPNER KL, 1995, ORTHOP CLIN N AM, V26, P249
   Wong J, 2002, AM J SPORT MED, V30, P565, DOI 10.1177/03635465020300041701
   YASUDA K, 1995, AM J SPORT MED, V23, P706, DOI 10.1177/036354659502300613
NR 34
TC 82
Z9 86
U1 0
U2 1
PU LIPPINCOTT WILLIAMS & WILKINS
PI PHILADELPHIA
PA TWO COMMERCE SQ, 2001 MARKET ST, PHILADELPHIA, PA 19103 USA
SN 1050-642X
EI 1536-3724
J9 CLIN J SPORT MED
JI Clin. J. Sport Med.
PD MAR
PY 2005
VL 15
IS 2
BP 56
EP 61
DI 10.1097/01.jsm.0000152714.05097.ef
PG 6
WC Orthopedics; Physiology; Sport Sciences
WE Science Citation Index Expanded (SCI-EXPANDED)
SC Orthopedics; Physiology; Sport Sciences
GA 943CF
UT WOS:000230328800003
PM 15782047
DA 2022-07-22
ER

PT J
AU Maffulli, N
   Reaper, JA
   Waterston, SW
   Ahya, R
AF Maffulli, N
   Reaper, JA
   Waterston, SW
   Ahya, R
TI ABO blood groups and Achilles tendon rupture in the Grampian Region of
   Scotland
SO CLINICAL JOURNAL OF SPORT MEDICINE
LA English
DT Article
DE blood groups; Achilles tendon rupture
ID GENE; ASSOCIATION
AB Objective: To test whether the association between blood groups and Achilles tendon rupture (ATR) reported in some Scandinavian countries and in Hungary was present in our region.
   Methods: We studied 78 patients treated at Aberdeen Royal Infirmary from 1990 to 1996, and compared their distribution of ABO blood groups with that found in 24,501 blood donors typed at the Blood Transfusion Centre during the same period.
   Results: Overall, 47 of 78 (60%) of patients with an Achilles tendon rupture belonged to blood group O, compared with 51% of the population as a whole. Only 22 (28%) of the Achilles tendon rupture patients belonged to blood group A. whereas 35% of the general population were members of this group (NS). The A/O ratio was 0.47 for the tendon rupture patients, compared with 0.68 for the general population (NS).
   Conclusions: We could not demonstrate any significant association between the proportions of ABO blood groups and ATR in the Grampian Region of Scotland. The findings in other studies could be due to peculiarities in the distribution of the ABO groups in genetically segregated populations.
C1 Univ Aberdeen, Sch Med, Dept Orthopaed Surg, Aberdeen AB25 3ZD, Scotland.
RP Maffulli, N (通讯作者)，Univ Aberdeen, Sch Med, Dept Orthopaed Surg, Polwarth Bldg, Aberdeen AB25 3ZD, Scotland.
RI Maffulli, Nicola/A-1839-2014
OI Maffulli, Nicola/0000-0002-5327-3702
CR ADALSTEINSSON S, 1985, ANN HUM GENET, V49, P275, DOI 10.1111/j.1469-1809.1985.tb01703.x
   CavalliSforza LL, 1997, P NATL ACAD SCI USA, V94, P7719, DOI 10.1073/pnas.94.15.7719
   GREENSPAN DS, 1992, GENOMICS, V12, P836, DOI 10.1016/0888-7543(92)90320-R
   HALVORSEN TB, 1986, SCAND J GASTROENTERO, V21, P979, DOI 10.3109/00365528608996408
   HUEBNER K, 1992, GENOMICS, V14, P220, DOI 10.1016/S0888-7543(05)80209-5
   JOZSA L, 1989, J BONE JOINT SURG BR, V71, P272, DOI 10.1302/0301-620X.71B2.2494187
   JOZSA L, 1989, AM J SPORT MED, V17, P338, DOI 10.1177/036354658901700305
   JUVONEN T, 1992, BRIT MED J, V305, P26, DOI 10.1136/bmj.305.6844.26
   KUJALA UM, 1992, INJURY, V23, P131, DOI 10.1016/0020-1383(92)90050-3
   LANZA RP, 1982, NEW ENGL J MED, V307, P1275
   Leppilahti J, 1996, ANN CHIR GYNAECOL FE, V85, P369
   LOURIE JA, 1983, ANN HUM BIOL, V10, P381, DOI 10.1080/03014468300006551
   Maffulli N, 1999, CLIN J SPORT MED, V9, P157, DOI 10.1097/00042752-199907000-00007
   MAHRLEIN R, 1995, AKTUEL TRAUMATOL, V25, P13
   MOURANT A E, 1974, Haematologia, V8, P183
   RENWICK JH, 1955, ANN HUM GENET, V19, P312, DOI 10.1111/j.1469-1809.1955.tb01356.x
   Toriello HV, 1996, NAT GENET, V13, P361, DOI 10.1038/ng0796-361
   WITSCHEW N, 1987, Beitraege zur Orthopaedie und Traumatologie, V34, P515
   WONG FL, 1992, GENET EPIDEMIOL, V9, P405
NR 19
TC 31
Z9 31
U1 0
U2 0
PU LIPPINCOTT WILLIAMS & WILKINS
PI PHILADELPHIA
PA 530 WALNUT ST, PHILADELPHIA, PA 19106-3621 USA
SN 1050-642X
J9 CLIN J SPORT MED
JI Clin. J. Sport Med.
PD OCT
PY 2000
VL 10
IS 4
BP 269
EP 271
DI 10.1097/00042752-200010000-00008
PG 3
WC Orthopedics; Physiology; Sport Sciences
WE Science Citation Index Expanded (SCI-EXPANDED)
SC Orthopedics; Physiology; Sport Sciences
GA 389HK
UT WOS:000166232400008
PM 11086753
DA 2022-07-22
ER

PT J
AU Eliasson, P
   Agergaard, AS
   Couppe, C
   Svensson, R
   Hoeffner, R
   Warming, S
   Warming, N
   Holm, C
   Jensen, MH
   Krogsgaard, M
   Kjaer, M
   Magnusson, SP
AF Eliasson, Pernilla
   Agergaard, Anne-Sofie
   Couppe, Christian
   Svensson, Rene
   Hoeffner, Rikke
   Warming, Susan
   Warming, Nichlas
   Holm, Christina
   Jensen, Mikkel Holm
   Krogsgaard, Michael
   Kjaer, Michael
   Magnusson, S. Peter
TI The Ruptured Achilles Tendon Elongates for 6 Months After Surgical
   Repair Regardless of Early or Late Weightbearing in Combination With
   Ankle Mobilization: A Randomized Clinical Trial
SO AMERICAN JOURNAL OF SPORTS MEDICINE
LA English
DT Article
DE Achilles tendon rupture; tendon strain; tendon healing; tendon
   elongation; weightbearing
ID CROSS-SECTIONAL AREA; NONSURGICAL TREATMENT; ACCELERATED REHABILITATION;
   MECHANICAL-PROPERTIES; FUNCTIONAL TREATMENT; FOLLOW-UP; METAANALYSIS;
   MUSCLE; PROTOCOL; DEFICITS
AB Background: Treatment strategies for Achilles tendon rupture vary considerably, and clinical outcome may depend on the magnitude of tendon elongation after surgical repair. The aim of this project was to examine whether tendon elongation, mechanical properties, and functional outcomes during rehabilitation of surgically repaired acute Achilles tendon ruptures were influenced by different rehabilitation regimens during the early postsurgical period.
   Hypothesis: Restricted early weightbearing that permits only limited motion about the ankle in the early phase of tendon healing limits tendon elongation and improves functional outcome.
   Study Design:Randomized controlled trial; Level of evidence, 1.
   Methods: 75 consecutive patients with an acute Achilles tendon rupture were included. They underwent surgical repair, and tantalum beads were placed in the distal and proximal parts of the tendon; thereafter, the patients were randomized into 3 groups. The first group was completely restricted from weightbearing until week 7. The second group was completely restricted from weightbearing until week 7 but performed ankle joint mobilization exercises. The first and second groups were allowed full weightbearing after week 8. The third group was allowed partial weightbearing from day 1 and full weightbearing from week 5. All patients received the same instructions in home exercise guidelines starting from week 9.
   Results: The rehabilitation regimen in the initial 8 weeks did not significantly influence any of the measured outcomes including tendon elongation. Achilles tendon elongation and tendon compliance continued for up to 6 months after surgery, and muscle strength, muscle endurance, and patient-reported functional scores did not reach normal values at 12 months.
   Conclusion: Differences in rehabilitation loading pattern in the initial 8 weeks after the repair of an Achilles tendon rupture did not measurably alter the outcome. The time to recover full function after an Achilles tendon rupture is at least 12 months.
C1 [Eliasson, Pernilla; Agergaard, Anne-Sofie; Couppe, Christian; Svensson, Rene; Hoeffner, Rikke; Warming, Susan; Warming, Nichlas; Holm, Christina; Jensen, Mikkel Holm; Krogsgaard, Michael; Kjaer, Michael; Magnusson, S. Peter] Bispebjerg Frederiksberg Hosp, Copenhagen, Denmark.
   [Eliasson, Pernilla; Agergaard, Anne-Sofie; Couppe, Christian; Svensson, Rene; Hoeffner, Rikke; Warming, Susan; Warming, Nichlas; Holm, Christina; Jensen, Mikkel Holm; Kjaer, Michael; Magnusson, S. Peter] Univ Copenhagen, Inst Sports Med Copenhagen, Dept Orthoped Surg, Bispebjerg Frederiksberg Hosp, Copenhagen, Denmark.
   [Eliasson, Pernilla; Agergaard, Anne-Sofie; Couppe, Christian; Svensson, Rene; Hoeffner, Rikke; Warming, Susan; Warming, Nichlas; Holm, Christina; Jensen, Mikkel Holm; Kjaer, Michael; Magnusson, S. Peter] Univ Copenhagen, Ctr Hlth Aging, Fac Hlth Sci, Copenhagen, Denmark.
   [Eliasson, Pernilla] Linkoping Univ, Dept Clin & Expt Med, Linkoping, Sweden.
   [Agergaard, Anne-Sofie; Couppe, Christian; Hoeffner, Rikke; Warming, Susan; Magnusson, S. Peter] Bispebjerg Frederiksberg Hosp, Dept Phys Therapy, Copenhagen, Denmark.
   [Krogsgaard, Michael] Bispebjerg Frederiksberg Hosp, Sect Sports Traumatol, Dept Orthoped Surg, Copenhagen, Denmark.
RP Eliasson, P (通讯作者)，Bispebjerg Frederiksberg Hosp, Inst Sports Med Copenhagen, Entrance 8,1st Floor,Nielsine Nielsens Vej 11, DK-2400 Copenhagen NV, Denmark.
EM pernilla.eliasson@gmail.com
RI krogsgaard, michael/ABF-4102-2020; Kjaer, Michael/W-6842-2019; Eliasson,
   Pernilla/AAY-2126-2020; Magnusson, Peter/AAN-1631-2020; Magnusson, S.
   Peter/AAD-5903-2022; Svensson, Rene B./AGZ-3181-2022; Krogsgaard,
   Michael Rindom/ABC-6014-2020
OI Kjaer, Michael/0000-0002-4582-8755; Eliasson,
   Pernilla/0000-0001-6718-034X; Magnusson, S. Peter/0000-0002-4308-5484;
   Svensson, Rene B./0000-0002-1607-5379;
FU Lundbeck foundation; Nordea Foundation (Center of Healthy Aging); IOC
   Research Center Sports Medicine Copenhagen; Danish Medical Research
   Council; Swedish Society for Medical Research
FX One or more of the authors have declared the following potential
   conflict of interest or source of funding: This study was supported by
   grants from Lundbeck foundation, Nordea Foundation (Center of Healthy
   Aging), IOC Research Center Sports Medicine Copenhagen, Danish Medical
   Research Council, and Swedish Society for Medical Research.
CR Amin NH, 2013, AM J SPORT MED, V41, P1864, DOI 10.1177/0363546513490659
   Andersson T, 2009, J APPL PHYSIOL, V107, P417, DOI 10.1152/japplphysiol.00414.2009
   Barfod KW, 2013, J FOOT ANKLE SURG, V52, P629, DOI 10.1053/j.jfas.2013.04.012
   Barfod KW, 2017, J FOOT ANKLE SURG, V56, P440, DOI 10.1053/j.jfas.2017.01.004
   Barfod KW, 2014, J BONE JOINT SURG AM, V96A, P1497, DOI 10.2106/JBJS.M.01273
   Brumann M, 2014, INJURY, V45, P1782, DOI 10.1016/j.injury.2014.06.022
   Cottom JM, 2017, J FOOT ANKLE SURG, V56, P423, DOI 10.1053/j.jfas.2016.10.012
   Couppe C, 2014, CLIN PHYSIOL FUNCT I, V34, P237, DOI 10.1111/cpf.12086
   Eliasson P, 2016, EUR J NUCL MED MOL I, V43, P1868, DOI 10.1007/s00259-016-3379-4
   Finni T, 1998, EUR J APPL PHYSIOL O, V77, P289, DOI 10.1007/s004210050335
   Finni T, 2006, CLIN BIOMECH, V21, P67, DOI 10.1016/j.clinbiomech.2005.08.007
   Froberg A, 2009, AM J SPORT MED, V37, P1200, DOI 10.1177/0363546508330126
   FUKUNAGA T, 1992, J ORTHOPAED RES, V10, P926, DOI 10.1002/jor.1100100623
   Gebauer M, 2007, ARCH ORTHOP TRAUM SU, V127, P795, DOI 10.1007/s00402-007-0325-8
   Hansen MS, 2016, KNEE SURG SPORT TR A, V24, P1365, DOI 10.1007/s00167-015-3974-0
   Heikkinen J, 2017, AM J SPORT MED, V45, P1395, DOI 10.1177/0363546517694610
   Herbort M, 2008, ARCH ORTHOP TRAUM SU, V128, P1273, DOI 10.1007/s00402-008-0602-1
   Houshian S, 1998, INJURY, V29, P651, DOI 10.1016/S0020-1383(98)00147-8
   Ismail M, 2008, FOOT ANKLE INT, V29, P808, DOI 10.3113/FAI.2008.0808
   Kangas J, 2007, AM J SPORT MED, V35, P59, DOI 10.1177/0363546506293255
   Khan RJK, 2010, COCHRANE DB SYST REV, DOI 10.1002/14651858.CD003674.pub4
   Khan RJK, 2005, J BONE JOINT SURG AM, V87A, P2202, DOI 10.2106/JBJS.D.03049
   Kjaer M, 2004, PHYSIOL REV, V84, P649, DOI 10.1152/physrev.00031.2003
   KOMI PV, 1987, INT J SPORTS MED, V8, P3
   KOMI PV, 1992, CLIN SPORT MED, V11, P521
   Kongsgaard M, 2005, J APPL PHYSIOL, V99, P1965, DOI 10.1152/japplphysiol.00384.2005
   Kongsgaard M, 2011, CLIN BIOMECH, V26, P772, DOI 10.1016/j.clinbiomech.2011.02.011
   Konor MM, 2012, INT J SPORTS PHYS TH, V7, P279
   Lantto I, 2015, AM J SPORT MED, V43, P2302, DOI 10.1177/0363546515591267
   Lee SJ, 2009, AM J SPORT MED, V37, P786, DOI 10.1177/0363546508328595
   Leppilahti J, 1996, ACTA ORTHOP SCAND, V67, P277, DOI 10.3109/17453679608994688
   Maffulli N, 1999, CLIN J SPORT MED, V9, P157, DOI 10.1097/00042752-199907000-00007
   Magnusson SP, 2010, NAT REV RHEUMATOL, V6, P262, DOI 10.1038/nrrheum.2010.43
   Mark-Christensen T, 2016, KNEE SURG SPORT TR A, V24, P1852, DOI 10.1007/s00167-014-3180-5
   Moller M, 2002, SCAND J MED SCI SPOR, V12, P9, DOI 10.1034/j.1600-0838.2002.120103.x
   Mortensen NHM, 1999, J BONE JOINT SURG AM, V81A, P983, DOI 10.2106/00004623-199907000-00011
   Mullaney MJ, 2006, AM J SPORT MED, V34, P1120, DOI 10.1177/0363546505284186
   NILLIUS SA, 1976, ACTA ORTHOP SCAND, V47, P118, DOI 10.3109/17453677608998983
   Nilsson-Helander K, 2007, AM J SPORT MED, V35, P421, DOI 10.1177/0363546506294856
   Nilsson-Helander K, 2010, AM J SPORT MED, V38, P2186, DOI 10.1177/0363546510376052
   Olsson N, 2014, AM J SPORT MED, V42, P1448, DOI 10.1177/0363546514527409
   Olsson N, 2013, AM J SPORT MED, V41, P2867, DOI 10.1177/0363546513503282
   Olsson N, 2011, KNEE SURG SPORT TR A, V19, P1385, DOI 10.1007/s00167-011-1511-3
   Pajala A, 2009, J BONE JOINT SURG AM, V91A, P1092, DOI 10.2106/JBJS.G.01089
   Porter MD, 2015, ANZ J SURG, V85, P373, DOI 10.1111/ans.12910
   Rosso C, 2015, KNEE SURG SPORT TR A, V23, P890, DOI 10.1007/s00167-013-2726-2
   Schepull T, 2015, KNEE SURG SPORT TR A, V23, P884, DOI 10.1007/s00167-013-2720-8
   Schepull T, 2013, AM J SPORT MED, V41, P2550, DOI 10.1177/0363546513501785
   Schepull T, 2011, AM J SPORT MED, V39, P38, DOI 10.1177/0363546510383515
   Schepull T, 2007, BMC MUSCULOSKEL DIS, V8, DOI 10.1186/1471-2474-8-116
   Silbernagel KG, 2012, AM J SPORT MED, V40, P1564, DOI 10.1177/0363546512447926
   Silbernagel KG, 2010, KNEE SURG SPORT TR A, V18, P258, DOI 10.1007/s00167-009-0889-7
   Soroceanu A, 2012, J BONE JOINT SURG AM, V94A, P2136, DOI 10.2106/JBJS.K.00917
   Speck M, 1998, AM J SPORT MED, V26, P789, DOI 10.1177/03635465980260060901
   Suchak AA, 2006, CLIN ORTHOP RELAT R, P216, DOI 10.1097/01.blo.0000203458.05135.74
   Willits K, 2010, J BONE JOINT SURG AM, V92A, P2767, DOI 10.2106/JBJS.I.01401
NR 56
TC 40
Z9 42
U1 3
U2 13
PU SAGE PUBLICATIONS INC
PI THOUSAND OAKS
PA 2455 TELLER RD, THOUSAND OAKS, CA 91320 USA
SN 0363-5465
EI 1552-3365
J9 AM J SPORT MED
JI Am. J. Sports Med.
PD AUG
PY 2018
VL 46
IS 10
BP 2492
EP 2502
DI 10.1177/0363546518781826
PG 11
WC Orthopedics; Sport Sciences
WE Science Citation Index Expanded (SCI-EXPANDED)
SC Orthopedics; Sport Sciences
GA GO9PH
UT WOS:000440439000022
PM 29965789
OA Green Submitted
DA 2022-07-22
ER

PT J
AU Fryhofer, GW
   Freedman, BR
   Hillin, CD
   Salka, NS
   Pardes, AM
   Weiss, SN
   Farber, DC
   Soslowsky, LJ
AF Fryhofer, George W.
   Freedman, Benjamin R.
   Hillin, Cody D.
   Salka, Nabeel S.
   Pardes, Adam M.
   Weiss, Stephanie N.
   Farber, Daniel C.
   Soslowsky, Louis J.
TI Postinjury biomechanics of Achilles tendon vary by sex and hormone
   status
SO JOURNAL OF APPLIED PHYSIOLOGY
LA English
DT Article
DE Achilles tendon; tendon healing; biomechanics; sex differences;
   ovariectomy; female athlete
ID CRUCIATE LIGAMENT INJURY; ESTROGEN; RUPTURE; IMAGE; PROGESTERONE;
   ACTIVATION; CELLS; GAIT
AB Achilles tendon ruptures are common injuries. Sex differences are present in mechanical properties of uninjured Achilles tendon, but it remains unknown if these differences extend to tendon healing. We hypothesized that ovariectomized females (OVX) and males would exhibit inferior postinjury tendon properties compared with females. Male, female, and OVX Sprague-Dawley rats (n = 32/group) underwent acclimation and treadmill training before blunt transection of the Achilles tendon midsubstance. Injured hindlimbs were immobilized for 1 wk, followed by gradual return to activity and assessment of active and passive hindlimb function. Animals were euthanized at 3 or 6 wk postinjury to assess tendon structure, mechanics, and composition. Passive ankle stiffness and range of motion were superior in females at 3 wk; however, by 6 wk, passive and active function were similar in males and females but remained inferior in OVX. At 6 wk, female tendons had greater normalized secant modulus, viscoelastic behavior, and laxity compared with males. Normalized secant modulus, crosssectional area and tendon glycosaminoglycan composition were inferior in OVX compared with females at 6 wk. Total fatigue cycles until tendon failure were similar among groups. Postinjury muscle fiber size was better preserved in females compared with males, and females had greater collagen III at the tendon injury site compared with males at 6 wk. Despite male and female Achilles tendons withstanding similar durations of fatigue loading, early passive hindlimb function and tendon mechanical properties, including secant modulus, suggest superior healing in females. Ovarian hormone loss was associated with inferior Achilles tendon healing.
C1 [Fryhofer, George W.; Freedman, Benjamin R.; Hillin, Cody D.; Salka, Nabeel S.; Pardes, Adam M.; Weiss, Stephanie N.; Farber, Daniel C.; Soslowsky, Louis J.] Univ Penn, McKay Orthopaed Lab, Dept Orthopaed Surg, Philadelphia, PA 19104 USA.
RP Soslowsky, LJ (通讯作者)，Univ Penn, McKay Orthopaed Res Lab, 424 Stemmler Hall,3450 Hamilton Walk, Philadelphia, PA 19104 USA.
EM soslowsk@upenn.edu
RI Fryhofer, George/L-1954-2016
OI Fryhofer, George/0000-0002-8105-0637
FU National Institute of Arthritis and Musculoskeletal and Skin Diseases
   (NIAMS) [R01AR064216S1]; NIAMS [P30 AR050950]; National Center for
   Advancing Translational Sciences [TL1TR000138]; National Institute of
   Arthritis and Musculoskeletal and Skin Diseases [T32AR007132]; National
   Science Foundation Graduate Research Fellowship Program; NATIONAL
   INSTITUTE OF ARTHRITIS AND MUSCULOSKELETAL AND SKIN DISEASES
   [R01AR064216, T32AR007132, P30AR069619] Funding Source: NIH RePORTER
FX This study was funded by National Institute of Arthritis and
   Musculoskeletal and Skin Diseases (NIAMS) Grant (R01AR064216S1), the
   NIAMS supported Penn Center for Musculoskeletal Disorders (P30
   AR050950), the National Center for Advancing Translational Sciences
   (TL1TR000138), the National Institute of Arthritis and Musculoskeletal
   and Skin Diseases (T32AR007132), and National Science Foundation
   Graduate Research Fellowship Program.
CR Bates NA, 2016, J BIOMECH, V49, P1429, DOI 10.1016/j.jbiomech.2016.03.001
   Bridgeman JT, 2010, FOOT ANKLE INT, V31, P1081, DOI 10.3113/FAI.2010.1081
   Bryant AL, 2008, J APPL PHYSIOL, V105, P1035, DOI 10.1152/japplphysiol.01281.2007
   Chiodo CP, 2010, J BONE JOINT SURG AM, V92A, P2466
   Circi E, 2009, INT ORTHOP, V33, P1461, DOI 10.1007/s00264-009-0778-1
   Eliasson P, 2012, J ORTHOP RES, V30, P274, DOI 10.1002/jor.21511
   Favata M., 2006, BIOENGINEERING
   Fisher JS, 2000, J APPL PHYSIOL, V88, P265, DOI 10.1063/1.373651
   Freedman Benjamin R, 2014, Muscles Ligaments Tendons J, V4, P245
   Freedman BR, 2015, J ORTHOP RES, V33, P904, DOI 10.1002/jor.22875
   Freedman BR, 2014, J BIOMECH, V47, P2028, DOI 10.1016/j.jbiomech.2013.10.054
   Freedman BR, J ORTHOP RE IN PRESS
   Gillis GB, 2001, J EXP BIOL, V204, P2717
   Gordon JA, 2015, J BIOMECH, V48, P2110, DOI 10.1016/j.jbiomech.2015.02.058
   Hansen MS, 2016, KNEE SURG SPORT TR A, V24, P1365, DOI 10.1007/s00167-015-3974-0
   Hansen M, 2016, ADV EXP MED BIOL, V920, P139, DOI 10.1007/978-3-319-33943-6_13
   Hansen M, 2014, EXERC SPORT SCI REV, V42, P183, DOI 10.1249/JES.0000000000000026
   Hewett TE, 2005, BRIT J SPORT MED, V39, P347, DOI 10.1136/bjsm.2005.018572
   Houshian S, 1998, INJURY, V29, P651, DOI 10.1016/S0020-1383(98)00147-8
   Huisman ES, 2014, J ANAT, V224, P538, DOI 10.1111/joa.12169
   Huttunen TT, 2014, AM J SPORT MED, V42, P2419, DOI 10.1177/0363546514540599
   JOZSA L, 1989, AM J SPORT MED, V17, P338, DOI 10.1177/036354658901700305
   Kiapour AM, 2014, BONE JOINT RES, V3, P20, DOI 10.1302/2046-3758.32.2000241
   Kiapour AM, 2015, AM J SPORT MED, V43, P1623, DOI 10.1177/0363546515582024
   Lambers FM, 2012, CALCIFIED TISSUE INT, V90, P108, DOI 10.1007/s00223-011-9553-6
   Lenth RV, 2016, J STAT SOFTW, V69, P1, DOI 10.18637/jss.v069.i01
   Leppilahti J, 1996, ACTA ORTHOP SCAND, V67, P277, DOI 10.3109/17453679608994688
   Liu SH, 1996, J ORTHOPAED RES, V14, P526, DOI 10.1002/jor.1100140405
   MARCONDES F. K., 2002, Braz. J. Biol., V62, P609, DOI 10.1590/S1519-69842002000400008
   Moller A, 1996, ACTA ORTHOP SCAND, V67, P479, DOI 10.3109/17453679608996672
   Pardes AM, 2016, ANN BIOMED ENG, V44, P2901, DOI 10.1007/s10439-016-1635-1
   Pardes AM, 2016, J BIOMECH, V49, P376, DOI 10.1016/j.jbiomech.2015.12.027
   Peltz CD, 2009, J BONE JOINT SURG AM, V91A, P2421, DOI 10.2106/JBJS.H.01121
   Riggin CN, 2014, J BIOMECH ENG-T ASME, V136, DOI 10.1115/1.4026285
   Sandberg OH, 2015, MLTJ-MUSCLE LIGAMENT, V5, P63, DOI 10.11138/mltj/2015.5.2.063
   Sarver JJ, 2010, J BIOMECH, V43, P778, DOI 10.1016/j.jbiomech.2009.10.031
   Schindelin J, 2012, NAT METHODS, V9, P676, DOI [10.1038/NMETH.2019, 10.1038/nmeth.2019]
   Schneider CA, 2012, NAT METHODS, V9, P671, DOI 10.1038/nmeth.2089
   Silbernagel KG, 2015, ORTHOP J SPORTS MED, V3, DOI 10.1177/2325967115586768
   Smith LR, 2014, SKELET MUSCLE, V4, DOI 10.1186/2044-5040-4-21
   Soroceanu A, 2012, J BONE JOINT SURG AM, V94A, P2136, DOI 10.2106/JBJS.K.00917
   Sullivan BE, 2009, J APPL PHYSIOL, V106, P468, DOI 10.1152/japplphysiol.91341.2008
   Team R. C, 2016, R LANG ENV STAT COMP
   Torricelli P, 2013, AGE, V35, P2125, DOI 10.1007/s11357-012-9500-0
   Veronesi F, 2015, CYTOTHERAPY, V17, P1536, DOI 10.1016/j.jcyt.2015.07.007
   Vosseller JT, 2013, FOOT ANKLE INT, V34, P49, DOI 10.1177/1071100712460223
   Witte MM, 2010, GEN COMP ENDOCR, V166, P520, DOI 10.1016/j.ygcen.2010.01.006
   Yu WD, 2001, CLIN ORTHOP RELAT R, P268, DOI 10.1097/00003086-200102000-00031
NR 48
TC 14
Z9 13
U1 1
U2 10
PU AMER PHYSIOLOGICAL SOC
PI BETHESDA
PA 9650 ROCKVILLE PIKE, BETHESDA, MD 20814 USA
SN 8750-7587
EI 1522-1601
J9 J APPL PHYSIOL
JI J. Appl. Physiol.
PD NOV 1
PY 2016
VL 121
IS 5
BP 1106
EP 1114
DI 10.1152/japplphysiol.00620.2016
PG 9
WC Physiology; Sport Sciences
WE Science Citation Index Expanded (SCI-EXPANDED)
SC Physiology; Sport Sciences
GA EE5MH
UT WOS:000389651700009
PM 27633741
OA Green Published
DA 2022-07-22
ER

PT J
AU Ahn, JH
   Choy, WS
AF Ahn, Jae Hoon
   Choy, Won-Sik
TI Tendon Adhesion after Percutaneous Repair of the Achilles Tendon: A Case
   Report
SO JOURNAL OF FOOT & ANKLE SURGERY
LA English
DT Article
DE ankle; leg; surgery; trauma
ID RUPTURE; REHABILITATION
AB Percutaneous repair of the Achilles tendon is known to minimize tendon adhesions and wound complication through a minimally invasive technique. The usually reported complications are sural nerve injury and inadequate contact of the tendon stumps, which can lead to rerupture or tendon lengthening. We experienced an interesting case of Achilles tendon adhesion after percutaneous repair, which had been initially misdiagnosed as a rerupture. To our knowledge, this is the only report of Achilles tendon adhesion as a complication after percutaneous repair, which stresses the importance of functional postoperative rehabilitation. (C) 2011 by the American College of Foot and Ankle Surgeons. All rights reserved.
C1 [Ahn, Jae Hoon; Choy, Won-Sik] Eulji Univ, Coll Med, Dept Orthopaed Surg, Taejon 302799, South Korea.
RP Ahn, JH (通讯作者)，Eulji Univ, Coll Med, Dept Orthoped Surg, 1306 Dunsan Dong, Taejon 302799, South Korea.
EM jhahn@eulji.ac.kr
CR ARNER O, 1959, ACTA CHIR SCAND, V116, P7
   Assal M, 2002, J BONE JOINT SURG AM, V84A, P161, DOI 10.2106/00004623-200202000-00001
   BRADLEY JP, 1990, AM J SPORT MED, V18, P188, DOI 10.1177/036354659001800213
   Calder JDF, 2005, BRIT J SPORT MED, V39, P857, DOI 10.1136/bjsm.2004.017509
   CETTI R, 1993, AM J SPORT MED, V21, P791, DOI 10.1177/036354659302100606
   Halasi T, 2003, KNEE SURG SPORT TR A, V11, P409, DOI 10.1007/s00167-003-0397-0
   INGLIS AE, 1976, J BONE JOINT SURG AM, V58, P990, DOI 10.2106/00004623-197658070-00015
   KAKIUCHI M, 1995, J BONE JOINT SURG BR, V77B, P60, DOI 10.1302/0301-620X.77B1.7822398
   Leppilahti J, 1998, CLIN ORTHOP RELAT R, P152
   MA GWC, 1977, CLIN ORTHOP RELAT R, P247
   Maffulli N, 1999, J BONE JOINT SURG AM, V81A, P1019, DOI 10.2106/00004623-199907000-00017
   Maffulli N, 1999, CLIN J SPORT MED, V9, P157, DOI 10.1097/00042752-199907000-00007
   MANDELBAUM BR, 1995, AM J SPORT MED, V23, P392, DOI 10.1177/036354659502300403
   Moller M, 2001, J BONE JOINT SURG BR, V83B, P843
   Mortensen NHM, 1999, J BONE JOINT SURG AM, V81A, P983, DOI 10.2106/00004623-199907000-00011
NR 15
TC 5
Z9 6
U1 0
U2 0
PU ELSEVIER SCIENCE INC
PI NEW YORK
PA 360 PARK AVE SOUTH, NEW YORK, NY 10010-1710 USA
SN 1067-2516
EI 1542-2224
J9 J FOOT ANKLE SURG
JI J. Foot Ankle Surg.
PD JAN-FEB
PY 2011
VL 50
IS 1
BP 93
EP 95
DI 10.1053/j.jfas.2010.07.008
PG 3
WC Orthopedics; Surgery
WE Science Citation Index Expanded (SCI-EXPANDED)
SC Orthopedics; Surgery
GA 697AC
UT WOS:000285481500022
PM 20833567
DA 2022-07-22
ER

PT J
AU Aderinto, J
   Gross, A
AF Aderinto, Joseph
   Gross, Allan
TI Delayed Repair of Tibialis Anterior Tendon Rupture with Achilles Tendon
   Allograft
SO JOURNAL OF FOOT & ANKLE SURGERY
LA English
DT Article
DE ankle; foot; hypothyroidism; injury; surgery; trauma
ID SUBCUTANEOUS RUPTURE
AB Few cases of spontaneous rupture of the tibialis anterior tendon have been reported in the literature. We report a case of a 66-year-old male with a spontaneous rupture of his left tibialis anterior tendon, with a delayed presentation of approximately 3 months. At the time of reconstruction there was marked retraction of the proximal portion of the tibialis anterior tendon, which precluded an end-to-end repair; therefore, we used an Achilles tendon allograft. (C) 2011 by the American College of Foot and Ankle Surgeons. All rights reserved.
C1 [Aderinto, Joseph; Gross, Allan] Mt Sinai Hosp, Div Orthopaed, Toronto, ON M5G 1X5, Canada.
RP Gross, A (通讯作者)，Mt Sinai Hosp, Div Orthopaed, Appt 476A,600 Univ Ave, Toronto, ON M5G 1X5, Canada.
EM Allan.gross@utoronto.ca
CR Anagnostakos K, 2006, FOOT ANKLE INT, V27, P330, DOI 10.1177/107110070602700504
   Boyette DM, 2008, TECH FOOT ANKLE SURG, V7, P120, DOI 10.1097/btf.0b013e318165decc
   Burman MS, 1934, ANN SURG, V100, P368, DOI 10.1097/00000658-193408000-00013
   CIRINCIONE RJ, 1975, J BONE JOINT SURG BR, V57, P852
   DiDomenico LA, 2008, J FOOT ANKLE SURG, V47, P463, DOI 10.1053/j.jfas.2008.05.007
   DOOLEY BJ, 1980, J BONE JOINT SURG BR, V62, P471, DOI 10.1302/0301-620X.62B4.7430226
   KASHYAP S, 1987, CLIN ORTHOP RELAT R, P159
   Kausch T, 1998, ARCH ORTHOP TRAUM SU, V117, P290, DOI 10.1007/s004020050250
   KLEINMAN M, 1983, J BONE JOINT SURG AM, V65, P1345, DOI 10.2106/00004623-198365090-00019
   Mankey MG, 1994, OPERATIVE TECHNIQUES, V4, P141
   McGarvey WC, 1996, FOOT ANKLE INT, V17, P496, DOI 10.1177/107110079601700811
   *MED RES COUNC, 1954, SPEC REP SER, V282, P64
   MENSOR MC, 1953, J BONE JOINT SURG AM, V35-A, P675, DOI 10.2106/00004623-195335030-00014
   MOBERG E, 1947, ACTA CHIR SCAND, V95, P455
   OMARI AM, 1999, FOOT ANKLE SURG, V5, P251
   Patten A, 2000, FOOT ANKLE INT, V21, P697, DOI 10.1177/107110070002100814
   Rao S K, 2005, J Orthop Surg (Hong Kong), V13, P178
   RIMOLDI RL, 1991, FOOT ANKLE, V12, P176, DOI 10.1177/107110079101200308
   TOMFORD WW, 1995, J BONE JOINT SURG AM, V77A, P1742, DOI 10.2106/00004623-199511000-00017
   WENER JA, 1974, J BONE JOINT SURG AM, VA 56, P823, DOI 10.2106/00004623-197456040-00021
NR 20
TC 14
Z9 14
U1 0
U2 0
PU ELSEVIER SCIENCE INC
PI NEW YORK
PA 360 PARK AVE SOUTH, NEW YORK, NY 10010-1710 USA
SN 1067-2516
J9 J FOOT ANKLE SURG
JI J. Foot Ankle Surg.
PD MAY-JUN
PY 2011
VL 50
IS 3
BP 340
EP 342
DI 10.1053/j.jfas.2011.02.009
PG 3
WC Orthopedics; Surgery
WE Science Citation Index Expanded (SCI-EXPANDED)
SC Orthopedics; Surgery
GA 753NB
UT WOS:000289783800014
PM 21435914
DA 2022-07-22
ER

PT J
AU Marcel, JJ
   Sage, K
   Guyton, GP
AF Marcel, John J., Jr.
   Sage, Katherine
   Guyton, Gregory P.
TI Complications of Supine Surgical Achilles Tendon Repair
SO FOOT & ANKLE INTERNATIONAL
LA English
DT Article
DE Achilles repair; Achilles rupture; supine positioning; posteromedial
   approach
ID LARYNGEAL MASK AIRWAY; PRONE POSITION; NONOPERATIVE TREATMENT;
   CLINICAL-IMPLICATIONS; ACUTE RUPTURE; SURGERY; ANGIOSOMES; METAANALYSIS;
   TRIALS; ANKLE
AB Background: Open Achilles tendon surgery with the patient in the supine position potentially avoids the complications of the prone position, but the safety and viability of the supine position for this procedure are not known. The aim of this study was to test the hypothesis that supine positioning for open repair of acute Achilles tendon ruptures would be safe, with low wound and neurologic complication rates.
   Methods: Supine position safety in acute Achilles tendon repair was investigated. Consecutive cases of supine Achilles tendon surgical repair performed by one surgeon from 2010 to 2015 were retrospectively reviewed. Patients were included if they were surgically treated with primary repair in the supine position within 15 days of injury and did not undergo concomitant surgery. A paramedian incision 1 cm medial to the Achilles sheath was used. Initial chart review identified 161 patients who underwent any type of Achilles tendon surgery in the supine position, of whom 45 patients met the inclusion criteria. This group included 39 men and 6 women with an average age of 41 years (range, 20-66 years). Median length of follow-up was 116 days (range, 25-1,589 days). Average body mass index was 29 kg/m(2) (range, 23-36 kg/m(2)).
   Results: There were no infections, sural nerve injuries, or reruptures.
   Conclusions: The supine position was safe for primary open Achilles tendon repair, with no wound or neurologic complications.
   Level of Evidence: Level IV, case series.
C1 [Marcel, John J., Jr.; Sage, Katherine; Guyton, Gregory P.] MedStar Union Mem Hosp, Dept Orthopaed Surg, 3333 North Calvert St,Suite 400, Baltimore, MD 21218 USA.
RP Guyton, GP (通讯作者)，MedStar Union Mem Hosp, Dept Orthopaed Surg, 3333 North Calvert St,Suite 400, Baltimore, MD 21218 USA.
EM lyn.camire@medstar.net
CR Attinger CE, 2006, PLAST RECONSTR SURG, V117, p261S, DOI 10.1097/01.prs.0000222582.84385.54
   Beck D, 2017, FOOT ANKLE ORTHOP, DOI [10.1177/2473011417S000114, DOI 10.1177/2473011417S000114]
   Brodsky JB, 2001, J CLIN ANESTH, V13, P138, DOI 10.1016/S0952-8180(01)00230-6
   Brown J, 2001, RESUSCITATION, V50, P233, DOI 10.1016/S0300-9572(01)00362-8
   Bruggeman NB, 2004, CLIN ORTHOP RELAT R, P63, DOI 10.1097/01.blo.0000144475.05543.e7
   Gluck GS, 2008, J FOOT ANKLE SURG, V47, P603, DOI 10.1053/j.jfas.2008.08.001
   Gougoulias N, 2013, BONE JOINT J, V95B, P1317, DOI 10.1302/0301-620X.95B10.32527
   Hans P, 2006, EUR J ANAESTH, V23, P83, DOI 10.1017/S0265021505231824
   Highlander Peter, 2011, Foot Ankle Spec, V4, P361, DOI 10.1177/1938640011418488
   Hsu AR, 2015, FOOT ANKLE INT, V36, P1279, DOI 10.1177/1071100715589632
   Keating JF, 2011, J BONE JOINT SURG BR, V93B, P1071, DOI 10.1302/0301-620X.93B8.25998
   Khan RJK, 2005, J BONE JOINT SURG AM, V87A, P2202, DOI 10.2106/JBJS.D.03049
   Kwee MM, 2015, INT SURG, V100, P292, DOI 10.9738/INTSURG-D-13-00256.1
   Lee LA, 2012, ANESTHESIOLOGY, V116, P15, DOI 10.1097/ALN.0b013e31823d012a
   Moller M, 2001, J BONE JOINT SURG BR, V83B, P843
   Pajala A, 2009, J BONE JOINT SURG AM, V91A, P1092, DOI 10.2106/JBJS.G.01089
   Roth S, 2009, Br J Anaesth, V103 Suppl 1, pi31, DOI 10.1093/bja/aep295
   Soroceanu A, 2012, J BONE JOINT SURG AM, V94A, P2136, DOI 10.2106/JBJS.K.00917
   Stambough JL, 2007, J AM ACAD ORTHOP SUR, V15, P156, DOI 10.5435/00124635-200703000-00005
   Tan GJ, 2009, FOOT ANKLE INT, V30, P1124, DOI 10.3113/FAI.2009.1124
   TAYLOR GI, 1987, BRIT J PLAST SURG, V40, P113, DOI 10.1016/0007-1226(87)90185-8
   Taylor GI, 1998, PLAST RECONSTR SURG, V102, P599, DOI 10.1097/00006534-199809030-00001
   van Esch BF, 2017, J CLIN ANESTH, V36, P142, DOI 10.1016/j.jclinane.2016.10.004
   Williams E Lynne, 2002, Anesthesiol Clin North Am, V20, P605
   Willits K, 2010, J BONE JOINT SURG AM, V92A, P2767, DOI 10.2106/JBJS.I.01401
   Yano T, 2008, J ANESTH, V22, P312, DOI 10.1007/s00540-008-0635-1
   Yepes H, 2010, J BONE JOINT SURG AM, V92A, P1215, DOI 10.2106/JBJS.I.00743
NR 27
TC 4
Z9 4
U1 0
U2 0
PU SAGE PUBLICATIONS INC
PI THOUSAND OAKS
PA 2455 TELLER RD, THOUSAND OAKS, CA 91320 USA
SN 1071-1007
EI 1944-7876
J9 FOOT ANKLE INT
JI Foot Ankle Int.
PD JUN
PY 2018
VL 39
IS 6
BP 720
EP 724
DI 10.1177/1071100718755474
PG 5
WC Orthopedics
WE Science Citation Index Expanded (SCI-EXPANDED)
SC Orthopedics
GA GH9YO
UT WOS:000434026300012
PM 29448826
DA 2022-07-22
ER

PT J
AU Webb, J
   Moorjani, N
   Radford, M
AF Webb, J
   Moorjani, N
   Radford, M
TI Anatomy of the sural nerve and its relation to the Achilles Tendon
SO FOOT & ANKLE INTERNATIONAL
LA English
DT Article
DE sural nerve; tendo achilles rupture; percutaneous tendo achilles repair
ID NON-SURGICAL TREATMENT; RUPTURES; REPAIR
AB Sural nerve injury is a complication of Achilles Tendon (TA) rupture. We dissected 30 cadaveric lower limbs to describe the course of the sural nerve in relation to the TA. At the level of insertion of the TA into the calcaneum, the sural nerve was a mean 18.8 mm from the lateral border of the TA, The proximal course of the nerve was towards the midline such that it crossed the lateral border of the TA at a mean distance of 9.8 cm from the calcaneum, The significant individual variation in the position of the sural nerve in relation to the achilles tendon should be borne in mind when placing sutures in the proximal part of the achilles tendon. Percutaneous sutures should not be placed in the lateral half of the TA.
C1 Nuffield Orthopaed Ctr, Dept Orthopaed Surg, Oxford OX3 7LD, England.
   John Radcliffe Hosp, Dept Trauma, Oxford OX3 9DU, England.
RP Webb, J (通讯作者)，Southmead Gen Hosp, N Bristol NHS Trust, Dept Trauma & Orthoped Surg, Bristol BS10 5NB, Avon, England.
EM webbjonathan@hotmail.com
CR ARNER O, 1959, ACTA CHIR SCAND    S, V239, P1
   BRADLEY JP, 1990, AM J SPORT MED, V18, P188, DOI 10.1177/036354659001800213
   EASTWOOD DM, 1992, FOOT ANKLE, V13, P199, DOI 10.1177/107110079201300406
   GILLIES H, 1970, J BONE JOINT SURG AM, VA 52, P337, DOI 10.2106/00004623-197052020-00015
   HOCKENBURY RT, 1990, FOOT ANKLE, V11, P67, DOI 10.1177/107110079001100202
   HUENE DB, 1995, J BONE JOINT SURG AM, V77A, P1021, DOI 10.2106/00004623-199507000-00007
   INGLIS AE, 1976, J BONE JOINT SURG AM, V58, P990, DOI 10.2106/00004623-197658070-00015
   KLEIN W, 1991, Chirurgia degli Organi di Movimento, V76, P223
   LAWRENCE SJ, 1994, FOOT ANKLE INT, V15, P490, DOI 10.1177/107110079401500906
   LEA RB, 1972, J BONE JOINT SURG AM, VA 54, P1398, DOI 10.2106/00004623-197254070-00002
   MA GWC, 1977, CLIN ORTHOP RELAT R, P247
   NISTOR L, 1981, J BONE JOINT SURG AM, V63, P394, DOI 10.2106/00004623-198163030-00012
   ORTIGUELA ME, 1987, J HAND SURG-AM, V12A, P1119, DOI 10.1016/S0363-5023(87)80129-6
   QUIGLEY TB, 1980, AM J SPORT MED, V8, P244, DOI 10.1177/036354658000800406
   ROWLEY DI, 1982, INJURY, V14, P252, DOI 10.1016/0020-1383(82)90138-3
   Webb JM, 1999, J BONE JOINT SURG BR, V81B, P877, DOI 10.1302/0301-620X.81B5.9784
NR 16
TC 130
Z9 135
U1 0
U2 2
PU SAGE PUBLICATIONS INC
PI THOUSAND OAKS
PA 2455 TELLER RD, THOUSAND OAKS, CA 91320 USA
SN 1071-1007
EI 1944-7876
J9 FOOT ANKLE INT
JI Foot Ankle Int.
PD JUN
PY 2000
VL 21
IS 6
BP 475
EP 477
DI 10.1177/107110070002100604
PG 3
WC Orthopedics
WE Science Citation Index Expanded (SCI-EXPANDED)
SC Orthopedics
GA 326XB
UT WOS:000087761600004
PM 10884105
DA 2022-07-22
ER

PT J
AU Moller, M
   Karlsson, J
   Lind, K
   Mark, H
   Fogdestam, I
AF Moller, M
   Karlsson, J
   Lind, K
   Mark, H
   Fogdestam, I
TI Tissue expansion for repair of severely complicated Achilles tendon
   ruptures
SO KNEE SURGERY SPORTS TRAUMATOLOGY ARTHROSCOPY
LA English
DT Article
DE Achilles tendon; re-rupture; tissue expansion
ID NONOPERATIVE TREATMENT; MICROVASCULAR FLAPS; RECONSTRUCTION; DEFECTS;
   REGION
AB We report on two patients with severely complicated Achilles tendon ruptures (ATR), including rerupture, postoperative deep infection and subsequent tissue loss. Tissue expansion was used to facilitate tendon reconstruction and to ensure good healing potential with skin closure without tension. Tissue expansion is a valid option in patients with complicated ATR. The outcome for the two patients described here was good, with improved function and no major complications.
C1 Sahlgrens Univ Hosp, Dept Orthopaed, S-41685 Gothenburg, Sweden.
   Sahlgrens Univ Hosp, S-41345 Gothenburg, Sweden.
RP Moller, M (通讯作者)，Sahlgrens Univ Hosp, Dept Orthopaed, S-41685 Gothenburg, Sweden.
EM michael.moller@swipnet.se
CR ABRAHAM E, 1975, J BONE JOINT SURG AM, VA 57, P253, DOI 10.2106/00004623-197557020-00019
   BORGES PT, 1991, CLIN PLAST SURG, V18, P593
   BOSWORTH DM, 1956, J BONE JOINT SURG AM, V38, P111, DOI 10.2106/00004623-195638010-00011
   CETTI R, 1993, AM J SPORT MED, V21, P791, DOI 10.1177/036354659302100606
   HAGGMARK T, 1986, ORTHOPEDICS, V9, P160
   LANDVATER SJ, 1992, CLIN SPORT MED, V11, P741
   Leppilahti J, 1996, CLIN ORTHOP RELAT R, P171
   LIDMAN D, 1987, SCAND J PLAST RECONS, V21, P213, DOI 10.3109/02844318709078102
   Lo IKY, 1997, CLIN J SPORT MED, V7, P207, DOI 10.1097/00042752-199707000-00010
   MOHAMMED A, 1995, J BONE JOINT SURG BR, V77B, P64, DOI 10.1302/0301-620X.77B1.7822399
   NISTOR L, 1981, J BONE JOINT SURG AM, V63, P394, DOI 10.2106/00004623-198163030-00012
   SUOMINEN E, 1992, ACTA ORTHOP SCAND, V63, P482, DOI 10.3109/17453679209154719
   Thermann H, 2000, ORTHOPADE, V29, P235
   VOGELIN E, 1995, BRIT J PLAST SURG, V48, P579, DOI 10.1016/0007-1226(95)90048-9
   WARIS TH, 1991, SCAND J PLAST RECONS, V25, P87, DOI 10.3109/02844319109034929
   WEI FC, 1988, PLAST RECONSTR SURG, V81, P579, DOI 10.1097/00006534-198804000-00015
NR 16
TC 10
Z9 10
U1 0
U2 1
PU SPRINGER
PI NEW YORK
PA 233 SPRING ST, NEW YORK, NY 10013 USA
SN 0942-2056
EI 1433-7347
J9 KNEE SURG SPORT TR A
JI Knee Surg. Sports Traumatol. Arthrosc.
PD JUL
PY 2001
VL 9
IS 4
BP 228
EP +
DI 10.1007/s001670000185
PG 6
WC Orthopedics; Sport Sciences; Surgery
WE Science Citation Index Expanded (SCI-EXPANDED)
SC Orthopedics; Sport Sciences; Surgery
GA 457UK
UT WOS:000170155100009
PM 11522080
DA 2022-07-22
ER

PT J
AU Davlin, C
   Jenike, J
AF Davlin, C
   Jenike, J
TI Traumatic Achilles tendon rupture in a female college basketball player
SO JOURNAL OF SPORT REHABILITATION
LA English
DT Article
DE basketball; injury; rehabilitation
ID NON-SURGICAL TREATMENT; REPAIR; MOTION; MUSCLE; IMMOBILIZATION;
   WEIGHTBEARING
AB Objective: To present the case of a Division I female college basketball player with a complete Achilles tendon rupture. Background: A 19-year-old, female college basketball player ruptured her right Achilles tendon during preseason conditioning. She had no previous history of heel cord symptoms. Treatment: The athlete underwent open surgical repair of Achilles tendon. The athlete successfully progressed through a functional rehabilitation program focused on early mobilization and weight bearing. The rehabilitation program was continually modified to address deficiencies and to keep the athlete actively engaged. She was cleared for full, unrestricted activity 15 weeks and 3 days after surgery and returned to game participation in 16 weeks. Conclusions: This case provides evidence that early mobilization and weight bearing can be used while still protecting the repaired tendon.
C1 Xavier Univ, Sport Studies Dept, Cincinnati, OH 45207 USA.
   Xavier Univ, Athlet Dept, Cincinnati, OH 45207 USA.
RP Davlin, C (通讯作者)，Xavier Univ, Sport Studies Dept, Cincinnati, OH 45207 USA.
CR Aoki M, 1998, AM J SPORT MED, V26, P794, DOI 10.1177/03635465980260061001
   ARNER O, 1959, ACTA CHIR SCAND    S, V239, P1
   BESKIN JL, 1987, AM J SPORT MED, V15, P1, DOI 10.1177/036354658701500101
   BOOTH FW, 1987, CLIN ORTHOP RELAT R, P15
   CARTER TR, 1992, AM J SPORT MED, V20, P459, DOI 10.1177/036354659202000417
   CETTI R, 1993, AM J SPORT MED, V21, P791, DOI 10.1177/036354659302100606
   CHRISTENSEN I, 1953, ACTA CHIR SCAND, V106, P50
   Fahlstrom M, 1998, AM J SPORT MED, V26, P467, DOI 10.1177/03635465980260032201
   FernandezFairen M, 1997, AM J SPORT MED, V25, P177, DOI 10.1177/036354659702500206
   Grisogono V., 1989, PHYSIOTHERAPY, V75, P562, DOI [10.1016/S0031-9406(10)62335-8, DOI 10.1016/S0031-9406(10)62335-8]
   HAGGMARK T, 1986, ORTHOPEDICS, V9, P160
   INGLIS AE, 1976, J BONE JOINT SURG AM, V58, P990, DOI 10.2106/00004623-197658070-00015
   INGLIS AE, 1981, CLIN ORTHOP RELAT R, P160
   JOZSA L, 1989, AM J SPORT MED, V17, P338, DOI 10.1177/036354658901700305
   Kannus P, 1997, SCAND J MED SCI SPOR, V7, P107
   Leppilahti J, 1998, SPORTS MED, V25, P79, DOI 10.2165/00007256-199825020-00002
   Leppilahti J, 1996, INT J SPORTS MED, V17, P619, DOI 10.1055/s-2007-972905
   MANDELBAUM BR, 1995, AM J SPORT MED, V23, P392, DOI 10.1177/036354659502300403
   Neumann D, 1997, FOOT ANKLE INT, V18, P339, DOI 10.1177/107110079701800605
   NISTOR L, 1981, J BONE JOINT SURG AM, V63, P394, DOI 10.2106/00004623-198163030-00012
   OBRIEN M, 1992, CLIN SPORT MED, V11, P505
   Pneumaticos SG, 2000, FOOT ANKLE INT, V21, P551, DOI 10.1177/107110070002100704
   ROTHMAN RH, 1967, SURG GYNECOL OBSTETR, V124, P1064
   Schepsis AA, 2002, AM J SPORT MED, V30, P287, DOI 10.1177/03635465020300022501
   SOLVEBORN SA, 1994, AM J SPORT MED, V22, P607, DOI 10.1177/036354659402200507
   Speck M, 1998, AM J SPORT MED, V26, P789, DOI 10.1177/03635465980260060901
   THOMPSON T C, 1962, J Trauma, V2, P126, DOI 10.1097/00005373-196203000-00003
   Wong J, 2002, AM J SPORT MED, V30, P565, DOI 10.1177/03635465020300041701
NR 28
TC 0
Z9 0
U1 2
U2 3
PU HUMAN KINETICS PUBL INC
PI CHAMPAIGN
PA 1607 N MARKET ST, CHAMPAIGN, IL 61820-2200 USA
SN 1056-6716
J9 J SPORT REHABIL
JI J. Sport Rehabil.
PD MAY
PY 2004
VL 13
IS 2
BP 151
EP 166
DI 10.1123/jsr.13.2.151
PG 16
WC Rehabilitation; Sport Sciences
WE Science Citation Index Expanded (SCI-EXPANDED)
SC Rehabilitation; Sport Sciences
GA 824UR
UT WOS:000221713000005
DA 2022-07-22
ER

PT J
AU Fenelon, C
   Galbraith, JG
   Hession, P
   D'Souza, LG
AF Fenelon, Christopher
   Galbraith, John G.
   Hession, Paul
   D'Souza, Lester G.
TI Complete tendon Achilles rupture following injection of Aethoxysklerol
   (polidocanol) for the treatment of chronic Achilles tendinopathy
SO FOOT AND ANKLE SURGERY
LA English
DT Article
DE Aethoxysklerol; Polidocanol; Ethoxysclerol; Achilles tendon; Rupture;
   Achilles tendinopathy; Chronic
ID LOCAL STEROID INJECTION; SCLEROSING INJECTIONS; DOUBLE-BLIND;
   TENDINOSIS; NEOVASCULARIZATION; NEOVESSELS
AB Achilles tendinopathy can be a chronic disabling condition affecting both athletic and sedentary patients. Multiple new treatment approaches have developed, including shock wave therapy and various types of injection. One of the novel treatment methods used is the injection of Aethoxysklerol or polidocanol, a sclerosing substance injected under ultrasound guidance targeting areas of neovascularisation. We report the case of a 78-year-old lady who suffered a complete Achilles tendon rupture following injection of Aethoxysklerol. This is the first case of Achilles tendon rupture following Aethoxysklerol injection in isolation to our knowledge in the literature and the first published complication of this treatment. (C) 2016 European Foot and Ankle Society. Published by Elsevier Ltd. All rights reserved.
C1 [Fenelon, Christopher; Galbraith, John G.; D'Souza, Lester G.] Univ Hosp Limerick, Dept Orthopaed Surg, Limerick, Ireland.
   [Hession, Paul] Univ Hosp Limerick, Dept Radiol, Limerick, Ireland.
RP Fenelon, C (通讯作者)，18 Crescent Court,Father Griffin Rd, Galway, Ireland.
EM cgcfenelon@gmail.com; john@gmail.com; paul.hession@hse.ie;
   lgdsouza7@hotmail.com
CR Alfredson H, 2005, KNEE SURG SPORT TR A, V13, P74, DOI 10.1007/s00167-004-0549-x
   Alfredson H, 2005, KNEE SURG SPORT TR A, V13, P338, DOI 10.1007/s00167-004-0585-6
   Alfredson H, 2006, KNEE SURG SPORT TR A, V14, P1321, DOI 10.1007/s00167-006-0205-8
   Clementson M, 2008, KNEE SURG SPORT TR A, V16, P887, DOI 10.1007/s00167-008-0568-0
   FORD LT, 1979, SOUTH MED J, V72, P827, DOI 10.1097/00007611-197907000-00019
   Fredberg U, 2004, SCAND J RHEUMATOL, V33, P94, DOI 10.1080/03009740310004126
   Hamilton B, 2008, J SCI MED SPORT, V11, P566, DOI 10.1016/j.jsams.2007.03.008
   KLEINMAN M, 1983, J BONE JOINT SURG AM, V65, P1345, DOI 10.2106/00004623-198365090-00019
   Lind B, 2006, KNEE SURG SPORT TR A, V14, P1327, DOI 10.1007/s00167-006-0161-3
   Ohberg L, 2002, BRIT J SPORT MED, V36, P173, DOI 10.1136/bjsm.36.3.173
   Vallone G, 2014, J ULTRASOUND, V17, P165, DOI 10.1007/s40477-014-0066-9
   van Sterkenburg MN, 2010, AM J SPORT MED, V38, P2226, DOI 10.1177/0363546510373467
   Zanetti M, 2003, RADIOLOGY, V227, P556, DOI 10.1148/radiol.2272012069
   Zeisig E, 2006, KNEE SURG SPORT TR A, V14, P1218, DOI 10.1007/s00167-006-0156-0
NR 14
TC 1
Z9 1
U1 0
U2 2
PU ELSEVIER SCIENCE BV
PI AMSTERDAM
PA PO BOX 211, 1000 AE AMSTERDAM, NETHERLANDS
SN 1268-7731
EI 1460-9584
J9 FOOT ANKLE SURG
JI Foot Ankle Surg.
PD DEC
PY 2017
VL 23
IS 4
BP E7
EP E8
DI 10.1016/j.fas.2016.11.005
PG 2
WC Orthopedics
WE Science Citation Index Expanded (SCI-EXPANDED)
SC Orthopedics
GA FO9ZV
UT WOS:000417258500001
PM 29203002
DA 2022-07-22
ER

PT J
AU Yasuda, T
   Shima, H
   Mori, K
   Kizawa, M
   Neo, M
AF Yasuda, Toshito
   Shima, Hiroaki
   Mori, Katsunori
   Kizawa, Momoko
   Neo, Masashi
TI Direct Repair of Chronic Achilles Tendon Ruptures Using Scar Tissue
   Located Between the Tendon Stumps
SO JOURNAL OF BONE AND JOINT SURGERY-AMERICAN VOLUME
LA English
DT Article
ID NEGLECTED RUPTURE; RECONSTRUCTION; MANAGEMENT; AUTOGRAFT; DEFECTS; GRAFT
AB Background: Several surgical procedures for chronically ruptured Achilles tendons have been reported. Resection of the interposed scar tissue located between the tendon stumps and reconstruction using normal autologous tissue have been well described. We developed a direct repair procedure that uses scar tissue, which obviates the need to use normal autologous tissue.
   Methods: Thirty consecutive patients with Achilles tendon ruptures with a delay in diagnosis of > 4 weeks underwent removal of a section of scar and healing tissue with direct primary suture of the ends of the tendon without the use of allograft or autograft. Patients were followed for a mean time of 33 months. Preoperative and postoperative clinical outcomes were measured with the Achilles Tendon Total Rupture Score (ATRS) and the American Orthopaedic Foot & Ankle Society (AOFAS) ankle-hindfoot score. In addition, the patients underwent preoperative and postoperative functional measurements and magnetic resonance imaging. Lastly, we evaluated the histology of the interposed healing tissue.
   Results: The mean AOFAS scores were 82.8 points preoperatively and 98.1 points postoperatively. The mean postoperative ATRS was 92.0 points. At the time of the latest follow-up, none of the patients had experienced tendon reruptures or difficulties in walking or climbing stairs, and all except 2 patients could perform a single-limb heel rise. All athletes had returned to their pre-injury level of sports participation. Preoperative T2-weighted magnetic resonance imaging showed that 22 Achilles tendons were thickened with diffuse intratendinous high-signal alterations, and 8 Achilles tendons were thinned. Postoperative T2-weighted magnetic resonance imaging findings included fusiform-shaped tendon thickening and homogeneous low-signal alterations of the tendons in all patients. Histologically, the interposed scar tissue consisted of dense collagen fibers.
   Conclusions: Shortening of the tissue between the 2 tendon ends that included healing scar and direct repair of healing tendon without allograft or autograft can be effective for treatment-delayed or neglected Achilles tendon rupture.
C1 [Yasuda, Toshito; Shima, Hiroaki; Mori, Katsunori; Kizawa, Momoko; Neo, Masashi] Osaka Med Coll, Dept Orthoped Surg, Osaka, Japan.
RP Yasuda, T (通讯作者)，Osaka Med Coll, Dept Orthoped Surg, Osaka, Japan.
EM ort028@osaka-med.ac.jp; ort125@osaka-med.ac.jp; ort166@osaka-med.ac.jp;
   ort211@osaka-med.ac.jp; neo@osaka-med.ac.jp
RI SHIMA, HIROAKI/S-6153-2019
OI SHIMA, HIROAKI/0000-0003-4930-0007; Neo, Masashi/0000-0001-9208-6419
CR BARNES MJ, 1986, J BONE JOINT SURG BR, V68, P121, DOI 10.1302/0301-620X.68B1.3941128
   Bate J T, 1966, Clin Orthop Relat Res, V47, P151
   BECKER H, 1984, ORTHOP REV, V13, P65
   BOSWORTH DM, 1956, J BONE JOINT SURG AM, V38, P111, DOI 10.2106/00004623-195638010-00011
   BUGG EI, 1968, CLIN ORTHOP RELAT R, P73
   El Shazly O, 2014, ARTHROSCOPY, V30, P622, DOI 10.1016/j.arthro.2014.02.019
   El Shewy MT, 2009, AM J SPORT MED, V37, P1570, DOI 10.1177/0363546509333009
   GABEL S, 1994, FOOT ANKLE INT, V15, P512, DOI 10.1177/107110079401500912
   KANNUS P, 1991, J BONE JOINT SURG AM, V73A, P1507, DOI 10.2106/00004623-199173100-00009
   Kissel C G, 1994, J Foot Ankle Surg, V33, P46
   KITAOKA HB, 1994, FOOT ANKLE INT, V15, P349, DOI 10.1177/107110079401500701
   Kumar V., 2013, ROBBINS BASIC PATHOL, V9th ed., P29
   LANDIS JR, 1977, BIOMETRICS, V33, P159, DOI 10.2307/2529310
   Lee Daniel K, 2007, J Foot Ankle Surg, V46, P451, DOI 10.1053/j.jfas.2007.05.007
   Lee KB, 2009, KNEE SURG SPORT TR A, V17, P316, DOI 10.1007/s00167-008-0693-9
   Lee YS, 2005, ORTHOPEDICS, V28, P647, DOI 10.3928/0147-7447-20050701-10
   Leslie Hamish D H, 2005, Foot Ankle Clin, V10, P357, DOI 10.1016/j.fcl.2005.01.009
   Maffulli N, 1999, J BONE JOINT SURG AM, V81A, P1019, DOI 10.2106/00004623-199907000-00017
   Maffulli N, 2015, BONE JOINT J, V97B, P353, DOI 10.1302/0301-620X.97B3.33732
   Maffulli N, 2008, J BONE JOINT SURG AM, V90A, P1348, DOI 10.2106/JBJS.G.01241
   Maffulli N, 2013, AM J SPORT MED, V41, P1100, DOI 10.1177/0363546513479017
   Maffulli N, 2012, J BONE JOINT SURG AM, V94A, P906, DOI 10.2106/JBJS.K.00869
   MANDELBAUM BR, 1995, AM J SPORT MED, V23, P392, DOI 10.1177/036354659502300403
   MANN RA, 1991, J BONE JOINT SURG AM, V73A, P214, DOI 10.2106/00004623-199173020-00009
   Miskulin Mladen, 2005, J Foot Ankle Surg, V44, P49, DOI 10.1053/j.jfas.2004.11.003
   Mutlu EK, 2015, KNEE SURG SPORT TR A, V23, P2427, DOI 10.1007/s00167-014-3042-1
   Myerson M S, 1999, Instr Course Lect, V48, P219
   Nilsson-Helander K, 2007, AM J SPORT MED, V35, P421, DOI 10.1177/0363546506294856
   Patil SSD, 2014, FOOT ANKLE INT, V35, P699, DOI 10.1177/1071100714531228
   Porter DA, 1997, FOOT ANKLE INT, V18, P557, DOI 10.1177/107110079701800905
   Rahm S, 2013, FOOT ANKLE INT, V34, P1100, DOI 10.1177/1071100713487725
   Sarzaeem MM, 2012, KNEE SURG SPORT TR A, V20, P1386, DOI 10.1007/s00167-011-1703-x
   Us AK, 1997, ARCH ORTHOP TRAUM SU, V116, P408
   Wapner K L, 1993, Foot Ankle, V14, P443
   Wegrzyn J, 2010, INT ORTHOP, V34, P1187, DOI 10.1007/s00264-009-0859-1
   Yasuda T, 2000, ACTA ORTHOP SCAND, V71, P69, DOI 10.1080/00016470052943937
   Yasuda T, 2007, AM J SPORT MED, V35, P582, DOI 10.1177/0363546506295939
NR 37
TC 18
Z9 23
U1 0
U2 6
PU LIPPINCOTT WILLIAMS & WILKINS
PI PHILADELPHIA
PA TWO COMMERCE SQ, 2001 MARKET ST, PHILADELPHIA, PA 19103 USA
SN 0021-9355
EI 1535-1386
J9 J BONE JOINT SURG AM
JI J. Bone Joint Surg.-Am. Vol.
PD JUL 20
PY 2016
VL 98
IS 14
BP 1168
EP 1175
DI 10.2106/JBJS.15.00865
PG 8
WC Orthopedics; Surgery
WE Science Citation Index Expanded (SCI-EXPANDED)
SC Orthopedics; Surgery
GA DR5JB
UT WOS:000379938200009
PM 27440564
DA 2022-07-22
ER

PT J
AU Ding, WG
   Yan, WH
   Zhu, YP
   Liu, ZW
AF Ding, Wenge
   Yan, Weihong
   Zhu, Yaping
   Liu, Zhiwei
TI Treatment of acute and closed Achilles tendon ruptures by minimally
   invasive tenocutaneous suturing
SO ULUSAL TRAVMA VE ACIL CERRAHI DERGISI-TURKISH JOURNAL OF TRAUMA &
   EMERGENCY SURGERY
LA English
DT Article
DE Ankle; injury; surgery; trauma
ID NONOPERATIVE TREATMENT; FUNCTIONAL AFTERTREATMENT; SURGICAL TECHNIQUE;
   REPAIR
AB BACKGROUND
   Achilles tendon rupture is a common injury, and its complications can impair function. Numerous operations have been described for reconstructing the ruptured tendon, but these methods can compromise microcirculation in the tendon and can seriously impair its healing. Suturing with a minimally invasive tenocutaneous technique soon after the rupture and systematic functional exercise can greatly reduce the possibility of complications.
   METHODS
   Between June 1996 and February 2009, we treated 88 patients (54 males; age range, 21-66 years) with this method.
   RESULTS
   After follow-up ranging from 1-7 years, the mean American Orthopedic Foot and Ankle Society ankle-hind foot score was 95 (range, 90-98), and the maximum length of postoperative scarring was 3 cm. One patient re-ruptured his Achilles tendon one year after surgery in an accident, but after 10 months, the repaired tendon was still intact. In another patient, the nervus suralis was damaged during surgery by piercing the tension suture at the near end, causing postoperative numbness and swelling. The tension suture was quickly removed, and the patient recovered well with conservative treatment. No large irregular scars, such as those sustained during immobilization, were present over the Achilles tendon.
   CONCLUSION
   Minimally invasive percutaneous suturing can restore the original length and continuity of the Achilles tendon, is minimally invasive, and has fewer postoperative complications than other methods.
C1 [Ding, Wenge; Yan, Weihong; Zhu, Yaping; Liu, Zhiwei] Suzhou Univ, Dept Orthopaed Surg, Affiliated Hosp 3, Suzhou, Peoples R China.
RP Yan, WH (通讯作者)，Ju Qian St 185, Changzhou, Peoples R China.
EM sineboat@126.com
CR Ahmed IM, 1998, J ORTHOPAED RES, V16, P591, DOI 10.1002/jor.1100160511
   Boushel R, 2000, J PHYSIOL-LONDON, V524, P305, DOI 10.1111/j.1469-7793.2000.t01-2-00305.x
   BURCHHARDT H, 1992, Z ORTHOP GRENZGEB, V130, P109, DOI 10.1055/s-2008-1040122
   CARR AJ, 1989, J BONE JOINT SURG BR, V71, P100, DOI 10.1302/0301-620X.71B1.2914976
   CETTI R, 1993, AM J SPORT MED, V21, P791, DOI 10.1177/036354659302100606
   Cretnik A, 2005, AM J SPORT MED, V33, P1369, DOI 10.1177/0363546504271501
   Doral MN, 2009, ARCH ORTHOP TRAUM SU, V129, P1093, DOI 10.1007/s00402-009-0880-2
   GABEL S, 1994, FOOT ANKLE INT, V15, P512, DOI 10.1177/107110079401500912
   Gorschewsky O, 1999, INJURY, V30, P315, DOI 10.1016/S0020-1383(99)00089-3
   Ingvar J, 2005, ACTA ORTHOP, V76, P597, DOI 10.1080/17453670510041619
   JOZSA L, 1989, AM J SPORT MED, V17, P338, DOI 10.1177/036354658901700305
   KITAOKA HB, 1994, FOOT ANKLE INT, V15, P349, DOI 10.1177/107110079401500701
   Lansdaal JR, 2007, INJURY, V38, P839, DOI 10.1016/j.injury.2006.12.010
   Leppilahti J, 1996, ACTA ORTHOP SCAND, V67, P277, DOI 10.3109/17453679608994688
   Lo IKY, 1997, CLIN J SPORT MED, V7, P207, DOI 10.1097/00042752-199707000-00010
   Maffulli N, 1999, J BONE JOINT SURG AM, V81A, P1019, DOI 10.2106/00004623-199907000-00017
   Majewski M, 2000, ORTHOPADE, V29, P670
   Metz R, 2011, AM J SPORT MED, V39, P820, DOI 10.1177/0363546510392012
   Pavic R, 2008, INJURY, V39, P499, DOI 10.1016/j.injury.2007.11.423
   Rebeccato A, 2001, J Foot Ankle Surg, V40, P188
   Riedl S, 2002, CHIRURG, V73, P607, DOI 10.1007/s00104-002-0452-2
   Rothenbuhler J M, 1991, Z Unfallchir Versicherungsmed, V84, P25
   Sirovy M, 2007, Rozhl Chir, V86, P594
   Valente Maurizio, 2012, Musculoskelet Surg, V96, P35, DOI 10.1007/s12306-011-0158-z
   Weber M, 2003, AM J SPORT MED, V31, P685, DOI 10.1177/03635465030310050901
   WILLS CA, 1986, CLIN ORTHOP RELAT R, P156
   Yinger K, 2002, CLIN PODIATR MED SUR, V19, P231
   Yinger Kent, 2002, Clin Podiatr Med Surg, V19, P231, DOI 10.1016/S0891-8422(02)00002-2
NR 28
TC 1
Z9 3
U1 0
U2 2
PU TURKISH ASSOC TRAUMA EMERGENCY SURGERY
PI ISTANBUL
PA KOPRULU MEHMET PASA SOC, DENIZ ABDAL MAH, DADASOGLU AP, 25-1 SEHREMINI,
   ISTANBUL, 00000, TURKEY
SN 1306-696X
J9 ULUS TRAVMA ACIL CER
JI Ulus. Travma Acil Cerrahi Derg.
PD SEP
PY 2012
VL 18
IS 5
BP 405
EP 410
DI 10.5505/tjtes.2012.59376
PG 6
WC Emergency Medicine
WE Science Citation Index Expanded (SCI-EXPANDED)
SC Emergency Medicine
GA 032LY
UT WOS:000310721500006
PM 23188601
OA Bronze
DA 2022-07-22
ER

PT J
AU Park, JH
   Kim, SB
   Shin, HS
   Jung, GH
   Jung, YS
   Rim, H
AF Park, Jin Hee
   Kim, Sung Bin
   Shin, Ho Sik
   Jung, Gu Hee
   Jung, Yeon Soon
   Rim, Hark
TI Spontaneous and serial rupture of both Achilles tendons associated with
   secondary hyperparathyroidism in a patient receiving long-term
   hemodialysis
SO INTERNATIONAL UROLOGY AND NEPHROLOGY
LA English
DT Article
DE Achilles tendon; Spontaneous rupture; Hemodialysis
AB The spontaneous and serial rupture of the bilateral Achilles tendons without history of significant trauma is an uncommon complication in long-term hemodialysis (HD) patients. The majority of these patients have additional predisposing factors, such as previous use of fluoroquinolone antibiotics or corticosteroids. In general, this condition is associated with a coexisting systemic disease, including chronic kidney disease (CKD), secondary hyperparathyroidism, systemic lupus erythematosus (SLE), and diabetes mellitus (DM). Here, we report a 46-year-old man who had been undergoing regular HD for 11 years. He developed a spontaneous and consecutive rupture of both Achilles tendons. Based on previous reports of tendon ruptures in uremic patients and on the patient's lack of corticosteroid or fluoroquinolone use, we believe that secondary hyperparathyroidism was the predisposing factor in this patient. The mechanism seems to be related to a high parathyroid hormone (PTH) level, which results in osteolytic bone resorption at the tendon insertion site. Treatment and prevention of such tendon ruptures include early surgical repair and control of secondary hyperparathyroidism, by use of vitamin D analogs, and total parathyroidectomy, with or without autotransplantation of a parathyroid gland.
C1 [Park, Jin Hee; Kim, Sung Bin; Shin, Ho Sik; Jung, Yeon Soon; Rim, Hark] Kosin Univ, Coll Med, Dept Internal Med, 262 Gamcheon Ro, Pusan 602702, South Korea.
   [Jung, Gu Hee] Kosin Univ, Coll Med, Dept Orthoped Surg, Pusan 602702, South Korea.
RP Shin, HS (通讯作者)，Kosin Univ, Coll Med, Dept Internal Med, 262 Gamcheon Ro, Pusan 602702, South Korea.
EM danieljoseph@hanmail.net
CR Adhikari S, 2012, AM J EMERG MED, V30, DOI 10.1016/j.ajem.2011.01.029
   Basic-Jukic N, 2009, KIDNEY BLOOD PRESS R, V32, P32, DOI 10.1159/000201792
   Brountzos E, 2009, J NEPHROL, V22, P476
   DEFRANCO P, 1994, AM J KIDNEY DIS, V24, P951, DOI 10.1016/S0272-6386(12)81067-5
   Finlayson GR, 1964, JAMA-J AM MED ASSOC, V187, P657
   Hartgerink P, 2001, RADIOLOGY, V220, P406, DOI 10.1148/radiology.220.2.r01au41406
   Hess Gregory William, 2010, Foot Ankle Spec, V3, P29, DOI 10.1177/1938640009355191
   KURER MHJ, 1991, J BONE JOINT SURG BR, V73, P271, DOI 10.1302/0301-620X.73B2.2005153
   Malaguti M, 2001, J NEPHROL, V14, P431
   Palmer S, 2004, NEPHROLOGY, V9, P262, DOI 10.1111/j.1440-1797.2004.00269.x
   Shiota E, 2002, CLIN ORTHOP RELAT R, P236
   Tsourvakas S, 2004, ARCH ORTHOP TRAUM SU, V124, P278, DOI 10.1007/s00402-003-0628-3
   Ureten K, 2008, INT UROL NEPHROL, V40, P1103, DOI 10.1007/s11255-008-9421-0
   van der Linden PD, 2003, ARCH INTERN MED, V163, P1801, DOI 10.1001/archinte.163.15.1801
   Willits K, 2010, J BONE JOINT SURG AM, V92A, P2767, DOI 10.2106/JBJS.I.01401
   Worth N, 2007, J Orthop Surg (Hong Kong), V15, P311
NR 16
TC 10
Z9 10
U1 0
U2 5
PU SPRINGER
PI DORDRECHT
PA VAN GODEWIJCKSTRAAT 30, 3311 GZ DORDRECHT, NETHERLANDS
SN 0301-1623
EI 1573-2584
J9 INT UROL NEPHROL
JI Int. Urol. Nephrol.
PD APR
PY 2013
VL 45
IS 2
BP 587
EP 590
DI 10.1007/s11255-011-0111-y
PG 4
WC Urology & Nephrology
WE Science Citation Index Expanded (SCI-EXPANDED)
SC Urology & Nephrology
GA 108YZ
UT WOS:000316333600038
PM 22241073
DA 2022-07-22
ER

PT J
AU Sarzaeem, MM
   Lemraski, MMB
   Safdari, F
AF Sarzaeem, Mohammad Mahdi
   Lemraski, Mohammad Mahdi Bagherian
   Safdari, Farshad
TI Chronic Achilles tendon rupture reconstruction using a free
   semitendinosus tendon graft transfer
SO KNEE SURGERY SPORTS TRAUMATOLOGY ARTHROSCOPY
LA English
DT Article
DE Achilles tendon; Chronic rupture; Semitendinosus; Tendon autograft;
   Surgery
ID LESS-INVASIVE RECONSTRUCTION; HALLUCIS LONGUS TRANSFER; NEGLECTED TEARS;
   REPAIR; AUGMENTATION; MANAGEMENT; FLAPS; MORBIDITY
AB The purpose of this study was to evaluate the outcomes following reconstruction of the chronic Achilles tendon ruptures with large gaps (> 6 cm) using free semitendinosus tendon graft transfer.
   There were 11 consecutive patients underwent the above-mentioned surgical technique for the treatment of chronically ruptured Achilles tendon contributed in current study and were followed up prospectively for a mean of 25 +/- A 3 months. The intraoperative tendon defect was greater than 6 cm in all of the patients. Functional and clinical assessment was performed using The American Orthopaedic Foot and Ankle Society (AOFAS) and Achilles Tendon Rupture Score (ATRS).
   The average AOFAS and ATRS improved significantly from 70 +/- A 5 and 32 +/- A 6 preoperatively, to 92 +/- A 5 and 89 +/- A 4 points post-operatively (P = 0.001). The range of dorsiflexion was significantly limited on the operated side (13 +/- A 4A degrees vs. 17 +/- A 4A degrees) (P = 0.04). All patients were able to stand on the tiptoe of injured leg, and no patient walked with a visible limp. Post-operative complications included one patient with symptomatic DVT and 2 patients with superficial infection treated nonoperatively.
   The technique offers good clinical and functional outcomes and is safe. Reconstruction of the chronic Achilles tendon ruptures with free semitendinosus tendon graft in patients with defects greater than 6 cm is recommended.
   IV.
C1 [Sarzaeem, Mohammad Mahdi; Lemraski, Mohammad Mahdi Bagherian] Shahid Beheshti Med Univ, Imam Hosein Hosp, Dept Orthopaed, Tehran, Iran.
   [Safdari, Farshad] Shahid Beheshti Med Univ, Akhtar Orthopaed Hosp, Akhtar Orthopaed Res Ctr, Tehran, Iran.
RP Sarzaeem, MM (通讯作者)，Shahid Beheshti Med Univ, Imam Hosein Hosp, Dept Orthopaed, Tehran, Iran.
EM mmszaeem@gmail.com
RI Sarzaeem, Mohammad Mahdi/AAQ-7439-2021
OI Sarzaeem, Mohammad Mahdi/0000-0002-6256-1036
CR Coull R, 2003, FOOT ANKLE INT, V24, P931, DOI 10.1177/107110070302401211
   El Shewy MT, 2009, AM J SPORT MED, V37, P1570, DOI 10.1177/0363546509333009
   FernandezFairen M, 1997, AM J SPORT MED, V25, P177, DOI 10.1177/036354659702500206
   Hahn F, 2008, CLIN BIOMECH, V23, P109, DOI 10.1016/j.clinbiomech.2007.08.015
   Ibrahim SAR, 2009, J FOOT ANKLE SURG, V48, P340, DOI 10.1053/j.jfas.2009.02.007
   Jennings AG, 2002, J BONE JOINT SURG BR, V84B, P361, DOI 10.1302/0301-620X.84B3.11559
   Ji JH, 2007, FOOT ANKLE INT, V28, P1100, DOI 10.3113/FAI.2007.1100
   Karousou E, 2008, CLIN ORTHOP RELAT R, V466, P1577, DOI 10.1007/s11999-008-0255-y
   KITAOKA HB, 1994, FOOT ANKLE INT, V15, P349, DOI 10.1177/107110079401500701
   Kosanovic M, 2008, ARCH ORTHOP TRAUM SU, V128, P211, DOI 10.1007/s00402-007-0514-5
   Lee KB, 2009, KNEE SURG SPORT TR A, V17, P316, DOI 10.1007/s00167-008-0693-9
   Lee YS, 2005, ORTHOPEDICS, V28, P647, DOI 10.3928/0147-7447-20050701-10
   LIEBERMAN JR, 1988, CLIN ORTHOP RELAT R, P204
   Lui TH, 2007, KNEE SURG SPORT TR A, V15, P1163, DOI 10.1007/s00167-007-0352-6
   Maffulli N, 2005, CLIN J SPORT MED, V15, P56, DOI 10.1097/01.jsm.0000152714.05097.ef
   Maffulli N, 2008, J BONE JOINT SURG AM, V90A, P1348, DOI 10.2106/JBJS.G.01241
   Maffulli N, 2008, BMC MUSCULOSKEL DIS, V9, DOI 10.1186/1471-2474-9-100
   Maffulli N, 2010, AM J SPORT MED, V38, P2304, DOI 10.1177/0363546510376619
   Maffulli N, 2010, KNEE SURG SPORT TR A, V18, P269, DOI 10.1007/s00167-009-0968-9
   Mahajan Ravindra H, 2009, J Orthop Surg (Hong Kong), V17, P194
   Nilsson-Helander K, 2008, KNEE SURG SPORT TR A, V16, P614, DOI 10.1007/s00167-008-0492-3
   Nilsson-Helander K, 2007, AM J SPORT MED, V35, P421, DOI 10.1177/0363546506294856
   Nilsson-Helander K, 2009, KNEE SURG SPORT TR A, V17, P1234, DOI 10.1007/s00167-009-0727-y
   OZAKI J, 1989, CLIN ORTHOP RELAT R, P204
   Padanilam TG, 2009, FOOT ANKLE CLIN, V14, P711, DOI 10.1016/j.fcl.2009.08.001
   Pintore E, 2001, J TRAUMA, V50, P71, DOI 10.1097/00005373-200101000-00013
   Richardson DR, 2009, FOOT ANKLE INT, V30, P627, DOI 10.3113/FAI.2009.0627
   Takao M, 2003, ARCH ORTHOP TRAUM SU, V123, P471, DOI 10.1007/s00402-002-0443-2
   Tay D, 2010, ANN ACAD MED SINGAP, V39, P58
   Wegrzyn J, 2010, INT ORTHOP, V34, P1187, DOI 10.1007/s00264-009-0859-1
NR 30
TC 30
Z9 33
U1 0
U2 4
PU SPRINGER
PI NEW YORK
PA 233 SPRING ST, NEW YORK, NY 10013 USA
SN 0942-2056
EI 1433-7347
J9 KNEE SURG SPORT TR A
JI Knee Surg. Sports Traumatol. Arthrosc.
PD JUL
PY 2012
VL 20
IS 7
BP 1386
EP 1391
DI 10.1007/s00167-011-1703-x
PG 6
WC Orthopedics; Sport Sciences; Surgery
WE Science Citation Index Expanded (SCI-EXPANDED)
SC Orthopedics; Sport Sciences; Surgery
GA 962FP
UT WOS:000305527400021
PM 22037808
DA 2022-07-22
ER

PT J
AU Medeiros, DM
AF Medeiros, Diulian Muniz
TI Conservative treatment of Achilles tendon partial tear in a futsal
   player: A case report
SO PHYSIOTHERAPY THEORY AND PRACTICE
LA English
DT Article
DE Plantarflexors; rehabilitation; triceps surae
ID LEVEL LASER THERAPY; ECCENTRIC EXERCISE; TENDINOPATHY; RUPTURE; SPORT;
   RECOVERY; PHOTOBIOMODULATION; REHABILITATION; PERFORMANCE; MANAGEMENT
AB Background and purpose: Achilles tendon is the most commonly injured part in the lower limb, especially in athletes. Treatment options for Achilles tendinopathy and total rupture are well described; however, there is a lack of information regarding treatment protocols for partial tears. Thus, the purpose of this case report was to describe the examination, intervention and outcomes of patient who suffered an acute Achilles tendon partial tear. Case description: A case is described here, in which the diagnosis of Achilles tendon partial tear was based on both magnetic resonance imaging and physical evaluation. Both the patient and the physical therapy team opted for non-surgical treatment. A 12-week course of conservative treatment including exercise, tendon loading, electrical stimulation, and photobiomodulation is described. Outcomes: Ankle dorsiflexion range of motion and hop tests. Discussion: This case report demonstrated that non-surgical treatment for Achilles tendon partial tear was effective for this patient and enabled the athlete to return to pre-injury levels of activity 6 months following the injury.
C1 [Medeiros, Diulian Muniz] Fed Univ Hlth Sci Porto Alegre, Phys Therapy Dept, Grad Program Rehabil Sci, Rua Sarmento Leite 245, BR-90050170 Porto Alegre, RS, Brazil.
RP Medeiros, DM (通讯作者)，Fed Univ Hlth Sci Porto Alegre, Phys Therapy Dept, Grad Program Rehabil Sci, Rua Sarmento Leite 245, BR-90050170 Porto Alegre, RS, Brazil.
EM Diulian.medeiros@yahoo.com
CR Alfredson H, 2007, BRIT J SPORT MED, V41, P211, DOI 10.1136/bjsm.2007.035543
   ALLENMARK C, 1992, CLIN SPORT MED, V11, P759
   Antonialli FC, 2014, LASER MED SCI, V29, P1967, DOI 10.1007/s10103-014-1611-7
   Ardern CL, 2016, BRIT J SPORT MED, V50, P853, DOI 10.1136/bjsports-2016-096278
   Ardern CL, 2013, BRIT J SPORT MED, V47, P1120, DOI 10.1136/bjsports-2012-091203
   Askling CM, 2007, AM J SPORT MED, V35, P1716, DOI 10.1177/0363546507303563
   Bayer ML, 2017, NEW ENGL J MED, V377, P1300, DOI 10.1056/NEJMc1708134
   Beyer R, 2015, AM J SPORT MED, V43, P1704, DOI 10.1177/0363546515584760
   Biewener AA, 2004, J APPL PHYSIOL, V97, P2266, DOI 10.1152/japplphysiol.00003.2004
   Bohm H, 2006, J APPL BIOMECH, V22, P3, DOI 10.1123/jab.22.1.3
   Cohen DB, 2006, AM J SPORT MED, V34, P362, DOI 10.1177/0363546505280428
   De Marchi T, 2017, LASER MED SCI, V32, P429, DOI 10.1007/s10103-016-2139-9
   Doral MN, 2010, KNEE SURG SPORT TR A, V18, P638, DOI 10.1007/s00167-010-1083-7
   Dorn TW, 2012, J EXP BIOL, V215, P1944, DOI 10.1242/jeb.064527
   ELVERU RA, 1988, PHYS THER, V68, P672, DOI 10.1093/ptj/68.5.672
   Emerson C, 2010, MANUAL THER, V15, P37, DOI 10.1016/j.math.2009.05.008
   FUKASHIRO S, 1995, EUR J APPL PHYSIOL O, V71, P453, DOI 10.1007/BF00635880
   Hess Gregory William, 2010, Foot Ankle Spec, V3, P29, DOI 10.1177/1938640009355191
   Hutchison AM, 2015, BONE JOINT J, V97B, P510, DOI 10.1302/0301-620X.97B4.35314
   JOZSA L, 1989, AM J SPORT MED, V17, P338, DOI 10.1177/036354658901700305
   Kannus Pekka, 1992, Scandinavian Journal of Medicine and Science in Sports, V2, P164
   Khan KM, 2003, BRIT J SPORT MED, V37, P149, DOI 10.1136/bjsm.37.2.149
   Khan RJK, 2009, COCHRANE DB SYST REV, DOI 10.1002/14651858.CD003674.pub3
   Kjaer M., 2005, Journal of Musculoskeletal & Neuronal Interactions, V5, P41
   KOMI PV, 1990, J BIOMECH, V23, P23, DOI 10.1016/0021-9290(90)90038-5
   KOMI PV, 1992, CLIN SPORT MED, V11, P521
   KVIST M, 1994, SPORTS MED, V18, P173, DOI 10.2165/00007256-199418030-00004
   Maffulli N, 2019, BMJ-BRIT MED J, V364, DOI 10.1136/bmj.k5344
   Magnan B, 2014, FOOT ANKLE SURG, V20, P154, DOI 10.1016/j.fas.2014.02.010
   Malliaras P, 2013, SPORTS MED, V43, P267, DOI 10.1007/s40279-013-0019-z
[truncated: 18,962,277 more chars]
